# LAYLA: development of a comprehensive and cross-sample program for detecting structural variants and its application to citrus cultivars

**DOI:** 10.1270/jsbbs.25020

**Published:** 2025-10-28

**Authors:** Tomoaki Watanabe, Tomoko Endo, Yoshihiro Kawahara, Kenta Shirasawa, Keisuke Nonaka, Sachiko Isobe, Takehiko Shimada, Shingo Goto, Hiroshi Fujii

**Affiliations:** 1 Institute of Fruit Tree and Tea Science, National Agriculture and Food Research Organization (NARO), 2-1 Fujimoto, Tsukuba, Ibaraki 305-8605, Japan; 2 Research Center for Advanced Analysis, NARO, 2-1-2 Kannondai, Tsukuba, Ibaraki 305-8602, Japan; 3 Kazusa DNA Research Institute, Kisarazu, Chiba 292-0818, Japan; 4 Graduate School of Agricultural and Life Sciences, The University of Tokyo, Tokyo 113-8657, Japan; 5 Faculty of Agriculture, Shizuoka University, 836 Ohya, Suruga-ku, Shizuoka 422-8529, Japan

**Keywords:** indel, mapping, long read, python, genome, *Citrus unshiu*, structural variant

## Abstract

Structural variants (SVs) are genomic mutations that are typically 50 bp or larger. Given their larger scale compared to single nucleotide polymorphisms and small insertions and deletions, SVs are expected to be associated with various traits in several crops and fruit species. They can also be used to identify plant cultivars. However, it is challenging to detect SVs using short-read next-generation sequencing (NGS), which, until recently, has been the mainstream method, due to its short read length compared to SVs. In recent years, long-read NGS, which generates reads exceeding the length of SVs, has made SV detection more feasible. To take advantage of this, we developed LAYLA (Large indel AnalYzer for muLti-sAmple), a pipeline program designed to comprehensively detect and visualize SVs across multiple samples using long-read data. Here, we applied LAYLA to 13 citrus founder cultivars used in Japanese breeding and *Citrus unshiu* Marc. We identified SVs at 59,983 positions in the reference genome. This analysis revealed both common and cultivar-specific SVs. Furthermore, we designed primers targeting nine selected SVs and conducted experimental validation, confirming the presence of SVs detected by LAYLA. In the future, LAYLA can be applied to other plant species to detect SVs.

## Introduction

Structural variants (SVs) are genomic variations that are 50 base pairs (bp) or longer (Chiang *et al.* 2017, Ho *et al.* 2020). They encompass various mutations, including deletions, insertions, duplications, inversions, and translocations (Escaramís *et al.* 2015, Ho *et al.* 2020).

SVs play crucial roles in genetic variations as well as evolution. In a recent study (Liu *et al.* 2021), approximately 49% of deletions exhibited low linkage disequilibrium with adjacent single-nucleotide polymorphisms (SNPs), suggesting SVs’ probable association with genetic variation not captured by SNPs. Furthermore, SVs can cause gene loss, gene duplication, and the generation of new genes, which may significantly influence complex agronomic traits (Yuan *et al.* 2021).

Several studies have demonstrated a correlation between SVs and economically significant traits in several crop species. For example, an insertion in the promoter region of the *ZmVPP1* gene in maize enhances photosynthetic efficiency and root development (Wang *et al.* 2016), and a 182 bp deletion in the *AT1G11520* gene of *Arabidopsis thaliana* is associated with delayed flowering (Liu *et al.* 2021). Additionally, SVs in tomatoes influence fruit flavor, size, and productivity (Alonge *et al.* 2020).

In fruit tree research, studies have increasingly focused on the role of transposons in shaping SVs and their subsequent influence on the evolution of key traits. Transposon insertion within the *MYB* gene in grapes is associated with variation in skin color (Azuma *et al.* 2007, Kobayashi *et al.* 2004). In firm-flesh peach cultivars, a transposon inserted into an auxin gene (which induces ethylene biosynthesis) suppresses fruit softening (Tatsuki *et al.* 2018). A transposon inserted upstream of a gibberellin synthesis gene in apple triggers gibberellin expression in aboveground tissues, resulting in the characteristic columnar growth form (Okada *et al.* 2016, 2020). Polyembryony in citrus is initiated by the insertion of a MITE-type transposon (Shimada *et al.* 2018).

These examples illustrate how SVs directly affect important agronomic traits and can be utilized for crop improvement. Insertions and deletions (InDels) can serve as useful genetic markers and have been successfully implemented for citrus cultivar identification (Endo *et al.* 2022). InDels offer distinct advantages over SNP- or SSR-based markers because they can be detected using PCR amplification and electrophoresis. Specifically, they can be adapted to simple screening methods—such as chromatographic printed array strip method (Monden *et al.* 2023)—even in facilities with limited laboratory resources (such as customs offices). This ability helps identify cultivars that may infringe on breeders’ rights in Japan (Okamoto *et al.* 2023).

Detection of SVs remains a challenging task due to their inherent complexity and diversity (Mahmoud *et al.* 2019). Short-read sequencing, which is the predominant method used in next-generation sequencing (NGS), has limitations in detecting large-scale or complex SVs because the size of some variants often exceeds the length of sequencing reads. Consequently, although SVs—particularly large InDels (those exceeding 50 bp)—hold great potential, including for cultivar identification, the availability of such SV information for a wide range of cultivars remains insufficient.

Long-read technology is effective in detecting SVs in the human genome (Chaisson *et al.* 2019), and long-read sequencing techniques have been used to analyze large-scale SVs in tomatoes (Alonge *et al.* 2020). The impact of long-read sequencing parameters on SV detection has been comprehensively evaluated, leading to the standardization of SV analysis (Jiang *et al.* 2021). Subsequent studies have applied long-read-based SV analyses to various major crops, including bread wheat, thereby solidifying its role as a crucial instrument in crop genome research (Athiyannan *et al.* 2022, Kajiya-Kanegae *et al.* 2021).

The advent of long-read technologies has fueled the development of corresponding software. For example, minimap2 (Li 2018) is a versatile aligner capable of mapping read sequences from various NGS platforms, including Sequel System (Pacific Biosciences) and PromethION (Oxford Nanopore Technologies), offering fast and accurate alignments. It has also been effective in detecting SVs from long reads (Li 2018). Similarly, Sniffles (Sedlazeck *et al.* 2018) is designed to filter out erroneous SV signals often arising from unreliable reads and can detect multiple SV types (such as insertions, deletions, duplications, inversions, and breakends) using long-read alignments. Recent advancements and improvements in these tools enabled high-accuracy SV detection from long reads.

Though SV detection has become increasingly accessible, it still requires combining multiple tools for variant calling, filtering, and interpretation. In addition to Sniffles, useful programs such as cuteSV (Jiang *et al.* 2020) and VolcanoSV (Luo *et al.* 2024) have been developed for accurate SV detection from long-read data. CuteSV focuses on fast, single-sample SV calling via signal clustering, whereas VolcanoSV employs haplotype-resolved, assembly-based detection. However, these tools are not designed for cross-sample or comparative SV analysis.

Sniffles2 (Smolka *et al.* 2024), an updated version of Sniffles, supports joint SV genotyping across samples by merging individual results. However, the workflow remains complex: each sample must be processed separately, and the resulting multi-sample variant call format (VCF) file is often difficult to interpret directly. In addition, existing tools often lack (i) automated retrieval of flanking sequences of SVs, (ii) flexible filtering options—such as by gene region—or (iii) clear visualization of SV patterns across samples.

To address these limitations, we developed a pipeline program that facilitates SV detection, filtering, and visualization across multiple samples. We named this program “Large indel AnalYzer for muLti-sAmple” (LAYLA). LAYLA was designed to integrate various programs, allowing the identification of SVs of each cultivar based on the positional information of the reference genome. As noted above, this information can be utilized for various purposes, including the development of markers for cultivar identification.

Furthermore, as a case study, we applied LAYLA to the founder cultivars used in Japanese citrus breeding, to comprehensively list the SVs present in the founder cultivars and in the progeny cultivars that inherited their genomes. Using these findings, we evaluated the utility of LAYLA. Additionally, we designed primers for several of the identified SVs, performed PCR, and conducted electrophoresis to directly validate the accuracy of results obtained using LAYLA.

## Materials and Methods

### Software required for LAYLA implementation

LAYLA was developed in Python version 3.11.0. Three software packages, namely minimap2 version 2.26-r1175, Sniffles version 2.2, and samtools version 1.13, (Danecek *et al.* 2021) are required to run the program. LAYLA relies on several external Python libraries, which include pandas version 2.1.4 (McKinney 2010), PyVCF version 0.6.8 (https://pypi.org/project/PyVCF/), Numpy version 1.26.3 (Harris *et al.* 2020), and openpyxl version 3.1.2 (https://pypi.org/project/openpyxl/).

### Sample data

A total of 14 samples were used in this study: 13 cultivars that serve as founders in Japanese citrus breeding (Endo *et al.* 2022, Imai *et al.* 2018) and *Citrus unshiu* Marc. ‘Miyagawa wase’ (hereafter referred to as Satsuma mandarin), with parents estimated to be *C. kinokuni* hort. ex Tanaka (referred to as Kishu mikan) and *C. nobilis* Lour. var. kunep Tanaka (referred to as Kunenbo) (Fujii *et al.* 2016). The 13 cultivars included *C. maxima* (Burm.) Merr. ‘Tosa-buntan’ (referred to as Pummelo), *C. hassaku* hort. ex Tanaka (referred to as Hassaku), *C. paradisi* Macf. (referred to as Grapefruit), *C. sinensis* (L.) Osbeck (referred to as Sweet orange), *C. tamurana* hort. ex Tanaka (referred to as Hyuganatsu), *C. iyo* hort. ex Tanaka (referred to as Iyokan), Kishu mikan, Kunenbo, *C. reticulata* Blanco (referred to as Ponkan), *C. tangerina* hort. ex Tanaka (referred to as Dancy tangerine), *C. nobilis* Lour. (referred to as King mandarin), a tangor cultivar ‘Murcott’ (referred to as Murcott tangor), and *C. deliciosa* Tenore (referred to as Mediterranean mandarin). We used the long-read PacBio CCS data (BioProject Accession: PRJDB15866 [BioSample Accession: SAMD00608599, SAMD00608600, SAMD00608601], PRJDB15974 [SAMD00622473, SAMD00622475, SAMD00622477, SAMD00622478, SAMD00622479, SAMD00622480, SAMD00622482, SAMD00622486, SAMD00622488, SAMD00622491, SAMD00622492]) from the 14 samples, which was obtained following a previously described method (https://doi.org/10.1101/2023.06.02.543356) to execute LAYLA.

Across these 14 cultivars, the mean read length was 22,776 bp; the mean read quality was 25; the mean number of reads was 325,985; the mean N50 was 23,264 bp; the mean standard deviation was 5,310 bp; and the mean total base count was approximately 7,302 Mb. While there was some variation in the mean read count and the total base count among the cultivars, even Dancy tangerine—the cultivar with the fewest reads—yielded 166,960 reads and approximately 4,085 Mb of total bases. This indicated that the data obtained for each cultivar were of high quality (Supplemental Table 1).

The unphased genome (version r1.0) of Satsuma mandarin (https://doi.org/10.1101/2023.06.02.543356) was obtained from Mikan Genome Database (Kawahara *et al.* 2020) and used as the reference genome for mapping. Note that the reference genome used in this study was constructed from the long-read sequences of Satsuma mandarin provided for LAYLA.

### Algorithm of the LAYLA software

**Fig. 1.**
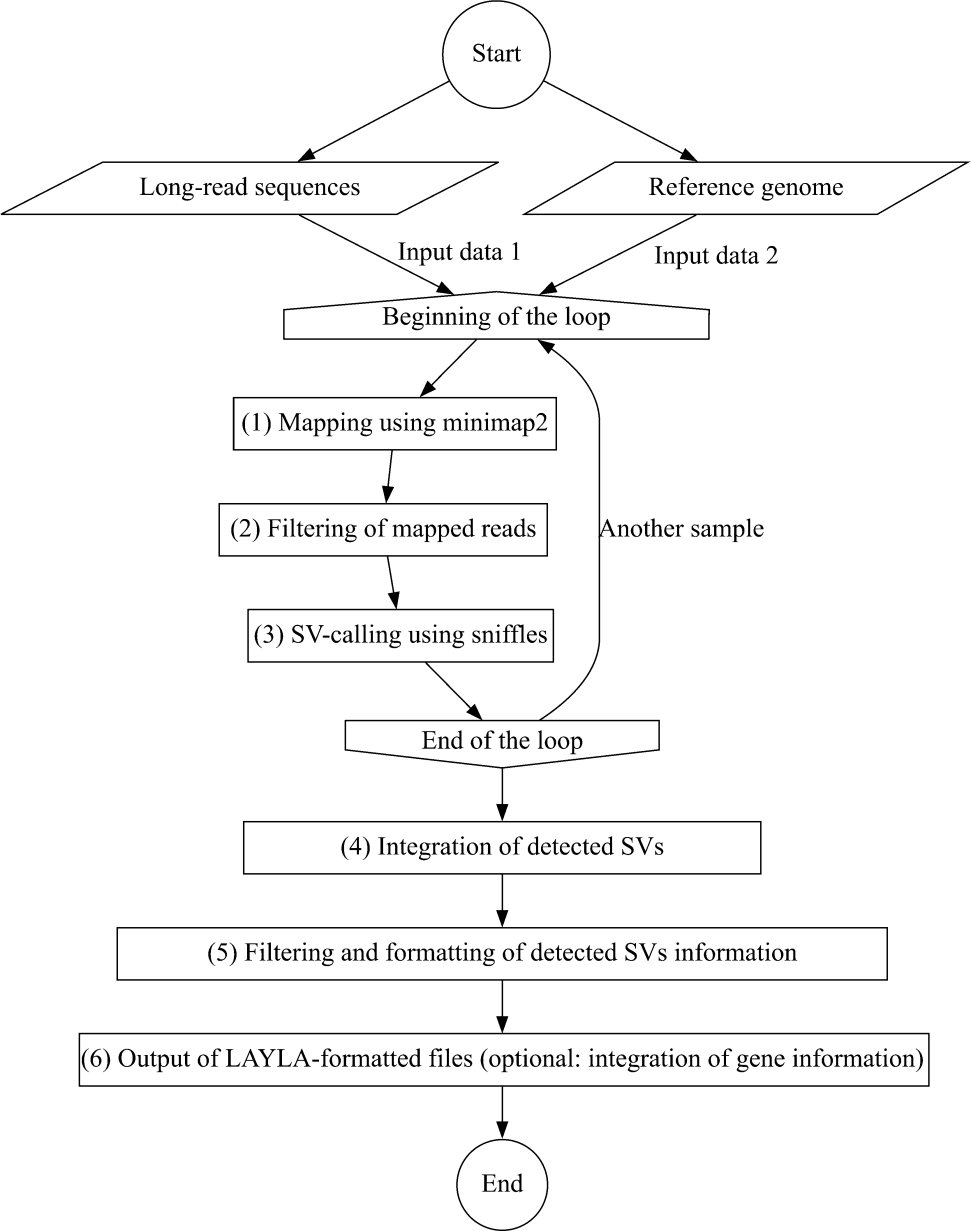
Process flow of LAYLA program. This flowchart illustrates the comprehensive workflow of the LAYLA program, starting with the input of long-read sequencing data and a reference genome. The program systematically processes these inputs to detect structural variants (SVs).

LAYLA was developed to comprehensively detect SVs shared among multiple cultivars or unique to individual cultivars, using PacBio CCS reads and a reference genome as input. The pipeline consisted of the following six primary steps (Fig. 1): (1) mapping long reads to the reference genome using minimap2, (2) filtering the mapped reads, (3) SV-calling using sniffles, (4) integrating the SVs detected in each sample based on their positions in the reference genome, (5) filtering the integrated SVs, and (6) generating the final output in the LAYLA format.

### Mapping long reads and filtering of mapped reads

In the first step, the provided PacBio CCS long-read sequences were mapped to the reference genome using minimap2 with the PacBio CCS option (Fig. 1). Subsequently, samtools was used to remove secondary mapped reads from the results, and the output was generated as a SAM file (Fig. 1).

The SAM file was converted into a BAM file, then sorted and indexed using samtools. Reads with low-quality scores (which, by default, includes a mapping score <60) were removed, and the resulting BAM file was then produced and stored for further analysis (Fig. 1).

### SV-calling

Using the BAM file as input, Sniffles was used for SV detection. Under default settings, only genomic regions with a coverage of five reads or more are considered for SV detection, and only SVs of 50 bp or longer are reported. However, these parameters can be adjusted using the optional settings of LAYLA. The procedures for long-reads mapping, mapped-reads filtering, and SV-calling were repeated for multiple samples, which generated an SNF file (defined in Sniffles) for each sample containing the corresponding SV information (Fig. 1). The detected SVs were classified by Sniffles into different types. Insertions were identified when sequences that did not exist in the reference genome were found in the mapped long reads. Deletions were detected when sequences present in the reference genome were absent in the mapped long reads. Inversions were identified when the orientation of a genomic region was reversed compared to the reference. Duplications were detected when a genomic sequence was present in more copies than in the reference genome. Sniffles also classified other types of structural variants as breakends (BNDs).

### Integration of SV-calling results

Sniffles was then executed in Multi-Sample SV Calling mode using the obtained SNF files. In this mode, SVs detected at identical or closely adjacent positions in the reference genome across different cultivars were considered to be the same SV. The threshold for positional offset was calculated as: M × sqrt(min(SV_length_a, SV_length_b)). Here, M is a constant defined in Sniffles (default: 250), and SV_length_a and SV_length_b represent the lengths of the two SVs being compared, respectively. “sqrt” is a function that returns the square root of the given argument, and “min” is a function that returns the minimum value among the given arguments. Increasing M enhances the tolerance of positional discrepancies. Consequently, the SVs from each sample were aligned to the corresponding positions in the reference genome, and a single VCF file was produced. This file included genotype data for each cultivar for all matching SVs.

### Filtering of SVs

Low-confidence SVs were removed from the VCF file if they met any of the following criteria: (1) the standard deviation of detected position (STDEV_POS) was one or more, (2) the standard deviation of SV length (STDEV_LEN) was one or more, or (3) the IMPRECISE flag (denied by Sniffles) was set. These values served as default thresholds, but the thresholds for STDEV_POS and STDEV_LEN could be modified via optional parameters. This filtering step was implemented within LAYLA’s custom Python code by reading the VCF file generated by Sniffles into a pandas DataFrame and applying the above conditions programmatically.

### Output of files defined by LAYLA

**Fig. 2.**
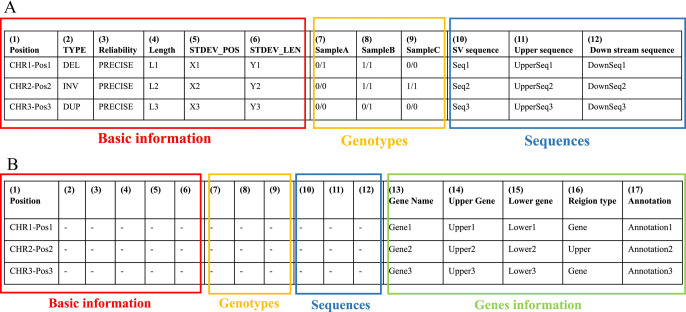
Overview of LAYLA and LAYLA_gene files. This figure illustrates the structure of two types of output files defined by LAYLA. (A) Description of LAYLA files. LAYLA files contain structural variant (SV) information, organized into multiple columns. Columns (1)–(6) provide basic SV details, columns (7)–(9) describe genotypes (varying based on the number of samples), and columns (10)–(12) present SV sequences and their surrounding regions. The details of each column are as follows: (1) The position of the reference genome where the SV was detected. The chromosome and position are written with a hyphen as a separator. (2) The type of detected SV. (3) The reliability of the detected SV (defined by Sniffles). (4) The length of the detected SV. (5) STDEV_POS is a numerical value indicating the degree of variation in the detected SV positions among different samples. (6) STDEV_LEN is a numerical value indicating the degree of variation in the lengths of the detected SVs among different samples. (7)–(9) The SV detection results for Samples A–C (the number of columns is variable and corresponds to the number of samples). The genotype is listed for each position in the reference genome: (i) 0/0 indicates that the SV was not detected. (ii) 0/1 indicates a heterozygous SV. (iii) 1/1 indicates a homozygous SV. (10) The sequence of the detected SV. (11) The upstream sequence of the detected SV, with a default length of 300 bp. (12) The downstream sequence of the detected SV, with a default length of 300 bp. (B) Description of LAYLA_gene files. LAYLA_gene files extend the LAYLA file format by incorporating additional gene-related information. This file format only includes SVs located in the gene coding region or gene upstream region. In addition to columns (1)–(12) from LAYLA files, it includes the following columns: (13) The name of the gene. (14) Upstream neighboring gene name. (15) Downstream neighboring gene name. (16) The region where the SV is located, indicating whether the SV is present in the coding region of the gene or in the upstream region. (17) Gene annotation. The number of columns varies depending on the content of the input annotation file provided at runtime.

Based on the filtered SVs, a “LAYLA file” (defined in LAYLA) was generated (Fig. 2A). In this file, the following output was generated: the position of the SV, the type of SV, the length of SV, the genotype of each sample relative to the reference (one of 0/0 [no SV], 0/1 [heterozygous SV], or 1/1 [homozygous SV]), the base sequence of the SV, the reference base sequence upstream of the SV, and the reference base sequence downstream of the SV. By default, 300 bp of the reference genome’s upstream and downstream sequences were extracted, but this length could be edited using an option.

Even when the SVs are derived from the same ancestor, they may exhibit slight discrepancies in detected SV positions and lengths due to variations between the reference genome and the sample’s long reads, sequencing errors, or software-related issues. In LAYLA, if SV positions differed between cultivars but still met the threshold set by Sniffles, they were treated as SVs at the same position. This allowed the genotype of each sample to be organized and output at the same reference position. The allowable positional discrepancy could be adjusted by changing the value of the “-M” option (refer to Section “*Integration of SV-calling results*”).

When the “--anno” option in LAYLA was executed, a “LAYLA_gene” (defined in LAYLA) file was generated, in addition to the LAYLA file (Fig. 2B). This file contained the information for only those SVs that were present in gene regions and their upstream regions, along with added gene annotations. In addition to the information in the LAYLA file, the following details were also generated: the name of the gene in which the SV occurred, the name of the gene upstream of that position, the name of the gene downstream of that position, and gene annotations.

When using the “--anno” option, it is essential to provide a Browser Extensible Data (BED) file as input. This BED file must include columns for chromosome number, gene number, gene start position, and the gene end position, as well as an arbitrary column for gene annotations. By default, LAYLA defines a gene’s upstream region as the 2,000 bp upstream from the start of its coding region, or up to the end of any upstream gene if that boundary is within 2,000 bp. However, the “--region” option is provided to change the default upstream region length.

### Running LAYLA for a test

We executed LAYLA using the Satsuma mandarin genome as the reference genome and the long-read data from the 14 citrus cultivars described in Section “*Sample data*”. Additionally, we provided gene information to LAYLA using the annotation option (--anno) and set the threshold value for filtering mapped reads to 50. The annotation BED file specified with the --anno option was generated by converting the general feature format file registered in the Mikan Genome Database (CUNuph_r1.0_gene_primary.gff3), and gene regions were defined according to this file.

### Principal component analysis (PCA) using SVs

To assess the genomic similarity between samples, PCA was performed using SV genotypes detected by LAYLA. For each SV genotype in the LAYLA result files for each cultivar, 0/0 (no SVs) was converted to 0, 0/1 (heterozygous SVs) to 1, and 1/1 (homozygous SVs) to 2. PCA was conducted using the prcomp function in R with center = TRUE and scale. = TRUE, while all other parameters were kept at their default values.

### Verification of LAYLA execution results

To confirm the presence of SVs detected by LAYLA, we designed PCR primers using Primer3 (Koressaar and Remm 2007) to amplify regions encompassing the identified SVs. The parameters of Primer3 were configured such that the designed primers would be 29 nucleotides in length. Other settings were left at their default values. These primers were applied to the 14 cultivars, and the resulting PCR band patterns were analyzed. DNA from the 14 cultivars was extracted using the DNeasy Plant Mini Kit (Qiagen), following the manufacturer’s protocol (Abdel-Latif and Osman 2017). The band pattern validation followed a previously established protocol (Endo *et al.* 2022), and PCR amplification was conducted using KOD One^®^ PCR Master Mix (TOYOBO).

### Availability

The copyright for LAYLA belongs to National Agriculture and Food Research Organization (NARO), and permission from NARO is required for its use. Usage permission can be obtained by contacting NARO through their website (https://prd.form.naro.go.jp/form/pub/naro01/program). Additionally, outsourced computation using LAYLA can be conducted at NARO.

## Results

### Implementation and requirements of LAYLA

LAYLA can be installed on Linux/UNIX-like operating systems such as Red Hat Enterprise Linux, Ubuntu, and Mac OS X, and can be executed with a single command. Different options are available to modify its behavior (Supplemental Table 2); detailed instructions for using LAYLA are provided in the user manual (which is bundled with LAYLA).

### Analysis across the whole genome

Upon analyzing the 14 cultivars, a total of 59,983 SVs were identified in the LAYLA file (Supplemental Table 3, excluding sequence data for readability and size constraints). These SVs included 29,058 deletions (~48.44%), 24,867 insertions (~41.46%), 545 inversions (~0.90%), and 470 duplications (~0.78%). Across the entire genomic region of 372.92 Mb, the density of SVs was approximately 160.85 per Mb. It should be noted that the total number of SVs refers to the number of positions in the reference genome where SVs were detected. This corresponds to the number of record rows in the LAYLA file. Each row in the LAYLA file represents the genotype of an SV detected at a specific position in the reference genome across multiple samples (including 0/0, which indicates that no SV was detected at that position in a given sample). It is important to note that this total number is unrelated to the genotype distribution within samples. Additionally, each row contains information about only one type of SV; different types of SVs are not mixed within a single row.

### Number of SVs for each chromosome

The highest number of SVs was detected on chromosome 3 with 10,056 SVs, whereas the lowest number was detected on chromosome 4 with 5,633 SVs. Additionally, the density of SVs detected per Mb on each chromosome ranged from 112.58 SVs/Mb (detected in chromosome 8) to 197.33 SVs/Mb (detected in chromosome 7) (Table 1).

### Genomic distribution of SVs

**Fig. 3.**
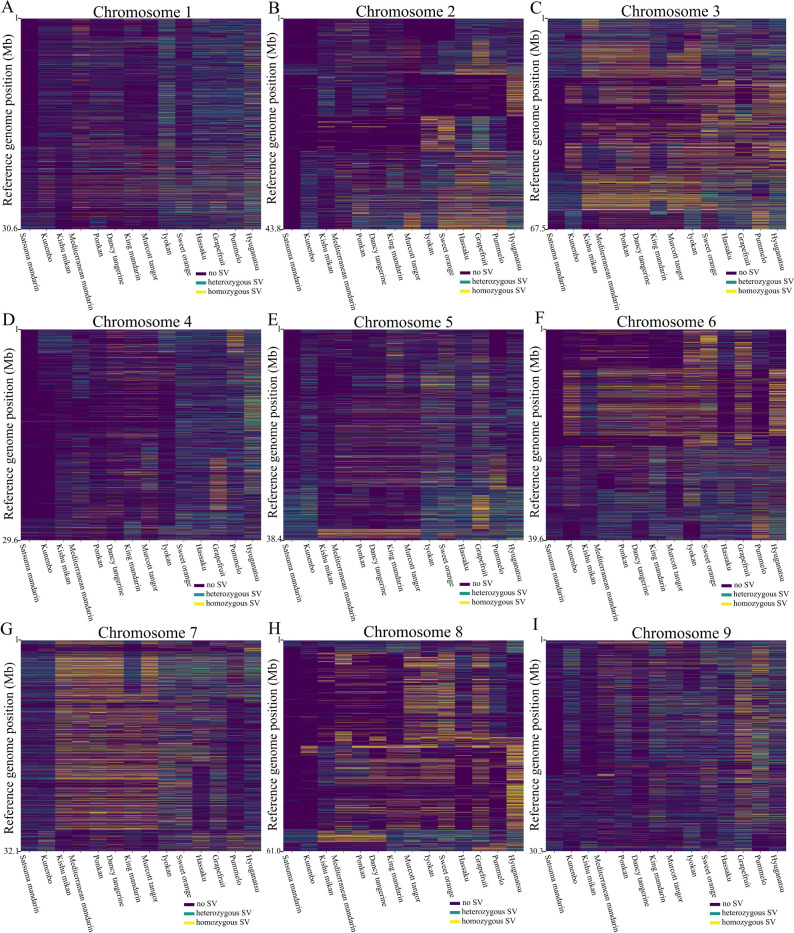
Distribution of structural variants genotypes for each cultivar across chromosomes. This figure provides an overview of the genotypes of detected structural variants (SVs) along each chromosome, from upstream to downstream at their respective positions in the reference genome. The Y-axis represents positions on the reference genome where structural variants SVs were detected in at least one cultivar. Due to the large number of detected sites, tick marks are omitted for readability, except at the top and bottom ends (corresponding to the ends of the chromosome). The X-axis corresponds to each cultivar. The detection results for each cultivar are indicated using one of three colors corresponding to genotypes of SVs. Dark blue indicates that SVs were detected in other cultivars, but not in the cultivar corresponding to the X-axis (0/0), green indicates a heterozygous SV (0/1), and yellow indicates a homozygous SV (1/1). To ensure consistent scaling across chromosomes, additional points were inserted every 50,000 bp as well as at both ends of each chromosome, even in regions where no SVs were detected. Panels (A)–(I) correspond to chromosomes 1–9.

The distribution of SVs in each chromosome was visualized by color-coding the genotypes for each cultivar (dark blue indicates the absence of an SV at the corresponding position in the reference genome, green indicates heterozygous SVs, and yellow indicates homozygous SVs) (Fig. 3). The analysis revealed that the SVs in the sample data were distributed across the entire genome. The distribution of SV genotypes detected on each chromosome showed different patterns, which are likely to reflect differences in genomic structure among the chromosomes and cultivars. On chromosome 7, the distribution of homozygous SVs, shown in yellow, exhibited a somewhat similar pattern across Kishu mikan, Mediterranean mandarin, Ponkan, Dancy tangerine, and Murcott tangor. This suggests that the genomic structures in these regions of chromosome 7 may be similar among these cultivars. However, homozygous SVs were also detected in Satsuma mandarin, even though the long-read sequences used in LAYLA were derived from the same individual as the reference genome (refer to Section “*Sample data*”). This suggests that some of the detected SVs may be attributable to sequencing errors in the long-read data or inaccuracies in the reference genome itself.

Although SVs were detected across extensive regions of the genome relative to the reference, the number detected in each cultivar varied by genomic region (Supplemental Fig. 1). These region-specific differences likely reflect cultivar-dependent genome structures, and regions where all cultivars show comparatively few SVs may correspond to highly conserved genome regions.

### Distribution of lengths of SVs

**Fig. 4.**
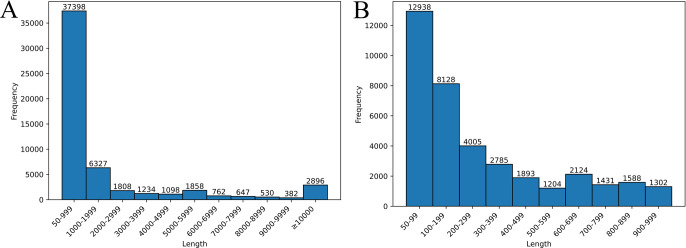
Histogram showing the number of structural variants categorized by length. This figure shows the distribution of structural variants (SVs) by length. The y-axis represents the count of SVs in each length interval. (A) includes all lengths, with SVs longer than 10,000 bp grouped into a single bin. (B) displays data with a length upper limit of 1,000 bp.

The detected SVs were aggregated based on their length, excluding SVs for which length information was not reported by sniffles (Fig. 4). The most prevalent SVs ranged from 50 to 100 bp in size (SVs that were shorter than 50 bp were excluded by LAYLA as described in “Materials and Methods”), with 12,938 variants representing approximately 21.57% of all SVs. As the length of the SVs increased, their number decreased; nevertheless, large SVs exceeding 10,000 bp were relatively common, with 2,896 such SVs identified.

### Proportion of homozygous and heterozygous SVs

The number of homozygous and heterozygous SVs detected in each cultivar was aggregated (Table 2). Among cultivars other than Satsuma mandarin, the number of homozygous variants was smaller than that of heterozygous variants, with an average ratio of about 0.64. The number of homozygous SVs in Satsuma mandarin was 158, while the number of heterozygous SVs was 6,048, resulting in a homozygous variant ratio of approximately 0.026. This proportion was clearly lower than other cultivars. This result is consistent with the fact that both the reference genome and the long-read sequences were derived from the same individual (refer to Section “*Sample data*”).

Additionally, 17,516 SVs (approximately 29.2%) exhibited all three genotypes across the 14 cultivars. The three genotypes 0/0, 0/1, and 1/1 in the LAYLA file represented no SVs, heterozygous SVs, and homozygous SVs, respectively. These SVs, which exhibited high genetic diversity, were distributed across chromosomes, ranging from 1,098 to 3,263 per chromosome (Table 1).

### Detection of transposable elements in SV sequences

The SV sequences corresponding to insertions and deletions—defined here as the variant regions themselves, not including flanking sequences—were analyzed for transposon detection using RepeatMasker (https://www.repeatmasker.org/). Transposable elements (TEs) were identified in 40.43% of these SVs. Further analysis showed that 35.75% of the SVs contained retrotransposons, while 7.77% contained DNA transposons.

### Number of SVs for each cultivar

The number of SVs detected per cultivar ranged from 11,693 in Dancy tangerine to 21,217 in Hyuganatsu, excluding Satsuma mandarin and its parental cultivars, Kishu mikan and Kunenbo (Table 2). The number of SVs detected in Satsuma mandarin, Kishu mikan, and Kunenbo were 6,206, 11,510, and 11,592, respectively, which is fewer than in the other cultivars. This can be attributed to the Satsuma mandarin genome being used as the reference genome. Aggregating the detected SVs by category showed the highest numbers to be 12,213 deletions in Grapefruit, 8,012 insertions in Hyuganatsu, 140 inversions and 117 duplications in Sweet orange.

### Results for PCA using SVs

**Fig. 5.**
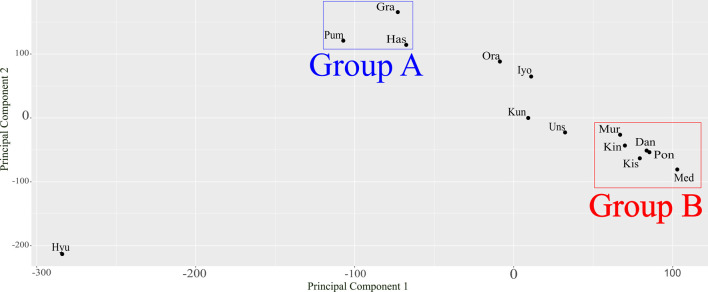
Principal component analysis results based on the structural variants genotypes. Each point represents a sample, with the proximity of points reflecting their genetic relatedness. The three closely positioned cultivars, Gra, Has, and Pum, were designated as Group A, while the six closely positioned cultivars, Mur, Kin, Dan, Kis, Pon, and Med, were designated as Group B. Hyu: Hyuganatsu; Pum: Pummelo; Gra: Grapefruit; Has: Hassaku; Ora: Sweet orange; Iyo: Iyokan; Kun: Kunenbo; Uns: Satsuma mandarin; Mur: Murcott tangor; Kin: King mandarin; Kis: Kishu mikan; Dan: Dancy tangerine; Pon: Ponkan; Med: Mediterranean mandarin.

According to the results of PCA based on SV genotypes detected by LAYLA (Fig. 5), Hyuganatsu was positioned in a distinct cluster from other cultivars. Grapefruit, Hassaku, and Pummelo were found in close proximity and were considered to form the same cluster (Group A). Similarly, Murcott tangor, King mandarin, Dancy tangerine, Kishu mikan, Ponkan, and Mediterranean mandarin were also in relatively close proximity, forming another cluster (Group B). Other cultivars, namely Sweet orange, Iyokan, Kunenbo, and Satsuma mandarin were located between Groups A and B. However, Sweet orange and Iyokan were closer to group A, whereas Kunenbo and Satsuma mandarin were closer to group B.

### Distribution of SVs in gene regions and their upstream regions

The LAYLA_gene output obtained from the sample data was used to compile the number of SVs detected in upstream and genic regions. Among these, 7,159 SVs were identified within gene regions, and 11,803 SVs were identified in the upstream regions, accounting for approximately 31.61% of the total SVs detected across the entire genome. Additionally, out of all 27,818 genes in Satsuma mandarin, 5,420 genes were found to harbor the SVs detected in this study, which accounted for 19.48% of the total genes.

### Validation of LAYLA results

To validate the output of LAYLA, we selected one SV per chromosome and designed primers accordingly (Supplemental Table 4). The selected SVs included insertions or deletions of 1,000 bp or less, and only those with three distinguishable genotypes (0/0, 0/1, 1/1) among the cultivars were considered. Furthermore, we assumed that any detected homozygous SVs in Satsuma mandarin would be unreliable because theoretically at least 50% of the long-read sequences were expected to match the reference genome sequence, as both the reference genome and the long-read sequences were derived from the same individual (refer to Section “*Sample data*”). While some of these may represent true SVs, we excluded them due to concerns about their reliability.

Electrophoresis (Supplemental Fig. 2) revealed that the predicted genotypes based on the band patterns of each marker were almost entirely consistent with those predicted by LAYLA (Table 3). Notably, for marker4, marker6, and marker7, the genotypes inferred from the electrophoresis band patterns matched the predictions of LAYLA for all cultivars. However, discrepancies were observed in some cases. For marker1 and marker8, the genotype of Dancy tangerine contradicted the electrophoresis results (Table 3). For marker2, marker5, and marker9, discrepancies were found in the genotype of Pummelo (Table 3), whereas for marker3, discrepancies were observed in the genotypes of Murcott tangor and Pummelo (Table 3).

## Discussion

### Overview of LAYLA

In this study, we developed LAYLA, a program that comprehensively detects SVs, and used it to analyze SVs using long-read data from 14 citrus cultivars. The output of LAYLA, presented as “LAYLA files”, facilitated comprehensive visualization of SVs along the reference genome across various groups of cultivars, hybrid populations, or family populations (Fig. 2). These files include the SV sequences and the flanking nucleotide sequences, which were used to design primers that span SV regions (Fig. 2). Consequently, LAYLA facilitated the comprehensive and cross-sample identification of SVs that were previously cumbersome to detect, even with long-read sequencing data. Furthermore, it can be used to develop molecular markers and support other applications.

### Potential of LAYLA

Approximately 160.85 SVs were detected per 1 Mb of chromosome when 14 citrus cultivars were compared with the reference genome (Table 1). Given the genome-wide distribution of SVs (Fig. 3), these SVs can serve as markers for linkage analysis, genome-wide association studies, and cultivar identification.

In genetic statistical analyses aimed at identifying regions associated with breeding traits, SVs are more likely to be directly related to traits compared to SNPs or SSRs. Therefore, the relationship between the SV genotypes obtained through LAYLA in these regions and the traits can be used to identify causal SVs. Even if direct identification is not achieved, SVs in the relevant regions can still be applied for fine mapping.

In the present study, the LAYLA file contained not only information on indel length but also the genotypic information for samples (Fig. 2), enabling the efficient development of co-dominant markers with higher detection power. In test cases, many SVs were found to exhibit three genotypes across the 14 cultivars (ranging from 1,098 to 3,263 per chromosome, Table 1). Furthermore, SVs smaller than 1,000 bp were predominant (Fig. 4). This suggests that LAYLA can be used to design co-dominant markers at any location within the genome.

### Detection results of LAYLA in test cases

In this study, approximately 31.61% of the detected SVs were located within genes or upstream regions of genes (refer to Section “*Distribution of SVs in gene regions and their upstream regions*”). Thus, using LAYLA enabled the detection of many SVs associated with certain gene functions. The LAYLA_gene file (Fig. 2B) contained the names of genes with SVs and the genotypes of each sample. Therefore, if trait data for each sample is available, the relationship between specific breeding traits and SVs can likely be elucidated.

In this study, transposable element sequences were detected in approximately 40.43% of the SVs (refer to Section “*Detection of transposable elements in SV sequences*”). Thus, using LAYLA to detect SVs can help assess SVs that are derived from transposons. Furthermore, integration of SV data with trait information can provide insights into the relationship between specific traits and transposon-derived SVs.

### PCA using SVs

Many citrus cultivars, including Satsuma mandarin, Sweet orange, and Grapefruit, are hybrids of mandarin and pummelo, and their genomes exhibit a mosaic structure composed of genomic segments derived from both mandarin and pummelo (Wu *et al.* 2018). In this study, the reference genome of Satsuma mandarin, which comprises approximately three-quarters of genomic segments derived from mandarin and one-quarter derived from pummelo, was utilized (Wu *et al.* 2014, 2018, 2021). Numerous SVs were detected in cultivars such as Grapefruit, Sweet orange, Hassaku, and Iyokan, all of which harbor substantial pummelo-derived genomes (Table 2). Conversely, fewer SVs were detected in cultivars predominantly carrying mandarin-derived genomes (Table 2). These results could be attributed to genomic similarity and were consistent with the results of PCA performed using SVs (Fig. 5). Therefore, the PCA results based on the genotypes of SVs detected by LAYLA are considered to reflect genomic similarity, which may be further useful for inferring phylogenetic relationships.

### LAYLA validation using electrophoresis

The differences in the length of PCR amplification product sizes between genotypes at each marker are correspond well with the SV lengths predicted by LAYLA (Supplemental Fig. 2). Therefore, the result of the electrophoresis band patterns obtained using the designed primers is considered to accurately represent the presence or absence of SVs. The concordance rate between the genotyping results estimated by LAYLA and the genotypes predicted from electrophoresis results was, on average, 94.4% (Table 3), indicating that the results obtained using LAYLA are highly reliable. Additionally, none of the designed markers exhibited monomorphism, as all markers showed polymorphism among the cultivars. Consequently, the regions where SVs were detected by LAYLA are potentially valuable for breeding due to the polymorphisms found among the cultivars. However, for some cultivars (namely, Murcott tangor, Dancy tangerine, and Pummelo), the genotyping results estimated by LAYLA did not match those predicted from electrophoresis (Table 3). These cultivars tended to have a relatively low number of long-read sequences used as input (Supplemental Table 1). This suggests that providing higher-quality long-read data as input is crucial for improving the reliability of LAYLA.

In the electrophoresis results for marker 6, a third band, slightly larger in size compared to the other bands, was specifically observed in cultivars with a heterozygous SV (Supplemental Fig. 2F). This additional band is likely due to the formation of a heteroduplex (Noda *et al.* 2021).

### Limitations

While LAYLA outputs the genotype (heterozygous or homozygous) of each detected SV for each sample, some discrepancies were observed between these results and the validation experiments. This suggests that experimental validation remains crucial for confirming SV genotypes, particularly in cases where high accuracy in SV genotyping is critical. Additionally, variations in genotype estimation accuracy were observed among samples. Differences in the number of long reads across samples indicated that data quality could influence LAYLA’s estimation accuracy.

Though LAYLA was only applied to citrus cultivars in the present study, the algorithm of LAYLA is versatile and can be applied to other organisms in further studies. However, for broader and larger-scale analyses, the cost of long-read sequencing remains a practical limitation. One realistic strategy would be to first identify structural variants (SVs) in the parents using LAYLA, and then develop indel markers based on those SVs for genotyping individuals in a population. In addition, combining long-read results with short-read sequencing data could enable the identification of SNPs linked to SVs. If such SNPs are available, conventional genotyping methods could be used to indirectly utilize SV information. Through such integrative approaches, it should be possible to extend the applicability of LAYLA to large populations while mitigating cost-related constraints.

The current version of LAYLA assumes alignment to a single genome sequence and does not explicitly incorporate haplotype structure. Incorporating phased reference genomes may help resolve haplotype-specific structural variants (SVs), particularly in highly heterozygous species. For example, a future extension could involve running LAYLA separately on each haplotype genome and integrating the results, which may improve the interpretation of parental origin and haplotype specificity of SVs.

### Conclusion

In this study, we developed a novel pipeline program named LAYLA, designed for the comprehensive and cross-sample detection of structural variations (SVs) using long-read sequencing data from multiple samples along with reference genome sequences. The output was provided in a human-readable tabular format, facilitating the identification of SVs that were either shared across samples or unique to specific ones. In addition, by incorporating genome annotation data, LAYLA enabled the selective extraction of SVs located within gene bodies or upstream regulatory regions.

We applied LAYLA to 14 citrus cultivars of significant importance in Japanese breeding programs. As a result, SVs were detected at 59,983 genomic sites, corresponding to a density of 160.85 SVs per megabase. To validate these findings, PCR and electrophoresis experiments were performed using primers designed to target the detected SVs, confirming their presence. The capacity of LAYLA to detect SVs across multiple samples is expected to support various applications, such as the development of DNA markers for cultivar identification and genotyping markers for specific genes.

## Author Contribution Statement

T.W. wrote the manuscript, developed the program, and conducted the validation experiments. T.E., K.S., K.N., S.I., and T.S. contributed to the acquisition of long-read data used for analysis. S.G. contributed to the validation experiments. Y.K. contributed to the analysis using RepeatMasker. H.F. contributed to the acquisition of long-read data and the development of the program’s algorithm.

## Supplementary Material

Supplemental Figures

Supplemental Tables

## Figures and Tables

**Table 1.** Number and density of structural variants per chromosome. The table shows the number of structural variants (SVs) detected on each chromosome and their density per megabase (second column and third column). The fourth column shows the number of SVs for which all three genotypes (homozygous SV, heterozygous SV, and reference/no SV) were observed across samples at the corresponding positions in the reference genome.

Chromosome	Number of SVs	Density of SVs (Count per megabase)	Number of SVs with three genotypes
Chromosome 1	5,773	188.82	1,098
Chromosome 2	7,111	162.30	2,381
Chromosome 3	10,056	148.91	3,263
Chromosome 4	5,633	190.00	1,381
Chromosome 5	6,821	177.86	1,685
Chromosome 6	5,683	143.36	1,683
Chromosome 7	6,329	197.33	2,141
Chromosome 8	6,863	112.58	2,158
Chromosome 9	5,714	188.41	1,726
Total	59,983	160.85	17,516

**Table 2.** Number of structural variants detected in each cultivar and their genotypic distribution. For each cultivar, the table shows the number of structural variants (SVs) detected by type (as defined by Sniffles) and the total number of SVs. The numbers in parentheses in the “Total” column indicate the counts of homozygous and heterozygous SVs, for each cultivar, respectively.

Cultivar	Deletion	Insertion	Breakend	Inversion	Duplication	Total (Homozygous/Heterozygous)
Satsuma mandarin	3,944	1,958	246	37	21	6,206 (158/6,048)
Kunenbo	7,218	3,636	608	68	62	11,592 (1,819/9,773)
Kishu mikan	6,882	3,927	581	78	42	11,510 (3,755/7,755)
Mediterranean mandarin	7,907	4,651	744	82	77	13,461 (6,447/7,014)
Ponkan	8,816	4,505	899	92	90	14,402 (6,236/8,166)
Dancy tangerine	7,336	3,525	666	87	79	11,693 (6,108/5,585)
King Mandarin	8,436	4,672	828	91	74	14,101 (4,950/9,151)
Murcott tangor	8,306	4,058	783	85	85	13,317 (6,704/6,613)
Iyokan	10,838	5,423	976	118	98	17,453 (6,615/10,838)
Sweet orange	11,810	6,548	940	140	117	19,555 (7,489/12,066)
Hassaku	10,818	5,869	723	103	87	17,600 (4,354/13,246)
Grapefruit	12,213	6,523	905	139	92	19,872 (8,240/11,632)
Pummelo	10,478	5,778	668	98	85	17,107 (5,732/11,375)
Hyuganatsu	11,960	8,012	1,012	130	103	21,217 (7,460/13,757)

**Table 3.** Genotypes of each marker by cultivar. The table lists, for every cultivar, the genotype of each marker targeting a structural variant (SV). For each cultivar, the genotype estimated by LAYLA is shown in the upper row, while the genotype inferred from electrophoresis results is shown in the lower row (separated by a bold horizontal line). The “Discrepant cultivar” row indicates cultivars where the LAYLA estimate conflicts with the electrophoresis result; these conflicting genotypes are highlighted with gray cell shading. The “Length” row presents the SV length, including the value estimated by LAYLA and the approximate length inferred from electrophoresis.

	Method	Marker1	Marker2	Marker3	Marker4	Marker5	Marker6	Marker7	Marker8	Marker9
Chromosome		1	2	3	4	5	6	7	8	9
Position		1,671,715	4,213,105	15,527,721	6,092,891	360,86,970	39,199,213	7,498,561	60,820,786	59,79,910
SV Type		insertion	deletion	deletion	deletion	insertion	insertion	deletion	insertion	insertion
Length	L*^a^*	162	63	128	72	331	58	132	135	132
	E*^b^*	100–200	0–100	100–200	0–100	300–400	0–100	100–200	100–200	100–200
Consistency rate		13/14	13/14	12/14	14/14	13/14	14/14	14/14	13/14	13/14
discrepant cultivar		Dancy tangerine	Pummelo	Murcott tangor, Pummelo		Pummelo			Dancy tangerine	Pummelo
Satsuma mandarin	L	0/0	0/0	0/0	0/0	0/1	0/0	0/1	0/0	0/0
	E	0/0	0/0	0/0	0/0	0/1	0/0	0/1	0/0	0/0
Kunenbo	L	0/1	0/0	0/0	0/1	0/1	0/0	0/1	0/1	0/1
	E	0/1	0/0	0/0	0/1	0/1	0/0	0/1	0/1	0/1
Kishu mikan	L	0/1	0/0	0/0	0/1	0/0	0/0	1/1	0/0	0/0
	E	0/1	0/0	0/0	0/1	0/0	0/0	1/1	0/0	0/0
Mediterranean mandarin	L	1/1	0/0	1/1	0/0	0/0	0/1	1/1	0/1	0/0
	E	1/1	0/0	1/1	0/0	0/0	0/1	1/1	0/1	0/0
Ponkan	L	0/1	0/0	0/0	0/0	0/0	0/1	1/1	0/1	0/1
	E	0/1	0/0	0/0	0/0	0/0	0/1	1/1	0/1	0/1
Dancy tangerine	L	1/1	0/0	0/0	1/1	0/0	0/0	1/1	0/0	0/0
	E	0/1	0/0	0/0	1/1	0/0	0/0	1/1	0/1	0/0
King mandarin	L	1/1	0/0	1/1	1/1	0/0	0/0	0/1	1/1	0/1
	E	1/1	0/0	1/1	1/1	0/0	0/0	0/1	1/1	0/1
Murcott tangor	L	0/1	0/0	1/1	1/1	0/0	0/1	1/1	1/1	0/1
	E	0/1	0/0	0/1	1/1	0/0	0/1	1/1	1/1	0/1
Iyokan	L	0/1	0/0	0/0	1/1	0/1	0/0	0/1	1/1	0/0
	E	0/1	0/0	0/0	1/1	0/1	0/0	0/1	1/1	0/0
Sweet orange	L	0/0	0/1	0/0	0/0	0/1	0/1	0/1	1/1	0/1
	E	0/0	0/1	0/0	0/0	0/1	0/1	0/1	1/1	0/1
Hassaku	L	0/0	0/1	0/0	0/0	0/1	0/1	0/1	0/1	0/1
	E	0/0	0/1	0/0	0/0	0/1	0/1	0/1	0/1	0/1
Grapefruit	L	0/0	1/1	0/0	0/0	1/1	0/1	0/1	1/1	1/1
	E	0/0	1/1	0/0	0/0	1/1	0/1	0/1	1/1	1/1
Pummelo	L	0/0	0/1	0/1	1/1	0/1	1/1	0/0	1/1	0/1
	E	0/0	1/1	0/0	1/1	1/1	1/1	0/0	1/1	1/1
Hyuganatsu	L	0/0	0/0	0/0	1/1	0/1	0/1	0/1	1/1	0/1
	E	0/0	0/0	0/0	1/1	0/1	0/1	0/1	1/1	0/1

*^a^* L indicates LAYLA. *^b^* E indicates Electrophoresis.
