# Supplementary material for: LAYLA: development of a comprehensive and cross-sample program for detecting structural variants and its application to citrus cultivars
Source: Breed Sci. 2025 Oct 28;75(5):442–54. doi: 10.1270/jsbbs.25020 (PMC13129578; doi:10.1270/jsbbs.25020)
Supplement: Supplementary file 1 — Supplemental Figures [file 75_442_s1.pdf]

A Chromosome 1

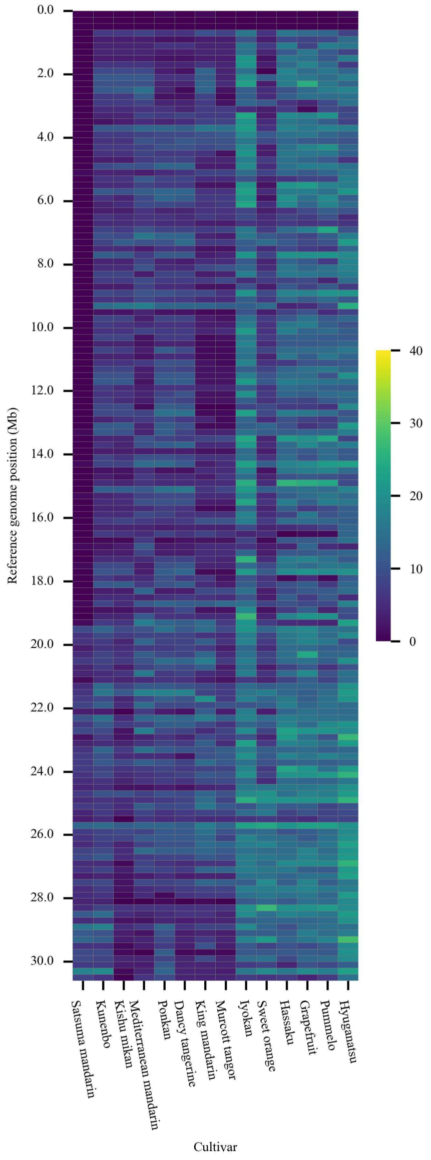

B

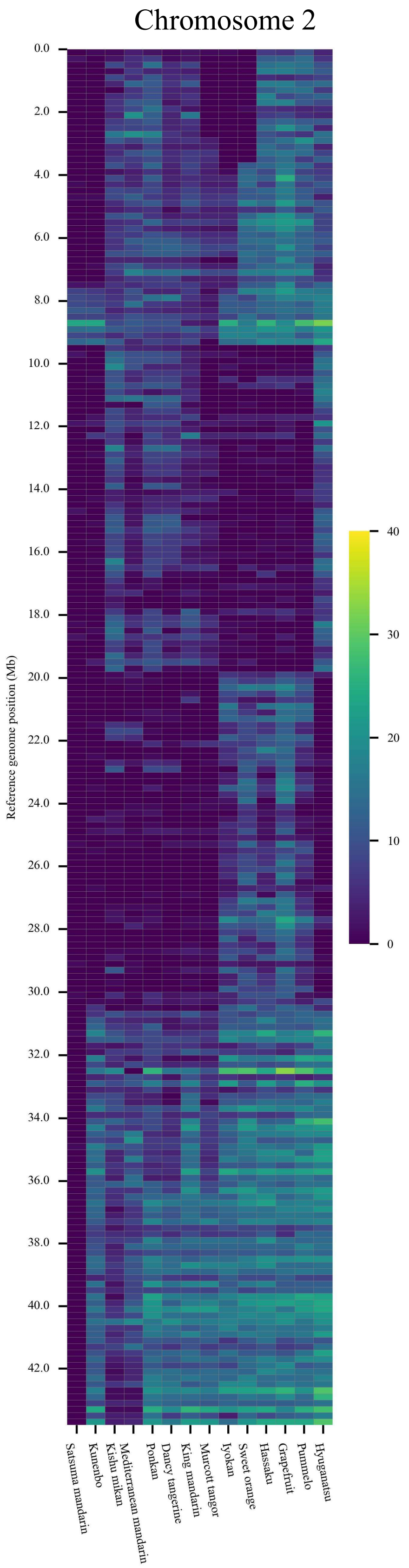

Chromosome 2
Reference genome position (Mb)
0.0
2.0
4.0
6.0
8.0
10.0
12.0
14.0
16.0
18.0
20.0
22.0
24.0
26.0
28.0
30.0
32.0
34.0
36.0
38.0
40.0
42.0
Satsuma mandarin
Kunenbo
Kishu mikan
Mediterranean mandarin
Ponkan
Dancy tangerine
King mandarin
Murcott tangor
Iyokan
Sweet orange
Hassaku
Grapefruit
Pummelo
Hyuganatsu
Cultivar
40
30
20
10
0

C Chromosome 3

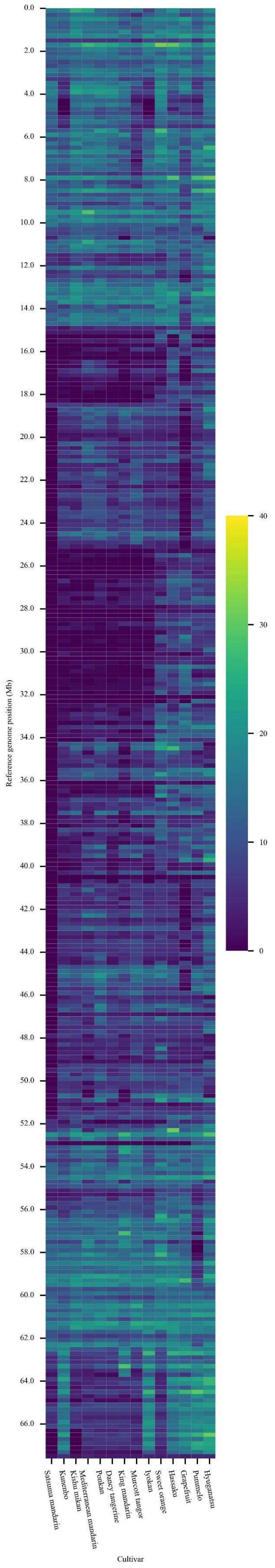

D Chromosome 4

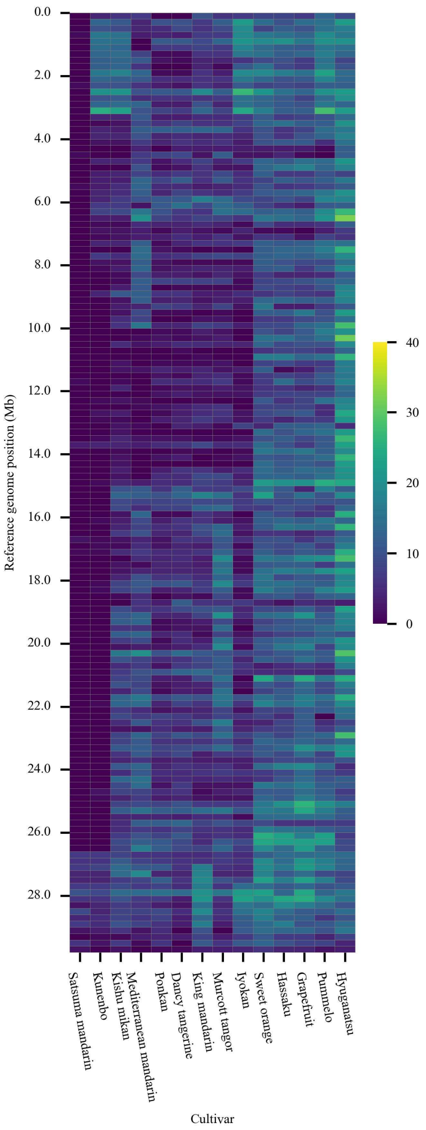

E

Chromosome 5

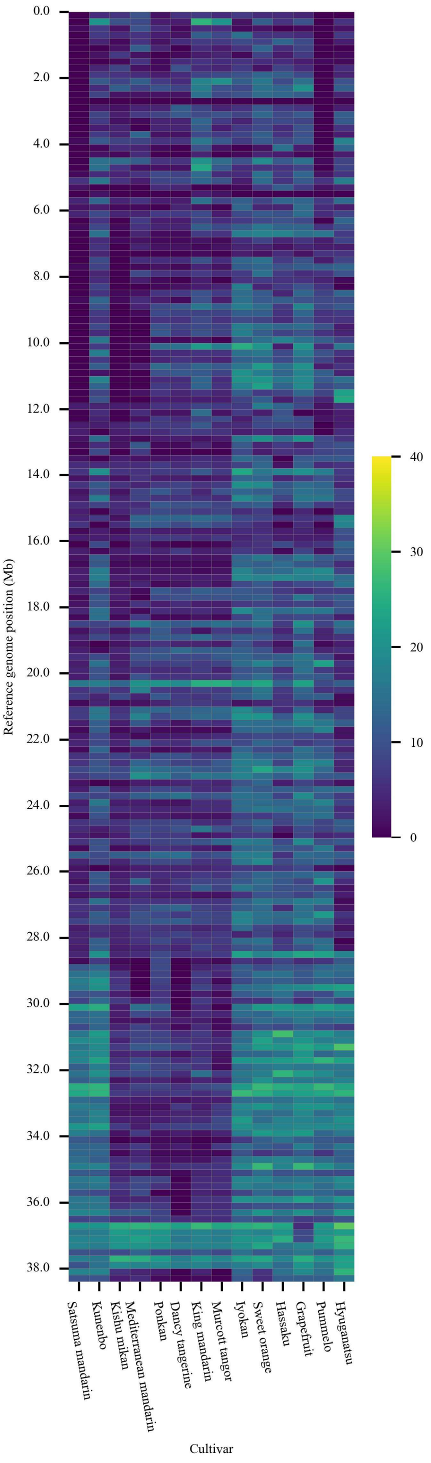

F

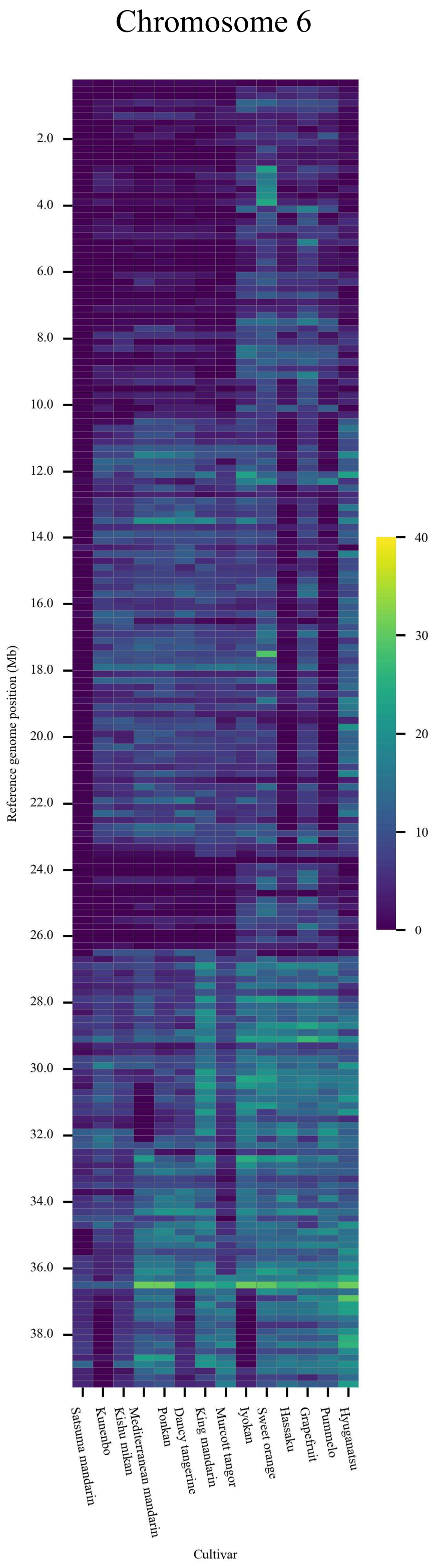

Chromosome 6
Reference genome position (Mb)
2.0
4.0
6.0
8.0
10.0
12.0
14.0
16.0
18.0
20.0
22.0
24.0
26.0
28.0
30.0
32.0
34.0
36.0
38.0
40
30
20
10
0
Satsuma mandarin
Kunenbo
Kishu mikan
Mediterranean mandarin
Ponkan
Dancy tangerine
King mandarin
Murcott tangor
Iyokan
Sweet orange
Hassaku
Grapefruit
Pummelo
Hyuganatsu
Cultivar

G

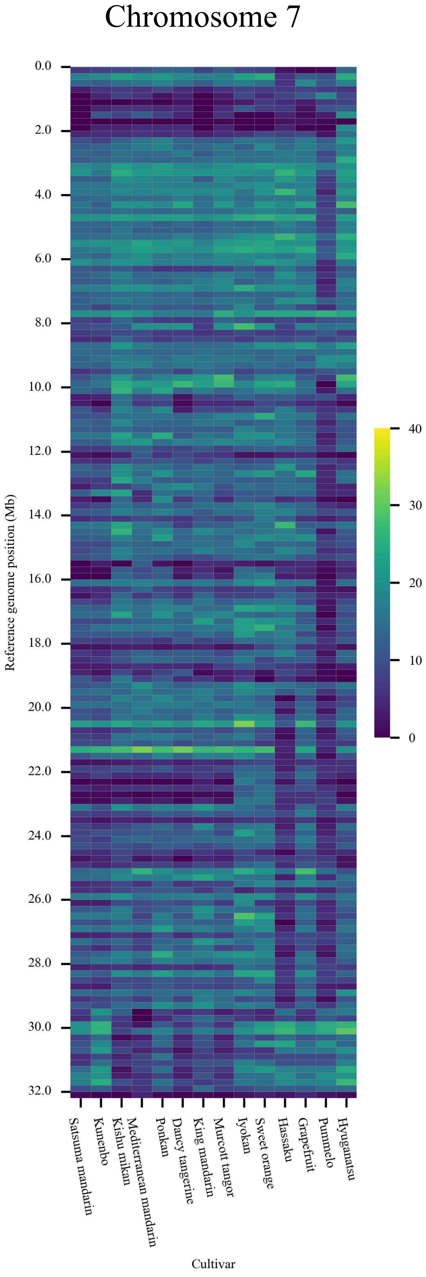

Chromosome 7
0.0
2.0
4.0
6.0
8.0
10.0
12.0
14.0
16.0
18.0
20.0
22.0
24.0
26.0
28.0
30.0
32.0
Reference genome position (Mb)
40
30
20
10
0
Satsuma mandarin
Kunenbo
Kishu mikan
Mediterranean mandarin
Ponkan
Dancy tangerine
King mandarin
Murcott tangor
Iyokan
Sweet orange
Hassaku
Grapefruit
Pummelo
Hyuganatsu
Cultivar

H Chromosome 8

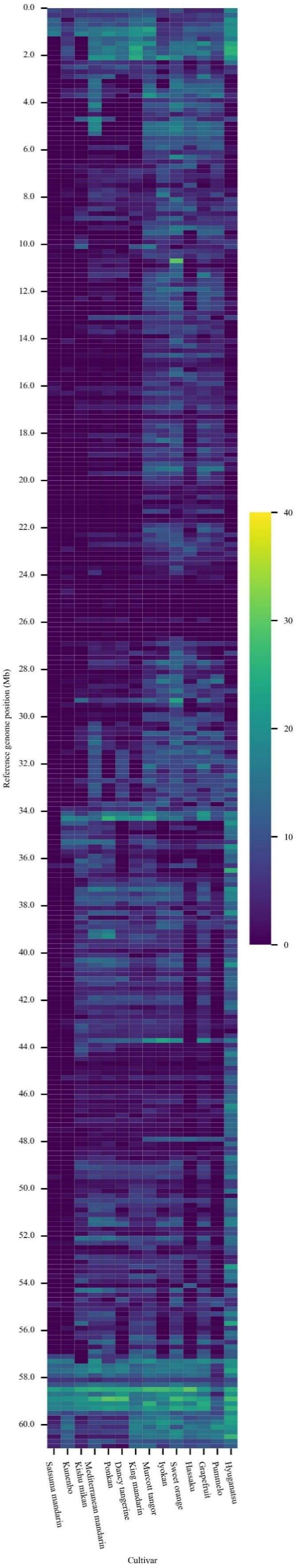

I

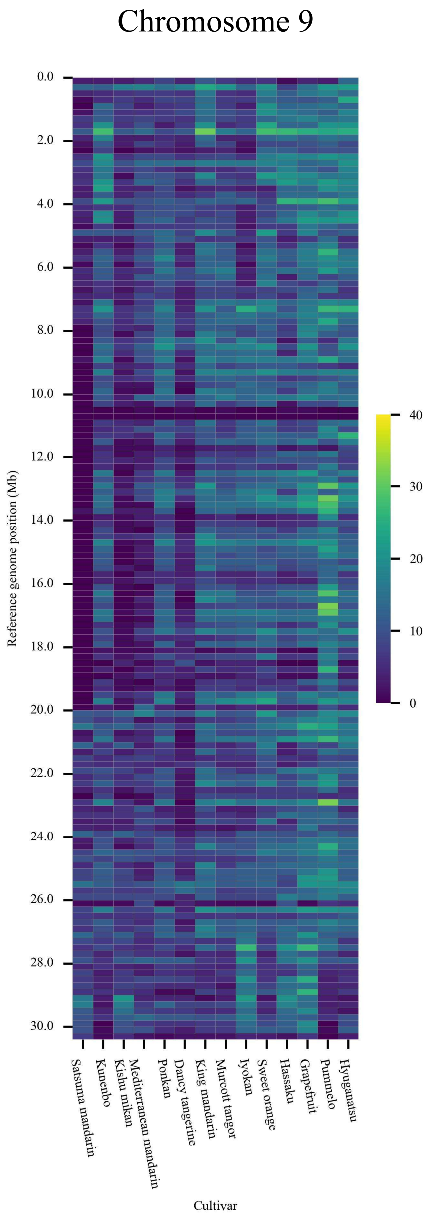
Chromosome 9
Reference genome position (Mb)
0.0
2.0
4.0
6.0
8.0
10.0
12.0
14.0
16.0
18.0
20.0
22.0
24.0
26.0
28.0
30.0
Satsuma mandarin
Kunenbo
Kishu mikan
Mediterranean mandarin
Ponkan
Dancy tangerine
King mandarin
Murcott tangor
Iyokan
Sweet orange
Hassaku
Grapefruit
Pummelo
Hyuganatsu
Cultivar
40
30
20
10
0

Supplemental Fig. 1. Heatmap of structural variant counts per cultivar across the reference genome. The reference genome was divided into 200 kbp windows. Colors represent structural variant density (dark blue = low, green = medium, yellow = high) with a fixed scale of 0–40 counts. Panels A–I correspond to chromosomes 1–9, respectively. Vertical length differences reflect variations in chromosome lengths, except for Figures C (chromosome 3) and H (chromosome 8), which were adjusted to fit the page layout.

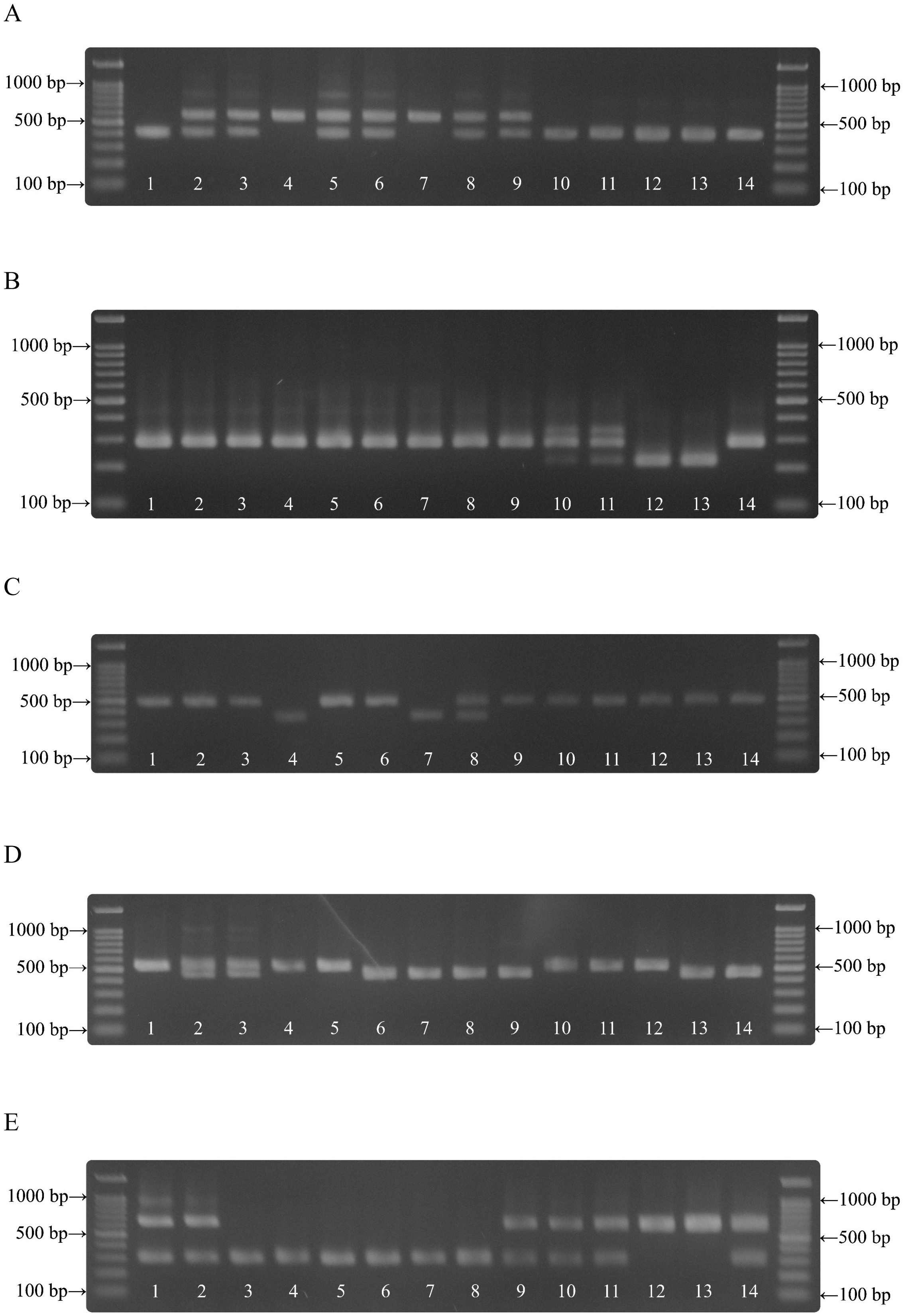
A
1000 bp→
500 bp→
100 bp→
1 2 3 4 5 6 7 8 9 10 11 12 13 14
←1000 bp
←500 bp
←100 bp
B
1000 bp→
500 bp→
100 bp→
1 2 3 4 5 6 7 8 9 10 11 12 13 14
←1000 bp
←500 bp
←100 bp
C
1000 bp→
500 bp→
100 bp→
1 2 3 4 5 6 7 8 9 10 11 12 13 14
←1000 bp
←500 bp
←100 bp
D
1000 bp→
500 bp→
100 bp→
1 2 3 4 5 6 7 8 9 10 11 12 13 14
←1000 bp
←500 bp
←100 bp
E
1000 bp→
500 bp→
100 bp→
1 2 3 4 5 6 7 8 9 10 11 12 13 14
←1000 bp
←500 bp
←100 bp

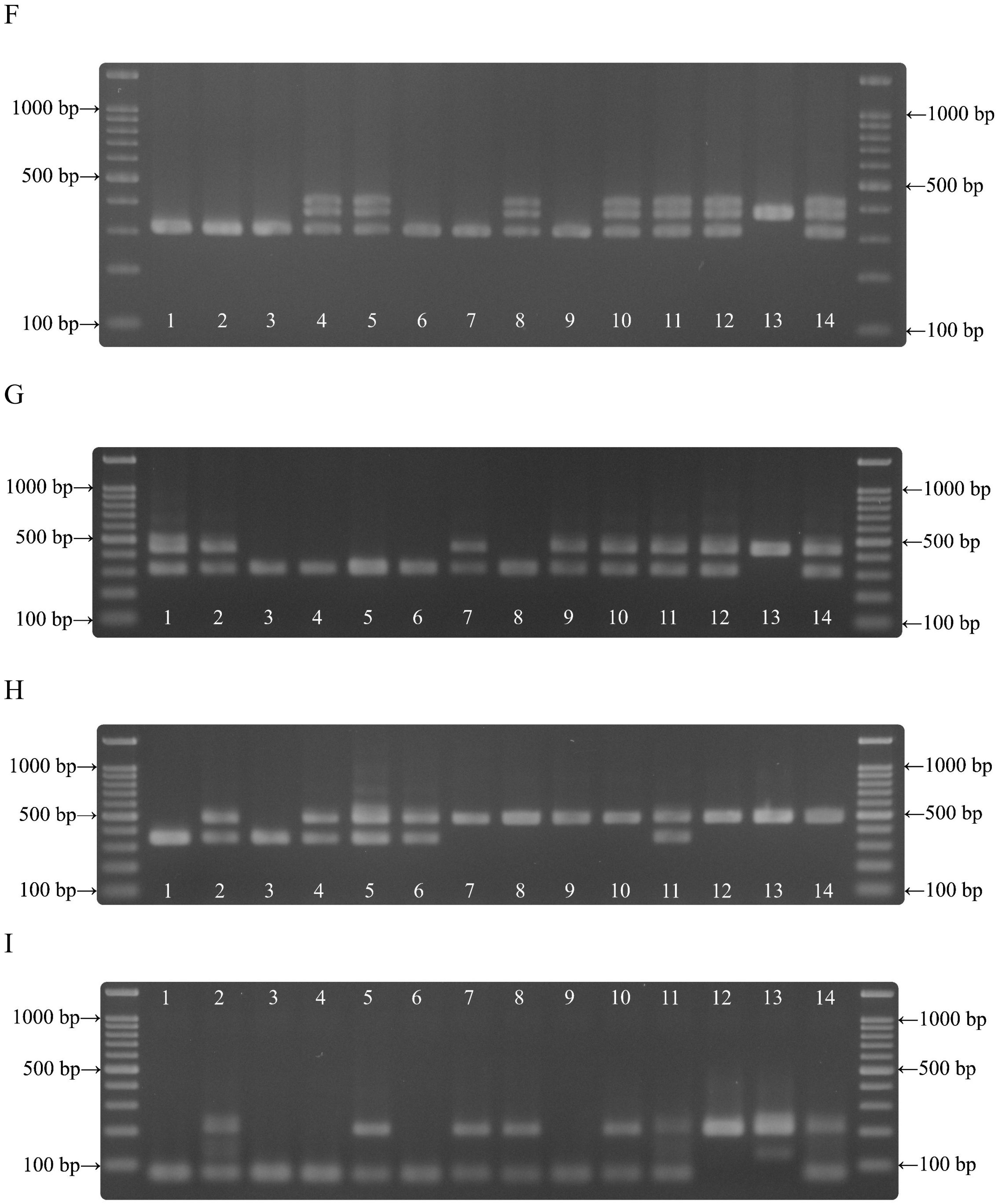

Supplemental Fig. 2. The electrophoresis results of the markers designed based on LAYLA's results.

This figure shows the electrophoresis results of markers designed based on the LAYLA analysis. Among the 16 lanes, both ends contain ladders. The samples loaded in each lane are as follows: Lane 1: Satsuma mandarin, Lane 2: Kunenbo, Lane 3: Kishu mikan, Lane 4: Mediterranean mandarin, Lane 5: Ponkan, Lane 6: Dancy tangerine, Lane 7: King mandarin, Lane 8: Murcott tangor, Lane 9: Iyokan, Lane 10: Sweet orange, Lane 11:

Hassaku, Lane 12: Grapefruit, Lane 13: Pummelo, Lane 14: Hyuganatsu. The lower end of the ladder corresponds to 100 bp, with bands spaced at 100 bp intervals up to 1000 bp (the 500 bp band appears more intense than others), and the upper end corresponds to 1500 bp. Electrophoresis results of markers 1 to 9 are shown in (A) to (I), respectively.
