# Supplementary material for: LAYLA: development of a comprehensive and cross-sample program for detecting structural variants and its application to citrus cultivars
Source: Breed Sci. 2025 Oct 28;75(5):442–54. doi: 10.1270/jsbbs.25020 (PMC13129578; doi:10.1270/jsbbs.25020)
Supplement: Supplementary file 2 — Supplemental Tables [file 75_442_s2.pdf]

doi: 10.1270/jsbbs.25020

Supplemental Table 1. Basic read statistics

The table presents the basic statistics of the long-read data used for the analysis.

Sample	Satsuma mandarin	Kunenbo	Kishu mikan	Mediterranean mandarin	Ponkan
Mean read length (bp)	21,144.60	21,167.30	22,832.30	22,933.10	22,476.10
Mean read quality	25.9	26	25.5	25	25.5
Median read length (bp)	20,281	20,458	22,062	21,933	21,615
Median read quality (bp)	28.8	29.1	28.1	27.2	28.0
Number of reads	729,961	446,899	487,706	360,406	344,866
Read length N50 (bp)	21,657	21,250	23,215	23,585	22,722
STDEV read length (bp)	5,244.30	4,208.20	4,924.40	5,946.50	4,780.10
Total bases (bp)	15,434,762,341	9,459,642,704	11,135,443,762	8,265,216,075	7,751,258,472

Sample	Dancy tangerine	King mandarin	Murcott tangor	Iyokan	Sweet orange
Mean read length (bp)	24,469.90	21,377.60	23,513.90	22,522.20	23,322.50
Mean read quality	25	25.8	25.3	25.3	25.4
Median read length (bp)	23,600	20,709	22,596	21,631	22,415
Median read quality (bp)	27	28.6	27.7	27.6	27.8
Number of reads	166,960	301,612	203,924	307,528	247,192
Read length N50 (bp)	25,196	21,530	24,064	23,178	23,845
STDEV read length (bp)	5,999.40	4,102.80	5,584.70	5,588.90	5,491.20
Total bases (bp)	4,085,486,816	6,447,730,137	4,795,051,263	6,926,207,603	5,765,137,427

Sample	Hassaku	Grapefruit	Pummelo	Hyuganatsu
Mean read length (bp)	24,174	24,668	23,949	20,310
Mean read quality	25.2	24.8	25.1	25.9
Median read length (bp)	23,243	23,697	23,072	19,560
Median read quality (bp)	27.4	26.8	27.2	28.8
Number of reads	248,777	197,330	197,699	322,924
Read length N50 (bp)	24,830	25,420	24,567	20,632
STDEV read length (bp)	5,888.80	6,314.40	5,795.30	4,465.60
Total bases (bp)	6,013,936,378	4,867,728,280	4,734,875,458	6,558,877,385

Supplemental Table 2. List of major options in LAYLA program. The table presents the options and configuration items that can be specified when executing LAYLA.

Option	Definition
**-i**	Input directory path (containing long-read sequence data files)
**-o**	Output directory path
**-g**	Path of a reference genome file
**-q**	Threshold for mapping quality of reads to be filtered
**--min_svlen**	Minimum length of structural variants to be detected
**--anno**	Path to gene annotation file
**--thre_abs**	Threshold for filtering STDEV_POS
**--seq_len**	Length of upstream and downstream sequences of structural variants to be extracted
**--region**	Length of the upstream region of a gene to be considered
**-M**	Constant used by sniffles for calculating the threshold to identify structural variants at the same position

Supplemental Table 3. The result of the structural variant detection by LAYLA in 14 citrus cultivars.

The table shows the structural variants (SVs) detected by LAYLA using long-read sequencing data from 14 citrus cultivars and the reference genome of Satsuma mandarin.

POS	TYPE	Reliability	LENGTH	STDEV_POS	STDEV_LEN	Satsuma mandarin	Kunenbo	Kishu Mikan	Mediterranean Mandarin	Ponkan	Dancy Tangerine	King Mandarin	Murcott tangor	Iyokan	Sweet orange	Hassaku	Grapefruit	Pummelo	Hyuganatsu
Chromosome1-11103	DUP	PRECISE	4228281	0	0	0/0	0/0	0/0	0/0	0/1	0/0	0/1	0/0	0/0	0/0	0/0	0/0	0/0	0/1
Chromosome1-602629	BND	PRECISE	0	0	0	0/0	0/0	0/1	0/1	0/0	0/0	0/1	0/1	0/0	0/0	0/1	0/0	0/0	0/0
Chromosome1-603089	DEL	PRECISE	129	0	0	0/0	0/0	0/0	0/0	0/0	0/0	0/0	0/0	0/0	0/0	0/0	0/0	0/0	0/1
Chromosome1-625082	DEL	PRECISE	192	0	0	0/0	0/0	0/0	0/0	0/0	0/0	0/0	0/0	0/1	0/1	0/1	0/1	0/1	0/0
Chromosome1-627207	INS	PRECISE	700	0	0.837	0/0	0/0	0/0	0/0	0/0	0/0	0/0	0/0	0/1	0/1	0/1	0/1	0/1	0/0
Chromosome1-638980	DEL	PRECISE	59	0	0	0/0	0/1	0/1	1/1	0/1	0/1	1/1	0/1	0/1	0/0	0/1	0/0	0/0	0/1
Chromosome1-648231	DEL	PRECISE	110	0	0	0/0	0/0	0/0	0/0	0/0	0/0	0/0	0/0	0/1	0/1	0/1	0/1	0/1	0/0
Chromosome1-649977	INS	PRECISE	1608	0	0	0/0	0/0	0/0	0/0	0/0	0/0	0/0	0/0	1/1	0/0	0/1	0/0	0/0	0/0
Chromosome1-650651	DEL	PRECISE	992	0	0	0/0	0/1	0/1	1/1	0/1	0/1	1/1	1/1	1/1	0/0	1/1	0/1	0/0	0/1
Chromosome1-660398	DEL	PRECISE	4312	0.577	0.577	0/0	0/0	0/0	0/0	0/1	0/1	0/0	0/0	0/0	0/0	0/0	0/0	0/0	0/0
Chromosome1-660952	DEL	PRECISE	2453	0	0	0/0	0/0	0/0	0/0	0/0	0/0	0/0	0/0	0/0	0/0	0/0	0/0	0/0	0/1
Chromosome1-664736	INV	PRECISE	19399429	0	0	0/0	0/0	0/0	0/0	0/0	0/0	0/1	0/0	0/0	0/0	0/0	0/0	0/0	0/0
Chromosome1-667494	INS	PRECISE	131	0	0.5	0/0	0/0	0/0	0/0	0/0	0/0	0/0	0/0	1/1	0/0	0/1	0/0	0/0	0/0
Chromosome1-668518	DEL	PRECISE	65	0	0	0/0	0/0	0/0	0/0	0/0	0/0	0/0	0/0	1/1	0/0	0/1	0/1	0/0	0/0
Chromosome1-677161	DEL	PRECISE	209	0	0	0/0	0/0	0/0	0/0	0/0	0/0	0/0	0/0	0/1	0/1	0/1	0/1	0/1	0/0
Chromosome1-687529	INV	PRECISE	302	0	0	0/0	0/0	0/0	0/0	0/1	0/1	0/0	0/0	0/1	0/0	0/0	0/0	0/0	0/0
Chromosome1-701983	INS	PRECISE	1550	0	0	0/0	0/0	0/0	0/0	0/0	0/0	0/0	0/0	0/0	0/0	0/0	0/0	0/1	0/0
Chromosome1-702233	INS	PRECISE	369	0	0.674	0/0	0/1	0/1	1/1	0/0	0/0	1/1	0/1	0/1	0/1	1/1	0/1	0/1	0/1
Chromosome1-707099	INS	PRECISE	3586	0	0	0/0	0/0	0/0	0/0	0/0	0/0	0/0	0/0	0/0	0/0	0/0	0/0	0/0	1/1
Chromosome1-714581	INS	PRECISE	967	0	0	0/0	0/0	0/0	0/0	0/0	0/0	0/0	0/0	0/0	0/0	0/0	0/0	0/0	0/1
Chromosome1-735570	DEL	PRECISE	56	0	0	0/0	0/1	0/1	1/1	0/0	0/0	1/1	1/1	0/0	0/0	0/0	0/0	0/0	0/0
Chromosome1-749106	DEL	PRECISE	86	0	0	0/0	0/0	0/0	0/0	0/0	0/0	0/0	0/0	0/1	0/0	0/1	0/1	0/0	0/0
Chromosome1-759074	INS	PRECISE	126	0	0	0/0	0/0	0/0	0/0	0/0	0/0	0/0	0/0	0/1	0/0	0/1	0/1	0/1	0/0
Chromosome1-766386	DEL	PRECISE	150	0	0	0/0	0/0	0/0	0/0	0/0	0/0	0/0	0/0	1/1	0/0	0/1	0/0	0/0	0/0
Chromosome1-769199	DEL	PRECISE	718	0	0	0/0	0/1	0/1	1/1	0/1	0/1	1/1	0/1	0/1	0/0	1/1	0/0	0/0	0/0
Chromosome1-770249	DEL	PRECISE	111	0	0	0/0	0/0	0/0	0/0	0/0	0/0	0/0	0/0	0/0	0/0	0/0	0/1	0/1	0/0
Chromosome1-773949	INS	PRECISE	121	0	0	0/0	0/0	0/0	0/0	0/0	0/0	0/0	0/0	0/0	0/0	0/0	0/0	0/1	0/0
Chromosome1-774964	INS	PRECISE	333	0	0	0/0	0/0	0/0	0/0	0/0	0/0	0/0	0/0	0/0	0/0	0/0	0/0	0/1	0/0
Chromosome1-777128	DEL	PRECISE	703	0	0	0/0	0/1	0/1	1/1	0/1	0/1	1/1	0/1	1/1	0/1	1/1	0/1	0/1	0/1
Chromosome1-782394	INS	PRECISE	122	0	0	0/0	0/0	0/0	0/0	0/0	0/0	0/0	0/0	0/0	0/0	0/0	0/1	0/0	0/0
Chromosome1-786320	DEL	PRECISE	114	0	0	0/0	0/0	0/0	0/0	0/0	0/0	0/0	0/0	0/0	0/0	0/0	0/1	0/1	0/0
Chromosome1-790885	BND	PRECISE	0	0	0	0/0	0/0	0/0	0/0	0/0	0/0	0/1	0/0	0/0	0/0	0/0	0/0	0/0	0/0
Chromosome1-790886	BND	PRECISE	0	0	0	0/0	0/1	0/0	0/1	0/0	0/0	0/1	0/1	0/0	0/0	0/0	0/0	0/0	0/0
Chromosome1-797834	DEL	PRECISE	50	0	0	0/0	0/0	0/0	0/0	0/0	0/0	0/0	0/0	0/0	0/0	0/0	0/1	0/1	0/0
Chromosome1-809478	INS	PRECISE	211	0	0	0/0	0/0	0/0	0/0	0/0	0/0	0/0	0/0	0/0	0/0	0/0	0/0	0/0	0/1
Chromosome1-813759	INS	PRECISE	4631	0	0	0/0	0/0	0/0	0/0	0/0	0/0	0/0	0/0	0/0	0/0	0/0	0/0	0/0	0/1
Chromosome1-819503	INS	PRECISE	824	0	0.707	0/0	0/0	0/0	0/0	0/0	0/0	0/0	0/0	0/1	0/0	0/1	0/0	0/0	0/0
Chromosome1-820049	INS	PRECISE	2145	0	0	0/0	0/0	0/0	0/0	0/0	0/0	0/0	0/0	0/0	0/0	0/0	0/0	0/0	0/1
Chromosome1-825408	DEL	PRECISE	599	0	0	0/0	0/1	0/1	1/1	0/0	0/0	1/1	0/1	0/1	0/0	1/1	0/1	0/0	0/1
Chromosome1-834856	INS	PRECISE	503	0	0	0/0	0/0	0/0	0/0	0/0	0/0	0/0	0/0	0/0	0/0	0/0	0/0	0/0	0/1
Chromosome1-836175	INS	PRECISE	51	0	0	0/0	0/0	0/0	0/0	0/0	0/0	0/0	0/0	0/0	0/0	0/0	0/0	1/1	0/0
Chromosome1-836811	DEL	PRECISE	56	0	0	0/0	0/0	0/0	0/0	0/0	0/0	0/0	0/0	0/1	0/0	0/1	0/0	0/0	0/0
Chromosome1-853906	INS	PRECISE	165	0	0.5	0/0	0/0	0/0	0/0	0/0	0/0	0/0	0/0	0/1	0/0	0/1	0/1	0/1	0/0
Chromosome1-856651	DEL	PRECISE	167	0	0	0/0	0/0	0/0	0/0	0/0	0/0	0/0	0/0	0/0	0/0	0/0	0/0	0/0	0/1
Chromosome1-867561	INS	PRECISE	1003	0	0.707	0/0	0/0	0/0	0/0	0/0	0/0	0/0	0/0	0/0	0/0	0/0	0/1	0/1	0/0
Chromosome1-875688	INS	PRECISE	291	0	0	0/0	0/0	0/0	0/0	0/0	0/0	0/0	0/0	0/0	0/0	0/0	0/1	0/0	0/0
Chromosome1-882583	INS	PRECISE	137	0	0	0/0	0/0	0/0	0/0	0/0	0/0	0/0	0/0	0/0	0/0	0/0	0/0	0/0	0/1
Chromosome1-887641	INS	PRECISE	1982	0	0	0/0	0/0	0/0	0/0	0/0	0/0	0/0	0/0	0/0	0/0	0/0	0/0	0/0	0/1
Chromosome1-900191	DEL	PRECISE	52	0	0	0/0	0/0	0/0	0/0	0/0	0/0	0/0	0/0	0/0	0/0	0/0	0/0	0/1	0/0
Chromosome1-914689	DEL	PRECISE	145	0	0	0/0	0/0	0/0	0/0	0/0	0/0	0/0	0/0	0/1	0/0	0/1	0/1	0/1	0/0
Chromosome1-917121	INS	PRECISE	211	0	0	0/0	0/0	0/0	0/0	0/0	0/0	0/0	0/0	0/0	0/1	0/0	0/0	0/0	0/0
Chromosome1-918088	INS	PRECISE	160	0	0	0/0	0/0	0/0	0/0	0/0	0/0	0/0	0/0	0/0	0/0	0/0	0/0	0/0	0/1
Chromosome1-918406	DEL	PRECISE	211	0	0	0/0	0/1	0/1	1/1	0/1	0/1	1/1	0/1	1/1	0/1	1/1	0/1	0/1	0/1
Chromosome1-923813	INS	PRECISE	2261	0	0	0/0	0/0	0/0	0/0	0/0	0/0	0/0	0/0	0/1	0/0	0/1	0/0	0/0	0/0
Chromosome1-923884	INS	PRECISE	12529	0	0	0/0	0/0	0/0	0/0	0/0	0/0	0/0	0/0	0/0	0/0	0/0	0/0	0/0	0/1
Chromosome1-923890	BND	PRECISE	0	0	0	0/0	0/0	0/0	0/0	0/0	0/0	0/0	0/0	0/0	0/0	0/0	0/0	0/0	0/1
Chromosome1-945601	DEL	PRECISE	1008	0	0	0/0	0/1	0/1	1/1	0/1	0/1	1/1	0/1	1/1	0/1	1/1	0/1	0/1	0/1
Chromosome1-955271	DEL	PRECISE	54	0	0	0/0	0/0	0/0	0/0	0/0	0/0	0/0	0/0	0/0	0/0	0/0	0/0	0/0	0/1
Chromosome1-980111	DEL	PRECISE	877	0	0	0/0	0/0	0/0	0/0	0/0	0/0	0/0	0/0	1/1	0/0	1/1	0/0	0/1	0/0
Chromosome1-985022	DEL	PRECISE	248	0	0	0/0	0/0	0/0	0/0	0/0	0/0	0/0	0/0	0/1	0/0	0/0	0/1	0/1	0/0
Chromosome1-1010177	INS	PRECISE	211	0	0	0/0	0/0	0/0	0/0	0/1	0/1	0/0	0/0	0/1	0/0	0/0	0/0	0/0	0/0
Chromosome1-1012226	INS	PRECISE	455	0	0	0/0	0/0	0/0	0/0	0/0	0/0	0/0	0/0	0/0	0/1	0/0	0/0	0/0	0/0
Chromosome1-1013321	INS	PRECISE	830	0	0	0/0	0/0	0/0	0/0	0/0	0/0	0/0	0/0	0/0	0/0	0/0	0/0	0/0	0/1
Chromosome1-1020434	INS	PRECISE	62	0	0	0/0	0/0	0/0	0/0	0/1	0/1	0/0	0/0	1/1	0/0	0/0	0/0	0/0	0/0
Chromosome1-1025108	DEL	PRECISE	66	0	0	0/0	0/0	0/0	0/0	0/0	0/0	0/0	0/0	0/1	0/0	0/1	0/1	0/1	0/0
Chromosome1-1036301	DEL	PRECISE	90	0	0	0/0	0/0	0/0	0/0	0/0	0/0	0/0	0/0	0/1	0/0	0/1	0/0	0/1	0/0
Chromosome1-1036330	INS	PRECISE	5550	0	0	0/0	0/0	0/0	0/0	0/0	0/0	0/0	0/0	0/0	1/1	0/0	0/0	0/0	0/0
Chromosome1-1038304	DEL	PRECISE	51	0	0	0/0	0/0	0/0	0/0	0/0	0/0	0/0	0/0	0/1	0/0	0/1	0/0	0/1	0/0
Chromosome1-1039098	INS	PRECISE	1943	0	0	0/0	0/0	0/0	0/0	0/0	0/0	0/0	0/0	0/0	0/0	0/0	0/1	0/0	0/0
Chromosome1-1044243	DEL	PRECISE	137	0	0	0/0	0/1	0/1	1/1	0/1	0/1	1/1	0/1	1/1	0/1	1/1	0/0	0/0	0/1

Chromosome1-1048998	DEL	PRECISE	811	0.894	0.894	0/0	0/0	0/0	0/0	0/0	0/0	0/0	0/0	0/0	0/0	0/1	0/1	0/1	0/1
Chromosome1-1066320	DEL	PRECISE	1015	0	0	0/0	0/1	0/1	1/1	0/1	1/1	1/1	0/1	1/1	0/1	1/1	0/0	0/0	0/1
Chromosome1-1074126	DEL	PRECISE	121	0	0	0/0	0/0	0/0	0/0	0/0	0/0	0/0	0/0	0/1	0/0	0/1	0/0	0/1	0/0
Chromosome1-1075837	DEL	PRECISE	706	0.577	0.577	0/0	0/0	0/0	0/0	0/0	0/0	0/0	0/0	0/1	0/0	0/1	0/0	0/1	0/0
Chromosome1-1076934	INS	PRECISE	621	0	0	0/0	0/0	0/0	0/0	0/0	0/0	0/0	0/0	0/0	0/0	0/0	0/0	0/0	0/1
Chromosome1-1080874	DEL	PRECISE	189	0	0	0/0	0/0	0/0	0/0	0/0	0/0	0/0	0/0	0/0	0/0	0/1	0/0	0/1	0/0
Chromosome1-1087764	DEL	PRECISE	212	0	0	0/0	0/0	0/0	0/0	0/0	0/0	0/0	0/0	0/0	0/0	0/1	0/0	0/0	0/1
Chromosome1-1089710	DEL	PRECISE	52	0	0	0/0	0/0	0/0	0/0	0/0	0/0	0/0	0/0	0/0	0/0	0/0	0/0	0/0	0/1
Chromosome1-1094889	INS	PRECISE	83	0	0	0/0	0/1	0/1	1/1	0/0	0/0	1/1	0/1	0/0	0/0	1/1	0/0	0/0	0/1
Chromosome1-1094889	INS	PRECISE	10698	0	0	0/0	0/0	0/0	0/0	0/0	0/0	0/0	0/0	0/0	0/1	0/0	0/0	0/0	0/0
Chromosome1-1096897	DEL	PRECISE	87	0	0	0/0	0/0	0/0	0/0	0/0	0/0	0/0	0/0	0/0	0/0	0/0	0/0	0/0	0/1
Chromosome1-1112213	DEL	PRECISE	61	0	0	0/0	0/0	0/0	0/0	0/0	0/0	0/0	0/0	0/0	0/0	0/0	0/0	0/1	0/0
Chromosome1-1120941	DEL	PRECISE	56	0	0	0/0	0/0	0/0	0/0	0/0	0/0	0/0	0/0	0/0	0/0	0/0	0/0	0/0	0/1
Chromosome1-1121091	INS	PRECISE	248	0	0	0/0	0/0	0/0	0/0	0/0	0/0	0/0	0/0	0/0	0/0	0/0	0/0	0/1	0/0
Chromosome1-1123866	DEL	PRECISE	138	0	0	0/0	0/0	0/0	0/0	0/0	0/0	0/0	0/0	0/0	0/0	0/0	0/1	0/1	0/0
Chromosome1-1129499	DEL	PRECISE	139	0	0	0/0	0/0	0/0	0/0	0/0	0/0	0/0	0/0	0/1	0/0	0/1	0/1	0/1	0/0
Chromosome1-1134523	DEL	PRECISE	173	0	0	0/0	0/0	0/0	0/0	0/0	0/0	0/0	0/0	0/1	0/0	0/1	0/0	0/1	0/0
Chromosome1-1137465	DEL	PRECISE	641	0	0	0/0	0/0	0/0	0/0	0/0	0/0	0/0	0/0	0/0	0/0	0/0	0/0	0/0	0/1
Chromosome1-1141370	INS	PRECISE	690	0	0	0/0	0/0	0/0	0/0	0/0	0/0	0/0	0/0	0/0	0/0	0/0	0/0	0/0	0/1
Chromosome1-1146610	DEL	PRECISE	251	0	0	0/0	0/0	0/0	0/0	0/0	0/0	0/0	0/0	0/0	0/0	0/0	0/0	0/0	0/1
Chromosome1-1147930	DEL	PRECISE	172	0	0	0/0	0/0	0/0	0/0	0/0	0/0	0/0	0/0	0/0	0/0	0/0	0/0	0/1	0/0
Chromosome1-1153935	INS	PRECISE	74	0	0	0/0	0/0	0/0	0/0	0/0	0/0	0/0	0/0	0/1	0/0	0/1	0/0	0/1	0/0
Chromosome1-1158034	DEL	PRECISE	60	0	0	0/0	0/0	0/0	0/0	0/0	0/0	0/0	0/0	0/0	0/0	0/0	0/1	0/1	0/0
Chromosome1-1162320	INS	PRECISE	55	0	0	0/0	0/1	0/1	1/1	0/1	0/1	1/1	0/1	1/1	0/1	1/1	0/1	0/0	0/0
Chromosome1-1166644	DEL	PRECISE	627	0	0	0/0	0/0	0/0	0/0	0/1	0/1	0/0	0/0	0/1	0/0	0/0	0/0	0/0	0/0
Chromosome1-1169418	INS	PRECISE	4681	0	0	0/0	0/0	0/0	0/0	0/0	0/0	0/0	0/0	0/0	0/0	0/0	0/0	0/0	0/1
Chromosome1-1169871	INS	PRECISE	200	0	0	0/0	0/0	0/0	0/0	0/0	0/0	0/0	0/0	0/0	0/0	0/0	0/0	0/0	0/1
Chromosome1-1175063	DEL	PRECISE	137	0.577	0.577	0/0	0/0	0/0	0/0	0/0	0/0	0/0	0/0	0/1	0/0	0/1	0/0	0/1	0/0
Chromosome1-1189855	INS	PRECISE	4915	0	0	0/0	0/0	0/0	0/0	0/0	0/0	0/0	0/0	0/0	0/0	0/0	0/0	0/0	0/1
Chromosome1-1190439	INS	PRECISE	67	0	0	0/0	0/0	0/0	0/0	0/0	0/0	0/0	0/0	0/0	0/0	0/0	0/0	0/1	0/0
Chromosome1-1192566	INS	PRECISE	1190	0	0	0/0	0/0	0/0	0/0	0/0	0/0	0/0	0/0	0/0	0/0	0/1	0/0	0/0	0/0
Chromosome1-1196054	INS	PRECISE	9969	0	0	0/0	0/0	0/0	0/0	0/0	0/0	0/0	0/0	0/1	0/0	0/0	0/0	0/0	0/0
Chromosome1-1196688	INS	PRECISE	1764	0	0	0/0	0/0	0/0	0/0	0/0	0/0	0/0	0/0	0/0	0/1	0/0	0/0	0/0	0/0
Chromosome1-1196857	INS	PRECISE	6131	0	0	0/0	0/0	0/0	0/0	0/0	0/0	0/0	0/0	0/0	0/0	0/0	1/1	0/0	0/0
Chromosome1-1200436	INS	PRECISE	649	0	0	0/0	0/0	0/0	0/0	0/0	0/0	0/0	0/0	0/1	0/0	0/0	0/0	0/0	0/0
Chromosome1-1202892	INS	PRECISE	1604	0	0	0/0	0/0	0/0	0/0	0/0	0/0	0/0	0/0	0/0	0/1	0/0	0/0	0/0	0/0
Chromosome1-1210683	INS	PRECISE	5939	0	0	0/0	0/0	0/0	0/0	0/0	0/0	0/0	0/0	0/0	0/0	0/0	0/0	0/1	0/0
Chromosome1-1218211	DEL	PRECISE	67	0	0	0/0	0/0	0/0	0/0	0/0	0/0	0/0	0/0	0/0	0/0	0/0	0/0	0/1	0/0
Chromosome1-1234797	DEL	PRECISE	5100	0	0	0/0	0/0	0/0	0/0	0/0	0/0	0/0	0/0	0/0	0/0	0/0	0/1	0/0	0/0
Chromosome1-1240467	INS	PRECISE	201	0	0	0/0	0/0	0/0	0/0	0/0	0/0	0/0	0/0	0/0	0/0	0/0	0/1	0/0	0/0
Chromosome1-1244207	DEL	PRECISE	63	0	0	0/0	0/0	0/0	0/0	0/0	0/0	0/0	0/0	0/1	0/0	0/1	0/1	0/0	0/0
Chromosome1-1248917	INS	PRECISE	1553	0	0	0/0	0/0	0/0	0/0	0/0	0/0	0/0	0/0	0/0	0/0	0/0	0/1	0/0	0/0
Chromosome1-1254698	DEL	PRECISE	104	0	0	0/0	0/0	0/0	0/0	0/0	0/0	0/0	0/0	0/1	0/0	0/1	0/0	0/0	0/0
Chromosome1-1258124	INS	PRECISE	211	0	0.577	0/0	0/0	0/0	0/0	0/1	0/1	0/0	0/0	0/1	0/0	0/0	0/0	0/0	0/0
Chromosome1-1275750	INS	PRECISE	50	0.5	0	0/0	0/0	0/0	0/0	0/0	0/0	0/0	0/0	0/0	0/0	1/1	0/1	0/1	0/0
Chromosome1-1292466	INS	PRECISE	50	0	0	0/0	0/0	0/0	0/0	0/0	0/0	0/0	0/0	0/1	0/0	0/1	0/1	0/1	0/0
Chromosome1-1305678	DEL	PRECISE	1196	0	0	0/0	0/0	0/0	0/0	0/0	0/0	0/0	0/0	0/0	0/0	0/0	0/1	0/0	0/0
Chromosome1-1306492	INS	PRECISE	1336	0	0.548	0/0	0/1	0/1	1/1	0/0	0/0	1/1	0/1	0/0	0/0	0/0	0/0	0/0	0/0
Chromosome1-1310634	DEL	PRECISE	161	0	0	0/0	0/1	0/1	1/1	0/1	0/1	1/1	0/1	1/1	0/0	0/0	0/1	0/0	0/1
Chromosome1-1321412	INS	PRECISE	61	0	0.5	0/0	0/0	0/0	0/0	0/0	0/0	0/0	0/0	0/1	0/0	0/1	0/1	0/1	0/0
Chromosome1-1322324	DEL	PRECISE	101	0	0	0/0	0/0	0/0	0/0	0/0	0/0	0/0	0/0	0/1	0/0	0/0	0/0	0/0	0/0
Chromosome1-1323374	DEL	PRECISE	389	0	0	0/0	0/0	0/0	0/0	0/0	0/0	0/0	0/0	0/1	0/0	0/0	0/0	0/0	0/0
Chromosome1-1331609	DEL	PRECISE	83	0	0	0/0	0/0	0/0	0/0	0/0	0/0	0/0	0/0	0/0	0/0	0/0	0/0	0/1	0/0
Chromosome1-1331790	INS	PRECISE	7224	0	0	0/0	0/0	0/0	0/0	0/0	0/0	0/0	0/0	0/0	0/0	0/0	0/0	0/0	1/1
Chromosome1-1332883	INS	PRECISE	50	0	0.957	0/0	0/0	0/0	0/0	0/0	0/0	0/0	0/0	0/1	0/0	0/1	0/1	0/1	0/0
Chromosome1-1335771	INS	PRECISE	329	0	0	0/0	0/0	0/0	0/0	0/0	0/0	0/0	0/0	0/0	0/0	0/0	0/1	0/0	0/0
Chromosome1-1341229	INS	PRECISE	111	0	0	0/0	0/1	0/1	1/1	0/1	0/1	1/1	0/1	1/1	0/1	0/1	0/1	0/1	0/1
Chromosome1-1345264	INS	PRECISE	132	0	0	0/0	0/0	0/0	0/0	0/0	0/0	0/0	0/0	0/0	0/0	0/0	0/0	0/0	0/1
Chromosome1-1346185	BND	PRECISE	0	0	0	0/0	0/1	0/1	0/1	0/0	0/0	0/1	0/1	0/0	0/1	0/0	0/0	0/0	0/0
Chromosome1-1363094	DEL	PRECISE	426	0	0	0/0	0/0	0/0	0/0	0/0	0/0	0/0	0/0	0/0	0/0	0/0	0/0	0/1	0/0
Chromosome1-1366627	DEL	PRECISE	2063	0	0	0/0	0/0	0/0	0/0	0/0	0/0	0/0	0/0	0/1	0/0	0/1	0/0	0/1	0/0
Chromosome1-1379152	DEL	PRECISE	69	0	0	0/0	0/0	0/0	0/0	0/0	0/0	0/0	0/0	0/0	0/1	0/0	0/0	0/0	0/0
Chromosome1-1386474	INS	PRECISE	2323	0	0	0/0	0/0	0/0	0/0	0/0	0/0	0/0	0/0	0/0	0/0	0/0	0/0	0/0	0/1
Chromosome1-1388876	DEL	PRECISE	298	0	0	0/0	0/0	0/0	0/0	0/0	0/0	0/0	0/0	0/0	0/0	0/0	0/0	0/0	0/1
Chromosome1-1389927	INS	PRECISE	771	0	0.707	0/0	0/0	0/0	0/0	0/0	0/0	0/0	0/0	0/1	0/0	0/1	0/0	0/0	0/0
Chromosome1-1390351	INS	PRECISE	3339	0	0	0/0	0/0	0/0	0/0	0/0	0/0	0/0	0/0	0/0	0/0	0/0	0/0	0/0	1/1
Chromosome1-1391431	INS	PRECISE	53	0	0	0/0	0/0	0/0	0/0	0/0	0/0	0/0	0/0	0/1	0/0	0/1	0/1	0/0	0/0
Chromosome1-1393055	DEL	PRECISE	2488	0	0	0/0	0/0	0/0	0/0	0/0	0/0	0/0	0/0	0/1	0/0	0/1	0/1	0/1	0/1
Chromosome1-1398184	DEL	PRECISE	78	0	0	0/0	0/0	0/0	0/0	0/0	0/0	0/0	0/0	0/0	0/0	0/0	0/1	0/0	0/0
Chromosome1-1398697	INS	PRECISE	144	0	0	0/0	0/0	0/0	0/0	0/0	0/0	0/0	0/0	0/0	0/0	0/0	0/1	0/0	0/0
Chromosome1-1406095	INS	PRECISE	726	0	0	0/0	0/0	0/0	0/0	0/0	0/0	0/0	0/0	0/0	1/1	0/0	0/0	0/0	0/0
Chromosome1-1407534	DEL	PRECISE	3158	0	0	0/0	0/0	0/0	0/0	0/0	0/0	0/0	0/0	0/1	0/1	0/1	0/1	0/1	0/1
Chromosome1-1416387	DEL	PRECISE	95	0	0	0/0	0/0	0/0	0/0	0/0	0/0	0/0	0/0	0/1	0/0	0/1	0/1	0/1	0/0

Chromosome1-1420254	INS	PRECISE	52	0	0	0/0	0/0	0/0	0/0	0/0	0/0	0/0	0/0	0/0	0/0	0/0	0/1	0/0	0/0
Chromosome1-1423285	DEL	PRECISE	390	0	0	0/0	0/0	0/0	0/0	0/0	0/0	0/0	0/0	0/1	0/0	0/1	0/0	0/1	0/0
Chromosome1-1424254	DEL	PRECISE	159	0.577	0.577	0/0	0/0	0/0	0/0	0/0	0/0	0/0	0/0	0/1	0/0	0/1	0/0	0/1	0/0
Chromosome1-1437016	INS	PRECISE	446	0	0.816	0/0	0/0	0/0	0/0	0/0	0/0	0/0	0/0	0/1	0/0	0/1	0/1	0/1	0/0
Chromosome1-1441417	INS	PRECISE	830	0	0	0/0	0/0	0/0	0/0	0/0	0/0	0/0	0/0	0/0	0/0	0/0	0/1	0/0	0/0
Chromosome1-1441682	INS	PRECISE	58	0.894	0.548	0/0	0/0	0/0	0/0	0/0	0/0	0/0	0/0	0/1	0/0	0/1	0/1	0/1	0/1
Chromosome1-1451110	DEL	PRECISE	124	0.5	0	0/0	0/0	0/0	0/0	0/0	0/0	0/0	0/0	0/1	0/0	0/1	0/1	0/1	0/0
Chromosome1-1451425	INS	PRECISE	245	0	0.5	0/0	0/0	0/0	0/0	0/0	0/0	0/0	0/0	0/1	0/0	0/1	0/1	0/1	0/0
Chromosome1-1455339	INS	PRECISE	387	0	0	0/0	0/0	0/0	0/0	0/0	0/0	0/0	0/0	0/0	0/0	0/0	0/1	0/0	0/0
Chromosome1-1459051	INS	PRECISE	589	0	0	0/0	0/0	0/0	0/0	0/0	0/0	0/0	0/0	0/0	0/0	0/0	0/0	0/0	0/1
Chromosome1-1468204	INS	PRECISE	66	0	0	0/0	0/0	0/0	0/0	0/0	0/0	0/0	0/0	0/0	0/0	0/0	0/0	0/0	0/1
Chromosome1-1476912	INS	PRECISE	9689	0	0	0/0	0/0	0/0	0/0	0/0	0/0	0/0	0/0	0/0	0/0	0/0	0/0	0/0	1/1
Chromosome1-1496046	INS	PRECISE	136	0	0	0/0	0/0	0/0	0/0	0/0	0/0	0/0	0/0	0/0	0/0	0/0	0/0	0/0	0/1
Chromosome1-1509402	INS	PRECISE	2081	0	0	0/0	0/0	0/0	0/0	0/0	0/0	0/0	0/0	0/0	0/0	0/0	0/0	0/0	0/1
Chromosome1-1512051	DEL	PRECISE	539	0	0	0/0	0/0	0/0	0/0	0/0	0/0	0/0	0/0	0/0	0/0	0/0	0/0	0/0	0/1
Chromosome1-1521940	DEL	PRECISE	144	0	0	0/0	0/0	0/0	0/0	0/0	0/0	0/0	0/0	0/0	0/0	0/0	0/1	1/1	0/0
Chromosome1-1523840	INS	PRECISE	107	0	0	0/0	0/0	0/0	0/0	0/0	0/0	0/0	0/0	0/1	0/0	0/1	0/1	0/1	0/0
Chromosome1-1527625	INS	PRECISE	119	0	0	0/0	0/0	0/0	0/0	0/0	0/0	0/0	0/0	0/1	0/0	0/1	0/0	0/0	0/0
Chromosome1-1537849	DEL	PRECISE	281	0	0	0/0	0/1	0/1	1/1	0/1	0/1	1/1	0/1	0/1	0/0	0/0	0/0	0/0	0/0
Chromosome1-1542447	INS	PRECISE	1314	0	0	0/0	0/0	0/0	0/0	0/0	0/0	0/0	0/0	0/0	0/0	0/0	0/0	0/0	0/1
Chromosome1-1543852	DEL	PRECISE	871	0	0	0/0	0/0	0/0	0/0	0/0	0/0	0/0	0/0	0/1	0/0	1/1	0/1	1/1	0/1
Chromosome1-1551854	INS	PRECISE	889	0	0	0/0	0/0	0/0	0/0	0/0	0/0	0/0	0/0	0/0	0/0	0/0	0/0	0/0	0/1
Chromosome1-1554024	INS	PRECISE	72	0	0	0/0	0/0	0/0	0/0	0/0	0/0	0/0	0/0	0/0	0/0	0/0	0/0	0/0	0/1
Chromosome1-1556802	DEL	PRECISE	55	0	0	0/0	0/1	0/1	1/1	0/1	0/1	1/1	0/1	0/1	0/0	0/0	0/0	0/0	0/0
Chromosome1-1558663	DUP	PRECISE	8368	0	0	0/0	0/0	0/0	0/0	0/0	0/0	0/0	0/0	0/1	0/0	0/0	0/0	0/0	0/0
Chromosome1-1563181	INS	PRECISE	1554	0	0	0/0	0/0	0/0	0/0	0/0	0/0	0/0	0/0	0/0	0/0	0/0	0/0	0/0	0/1
Chromosome1-1564584	INS	PRECISE	7710	0	0	0/0	0/0	0/0	0/0	0/0	0/0	0/0	0/0	0/0	0/0	0/0	0/0	0/0	0/0
Chromosome1-1564713	DEL	PRECISE	205	0	0	0/0	0/0	0/0	0/0	0/0	0/0	0/0	0/0	0/1	0/0	0/1	0/0	0/0	0/0
Chromosome1-1564717	INS	PRECISE	9336	0	0	0/0	0/0	0/0	0/0	0/0	0/0	0/0	0/0	0/1	0/0	0/0	0/0	0/0	0/0
Chromosome1-1570820	DEL	PRECISE	185	0	0	0/0	0/0	0/0	0/0	0/0	0/0	0/0	0/0	1/1	0/0	0/1	0/1	0/0	0/0
Chromosome1-1570937	INS	PRECISE	94	0	0	0/0	0/0	0/0	0/0	0/0	0/0	0/0	0/0	0/0	0/0	0/0	0/0	0/0	0/1
Chromosome1-1572116	INS	PRECISE	103	0	0	0/0	0/1	0/1	1/1	0/0	0/0	1/1	0/1	0/0	0/0	0/0	0/0	0/0	0/0
Chromosome1-1572450	DEL	PRECISE	205	0	0	0/0	0/0	0/0	0/0	0/0	0/0	0/0	0/0	1/1	0/0	0/1	0/1	0/0	0/1
Chromosome1-1576426	DEL	PRECISE	53	0	0	0/0	0/0	0/0	0/0	0/0	0/0	0/0	0/0	1/1	0/0	0/1	0/0	0/0	0/0
Chromosome1-1585299	BND	PRECISE	0	0	0	0/0	0/0	0/0	0/0	0/0	0/0	0/0	0/0	0/1	0/0	0/1	0/0	0/0	0/0
Chromosome1-1585426	INS	PRECISE	5685	0	0	0/0	0/0	0/0	0/0	0/0	0/0	0/0	0/0	1/1	0/0	0/0	0/0	0/0	0/0
Chromosome1-1586160	DEL	PRECISE	64	0	0	0/0	0/0	0/0	0/0	0/0	0/0	0/0	0/0	0/0	0/0	0/0	0/0	0/0	0/1
Chromosome1-1597593	DEL	PRECISE	91	0	0	0/0	0/0	0/0	0/0	0/0	0/0	0/0	0/0	0/0	0/0	0/0	0/0	0/0	0/1
Chromosome1-1598755	INS	PRECISE	277	0	0	0/0	0/0	0/0	0/0	0/0	0/0	0/0	0/0	0/0	0/0	0/0	0/1	0/0	0/0
Chromosome1-1601542	DEL	PRECISE	78	0	0	0/0	0/0	0/0	0/0	0/0	0/0	0/0	0/0	0/0	0/1	0/0	0/0	0/0	0/0
Chromosome1-1611335	DEL	PRECISE	146	0	0	0/0	0/0	0/0	0/0	0/0	0/0	0/0	0/0	0/1	0/0	0/1	0/0	0/0	0/0
Chromosome1-1614754	INS	PRECISE	137	0	0.354	0/0	0/1	0/1	1/1	0/1	0/1	1/1	0/1	0/1	0/0	0/0	0/0	0/0	0/0
Chromosome1-1617517	INS	PRECISE	619	0	0	0/0	0/0	0/0	0/0	0/0	0/0	0/0	0/0	0/0	0/0	0/0	0/0	0/0	0/1
Chromosome1-1619077	INS	PRECISE	481	0	0	0/0	0/0	0/0	0/0	0/0	0/0	0/0	0/0	0/0	0/0	0/0	0/0	0/0	0/1
Chromosome1-1630508	DEL	PRECISE	143	0	0	0/0	0/0	0/0	0/0	0/0	0/0	0/0	0/0	0/0	0/0	0/0	0/1	0/0	0/0
Chromosome1-1631335	INS	PRECISE	152	0	0	0/0	0/0	0/0	0/0	0/0	0/0	0/0	0/0	0/1	0/0	0/1	0/1	0/1	0/0
Chromosome1-1632716	DEL	PRECISE	80	0	0	0/0	0/0	0/0	0/0	0/0	0/0	0/0	0/0	0/0	0/1	0/0	0/0	0/0	0/0
Chromosome1-1634015	INS	PRECISE	570	0	0.577	0/0	0/0	0/0	0/0	0/0	0/0	0/0	0/0	0/1	0/0	0/1	0/1	0/0	0/0
Chromosome1-1634743	INS	PRECISE	57	0	0	0/0	0/1	0/1	1/1	0/1	0/1	1/1	1/1	1/1	0/0	0/1	0/1	0/1	0/1
Chromosome1-1635023	DEL	PRECISE	64	0	0	0/0	0/0	0/0	0/0	0/0	0/0	0/0	0/0	0/1	0/0	0/1	0/0	0/0	0/0
Chromosome1-1637407	DEL	PRECISE	312	0	0	0/0	0/1	0/1	1/1	0/1	0/1	1/1	0/1	0/1	0/0	0/0	0/0	0/0	0/0
Chromosome1-1641910	DEL	PRECISE	2357	0	0	0/0	0/0	0/0	0/0	0/0	0/0	0/0	0/0	0/0	0/0	0/0	0/1	0/0	0/0
Chromosome1-1644004	INS	PRECISE	211	0	0	0/0	0/0	0/0	0/0	0/0	0/0	0/0	0/0	0/1	0/0	0/1	0/0	0/0	0/0
Chromosome1-1644995	INS	PRECISE	768	0	0	0/0	0/0	0/0	0/0	0/0	0/0	0/0	0/0	0/0	0/0	0/0	0/0	0/1	0/0
Chromosome1-1648464	INS	PRECISE	1289	0	0.707	0/0	0/0	0/0	0/0	0/0	0/0	0/0	0/0	0/1	0/0	0/1	0/0	0/0	0/0
Chromosome1-1650524	INS	PRECISE	128	0	0	0/0	0/0	0/0	0/0	0/0	0/0	0/0	0/0	0/0	0/0	0/0	1/1	0/0	0/0
Chromosome1-1651652	DEL	PRECISE	67	0	0	0/0	0/0	0/0	0/0	0/0	0/0	0/0	0/0	0/1	0/0	0/1	1/1	1/1	0/0
Chromosome1-1656966	DEL	PRECISE	61	0	0	0/0	0/0	0/0	0/0	0/0	0/0	0/0	0/0	0/0	0/0	0/0	0/1	0/0	0/0
Chromosome1-1664660	INS	PRECISE	70	0	0	0/0	0/0	0/0	0/0	0/0	0/0	0/0	0/0	0/1	0/0	0/1	0/1	0/1	0/0
Chromosome1-1671715	INS	PRECISE	162	0	0	0/0	0/1	0/1	1/1	0/1	1/1	1/1	0/1	0/1	0/0	0/0	0/0	0/0	0/0
Chromosome1-1691564	DEL	PRECISE	97	0	0	0/0	0/0	0/0	0/0	0/0	0/0	0/0	0/0	0/1	0/0	0/1	0/1	0/1	0/1
Chromosome1-1692895	INS	PRECISE	150	0	0.577	0/0	0/0	0/0	0/0	0/0	0/0	0/0	0/0	0/1	0/0	0/1	0/1	0/1	0/0
Chromosome1-1694403	DEL	PRECISE	50	0	0	0/0	0/0	0/0	0/0	0/0	0/0	0/0	0/0	1/1	0/0	0/1	0/1	0/1	0/0
Chromosome1-1698465	INS	PRECISE	186	0	0	0/0	0/0	0/0	0/0	0/0	0/0	0/0	0/0	0/1	0/0	0/1	0/1	0/1	0/1
Chromosome1-1720494	DEL	PRECISE	301	0	0	0/0	0/0	0/0	0/0	0/0	0/0	0/0	0/0	0/0	0/0	0/0	0/0	0/1	0/0
Chromosome1-1731717	DEL	PRECISE	282	0	0	0/0	0/0	0/0	0/0	0/0	0/0	0/0	0/0	0/1	0/0	0/0	0/0	0/0	0/0
Chromosome1-1732238	INS	PRECISE	11072	0	0	0/0	0/0	0/0	0/0	0/0	0/0	0/0	0/0	0/0	1/1	0/0	0/0	0/0	0/0
Chromosome1-1733083	INS	PRECISE	138	0	0.816	0/0	0/0	0/0	0/0	0/0	0/0	0/0	0/0	0/1	0/0	0/0	0/1	0/1	0/0
Chromosome1-1737120	INS	PRECISE	91	0	0	0/0	0/0	0/0	0/0	0/0	0/0	0/0	0/0	0/0	0/0	0/0	0/0	0/0	0/1
Chromosome1-1742726	DEL	PRECISE	60	0	0	0/0	0/0	0/0	0/0	0/0	0/0	0/0	0/0	0/1	0/0	0/1	0/1	0/1	0/0
Chromosome1-1774940	INS	PRECISE	706	0	0	0/0	0/0	0/0	0/0	0/0	0/0	0/0	0/0	0/0	0/0	0/0	0/1	0/0	0/0
Chromosome1-1781434	INS	PRECISE	148	0	0.957	0/0	0/0	0/0	0/0	0/0	0/0	0/0	0/0	0/1	0/0	0/0	0/1	0/1	0/0
Chromosome1-1784007	DEL	PRECISE	284	0	0	0/0	0/0	0/0	0/0	0/0	0/0	0/0	0/0	0/0	0/0	0/0	0/0	0/1	0/0

Chromosome1-1791159 INS	PRECISE	628	0	0	0/0	0/1	0/1	1/1	0/0	0/0	0/1	0/0	0/0	0/0	0/0	0/0	0/0	0/0
Chromosome1-1791666 DEL	PRECISE	114	0	0	0/0	0/0	0/0	0/0	0/0	0/0	0/0	0/0	0/0	0/0	0/0	0/0	0/0	0/1
Chromosome1-1797116 DEL	PRECISE	61	0	0	0/0	0/0	0/0	0/0	0/0	0/0	0/0	0/0	0/1	0/0	0/0	0/1	0/0	0/0
Chromosome1-1797604 INS	PRECISE	358	0	0.5	0/0	0/1	0/1	1/1	0/0	0/0	0/1	0/0	0/0	0/0	0/0	0/0	0/0	0/0
Chromosome1-1801644 DEL	PRECISE	71	0	0	0/0	0/0	0/0	0/0	0/0	0/0	0/0	0/0	0/0	0/0	0/0	0/0	0/0	0/1
Chromosome1-1805455 DEL	PRECISE	337	0	0	0/0	0/1	0/1	1/1	0/0	0/0	1/1	0/1	0/1	0/0	0/1	0/1	0/1	0/1
Chromosome1-1808925 DEL	PRECISE	201	0	0	0/0	0/0	0/0	0/0	0/0	0/0	0/0	0/0	0/1	0/0	0/1	0/1	0/1	0/0
Chromosome1-1811182 INS	PRECISE	725	0	0	0/0	0/0	0/0	0/0	0/0	0/0	0/0	0/0	0/0	0/0	0/0	0/0	0/1	0/0
Chromosome1-1820135 INS	PRECISE	996	0	0.577	0/0	0/0	0/0	0/0	0/1	0/1	0/0	0/0	0/1	0/0	0/0	0/0	0/0	0/0
Chromosome1-1823936 INS	PRECISE	51	0	0	0/0	0/0	0/0	0/0	0/0	0/0	0/0	0/0	0/1	0/0	1/1	0/0	0/0	0/0
Chromosome1-1824765 DEL	PRECISE	396	0.5	0.5	0/0	0/0	0/0	0/0	0/0	0/0	0/0	0/0	0/1	0/0	0/1	0/1	0/1	0/0
Chromosome1-1827556 INS	PRECISE	2795	0	0	0/0	0/0	0/0	0/0	0/0	0/0	0/0	0/0	0/0	0/0	0/0	0/0	0/0	1/1
Chromosome1-1827670 DEL	PRECISE	94	0	0	0/0	0/0	0/0	0/0	0/0	0/0	0/0	0/0	0/0	0/0	0/0	0/1	0/1	0/0
Chromosome1-1828310 INS	PRECISE	4865	0	0	0/0	0/0	0/0	0/0	0/0	0/0	0/0	0/0	0/0	0/0	0/0	0/0	0/0	1/1
Chromosome1-1836310 DEL	PRECISE	17476	0	0	0/0	0/0	0/0	0/0	0/0	0/0	0/0	0/0	0/0	0/0	0/0	0/0	0/0	0/1
Chromosome1-1842629 INS	PRECISE	1554	0	0	0/0	0/0	0/0	0/0	0/0	0/0	0/0	0/0	0/1	0/0	0/1	0/0	0/0	0/0
Chromosome1-1851432 INS	PRECISE	1386	0	0.816	0/0	0/1	0/1	1/1	0/0	0/0	0/1	0/0	0/0	0/0	0/0	0/0	0/0	0/0
Chromosome1-1853986 INS	PRECISE	5530	0	0	0/0	0/0	0/0	0/0	0/0	0/0	0/0	0/0	0/0	0/0	0/0	0/1	0/0	0/0
Chromosome1-1857427 INS	PRECISE	5670	0	0	0/0	0/0	0/0	0/0	0/0	0/0	0/0	0/0	0/0	0/0	0/0	0/0	0/1	0/0
Chromosome1-1863845 INS	PRECISE	695	0	0.577	0/0	0/0	0/0	0/0	0/0	0/0	0/0	0/0	0/1	0/0	0/1	0/1	0/1	0/0
Chromosome1-1864309 INS	PRECISE	137	0	0	0/0	0/0	0/0	0/0	0/1	0/1	0/1	0/1	0/1	0/0	0/0	0/0	0/0	0/0
Chromosome1-1864933 DEL	PRECISE	77	0	0	0/0	0/0	0/0	0/0	0/0	0/0	0/0	0/0	0/0	0/0	0/0	0/0	0/0	0/1
Chromosome1-1869783 INS	PRECISE	157	0	0	0/0	0/1	0/1	1/1	0/0	0/0	0/1	0/0	0/0	0/0	0/0	0/0	0/0	0/1
Chromosome1-1870619 INS	PRECISE	1203	0	0	0/0	0/0	0/0	0/0	0/0	0/0	0/0	0/0	0/0	0/0	0/0	0/0	0/0	0/1
Chromosome1-1890199 DEL	PRECISE	6644	0	0	0/0	0/1	0/1	1/1	0/0	0/0	0/1	0/0	0/0	0/0	0/0	0/0	0/0	0/0
Chromosome1-1894508 INS	PRECISE	3314	0	0	0/0	0/0	0/0	0/0	0/0	0/0	0/0	0/0	0/0	0/0	0/0	0/0	0/0	0/1
Chromosome1-1903366 INS	PRECISE	3273	0	0	0/0	0/0	0/0	0/0	0/0	0/0	0/0	0/0	0/0	0/0	0/0	0/0	0/0	1/1
Chromosome1-1910122 INS	PRECISE	419	0	0.5	0/0	0/0	0/0	0/0	0/0	0/0	0/0	0/0	0/1	0/0	0/1	1/1	1/1	0/0
Chromosome1-1911188 DEL	PRECISE	114	0	0	0/0	0/1	0/1	1/1	0/0	0/0	0/1	0/0	0/0	0/0	0/0	0/0	0/0	0/0
Chromosome1-1912135 INS	PRECISE	186	0	0.577	0/0	0/0	0/0	0/0	0/0	0/0	0/0	0/0	0/0	0/0	0/1	0/1	0/1	0/0
Chromosome1-1914159 DEL	PRECISE	103	0	0	0/0	0/0	0/0	0/0	0/0	0/0	0/0	0/0	0/0	0/0	0/1	0/1	0/1	0/0
Chromosome1-1916473 INS	PRECISE	71	0	0	0/0	0/0	0/0	0/0	0/0	0/0	0/0	0/0	0/0	0/0	0/1	0/1	0/1	0/0
Chromosome1-1918523 DEL	PRECISE	168	0.707	0.707	0/0	0/0	0/0	0/0	0/0	0/0	0/0	0/0	0/1	0/0	0/1	0/0	0/0	0/0
Chromosome1-1921928 DEL	PRECISE	127	0	0	0/0	0/0	0/0	0/0	0/0	0/0	0/0	0/0	0/0	0/0	0/1	0/1	0/1	0/0
Chromosome1-1925426 INS	PRECISE	161	0	0	0/0	0/0	0/0	0/0	0/0	0/0	0/0	0/0	0/0	0/0	0/1	0/0	0/0	0/0
Chromosome1-1925693 DEL	PRECISE	96	0	0	0/0	0/0	0/0	0/0	0/1	0/0	0/0	0/0	1/1	0/0	0/0	0/0	0/0	0/0
Chromosome1-1927310 DEL	PRECISE	864	0	0	0/0	0/0	0/0	0/0	0/0	0/0	0/0	0/0	0/0	0/0	0/1	0/1	0/1	0/0
Chromosome1-1928008 INS	PRECISE	63	0	0	0/0	0/1	0/1	1/1	0/0	0/0	0/1	0/0	0/0	0/0	0/0	0/0	0/0	0/0
Chromosome1-1934268 INS	PRECISE	1299	0	0.5	0/0	0/0	0/0	0/0	0/0	0/0	0/0	0/0	0/1	0/0	0/1	0/1	0/1	0/0
Chromosome1-1943624 INS	PRECISE	11518	0	0	0/0	0/0	0/0	0/0	0/0	0/0	0/0	0/0	0/0	0/0	0/0	0/0	0/0	0/1
Chromosome1-1954236 INS	PRECISE	202	0	0	0/0	0/0	0/0	0/0	0/0	0/0	0/0	0/0	0/0	0/0	0/0	0/0	0/0	0/1
Chromosome1-1960186 DEL	PRECISE	131	0	0	0/0	0/0	0/0	0/0	0/0	0/0	0/0	0/0	0/1	0/0	0/1	0/0	0/0	0/0
Chromosome1-1963189 BND	PRECISE	0	0	0	0/0	0/0	0/0	0/0	0/0	0/0	0/0	0/0	0/1	0/0	0/1	0/0	0/0	0/0
Chromosome1-1975521 DEL	PRECISE	182	0	0	0/0	0/1	0/1	1/1	0/0	0/0	0/1	0/0	0/0	0/0	0/0	0/0	0/0	0/0
Chromosome1-1983457 DEL	PRECISE	84	0	0	0/0	0/0	0/0	0/0	0/0	0/0	0/1	0/1	0/0	0/0	0/0	0/0	0/0	0/0
Chromosome1-1984723 DEL	PRECISE	570	0	0	0/0	0/0	0/0	0/0	0/0	0/0	0/1	0/1	0/0	0/0	0/0	0/0	0/0	0/0
Chromosome1-1985442 DEL	PRECISE	214	0	0	0/0	0/0	0/0	0/0	0/0	0/0	0/0	0/0	0/1	0/0	0/1	0/1	0/1	0/0
Chromosome1-1989890 DEL	PRECISE	91	0	0	0/0	0/1	0/1	1/1	0/0	0/0	0/1	0/0	0/0	0/0	0/0	0/0	0/0	0/0
Chromosome1-1993620 INS	PRECISE	135	0	0	0/0	0/0	0/0	0/0	0/0	0/0	0/0	0/0	0/0	0/0	0/0	0/0	0/0	0/1
Chromosome1-1994685 INS	PRECISE	211	0	0.5	0/0	0/1	0/1	1/1	0/0	0/0	0/1	0/0	0/0	0/0	0/0	0/0	0/0	0/0
Chromosome1-1995247 INS	PRECISE	736	0	0	0/0	0/0	0/0	0/0	0/0	0/0	0/0	0/0	0/0	0/0	0/0	0/0	0/1	0/0
Chromosome1-1997515 BND	PRECISE	0	0	0	0/0	0/0	0/0	0/0	0/0	0/0	0/1	0/0	0/0	0/0	0/0	0/0	0/0	0/0
Chromosome1-2012511 DEL	PRECISE	63	0	0	0/0	0/0	0/0	0/0	0/0	0/0	0/0	0/0	0/1	0/0	0/1	0/0	0/1	0/0
Chromosome1-2016006 INS	PRECISE	6941	0	0	0/0	0/0	0/0	0/0	0/0	0/0	0/0	0/0	0/0	1/1	0/0	0/0	0/0	0/0
Chromosome1-2018410 DEL	PRECISE	249	0	0	0/0	0/0	0/0	0/0	0/1	0/1	0/1	0/1	0/1	0/1	0/0	0/0	0/0	0/0
Chromosome1-2021845 DEL	PRECISE	162	0	0	0/0	0/0	0/0	0/0	0/0	0/0	0/0	0/0	0/1	0/0	0/1	0/1	0/1	0/0
Chromosome1-2022009 INS	PRECISE	543	0	0	0/0	0/0	0/0	0/0	0/0	0/0	0/0	0/0	0/0	0/0	0/0	0/0	0/0	0/1
Chromosome1-2031932 DEL	PRECISE	1107	0	0	0/0	0/0	0/0	0/0	0/0	0/0	0/0	0/0	0/1	0/0	0/1	0/1	0/1	0/0
Chromosome1-2033661 DEL	PRECISE	539	0.577	0.577	0/0	0/1	0/1	1/1	0/1	0/1	1/1	0/1	1/1	0/0	0/1	0/1	0/1	0/1
Chromosome1-2037908 DEL	PRECISE	137	0	0	0/0	0/1	0/1	1/1	0/0	0/0	0/1	0/0	0/1	0/0	0/1	0/1	0/1	1/1
Chromosome1-2039618 DEL	PRECISE	173	0	0	0/0	0/1	0/1	1/1	0/0	0/0	0/1	0/0	0/0	0/1	0/0	0/0	0/0	0/1
Chromosome1-2045881 INS	PRECISE	57	0	0	0/0	0/0	0/0	0/0	0/0	0/0	0/0	0/0	0/0	0/1	0/0	0/0	0/0	0/1
Chromosome1-2065386 DEL	PRECISE	79	0	0	0/0	0/1	0/1	1/1	0/0	0/0	0/1	0/0	0/0	0/1	0/0	0/0	0/0	0/1
Chromosome1-2074045 DEL	PRECISE	2620	0	0	0/0	0/0	0/0	0/0	0/0	0/0	0/0	0/0	0/0	0/1	0/0	0/0	0/0	0/0
Chromosome1-2079570 INS	PRECISE	861	0	0.577	0/0	0/0	0/0	0/0	0/1	0/1	0/0	0/0	0/1	0/0	0/0	0/0	0/0	0/0
Chromosome1-2081991 DEL	PRECISE	161	0.48	0	0/0	0/1	0/1	1/1	0/1	0/1	1/1	0/1	1/1	1/1	0/1	0/1	0/1	1/1
Chromosome1-2091098 DEL	PRECISE	616	0	0	0/0	0/1	0/1	1/1	0/0	0/0	0/1	0/0	0/0	0/1	0/0	0/0	0/0	1/1
Chromosome1-2097808 INS	PRECISE	102	0	0	0/0	0/1	0/1	1/1	0/0	0/0	0/1	0/0	0/0	0/1	0/0	0/0	0/0	0/1
Chromosome1-2098750 DEL	PRECISE	67	0	0	0/0	0/0	0/0	0/0	0/0	0/0	0/0	0/0	0/0	0/0	0/0	0/1	0/0	0/0
Chromosome1-2111762 DUP	PRECISE	2473	0	0	0/0	0/0	0/0	0/0	0/0	0/0	0/0	0/0	0/0	0/0	0/0	0/0	0/0	0/1
Chromosome1-2112169 INS	PRECISE	3013	0	0	0/0	0/0	0/0	0/0	0/0	0/0	0/0	0/0	0/0	0/0	0/0	0/0	0/0	0/1
Chromosome1-2114796 INS	PRECISE	150	0	0	0/0	0/0	0/0	0/0	0/0	0/0	0/0	0/0	0/0	0/0	0/0	0/0	0/0	0/1
Chromosome1-2119846 INS	PRECISE	50	0	0	0/0	0/0	0/0	0/0	0/0	0/0	0/0	0/0	0/1	0/0	0/1	0/1	0/1	0/0

Chromosome1-2121470	DEL	PRECISE	63	0	0	0/0	0/0	0/0	0/0	0/0	0/0	0/0	0/0	0/1	0/0	0/1	0/1	0/1	0/0
Chromosome1-2129578	DEL	PRECISE	61	0	0	0/0	0/0	0/0	0/0	0/0	0/0	0/0	0/0	0/0	0/0	0/0	0/1	0/0	0/0
Chromosome1-2129812	DEL	PRECISE	1022	0	0	0/0	0/0	0/0	0/0	0/0	0/0	0/0	0/0	0/0	0/0	0/0	0/1	0/0	0/0
Chromosome1-2132996	DEL	PRECISE	448	0	0	0/0	0/0	0/0	0/0	0/0	0/0	0/0	0/0	0/1	0/0	0/1	0/0	0/1	0/0
Chromosome1-2138706	DEL	PRECISE	61	0	0	0/0	0/0	0/0	0/0	0/0	0/0	0/0	0/0	0/0	0/0	0/0	0/1	0/0	0/0
Chromosome1-2138753	INS	PRECISE	1119	0	0	0/0	0/0	0/0	0/0	0/0	0/0	0/0	0/0	0/0	0/0	0/0	0/0	0/0	0/1
Chromosome1-2140740	INS	PRECISE	451	0	0.957	0/0	0/0	0/0	0/0	0/0	0/0	0/0	0/0	0/1	0/0	0/1	0/1	0/1	0/0
Chromosome1-2145768	DEL	PRECISE	119	0	0	0/0	0/0	0/0	0/0	0/0	0/0	0/0	0/0	0/0	0/0	0/0	0/0	0/0	0/1
Chromosome1-2146441	DUP	PRECISE	18228447	0	0	0/0	0/0	0/0	0/0	0/1	0/1	0/0	0/0	0/1	0/0	0/0	0/0	0/0	0/0
Chromosome1-2146446	BND	PRECISE	0	0	0	0/0	0/0	0/0	0/0	0/0	0/0	0/0	0/0	0/1	0/0	0/0	0/0	0/0	0/0
Chromosome1-2147977	DEL	PRECISE	538	0	0	0/0	0/0	0/0	0/0	0/0	0/0	0/0	0/0	0/1	0/0	0/1	0/0	0/0	0/0
Chromosome1-2148741	DEL	PRECISE	198	0	0	0/0	0/0	0/0	0/0	0/0	0/0	0/0	0/0	0/1	0/0	0/1	0/0	0/0	0/0
Chromosome1-2150424	DEL	PRECISE	1774	0	0	0/0	0/0	0/0	0/0	0/0	0/0	0/0	0/0	0/0	0/0	0/1	0/1	0/0	0/0
Chromosome1-2174559	DEL	PRECISE	938	0	0	0/0	0/0	0/0	0/0	0/0	0/0	0/0	0/0	0/1	0/0	0/1	0/1	0/1	0/0
Chromosome1-2176843	INS	PRECISE	323	0	0	0/0	0/0	0/0	0/0	0/0	0/0	0/0	0/0	0/0	0/1	0/0	0/0	0/0	0/0
Chromosome1-2181110	DEL	PRECISE	348	0	0	0/0	0/1	0/1	1/1	0/0	0/0	0/1	0/0	0/1	0/1	0/1	0/1	0/1	0/1
Chromosome1-2182007	INS	PRECISE	54	0	0	0/0	0/1	0/1	1/1	0/0	0/0	0/1	0/0	0/0	0/0	0/0	0/0	0/0	0/1
Chromosome1-2185982	DEL	PRECISE	4967	0	0	0/0	0/1	0/1	1/1	0/1	0/1	1/1	0/1	1/1	0/1	0/1	0/1	0/1	0/1
Chromosome1-2196151	INS	PRECISE	79	0.333	0.866	0/0	0/1	0/1	1/1	0/0	0/0	0/1	0/0	0/1	0/0	0/1	0/0	0/1	0/1
Chromosome1-2208295	DEL	PRECISE	53	0	0	0/0	0/0	0/0	0/0	0/0	0/0	0/0	0/0	0/0	0/0	0/0	0/1	0/0	0/0
Chromosome1-2208566	INS	PRECISE	5467	0	0	0/0	0/0	0/0	0/0	0/0	0/0	0/0	0/0	0/0	0/0	0/0	0/0	1/1	0/0
Chromosome1-2227580	DEL	PRECISE	324	0	0	0/0	0/0	0/0	0/0	0/0	0/0	0/0	0/0	0/1	0/0	0/1	0/1	0/0	0/0
Chromosome1-2228260	INS	PRECISE	150	0	0	0/0	0/0	0/0	0/0	0/0	0/0	0/0	0/0	0/1	0/0	0/1	0/1	0/0	0/0
Chromosome1-2231240	DEL	PRECISE	222	0	0	0/0	0/0	0/0	0/0	0/0	0/0	0/0	0/0	0/0	0/0	0/0	0/0	1/1	0/0
Chromosome1-2243820	DEL	PRECISE	905	0	0	0/0	0/1	0/1	1/1	0/1	0/1	1/1	0/1	1/1	0/1	0/1	0/1	1/1	1/1
Chromosome1-2252006	DEL	PRECISE	79	0	0	0/0	0/0	0/0	0/0	0/0	0/0	0/0	0/0	0/0	0/0	0/0	0/1	0/0	0/0
Chromosome1-2258064	INS	PRECISE	1195	0	0	0/0	0/0	0/0	0/0	0/0	0/0	0/0	0/0	0/0	0/0	0/0	0/0	0/1	0/0
Chromosome1-2261610	INS	PRECISE	63	0	0	0/0	0/0	0/0	0/0	0/0	0/0	0/0	0/0	0/1	0/0	0/1	0/1	0/1	0/0
Chromosome1-2271085	DEL	PRECISE	82	0	0	0/0	0/0	0/0	0/0	0/0	0/0	0/0	0/0	0/1	0/0	0/1	0/1	0/1	0/0
Chromosome1-2272434	DEL	PRECISE	166	0	0	0/0	0/0	0/0	0/0	0/0	0/0	0/0	0/0	0/1	0/0	0/1	0/1	0/1	0/0
Chromosome1-2284558	DEL	PRECISE	50	0	0	0/0	0/0	0/0	0/0	0/0	0/0	0/0	0/0	0/1	0/0	0/1	0/0	0/0	0/0
Chromosome1-2290982	DEL	PRECISE	508	0	0	0/0	0/0	0/0	0/0	0/0	0/0	0/0	0/0	0/1	0/0	0/1	0/1	0/1	0/0
Chromosome1-2305276	INS	PRECISE	78	0	0	0/0	0/1	0/1	1/1	0/0	0/0	0/1	0/0	0/0	0/0	0/0	0/0	0/0	0/1
Chromosome1-2305853	DEL	PRECISE	363	0.333	0	0/0	0/1	0/1	1/1	0/0	0/0	0/1	0/0	0/1	0/0	0/1	0/1	0/1	0/1
Chromosome1-2306269	INS	PRECISE	160	0	0.447	0/0	0/0	0/0	0/0	0/1	0/1	0/1	0/1	0/1	0/0	0/0	0/0	0/0	0/0
Chromosome1-2309981	DEL	PRECISE	55	0	0	0/0	0/1	0/1	0/1	0/0	0/0	0/1	0/0	0/0	0/0	0/0	0/0	0/0	0/1
Chromosome1-2317438	DEL	PRECISE	513	0	0	0/0	0/0	0/0	0/0	0/0	0/0	0/0	0/0	0/1	0/0	0/0	0/0	0/0	0/0
Chromosome1-2318768	INS	PRECISE	277	0	0	0/0	0/0	0/0	0/0	0/0	0/0	0/0	0/0	0/0	0/0	0/0	0/0	0/0	0/1
Chromosome1-2318852	DEL	PRECISE	82	0	0	0/0	0/0	0/0	0/0	0/0	0/0	0/0	0/0	0/1	0/0	0/0	0/0	0/0	0/0
Chromosome1-2320767	INS	PRECISE	150	0	0.447	0/0	0/1	0/1	1/1	0/0	0/0	0/1	0/0	0/0	0/0	0/0	0/0	0/0	0/1
Chromosome1-2321261	DEL	PRECISE	477	0	0	0/0	0/1	0/1	1/1	0/0	0/0	1/1	0/0	1/1	0/0	0/1	0/1	0/0	1/1
Chromosome1-2321929	DEL	PRECISE	1700	0	0	0/0	0/1	0/1	1/1	0/1	0/1	1/1	0/1	1/1	0/1	0/1	0/1	0/0	1/1
Chromosome1-2338189	DEL	PRECISE	51	0	0	0/0	0/0	0/0	0/0	0/0	0/0	0/0	0/0	1/1	0/0	0/0	0/1	0/0	0/0
Chromosome1-2345028	DEL	PRECISE	154	0	0	0/0	0/0	0/0	0/0	0/0	0/0	0/0	0/0	0/1	0/0	0/0	0/1	0/1	0/0
Chromosome1-2346844	DEL	PRECISE	54	0	0	0/0	0/0	0/0	0/0	0/0	0/0	0/0	0/0	0/0	0/0	0/0	0/0	0/0	0/1
Chromosome1-2348875	DEL	PRECISE	53	0	0	0/0	0/0	0/0	0/0	0/0	0/0	0/0	0/0	0/0	0/0	0/0	0/1	0/0	0/0
Chromosome1-2354623	DEL	PRECISE	637	0	0	0/0	0/0	0/0	0/0	0/0	0/0	0/0	0/0	0/1	0/0	0/1	0/1	0/1	0/0
Chromosome1-2364119	INS	PRECISE	540	0	0.548	0/0	0/1	0/1	1/1	0/0	0/0	0/1	0/0	0/0	0/0	0/0	0/0	0/0	0/1
Chromosome1-2366659	INS	PRECISE	2902	0	0	0/0	0/0	0/0	0/0	0/0	0/0	0/0	0/0	0/0	1/1	0/0	0/0	0/0	0/0
Chromosome1-2372140	DEL	PRECISE	885	0	0	0/0	0/1	0/1	0/1	0/0	0/0	0/1	0/0	0/1	0/0	0/1	0/1	0/1	1/1
Chromosome1-2376609	INS	PRECISE	364	0	0.447	0/0	0/1	0/1	0/1	0/0	0/0	0/1	0/0	0/0	0/0	0/0	0/0	0/0	0/1
Chromosome1-2377651	INS	PRECISE	54	0	0	0/0	0/0	0/0	0/0	0/0	0/0	0/0	0/0	0/0	0/0	0/0	0/1	0/0	0/0
Chromosome1-2381758	INS	PRECISE	126	0	0	0/0	0/0	0/0	0/0	0/0	0/0	0/0	0/0	0/0	0/0	0/0	0/0	0/0	0/1
Chromosome1-2382467	DEL	PRECISE	240	0	0	0/0	0/0	0/0	0/0	0/0	0/0	0/0	0/0	0/1	0/0	0/1	0/1	0/0	0/0
Chromosome1-2382988	INS	PRECISE	8773	0	0	0/0	0/0	0/0	0/0	0/0	0/0	0/0	0/0	0/0	0/0	0/0	0/0	0/0	0/0
Chromosome1-2383403	INS	PRECISE	9378	0	0	0/0	0/0	0/0	0/0	0/0	0/0	0/0	0/0	0/0	0/0	0/0	1/1	0/0	0/0
Chromosome1-2383626	BND	PRECISE	0	0	0	0/0	0/0	0/0	0/0	0/0	0/0	0/0	0/0	0/0	0/0	0/0	0/1	0/0	0/0
Chromosome1-2385698	DEL	PRECISE	599	0	0	0/0	0/0	0/0	0/0	0/0	0/0	0/0	0/0	0/0	0/0	0/0	0/1	0/1	0/0
Chromosome1-2386042	INS	PRECISE	211	0	0	0/0	0/0	0/0	0/0	0/0	0/0	0/1	0/1	0/0	0/0	0/0	0/0	0/0	0/0
Chromosome1-2391434	DEL	PRECISE	103	0	0	0/0	0/0	0/0	0/0	0/1	0/1	0/1	0/1	0/1	0/0	0/0	0/0	0/0	0/0
Chromosome1-2392753	DEL	PRECISE	117	0	0	0/0	0/0	0/0	0/0	0/0	0/0	0/0	0/0	0/1	0/0	0/1	0/1	0/1	0/0
Chromosome1-2396971	DEL	PRECISE	249	0	0	0/0	0/0	0/0	0/0	0/0	0/0	0/0	0/0	0/1	0/0	0/1	0/1	0/1	0/0
Chromosome1-2397544	INS	PRECISE	141	0	0.5	0/0	0/0	0/0	0/0	0/0	0/0	0/0	0/0	0/1	0/0	0/1	0/1	0/1	0/0
Chromosome1-2401297	INS	PRECISE	174	0	0	0/0	0/0	0/0	0/0	0/0	0/0	0/0	0/0	0/1	0/0	0/1	0/1	0/1	0/0
Chromosome1-2403866	INS	PRECISE	451	0	0	0/0	0/1	0/1	0/1	0/0	0/0	0/1	0/0	0/0	0/0	0/0	0/0	0/0	0/1
Chromosome1-2405443	INS	PRECISE	1021	0	0.837	0/0	0/1	0/1	0/1	0/0	0/0	0/1	0/0	0/0	0/0	0/0	0/0	0/0	0/1
Chromosome1-2422182	DEL	PRECISE	67	0	0	0/0	0/1	0/1	0/1	0/0	0/0	0/1	0/0	0/0	0/0	0/0	0/0	0/0	0/1
Chromosome1-2428007	DEL	PRECISE	84	0	0	0/0	0/1	0/1	0/1	0/0	0/0	0/1	0/0	0/0	0/0	0/0	0/0	0/0	0/1
Chromosome1-2431631	INS	PRECISE	359	0	0.707	0/0	0/0	0/0	0/0	0/0	0/0	0/0	0/0	0/1	0/0	0/1	0/0	0/0	0/0
Chromosome1-2437081	DEL	PRECISE	51	0	0	0/0	0/0	0/0	0/0	0/0	0/0	0/0	0/0	0/1	0/0	0/1	0/0	0/0	0/0
Chromosome1-2444293	DEL	PRECISE	71	0	0	0/0	0/0	0/0	0/0	0/0	0/0	0/0	0/0	0/0	0/0	0/0	0/0	0/1	0/0
Chromosome1-2447292	INS	PRECISE	72	0.905	0.905	0/0	0/1	0/1	0/1	0/1	0/1	1/1	0/1	1/1	0/0	0/1	0/1	0/1	0/1
Chromosome1-2447648	INS	PRECISE	804	0	0	0/0	0/0	0/0	0/0	0/0	0/0	0/0	0/0	0/0	0/0	0/0	0/0	0/1	0/0

Chromosome1-2450359	INS	PRECISE	100	0	0	0/0	0/0	0/0	0/0	0/0	0/0	0/0	0/0	0/0	0/0	0/0	0/0	0/1	0/0
Chromosome1-2452522	INS	PRECISE	4061	0	0	0/0	0/0	0/0	0/0	0/0	0/0	0/0	0/0	0/0	0/0	0/0	0/0	1/1	0/0
Chromosome1-2455855	INS	PRECISE	150	0	0	0/0	0/0	0/0	0/0	0/0	0/0	0/0	0/0	0/0	0/0	0/0	0/0	0/1	0/0
Chromosome1-2457415	BND	PRECISE	0	0	0	0/0	0/0	0/0	0/1	0/0	0/0	0/1	0/0	0/0	0/0	0/0	0/0	0/0	0/0
Chromosome1-2464492	BND	PRECISE	0	0	0	0/0	0/0	0/0	0/0	0/0	0/0	0/0	0/0	0/0	0/0	0/0	0/0	0/1	0/0
Chromosome1-2467762	INS	PRECISE	6685	0	0.707	0/0	0/0	0/0	0/0	0/0	0/0	0/0	0/0	0/0	0/0	1/1	0/0	0/0	0/0
Chromosome1-2469412	DEL	PRECISE	52	0	0	0/0	0/0	0/0	0/0	0/0	0/0	0/0	0/0	0/0	0/0	0/1	0/1	0/1	0/0
Chromosome1-2473126	INS	PRECISE	3878	0	0	0/0	0/0	0/0	0/0	0/0	0/0	0/0	0/0	0/0	0/0	0/0	1/1	0/0	0/0
Chromosome1-2473130	BND	PRECISE	0	0	0	0/0	0/0	0/0	0/0	0/0	0/0	0/0	0/0	0/0	0/0	0/0	0/1	0/0	0/0
Chromosome1-2473512	INS	PRECISE	156	0	0	0/0	0/0	0/0	0/0	0/0	0/0	0/0	0/0	0/0	0/0	0/0	0/1	0/0	0/0
Chromosome1-2477594	DEL	PRECISE	979	0.756	0.756	0/0	0/1	0/1	0/1	0/0	0/0	0/1	0/0	0/0	0/0	0/0	0/1	0/1	0/1
Chromosome1-2489439	INS	PRECISE	233	0	0.577	0/0	0/0	0/0	0/0	0/0	0/0	0/0	0/0	0/1	0/0	0/1	0/1	0/0	0/0
Chromosome1-2489566	DEL	PRECISE	89	0	0	0/0	0/0	0/0	0/0	0/0	0/0	0/0	0/0	0/0	0/0	0/0	0/0	0/0	0/1
Chromosome1-2490693	DEL	PRECISE	128	0	0	0/0	0/0	0/0	0/0	0/0	0/0	0/0	0/0	0/1	0/0	0/1	0/1	0/0	0/0
Chromosome1-2497091	INS	PRECISE	8824	0	0	0/0	0/0	0/0	0/0	0/0	0/0	0/0	0/0	0/0	0/0	0/0	0/0	0/0	0/1
Chromosome1-2498548	INS	PRECISE	332	0	0.577	0/0	0/0	0/0	0/0	0/0	0/0	0/0	0/0	0/1	0/0	0/1	0/1	0/0	0/0
Chromosome1-2500291	INS	PRECISE	162	0	0	0/0	0/0	0/0	0/0	0/0	0/0	0/0	0/0	0/0	0/0	0/0	0/0	0/1	0/0
Chromosome1-2513844	INV	PRECISE	10996098	0	0	0/0	0/0	0/1	0/0	0/0	0/0	0/0	0/0	0/0	0/0	0/0	0/0	0/0	0/0
Chromosome1-2513951	INS	PRECISE	162	0	0	0/0	0/0	0/0	0/0	0/0	0/0	0/0	0/0	0/0	0/0	0/0	0/0	0/0	0/1
Chromosome1-2515037	INS	PRECISE	50	0	0	0/0	0/1	0/1	0/1	0/0	0/0	0/1	0/0	0/0	0/0	0/0	0/0	0/0	0/1
Chromosome1-2517803	INS	PRECISE	1776	0	0	0/0	0/0	0/0	0/1	0/0	0/0	0/0	0/0	0/0	0/0	0/0	0/0	0/0	0/0
Chromosome1-2521468	DEL	PRECISE	77	0	0	0/0	0/1	0/1	1/1	0/0	0/0	0/1	0/0	0/0	0/1	0/0	0/0	0/0	1/1
Chromosome1-2529031	DEL	PRECISE	131	0	0	0/0	0/0	0/0	0/0	0/0	0/0	0/0	0/0	0/0	0/0	0/1	0/0	0/1	0/0
Chromosome1-2530656	DEL	PRECISE	210	0	0	0/0	0/0	0/0	0/0	0/0	0/0	0/0	0/0	0/0	0/0	0/0	0/0	0/1	0/1
Chromosome1-2533164	INS	PRECISE	164	0	0	0/0	0/0	0/0	0/0	0/0	0/0	0/0	0/0	0/0	0/0	0/0	0/0	1/1	0/0
Chromosome1-2538487	DEL	PRECISE	673	0	0	0/0	0/0	0/0	0/0	0/0	0/0	0/0	0/0	0/0	0/0	0/0	0/1	0/0	0/0
Chromosome1-2543612	INS	PRECISE	62	0	0.548	0/0	0/1	0/1	1/1	0/0	0/0	0/1	0/0	0/0	0/0	0/0	0/0	0/0	1/1
Chromosome1-2543843	DEL	PRECISE	6610	0	0	0/0	0/0	0/0	0/0	0/0	0/0	0/0	0/0	0/0	0/0	0/0	0/1	0/0	0/0
Chromosome1-2556783	DEL	PRECISE	91	0	0	0/0	0/0	0/0	0/0	0/0	0/0	0/0	0/0	0/0	0/0	0/0	0/0	0/0	0/1
Chromosome1-2558500	INS	PRECISE	5162	0	0	0/0	0/0	0/0	0/0	0/0	0/0	0/0	0/0	0/0	0/0	0/0	0/0	0/1	0/0
Chromosome1-2559038	BND	PRECISE	0	0	0	0/0	0/0	0/0	0/0	0/1	0/0	0/0	0/0	0/0	0/0	0/0	0/0	0/0	0/0
Chromosome1-2559083	INS	PRECISE	1261	0	0	0/0	0/0	0/0	0/0	0/0	0/0	0/0	0/0	0/0	0/1	0/0	0/0	0/0	0/0
Chromosome1-2577999	INS	PRECISE	243	0	0	0/0	0/0	0/0	0/0	0/0	0/0	0/0	0/0	0/0	0/0	0/0	0/0	0/1	0/0
Chromosome1-2578774	DEL	PRECISE	878	0	0	0/0	0/0	0/0	0/0	0/0	0/0	0/0	0/0	0/1	0/0	0/0	0/1	0/1	0/0
Chromosome1-2580391	INS	PRECISE	734	0	0	0/0	0/0	0/0	0/0	0/0	0/0	0/0	0/0	0/0	0/1	0/0	0/0	0/0	0/0
Chromosome1-2580933	INS	PRECISE	137	0	0	0/0	0/0	0/0	0/0	0/0	0/0	0/0	0/0	0/0	0/0	0/0	0/0	0/0	0/1
Chromosome1-2582341	DEL	PRECISE	67	0	0	0/0	0/0	0/0	0/1	0/0	0/0	0/0	0/0	0/0	0/0	0/0	0/0	0/0	0/0
Chromosome1-2584874	DEL	PRECISE	56	0	0	0/0	0/0	0/0	0/0	0/0	0/0	0/0	0/0	0/1	0/0	0/0	0/1	0/0	0/0
Chromosome1-2586358	INS	PRECISE	130	0	0	0/0	0/0	0/0	0/0	0/0	0/0	0/0	0/0	0/0	0/1	0/0	0/0	0/0	0/0
Chromosome1-2594913	DUP	PRECISE	185	0	0	0/0	0/0	0/0	0/0	0/0	0/0	0/0	0/0	0/0	0/0	0/0	0/0	0/0	0/1
Chromosome1-2595694	DEL	PRECISE	137	0	0	0/0	0/1	0/1	1/1	0/1	0/0	1/1	0/1	0/1	0/0	0/0	0/0	0/0	1/1
Chromosome1-2596883	BND	PRECISE	0	0	0	0/0	0/0	0/0	0/0	0/1	0/0	0/1	0/0	1/1	0/1	0/0	0/0	0/0	0/0
Chromosome1-2596904	BND	PRECISE	0	0	0	0/0	0/0	0/0	0/0	0/0	0/0	0/0	0/0	0/1	0/0	0/0	0/0	0/0	0/0
Chromosome1-2597112	DEL	PRECISE	696	0	0	0/0	0/0	0/0	0/1	0/0	0/0	0/0	0/0	0/0	0/0	0/0	0/0	0/0	0/0
Chromosome1-2598009	DEL	PRECISE	3789	0	0	0/0	0/1	0/1	0/1	0/0	0/0	1/1	0/0	0/0	0/0	0/0	0/0	0/0	1/1
Chromosome1-2604983	DEL	PRECISE	420	0	0	0/0	0/0	0/0	0/1	0/0	0/0	0/0	0/0	0/0	0/0	0/0	0/0	0/0	0/0
Chromosome1-2606439	INS	PRECISE	52	0	0.816	0/0	0/1	0/1	0/1	0/0	0/0	0/1	0/0	0/0	0/1	0/0	0/0	0/0	0/1
Chromosome1-2616068	DEL	PRECISE	164	0	0	0/0	0/0	0/0	0/0	0/0	0/0	0/0	0/0	0/0	0/0	0/0	0/0	0/0	0/1
Chromosome1-2619372	BND	PRECISE	0	0	0	0/0	0/0	0/0	0/0	0/0	0/0	0/0	0/0	0/0	0/1	0/0	0/0	0/0	0/0
Chromosome1-2621274	DEL	PRECISE	1389	0	0	0/0	0/0	0/0	0/1	0/1	0/1	0/1	0/1	1/1	0/1	0/0	0/1	0/0	0/0
Chromosome1-2627028	DEL	PRECISE	71	0	0	0/0	0/1	0/1	0/1	0/0	0/0	0/1	0/0	0/0	0/0	0/0	0/0	0/0	0/1
Chromosome1-2628835	DEL	PRECISE	242	0	0	0/0	0/0	0/0	0/0	0/0	0/0	0/0	0/0	0/1	0/0	0/1	0/1	0/1	0/0
Chromosome1-2630954	INS	PRECISE	565	0	0.5	0/0	0/0	0/0	0/1	0/1	0/1	0/0	0/0	0/1	0/0	0/0	0/0	0/0	0/0
Chromosome1-2639330	INS	PRECISE	5155	0	0	0/0	0/0	0/0	1/1	0/0	0/0	0/0	0/0	0/0	0/0	0/0	0/0	0/0	0/0
Chromosome1-2643548	DEL	PRECISE	186	0	0	0/0	0/0	0/0	0/0	0/0	0/0	0/0	0/0	0/1	0/0	0/1	0/1	0/1	0/0
Chromosome1-2654607	DEL	PRECISE	221	0	0	0/0	0/0	0/0	0/0	0/0	0/0	0/0	0/0	0/1	0/0	0/1	0/1	0/1	0/0
Chromosome1-2660078	DEL	PRECISE	271	0	0	0/0	0/1	0/1	0/1	0/0	0/0	0/1	0/0	0/1	0/0	0/1	0/1	0/0	1/1
Chromosome1-2663825	INS	PRECISE	1943	0	0	0/0	0/0	0/0	0/0	0/0	0/0	0/0	0/0	0/0	0/0	0/0	0/0	0/0	0/1
Chromosome1-2687926	DEL	PRECISE	1307	0	0	0/0	0/0	0/0	0/0	0/0	0/0	0/0	0/0	0/0	0/0	0/0	0/0	0/0	0/1
Chromosome1-2690134	DEL	PRECISE	777	0	0	0/0	0/0	0/0	0/0	0/0	0/0	0/0	0/0	0/0	0/0	0/1	0/0	0/0	0/0
Chromosome1-2691183	BND	PRECISE	0	0	0	0/0	0/0	0/0	0/0	0/0	0/0	0/0	0/0	0/0	0/0	0/0	0/0	0/1	0/0
Chromosome1-2701473	INS	PRECISE	1472	0	0.707	0/0	0/0	0/0	0/0	0/0	0/0	0/0	0/0	0/0	0/0	0/0	1/1	0/1	0/0
Chromosome1-2704145	INS	PRECISE	140	0	0	0/0	0/0	0/0	0/0	0/0	0/0	0/0	0/0	0/0	0/0	0/0	1/1	0/1	0/0
Chromosome1-2709608	INS	PRECISE	175	0	0	0/0	0/0	0/0	0/0	0/0	0/0	0/0	0/0	0/0	0/0	0/0	0/0	0/0	0/1
Chromosome1-2712477	INS	PRECISE	140	0	0.5	0/0	0/0	0/0	0/0	0/0	0/0	0/0	0/0	0/1	0/0	0/1	1/1	0/1	0/0
Chromosome1-2715864	INS	PRECISE	859	0	0	0/0	0/0	0/0	0/0	0/0	0/0	0/0	0/0	0/0	0/0	0/0	0/1	0/0	0/0
Chromosome1-2719885	DEL	PRECISE	349	0	0	0/0	0/0	0/0	0/0	0/0	0/0	0/0	0/0	0/0	0/0	0/0	0/0	0/0	0/1
Chromosome1-2723043	INS	PRECISE	60	0	0.516	0/0	0/1	0/1	1/1	0/0	0/0	0/1	0/0	0/0	0/1	0/0	0/0	0/0	1/1
Chromosome1-2726079	INS	PRECISE	66	0	0	0/0	0/0	0/0	0/0	0/0	0/0	0/0	0/0	0/0	0/0	0/0	0/0	0/0	0/1
Chromosome1-2729564	INS	PRECISE	61	0	0	0/0	0/1	0/1	0/1	0/0	0/0	0/1	0/0	0/0	0/0	0/0	0/0	0/0	0/1
Chromosome1-2748920	INS	PRECISE	5721	0	0	0/0	0/0	0/0	0/0	0/0	0/0	0/0	0/0	0/0	0/0	0/0	0/0	1/1	0/0
Chromosome1-2750135	INS	PRECISE	9083	0	0	0/0	0/0	0/0	0/0	0/0	0/0	0/0	0/0	0/0	0/0	0/0	0/0	1/1	0/0
Chromosome1-2750748	DEL	PRECISE	128	0	0	0/0	0/0	0/0	0/0	0/0	0/0	0/0	0/0	0/0	0/0	0/1	0/1	0/1	0/0

Chromosome1-2751093	INS	PRECISE	1297	0	0	0/0	0/0	0/0	0/0	0/0	0/0	0/0	0/0	0/0	0/0	0/0	0/1	0/0	0/0
Chromosome1-2760650	DEL	PRECISE	332	0	0	0/0	0/0	0/0	0/0	0/0	0/0	0/0	0/0	0/1	0/0	0/1	0/1	0/1	0/0
Chromosome1-2760919	INS	PRECISE	362	0	0.548	0/0	0/1	0/1	0/1	0/0	0/0	0/1	0/0	0/0	0/0	0/0	0/0	0/0	0/1
Chromosome1-2762061	INS	PRECISE	201	0	0.707	0/0	0/0	0/0	0/0	0/0	0/0	0/0	0/0	0/1	0/0	0/1	0/0	0/0	0/0
Chromosome1-2764454	DEL	PRECISE	63	0	0	0/0	0/0	0/0	0/0	0/0	0/0	0/0	0/0	0/1	0/0	0/1	0/1	0/1	0/0
Chromosome1-2765623	DEL	PRECISE	2310	0.667	0	0/0	0/1	0/1	0/1	0/0	0/0	0/1	0/0	0/1	0/0	0/1	0/1	0/1	1/1
Chromosome1-2766347	INS	PRECISE	90	0	0	0/0	0/0	0/0	0/0	0/0	0/0	0/0	0/0	0/0	0/1	0/0	0/0	0/0	0/0
Chromosome1-2780240	INS	PRECISE	262	0	0	0/0	0/0	0/0	0/0	0/0	0/0	0/0	0/0	0/1	0/0	0/1	0/1	0/1	0/0
Chromosome1-2784174	INS	PRECISE	1138	0	0	0/0	0/0	0/0	0/0	0/0	0/0	0/0	0/0	0/0	0/0	0/0	0/0	0/0	0/1
Chromosome1-2787161	BND	PRECISE	0	0	0	0/0	0/0	0/0	0/0	0/0	0/0	0/0	0/0	0/0	0/0	0/0	0/0	0/0	0/1
Chromosome1-2802706	DEL	PRECISE	68	0	0	0/0	0/0	0/0	0/0	0/0	0/0	0/0	0/0	0/1	0/0	0/0	0/0	0/1	0/0
Chromosome1-2805685	INS	PRECISE	3985	0	0	0/0	0/0	0/0	0/0	0/0	0/0	0/0	0/0	0/0	0/0	0/0	0/1	0/0	0/0
Chromosome1-2806524	DEL	PRECISE	1482	0	0	0/0	0/1	0/0	0/0	0/0	0/0	0/0	0/0	0/0	0/0	0/0	0/0	0/0	0/0
Chromosome1-2820534	DEL	PRECISE	860	0	0	0/0	0/0	0/0	0/0	0/0	0/0	0/0	0/0	0/0	0/1	0/0	0/0	0/0	0/0
Chromosome1-2823282	INS	PRECISE	3055	0	0	0/0	0/0	0/0	0/0	0/0	0/0	0/0	0/0	0/0	1/1	0/0	0/0	0/0	0/0
Chromosome1-2825228	BND	PRECISE	0	0	0	0/0	0/1	0/1	0/0	0/0	0/0	0/0	0/0	0/0	0/0	0/0	0/0	0/0	0/0
Chromosome1-2825228	BND	PRECISE	0	0	0	0/0	0/0	0/0	0/0	0/0	0/0	0/1	0/0	0/0	0/0	0/0	0/0	0/0	0/0
Chromosome1-2835688	DEL	PRECISE	68	0	0	0/0	0/1	0/1	0/1	0/0	0/0	0/1	0/0	0/0	0/0	0/0	0/0	0/0	0/1
Chromosome1-2839134	DEL	PRECISE	4758	0	0	0/0	0/0	0/0	0/0	0/0	0/0	0/0	0/0	0/0	0/1	0/0	0/0	0/0	0/0
Chromosome1-2844548	DEL	PRECISE	594	0	0	0/0	0/0	0/0	0/0	0/0	0/0	0/0	0/0	0/0	0/1	0/0	0/0	0/0	0/0
Chromosome1-2850292	INS	PRECISE	5714	0	0	0/0	0/0	0/0	0/0	0/0	0/0	0/0	0/0	0/0	0/0	0/0	0/0	0/0	0/0
Chromosome1-2854977	BND	PRECISE	0	0	0	0/0	0/0	0/0	0/0	0/0	0/0	0/0	0/0	0/0	0/1	0/0	0/0	0/0	0/0
Chromosome1-2855098	INS	PRECISE	4842	0	0	0/0	0/0	0/0	0/0	0/0	0/0	0/0	0/0	0/0	0/0	0/0	0/0	0/0	0/1
Chromosome1-2856245	DEL	PRECISE	59	0	0	0/0	1/1	0/1	0/1	0/0	0/0	1/1	0/0	0/0	0/0	0/0	0/0	0/0	1/1
Chromosome1-2864889	BND	PRECISE	0	0	0	0/0	0/0	0/0	0/1	0/0	0/0	0/0	0/0	0/0	0/0	0/0	0/0	0/0	0/1
Chromosome1-2877999	DEL	PRECISE	5691	0	0	0/0	0/1	0/1	1/1	0/1	0/1	1/1	0/1	1/1	0/1	0/0	0/0	0/0	1/1
Chromosome1-2884965	BND	PRECISE	0	0	0	0/0	0/0	0/0	0/0	0/1	0/0	0/0	0/0	0/1	0/0	0/0	0/0	0/0	0/0
Chromosome1-2912701	INS	PRECISE	421	0	0	0/0	0/0	0/0	0/0	0/0	0/1	0/0	0/0	1/1	0/0	0/0	0/0	0/0	0/0
Chromosome1-2914810	INS	PRECISE	5375	0	0	0/0	0/0	0/0	0/1	0/0	0/0	0/0	0/0	0/0	0/0	0/0	0/0	0/0	0/0
Chromosome1-2918070	DEL	PRECISE	4965	0	0	0/0	0/0	0/0	0/0	0/0	0/0	0/0	0/0	0/1	0/0	0/1	0/0	0/1	0/0
Chromosome1-2925913	INS	PRECISE	419	0	0	0/0	0/0	0/0	0/0	0/0	0/0	0/0	0/0	0/0	0/1	0/0	0/0	0/0	0/0
Chromosome1-2927071	INS	PRECISE	90	0	0	0/0	0/0	0/0	0/0	0/0	0/0	0/0	0/0	0/1	0/0	0/1	0/0	0/1	0/0
Chromosome1-2927335	DEL	PRECISE	313	0	0	0/0	0/0	0/0	0/0	0/0	0/0	0/0	0/0	0/1	0/0	0/1	0/1	0/0	0/0
Chromosome1-2928448	DEL	PRECISE	611	0	0	0/0	0/0	0/0	0/0	0/0	0/0	0/0	0/0	0/1	0/0	0/0	0/0	0/1	0/0
Chromosome1-2928793	DEL	PRECISE	1482	0	0	0/0	0/1	0/1	0/1	0/0	0/0	0/1	0/0	0/0	0/0	0/0	0/0	0/0	0/1
Chromosome1-2930382	DEL	PRECISE	2171	0	0	0/0	0/0	0/0	0/0	0/0	0/0	0/0	0/0	0/0	0/0	0/0	0/0	0/0	0/1
Chromosome1-2932732	BND	PRECISE	0	0.447	0	0/0	0/0	0/1	0/1	0/0	0/0	0/0	0/0	0/0	0/0	0/0	0/0	0/0	0/0
Chromosome1-2933663	DEL	PRECISE	287	0	0	0/0	0/0	0/0	0/0	0/0	0/0	0/0	0/0	0/1	0/0	0/0	0/0	0/1	0/0
Chromosome1-2936389	DEL	PRECISE	161	0	0	0/0	0/0	0/0	0/0	0/0	0/0	0/0	0/0	0/1	0/0	0/1	0/1	0/1	0/0
Chromosome1-2939138	DEL	PRECISE	8689	0	0	0/0	0/0	0/0	0/0	0/0	0/0	0/0	0/0	0/0	0/0	0/0	0/0	0/0	0/1
Chromosome1-2946611	INS	PRECISE	323	0	0	0/0	0/0	0/0	0/0	0/0	0/0	0/0	0/0	0/0	0/1	0/0	0/0	0/0	0/0
Chromosome1-2950688	DEL	PRECISE	66	0	0	0/0	0/0	0/0	0/0	0/1	0/1	0/0	0/0	1/1	0/0	0/0	0/0	0/0	0/1
Chromosome1-2953983	DEL	PRECISE	645	0	0	0/0	0/0	0/0	0/0	0/0	0/0	0/0	0/0	0/0	0/1	0/0	0/0	0/0	0/1
Chromosome1-2955779	BND	PRECISE	0	0	0	0/0	0/0	0/0	0/0	0/0	0/0	0/0	0/0	0/0	0/0	0/0	0/0	0/1	0/0
Chromosome1-2955780	INV	PRECISE	22005295	0	0	0/0	0/0	0/0	0/0	0/0	0/0	0/0	0/0	0/0	0/0	0/0	0/0	0/0	0/1
Chromosome1-2955780	INV	PRECISE	11416568	0	0	0/0	0/0	0/0	0/0	0/0	0/0	0/0	0/0	0/0	0/0	0/0	0/0	0/0	0/1
Chromosome1-2964604	DEL	PRECISE	13192625	0	0	0/0	0/0	0/0	0/0	0/0	0/0	0/0	0/0	0/0	0/1	0/0	0/0	0/0	0/1
Chromosome1-2964604	BND	PRECISE	0	0	0	0/0	0/0	0/0	0/0	0/0	0/0	0/0	0/0	0/0	0/0	0/0	0/0	0/0	0/1
Chromosome1-2968529	BND	PRECISE	0	0	0	0/0	0/0	0/0	0/0	0/0	0/0	0/1	0/0	0/0	0/0	0/0	0/0	0/0	0/0
Chromosome1-2969461	DEL	PRECISE	51	0	0	0/0	0/0	0/0	0/0	0/0	0/0	0/0	0/1	0/0	0/0	0/0	0/0	0/0	0/0
Chromosome1-2985497	INS	PRECISE	540	0	0	0/0	0/0	0/0	0/0	0/0	0/0	0/0	0/0	0/0	0/1	0/0	0/0	0/0	0/0
Chromosome1-2986636	DEL	PRECISE	830	0	0	0/0	0/0	0/0	0/0	0/0	0/0	0/0	0/0	0/1	0/0	0/1	0/1	0/1	0/0
Chromosome1-2995782	INS	PRECISE	419	0	0.535	0/0	0/1	0/1	0/1	0/1	0/1	0/1	0/0	0/0	0/0	0/0	0/0	0/0	0/1
Chromosome1-2997450	DEL	PRECISE	111	0	0	0/0	0/0	0/0	0/0	0/0	0/0	0/0	0/0	0/1	0/0	0/1	0/0	0/1	0/0
Chromosome1-3013195	DEL	PRECISE	1669	0.302	0.302	0/0	0/1	0/1	0/1	0/1	0/1	0/1	0/1	0/1	0/0	0/1	0/0	0/1	1/1
Chromosome1-3018425	INS	PRECISE	619	0	0	0/0	0/0	0/0	0/0	0/0	0/0	0/0	0/0	0/0	0/0	0/0	0/0	1/1	0/0
Chromosome1-3018487	DEL	PRECISE	2494	0	0	0/0	0/0	0/0	0/0	0/0	0/0	0/0	0/0	0/1	0/0	0/1	0/0	0/0	0/0
Chromosome1-3019524	DEL	PRECISE	709	0	0	0/0	0/1	0/1	0/1	0/1	0/1	0/1	0/1	0/0	0/0	0/0	0/0	0/1	1/1
Chromosome1-3020869	DEL	PRECISE	112	0	0	0/0	0/0	0/0	0/0	0/0	0/0	0/0	0/0	0/0	0/0	0/0	0/0	0/1	0/0
Chromosome1-3025068	INS	PRECISE	451	0	0.957	0/0	0/0	0/0	0/0	0/0	0/0	0/0	0/0	0/1	0/0	0/1	0/0	0/1	0/0
Chromosome1-3026283	INS	PRECISE	702	0	0.707	0/0	0/0	0/0	0/1	0/0	0/0	0/0	0/0	0/0	0/1	0/0	0/0	0/0	0/0
Chromosome1-3034024	INS	PRECISE	350	0	0	0/0	0/0	0/0	0/0	0/0	0/0	0/0	0/0	0/0	0/0	0/0	0/0	1/1	0/0
Chromosome1-3039421	INS	PRECISE	5164	0	0	0/0	0/0	0/0	0/0	1/1	0/0	0/0	0/0	0/0	0/0	0/0	0/0	0/0	0/0
Chromosome1-3046337	DEL	PRECISE	150	0	0	0/0	0/0	0/0	0/0	0/0	0/0	0/0	0/0	0/0	0/0	0/1	0/0	0/1	0/0
Chromosome1-3047424	INS	PRECISE	119	0	0.577	0/0	0/0	0/0	0/0	0/0	0/0	0/0	0/0	0/0	0/0	0/1	0/0	0/0	0/0
Chromosome1-3050831	INS	PRECISE	235	0	0	0/0	0/0	0/0	0/0	0/0	0/0	0/1	0/1	0/0	0/0	0/0	0/0	0/0	0/0
Chromosome1-3060152	INS	PRECISE	99	0	0.577	0/0	0/0	0/0	0/0	0/0	0/0	0/0	0/0	0/1	0/0	0/1	0/1	0/0	0/0
Chromosome1-3064794	INS	PRECISE	162	0	0	0/0	0/0	0/0	0/0	0/0	0/0	0/0	0/0	0/0	0/0	0/0	0/0	0/1	0/0
Chromosome1-3065985	INS	PRECISE	14863	0	0	0/0	0/0	0/0	0/0	0/0	0/0	0/0	0/0	0/0	0/0	0/0	0/0	1/1	0/0
Chromosome1-3069247	DEL	PRECISE	88	0	0	0/0	0/1	0/1	1/1	0/1	0/0	1/1	1/1	0/0	0/1	0/0	0/0	0/0	0/1
Chromosome1-3069968	INS	PRECISE	132	0	0	0/0	0/0	0/0	0/0	0/0	0/0	0/1	0/1	0/0	0/0	0/0	0/0	0/0	0/0
Chromosome1-3090401	DEL	PRECISE	129	0	0	0/0	0/1	0/1	0/1	0/1	0/1	0/1	0/1	0/0	0/0	0/0	0/0	0/0	0/1
Chromosome1-3091666	INS	PRECISE	323	0	0	0/0	0/0	0/0	0/0	0/0	0/0	0/0	0/0	0/0	0/0	0/0	0/0	0/0	0/1

Chromosome1-3126105	INS	PRECISE	196	0	0	0/0	0/0	0/0	0/0	0/0	0/0	0/0	0/0	0/0	0/0	0/0	0/0	0/0	0/1
Chromosome1-3141844	INS	PRECISE	7162	0	0	0/0	0/0	0/0	0/0	0/0	0/0	0/0	0/0	0/0	1/1	0/0	0/0	0/0	0/0
Chromosome1-3178433	DEL	PRECISE	3216	0	0	0/0	0/1	0/1	0/1	0/1	0/1	0/1	0/1	0/1	0/0	0/1	0/0	0/0	0/1
Chromosome1-3181754	INS	PRECISE	4863	0	0	0/0	0/0	0/0	0/0	0/0	0/0	0/0	0/0	0/0	0/0	0/0	0/0	0/0	1/1
Chromosome1-3201126	INS	PRECISE	58	0	0.378	0/0	0/0	0/0	0/0	0/0	0/0	0/1	0/1	0/1	0/0	0/1	0/1	0/1	0/1
Chromosome1-3201736	DEL	PRECISE	81	0	0	0/0	0/0	0/0	0/0	0/0	0/0	0/0	0/0	1/1	0/0	0/1	0/1	0/1	0/0
Chromosome1-3201835	DEL	PRECISE	2139	0	0	0/0	0/0	0/0	0/1	0/0	0/0	0/1	0/1	0/0	0/0	0/0	0/0	0/0	0/0
Chromosome1-3202503	DEL	PRECISE	441	0	0	0/0	0/0	0/0	0/0	0/0	0/0	0/0	0/0	1/1	0/0	0/1	0/1	0/1	0/0
Chromosome1-3202942	INS	PRECISE	68	0	0	0/0	0/1	0/1	0/1	1/1	1/1	0/1	0/1	0/0	0/1	0/0	0/0	0/0	1/1
Chromosome1-3205043	DEL	PRECISE	1281	0	0	0/0	0/0	0/0	0/1	0/0	0/0	0/1	0/1	1/1	0/0	0/1	0/1	0/1	0/0
Chromosome1-3205550	DEL	PRECISE	71	0	0	0/0	0/0	0/0	0/0	0/0	0/0	0/0	0/0	0/0	0/0	0/0	0/0	0/0	0/1
Chromosome1-3206442	INS	PRECISE	121	0	0	0/0	0/0	0/0	0/0	0/0	0/0	0/0	0/0	0/0	0/0	0/0	0/0	0/0	0/1
Chromosome1-3214167	INS	PRECISE	115	0	0.577	0/0	0/0	0/0	0/0	0/0	0/0	0/0	0/0	0/1	0/0	0/1	0/0	0/1	0/0
Chromosome1-3219044	INS	PRECISE	993	0	0	0/0	0/0	0/0	0/1	0/0	0/0	0/0	0/0	0/0	0/0	0/0	0/0	0/0	0/0
Chromosome1-3222389	INS	PRECISE	622	0	0.577	0/0	0/0	0/0	0/0	0/1	0/1	0/0	0/0	0/1	0/0	0/0	0/0	0/0	0/0
Chromosome1-3235093	INS	PRECISE	121	0	0	0/0	0/0	0/0	0/0	0/0	0/0	0/0	0/0	0/1	0/0	0/1	0/1	0/1	0/0
Chromosome1-3238843	INS	PRECISE	62	0	0	0/0	0/1	0/1	0/1	0/1	0/1	0/1	0/1	0/0	0/0	0/0	0/0	0/0	1/1
Chromosome1-3240379	INS	PRECISE	66	0	0	0/0	0/0	0/0	0/0	0/0	0/0	0/0	0/0	0/1	0/0	0/1	0/1	0/1	0/0
Chromosome1-3245395	INS	PRECISE	97	0	0	0/0	0/0	0/0	0/0	0/0	0/0	0/0	0/0	0/1	0/0	0/1	0/0	0/1	0/0
Chromosome1-3251886	INS	PRECISE	9251	0	0	0/0	0/0	0/0	0/0	0/0	0/0	0/0	0/0	0/0	1/1	0/0	0/0	0/0	0/0
Chromosome1-3253902	DEL	PRECISE	53	0.447	0.447	0/0	0/0	0/0	0/0	0/0	0/0	0/0	0/0	0/1	0/1	0/1	0/1	0/1	0/0
Chromosome1-3256320	INS	PRECISE	53	0	0.5	0/0	0/0	0/0	0/0	0/0	0/0	0/0	0/0	0/1	0/0	0/1	1/1	0/1	0/0
Chromosome1-3282262	DEL	PRECISE	107	0	0	0/0	0/0	0/0	0/1	0/0	0/0	0/0	0/0	0/0	0/0	0/0	0/0	0/0	0/0
Chromosome1-3287494	DEL	PRECISE	132	0	0	0/0	0/0	0/0	0/0	0/0	0/0	0/0	0/0	0/1	0/0	0/0	0/1	0/1	0/0
Chromosome1-3295447	INS	PRECISE	483	0	0.5	0/0	0/0	0/0	0/0	0/0	0/0	0/0	0/0	0/1	0/0	0/0	0/1	0/1	0/0
Chromosome1-3299218	DEL	PRECISE	710	0	0	0/0	0/0	0/0	0/0	0/0	0/0	0/0	0/0	0/1	0/0	0/1	0/1	0/1	0/0
Chromosome1-3303190	INS	PRECISE	135	0	0	0/0	0/0	0/0	0/0	0/1	0/1	0/0	0/0	0/1	0/0	0/0	0/0	0/0	0/0
Chromosome1-3323797	INV	PRECISE	1929	0	0	0/0	0/0	0/0	0/0	0/0	0/1	0/0	0/0	0/0	0/0	0/0	0/0	0/0	0/0
Chromosome1-3324135	DEL	PRECISE	1591	0	0	0/0	0/0	0/0	0/0	0/1	0/1	0/0	0/0	0/1	0/0	0/0	0/0	0/0	0/0
Chromosome1-3329718	DEL	PRECISE	760	0	0	0/0	0/1	0/1	0/1	0/1	0/1	0/1	0/1	0/1	0/0	0/1	0/1	0/1	0/1
Chromosome1-3340021	INS	PRECISE	10879	0	0	0/0	0/0	0/0	0/0	0/0	0/0	0/0	0/0	0/0	0/0	0/0	0/0	0/0	1/1
Chromosome1-3341978	DEL	PRECISE	55	0	0	0/0	0/0	0/0	0/0	0/0	0/0	0/0	0/0	0/1	0/0	0/1	0/0	0/1	0/0
Chromosome1-3365484	DEL	PRECISE	785	0	0	0/0	0/0	0/0	0/0	0/0	0/0	0/0	0/0	0/1	0/0	0/1	0/1	0/1	0/0
Chromosome1-3368922	INS	PRECISE	91	0	0	0/0	0/1	0/1	1/1	1/1	1/1	1/1	1/1	1/1	0/0	0/1	0/1	0/1	1/1
Chromosome1-3371778	DEL	PRECISE	50	0	0	0/0	0/0	0/0	0/0	0/0	0/0	0/0	0/0	0/0	0/0	0/0	0/0	0/0	0/1
Chromosome1-3379861	DEL	PRECISE	238	0	0	0/0	0/0	0/0	0/0	0/0	0/0	0/0	0/0	0/1	0/0	0/1	0/1	0/1	0/0
Chromosome1-3384166	INS	PRECISE	228	0	0.5	0/0	0/0	0/0	0/0	0/0	0/0	0/0	0/0	0/1	0/0	0/1	1/1	0/1	0/0
Chromosome1-3395852	INS	PRECISE	323	0	0.5	0/0	0/0	0/0	0/0	0/0	0/0	0/0	0/0	0/1	0/0	0/1	0/1	0/1	0/0
Chromosome1-3396822	DEL	PRECISE	416	0	0	0/0	0/0	0/0	0/0	0/0	0/0	0/0	0/0	0/1	0/0	1/1	1/1	1/1	0/0
Chromosome1-3422873	INS	PRECISE	211	0	0	0/0	0/0	0/0	0/0	0/0	0/0	0/0	0/0	0/0	0/0	0/0	0/0	0/0	0/1
Chromosome1-3425396	DEL	PRECISE	121	0	0	0/0	0/0	0/0	0/0	0/0	0/0	0/0	0/0	0/1	0/0	0/1	0/1	0/0	0/0
Chromosome1-3425980	INS	PRECISE	270	0	0	0/0	0/0	0/0	0/0	0/0	0/0	0/0	0/0	0/1	0/0	0/1	0/1	0/0	0/0
Chromosome1-3432796	BND	PRECISE	0	0	0	0/0	0/0	0/0	1/1	0/1	0/1	0/1	1/1	1/1	1/1	0/0	0/0	0/0	0/0
Chromosome1-3454580	DEL	PRECISE	1900	0	0	0/0	0/1	0/1	0/1	1/1	1/1	1/1	1/1	0/0	0/0	0/0	0/0	0/0	0/1
Chromosome1-3455418	INS	PRECISE	977	0	0.577	0/0	0/0	0/0	0/0	0/0	0/0	0/0	0/0	0/1	0/0	0/1	0/0	0/1	0/0
Chromosome1-3460643	INS	PRECISE	1307	0	0.707	0/0	0/0	0/0	0/1	0/0	0/0	0/0	0/0	1/1	0/0	0/0	0/0	0/0	0/0
Chromosome1-3461867	DEL	PRECISE	885	0.447	0.447	0/0	0/0	0/0	0/0	0/0	0/0	0/1	0/1	1/1	0/0	0/0	0/0	0/0	0/0
Chromosome1-3465245	BND	PRECISE	0	0	0	0/0	0/0	0/0	0/0	0/0	0/0	0/0	0/0	0/1	0/0	0/1	0/1	0/0	0/0
Chromosome1-3467892	DEL	PRECISE	73	0	0	0/0	0/0	0/0	0/0	0/0	0/0	0/0	0/0	0/1	0/0	0/1	1/1	0/1	0/0
Chromosome1-3470617	INS	PRECISE	937	0	0	0/0	0/0	0/0	0/0	0/0	0/0	0/0	0/0	0/0	0/0	0/0	0/0	0/0	0/1
Chromosome1-3478261	INS	PRECISE	248	0	0.447	0/0	0/0	0/0	0/0	0/1	0/1	0/1	0/1	0/1	0/0	0/0	0/0	0/0	0/0
Chromosome1-3478522	DEL	PRECISE	136	0	0	0/0	0/0	0/0	0/0	0/0	0/0	0/0	0/0	0/1	0/0	0/1	0/1	0/1	0/0
Chromosome1-3485923	DEL	PRECISE	647	0	0	0/0	0/1	0/1	1/1	0/1	0/1	0/1	0/1	0/1	0/1	0/1	0/1	0/1	1/1
Chromosome1-3507886	DEL	PRECISE	93	0	0	0/0	0/0	0/0	0/0	0/0	0/0	0/1	0/1	0/0	0/0	0/0	0/0	0/0	0/0
Chromosome1-3509486	INS	PRECISE	2827	0	0	0/0	0/0	1/1	0/0	0/0	0/0	0/0	0/0	0/0	0/0	0/0	0/0	0/0	0/0
Chromosome1-3510266	DEL	PRECISE	72	0	0	0/0	0/0	0/0	0/0	0/0	0/0	0/0	0/0	0/1	0/0	0/1	0/1	0/1	0/0
Chromosome1-3514538	INS	PRECISE	213	0	0.5	0/0	0/0	0/0	0/0	0/0	0/1	0/0	0/0	0/1	0/1	0/0	0/0	0/0	0/0
Chromosome1-3514587	DEL	PRECISE	830	0	0	0/0	0/0	0/0	0/0	0/0	0/0	0/0	0/0	0/1	0/0	0/1	0/1	0/1	0/1
Chromosome1-3524065	INS	PRECISE	5523	0	0	0/0	0/0	0/0	1/1	0/0	0/0	0/0	0/0	0/0	0/0	0/0	0/0	0/0	0/0
Chromosome1-3524306	INS	PRECISE	89	0	0.5	0/0	0/0	0/0	0/0	0/0	0/0	0/0	0/0	0/1	0/0	1/1	0/1	0/1	0/0
Chromosome1-3532113	INS	PRECISE	657	0	0	0/0	0/0	0/0	0/0	0/0	0/0	0/0	0/0	0/0	0/0	0/0	0/0	0/0	0/1
Chromosome1-3549481	DEL	PRECISE	1311	0	0	0/0	0/0	0/0	0/0	0/0	0/0	0/0	0/0	0/1	0/0	0/1	0/1	0/1	0/0
Chromosome1-3558390	INS	PRECISE	132	0	0.5	0/0	0/0	0/0	0/0	0/0	0/0	0/0	0/0	0/1	0/0	0/1	0/1	0/0	0/0
Chromosome1-3562103	DEL	PRECISE	274	0	0	0/0	0/0	0/0	0/0	0/0	0/0	0/0	0/0	0/0	0/1	0/0	0/0	0/0	0/0
Chromosome1-3565062	DEL	PRECISE	316	0	0	0/0	0/0	0/0	0/0	0/0	0/0	0/0	0/0	0/1	0/0	0/1	0/1	0/1	0/0
Chromosome1-3567410	INS	PRECISE	142	0	0.5	0/0	0/0	0/0	0/0	0/0	0/0	0/0	0/0	0/1	0/0	0/1	0/1	0/1	0/0
Chromosome1-3577930	DEL	PRECISE	94	0	0	0/0	0/0	0/0	0/0	0/0	0/0	0/0	0/0	0/1	0/0	0/1	0/1	0/1	0/0
Chromosome1-3579044	DEL	PRECISE	80	0	0	0/0	0/0	0/0	0/0	0/0	0/0	0/1	0/1	0/0	0/0	0/0	0/0	0/0	0/0
Chromosome1-3580394	DEL	PRECISE	203	0	0	0/0	0/0	0/0	0/0	0/0	0/0	0/0	0/0	0/1	0/0	0/1	0/1	0/1	0/0
Chromosome1-3586712	INS	PRECISE	381	0	0	0/0	0/0	0/0	0/0	0/0	0/0	0/0	0/0	0/0	1/1	0/0	0/0	0/0	0/1
Chromosome1-3587802	INS	PRECISE	363	0	0	0/0	0/0	0/0	0/0	0/0	0/0	0/1	0/1	0/0	0/1	0/0	0/0	0/0	0/0
Chromosome1-3597230	INS	PRECISE	285	0	0	0/0	0/0	0/0	0/0	0/0	0/0	0/0	0/0	0/0	0/0	0/0	0/0	0/0	0/1
Chromosome1-3611257	INS	PRECISE	161	0	0	0/0	0/0	0/0	0/0	0/0	0/0	0/0	0/0	0/0	0/0	0/0	0/0	0/0	0/1

Chromosome1-3616956	INS	PRECISE	212	0	0.577	0/0	0/0	0/0	0/0	0/0	0/0	0/0	0/0	0/1	0/0	0/1	0/1	0/1	0/0
Chromosome1-3623965	DUP	PRECISE	21774276	0	0	0/0	0/0	0/0	0/0	0/0	0/0	0/0	0/0	0/1	0/0	0/1	0/0	0/0	0/0
Chromosome1-3631754	INS	PRECISE	49	0	0.354	0/0	0/1	0/1	0/1	0/1	0/1	0/1	0/1	0/0	0/0	0/0	0/0	0/0	0/1
Chromosome1-3633428	DEL	PRECISE	70	0	0	0/0	0/1	0/1	0/1	0/1	0/1	0/1	0/1	0/0	0/0	0/0	0/0	0/0	0/1
Chromosome1-3634811	INS	PRECISE	1184	0	0	0/0	0/0	0/0	0/0	0/0	0/0	0/0	0/0	0/0	0/1	0/0	0/0	0/0	0/0
Chromosome1-3660685	DEL	PRECISE	51	0	0	0/0	0/0	0/0	0/0	0/0	0/0	0/0	0/0	0/1	0/0	0/0	0/0	0/0	0/0
Chromosome1-3671141	INS	PRECISE	63	0	0.439	0/0	0/1	0/1	0/0	1/1	1/1	1/1	1/1	1/1	0/1	0/1	0/1	0/1	1/1
Chromosome1-3671703	DEL	PRECISE	67	0	0	0/0	0/0	0/0	1/1	0/0	0/0	0/0	0/0	0/0	0/0	0/0	0/0	0/0	0/0
Chromosome1-3677808	DEL	PRECISE	56	0	0	0/0	0/0	0/0	0/0	0/0	0/0	0/0	0/0	0/1	0/0	0/1	0/0	0/1	0/0
Chromosome1-3678888	BND	PRECISE	0	0	0	0/0	0/0	0/0	0/0	0/0	0/0	0/0	0/0	0/1	0/0	0/0	0/0	0/1	0/0
Chromosome1-3680962	DEL	PRECISE	1241	0	0	0/0	0/0	0/0	0/0	0/0	0/0	0/0	0/0	0/1	0/0	0/1	0/1	0/1	0/0
Chromosome1-3681169	INS	PRECISE	449	0	0	0/0	0/0	0/0	0/1	0/0	0/0	0/0	0/0	0/0	0/0	0/0	0/0	0/0	0/0
Chromosome1-3699466	DEL	PRECISE	100	0	0	0/0	0/0	0/0	0/0	0/0	0/0	0/0	0/0	0/1	0/0	0/1	0/1	0/1	0/0
Chromosome1-3705165	BND	PRECISE	0	0	0	0/0	0/0	0/0	0/0	0/1	0/0	0/0	0/0	0/0	0/0	0/0	0/0	0/0	0/0
Chromosome1-3706547	INS	PRECISE	950	0	0.886	0/0	0/1	0/1	0/1	0/1	0/1	0/1	0/1	0/0	0/0	0/0	0/0	0/0	0/1
Chromosome1-3707101	DEL	PRECISE	351	0	0	0/0	0/0	0/0	0/0	0/0	0/0	0/0	0/0	0/1	0/0	0/1	0/1	0/1	0/0
Chromosome1-3715465	DEL	PRECISE	53	0	0	0/0	0/0	0/0	0/0	0/0	0/0	0/1	0/1	0/0	0/0	0/0	0/0	0/0	0/0
Chromosome1-3717600	INS	PRECISE	1132	0	0.957	0/0	0/0	0/0	0/0	0/0	0/0	0/0	0/0	0/1	0/0	0/1	0/1	0/1	0/0
Chromosome1-3718142	INS	PRECISE	152	0	0.5	0/0	0/0	0/0	0/0	0/0	0/0	0/0	0/0	0/1	0/0	0/1	0/1	0/1	0/0
Chromosome1-3723085	DEL	PRECISE	987	0	0	0/0	0/0	0/0	0/0	0/0	0/0	0/0	0/0	0/1	0/0	0/1	0/1	0/1	0/0
Chromosome1-3723329	INS	PRECISE	656	0	0.577	0/0	0/0	0/0	0/0	0/0	0/0	0/1	0/1	0/0	0/1	0/0	0/0	0/0	0/0
Chromosome1-3725156	DEL	PRECISE	711	0	0	0/0	0/1	0/1	0/1	0/1	0/1	0/1	0/1	0/1	0/0	0/1	0/1	0/1	0/1
Chromosome1-3728134	DEL	PRECISE	527	0	0	0/0	0/0	0/0	0/0	0/0	0/0	0/1	0/1	0/0	0/0	0/0	0/0	0/0	0/0
Chromosome1-3730907	DEL	PRECISE	542	0	0	0/0	0/0	0/0	0/0	0/0	0/0	0/0	0/0	0/1	0/0	0/1	0/1	0/1	0/1
Chromosome1-3732863	DEL	PRECISE	88	0	0	0/0	0/1	0/1	1/1	1/1	1/1	1/1	1/1	0/1	0/0	0/0	0/0	0/0	1/1
Chromosome1-3736576	INS	PRECISE	558	0	0.577	0/0	0/0	0/0	0/0	0/0	0/0	0/1	1/1	0/0	0/1	0/0	0/0	0/0	0/0
Chromosome1-3737914	INS	PRECISE	127	0	0	0/0	0/1	0/1	0/1	0/1	0/1	0/1	0/0	0/0	0/0	0/0	0/0	0/0	0/1
Chromosome1-3741802	INS	PRECISE	979	0	0	0/0	0/0	0/0	0/0	0/0	0/0	0/0	0/0	0/0	0/0	0/0	0/0	0/0	0/1
Chromosome1-3743579	INS	PRECISE	6370	0	0	0/0	0/0	0/0	0/1	0/0	0/0	0/0	0/0	0/0	0/0	0/0	0/0	0/0	0/0
Chromosome1-3750415	DEL	PRECISE	50	0	0	0/0	0/1	0/1	0/1	0/1	0/1	0/1	0/1	0/0	0/0	0/0	0/0	0/0	1/1
Chromosome1-3751589	INS	PRECISE	5285	0	0	0/0	0/0	0/0	0/0	0/0	0/0	0/0	0/0	0/0	0/0	0/0	0/0	0/0	1/1
Chromosome1-3762437	INS	PRECISE	785	0	0.5	0/0	0/0	0/0	0/0	0/0	0/0	0/0	0/0	0/1	0/0	0/1	0/1	0/1	0/0
Chromosome1-3764539	DEL	PRECISE	1799	0	0	0/0	0/1	0/1	1/1	1/1	1/1	1/1	1/1	1/1	0/1	0/1	0/1	0/1	1/1
Chromosome1-3768075	INS	PRECISE	85	0	0	0/0	0/0	0/0	0/0	0/0	0/0	0/0	0/0	0/1	0/0	0/1	0/1	0/1	0/0
Chromosome1-3769912	INS	PRECISE	5436	0	0	0/0	0/0	0/0	0/0	0/0	0/0	0/0	0/0	0/0	0/0	0/0	0/0	0/0	1/1
Chromosome1-3770886	DEL	PRECISE	5151	0	0	0/0	0/0	0/0	0/0	0/0	0/0	0/0	0/0	0/1	0/0	0/1	0/1	0/1	0/0
Chromosome1-3772900	INS	PRECISE	1366	0	0	0/0	0/0	0/0	0/0	0/0	0/0	0/0	0/0	0/0	0/0	0/0	0/0	0/0	0/1
Chromosome1-3788558	INS	PRECISE	482	0	0	0/0	0/0	0/0	0/0	0/0	0/0	0/1	0/1	0/0	0/0	0/0	0/0	0/0	0/0
Chromosome1-3790585	DEL	PRECISE	343	0	0	0/0	0/1	0/1	0/1	0/1	0/1	0/1	0/1	0/0	0/0	0/0	0/0	0/0	1/1
Chromosome1-3792210	DEL	PRECISE	1182	0	0	0/0	0/1	0/1	1/1	1/1	1/1	1/1	1/1	1/1	0/1	0/1	0/1	0/1	1/1
Chromosome1-3794148	INS	PRECISE	211	0	0.577	0/0	0/0	0/0	0/0	0/1	0/1	0/0	0/0	0/1	0/0	0/0	0/0	0/0	0/0
Chromosome1-3795063	DEL	PRECISE	65	0	0	0/0	0/0	0/0	0/0	0/0	0/0	0/0	0/0	0/1	0/0	0/1	0/1	0/1	0/0
Chromosome1-3804494	DEL	PRECISE	66	0	0	0/0	0/0	0/0	0/0	0/0	0/0	0/1	0/1	0/0	0/0	0/0	0/0	0/0	0/0
Chromosome1-3812235	INS	PRECISE	211	0	0	0/0	0/0	0/0	0/0	0/0	0/0	0/0	0/0	0/1	0/0	0/1	0/1	0/1	0/0
Chromosome1-3813833	DEL	PRECISE	54	0	0	0/0	0/0	0/0	0/1	0/0	0/0	0/1	0/1	0/0	0/1	0/0	0/0	0/0	0/0
Chromosome1-3819622	INS	PRECISE	151	0	0	0/0	0/0	0/0	0/0	0/0	0/0	0/0	0/0	0/0	0/0	0/0	0/0	0/0	0/1
Chromosome1-3831460	DEL	PRECISE	52	0	0	0/0	0/0	0/0	0/0	0/0	0/0	0/0	0/0	0/1	0/0	0/1	0/1	0/1	0/0
Chromosome1-3831879	INS	PRECISE	50	0	0.5	0/0	0/0	0/0	0/0	0/0	0/0	0/0	0/0	0/1	0/0	0/1	0/1	0/1	0/0
Chromosome1-3833992	INS	PRECISE	56	0	0	0/0	0/0	0/0	0/0	0/0	0/0	0/0	0/0	0/1	0/0	0/1	0/1	0/1	0/0
Chromosome1-3842360	INS	PRECISE	134	0	0.577	0/0	0/0	0/0	0/0	0/1	0/1	0/0	0/0	0/1	0/0	0/0	0/0	0/0	0/0
Chromosome1-3856590	DEL	PRECISE	594	0	0	0/0	0/0	0/0	0/0	0/0	0/0	0/0	0/0	0/0	0/0	0/0	0/1	0/0	0/0
Chromosome1-3858360	INS	PRECISE	117	0	0	0/0	0/0	0/0	0/0	0/0	0/0	0/0	0/0	0/0	0/0	0/0	0/1	0/0	0/0
Chromosome1-3871181	INS	PRECISE	117	0	0.354	0/0	0/1	0/1	0/1	0/1	0/1	0/1	0/1	0/0	0/0	0/0	0/0	0/0	0/1
Chromosome1-3876301	INS	PRECISE	482	0	0	0/0	0/0	0/0	0/0	0/0	0/0	0/0	0/0	0/0	0/0	0/0	0/0	0/0	0/1
Chromosome1-3888891	INS	PRECISE	74	0	0	0/0	0/0	0/0	0/0	0/0	0/0	0/0	0/0	0/1	0/0	0/1	0/1	0/1	0/0
Chromosome1-3888936	DEL	PRECISE	80	0	0	0/0	0/0	0/0	0/0	0/0	0/0	0/0	0/0	0/1	0/0	0/1	0/1	0/1	0/0
Chromosome1-3891494	INS	PRECISE	55	0	0	0/0	0/0	0/0	0/0	0/0	0/0	0/0	0/0	0/1	0/0	0/1	0/1	0/1	0/0
Chromosome1-3912503	DEL	PRECISE	905	0	0	0/0	0/1	0/1	1/1	1/1	1/1	1/1	1/1	0/1	0/1	0/0	0/0	0/0	1/1
Chromosome1-3912509	INS	PRECISE	288	0	0.957	0/0	0/0	0/0	0/0	0/0	0/0	0/0	0/0	0/1	0/0	0/1	0/1	0/1	0/0
Chromosome1-3913407	DEL	PRECISE	298	0	0	0/0	0/0	0/0	0/0	0/0	0/0	0/0	0/0	0/1	0/0	0/1	0/1	0/1	0/0
Chromosome1-3914317	DEL	PRECISE	1378	0	0	0/0	0/0	0/0	0/0	0/1	0/1	0/0	0/0	1/1	0/1	0/1	0/1	0/1	0/1
Chromosome1-3923377	INS	PRECISE	13834	0	0	0/0	0/0	0/0	0/0	0/0	0/0	0/0	0/0	0/0	0/1	0/0	0/0	0/0	0/0
Chromosome1-3927103	DEL	PRECISE	61	0.333	0	0/0	0/1	0/1	0/1	0/1	0/1	0/1	0/1	0/0	0/1	0/0	0/0	0/0	1/1
Chromosome1-3935613	INS	PRECISE	1481	0	0	0/0	0/0	0/0	0/0	0/0	0/0	0/0	0/0	0/0	0/0	0/0	0/0	0/0	0/1
Chromosome1-3945249	INS	PRECISE	51	0	0	0/0	0/0	0/0	0/0	0/0	0/0	0/0	0/0	0/1	0/0	0/1	0/1	0/0	0/0
Chromosome1-3945827	DEL	PRECISE	1212	0	0	0/0	0/1	0/1	0/1	0/0	0/1	0/0	0/1	0/1	0/1	0/1	0/1	0/0	1/1
Chromosome1-3947441	DEL	PRECISE	353	0	0	0/0	0/0	0/0	0/0	0/0	0/0	0/0	0/0	0/0	0/0	0/0	0/0	0/0	0/1
Chromosome1-3949634	DEL	PRECISE	191	0	0	0/0	0/0	0/0	0/0	0/0	0/0	0/0	0/0	0/1	0/0	0/1	0/0	0/1	0/0
Chromosome1-3954819	DEL	PRECISE	51	0	0	0/0	0/0	0/0	0/0	0/0	0/0	1/1	1/1	0/0	0/0	0/0	0/0	0/0	0/0
Chromosome1-3958546	DEL	PRECISE	74	0	0	0/0	0/0	0/0	0/0	1/1	0/1	0/0	0/0	0/1	0/0	0/0	0/0	0/0	0/0
Chromosome1-3964247	INS	PRECISE	1381	0	0	0/0	0/0	0/0	0/0	0/0	0/0	0/0	0/0	0/0	0/0	0/0	0/0	0/0	0/1
Chromosome1-3977632	DEL	PRECISE	666	0	0	0/0	0/1	0/1	0/1	0/1	0/1	0/1	0/1	0/1	0/1	0/1	0/1	0/1	1/1
Chromosome1-3979221	INS	PRECISE	193	0.894	0.837	0/0	0/0	0/0	0/0	0/0	0/0	0/0	0/0	0/1	0/1	0/1	0/1	0/1	0/0

Chromosome1-3987299	INS	PRECISE	700	0	0	0/0	0/0	0/0	0/0	0/0	0/0	1/1	1/1	0/0	0/0	0/0	0/0	0/0	0/0
Chromosome1-3988664	DEL	PRECISE	1474	0.949	0.949	0/0	1/1	1/1	0/1	1/1	0/0	1/1	0/1	0/0	1/1	0/0	0/0	0/0	1/1
Chromosome1-3991265	INS	PRECISE	741	0	0.919	0/0	0/1	0/1	0/1	1/1	1/1	0/1	0/1	1/1	0/1	0/0	0/0	0/0	0/0
Chromosome1-3992821	INS	PRECISE	295	0	0	0/0	0/0	0/0	0/0	0/0	0/0	0/0	0/0	0/0	0/1	0/0	0/0	0/0	0/0
Chromosome1-4006144	DEL	PRECISE	469	0.354	0.354	0/0	0/1	0/1	0/1	0/1	0/1	0/1	0/1	0/0	0/0	0/0	0/0	0/0	0/1
Chromosome1-4006367	INS	PRECISE	160	0	0	0/0	0/0	0/0	0/0	0/0	0/0	0/0	0/0	0/1	0/0	0/1	0/1	0/1	0/0
Chromosome1-4006482	INS	PRECISE	7196	0	0	0/0	0/0	0/0	0/0	0/0	0/0	0/0	0/0	0/0	0/0	0/0	0/0	0/0	1/1
Chromosome1-4006687	INS	PRECISE	1548	0	0.707	0/0	0/0	0/0	0/0	0/0	0/0	0/1	0/1	0/0	0/0	0/0	0/0	0/0	0/0
Chromosome1-4009327	INS	PRECISE	1197	0	0	0/0	0/0	0/0	0/0	0/0	0/0	0/0	0/0	0/0	0/0	0/0	0/0	0/0	0/1
Chromosome1-4024126	DEL	PRECISE	48511	0	0	0/0	1/1	0/1	1/1	0/1	1/1	1/1	1/1	0/0	0/0	0/0	0/0	0/0	0/0
Chromosome1-4032030	DEL	PRECISE	3390	0	0	0/0	0/0	0/0	0/1	0/0	0/0	0/0	0/0	0/0	0/0	0/0	0/0	0/0	0/0
Chromosome1-4034665	DEL	PRECISE	1310	0	0	0/0	0/0	0/0	0/0	0/0	0/0	0/0	0/0	0/0	0/0	0/0	0/0	0/1	0/0
Chromosome1-4034678	INS	PRECISE	51	0	0.289	0/0	0/1	0/1	0/1	0/0	0/1	0/1	0/1	0/1	0/1	0/1	0/1	0/1	1/1
Chromosome1-4037763	DEL	PRECISE	58	0	0	0/0	0/0	0/0	0/0	0/0	0/0	0/0	0/0	0/0	0/1	0/0	0/0	0/0	0/0
Chromosome1-4039257	INS	PRECISE	137	0.408	0.408	0/0	0/0	0/0	0/0	1/1	1/1	1/1	1/1	1/1	0/1	0/0	0/0	0/0	0/0
Chromosome1-4047374	DEL	PRECISE	125	0	0	0/0	0/0	0/0	0/0	0/0	0/0	0/0	0/0	0/0	0/1	0/0	0/0	0/0	0/0
Chromosome1-4058831	BND	PRECISE	0	0	0	0/0	0/0	0/0	0/0	0/0	0/0	0/0	0/0	0/0	0/0	0/1	0/0	0/1	0/0
Chromosome1-4067241	INS	PRECISE	61	0	0	0/0	0/0	0/0	0/0	1/1	1/1	0/0	0/0	1/1	0/1	0/0	0/0	0/0	0/0
Chromosome1-4080768	DEL	PRECISE	173	0	0	0/0	0/0	0/0	0/0	0/0	0/0	0/0	0/0	0/1	0/0	0/1	0/1	0/1	0/0
Chromosome1-4085982	DEL	PRECISE	192	0	0	0/0	0/0	0/0	0/0	0/0	0/0	0/0	0/0	0/1	0/0	0/1	0/1	0/1	0/0
Chromosome1-4088559	INS	PRECISE	133	0	0.5	0/0	0/0	0/0	0/0	0/0	0/0	0/0	0/0	0/1	0/0	0/1	0/0	0/0	0/0
Chromosome1-4090873	INS	PRECISE	109	0	0	0/0	0/0	0/0	0/0	0/0	0/0	0/0	0/0	0/1	0/0	0/1	0/1	0/0	0/0
Chromosome1-4091645	DEL	PRECISE	908	0	0	0/0	0/0	0/0	0/0	0/0	0/0	0/0	0/0	0/1	0/0	0/1	0/1	0/0	0/0
Chromosome1-4091921	INS	PRECISE	212	0	0	0/0	0/0	0/0	0/0	0/0	0/0	0/0	0/0	0/0	0/0	0/0	0/0	0/0	0/1
Chromosome1-4098294	INS	PRECISE	130	0	0	0/0	0/0	0/0	0/0	0/0	0/0	0/0	0/0	0/0	0/1	0/0	0/0	0/0	0/0
Chromosome1-4101378	INS	PRECISE	211	0	0.354	0/0	0/1	0/1	0/1	0/1	0/1	0/1	0/1	0/0	0/0	0/0	0/0	0/0	0/1
Chromosome1-4109410	DEL	PRECISE	66	0	0	0/0	0/0	0/0	0/0	0/0	0/0	0/0	0/0	0/1	0/0	0/1	0/1	0/1	0/0
Chromosome1-4112974	INS	PRECISE	322	0	0	0/0	0/0	0/0	0/0	0/0	0/0	0/0	0/0	0/0	0/0	0/0	0/0	0/0	0/1
Chromosome1-4117732	INS	PRECISE	126	0	0.5	0/0	0/0	0/0	0/0	0/0	0/0	0/0	0/0	0/1	0/0	0/1	0/1	0/1	0/0
Chromosome1-4117929	DEL	PRECISE	1487	0	0	0/0	0/0	0/0	0/0	0/0	0/0	0/0	0/0	0/1	0/0	0/1	0/1	0/1	0/0
Chromosome1-4119625	DEL	PRECISE	215	0	0	0/0	0/0	0/0	0/0	0/0	0/0	0/0	0/0	0/1	0/0	0/1	0/1	0/1	0/0
Chromosome1-4137712	BND	PRECISE	0	0	0	0/0	0/0	0/0	0/1	0/0	0/0	0/0	0/0	0/0	0/0	0/0	0/0	0/0	0/0
Chromosome1-4137712	BND	PRECISE	0	0	0	0/0	0/0	0/0	0/1	0/0	0/0	0/0	0/0	0/0	0/1	0/0	0/0	0/0	0/0
Chromosome1-4149543	DEL	PRECISE	1303	0	0	0/0	0/0	0/0	1/1	0/0	0/1	0/0	0/0	0/0	0/1	0/0	0/0	0/0	0/0
Chromosome1-4168949	INS	PRECISE	994	0	0	0/0	0/0	0/0	0/0	0/0	0/0	0/0	0/0	0/0	0/1	0/0	0/0	0/0	0/0
Chromosome1-4169433	DEL	PRECISE	1556	0	0	0/0	0/1	0/1	0/1	0/1	0/1	0/1	0/1	0/1	0/0	0/1	0/1	0/1	1/1
Chromosome1-4171395	INS	PRECISE	5090	0	0	0/0	0/0	0/0	0/0	0/0	0/0	0/0	0/0	0/0	0/0	0/0	0/0	0/0	0/1
Chromosome1-4173526	DEL	PRECISE	92	0	0	0/0	0/0	0/0	0/0	0/1	0/1	0/1	0/1	0/1	0/0	0/0	0/0	0/0	0/0
Chromosome1-4176031	INS	PRECISE	627	0	0.991	0/0	0/1	0/1	0/1	0/1	0/1	0/1	0/1	0/0	0/0	0/0	0/0	0/0	0/1
Chromosome1-4197277	INS	PRECISE	788	0	0.894	0/0	0/0	0/0	0/0	0/1	0/1	0/1	0/1	0/1	0/0	0/0	0/0	0/0	0/0
Chromosome1-4206062	INS	PRECISE	83	0	0	0/0	0/0	0/0	0/0	0/0	0/0	0/0	0/0	0/1	0/0	0/1	0/1	0/1	0/0
Chromosome1-4210737	BND	PRECISE	0	0	0	0/0	0/0	0/0	0/0	0/0	0/0	0/0	0/0	0/0	0/0	0/0	0/1	0/0	0/0
Chromosome1-4214925	DEL	PRECISE	713	0	0	0/0	0/0	0/0	0/0	0/0	0/0	0/0	0/0	0/1	0/0	0/1	0/1	1/1	0/0
Chromosome1-4236459	INS	PRECISE	199	0	0.354	0/0	0/1	0/0	0/1	0/1	0/1	0/1	0/1	0/0	0/0	0/0	0/0	0/0	0/1
Chromosome1-4242290	BND	PRECISE	0	0	0	0/0	0/0	0/1	0/1	0/1	0/1	0/1	0/1	0/0	0/0	0/0	0/0	0/0	0/1
Chromosome1-4245266	DEL	PRECISE	152	0.957	0.577	0/0	0/0	0/0	0/0	0/0	0/0	0/0	0/0	0/1	0/0	0/0	0/1	0/1	0/0
Chromosome1-4251072	INS	PRECISE	538	0	0.577	0/0	0/0	0/0	0/0	0/1	0/1	0/0	0/0	0/1	0/0	0/0	0/0	0/0	0/0
Chromosome1-4252942	INS	PRECISE	276	0	0.577	0/0	0/0	0/0	0/0	0/0	0/0	0/0	0/0	0/1	0/0	0/0	0/1	0/1	0/0
Chromosome1-4270513	DEL	PRECISE	157	0	0	0/0	0/0	0/0	0/0	0/0	0/0	0/0	0/0	0/1	0/0	0/1	0/1	0/1	0/0
Chromosome1-4272276	INS	PRECISE	183	0	0.5	0/0	0/0	0/0	0/0	0/0	0/0	0/0	0/0	0/1	0/0	0/1	0/1	0/1	0/0
Chromosome1-4272558	INS	PRECISE	5415	0	0	0/0	0/0	0/0	0/0	0/0	0/0	0/0	0/0	0/0	0/0	0/0	0/0	0/0	0/1
Chromosome1-4287825	INS	PRECISE	53	0	0	0/0	0/1	0/1	0/1	0/1	0/1	0/1	0/1	0/0	0/0	0/0	0/0	0/0	0/1
Chromosome1-4292302	DEL	PRECISE	3408	0	0	0/0	0/0	0/0	0/0	0/0	0/0	0/0	0/0	0/0	0/0	0/0	0/0	0/0	0/1
Chromosome1-4296248	INS	PRECISE	840	0	0.707	0/0	0/0	0/0	0/0	0/0	0/0	0/0	0/0	0/0	0/0	0/0	0/0	0/1	0/0
Chromosome1-4300401	INS	PRECISE	442	0	0.866	0/0	0/1	0/1	1/1	1/1	1/1	1/1	1/1	1/1	0/0	0/0	0/0	0/0	0/1
Chromosome1-4301179	DEL	PRECISE	759	0	0	0/0	0/0	0/0	0/0	0/0	0/0	0/0	0/0	0/0	0/0	0/1	0/0	0/1	0/0
Chromosome1-4301500	INS	PRECISE	51	0	0	0/0	0/0	0/0	0/0	0/0	0/0	0/0	0/0	0/0	0/1	0/0	0/0	0/0	0/1
Chromosome1-4308778	INS	PRECISE	276	0	0.957	0/0	0/0	0/0	0/0	0/0	0/0	0/0	0/0	0/1	0/0	0/0	0/1	0/1	0/0
Chromosome1-4313171	DEL	PRECISE	66	0	0	0/0	0/0	0/0	0/0	0/0	0/0	0/0	0/0	0/1	0/0	0/1	0/1	0/1	0/0
Chromosome1-4321442	INS	PRECISE	845	0	0	0/0	0/0	0/0	0/0	0/0	0/0	0/0	0/0	0/1	0/0	0/1	0/1	0/1	0/0
Chromosome1-4325326	DEL	PRECISE	811	0	0	0/0	0/1	0/1	0/1	1/1	1/1	1/1	1/1	1/1	0/0	0/1	0/0	0/1	1/1
Chromosome1-4326407	DEL	PRECISE	354	0	0	0/0	0/0	0/0	0/0	0/0	0/0	0/1	1/1	0/1	0/0	0/1	0/1	0/1	0/0
Chromosome1-4339459	INS	PRECISE	120	0	0.5	0/0	0/0	0/0	0/0	0/0	0/0	0/0	0/0	0/1	0/0	0/1	0/1	1/1	0/0
Chromosome1-4340216	DEL	PRECISE	297	0	0	0/0	0/0	0/0	0/0	0/0	0/0	0/0	0/0	0/1	0/0	0/1	0/1	1/1	0/0
Chromosome1-4355998	DEL	PRECISE	82	0	0	0/0	0/1	0/1	0/1	1/1	1/1	0/1	0/1	0/1	0/0	0/0	0/0	0/0	1/1
Chromosome1-4360763	INS	PRECISE	371	0	0.957	0/0	0/0	0/0	0/0	0/0	0/0	0/0	0/0	0/0	0/0	0/1	0/1	0/1	0/0
Chromosome1-4361679	DEL	PRECISE	53	0	0	0/0	0/0	0/0	0/0	0/0	0/0	0/0	0/0	0/0	0/0	0/1	0/1	0/1	0/0
Chromosome1-4363829	INS	PRECISE	52	0	0	0/0	0/0	0/0	0/0	0/0	0/0	0/0	0/0	0/0	0/0	0/0	0/0	0/0	0/1
Chromosome1-4372007	DEL	PRECISE	105	0	0	0/0	0/0	0/0	0/0	0/0	0/0	0/0	0/0	0/0	0/0	0/0	0/1	0/1	0/0
Chromosome1-4372465	INS	PRECISE	888	0	0	0/0	0/0	0/0	0/0	0/0	0/0	0/0	0/0	0/0	0/0	0/0	0/0	0/0	0/1
Chromosome1-4374398	DEL	PRECISE	7545	0	0	0/0	0/0	0/0	0/0	0/0	0/0	0/0	1/1	0/0	0/0	0/0	0/0	0/0	0/0
Chromosome1-4374417	DEL	PRECISE	1873	0	0	0/0	0/1	0/1	0/1	0/1	0/1	0/1	0/1	0/0	0/0	0/0	0/0	0/0	0/1
Chromosome1-4385322	DEL	PRECISE	112	0.333	0.333	0/0	0/1	0/1	0/1	0/1	0/1	1/1	0/1	0/0	0/1	0/0	0/0	0/0	0/1

Chromosome1-4385423	INS	PRECISE	2062	0	0.957	0/0	0/0	0/0	0/0	0/0	0/0	0/0	0/0	0/1	0/0	0/1	0/1	0/1	0/0
Chromosome1-4390611	INS	PRECISE	626	0	0.5	0/0	0/0	0/0	0/0	0/1	0/1	0/0	0/0	0/1	0/1	0/0	0/0	0/0	0/0
Chromosome1-4394850	DEL	PRECISE	1133	0	0	0/0	0/0	0/0	0/0	0/0	0/0	0/0	0/0	0/1	0/0	0/1	0/1	0/1	0/0
Chromosome1-4402883	DEL	PRECISE	61	0	0	0/0	0/0	0/0	0/0	0/1	0/1	0/0	0/0	0/1	0/0	0/0	0/0	0/0	0/0
Chromosome1-4414111	INS	PRECISE	62	0	0	0/0	0/0	0/0	0/0	0/0	0/0	0/0	0/0	0/1	0/0	0/1	0/1	0/1	0/0
Chromosome1-4415548	DEL	PRECISE	1286	0	0	0/0	0/0	0/0	0/0	0/0	0/0	0/0	0/0	0/1	0/0	0/1	0/1	0/1	0/0
Chromosome1-4417677	INS	PRECISE	615	0	0	0/0	0/0	0/0	0/0	0/0	0/0	0/0	0/0	0/0	0/0	0/0	0/0	0/0	0/1
Chromosome1-4420929	BND	PRECISE	0	0	0	0/0	0/0	0/0	0/0	0/0	0/0	0/0	0/0	0/1	0/0	0/0	0/0	0/0	0/0
Chromosome1-4420929	BND	PRECISE	0	0	0	0/0	0/0	0/0	0/0	0/0	0/0	0/0	0/0	0/1	0/0	0/0	0/0	0/0	0/0
Chromosome1-4421337	DEL	PRECISE	402	0	0	0/0	0/0	0/0	0/0	0/0	0/0	0/0	0/0	1/1	0/0	1/1	0/1	0/1	0/0
Chromosome1-4441637	INS	PRECISE	75	0	0	0/0	0/0	0/0	0/0	0/0	0/0	0/0	0/0	0/1	0/0	0/1	0/1	0/1	0/0
Chromosome1-4453522	DEL	PRECISE	271	0	0	0/0	0/0	0/0	0/1	0/0	0/0	0/0	0/0	0/0	0/0	0/0	0/0	0/0	0/0
Chromosome1-4454735	DEL	PRECISE	117	0	0	0/0	0/0	0/0	0/1	0/0	0/0	0/0	0/0	0/0	0/0	0/0	0/0	0/0	0/0
Chromosome1-4463905	INS	PRECISE	5675	0	0	0/0	0/0	0/0	0/0	0/0	0/0	0/0	0/0	0/0	0/0	0/0	0/0	0/0	0/1
Chromosome1-4465729	DEL	PRECISE	423	0	0	0/0	0/0	0/0	0/0	0/0	0/0	0/0	0/0	0/1	0/0	0/1	0/1	0/1	0/0
Chromosome1-4470619	DEL	PRECISE	61	0	0	0/0	0/0	0/0	0/0	0/0	0/0	0/0	0/0	0/1	0/0	0/1	0/1	0/1	0/0
Chromosome1-4472420	DEL	PRECISE	490	0	0	0/0	0/1	0/1	0/0	0/1	0/1	0/1	0/0	0/1	0/0	0/1	0/1	0/1	1/1
Chromosome1-4477823	INS	PRECISE	553	0	0.577	0/0	0/0	0/0	0/0	0/0	0/0	0/0	0/0	0/1	0/0	0/1	0/1	0/1	0/0
Chromosome1-4482029	INS	PRECISE	876	0	0	0/0	0/0	0/0	0/0	0/0	0/0	0/0	0/0	0/0	0/0	0/0	0/0	0/0	0/1
Chromosome1-4495291	INS	PRECISE	144	0	0.577	0/0	0/0	0/0	0/0	0/0	0/0	0/0	0/0	0/1	0/0	0/1	0/1	0/1	0/0
Chromosome1-4495734	DEL	PRECISE	175	0	0	0/0	0/0	0/0	0/0	0/0	0/0	0/0	0/0	0/1	0/0	0/1	0/1	0/1	0/0
Chromosome1-4498953	DEL	PRECISE	61	0	0	0/0	0/0	0/0	0/0	0/0	0/0	0/0	0/0	0/0	0/0	0/0	0/0	0/0	0/1
Chromosome1-4506578	INS	PRECISE	353	0	0	0/0	0/0	0/0	0/0	0/0	0/0	0/0	0/0	0/0	0/0	0/0	0/0	0/0	0/1
Chromosome1-4512848	INS	PRECISE	1022	0	0	0/0	0/0	0/0	0/1	0/0	0/0	0/0	0/0	0/0	0/0	0/0	0/0	0/0	0/0
Chromosome1-4518220	DEL	PRECISE	688	0	0	0/0	0/0	0/0	0/0	0/0	0/0	0/0	0/0	0/1	0/0	1/1	1/1	1/1	0/0
Chromosome1-4520963	INS	PRECISE	562	0	0.816	0/0	0/0	0/0	0/0	0/0	0/0	0/0	0/0	0/1	0/0	0/1	0/1	0/1	0/0
Chromosome1-4522775	DEL	PRECISE	90	0	0	0/0	0/0	0/0	0/1	0/0	0/0	0/0	0/0	0/0	0/0	0/0	0/0	0/0	0/0
Chromosome1-4523564	INS	PRECISE	121	0	0.5	0/0	0/0	0/0	0/0	0/0	0/0	0/0	0/0	0/1	0/0	0/1	0/1	0/1	0/0
Chromosome1-4524125	INS	PRECISE	871	0	0.816	0/0	0/0	0/0	0/0	0/0	0/0	0/0	0/0	0/1	0/0	0/1	0/1	0/1	0/0
Chromosome1-4535124	DEL	PRECISE	88	0	0	0/0	0/0	0/0	0/0	0/0	0/0	0/0	0/0	0/1	0/0	0/1	0/1	0/1	0/0
Chromosome1-4546868	INS	PRECISE	10719	0	0	0/0	0/0	0/0	1/1	0/0	0/0	0/0	0/0	0/0	0/0	0/0	0/0	0/0	0/0
Chromosome1-4546875	BND	PRECISE	0	0	0	0/0	0/0	0/0	0/1	0/0	0/0	0/0	0/0	0/0	0/0	0/0	0/0	0/0	0/0
Chromosome1-4547655	DEL	PRECISE	173	0	0	0/0	0/0	0/0	0/0	0/0	0/0	0/0	0/0	0/1	0/0	0/1	0/1	0/1	0/0
Chromosome1-4550068	DEL	PRECISE	71	0	0	0/0	0/1	0/1	0/0	0/1	0/1	0/1	0/0	0/0	0/0	0/0	0/0	0/0	1/1
Chromosome1-4550586	INS	PRECISE	898	0	0	0/0	0/0	0/0	0/0	0/0	0/0	0/0	0/0	0/0	0/0	0/0	0/0	0/0	0/1
Chromosome1-4552379	DEL	PRECISE	211	0	0	0/0	0/1	0/1	1/1	1/1	1/1	1/1	0/1	1/1	0/1	0/0	0/0	0/0	1/1
Chromosome1-4555536	INS	PRECISE	401	0	0.516	0/0	0/1	0/1	0/0	0/1	0/1	0/1	0/0	0/0	0/0	0/0	0/0	0/0	1/1
Chromosome1-4558548	DEL	PRECISE	163	0	0	0/0	0/0	0/0	0/0	1/1	1/1	0/0	0/1	0/1	0/0	0/0	0/0	0/0	0/0
Chromosome1-4563334	DEL	PRECISE	140	0	0	0/0	0/0	0/0	0/0	0/0	0/0	0/0	0/0	0/0	0/1	0/0	0/0	0/0	0/0
Chromosome1-4563484	BND	PRECISE	0	0	0	0/0	0/1	0/1	0/0	0/0	0/0	0/1	0/0	0/0	0/0	0/0	0/0	0/0	0/1
Chromosome1-4568491	INS	PRECISE	311	0	0	0/0	0/0	0/0	0/1	0/0	0/0	0/0	0/0	0/0	0/1	0/0	0/0	0/0	0/0
Chromosome1-4589334	INS	PRECISE	438	0	0.894	0/0	0/1	0/1	0/0	0/1	0/1	1/1	0/0	0/0	0/0	0/0	0/0	0/0	1/1
Chromosome1-4602171	DEL	PRECISE	86	0	0	0/0	0/0	0/0	1/1	0/1	0/1	0/0	0/0	1/1	0/1	0/0	0/0	0/0	0/0
Chromosome1-4621575	DEL	PRECISE	163	0	0	0/0	0/0	0/0	0/0	0/1	0/1	0/0	0/0	0/1	0/0	0/0	0/0	0/0	0/0
Chromosome1-4623035	DEL	PRECISE	633	0.378	0.378	0/0	0/0	0/0	1/1	0/1	0/1	1/1	0/1	0/1	1/1	0/0	0/0	0/0	0/0
Chromosome1-4631032	DEL	PRECISE	53	0	0	0/0	0/0	0/0	1/1	0/0	0/0	0/0	0/0	0/0	0/1	0/0	0/0	0/0	0/0
Chromosome1-4631170	INS	PRECISE	65	0	0	0/0	0/0	0/0	0/0	0/0	0/0	0/0	0/0	1/1	0/0	0/1	0/1	0/0	0/0
Chromosome1-4631530	DEL	PRECISE	633	0	0	0/0	0/0	0/0	1/1	0/0	0/0	0/0	0/0	0/0	0/1	0/0	0/0	0/0	0/0
Chromosome1-4633601	INS	PRECISE	57	0	0	0/0	0/0	0/0	0/0	0/0	0/0	0/0	0/0	1/1	0/0	0/1	0/1	0/0	0/0
Chromosome1-4639354	BND	PRECISE	0	0	0	0/0	0/0	0/0	0/1	0/0	0/0	0/0	0/0	0/0	0/1	0/0	0/0	0/0	0/0
Chromosome1-4640879	INS	PRECISE	1471	0	0.707	0/0	0/0	0/0	0/0	0/0	0/0	0/1	0/1	0/0	0/0	0/0	0/0	0/0	0/0
Chromosome1-4643229	INS	PRECISE	86	0	0	0/0	0/0	0/0	0/0	0/0	0/0	0/0	0/0	0/1	0/0	0/1	0/1	0/1	0/0
Chromosome1-4645549	DEL	PRECISE	2735	0	0	0/0	0/0	0/0	0/0	0/0	0/0	0/0	0/0	0/1	0/0	0/1	0/1	0/1	0/0
Chromosome1-4654241	INS	PRECISE	245	0	0.577	0/0	0/0	0/0	0/0	0/0	0/0	0/0	0/0	0/1	0/0	0/1	0/1	0/1	0/0
Chromosome1-4658694	DEL	PRECISE	72	0	0	0/0	0/0	0/0	0/0	0/0	0/0	0/0	0/0	0/1	0/0	1/1	0/1	0/1	0/0
Chromosome1-4670641	DEL	PRECISE	136	0.707	0.707	0/0	0/0	0/0	0/0	0/0	0/0	0/1	0/1	0/0	0/0	0/0	0/0	0/0	0/0
Chromosome1-4677141	DEL	PRECISE	472	0	0	0/0	0/0	0/0	0/1	1/1	1/1	0/1	0/1	1/1	0/1	0/1	0/1	0/1	1/1
Chromosome1-4679774	DEL	PRECISE	150	0	0	0/0	0/0	0/0	0/0	0/0	0/0	0/0	0/0	0/1	0/0	0/1	0/1	0/1	0/0
Chromosome1-4691367	DEL	PRECISE	135	0	0	0/0	0/0	0/0	0/1	0/0	0/0	0/0	0/0	0/0	0/0	0/0	0/0	0/0	0/0
Chromosome1-4699316	DEL	PRECISE	69	0	0	0/0	0/0	0/0	0/0	0/0	0/0	0/0	0/0	0/0	0/0	0/0	0/0	0/0	0/1
Chromosome1-4703051	DEL	PRECISE	63	0	0	0/0	0/0	0/0	0/0	0/1	0/1	0/0	0/0	1/1	0/0	0/0	0/0	0/0	0/0
Chromosome1-4728555	DEL	PRECISE	343	0	0	0/0	0/0	0/0	0/0	0/0	0/0	0/0	0/0	0/1	0/1	0/1	0/0	0/1	0/0
Chromosome1-4739640	INS	PRECISE	230	0	0	0/0	0/0	0/0	0/0	0/0	0/0	0/0	0/0	0/1	0/0	0/1	0/1	0/0	0/0
Chromosome1-4745523	DEL	PRECISE	223	0	0	0/0	0/0	0/0	0/0	0/0	0/0	0/0	0/0	0/1	0/0	0/1	0/1	0/1	0/0
Chromosome1-4755106	DEL	PRECISE	81	0	0	0/0	0/0	0/0	0/0	0/0	0/0	0/0	0/0	0/1	0/0	0/1	0/1	0/1	0/0
Chromosome1-4758881	INS	PRECISE	57	0	0	0/0	0/1	0/1	0/0	0/1	0/1	0/0	0/0	0/0	0/0	0/0	0/0	0/0	0/1
Chromosome1-4764872	INS	PRECISE	700	0	0	0/0	0/0	0/0	0/0	0/0	0/0	0/0	0/0	0/0	0/0	0/0	0/0	0/0	1/1
Chromosome1-4767791	DEL	PRECISE	255	0	0	0/0	0/1	0/1	0/0	0/1	0/1	0/0	0/0	0/0	0/0	0/0	0/0	0/0	1/1
Chromosome1-4768433	DEL	PRECISE	989	0	0	0/0	0/1	0/1	0/0	0/1	0/1	0/0	0/0	0/0	0/0	0/1	0/0	0/1	1/1
Chromosome1-4780991	DEL	PRECISE	56	0	0	0/0	0/0	0/0	0/0	0/0	0/0	0/0	0/0	0/0	0/0	0/1	0/1	0/0	0/0
Chromosome1-4795827	DEL	PRECISE	51	0	0	0/0	0/0	0/0	0/0	0/0	0/0	0/0	0/0	0/1	0/0	0/1	0/1	0/1	0/0
Chromosome1-4800502	DEL	PRECISE	704	0	0	0/0	0/0	0/0	0/0	0/0	0/0	0/0	0/0	0/1	0/0	0/1	0/1	0/1	0/0
Chromosome1-4817450	INS	PRECISE	253	0	0	0/0	0/0	0/0	0/0	0/0	0/0	0/0	0/0	0/1	0/0	0/1	0/1	0/1	0/0

Chromosome1-4820607 DEL	PRECISE	65	0	0	0/0	0/0	0/0	1/1	0/0	0/0	0/0	0/0	0/0	0/0	0/0	0/0	0/0	0/0
Chromosome1-4827935 INS	PRECISE	615	0	0.548	0/0	0/1	0/1	0/0	0/1	0/1	0/0	0/0	0/0	0/0	0/0	0/0	0/0	1/1
Chromosome1-4828996 DEL	PRECISE	10159	0	0	0/0	0/0	0/0	0/0	0/0	0/0	0/0	0/0	0/0	0/0	0/0	0/0	0/0	0/1
Chromosome1-4828998 DEL	PRECISE	8482	0	0	0/0	0/1	0/1	0/0	0/1	0/1	0/0	0/0	0/0	0/0	0/0	0/0	0/0	1/1
Chromosome1-4837629 DEL	PRECISE	1526	0	0	0/0	0/1	0/1	0/0	0/1	0/1	0/0	0/0	0/1	0/0	0/1	0/1	0/1	1/1
Chromosome1-4839751 INS	PRECISE	170	0	0.577	0/0	0/0	0/0	0/0	0/0	0/0	0/0	0/0	0/1	0/0	0/1	0/1	0/1	0/0
Chromosome1-4843832 INS	PRECISE	1183	0	0	0/0	0/0	0/0	0/0	0/0	0/0	0/0	0/0	0/0	0/0	0/0	0/0	0/0	0/1
Chromosome1-4843978 DEL	PRECISE	131	0	0	0/0	0/0	0/0	0/0	0/0	0/0	0/0	0/0	0/1	0/0	0/1	0/1	0/1	0/0
Chromosome1-4861172 DEL	PRECISE	127	0	0	0/0	0/0	0/0	0/0	0/0	0/0	0/0	0/0	0/0	0/1	0/0	0/0	0/0	0/0
Chromosome1-4871561 BND	PRECISE	0	0	0	0/0	0/0	0/0	0/0	0/1	0/1	0/0	0/0	0/0	0/0	0/0	0/0	0/0	0/0
Chromosome1-4873284 INS	PRECISE	739	0	0	0/0	0/0	0/0	0/0	0/0	0/0	0/0	0/0	0/0	0/0	0/0	0/0	0/0	0/1
Chromosome1-4895307 BND	PRECISE	0	0	0	0/0	0/0	0/0	0/0	0/1	0/1	0/0	0/0	0/0	0/0	0/0	0/0	0/0	0/0
Chromosome1-4897284 DEL	PRECISE	325	0	0	0/0	0/0	0/0	0/0	0/0	0/0	0/0	0/0	0/1	0/0	0/0	0/1	0/1	0/0
Chromosome1-4908346 INS	PRECISE	137	0	0	0/0	0/0	0/0	0/0	0/0	0/0	0/0	0/0	1/1	0/0	0/1	0/1	0/1	0/0
Chromosome1-4908508 INS	PRECISE	1100	0	0	0/0	0/0	0/0	0/1	0/0	0/0	0/0	0/0	0/0	0/0	0/0	0/0	0/0	0/0
Chromosome1-4910811 BND	PRECISE	0	0	0	0/0	0/0	0/0	0/0	0/0	0/0	0/0	0/0	0/1	0/0	0/0	0/0	0/0	0/0
Chromosome1-4913841 INS	PRECISE	211	0	0	0/0	0/0	0/0	0/0	0/0	0/0	0/0	0/0	0/0	0/0	0/0	0/0	0/0	0/1
Chromosome1-4920548 BND	PRECISE	0	0	0	0/0	0/0	0/0	0/0	0/1	0/1	0/0	0/0	1/1	0/0	0/0	0/0	0/0	0/0
Chromosome1-4923423 INS	PRECISE	211	0	0.577	0/0	0/0	0/0	0/0	0/0	0/0	0/0	0/0	0/1	0/0	0/0	0/1	0/1	0/0
Chromosome1-4929549 DEL	PRECISE	4202	0	0	0/0	0/0	0/0	0/0	0/0	0/0	0/0	0/0	0/1	0/0	0/1	0/1	0/1	0/0
Chromosome1-4929605 INS	PRECISE	152	0	0	0/0	0/0	0/0	0/0	0/0	0/0	0/0	0/0	0/0	0/1	0/0	0/0	0/0	0/0
Chromosome1-4930835 DEL	PRECISE	1315	0	0	0/0	0/0	0/0	0/0	0/0	0/0	0/0	0/0	0/0	0/0	0/0	0/0	0/0	0/1
Chromosome1-4933751 INS	PRECISE	57	0	0	0/0	0/0	0/0	0/0	0/0	0/0	0/1	0/0	0/0	0/0	0/0	0/0	0/0	0/0
Chromosome1-4939606 INS	PRECISE	489	0	0.5	0/0	0/0	0/0	0/0	0/0	0/0	0/0	0/0	1/1	0/0	0/1	1/1	1/1	0/0
Chromosome1-4940202 DEL	PRECISE	1387	0.751	0.277	0/0	0/1	0/1	0/1	0/1	0/1	0/1	0/1	0/1	0/1	0/1	0/1	0/1	1/1
Chromosome1-4943378 DEL	PRECISE	75	0	0	0/0	0/1	0/1	0/1	0/1	0/1	0/0	0/0	0/0	0/0	0/0	0/0	0/0	0/1
Chromosome1-4943969 INS	PRECISE	85	0	0.5	0/0	0/0	0/0	0/0	0/0	0/0	0/0	0/0	0/1	0/0	0/1	0/1	0/1	0/0
Chromosome1-4949920 INS	PRECISE	125	0	0.5	0/0	0/0	0/0	0/0	0/0	0/0	0/0	0/0	0/1	0/0	0/1	0/1	0/1	0/0
Chromosome1-4952875 INS	PRECISE	328	0	0.577	0/0	0/0	0/0	0/0	0/0	0/0	0/0	0/0	0/1	0/0	0/1	0/1	0/1	0/0
Chromosome1-4954494 INS	PRECISE	322	0	0	0/0	0/0	0/0	0/0	0/0	0/0	0/0	0/0	0/0	0/1	0/0	0/0	0/0	0/0
Chromosome1-4958511 INS	PRECISE	878	0	0	0/0	0/0	0/0	0/1	0/0	0/0	0/0	0/0	0/0	0/0	0/0	0/0	0/0	0/0
Chromosome1-4961090 DEL	PRECISE	865	0	0	0/0	0/0	0/0	0/0	0/0	0/0	0/0	0/0	0/1	0/0	0/1	0/1	0/1	0/0
Chromosome1-4965531 DEL	PRECISE	818	0	0	0/0	0/1	0/1	0/1	0/1	0/1	0/0	0/0	0/0	0/0	0/0	0/0	0/0	0/1
Chromosome1-4971295 INS	PRECISE	542	0	0.816	0/0	0/1	0/1	0/1	0/1	0/1	0/0	0/0	0/0	0/0	0/0	0/0	0/0	0/1
Chromosome1-4971850 DEL	PRECISE	55	0	0	0/0	0/1	0/1	0/1	0/1	0/1	0/0	0/0	0/0	0/0	0/0	0/0	0/0	0/1
Chromosome1-4976623 INS	PRECISE	54	0	0	0/0	0/0	0/1	1/1	1/1	1/1	0/1	0/1	1/1	0/1	0/1	0/1	0/1	0/1
Chromosome1-4987526 DEL	PRECISE	786	0.515	0.9	0/0	0/1	0/1	1/1	1/1	1/1	0/0	0/1	1/1	0/0	0/1	1/1	0/1	1/1
Chromosome1-5010803 DEL	PRECISE	56	0	0	0/0	0/0	0/0	0/0	0/0	0/0	0/1	0/1	0/0	0/0	0/0	0/0	0/0	0/0
Chromosome1-5019526 DEL	PRECISE	307	0	0	0/0	0/0	0/0	0/0	0/0	0/0	0/0	0/0	0/0	0/0	0/0	0/1	0/1	0/0
Chromosome1-5030622 DEL	PRECISE	5819	0	0	0/0	0/0	0/0	0/0	0/0	0/0	0/0	0/0	0/0	0/0	0/0	0/1	0/0	0/0
Chromosome1-5031316 DEL	PRECISE	1341	0	0	0/0	0/1	0/1	0/1	0/1	0/1	0/1	0/1	0/0	0/1	0/0	0/0	0/0	1/1
Chromosome1-5036454 DEL	PRECISE	2359	0.707	0	0/0	0/0	0/0	0/0	0/0	0/0	0/0	0/0	0/0	0/0	0/0	0/1	0/0	0/0
Chromosome1-5041514 INS	PRECISE	160	0	0	0/0	0/0	0/0	0/0	0/0	0/1	0/0	0/0	1/1	0/0	0/0	0/0	0/0	0/0
Chromosome1-5044327 DEL	PRECISE	444	0	0	0/0	0/0	0/0	0/0	0/0	0/0	0/1	0/1	0/0	0/1	0/0	0/0	0/0	0/0
Chromosome1-5045735 DEL	PRECISE	100	0	0	0/0	0/0	0/0	0/0	0/0	0/0	0/0	0/0	0/0	0/0	0/0	0/1	0/0	0/0
Chromosome1-5047349 INS	PRECISE	7145	0	0	0/0	0/0	0/0	0/0	0/0	0/0	0/0	0/0	0/0	1/1	0/0	0/0	0/0	0/0
Chromosome1-5047358 BND	PRECISE	0	0	0	0/0	0/0	0/0	0/0	0/0	0/0	0/0	0/0	0/0	0/1	0/0	0/0	0/0	0/0
Chromosome1-5054120 DEL	PRECISE	88	0	0	0/0	0/0	0/0	0/0	0/0	0/0	0/1	0/1	0/0	0/1	0/0	0/0	0/0	0/0
Chromosome1-5071249 INS	PRECISE	159	0	0	0/0	0/0	0/0	0/0	0/0	0/0	0/0	0/0	0/0	0/0	0/0	0/0	0/0	0/1
Chromosome1-5072365 INS	PRECISE	426	0	0.957	0/0	0/0	0/0	0/0	0/0	0/0	0/0	0/0	0/1	0/0	0/1	0/0	0/1	0/0
Chromosome1-5077185 INS	PRECISE	352	0	0.5	0/0	0/0	0/0	0/0	0/0	0/0	0/0	0/0	0/1	0/0	0/1	0/0	0/1	0/0
Chromosome1-5078413 INS	PRECISE	851	0	0	0/0	0/0	0/0	0/0	0/0	0/0	0/0	0/0	0/0	0/0	0/0	0/0	0/0	0/1
Chromosome1-5079078 INS	PRECISE	149	0	0	0/0	0/0	0/0	0/0	0/0	0/0	0/0	0/0	0/1	0/0	0/1	0/0	0/1	0/0
Chromosome1-5080279 DEL	PRECISE	1182	0	0	0/0	0/1	0/1	1/1	1/1	1/1	0/1	0/1	1/1	0/0	0/1	0/0	0/0	1/1
Chromosome1-5082945 DEL	PRECISE	1192	0	0	0/0	0/0	0/0	0/0	0/0	0/0	0/0	0/0	0/0	0/0	0/0	0/0	0/0	0/1
Chromosome1-5089418 DEL	PRECISE	54	0	0	0/0	0/0	0/0	0/0	0/0	0/0	0/1	0/1	0/0	0/0	0/0	0/0	0/0	0/1
Chromosome1-5089700 DEL	PRECISE	1347	0	0	0/0	0/0	0/0	0/0	0/0	0/0	0/0	0/0	0/1	0/0	0/1	0/0	0/1	0/0
Chromosome1-5092219 DEL	PRECISE	423	0	0	0/0	0/0	0/0	0/0	0/0	0/0	0/0	0/0	0/1	0/0	0/1	0/0	0/1	0/0
Chromosome1-5103043 INS	PRECISE	868	0	0	0/0	0/0	0/0	0/0	0/0	0/0	0/0	0/0	0/0	0/0	0/0	0/0	0/0	0/1
Chromosome1-5107527 INS	PRECISE	66	0	0.5	0/0	0/0	0/0	0/0	0/0	0/0	0/0	0/0	0/1	0/0	0/1	0/1	0/1	0/0
Chromosome1-5117533 INS	PRECISE	55	0	0.5	0/0	0/0	0/0	0/0	0/0	0/0	0/0	0/0	0/0	0/0	0/1	0/1	0/1	0/0
Chromosome1-5117546 INS	PRECISE	1059	0	0	0/0	0/0	0/0	0/0	0/0	0/0	0/0	0/0	0/0	0/0	0/0	0/0	0/0	0/1
Chromosome1-5120043 INS	PRECISE	7478	0	0	0/0	0/0	0/0	0/0	0/0	0/0	0/0	0/0	0/0	0/0	0/0	0/0	0/0	1/1
Chromosome1-5124729 DEL	PRECISE	969	0	0	0/0	0/0	0/0	0/0	0/0	0/0	0/0	0/0	0/1	0/0	0/1	0/1	0/1	0/0
Chromosome1-5132398 INS	PRECISE	12550	0	0	0/0	0/0	0/0	0/0	0/0	0/0	0/0	0/0	0/0	1/1	0/0	0/0	0/0	0/0
Chromosome1-5135407 DEL	PRECISE	1066	0	0	0/1	1/1	1/1	0/0	1/1	1/1	1/1	1/1	1/1	1/1	1/1	1/1	1/1	1/1
Chromosome1-5137192 DEL	PRECISE	64	0	0	0/0	0/0	0/0	0/0	0/0	0/0	0/0	0/0	0/0	0/0	0/0	0/0	0/0	0/1
Chromosome1-5152567 INS	PRECISE	100	0	0	0/0	0/1	0/1	0/1	1/1	0/1	0/0	0/0	0/0	0/1	0/0	0/0	0/0	1/1
Chromosome1-5154283 DEL	PRECISE	3061	0	0	0/0	0/0	0/0	0/0	0/0	0/0	0/0	0/0	0/1	0/0	0/1	0/1	1/1	0/0
Chromosome1-5174811 BND	PRECISE	0	0	0	0/0	0/0	0/0	0/0	0/1	0/1	0/0	0/0	1/1	0/0	0/0	0/0	0/0	0/0
Chromosome1-5177821 INS	PRECISE	9896	0	0	0/0	0/0	0/0	0/0	0/0	0/0	0/0	0/0	0/0	0/0	0/0	0/0	0/0	0/1
Chromosome1-5177821 BND	PRECISE	0	0	0	0/0	0/0	0/0	0/0	0/0	0/0	0/0	0/0	0/0	0/0	0/0	0/0	0/0	0/1
Chromosome1-5179935 INS	PRECISE	703	0	0	0/0	0/0	0/0	0/0	0/0	0/0	0/0	0/0	0/0	0/0	0/0	0/0	0/0	0/1

Chromosome1-5180347	INS	PRECISE	55	0	0	0/0	0/0	0/0	0/0	0/0	0/0	0/1	1/1	0/0	0/0	0/0	0/0	0/0	0/0
Chromosome1-5183314	DEL	PRECISE	60	0	0	0/0	0/0	0/0	0/0	0/0	0/0	0/0	0/0	0/1	0/0	0/1	0/1	0/1	0/0
Chromosome1-5183426	INS	PRECISE	71	0	0.447	0/0	0/0	0/0	0/0	0/0	0/0	0/0	0/0	0/1	0/0	0/1	0/1	0/1	0/1
Chromosome1-5213381	DEL	PRECISE	184	0	0	0/0	0/0	0/0	0/0	0/0	0/0	0/0	0/0	0/1	0/0	0/1	1/1	0/1	0/0
Chromosome1-5217843	BND	PRECISE	0	0	0	0/0	0/0	0/0	0/0	0/0	0/0	0/0	0/0	0/0	0/0	0/0	0/1	0/0	0/0
Chromosome1-5219340	INS	PRECISE	329	0	0.577	0/0	0/0	0/0	0/0	0/0	0/0	0/0	0/0	0/1	0/0	0/1	1/1	0/1	0/0
Chromosome1-5221630	INS	PRECISE	392	0	0.5	0/0	0/0	0/0	0/0	0/0	0/0	0/0	0/0	1/1	0/0	0/1	1/1	0/1	0/0
Chromosome1-5223164	BND	PRECISE	0	0	0	0/0	0/0	0/0	0/1	0/0	0/0	0/1	0/1	1/1	0/0	0/0	0/0	0/0	0/0
Chromosome1-5224708	DUP	PRECISE	24672605	0	0	0/0	0/0	0/0	0/0	0/0	0/0	0/1	0/1	0/0	0/0	0/0	0/0	0/0	0/0
Chromosome1-5224801	INS	PRECISE	4921	0	0	0/0	0/0	0/0	0/0	0/0	0/0	0/0	0/0	0/0	0/1	0/0	0/0	0/0	0/0
Chromosome1-5233580	DEL	PRECISE	60	0	0	0/0	0/0	0/0	0/0	0/0	0/0	0/0	0/0	0/0	0/0	0/0	0/0	0/0	0/1
Chromosome1-5235685	DEL	PRECISE	76	0	0	0/0	0/0	0/0	0/1	0/0	0/0	0/0	0/0	0/0	0/0	0/0	0/0	0/0	0/0
Chromosome1-5236388	DEL	PRECISE	4830	0	0	0/0	0/0	0/0	0/0	0/0	0/0	0/0	0/0	0/0	0/0	0/0	0/0	0/0	0/1
Chromosome1-5243981	DEL	PRECISE	51	0	0	0/0	0/0	0/0	0/1	0/0	0/0	0/0	0/0	0/0	0/1	0/0	0/0	0/0	0/0
Chromosome1-5249490	DUP	PRECISE	4297263	0	0	0/0	0/0	0/0	0/1	0/0	0/0	0/0	0/0	0/0	0/0	0/0	0/0	0/0	0/0
Chromosome1-5261476	DEL	PRECISE	54	0	0	0/0	0/0	0/0	0/1	0/0	0/0	0/0	0/0	0/0	0/0	0/0	0/0	0/0	0/0
Chromosome1-5263507	DEL	PRECISE	275	0	0	0/0	0/1	0/1	0/1	0/1	0/1	0/0	0/0	0/1	0/0	0/1	0/1	0/1	1/1
Chromosome1-5270308	DEL	PRECISE	77	0	0	0/0	0/1	0/1	0/1	0/1	0/1	0/0	0/0	0/0	0/0	0/0	0/0	0/0	0/1
Chromosome1-5290716	INS	PRECISE	284	0	0.5	0/0	0/0	0/0	0/0	0/0	0/0	0/0	0/0	0/0	0/0	0/0	0/0	0/1	0/0
Chromosome1-5291257	INS	PRECISE	7075	0	0	0/0	0/0	0/0	0/0	0/0	0/0	0/0	0/0	0/0	0/0	0/0	0/0	0/0	0/1
Chromosome1-5296636	DEL	PRECISE	136	0	0	0/0	0/0	0/0	0/0	0/0	0/0	0/0	0/0	0/0	0/0	0/0	0/0	0/0	0/1
Chromosome1-5304890	DEL	PRECISE	57	0	0	0/0	0/0	0/0	0/0	0/0	0/0	0/1	0/1	0/0	0/0	0/0	0/0	0/0	0/0
Chromosome1-5309864	INS	PRECISE	137	0	0	0/0	0/0	0/0	0/0	0/1	0/1	0/0	0/0	0/1	0/0	0/0	0/0	0/0	0/0
Chromosome1-5311215	DEL	PRECISE	285	0	0	0/0	0/1	0/1	0/1	0/1	0/1	0/0	0/0	0/0	0/0	0/0	0/0	0/0	0/1
Chromosome1-5313365	INS	PRECISE	212	0	0	0/0	0/0	0/0	0/0	0/0	0/0	0/0	0/0	0/0	0/0	0/0	0/0	0/0	0/1
Chromosome1-5322785	DEL	PRECISE	732	0	0	0/0	0/1	0/1	0/1	0/1	0/1	0/0	0/0	0/0	0/0	0/0	0/0	0/0	0/1
Chromosome1-5324659	DEL	PRECISE	227	0	0	0/0	0/0	0/0	0/0	0/0	0/0	0/0	0/0	0/0	0/0	0/0	0/0	0/0	0/1
Chromosome1-5325961	DEL	PRECISE	354	0	0	0/0	0/1	0/1	1/1	0/1	0/1	0/0	0/0	0/0	0/1	0/0	0/0	0/0	1/1
Chromosome1-5329072	DEL	PRECISE	1682	0	0.577	0/0	0/0	0/0	0/0	0/0	0/0	0/0	0/0	0/0	0/0	0/0	0/1	0/0	0/0
Chromosome1-5338296	DEL	PRECISE	147	0	0	0/0	0/0	0/0	0/0	0/0	0/0	0/0	0/0	0/0	0/0	0/0	0/0	0/0	0/1
Chromosome1-5339377	DEL	PRECISE	10094	0	0.447	0/0	0/0	0/0	0/0	0/0	0/0	0/0	0/0	0/1	0/0	0/1	1/1	0/1	0/1
Chromosome1-5346741	DEL	PRECISE	549	0	0	0/0	0/0	0/0	0/0	0/0	0/0	0/1	0/1	0/0	0/0	0/0	0/0	0/0	0/0
Chromosome1-5358043	DEL	PRECISE	50	0	0	0/0	0/0	0/0	0/0	0/0	0/0	0/0	0/0	0/0	0/0	0/0	0/0	0/0	0/1
Chromosome1-5359356	INS	PRECISE	82	0	0	0/0	0/0	0/0	0/0	0/0	0/0	0/0	0/0	0/0	0/1	0/0	0/0	0/0	0/0
Chromosome1-5361225	DEL	PRECISE	276	0	0	0/0	0/1	0/1	0/1	0/1	0/1	0/0	0/0	0/1	0/0	0/0	0/0	0/1	0/1
Chromosome1-5374731	DEL	PRECISE	92	0	0	0/0	0/0	0/0	0/0	0/0	0/0	0/0	0/0	0/0	0/0	0/0	0/0	0/0	0/1
Chromosome1-5383585	INS	PRECISE	971	0	0	0/0	0/0	0/0	0/0	0/0	0/0	0/0	0/0	0/0	0/0	0/0	0/0	0/0	0/1
Chromosome1-5394314	DEL	PRECISE	5526	0	0	0/0	0/0	0/0	0/0	0/0	0/0	0/0	0/0	0/1	0/0	1/1	1/1	0/1	0/0
Chromosome1-5394885	INS	PRECISE	88	0	0	0/0	0/0	0/0	0/0	0/1	0/1	0/1	0/1	0/1	0/0	0/0	0/0	0/0	0/0
Chromosome1-5413477	DEL	PRECISE	814	0	0	0/0	0/0	0/0	0/0	0/0	0/0	0/0	0/0	0/1	0/0	0/1	1/1	0/1	0/0
Chromosome1-5414964	DEL	PRECISE	50	0	0	0/0	0/0	0/0	0/0	0/0	0/0	0/0	0/0	0/0	0/0	0/0	0/0	0/0	0/1
Chromosome1-5421356	DEL	PRECISE	76	0	0	0/0	0/1	0/1	0/1	0/1	0/1	0/0	0/0	0/0	0/0	0/0	0/0	0/0	0/1
Chromosome1-5423701	DEL	PRECISE	174	0	0	0/0	0/0	0/0	0/0	0/0	0/0	0/0	0/0	0/0	0/0	0/0	0/0	0/0	0/1
Chromosome1-5436086	DEL	PRECISE	975	0	0	0/0	0/0	0/0	0/0	0/0	0/0	0/0	0/0	0/1	0/0	0/1	0/1	0/1	0/0
Chromosome1-5438093	DEL	PRECISE	1684	0	0	0/0	0/0	0/0	0/1	0/0	0/0	0/0	0/0	0/0	0/1	0/0	0/0	0/0	0/0
Chromosome1-5439007	DEL	PRECISE	114	0	0	0/0	0/0	0/0	0/0	0/0	0/0	0/0	0/0	0/1	0/0	0/1	0/1	0/1	0/0
Chromosome1-5444593	INS	PRECISE	373	0	0.823	0/0	0/1	0/1	0/1	0/1	0/1	0/0	0/0	0/1	0/0	0/1	0/1	0/1	0/1
Chromosome1-5446940	DEL	PRECISE	252	0	0	0/0	0/0	0/0	0/0	0/0	0/0	0/0	0/0	0/1	0/0	0/1	0/1	0/1	0/0
Chromosome1-5447741	INS	PRECISE	109	0	0	0/0	0/0	0/0	0/0	0/0	0/0	0/0	0/0	0/1	0/0	0/1	0/1	0/1	0/0
Chromosome1-5447840	DEL	PRECISE	527	0	0	0/0	0/0	0/0	0/0	0/0	0/0	0/0	0/0	0/1	0/0	0/1	0/1	0/1	0/0
Chromosome1-5448876	DEL	PRECISE	5493	0	0	0/0	0/0	0/0	0/0	0/0	0/0	0/0	0/0	0/1	0/0	0/1	0/1	0/1	0/0
Chromosome1-5460750	INS	PRECISE	1307	0	0	0/0	0/0	0/0	0/0	0/0	0/0	0/0	0/0	0/0	0/0	0/1	0/1	0/1	0/0
Chromosome1-5462268	INS	PRECISE	12577	0	0	0/0	0/0	0/0	0/0	0/0	0/0	0/0	0/0	0/0	0/1	0/0	0/0	0/0	0/0
Chromosome1-5468766	DEL	PRECISE	1358	0	0	0/0	0/0	0/0	0/0	0/0	0/0	0/0	0/0	0/0	0/0	0/0	0/0	0/0	0/1
Chromosome1-5487389	INS	PRECISE	1319	0	0.837	0/0	0/0	0/0	0/0	0/1	0/1	0/1	0/1	1/1	0/0	0/0	0/0	0/0	0/0
Chromosome1-5487462	DEL	PRECISE	72	0	0	0/0	0/0	0/0	0/0	0/0	0/0	0/0	0/0	0/0	0/0	0/0	0/0	0/0	0/1
Chromosome1-5488275	INS	PRECISE	153	0	0.577	0/0	0/0	0/0	0/0	0/0	0/0	0/0	0/0	0/0	0/0	0/1	0/1	0/1	0/0
Chromosome1-5488670	DEL	PRECISE	6940	0	0	0/0	0/0	0/0	0/0	0/0	0/0	0/0	0/0	0/0	0/0	1/1	0/1	0/1	0/0
Chromosome1-5501093	INS	PRECISE	53	0	0	0/0	0/0	0/0	0/0	0/0	0/0	0/0	0/0	0/0	0/0	0/0	0/0	0/0	0/1
Chromosome1-5504127	INS	PRECISE	1330	0	0	0/0	0/0	0/0	0/1	0/0	0/0	0/0	0/0	0/0	0/0	0/0	0/0	0/0	0/0
Chromosome1-5508069	DEL	PRECISE	591	0	0	0/0	0/0	0/0	0/0	0/0	0/0	0/0	0/0	0/1	0/0	0/1	0/1	1/1	0/0
Chromosome1-5509119	DEL	PRECISE	70	0	0	0/0	0/0	0/0	0/0	0/0	0/0	0/0	0/0	0/0	0/0	0/0	0/0	0/0	0/1
Chromosome1-5513221	INS	PRECISE	128	0	0.548	0/0	0/1	0/1	0/1	0/1	0/1	0/0	0/0	0/0	0/0	0/0	0/0	0/0	1/1
Chromosome1-5525062	DEL	PRECISE	144	0	0	0/0	0/0	0/0	0/0	0/0	0/0	0/0	0/0	0/1	0/0	0/1	0/1	0/1	0/0
Chromosome1-5531609	INS	PRECISE	138	0	0.5	0/0	0/0	0/0	0/0	0/0	0/0	0/0	0/0	0/1	0/0	0/1	0/1	0/1	0/0
Chromosome1-5532505	INS	PRECISE	314	0	0	0/0	0/0	0/0	0/0	0/0	0/0	0/0	0/0	0/0	0/0	0/0	0/0	0/0	0/1
Chromosome1-5535106	INS	PRECISE	167	0.5	0.5	0/0	0/0	0/0	0/0	0/0	0/0	0/0	0/0	0/1	0/0	0/1	0/1	0/1	0/0
Chromosome1-5544902	INS	PRECISE	92	0	0	0/0	0/0	0/0	0/0	0/0	0/0	0/0	0/0	0/1	0/0	0/1	0/1	0/1	0/0
Chromosome1-5546960	DEL	PRECISE	568	0.5	0.5	0/0	0/0	0/0	0/0	0/0	0/0	0/0	0/0	0/1	0/0	0/1	0/1	0/1	0/0
Chromosome1-5552512	INS	PRECISE	211	0	0	0/0	0/0	0/0	0/0	0/0	0/0	0/0	0/0	0/0	0/0	0/0	0/0	0/0	1/1
Chromosome1-5562026	INS	PRECISE	108	0	0.5	0/0	0/0	0/0	0/0	0/0	0/0	0/0	0/0	0/1	0/0	0/1	0/0	0/0	0/0
Chromosome1-5562462	DEL	PRECISE	195	0	0	0/0	0/0	0/0	0/1	0/0	0/0	0/0	0/0	0/0	0/0	0/0	0/0	0/0	0/0
Chromosome1-5563544	INS	PRECISE	79	0	0	0/0	0/0	0/0	0/0	0/0	0/0	0/0	0/0	0/1	0/0	0/1	0/1	0/0	0/0

Chromosome1-5565990	INS	PRECISE	212	0	0	0/0	0/0	0/0	0/0	0/0	0/0	0/0	0/0	0/0	0/0	0/0	0/0	0/0	0/1
Chromosome1-5568106	INS	PRECISE	170	0	0	0/0	0/0	0/0	0/0	0/0	0/0	0/0	0/0	0/1	0/0	0/1	0/1	0/0	0/0
Chromosome1-5570541	INS	PRECISE	60	0	0.707	0/0	0/0	0/0	0/0	0/1	0/1	0/0	0/0	0/0	0/0	0/0	0/0	0/0	0/0
Chromosome1-5571421	BND	PRECISE	0	0	0	0/0	0/0	0/0	0/0	0/0	0/0	0/0	0/0	0/0	0/0	0/0	0/0	0/0	0/1
Chromosome1-5577388	DEL	PRECISE	18603	0	0	0/0	0/1	0/1	1/1	1/1	1/1	0/0	0/0	1/1	0/0	1/1	0/0	0/1	1/1
Chromosome1-5577388	DEL	PRECISE	884	0	0	0/0	0/0	0/0	0/0	0/0	0/0	0/0	0/0	1/1	0/0	0/0	0/1	0/0	1/1
Chromosome1-5578291	DEL	PRECISE	450	0	0	0/0	0/0	0/0	0/0	0/0	0/0	0/0	0/0	1/1	0/0	0/0	0/1	0/0	1/1
Chromosome1-5578866	DEL	PRECISE	17125	0.577	0.577	0/0	0/0	0/0	0/0	0/0	0/0	0/1	0/1	0/0	0/0	0/0	0/1	0/0	0/0
Chromosome1-5598656	DEL	PRECISE	122	0	0	0/0	0/1	0/1	0/1	0/1	0/1	0/0	0/0	0/0	0/0	0/0	0/0	0/0	0/1
Chromosome1-5607099	INS	PRECISE	161	0	0	0/0	0/0	0/0	0/0	0/0	0/0	0/1	1/1	0/0	0/0	0/0	0/0	0/0	0/0
Chromosome1-5616732	BND	PRECISE	0	0	0	0/0	0/1	0/0	0/0	0/0	0/1	0/0	0/0	0/1	0/0	0/0	0/0	0/0	0/0
Chromosome1-5621654	INS	PRECISE	711	0	0	0/0	0/0	0/0	0/0	0/0	0/0	0/0	0/0	0/0	0/0	0/0	0/0	0/0	0/1
Chromosome1-5632203	DEL	PRECISE	51	0	0	0/0	0/0	0/0	0/0	0/0	0/0	0/0	0/0	0/1	0/0	0/1	0/1	0/1	0/0
Chromosome1-5642796	INS	PRECISE	1446	0	0	0/0	0/0	0/0	0/0	0/0	0/0	0/0	0/0	0/0	0/0	0/0	0/0	0/0	0/1
Chromosome1-5652821	INS	PRECISE	4982	0	0	0/0	0/0	0/0	1/1	0/0	0/0	0/0	0/0	0/0	0/0	0/0	0/0	0/0	0/0
Chromosome1-5656191	INS	PRECISE	216	0.5	0	0/0	0/0	0/0	0/0	0/0	0/0	0/0	0/0	0/1	0/0	0/1	0/1	0/1	0/0
Chromosome1-5660284	INS	PRECISE	162	0	0	0/0	0/0	0/0	0/0	0/1	0/1	0/0	0/0	0/1	0/0	0/0	0/0	0/0	0/0
Chromosome1-5662006	DEL	PRECISE	51	0	0	0/0	0/1	0/1	1/1	1/1	1/1	0/0	0/0	0/1	0/0	0/0	0/0	0/0	1/1
Chromosome1-5667873	DEL	PRECISE	325	0	0	0/0	0/1	0/1	1/1	0/1	0/1	0/0	0/0	0/1	0/0	0/1	0/1	0/1	1/1
Chromosome1-5670684	DEL	PRECISE	57	0	0	0/0	0/0	0/0	0/0	0/0	0/0	0/0	0/0	0/1	0/0	0/1	0/1	0/1	0/0
Chromosome1-5672226	INS	PRECISE	168	0	0.577	0/0	0/0	0/0	0/0	0/0	0/0	0/0	0/0	0/1	0/0	0/1	0/1	0/1	0/0
Chromosome1-5673721	DEL	PRECISE	73	0	0	0/0	0/1	0/1	1/1	1/1	1/1	0/0	0/0	0/1	0/0	0/0	0/0	0/0	1/1
Chromosome1-5675405	INS	PRECISE	66	0	0	0/0	0/1	0/1	1/1	0/1	0/1	0/1	0/1	0/0	0/0	0/0	0/0	0/0	1/1
Chromosome1-5678820	DEL	PRECISE	70	0	0	0/0	0/0	0/0	0/0	0/0	0/0	0/0	0/0	0/1	0/0	0/1	0/1	0/1	0/0
Chromosome1-5680112	DEL	PRECISE	616	0	0	0/0	0/1	0/1	1/1	1/1	1/1	0/1	0/1	1/1	0/0	0/1	0/1	0/1	1/1
Chromosome1-5685641	INS	PRECISE	148	0	0.5	0/0	0/0	0/0	0/0	0/0	0/0	0/0	0/0	0/1	0/0	0/1	0/1	0/1	0/0
Chromosome1-5688815	DEL	PRECISE	846	0	0	0/0	0/0	0/0	0/0	0/0	0/0	0/0	0/0	0/1	0/0	0/1	0/1	0/0	0/0
Chromosome1-5689833	INS	PRECISE	51	0	0	0/0	0/0	0/0	0/0	0/0	0/0	0/0	0/0	0/1	0/0	0/1	0/1	0/1	0/1
Chromosome1-5699356	DEL	PRECISE	50	0.5	0.5	0/0	0/0	0/0	0/0	0/0	0/0	0/0	0/0	0/1	0/0	0/1	0/1	0/1	0/0
Chromosome1-5703990	DEL	PRECISE	55	0	0	0/0	0/0	0/0	0/0	0/0	0/0	0/0	0/0	0/1	0/0	0/1	0/1	0/1	0/0
Chromosome1-5713481	DEL	PRECISE	532	0	0	0/0	0/0	0/0	0/0	0/0	0/0	0/0	0/0	0/1	0/0	0/1	0/1	0/0	0/0
Chromosome1-5715613	INS	PRECISE	3572	0	0	0/0	0/0	0/0	0/0	0/0	0/0	0/0	0/0	0/0	0/0	0/0	0/0	0/0	0/1
Chromosome1-5718270	DEL	PRECISE	66	0	0	0/0	0/0	0/0	0/0	0/0	0/0	0/0	0/0	0/0	0/1	0/0	0/0	0/0	0/0
Chromosome1-5722091	INS	PRECISE	1094	0	0.577	0/0	0/0	0/0	0/0	0/0	0/0	0/1	0/1	0/0	0/1	0/0	0/0	0/0	0/0
Chromosome1-5723094	DEL	PRECISE	59	0	0	0/0	0/0	0/0	0/0	0/0	0/0	0/0	0/0	0/0	0/0	0/0	0/0	0/0	0/1
Chromosome1-5723753	INS	PRECISE	330	0	0.516	0/0	0/1	0/1	1/1	0/1	0/1	0/0	0/0	0/0	0/0	0/0	0/0	0/0	0/1
Chromosome1-5725672	DEL	PRECISE	284	0	0	0/0	0/0	0/0	0/0	0/0	0/0	0/1	0/1	0/0	0/0	0/0	0/0	0/0	0/0
Chromosome1-5725919	INS	PRECISE	1429	0	0	0/0	0/0	0/0	0/0	0/0	0/0	0/0	0/0	0/0	0/0	0/0	0/0	0/0	0/1
Chromosome1-5731288	INS	PRECISE	146	0	0	0/0	0/0	0/0	0/0	0/0	0/0	0/0	0/0	0/0	0/0	0/0	0/0	0/0	0/1
Chromosome1-5737631	INS	PRECISE	57	0	0	0/0	0/0	0/0	0/0	0/1	0/1	0/0	0/0	1/1	0/0	0/0	0/0	0/0	0/0
Chromosome1-5738768	INS	PRECISE	401	0	0	0/0	0/1	0/1	1/1	0/1	0/1	0/0	0/0	0/0	0/0	0/0	0/0	0/0	1/1
Chromosome1-5745570	DEL	PRECISE	492	0	0	0/0	0/0	0/0	0/0	0/0	0/0	0/0	0/0	0/0	0/0	0/0	0/0	0/0	0/1
Chromosome1-5749059	INS	PRECISE	225	0	0.5	0/0	0/0	0/0	0/0	0/0	0/0	0/0	0/0	0/1	0/0	0/1	0/1	0/1	0/0
Chromosome1-5750863	DEL	PRECISE	774	0	0	0/0	0/0	0/0	0/0	0/0	0/0	0/0	0/0	0/1	0/0	0/1	0/1	0/1	0/1
Chromosome1-5753620	INS	PRECISE	4968	0	0	0/0	0/0	0/0	0/0	0/0	0/0	0/0	0/0	0/0	0/0	0/0	0/0	0/0	1/1
Chromosome1-5755774	DEL	PRECISE	709	0	0	0/0	0/0	0/0	0/0	0/0	0/0	0/0	0/0	0/1	0/0	0/1	0/1	0/1	0/0
Chromosome1-5757723	DEL	PRECISE	150	0	0	0/0	0/1	0/1	1/1	0/1	0/1	0/0	0/0	0/0	0/0	0/0	0/0	0/0	0/1
Chromosome1-5762286	DEL	PRECISE	55	0.816	0.408	0/0	0/1	0/1	1/1	0/1	0/1	0/0	0/0	0/0	0/0	0/0	0/0	0/0	1/1
Chromosome1-5762482	INS	PRECISE	6120	0	0	0/0	0/0	0/0	0/0	0/0	0/0	0/0	0/0	0/0	1/1	0/0	0/0	0/0	0/0
Chromosome1-5762487	BND	PRECISE	0	0	0	0/0	0/0	0/0	0/0	0/0	0/0	0/0	0/0	0/0	0/1	0/0	0/0	0/0	0/0
Chromosome1-5763562	DEL	PRECISE	7707	0.302	0.302	0/0	0/1	0/1	1/1	1/1	1/1	0/0	0/0	1/1	0/1	0/1	0/1	0/1	1/1
Chromosome1-5771976	DEL	PRECISE	77	0	0	0/0	0/0	0/0	0/0	0/0	0/0	0/0	0/0	0/0	0/0	0/0	0/0	0/0	0/1
Chromosome1-5778954	INV	PRECISE	13950828	0	0	0/0	0/0	0/0	0/1	0/0	0/0	0/1	0/0	0/0	0/0	0/0	0/0	0/0	0/0
Chromosome1-5789973	BND	PRECISE	0	0	0	0/0	0/0	0/0	0/0	0/1	0/0	0/0	0/1	0/1	0/0	0/0	0/0	0/0	0/0
Chromosome1-5805505	INS	PRECISE	152	0	0	0/0	0/0	0/0	0/0	0/0	0/0	0/0	0/0	0/0	0/0	0/0	0/0	0/0	0/1
Chromosome1-5823065	DEL	PRECISE	96	0	0	0/0	0/0	0/0	0/0	0/0	0/0	0/0	0/0	0/1	0/0	0/1	0/1	0/1	0/0
Chromosome1-5823634	INS	PRECISE	722	0	0.816	0/0	0/1	0/1	1/1	0/1	0/1	0/0	0/0	0/0	0/0	0/0	0/0	0/0	0/1
Chromosome1-5824924	INS	PRECISE	650	0	0.753	0/0	0/1	0/1	1/1	0/1	0/1	0/0	0/0	0/0	0/0	0/0	0/0	0/0	1/1
Chromosome1-5828019	DEL	PRECISE	269	0	0	0/0	0/0	0/0	0/0	0/1	0/1	0/0	0/0	0/1	0/0	0/0	0/0	0/0	0/0
Chromosome1-5833875	INS	PRECISE	75	0	0.316	0/0	0/1	0/1	1/1	0/1	0/1	0/0	0/0	0/0	0/0	0/1	0/1	0/1	1/1
Chromosome1-5839036	INS	PRECISE	105	0	0	0/0	0/0	0/0	0/0	0/1	0/1	1/1	0/1	1/1	0/0	0/0	0/0	0/0	0/0
Chromosome1-5839992	INS	PRECISE	751	0	0.548	0/0	0/0	0/0	0/0	0/1	0/1	1/1	0/1	1/1	0/0	0/0	0/0	0/0	0/0
Chromosome1-5849684	DEL	PRECISE	51	0	0	0/0	0/0	0/0	0/0	0/0	0/0	0/0	0/0	0/0	0/0	0/1	0/1	0/1	0/0
Chromosome1-5851464	INS	PRECISE	132	0	0.408	0/0	0/1	0/1	1/1	0/1	0/0	0/0	0/0	0/0	0/0	0/0	0/0	0/0	0/1
Chromosome1-5851835	INV	PRECISE	14805070	0	0	0/0	0/0	0/0	0/0	0/0	0/0	0/1	0/0	0/0	0/0	0/0	0/0	0/0	0/0
Chromosome1-5858437	BND	PRECISE	0	0	0	0/0	0/1	0/0	0/0	0/1	0/1	0/0	0/0	0/0	0/0	0/0	0/0	0/0	0/1
Chromosome1-5860348	DEL	PRECISE	150	0	0	0/0	0/0	0/0	0/0	0/0	0/0	0/0	0/0	0/0	0/0	0/1	0/1	0/1	0/0
Chromosome1-5864347	INS	PRECISE	878	0	0.5	0/0	0/0	0/0	0/0	0/0	0/0	0/0	0/0	0/0	0/0	0/1	0/1	0/1	0/0
Chromosome1-5872235	DEL	PRECISE	130	0	0	0/0	0/0	0/0	1/1	1/1	1/1	0/1	0/1	1/1	0/1	0/1	0/1	0/1	0/0
Chromosome1-5881323	INS	PRECISE	69	0.555	0.277	0/0	0/1	0/0	1/1	1/1	1/1	0/1	0/1	1/1	0/1	0/1	0/1	0/1	1/1
Chromosome1-5883098	INS	PRECISE	183	0.5	0.5	0/0	0/0	0/0	0/0	0/0	0/0	0/0	0/0	0/1	0/0	0/1	0/1	0/1	0/0
Chromosome1-5884052	DEL	PRECISE	631	0	0	0/0	0/0	0/0	0/0	0/0	0/0	0/0	0/0	1/1	0/0	0/1	0/1	0/1	0/0
Chromosome1-5892993	INS	PRECISE	58	0	0	0/0	0/0	0/0	0/0	0/0	0/0	0/0	0/0	0/1	0/0	0/1	0/1	1/1	0/0

Chromosome1-5893465	INS	PRECISE	4474	0	0	0/0	0/0	0/0	0/0	0/0	0/0	0/0	0/0	0/0	0/0	0/0	0/0	0/0	1/1
Chromosome1-5893965	INS	PRECISE	161	0	0	0/0	0/0	0/0	0/0	0/0	0/0	0/0	0/0	0/1	0/0	0/1	0/1	1/1	0/0
Chromosome1-5900897	INS	PRECISE	317	0	0.816	0/0	0/0	0/0	0/1	0/1	1/1	0/0	0/0	0/1	0/0	0/0	0/0	0/0	0/0
Chromosome1-5903241	INS	PRECISE	261	0	0	0/0	0/0	0/0	0/0	0/0	0/0	0/0	0/0	0/0	0/0	0/0	0/0	0/0	0/1
Chromosome1-5904943	DEL	PRECISE	175	0	0	0/0	0/0	0/0	0/0	0/0	0/0	0/0	0/0	0/0	0/0	0/1	0/0	0/0	0/0
Chromosome1-5910436	DEL	PRECISE	78	0	0	0/0	0/0	0/0	0/0	0/0	0/0	0/0	0/0	0/0	0/0	0/0	0/0	0/0	0/1
Chromosome1-5913166	DEL	PRECISE	1003	0	0	0/0	0/0	0/0	0/0	0/0	0/0	0/0	0/0	0/1	0/0	0/1	0/1	0/1	0/0
Chromosome1-5918646	DEL	PRECISE	1263	0	0	0/0	0/0	0/0	0/0	0/0	0/0	0/0	0/0	0/0	0/0	0/0	0/0	0/0	0/1
Chromosome1-5919529	INS	PRECISE	456	0	0.5	0/0	0/0	0/0	0/0	0/0	0/0	0/0	0/0	0/1	0/0	0/1	0/0	0/1	0/0
Chromosome1-5942832	INS	PRECISE	1073	0	0.753	0/0	0/0	0/0	0/1	0/1	0/1	0/1	0/1	0/1	0/0	0/0	0/0	0/0	0/0
Chromosome1-5943739	DEL	PRECISE	53	0	0	0/0	0/0	0/0	0/0	0/0	0/0	0/0	0/0	0/1	0/0	0/1	0/0	0/1	0/0
Chromosome1-5944494	INS	PRECISE	1256	0	0.5	0/0	0/0	0/0	0/1	0/1	0/1	0/0	0/0	0/1	0/0	0/0	0/0	0/0	0/0
Chromosome1-5944494	BND	PRECISE	0	0	0	0/0	0/0	0/0	0/0	0/0	0/0	0/0	0/1	0/0	0/0	0/0	0/0	0/0	0/0
Chromosome1-5950105	DEL	PRECISE	176	0	0	0/0	0/0	0/0	0/0	0/0	0/0	0/0	0/0	0/1	0/0	0/1	0/0	0/1	0/0
Chromosome1-5955543	INS	PRECISE	53	0	0.5	0/0	0/0	0/0	0/0	0/0	0/0	0/0	0/0	0/1	0/0	0/1	0/1	0/1	0/0
Chromosome1-5963604	DEL	PRECISE	118	0.5	0.5	0/0	0/0	0/0	0/0	0/0	0/0	0/0	0/0	0/1	0/0	0/1	0/1	0/1	0/0
Chromosome1-5972150	INS	PRECISE	994	0	0.957	0/0	0/0	0/0	0/0	0/0	0/0	0/0	0/0	0/1	0/0	0/1	0/0	0/1	0/0
Chromosome1-5985429	DEL	PRECISE	483	0	0	0/0	0/0	0/0	0/0	0/0	0/0	0/1	0/1	0/0	0/0	0/0	0/0	0/0	0/0
Chromosome1-5985451	DEL	PRECISE	7132	0	0	0/0	0/0	0/0	0/0	0/0	0/0	0/0	0/0	1/1	0/0	1/1	1/1	1/1	0/0
Chromosome1-5990584	DEL	PRECISE	54	0	0	0/0	0/0	0/0	0/0	0/0	0/0	0/0	0/0	0/0	0/0	0/0	0/0	0/0	0/1
Chromosome1-6005414	DEL	PRECISE	60	0	0	0/0	0/0	0/1	0/0	0/0	0/0	0/1	0/1	0/0	0/0	0/0	0/0	0/0	0/0
Chromosome1-6007270	DEL	PRECISE	1297	0	0	0/0	0/0	0/0	0/0	0/0	0/0	0/0	0/1	0/0	0/0	0/0	0/0	0/0	0/0
Chromosome1-6009415	DEL	PRECISE	120	0	0	0/0	0/0	0/0	0/1	0/0	0/0	0/0	0/0	0/0	0/0	0/0	0/0	0/0	0/0
Chromosome1-6029845	INS	PRECISE	1191	0	0.69	0/0	0/1	0/1	0/1	0/1	0/1	0/0	0/0	0/0	0/1	0/0	0/0	0/0	0/1
Chromosome1-6030241	DEL	PRECISE	338	0	0	0/0	0/0	0/0	0/0	0/0	0/0	0/0	0/0	0/1	0/0	0/1	0/1	0/1	0/0
Chromosome1-6036207	INS	PRECISE	14296	0	0	0/0	0/0	0/0	0/0	0/0	0/0	0/0	0/0	0/0	0/0	0/1	0/0	0/0	0/0
Chromosome1-6047219	DUP	PRECISE	21413297	0	0	0/0	0/0	0/0	0/0	0/0	0/0	0/1	0/0	0/0	0/0	0/0	0/0	0/0	0/0
Chromosome1-6050861	INS	PRECISE	75	0	0	0/0	0/0	0/0	0/1	0/0	0/0	0/0	0/1	0/0	0/0	0/0	0/0	0/0	0/0
Chromosome1-6059158	BND	PRECISE	0	0	0	0/0	0/0	0/0	0/1	0/1	0/1	0/0	0/0	0/1	0/1	0/0	0/0	0/0	0/0
Chromosome1-6060100	INS	PRECISE	140	0	0.354	0/0	0/0	0/0	0/0	0/1	0/1	0/0	0/0	1/1	0/0	0/1	0/1	0/1	0/1
Chromosome1-6061860	INS	PRECISE	131	0	0.5	0/0	0/0	0/0	0/0	0/0	0/0	0/0	0/0	0/1	0/0	0/1	0/1	0/1	0/0
Chromosome1-6068522	DEL	PRECISE	425	0	0	0/0	0/0	0/0	0/0	0/0	0/0	0/1	0/1	0/0	0/0	0/0	0/0	0/0	0/0
Chromosome1-6069232	DEL	PRECISE	119	0	0	0/0	0/0	0/0	0/0	0/0	0/0	0/0	0/0	0/1	0/0	0/1	0/1	0/1	0/0
Chromosome1-6072737	INS	PRECISE	77	0	0	0/0	0/0	0/0	0/0	0/0	0/0	0/0	0/0	0/1	0/0	0/1	0/1	0/1	0/0
Chromosome1-6075750	DEL	PRECISE	391	0	0	0/0	0/0	0/0	0/0	0/0	0/0	0/0	0/0	0/1	0/0	0/1	0/1	0/1	0/0
Chromosome1-6092060	DEL	PRECISE	915	0	0	0/0	0/0	0/0	0/0	0/0	0/0	0/0	0/0	0/1	0/0	0/1	0/1	0/1	0/1
Chromosome1-6098926	DEL	PRECISE	621	0	0	0/0	0/0	0/0	0/0	0/0	0/0	0/0	0/0	0/1	0/0	0/1	0/1	0/1	0/0
Chromosome1-6099172	DEL	PRECISE	7830	0	0	0/0	0/0	0/0	0/0	0/0	0/0	0/0	0/0	0/0	0/0	0/0	0/0	0/0	0/1
Chromosome1-6111034	INS	PRECISE	137	0	0	0/0	0/0	0/0	0/0	0/0	0/0	0/0	0/0	0/0	0/0	0/0	0/0	0/0	0/1
Chromosome1-6114489	INS	PRECISE	5420	0	0	0/0	0/0	0/0	0/0	0/0	0/0	0/0	0/0	0/0	0/0	0/0	0/0	0/0	0/1
Chromosome1-6114498	BND	PRECISE	0	0	0	0/0	0/0	0/0	0/0	0/0	0/0	0/0	0/0	0/0	0/0	0/0	0/0	0/0	0/1
Chromosome1-6114815	BND	PRECISE	0	0	0	0/0	0/0	0/0	0/0	0/0	0/0	0/0	0/0	0/1	0/0	0/1	0/0	0/0	0/0
Chromosome1-6117707	DEL	PRECISE	12473	0	0	0/0	0/1	0/1	1/1	0/1	0/1	0/0	0/0	0/0	0/1	0/0	0/0	0/0	1/1
Chromosome1-6117757	INS	PRECISE	76	0	0.5	0/0	0/0	0/0	0/1	0/1	0/0	0/0	0/0	0/1	0/0	0/0	0/0	0/0	0/0
Chromosome1-6121226	INS	PRECISE	110	0	0	0/0	0/0	0/0	0/0	0/0	0/0	0/1	0/1	0/0	0/0	0/0	0/0	0/0	0/0
Chromosome1-6135943	DEL	PRECISE	66	0	0	0/0	0/0	0/0	0/0	0/0	0/0	0/0	0/0	0/1	0/0	0/1	0/1	0/1	0/0
Chromosome1-6138441	DEL	PRECISE	339	0	0	0/0	0/0	0/0	0/0	0/0	0/0	0/0	0/0	0/1	0/0	0/1	0/1	0/1	0/0
Chromosome1-6140107	INS	PRECISE	211	0	0	0/0	0/0	0/0	0/0	0/0	0/0	0/0	0/0	0/1	0/0	0/1	0/0	0/1	0/0
Chromosome1-6140624	DEL	PRECISE	360	0	0	0/0	0/0	0/0	0/0	0/0	0/0	0/0	0/0	0/1	0/0	0/1	0/0	0/1	0/0
Chromosome1-6141279	DEL	PRECISE	983	0	0	0/0	0/0	0/0	0/0	0/0	0/0	0/0	0/0	0/1	0/0	0/1	0/0	0/1	0/0
Chromosome1-6142114	BND	PRECISE	0	0	0	0/0	0/0	0/0	0/0	0/1	0/0	0/0	0/0	0/1	0/0	0/0	0/0	0/0	0/0
Chromosome1-6142637	DEL	PRECISE	708	0	0	0/0	0/0	0/0	0/0	0/0	0/0	0/0	0/0	0/1	0/0	0/1	0/0	0/1	0/0
Chromosome1-6144886	DEL	PRECISE	1427	0	0	0/0	0/1	0/1	0/1	1/1	1/1	0/1	0/1	1/1	0/1	0/0	0/0	0/0	1/1
Chromosome1-6148802	INS	PRECISE	430	0	0	0/0	0/0	0/0	0/0	0/0	0/0	0/0	0/0	0/0	0/0	0/0	0/0	0/0	0/1
Chromosome1-6151898	INS	PRECISE	5082	0	0	0/0	0/0	0/0	0/0	0/0	0/0	0/0	0/0	0/0	0/0	0/0	0/0	0/0	0/1
Chromosome1-6152152	DEL	PRECISE	62	0	0	0/0	0/0	0/0	0/0	0/0	0/0	0/0	0/0	0/1	0/0	0/1	0/1	0/1	0/0
Chromosome1-6161249	INS	PRECISE	124	0	0	0/0	0/0	0/0	0/0	0/0	0/0	0/0	0/0	0/0	0/0	0/0	0/0	0/0	0/1
Chromosome1-6162215	INV	PRECISE	238	0	0	0/0	0/0	0/0	0/0	0/1	0/1	0/0	0/0	0/1	0/0	0/0	0/0	0/0	0/0
Chromosome1-6165369	INS	PRECISE	363	0	0.447	0/0	0/0	0/0	0/0	0/0	1/1	0/1	0/1	0/1	0/0	0/0	0/0	0/0	0/0
Chromosome1-6167211	INS	PRECISE	161	0	0	0/0	0/0	0/0	0/1	0/0	0/0	0/0	0/0	0/0	0/0	0/0	0/0	0/0	0/0
Chromosome1-6176771	INS	PRECISE	624	0	0	0/0	0/0	0/0	0/0	0/0	0/0	0/0	0/0	0/0	0/0	0/0	0/0	0/0	0/1
Chromosome1-6182267	DEL	PRECISE	276	0	0	0/0	0/1	0/1	1/1	0/1	0/1	0/0	0/0	0/1	0/1	0/1	0/1	0/1	1/1
Chromosome1-6186792	INS	PRECISE	89	0	0	0/0	0/0	0/0	0/0	0/0	0/0	0/0	0/0	0/0	0/0	0/0	0/0	0/1	0/0
Chromosome1-6192934	INS	PRECISE	87	0	0.5	0/0	0/0	0/0	0/0	0/0	0/0	0/0	0/0	0/1	0/0	0/1	0/1	0/1	0/0
Chromosome1-6200623	INS	PRECISE	150	0	0.577	0/0	0/0	0/0	0/0	0/0	0/0	0/0	0/0	0/1	0/0	0/1	0/1	0/0	0/0
Chromosome1-6202624	INS	PRECISE	378	0	0	0/0	0/0	0/0	0/1	0/0	0/0	0/0	0/0	0/0	0/0	0/0	0/0	0/0	0/0
Chromosome1-6208729	INS	PRECISE	154	0	0	0/0	0/0	0/0	0/0	0/0	0/0	0/0	0/0	0/0	0/0	0/0	0/0	0/1	0/0
Chromosome1-6209714	DEL	PRECISE	123	0	0	0/0	0/1	0/1	0/1	0/1	0/1	0/0	0/0	0/0	0/1	0/0	0/0	0/0	0/1
Chromosome1-6209741	BND	PRECISE	0	0	0	0/0	0/0	0/0	0/0	0/0	0/0	0/1	0/1	0/0	0/0	0/0	0/0	0/0	0/0
Chromosome1-6211968	INS	PRECISE	59	0	0	0/0	0/1	0/1	0/1	0/1	0/1	0/1	0/1	0/0	0/1	0/0	0/0	0/0	1/1
Chromosome1-6212028	DEL	PRECISE	95	0	0	0/0	0/0	0/0	0/0	0/0	0/0	0/1	0/1	0/0	0/0	0/0	0/0	0/0	0/0
Chromosome1-6215937	DEL	PRECISE	2390	0	0	0/0	0/0	0/0	0/0	0/0	0/0	0/1	0/1	0/0	0/0	0/0	0/0	0/1	0/0
Chromosome1-6230396	INS	PRECISE	184	0	0.5	0/0	0/0	0/0	0/0	0/0	0/0	0/0	0/0	0/0	0/0	0/1	0/1	0/1	0/0

Chromosome1-6232522	INS	PRECISE	6033	0	0	0/0	0/0	0/0	0/0	0/0	0/0	0/0	0/0	0/0	0/0	0/0	0/0	0/0	0/1
Chromosome1-6239742	DEL	PRECISE	405	0	0	0/0	0/0	0/0	0/0	0/1	0/1	0/0	0/0	0/1	0/0	0/0	0/0	0/0	0/0
Chromosome1-6242636	INS	PRECISE	53	0	0	0/0	0/1	0/1	0/1	1/1	1/1	0/0	0/0	0/1	0/1	0/0	0/0	0/0	0/1
Chromosome1-6246986	DEL	PRECISE	351	0	0	0/0	0/0	0/0	0/0	0/0	0/0	0/0	0/0	0/1	0/0	0/1	0/1	0/1	0/0
Chromosome1-6248364	DEL	PRECISE	977	0	0	0/0	0/0	0/0	0/0	0/0	0/0	0/0	0/0	0/1	0/0	0/0	0/1	0/1	0/0
Chromosome1-6267862	DEL	PRECISE	379	0	0	0/0	0/0	0/0	0/0	0/0	0/0	0/1	0/1	0/0	0/0	0/0	0/0	0/0	0/0
Chromosome1-6268141	INS	PRECISE	3400	0	0	0/0	0/0	0/0	1/1	0/0	0/0	0/0	0/0	0/0	0/0	0/0	0/0	0/0	0/0
Chromosome1-6298909	INS	PRECISE	974	0	0	0/0	0/0	0/0	0/0	0/0	0/0	0/0	0/0	0/0	0/0	0/0	0/0	0/0	0/1
Chromosome1-6301444	DEL	PRECISE	1318	0	0	0/0	0/0	0/0	0/0	0/0	0/0	0/0	0/0	0/0	0/0	0/0	0/0	0/0	0/1
Chromosome1-6306494	DEL	PRECISE	5159	0	0	0/0	0/0	0/0	0/0	0/0	0/0	0/0	0/0	0/0	0/0	0/0	0/0	0/0	0/1
Chromosome1-6312267	DEL	PRECISE	5202	0	0	0/0	0/0	0/0	0/0	0/0	0/0	0/0	0/0	0/0	0/0	0/0	0/0	1/1	0/1
Chromosome1-6326219	INS	PRECISE	7111	0	0	0/0	0/0	0/0	0/0	0/0	0/0	0/0	0/0	0/0	0/0	0/0	0/0	0/0	1/1
Chromosome1-6328840	INS	PRECISE	8363	0	0	0/0	0/0	0/0	0/0	0/0	0/0	0/0	0/0	0/0	0/0	0/0	0/0	0/1	0/0
Chromosome1-6331965	DEL	PRECISE	5520	0	0	0/0	0/0	0/0	0/0	0/0	0/0	0/0	0/0	0/0	0/0	0/0	0/0	0/1	0/0
Chromosome1-6338408	INS	PRECISE	2015	0	0	0/0	0/0	0/0	0/0	0/0	0/0	0/0	0/0	0/0	0/0	0/0	0/0	0/1	0/0
Chromosome1-6344423	DEL	PRECISE	877	0	0	0/0	0/0	0/0	0/0	0/0	0/0	0/1	0/1	0/1	0/0	0/1	0/1	0/1	0/1
Chromosome1-6354855	DEL	PRECISE	1409	0	0	0/0	0/0	0/0	0/0	0/0	0/0	0/0	0/0	0/0	0/0	0/0	0/1	0/0	0/0
Chromosome1-6357167	BND	PRECISE	0	0	0	0/0	0/0	0/0	0/0	0/0	0/0	0/0	0/0	0/1	0/0	0/1	1/1	0/0	0/0
Chromosome1-6357445	INS	PRECISE	669	0	0	0/0	0/0	0/0	0/0	0/0	0/0	0/0	0/0	0/0	0/0	0/0	0/0	0/1	0/0
Chromosome1-6358220	INS	PRECISE	1012	0	0	0/0	0/0	0/0	0/0	0/0	0/0	0/0	0/0	0/0	0/0	0/0	0/0	0/1	0/0
Chromosome1-6369187	BND	PRECISE	0	0	0	0/0	0/0	0/0	0/0	0/0	0/0	0/0	0/0	0/0	0/0	0/0	0/1	0/0	0/0
Chromosome1-6373510	INS	PRECISE	201	0	0	0/0	0/0	0/0	0/0	0/0	0/0	0/0	0/0	0/1	0/0	0/1	1/1	0/0	0/0
Chromosome1-6377023	INS	PRECISE	12444	0	0	0/0	0/0	0/0	1/1	0/0	0/0	0/0	0/0	0/0	0/0	0/0	0/0	0/0	0/0
Chromosome1-6401631	DEL	PRECISE	70	0	0	0/0	0/1	0/1	0/1	0/1	1/1	0/0	0/0	0/0	0/1	0/0	0/0	0/0	0/1
Chromosome1-6407648	DEL	PRECISE	168	0	0	0/0	0/0	0/0	0/0	0/0	0/0	0/0	0/0	0/1	0/0	0/0	0/0	0/0	0/0
Chromosome1-6411503	INS	PRECISE	74	0	0	0/0	0/0	0/0	0/0	0/0	0/0	0/0	0/0	0/1	0/0	0/0	0/0	0/0	0/0
Chromosome1-6413072	DEL	PRECISE	73640	0	0	0/0	0/0	0/0	0/0	0/0	0/0	0/0	0/0	0/0	0/0	0/0	0/0	1/1	0/0
Chromosome1-6414688	INS	PRECISE	402	0	0	0/0	0/0	0/0	0/1	0/0	0/0	0/0	0/0	0/0	0/0	0/0	0/0	0/0	0/0
Chromosome1-6426713	DEL	PRECISE	1372	0	0	0/0	0/0	0/0	0/0	0/0	0/0	0/1	0/1	0/0	0/0	0/0	0/0	0/0	0/0
Chromosome1-6434286	INS	PRECISE	1966	0	0	0/0	0/0	0/0	0/1	0/0	0/0	0/0	0/0	0/0	0/0	0/0	0/0	0/0	0/0
Chromosome1-6444675	DEL	PRECISE	28161	0	0	0/0	1/1	0/1	1/1	0/1	0/1	0/0	0/0	0/0	0/1	0/0	0/0	0/0	1/1
Chromosome1-6484272	DEL	PRECISE	755	0	0	0/0	0/1	0/1	0/1	0/1	0/1	0/0	0/0	0/0	0/1	0/0	0/0	0/0	0/1
Chromosome1-6487245	DEL	PRECISE	405	0	0	0/0	0/0	0/0	0/0	0/0	0/0	0/0	0/1	0/1	0/0	0/1	0/1	0/1	0/0
Chromosome1-6492810	DEL	PRECISE	1189	0	0	0/0	0/0	0/0	0/0	0/0	0/0	0/1	0/0	0/0	0/0	0/0	0/0	0/0	0/0
Chromosome1-6500957	DEL	PRECISE	53	0	0	0/0	0/0	0/0	0/0	0/0	0/0	0/1	0/1	0/1	0/0	0/1	0/1	0/1	0/0
Chromosome1-6505672	DEL	PRECISE	3074	0	0	0/0	0/0	0/0	0/0	0/0	0/0	0/0	0/0	0/1	0/0	0/1	0/1	0/1	0/0
Chromosome1-6509142	DEL	PRECISE	2247	0	0	0/0	0/0	0/0	0/0	0/0	0/0	0/0	0/0	0/1	0/0	0/1	0/1	0/1	0/0
Chromosome1-6510507	DEL	PRECISE	158	0	0	0/0	0/0	0/0	0/0	0/0	0/0	0/1	0/1	0/0	0/0	0/0	0/0	0/0	0/0
Chromosome1-6512242	DEL	PRECISE	520	0	0	0/0	0/0	0/0	0/0	0/0	0/0	0/1	0/1	0/0	0/0	0/0	0/0	0/0	0/0
Chromosome1-6515521	DEL	PRECISE	723	0	0	0/0	0/0	0/0	0/0	0/0	0/0	0/0	0/0	0/1	0/0	0/1	0/1	0/1	0/0
Chromosome1-6516487	DEL	PRECISE	297474	0	0	0/0	0/0	0/0	0/0	0/0	0/0	0/1	0/1	0/0	0/0	0/0	0/0	0/0	0/0
Chromosome1-6516720	DEL	PRECISE	1297	0	0	0/0	0/0	0/0	0/0	0/0	0/0	0/0	0/0	0/0	0/0	0/1	0/0	0/1	0/0
Chromosome1-6520114	DEL	PRECISE	1560	0	0	0/0	0/0	0/0	0/0	0/0	0/0	0/0	0/0	0/1	0/0	0/1	0/1	0/1	0/0
Chromosome1-6520121	INS	PRECISE	52	0	0.577	0/0	0/0	0/0	0/0	0/0	0/0	0/0	0/0	0/0	0/0	0/1	0/0	0/0	0/0
Chromosome1-6520210	INS	PRECISE	7330	0	0	0/0	0/0	0/0	0/0	0/0	0/0	0/0	0/0	0/0	0/0	0/0	0/0	0/0	0/0
Chromosome1-6533312	INS	PRECISE	7695	0	0	0/0	0/0	0/0	0/0	0/0	0/0	0/0	0/0	0/0	0/0	0/0	0/0	0/1	0/0
Chromosome1-6541132	INS	PRECISE	70	0	0.577	0/0	0/0	0/0	0/0	0/0	0/0	0/0	0/0	0/1	0/0	0/0	0/1	0/0	0/0
Chromosome1-6576772	DEL	PRECISE	51	0	0	0/0	0/1	0/1	0/1	0/1	0/1	0/0	0/0	0/0	0/1	0/0	0/0	0/0	0/1
Chromosome1-6581098	BND	PRECISE	0	0	0	0/0	0/0	0/0	0/0	0/0	0/0	0/1	0/0	0/0	0/0	0/0	0/0	0/1	0/0
Chromosome1-6596283	BND	PRECISE	0	0	0	0/0	0/1	0/0	0/0	0/1	0/0	0/0	0/0	0/0	0/0	0/0	0/0	0/0	0/0
Chromosome1-6598108	BND	PRECISE	0	0	0	0/0	0/1	0/1	0/0	0/1	0/1	0/0	0/0	0/0	1/1	0/0	0/0	0/0	0/1
Chromosome1-6647004	DEL	PRECISE	51	0.5	0.5	0/0	0/0	0/0	0/0	0/0	0/0	0/0	0/0	0/1	0/0	0/1	0/1	0/1	0/0
Chromosome1-6651828	DEL	PRECISE	57	0	0	0/0	0/0	0/0	0/0	0/0	0/0	0/0	0/0	0/1	0/0	0/1	0/1	0/1	0/0
Chromosome1-6652392	DEL	PRECISE	1150	0	0	0/0	0/1	0/1	0/1	0/1	0/1	0/0	0/0	0/0	0/1	0/0	0/0	0/0	0/1
Chromosome1-6655235	DEL	PRECISE	52	0	0	0/0	0/1	0/1	1/1	0/1	0/1	0/0	0/0	0/1	0/1	0/1	0/1	0/1	1/1
Chromosome1-6656368	INS	PRECISE	1553	0	0	0/0	0/0	0/0	0/0	0/0	0/0	0/0	0/0	0/0	0/0	0/0	0/0	0/1	0/0
Chromosome1-6656932	INS	PRECISE	215	0	0.378	0/0	0/1	0/1	0/1	0/1	0/1	0/0	0/0	0/0	0/1	0/0	0/0	0/0	0/1
Chromosome1-6676126	DEL	PRECISE	1675	0	0	0/0	0/1	0/1	0/1	0/1	0/1	0/0	0/0	0/0	0/1	0/0	0/0	0/0	0/1
Chromosome1-6715009	DEL	PRECISE	1828	0	0	0/0	0/0	0/0	0/0	0/0	0/0	0/0	0/0	0/0	0/0	0/0	0/0	0/1	0/0
Chromosome1-6727218	DEL	PRECISE	2755	0	0	0/0	0/0	0/0	0/0	0/0	0/0	0/1	0/1	0/0	0/0	0/0	0/0	0/0	0/0
Chromosome1-6751078	DUP	PRECISE	98359	0	0	0/0	0/0	0/0	0/0	0/0	0/0	0/0	0/0	0/1	0/0	0/1	0/1	0/0	0/0
Chromosome1-6754372	INS	PRECISE	13282	0	0	0/0	0/0	0/0	0/0	0/0	0/0	0/0	0/0	0/0	0/0	0/0	0/0	0/0	0/0
Chromosome1-6755185	INS	PRECISE	7477	0	0	0/0	0/0	0/0	0/0	0/0	0/0	0/0	0/0	0/0	0/0	0/0	0/0	0/0	0/0
Chromosome1-6763101	DEL	PRECISE	430	0	0	0/0	0/0	0/0	0/1	0/0	0/0	0/0	0/0	0/0	0/0	0/0	0/0	0/0	0/1
Chromosome1-6769273	INS	PRECISE	51	0	0	0/0	0/0	0/0	0/0	0/0	0/0	0/1	0/1	0/1	0/0	0/1	0/1	0/1	0/0
Chromosome1-6771269	DEL	PRECISE	1983	0	0	0/0	0/0	0/0	0/0	0/0	0/0	0/0	0/0	0/0	0/0	0/1	0/0	0/0	0/0
Chromosome1-6792737	INS	PRECISE	218	0	0.516	0/0	0/0	0/0	0/0	0/0	0/0	0/1	0/1	0/1	0/0	1/1	0/1	0/1	0/0
Chromosome1-6797943	DEL	PRECISE	12535	0	0	0/0	0/1	0/1	1/1	0/1	0/1	0/0	0/0	0/0	0/1	0/0	0/0	0/0	1/1
Chromosome1-6818666	INS	PRECISE	9592	0	0	0/0	0/0	0/0	0/0	0/0	0/0	0/0	0/0	0/0	0/0	0/0	0/0	0/1	0/0
Chromosome1-6825306	INS	PRECISE	60	0	0	0/0	0/0	0/0	0/0	0/0	0/0	0/0	0/0	0/0	0/0	0/0	0/0	0/1	0/0
Chromosome1-6825713	DEL	PRECISE	15359	0	0	0/0	0/0	0/0	0/0	0/0	0/0	0/0	0/0	0/0	0/0	0/1	0/1	0/0	0/0
Chromosome1-6833936	INS	PRECISE	347	0	0.408	0/0	0/0	0/0	0/0	0/0	0/0	0/1	0/1	0/1	0/0	0/0	0/0	1/1	0/0
Chromosome1-6835491	DEL	PRECISE	2197	0	0	0/0	0/0	0/0	0/0	0/0	0/0	0/0	0/1	0/0	0/0	0/0	0/0	0/0	0/0

Chromosome1-6846917	DEL	PRECISE	275	0	0	0/0	0/0	0/0	0/0	0/0	0/0	0/1	0/1	0/0	0/0	0/0	0/0	0/0	0/0
Chromosome1-6847451	INS	PRECISE	62	0	0	0/0	0/0	0/0	0/0	0/0	0/0	0/0	0/0	0/1	0/0	0/1	0/1	0/0	0/0
Chromosome1-6850651	DEL	PRECISE	160	0	0	0/0	0/0	0/0	0/0	0/0	0/0	0/0	0/0	0/1	0/0	0/1	0/1	0/1	0/0
Chromosome1-6890357	DEL	PRECISE	1215	0	0	0/0	0/0	0/0	0/0	0/0	0/0	0/0	0/0	0/0	0/0	0/0	0/0	0/1	0/0
Chromosome1-6900304	INS	PRECISE	1100	0	0	0/0	0/0	0/0	0/0	0/0	0/0	0/0	0/0	0/0	0/0	0/0	0/0	0/1	0/0
Chromosome1-6903137	INS	PRECISE	50	0	0.577	0/0	0/0	0/0	0/0	0/0	0/0	0/0	0/0	0/1	0/0	0/1	0/1	0/0	0/0
Chromosome1-6904435	DEL	PRECISE	2859	0	0	0/0	0/0	0/0	0/0	0/0	0/0	0/0	0/0	0/0	0/0	0/0	0/0	0/1	0/0
Chromosome1-6904593	DEL	PRECISE	1320	0	0	0/0	0/0	0/0	0/0	0/0	0/0	0/0	0/0	0/1	0/0	0/1	0/1	0/0	0/0
Chromosome1-6911078	INS	PRECISE	385	0	0	0/0	0/0	0/0	0/0	0/0	0/0	0/0	0/0	0/0	0/0	0/0	0/0	0/1	0/0
Chromosome1-6915102	INS	PRECISE	697	0	0.756	0/0	0/1	0/1	0/1	0/1	0/1	0/0	0/0	0/0	0/1	0/0	0/0	0/0	0/1
Chromosome1-6915911	INS	PRECISE	8390	0	0	0/0	0/0	0/0	0/0	0/0	0/0	0/0	0/0	0/0	0/0	0/0	0/0	1/1	0/0
Chromosome1-6915915	BND	PRECISE	0	0	0	0/0	0/0	0/0	0/0	0/0	0/0	0/0	0/0	0/0	0/0	0/0	0/0	0/1	0/0
Chromosome1-6919202	DEL	PRECISE	83	0	0	0/0	0/0	0/0	0/0	0/0	0/0	0/1	1/1	0/0	0/0	0/0	0/0	0/0	0/0
Chromosome1-6932622	INS	PRECISE	403	0	0.577	0/0	0/0	0/0	0/0	0/0	0/0	0/0	0/0	0/1	0/0	0/1	0/1	0/0	0/0
Chromosome1-6934830	DEL	PRECISE	211	0	0	0/0	0/0	0/0	0/0	0/0	0/0	0/0	0/0	0/1	0/0	0/1	0/1	0/0	0/0
Chromosome1-6939237	DEL	PRECISE	1019	0	0	0/0	0/0	0/0	0/0	0/0	0/0	0/0	0/0	0/1	0/0	0/1	0/1	0/1	0/0
Chromosome1-6942819	BND	PRECISE	0	0	0	0/0	0/0	0/0	0/0	0/0	0/0	0/0	0/0	0/1	0/0	0/1	0/1	0/0	0/0
Chromosome1-6943835	DEL	PRECISE	981	0	0	0/0	0/1	0/1	0/1	1/1	1/1	0/0	0/0	0/0	1/1	0/0	0/0	0/0	1/1
Chromosome1-6949108	DEL	PRECISE	472	0	0	0/0	0/0	0/0	0/0	0/0	0/0	0/0	0/0	0/0	0/0	0/0	0/0	0/1	0/0
Chromosome1-6952331	DEL	PRECISE	886	0	0	0/0	0/0	0/0	0/0	0/0	0/0	0/0	0/0	0/0	0/0	0/0	0/0	0/1	0/0
Chromosome1-6953340	DEL	PRECISE	1072	0	0	0/0	0/0	0/0	0/0	0/0	0/0	0/0	0/0	0/0	0/0	0/0	0/0	0/1	0/0
Chromosome1-6956096	DEL	PRECISE	824	0	0	0/0	0/1	0/1	0/1	0/1	0/1	0/0	0/0	0/0	0/1	0/0	0/0	0/0	1/1
Chromosome1-6957878	DEL	PRECISE	157	0	0	0/0	0/0	0/0	0/0	0/0	0/0	0/0	0/0	0/1	0/0	0/0	0/1	0/0	0/0
Chromosome1-6958280	DEL	PRECISE	4851	0	0	0/0	0/0	0/0	0/0	0/0	0/0	0/0	0/0	0/1	0/0	0/0	0/1	0/0	0/0
Chromosome1-6958784	INS	PRECISE	730	0	0	0/0	0/0	0/0	0/0	0/0	0/0	0/0	0/0	0/0	0/0	0/0	0/0	0/1	0/0
Chromosome1-6960444	INS	PRECISE	1403	0	0	0/0	0/0	0/0	0/0	0/0	0/0	0/0	0/0	0/0	0/0	0/0	0/0	0/0	0/1
Chromosome1-6965714	INS	PRECISE	701	0	0	0/0	0/0	0/0	0/0	0/0	0/0	0/0	0/0	0/0	0/0	0/0	0/0	1/1	0/0
Chromosome1-6968387	DEL	PRECISE	626	0	0	0/0	0/1	0/0	0/1	0/1	0/1	0/0	0/0	0/0	0/0	0/0	0/0	0/1	1/1
Chromosome1-6969844	DEL	PRECISE	740	0	0	0/0	0/0	0/0	0/0	0/0	0/0	0/0	0/0	0/1	0/0	0/1	0/1	0/1	0/0
Chromosome1-6970121	INV	PRECISE	14782575	0	0	0/0	0/0	0/0	0/1	0/0	0/0	0/0	0/0	0/0	0/0	0/0	0/0	0/0	0/0
Chromosome1-6970691	DEL	PRECISE	144	0	0	0/0	0/1	0/1	0/1	0/1	0/1	0/0	0/0	0/0	0/1	0/0	0/0	0/0	0/1
Chromosome1-6972648	INS	PRECISE	61	0	0	0/0	0/0	0/0	0/0	0/0	0/0	0/0	0/0	0/0	0/0	0/0	0/0	0/1	0/0
Chromosome1-6974685	INS	PRECISE	139	0.439	0.277	0/0	0/1	0/1	1/1	0/1	0/1	0/1	0/1	0/1	0/1	0/1	0/1	0/1	1/1
Chromosome1-6987085	INS	PRECISE	19144	0	0	0/0	0/0	0/0	0/0	0/0	0/0	0/0	0/0	0/0	0/0	0/0	0/0	0/1	0/0
Chromosome1-6987197	DEL	PRECISE	90	0	0	0/0	0/0	0/0	0/0	0/0	0/0	0/0	0/0	0/0	0/0	0/0	0/1	0/0	0/0
Chromosome1-6989865	DEL	PRECISE	156	0	0	0/0	0/0	0/0	0/0	0/0	0/0	0/1	0/1	0/1	0/0	0/1	0/1	0/1	0/0
Chromosome1-6990054	INS	PRECISE	65	0	0	0/0	0/0	0/0	0/0	0/0	0/0	0/0	0/0	0/0	0/0	0/0	0/0	0/0	0/1
Chromosome1-6991449	DEL	PRECISE	243	0	0	0/0	0/1	0/1	1/1	0/1	0/1	0/1	0/1	0/1	0/1	0/1	0/1	0/1	1/1
Chromosome1-6993931	DEL	PRECISE	328	0	0	0/0	0/1	0/1	1/1	0/1	0/1	0/1	0/1	0/1	0/1	0/1	0/1	0/1	1/1
Chromosome1-6996723	INS	PRECISE	1366	0	0	0/0	0/0	0/0	0/1	0/0	0/0	0/0	0/0	0/0	0/0	0/0	0/0	0/0	0/0
Chromosome1-7001097	INS	PRECISE	401	0	0	0/0	0/0	0/0	0/0	0/0	0/0	0/0	0/0	0/0	0/0	0/0	0/0	0/0	0/1
Chromosome1-7002574	DEL	PRECISE	2632	0	0	0/0	0/0	0/0	0/0	0/0	0/0	0/0	0/0	0/1	0/0	0/1	0/1	1/1	0/0
Chromosome1-7006795	DEL	PRECISE	622	0	0	0/0	0/0	0/0	0/0	0/0	0/0	0/0	0/0	0/1	0/0	0/1	0/1	0/1	0/0
Chromosome1-7008499	DEL	PRECISE	59	0	0	0/0	0/0	0/0	0/0	0/0	0/0	0/0	0/0	0/1	0/0	0/1	0/1	0/1	0/0
Chromosome1-7009516	INS	PRECISE	161	0	0	0/0	0/0	0/0	0/0	0/0	0/0	0/0	0/0	0/1	0/0	0/1	0/1	0/1	0/0
Chromosome1-7011582	DEL	PRECISE	70	0	0	0/0	0/0	0/0	0/0	0/0	0/0	0/0	0/0	0/0	0/0	0/0	0/0	0/1	0/0
Chromosome1-7021392	INS	PRECISE	5587	0	0	0/0	0/0	0/0	0/0	0/0	0/0	0/0	0/0	0/0	1/1	0/0	0/0	0/0	0/0
Chromosome1-7023080	DEL	PRECISE	9894	0	0	0/0	0/0	0/0	0/0	0/0	0/0	0/0	0/0	0/0	0/0	0/0	0/0	0/0	0/1
Chromosome1-7023674	INS	PRECISE	133	0	0	0/0	0/0	0/0	0/1	0/0	0/0	0/0	0/0	0/0	0/0	0/0	0/0	0/0	0/0
Chromosome1-7024069	DEL	PRECISE	350	0	0	0/0	0/1	0/1	0/1	0/1	0/1	0/0	0/0	0/0	0/1	0/0	0/0	0/0	0/1
Chromosome1-7031577	DEL	PRECISE	206	0	0	0/0	0/0	0/0	0/0	0/0	0/0	0/0	0/0	0/1	0/0	0/1	0/1	0/1	0/0
Chromosome1-7035874	INS	PRECISE	338	0	0	0/0	0/0	0/0	0/0	0/0	0/0	0/0	0/0	0/0	0/0	0/0	0/0	0/0	0/1
Chromosome1-7043435	DEL	PRECISE	74	0	0	0/0	0/0	0/0	0/0	0/0	0/0	0/0	0/0	0/0	0/0	0/0	0/0	0/1	0/0
Chromosome1-7044757	DEL	PRECISE	250	0	0	0/0	0/0	0/0	0/0	0/0	0/0	0/0	0/0	0/1	0/0	0/1	0/0	0/1	0/0
Chromosome1-7045326	INS	PRECISE	53	0	0	0/0	0/0	0/0	0/0	0/0	0/0	0/0	0/0	0/0	0/0	0/0	0/0	0/1	0/0
Chromosome1-7047691	INS	PRECISE	696	0	0	0/0	0/0	0/0	0/0	0/0	0/0	0/0	0/0	0/0	0/0	0/0	0/0	0/1	0/0
Chromosome1-7054075	INS	PRECISE	7243	0	0	0/0	0/0	0/0	0/0	0/0	0/0	0/0	0/0	0/0	0/0	1/1	0/0	0/0	0/0
Chromosome1-7054724	DEL	PRECISE	55	0	0	0/0	0/0	0/0	0/0	0/0	0/0	0/0	0/0	0/1	0/0	0/1	0/0	0/1	0/0
Chromosome1-7058165	DEL	PRECISE	189	0	0	0/0	0/0	0/0	0/0	0/0	0/0	0/1	0/1	0/0	0/0	0/0	0/0	0/0	0/0
Chromosome1-7060660	INS	PRECISE	76	0	0.555	0/0	0/1	0/1	1/1	1/1	1/1	0/1	0/1	1/1	0/1	0/1	0/1	0/0	1/1
Chromosome1-7061101	DEL	PRECISE	1380	0	0	0/0	0/0	0/0	0/0	0/0	0/0	0/0	0/0	0/1	0/0	0/1	0/1	0/0	0/0
Chromosome1-7062596	BND	PRECISE	0	0	0	0/0	0/0	0/0	0/0	0/0	0/0	0/1	0/0	0/0	0/0	0/0	0/0	0/0	0/0
Chromosome1-7073246	INS	PRECISE	71	0	0	0/0	0/0	0/0	0/0	0/0	0/0	0/0	0/0	0/0	0/0	0/0	0/0	0/1	0/0
Chromosome1-7083976	DEL	PRECISE	60	0	0	0/0	0/0	0/0	0/1	0/1	0/1	0/0	0/0	0/1	0/0	0/0	0/0	0/0	0/0
Chromosome1-7084846	BND	PRECISE	0	0	0	0/0	0/0	0/0	0/0	0/0	0/0	0/1	0/1	0/0	0/0	0/0	0/0	0/0	0/0
Chromosome1-7085131	DEL	PRECISE	61	0	0	0/0	0/0	0/0	0/1	0/1	0/1	0/0	0/0	0/1	0/0	0/0	0/0	0/0	0/1
Chromosome1-7089299	DEL	PRECISE	166	0	0	0/0	0/0	0/0	0/1	0/1	0/1	0/0	0/0	0/1	0/0	0/0	0/0	0/0	0/1
Chromosome1-7089860	DEL	PRECISE	788	0	0	0/0	0/0	0/0	0/1	1/1	1/1	0/0	0/0	1/1	0/0	0/0	0/0	0/0	1/1
Chromosome1-7097316	INS	PRECISE	511	0	0.516	0/0	0/1	0/0	1/1	0/1	0/0	0/0	0/0	0/0	0/1	0/0	0/0	0/0	1/1
Chromosome1-7103665	DEL	PRECISE	712	0	0	0/0	0/0	0/1	1/1	0/1	0/0	0/0	0/0	0/0	0/1	0/0	0/0	0/0	1/1
Chromosome1-7115743	DEL	PRECISE	7341	0.756	0.378	0/0	0/1	0/1	1/1	0/1	0/1	0/0	0/0	0/0	0/1	0/0	0/0	0/0	0/1
Chromosome1-7132146	DEL	PRECISE	178	0	0	0/0	0/1	0/1	1/1	0/1	0/1	0/0	0/0	0/0	0/1	0/0	0/0	0/0	1/1
Chromosome1-7144282	DEL	PRECISE	735	0	0	0/0	0/1	0/1	1/1	1/1	1/1	0/0	0/0	1/1	0/1	0/0	0/0	0/0	1/1

Chromosome1-7166871 DEL	PRECISE	1330	0	0	0/0	0/1	0/1	1/1	1/1	1/1	0/0	0/0	1/1	0/1	0/0	0/0	0/0	1/1
Chromosome1-7193169 INS	PRECISE	130	0.756	0.816	0/0	0/1	0/0	0/1	0/0	0/1	0/0	0/0	0/0	0/0	0/0	0/0	0/0	1/1
Chromosome1-7194069 INS	PRECISE	650	0	0.756	0/0	1/1	0/1	1/1	0/0	0/1	0/0	0/0	0/0	0/1	0/0	0/0	0/0	1/1
Chromosome1-7195342 DEL	PRECISE	2246	0	0	0/0	0/1	0/1	1/1	0/1	0/1	0/0	0/0	0/0	0/1	0/0	0/0	0/0	1/1
Chromosome1-7198357 DEL	PRECISE	289	0	0	0/0	0/0	0/0	0/0	0/0	0/0	0/0	0/0	0/1	0/0	0/0	0/1	0/0	0/0
Chromosome1-7201094 INS	PRECISE	237	0	0.707	0/0	0/0	0/0	0/0	0/0	0/0	0/0	0/0	0/1	0/0	0/0	0/0	0/0	0/0
Chromosome1-7201712 BND	PRECISE	0	0	0	0/0	0/0	0/0	0/0	0/0	0/0	0/0	0/0	0/0	0/0	0/0	0/0	0/1	0/0
Chromosome1-7202373 DEL	PRECISE	864	0	0	0/0	0/0	0/0	0/0	0/0	0/0	0/0	0/0	0/1	0/0	0/0	0/1	0/0	0/0
Chromosome1-7205491 DEL	PRECISE	62	0	0	0/0	0/1	0/1	0/1	0/1	0/1	0/0	0/0	0/0	0/1	0/0	0/0	0/0	0/1
Chromosome1-7207971 DEL	PRECISE	51	0	0	0/0	0/0	0/0	0/0	0/0	0/0	0/0	0/0	0/1	0/0	0/1	0/1	0/0	0/0
Chromosome1-7214788 INS	PRECISE	2180	0	0.707	0/0	0/0	0/0	0/0	0/0	0/0	0/1	0/1	0/0	0/0	0/0	0/0	0/0	0/0
Chromosome1-7225847 DEL	PRECISE	994	0	0	0/0	0/1	0/1	0/1	0/1	1/1	0/1	0/1	0/1	0/1	0/1	0/1	0/1	1/1
Chromosome1-7227687 INS	PRECISE	127	0	0.816	0/0	0/0	0/0	0/0	0/0	0/0	0/0	0/0	0/1	0/0	0/1	0/1	0/1	0/0
Chromosome1-7235856 DEL	PRECISE	209	0	0	0/0	0/1	0/1	0/1	0/1	0/1	0/0	0/0	0/0	0/1	0/0	0/0	0/0	1/1
Chromosome1-7237443 DEL	PRECISE	75	0	0	0/0	0/1	0/1	0/1	0/1	0/1	0/0	0/0	0/0	0/1	0/0	0/0	0/0	0/1
Chromosome1-7238203 INS	PRECISE	78	0	0	0/0	0/0	0/0	0/0	0/0	0/0	0/0	0/0	0/1	0/0	0/1	0/1	0/1	0/0
Chromosome1-7247288 INS	PRECISE	176	0	0	0/0	0/0	0/0	0/0	0/0	0/0	0/0	0/0	0/0	0/0	0/0	0/0	0/0	0/1
Chromosome1-7257989 INS	PRECISE	2735	0	0	0/0	0/0	0/0	0/0	0/0	0/0	0/0	0/0	0/0	0/0	0/0	0/0	0/1	0/0
Chromosome1-7263377 DEL	PRECISE	1007	0	0	0/0	0/1	0/1	0/1	0/1	0/1	0/1	0/1	0/1	0/1	0/1	0/1	0/1	1/1
Chromosome1-7283346 INS	PRECISE	692	0	0	0/0	0/0	0/0	0/0	0/0	0/0	0/0	0/0	0/0	0/0	0/0	0/0	0/0	0/1
Chromosome1-7298513 DEL	PRECISE	137	0	0	0/0	0/0	0/0	0/0	0/0	0/0	0/0	0/0	0/0	0/0	0/1	0/0	0/1	0/0
Chromosome1-7300806 DEL	PRECISE	2077	0	0	0/0	0/1	0/1	0/0	0/1	0/1	0/0	0/0	0/0	0/1	0/0	0/0	0/0	0/1
Chromosome1-7301732 DEL	PRECISE	87	0	0	0/0	0/0	0/0	0/0	0/0	0/0	0/0	0/0	0/0	0/0	0/0	0/0	0/1	0/0
Chromosome1-7308387 DEL	PRECISE	179	0	0	0/0	0/0	0/1	0/0	0/0	0/1	0/0	0/0	0/0	0/1	0/0	0/0	0/0	0/1
Chromosome1-7308720 INS	PRECISE	138	0	0	0/0	0/0	0/1	0/0	0/0	0/1	0/0	0/0	0/0	0/1	0/0	0/0	0/0	0/1
Chromosome1-7308820 DEL	PRECISE	1736	0	0	0/0	0/0	0/1	0/0	0/0	0/1	0/0	0/0	0/0	0/1	0/0	0/0	0/0	0/1
Chromosome1-7310684 DEL	PRECISE	19450	0	0	0/0	0/0	0/1	0/0	0/0	0/1	0/0	0/0	0/0	0/1	0/0	0/0	0/0	0/1
Chromosome1-7313683 DEL	PRECISE	12223	0	0	0/0	0/0	0/0	0/0	0/0	0/0	0/0	0/0	0/0	0/0	0/0	0/0	0/0	0/1
Chromosome1-7319244 DEL	PRECISE	60	0	0	0/0	0/0	0/0	0/0	0/0	0/0	0/1	0/1	0/0	0/0	0/0	0/0	0/0	0/0
Chromosome1-7319321 DEL	PRECISE	5825	0	0	0/0	0/0	0/0	0/0	0/0	0/0	0/1	0/1	0/0	0/0	0/0	0/0	0/0	0/0
Chromosome1-7332747 DEL	PRECISE	250	0.5	0.5	0/0	0/0	0/0	0/0	0/0	0/0	0/0	0/0	0/1	0/0	0/1	0/1	0/0	0/0
Chromosome1-7337666 INS	PRECISE	112	0	0.316	0/0	0/1	0/1	0/0	0/1	0/1	0/0	0/0	0/1	0/1	0/1	0/1	0/0	0/1
Chromosome1-7340118 DEL	PRECISE	88	0	0	0/0	0/0	0/0	0/0	0/0	0/0	0/0	0/0	0/0	0/0	0/0	0/0	0/0	0/1
Chromosome1-7341032 INS	PRECISE	1302	0	0.957	0/0	0/0	0/0	0/0	0/0	0/0	0/0	0/0	0/0	0/1	0/0	0/0	0/0	0/0
Chromosome1-7342770 DEL	PRECISE	1163	0	0	0/0	0/0	0/0	0/0	1/1	1/1	0/0	0/0	1/1	0/0	0/0	0/0	0/0	0/0
Chromosome1-7352193 DEL	PRECISE	8276	0	0	0/0	0/0	0/0	0/0	0/0	0/0	0/0	0/0	0/0	0/0	0/0	0/0	0/0	0/1
Chromosome1-7353748 DEL	PRECISE	23479	0	0	0/0	0/0	0/0	0/0	0/0	0/0	0/0	0/0	0/0	0/1	0/0	0/0	0/0	0/0
Chromosome1-7360558 DEL	PRECISE	3613	0	0	0/0	0/0	0/0	0/0	0/0	0/0	0/0	0/0	0/0	0/0	0/0	0/0	0/0	0/1
Chromosome1-7363183 DEL	PRECISE	136	0	0	0/0	0/0	0/0	0/1	0/0	0/0	0/0	0/0	0/0	0/0	0/0	0/0	0/0	0/0
Chromosome1-7364151 DEL	PRECISE	13073	0	0	0/0	0/0	0/0	0/0	0/0	0/0	0/0	0/0	0/0	0/0	0/0	0/0	0/0	0/1
Chromosome1-7365170 INS	PRECISE	51	0	0	0/0	0/0	0/0	0/0	0/0	0/0	0/0	0/0	0/0	0/0	0/0	0/0	0/0	0/1
Chromosome1-7371249 INS	PRECISE	150	0	0.408	0/0	0/0	0/0	0/1	1/1	1/1	0/1	0/1	1/1	0/0	0/0	0/0	0/0	0/0
Chromosome1-7371251 INV	PRECISE	1286	0	0	0/0	0/0	0/0	0/0	0/0	0/0	0/0	0/0	0/0	0/0	0/0	0/0	0/0	0/1
Chromosome1-7380584 INS	PRECISE	350	0	0	0/0	0/0	0/0	0/0	0/0	0/0	0/0	0/0	0/0	0/0	0/0	0/0	0/0	0/1
Chromosome1-7382313 INS	PRECISE	1271	0	0	0/0	0/0	0/0	0/1	0/0	0/0	0/0	0/0	0/0	0/0	0/0	0/0	0/0	0/0
Chromosome1-7388544 DEL	PRECISE	137	0	0	0/0	0/1	0/1	0/1	1/1	1/1	0/1	0/1	1/1	0/1	0/1	0/1	0/1	1/1
Chromosome1-7389289 INS	PRECISE	224	0	0	0/0	0/0	0/0	0/0	0/0	0/0	0/0	0/0	0/0	0/0	0/1	0/0	0/0	0/0
Chromosome1-7390388 INS	PRECISE	1182	0	0	0/0	0/0	0/0	0/1	0/0	0/0	0/0	0/0	0/0	0/0	0/0	0/0	0/0	0/0
Chromosome1-7391532 DEL	PRECISE	94	0	0	0/0	0/0	0/0	0/0	0/0	0/0	0/0	0/0	0/0	0/0	0/0	0/0	0/1	0/0
Chromosome1-7393575 INS	PRECISE	8512	0	0	0/0	0/0	0/0	0/0	0/0	0/0	0/0	0/0	0/0	0/0	0/1	0/0	0/0	0/0
Chromosome1-7419298 DEL	PRECISE	143	0	0	0/0	0/0	0/0	0/0	0/0	0/0	0/1	0/1	0/0	0/0	0/0	0/0	0/0	0/1
Chromosome1-7430385 INS	PRECISE	1331	0	0	0/0	0/0	0/0	0/0	0/0	0/0	0/0	0/0	0/0	0/0	1/1	0/0	0/0	0/0
Chromosome1-7431723 DEL	PRECISE	467	0	0	0/0	0/0	0/0	0/0	0/0	0/0	0/0	0/0	0/0	0/0	0/1	0/0	0/0	0/0
Chromosome1-7436199 DEL	PRECISE	68	0	0	0/0	0/0	0/0	0/0	0/0	0/0	0/0	0/0	0/1	0/0	0/1	0/1	0/1	0/0
Chromosome1-7436665 INS	PRECISE	1144	0	0	0/0	0/0	0/0	0/0	0/0	0/0	0/0	0/0	0/0	0/0	0/0	0/0	0/0	0/1
Chromosome1-7443753 DEL	PRECISE	238	0	0	0/0	0/0	0/0	0/0	0/0	0/0	0/0	0/0	0/1	0/0	0/1	0/1	0/1	0/0
Chromosome1-7444651 DEL	PRECISE	80	0	0	0/0	0/0	0/0	0/0	0/0	0/0	0/0	0/0	0/1	0/0	0/0	0/1	0/1	0/0
Chromosome1-7457370 DEL	PRECISE	8747	0	0	0/0	0/0	0/0	0/0	0/0	0/0	0/0	0/0	0/0	0/0	0/0	0/0	0/0	0/1
Chromosome1-7458640 DEL	PRECISE	620	0	0	0/0	0/0	0/0	0/1	0/0	0/0	0/0	0/0	0/0	0/0	0/0	0/0	0/0	0/0
Chromosome1-7468332 INS	PRECISE	1405	0	0.753	0/0	0/1	0/1	0/0	0/1	0/1	0/0	0/0	0/0	0/1	0/0	0/0	0/0	0/1
Chromosome1-7484414 DEL	PRECISE	1339	0	0	0/0	0/1	0/1	0/1	1/1	1/1	0/0	0/0	1/1	0/1	0/0	0/0	0/0	1/1
Chromosome1-7502750 DEL	PRECISE	673	0	0	0/0	0/0	0/0	0/0	0/0	0/0	0/0	0/0	0/0	0/0	0/0	0/0	0/0	0/1
Chromosome1-7536468 INS	PRECISE	4286	0	0	0/0	0/0	0/0	0/0	0/0	0/0	0/0	0/0	0/0	0/0	0/0	0/0	0/0	1/1
Chromosome1-7541389 DEL	PRECISE	5427	0	0	0/0	0/1	0/1	0/0	1/1	1/1	0/0	0/0	1/1	0/1	0/0	0/0	0/0	1/1
Chromosome1-7546921 DEL	PRECISE	4767	0	0	0/0	0/1	0/1	1/1	1/1	1/1	0/1	1/1	1/1	0/1	0/0	0/0	0/0	1/1
Chromosome1-7556189 INS	PRECISE	196	0	0	0/0	0/0	0/0	0/0	0/0	0/0	0/0	0/0	0/0	0/0	0/0	0/0	0/0	0/1
Chromosome1-7556436 DEL	PRECISE	997	0	0	0/0	0/0	0/0	0/0	0/0	0/0	0/0	0/0	0/1	0/0	0/1	0/1	0/0	0/0
Chromosome1-7557282 INS	PRECISE	55	0	0	0/0	0/0	0/0	0/0	0/1	0/1	0/0	0/0	0/1	0/0	0/0	0/0	0/0	0/0
Chromosome1-7570590 DEL	PRECISE	2842	0	0	0/0	0/0	0/0	0/0	0/0	0/0	0/0	0/0	0/0	0/0	0/0	0/0	0/1	0/0
Chromosome1-7584499 DEL	PRECISE	452	0	0	0/0	0/0	0/0	0/0	0/0	0/0	0/0	0/0	0/1	0/0	0/1	1/1	0/0	0/1
Chromosome1-7592394 INS	PRECISE	65	0	0	0/0	0/1	0/1	0/0	0/1	0/1	0/0	0/0	0/1	0/1	0/1	1/1	0/1	1/1
Chromosome1-7595725 INS	PRECISE	330	0	0	0/0	0/0	0/0	0/0	0/0	0/0	0/0	0/0	0/0	0/0	0/1	0/0	0/0	0/0
Chromosome1-7597366 DEL	PRECISE	137	0	0	0/0	0/0	0/0	0/0	0/0	0/0	0/0	0/0	0/1	0/0	0/1	0/1	0/1	0/1

Chromosome1-7600231	INS	PRECISE	161	0	0	0/0	0/1	0/1	0/0	0/1	0/1	0/0	0/0	0/0	0/1	0/0	0/0	0/0	0/1
Chromosome1-7604725	DEL	PRECISE	114	0.5	0	0/0	0/0	0/0	0/0	0/0	0/0	0/0	0/0	0/1	0/0	0/1	0/1	0/1	0/0
Chromosome1-7606672	DEL	PRECISE	135	0	0	0/0	0/0	0/0	0/0	0/0	0/0	0/0	0/0	0/0	0/0	0/0	0/0	0/1	0/0
Chromosome1-7614802	INS	PRECISE	175	0	0	0/0	0/0	0/0	0/1	0/0	0/0	0/0	0/1	0/0	0/0	0/0	0/0	0/0	0/0
Chromosome1-7614802	BND	PRECISE	0	0	0	0/0	0/1	0/0	0/0	0/0	0/0	0/0	0/0	0/0	0/0	0/0	0/0	0/0	0/0
Chromosome1-7619620	DEL	PRECISE	699	0	0	0/0	0/1	0/1	0/1	1/1	1/1	0/1	0/1	1/1	0/0	0/1	0/0	0/1	1/1
Chromosome1-7624883	DEL	PRECISE	5467	0	0	0/0	0/1	0/1	0/0	0/1	1/1	0/0	0/0	0/0	0/0	0/0	0/0	0/0	1/1
Chromosome1-7631213	DEL	PRECISE	458	0	0	0/0	0/0	0/0	0/0	0/0	0/0	0/0	0/0	0/1	0/0	0/1	0/1	0/0	0/0
Chromosome1-7637117	DEL	PRECISE	2120	0	0	0/0	0/0	0/0	0/0	0/0	0/0	0/0	0/0	0/0	0/0	0/0	0/0	0/0	0/1
Chromosome1-7645024	INS	PRECISE	211	0	0	0/0	0/1	0/1	0/0	0/1	0/1	0/0	0/0	0/0	0/1	0/0	0/0	0/0	0/1
Chromosome1-7649930	DEL	PRECISE	98	0.5	0.5	0/0	0/0	0/0	0/0	0/0	0/0	0/0	0/0	0/1	0/0	0/1	0/1	0/1	0/0
Chromosome1-7655273	DEL	PRECISE	778	0	0	0/0	0/0	0/0	0/0	0/0	0/0	0/0	0/0	0/1	0/0	0/1	0/1	0/0	0/0
Chromosome1-7657499	BND	PRECISE	0	0	0	0/0	0/0	0/0	0/0	0/0	0/0	0/0	0/0	0/1	0/0	0/0	0/1	0/0	0/0
Chromosome1-7657569	INS	PRECISE	6137	0	0	0/0	0/0	0/0	0/0	0/0	0/0	0/0	0/0	0/0	0/0	0/0	0/0	0/1	0/0
Chromosome1-7657605	DEL	PRECISE	1550	0	0	0/0	0/0	0/0	0/0	0/0	0/0	0/0	0/0	0/0	0/0	0/0	0/0	0/0	0/1
Chromosome1-7659009	INS	PRECISE	595	0	0	0/0	0/0	0/0	0/0	0/0	0/0	0/0	0/0	0/0	0/0	0/1	0/0	0/0	0/0
Chromosome1-7659850	DEL	PRECISE	60	0	0	0/0	0/0	0/0	0/1	0/0	0/0	0/0	0/0	0/0	0/0	0/0	0/0	0/0	0/0
Chromosome1-7661567	DEL	PRECISE	181	0	0	0/0	0/0	0/0	0/0	0/0	0/0	0/0	0/0	0/1	0/0	0/1	0/1	0/1	0/0
Chromosome1-7668297	DEL	PRECISE	86	0.632	0.316	0/0	0/1	0/1	0/0	0/1	0/1	0/0	0/0	0/1	0/1	0/1	0/1	0/1	0/1
Chromosome1-7678916	INS	PRECISE	1000	0.5	0.957	0/0	0/0	0/0	0/0	0/0	0/0	0/0	0/0	0/1	0/0	1/1	0/1	0/1	0/0
Chromosome1-7680602	INS	PRECISE	884	0	0	0/0	0/0	0/0	0/0	0/0	0/0	0/0	0/0	0/0	0/0	0/0	0/0	0/1	0/0
Chromosome1-7684269	DEL	PRECISE	50	0	0	0/0	0/0	0/0	0/0	0/0	0/0	0/0	0/0	1/1	0/0	1/1	0/1	0/1	0/0
Chromosome1-7692086	INS	PRECISE	1136	0	0.816	0/0	0/0	0/0	0/0	0/0	0/0	0/0	0/0	0/1	0/0	1/1	0/1	0/1	0/0
Chromosome1-7692775	DEL	PRECISE	66	0	0	0/0	0/0	0/0	0/0	0/0	0/0	0/0	0/0	0/1	0/0	1/1	0/1	0/1	0/0
Chromosome1-7696104	DEL	PRECISE	1378	0	0	0/0	0/0	0/0	0/0	0/0	0/0	0/0	0/0	0/1	0/0	0/1	0/1	0/1	0/0
Chromosome1-7697693	INS	PRECISE	9511	0	0	0/0	0/0	0/0	0/0	0/0	0/0	0/0	0/0	0/0	0/0	0/0	0/0	0/0	0/1
Chromosome1-7702706	INS	PRECISE	625	0	0	0/0	0/0	0/0	0/0	0/0	0/0	0/0	0/0	0/0	0/0	0/0	0/0	0/0	0/1
Chromosome1-7703962	DEL	PRECISE	51	0	0	0/0	0/0	0/0	0/0	0/0	0/0	0/0	0/0	0/0	0/0	0/0	0/0	0/1	0/0
Chromosome1-7709763	INV	PRECISE	2168	0	0	0/0	0/0	0/0	0/0	0/0	0/0	0/0	0/0	0/1	0/0	0/1	0/1	0/1	0/0
Chromosome1-7713211	INS	PRECISE	12457	0	0	0/0	0/0	0/0	0/1	0/0	0/0	0/0	0/0	0/0	0/0	0/0	0/0	0/0	0/0
Chromosome1-7721890	INS	PRECISE	12447	0	0	0/0	0/0	0/0	0/0	0/0	0/0	0/0	0/0	0/0	0/0	0/0	0/0	0/0	0/1
Chromosome1-7724150	DEL	PRECISE	1099	0	0	0/0	0/0	0/0	0/0	0/0	0/0	0/0	0/0	0/1	0/0	0/1	0/1	0/1	0/0
Chromosome1-7728043	BND	PRECISE	0	0	0	0/0	0/0	0/0	0/0	0/0	0/0	0/0	0/0	0/1	0/0	0/1	0/1	0/0	0/0
Chromosome1-7731518	DEL	PRECISE	426	0	0	0/0	0/0	0/0	0/0	0/0	0/0	0/0	0/0	0/1	0/0	0/1	0/1	0/1	0/0
Chromosome1-7732166	INS	PRECISE	59	0	0	0/0	0/0	0/0	0/0	0/0	0/0	0/0	0/0	0/1	0/0	0/1	0/1	0/1	0/0
Chromosome1-7732181	DEL	PRECISE	56	0	0	0/0	0/1	0/1	0/0	0/1	0/1	0/0	0/0	0/0	0/1	0/0	0/0	0/0	0/1
Chromosome1-7732194	INS	PRECISE	5584	0	0	0/0	0/0	0/0	0/1	0/0	0/0	0/0	0/0	0/0	0/0	0/0	0/0	0/0	0/0
Chromosome1-7743325	INS	PRECISE	5624	0	0	0/0	0/0	0/0	0/0	0/0	0/0	0/0	0/0	0/0	0/0	0/0	0/0	0/0	1/1
Chromosome1-7748270	INS	PRECISE	733	0	0	0/0	0/0	0/0	0/0	0/0	0/0	0/0	0/0	0/0	0/0	0/0	0/0	0/0	0/1
Chromosome1-7748710	DEL	PRECISE	79	0	0	0/0	0/1	0/1	0/0	0/1	0/1	0/0	0/0	0/1	0/1	1/1	1/1	0/1	1/1
Chromosome1-7753753	INS	PRECISE	62	0.408	0	0/0	0/1	0/1	0/0	0/1	0/1	0/0	0/0	0/0	0/1	0/0	0/0	0/0	0/1
Chromosome1-7755524	DEL	PRECISE	104	0	0	0/0	0/1	0/1	1/1	0/1	0/1	0/1	0/1	0/1	0/1	0/1	0/1	0/1	1/1
Chromosome1-7789340	INS	PRECISE	58	0	0.405	0/0	0/1	0/1	0/1	0/1	0/1	0/1	0/1	0/0	0/0	0/0	0/0	0/0	1/1
Chromosome1-7790219	INS	PRECISE	611	0	0.548	0/0	0/1	1/1	0/0	0/1	0/1	0/0	0/0	0/0	0/1	0/0	0/0	0/0	1/1
Chromosome1-7790391	DEL	PRECISE	1706	0	0	0/0	0/0	0/0	0/0	0/0	0/0	0/0	0/0	0/0	0/0	0/0	0/0	0/0	0/1
Chromosome1-7799219	DEL	PRECISE	840	0	0	0/0	0/0	0/0	0/0	0/0	0/0	0/0	0/0	0/1	0/0	0/0	0/1	0/1	0/0
Chromosome1-7806223	DEL	PRECISE	75	0	0	0/0	0/0	0/0	0/0	0/0	0/0	0/0	0/0	0/1	0/0	0/1	0/1	0/1	0/0
Chromosome1-7811660	INS	PRECISE	76	0	0	0/0	0/1	0/1	0/0	0/1	0/1	0/0	0/0	0/0	0/1	0/0	0/0	0/0	0/1
Chromosome1-7816652	DEL	PRECISE	798	0	0	0/0	0/0	0/0	0/0	0/0	0/0	0/0	0/0	0/1	0/0	0/1	0/1	0/1	0/0
Chromosome1-7828405	DEL	PRECISE	121	0	0	0/0	0/0	0/0	0/1	0/0	0/0	0/0	0/0	0/0	0/0	0/0	0/0	0/0	0/0
Chromosome1-7834950	DEL	PRECISE	51	0	0	0/0	0/0	0/0	0/0	0/0	0/0	0/0	0/0	0/0	0/0	0/0	0/0	0/0	0/1
Chromosome1-7835035	DEL	PRECISE	1332	0	0	0/0	0/0	0/0	0/0	0/0	0/0	0/0	0/0	0/1	0/0	0/1	0/1	0/1	0/1
Chromosome1-7842014	DEL	PRECISE	87	0	0	0/0	0/0	0/0	0/0	0/0	0/0	0/0	0/0	0/0	0/0	0/0	0/0	0/0	0/1
Chromosome1-7852925	DEL	PRECISE	89	0	0	0/0	0/0	0/0	0/0	0/0	0/0	0/0	0/0	0/0	0/0	0/0	0/0	0/0	0/1
Chromosome1-7855926	INS	PRECISE	53	0	0	0/0	0/0	0/0	0/0	0/0	0/0	0/0	0/0	0/0	0/0	0/0	0/0	0/0	0/1
Chromosome1-7858709	INS	PRECISE	17088	0	0	0/0	0/0	0/0	0/0	0/1	0/0	0/0	0/0	0/0	0/0	0/0	0/0	0/0	0/0
Chromosome1-7869450	INS	PRECISE	391	0	0	0/0	0/0	0/0	0/0	0/0	0/0	0/0	0/0	0/0	0/0	0/0	0/0	0/0	0/1
Chromosome1-7872172	DEL	PRECISE	810	0	0	0/0	0/0	0/0	0/0	0/0	0/0	0/0	0/0	0/0	0/0	0/0	0/0	0/0	0/1
Chromosome1-7881532	INS	PRECISE	1567	0	0	0/0	0/0	0/0	0/0	0/0	0/0	0/0	0/0	0/0	0/0	0/0	0/0	0/0	0/1
Chromosome1-7881642	BND	PRECISE	0	0	0	0/0	0/0	0/0	0/1	0/0	0/0	0/1	0/1	0/0	0/0	0/0	0/0	0/0	0/0
Chromosome1-7881651	BND	PRECISE	0	0	0	0/0	0/0	0/0	0/1	0/0	0/0	0/0	0/0	0/0	0/0	0/0	0/0	0/0	0/0
Chromosome1-7885998	DEL	PRECISE	12733	0	0	0/0	0/0	0/0	0/0	0/0	0/0	0/0	0/0	0/0	0/0	0/1	0/0	0/0	0/0
Chromosome1-7891359	DEL	PRECISE	125	0	0	0/0	0/0	0/0	0/1	0/0	0/0	0/1	0/1	0/0	0/0	0/0	0/0	0/0	0/0
Chromosome1-7891749	INS	PRECISE	59	0	0.548	0/0	0/0	0/0	0/1	0/0	0/0	0/1	0/1	0/1	0/0	0/0	0/1	0/0	0/0
Chromosome1-7894060	DEL	PRECISE	130	0	0	0/0	0/0	0/0	0/0	0/0	0/0	0/0	0/0	0/1	0/0	0/0	0/1	0/0	0/0
Chromosome1-7903116	BND	PRECISE	0	0	0	0/0	0/0	0/0	0/1	0/0	0/0	0/0	0/0	0/0	0/0	0/0	0/0	0/0	0/0
Chromosome1-7908377	DEL	PRECISE	466	0	0	0/0	0/1	0/1	0/1	0/1	0/0	0/1	0/1	0/1	0/1	0/0	1/1	0/1	1/1
Chromosome1-7914567	DEL	PRECISE	76	0	0	0/0	0/0	0/0	0/0	0/0	0/0	0/0	0/0	0/0	0/0	0/1	0/0	0/0	0/0
Chromosome1-7921741	DEL	PRECISE	118	0	0	0/0	0/0	0/0	0/0	0/0	0/0	0/0	0/0	0/0	0/0	0/0	0/0	0/0	0/1
Chromosome1-7922302	INS	PRECISE	347	0	0	0/0	0/0	0/0	0/0	0/0	0/0	0/0	0/0	0/1	0/0	0/0	1/1	0/0	0/0
Chromosome1-7925052	INS	PRECISE	136	0	0.5	0/0	0/0	0/0	0/0	0/0	0/0	0/0	0/0	0/1	0/0	0/1	1/1	1/1	0/0
Chromosome1-7927693	DEL	PRECISE	278	0	0	0/0	0/0	0/0	0/0	0/0	0/0	0/0	0/0	0/1	0/0	1/1	1/1	1/1	0/1
Chromosome1-7928751	INS	PRECISE	877	0	0	0/0	0/0	0/0	0/0	0/0	0/0	0/0	0/0	0/0	0/0	0/0	0/0	0/0	0/1

Chromosome1-7934988 DEL	PRECISE	82	0	0	0/0	0/0	0/0	0/0	0/0	0/0	0/0	0/0	0/1	0/0	0/0	0/1	0/0	0/0
Chromosome1-7935370 INS	PRECISE	357	0	0	0/0	0/0	0/0	0/0	0/0	0/0	0/0	0/0	0/0	0/0	0/0	0/0	1/1	0/0
Chromosome1-7935511 INS	PRECISE	9653	0	0	0/0	0/0	0/0	0/0	0/0	0/0	0/0	0/0	0/0	0/0	0/0	0/0	0/0	0/1
Chromosome1-7937310 INS	PRECISE	884	0	0	0/0	0/0	0/0	0/0	0/0	0/0	0/0	0/0	0/0	0/0	0/0	0/0	0/0	0/1
Chromosome1-7939788 INS	PRECISE	3075	0	0	0/0	0/0	0/0	0/0	0/0	0/0	0/0	0/0	0/0	0/0	1/1	0/0	0/0	0/0
Chromosome1-7944885 DEL	PRECISE	3156	0	0	0/0	0/1	0/1	0/0	0/0	0/1	0/0	0/0	0/0	0/1	0/0	0/0	0/0	0/0
Chromosome1-7949356 INS	PRECISE	1359	0	0	0/0	0/0	0/0	0/0	0/0	0/0	0/0	0/0	0/0	0/0	0/1	0/0	0/0	0/0
Chromosome1-7950598 DEL	PRECISE	120	0	0	0/0	0/0	0/0	0/0	0/0	0/0	0/0	0/0	0/0	0/0	0/1	0/0	0/0	0/0
Chromosome1-7951701 INS	PRECISE	50	0	0	0/0	0/0	0/0	0/0	0/0	0/0	0/0	0/0	0/0	0/0	0/1	0/0	0/0	0/0
Chromosome1-7960243 INS	PRECISE	70	0	0	0/0	0/0	0/0	0/0	0/0	0/0	0/0	0/0	0/0	0/0	0/0	0/0	0/0	0/1
Chromosome1-7977595 INS	PRECISE	996	0	0	0/0	0/0	0/0	0/0	0/0	0/0	0/0	0/0	0/0	0/0	0/0	0/0	0/0	0/1
Chromosome1-7978108 DEL	PRECISE	57	0	0	0/0	0/0	0/0	0/0	0/0	0/0	0/0	0/0	0/0	0/0	0/1	0/0	0/0	0/0
Chromosome1-7980220 INS	PRECISE	137	0	0	0/0	0/1	0/1	0/0	0/1	0/1	0/0	0/0	0/0	0/1	0/0	0/0	0/0	0/1
Chromosome1-7997275 DEL	PRECISE	2645	0.577	0.577	0/0	0/0	0/0	0/0	0/0	0/0	0/0	0/0	0/1	0/0	0/1	0/1	0/0	0/0
Chromosome1-7998849 DEL	PRECISE	463	0	0	0/0	0/0	0/0	0/1	0/0	0/0	0/1	0/1	0/0	0/0	0/0	0/0	0/0	0/0
Chromosome1-8007716 INS	PRECISE	2431	0	0.816	0/0	0/0	0/0	0/1	0/0	0/0	0/1	0/1	0/0	0/0	0/0	0/0	0/0	0/1
Chromosome1-8009730 INS	PRECISE	75	0	0.408	0/0	0/1	0/1	0/0	0/1	0/1	0/0	0/0	0/0	0/1	0/0	0/0	0/0	0/1
Chromosome1-8011872 DEL	PRECISE	91	0	0	0/0	0/0	0/0	0/1	0/0	0/0	0/1	0/1	0/0	0/0	0/0	0/0	0/0	0/1
Chromosome1-8013359 INS	PRECISE	115	0	0.577	0/0	0/0	0/0	0/1	0/0	0/0	0/1	0/1	0/0	0/0	0/0	0/0	0/0	0/0
Chromosome1-8024344 DEL	PRECISE	2535	0	0	0/0	0/0	0/0	0/0	0/0	0/0	0/0	0/0	0/0	0/0	0/1	0/0	0/0	0/0
Chromosome1-8030013 INS	PRECISE	5462	0	0	0/0	0/0	0/0	0/0	0/0	0/0	0/0	0/0	0/0	0/0	0/0	0/0	0/0	0/1
Chromosome1-8030013 BND	PRECISE	0	0	0	0/0	0/0	0/0	0/0	0/0	0/0	0/0	0/0	0/0	0/0	0/0	0/0	0/0	0/1
Chromosome1-8030126 INS	PRECISE	676	0	0	0/0	0/0	0/0	0/0	0/0	0/0	0/0	0/0	0/0	0/0	0/0	0/0	0/1	0/0
Chromosome1-8031345 DEL	PRECISE	111	0	0	0/0	0/0	0/0	0/0	0/0	0/0	0/0	0/0	0/1	0/0	0/1	0/1	0/1	0/0
Chromosome1-8039448 DEL	PRECISE	7484	0	0	0/0	0/0	0/0	0/1	0/0	0/0	0/1	0/1	0/0	0/0	0/0	0/0	0/0	0/1
Chromosome1-8047965 DEL	PRECISE	372	0	0	0/0	0/0	0/0	0/1	0/0	0/0	0/1	0/1	0/0	0/0	0/0	0/0	0/0	0/0
Chromosome1-8050833 DEL	PRECISE	625	0	0	0/0	0/0	0/0	0/1	0/0	0/0	0/1	0/1	0/1	0/0	0/1	0/1	0/1	0/1
Chromosome1-8061663 INS	PRECISE	137	0	0	0/0	0/1	0/1	0/1	0/1	0/1	0/0	0/0	0/0	0/1	0/0	0/0	0/0	0/1
Chromosome1-8062484 DEL	PRECISE	132	0	0	0/0	0/1	0/1	1/1	0/1	0/1	0/1	0/1	0/1	0/1	0/1	0/1	0/1	1/1
Chromosome1-8067182 INS	PRECISE	5463	0	0	0/0	0/0	0/0	0/0	0/0	0/0	0/0	0/0	0/0	0/0	0/0	0/0	0/0	1/1
Chromosome1-8069067 DEL	PRECISE	554	0	0	0/0	0/0	0/0	0/0	0/0	0/0	0/0	0/0	0/1	0/0	0/1	0/0	0/0	0/0
Chromosome1-8070412 DEL	PRECISE	176	0	0	0/0	0/0	0/0	0/0	0/0	0/0	0/0	0/0	0/0	0/0	0/0	0/0	0/0	0/1
Chromosome1-8073535 DEL	PRECISE	912	0	0	0/0	0/1	0/1	0/0	0/1	0/1	0/0	0/0	0/0	0/0	0/0	0/0	0/0	0/1
Chromosome1-8087149 DEL	PRECISE	411	0	0	0/0	0/0	0/0	0/0	0/0	0/0	0/0	0/1	0/0	0/0	0/0	0/0	0/0	0/0
Chromosome1-8100753 DEL	PRECISE	425	0	0	0/0	0/0	0/0	0/0	0/0	0/0	0/0	0/0	0/0	0/1	0/0	0/0	0/0	0/1
Chromosome1-8104946 INS	PRECISE	421	0	0.816	0/0	0/0	0/0	0/0	0/0	0/0	0/0	0/0	0/1	0/0	0/1	0/1	0/1	0/0
Chromosome1-8116711 DEL	PRECISE	858	0	0	0/0	0/0	0/0	0/0	0/0	0/0	0/0	0/0	0/1	0/0	0/1	0/1	0/1	0/0
Chromosome1-8118139 DEL	PRECISE	606	0	0	0/0	0/0	0/0	0/0	0/0	0/0	0/0	0/0	0/1	0/0	0/1	0/1	0/1	0/0
Chromosome1-8122519 INS	PRECISE	118	0	0	0/0	0/0	0/0	0/1	0/0	0/0	0/1	0/1	0/0	0/0	0/0	0/0	0/0	0/1
Chromosome1-8127413 BND	PRECISE	0	0	0	0/0	0/0	0/0	0/0	0/0	0/0	0/0	0/0	0/0	0/0	0/1	0/1	0/1	0/0
Chromosome1-8127422 BND	PRECISE	0	0.577	0	0/0	0/0	0/0	0/0	0/0	0/0	0/0	0/0	0/1	0/0	0/1	0/1	0/1	0/0
Chromosome1-8145544 INS	PRECISE	65	0	0	0/0	0/0	0/0	0/0	0/0	0/0	0/0	0/0	0/1	0/0	0/1	0/1	0/1	0/0
Chromosome1-8146677 DEL	PRECISE	355	0	0	0/0	0/0	0/0	0/0	0/0	0/0	0/0	0/0	0/1	0/0	0/1	0/1	0/1	0/0
Chromosome1-8147704 DEL	PRECISE	137	0	0	0/0	0/1	0/1	0/1	0/1	0/1	0/1	0/1	0/1	0/1	0/1	0/1	0/1	1/1
Chromosome1-8148454 DEL	PRECISE	934	0	0	0/0	0/0	0/0	0/0	0/0	0/0	0/0	0/0	0/1	0/0	0/1	0/1	0/1	0/0
Chromosome1-8159169 INS	PRECISE	90	0	0	0/0	0/0	0/0	0/0	0/0	0/0	0/0	0/0	0/0	0/0	0/0	0/0	0/1	0/0
Chromosome1-8183291 INS	PRECISE	1167	0	0	0/0	0/0	0/0	0/0	0/0	0/0	0/0	0/0	0/0	0/0	0/0	0/0	0/0	0/1
Chromosome1-8191270 DEL	PRECISE	55	0	0	0/0	0/0	0/0	0/0	0/0	0/0	0/0	0/0	0/0	0/0	0/0	0/0	0/0	0/1
Chromosome1-8192299 INS	PRECISE	8853	0	0	0/0	0/0	0/0	0/0	0/0	0/0	0/0	0/0	0/0	0/0	0/0	0/0	0/0	1/1
Chromosome1-8197526 INS	PRECISE	136	0	0	0/0	0/0	0/0	0/0	0/0	0/0	0/0	0/0	0/0	0/0	0/1	0/0	0/0	0/0
Chromosome1-8198679 INS	PRECISE	74	0	0.577	0/0	0/0	0/0	0/0	0/0	0/0	0/0	0/0	0/1	0/0	0/1	0/1	0/1	0/0
Chromosome1-8200419 INS	PRECISE	886	0	0	0/0	0/0	0/0	0/0	0/0	0/0	0/0	0/0	0/0	0/0	0/1	0/0	0/0	0/0
Chromosome1-8201345 INS	PRECISE	359	0	0.5	0/0	0/0	0/0	0/0	0/0	0/0	0/0	0/0	0/1	0/0	0/1	0/1	0/1	0/0
Chromosome1-8202551 INS	PRECISE	234	0	0.707	0/0	0/0	0/0	0/0	0/0	0/0	0/0	0/0	0/1	0/0	0/0	0/1	0/0	0/0
Chromosome1-8224057 DEL	PRECISE	785	0	0	0/0	0/1	0/1	0/0	0/1	0/1	0/0	0/0	0/0	0/1	0/0	0/0	0/0	1/1
Chromosome1-8234981 INS	PRECISE	926	0	0.753	0/0	0/1	0/1	0/0	0/1	0/1	0/0	0/0	0/0	0/1	0/0	0/0	0/0	0/1
Chromosome1-8239118 DEL	PRECISE	83	0.316	0.316	0/0	0/1	0/1	0/1	0/1	0/1	0/0	0/1	0/0	0/1	0/0	0/0	0/0	0/0
Chromosome1-8242412 BND	PRECISE	0	0	0	0/0	0/0	0/0	0/0	0/0	0/0	0/1	0/0	0/0	0/0	0/0	0/0	0/0	0/0
Chromosome1-8242412 BND	PRECISE	0	0	0	0/0	0/0	0/0	0/0	0/0	0/0	0/0	0/1	0/0	0/0	0/0	0/0	0/0	0/0
Chromosome1-8245932 INS	PRECISE	58	0	0	0/0	0/0	0/0	0/0	0/0	0/0	0/0	0/0	0/1	0/0	0/1	0/1	0/0	0/0
Chromosome1-8246245 DEL	PRECISE	130	0	0	0/0	0/0	0/0	0/1	0/0	0/0	0/1	0/1	0/0	0/0	0/0	0/0	0/0	0/0
Chromosome1-8246324 INS	PRECISE	9659	0	0	0/0	0/0	0/0	0/0	0/0	0/0	0/0	0/0	0/0	0/0	0/0	0/0	0/0	0/1
Chromosome1-8250357 INS	PRECISE	1416	0	0.707	0/0	0/0	0/0	0/0	0/0	0/0	0/0	0/0	0/1	0/0	0/0	0/1	0/0	0/0
Chromosome1-8256829 INS	PRECISE	54	0	0	0/0	0/0	0/0	0/0	0/0	0/0	0/0	0/0	0/0	0/0	0/0	0/0	0/0	0/1
Chromosome1-8267056 DEL	PRECISE	893	0	0	0/0	0/0	0/0	0/0	0/0	0/0	0/0	0/0	0/0	0/0	0/0	0/0	0/0	0/1
Chromosome1-8284784 INS	PRECISE	324	0	0	0/0	0/0	0/0	0/0	0/0	0/0	0/0	0/0	0/0	0/0	0/0	0/0	0/0	0/1
Chromosome1-8286942 DEL	PRECISE	988	0	0	0/0	0/0	0/0	0/0	0/0	0/0	0/0	0/0	0/0	0/0	0/1	0/0	0/0	0/0
Chromosome1-8294406 DEL	PRECISE	199	0	0	0/0	0/0	0/0	0/0	0/0	0/0	0/0	0/0	0/1	0/0	0/1	0/1	0/1	0/0
Chromosome1-8300762 INS	PRECISE	66	0	0	0/0	0/0	0/0	0/0	0/0	0/0	0/0	0/0	1/1	0/0	0/1	0/1	0/1	0/0
Chromosome1-8307618 DEL	PRECISE	161	0	0	0/0	0/0	0/0	0/0	0/0	0/0	0/0	0/0	0/0	0/0	0/0	0/0	0/1	0/0
Chromosome1-8309369 DEL	PRECISE	151	0	0	0/0	0/0	0/0	0/0	0/0	0/0	0/0	0/0	0/1	0/0	0/1	0/1	0/1	0/0
Chromosome1-8317054 INS	PRECISE	118	0	0	0/0	0/1	0/1	0/0	0/1	0/1	0/0	0/0	0/0	0/1	0/0	0/0	0/0	0/1
Chromosome1-8317555 DEL	PRECISE	480	0	0	0/0	0/0	0/0	0/0	0/0	0/0	0/0	0/0	0/1	0/0	0/0	0/1	0/1	0/0

Chromosome1-8318405	DEL	PRECISE	1061	0	0	0/0	0/0	0/0	0/0	0/0	0/0	0/0	0/0	0/1	0/0	0/0	0/1	0/0	0/0
Chromosome1-8320299	INS	PRECISE	10641	0	0	0/0	0/0	0/0	0/0	0/0	0/0	0/0	0/0	0/0	0/0	0/0	0/0	0/1	0/0
Chromosome1-8324896	INS	PRECISE	285	0	0.408	0/0	0/1	0/1	0/0	0/1	0/1	0/0	0/0	0/0	0/1	0/0	0/0	0/0	0/1
Chromosome1-8328442	DEL	PRECISE	121	0	0	0/0	0/0	0/0	0/0	0/0	0/0	0/0	0/0	0/0	0/0	0/0	0/0	0/0	0/1
Chromosome1-8329794	INS	PRECISE	68	0	0	0/0	0/0	0/0	0/0	0/0	0/0	0/0	0/0	0/0	0/0	0/0	0/0	0/0	0/1
Chromosome1-8331806	INS	PRECISE	3153	0	0	0/0	0/0	0/0	0/0	0/0	0/0	0/0	0/0	0/0	0/0	0/0	0/0	0/0	1/1
Chromosome1-8335826	DEL	PRECISE	87	0	0	0/0	0/1	0/1	0/0	0/1	0/1	0/0	0/0	0/0	0/1	0/0	0/0	0/0	0/1
Chromosome1-8339013	DUP	PRECISE	2740200	0	0	0/0	0/0	0/0	0/0	0/0	0/0	0/0	0/0	0/1	0/0	0/1	0/1	0/1	0/0
Chromosome1-8347924	DEL	PRECISE	335	0	0	0/0	0/0	0/0	0/0	0/0	0/0	0/0	0/0	0/0	0/0	0/1	0/0	0/0	0/0
Chromosome1-8348926	INS	PRECISE	51	0	0.5	0/0	0/0	0/0	0/0	0/0	0/0	0/0	0/0	0/1	0/0	0/1	0/1	0/1	0/0
Chromosome1-8358825	INS	PRECISE	1438	0	0.816	0/0	0/0	0/0	0/0	0/0	0/0	0/0	0/0	0/1	0/0	0/1	0/1	0/1	0/0
Chromosome1-8365170	DEL	PRECISE	150	0	0	0/0	0/0	0/0	0/0	0/0	0/0	0/0	0/0	0/1	0/0	0/1	0/1	0/1	0/0
Chromosome1-8370053	INS	PRECISE	909	0	0	0/0	0/0	0/0	0/0	0/0	0/0	0/0	0/0	0/0	0/0	0/0	0/0	0/0	0/1
Chromosome1-8372420	DEL	PRECISE	673	0	0	0/0	0/0	0/0	0/0	0/0	0/0	0/0	0/0	0/0	0/0	0/0	0/0	0/0	0/1
Chromosome1-8375168	DEL	PRECISE	657	0	0	0/0	0/0	0/0	0/0	0/0	0/0	0/0	0/0	0/1	0/0	0/1	0/1	0/1	0/0
Chromosome1-8377260	INS	PRECISE	158	0	0	0/0	0/0	0/0	0/0	0/0	0/0	0/0	0/0	0/0	0/0	0/0	0/0	0/0	0/1
Chromosome1-8382581	INS	PRECISE	1353	0	0	0/0	0/0	0/0	0/0	0/0	0/0	0/0	0/0	0/0	0/0	0/1	0/0	0/0	0/0
Chromosome1-8383681	BND	PRECISE	0	0	0	0/0	0/0	0/0	0/0	0/0	0/0	0/0	0/0	0/0	0/0	0/0	0/0	0/1	0/0
Chromosome1-8383883	BND	PRECISE	0	0	0	0/0	0/0	0/0	0/0	0/0	0/0	0/0	0/0	0/1	0/0	0/0	0/0	0/0	0/0
Chromosome1-8386854	DEL	PRECISE	78	0	0	0/0	0/1	0/1	0/0	0/1	0/1	0/0	0/0	0/0	0/1	0/0	0/0	0/0	0/1
Chromosome1-8386940	INS	PRECISE	55	0	0	0/0	0/0	0/0	0/0	0/0	0/0	0/0	0/0	0/0	0/0	0/1	0/0	0/0	0/0
Chromosome1-8390686	DEL	PRECISE	698	0	0	0/0	0/1	0/1	0/1	0/1	0/1	0/1	0/1	0/1	0/1	0/0	0/1	0/1	1/1
Chromosome1-8397491	INS	PRECISE	76	0	0	0/0	0/0	0/0	0/0	0/0	0/0	0/0	0/0	0/1	0/0	0/1	0/1	0/1	0/0
Chromosome1-8402105	INS	PRECISE	71	0	0	0/0	0/0	0/0	0/0	0/0	0/0	0/0	0/0	0/0	0/0	0/1	0/0	0/0	0/0
Chromosome1-8417816	INS	PRECISE	470	0	0.527	0/0	0/1	0/1	0/1	0/1	0/1	0/1	0/1	0/0	0/1	0/0	0/0	0/0	0/1
Chromosome1-8444834	INS	PRECISE	128	0	0	0/0	0/1	0/1	0/0	0/1	0/1	0/0	0/0	0/0	0/1	0/0	0/0	0/0	0/1
Chromosome1-8445547	DEL	PRECISE	1014	0	0	0/0	0/1	0/1	0/1	0/1	0/1	0/1	0/1	0/0	0/1	0/0	0/0	0/0	1/1
Chromosome1-8448448	INS	PRECISE	122	0	0.354	0/0	0/1	0/1	0/0	0/1	0/1	0/0	0/0	0/0	0/1	0/0	0/0	0/0	1/1
Chromosome1-8460084	INS	PRECISE	50	0	0	0/0	0/0	0/0	0/0	0/0	0/0	0/0	0/0	0/0	0/0	0/1	0/1	0/1	0/0
Chromosome1-8462537	DEL	PRECISE	77	0	0	0/0	0/0	0/0	0/0	0/0	0/0	0/0	0/0	0/0	0/0	0/1	0/0	0/0	0/0
Chromosome1-8467084	BND	PRECISE	0	0	0	0/0	0/0	0/0	0/0	0/0	0/0	0/0	0/0	0/0	0/0	0/1	0/1	1/1	0/0
Chromosome1-8471046	INS	PRECISE	65	0.5	0.5	0/0	0/0	0/0	0/0	0/0	0/0	0/0	0/0	0/1	0/0	0/1	0/1	0/1	0/0
Chromosome1-8474766	DEL	PRECISE	71	0	0	0/0	0/0	0/0	0/0	0/0	0/0	0/0	0/0	0/1	0/0	0/0	0/1	0/0	0/0
Chromosome1-8483991	DEL	PRECISE	655	0	0	0/0	0/1	0/1	0/1	0/1	0/1	0/1	0/1	0/0	0/1	0/0	0/0	0/0	1/1
Chromosome1-8486346	DEL	PRECISE	61	0	0	0/0	0/0	0/0	0/1	0/0	0/0	0/1	0/1	0/0	0/0	0/0	0/0	0/0	0/0
Chromosome1-8489891	DEL	PRECISE	50	0	0	0/0	0/0	0/0	0/0	0/0	0/0	0/0	0/0	0/0	0/0	0/1	0/0	0/0	0/0
Chromosome1-8490000	INS	PRECISE	94	0	0	0/0	0/0	0/0	0/0	0/0	0/0	0/0	0/0	0/1	0/0	0/0	0/1	0/0	0/0
Chromosome1-8503988	INS	PRECISE	149	0	0	0/0	0/0	0/0	0/0	0/0	0/0	0/0	0/0	0/0	0/0	0/0	0/0	0/0	0/1
Chromosome1-8506827	INS	PRECISE	1024	0	0	0/0	0/0	0/0	0/0	0/0	0/0	0/0	0/0	0/0	0/0	0/0	0/0	0/0	0/1
Chromosome1-8507545	DEL	PRECISE	66	0.577	0.577	0/0	0/0	0/0	0/0	0/0	0/0	0/0	0/0	0/1	0/0	0/0	0/1	0/0	0/0
Chromosome1-8508995	INS	PRECISE	1003	0	0	0/0	0/0	0/0	0/1	0/0	0/0	0/0	0/0	0/0	0/0	0/0	0/0	0/0	0/0
Chromosome1-8511553	INS	PRECISE	57	0	0	0/0	0/0	0/0	1/1	0/0	0/0	0/1	0/1	0/0	0/0	0/0	0/0	0/0	0/0
Chromosome1-8520079	BND	PRECISE	0	0	0	0/0	0/0	0/0	0/0	0/0	0/0	0/0	0/0	0/0	0/0	0/0	0/1	0/0	0/0
Chromosome1-8525714	INS	PRECISE	3229	0	0.577	0/0	0/0	0/0	1/1	0/0	0/0	1/1	1/1	0/0	0/0	0/0	0/0	0/0	0/0
Chromosome1-8545303	DEL	PRECISE	69	0	0	0/0	0/0	0/0	0/1	0/0	0/0	0/1	0/1	0/0	0/0	0/0	0/0	0/0	0/0
Chromosome1-8546993	DEL	PRECISE	72	0	0	0/0	0/0	0/0	0/0	0/0	0/0	0/0	0/0	0/1	0/0	0/1	0/1	0/1	0/1
Chromosome1-8551729	INS	PRECISE	12518	0	0	0/0	0/0	0/0	0/0	0/0	0/0	0/0	1/1	0/0	0/0	0/0	0/0	0/0	0/0
Chromosome1-8554496	DEL	PRECISE	5096	0	0	0/0	0/0	0/0	0/0	0/0	0/0	0/0	0/0	0/0	0/0	0/0	0/1	0/0	0/0
Chromosome1-8557907	INS	PRECISE	12620	0	0	0/0	0/0	0/0	0/0	0/0	0/0	0/0	0/1	0/0	0/0	0/0	0/0	0/0	0/0
Chromosome1-8560352	DEL	PRECISE	419	0	0	0/0	0/0	0/0	0/0	0/0	0/0	0/0	0/0	0/0	0/0	0/0	0/0	0/0	0/1
Chromosome1-8563647	DEL	PRECISE	56	0	0	0/0	0/0	0/0	0/0	0/0	0/0	0/0	0/0	0/0	0/0	0/0	0/0	0/0	0/1
Chromosome1-8572311	INS	PRECISE	1793	0	0	0/0	0/0	0/0	0/0	0/0	0/0	0/0	0/0	0/0	0/0	0/0	0/0	0/0	0/1
Chromosome1-8573465	INS	PRECISE	1134	0	0.753	0/0	0/1	0/1	0/0	0/1	0/1	0/0	0/0	0/0	0/1	0/0	0/0	0/0	1/1
Chromosome1-8576641	INS	PRECISE	1079	0	0	0/0	0/0	0/0	0/1	0/0	0/0	0/0	0/0	0/0	0/0	0/0	0/0	0/0	0/0
Chromosome1-8577735	DEL	PRECISE	467	0	0	0/0	0/1	0/1	0/0	0/1	0/1	0/0	0/0	0/0	0/1	0/0	0/0	0/0	0/1
Chromosome1-8589689	INS	PRECISE	51	0	0	0/0	0/0	0/0	0/0	0/0	0/0	0/0	0/0	0/0	0/0	0/1	0/0	0/0	0/0
Chromosome1-8595487	INS	PRECISE	173	0	0	0/0	0/0	0/0	0/0	0/0	0/0	0/0	0/0	0/0	0/0	0/0	0/0	0/0	0/1
Chromosome1-8608118	INS	PRECISE	425	0	0.577	0/0	0/0	0/0	0/0	0/0	0/0	0/0	0/0	0/1	0/0	0/1	0/1	1/1	0/0
Chromosome1-8612022	INS	PRECISE	330	0	0	0/0	0/0	0/0	0/0	0/0	0/0	0/0	0/0	0/0	0/0	0/1	0/0	0/1	0/0
Chromosome1-8613719	DEL	PRECISE	70	0	0	0/0	0/0	0/0	0/0	0/0	0/0	0/0	0/0	0/0	0/0	0/0	0/0	0/0	0/1
Chromosome1-8619642	DEL	PRECISE	2852	0	0	0/0	0/1	0/1	0/0	0/1	0/1	0/0	0/0	0/0	0/1	0/0	0/0	0/0	0/1
Chromosome1-8630039	INS	PRECISE	152	0	0	0/0	0/0	0/0	0/0	0/0	0/0	0/0	0/0	0/0	0/0	0/0	0/0	0/0	0/1
Chromosome1-8643878	INS	PRECISE	12515	0	0	0/0	0/0	0/0	1/1	0/0	0/0	0/0	0/0	0/0	0/0	0/0	0/0	0/0	0/0
Chromosome1-8644005	DEL	PRECISE	1274	0	0	0/0	0/0	0/0	0/0	0/0	0/0	0/0	0/0	0/1	0/0	0/1	0/1	0/1	0/0
Chromosome1-8648928	INS	PRECISE	54	0	0	0/0	0/0	0/0	0/0	0/0	0/0	0/0	0/0	0/0	0/0	0/1	0/0	0/1	0/0
Chromosome1-8650899	INS	PRECISE	133	0	0.707	0/0	0/0	0/0	0/0	0/0	0/0	0/0	0/0	0/0	0/0	0/1	0/0	0/1	0/0
Chromosome1-8651662	DEL	PRECISE	154	0	0	0/0	0/0	0/0	0/0	0/0	0/0	0/0	0/0	0/1	0/0	0/1	0/1	0/1	0/0
Chromosome1-8653014	DEL	PRECISE	71	0	0	0/0	0/0	0/0	0/0	0/0	0/0	0/0	0/0	0/1	0/0	0/1	0/1	0/1	0/0
Chromosome1-8661355	INS	PRECISE	54	0	0	0/0	0/0	0/0	0/0	0/0	0/0	0/0	0/0	0/0	0/0	0/1	0/0	0/1	0/0
Chromosome1-8672009	INS	PRECISE	56	0	0	0/0	0/0	0/0	0/1	0/0	0/0	0/0	0/0	0/0	0/0	0/0	0/0	0/0	0/0
Chromosome1-8678294	DEL	PRECISE	646	0	0	0/0	0/0	0/0	0/0	0/0	0/0	0/0	0/0	1/1	0/0	0/0	0/1	0/0	0/0
Chromosome1-8684794	INS	PRECISE	1848	0	0	0/0	0/0	0/0	0/1	0/0	0/0	0/0	0/0	0/0	0/0	0/0	0/0	0/0	0/0
Chromosome1-8699638	INS	PRECISE	74	0	0	0/0	0/0	0/0	0/0	0/0	0/0	0/0	0/0	0/0	0/0	0/1	0/0	0/1	0/0

Chromosome1-8702427	DEL	PRECISE	4910	0	0	0/0	0/1	0/1	0/0	0/1	0/1	0/0	0/0	0/1	0/1	0/1	0/1	0/1	0/1
Chromosome1-8721289	INS	PRECISE	61	0	0.5	0/0	0/0	0/0	0/0	0/1	0/1	0/0	0/0	1/1	0/0	0/0	0/1	0/0	0/0
Chromosome1-8726972	BND	PRECISE	0	0	0	0/0	0/0	0/0	0/0	0/0	0/0	1/1	1/1	0/0	0/0	0/0	0/0	0/0	1/1
Chromosome1-8727154	BND	PRECISE	0	0	0	0/0	0/0	0/0	0/0	0/0	0/0	0/0	0/0	1/1	0/0	0/0	0/0	0/0	0/0
Chromosome1-8727544	INS	PRECISE	1618	0	0	0/0	0/0	0/0	1/1	0/0	0/0	0/0	0/0	0/0	0/0	0/0	0/0	0/0	0/0
Chromosome1-8739841	DEL	PRECISE	418	0	0	0/0	0/0	0/0	1/1	0/0	0/0	0/0	0/0	0/0	0/0	0/0	0/0	0/0	0/0
Chromosome1-8761431	DEL	PRECISE	153	0	0	0/0	0/0	0/0	0/0	0/1	0/1	0/0	0/0	1/1	0/0	0/0	0/0	0/0	0/0
Chromosome1-8781781	DEL	PRECISE	55	0	0	0/0	0/0	0/0	1/1	0/0	0/0	0/0	0/0	0/0	0/0	0/0	0/0	0/0	0/0
Chromosome1-8781886	DEL	PRECISE	2803	0	0	0/0	0/0	0/0	1/1	0/0	0/0	0/0	0/0	0/0	0/0	0/0	0/0	0/0	0/0
Chromosome1-8788750	INS	PRECISE	2110	0	0.707	0/0	0/0	0/0	0/0	0/0	0/0	0/0	0/0	0/1	0/0	0/0	0/1	0/0	0/0
Chromosome1-8792918	INS	PRECISE	306	0	0.577	0/0	0/0	0/0	0/0	0/0	0/0	0/1	0/1	0/0	0/0	0/0	0/0	0/0	0/1
Chromosome1-8793064	DEL	PRECISE	305	0	0	0/0	0/0	0/0	0/0	0/0	0/0	0/0	0/0	0/0	0/0	0/1	0/0	0/1	0/0
Chromosome1-8796716	DEL	PRECISE	2267	0	0	0/0	0/0	0/0	0/1	0/0	0/0	0/0	0/0	0/0	0/0	0/0	0/0	0/0	0/0
Chromosome1-8798372	INS	PRECISE	215	0	0.408	0/0	0/1	0/1	0/0	0/1	0/1	0/0	0/0	0/0	0/1	0/0	0/0	0/0	0/1
Chromosome1-8800722	DEL	PRECISE	101	0	0	0/0	0/0	0/0	0/0	0/0	0/0	0/0	0/0	0/0	0/0	0/1	0/0	0/1	0/0
Chromosome1-8801766	DEL	PRECISE	6328	0	0	0/0	0/0	0/0	0/0	0/0	0/0	0/0	0/0	0/0	0/0	0/1	0/0	0/1	0/0
Chromosome1-8806392	INS	PRECISE	105	0	0	0/0	0/1	0/0	0/1	0/1	0/1	0/1	0/1	0/0	0/1	0/0	0/0	0/0	1/1
Chromosome1-8809383	DEL	PRECISE	51	0	0	0/0	0/1	0/1	0/0	0/1	0/1	0/0	0/0	0/0	0/1	0/0	0/0	0/0	0/1
Chromosome1-8809960	DEL	PRECISE	1723	0	0	0/0	0/0	0/0	0/0	0/0	0/0	0/0	0/0	0/0	0/0	1/1	0/0	1/1	0/0
Chromosome1-8810399	DEL	PRECISE	398	0	0	0/0	0/0	0/0	0/0	0/0	0/0	0/1	0/1	0/1	0/0	0/0	0/1	0/0	0/0
Chromosome1-8811713	INS	PRECISE	625	0	0	0/0	0/0	0/0	0/1	0/0	0/0	0/0	0/0	0/0	0/0	0/0	0/0	0/0	0/0
Chromosome1-8815505	DEL	PRECISE	342	0	0	0/0	0/0	0/0	0/0	0/0	0/0	0/0	0/0	0/1	0/0	0/0	0/1	0/0	1/1
Chromosome1-8818972	DEL	PRECISE	176	0	0	0/0	0/0	0/0	0/0	0/0	0/0	0/0	0/0	0/1	0/0	0/0	0/1	0/0	0/0
Chromosome1-8819546	INS	PRECISE	83	0	0.707	0/0	0/0	0/0	0/0	0/0	0/0	0/0	0/0	0/1	0/0	0/0	0/1	0/0	0/0
Chromosome1-8820674	INS	PRECISE	282	0	0	0/0	0/0	0/0	0/0	0/0	0/0	0/0	0/0	0/1	0/0	0/0	0/1	0/0	0/0
Chromosome1-8821771	INS	PRECISE	919	0	0.707	0/0	0/0	0/0	0/0	0/0	0/0	0/0	0/0	0/0	0/0	0/1	0/0	0/1	0/0
Chromosome1-8825321	INS	PRECISE	311	0	0.816	0/0	0/1	0/1	0/0	0/1	0/1	0/0	0/0	0/0	0/1	0/0	0/0	0/0	0/1
Chromosome1-8830022	INS	PRECISE	50	0	0	0/0	0/0	0/0	0/1	0/0	0/0	0/0	0/0	0/0	0/0	0/0	0/0	0/0	0/0
Chromosome1-8833318	DEL	PRECISE	1038	0	0	0/0	0/0	0/0	0/0	0/0	0/0	0/0	0/0	0/1	0/0	0/1	0/1	0/1	0/0
Chromosome1-8847804	INS	PRECISE	6424	0	0	0/0	0/0	0/0	0/0	0/1	0/0	0/0	0/0	0/0	0/0	0/0	0/0	0/0	0/0
Chromosome1-8848670	INS	PRECISE	878	0	0.983	0/0	0/1	0/1	0/0	0/0	0/1	0/0	0/0	0/0	0/1	0/0	0/0	0/0	0/1
Chromosome1-8858328	BND	PRECISE	0	0	0	0/0	0/0	0/0	0/0	0/0	0/0	0/0	0/0	0/1	0/0	0/0	0/0	0/1	0/0
Chromosome1-8860437	INS	PRECISE	4818	0	0	0/0	0/0	0/0	1/1	0/0	0/0	0/0	0/0	0/0	0/0	0/0	0/0	0/0	0/0
Chromosome1-8860639	DEL	PRECISE	5649	0	0	0/0	0/0	0/0	0/0	0/0	0/0	0/0	0/0	0/1	0/0	0/1	0/0	1/1	0/0
Chromosome1-8864282	DEL	PRECISE	410	0	0	0/0	0/1	0/1	0/0	0/1	0/1	0/0	0/0	0/0	0/1	0/0	0/0	0/0	0/1
Chromosome1-8874046	INS	PRECISE	663	0	0	0/0	0/0	0/0	0/0	0/0	0/0	0/1	0/0	0/0	0/0	0/0	0/0	0/0	0/0
Chromosome1-8874089	DEL	PRECISE	664	0	0	0/0	0/1	0/1	0/0	0/1	0/1	0/0	0/0	0/0	1/1	0/1	0/0	0/1	0/1
Chromosome1-8874623	INS	PRECISE	51	0	0	0/0	0/0	0/0	0/0	0/0	0/0	0/1	0/0	0/0	0/0	0/0	0/0	0/0	0/0
Chromosome1-8875530	DEL	PRECISE	489	0	0	0/0	0/0	0/0	0/1	0/0	0/0	0/0	0/0	0/0	0/0	0/1	0/0	0/1	0/1
Chromosome1-8878536	DEL	PRECISE	67	0	0	0/0	0/0	0/0	0/0	0/0	0/0	0/0	0/0	0/0	0/0	0/1	0/0	0/1	0/0
Chromosome1-8879517	DEL	PRECISE	150	0	0	0/0	0/0	0/0	0/1	0/0	0/0	0/0	0/0	0/0	0/0	0/0	0/0	0/0	0/0
Chromosome1-8885835	INS	PRECISE	14340	0	0	0/0	0/0	0/0	0/0	0/0	0/0	0/0	0/0	0/0	0/0	0/0	0/1	0/0	0/0
Chromosome1-8887745	DEL	PRECISE	382	0	0	0/0	0/0	0/0	0/0	0/0	0/0	0/0	0/0	0/0	0/0	0/0	0/1	0/0	0/0
Chromosome1-8889121	DEL	PRECISE	711	0.555	0.277	0/0	0/1	0/1	1/1	1/1	1/1	0/1	0/1	1/1	0/1	0/1	0/1	0/1	1/1
Chromosome1-8890896	DEL	PRECISE	104	0	0	0/0	0/0	0/0	0/0	0/0	0/0	0/1	0/1	0/0	0/0	0/0	0/0	0/0	0/0
Chromosome1-8892099	INS	PRECISE	164	0	0.707	0/0	0/0	0/0	0/0	0/0	0/0	0/1	0/1	0/0	0/0	0/0	0/0	0/0	0/0
Chromosome1-8895403	INS	PRECISE	50	0	0	0/0	0/0	0/0	0/0	0/0	0/0	0/0	0/0	0/0	0/0	0/0	0/0	0/0	0/1
Chromosome1-8898166	INS	PRECISE	1424	0	0.707	0/0	0/0	0/0	0/0	0/0	0/0	0/0	0/0	0/1	0/0	0/0	0/1	0/0	0/0
Chromosome1-8898547	INS	PRECISE	241	0	0.707	0/0	0/0	0/0	0/0	0/0	0/0	0/0	0/0	0/0	0/0	0/1	0/0	0/1	0/0
Chromosome1-8910770	DEL	PRECISE	66	0	0	0/0	0/0	0/0	0/0	0/0	0/0	0/0	0/0	0/0	0/0	0/1	0/0	0/1	0/0
Chromosome1-8916753	INS	PRECISE	218	0	0	0/0	0/0	0/0	0/1	0/0	0/0	0/0	0/0	0/0	0/0	0/0	0/0	0/0	0/0
Chromosome1-8918311	INS	PRECISE	4090	0	0	0/0	0/0	0/0	0/0	0/0	0/0	0/0	0/0	1/1	0/0	0/0	0/0	0/0	0/0
Chromosome1-8918639	INS	PRECISE	6051	0	0	0/0	0/0	0/0	0/0	0/0	0/0	0/0	0/0	1/1	0/0	0/0	0/0	0/0	0/0
Chromosome1-8920471	INS	PRECISE	655	0	0.463	0/0	0/1	0/1	0/1	0/1	0/0	0/1	0/0	0/0	0/1	0/0	0/0	0/0	0/0
Chromosome1-8925602	INS	PRECISE	527	0	0	0/0	0/0	0/0	0/0	0/0	0/0	0/0	0/0	0/0	0/0	0/0	0/0	0/0	1/1
Chromosome1-8928967	INS	PRECISE	1325	0	0	0/0	0/0	0/0	0/0	0/0	0/0	0/0	0/0	0/0	0/0	0/0	0/0	0/0	1/1
Chromosome1-8932841	DEL	PRECISE	579	0	0	0/0	0/0	0/0	0/0	0/0	0/0	0/0	0/0	0/1	0/0	0/0	0/1	0/1	0/0
Chromosome1-8935394	DEL	PRECISE	567	0	0	0/0	0/0	0/0	0/0	0/0	0/0	0/0	0/0	0/1	0/0	0/0	0/1	0/1	0/0
Chromosome1-8950638	DEL	PRECISE	128	0	0	0/0	0/0	0/0	0/0	0/0	0/0	0/0	0/0	0/1	0/0	0/1	0/1	0/1	0/0
Chromosome1-8950812	INS	PRECISE	111	0	0.957	0/0	0/0	0/0	0/0	0/0	0/0	0/0	0/0	0/1	0/0	0/1	0/1	0/1	0/0
Chromosome1-8951697	DEL	PRECISE	137	0	0	0/0	0/0	0/0	0/0	0/0	0/0	0/0	0/0	0/1	0/0	0/1	0/1	0/1	0/0
Chromosome1-8959538	DEL	PRECISE	200	0	0	0/0	0/0	0/0	0/0	0/0	0/0	0/0	0/0	0/1	0/0	0/1	0/1	0/1	0/0
Chromosome1-8959756	DEL	PRECISE	1378	0	0	0/0	0/0	0/0	0/0	0/0	0/0	0/0	0/0	1/1	0/0	0/1	0/1	0/1	0/0
Chromosome1-8959831	BND	PRECISE	0	0	0	0/0	0/0	0/0	0/0	0/1	1/1	0/0	0/0	1/1	0/0	0/0	0/0	0/0	0/0
Chromosome1-8975534	DEL	PRECISE	1206	0	0	0/0	0/0	0/0	0/0	0/0	0/0	0/0	0/0	0/0	0/0	0/0	0/0	0/0	0/1
Chromosome1-8976996	DEL	PRECISE	3283	0	0	0/0	0/0	0/0	0/0	0/0	0/0	0/0	0/0	0/0	0/0	0/0	0/0	0/0	0/1
Chromosome1-8978742	INS	PRECISE	146	0	0	0/0	0/1	0/1	0/0	0/1	0/1	0/0	0/0	0/0	0/1	0/0	0/0	0/0	0/1
Chromosome1-8980383	DEL	PRECISE	301	0	0	0/0	0/0	0/0	0/0	0/0	0/0	0/0	0/0	0/0	0/0	0/0	0/0	0/0	0/1
Chromosome1-8980710	DEL	PRECISE	3752	0	0	0/0	0/0	0/0	0/0	0/0	0/0	0/0	0/0	0/0	0/0	0/0	0/0	0/0	0/1
Chromosome1-8984535	DEL	PRECISE	1042	0	0	0/0	0/0	0/0	0/0	0/0	0/0	0/0	0/0	0/0	0/0	0/0	0/0	0/0	0/1
Chromosome1-8985631	DEL	PRECISE	498	0	0	0/0	0/0	0/0	0/0	0/0	0/0	0/0	0/0	0/0	0/0	0/0	0/0	0/0	0/1
Chromosome1-8986189	DEL	PRECISE	2711	0	0	0/0	0/0	0/0	0/0	0/0	0/0	0/0	0/0	0/0	0/0	0/0	0/0	0/0	1/1
Chromosome1-8987874	DEL	PRECISE	1865	0	0	0/0	0/0	0/0	1/1	0/0	0/0	0/1	0/1	0/0	0/0	0/0	0/0	0/0	0/0

Chromosome1-8994312 DEL	PRECISE	783	0	0	0/0	0/0	0/0	0/0	0/0	0/0	0/0	0/0	0/1	0/0	0/1	0/1	0/1	1/1
Chromosome1-9012170 INS	PRECISE	5716	0	0	0/0	0/0	0/0	0/0	0/0	0/0	0/0	0/0	0/0	0/0	0/0	1/1	0/0	0/0
Chromosome1-9021427 DEL	PRECISE	1158	0	0	0/0	0/0	0/0	0/0	0/0	0/0	0/0	0/0	0/1	0/0	0/1	0/1	0/1	0/0
Chromosome1-9024106 DEL	PRECISE	182	0	0	0/0	0/0	0/0	0/0	0/0	0/0	0/0	0/0	0/1	0/0	0/1	0/1	0/1	0/0
Chromosome1-9027130 DEL	PRECISE	1559	0	0	0/0	0/0	0/0	0/0	0/0	0/0	0/0	0/0	0/0	0/0	0/1	0/0	0/1	0/0
Chromosome1-9027408 DEL	PRECISE	6718	0	0	0/0	0/1	0/1	0/0	0/1	0/1	0/0	0/0	0/0	0/1	0/0	0/0	0/0	0/1
Chromosome1-9034750 INS	PRECISE	1304	0	0	0/0	0/0	0/0	0/1	0/0	0/0	0/0	0/0	0/0	0/0	0/0	0/0	0/0	0/0
Chromosome1-9037574 INS	PRECISE	141	0	0	0/0	0/1	0/1	0/0	0/1	0/1	0/0	0/0	0/0	1/1	0/0	0/0	0/0	0/1
Chromosome1-9041259 INS	PRECISE	60	0	0	0/0	0/0	0/0	0/1	0/0	0/0	0/0	0/0	0/0	0/0	0/0	0/0	0/0	0/0
Chromosome1-9042497 INS	PRECISE	201	0	0.816	0/0	0/0	0/0	0/1	0/1	0/1	0/0	0/0	0/1	0/0	0/0	0/0	0/0	0/0
Chromosome1-9042626 INS	PRECISE	5965	0	0	0/0	0/0	0/0	0/0	0/0	0/0	0/0	0/0	0/0	0/0	0/0	0/0	0/0	1/1
Chromosome1-9042643 BND	PRECISE	0	0	0	0/0	0/0	0/0	0/0	0/0	0/0	0/0	0/0	0/0	0/0	0/0	0/0	0/0	0/1
Chromosome1-9050383 DUP	PRECISE	5793314	0	0	0/0	0/0	0/0	0/0	0/0	0/0	0/0	0/0	0/0	0/0	0/0	0/0	0/0	0/1
Chromosome1-9051693 DEL	PRECISE	756	0	0	0/0	0/0	0/0	0/0	0/0	0/0	0/0	0/0	0/1	0/0	0/1	0/1	0/1	0/0
Chromosome1-9059514 DEL	PRECISE	54	0	0	0/0	0/0	0/0	0/1	0/0	0/0	0/1	0/1	0/0	0/0	0/0	0/0	0/0	0/0
Chromosome1-9062722 INS	PRECISE	12560	0	0	0/0	0/0	0/0	0/0	0/0	0/0	0/0	0/0	0/0	0/1	0/0	0/0	0/0	0/0
Chromosome1-9064469 INS	PRECISE	1371	0	0.707	0/0	0/0	0/0	0/0	0/0	0/0	0/1	0/1	0/0	0/0	0/0	0/0	0/0	0/0
Chromosome1-9064474 INS	PRECISE	10581	0	0	0/0	0/0	0/0	0/1	0/0	0/0	0/0	0/0	0/0	0/0	0/0	0/0	0/0	0/0
Chromosome1-9065169 DEL	PRECISE	75	0	0	0/0	0/0	0/0	0/0	0/0	0/0	0/0	0/0	0/0	0/0	0/0	0/0	0/0	0/1
Chromosome1-9066228 DEL	PRECISE	62	0	0	0/0	0/1	0/1	0/0	0/1	0/1	0/0	0/0	0/0	0/1	0/0	0/0	0/0	0/1
Chromosome1-9069855 DEL	PRECISE	233	0	0	0/0	0/1	0/1	0/1	0/1	0/1	0/1	0/1	0/0	0/1	0/0	0/0	0/0	0/1
Chromosome1-9070076 BND	PRECISE	0	0	0	0/0	0/0	0/0	0/0	0/0	0/0	0/0	0/0	0/0	0/0	0/1	0/0	0/1	0/0
Chromosome1-9072747 DEL	PRECISE	51	0	0	0/0	0/1	0/1	0/0	0/1	0/1	0/0	0/0	0/0	0/1	0/0	0/0	0/0	0/1
Chromosome1-9077513 DEL	PRECISE	211	0	0	0/0	0/1	0/1	1/1	1/1	1/1	0/1	0/1	1/1	0/1	0/1	0/1	0/1	1/1
Chromosome1-9083851 DEL	PRECISE	137	0	0	0/0	0/0	0/0	0/0	0/0	0/0	0/0	0/0	0/1	0/0	0/1	0/1	1/1	0/0
Chromosome1-9088124 INS	PRECISE	80	0	0	0/0	0/0	0/0	0/0	0/0	0/0	0/0	0/0	0/1	0/0	0/1	0/1	0/1	0/0
Chromosome1-9093593 DEL	PRECISE	50	0	0	0/0	0/0	0/0	0/0	0/0	0/0	0/0	0/0	0/1	0/0	0/1	0/1	0/1	0/0
Chromosome1-9114761 INS	PRECISE	343	0	0.577	0/0	0/0	0/0	0/1	0/0	0/0	0/1	0/1	0/0	0/0	0/0	0/0	0/0	0/0
Chromosome1-9153640 INS	PRECISE	128	0	0	0/0	0/0	0/0	0/0	0/0	0/0	0/0	0/0	0/1	0/0	0/0	0/1	0/0	0/0
Chromosome1-9154281 INS	PRECISE	627	0	0	0/0	0/0	0/0	0/0	0/0	0/0	0/0	0/0	0/0	0/0	0/0	0/0	0/0	0/1
Chromosome1-9154490 DEL	PRECISE	147	0	0	0/0	0/0	0/0	0/0	0/0	0/0	0/0	0/0	0/1	0/0	0/0	0/1	0/0	0/0
Chromosome1-9181515 DEL	PRECISE	69	0	0	0/0	0/0	0/0	0/0	0/0	0/0	0/0	0/0	0/1	0/0	0/0	0/0	0/0	0/0
Chromosome1-9183147 DEL	PRECISE	130	0	0	0/0	0/0	0/0	0/0	0/0	0/0	0/0	0/0	0/0	0/0	0/1	0/0	0/1	0/0
Chromosome1-9186948 INS	PRECISE	60	0	0	0/0	0/0	0/0	0/0	0/0	0/0	0/0	0/0	0/0	0/0	0/1	0/0	0/1	0/0
Chromosome1-9186949 DEL	PRECISE	65	0	0	0/0	0/0	0/0	0/0	0/0	0/0	0/0	0/0	0/1	0/0	0/0	0/1	0/0	0/0
Chromosome1-9188506 DEL	PRECISE	57	0	0	0/0	0/0	0/0	0/1	0/0	0/0	0/0	0/0	0/0	0/0	0/0	0/0	0/0	0/0
Chromosome1-9188978 INS	PRECISE	1332	0	0	0/0	0/0	0/0	0/0	0/0	0/0	0/0	0/0	0/0	0/0	0/1	0/0	0/1	0/0
Chromosome1-9193456 DEL	PRECISE	210	0	0	0/0	0/0	0/0	0/0	0/0	0/0	0/0	0/0	0/0	0/0	0/1	0/0	0/1	0/0
Chromosome1-9194164 INS	PRECISE	86	0	0	0/0	0/0	0/0	0/0	0/0	0/0	0/0	0/0	0/0	0/0	0/1	0/0	0/1	0/0
Chromosome1-9200471 DEL	PRECISE	503	0	0	0/0	0/0	0/0	0/1	0/0	0/0	0/0	0/0	0/0	0/0	0/0	0/0	0/0	0/0
Chromosome1-9215801 DEL	PRECISE	121	0	0	0/0	0/0	0/0	0/0	0/0	0/0	0/0	0/0	0/1	0/0	0/1	0/1	0/1	0/0
Chromosome1-9223134 INS	PRECISE	97	0	0.577	0/0	0/0	0/0	0/0	0/0	0/0	0/0	0/0	0/0	0/0	0/0	0/0	0/1	0/0
Chromosome1-9229876 DEL	PRECISE	2289	0	0	0/0	0/1	0/1	0/0	0/1	0/0	0/0	0/0	0/0	0/0	0/0	0/0	0/0	0/1
Chromosome1-9230078 BND	PRECISE	0	0	0	0/0	0/0	0/0	0/0	0/0	0/0	0/0	0/0	0/1	0/0	0/0	0/0	1/1	0/0
Chromosome1-9230087 BND	PRECISE	0	0	0	0/0	0/0	0/0	0/0	0/0	0/0	0/0	0/0	0/1	0/0	0/0	0/0	0/1	0/0
Chromosome1-9232461 DEL	PRECISE	323	0	0	0/0	0/0	0/0	0/1	0/0	0/0	0/1	0/1	0/0	0/0	0/0	0/0	0/0	0/0
Chromosome1-9243051 INS	PRECISE	70	0.555	0.439	0/0	0/1	0/1	1/1	0/1	0/1	0/1	0/0	0/1	0/1	0/0	0/1	0/1	1/1
Chromosome1-9247031 BND	PRECISE	0	0	0	0/0	0/1	0/1	0/0	0/0	0/0	0/0	0/0	0/0	0/0	0/0	0/0	0/0	0/0
Chromosome1-9250874 DEL	PRECISE	118	0	0	0/0	0/0	0/0	0/0	0/0	0/0	0/0	0/0	0/1	0/0	0/0	0/0	0/0	0/0
Chromosome1-9260671 DEL	PRECISE	986	0	0	0/0	0/0	0/0	0/0	0/0	0/0	0/0	0/0	0/0	0/0	0/0	0/0	0/0	1/1
Chromosome1-9265816 DEL	PRECISE	72	0	0	0/0	0/0	0/0	0/0	0/0	0/0	0/0	0/0	0/1	0/0	0/0	0/0	0/0	0/0
Chromosome1-9265921 BND	PRECISE	0	0	0	0/0	0/0	0/0	0/0	0/0	0/0	0/0	0/0	0/0	0/0	0/0	0/0	0/1	0/0
Chromosome1-9266839 BND	PRECISE	0	0	0	0/0	0/1	0/0	0/0	0/0	0/0	0/0	0/0	0/0	0/0	0/0	0/0	0/0	0/0
Chromosome1-9267426 INS	PRECISE	1278	0	0.894	0/0	0/0	0/1	0/0	0/1	0/0	0/0	0/0	0/0	0/1	0/0	0/0	0/0	0/0
Chromosome1-9268066 INS	PRECISE	136	0	0	0/0	0/0	0/0	0/0	0/0	0/0	0/0	0/0	0/0	0/0	0/0	0/0	0/0	1/1
Chromosome1-9269716 DEL	PRECISE	275	0.577	0.577	0/0	0/0	0/0	0/0	0/0	0/0	0/0	0/0	0/1	0/0	0/0	0/0	0/1	0/0
Chromosome1-9293899 DEL	PRECISE	555	0	0	0/0	0/0	0/0	0/0	0/0	0/0	0/0	0/0	0/0	0/0	0/0	0/0	0/0	0/1
Chromosome1-9296947 INS	PRECISE	1224	0	0	0/0	0/0	0/0	0/1	0/0	0/0	0/0	0/0	0/0	0/0	0/0	0/0	0/0	0/0
Chromosome1-9303104 DEL	PRECISE	3025	0	0	0/0	0/0	0/0	0/1	0/0	0/0	0/0	0/0	0/0	0/0	0/0	0/0	0/0	0/0
Chromosome1-9305415 INS	PRECISE	54	0	0.5	0/0	0/0	0/0	0/0	0/0	0/0	0/0	0/0	0/1	0/0	0/1	0/1	0/0	0/0
Chromosome1-9307606 DEL	PRECISE	56	0.966	0	0/0	0/1	0/1	0/0	0/1	0/1	0/0	0/0	0/1	0/1	0/1	0/1	0/0	0/1
Chromosome1-9308195 DEL	PRECISE	549	0	0	0/0	0/0	0/0	0/0	0/0	0/0	0/0	0/0	0/0	0/0	0/1	0/1	0/0	0/0
Chromosome1-9309471 INS	PRECISE	58	0	0	0/0	0/0	0/0	0/0	0/0	0/0	0/0	0/0	0/1	0/0	0/0	0/0	0/0	0/0
Chromosome1-9312793 INS	PRECISE	1365	0	0.753	0/0	0/1	0/1	0/0	0/1	0/1	0/0	0/0	0/0	0/1	0/0	0/0	0/0	0/1
Chromosome1-9312793 INS	PRECISE	8385	0	0	0/0	0/0	0/0	1/1	0/0	0/0	0/0	0/0	0/0	0/0	0/0	0/0	0/0	0/0
Chromosome1-9313361 DEL	PRECISE	166	0	0	0/0	0/0	0/0	0/0	0/0	0/0	0/0	0/0	0/0	0/0	0/0	0/0	0/0	0/1
Chromosome1-9316203 DEL	PRECISE	1372	0.333	0.333	0/0	0/1	0/1	1/1	0/1	0/1	0/1	0/1	0/0	0/1	0/0	0/0	0/0	1/1
Chromosome1-9318828 BND	PRECISE	0	0	0	0/0	0/0	0/1	0/0	0/0	0/1	0/0	0/0	0/0	0/1	0/0	0/0	0/0	0/1
Chromosome1-9318837 BND	PRECISE	0	0	0	0/0	0/0	0/1	0/0	0/0	0/1	0/0	0/0	0/0	0/1	0/0	0/0	0/0	0/1
Chromosome1-9325081 INS	PRECISE	54	0	0	0/0	0/0	0/0	0/0	0/0	0/0	0/0	0/0	0/0	0/0	0/0	0/0	0/0	0/1
Chromosome1-9326736 INS	PRECISE	116	0	0	0/0	0/0	0/0	0/0	0/0	0/0	0/0	0/0	0/0	0/0	0/0	0/0	0/0	0/1
Chromosome1-9328602 INS	PRECISE	84	0	0	0/0	0/0	0/0	0/1	0/0	0/0	0/1	0/1	0/0	0/0	0/0	0/0	0/0	0/0
Chromosome1-9332673 INS	PRECISE	56	0	0	0/0	0/0	0/0	0/0	0/0	0/0	0/0	0/0	0/0	0/0	0/0	0/0	0/0	0/1

Chromosome1-9335144	DEL	PRECISE	97	0	0	0/0	0/0	0/0	1/1	0/0	0/0	0/1	0/1	0/0	0/0	0/0	0/0	0/0	0/0
Chromosome1-9337651	DEL	PRECISE	193	0	0	0/0	0/1	0/1	1/1	0/1	0/1	0/1	0/1	0/0	0/1	0/0	0/0	0/0	0/1
Chromosome1-9339522	INS	PRECISE	213	0	0.577	0/0	0/0	0/0	0/1	0/0	0/0	0/1	0/1	0/0	0/0	0/0	0/0	0/0	0/0
Chromosome1-9349194	DEL	PRECISE	176	0	0	0/0	0/0	0/0	0/0	0/0	0/0	0/0	0/0	0/0	0/0	0/0	0/0	0/0	0/1
Chromosome1-9349793	INS	PRECISE	787	0	0.833	0/0	0/1	0/1	1/1	0/1	0/1	0/1	0/1	0/0	0/1	0/0	0/0	0/0	1/1
Chromosome1-9360498	DEL	PRECISE	56	0	0	0/0	0/0	0/0	0/0	0/0	0/0	0/0	0/0	0/0	0/0	0/0	0/0	0/0	0/1
Chromosome1-9365949	DEL	PRECISE	626	0	0	0/0	0/1	0/1	1/1	0/1	0/1	0/1	0/1	0/0	0/1	0/0	0/0	0/0	1/1
Chromosome1-9376675	DEL	PRECISE	401	0	0	0/0	0/0	0/0	0/0	0/0	0/0	0/0	0/0	0/1	0/0	0/0	0/0	0/0	0/0
Chromosome1-9376993	INS	PRECISE	1414	0	0.928	0/0	0/1	0/1	1/1	0/1	0/1	0/1	0/1	0/0	0/1	0/0	0/0	0/0	0/1
Chromosome1-9381406	INS	PRECISE	657	0	0	0/0	0/0	0/0	0/0	0/0	0/0	0/0	0/0	0/0	0/0	0/0	0/0	0/0	0/1
Chromosome1-9384668	INS	PRECISE	62	0	0	0/0	0/0	0/0	0/0	0/0	0/0	0/0	0/0	0/0	0/0	0/0	0/0	0/0	0/1
Chromosome1-9387811	DEL	PRECISE	7305	0	0	0/0	0/0	0/0	0/0	0/0	0/0	0/0	0/0	0/1	0/0	0/0	0/0	0/0	0/0
Chromosome1-9391683	INS	PRECISE	72	0	0	0/0	0/1	0/1	1/1	0/1	0/1	0/1	0/1	0/0	0/1	0/0	0/0	0/0	1/1
Chromosome1-9397751	INS	PRECISE	53	0	0.277	0/0	0/1	0/1	1/1	0/1	0/1	0/1	0/1	0/0	0/1	0/0	0/0	0/1	1/1
Chromosome1-9398230	DEL	PRECISE	1958	0	0	0/0	0/1	0/1	1/1	0/1	0/1	0/1	0/1	0/0	0/1	0/0	0/0	0/1	1/1
Chromosome1-9423823	DEL	PRECISE	66	0.9	0.492	0/0	0/1	0/1	1/1	0/1	0/1	0/1	0/1	0/1	0/1	0/0	0/0	0/0	1/1
Chromosome1-9437721	INV	PRECISE	295	0	0	0/0	0/0	0/0	0/0	0/0	0/0	0/0	0/0	0/1	0/0	0/0	0/1	0/0	0/0
Chromosome1-9445734	INS	PRECISE	4630	0	0	0/0	0/0	0/0	0/0	0/0	0/0	0/0	0/0	0/0	0/0	0/0	0/0	0/0	0/1
Chromosome1-9447537	INS	PRECISE	1351	0	0	0/0	0/0	0/0	0/0	0/0	0/0	0/0	0/0	0/0	0/0	0/0	0/0	0/0	0/1
Chromosome1-9449625	BND	PRECISE	0	0	0	0/0	0/0	0/0	0/0	0/0	0/0	0/0	0/0	0/0	0/0	0/0	0/0	0/1	0/0
Chromosome1-9449900	DEL	PRECISE	416	0	0	0/0	0/0	0/0	0/0	0/0	0/0	0/0	0/0	0/1	0/0	0/0	0/1	0/0	0/0
Chromosome1-9451026	DEL	PRECISE	721	0	0	0/0	0/0	0/0	0/0	0/0	0/0	0/0	0/0	0/1	0/0	0/0	0/1	0/0	0/0
Chromosome1-9454834	BND	PRECISE	0	0	0	0/0	0/0	0/0	0/0	0/0	0/0	0/0	0/0	0/0	0/1	0/0	0/0	0/0	0/0
Chromosome1-9460673	INS	PRECISE	702	0	0	0/0	0/0	0/0	0/0	0/0	0/0	0/0	0/0	0/0	0/0	0/0	0/1	0/0	0/0
Chromosome1-9468477	BND	PRECISE	0	0	0	0/0	0/0	0/0	0/0	0/0	0/0	0/0	0/0	0/1	0/0	0/0	0/0	0/0	0/0
Chromosome1-9469025	INS	PRECISE	308	0	0	0/0	0/0	0/0	0/0	0/0	0/0	0/0	0/0	0/0	0/0	0/0	0/0	0/0	0/1
Chromosome1-9469539	DEL	PRECISE	50	0	0	0/0	0/0	0/0	0/0	0/0	0/0	0/0	0/0	0/1	0/0	0/1	0/1	0/0	0/0
Chromosome1-9484479	INS	PRECISE	56	0	0.577	0/0	0/0	0/0	0/0	0/0	0/0	0/0	0/0	0/0	0/0	0/1	0/0	0/0	0/0
Chromosome1-9485849	DEL	PRECISE	192	0	0	0/0	0/0	0/0	0/0	0/0	0/0	0/0	0/0	0/0	0/0	0/1	0/0	0/0	0/0
Chromosome1-9486649	DEL	PRECISE	1566	0.577	0.577	0/0	0/0	0/0	0/0	0/0	0/0	0/0	0/0	0/1	0/0	0/1	0/1	0/0	0/0
Chromosome1-9488713	DEL	PRECISE	60	0	0	0/0	0/0	0/0	0/0	0/0	0/0	0/0	0/0	0/0	0/0	0/0	0/0	0/0	0/1
Chromosome1-9500558	INS	PRECISE	275	0	0.632	0/0	0/1	0/1	0/0	0/1	0/1	0/0	0/0	0/0	0/1	0/0	0/0	0/0	1/1
Chromosome1-9503605	DEL	PRECISE	1455	0	0	0/0	0/0	0/0	0/0	0/0	0/0	0/0	0/0	0/0	0/0	0/0	0/0	0/0	0/1
Chromosome1-9518724	INS	PRECISE	5931	0	0	0/0	0/0	0/0	1/1	0/0	0/0	0/0	0/0	0/0	0/0	0/0	0/0	0/0	0/0
Chromosome1-9525559	DEL	PRECISE	54	0	0	0/0	0/0	0/0	0/0	0/0	0/0	0/0	0/0	0/1	0/0	0/1	0/0	0/1	0/0
Chromosome1-9530673	INS	PRECISE	193	0	0	0/0	0/0	0/0	0/1	0/0	0/0	0/0	0/0	0/0	0/0	0/0	0/0	0/0	0/0
Chromosome1-9545413	INS	PRECISE	12297	0	0	0/0	0/0	0/0	0/0	0/0	0/0	0/0	0/0	0/0	0/0	0/0	0/1	0/0	0/0
Chromosome1-9549753	DEL	PRECISE	8670	0	0	0/0	0/0	0/0	0/0	0/0	0/0	0/0	0/0	0/1	0/0	0/1	0/1	0/1	0/0
Chromosome1-9557061	INS	PRECISE	173	0	0	0/0	0/0	0/0	0/1	0/0	0/0	0/0	0/0	0/0	0/0	0/0	0/0	0/0	0/0
Chromosome1-9564044	INS	PRECISE	212	0	0.577	0/0	0/0	0/0	0/0	0/0	0/0	0/0	0/0	0/0	0/0	0/1	0/1	0/1	0/0
Chromosome1-9574519	BND	PRECISE	0	0	0	0/0	0/0	0/0	0/0	0/0	0/0	0/0	0/0	0/0	0/0	0/0	0/0	0/0	0/1
Chromosome1-9593503	DEL	PRECISE	576	0	0	0/0	0/0	0/0	0/0	0/0	0/0	0/0	0/0	0/1	0/0	0/1	0/1	0/1	0/0
Chromosome1-9603717	DEL	PRECISE	418	0	0	0/0	0/0	0/0	0/0	0/0	0/0	0/0	0/0	0/0	0/0	0/0	0/0	0/0	0/1
Chromosome1-9608086	DEL	PRECISE	54	0	0	0/0	0/0	0/0	0/0	0/0	0/0	0/0	0/0	0/0	0/0	0/0	0/1	0/0	0/0
Chromosome1-9609010	INS	PRECISE	602	0	0	0/0	0/0	0/0	0/0	0/0	0/0	0/0	0/0	0/0	0/0	0/0	0/0	0/0	0/1
Chromosome1-9609675	INS	PRECISE	10228	0	0	0/0	0/0	0/0	0/0	0/0	0/0	0/0	0/0	0/0	0/0	0/0	0/1	0/0	0/0
Chromosome1-9623671	DEL	PRECISE	6250	0	0	0/0	0/0	0/0	0/0	0/0	0/0	0/0	0/0	0/0	0/0	0/0	0/1	0/0	0/0
Chromosome1-9627724	DEL	PRECISE	12576	0	0	0/0	0/0	0/0	0/1	0/0	0/0	0/0	0/0	0/0	0/0	0/0	0/0	0/0	0/0
Chromosome1-9632574	INS	PRECISE	1287	0	0	0/0	0/0	0/0	0/0	0/0	0/0	0/0	0/0	0/0	0/0	0/0	0/1	0/0	0/0
Chromosome1-9634176	DEL	PRECISE	1552	0	0	0/0	0/1	0/1	0/0	0/1	0/1	0/0	0/0	0/0	0/1	0/0	0/0	0/0	1/1
Chromosome1-9675205	DEL	PRECISE	3791	0	0	0/0	0/0	0/0	0/0	0/0	0/0	0/0	0/0	0/1	0/0	0/1	0/1	0/1	0/0
Chromosome1-9684985	INS	PRECISE	106	0	0.5	0/0	0/0	0/0	0/0	0/0	0/0	0/0	0/0	0/1	0/0	0/1	0/0	0/1	0/0
Chromosome1-9686129	INS	PRECISE	104	0	0.5	0/0	0/0	0/0	0/0	0/0	0/0	0/0	0/0	0/1	0/0	0/1	0/0	0/1	0/0
Chromosome1-9686366	INS	PRECISE	8232	0	0	0/0	0/0	0/0	0/0	0/0	0/0	0/0	0/0	0/0	0/0	0/0	0/0	0/0	0/1
Chromosome1-9690463	INS	PRECISE	831	0	0	0/0	0/0	0/0	0/0	0/0	0/0	0/0	0/0	0/0	0/0	0/0	0/1	0/0	0/0
Chromosome1-9693069	DEL	PRECISE	53	0	0	0/0	0/0	0/0	0/0	0/0	0/0	0/0	0/0	0/1	0/0	0/1	0/1	0/1	0/0
Chromosome1-9694527	INS	PRECISE	1321	0	0	0/0	0/0	0/0	0/0	0/0	0/0	0/0	0/0	0/1	0/0	0/0	0/0	0/0	0/0
Chromosome1-9698227	INS	PRECISE	64	0	0	0/0	0/0	0/0	0/0	0/0	0/0	0/0	0/0	0/1	0/0	0/0	0/0	0/0	0/0
Chromosome1-9698612	DEL	PRECISE	7544	0	0	0/0	0/1	0/1	1/1	0/1	0/1	0/1	0/1	0/1	0/1	0/0	0/1	0/1	1/1
Chromosome1-9703913	DEL	PRECISE	121	0	0	0/0	0/0	0/0	0/0	0/0	0/0	0/1	0/0	0/0	0/0	0/0	0/0	0/0	0/0
Chromosome1-9708024	INS	PRECISE	161	0	0.408	0/0	0/1	0/1	0/0	0/1	0/1	0/0	0/0	0/0	0/1	0/0	0/0	0/0	0/1
Chromosome1-9710030	INS	PRECISE	1779	0	0	0/0	0/0	0/0	0/0	0/0	0/0	0/0	0/0	0/1	0/0	0/0	0/0	0/0	0/0
Chromosome1-9710140	INS	PRECISE	159	0	0.577	0/0	0/0	0/0	0/0	0/0	0/0	0/0	0/0	0/0	0/0	0/1	0/1	0/1	0/0
Chromosome1-9714612	INS	PRECISE	5180	0	0	0/0	0/0	0/0	0/0	0/0	0/0	0/0	0/0	1/1	0/0	0/0	0/0	0/0	0/0
Chromosome1-9725044	INS	PRECISE	79	0	0	0/0	0/1	0/1	0/0	0/1	0/1	0/0	0/0	0/0	0/1	0/0	0/0	0/0	0/1
Chromosome1-9736464	BND	PRECISE	0	0	0	0/0	0/0	0/0	0/0	0/0	0/0	0/0	0/0	0/1	0/0	0/0	0/0	0/0	0/0
Chromosome1-9737053	DEL	PRECISE	836	0	0	0/0	0/0	0/0	0/0	0/0	0/0	0/0	0/0	0/0	0/0	0/0	1/1	0/1	0/0
Chromosome1-9743202	DEL	PRECISE	528	0	0	0/0	0/1	0/1	0/0	0/1	0/1	0/0	0/0	0/0	0/0	0/0	0/0	0/0	1/1
Chromosome1-9748861	INS	PRECISE	238	0	0.333	0/0	0/1	0/1	0/0	0/1	0/1	0/0	0/0	0/0	0/1	0/0	0/1	0/1	1/1
Chromosome1-9750538	BND	PRECISE	0	0	0	0/0	0/0	0/0	0/0	0/0	0/0	0/0	0/0	0/1	0/0	0/0	0/0	0/0	0/0
Chromosome1-9751859	INS	PRECISE	3546	0	0	0/0	0/0	0/0	0/0	0/0	0/0	0/0	0/0	1/1	0/0	0/0	0/0	0/0	0/0
Chromosome1-9760859	INS	PRECISE	51	0	0	0/0	0/0	0/0	0/0	0/0	0/0	0/0	0/0	0/1	0/0	0/1	0/1	0/1	0/0
Chromosome1-9766993	DEL	PRECISE	16403	0	0	0/0	0/1	0/1	0/0	0/1	0/1	0/0	0/0	0/0	0/1	0/0	0/0	0/0	0/1

Chromosome1-9767041	INS	PRECISE	97	0	0.447	0/0	0/0	0/0	0/0	0/0	0/0	0/0	0/0	0/0	0/0	0/1	0/0	0/1	0/1
Chromosome1-9777200	INS	PRECISE	128	0	0.5	0/0	0/0	0/0	0/0	0/0	0/0	0/0	0/0	0/1	0/0	0/1	0/1	0/1	0/0
Chromosome1-9782039	DEL	PRECISE	6771	0	0	0/0	0/0	0/0	0/0	0/0	0/0	0/0	0/0	0/0	0/0	0/1	0/1	0/0	0/0
Chromosome1-9784420	DEL	PRECISE	24452	0	0	0/0	0/0	0/0	0/0	0/0	0/0	0/0	0/0	0/1	0/0	0/0	0/0	0/0	1/1
Chromosome1-9789839	INS	PRECISE	54	0	0	0/0	0/0	0/0	0/0	0/0	0/0	0/0	0/0	0/0	0/0	0/1	0/0	0/0	0/0
Chromosome1-9791541	DEL	PRECISE	1565	0.354	0.354	0/0	0/1	0/1	0/0	0/1	0/1	0/0	0/0	0/0	0/1	0/1	0/1	0/0	1/1
Chromosome1-9792023	DEL	PRECISE	680	0	0	0/0	0/0	0/0	1/1	0/0	0/0	0/1	0/1	0/0	0/0	0/0	0/0	0/0	0/0
Chromosome1-9802480	INS	PRECISE	73	0	0	0/0	0/0	0/0	0/0	0/0	0/0	0/0	0/0	0/0	0/0	0/1	0/0	0/0	0/0
Chromosome1-9807922	INS	PRECISE	5897	0	0	0/0	0/0	0/0	0/1	0/0	0/0	0/0	0/0	0/0	0/0	0/0	0/0	0/0	0/0
Chromosome1-9810219	INS	PRECISE	1480	0	0.5	0/0	0/0	0/0	0/0	0/0	0/0	0/0	0/0	0/0	0/0	0/1	0/1	0/1	0/0
Chromosome1-9816510	INS	PRECISE	977	0	0.577	0/0	0/0	0/0	0/0	0/0	0/0	0/0	0/0	0/0	0/0	0/1	0/1	0/1	0/0
Chromosome1-9816529	INS	PRECISE	5083	0	0	0/0	0/0	0/0	0/0	0/0	0/0	0/0	0/0	0/1	0/0	0/0	0/0	0/0	0/0
Chromosome1-9817235	INS	PRECISE	480	0	0.577	0/0	0/0	0/0	0/0	0/0	0/0	0/0	0/0	0/0	0/0	0/1	0/1	0/1	0/0
Chromosome1-9820817	DEL	PRECISE	7872	0.577	0	0/0	0/0	0/0	0/0	0/0	0/0	0/0	0/0	0/1	0/0	0/1	0/1	0/0	0/0
Chromosome1-9841579	DEL	PRECISE	113	0	0	0/0	0/0	0/0	0/1	0/0	0/0	0/1	0/1	0/0	0/0	0/0	0/0	0/0	0/0
Chromosome1-9844698	INS	PRECISE	66	0	0	0/0	0/0	0/0	0/1	0/0	0/0	0/0	0/0	0/0	0/0	0/0	0/0	0/0	0/0
Chromosome1-9846397	INS	PRECISE	50	0	0	0/0	0/0	0/0	0/0	0/0	0/0	0/0	0/0	0/1	0/0	0/0	0/0	0/0	0/0
Chromosome1-9846897	DEL	PRECISE	1157	0	0	0/0	0/0	0/0	0/0	0/0	0/0	0/0	0/0	0/1	0/0	0/0	0/1	0/0	0/0
Chromosome1-9851587	DEL	PRECISE	655	0.333	0.333	0/0	0/1	0/1	0/0	0/1	0/1	0/0	0/0	0/1	0/1	0/0	0/1	0/0	1/1
Chromosome1-9854590	DEL	PRECISE	138	0.577	0.577	0/0	0/0	0/0	0/0	0/0	0/0	0/0	0/0	0/1	0/0	0/1	0/1	0/0	0/0
Chromosome1-9856175	DEL	PRECISE	137	0	0	0/0	0/0	0/0	0/0	0/0	0/0	0/0	0/0	0/1	0/0	0/1	0/1	0/0	0/0
Chromosome1-9857491	DEL	PRECISE	61	0	0	0/0	0/0	0/0	0/0	0/0	0/0	0/0	0/0	0/1	0/0	0/1	0/1	0/0	0/0
Chromosome1-9870172	INS	PRECISE	668	0	0.837	0/0	0/1	0/1	0/0	0/1	0/0	0/0	0/0	0/0	0/1	0/0	0/0	0/0	0/1
Chromosome1-9871646	INS	PRECISE	454	0	0	0/0	0/0	0/0	0/0	0/0	0/0	0/0	0/0	0/1	0/0	0/0	0/0	0/0	0/0
Chromosome1-9881937	INS	PRECISE	3545	0	0	0/0	0/0	0/0	0/0	0/0	0/0	0/0	0/0	0/1	0/0	0/0	0/0	0/0	0/0
Chromosome1-9885434	DEL	PRECISE	1213	0	0	0/0	0/0	0/0	0/0	0/0	0/0	0/0	0/0	0/1	0/0	0/1	0/1	0/1	0/0
Chromosome1-9887382	DEL	PRECISE	144	0	0	0/0	0/1	0/1	0/0	0/1	0/1	0/0	0/0	0/1	0/1	0/1	0/1	0/1	0/1
Chromosome1-9890346	DEL	PRECISE	52	0	0	0/0	0/0	0/0	0/0	0/0	0/0	0/0	0/0	0/0	0/0	0/1	0/1	0/1	0/0
Chromosome1-9892807	DEL	PRECISE	192	0	0	0/0	0/0	0/0	0/0	0/0	0/0	0/0	0/0	0/1	0/0	0/1	0/1	0/1	0/0
Chromosome1-9898312	DEL	PRECISE	724	0	0	0/0	0/0	0/0	0/0	0/0	0/0	0/0	0/0	0/1	0/0	0/1	0/1	0/1	0/0
Chromosome1-9907977	INS	PRECISE	142	0	0	0/0	0/0	0/0	0/0	0/0	0/0	0/0	0/0	0/0	0/0	0/0	0/0	0/0	0/1
Chromosome1-9913734	DEL	PRECISE	2305	0	0	0/0	0/1	0/1	0/0	0/1	0/1	0/0	0/0	0/0	0/1	0/0	0/0	0/0	0/1
Chromosome1-9916302	INS	PRECISE	553	0	0	0/0	0/0	0/0	0/0	0/0	0/0	0/0	0/0	0/0	0/0	0/0	0/0	0/0	0/1
Chromosome1-9924511	DEL	PRECISE	94	0	0	0/0	0/1	0/1	0/0	0/1	0/1	0/0	0/0	0/0	0/1	0/0	0/0	0/0	0/1
Chromosome1-9929814	BND	PRECISE	0	0.447	0	0/0	0/1	0/0	0/0	0/0	0/1	0/0	0/0	0/0	0/0	0/0	0/0	0/0	0/0
Chromosome1-9938451	DEL	PRECISE	2903	0	0	0/0	0/0	0/0	0/0	0/0	0/0	0/0	0/0	0/1	0/0	0/1	0/1	0/1	0/0
Chromosome1-9944232	INS	PRECISE	554	0	0.577	0/0	0/0	0/0	0/0	0/0	0/0	0/0	0/0	0/0	0/0	0/1	0/0	0/0	0/0
Chromosome1-9946935	INS	PRECISE	530	0	0.408	0/0	0/1	0/1	0/0	0/1	0/1	0/0	0/0	0/0	0/1	0/0	0/0	0/0	0/1
Chromosome1-9951286	DEL	PRECISE	111	0	0	0/0	0/0	0/0	0/0	0/0	0/0	0/0	0/0	0/1	0/0	0/1	0/1	0/1	0/0
Chromosome1-9958577	DEL	PRECISE	14012	0	0	0/0	0/0	0/0	0/0	0/0	0/0	0/0	0/0	0/1	0/0	0/0	0/1	0/1	0/0
Chromosome1-9965171	DEL	PRECISE	115043	0	0	0/0	0/0	0/0	0/0	0/0	0/0	0/0	0/0	0/0	0/0	0/0	0/0	0/0	1/1
Chromosome1-9974650	INS	PRECISE	8485	0	0	0/0	0/0	0/0	1/1	0/0	0/0	0/0	0/0	0/0	0/0	0/0	0/0	0/0	0/0
Chromosome1-9976684	DEL	PRECISE	62	0	0	0/0	0/0	0/0	0/1	0/0	0/0	0/1	0/1	0/0	0/0	0/0	0/0	0/0	0/0
Chromosome1-9986039	BND	PRECISE	0	0	0	0/0	0/0	0/0	0/0	0/0	0/0	0/0	0/1	0/0	0/0	0/0	0/0	0/0	0/0
Chromosome1-9986851	INS	PRECISE	53	0.447	0.837	0/0	0/0	0/0	0/0	0/0	0/0	0/0	0/0	0/0	0/0	0/0	0/0	0/1	0/1
Chromosome1-9993914	INS	PRECISE	590	0	0	0/0	0/0	0/0	0/0	0/0	0/0	0/1	0/0	0/0	0/0	0/0	0/0	0/0	0/0
Chromosome1-1000214	INS	PRECISE	505	0	0.718	0/0	0/1	0/1	1/1	1/1	1/1	0/1	0/1	1/1	0/1	0/0	0/1	0/1	1/1
Chromosome1-1001516	DEL	PRECISE	103	0	0	0/0	0/0	0/0	0/0	0/0	0/0	0/0	0/0	0/0	0/0	0/1	0/0	0/0	0/0
Chromosome1-1001549	INS	PRECISE	120	0	0	0/0	0/0	0/0	0/0	0/0	0/0	0/0	0/0	0/0	0/0	0/1	0/0	0/0	0/0
Chromosome1-1001877	INS	PRECISE	8815	0	0	0/0	0/0	0/0	0/0	0/0	0/0	0/0	0/0	0/0	0/0	0/1	0/0	0/0	0/0
Chromosome1-1002307	DEL	PRECISE	1415	0	0	0/0	0/0	0/0	0/0	0/0	0/0	0/0	0/0	0/1	0/0	0/1	0/1	0/1	0/0
Chromosome1-1003190	DEL	PRECISE	131	0	0	0/0	0/1	0/1	0/0	0/1	0/1	0/0	0/0	0/0	0/1	0/0	0/0	0/0	1/1
Chromosome1-1004111	DEL	PRECISE	78	0	0	0/0	0/0	0/0	0/0	0/0	0/0	0/0	0/0	0/1	0/0	0/1	0/1	0/1	0/0
Chromosome1-1004701	DEL	PRECISE	5897	0	0	0/0	0/0	0/0	0/0	0/0	0/0	0/0	0/0	0/1	0/0	0/1	1/1	0/1	0/0
Chromosome1-1005773	INS	PRECISE	2322	0	0.577	0/0	0/0	0/0	0/0	0/0	0/0	0/0	0/0	0/1	0/0	0/1	0/1	0/1	0/0
Chromosome1-1005792	INS	PRECISE	200	0	0	0/0	0/0	0/0	0/1	0/0	0/0	0/0	0/0	0/0	0/0	0/0	0/0	0/0	0/0
Chromosome1-1005813	DEL	PRECISE	793	0	0	0/0	0/0	0/0	0/1	0/0	0/0	0/1	0/1	0/0	0/0	0/0	0/0	0/0	0/0
Chromosome1-1006408	DEL	PRECISE	107	0	0	0/0	0/0	0/0	0/0	0/0	0/0	0/0	0/0	0/1	0/0	0/1	0/1	0/1	0/0
Chromosome1-1006908	INS	PRECISE	547	0	0.577	0/0	0/0	0/0	0/0	0/0	0/0	0/0	0/0	0/1	0/0	0/1	0/1	0/1	0/0
Chromosome1-1007171	INS	PRECISE	70	0	0.316	0/0	0/1	0/1	0/0	0/1	0/1	0/0	0/0	0/1	0/1	0/1	0/1	0/1	1/1
Chromosome1-1007601	BND	PRECISE	0	0	0	0/0	0/0	0/0	0/0	0/0	0/0	0/0	0/0	0/0	0/0	0/1	0/0	0/0	0/0
Chromosome1-1007700	DEL	PRECISE	269	0	0	0/0	0/0	0/0	0/0	0/0	0/0	0/0	0/0	0/1	0/0	0/0	0/0	0/0	0/0
Chromosome1-1008275	INS	PRECISE	80	0	0	0/0	0/0	0/0	0/0	0/0	0/0	0/0	0/0	0/1	0/0	0/1	0/1	0/1	0/0
Chromosome1-1009290	INV	PRECISE	3239	0	0	0/0	0/0	0/0	0/0	0/0	0/0	0/0	0/0	0/0	0/0	0/0	0/0	0/0	0/1
Chromosome1-1009571	INV	PRECISE	460	0	0	0/0	0/0	0/0	0/0	0/0	0/0	0/0	0/0	0/0	0/0	0/0	0/0	0/0	0/1
Chromosome1-1009645	DEL	PRECISE	836	0	0	0/0	0/0	0/0	0/0	0/0	0/0	0/0	0/0	0/1	0/0	0/1	0/1	0/1	0/0
Chromosome1-1010083	INS	PRECISE	4467	0	0	0/0	0/0	0/0	1/1	0/0	0/0	0/0	0/0	0/0	0/0	0/0	0/0	0/0	0/0
Chromosome1-1010110	DEL	PRECISE	1196	0	0	0/0	0/0	0/0	0/1	0/0	0/0	0/1	0/1	0/0	0/0	0/0	0/0	0/0	0/0
Chromosome1-1010311	INS	PRECISE	150	0	0.5	0/0	0/0	0/0	0/0	0/0	0/0	0/0	0/0	0/1	0/0	0/1	0/1	0/1	0/0
Chromosome1-1010595	DEL	PRECISE	67	0	0	0/0	0/0	0/0	0/0	0/1	0/1	0/0	0/0	0/1	0/0	0/0	0/0	0/0	0/0
Chromosome1-1010870	DEL	PRECISE	348	0	0	0/0	0/1	0/1	0/0	1/1	1/1	0/0	0/0	1/1	0/1	0/0	0/0	0/0	1/1
Chromosome1-1011667	INS	PRECISE	879	0	0	0/0	0/0	0/0	0/0	0/0	0/0	0/0	0/0	0/0	0/0	0/0	0/0	0/0	0/1
Chromosome1-1012701	INS	PRECISE	261	0	0	0/0	0/0	0/0	0/0	0/0	0/0	0/0	0/0	0/0	0/0	0/0	0/0	0/0	0/1

Chromosome1-1012920	INS	PRECISE	211	0	0.5	0/0	0/0	0/0	0/0	0/0	0/0	0/0	0/0	0/1	0/0	0/1	0/1	0/1	0/0
Chromosome1-1012953	INS	PRECISE	1483	0	0	0/0	0/0	0/0	0/0	0/0	0/0	0/1	0/1	0/0	0/0	0/0	0/0	0/0	0/0
Chromosome1-1013443	INS	PRECISE	9572	0	0	0/0	0/0	0/0	0/0	0/0	0/0	0/0	0/0	1/1	0/0	0/0	0/0	0/0	0/0
Chromosome1-1013446	BND	PRECISE	0	0	0	0/0	0/0	0/0	0/0	0/0	0/0	0/0	0/0	0/1	0/0	0/0	0/0	0/0	0/0
Chromosome1-1013912	DEL	PRECISE	1383	0	0	0/0	0/0	0/0	0/0	0/0	0/0	0/0	0/0	0/1	0/0	0/1	0/1	0/1	0/0
Chromosome1-1014557	INS	PRECISE	1141	0	0	0/0	0/0	0/0	0/1	0/0	0/0	0/0	0/0	0/0	0/0	0/0	0/0	0/0	0/0
Chromosome1-1014765	INS	PRECISE	214	0	0	0/0	0/0	0/0	0/0	0/0	0/0	0/0	0/0	0/0	0/0	0/0	0/0	0/0	0/1
Chromosome1-1015090	DEL	PRECISE	51	0	0	0/0	0/0	0/0	0/0	0/0	0/0	0/0	0/0	0/1	0/0	0/0	0/0	0/0	0/0
Chromosome1-1017487	INS	PRECISE	846	0	0	0/0	0/0	0/0	0/0	0/0	0/0	0/0	0/0	0/1	0/0	0/0	0/0	0/0	0/0
Chromosome1-1018462	INS	PRECISE	3328	0	0	0/0	0/0	0/0	0/0	0/0	0/0	0/0	0/0	1/1	0/0	0/0	0/0	0/0	0/0
Chromosome1-1018599	INS	PRECISE	211	0	0	0/0	0/0	0/0	0/1	0/0	0/0	0/0	0/0	0/0	0/0	0/0	0/0	0/0	0/0
Chromosome1-1018637	DEL	PRECISE	1868	0	0	0/0	0/1	0/1	0/0	0/1	0/1	0/0	0/0	0/0	0/1	0/0	0/0	0/0	0/1
Chromosome1-1018670	DEL	PRECISE	1181	0	0	0/0	0/0	0/0	0/1	0/0	0/0	0/0	0/1	0/0	0/0	0/0	0/0	0/0	0/1
Chromosome1-1018890	DEL	PRECISE	2538	0.667	0.667	0/0	0/1	0/1	0/0	0/1	0/1	0/0	0/0	0/1	0/1	0/1	0/0	0/1	1/1
Chromosome1-1020318	DEL	PRECISE	97	0	0	0/0	0/0	0/0	0/0	0/0	0/0	0/0	0/0	0/0	0/0	0/1	0/1	0/1	0/0
Chromosome1-1020680	INS	PRECISE	272	0	0.699	0/0	0/1	0/1	0/0	0/1	0/1	0/0	0/0	0/1	0/1	0/1	0/1	0/1	1/1
Chromosome1-1020958	INS	PRECISE	5234	0	0	0/0	0/0	0/0	0/0	0/0	0/0	0/0	0/0	1/1	0/0	0/0	0/0	0/0	0/0
Chromosome1-1020958	BND	PRECISE	0	0	0	0/0	0/0	0/0	0/0	0/0	0/0	0/0	0/0	0/1	0/0	0/0	0/0	0/0	0/0
Chromosome1-1021066	INS	PRECISE	614	0	0	0/0	0/0	0/0	0/0	0/0	0/0	0/0	0/0	1/1	0/0	0/0	0/0	0/0	0/0
Chromosome1-1021397	INS	PRECISE	6033	0	0	0/0	0/0	0/0	0/0	0/0	0/0	0/0	0/0	1/1	0/0	0/0	0/0	0/0	0/0
Chromosome1-1022809	DEL	PRECISE	118	0	0	0/0	0/0	0/0	0/0	0/0	0/0	0/0	0/0	0/1	0/0	0/1	0/1	0/0	0/0
Chromosome1-1023173	INS	PRECISE	2989	0	0	0/0	0/0	0/0	0/0	0/0	0/0	0/0	0/0	0/0	0/0	0/0	0/0	0/0	1/1
Chromosome1-1023720	DEL	PRECISE	624	0	0	0/0	0/0	0/0	0/0	0/0	0/0	0/0	0/0	0/1	0/0	0/0	0/0	0/0	0/0
Chromosome1-1023792	INS	PRECISE	888	0	0.983	0/0	0/1	0/1	0/0	0/1	0/1	0/0	0/0	0/0	0/1	0/0	0/0	0/0	0/1
Chromosome1-1024872	DEL	PRECISE	905	0	0	0/0	0/1	0/1	0/0	0/1	0/1	0/0	0/0	0/0	0/1	0/1	0/1	0/1	1/1
Chromosome1-1025167	DEL	PRECISE	88	0	0	0/0	0/1	0/1	0/0	0/1	0/1	0/0	0/0	0/0	0/1	0/0	0/0	0/0	0/1
Chromosome1-1025278	DEL	PRECISE	53	0	0	0/0	0/1	0/1	0/1	0/1	0/1	0/1	0/1	0/0	0/1	0/0	0/0	0/0	1/1
Chromosome1-1025735	INS	PRECISE	233	0	0.577	0/0	0/0	0/0	0/0	0/0	0/0	0/0	0/0	0/0	0/0	0/1	0/1	0/1	0/0
Chromosome1-1025984	DEL	PRECISE	616	0.447	0.447	0/0	0/0	0/0	0/1	0/0	0/0	0/0	0/0	1/1	0/0	0/1	1/1	1/1	0/0
Chromosome1-1027072	DEL	PRECISE	426	0	0	0/0	0/1	0/1	0/0	0/1	0/1	0/0	0/0	0/1	0/1	0/1	1/1	0/1	1/1
Chromosome1-1028059	INV	PRECISE	3861342	0	0	0/0	0/0	0/0	0/0	0/0	0/0	0/0	0/0	0/0	0/0	0/0	0/0	0/0	0/1
Chromosome1-1028331	INS	PRECISE	10403	0	0	0/0	0/0	0/0	0/0	0/0	0/0	0/0	0/0	0/1	0/0	0/0	0/0	0/0	0/0
Chromosome1-1032380	INS	PRECISE	1039	0	0.5	0/0	0/0	0/0	0/0	0/0	0/0	0/0	0/0	1/1	0/0	0/1	0/1	0/1	0/0
Chromosome1-1034416	DEL	PRECISE	712	0	0	0/0	0/0	0/0	0/0	0/0	0/0	0/0	0/0	0/0	0/0	0/1	0/1	0/1	0/0
Chromosome1-1034570	DEL	PRECISE	1650	0	0	0/0	0/0	0/0	0/0	0/0	0/0	0/0	0/0	0/0	0/0	0/1	0/1	0/1	0/0
Chromosome1-1034751	DEL	PRECISE	61	0	0	0/0	0/0	0/0	0/0	0/0	0/0	0/0	0/0	0/0	0/0	0/1	0/1	0/1	0/0
Chromosome1-1035664	INS	PRECISE	7801	0	0	0/0	0/0	0/0	0/0	0/0	0/0	0/0	0/0	0/1	0/0	0/0	0/0	0/0	0/0
Chromosome1-1036342	BND	PRECISE	0	0	0	0/0	0/0	0/0	0/0	0/0	0/0	0/0	0/1	0/0	0/0	0/0	0/0	0/0	0/0
Chromosome1-1036525	DEL	PRECISE	66	0	0	0/0	0/0	0/0	0/0	0/0	0/0	0/0	0/0	0/1	0/0	0/0	0/0	0/0	0/0
Chromosome1-1036617	INS	PRECISE	1005	0	0	0/0	0/0	0/0	0/0	0/0	0/0	0/0	0/0	0/1	0/0	0/0	0/0	0/0	0/0
Chromosome1-1036801	INS	PRECISE	2263	0	0	0/0	0/0	0/0	0/0	0/0	0/0	0/0	0/0	0/0	0/0	0/0	0/0	0/0	0/1
Chromosome1-1036872	INS	PRECISE	1549	0	0	0/0	0/0	0/0	0/0	0/0	0/0	0/0	0/0	0/1	0/0	0/0	0/0	0/0	0/0
Chromosome1-1038516	DEL	PRECISE	616	0	0	0/0	0/0	0/0	0/0	0/0	0/0	0/0	0/0	0/0	0/0	0/0	0/0	0/0	0/1
Chromosome1-1039784	BND	PRECISE	0	0	0	0/0	0/1	0/1	0/0	0/0	0/0	0/0	0/0	0/0	0/0	0/0	0/0	0/0	0/0
Chromosome1-1039903	DEL	PRECISE	5054	0	0	0/0	0/1	0/1	0/0	0/1	0/1	0/0	0/0	0/1	0/1	0/1	0/1	0/1	1/1
Chromosome1-1040569	INS	PRECISE	58	0	0	0/0	0/0	0/0	0/1	0/0	0/0	0/0	0/0	0/0	0/0	0/0	0/0	0/0	0/0
Chromosome1-1041606	DEL	PRECISE	1483	0	0	0/0	0/0	0/0	0/0	0/0	0/0	0/0	0/0	0/1	0/0	0/1	0/1	0/1	0/0
Chromosome1-1041964	DEL	PRECISE	1428	0	0	0/0	0/0	0/0	0/0	0/0	0/0	0/0	0/0	0/1	0/0	0/0	0/0	0/0	0/0
Chromosome1-1042116	DEL	PRECISE	208	0	0	0/0	0/0	0/0	0/0	0/0	0/0	0/0	0/0	0/1	0/0	0/0	0/0	0/0	0/0
Chromosome1-1043181	INS	PRECISE	10352	0	0	0/0	0/0	0/0	0/0	0/0	0/0	0/0	0/0	0/0	0/0	0/0	0/0	0/0	1/1
Chromosome1-1043347	DEL	PRECISE	4906	0	0	0/0	0/1	0/1	0/0	0/1	0/1	0/0	0/0	0/1	0/1	0/1	0/1	0/1	1/1
Chromosome1-1044912	DEL	PRECISE	149	0	0	0/0	0/0	0/0	0/0	0/0	0/0	0/0	0/0	0/1	0/0	0/1	0/1	0/1	0/0
Chromosome1-1044974	INS	PRECISE	426	0	0.5	0/0	0/0	0/0	0/0	0/0	0/0	0/0	0/0	0/1	0/0	0/1	0/1	0/1	0/0
Chromosome1-1046446	DEL	PRECISE	57	0	0	0/0	0/0	0/0	0/0	0/0	0/0	0/0	0/0	0/1	0/0	0/0	0/0	0/0	0/0
Chromosome1-1047031	DEL	PRECISE	193	0	0	0/0	0/0	0/0	0/0	0/0	0/0	0/0	0/0	0/1	0/0	0/1	0/1	0/1	0/0
Chromosome1-1047212	INS	PRECISE	161	0	0	0/0	0/0	0/0	0/0	0/0	0/0	0/0	0/0	0/0	0/0	0/0	0/0	0/0	0/1
Chromosome1-1047643	DEL	PRECISE	9630	0	0	0/0	0/0	0/0	0/0	0/0	0/0	0/0	0/0	0/1	0/0	0/1	0/0	0/1	0/0
Chromosome1-1047663	DEL	PRECISE	72	0	0	0/0	0/1	0/1	0/0	0/1	0/1	0/0	0/0	0/0	0/1	0/0	0/0	0/0	0/1
Chromosome1-1048605	BND	PRECISE	0	0	0	0/0	0/0	0/0	0/0	0/0	0/0	0/0	0/0	0/0	0/0	0/0	0/0	0/0	0/1
Chromosome1-1049456	INS	PRECISE	53	0	0	0/0	0/0	0/0	0/0	0/0	0/0	0/0	0/0	0/0	0/0	0/0	0/1	0/0	0/0
Chromosome1-1050867	BND	PRECISE	0	0.408	0	0/0	0/0	0/1	0/0	0/0	0/0	0/0	0/0	0/0	0/1	0/0	0/0	0/0	0/0
Chromosome1-1051563	DEL	PRECISE	2281	0	0	0/0	0/0	0/0	0/0	0/0	0/0	0/0	0/0	0/0	0/0	0/0	0/0	0/0	0/1
Chromosome1-1051849	DEL	PRECISE	95	0	0	0/0	0/0	0/0	0/0	0/0	0/0	0/0	0/0	0/0	0/0	0/0	0/0	0/0	0/1
Chromosome1-1052450	INS	PRECISE	295	0	0	0/0	0/0	0/0	0/0	0/0	0/0	0/0	0/0	0/0	0/0	0/0	0/0	0/0	0/1
Chromosome1-1052529	INS	PRECISE	62	0	0	0/0	0/0	0/0	0/0	0/0	0/0	0/0	0/0	0/1	0/0	0/1	0/0	0/1	0/0
Chromosome1-1052724	INS	PRECISE	888	0	0.5	0/0	0/0	0/0	0/0	0/0	0/0	0/0	0/0	0/1	0/0	0/1	0/1	0/1	0/0
Chromosome1-1053328	INS	PRECISE	58	0	0	0/0	0/0	0/0	0/0	0/0	0/0	0/0	0/0	0/1	0/0	0/0	0/0	0/0	0/0
Chromosome1-1054467	INS	PRECISE	211	0	0	0/0	0/0	0/0	0/0	0/0	0/0	0/0	0/0	0/0	0/0	0/0	0/0	0/0	0/1
Chromosome1-1054776	DEL	PRECISE	89	0	0	0/0	0/0	0/0	0/0	0/0	0/0	0/0	0/0	0/1	0/0	0/0	0/0	0/0	0/1
Chromosome1-1054828	DEL	PRECISE	15569	0	0	0/0	0/0	0/0	0/0	0/0	0/0	0/0	0/0	0/1	0/0	1/1	0/1	0/1	0/0
Chromosome1-1054927	DEL	PRECISE	12592	0	0	0/0	0/1	0/1	1/1	1/1	1/1	0/1	0/1	1/1	0/1	0/0	0/0	0/0	1/1
Chromosome1-1057819	INS	PRECISE	1547	0	0	0/0	0/0	0/0	0/0	0/0	0/0	0/0	0/0	0/1	0/0	0/0	0/0	0/0	0/0
Chromosome1-1057835	DEL	PRECISE	1157	0	0	0/0	0/0	0/0	0/0	0/0	0/0	0/0	0/0	0/1	0/0	0/1	0/1	0/1	0/0

Chromosome1-1058685	INS	PRECISE	10101	0	0	0/0	0/0	0/0	0/0	0/0	0/0	0/0	0/0	0/0	0/0	0/0	0/0	0/0	1/1
Chromosome1-1058831	DEL	PRECISE	57	0	0	0/0	0/0	0/0	0/0	0/0	0/0	0/0	0/0	0/1	0/0	0/0	0/1	0/1	0/0
Chromosome1-1059195	INS	PRECISE	1363	0	0.577	0/0	0/0	0/0	0/0	0/0	0/0	0/0	0/0	0/0	0/0	1/1	0/1	1/1	0/0
Chromosome1-1059841	BND	PRECISE	0	0	0	0/0	0/0	0/0	0/0	0/0	0/0	0/0	0/0	0/0	0/1	0/0	0/0	0/0	0/0
Chromosome1-1059841	BND	PRECISE	0	0	0	0/0	0/1	0/0	0/0	0/1	0/1	0/0	0/0	0/0	0/0	0/0	0/0	0/0	0/0
Chromosome1-1059861	INS	PRECISE	1305	0	0	0/0	0/0	0/0	0/0	0/0	0/0	0/0	0/0	0/0	0/0	0/0	0/0	0/0	0/1
Chromosome1-1060016	INS	PRECISE	158	0	0.408	0/0	0/1	0/1	0/0	0/1	0/1	0/0	0/0	0/0	0/1	0/0	0/0	0/0	0/1
Chromosome1-1060047	DEL	PRECISE	673	0	0	0/0	0/0	0/0	0/0	0/0	0/0	0/0	0/0	0/1	0/0	1/1	0/1	1/1	0/0
Chromosome1-1060151	INS	PRECISE	279	0	0.577	0/0	0/0	0/0	0/0	0/0	0/0	0/0	0/0	0/0	0/0	0/0	0/0	0/0	0/1
Chromosome1-1060550	DEL	PRECISE	5870	0	0	0/0	0/0	0/0	0/0	0/0	0/0	0/0	0/0	1/1	0/0	0/1	0/1	0/1	0/0
Chromosome1-1060789	DEL	PRECISE	5371	0	0	0/0	0/0	0/0	0/0	0/0	0/0	0/0	0/0	0/0	0/0	0/0	0/0	0/0	0/1
Chromosome1-1061472	INS	PRECISE	5953	0	0	0/0	0/0	0/0	0/0	0/0	0/0	0/0	0/0	0/0	0/0	0/0	0/0	0/0	1/1
Chromosome1-1061562	INS	PRECISE	426	0	0.5	0/0	0/0	0/0	0/0	0/0	0/0	0/0	0/0	0/1	0/0	0/1	0/0	0/1	0/0
Chromosome1-1062008	DEL	PRECISE	73	0	0	0/0	0/0	0/0	0/1	0/0	0/0	0/1	0/1	0/0	0/0	0/0	0/0	0/0	0/0
Chromosome1-1063078	DEL	PRECISE	2223	0	0	0/0	0/0	0/0	0/0	0/0	0/0	0/0	0/0	0/0	0/0	0/1	0/1	0/1	0/0
Chromosome1-1063079	DEL	PRECISE	12819	0	0	0/0	0/0	0/0	0/0	0/0	0/0	0/0	0/0	0/1	0/0	0/0	0/0	0/0	0/0
Chromosome1-1063449	DEL	PRECISE	617	0	0	0/0	0/1	0/1	0/0	0/1	0/1	0/0	0/0	0/0	0/1	0/0	0/0	0/0	0/1
Chromosome1-1063703	BND	PRECISE	0	0.408	0	0/0	0/0	0/0	0/0	0/0	0/0	0/0	0/0	0/0	0/0	0/0	0/0	0/0	0/1
Chromosome1-1065936	DEL	PRECISE	793	0	0	0/0	0/0	0/0	0/1	0/1	0/1	0/0	0/0	1/1	0/0	0/0	0/0	0/0	0/0
Chromosome1-1066834	DEL	PRECISE	498	0	0	0/0	0/0	0/0	0/0	0/0	0/0	0/0	0/0	0/0	0/0	0/0	0/0	0/1	0/0
Chromosome1-1069096	DEL	PRECISE	82	0	0	0/0	0/1	0/1	0/0	0/1	0/1	0/0	0/0	0/0	0/1	0/0	0/0	0/0	0/1
Chromosome1-1069409	DEL	PRECISE	498	0	0	0/0	0/0	0/0	0/0	0/0	0/0	0/0	0/0	0/1	0/0	0/0	0/0	0/1	0/0
Chromosome1-1069553	DEL	PRECISE	492	0	0	0/0	0/1	0/1	0/0	0/1	0/1	0/0	0/0	0/0	0/1	0/0	0/0	0/0	0/1
Chromosome1-1069647	DEL	PRECISE	101	0	0	0/0	0/0	0/0	0/0	0/0	0/0	0/0	0/0	0/0	0/0	0/0	0/0	0/0	0/1
Chromosome1-1072228	DEL	PRECISE	103	0	0	0/0	0/0	0/0	0/0	0/0	0/0	0/0	0/0	0/1	0/0	0/1	0/1	0/1	0/0
Chromosome1-1072390	INS	PRECISE	63	0	0.5	0/0	0/0	0/0	0/0	0/0	0/0	0/0	0/0	0/1	0/0	0/1	0/1	0/1	0/0
Chromosome1-1072420	INS	PRECISE	1027	0	0.816	0/0	0/0	0/0	0/0	0/0	0/0	0/0	0/0	0/1	0/0	0/1	0/1	0/1	0/0
Chromosome1-1073115	INS	PRECISE	211	0	0	0/0	0/0	0/0	0/0	0/0	0/0	0/0	0/0	0/1	0/0	0/1	0/1	0/1	0/0
Chromosome1-1075330	DEL	PRECISE	1944	0	0	0/0	0/0	0/0	0/0	0/0	0/0	0/0	0/0	0/1	0/0	0/1	0/1	0/1	0/0
Chromosome1-1075499	INS	PRECISE	57	0	0	0/0	0/1	0/1	0/0	0/1	0/1	0/0	0/0	0/0	0/1	0/0	0/0	0/0	0/1
Chromosome1-1075539	DEL	PRECISE	5470	0	0	0/0	0/0	0/0	0/0	0/0	0/0	0/0	0/0	0/1	0/0	0/1	0/1	0/1	0/0
Chromosome1-1075905	DEL	PRECISE	1380	0	0	0/0	0/0	0/0	0/0	0/0	0/0	0/0	0/0	0/0	0/0	0/0	0/0	0/0	0/1
Chromosome1-1076135	INS	PRECISE	4908	0	0	0/0	0/0	0/0	0/0	0/0	0/0	0/0	0/0	0/0	0/0	0/0	0/0	0/0	0/1
Chromosome1-1076733	BND	PRECISE	0	0.894	0	0/0	0/0	0/0	0/0	0/1	0/0	0/0	0/0	0/0	0/0	0/0	0/0	0/0	0/0
Chromosome1-1076826	INS	PRECISE	216	0	0	0/0	0/0	0/0	0/1	0/1	0/1	0/0	0/0	0/1	0/0	0/0	0/0	0/0	0/0
Chromosome1-1077152	DEL	PRECISE	1628	0	0	0/0	0/0	0/0	0/0	0/0	0/0	0/0	0/0	0/1	0/0	0/1	0/1	0/1	0/0
Chromosome1-1077215	BND	PRECISE	0	0	0	0/0	0/0	0/0	0/0	0/1	0/0	0/0	0/0	0/0	0/0	0/0	0/1	0/0	0/0
Chromosome1-1077454	DEL	PRECISE	182	0	0	0/0	0/1	0/0	0/0	0/1	0/0	0/0	0/0	0/0	0/1	0/1	0/1	0/0	0/1
Chromosome1-1078000	DEL	PRECISE	20164	0	0	0/0	0/0	0/0	0/0	0/0	0/0	0/0	0/0	1/1	0/0	1/1	0/1	1/1	0/0
Chromosome1-1078436	BND	PRECISE	0	0	0	0/0	0/0	0/0	0/0	0/0	0/0	0/0	0/0	0/1	0/0	1/1	0/1	0/0	0/0
Chromosome1-1079101	DEL	PRECISE	64	0	0	0/0	0/1	0/1	0/0	0/1	0/1	0/0	0/0	0/0	0/1	0/0	0/0	0/0	0/1
Chromosome1-1079494	INV	PRECISE	524	0	0	0/0	0/0	0/0	0/0	0/0	0/0	0/0	0/0	0/0	0/0	0/0	0/0	0/0	0/1
Chromosome1-1079841	INS	PRECISE	264	0	0.516	0/0	0/1	0/1	0/0	0/1	0/1	0/0	0/0	0/0	0/1	0/0	0/0	0/0	1/1
Chromosome1-1080308	BND	PRECISE	0	0	0	0/0	0/0	0/0	0/0	0/0	0/0	0/0	0/0	0/0	0/0	0/0	0/0	0/0	0/1
Chromosome1-1080493	BND	PRECISE	0	0.5	0	0/0	0/0	0/0	0/0	0/0	0/0	0/0	0/0	0/1	0/0	0/1	0/0	0/1	0/0
Chromosome1-1080522	INS	PRECISE	359	0	0	0/0	0/0	0/0	0/0	0/0	0/0	0/0	0/0	0/0	0/0	0/0	0/0	0/0	0/1
Chromosome1-1081237	INS	PRECISE	347	0	0	0/0	0/0	0/0	0/0	0/0	0/0	0/0	0/0	0/0	0/0	0/0	0/0	0/0	0/1
Chromosome1-1081409	INS	PRECISE	226	0	0.5	0/0	0/0	0/0	0/0	0/0	0/0	0/0	0/0	0/1	0/0	0/1	0/0	0/1	0/0
Chromosome1-1081627	DEL	PRECISE	547	0	0	0/0	0/0	0/0	0/0	0/0	0/0	0/0	0/0	0/1	0/0	1/1	0/1	0/1	0/0
Chromosome1-1081763	INS	PRECISE	275	0	0.577	0/0	0/0	0/0	0/0	0/0	0/0	0/0	0/0	0/1	0/0	0/1	0/1	0/1	0/0
Chromosome1-1083312	DEL	PRECISE	56	0.5	0.5	0/0	0/0	0/0	0/0	0/0	0/0	0/0	0/0	0/1	0/0	0/1	0/1	0/1	0/0
Chromosome1-1084545	DEL	PRECISE	63	0	0	0/0	0/1	0/1	0/0	0/1	0/1	0/0	0/0	0/0	0/1	0/0	0/0	0/0	0/1
Chromosome1-1084940	DEL	PRECISE	193	0	0	0/0	0/1	0/1	0/0	0/1	0/1	0/0	0/0	0/0	0/1	0/0	0/0	0/0	1/1
Chromosome1-1086002	DEL	PRECISE	3616	0	0	0/0	0/0	0/0	0/0	0/0	0/0	0/0	0/0	0/0	0/0	0/0	0/0	0/0	0/1
Chromosome1-1086165	INS	PRECISE	8165	0	0	0/0	0/0	0/0	0/0	0/0	0/0	0/0	0/0	0/0	0/0	0/0	0/0	0/0	0/0
Chromosome1-1086307	INS	PRECISE	10156	0	0	0/0	0/0	0/0	0/0	0/0	0/0	0/0	0/0	0/0	0/0	0/0	0/0	0/0	0/0
Chromosome1-1087800	DEL	PRECISE	56	0	0	0/0	0/0	0/0	0/0	0/0	0/0	0/0	0/0	0/0	0/0	0/0	0/0	0/0	0/1
Chromosome1-1087945	INS	PRECISE	839	0	0.816	0/0	0/0	0/0	0/0	0/0	0/0	0/0	0/0	0/0	0/0	0/1	0/1	0/1	0/0
Chromosome1-1088042	INS	PRECISE	14322	0	0	0/0	0/0	0/0	0/0	0/0	0/0	0/0	0/0	0/1	0/0	0/0	0/0	0/0	0/0
Chromosome1-1088400	INS	PRECISE	135	0	0	0/0	0/0	0/0	0/0	0/0	0/0	0/0	0/0	0/1	0/0	0/1	0/1	0/1	0/0
Chromosome1-1088722	INS	PRECISE	91	0	0.5	0/0	0/0	0/0	0/0	0/0	0/0	0/0	0/0	0/1	0/0	0/1	0/1	0/1	0/0
Chromosome1-1089001	INS	PRECISE	469	0	0	0/0	0/0	0/0	0/0	0/0	0/0	0/0	0/0	0/0	0/0	0/0	0/0	0/0	0/1
Chromosome1-1089329	INS	PRECISE	53	0	0	0/0	0/0	0/0	0/0	0/0	0/0	0/0	0/0	0/0	0/0	0/1	0/1	0/1	0/0
Chromosome1-1089514	DEL	PRECISE	3393	0	0	0/0	0/1	0/1	0/0	0/1	0/1	0/0	0/0	0/1	0/1	0/1	0/1	0/1	1/1
Chromosome1-1089867	DEL	PRECISE	431	0	0	0/0	0/0	0/0	0/0	0/0	0/0	0/0	0/0	0/0	0/0	0/0	0/0	0/0	0/1
Chromosome1-1089987	DEL	PRECISE	5879	0	0	0/0	0/0	0/0	0/0	0/0	0/0	0/0	0/0	0/0	0/0	0/0	0/0	0/0	0/1
Chromosome1-1090470	INS	PRECISE	1469	0	0.5	0/0	0/0	0/0	0/0	0/0	0/0	0/0	0/0	0/1	0/0	0/1	0/1	0/1	0/0
Chromosome1-1090643	DEL	PRECISE	79	0	0	0/0	0/0	0/0	0/0	0/0	0/0	0/0	0/0	0/1	0/0	0/1	0/1	0/1	0/0
Chromosome1-1090775	DEL	PRECISE	5352	0	0	0/0	0/0	0/0	0/0	0/0	0/0	0/0	0/0	0/0	0/0	0/0	0/0	0/0	0/1
Chromosome1-1091431	INS	PRECISE	57	0	0	0/0	0/0	0/0	0/1	0/0	0/0	0/1	0/1	0/0	0/0	0/0	0/0	0/0	0/0
Chromosome1-1092099	DEL	PRECISE	2025	0	0	0/0	0/0	0/0	0/0	0/0	0/0	0/0	0/0	0/0	0/0	0/1	0/0	0/1	0/0
Chromosome1-1092986	DEL	PRECISE	12341	0	0	0/0	0/0	0/0	0/0	0/0	0/0	0/0	0/0	0/0	0/0	0/1	0/1	0/1	0/0
Chromosome1-1094791	DEL	PRECISE	440	0	0	0/0	0/1	0/1	0/0	0/1	0/1	0/0	0/0	0/0	0/1	0/0	0/0	0/0	1/1

Chromosome1-1096054 DEL	PRECISE	425	0	0	0/0	0/0	0/0	0/0	0/0	0/0	0/0	0/0	0/1	0/0	0/1	0/1	0/1	0/0
Chromosome1-1097502 INS	PRECISE	283	0	0	0/0	0/0	0/0	0/1	0/1	0/1	0/0	0/0	1/1	0/0	0/0	0/0	0/0	0/0
Chromosome1-1098932 INS	PRECISE	114	0	0	0/0	0/0	0/0	0/0	0/0	0/0	0/0	0/0	0/1	0/0	0/0	0/0	0/0	0/0
Chromosome1-1099958 DEL	PRECISE	203	0	0	0/0	0/0	0/0	0/0	0/0	0/0	0/0	0/0	0/1	0/0	0/1	0/0	0/1	0/0
Chromosome1-1100218 DEL	PRECISE	93	0	0	0/0	0/0	0/0	0/0	0/0	0/0	0/0	0/0	0/0	0/0	0/0	0/0	0/0	0/1
Chromosome1-1102135 BND	PRECISE	0	0	0	0/0	0/0	0/0	0/0	0/0	0/0	0/0	0/0	0/0	0/0	0/0	0/0	0/0	0/1
Chromosome1-1103136 BND	PRECISE	0	0	0	0/0	0/0	0/0	0/0	0/0	0/0	0/0	0/0	0/1	0/0	0/1	0/0	0/0	0/0
Chromosome1-1103136 BND	PRECISE	0	0	0	0/0	0/0	0/0	0/0	0/0	0/0	0/0	0/0	0/1	0/0	0/0	0/1	0/1	0/0
Chromosome1-1104645 DEL	PRECISE	30543	0	0	0/0	0/0	0/0	0/0	0/0	0/0	0/0	0/0	1/1	0/0	1/1	1/1	1/1	0/0
Chromosome1-1105603 DEL	PRECISE	267	0	0	0/0	0/0	0/0	0/1	0/1	0/1	0/0	0/0	1/1	0/0	0/0	0/0	0/0	0/0
Chromosome1-1105663 DEL	PRECISE	1279	0	0	0/0	0/0	0/0	0/1	0/1	0/1	0/0	0/0	1/1	0/0	0/0	0/0	0/0	0/0
Chromosome1-1105826 INS	PRECISE	55	0	0	0/0	0/1	0/1	0/0	0/1	0/1	0/0	0/0	0/0	0/1	0/0	0/0	0/0	1/1
Chromosome1-1106260 DEL	PRECISE	4143	0	0	0/0	0/0	0/0	0/0	0/0	0/0	0/0	0/0	0/0	0/0	0/0	0/0	0/0	0/1
Chromosome1-1107839 DEL	PRECISE	194	0	0	0/0	0/0	0/0	0/0	0/0	0/0	0/0	0/0	0/1	0/0	0/1	0/1	0/1	0/0
Chromosome1-1107957 DEL	PRECISE	276	0	0	0/0	0/0	0/0	0/0	0/0	0/0	0/0	0/0	0/1	0/0	0/1	0/1	0/1	0/0
Chromosome1-1108476 INS	PRECISE	323	0	0.577	0/0	0/0	0/0	0/0	0/0	0/0	0/0	0/0	0/1	0/0	0/1	0/1	0/1	0/0
Chromosome1-1108680 DEL	PRECISE	10320	0	0	0/0	0/0	0/0	0/1	0/0	0/0	0/0	0/0	0/0	0/0	0/0	0/0	0/0	0/0
Chromosome1-1108954 DEL	PRECISE	1562	0	0	0/0	0/0	0/0	0/0	0/0	0/0	0/0	0/0	0/0	0/0	0/0	0/0	0/0	0/1
Chromosome1-1110079 DEL	PRECISE	12301	0	0	0/0	0/1	0/1	1/1	1/1	1/1	0/1	0/1	1/1	0/1	0/0	0/0	0/0	1/1
Chromosome1-1111309 INV	PRECISE	8498614	0	0	0/0	0/0	0/0	0/0	0/0	0/0	0/1	0/0	0/0	0/0	0/0	0/0	0/0	0/0
Chromosome1-1112135 INS	PRECISE	51	0	0.408	0/0	0/1	0/1	0/0	0/1	0/1	0/0	0/0	0/0	0/1	0/0	0/0	0/0	0/1
Chromosome1-1112494 DEL	PRECISE	105	0	0	0/0	0/1	0/1	0/1	1/1	1/1	0/0	0/0	1/1	0/1	0/0	0/0	0/0	1/1
Chromosome1-1112710 INS	PRECISE	5928	0	0	0/0	0/0	0/0	0/0	0/0	0/0	0/0	0/0	0/0	0/0	0/0	0/0	0/0	1/1
Chromosome1-1113877 DEL	PRECISE	2063	0.5	0.5	0/0	0/0	0/0	0/0	0/0	0/0	0/0	0/0	0/1	0/0	0/0	0/0	0/0	0/0
Chromosome1-1114147 DEL	PRECISE	1481	0	0	0/0	0/0	0/0	0/0	0/0	0/0	0/0	0/0	0/1	0/0	0/1	0/1	0/1	0/0
Chromosome1-1114371 DEL	PRECISE	54	0	0	0/0	0/0	0/0	0/1	0/1	0/1	0/0	0/0	0/1	0/0	0/0	0/0	0/0	0/0
Chromosome1-1114766 DEL	PRECISE	74	0	0	0/0	0/0	0/0	0/0	0/0	0/0	0/0	0/0	0/1	0/0	0/0	0/1	0/1	0/0
Chromosome1-1115279 INS	PRECISE	78	0	0.843	0/0	0/1	0/1	0/0	0/1	0/1	0/0	0/0	0/1	0/1	0/1	0/1	0/1	1/1
Chromosome1-1115353 DEL	PRECISE	56	0	0	0/0	0/0	0/0	0/0	0/0	0/0	0/0	0/0	0/1	0/0	0/0	0/1	0/1	0/0
Chromosome1-1116973 DEL	PRECISE	68	0.408	0.408	0/0	0/1	0/1	0/0	0/1	0/1	0/0	0/0	0/0	0/1	0/0	0/0	0/0	0/1
Chromosome1-1118015 BND	PRECISE	0	0	0	0/0	0/0	0/0	0/0	0/0	0/0	0/0	0/0	0/0	0/0	0/0	0/1	0/1	0/0
Chromosome1-1119296 BND	PRECISE	0	0	0	0/0	0/0	0/0	0/0	0/0	0/0	0/0	0/0	0/1	0/0	0/0	0/0	0/0	0/0
Chromosome1-1119677 DEL	PRECISE	12527	0	0	0/0	0/0	0/0	0/1	1/1	0/1	0/0	0/0	0/1	0/0	0/0	0/0	0/0	0/0
Chromosome1-1121248 DEL	PRECISE	96	0	0	0/0	0/0	0/0	0/0	0/0	0/0	0/0	0/0	0/1	0/0	0/1	0/1	0/1	0/0
Chromosome1-1122658 INS	PRECISE	114	0	0	0/0	0/0	0/0	0/0	0/0	0/0	0/0	0/0	0/0	0/0	0/0	0/0	0/1	0/0
Chromosome1-1123306 INS	PRECISE	165	0	0	0/0	0/0	0/0	0/0	0/0	0/0	0/0	0/0	0/0	0/0	0/0	0/0	0/0	0/1
Chromosome1-1124215 INS	PRECISE	1305	0	0.753	0/0	0/1	0/1	0/0	0/1	0/1	0/0	0/0	0/0	0/1	0/0	0/0	0/0	0/1
Chromosome1-1124576 DEL	PRECISE	5087	0	0	0/0	0/0	0/0	0/0	0/0	0/0	0/0	0/0	0/0	0/0	0/1	0/1	0/1	0/0
Chromosome1-1124614 DEL	PRECISE	302	0	0	0/0	0/1	0/1	0/0	0/1	0/1	0/0	0/0	0/0	0/1	0/0	0/0	0/0	1/1
Chromosome1-1124759 DEL	PRECISE	81	0	0	0/0	0/0	0/0	0/0	0/0	0/0	0/0	0/0	0/0	0/0	0/0	0/0	0/0	0/1
Chromosome1-1125110 DEL	PRECISE	605	0	0	0/0	0/0	0/0	0/0	0/0	0/0	0/0	0/0	0/0	0/0	0/1	0/1	0/1	0/0
Chromosome1-1126844 DEL	PRECISE	826	0	0	0/0	0/0	0/0	0/0	0/0	0/0	0/0	0/0	0/1	0/0	0/1	0/1	0/1	0/0
Chromosome1-1127181 INS	PRECISE	9760	0	0	0/0	0/0	0/0	0/0	0/0	0/0	0/0	0/0	0/0	0/0	0/0	0/0	0/0	0/1
Chromosome1-1128057 DEL	PRECISE	66	0	0	0/0	0/0	0/0	0/0	0/0	0/0	0/0	0/0	0/0	0/0	0/1	0/1	0/1	0/0
Chromosome1-1129905 INS	PRECISE	84	0	0	0/0	0/1	0/1	0/0	0/1	0/1	0/0	0/0	0/0	0/1	0/0	0/0	0/0	0/1
Chromosome1-1130712 DEL	PRECISE	7038	0	0	0/0	0/0	0/0	0/0	0/0	0/0	0/0	0/0	0/0	0/0	0/0	0/0	0/0	0/1
Chromosome1-1131085 DEL	PRECISE	7304	0	0	0/0	0/0	0/0	0/1	0/1	0/1	0/0	0/0	0/1	0/0	0/0	0/0	0/0	0/0
Chromosome1-1132654 INS	PRECISE	8438	0	0	0/0	0/0	0/0	0/0	0/0	0/0	0/0	0/0	0/0	0/0	0/0	0/0	0/0	0/0
Chromosome1-1133100 INS	PRECISE	5893	0	0	0/0	0/0	0/0	0/0	0/0	0/0	0/0	0/0	0/0	0/0	0/0	0/0	0/0	0/1
Chromosome1-1133106 DEL	PRECISE	129	0	0	0/0	0/1	0/1	0/0	0/1	0/1	0/0	0/0	0/1	0/1	0/0	0/1	0/1	0/1
Chromosome1-1133124 INS	PRECISE	137	0	0.408	0/0	0/1	0/1	0/0	0/1	0/1	0/0	0/0	0/0	0/1	0/0	0/0	0/0	0/1
Chromosome1-1133300 DEL	PRECISE	87	0	0	0/0	0/0	0/0	0/0	0/0	0/0	0/0	0/0	0/1	0/0	0/0	0/1	0/1	0/0
Chromosome1-1134220 INS	PRECISE	191	0	0.5	0/0	0/0	0/0	0/0	0/0	0/0	0/0	0/0	0/1	0/0	0/1	0/1	0/1	0/0
Chromosome1-1135228 INS	PRECISE	137	0.577	0.577	0/0	0/0	0/0	0/1	0/0	0/0	0/1	0/1	0/0	0/0	0/0	0/0	0/0	0/0
Chromosome1-1135321 INV	PRECISE	4976249	0	0	0/0	0/0	0/0	0/0	0/0	0/0	0/0	0/0	0/0	0/0	0/1	0/1	0/1	0/0
Chromosome1-1135374 INS	PRECISE	874	0	0.983	0/0	0/1	0/1	0/0	0/1	0/1	0/0	0/0	0/0	0/1	0/0	0/0	0/0	0/1
Chromosome1-1135549 DEL	PRECISE	1172	0	0	0/0	0/0	0/0	0/0	0/0	0/0	0/0	0/0	0/1	0/0	0/1	0/1	0/1	0/0
Chromosome1-1135670 DEL	PRECISE	426	0	0	0/0	0/0	0/0	0/0	0/0	0/0	0/0	0/0	0/1	0/0	0/1	0/1	0/1	0/0
Chromosome1-1135802 BND	PRECISE	0	0	0	0/0	0/1	0/1	0/0	0/1	0/0	0/0	0/0	0/0	0/1	0/0	0/0	0/0	0/0
Chromosome1-1136159 DEL	PRECISE	1480	0	0	0/0	0/0	0/0	0/0	0/0	0/0	0/0	0/0	0/1	0/0	0/1	0/1	0/1	0/0
Chromosome1-1136306 DEL	PRECISE	77	0	0	0/0	0/0	0/0	0/0	0/0	0/0	0/0	0/0	0/0	0/0	0/0	0/0	0/0	0/1
Chromosome1-1136577 DEL	PRECISE	82	0	0	0/0	0/0	0/0	0/0	0/0	0/0	0/0	0/0	0/1	0/0	0/1	0/1	0/1	0/0
Chromosome1-1138551 DEL	PRECISE	6341	0	0	0/0	0/0	0/0	0/0	0/0	0/0	0/0	0/0	0/1	0/0	1/1	1/1	1/1	0/0
Chromosome1-1139291 BND	PRECISE	0	0	0	0/0	0/0	0/0	0/0	0/0	0/0	0/0	0/0	0/0	0/0	1/1	1/1	0/1	0/0
Chromosome1-1139723 DEL	PRECISE	13596	0	0	0/0	0/1	0/1	1/1	1/1	1/1	0/1	1/1	1/1	1/1	0/0	0/0	0/0	1/1
Chromosome1-1140206 INS	PRECISE	2168	0	0.707	0/0	0/0	0/0	0/0	0/0	0/0	0/0	0/0	0/0	0/0	0/0	0/0	0/0	0/1
Chromosome1-1140349 DEL	PRECISE	2777	0	0	0/0	0/0	0/0	0/0	0/0	0/0	0/0	0/0	0/0	0/0	0/0	0/0	0/0	0/1
Chromosome1-1140640 DEL	PRECISE	1633	0	0	0/0	0/0	0/0	0/0	0/0	0/0	0/0	0/0	0/0	0/0	0/0	0/0	0/0	0/1
Chromosome1-1141082 BND	PRECISE	0	0	0	0/0	0/0	0/0	0/1	0/0	0/0	0/0	0/0	0/0	0/0	0/0	0/0	0/0	0/0
Chromosome1-1141815 DEL	PRECISE	5035	0	0	0/0	0/1	0/1	1/1	1/1	1/1	0/1	0/1	1/1	0/1	0/0	0/0	0/0	1/1
Chromosome1-1142400 BND	PRECISE	0	0	0	0/0	0/0	0/0	0/0	0/0	0/0	0/0	0/0	0/1	0/0	0/0	0/0	0/0	0/0
Chromosome1-1142526 DEL	PRECISE	5177	0	0	0/0	0/1	0/1	0/0	0/1	0/1	0/0	0/0	0/1	0/1	0/1	0/1	0/1	1/1
Chromosome1-1143845 INS	PRECISE	92	0	0	0/0	0/0	0/0	0/0	0/0	0/0	0/0	0/0	0/1	0/0	0/1	0/1	0/1	0/0

Chromosome1-1143944	DEL	PRECISE	914	0	0	0/0	0/1	0/1	0/0	0/1	0/1	0/0	0/0	0/1	0/1	0/1	0/1	0/1	1/1
Chromosome1-1144925	DEL	PRECISE	956	0.5	0.5	0/0	0/0	0/0	0/0	0/0	0/0	0/0	0/0	0/1	0/0	0/1	0/1	0/0	0/0
Chromosome1-1145067	DEL	PRECISE	5900	0	0	0/0	0/0	0/0	0/0	0/0	0/0	0/0	0/0	0/1	0/0	0/1	0/1	0/0	0/0
Chromosome1-1145069	DEL	PRECISE	86	0	0	0/0	0/1	0/1	0/0	0/1	0/1	0/0	0/0	0/0	0/1	0/0	0/0	0/0	0/1
Chromosome1-1145771	DEL	PRECISE	363	0	0	0/0	0/1	0/1	0/0	0/1	0/1	0/0	0/0	0/1	0/1	0/1	0/1	0/0	1/1
Chromosome1-1145864	INS	PRECISE	159	0	0.5	0/0	0/0	0/0	0/0	0/0	0/0	0/0	0/0	0/1	0/0	0/1	0/1	0/0	0/0
Chromosome1-1146060	BND	PRECISE	0	0	0	0/0	0/0	0/0	0/0	0/0	0/0	0/0	0/0	0/0	0/0	0/1	0/0	0/0	0/0
Chromosome1-1146859	INS	PRECISE	4303	0	0	0/0	0/0	0/0	0/0	0/0	0/0	0/0	0/0	0/0	0/0	0/0	0/0	0/0	1/1
Chromosome1-1146859	BND	PRECISE	0	0	0	0/0	0/0	0/0	0/0	0/0	0/0	0/0	0/0	0/0	0/0	0/0	0/0	0/0	0/1
Chromosome1-1147852	DEL	PRECISE	615	0	0	0/0	0/0	0/0	0/0	0/0	0/0	0/0	0/0	0/1	0/0	0/1	0/1	0/1	0/0
Chromosome1-1148286	INS	PRECISE	93	0	0	0/0	0/0	0/0	0/0	0/0	0/0	0/0	0/0	0/1	0/0	0/0	0/0	0/0	0/0
Chromosome1-1148348	INS	PRECISE	1299	0	0	0/0	0/0	0/0	0/0	0/0	0/0	0/0	0/0	0/1	0/0	0/0	0/1	0/1	0/0
Chromosome1-1148571	INS	PRECISE	1522	0	0	0/0	0/0	0/0	0/0	0/0	0/0	0/0	0/0	0/0	0/0	0/0	0/0	0/0	0/1
Chromosome1-1148836	DEL	PRECISE	79	0	0	0/0	0/0	0/0	0/0	0/0	0/0	0/0	0/0	0/0	0/0	0/0	0/0	0/0	0/1
Chromosome1-1149188	DEL	PRECISE	556	0	0	0/0	0/0	0/0	0/0	0/0	0/0	0/0	0/0	0/1	0/0	0/1	0/1	0/1	0/0
Chromosome1-1149220	INS	PRECISE	67	0	0.548	0/0	0/1	0/1	0/0	0/1	0/1	0/0	0/0	0/0	0/1	0/0	0/0	0/0	1/1
Chromosome1-1149436	BND	PRECISE	0	0	0	0/0	0/0	0/0	0/0	0/0	0/0	0/0	0/0	0/0	0/0	0/1	0/1	0/1	0/0
Chromosome1-1149545	DEL	PRECISE	994	0	0	0/0	0/0	0/0	1/1	0/1	0/1	0/1	0/1	0/1	0/0	0/0	0/0	0/0	0/0
Chromosome1-1150570	DEL	PRECISE	5762	0	0	0/0	0/0	0/0	0/0	0/0	0/0	0/0	0/0	0/0	0/0	0/0	0/0	0/0	0/1
Chromosome1-1152811	INS	PRECISE	7348	0	0	0/0	0/0	0/0	0/0	0/0	0/0	0/0	0/0	0/0	0/0	0/0	0/0	0/0	1/1
Chromosome1-1153287	DEL	PRECISE	2074	0	0	0/0	0/1	0/1	0/0	0/1	0/1	0/0	0/0	0/1	0/1	0/1	0/1	0/1	1/1
Chromosome1-1153535	DEL	PRECISE	9680	0	0	0/0	0/0	0/0	0/0	0/0	0/0	0/0	0/0	1/1	0/0	0/1	1/1	1/1	0/0
Chromosome1-1153612	DEL	PRECISE	525	0	0	0/0	0/1	0/1	0/0	0/1	0/1	0/0	0/0	0/0	0/1	0/0	0/0	0/0	0/1
Chromosome1-1153666	DEL	PRECISE	3171	0	0	0/0	0/1	0/1	0/0	0/1	0/1	0/0	0/0	0/0	0/1	0/0	0/0	0/0	0/1
Chromosome1-1154034	DEL	PRECISE	4183	0	0	0/0	0/0	0/0	0/0	0/0	0/0	0/0	0/0	0/0	0/0	0/0	0/0	0/0	0/1
Chromosome1-1159799	DEL	PRECISE	4609	0	0	0/0	0/0	0/0	0/0	0/0	0/0	0/0	0/0	0/1	0/0	0/1	0/1	0/1	0/0
Chromosome1-1159966	INS	PRECISE	92	0	0	0/0	0/1	0/1	0/0	0/1	0/1	0/0	0/0	0/0	0/1	0/0	0/0	0/0	1/1
Chromosome1-1160370	INS	PRECISE	626	0	0	0/0	0/0	0/0	0/0	0/0	0/0	0/0	0/0	0/0	0/0	0/0	0/0	0/0	0/1
Chromosome1-1160450	DEL	PRECISE	57	0	0	0/0	0/0	0/0	0/0	0/0	0/0	0/0	0/0	0/1	0/0	0/1	0/1	0/1	0/0
Chromosome1-1161603	DEL	PRECISE	11502	0	0	0/0	0/1	0/1	0/0	0/1	0/1	0/0	0/0	0/0	0/1	0/0	0/0	0/0	0/1
Chromosome1-1162044	INS	PRECISE	1318	0	0.816	0/0	0/0	0/0	0/0	0/0	0/0	0/0	0/0	0/1	0/0	0/1	0/1	0/1	0/0
Chromosome1-1162705	DEL	PRECISE	3313	0	0	0/0	0/0	0/0	0/0	0/0	0/0	0/0	0/0	1/1	0/0	0/1	0/0	0/1	0/0
Chromosome1-1163367	DEL	PRECISE	77	0.5	0.5	0/0	0/0	0/0	0/0	0/0	0/0	0/0	0/0	1/1	0/0	0/1	0/1	0/1	0/0
Chromosome1-1164682	BND	PRECISE	0	0	0	0/0	0/0	0/0	0/0	0/0	0/0	0/0	0/0	0/1	0/0	0/0	0/1	0/1	0/0
Chromosome1-1164682	BND	PRECISE	0	0	0	0/0	0/0	0/0	0/0	0/0	0/0	0/0	0/0	0/1	0/0	0/1	0/0	0/0	0/0
Chromosome1-1164813	BND	PRECISE	0	0	0	0/0	0/0	0/0	0/0	0/0	0/0	0/0	0/0	0/1	0/0	0/0	0/0	0/0	0/0
Chromosome1-1165197	DEL	PRECISE	284	0	0	0/0	0/0	0/0	0/0	0/0	0/0	0/0	0/0	0/1	0/0	0/1	1/1	0/1	0/0
Chromosome1-1165938	DEL	PRECISE	5486	0.816	0.816	0/0	0/1	0/1	0/0	0/1	0/1	0/0	0/0	0/0	0/1	0/0	0/0	0/0	0/1
Chromosome1-1166283	INS	PRECISE	2843	0	0	0/0	0/0	0/0	0/0	0/0	0/0	0/0	0/0	0/0	0/0	0/0	0/0	0/0	0/0
Chromosome1-1166375	DEL	PRECISE	130	0	0	0/0	0/0	0/0	0/0	0/0	0/0	0/0	0/0	0/1	0/0	0/1	0/1	0/1	0/0
Chromosome1-1166885	DEL	PRECISE	2351	0	0	0/0	0/1	0/1	0/0	0/1	0/1	0/0	0/0	0/0	0/1	0/0	0/0	0/0	0/1
Chromosome1-1167229	DEL	PRECISE	452	0	0	0/0	0/1	0/1	0/0	0/1	0/1	0/0	0/0	0/1	0/1	0/1	0/1	0/1	1/1
Chromosome1-1167531	DEL	PRECISE	679	0	0	0/0	0/1	0/1	0/0	0/1	0/1	0/0	0/0	0/1	0/1	0/1	0/1	0/1	1/1
Chromosome1-1169999	INS	PRECISE	1345	0	0.894	0/0	0/1	0/1	0/0	0/1	0/1	0/0	0/0	0/0	0/1	0/0	0/0	0/0	0/1
Chromosome1-1170081	INS	PRECISE	279	0	0.277	0/0	0/1	0/1	1/1	1/1	1/1	0/1	0/1	1/1	0/1	0/1	0/1	0/1	1/1
Chromosome1-1171690	BND	PRECISE	0	0	0	0/0	0/0	0/0	0/0	0/0	0/0	1/1	1/1	0/0	0/0	0/0	0/0	0/0	0/0
Chromosome1-1171879	DEL	PRECISE	41153	0	0	0/0	0/0	0/0	1/1	1/1	1/1	0/0	0/0	1/1	0/0	0/0	0/0	0/0	0/0
Chromosome1-1171957	BND	PRECISE	0	0	0	0/0	0/0	0/0	0/0	0/0	0/0	0/0	0/0	1/1	0/0	0/1	0/0	0/1	0/0
Chromosome1-1172427	INS	PRECISE	84	0	0	0/0	0/0	0/0	0/0	0/0	0/0	0/0	0/0	0/0	0/0	0/0	0/0	0/0	0/1
Chromosome1-1172789	DEL	PRECISE	2701	0	0	0/0	0/0	0/0	0/0	0/0	0/0	0/0	0/0	0/1	0/0	0/0	0/0	0/0	0/0
Chromosome1-1172815	INS	PRECISE	75	0	0	0/0	0/0	0/0	0/0	0/0	0/0	0/0	0/0	0/0	0/0	0/0	0/0	0/0	0/1
Chromosome1-1173184	DEL	PRECISE	2376	0	0	0/0	0/0	0/0	0/0	0/0	0/0	0/0	0/0	1/1	0/0	0/0	0/0	0/0	0/0
Chromosome1-1174084	DEL	PRECISE	4140	0	0	0/0	0/0	0/0	0/0	0/0	0/0	0/0	0/0	1/1	0/0	0/1	0/1	0/0	0/0
Chromosome1-1174416	DEL	PRECISE	262	0	0	0/0	0/1	0/1	0/0	1/1	1/1	0/0	0/0	0/0	0/1	0/0	0/0	0/0	0/1
Chromosome1-1174527	DEL	PRECISE	146	0	0	0/0	0/1	0/1	0/0	1/1	1/1	0/0	0/0	0/0	0/1	0/0	0/0	0/0	0/1
Chromosome1-1175533	DEL	PRECISE	7198	0	0	0/0	0/0	0/0	0/0	0/0	0/0	0/0	0/0	0/1	0/0	0/1	0/1	0/1	0/0
Chromosome1-1176391	DEL	PRECISE	55	0	0	0/0	0/0	0/0	0/0	0/0	0/0	0/0	0/0	0/0	0/0	0/0	0/0	0/0	0/1
Chromosome1-1176395	DEL	PRECISE	4031	0	0	0/0	0/0	0/0	0/0	0/0	0/0	0/0	0/0	0/1	0/0	0/1	0/1	0/1	0/0
Chromosome1-1177203	INS	PRECISE	56	0	0	0/0	0/0	0/0	0/0	0/0	0/0	0/0	0/0	0/1	0/0	0/1	0/1	0/1	0/0
Chromosome1-1177289	BND	PRECISE	0	0	0	0/0	0/0	0/0	0/0	0/0	0/0	0/0	0/0	0/0	0/0	0/0	0/1	0/0	0/0
Chromosome1-1177330	INS	PRECISE	54	0	0	0/0	0/1	0/1	0/0	0/1	0/1	0/0	0/0	0/0	0/1	0/0	0/0	0/0	0/1
Chromosome1-1178520	INS	PRECISE	63	0	0	0/0	0/1	0/1	0/0	0/1	0/1	0/0	0/0	0/0	0/1	0/0	0/0	0/0	0/1
Chromosome1-1178520	DEL	PRECISE	57	0	0	0/0	0/0	0/0	0/0	0/0	0/0	0/0	0/0	0/1	0/0	0/1	0/1	0/0	0/0
Chromosome1-1179317	DEL	PRECISE	673	0	0	0/0	0/0	0/0	0/0	0/0	0/0	0/0	0/0	0/1	0/0	0/1	0/1	0/1	0/0
Chromosome1-1180128	BND	PRECISE	0	0	0	0/0	0/0	0/0	0/0	0/0	0/0	0/0	0/0	0/1	0/0	0/1	0/0	0/0	0/0
Chromosome1-1180534	BND	PRECISE	0	0	0	0/0	0/0	0/0	0/0	0/0	0/0	0/0	0/0	0/1	0/0	1/1	1/1	0/1	0/0
Chromosome1-1180851	DEL	PRECISE	73	0	0	0/0	0/0	0/0	0/0	0/0	0/0	0/0	0/0	0/0	0/0	0/0	0/0	0/0	0/1
Chromosome1-1184030	DEL	PRECISE	50	0	0	0/0	0/1	0/1	0/0	0/1	0/1	0/0	0/0	0/0	0/1	0/0	0/0	0/0	0/1
Chromosome1-1184367	DEL	PRECISE	8011	0	0	0/0	0/0	0/0	0/0	0/0	0/0	0/0	0/0	0/1	0/0	1/1	1/1	0/1	0/0
Chromosome1-1184948	DEL	PRECISE	90	0	0	0/0	0/1	0/1	0/0	0/1	0/1	0/0	0/0	0/0	0/1	0/0	0/0	0/0	0/1
Chromosome1-1185307	DEL	PRECISE	460	0	0	0/0	0/0	0/0	0/0	0/0	0/0	0/0	0/0	0/1	0/0	0/1	0/1	0/1	0/0
Chromosome1-1185944	DEL	PRECISE	137	0	0	0/0	0/1	0/1	1/1	1/1	1/1	0/1	0/1	1/1	0/1	0/1	0/1	0/1	1/1
Chromosome1-1186057	DEL	PRECISE	52	0	0	0/0	0/0	0/0	0/0	0/0	0/0	0/0	0/0	0/1	0/0	0/1	0/1	0/1	0/0

Chromosome1-1186635 DEL	PRECISE	14189	0	0	0/0	0/0	0/0	0/0	0/0	0/0	0/0	0/0	0/1	0/0	0/1	0/1	0/1	0/0
Chromosome1-1186775 INS	PRECISE	207	0	0	0/0	0/0	0/0	0/0	0/0	0/0	0/0	0/0	0/0	0/0	0/0	0/0	0/0	0/1
Chromosome1-1187739 INS	PRECISE	55	0	0	0/0	0/0	0/0	0/0	0/0	0/0	0/0	0/0	0/0	0/0	0/0	0/0	0/0	0/1
Chromosome1-1188512 INS	PRECISE	5243	0	0.707	0/0	0/0	0/0	0/0	0/0	0/0	1/1	1/1	0/0	0/0	0/0	0/0	0/0	0/0
Chromosome1-1188908 DEL	PRECISE	1233	0	0	0/0	0/0	0/0	0/0	0/0	0/0	0/0	0/0	0/1	0/0	0/1	0/1	0/1	0/0
Chromosome1-1190314 INS	PRECISE	63	0	0.577	0/0	0/0	0/0	0/1	0/0	0/0	0/1	0/1	0/0	0/0	0/0	0/0	0/0	0/0
Chromosome1-1191014 INS	PRECISE	906	0	0	0/0	0/0	0/0	0/0	0/0	0/0	0/0	0/0	0/0	0/0	0/0	0/0	0/0	0/1
Chromosome1-1191411 DEL	PRECISE	9747	0.5	0.5	0/0	0/0	0/0	0/0	0/0	0/0	0/0	0/0	0/1	0/0	0/1	0/1	0/1	0/0
Chromosome1-1192583 INS	PRECISE	51	0	0	0/0	0/0	0/0	0/0	0/0	0/0	0/0	0/0	0/0	0/0	0/1	0/1	0/1	0/0
Chromosome1-1192944 BND	PRECISE	0	0	0	0/0	0/1	0/0	0/0	0/1	0/1	0/0	0/0	0/0	0/1	1/1	0/1	0/0	0/1
Chromosome1-1194280 BND	PRECISE	0	0	0	0/0	0/1	0/0	0/0	0/0	0/0	0/0	0/0	0/0	0/0	0/0	0/0	0/0	0/0
Chromosome1-1195117 DEL	PRECISE	722	0	0	0/0	0/0	0/0	0/1	0/0	0/0	0/1	1/1	0/0	0/0	0/0	0/0	0/0	0/0
Chromosome1-1195119 INS	PRECISE	1392	0	0	0/0	0/0	0/0	0/0	0/0	0/0	0/0	0/0	0/0	0/0	0/0	0/0	0/0	0/1
Chromosome1-1196637 DEL	PRECISE	925	0	0	0/0	0/1	0/1	0/0	0/1	0/1	0/0	0/0	0/1	0/1	0/1	0/1	0/1	1/1
Chromosome1-1198474 INS	PRECISE	52	0	0.316	0/0	0/1	0/1	0/0	0/1	0/1	0/0	0/0	0/1	0/1	0/1	0/1	0/1	1/1
Chromosome1-1199237 DEL	PRECISE	2501	0	0	0/0	0/0	0/0	0/0	0/0	0/0	0/0	0/0	0/1	0/0	0/1	0/1	0/1	0/0
Chromosome1-1201072 DEL	PRECISE	2237	0	0	0/0	0/0	0/0	0/0	0/0	0/0	0/0	0/0	0/1	0/0	0/1	0/1	0/1	0/0
Chromosome1-1203078 DEL	PRECISE	950	0	0	0/0	0/0	0/0	0/0	0/0	0/0	0/0	0/0	0/1	0/0	0/1	0/0	0/1	0/0
Chromosome1-1204908 DEL	PRECISE	1365	0	0	0/0	0/0	0/0	0/0	0/0	0/0	0/0	0/0	0/1	0/0	0/1	0/1	0/1	0/0
Chromosome1-1205148 DEL	PRECISE	378	0	0	0/0	0/0	0/0	0/0	0/0	0/0	0/0	0/0	0/1	0/0	0/1	0/1	0/1	0/0
Chromosome1-1206523 INS	PRECISE	742	0	0.816	0/0	0/0	0/0	0/0	0/0	0/0	0/0	0/0	0/1	0/0	0/1	0/1	0/1	0/0
Chromosome1-1207048 DEL	PRECISE	130	0	0	0/0	0/0	0/0	0/0	0/0	0/0	0/0	0/0	0/1	0/0	0/1	0/1	0/1	0/0
Chromosome1-1207079 INS	PRECISE	1405	0	0.753	0/0	0/1	0/1	0/0	0/1	0/1	0/0	0/0	0/0	0/1	0/0	0/0	0/0	0/1
Chromosome1-1208831 DEL	PRECISE	55	0	0	0/0	0/1	0/1	0/0	0/1	0/1	0/0	0/0	0/0	0/1	0/0	0/0	0/0	0/1
Chromosome1-1208833 DEL	PRECISE	1031	0	0	0/0	0/0	0/0	0/0	0/0	0/0	0/0	0/0	0/1	0/0	0/1	0/1	0/1	0/0
Chromosome1-1209055 DEL	PRECISE	8504913	0.5	0	0/0	0/0	0/0	0/0	0/0	0/0	0/0	0/0	0/1	0/0	0/0	0/1	0/1	0/0
Chromosome1-1209967 INS	PRECISE	93	0	0	0/0	0/1	0/1	0/0	0/1	0/1	0/0	0/0	0/0	0/1	0/0	0/0	0/0	0/1
Chromosome1-1209986 DEL	PRECISE	227	0	0	0/0	0/0	0/0	0/0	0/0	0/0	0/0	0/0	0/0	0/0	0/0	0/0	0/0	0/1
Chromosome1-1210088 DEL	PRECISE	78	0	0	0/0	0/0	0/0	0/0	0/0	0/0	0/0	0/0	0/1	0/0	0/1	0/1	0/1	0/0
Chromosome1-1210178 DEL	PRECISE	8886	0	0	0/0	0/1	0/1	0/0	1/1	1/1	0/0	0/0	0/0	0/1	0/0	0/0	0/0	1/1
Chromosome1-1210326 DEL	PRECISE	7332	0	0	0/0	0/0	0/0	0/1	0/0	0/0	0/0	0/0	1/1	0/0	0/0	0/0	0/0	0/0
Chromosome1-1210369 DEL	PRECISE	54	0	0	0/0	0/0	0/0	0/1	0/0	0/0	0/1	0/1	0/0	0/0	0/0	0/0	0/0	0/0
Chromosome1-1212108 BND	PRECISE	0	0	0	0/0	0/0	0/0	0/1	0/0	0/1	0/0	0/0	1/1	0/0	0/0	0/0	0/0	0/0
Chromosome1-1212426 BND	PRECISE	0	0	0	0/0	0/0	0/0	0/0	0/0	0/1	0/0	0/0	0/0	0/1	0/0	0/0	0/0	0/1
Chromosome1-1212426 BND	PRECISE	0	0	0	0/0	0/0	0/0	0/0	0/0	0/0	0/0	0/0	0/0	0/0	0/0	0/0	0/0	0/1
Chromosome1-1213414 DEL	PRECISE	3834	0	0	0/0	0/0	0/0	0/0	0/0	0/0	0/0	0/0	0/1	0/0	0/1	0/1	0/1	0/0
Chromosome1-1213414 DEL	PRECISE	59	0	0	0/0	0/0	0/0	0/1	0/1	0/1	0/0	0/0	0/1	0/0	0/0	0/0	0/0	0/0
Chromosome1-1215081 DEL	PRECISE	70	0	0	0/0	0/1	0/1	0/0	0/1	0/1	0/0	0/0	0/0	0/1	0/0	0/0	0/0	0/1
Chromosome1-1215570 INS	PRECISE	9757	0	0	0/0	0/0	0/0	0/0	0/0	0/0	0/0	0/0	0/0	0/0	0/0	0/0	0/0	0/1
Chromosome1-1215570 DUP	PRECISE	2687994	0	0	0/0	0/0	0/0	0/0	0/0	0/0	0/0	0/0	0/0	0/0	0/0	0/0	0/0	0/1
Chromosome1-1215618 INS	PRECISE	66	0	0	0/0	0/0	0/0	0/0	0/0	0/0	0/0	0/0	0/1	0/0	0/0	0/0	0/0	0/0
Chromosome1-1215882 DEL	PRECISE	711	0	0	0/0	0/0	0/0	0/0	0/0	0/0	0/0	0/0	0/1	0/0	0/1	0/1	0/1	0/0
Chromosome1-1215969 DEL	PRECISE	6273	0	0	0/0	0/0	0/0	0/0	0/0	0/0	0/0	0/0	0/1	0/0	0/1	0/1	0/1	0/0
Chromosome1-1216358 DEL	PRECISE	368	0	0	0/0	0/0	0/0	0/0	0/0	0/0	0/1	0/1	0/0	0/0	0/0	0/0	0/0	0/0
Chromosome1-1217014 INS	PRECISE	682	0	0	0/0	0/0	0/0	0/0	0/0	0/0	0/0	0/0	0/0	0/0	0/0	0/0	0/0	0/1
Chromosome1-1217016 DEL	PRECISE	83	0	0	0/0	0/0	0/0	0/0	0/1	0/1	0/0	0/0	0/1	0/0	0/0	0/0	0/0	0/0
Chromosome1-1217416 DEL	PRECISE	1019	0	0	0/0	0/1	0/1	0/0	0/1	0/1	0/0	0/0	0/1	0/1	0/1	1/1	0/1	1/1
Chromosome1-1218476 BND	PRECISE	0	0	0	0/0	0/0	0/0	0/0	0/0	0/0	0/0	0/0	0/1	0/0	0/0	0/0	0/0	0/0
Chromosome1-1218852 INS	PRECISE	3524	0	0	0/0	0/0	0/0	0/0	0/0	0/0	0/0	0/0	0/0	0/0	0/0	0/0	0/0	1/1
Chromosome1-1219275 INS	PRECISE	131	0	0.577	0/0	0/0	0/0	0/0	0/0	0/0	0/0	0/0	0/1	0/0	0/1	0/0	0/1	0/0
Chromosome1-1219933 INS	PRECISE	5434	0	0	0/0	0/0	0/0	0/0	0/0	0/0	0/0	0/0	0/0	0/0	0/0	0/0	0/0	1/1
Chromosome1-1219934 BND	PRECISE	0	0	0	0/0	0/0	0/0	0/0	0/0	0/0	0/0	0/0	0/0	0/0	0/0	0/0	0/0	0/1
Chromosome1-1221061 DEL	PRECISE	51	0	0	0/0	0/0	0/0	0/0	0/0	0/0	0/0	0/0	0/0	0/0	0/0	0/0	0/0	0/1
Chromosome1-1222042 DEL	PRECISE	7727	0	0	0/0	0/0	0/0	0/0	0/0	0/0	0/0	0/0	0/0	0/0	0/1	0/1	0/1	0/0
Chromosome1-1222989 INS	PRECISE	844	0	0	0/0	0/0	0/0	0/0	0/0	0/0	0/0	0/0	0/0	0/0	0/0	0/0	0/0	0/1
Chromosome1-1223174 INS	PRECISE	999	0	0	0/0	0/0	0/0	0/0	0/0	0/0	0/0	0/0	0/0	0/0	0/0	0/0	0/0	0/1
Chromosome1-1226705 INS	PRECISE	57	0	0.408	0/0	0/0	0/1	0/0	0/1	0/0	0/0	0/0	0/0	0/0	0/0	0/0	0/0	0/0
Chromosome1-1226905 BND	PRECISE	0	0	0	0/0	0/0	0/0	0/0	0/0	0/0	0/0	0/0	0/1	0/0	0/0	0/0	0/0	0/0
Chromosome1-1228646 BND	PRECISE	0	0	0	0/0	0/0	0/0	0/0	0/0	0/0	0/0	0/0	0/1	0/0	0/0	0/0	0/1	0/0
Chromosome1-1228822 INS	PRECISE	109	0	0.5	0/0	0/0	0/0	0/0	0/0	0/0	0/0	0/0	0/1	0/0	0/1	0/1	0/1	0/0
Chromosome1-1229823 INS	PRECISE	54	0	0	0/0	0/0	0/0	0/0	0/0	0/0	0/0	0/0	0/1	0/0	0/0	0/0	0/0	0/0
Chromosome1-1230302 DEL	PRECISE	5372	0	0	0/0	0/0	0/0	0/0	0/0	0/0	0/0	0/0	1/1	0/0	1/1	0/1	1/1	0/0
Chromosome1-1230803 INS	PRECISE	82	0	0	0/0	0/0	0/0	0/1	0/1	0/1	0/0	0/0	1/1	0/0	0/0	0/0	0/0	0/0
Chromosome1-1231093 DEL	PRECISE	175	0	0	0/0	0/1	0/0	1/1	1/1	1/1	0/1	0/1	1/1	0/1	0/1	0/1	0/1	1/1
Chromosome1-1231268 INS	PRECISE	174	0	0.738	0/0	0/1	0/0	0/0	0/1	0/1	0/0	0/0	0/1	0/1	0/1	0/1	0/1	1/1
Chromosome1-1231362 INS	PRECISE	462	0	0.5	0/0	0/0	0/0	0/0	0/0	0/0	0/0	0/0	0/1	0/0	0/1	0/1	0/1	0/0
Chromosome1-1231872 DEL	PRECISE	211	0	0	0/0	0/1	0/0	0/0	0/1	0/1	0/0	0/0	0/1	0/1	0/1	0/1	0/1	1/1
Chromosome1-1232149 DEL	PRECISE	65	0	0	0/0	0/0	0/0	0/0	0/0	0/0	0/0	0/0	0/0	0/0	0/0	0/0	0/0	0/1
Chromosome1-1232898 INS	PRECISE	93	0	0	0/0	0/0	0/0	0/0	0/0	0/0	0/0	0/0	0/1	0/0	0/1	0/1	0/1	0/0
Chromosome1-1233079 INS	PRECISE	98	0	0.577	0/0	0/0	0/0	0/1	0/0	0/0	0/1	0/1	0/0	0/0	0/0	0/0	0/0	0/0
Chromosome1-1233178 BND	PRECISE	0	0	0	0/0	0/0	0/0	0/0	0/0	0/0	0/0	0/0	0/0	0/0	0/1	0/0	0/1	0/0
Chromosome1-1233178 BND	PRECISE	0	0	0	0/0	0/0	0/0	0/0	0/0	0/0	0/0	0/0	0/1	0/0	0/0	0/0	0/1	0/0
Chromosome1-1236058 DEL	PRECISE	59	0	0	0/0	0/1	0/1	0/0	0/1	0/1	0/0	0/0	0/0	0/1	0/0	0/0	0/0	0/1

Chromosome1-1236062	INS	PRECISE	257	0	0	0/0	0/0	0/0	0/0	0/0	0/0	0/0	0/0	0/0	0/0	0/0	0/0	0/0	0/1
Chromosome1-1237837	DEL	PRECISE	51	0	0	0/0	0/1	0/1	0/0	0/1	0/1	0/0	0/0	0/0	0/1	0/0	0/0	0/0	1/1
Chromosome1-1238701	INS	PRECISE	1336	0	0	0/0	0/0	0/0	0/0	0/0	0/0	0/0	0/0	0/0	0/0	0/0	0/0	0/0	0/1
Chromosome1-1239150	INS	PRECISE	75	0	0.408	0/0	0/1	0/1	0/0	0/1	0/1	0/0	0/0	0/0	0/1	0/0	0/0	0/0	1/1
Chromosome1-1239583	INS	PRECISE	479	0	0.632	0/0	0/1	0/1	0/0	0/1	0/1	0/0	0/0	0/0	0/0	0/0	0/0	0/0	1/1
Chromosome1-1240157	INS	PRECISE	390	0	0.516	0/0	0/1	0/1	0/0	0/1	0/1	0/0	0/0	0/0	0/1	0/0	0/0	0/0	0/1
Chromosome1-1241082	INS	PRECISE	57	0	0.408	0/0	0/1	0/1	0/0	0/1	0/1	0/0	0/0	0/0	0/1	0/0	0/0	0/0	1/1
Chromosome1-1241428	INS	PRECISE	12436	0	0	0/0	0/0	0/0	0/0	0/0	0/0	0/0	0/0	0/0	0/0	0/0	0/0	0/0	0/1
Chromosome1-1242087	DEL	PRECISE	623	0	0	0/0	0/1	0/1	0/0	0/1	0/1	0/0	0/0	0/0	0/1	0/0	0/0	0/0	0/1
Chromosome1-1242272	INS	PRECISE	1350	0	0	0/0	0/0	0/0	0/0	0/0	0/0	0/0	0/0	0/0	0/0	0/0	0/0	0/0	0/1
Chromosome1-1242616	INS	PRECISE	5811	0	0	0/0	0/0	0/0	0/0	0/0	0/0	0/0	0/0	0/0	0/0	0/0	0/0	0/0	1/1
Chromosome1-1242906	DEL	PRECISE	12395	0	0	0/0	0/0	0/0	0/0	0/0	0/0	0/0	0/0	0/0	0/0	0/0	0/0	0/0	0/1
Chromosome1-1245233	DEL	PRECISE	2985	0	0	0/0	0/0	0/0	0/0	0/0	0/0	0/0	0/0	0/1	0/0	0/1	0/1	0/1	0/0
Chromosome1-1246925	INS	PRECISE	367	0	0.577	0/0	0/0	0/0	0/0	0/0	0/0	0/0	0/0	0/1	0/0	0/0	0/1	0/0	0/0
Chromosome1-1247481	INS	PRECISE	470	0	0.548	0/0	0/1	0/1	0/0	0/1	0/1	0/0	0/0	0/0	0/1	0/0	0/0	0/0	0/1
Chromosome1-1247513	DEL	PRECISE	418	0	0	0/0	0/0	0/0	0/0	0/0	0/0	0/0	0/0	0/1	0/0	0/1	1/1	0/1	0/0
Chromosome1-1248349	INS	PRECISE	65	0	0.408	0/0	0/1	0/1	0/0	0/1	0/1	0/0	0/0	0/0	0/1	0/0	0/0	0/0	1/1
Chromosome1-1249687	DEL	PRECISE	4906	0	0	0/0	0/0	0/0	0/0	0/0	0/0	0/0	0/0	0/1	0/0	0/1	0/1	0/1	0/0
Chromosome1-1250463	INS	PRECISE	132	0	0	0/0	0/0	0/0	0/0	0/0	0/0	0/0	0/0	0/0	0/0	0/0	0/0	0/0	0/1
Chromosome1-1250710	INS	PRECISE	887	0	0	0/0	0/0	0/0	0/0	0/0	0/0	0/0	0/0	0/0	0/0	0/0	0/0	0/0	0/1
Chromosome1-1250958	INS	PRECISE	1629	0	0	0/0	0/0	0/0	0/0	0/0	0/0	0/0	0/0	0/0	0/0	0/0	0/0	0/0	0/1
Chromosome1-1252483	INV	PRECISE	1673774	0	0	0/0	0/0	0/0	0/0	0/1	0/0	0/0	0/0	0/0	0/0	0/0	0/0	0/0	0/0
Chromosome1-1253209	DEL	PRECISE	202	0	0	0/0	0/0	0/0	0/1	1/1	1/1	0/0	0/0	1/1	0/0	0/0	0/0	0/0	0/0
Chromosome1-1253580	DUP	PRECISE	21106	0	0	0/0	0/0	0/0	0/0	0/1	0/1	0/0	0/0	0/0	0/0	0/0	0/0	0/0	0/0
Chromosome1-1253666	INS	PRECISE	14225	0	0	0/0	0/0	0/0	0/0	0/0	0/0	0/0	0/0	0/0	0/0	0/0	0/0	0/0	1/1
Chromosome1-1254638	INS	PRECISE	150	0	0.5	0/0	0/0	0/0	0/0	0/0	0/0	0/0	0/0	0/1	0/0	1/1	0/1	0/1	0/0
Chromosome1-1255854	INS	PRECISE	66	0	0	0/0	0/0	0/0	0/0	0/0	0/0	0/0	0/0	0/0	0/0	0/1	0/0	0/0	0/0
Chromosome1-1256744	DEL	PRECISE	909	0	0	0/0	0/1	0/1	0/0	0/1	0/1	0/0	0/0	0/0	0/1	0/0	0/0	0/0	0/1
Chromosome1-1256838	DEL	PRECISE	1666	0	0	0/0	0/1	0/1	0/0	0/1	0/1	0/0	0/0	0/0	0/1	0/0	0/0	0/0	0/1
Chromosome1-1257728	DEL	PRECISE	695	0	0	0/0	0/0	0/0	0/0	0/0	0/0	0/0	0/0	0/0	0/0	0/0	0/0	0/0	0/1
Chromosome1-1258604	INS	PRECISE	576	0	0.516	0/0	0/1	0/1	0/0	0/1	0/1	0/0	0/0	0/0	0/1	0/0	0/0	0/0	1/1
Chromosome1-1259060	DEL	PRECISE	1326	0	0	0/0	0/0	0/0	0/0	0/0	0/0	0/0	0/0	0/1	0/0	0/1	0/1	0/1	0/0
Chromosome1-1259525	DEL	PRECISE	78	0	0	0/0	0/0	0/0	0/0	0/0	0/0	0/0	0/0	0/0	0/0	0/0	0/0	0/0	0/1
Chromosome1-1259631	DEL	PRECISE	1551	0	0	0/0	0/1	0/1	0/0	0/1	0/1	0/0	0/0	0/1	0/1	0/1	0/1	0/1	1/1
Chromosome1-1260084	DEL	PRECISE	1331	0	0	0/0	0/0	0/0	0/0	0/0	0/0	0/0	0/0	0/1	0/0	0/1	0/1	0/1	0/0
Chromosome1-1260339	DEL	PRECISE	12066	0	0	0/0	0/0	0/0	0/0	0/0	0/0	0/0	0/0	0/1	0/0	0/1	0/1	0/1	0/0
Chromosome1-1260375	INS	PRECISE	530	0	0.894	0/0	0/1	0/1	0/0	0/1	0/1	0/0	0/0	0/0	0/1	0/0	0/0	0/0	0/1
Chromosome1-1261667	DEL	PRECISE	864	0.5	0.5	0/0	0/0	0/0	0/0	0/0	0/0	0/0	0/0	0/1	0/0	0/1	0/1	0/1	0/0
Chromosome1-1262135	INS	PRECISE	150	0	0	0/0	0/0	0/0	0/0	0/0	0/0	0/0	0/0	0/1	0/0	0/1	0/1	0/1	0/0
Chromosome1-1262625	DEL	PRECISE	68	0	0	0/0	0/1	0/1	0/0	0/1	0/1	0/0	0/0	0/0	0/1	0/0	0/0	0/0	0/1
Chromosome1-1262975	INS	PRECISE	8896	0	0	0/0	0/0	0/0	1/1	0/0	0/0	0/0	0/0	0/0	0/0	0/0	0/0	0/0	0/0
Chromosome1-1262976	BND	PRECISE	0	0	0	0/0	0/0	0/0	0/1	0/0	0/0	0/0	0/0	0/0	0/0	0/0	0/0	0/0	0/0
Chromosome1-1263719	DEL	PRECISE	121	0	0	0/0	0/1	0/1	0/0	0/1	0/1	0/0	0/0	0/0	0/1	0/0	0/0	0/0	0/1
Chromosome1-1264514	DEL	PRECISE	59	0	0	0/0	0/1	0/1	0/0	0/1	0/1	0/0	0/0	0/0	0/1	0/0	0/0	0/0	0/1
Chromosome1-1264708	INS	PRECISE	54	0	0	0/0	0/0	0/0	0/0	0/0	0/0	0/0	0/0	0/1	0/0	0/1	0/1	0/1	0/0
Chromosome1-1265878	INS	PRECISE	196	0	0	0/0	0/0	0/0	0/0	0/0	0/0	0/0	0/0	0/1	0/0	0/0	0/0	0/1	0/1
Chromosome1-1265903	BND	PRECISE	0	0	0	0/0	0/0	0/0	0/0	0/0	0/0	0/0	0/0	0/0	0/0	0/1	0/0	0/0	0/0
Chromosome1-1266170	INS	PRECISE	121	0	0	0/0	0/0	0/0	0/0	0/0	0/0	0/0	0/0	0/1	0/0	0/0	0/0	0/0	0/0
Chromosome1-1267792	DEL	PRECISE	22710	0	0	0/0	1/1	1/1	0/0	1/1	1/1	0/0	0/0	0/0	1/1	0/0	0/0	0/0	0/0
Chromosome1-1267792	DEL	PRECISE	27387	0	0	0/0	0/0	0/0	0/0	0/0	0/0	0/0	0/0	0/0	0/0	0/1	0/1	0/0	0/0
Chromosome1-1268220	DEL	PRECISE	12567	0	0	0/0	0/0	0/0	1/1	1/1	1/1	0/1	0/1	1/1	0/0	0/0	0/0	0/0	0/0
Chromosome1-1269753	DEL	PRECISE	146	0	0	0/0	0/0	0/0	0/1	1/1	1/1	0/0	0/0	1/1	0/0	0/0	0/0	0/0	0/0
Chromosome1-1271308	DEL	PRECISE	248	0	0	0/0	0/0	0/0	0/0	0/0	0/0	0/0	0/0	0/1	0/0	0/1	0/1	0/0	0/0
Chromosome1-1272168	BND	PRECISE	0	0	0	0/0	0/0	0/0	0/0	0/0	0/0	0/0	0/0	0/1	0/0	0/1	0/0	0/0	0/0
Chromosome1-1272300	DEL	PRECISE	531	0	0	0/0	0/0	0/0	0/1	0/1	1/1	0/0	0/0	1/1	0/0	0/0	0/0	0/0	0/0
Chromosome1-1272798	INS	PRECISE	103	0	0.408	0/0	0/1	0/1	0/0	0/1	0/1	0/0	0/0	0/0	0/1	0/0	0/0	0/0	1/1
Chromosome1-1272925	INS	PRECISE	54	0	0.5	0/0	0/0	0/0	0/0	0/0	0/0	0/0	0/0	0/1	0/0	0/1	0/1	0/1	0/0
Chromosome1-1273314	DEL	PRECISE	153	0	0	0/0	0/0	0/0	0/1	0/1	0/1	0/0	0/0	0/1	0/0	0/0	0/0	0/0	0/0
Chromosome1-1273566	INS	PRECISE	118	0	0.5	0/0	0/0	0/0	0/0	0/0	0/0	0/0	0/0	0/1	0/0	0/1	0/1	0/1	0/0
Chromosome1-1273656	DEL	PRECISE	1657	0	0	0/0	0/0	0/0	0/0	0/0	0/0	0/0	0/0	0/1	0/0	0/1	0/1	0/1	0/0
Chromosome1-1273838	INS	PRECISE	151	0	0	0/0	0/1	0/1	0/0	0/1	0/1	0/0	0/0	0/0	0/1	0/0	0/0	0/0	0/1
Chromosome1-1274258	DEL	PRECISE	685	0	0	0/0	0/0	0/0	0/0	0/0	0/0	0/0	0/0	0/0	0/0	0/1	0/1	0/1	0/0
Chromosome1-1274851	INS	PRECISE	66	0	0	0/0	0/1	0/1	0/0	0/1	0/1	0/0	0/0	0/0	0/1	0/0	0/0	0/0	1/1
Chromosome1-1275218	DEL	PRECISE	162	0	0	0/0	0/0	0/0	0/0	0/0	0/0	0/0	0/0	0/1	0/0	0/1	0/1	0/1	0/0
Chromosome1-1275346	INS	PRECISE	85	0	0	0/0	0/0	0/0	0/0	0/0	0/0	0/0	0/0	0/1	0/0	0/1	0/1	0/1	0/0
Chromosome1-1275844	DEL	PRECISE	1730	0	0	0/0	0/0	0/0	0/1	0/1	0/1	0/0	0/0	0/1	0/0	0/0	0/0	0/0	0/0
Chromosome1-1276202	DEL	PRECISE	338	0	0	0/0	0/1	0/1	0/0	0/1	0/1	0/0	0/0	0/0	0/1	0/0	0/0	0/0	0/1
Chromosome1-1276507	INS	PRECISE	81	0	0	0/0	0/0	0/0	0/0	0/0	0/0	0/0	0/0	1/1	0/0	0/1	1/1	0/1	0/0
Chromosome1-1278142	DEL	PRECISE	51	0	0	0/0	0/0	0/0	0/1	0/1	0/1	0/0	0/0	0/1	0/0	0/0	0/0	0/0	0/0
Chromosome1-1278203	INS	PRECISE	63	0	0	0/0	0/0	0/0	0/0	0/0	0/0	0/0	0/0	0/1	0/0	0/1	0/1	0/1	0/0
Chromosome1-1278563	DEL	PRECISE	681	0	0	0/0	0/0	0/0	0/0	0/0	0/0	0/0	0/0	0/1	0/0	0/0	0/1	0/0	0/0
Chromosome1-1279304	DEL	PRECISE	53	0	0	0/0	0/0	0/0	0/0	0/0	0/0	0/0	0/0	0/0	0/0	0/0	0/0	0/0	0/1
Chromosome1-1280395	INS	PRECISE	65	0	0	0/0	0/0	0/0	0/0	0/0	0/0	0/0	0/0	0/1	0/0	0/0	0/0	0/0	0/0

Chromosome1-1280436	DEL	PRECISE	50	0	0	0/0	0/0	0/0	0/0	0/0	0/0	0/0	0/0	0/0	0/0	0/0	0/0	0/0	0/1
Chromosome1-1280613	INS	PRECISE	69	0	0	0/0	0/0	0/0	0/1	0/1	0/1	0/0	0/0	0/1	0/0	0/0	0/0	0/0	0/0
Chromosome1-1280633	DEL	PRECISE	1026	0	0	0/0	0/0	0/0	0/0	0/0	0/0	0/0	0/0	0/1	0/0	0/1	0/1	0/1	0/0
Chromosome1-1281790	DEL	PRECISE	3441	0	0	0/0	0/1	0/1	0/0	0/1	0/1	0/0	0/0	0/0	0/1	0/0	0/0	0/0	1/1
Chromosome1-1281875	DEL	PRECISE	2099	0	0	0/0	0/0	0/0	1/1	0/1	0/1	0/1	1/1	1/1	0/0	0/0	0/0	0/0	0/0
Chromosome1-1282401	INS	PRECISE	54	0	0	0/0	0/1	0/1	0/0	0/1	0/1	0/0	0/0	0/0	0/1	0/0	0/0	0/0	0/1
Chromosome1-1283123	BND	PRECISE	0	0	0	0/0	0/0	0/0	0/1	0/0	0/0	0/0	0/0	0/0	0/0	0/0	0/0	0/0	0/0
Chromosome1-1283123	INV	PRECISE	6932258	0	0	0/0	0/0	0/0	0/0	0/0	0/0	0/0	0/0	0/1	0/0	0/1	0/1	0/0	0/0
Chromosome1-1283298	DEL	PRECISE	1589	0.905	0	0/0	0/0	0/0	0/1	0/1	0/0	0/1	0/0	0/1	0/0	0/0	0/0	0/0	0/0
Chromosome1-1283726	INS	PRECISE	12583	0	0	0/0	0/0	0/1	0/0	0/0	0/0	0/0	0/0	0/0	0/0	0/0	0/0	0/0	0/0
Chromosome1-1284114	INV	PRECISE	6922353	0	0	0/0	0/0	0/0	0/0	0/0	0/0	0/0	0/0	0/1	0/0	0/1	0/1	0/0	0/0
Chromosome1-1284114	BND	PRECISE	0	0	0	0/0	0/0	0/0	0/1	0/1	0/0	0/1	0/0	0/0	0/0	0/0	0/0	0/0	0/0
Chromosome1-1284145	INS	PRECISE	227	0	0	0/0	0/0	0/0	0/0	0/0	0/0	0/0	0/0	0/1	0/0	0/1	0/1	0/1	0/0
Chromosome1-1285748	INS	PRECISE	112	0	0	0/0	0/0	0/0	0/0	0/0	0/0	0/0	0/0	0/0	0/0	0/0	0/0	0/0	0/1
Chromosome1-1286177	BND	PRECISE	0	0	0	0/0	0/0	0/0	0/0	0/0	0/0	0/0	0/0	0/0	0/1	0/0	0/0	0/0	0/0
Chromosome1-1287851	BND	PRECISE	0	0	0	0/0	0/0	0/0	0/0	0/0	0/0	0/0	0/0	0/0	0/0	0/1	0/0	0/0	0/0
Chromosome1-1288768	INS	PRECISE	2066	0	0	0/0	0/0	0/0	0/0	0/0	0/0	0/0	0/0	0/0	0/0	0/0	0/0	0/0	0/1
Chromosome1-1289279	DEL	PRECISE	1634	0	0	0/0	0/0	0/0	0/0	0/0	0/0	0/0	0/0	0/0	0/0	0/0	0/0	0/0	0/1
Chromosome1-1290365	DEL	PRECISE	54	0	0	0/0	0/1	0/1	0/0	0/1	0/1	0/0	0/0	0/0	0/1	0/0	0/0	0/0	0/1
Chromosome1-1290657	INS	PRECISE	10121	0	0	0/0	0/0	0/0	0/0	0/0	0/0	0/0	0/0	0/0	0/0	0/0	0/0	0/0	1/1
Chromosome1-1291370	INS	PRECISE	132	0	0.5	0/0	0/0	0/0	0/1	0/1	0/1	0/0	0/0	0/1	0/0	0/0	0/0	0/0	0/0
Chromosome1-1294246	INS	PRECISE	57	0	0	0/0	0/0	0/0	0/0	0/0	0/0	0/0	0/0	0/1	0/0	0/1	0/1	0/1	0/0
Chromosome1-1294690	INS	PRECISE	53	0	0	0/0	0/0	0/0	0/0	0/0	0/0	0/0	0/0	0/1	0/0	0/1	0/1	0/1	0/0
Chromosome1-1295307	DEL	PRECISE	1345	0	0	0/0	0/0	0/0	0/0	0/0	0/0	0/0	0/0	0/1	0/0	0/1	0/1	0/1	0/0
Chromosome1-1296315	INS	PRECISE	60	0	0	0/0	0/0	0/1	0/0	0/0	0/0	0/0	0/0	0/0	0/0	0/0	0/0	0/0	0/0
Chromosome1-1297528	BND	PRECISE	0	0	0	0/0	0/0	0/0	0/1	0/0	0/0	0/1	0/0	0/0	0/0	0/1	0/0	0/1	0/1
Chromosome1-1297733	DEL	PRECISE	12752	0	0	0/0	0/0	0/0	0/0	0/0	0/0	0/0	0/0	1/1	0/0	1/1	0/1	1/1	0/0
Chromosome1-1297777	INV	PRECISE	6204	0	0	0/0	0/0	0/0	0/0	0/0	0/0	0/0	0/0	0/0	0/0	0/0	0/0	0/0	0/1
Chromosome1-1297818	DEL	PRECISE	295	0	0	0/0	0/1	0/1	0/0	0/1	0/1	0/0	0/0	0/0	0/1	0/0	0/0	0/0	1/1
Chromosome1-1298353	INV	PRECISE	447	0	0	0/0	0/0	0/0	0/0	0/0	0/0	0/0	0/0	0/0	0/0	0/0	0/0	0/0	0/1
Chromosome1-1300315	INS	PRECISE	86	0	0	0/0	0/0	0/0	0/0	0/0	0/0	0/0	0/0	0/1	0/0	0/1	0/0	0/1	0/0
Chromosome1-1301811	BND	PRECISE	0	0	0	0/0	0/0	0/0	0/0	0/1	0/0	0/0	0/0	0/0	0/0	0/0	0/0	0/0	0/0
Chromosome1-1302397	DEL	PRECISE	161	0	0	0/0	0/0	0/0	0/0	0/0	0/0	0/0	0/0	0/1	0/0	0/1	0/0	0/1	0/0
Chromosome1-1303638	INS	PRECISE	176	0	0	0/0	0/0	0/0	0/0	0/0	0/0	0/0	0/0	0/1	0/0	0/1	0/0	0/0	0/0
Chromosome1-1303673	INS	PRECISE	7646	0	0	0/0	0/0	0/0	1/1	0/0	0/0	0/0	0/0	0/0	0/0	0/0	0/0	0/0	0/0
Chromosome1-1303830	INS	PRECISE	56	0	0	0/0	0/0	0/0	0/0	0/0	0/0	0/0	0/0	0/1	0/0	0/1	0/0	0/0	0/0
Chromosome1-1303995	INS	PRECISE	131	0	0.816	0/0	0/0	0/0	0/1	0/1	0/1	0/0	0/0	1/1	0/0	0/0	0/0	0/0	0/0
Chromosome1-1305277	DEL	PRECISE	1391	0	0	0/0	0/1	0/1	0/1	1/1	1/1	0/0	0/0	1/1	0/1	0/0	0/0	0/0	1/1
Chromosome1-1305709	DEL	PRECISE	54	0	0	0/0	0/0	0/0	0/0	0/0	0/0	0/0	0/0	0/1	0/0	0/1	0/1	0/1	0/0
Chromosome1-1305775	DEL	PRECISE	796	0.516	0	0/0	0/1	0/1	0/0	0/1	0/1	0/0	0/0	0/1	0/1	0/1	0/1	0/1	1/1
Chromosome1-1306046	DEL	PRECISE	7163	0	0	0/0	0/0	0/0	0/0	0/0	0/0	0/0	0/0	1/1	0/0	1/1	0/1	0/1	0/0
Chromosome1-1306274	DEL	PRECISE	165	0	0	0/0	0/1	0/1	0/0	0/1	0/1	0/0	0/0	0/0	0/1	0/0	0/0	0/0	0/1
Chromosome1-1306619	DEL	PRECISE	895	0	0	0/0	0/1	0/1	0/0	0/1	0/1	0/0	0/0	0/0	0/1	0/0	0/0	0/0	0/1
Chromosome1-1307629	DEL	PRECISE	54	0	0	0/0	0/0	0/1	0/0	0/1	0/1	0/0	0/0	0/0	0/1	0/0	0/0	0/0	0/1
Chromosome1-1308373	INS	PRECISE	6459	0	0	0/0	0/0	0/0	0/0	0/0	0/0	0/0	0/0	0/0	0/0	0/0	0/0	0/0	1/1
Chromosome1-1308973	DEL	PRECISE	69	0	0	0/0	0/1	0/1	0/0	1/1	0/1	0/0	0/0	0/0	0/1	0/0	0/0	0/0	0/1
Chromosome1-1309686	DEL	PRECISE	1757	0	0	0/0	0/1	0/0	0/0	0/1	0/0	0/0	0/0	0/0	0/1	0/0	0/0	0/0	0/1
Chromosome1-1310625	INV	PRECISE	1326	0	0	0/0	0/0	0/0	0/1	0/0	0/0	0/1	0/1	0/0	0/0	0/0	0/0	0/0	0/0
Chromosome1-1310769	DEL	PRECISE	1924	0	0	0/0	0/1	0/1	0/0	0/1	0/0	0/0	0/0	0/0	0/1	0/0	0/0	0/0	0/1
Chromosome1-1310967	DEL	PRECISE	1326	0	0	0/0	0/1	0/1	0/0	0/1	0/0	0/0	0/0	0/0	0/1	0/0	0/0	0/0	0/1
Chromosome1-1311003	INS	PRECISE	85	0	0.408	0/0	0/1	0/0	0/0	0/0	0/1	0/0	0/0	0/0	0/0	0/0	0/0	0/0	0/1
Chromosome1-1311804	INS	PRECISE	658	0	0.632	0/0	0/1	0/1	0/0	0/1	0/1	0/0	0/0	0/0	0/1	0/0	0/0	0/0	1/1
Chromosome1-1315018	DEL	PRECISE	62	0	0	0/0	0/0	0/0	0/0	0/0	0/0	0/0	0/0	0/0	0/0	0/0	0/0	0/0	0/1
Chromosome1-1315328	DEL	PRECISE	165	0	0	0/0	0/0	0/0	0/0	0/0	0/0	0/0	0/0	0/0	0/0	0/0	0/0	0/0	0/1
Chromosome1-1315412	DEL	PRECISE	1161	0	0	0/0	0/0	0/1	0/0	0/0	0/1	0/0	0/0	0/0	0/1	0/0	0/0	0/0	0/1
Chromosome1-1315961	DEL	PRECISE	100	0	0	0/0	0/0	0/0	0/0	0/0	0/0	0/0	0/0	0/0	0/0	0/0	0/0	0/0	0/1
Chromosome1-1316462	DEL	PRECISE	1017	0.408	0.408	0/0	0/1	0/1	0/0	0/0	0/0	0/0	0/0	0/0	0/1	0/0	0/0	0/0	1/1
Chromosome1-1316771	INS	PRECISE	296	0	0	0/0	0/0	0/0	0/0	0/0	0/0	0/0	0/0	0/0	0/0	0/0	0/0	0/0	0/1
Chromosome1-1316900	DEL	PRECISE	94	0	0	0/0	0/0	0/0	0/0	0/0	0/0	0/0	0/0	0/0	0/0	0/0	0/0	0/0	0/1
Chromosome1-1317594	INS	PRECISE	49	0	0.5	0/0	0/0	0/0	0/0	0/0	0/0	0/0	0/0	0/1	0/0	0/1	0/1	0/1	0/0
Chromosome1-1318004	INS	PRECISE	150	0	0	0/0	0/0	0/0	0/0	0/0	0/0	0/0	0/0	0/0	0/0	0/0	0/0	0/0	0/1
Chromosome1-1318889	DEL	PRECISE	15843359	0	0	0/0	0/0	0/1	0/0	0/0	0/0	0/0	0/0	0/0	0/1	0/0	0/0	0/0	0/1
Chromosome1-1319619	INS	PRECISE	9572	0	0	0/0	0/0	0/0	0/0	0/0	0/0	0/0	0/0	0/0	0/0	0/0	0/0	0/0	0/1
Chromosome1-1319737	DEL	PRECISE	59	0	0	0/0	0/0	0/0	0/0	0/0	0/0	0/0	0/0	0/1	0/0	0/1	0/1	0/1	0/0
Chromosome1-1319852	DEL	PRECISE	3742	0	0	0/0	0/0	0/0	0/0	0/0	0/0	0/0	0/0	0/1	0/0	0/1	1/1	1/1	0/0
Chromosome1-1321465	INS	PRECISE	1554	0	0	0/0	0/0	0/0	0/0	0/0	0/0	0/0	0/0	0/0	0/0	0/0	0/0	0/0	0/1
Chromosome1-1322396	DEL	PRECISE	124725	0	0	0/0	0/0	0/0	0/0	0/0	0/0	0/0	0/0	0/1	0/0	0/1	0/1	1/1	0/0
Chromosome1-1323405	DEL	PRECISE	83	0	0	0/0	0/0	0/0	0/0	0/0	0/0	0/0	0/0	0/1	0/0	0/1	0/1	0/1	0/0
Chromosome1-1323911	BND	PRECISE	0	0	0	0/0	0/1	0/1	0/0	0/1	0/0	0/0	0/0	0/0	0/1	0/0	0/0	0/0	0/1
Chromosome1-1323912	BND	PRECISE	0	0	0	0/0	0/1	0/0	0/0	0/0	0/0	0/0	0/0	0/0	0/1	0/0	0/0	0/0	0/0
Chromosome1-1323995	INS	PRECISE	159	0	0.5	0/0	0/0	0/0	0/0	0/0	0/0	0/0	0/0	0/1	0/0	0/1	0/1	0/1	0/0
Chromosome1-1324413	INS	PRECISE	336	0	0.816	0/0	0/0	0/0	0/0	0/0	0/0	0/0	0/0	0/1	0/0	0/1	0/1	0/1	0/0
Chromosome1-1324458	BND	PRECISE	0	0	0	0/0	0/0	0/0	0/0	0/0	0/0	0/0	0/0	0/0	0/1	0/0	0/0	0/0	0/0

Chromosome1-1324460	BND	PRECISE	0	0	0	0/0	0/1	0/1	0/0	0/1	0/0	0/0	0/0	0/0	0/1	0/0	0/0	0/0	0/0
Chromosome1-1324962	DEL	PRECISE	77	0.5	0.5	0/0	0/0	0/0	0/0	0/0	0/0	0/0	0/0	0/1	0/0	0/1	0/1	0/1	0/0
Chromosome1-1325470	DEL	PRECISE	388	0	0	0/0	0/1	0/1	1/1	1/1	1/1	0/1	0/1	1/1	0/1	0/1	0/1	0/1	1/1
Chromosome1-1325929	INS	PRECISE	82	0	0.577	0/0	0/0	0/0	0/0	0/0	0/0	0/0	0/0	0/1	0/0	0/1	0/1	0/1	0/0
Chromosome1-1326215	INS	PRECISE	71	0	0	0/1	0/1	0/1	0/1	0/1	0/1	0/1	0/1	0/1	0/1	0/0	0/1	0/0	0/1
Chromosome1-1326441	DEL	PRECISE	628	0	0	0/0	0/1	0/1	0/0	0/1	0/0	0/0	0/0	0/0	0/1	0/0	0/0	0/0	0/1
Chromosome1-1327016	BND	PRECISE	0	0	0	0/0	0/0	0/0	0/0	0/0	0/0	0/0	0/0	0/1	0/0	0/0	0/0	0/0	0/0
Chromosome1-1330589	DEL	PRECISE	115	0	0	0/0	0/0	0/0	0/0	1/1	1/1	0/1	0/1	0/0	0/0	0/0	0/0	0/0	1/1
Chromosome1-1330738	DEL	PRECISE	1015	0	0	0/0	0/0	0/0	0/0	0/0	0/0	0/0	0/0	0/0	0/0	0/0	0/0	0/0	1/1
Chromosome1-1330985	INS	PRECISE	343	0	0.408	0/0	0/1	0/1	0/0	0/1	0/1	0/0	0/0	0/0	0/1	0/0	0/0	0/0	0/1
Chromosome1-1331222	DEL	PRECISE	7568	0	0	0/0	0/0	0/0	0/0	0/0	0/0	0/0	0/0	0/0	0/0	0/0	0/0	0/0	0/1
Chromosome1-1331318	DEL	PRECISE	614	0	0	0/0	0/1	0/1	0/0	0/1	1/1	0/0	0/0	0/0	0/1	0/0	0/0	0/0	0/1
Chromosome1-1331808	INS	PRECISE	612	0	0.816	0/0	0/0	0/0	0/0	0/0	0/0	0/0	0/0	0/1	0/0	0/1	0/1	0/1	0/0
Chromosome1-1331938	INS	PRECISE	81	0	0	0/0	0/0	0/0	0/0	0/0	0/0	0/0	0/0	0/1	0/0	0/1	0/1	0/1	0/0
Chromosome1-1332710	INS	PRECISE	1017	0	0	0/0	0/0	0/0	0/0	0/0	0/0	0/0	0/0	0/0	0/0	0/0	0/0	0/0	0/1
Chromosome1-1333295	INV	PRECISE	587	0	0	0/0	0/0	0/0	0/1	0/1	0/0	0/0	0/0	1/1	0/0	0/0	0/0	0/0	0/0
Chromosome1-1333925	INS	PRECISE	5748	0	0	0/0	0/0	0/0	0/0	0/0	0/0	0/0	0/0	0/0	0/0	0/0	0/0	0/1	0/0
Chromosome1-1334221	INS	PRECISE	109	0	0	0/0	0/1	0/1	0/0	0/1	1/1	0/0	0/0	0/0	0/1	0/0	0/0	0/0	1/1
Chromosome1-1334664	DEL	PRECISE	2211	0	0	0/0	0/0	0/0	0/0	0/0	0/0	0/0	0/0	0/0	0/0	0/0	0/0	0/0	0/1
Chromosome1-1335363	INS	PRECISE	148	0	0	0/0	0/0	0/0	0/0	0/0	0/0	0/0	0/0	0/0	0/0	0/0	0/0	0/0	0/1
Chromosome1-1335528	INS	PRECISE	627	0	0	0/0	0/0	0/0	0/0	0/0	0/0	0/0	0/0	0/0	0/0	0/0	0/0	0/0	0/1
Chromosome1-1337066	DEL	PRECISE	211	0	0	0/0	0/1	0/1	1/1	1/1	1/1	0/1	0/1	1/1	0/1	0/1	0/1	0/1	1/1
Chromosome1-1337393	DEL	PRECISE	1455	0	0	0/0	0/0	0/0	0/0	0/0	0/0	0/0	0/0	0/1	0/0	0/1	0/1	0/1	0/0
Chromosome1-1337797	DEL	PRECISE	87	0	0	0/0	0/0	0/0	0/0	0/0	0/0	0/0	0/0	0/1	0/0	0/1	0/1	0/1	0/0
Chromosome1-1339869	INS	PRECISE	1015	0	0.408	0/0	0/1	0/1	0/0	0/1	0/1	0/0	0/0	0/0	0/1	0/0	0/0	0/0	0/1
Chromosome1-1341731	INS	PRECISE	50	0	0	0/0	0/0	0/0	0/0	0/0	0/0	0/0	0/0	1/1	0/0	1/1	1/1	1/1	0/0
Chromosome1-1341927	DEL	PRECISE	2852	0	0	0/0	0/0	0/0	0/0	0/0	0/0	0/0	0/0	0/1	0/0	1/1	1/1	1/1	0/0
Chromosome1-1342088	INS	PRECISE	410	0	0.738	0/0	0/0	0/0	0/0	0/0	0/0	0/0	0/1	0/0	0/0	0/0	0/0	0/0	0/0
Chromosome1-1342215	DEL	PRECISE	4271	0	0	0/0	0/0	0/0	0/0	0/0	0/0	0/0	0/0	0/1	0/0	1/1	1/1	1/1	0/0
Chromosome1-1343721	INV	PRECISE	1272699	0	0	0/0	0/0	0/0	0/0	0/0	0/0	0/0	0/0	0/0	0/0	0/0	0/0	0/0	0/1
Chromosome1-1344279	INS	PRECISE	58	0	0	0/0	0/0	0/0	0/0	0/0	0/0	0/0	0/0	1/1	0/0	1/1	1/1	1/1	0/0
Chromosome1-1344561	DEL	PRECISE	1144	0.5	0.5	0/0	0/0	0/0	0/1	0/1	0/1	0/0	0/0	0/0	0/0	0/0	0/0	0/0	0/0
Chromosome1-1344707	INS	PRECISE	4572	0	0	0/0	0/0	0/0	0/0	0/0	0/0	0/0	0/0	0/1	0/0	0/0	0/0	0/0	0/0
Chromosome1-1344708	BND	PRECISE	0	0	0	0/0	0/0	0/0	0/0	0/0	0/0	0/0	0/0	0/1	0/0	0/0	0/0	0/1	0/0
Chromosome1-1345278	DEL	PRECISE	396	0	0	0/0	0/0	0/0	0/0	0/0	0/0	0/0	0/0	1/1	0/0	1/1	1/1	1/1	0/0
Chromosome1-1345766	INS	PRECISE	184	0	0	0/0	0/0	0/0	0/0	0/0	0/0	0/0	0/0	0/1	0/0	0/1	0/1	0/1	0/0
Chromosome1-1345985	INS	PRECISE	62	0	0	0/0	0/0	0/0	0/0	0/0	0/0	0/0	0/0	0/1	0/0	0/1	0/1	0/1	0/0
Chromosome1-1346259	INS	PRECISE	657	0	0.5	0/0	0/0	0/0	0/1	0/1	0/1	0/0	0/0	0/1	0/0	0/0	0/0	0/0	0/0
Chromosome1-1346267	DEL	PRECISE	69	0	0	0/0	0/0	0/0	0/1	0/0	0/0	0/1	0/1	0/0	0/0	0/0	0/0	0/0	0/0
Chromosome1-1346374	INS	PRECISE	66	0	0	0/0	0/0	0/0	0/0	0/0	0/0	0/0	0/0	0/1	0/0	0/1	0/1	0/1	0/0
Chromosome1-1346535	INS	PRECISE	64	0	0	0/0	0/0	0/0	0/0	0/0	0/0	0/0	0/0	0/1	0/0	0/1	0/1	0/1	0/0
Chromosome1-1346885	DEL	PRECISE	11861	0	0	0/0	0/0	0/0	0/0	0/0	0/0	0/0	0/0	0/1	0/0	0/0	0/0	0/1	0/0
Chromosome1-1348013	INS	PRECISE	54	0	0.378	0/0	0/0	0/0	0/1	0/0	0/0	0/1	0/1	0/0	0/0	0/0	0/0	0/0	0/0
Chromosome1-1348599	DEL	PRECISE	6859	0	0	0/0	0/1	0/1	0/0	0/1	0/1	0/0	0/0	0/1	0/1	0/1	0/1	0/1	1/1
Chromosome1-1348756	DEL	PRECISE	1957	0	0	0/0	0/0	0/0	0/0	0/0	0/0	0/1	0/1	0/0	0/0	0/0	0/0	0/0	0/0
Chromosome1-1349120	INS	PRECISE	93	0	0	0/0	0/0	0/0	0/1	0/0	0/0	0/0	0/0	0/0	0/0	0/0	0/0	0/0	0/0
Chromosome1-1349206	DEL	PRECISE	4576	0	0	0/0	0/0	0/0	0/0	0/0	0/0	0/1	0/1	0/0	0/0	0/0	0/0	0/0	0/0
Chromosome1-1349356	INS	PRECISE	94	0	0	0/0	0/0	0/0	0/0	0/0	0/0	0/0	0/0	0/1	0/0	0/1	0/1	0/1	0/0
Chromosome1-1350621	DEL	PRECISE	837	0	0	0/0	0/1	0/1	0/0	0/1	0/1	0/0	0/0	0/0	0/1	0/0	0/0	0/0	0/1
Chromosome1-1350859	DEL	PRECISE	1346	0	0	0/0	0/1	0/1	1/1	1/1	1/1	0/1	0/1	1/1	0/1	0/1	0/1	0/1	1/1
Chromosome1-1351220	BND	PRECISE	0	0	0	0/0	0/0	0/0	0/0	0/0	0/0	0/0	0/0	0/1	0/0	0/0	0/0	0/0	0/0
Chromosome1-1352202	DEL	PRECISE	51	0	0	0/0	0/0	0/0	0/1	0/1	0/1	0/0	0/0	0/1	0/0	0/0	0/0	0/0	0/0
Chromosome1-1352493	DEL	PRECISE	63	0.577	0.577	0/0	0/0	0/0	0/0	0/0	0/0	0/0	0/0	0/1	0/0	0/1	0/1	0/1	0/0
Chromosome1-1353383	INS	PRECISE	68	0	0	0/0	0/0	0/0	0/0	0/0	0/0	0/0	0/0	0/1	0/0	0/1	0/1	0/1	0/0
Chromosome1-1353564	DEL	PRECISE	12049	0	0	0/0	0/0	0/0	0/1	0/0	0/0	0/1	0/1	0/0	0/0	0/0	0/0	0/0	0/0
Chromosome1-1353712	DEL	PRECISE	53	0	0	0/0	0/0	0/0	0/0	0/0	0/0	0/0	0/0	0/1	0/0	0/1	0/1	0/1	0/0
Chromosome1-1353811	INV	PRECISE	1171799	0	0	0/0	0/0	0/0	0/0	0/0	0/0	0/0	0/0	0/0	0/0	0/0	0/0	0/0	0/1
Chromosome1-1354685	INS	PRECISE	431	0	0	0/0	0/0	0/0	0/0	0/0	0/0	0/0	0/0	0/0	0/0	0/0	0/0	0/0	0/1
Chromosome1-1354839	DEL	PRECISE	6487	0	0	0/0	0/0	0/0	0/1	0/1	0/1	0/0	0/0	1/1	0/0	0/0	0/0	0/0	0/0
Chromosome1-1354863	DEL	PRECISE	3901	0	0	0/0	0/0	0/0	0/0	0/0	0/0	0/0	0/0	0/0	0/0	0/1	0/0	0/1	0/0
Chromosome1-1355256	DEL	PRECISE	3922	0	0	0/0	0/0	0/0	0/0	0/0	0/0	0/0	0/0	0/0	0/0	0/1	0/0	0/1	0/0
Chromosome1-1357069	DEL	PRECISE	98	0	0	0/0	0/0	0/0	0/0	0/0	0/0	0/0	0/0	0/1	0/0	0/1	0/1	0/1	0/0
Chromosome1-1357745	INS	PRECISE	19233	0	0	0/0	0/0	0/0	0/0	0/0	0/0	0/0	0/0	0/0	0/0	0/0	0/1	0/0	0/0
Chromosome1-1357804	INS	PRECISE	258	0	0.577	0/0	0/0	0/0	0/0	0/0	0/0	0/0	0/0	0/0	0/0	0/1	0/1	0/1	0/0
Chromosome1-1358069	INS	PRECISE	72	0	0	0/0	0/0	0/0	0/0	0/0	0/0	0/0	0/0	0/0	0/0	0/1	0/1	0/1	0/0
Chromosome1-1358334	INS	PRECISE	6855	0	0.707	0/0	0/0	0/0	0/0	0/0	0/0	0/1	1/1	0/0	0/0	0/0	0/0	0/0	0/0
Chromosome1-1358588	INS	PRECISE	105	0	0	0/0	0/0	0/0	0/0	0/0	0/0	0/0	0/0	0/0	0/0	0/0	0/0	0/0	0/1
Chromosome1-1359282	INS	PRECISE	194	0	0.527	0/0	0/1	0/1	0/0	0/1	0/0	0/0	0/0	0/0	0/1	0/1	0/1	0/1	1/1
Chromosome1-1359802	DEL	PRECISE	514	0	0	0/0	0/0	0/0	0/1	0/0	0/0	0/1	0/1	0/0	0/0	0/0	0/0	0/0	0/0
Chromosome1-1359939	DEL	PRECISE	367	0	0	0/0	0/0	0/0	0/0	0/0	0/0	0/0	0/0	0/1	0/0	0/1	0/0	0/1	0/0
Chromosome1-1360106	INS	PRECISE	6571	0	0	0/0	0/0	0/0	0/0	0/0	0/0	0/0	0/0	0/0	0/0	0/0	0/0	0/0	0/1
Chromosome1-1362157	INS	PRECISE	1015	0	0	0/0	0/0	0/0	0/0	0/0	0/0	0/0	0/0	0/0	0/0	0/0	0/0	0/0	0/1
Chromosome1-1362537	DEL	PRECISE	1171	0.5	0.5	0/0	0/0	0/0	0/0	0/0	0/0	0/0	0/0	0/1	0/0	0/1	0/1	0/1	0/0

Chromosome1-1362843	DEL	PRECISE	25138	0	0	0/0	1/1	1/1	1/1	1/1	1/1	1/1	1/1	1/1	0/1	1/1	1/1	1/1	1/1
Chromosome1-1363451	DEL	PRECISE	3518	0	0	0/0	0/0	0/0	0/0	0/0	0/1	0/0	0/0	0/1	0/1	0/0	0/1	0/0	0/0
Chromosome1-1363805	DEL	PRECISE	2650	0	0	0/0	0/1	0/0	0/1	0/1	0/0	0/0	0/0	0/1	0/0	0/0	0/0	0/0	1/1
Chromosome1-1364035	DEL	PRECISE	151	0	0	0/0	0/0	0/0	0/0	0/0	0/1	0/0	0/0	0/0	0/0	0/0	0/0	0/0	0/0
Chromosome1-1364312	DEL	PRECISE	1842	0	0	0/0	0/1	0/0	0/1	0/1	0/0	0/0	0/0	0/1	0/0	0/0	0/0	0/0	0/1
Chromosome1-1365009	INS	PRECISE	885	0	0	0/0	0/0	0/0	0/0	0/0	0/1	0/0	0/0	0/0	0/0	0/0	0/0	0/0	0/0
Chromosome1-1365396	DEL	PRECISE	5786	0	0	0/0	0/0	0/0	0/0	0/0	0/0	0/0	0/0	1/1	0/0	0/0	0/0	0/1	0/0
Chromosome1-1365463	DEL	PRECISE	12073	0	0	0/0	0/0	0/0	1/1	1/1	0/1	0/0	0/0	1/1	0/0	0/0	0/0	0/0	0/0
Chromosome1-1366579	DEL	PRECISE	1891	0	0	0/0	0/0	0/0	0/0	0/0	0/0	0/0	0/0	0/1	0/0	1/1	0/1	0/1	0/0
Chromosome1-1366986	INS	PRECISE	116	0	0	0/0	0/0	0/0	0/1	0/1	0/0	0/0	0/0	0/1	0/0	0/0	0/0	0/0	0/0
Chromosome1-1367670	DEL	PRECISE	672	0	0	0/0	0/0	0/0	0/0	0/0	0/0	0/0	0/0	0/0	0/0	0/1	0/1	0/1	0/0
Chromosome1-1367733	DEL	PRECISE	65	0	0	0/0	0/0	0/0	0/0	0/0	0/0	0/0	0/0	0/0	0/0	0/0	0/0	0/0	0/1
Chromosome1-1368247	BND	PRECISE	0	0	0	0/0	0/0	0/0	0/0	0/0	0/0	0/0	0/0	0/0	0/0	0/0	0/1	0/1	0/0
Chromosome1-1368758	DEL	PRECISE	401	0	0	0/0	0/0	0/0	0/0	0/0	0/0	0/0	0/0	0/0	0/0	0/0	0/0	0/0	0/1
Chromosome1-1369319	INS	PRECISE	6997	0	0	0/0	1/1	0/0	0/0	0/0	0/0	0/0	0/0	0/0	0/0	0/0	0/0	0/0	0/0
Chromosome1-1369601	INS	PRECISE	134	0	0	0/0	0/0	0/0	0/1	0/1	0/1	0/0	0/0	1/1	0/0	0/0	0/0	0/0	0/0
Chromosome1-1370244	DEL	PRECISE	52	0	0	0/0	0/0	0/0	0/1	0/0	0/0	0/1	0/1	0/0	0/0	0/0	0/0	0/0	0/0
Chromosome1-1370753	DEL	PRECISE	224	0	0	0/0	0/0	0/0	0/0	0/0	0/0	0/0	0/0	0/0	0/0	0/0	0/0	0/0	0/1
Chromosome1-1370902	DEL	PRECISE	7196	0	0	0/0	0/0	0/0	0/0	0/0	0/0	0/0	0/0	0/1	0/0	0/1	0/1	0/1	0/0
Chromosome1-1371624	DEL	PRECISE	1943	0	0	0/0	0/0	0/0	0/0	0/0	0/0	0/0	0/0	0/1	0/0	0/1	0/1	0/1	0/0
Chromosome1-1371909	DEL	PRECISE	113	0	0	0/0	0/0	0/0	0/0	0/0	0/0	0/0	0/0	0/1	0/0	0/1	0/1	0/1	0/0
Chromosome1-1372007	DEL	PRECISE	1024	0	0	0/0	0/0	0/0	0/0	0/0	0/0	0/0	0/0	0/1	0/0	0/1	0/1	0/1	0/0
Chromosome1-1372253	DEL	PRECISE	63	0	0	0/0	0/1	0/1	0/0	0/1	0/1	0/0	0/0	0/0	0/1	0/0	0/0	0/0	0/1
Chromosome1-1372640	DEL	PRECISE	66	0	0	0/0	0/0	0/0	0/1	0/0	0/0	0/1	0/1	0/0	0/0	0/0	0/0	0/0	0/0
Chromosome1-1372707	INS	PRECISE	72	0	0	0/0	0/0	0/0	0/0	0/0	0/0	0/0	0/0	0/1	0/0	0/1	0/1	0/1	0/0
Chromosome1-1372818	INS	PRECISE	6973	0	0	0/0	0/0	0/0	0/0	0/0	0/0	0/0	0/0	0/0	0/0	0/0	0/0	0/0	1/1
Chromosome1-1372818	BND	PRECISE	0	0	0	0/0	0/0	0/0	0/0	0/0	0/0	0/0	0/0	0/0	0/0	0/0	0/0	0/0	0/1
Chromosome1-1373058	DEL	PRECISE	21247	0	0	0/0	0/0	0/0	0/0	0/0	0/0	0/0	0/0	1/1	0/0	0/1	1/1	1/1	0/0
Chromosome1-1373217	INS	PRECISE	52	0	0.447	0/0	0/0	0/0	0/0	0/0	0/1	0/0	0/0	0/0	0/0	0/0	0/1	0/0	0/0
Chromosome1-1373308	DEL	PRECISE	110	0	0	0/0	0/0	0/0	0/0	0/0	0/1	0/0	0/0	0/0	0/0	0/0	0/1	0/0	0/0
Chromosome1-1373431	DEL	PRECISE	5027	0	0	0/0	0/0	0/0	1/1	0/1	0/1	0/1	0/1	0/1	0/0	0/0	0/0	0/0	1/1
Chromosome1-1373433	INS	PRECISE	878	0	0.894	0/0	0/0	0/0	0/0	0/1	0/1	0/0	0/0	0/0	0/0	0/0	0/1	0/0	0/0
Chromosome1-1373937	DEL	PRECISE	9038	0	0	0/0	0/0	0/0	1/1	0/1	0/1	0/1	0/1	0/1	0/1	0/0	0/0	0/0	1/1
Chromosome1-1374140	INS	PRECISE	12981	0	0	0/0	0/0	0/0	0/0	0/0	0/0	0/0	0/0	0/0	0/0	0/0	0/0	0/1	0/0
Chromosome1-1375258	INS	PRECISE	53	0	0.957	0/0	0/0	0/0	0/0	0/0	0/0	0/0	0/0	0/1	0/0	0/1	0/1	0/1	0/0
Chromosome1-1375780	DEL	PRECISE	1583	0	0	0/0	0/0	0/0	0/0	0/0	0/0	0/0	0/0	0/1	0/0	0/1	0/1	0/0	0/0
Chromosome1-1376103	DEL	PRECISE	72	0	0	0/0	0/0	0/0	0/0	0/0	0/0	0/0	0/0	0/1	0/0	0/1	0/1	0/0	0/0
Chromosome1-1376384	DEL	PRECISE	284	0	0	0/0	0/0	0/0	0/0	0/0	0/0	0/0	0/0	0/0	0/0	0/0	0/0	0/0	0/1
Chromosome1-1377505	DEL	PRECISE	378	0	0	0/0	0/1	0/1	0/0	0/1	0/1	0/0	0/0	0/0	0/1	0/0	0/0	0/0	0/1
Chromosome1-1377996	DEL	PRECISE	22708	0	0	0/0	0/0	0/0	0/0	0/0	0/0	0/0	0/0	0/0	0/0	0/0	0/0	0/0	1/1
Chromosome1-1378767	DEL	PRECISE	4576	0	0	0/0	0/0	0/1	0/0	0/1	0/0	0/0	0/0	0/1	0/0	0/1	1/1	0/1	1/1
Chromosome1-1381255	DEL	PRECISE	404	0	0	0/0	0/0	0/0	0/0	0/0	0/0	0/0	0/0	0/0	0/0	0/0	0/0	0/0	0/1
Chromosome1-1381972	DEL	PRECISE	3075	0	0	0/0	0/1	0/1	0/0	0/1	0/1	0/0	0/0	0/0	0/1	0/0	0/0	0/0	0/1
Chromosome1-1382382	DEL	PRECISE	395	0	0	0/0	0/0	0/0	0/0	0/0	0/0	0/0	0/0	0/0	0/0	0/0	0/0	0/0	0/1
Chromosome1-1383725	DEL	PRECISE	730	0	0	0/0	0/0	0/0	0/0	0/0	0/0	0/0	0/0	1/1	0/0	0/1	0/1	0/1	0/0
Chromosome1-1383926	DEL	PRECISE	75	0	0	0/0	0/0	0/0	0/0	0/0	0/0	0/0	0/0	0/0	0/0	0/0	0/0	0/0	0/1
Chromosome1-1385233	DEL	PRECISE	22183	0	0	0/0	0/0	0/0	0/0	0/0	0/0	0/0	0/0	1/1	0/0	0/1	1/1	0/1	0/0
Chromosome1-1386188	DEL	PRECISE	298	0.516	0.516	0/0	0/1	0/1	0/0	0/1	0/1	0/0	0/0	0/0	0/1	0/0	0/0	0/0	1/1
Chromosome1-1388409	DEL	PRECISE	253	0	0	0/0	0/0	0/0	0/0	0/0	0/0	0/0	0/0	0/1	0/0	0/1	0/1	0/1	0/0
Chromosome1-1389255	DEL	PRECISE	1258	0	0	0/0	0/0	0/0	0/1	0/0	0/0	0/0	0/0	0/0	0/0	0/0	0/0	0/0	0/0
Chromosome1-1389734	DEL	PRECISE	89	0	0	0/0	0/1	0/1	1/1	1/1	1/1	0/1	0/1	1/1	0/1	0/0	0/0	0/0	1/1
Chromosome1-1389837	DEL	PRECISE	104	0	0	0/0	0/0	0/0	0/0	0/0	0/0	0/0	0/0	0/0	0/0	0/0	0/0	0/0	0/1
Chromosome1-1391665	DEL	PRECISE	51	0	0	0/0	0/0	0/0	0/0	0/0	0/0	0/0	0/0	0/1	0/0	0/1	0/1	0/1	0/0
Chromosome1-1392634	DEL	PRECISE	69	0	0	0/0	0/0	0/0	0/0	0/0	0/0	0/0	0/0	0/1	0/0	0/1	0/1	0/1	0/0
Chromosome1-1393256	DEL	PRECISE	149	0	0	0/0	0/0	0/0	0/0	0/0	0/0	0/0	0/0	0/0	0/0	0/0	0/0	0/0	0/1
Chromosome1-1393825	INS	PRECISE	312	0	0	0/0	0/0	0/0	0/0	0/0	0/0	0/0	0/0	0/0	0/0	0/0	0/0	0/0	0/1
Chromosome1-1394331	INS	PRECISE	952	0	0	0/0	0/0	0/0	0/0	0/0	0/0	0/0	0/0	0/0	0/0	0/0	0/0	0/0	0/1
Chromosome1-1394457	DEL	PRECISE	139	0	0	0/0	0/0	0/0	0/1	0/0	0/0	1/1	0/1	0/0	0/0	0/0	0/0	0/0	0/0
Chromosome1-1394646	DEL	PRECISE	76	0	0	0/0	0/0	0/0	0/0	0/0	0/0	0/0	0/0	0/1	0/0	0/1	0/1	0/1	0/0
Chromosome1-1394975	DEL	PRECISE	902	0	0	0/0	0/0	0/0	0/0	0/0	0/0	0/0	0/0	0/1	0/0	0/1	0/1	1/1	0/0
Chromosome1-1395356	BND	PRECISE	0	0	0	0/0	0/0	0/0	0/0	0/0	0/0	0/0	0/0	0/1	0/0	1/1	0/1	1/1	0/0
Chromosome1-1395624	BND	PRECISE	0	0	0	0/0	0/0	0/0	0/0	0/0	0/0	0/0	0/0	0/0	0/0	0/1	0/0	0/1	0/0
Chromosome1-1395624	BND	PRECISE	0	0	0	0/0	0/0	0/0	0/0	0/0	0/0	0/0	0/0	0/0	0/0	0/0	0/0	0/1	0/0
Chromosome1-1396094	DEL	PRECISE	138	0	0	0/0	0/0	0/0	0/0	0/0	0/0	0/0	0/0	0/1	0/0	0/1	0/0	0/1	0/0
Chromosome1-1396654	DEL	PRECISE	126	0	0	0/0	0/0	0/0	0/0	0/0	0/0	0/0	0/0	0/0	0/0	0/0	0/0	0/0	0/1
Chromosome1-1397154	INS	PRECISE	57	0.5	0.5	0/0	0/0	0/0	0/0	0/0	0/0	0/0	0/0	0/1	0/0	0/1	0/1	0/1	0/0
Chromosome1-1397645	DEL	PRECISE	406	0	0	0/0	0/0	0/0	0/0	0/0	0/0	0/0	0/0	0/1	0/0	0/1	0/1	0/1	0/0
Chromosome1-1399109	INS	PRECISE	60	0	0	0/0	0/0	0/0	0/0	0/0	0/0	0/0	0/0	0/1	0/0	0/1	0/1	0/1	0/0
Chromosome1-1399885	INS	PRECISE	1004	0	0	0/0	0/0	0/0	0/0	0/0	0/0	0/0	0/0	0/1	0/0	0/0	0/0	0/0	0/0
Chromosome1-1400871	DEL	PRECISE	198	0	0	0/0	0/0	0/0	0/1	0/1	0/1	0/0	0/0	0/1	0/0	0/0	0/0	0/0	0/0
Chromosome1-1401196	BND	PRECISE	0	0	0	0/0	0/1	0/0	0/0	0/1	0/0	0/0	0/0	0/0	0/0	0/0	0/0	0/0	0/0
Chromosome1-1402066	DEL	PRECISE	9764	0	0	0/0	0/0	0/0	0/0	0/0	0/0	0/0	0/0	0/0	0/0	0/0	0/0	0/0	0/1
Chromosome1-1402066	DEL	PRECISE	14930	0	0	0/0	0/0	0/0	0/0	0/0	0/0	0/0	0/0	1/1	0/0	0/1	0/1	0/1	0/0

Chromosome1-1402468	DEL	PRECISE	1551	0	0	0/0	0/1	0/1	0/1	0/1	0/1	0/1	0/1	0/0	0/1	0/0	0/0	0/0	0/1
Chromosome1-1402995	DEL	PRECISE	62	0	0	0/0	0/0	0/0	0/1	0/1	0/1	0/0	0/0	1/1	0/0	0/0	0/0	0/0	0/0
Chromosome1-1403899	DEL	PRECISE	12270	0	0	0/0	0/0	0/0	0/0	0/0	0/0	0/0	0/0	0/1	0/0	0/1	0/1	0/1	0/0
Chromosome1-1404757	INS	PRECISE	65	0	0	0/0	0/0	0/0	0/0	0/0	0/0	0/0	0/0	0/0	0/0	0/0	0/0	0/0	0/1
Chromosome1-1405556	INS	PRECISE	285	0	0	0/0	0/0	0/0	0/0	0/0	0/0	0/0	0/0	0/0	0/0	0/0	0/0	0/0	0/1
Chromosome1-1405824	INS	PRECISE	9734	0	0	0/0	0/0	0/0	0/0	0/0	0/0	0/0	0/0	0/0	0/0	0/0	0/0	0/0	0/1
Chromosome1-1405846	INS	PRECISE	1078	0	0	0/0	0/0	0/0	0/0	0/0	0/0	0/0	0/0	0/0	0/0	0/0	0/0	0/0	0/1
Chromosome1-1406270	DEL	PRECISE	237	0	0	0/0	0/0	0/0	0/0	0/0	0/0	0/0	0/0	0/1	0/0	0/1	0/0	0/1	0/0
Chromosome1-1406388	INS	PRECISE	56	0	0	0/0	0/0	0/0	0/1	0/0	0/0	0/0	0/0	0/0	0/0	0/0	0/0	0/0	0/0
Chromosome1-1406453	INV	PRECISE	10124273	0	0	0/0	0/1	0/0	0/0	0/1	0/0	0/0	0/0	0/0	0/0	0/0	0/0	0/0	0/0
Chromosome1-1407067	INS	PRECISE	4948	0	0	0/0	0/0	0/0	1/1	0/0	0/0	0/0	0/0	0/0	0/0	0/0	0/0	0/0	0/0
Chromosome1-1407297	INS	PRECISE	52	0	0.316	0/0	0/1	0/1	1/1	1/1	1/1	0/1	0/1	0/1	0/1	0/0	0/0	0/0	0/1
Chromosome1-1408093	DEL	PRECISE	75	0	0	0/0	0/0	0/0	0/0	0/0	0/0	0/0	0/0	0/1	0/0	0/1	0/0	0/1	0/0
Chromosome1-1408843	INS	PRECISE	117	0	0	0/0	0/0	0/0	0/0	0/0	0/0	0/0	0/0	0/1	0/0	1/1	0/0	0/1	0/0
Chromosome1-1408966	DEL	PRECISE	4939	0	0	0/0	0/1	0/1	1/1	1/1	1/1	0/1	0/1	1/1	0/1	1/1	0/0	0/1	1/1
Chromosome1-1410668	INS	PRECISE	120	0	0	0/0	0/0	0/0	0/0	0/0	0/0	0/0	0/0	0/1	0/0	0/1	0/1	0/1	0/0
Chromosome1-1411065	DEL	PRECISE	103	0	0	0/0	0/0	0/0	0/1	0/0	0/0	0/1	0/1	0/0	0/0	0/0	0/0	0/0	0/0
Chromosome1-1411689	DEL	PRECISE	181	0	0	0/0	0/0	0/0	0/0	0/0	0/0	0/0	0/0	0/1	0/0	0/1	0/1	0/1	0/0
Chromosome1-1411844	DEL	PRECISE	148	0	0	0/0	0/0	0/0	0/0	0/0	0/0	0/0	0/0	0/0	0/0	0/1	0/1	0/1	0/0
Chromosome1-1411963	INS	PRECISE	58	0	0	0/0	0/0	0/0	0/0	0/0	0/0	0/0	0/0	0/0	0/0	0/1	0/1	0/1	0/0
Chromosome1-1413226	DEL	PRECISE	9681	0	0	0/0	0/0	0/0	0/0	0/0	0/0	0/0	0/0	0/0	0/0	0/1	0/1	0/1	0/0
Chromosome1-1415745	INS	PRECISE	162	0	0	0/0	0/1	0/1	0/0	0/1	0/1	0/0	0/0	0/0	0/1	0/0	0/0	0/0	0/1
Chromosome1-1416734	BND	PRECISE	0	0	0	0/0	0/0	0/0	0/0	0/0	0/0	0/0	0/0	0/1	0/0	0/1	0/1	0/1	0/0
Chromosome1-1418308	DEL	PRECISE	112	0	0	0/0	0/0	0/0	0/0	0/0	0/0	0/0	0/0	0/1	0/0	0/1	0/1	0/1	0/0
Chromosome1-1418887	INS	PRECISE	54	0	0	0/0	0/0	0/0	0/0	0/0	0/0	0/0	0/0	0/1	0/0	0/1	0/1	0/1	0/0
Chromosome1-1418981	DEL	PRECISE	68	0	0	0/0	0/1	1/1	0/1	0/1	0/1	0/1	0/1	0/0	0/1	0/0	0/0	0/0	1/1
Chromosome1-1419502	DEL	PRECISE	54	0.441	0.441	0/0	0/1	0/1	0/1	1/1	0/1	0/1	0/1	0/0	0/1	0/0	0/0	0/0	1/1
Chromosome1-1419981	INS	PRECISE	10923	0	0	0/0	0/0	0/0	0/0	0/0	0/1	0/0	0/0	0/0	0/0	0/0	0/0	0/0	0/0
Chromosome1-1419982	BND	PRECISE	0	0	0	0/0	0/0	0/0	0/0	0/0	0/0	0/0	0/0	0/1	0/0	0/0	0/0	0/0	0/0
Chromosome1-1420000	INS	PRECISE	9602	0	0	0/0	0/0	0/0	0/1	0/0	0/0	0/0	0/0	0/0	0/0	0/0	0/0	0/0	0/0
Chromosome1-1420751	BND	PRECISE	0	0	0	0/0	0/0	0/0	0/1	0/0	0/0	0/0	0/0	0/0	0/0	0/0	0/0	0/0	0/0
Chromosome1-1421021	INS	PRECISE	104	0	0	0/0	0/0	0/0	0/0	0/0	0/0	0/0	0/0	0/1	0/0	0/1	0/1	0/1	0/0
Chromosome1-1422474	INS	PRECISE	776	0	0.577	0/0	0/0	0/0	0/0	0/0	0/0	0/0	0/0	0/1	0/0	0/0	0/1	0/1	0/0
Chromosome1-1422718	DEL	PRECISE	17977	0	0	0/0	0/0	0/0	0/0	0/0	0/0	0/0	0/0	0/1	0/0	0/1	0/1	0/1	0/0
Chromosome1-1422720	DEL	PRECISE	85	0	0	0/0	0/1	0/1	0/0	0/1	0/1	0/0	0/0	0/0	0/1	0/0	0/0	0/0	0/1
Chromosome1-1423241	DEL	PRECISE	2220	0	0	0/0	0/0	0/0	0/0	0/0	0/0	0/0	0/0	0/0	0/0	0/0	0/0	0/0	0/1
Chromosome1-1423688	DEL	PRECISE	99	0	0	0/0	0/1	0/1	0/0	0/1	0/1	0/0	0/0	0/0	0/1	0/0	0/0	0/0	0/1
Chromosome1-1423988	INS	PRECISE	601	0	0.957	0/0	0/0	0/0	0/1	0/0	0/0	0/1	0/0	0/0	0/0	0/0	0/0	0/0	1/1
Chromosome1-1424399	INS	PRECISE	57	0	0	0/0	0/0	0/0	0/1	0/1	0/1	0/0	0/0	0/1	0/0	0/0	0/0	0/0	0/0
Chromosome1-1425080	DEL	PRECISE	5784	0	0	0/0	0/0	0/0	0/0	0/0	0/0	0/0	0/0	0/0	0/0	0/0	0/0	0/0	0/1
Chromosome1-1425222	DEL	PRECISE	151	0	0	0/0	0/0	0/0	0/0	0/0	0/0	0/0	0/0	0/1	0/0	0/1	0/1	0/1	0/0
Chromosome1-1425531	DEL	PRECISE	11754	0	0	0/0	0/0	0/0	0/0	0/0	0/0	0/0	0/0	0/1	0/0	0/0	0/1	0/1	0/0
Chromosome1-1425601	DEL	PRECISE	693	0.577	0.577	0/0	0/0	0/0	1/1	0/1	1/1	0/0	0/0	0/1	0/0	0/0	0/0	0/0	0/0
Chromosome1-1426566	INS	PRECISE	8431	0	0	0/0	0/0	0/0	0/1	0/0	0/0	0/0	0/0	0/0	0/0	0/0	0/0	0/0	0/0
Chromosome1-1426566	BND	PRECISE	0	0	0	0/0	0/0	0/0	0/1	0/0	0/0	0/0	0/0	0/0	0/0	0/0	0/0	0/0	0/0
Chromosome1-1426683	INS	PRECISE	230	0	0	0/0	0/0	0/0	0/0	0/0	0/0	0/0	0/0	0/0	0/1	0/0	0/0	0/0	0/0
Chromosome1-1426683	INS	PRECISE	12464	0	0	0/0	0/0	0/0	0/0	0/0	0/0	0/0	0/0	0/0	0/0	0/0	0/0	0/0	0/0
Chromosome1-1426986	DEL	PRECISE	123	0	0	0/0	0/0	0/0	0/1	0/1	0/0	0/0	0/1	0/0	0/1	0/1	0/0	0/1	0/0
Chromosome1-1427110	INS	PRECISE	90	0	0.376	0/1	0/0	0/0	0/1	0/1	0/0	0/0	0/1	0/0	0/1	0/1	0/0	0/1	0/1
Chromosome1-1427148	DEL	PRECISE	355	0	0	0/0	0/0	0/0	0/1	0/1	0/0	0/0	0/1	0/0	0/1	0/1	0/0	0/1	0/1
Chromosome1-1427425	BND	PRECISE	0	0	0	0/0	0/0	0/0	0/0	0/0	0/0	0/0	0/0	0/1	0/0	0/0	1/1	1/1	0/0
Chromosome1-1428726	DEL	PRECISE	22507	0	0	0/0	0/0	0/0	0/0	0/0	0/0	0/0	0/0	0/1	0/0	1/1	1/1	1/1	0/0
Chromosome1-1429220	DEL	PRECISE	844	0	0	0/0	0/1	0/1	0/0	0/1	0/1	0/0	0/0	0/0	0/1	0/0	0/0	0/0	0/1
Chromosome1-1429544	DEL	PRECISE	1527	0	0	0/0	0/1	0/1	1/1	1/1	1/1	0/1	0/1	1/1	0/1	0/0	0/0	0/0	1/1
Chromosome1-1430114	INS	PRECISE	103	0	0	0/0	0/1	0/1	0/0	0/1	0/1	0/0	0/0	0/0	0/1	0/0	0/0	0/0	1/1
Chromosome1-1430390	DEL	PRECISE	907	0	0	0/0	0/1	0/1	0/0	0/1	0/1	0/0	0/0	0/0	0/1	0/0	0/0	0/0	0/1
Chromosome1-1430742	DEL	PRECISE	168	0	0	0/0	0/1	0/1	0/0	0/1	0/1	0/0	0/0	0/0	0/1	0/0	0/0	0/0	0/1
Chromosome1-1431225	DEL	PRECISE	312	0.5	0.5	0/0	0/0	0/0	0/0	0/0	0/0	0/0	0/0	0/1	0/0	0/1	0/1	0/1	0/0
Chromosome1-1432282	INS	PRECISE	1252	0	0	0/0	0/0	0/0	0/0	0/0	0/0	0/0	0/0	0/0	0/0	0/0	0/0	0/0	0/1
Chromosome1-1432340	INS	PRECISE	57	0	0	0/0	0/0	0/0	0/0	0/0	0/0	0/0	0/0	0/0	0/0	0/0	0/0	0/0	0/1
Chromosome1-1432748	INS	PRECISE	5002	0	0	0/0	0/0	0/0	1/1	0/0	0/0	0/0	0/0	0/0	0/0	0/0	0/0	0/0	0/0
Chromosome1-1432751	INS	PRECISE	54	0	0	0/0	0/0	0/0	0/0	0/0	0/0	0/0	0/0	0/1	0/0	0/1	0/1	0/1	0/0
Chromosome1-1433000	DEL	PRECISE	519	0	0	0/0	0/0	0/0	0/0	0/0	0/0	0/0	0/0	0/0	0/0	0/0	0/0	0/0	0/1
Chromosome1-1433216	DEL	PRECISE	61	0	0	0/0	0/0	0/0	0/0	0/0	0/0	0/0	0/0	0/1	0/0	0/1	0/1	0/1	0/0
Chromosome1-1433389	DEL	PRECISE	1017	0	0	0/0	0/0	0/0	0/0	0/0	0/0	0/0	0/0	0/1	0/0	0/1	0/1	0/1	0/0
Chromosome1-1433853	INS	PRECISE	84	0	0	0/0	0/0	0/0	0/0	0/0	0/0	0/0	0/0	0/1	0/0	0/1	0/1	0/1	0/0
Chromosome1-1434621	DEL	PRECISE	1842	0	0	0/0	0/0	0/0	0/0	0/0	0/0	0/0	0/0	0/1	0/0	0/1	0/1	0/1	0/0
Chromosome1-1434713	INS	PRECISE	10099	0	0	0/0	0/0	0/0	0/0	0/0	0/0	0/0	0/0	0/0	0/0	0/0	0/0	0/0	0/1
Chromosome1-1434714	BND	PRECISE	0	0	0	0/0	0/0	0/0	0/0	0/0	0/0	0/0	0/0	0/0	0/0	0/0	0/0	0/0	0/1
Chromosome1-1434714	BND	PRECISE	0	0	0	0/0	0/0	0/0	0/0	0/0	0/0	0/0	0/0	0/0	0/0	0/0	0/0	0/0	0/1
Chromosome1-1435426	DEL	PRECISE	656	0	0	0/0	0/0	0/0	0/0	0/0	0/0	0/0	0/0	0/1	0/0	0/1	0/1	0/1	0/0
Chromosome1-1435656	INS	PRECISE	90	0	0	0/0	0/0	0/0	0/1	0/0	0/0	0/1	0/1	0/1	0/0	0/1	0/1	0/1	0/1
Chromosome1-1435862	DEL	PRECISE	198	0	0	0/0	0/0	0/0	0/0	0/0	0/0	0/0	0/0	0/1	0/0	0/1	0/1	0/1	0/0

Chromosome1-1436573 INS	PRECISE	439	0	0	0/0	0/0	0/0	0/0	0/0	0/0	0/0	0/0	0/1	0/0	0/0	0/1	0/1	0/0
Chromosome1-1436917 INS	PRECISE	124	0	0.5	0/0	0/0	0/0	0/0	0/0	0/0	0/0	0/0	0/1	0/0	0/1	0/1	0/1	0/0
Chromosome1-1437234 INS	PRECISE	8891	0	0	0/0	0/0	0/0	0/0	0/0	0/0	0/0	0/0	0/0	0/0	0/0	0/0	0/0	0/1
Chromosome1-1437773 DEL	PRECISE	232	0	0	0/0	0/0	0/0	0/0	0/0	0/0	0/0	0/0	0/1	0/0	0/1	0/1	0/1	0/0
Chromosome1-1438077 DEL	PRECISE	65	0	0	0/0	0/0	0/0	0/0	0/0	0/0	0/0	0/0	0/0	0/0	0/0	0/0	0/0	0/1
Chromosome1-1438453 INS	PRECISE	276	0	0	0/0	0/0	0/0	0/0	0/0	0/0	0/0	0/0	0/0	0/0	0/0	0/0	0/0	0/1
Chromosome1-1438571 DEL	PRECISE	54	0	0	0/0	0/0	0/0	0/1	0/1	0/1	0/0	0/0	1/1	0/0	0/0	0/0	0/0	0/0
Chromosome1-1438572 INS	PRECISE	58	0	0	0/0	0/1	0/1	0/0	0/1	0/1	0/0	0/0	0/0	0/1	0/0	0/0	0/0	0/1
Chromosome1-1440917 DEL	PRECISE	1128	0	0	0/0	0/0	0/0	0/0	0/0	0/0	0/0	0/0	0/1	0/0	0/0	0/0	0/1	0/0
Chromosome1-1441363 INS	PRECISE	4911	0	0	0/0	0/0	0/0	0/0	0/0	0/0	0/0	0/0	0/0	0/0	0/0	0/0	0/0	1/1
Chromosome1-1442897 DEL	PRECISE	58	0	0	0/0	0/0	0/0	0/0	0/0	0/0	0/0	0/0	0/1	0/0	0/1	0/1	0/1	0/0
Chromosome1-1443092 INS	PRECISE	530	0	0.5	0/0	0/0	0/0	0/0	0/0	0/0	0/0	0/0	0/1	0/0	0/1	0/1	0/1	0/0
Chromosome1-1443319 INS	PRECISE	10117	0	0	0/0	0/0	0/0	0/0	0/0	0/0	0/0	0/0	0/0	0/0	0/0	0/0	0/0	0/1
Chromosome1-1444184 INS	PRECISE	148	0	0.5	0/0	0/0	0/0	0/0	0/0	0/0	0/0	0/0	0/1	0/0	0/1	0/1	0/1	0/0
Chromosome1-1444276 INS	PRECISE	50	0	0	0/0	0/0	0/0	0/0	0/0	0/0	0/0	0/0	0/0	0/0	0/0	0/0	0/0	0/1
Chromosome1-1444289 DEL	PRECISE	85	0	0	0/0	0/0	0/0	0/0	0/0	0/0	0/0	0/0	0/0	0/0	0/0	0/0	0/0	0/1
Chromosome1-1444689 BND	PRECISE	0	0	0	0/0	0/0	0/0	0/0	0/0	0/0	0/0	0/0	1/1	0/0	1/1	0/1	0/1	0/0
Chromosome1-1445463 DEL	PRECISE	137	0	0	0/0	0/1	0/1	0/1	0/1	0/1	0/1	0/1	1/1	0/1	0/1	0/1	0/1	1/1
Chromosome1-1445592 DEL	PRECISE	1164	0	0	0/0	0/0	0/0	0/0	0/0	0/0	0/0	0/0	0/1	0/0	0/1	0/1	0/1	0/0
Chromosome1-1445690 DEL	PRECISE	74	0	0	0/0	0/0	0/0	0/1	0/0	0/0	0/1	0/1	0/0	0/0	0/0	0/0	0/0	0/0
Chromosome1-1445773 DEL	PRECISE	918	0	0	0/0	0/0	0/0	0/0	0/0	0/0	0/0	0/0	0/1	0/0	0/1	0/1	0/1	0/0
Chromosome1-1447184 INS	PRECISE	54	0	0	0/0	0/0	0/0	0/0	0/0	0/0	0/0	0/0	1/1	0/0	0/1	1/1	0/1	0/0
Chromosome1-1448505 INS	PRECISE	12529	0	0	0/0	0/0	0/0	0/0	0/0	0/0	0/0	0/0	0/0	0/0	0/0	0/0	0/0	0/1
Chromosome1-1449188 INS	PRECISE	448	0	0.957	0/0	0/0	0/0	0/0	0/0	0/0	0/0	0/0	0/1	0/0	0/0	0/1	0/1	0/0
Chromosome1-1449797 DEL	PRECISE	330	0	0	0/0	0/0	0/0	0/0	0/0	0/0	0/0	0/0	0/1	0/0	0/1	0/1	1/1	0/0
Chromosome1-1450937 DEL	PRECISE	8133	0	0	0/0	0/1	0/1	0/0	1/1	0/1	0/0	0/0	0/0	0/1	0/0	0/0	0/0	0/1
Chromosome1-1451594 INS	PRECISE	2067	0	0	0/0	0/0	0/0	0/0	0/0	0/0	0/0	0/0	0/0	0/0	0/0	0/0	0/0	0/1
Chromosome1-1451831 INS	PRECISE	4570	0	0	0/0	0/0	0/0	0/0	0/0	0/0	0/0	0/0	0/0	0/0	0/0	0/0	0/0	1/1
Chromosome1-1451912 DEL	PRECISE	5466	0	0	0/0	0/0	0/0	0/0	0/0	0/0	0/0	0/0	1/1	0/0	0/1	1/1	1/1	0/0
Chromosome1-1452614 DEL	PRECISE	52	0	0	0/0	0/0	0/0	0/0	0/0	0/0	0/0	0/0	0/0	0/0	0/0	0/0	0/0	0/1
Chromosome1-1453237 INS	PRECISE	284	0	0.5	0/0	0/0	0/0	0/0	0/0	0/0	0/0	0/0	0/1	0/0	0/1	0/1	0/1	0/0
Chromosome1-1453805 DEL	PRECISE	883	0	0	0/0	0/0	0/0	0/0	0/0	0/0	0/0	0/0	0/1	0/0	0/1	0/1	0/1	0/0
Chromosome1-1454469 INS	PRECISE	211	0	0.5	0/0	0/0	0/0	0/0	0/0	0/0	0/0	0/0	0/1	0/0	0/1	0/1	0/1	0/0
Chromosome1-1454472 INS	PRECISE	5408	0	0	0/0	0/0	0/0	0/0	0/0	0/0	0/0	0/0	0/0	0/0	0/0	0/0	0/0	0/1
Chromosome1-1455112 INS	PRECISE	680	0	0	0/0	0/0	0/0	0/0	0/0	0/0	0/0	0/0	0/1	0/0	0/0	0/0	0/0	0/0
Chromosome1-1455143 DEL	PRECISE	187	0	0	0/0	0/0	0/0	0/0	0/0	0/0	0/0	0/0	0/0	0/0	0/1	0/1	0/1	0/0
Chromosome1-1456672 INS	PRECISE	150	0	0	0/0	0/0	0/0	0/0	0/0	0/0	0/0	0/0	0/0	0/0	0/0	0/0	0/0	0/1
Chromosome1-1456698 DEL	PRECISE	8623	0.577	0.577	0/0	0/0	0/0	0/0	0/0	0/0	0/0	0/0	0/1	0/0	0/1	0/1	0/1	0/0
Chromosome1-1457025 DEL	PRECISE	1015	0	0	0/0	0/0	0/0	0/1	0/0	0/0	0/1	0/1	0/0	0/0	0/0	0/0	0/0	0/1
Chromosome1-1457658 DEL	PRECISE	381	0	0	0/0	0/0	0/0	0/1	0/0	0/0	0/1	0/1	0/0	0/0	0/0	0/0	0/0	0/0
Chromosome1-1459165 DEL	PRECISE	101	0	0	0/0	0/0	0/0	0/0	0/0	0/0	0/0	0/0	0/1	0/0	0/1	0/1	0/1	0/0
Chromosome1-1459507 DEL	PRECISE	48165	0	0	0/0	0/0	0/0	0/0	0/0	0/0	0/0	0/0	0/0	0/0	0/0	0/0	0/0	1/1
Chromosome1-1459877 INS	PRECISE	137	0	0	0/0	0/0	0/0	0/1	0/0	0/0	0/1	0/1	0/0	0/0	0/0	0/0	0/0	0/0
Chromosome1-1460049 DEL	PRECISE	495	0	0	0/0	0/0	0/0	0/0	0/0	0/0	0/0	0/0	0/0	0/0	0/0	0/1	0/0	0/0
Chromosome1-1460050 DEL	PRECISE	6290	0	0	0/0	0/0	0/0	0/0	0/0	0/0	0/0	0/0	1/1	0/0	1/1	1/1	0/1	0/0
Chromosome1-1460334 BND	PRECISE	0	0	0	0/0	0/0	0/0	0/0	0/0	0/0	0/0	0/0	0/1	0/0	0/0	0/0	0/0	0/0
Chromosome1-1460895 DEL	PRECISE	165	0.5	0	0/0	0/0	0/0	0/0	0/0	0/0	0/0	0/0	0/1	0/0	0/1	0/1	0/1	0/0
Chromosome1-1461604 INS	PRECISE	68	0	0	0/0	0/0	0/0	0/0	0/0	0/0	0/0	0/0	0/1	0/0	0/0	0/0	0/0	0/0
Chromosome1-1461751 DEL	PRECISE	72	0	0	0/0	0/0	0/0	0/1	0/0	0/0	0/1	0/1	0/1	0/0	0/1	0/1	0/0	0/0
Chromosome1-1464253 DEL	PRECISE	53	0.5	0.5	0/0	0/0	0/0	0/0	0/0	0/0	0/0	0/0	0/1	0/0	0/1	0/1	0/1	0/0
Chromosome1-1465595 INS	PRECISE	1611	0	0	0/0	0/0	0/0	0/0	0/0	0/0	0/0	0/0	0/0	0/0	0/0	0/0	0/0	0/1
Chromosome1-1465774 INS	PRECISE	148	0	0	0/0	0/0	0/0	0/0	0/0	0/0	0/0	0/0	0/1	0/0	0/1	0/1	0/1	0/0
Chromosome1-1466137 DEL	PRECISE	1907	0	0	0/0	0/0	0/0	0/0	0/0	0/0	0/0	0/0	0/0	0/0	0/0	0/0	0/0	0/1
Chromosome1-1466783 DEL	PRECISE	55	0	0	0/0	0/0	0/0	0/0	0/0	0/0	0/0	0/0	0/1	0/0	0/1	0/1	0/1	0/0
Chromosome1-1468425 DEL	PRECISE	1051	0	0	0/0	0/0	0/0	1/1	0/1	0/1	0/0	0/0	1/1	0/0	0/0	0/0	0/0	0/0
Chromosome1-1469965 INS	PRECISE	57	0	0	0/0	0/0	0/0	0/0	0/0	0/0	0/0	0/0	0/0	0/0	0/0	0/0	0/0	0/1
Chromosome1-1469970 DEL	PRECISE	57	0	0	0/0	0/0	0/0	0/0	0/0	0/0	0/0	0/0	0/0	0/1	0/0	0/0	0/0	0/0
Chromosome1-1471335 DEL	PRECISE	57	0	0	0/0	0/1	0/1	0/0	0/1	1/1	0/0	0/0	0/0	0/1	0/0	0/0	0/0	0/1
Chromosome1-1471411 INS	PRECISE	5414	0	0	0/0	0/0	0/0	0/0	0/0	0/0	0/0	0/0	0/1	0/0	0/0	0/0	0/0	0/0
Chromosome1-1471616 INS	PRECISE	150	0	0	0/0	0/0	0/0	0/0	0/0	0/0	0/0	0/0	0/0	0/0	0/0	0/0	0/0	0/1
Chromosome1-1471913 DEL	PRECISE	61	0	0	0/0	0/0	0/0	0/0	0/0	0/0	0/0	0/0	0/1	0/0	0/1	0/1	0/1	0/0
Chromosome1-1472042 INS	PRECISE	72	0	0	0/0	0/0	0/0	0/1	0/0	0/0	0/1	1/1	0/1	0/0	0/1	0/1	0/1	0/1
Chromosome1-1472256 INS	PRECISE	60	0	0	0/0	0/0	0/0	0/0	0/0	0/0	0/0	0/0	0/1	0/0	0/1	0/1	0/1	0/0
Chromosome1-1472649 DEL	PRECISE	1259	0	0	0/0	0/1	0/1	0/0	0/1	0/1	0/0	0/0	0/0	0/1	0/0	0/0	0/0	0/1
Chromosome1-1473199 INS	PRECISE	105	0	0.5	0/0	0/0	0/0	0/0	0/0	0/0	0/0	0/0	0/1	0/0	0/1	0/1	0/1	0/0
Chromosome1-1473905 DEL	PRECISE	23407	0	0	0/0	0/0	0/0	1/1	0/0	0/0	1/1	1/1	0/0	0/0	0/0	0/0	0/0	0/0
Chromosome1-1474283 INS	PRECISE	7620	0	0	0/0	0/0	0/0	0/0	0/0	0/0	0/0	0/0	0/0	0/0	0/0	0/0	0/1	0/0
Chromosome1-1476943 DEL	PRECISE	13979	0	0	0/0	0/1	0/0	0/0	0/1	0/1	0/0	0/0	0/0	0/1	0/0	0/0	0/0	1/1
Chromosome1-1477532 DEL	PRECISE	916	0	0	0/0	0/0	0/0	0/1	0/0	0/0	0/0	0/1	0/0	0/0	0/0	0/0	0/0	0/0
Chromosome1-1477626 DEL	PRECISE	3675	0	0	0/0	0/0	0/0	0/1	0/0	0/0	0/0	0/1	0/0	0/0	0/0	0/0	0/0	0/0
Chromosome1-1478813 INS	PRECISE	374	0	0.726	0/0	0/0	0/0	0/1	0/1	1/1	0/0	0/1	0/0	0/0	0/0	0/0	0/0	1/1
Chromosome1-1480441 INS	PRECISE	375	0	0.667	0/0	0/1	0/0	0/1	0/1	0/1	0/1	0/1	0/0	0/1	0/0	0/0	0/0	1/1
Chromosome1-1481631 DEL	PRECISE	209	0	0	0/0	0/0	0/0	0/1	0/0	0/0	0/1	0/1	0/0	0/0	0/0	0/0	0/0	0/0

Chromosome1-1481813	DEL	PRECISE	183	0	0	0/0	0/0	0/0	0/1	0/0	0/0	0/1	0/1	0/0	0/0	0/0	0/0	0/0	0/0
Chromosome1-1482046	DEL	PRECISE	112	0	0	0/0	0/0	0/0	0/0	0/0	0/0	0/0	0/0	0/0	0/0	0/1	0/1	0/1	0/0
Chromosome1-1482327	DEL	PRECISE	2069	0	0	0/0	0/1	0/1	1/1	1/1	1/1	0/1	0/1	1/1	0/1	0/1	0/1	0/1	1/1
Chromosome1-1482790	BND	PRECISE	0	0	0	0/0	0/0	0/0	0/0	0/0	0/0	0/0	0/0	0/1	0/0	0/1	0/0	0/1	0/0
Chromosome1-1483230	DEL	PRECISE	128	0	0	0/0	0/0	0/0	0/0	0/0	0/0	0/0	0/0	0/1	0/0	0/1	0/1	0/1	0/0
Chromosome1-1483394	DEL	PRECISE	9755	0	0	0/0	0/1	0/1	1/1	1/1	1/1	0/1	1/1	1/1	0/1	0/1	0/1	0/0	0/0
Chromosome1-1483394	INS	PRECISE	9903	0	0	0/0	0/0	0/0	0/0	0/0	0/0	0/0	0/0	0/0	0/0	0/0	0/0	0/0	1/1
Chromosome1-1483394	INV	PRECISE	7309748	0	0	0/0	0/0	0/0	0/0	0/0	0/0	0/0	0/0	0/0	0/0	0/0	0/0	0/0	0/1
Chromosome1-1485279	INS	PRECISE	235	0	0.957	0/0	0/0	0/0	0/0	0/0	0/0	0/0	0/0	0/1	0/0	0/1	0/1	0/1	0/0
Chromosome1-1485351	INS	PRECISE	5049	0	0	0/0	0/0	0/0	0/0	0/0	0/0	0/0	0/0	0/0	0/0	0/0	0/0	0/0	1/1
Chromosome1-1485546	DEL	PRECISE	64	0	0	0/0	0/0	0/0	0/0	0/0	0/0	0/0	0/0	0/1	0/0	0/1	0/1	0/1	0/0
Chromosome1-1485939	DEL	PRECISE	6435	0.5	0.5	0/0	0/0	0/0	0/0	0/0	0/0	0/0	0/0	0/1	0/0	0/1	0/1	0/1	0/0
Chromosome1-1486979	INS	PRECISE	176	0	0.5	0/0	0/0	0/0	0/0	0/0	0/0	0/0	0/0	0/1	0/0	0/1	0/1	0/1	0/0
Chromosome1-1488172	BND	PRECISE	0	0	0	0/0	0/0	0/0	0/0	0/0	0/0	0/0	0/0	0/0	0/0	0/0	0/0	0/0	0/1
Chromosome1-1488202	DEL	PRECISE	388	0	0	0/0	0/0	0/0	0/0	0/0	0/0	0/0	0/0	0/1	0/0	0/1	0/1	0/1	0/0
Chromosome1-1489360	DEL	PRECISE	50	0	0	0/0	0/0	0/0	0/0	0/0	0/0	0/0	0/0	0/1	0/0	0/1	0/1	0/1	0/0
Chromosome1-1489808	DEL	PRECISE	2297	0	0	0/0	0/0	0/0	0/0	0/0	0/0	0/0	0/0	0/0	0/0	0/0	0/0	0/0	0/1
Chromosome1-1490704	DEL	PRECISE	180	0	0	0/0	0/1	0/1	0/0	0/1	0/1	0/0	0/0	0/0	0/1	0/0	0/0	0/0	0/1
Chromosome1-1490707	DEL	PRECISE	12323	0	0	0/0	0/0	0/0	0/0	0/0	0/0	0/0	0/0	0/1	0/0	0/1	0/1	0/1	0/0
Chromosome1-1491400	DEL	PRECISE	57	0	0	0/0	0/0	0/0	0/1	0/0	0/0	0/0	0/0	0/0	0/0	0/0	0/0	0/0	0/0
Chromosome1-1492107	DEL	PRECISE	5485	0	0	0/0	0/0	0/0	0/0	0/0	0/0	0/0	0/0	0/1	0/0	0/1	0/1	0/1	0/0
Chromosome1-1492128	DEL	PRECISE	498	0	0	0/0	0/1	0/1	0/0	0/1	0/1	0/0	0/0	0/0	0/1	0/0	0/0	0/0	0/1
Chromosome1-1492882	DEL	PRECISE	150	0	0	0/0	0/0	0/0	0/0	0/0	0/0	0/0	0/0	0/1	0/0	0/1	0/1	0/1	0/0
Chromosome1-1493539	INS	PRECISE	324	0	0.816	0/0	0/0	0/0	0/0	0/0	0/0	0/0	0/0	0/1	0/0	0/1	0/1	0/1	0/0
Chromosome1-1493816	DEL	PRECISE	128	0	0	0/0	0/0	0/0	0/0	0/0	0/0	0/0	0/0	0/1	0/0	0/1	0/1	0/1	0/0
Chromosome1-1494029	DEL	PRECISE	91	0	0	0/0	0/0	0/0	0/0	0/0	0/0	0/0	0/0	0/1	0/0	0/1	0/1	0/1	0/0
Chromosome1-1495586	INS	PRECISE	895	0	0.577	0/0	0/0	0/0	0/0	0/0	0/0	0/0	0/0	0/1	0/0	0/1	0/1	0/1	0/0
Chromosome1-1496197	DEL	PRECISE	94	0	0	0/0	0/0	0/0	0/0	0/0	0/0	0/0	0/0	0/1	0/0	0/1	0/1	1/1	0/0
Chromosome1-1496754	DEL	PRECISE	1340	0	0	0/0	0/0	0/0	0/0	0/0	0/0	0/0	0/0	0/1	0/0	0/1	0/1	0/1	0/0
Chromosome1-1497052	DEL	PRECISE	745	0	0	0/0	0/0	0/0	0/0	0/0	0/0	0/0	0/0	0/1	0/0	0/1	0/1	0/1	0/0
Chromosome1-1497415	DEL	PRECISE	114	0	0	0/0	0/0	0/0	0/0	0/0	0/0	0/0	0/0	0/1	0/0	0/1	0/1	0/1	0/0
Chromosome1-1497912	DEL	PRECISE	85	0	0	0/0	0/0	0/0	0/0	0/0	0/0	0/0	0/0	0/1	0/0	0/1	0/1	0/1	0/0
Chromosome1-1498709	INS	PRECISE	154	0	0	0/0	0/0	0/0	0/0	0/0	0/0	0/0	0/0	0/1	0/0	0/1	0/1	0/1	0/0
Chromosome1-1498784	DEL	PRECISE	80	0	0	0/0	0/0	0/0	0/0	0/0	0/0	0/0	0/0	0/1	0/0	0/1	0/1	0/1	0/0
Chromosome1-1499284	INS	PRECISE	84	0	0.5	0/0	0/0	0/0	0/0	0/0	0/0	0/0	0/0	0/1	0/0	0/1	0/1	0/0	0/0
Chromosome1-1500233	INS	PRECISE	1184	0	0	0/0	0/0	0/0	0/0	0/0	0/0	0/0	0/0	0/1	0/0	0/0	0/0	0/0	0/0
Chromosome1-1500317	DEL	PRECISE	141	0	0	0/0	0/0	0/0	0/0	0/0	0/0	0/0	0/0	0/0	0/0	0/0	0/0	0/0	0/1
Chromosome1-1500432	INS	PRECISE	130	0	0.577	0/0	0/0	0/0	0/1	0/0	0/0	0/1	0/1	0/0	0/0	0/0	0/0	0/0	0/0
Chromosome1-1502509	DEL	PRECISE	703	0.316	0.316	0/0	0/1	0/1	0/0	0/1	0/1	0/0	0/0	0/1	0/1	0/1	0/1	0/0	0/1
Chromosome1-1502890	DEL	PRECISE	2881	0	0	0/0	0/1	0/1	0/1	1/1	1/1	0/0	0/0	1/1	0/1	0/1	0/1	0/1	1/1
Chromosome1-1503556	DEL	PRECISE	62	0	0	0/0	0/0	0/0	0/1	0/0	0/0	0/1	1/1	0/0	0/0	0/0	0/0	0/0	0/0
Chromosome1-1503650	INS	PRECISE	331	0	0	0/0	0/0	0/0	0/0	0/0	0/0	0/0	0/0	0/0	0/0	0/0	0/0	0/0	0/1
Chromosome1-1503843	INS	PRECISE	84	0	0	0/0	0/0	0/0	0/0	0/0	0/0	0/0	0/0	0/0	0/0	0/0	0/0	0/0	0/1
Chromosome1-1504132	DEL	PRECISE	273	0	0	0/0	0/0	0/0	0/0	0/0	0/0	0/0	0/0	0/0	0/0	0/0	0/0	0/0	0/1
Chromosome1-1504939	DEL	PRECISE	222	0	0	0/0	0/0	0/0	0/0	0/0	0/0	0/0	0/0	0/1	0/0	0/1	0/1	0/1	0/0
Chromosome1-1505061	INS	PRECISE	3277	0	0.577	0/0	0/0	0/0	0/0	0/0	0/0	0/0	0/0	1/1	0/0	1/1	1/1	1/1	0/0
Chromosome1-1505307	BND	PRECISE	0	0	0	0/0	0/0	0/0	0/1	0/0	0/1	0/0	0/0	0/1	0/0	0/0	0/0	0/0	0/0
Chromosome1-1505479	INS	PRECISE	1439	0.816	0.753	0/0	0/1	0/1	0/0	0/1	0/1	0/0	0/0	0/0	0/1	0/0	0/0	0/0	0/1
Chromosome1-1505483	INS	PRECISE	203	0	0.5	0/0	0/0	0/0	0/0	0/0	0/0	0/0	0/0	0/1	0/0	0/1	0/1	1/1	0/0
Chromosome1-1505618	DEL	PRECISE	91	0	0	0/0	0/1	0/1	0/0	0/1	0/1	0/0	0/0	0/0	0/1	0/0	0/0	0/0	0/1
Chromosome1-1506268	DEL	PRECISE	658	0	0	0/0	0/1	0/1	0/0	0/1	0/1	0/0	0/0	0/1	0/1	0/1	0/1	0/1	0/1
Chromosome1-1506567	INS	PRECISE	1462	0	0.957	0/0	0/0	0/0	0/0	0/0	0/0	0/0	0/0	0/1	0/0	0/1	0/1	0/1	0/0
Chromosome1-1508013	INS	PRECISE	85	0	0	0/0	0/1	0/1	0/0	0/1	0/1	0/0	0/0	0/1	0/1	0/1	0/1	0/1	0/1
Chromosome1-1508318	DEL	PRECISE	177	0	0	0/0	0/0	0/0	0/0	0/0	0/0	0/0	0/0	0/1	0/0	0/0	0/1	0/1	0/0
Chromosome1-1508459	DEL	PRECISE	441	0	0	0/0	0/0	0/0	0/0	0/0	0/0	0/0	0/0	1/1	0/0	0/1	1/1	1/1	0/0
Chromosome1-1508736	DEL	PRECISE	56	0.5	0.5	0/0	0/0	0/0	0/0	0/0	0/0	0/0	0/0	0/1	0/0	0/1	0/1	0/1	0/0
Chromosome1-1510140	DEL	PRECISE	81	0	0	0/0	0/0	0/0	0/0	0/0	0/0	0/0	0/0	0/0	0/0	0/1	0/1	0/1	0/0
Chromosome1-1510294	DEL	PRECISE	168	0	0	0/0	0/0	0/0	0/0	0/0	0/0	0/0	0/0	0/1	0/0	0/1	0/1	0/1	0/0
Chromosome1-1510853	DEL	PRECISE	4595	0	0	0/0	0/1	0/1	1/1	1/1	1/1	0/0	0/1	1/1	0/1	0/0	0/0	0/0	1/1
Chromosome1-1511315	DEL	PRECISE	892	0	0	0/0	0/1	0/1	1/1	1/1	1/1	0/0	0/1	1/1	0/1	0/0	0/0	0/0	1/1
Chromosome1-1511568	INS	PRECISE	366	0	0	0/0	0/0	0/0	0/0	0/0	0/0	0/0	0/0	0/0	0/0	0/0	0/0	0/0	0/1
Chromosome1-1512068	DEL	PRECISE	317	0	0	0/0	0/0	0/0	0/0	0/0	0/0	0/0	0/0	0/1	0/0	0/1	0/1	0/1	0/0
Chromosome1-1512183	BND	PRECISE	0	0	0	0/0	0/0	0/0	0/0	0/0	0/0	0/1	0/1	0/0	0/0	0/0	0/0	0/0	0/0
Chromosome1-1512574	DEL	PRECISE	2218	0.5	0	0/0	0/0	0/0	0/0	0/0	0/0	0/0	0/0	0/1	0/0	1/1	0/1	0/1	0/0
Chromosome1-1512604	DEL	PRECISE	17795	0	0	0/0	0/0	0/0	0/0	0/0	0/0	0/0	0/0	0/0	0/0	0/0	0/0	0/0	0/1
Chromosome1-1513160	DEL	PRECISE	362	0	0	0/0	0/0	0/0	0/1	0/1	0/1	0/1	0/1	1/1	0/0	0/0	0/0	0/0	0/0
Chromosome1-1514019	DEL	PRECISE	73	0	0	0/0	0/0	0/0	0/1	0/1	0/1	0/0	0/0	1/1	0/0	0/0	0/0	0/0	0/0
Chromosome1-1515010	DEL	PRECISE	142	0	0	0/0	0/0	0/0	0/1	0/1	0/1	0/1	0/1	0/1	0/0	0/0	0/0	0/0	0/0
Chromosome1-1516284	DEL	PRECISE	344	0	0	0/0	0/1	0/1	1/1	1/1	1/1	0/1	0/1	1/1	0/1	0/0	0/0	0/0	1/1
Chromosome1-1517169	INS	PRECISE	339	0	0	0/0	0/1	0/1	0/0	1/1	1/1	0/0	0/0	0/0	0/1	0/0	0/0	0/0	1/1
Chromosome1-1517645	INS	PRECISE	7420	0	0	0/0	0/0	0/0	0/1	0/0	0/0	0/0	0/0	0/0	0/0	0/0	0/0	0/0	0/0
Chromosome1-1518147	DEL	PRECISE	86	0	0	0/0	0/1	0/1	0/0	0/1	0/1	0/0	0/0	0/0	0/1	0/0	0/0	0/0	0/1
Chromosome1-1519568	DEL	PRECISE	55	0	0	0/0	0/1	0/1	0/0	0/1	0/1	0/0	0/0	0/0	0/1	0/0	0/0	0/0	0/1

Chromosome1-1520001	DEL	PRECISE	4493	0	0	0/0	0/0	0/0	0/0	0/0	0/0	0/0	0/0	0/1	0/0	0/1	0/1	0/1	0/0
Chromosome1-1520930	DEL	PRECISE	58	0	0	0/0	0/0	0/0	0/0	0/0	0/0	0/0	0/0	0/0	0/0	0/0	0/0	0/0	0/1
Chromosome1-1521863	INS	PRECISE	1333	0	0.577	0/0	0/0	0/0	0/0	0/0	0/0	0/0	0/0	0/1	0/0	0/1	0/1	0/1	0/0
Chromosome1-1521888	DEL	PRECISE	627	0	0	0/0	0/1	0/1	0/0	0/1	0/1	0/0	0/0	0/0	0/1	0/0	0/0	0/0	0/1
Chromosome1-1522929	DEL	PRECISE	58	0	0	0/0	0/0	0/0	0/0	0/0	0/0	0/0	0/0	0/1	0/0	0/1	0/1	0/1	0/0
Chromosome1-1523156	DEL	PRECISE	67	0	0	0/0	0/0	0/0	0/0	0/0	0/0	0/0	0/0	0/1	0/0	0/1	0/1	0/1	0/0
Chromosome1-1524830	DEL	PRECISE	5907	0	0	0/0	0/0	0/0	0/0	0/0	0/0	0/0	0/0	0/0	0/0	0/0	1/1	0/1	0/0
Chromosome1-1525561	DEL	PRECISE	625	0	0	0/0	0/0	0/0	0/0	0/0	0/0	0/0	0/0	0/1	0/0	0/1	0/1	0/1	0/0
Chromosome1-1525587	INS	PRECISE	5158	0	0	0/0	0/0	0/0	0/0	0/0	0/0	0/0	0/0	0/0	0/0	0/0	0/0	0/0	1/1
Chromosome1-1526057	DEL	PRECISE	110	0	0	0/0	0/0	0/0	0/1	0/1	0/1	0/0	0/0	0/1	0/0	0/0	0/0	0/0	0/0
Chromosome1-1526360	DEL	PRECISE	2465	0	0	0/0	0/0	0/0	0/0	0/0	0/0	0/0	0/0	0/0	0/0	0/0	0/0	0/0	0/1
Chromosome1-1526367	DEL	PRECISE	53	0	0	0/0	0/1	0/1	0/0	0/1	0/1	0/0	0/0	0/0	0/1	0/0	0/0	0/0	0/1
Chromosome1-1526480	DEL	PRECISE	62	0	0	0/0	0/0	0/0	0/0	0/0	0/0	0/0	0/0	0/1	0/0	0/1	0/1	0/1	0/0
Chromosome1-1527346	INS	PRECISE	178	0	0	0/0	0/0	0/0	0/0	0/0	0/0	0/0	0/0	0/1	0/0	0/1	0/1	0/1	0/0
Chromosome1-1528981	INS	PRECISE	197	0	0.675	0/0	0/1	0/1	1/1	1/1	1/1	0/1	0/1	1/1	0/1	0/0	0/0	0/0	1/1
Chromosome1-1529579	DEL	PRECISE	454	0	0	0/0	0/0	0/0	1/1	0/1	0/1	0/1	0/1	1/1	0/0	0/0	0/0	0/0	0/1
Chromosome1-1530875	INS	PRECISE	87	0.277	0	0/1	0/0	0/1	0/1	0/1	0/1	0/1	0/1	0/1	0/1	0/1	0/0	0/1	0/0
Chromosome1-1530937	DEL	PRECISE	319	0	0	0/0	0/0	0/0	0/0	0/0	0/0	0/0	0/0	0/0	0/0	0/0	0/0	0/1	0/0
Chromosome1-1532353	INS	PRECISE	57	0	0	0/0	0/0	0/0	0/1	1/1	1/1	0/1	0/1	1/1	0/0	0/0	0/0	0/0	1/1
Chromosome1-1532451	DEL	PRECISE	57	0	0	0/0	0/0	0/0	0/0	0/0	0/0	0/0	0/0	0/0	0/0	0/0	0/0	0/0	0/1
Chromosome1-1533023	DEL	PRECISE	133	0	0	0/0	0/0	0/0	0/0	0/0	0/0	0/0	0/0	0/0	0/0	0/0	0/0	0/0	0/1
Chromosome1-1536199	DEL	PRECISE	162	0	0	0/0	0/1	0/1	1/1	1/1	1/1	0/1	0/1	1/1	0/1	0/0	0/0	0/0	1/1
Chromosome1-1536235	INS	PRECISE	4492	0	0	0/0	0/0	0/0	0/0	0/0	0/0	0/0	0/0	0/0	0/0	0/0	0/0	0/0	0/1
Chromosome1-1536392	DEL	PRECISE	1006	0	0	0/0	0/0	0/0	0/0	0/0	0/0	0/0	0/0	0/0	0/0	0/1	0/1	0/1	0/0
Chromosome1-1536553	DEL	PRECISE	109	0	0	0/0	0/0	0/0	0/0	0/0	0/0	0/0	0/0	0/1	0/0	0/1	0/1	0/1	0/0
Chromosome1-1537474	DEL	PRECISE	175	0	0	0/0	0/0	0/0	0/0	0/0	0/0	0/0	0/0	0/0	0/0	0/0	0/0	0/0	0/1
Chromosome1-1537638	INS	PRECISE	75	0	0	0/0	0/0	0/0	0/1	0/0	0/0	0/0	0/0	0/0	0/0	0/0	0/0	0/0	0/0
Chromosome1-1537841	BND	PRECISE	0	0	0	0/0	0/0	0/0	0/1	0/1	0/1	0/0	0/0	0/0	0/0	0/0	0/0	0/0	0/0
Chromosome1-1538416	DEL	PRECISE	332	0	0	0/0	0/0	0/0	0/0	0/0	0/0	0/0	0/0	0/1	0/0	0/0	0/1	0/1	0/0
Chromosome1-1538613	INS	PRECISE	139	0	0.957	0/0	0/0	0/0	0/0	0/0	0/0	0/0	0/0	0/1	0/0	0/1	0/1	0/1	0/0
Chromosome1-1538639	DEL	PRECISE	208	0	0	0/0	0/0	0/0	0/0	0/0	0/0	0/0	0/0	0/1	0/0	0/0	0/1	0/1	0/0
Chromosome1-1538746	INS	PRECISE	211	0	0	0/0	0/0	0/0	0/0	0/0	0/0	0/0	0/0	0/0	0/0	0/0	0/0	0/0	0/1
Chromosome1-1539143	DEL	PRECISE	3096	0	0	0/0	0/0	0/0	0/0	0/0	0/0	0/0	0/0	0/1	0/0	0/1	0/1	0/1	0/0
Chromosome1-1540928	DEL	PRECISE	136	0	0	0/0	0/0	0/0	0/0	0/0	0/0	0/0	0/0	0/1	0/0	0/1	0/1	0/0	0/0
Chromosome1-1541050	INS	PRECISE	16543	0	0	0/0	0/0	0/0	1/1	0/0	0/0	0/0	0/0	0/0	0/0	0/0	0/0	0/0	0/0
Chromosome1-1541467	INS	PRECISE	76	0	0.5	0/0	0/0	0/0	0/0	0/0	0/0	0/0	0/0	0/1	0/0	0/1	0/1	0/1	0/0
Chromosome1-1542234	DEL	PRECISE	12518	0	0	0/0	0/1	0/1	1/1	1/1	1/1	0/1	0/1	1/1	0/1	0/0	0/0	0/0	1/1
Chromosome1-1543823	DEL	PRECISE	444	0	0	0/0	0/0	0/0	0/0	0/0	0/0	0/0	0/0	0/0	0/0	0/0	0/0	0/0	0/1
Chromosome1-1544011	INS	PRECISE	112	0	0.577	0/0	0/0	0/0	1/1	0/1	0/1	0/0	0/0	1/1	0/0	0/0	0/0	0/0	0/0
Chromosome1-1544386	DEL	PRECISE	421	0	0	0/0	0/0	0/0	0/0	0/0	0/0	0/0	0/0	0/0	0/0	0/0	0/0	0/0	0/1
Chromosome1-1545609	BND	PRECISE	0	0	0	0/0	0/0	0/0	0/0	0/1	0/1	0/0	0/0	0/0	0/1	0/0	0/0	0/0	0/1
Chromosome1-1546242	DEL	PRECISE	178	0.707	0	0/0	0/0	0/0	0/0	0/0	0/0	0/0	0/0	0/1	0/0	0/0	0/0	0/0	0/0
Chromosome1-1546873	DEL	PRECISE	771	0	0	0/0	0/0	0/0	0/0	0/0	0/0	0/0	0/0	0/1	0/0	0/1	0/0	1/1	0/0
Chromosome1-1547101	DEL	PRECISE	847	0.894	0	0/0	0/0	0/0	0/0	0/0	0/0	0/0	0/0	0/1	0/0	0/1	0/0	1/1	1/1
Chromosome1-1547302	INS	PRECISE	132	0	0	0/0	0/0	0/0	0/0	0/0	0/0	0/0	0/0	0/0	0/0	0/0	0/0	0/0	1/1
Chromosome1-1547363	DEL	PRECISE	130	0	0	0/0	0/0	0/0	0/0	0/0	0/0	0/0	0/0	0/1	0/0	0/1	0/0	1/1	0/0
Chromosome1-1548205	DEL	PRECISE	9795	0.408	0.408	0/0	0/1	0/1	0/0	0/1	0/1	0/0	0/0	0/0	0/1	0/0	0/0	0/0	0/1
Chromosome1-1548864	DEL	PRECISE	9774	0	0	0/0	0/0	0/0	0/0	0/0	0/0	0/0	0/0	0/1	0/0	1/1	0/1	1/1	0/0
Chromosome1-1549307	INS	PRECISE	52	0	0	0/0	0/1	0/1	0/0	0/0	0/1	0/0	0/0	0/0	0/1	0/0	0/0	0/0	1/1
Chromosome1-1549522	INS	PRECISE	179	0	0	0/0	0/0	0/0	0/1	1/1	0/1	0/0	0/0	1/1	0/0	0/0	0/0	0/0	0/0
Chromosome1-1549993	DEL	PRECISE	147	0	0	0/0	0/0	0/0	0/0	0/0	0/0	0/0	0/0	0/1	0/0	0/1	0/1	0/1	0/0
Chromosome1-1550019	INS	PRECISE	161	0	0	0/0	0/0	0/0	0/1	0/1	0/1	0/1	0/1	0/1	0/0	0/0	0/0	0/0	0/0
Chromosome1-1550419	INS	PRECISE	11959	0	0	0/0	0/0	0/0	0/1	0/0	0/0	0/0	0/0	0/0	0/0	0/0	0/0	0/0	0/0
Chromosome1-1550965	DEL	PRECISE	5171	0	0	0/0	0/0	0/0	0/0	0/0	0/0	0/0	0/0	0/1	0/0	0/1	0/1	0/1	0/0
Chromosome1-1551829	DEL	PRECISE	425	0	0	0/0	0/0	0/0	0/0	0/0	0/0	0/0	0/0	0/1	0/0	0/1	0/1	0/1	0/0
Chromosome1-1552381	INS	PRECISE	1101	0	0	0/0	0/0	0/0	0/0	0/0	0/0	0/0	0/0	0/0	0/0	0/0	0/0	0/0	0/1
Chromosome1-1552883	INS	PRECISE	623	0	0	0/0	0/0	0/0	0/0	0/0	0/0	0/0	0/0	0/0	0/0	0/0	0/0	0/0	0/1
Chromosome1-1553388	DEL	PRECISE	51	0	0	0/0	0/0	0/0	0/0	0/0	0/0	0/0	0/0	0/0	0/0	0/1	0/0	0/1	0/0
Chromosome1-1553696	DEL	PRECISE	523	0	0	0/0	0/0	0/0	0/1	0/1	0/0	0/0	0/0	0/1	0/0	0/0	0/0	0/0	0/0
Chromosome1-1553766	DEL	PRECISE	14469	0.5	0.5	0/0	0/0	0/0	0/1	0/1	1/1	0/0	0/0	1/1	0/0	0/0	0/0	0/0	0/0
Chromosome1-1554091	INS	PRECISE	1478	0	0	0/0	0/0	0/0	0/0	0/0	0/0	0/0	0/0	0/0	0/0	0/0	0/0	0/0	0/1
Chromosome1-1554729	DEL	PRECISE	60	0	0	0/0	0/0	0/0	0/0	0/0	0/0	0/0	0/0	0/0	0/0	0/0	0/0	0/0	0/1
Chromosome1-1554763	DEL	PRECISE	993	0.447	0.447	0/0	0/0	0/0	0/0	0/0	0/0	0/0	0/0	0/1	0/0	0/1	0/1	0/1	0/1
Chromosome1-1555080	DEL	PRECISE	1334	0	0	0/0	0/0	0/0	0/0	0/0	0/0	0/0	0/0	0/1	0/0	0/1	0/1	0/1	0/0
Chromosome1-1555310	DEL	PRECISE	641	0	0	0/0	0/0	0/0	0/0	0/0	0/0	0/0	0/0	0/0	0/0	0/1	0/0	0/1	0/0
Chromosome1-1555555	DEL	PRECISE	10911	0.302	0	0/0	0/1	0/1	0/1	1/1	1/1	0/0	0/0	1/1	0/1	0/1	0/1	1/1	1/1
Chromosome1-1556187	DEL	PRECISE	587	0	0	0/0	0/0	0/0	0/1	0/0	0/0	0/1	0/1	0/0	0/0	0/0	0/0	0/0	0/0
Chromosome1-1556993	BND	PRECISE	0	0	0	0/0	0/0	0/0	0/0	0/0	0/0	0/0	0/0	0/0	0/0	0/0	0/0	1/1	0/0
Chromosome1-1557687	INS	PRECISE	62	0	0	0/0	0/0	0/0	0/0	0/0	0/0	0/0	0/0	0/1	0/0	0/0	0/0	0/0	0/0
Chromosome1-1557941	DEL	PRECISE	287	0.632	0.632	0/0	0/1	0/1	0/0	0/1	0/1	0/0	0/0	0/1	0/1	0/0	0/0	0/0	1/1
Chromosome1-1558781	INS	PRECISE	370	0	0.5	0/0	0/0	0/0	0/0	0/0	0/0	0/0	0/0	0/1	0/0	0/1	0/1	0/1	0/0
Chromosome1-1558916	DEL	PRECISE	108	0	0	0/0	0/0	0/0	0/0	0/0	0/0	0/0	0/0	0/1	0/0	0/1	0/1	0/1	0/1
Chromosome1-1559355	DEL	PRECISE	1340	0	0	0/0	0/0	0/0	0/0	0/0	0/0	0/0	0/0	0/1	0/0	0/1	0/1	0/1	0/0

Chromosome1-1560655 DEL	PRECISE	83	0	0	0/0	0/0	0/0	0/1	0/0	0/0	0/0	0/0	0/0	0/0	0/0	0/0	0/0	0/0
Chromosome1-1561295 DEL	PRECISE	5295	0	0	0/0	0/0	0/0	0/0	0/0	0/0	0/0	0/0	0/1	0/0	0/1	0/1	0/1	0/0
Chromosome1-1562202 INS	PRECISE	105	0	0	0/0	0/0	0/0	0/1	0/0	0/0	0/0	0/0	0/0	0/0	0/0	0/0	0/0	0/0
Chromosome1-1562231 INS	PRECISE	1480	0	0.548	0/0	0/1	0/1	0/0	0/1	0/1	0/0	0/0	0/0	0/1	0/0	0/0	0/0	0/1
Chromosome1-1562292 DEL	PRECISE	954	0	0	0/0	0/0	0/0	0/0	0/0	0/0	0/0	0/0	0/1	0/0	0/1	0/1	0/1	0/0
Chromosome1-1562303 BND	PRECISE	0	0	0	0/0	0/0	0/0	0/0	0/1	0/0	0/0	0/0	0/0	0/0	0/0	0/0	0/0	0/0
Chromosome1-1562464 INS	PRECISE	6752	0	0	0/0	0/0	0/0	0/0	0/0	0/0	0/0	0/0	0/0	0/0	0/0	0/0	0/0	0/1
Chromosome1-1562560 INS	PRECISE	1555	0	0.516	0/0	0/1	0/1	0/0	0/1	0/1	0/0	0/0	0/0	0/1	0/0	0/0	0/0	0/1
Chromosome1-1562913 INS	PRECISE	70	0	0.5	0/0	0/0	0/0	0/1	0/0	0/0	0/1	0/1	0/0	0/0	0/0	0/0	0/0	0/1
Chromosome1-1563221 DEL	PRECISE	471	0	0	0/0	0/0	0/0	0/1	0/1	0/1	0/0	0/0	0/1	0/0	0/0	0/0	0/0	0/0
Chromosome1-1565008 DEL	PRECISE	487	0	0	0/0	0/0	0/0	0/0	0/0	0/0	0/0	0/0	0/1	0/0	0/0	0/0	0/0	0/0
Chromosome1-1566021 INS	PRECISE	1392	0	0.707	0/0	0/0	0/0	0/0	0/0	0/0	0/0	0/0	0/0	0/0	0/1	0/0	0/1	0/0
Chromosome1-1566155 INS	PRECISE	137	0	0.577	0/0	0/0	0/0	0/1	0/1	0/1	0/0	0/0	0/1	0/0	0/0	0/0	0/0	0/0
Chromosome1-1567260 INS	PRECISE	142	0	0	0/0	0/0	0/0	0/0	0/0	0/0	0/0	0/0	0/0	0/0	0/0	0/0	0/0	0/1
Chromosome1-1568373 INS	PRECISE	61	0	0	0/0	0/1	0/1	0/0	0/1	0/1	0/0	0/0	0/0	0/1	0/0	0/0	0/0	1/1
Chromosome1-1568595 INV	PRECISE	377675	0	0	0/0	0/0	0/0	0/0	0/0	0/0	0/0	0/0	0/1	0/0	0/0	0/0	0/0	0/0
Chromosome1-1570574 INS	PRECISE	1680	0	0	0/0	0/0	0/0	0/0	0/0	0/0	0/0	0/0	0/1	0/0	0/0	0/0	0/0	0/0
Chromosome1-1570651 INS	PRECISE	89	0	0.577	0/0	0/0	0/0	0/0	0/0	0/0	0/0	0/0	0/1	0/0	0/1	0/1	0/1	0/0
Chromosome1-1571220 INS	PRECISE	886	0	0	0/0	0/0	0/0	0/0	0/0	0/0	0/0	0/0	0/0	0/0	0/0	0/0	0/0	0/1
Chromosome1-1571681 DEL	PRECISE	1374	0	0	0/0	0/0	0/0	0/0	0/0	0/0	0/0	0/0	0/1	0/0	0/1	0/1	0/1	0/0
Chromosome1-1571914 DEL	PRECISE	313	0	0	0/0	0/1	0/1	0/0	0/1	0/1	0/0	0/0	0/0	0/1	0/0	0/0	0/0	0/1
Chromosome1-1572905 DEL	PRECISE	56	0	0	0/0	0/0	0/0	0/0	0/0	0/0	0/0	0/0	0/0	0/0	0/0	0/0	0/0	0/1
Chromosome1-1573929 INS	PRECISE	99	0	0	0/0	0/0	0/0	0/0	0/0	0/0	0/0	0/0	0/1	0/0	0/0	0/0	0/0	0/0
Chromosome1-1575187 INS	PRECISE	63	0	0.707	0/0	0/0	0/0	0/0	0/0	0/0	0/0	0/0	0/1	0/0	0/0	0/0	0/0	0/0
Chromosome1-1575260 DEL	PRECISE	63	0	0	0/0	0/0	0/0	0/0	0/0	0/0	0/0	0/0	0/1	0/0	0/1	0/0	0/0	0/0
Chromosome1-1575965 DEL	PRECISE	80	0.577	0.577	0/0	0/0	0/0	0/0	0/0	0/0	0/0	0/0	0/1	0/0	0/1	0/0	0/1	0/0
Chromosome1-1577823 BND	PRECISE	0	0	0	0/0	0/0	0/0	0/0	0/0	0/0	0/0	0/0	0/0	0/0	0/1	0/0	0/0	0/0
Chromosome1-1577984 BND	PRECISE	0	0	0	0/0	0/0	0/0	0/0	0/0	0/0	0/0	0/0	0/0	0/0	0/1	0/0	0/0	0/0
Chromosome1-1578853 DEL	PRECISE	61	0	0	0/0	0/0	0/0	0/0	0/0	0/0	0/0	0/0	0/0	0/0	0/0	0/0	0/0	0/1
Chromosome1-1578930 DEL	PRECISE	862	0.577	0.577	0/0	0/0	0/0	0/0	0/0	0/0	0/0	0/0	0/0	0/0	0/1	0/1	0/1	0/0
Chromosome1-1579115 DEL	PRECISE	55	0	0	0/0	0/0	0/0	0/0	0/0	0/0	0/0	0/0	0/0	0/0	0/0	0/0	0/0	0/1
Chromosome1-1579542 DEL	PRECISE	1130	0	0	0/0	0/0	0/0	0/0	0/0	0/0	0/0	0/0	0/0	0/0	0/1	0/1	0/1	0/0
Chromosome1-1579954 DEL	PRECISE	789	0	0	0/0	0/0	0/0	0/0	0/0	0/0	0/0	0/0	0/0	0/0	0/0	0/0	0/0	0/1
Chromosome1-1579990 INS	PRECISE	795	0	0.577	0/0	0/0	0/0	0/0	0/0	0/0	0/0	0/0	0/0	0/0	0/1	0/1	0/1	0/0
Chromosome1-1580260 INS	PRECISE	214	0	0.408	0/0	0/1	0/1	0/0	0/1	0/0	0/0	0/0	0/0	0/1	0/0	0/0	0/0	0/1
Chromosome1-1580264 DEL	PRECISE	584	0	0	0/0	0/0	0/0	0/0	0/0	0/0	0/0	0/0	0/0	0/0	0/1	0/1	0/1	0/0
Chromosome1-1581030 DEL	PRECISE	59	0	0	0/0	0/0	0/0	0/1	0/1	0/1	0/1	0/1	1/1	0/0	0/0	0/0	0/0	0/0
Chromosome1-1582079 INS	PRECISE	5958	0	0	0/0	0/0	0/0	0/0	0/0	0/0	0/0	0/0	0/0	0/0	0/0	0/0	0/0	1/1
Chromosome1-1582788 DEL	PRECISE	1926	0	0	0/0	0/1	0/1	0/1	0/1	0/1	0/0	0/0	0/1	0/1	0/1	0/1	0/1	1/1
Chromosome1-1583351 BND	PRECISE	0	0	0	0/0	0/0	0/0	0/0	0/1	0/0	0/0	0/0	0/0	0/0	0/0	0/0	0/0	0/0
Chromosome1-1583705 DEL	PRECISE	72	0	0	0/0	0/0	0/0	0/1	0/0	0/0	0/0	0/0	0/0	0/0	0/0	0/0	0/0	0/0
Chromosome1-1583966 DEL	PRECISE	1361	0	0	0/0	0/1	0/1	1/1	1/1	1/1	1/1	0/1	1/1	0/1	0/0	0/0	0/0	1/1
Chromosome1-1584669 INS	PRECISE	2078	0	0	0/0	0/0	0/0	0/1	0/0	0/0	0/0	0/0	0/0	0/0	0/0	0/0	0/0	0/0
Chromosome1-1584692 INS	PRECISE	10921	0	0	0/0	0/0	0/0	0/0	0/0	0/0	0/0	0/0	0/0	0/0	0/0	0/0	0/0	0/0
Chromosome1-1584802 INS	PRECISE	10912	0	0	0/0	0/0	0/0	0/0	0/0	0/0	0/0	0/0	0/0	0/0	0/0	0/0	0/0	0/0
Chromosome1-1584834 DEL	PRECISE	85	0	0	0/0	0/0	0/0	0/0	0/0	0/0	0/0	0/0	0/0	0/0	0/0	0/0	0/0	0/1
Chromosome1-1586058 INS	PRECISE	338	0	0.408	0/0	0/0	0/0	0/1	0/0	0/0	0/0	0/0	0/1	0/0	0/1	0/1	0/0	0/1
Chromosome1-1586255 INS	PRECISE	647	0	0	0/0	0/0	0/0	0/0	0/0	0/0	0/0	0/0	0/1	0/0	0/0	0/0	0/0	0/0
Chromosome1-1586340 INS	PRECISE	1136	0	0	0/0	0/0	0/0	0/0	0/0	0/0	0/0	0/0	0/1	0/0	0/0	0/0	0/0	0/0
Chromosome1-1586351 INS	PRECISE	211	0	0	0/0	0/0	0/0	0/0	0/0	0/0	0/0	0/0	0/0	0/0	0/0	0/0	0/0	0/1
Chromosome1-1587682 DEL	PRECISE	1186	0	0	0/0	0/0	0/0	0/0	0/0	0/0	0/0	0/0	0/0	0/0	0/0	0/1	0/0	0/0
Chromosome1-1587981 DEL	PRECISE	1677	0	0	0/0	0/0	0/0	1/1	0/1	0/1	0/1	0/1	1/1	0/0	0/0	0/1	0/0	0/0
Chromosome1-1589046 INS	PRECISE	132	0	0	0/0	0/0	0/0	0/0	0/0	0/0	0/0	0/0	0/1	0/0	0/0	0/1	0/0	0/0
Chromosome1-1589393 INS	PRECISE	1976	0	0	0/0	0/0	0/0	0/0	0/0	0/0	0/0	0/0	0/1	0/0	0/0	0/0	0/0	0/0
Chromosome1-1589715 DEL	PRECISE	126	0	0	0/0	0/0	0/0	0/0	0/0	0/0	0/0	0/0	0/1	0/0	0/0	0/1	0/0	0/0
Chromosome1-1590093 DEL	PRECISE	54	0	0	0/0	0/0	0/0	1/1	0/0	0/0	0/1	0/1	0/1	0/0	0/0	0/1	0/0	0/0
Chromosome1-1591427 DEL	PRECISE	51	0	0	0/0	0/0	0/0	0/0	0/0	0/0	0/0	0/0	0/1	0/0	0/0	0/1	0/1	0/0
Chromosome1-1592424 DEL	PRECISE	75	0	0	0/0	0/0	0/0	0/0	0/0	0/0	0/0	0/0	0/1	0/0	0/1	1/1	0/1	0/0
Chromosome1-1593326 DEL	PRECISE	11567	0	0	0/0	0/0	0/0	0/0	0/0	0/0	0/0	0/0	0/1	0/0	0/1	1/1	1/1	0/0
Chromosome1-1593563 DEL	PRECISE	2148	0	0	0/0	0/1	0/1	0/1	0/1	0/1	0/1	0/1	0/0	0/1	0/0	0/0	0/0	1/1
Chromosome1-1593819 DEL	PRECISE	5135	0	0	0/0	0/0	0/0	0/0	0/0	0/0	0/0	0/0	0/0	0/0	0/0	0/0	0/0	0/1
Chromosome1-1594045 DEL	PRECISE	1531	0	0	0/0	0/1	0/1	0/0	0/1	0/1	0/0	0/0	0/0	0/1	0/0	0/0	0/0	0/1
Chromosome1-1594705 INS	PRECISE	445	0	0.5	0/0	0/0	0/0	0/0	0/0	0/0	0/0	0/0	0/0	0/0	0/0	0/1	0/1	0/0
Chromosome1-1595539 INS	PRECISE	2470	0	0	0/0	0/0	0/0	0/0	0/0	0/0	0/0	0/0	0/0	0/0	0/0	0/0	0/0	0/1
Chromosome1-1595690 DEL	PRECISE	183	0	0	0/0	0/0	0/0	0/0	0/0	0/0	0/0	0/0	0/0	0/0	0/1	0/1	0/1	0/0
Chromosome1-1596620 INS	PRECISE	955	0	0	0/0	0/0	0/0	0/0	0/0	0/0	0/0	0/0	0/0	0/0	0/0	0/0	0/0	0/1
Chromosome1-1597211 INS	PRECISE	72	0	0	0/0	0/0	0/0	0/0	0/0	0/0	0/0	0/0	0/1	0/0	0/1	0/1	0/1	0/0
Chromosome1-1597507 INS	PRECISE	7549	0	0	0/0	0/0	0/0	0/0	0/0	0/0	0/1	1/1	0/0	0/0	0/0	0/0	0/0	0/0
Chromosome1-1599246 DEL	PRECISE	91	0	0	0/0	0/0	0/0	0/0	0/0	0/0	0/0	0/0	0/1	0/0	0/0	0/1	0/1	0/0
Chromosome1-1599526 INS	PRECISE	90	0	0.957	0/0	0/0	0/0	0/0	0/0	0/0	0/0	0/0	0/1	0/0	0/1	0/1	0/1	0/0
Chromosome1-1599798 INS	PRECISE	5154	0	0	0/0	0/0	0/0	0/0	0/0	0/0	0/0	0/0	1/1	0/0	0/0	0/0	0/0	0/0
Chromosome1-1601069 DEL	PRECISE	1684	0.832	0.832	0/0	0/1	0/1	0/1	0/1	0/1	0/1	0/1	0/1	0/1	0/1	0/1	0/1	1/1
Chromosome1-1602189 DEL	PRECISE	949	0	0	0/0	0/0	0/0	0/0	0/0	0/0	0/0	0/0	0/1	0/0	0/0	0/1	0/0	0/0

Chromosome1-1603579	DEL	PRECISE	62	0	0	0/0	0/0	0/0	0/0	0/0	0/0	0/0	0/0	0/1	0/0	0/1	0/1	0/1	0/0
Chromosome1-1603623	DEL	PRECISE	1122	0	0	0/0	0/0	0/0	0/0	0/0	0/0	0/0	0/0	0/1	0/0	0/1	0/1	0/1	0/0
Chromosome1-1603648	BND	PRECISE	0	0	0	0/0	0/0	0/0	0/0	0/0	0/0	0/0	0/0	0/0	0/0	0/0	0/0	0/0	0/1
Chromosome1-1603732	INS	PRECISE	10152	0	0	0/0	0/0	0/0	0/0	0/0	0/0	0/0	0/0	0/0	0/0	0/0	0/0	0/0	0/1
Chromosome1-1603932	INS	PRECISE	401	0	0	0/0	0/0	0/0	0/0	0/0	0/0	0/0	0/0	0/0	0/0	0/0	0/0	0/0	0/1
Chromosome1-1604546	DEL	PRECISE	260	0.289	0.289	0/0	0/1	0/1	0/0	0/1	0/1	0/1	0/1	0/1	0/1	0/1	0/1	0/1	1/1
Chromosome1-1605094	DEL	PRECISE	52	0	0	0/0	0/0	0/0	0/1	0/0	0/0	0/1	0/1	0/1	0/0	0/1	0/1	0/1	0/0
Chromosome1-1605118	INS	PRECISE	1170	0	0	0/0	0/0	0/0	0/1	0/0	0/0	0/0	0/0	0/0	0/0	0/0	0/0	0/0	0/0
Chromosome1-1605292	DEL	PRECISE	57	0	0	0/0	0/0	0/0	0/0	0/0	0/0	0/0	0/0	0/1	0/0	0/0	0/0	0/0	0/0
Chromosome1-1606362	BND	PRECISE	0	0	0	0/0	0/0	0/0	0/0	0/0	0/0	0/1	0/0	0/0	0/0	0/0	0/0	0/0	0/0
Chromosome1-1607049	DEL	PRECISE	54	0	0	0/0	0/0	0/0	0/0	0/0	0/0	0/0	0/1	0/0	0/0	0/0	0/0	0/0	0/0
Chromosome1-1607725	DEL	PRECISE	3182	0	0	0/0	0/1	0/1	0/0	0/1	0/1	0/1	0/1	0/0	0/1	0/0	0/0	0/0	0/1
Chromosome1-1607794	DEL	PRECISE	9792	0.577	0.577	0/0	0/0	0/0	0/0	0/0	0/0	0/0	0/0	1/1	0/0	0/1	0/1	1/1	0/0
Chromosome1-1607795	DUP	PRECISE	1512372	0	0	0/0	0/0	0/0	0/0	0/0	0/0	0/0	0/0	0/1	0/0	0/1	0/1	0/1	0/0
Chromosome1-1608774	DEL	PRECISE	1502577	0	0	0/0	0/0	0/0	0/0	0/0	0/0	0/0	0/0	0/0	0/0	0/1	0/1	0/1	0/0
Chromosome1-1609129	INS	PRECISE	704	0.905	0.754	0/0	0/1	0/1	0/0	0/1	0/1	0/1	0/1	0/1	0/1	0/0	0/1	0/1	0/1
Chromosome1-1610669	DEL	PRECISE	1295	0	0	0/0	0/0	0/0	0/1	0/0	0/0	0/0	0/0	0/0	0/0	0/0	0/0	0/0	0/0
Chromosome1-1611402	INS	PRECISE	867	0	0.707	0/0	0/0	0/0	0/0	0/0	0/0	0/1	0/1	0/0	0/0	0/0	0/0	0/0	0/0
Chromosome1-1611647	DEL	PRECISE	86	0	0	0/0	0/0	0/0	0/0	0/0	0/0	0/0	0/0	0/1	0/0	0/1	0/0	0/0	0/0
Chromosome1-1612179	DEL	PRECISE	102	0	0	0/0	0/0	0/0	0/1	0/0	0/0	0/0	0/0	0/0	0/0	0/0	0/0	0/0	0/0
Chromosome1-1612286	DEL	PRECISE	1324	0	0	0/0	0/0	0/0	0/0	0/0	0/0	0/0	0/0	1/1	0/0	1/1	1/1	0/1	0/0
Chromosome1-1612549	INS	PRECISE	7694	0	0	0/0	0/0	0/0	1/1	0/0	0/0	0/0	0/0	0/0	0/0	0/0	0/0	0/0	0/0
Chromosome1-1612777	INS	PRECISE	960	0	0.577	0/0	0/0	0/0	0/0	0/0	0/0	0/0	0/0	1/1	0/0	1/1	1/1	0/0	0/0
Chromosome1-1613639	DEL	PRECISE	1393	0	0	0/0	0/1	0/1	0/0	0/0	0/1	0/1	0/1	0/0	0/1	0/0	0/0	0/0	1/1
Chromosome1-1615588	INS	PRECISE	195	0	0	0/0	0/0	0/0	0/0	0/0	0/0	0/0	0/0	0/0	0/0	0/0	0/1	0/0	0/0
Chromosome1-1615627	DEL	PRECISE	575	0	0	0/0	0/0	0/1	0/0	0/0	0/1	0/1	0/1	0/0	0/0	0/0	0/0	0/0	0/0
Chromosome1-1616036	INS	PRECISE	69	0	0.555	0/0	0/1	0/1	1/1	0/1	0/1	0/1	0/1	0/1	0/1	0/1	0/1	0/1	1/1
Chromosome1-1617328	DEL	PRECISE	138	0.316	0.316	0/0	0/0	0/0	0/0	0/0	0/0	0/0	0/0	0/0	0/0	0/0	0/0	0/0	0/1
Chromosome1-1618310	BND	PRECISE	0	0	0	0/0	0/0	0/0	0/0	0/0	0/0	0/0	0/0	0/0	0/0	0/0	0/0	0/0	0/1
Chromosome1-1618522	INS	PRECISE	161	0	0.577	0/0	0/0	0/0	0/0	0/0	0/0	0/0	0/0	0/1	0/0	0/1	0/0	0/1	0/0
Chromosome1-1619574	INS	PRECISE	277	0	0.577	0/0	0/0	0/0	0/0	0/0	0/0	0/0	0/0	0/1	0/0	0/1	0/0	0/1	0/0
Chromosome1-1619673	INS	PRECISE	909	0	0	0/0	0/0	0/0	0/0	0/0	0/0	0/0	0/0	0/1	0/0	0/0	0/0	0/0	0/0
Chromosome1-1619885	DEL	PRECISE	1083	0	0	0/0	0/1	0/1	0/0	0/1	0/0	0/0	0/0	0/0	0/1	0/0	0/0	0/0	0/1
Chromosome1-1621640	DEL	PRECISE	19043	0	0	0/0	0/0	0/0	0/0	0/0	0/0	0/0	0/0	0/0	0/0	0/1	0/1	0/1	0/0
Chromosome1-1623372	DEL	PRECISE	4446	0	0	0/0	0/0	0/0	0/0	0/0	0/0	0/0	0/0	0/0	0/0	0/0	0/0	0/0	0/1
Chromosome1-1623747	DUP	PRECISE	13567	0	0	0/0	0/1	0/1	0/0	0/0	0/1	0/0	0/0	0/0	0/1	0/0	0/0	0/0	0/1
Chromosome1-1624304	INS	PRECISE	181	0.492	0.289	0/0	0/1	0/1	0/0	0/1	0/1	0/1	0/1	0/0	1/1	0/1	0/1	0/1	0/1
Chromosome1-1624742	INS	PRECISE	6034	0	0	0/0	0/0	0/0	0/0	0/0	0/0	0/0	0/0	0/0	0/0	0/0	1/1	0/0	0/0
Chromosome1-1624932	DEL	PRECISE	79	0	0	0/0	0/0	0/0	0/0	0/0	0/0	0/0	0/0	0/0	0/0	0/0	0/1	0/0	0/0
Chromosome1-1625072	DEL	PRECISE	9425	0	0	0/0	0/0	0/0	0/0	0/0	0/0	0/0	0/0	0/0	0/0	0/0	0/0	0/0	0/1
Chromosome1-1627701	DEL	PRECISE	233	0	0	0/0	0/0	0/0	0/0	0/0	0/0	0/0	0/0	0/1	0/0	0/1	0/0	0/1	0/0
Chromosome1-1627777	INS	PRECISE	137	0	0	0/0	0/0	0/0	0/0	0/0	0/0	0/1	0/1	0/0	0/0	0/0	0/0	0/0	0/0
Chromosome1-1628272	INS	PRECISE	1546	0	0	0/0	0/0	0/0	1/1	0/0	0/0	0/0	0/0	0/0	0/0	0/0	0/0	0/0	0/0
Chromosome1-1628828	INS	PRECISE	891	0	0	0/0	0/0	0/0	0/0	0/0	0/0	0/0	0/0	0/1	0/0	0/0	0/0	0/0	0/0
Chromosome1-1628963	INS	PRECISE	8807	0	0	0/0	0/0	0/0	0/0	0/0	0/0	0/0	0/0	0/0	0/0	0/0	0/0	0/0	0/1
Chromosome1-1629028	DEL	PRECISE	63	0	0	0/0	0/0	0/0	0/1	0/0	0/0	0/0	0/0	0/0	0/0	0/0	0/0	0/0	0/0
Chromosome1-1629156	DEL	PRECISE	99	0	0	0/0	0/0	0/0	0/0	0/0	0/0	0/0	0/0	0/0	0/0	0/0	0/0	0/0	0/1
Chromosome1-1629460	DEL	PRECISE	93	0	0	0/0	0/0	0/0	0/0	0/0	0/0	0/0	0/0	0/0	0/0	0/0	0/0	0/0	0/1
Chromosome1-1630460	DEL	PRECISE	1026	0	0	0/0	0/0	0/0	0/0	0/0	0/0	0/0	0/0	0/1	0/0	0/1	0/1	0/1	0/0
Chromosome1-1632812	DEL	PRECISE	1332	0	0	0/0	0/0	0/0	0/0	0/0	0/0	0/0	0/0	0/1	0/0	0/1	0/1	0/1	0/0
Chromosome1-1634531	INS	PRECISE	50	0	0	0/0	0/0	0/0	1/1	0/0	0/0	0/0	0/0	0/0	0/0	0/0	0/0	0/0	0/0
Chromosome1-1634819	INS	PRECISE	61	0	0	0/0	0/0	0/0	0/0	0/0	0/0	0/0	0/0	0/1	0/0	0/0	0/0	0/1	0/0
Chromosome1-1635268	DEL	PRECISE	9708	0	0	0/0	0/0	0/0	0/0	0/0	0/0	0/0	0/0	1/1	0/0	0/0	0/0	1/1	0/0
Chromosome1-1635771	INS	PRECISE	1362	0	0	0/0	0/0	0/0	0/0	0/0	0/0	0/0	0/0	0/0	0/0	0/0	0/0	0/0	0/1
Chromosome1-1636239	BND	PRECISE	0	0	0	0/0	0/0	0/0	0/0	0/0	0/0	0/0	0/0	0/1	0/0	0/0	0/0	0/0	0/0
Chromosome1-1636278	INS	PRECISE	208	0	0.707	0/0	0/0	0/0	0/0	0/0	0/0	0/0	0/0	0/1	0/0	0/0	0/0	0/1	0/0
Chromosome1-1637485	INS	PRECISE	142	0	0	0/0	0/0	0/0	0/0	0/0	0/0	0/0	0/0	0/0	0/0	0/0	0/0	0/0	0/1
Chromosome1-1637650	DEL	PRECISE	51	0	0	0/0	0/0	0/0	0/0	0/0	0/0	0/0	0/0	0/0	0/0	0/0	0/0	0/0	0/1
Chromosome1-1638054	DEL	PRECISE	104	0	0	0/0	0/1	0/1	0/0	0/1	0/1	0/1	0/1	0/0	0/1	0/0	0/0	0/0	1/1
Chromosome1-1638342	DEL	PRECISE	13492	0	0	0/0	0/0	0/0	0/0	0/0	0/0	0/0	0/0	0/0	0/0	0/0	0/0	0/0	0/1
Chromosome1-1639959	DEL	PRECISE	12551	0	0	0/0	0/1	0/1	1/1	1/1	1/1	0/1	1/1	1/1	0/1	0/0	0/0	0/0	1/1
Chromosome1-1641697	INS	PRECISE	2230	0	0	0/0	0/0	0/0	0/0	0/0	0/0	0/0	0/0	0/0	0/0	0/0	0/0	0/0	0/1
Chromosome1-1641892	DEL	PRECISE	123770	0	0	0/0	0/0	0/0	0/0	0/0	0/0	0/0	0/0	0/0	1/1	0/0	0/0	0/0	0/0
Chromosome1-1642173	DEL	PRECISE	13266	0	0	0/0	0/0	0/0	1/1	0/0	0/0	0/0	0/0	0/0	0/0	0/0	0/0	0/0	0/0
Chromosome1-1642279	DEL	PRECISE	57	0	0	0/0	0/0	0/0	0/0	0/0	0/0	0/1	0/1	0/0	0/0	0/0	0/0	0/0	0/0
Chromosome1-1642991	DEL	PRECISE	23613	0	0	0/0	0/0	0/0	0/0	0/0	0/0	0/0	0/0	0/0	0/0	0/0	0/0	0/0	0/1
Chromosome1-1643079	DEL	PRECISE	1302	0	0	0/0	0/1	0/1	0/0	0/1	0/1	0/1	0/1	0/0	0/0	0/0	0/0	0/0	1/1
Chromosome1-1643743	DEL	PRECISE	12494	0	0	0/0	0/1	0/1	1/1	1/1	1/1	0/1	0/1	1/1	0/0	0/0	0/0	0/0	1/1
Chromosome1-1646729	INS	PRECISE	363	0	0	0/0	0/0	0/0	0/0	0/0	0/0	0/0	0/0	0/0	0/0	0/0	0/0	0/0	0/1
Chromosome1-1648131	INS	PRECISE	69	0	0	0/0	0/0	0/0	0/0	0/0	0/0	0/0	0/0	0/1	0/0	0/0	0/1	0/1	0/0
Chromosome1-1649405	INS	PRECISE	1331	0	0	0/0	0/0	0/0	0/0	0/0	0/0	0/0	0/0	0/0	0/0	0/0	0/0	0/0	0/1
Chromosome1-1649992	INS	PRECISE	10586	0	0	0/0	0/0	0/0	0/0	0/0	0/0	0/0	0/0	0/0	0/0	0/0	0/0	0/0	1/1
Chromosome1-1650625	DEL	PRECISE	162	0	0	0/0	0/0	0/0	0/0	0/0	0/0	0/1	0/1	0/0	0/0	0/0	0/0	0/0	0/0

Chromosome1-1651492	BND	PRECISE	0	0	0	0/0	0/0	0/0	0/0	0/1	0/0	0/0	0/0	1/1	0/0	0/0	0/0	0/0	0/0
Chromosome1-1653448	DEL	PRECISE	6239	0	0	0/0	0/1	0/1	0/0	0/1	0/1	0/0	0/0	0/0	0/0	0/0	0/0	0/0	0/1
Chromosome1-1653923	INS	PRECISE	689	0	0	0/0	0/0	0/0	1/1	0/0	0/0	0/0	0/0	0/0	0/0	0/0	0/0	0/0	0/0
Chromosome1-1655557	INS	PRECISE	1184	0	0	0/0	0/0	0/0	1/1	0/0	0/0	0/0	0/0	0/0	0/0	0/0	0/0	0/0	0/0
Chromosome1-1657011	DEL	PRECISE	713	0	0	0/0	0/1	0/0	0/0	0/1	0/0	0/0	0/0	0/0	0/1	0/0	0/0	0/0	1/1
Chromosome1-1657147	INS	PRECISE	110	0.422	0.738	0/0	0/1	0/1	0/0	0/1	0/1	0/0	0/0	0/0	0/1	0/0	0/0	0/0	1/1
Chromosome1-1657565	INV	PRECISE	2369	0	0.707	0/0	0/0	0/0	0/0	0/0	0/0	0/1	0/0	0/0	0/0	0/0	0/0	0/0	0/0
Chromosome1-1657621	INV	PRECISE	1803	0	0	0/0	0/0	0/0	0/0	0/0	0/0	0/1	0/0	0/0	0/0	0/0	0/0	0/0	0/0
Chromosome1-1658308	INS	PRECISE	59	0	0.316	0/0	0/1	0/1	1/1	1/1	1/1	0/1	0/1	1/1	0/1	0/0	0/0	0/0	1/1
Chromosome1-1658481	INS	PRECISE	249	0	0.354	0/0	0/1	0/1	0/0	0/1	0/1	0/1	0/1	0/0	0/1	0/0	0/0	0/0	1/1
Chromosome1-1659732	INS	PRECISE	5612	0	0	0/0	0/0	0/0	0/0	0/0	0/0	0/0	0/0	0/0	0/0	0/0	0/0	0/0	1/1
Chromosome1-1660699	DEL	PRECISE	1553	0.447	0.447	0/0	0/0	0/0	0/0	0/0	0/0	0/0	0/0	0/0	0/0	0/1	0/0	0/1	0/1
Chromosome1-1663057	DEL	PRECISE	5254	0	0	0/0	0/0	0/0	0/0	0/0	0/0	0/0	0/0	0/1	0/0	0/1	0/1	0/1	0/0
Chromosome1-1664177	INS	PRECISE	12665	0	0	0/0	0/0	0/0	1/1	0/0	0/0	0/0	0/0	0/0	0/0	0/0	0/0	0/0	0/0
Chromosome1-1664177	BND	PRECISE	0	0	0	0/0	0/0	0/0	0/1	0/0	0/0	0/0	0/0	0/0	0/0	0/0	0/0	0/0	0/0
Chromosome1-1664178	BND	PRECISE	0	0	0	0/0	0/0	0/0	0/1	0/0	0/0	0/0	0/0	0/0	0/0	0/0	0/0	0/0	0/0
Chromosome1-1664440	DEL	PRECISE	69	0	0	0/0	0/0	0/0	0/0	0/0	0/0	0/0	0/0	0/0	0/0	0/0	0/0	0/0	0/1
Chromosome1-1665647	DEL	PRECISE	122	0	0	0/0	0/0	0/0	1/1	0/0	0/0	0/0	0/0	0/0	0/0	0/0	0/0	0/0	0/0
Chromosome1-1666991	DEL	PRECISE	9230	0	0	0/0	0/0	0/0	0/0	0/0	0/0	0/0	0/0	0/0	0/0	0/0	0/0	0/0	0/1
Chromosome1-1668388	INS	PRECISE	2238	0	0	0/0	0/0	0/0	0/0	0/0	0/0	0/0	0/0	0/0	0/0	0/0	0/0	0/0	0/1
Chromosome1-1668644	BND	PRECISE	0	0	0	0/0	0/0	0/0	0/0	0/0	0/0	0/0	0/0	0/0	0/0	0/0	0/0	0/0	0/1
Chromosome1-1669552	BND	PRECISE	0	0	0	0/0	0/0	0/0	0/0	0/0	0/0	0/0	0/0	0/0	0/0	0/0	0/0	0/0	0/1
Chromosome1-1669722	BND	PRECISE	0	0	0	0/0	0/0	0/0	0/0	0/0	0/1	0/0	0/0	0/0	0/0	0/0	0/0	0/0	0/0
Chromosome1-1669933	DEL	PRECISE	211	0	0	0/0	0/0	0/0	0/0	0/0	0/0	0/0	0/0	0/1	0/0	1/1	0/1	0/1	0/1
Chromosome1-1670248	BND	PRECISE	0	0	0	0/0	0/0	0/0	0/0	0/0	0/0	0/0	0/0	0/1	0/0	1/1	0/0	1/1	0/0
Chromosome1-1670284	DEL	PRECISE	68	0	0	0/0	0/0	0/0	1/1	0/0	0/0	0/0	0/0	0/0	0/0	0/0	0/0	0/0	0/0
Chromosome1-1670602	DEL	PRECISE	92	0	0	0/0	0/0	0/0	0/0	0/0	0/0	0/1	0/1	0/0	0/0	0/0	0/0	0/0	0/0
Chromosome1-1670616	INS	PRECISE	92	0	0	0/0	0/0	0/0	0/0	0/0	0/0	0/0	0/0	0/0	0/0	0/0	0/0	0/0	0/1
Chromosome1-1671555	DEL	PRECISE	1664	0	0	0/0	0/0	0/0	0/0	0/0	0/0	0/0	0/0	0/0	0/0	0/0	0/0	0/0	0/1
Chromosome1-1672380	INS	PRECISE	72	0	0	0/0	0/0	0/0	0/0	0/0	0/0	0/0	0/0	0/0	0/0	0/0	0/0	0/0	0/1
Chromosome1-1672388	DEL	PRECISE	71	0	0	0/0	0/0	0/0	1/1	0/0	0/0	0/0	0/0	0/0	0/0	0/0	0/0	0/0	0/0
Chromosome1-1673230	DEL	PRECISE	1099	0	0	0/0	0/0	0/0	0/0	0/0	0/0	0/0	0/0	0/0	0/0	0/0	0/0	0/0	0/1
Chromosome1-1673502	DEL	PRECISE	679	0	0	0/0	0/0	0/0	1/1	0/0	0/0	0/0	0/0	0/0	0/0	0/0	0/0	0/0	0/0
Chromosome1-1673842	DEL	PRECISE	8936	0	0	0/0	0/1	0/1	0/0	0/1	0/1	0/1	0/1	0/0	0/1	0/0	0/0	0/0	0/1
Chromosome1-1674536	DEL	PRECISE	71	0	0	0/0	0/0	0/0	0/0	0/0	0/0	0/0	0/0	0/0	0/0	0/0	0/1	0/0	0/0
Chromosome1-1674646	INS	PRECISE	93	0	0	0/0	0/0	0/0	0/0	0/0	0/0	0/0	0/0	0/0	0/0	0/0	0/0	0/0	0/1
Chromosome1-1674749	BND	PRECISE	0	0	0	0/0	0/0	0/0	0/0	0/0	0/0	0/0	0/0	0/0	0/0	0/0	0/1	0/0	0/0
Chromosome1-1674879	INS	PRECISE	54	0	0	0/0	0/0	0/0	0/0	0/0	0/0	0/0	0/0	0/0	0/0	0/0	0/0	0/0	0/1
Chromosome1-1675247	INS	PRECISE	1554	0	0	0/0	0/0	0/0	0/0	0/0	0/0	0/0	0/0	0/0	0/0	0/0	0/1	0/0	0/0
Chromosome1-1675533	DEL	PRECISE	479	0	0	0/0	0/0	0/0	0/0	0/0	0/0	0/0	0/0	0/0	0/0	0/0	0/0	0/0	0/1
Chromosome1-1675631	DEL	PRECISE	432	0.5	0	0/0	0/0	0/0	0/0	0/0	0/0	0/0	0/0	0/1	0/0	0/1	0/1	0/1	0/0
Chromosome1-1676275	DEL	PRECISE	121	0	0	0/0	0/0	0/0	0/0	0/0	0/0	0/0	0/0	0/1	0/0	0/1	0/1	0/1	0/0
Chromosome1-1676713	BND	PRECISE	0	0	0	0/0	0/0	0/0	0/0	0/0	0/0	0/0	0/0	0/0	0/0	0/1	0/1	0/0	0/0
Chromosome1-1676920	BND	PRECISE	0	0	0	0/0	0/0	0/0	0/0	0/0	0/0	0/0	0/0	0/0	0/0	0/0	0/0	0/1	0/0
Chromosome1-1677102	DEL	PRECISE	1654	0	0	0/0	0/0	0/0	0/0	0/0	0/0	0/0	0/0	0/1	0/0	0/1	0/1	0/1	0/0
Chromosome1-1678721	INS	PRECISE	211	0	0	0/0	0/0	0/0	0/0	0/0	0/0	0/0	0/0	0/0	0/0	0/1	0/0	0/1	0/0
Chromosome1-1679173	DEL	PRECISE	94	0	0	0/0	0/0	0/0	0/0	0/0	0/0	0/0	0/0	0/1	0/0	0/1	0/1	0/1	0/0
Chromosome1-1679987	DEL	PRECISE	564	0.5	0	0/0	0/0	0/0	0/0	0/0	0/0	0/0	0/0	0/1	0/0	0/1	0/1	0/1	0/0
Chromosome1-1680284	INS	PRECISE	132	0	0	0/0	0/0	0/0	0/0	0/0	0/0	0/0	0/0	0/0	0/0	0/0	0/0	0/0	0/1
Chromosome1-1680831	INS	PRECISE	1566	0	0	0/0	0/0	0/0	1/1	0/0	0/0	0/0	0/0	0/0	0/0	0/0	0/0	0/0	0/0
Chromosome1-1681465	INS	PRECISE	860	0	0	0/0	0/0	0/0	1/1	0/0	0/0	0/0	0/0	0/0	0/0	0/0	0/0	0/0	0/0
Chromosome1-1681488	INS	PRECISE	3781	0	0	0/0	0/0	0/0	1/1	0/0	0/0	0/0	0/0	0/0	0/0	0/0	0/0	0/0	0/0
Chromosome1-1681639	INS	PRECISE	211	0	0	0/0	0/0	0/0	0/0	0/0	0/0	0/0	0/0	0/0	0/0	0/0	0/0	0/0	0/1
Chromosome1-1682006	INS	PRECISE	555	0	0	0/0	0/0	0/0	0/0	0/0	0/0	0/0	0/0	0/0	0/0	0/0	0/1	0/0	0/0
Chromosome1-1682214	INS	PRECISE	429	0	0	0/0	0/0	0/0	0/0	0/0	0/0	0/0	0/0	0/0	0/0	0/0	0/1	0/0	0/0
Chromosome1-1683021	DEL	PRECISE	798	0	0	0/0	0/0	0/0	1/1	0/0	0/0	0/0	0/0	0/0	0/0	0/0	0/0	0/0	0/0
Chromosome1-1683265	BND	PRECISE	0	0	0	0/0	0/0	0/0	0/0	0/0	0/0	0/0	0/0	0/0	0/0	0/0	0/1	0/0	0/0
Chromosome1-1684139	DEL	PRECISE	801	0	0	0/1	0/0	0/1	0/1	0/1	0/1	0/1	0/1	0/1	0/1	0/0	0/1	0/0	0/0
Chromosome1-1685489	DEL	PRECISE	129	0	0	0/0	0/0	0/0	1/1	0/0	0/0	0/0	0/0	0/0	0/0	0/0	0/0	0/0	0/0
Chromosome1-1685894	DEL	PRECISE	86	0	0	0/0	0/0	0/0	0/0	0/0	0/0	0/0	0/0	0/0	0/0	0/0	0/1	0/1	0/0
Chromosome1-1686065	INS	PRECISE	92	0	0	0/0	0/0	0/0	0/0	0/0	0/0	0/0	0/0	0/1	0/0	0/0	0/1	0/1	0/0
Chromosome1-1686083	INS	PRECISE	2880	0	0	0/0	0/0	0/0	0/0	0/0	0/0	0/0	0/0	0/0	0/0	0/0	1/1	0/0	0/0
Chromosome1-1686530	DEL	PRECISE	477	0	0	0/0	0/0	0/0	0/0	0/0	0/0	0/0	0/0	0/0	0/0	0/0	0/1	0/1	0/0
Chromosome1-1687180	DEL	PRECISE	90	0	0	0/0	0/0	0/0	0/0	0/0	0/0	0/1	0/1	0/0	0/0	0/0	0/0	0/0	0/0
Chromosome1-1687365	DEL	PRECISE	1379	0	0	0/0	0/1	0/1	1/1	1/1	1/1	0/1	0/1	1/1	0/1	0/0	0/0	0/0	1/1
Chromosome1-1688029	INS	PRECISE	54	0	0	0/0	0/1	0/1	1/1	0/1	0/1	0/0	0/0	0/0	0/1	0/0	0/0	0/0	1/1
Chromosome1-1689210	DEL	PRECISE	629	0	0	0/0	0/0	0/0	0/0	0/0	0/0	0/0	0/0	0/1	0/0	0/1	0/1	0/1	0/0
Chromosome1-1689573	BND	PRECISE	0	0	0	0/0	0/0	0/0	0/0	0/0	0/0	0/0	0/0	1/1	0/0	0/1	0/1	0/1	0/0
Chromosome1-1689677	INS	PRECISE	104	0	0	0/0	0/0	0/0	1/1	0/0	0/0	0/0	0/0	0/0	0/0	0/0	0/0	0/0	0/0
Chromosome1-1690053	DEL	PRECISE	448	0	0	0/0	0/0	0/0	0/0	0/0	0/0	0/0	0/0	0/1	0/0	0/1	0/1	0/0	0/0
Chromosome1-1690245	INS	PRECISE	172	0	0	0/0	0/0	0/0	0/0	0/0	0/0	0/0	0/0	0/0	0/0	0/1	0/0	0/0	0/0
Chromosome1-1690567	INS	PRECISE	4534	0	0	0/0	0/0	0/0	1/1	0/0	0/0	0/0	0/0	0/0	0/0	0/0	0/0	0/0	0/0
Chromosome1-1692362	INS	PRECISE	1461	0	0	0/0	0/0	0/0	0/0	0/0	0/0	0/1	0/1	0/0	0/0	0/0	0/0	0/0	0/0

Chromosome1-1695478	INS	PRECISE	1147	0	0	0/0	0/0	0/0	0/0	0/0	0/0	0/0	0/0	0/0	0/0	0/0	0/1	0/0	0/0
Chromosome1-1696360	INS	PRECISE	1339	0	0	0/0	0/0	0/0	0/0	0/0	0/0	0/0	0/0	0/0	0/0	0/0	0/1	0/0	0/0
Chromosome1-1697802	DEL	PRECISE	90	0	0	0/0	0/0	0/0	0/1	0/0	0/0	0/0	0/0	0/0	0/0	0/0	0/0	0/0	0/0
Chromosome1-1699395	INS	PRECISE	996	0	0.707	0/0	0/0	0/0	0/0	0/0	0/0	0/1	0/1	0/0	0/0	0/0	0/0	0/0	0/0
Chromosome1-1700740	DEL	PRECISE	53	0	0	0/0	0/0	0/0	0/0	0/0	0/0	0/0	0/0	0/0	0/0	0/0	0/1	0/0	0/0
Chromosome1-1700773	INS	PRECISE	5044	0	0	0/0	0/0	0/0	0/0	0/0	0/0	0/0	0/0	0/0	0/0	0/0	0/0	0/0	1/1
Chromosome1-1700888	INS	PRECISE	724	0	0	0/0	0/0	0/0	0/0	0/0	0/0	0/0	0/0	0/1	0/0	0/0	0/0	0/0	0/0
Chromosome1-1701231	INS	PRECISE	58	0	0	0/0	0/0	0/0	1/1	0/0	0/0	0/0	0/0	0/0	0/0	0/0	0/0	0/0	0/0
Chromosome1-1701605	INS	PRECISE	2018	0	0.707	0/0	0/0	0/0	0/0	0/0	0/0	0/1	0/1	0/0	0/0	0/0	0/0	0/0	0/0
Chromosome1-1701974	DEL	PRECISE	97	0	0	0/0	0/0	0/0	1/1	0/0	0/0	0/0	0/0	0/0	0/0	0/0	0/0	0/0	0/1
Chromosome1-1702176	DEL	PRECISE	67	0	0	0/0	0/0	0/0	0/0	0/0	0/0	0/1	0/1	0/0	0/0	0/0	0/0	0/0	0/0
Chromosome1-1703521	INS	PRECISE	3234	0	0	0/0	0/0	0/0	0/0	0/0	0/0	0/0	0/0	1/1	0/0	0/0	0/0	0/0	0/0
Chromosome1-1703682	INS	PRECISE	366	0	0	0/0	0/0	0/0	0/0	0/0	0/0	0/0	0/0	0/1	0/0	0/0	0/0	0/0	0/0
Chromosome1-1704136	INS	PRECISE	54	0	0	0/0	0/0	0/0	0/0	0/0	0/0	0/0	0/0	0/1	0/0	0/0	0/1	0/1	0/0
Chromosome1-1705041	DEL	PRECISE	448	0	0	0/0	0/0	0/0	0/0	0/0	0/0	0/0	0/0	0/0	0/0	0/1	0/0	0/1	0/0
Chromosome1-1705388	INS	PRECISE	218	0	0	0/0	0/0	0/0	0/0	0/0	0/0	0/0	0/0	0/0	0/0	0/0	0/0	0/0	0/1
Chromosome1-1706666	INS	PRECISE	5162	0	0	0/0	0/0	0/0	0/0	0/0	0/0	0/0	0/0	0/0	0/0	0/0	0/1	0/0	0/0
Chromosome1-1707531	INS	PRECISE	2212	0	0	0/0	0/0	0/0	0/0	0/0	0/0	0/0	0/0	0/0	0/0	0/0	0/1	0/0	0/0
Chromosome1-1707607	INS	PRECISE	1373	0	0	0/0	0/0	0/0	0/0	0/0	0/0	0/0	0/0	0/1	0/0	0/0	0/0	0/0	0/0
Chromosome1-1708011	DEL	PRECISE	84	0	0	0/0	0/0	0/0	1/1	0/0	0/0	0/0	0/0	0/0	0/0	0/0	0/0	0/0	0/0
Chromosome1-1708067	DEL	PRECISE	4093	0.408	0.408	0/0	0/1	0/1	0/0	0/1	1/1	0/0	0/0	0/0	0/1	0/0	0/0	0/0	0/1
Chromosome1-1709652	DEL	PRECISE	4020	0	0	0/0	0/0	0/0	0/0	0/0	0/0	0/0	0/0	0/1	0/0	0/1	0/1	0/1	0/0
Chromosome1-1710347	INS	PRECISE	1394	0	0	0/0	0/0	0/0	0/0	0/0	0/0	0/0	0/0	0/0	0/0	0/0	0/0	0/0	0/1
Chromosome1-1710432	DEL	PRECISE	5040	0	0	0/0	0/0	0/0	0/0	0/0	0/0	0/0	0/0	0/1	0/0	0/1	0/1	0/1	0/0
Chromosome1-1711229	INS	PRECISE	354	0	0	0/0	0/0	0/0	0/0	0/0	0/0	0/0	0/0	0/0	0/0	0/0	0/0	0/0	0/1
Chromosome1-1711442	DEL	PRECISE	5657	0	0	0/0	0/0	0/0	0/0	0/0	0/0	0/0	0/0	0/0	0/0	0/1	0/1	0/1	0/0
Chromosome1-1711805	INS	PRECISE	1553	0	0	0/0	0/0	0/0	1/1	0/0	0/0	0/0	0/0	0/0	0/0	0/0	0/0	0/0	0/0
Chromosome1-1713089	INS	PRECISE	332	0	0	0/0	0/0	0/0	0/0	0/0	0/0	0/0	0/0	0/0	0/0	0/0	0/0	0/0	0/1
Chromosome1-1713218	INS	PRECISE	335	0	0	0/0	0/0	0/0	1/1	0/0	0/0	0/0	0/0	0/0	0/0	0/0	0/0	0/0	0/0
Chromosome1-1713256	DEL	PRECISE	376	0	0	0/0	0/0	0/0	0/0	0/0	0/0	0/0	0/0	0/0	0/0	0/1	0/1	0/1	0/1
Chromosome1-1713415	INS	PRECISE	3788	0	0	0/0	0/0	0/0	0/0	0/0	0/0	0/0	0/0	0/1	0/0	0/0	0/0	0/0	0/0
Chromosome1-1713541	BND	PRECISE	0	0	0	0/0	0/0	0/0	0/0	0/0	0/0	0/0	0/0	0/0	0/0	0/0	0/0	1/1	0/0
Chromosome1-1713541	BND	PRECISE	0	0	0	0/0	0/0	0/0	0/0	0/0	0/0	0/0	0/0	0/0	0/0	0/1	0/0	0/0	0/0
Chromosome1-1714343	DEL	PRECISE	180	0	0	0/0	0/1	0/1	1/1	0/1	0/1	0/1	0/1	0/1	0/1	0/1	0/1	0/1	1/1
Chromosome1-1714369	INS	PRECISE	654	0	0	0/0	0/0	0/0	1/1	0/0	0/0	0/0	0/0	0/0	0/0	0/0	0/0	0/0	0/0
Chromosome1-1714463	INS	PRECISE	132	0	0	0/0	0/0	0/0	0/1	0/0	0/0	0/0	0/0	0/0	0/0	0/0	0/0	0/0	0/0
Chromosome1-1715007	INS	PRECISE	996	0	0	0/0	0/0	0/0	0/0	0/0	0/0	0/0	0/0	0/0	0/0	0/0	0/0	0/0	0/1
Chromosome1-1715348	INS	PRECISE	74	0	0	0/0	0/0	0/0	0/0	0/0	0/0	0/0	0/0	0/1	0/0	0/1	0/1	0/1	0/0
Chromosome1-1715534	INS	PRECISE	102	0	0	0/0	0/0	0/0	0/0	0/0	0/0	0/0	0/0	0/1	0/0	0/1	0/1	0/1	0/0
Chromosome1-1716523	INS	PRECISE	4495	0	0	0/0	0/0	0/0	0/0	0/0	0/0	0/0	0/0	0/0	0/0	0/0	0/0	0/0	0/1
Chromosome1-1716523	INV	PRECISE	8741315	0	0	0/0	0/0	0/0	0/0	0/0	0/0	0/0	0/0	0/0	0/0	0/0	0/0	0/0	0/1
Chromosome1-1716710	INS	PRECISE	529	0	0.5	0/0	0/0	0/0	0/0	0/0	0/0	0/0	0/0	0/1	0/0	0/1	0/0	0/1	0/0
Chromosome1-1717582	INS	PRECISE	135	0	0	0/0	0/1	0/1	0/0	1/1	1/1	0/1	0/1	1/1	0/1	0/1	0/0	0/1	1/1
Chromosome1-1717693	INS	PRECISE	287	0.707	0.707	0/0	0/0	0/0	0/0	0/0	0/0	0/1	0/1	0/0	0/0	0/0	0/0	0/0	0/0
Chromosome1-1720352	INS	PRECISE	710	0	0	0/0	0/0	0/0	0/0	0/0	0/0	0/0	0/0	0/1	0/0	0/0	0/0	0/0	0/0
Chromosome1-1720450	DEL	PRECISE	82	0	0	0/0	0/1	0/1	0/0	1/1	1/1	0/1	0/1	1/1	0/1	0/1	0/1	0/1	1/1
Chromosome1-1721615	DEL	PRECISE	53	0	0	0/0	0/0	0/0	0/0	0/0	0/0	0/0	0/0	0/0	0/0	0/0	0/0	0/0	0/1
Chromosome1-1721888	INS	PRECISE	703	0	0	0/0	0/0	0/0	0/0	0/0	0/0	0/0	0/0	0/1	0/0	0/0	0/0	0/0	0/0
Chromosome1-1722322	INS	PRECISE	10943	0	0	0/0	0/0	0/0	0/0	0/0	0/0	0/0	0/0	0/0	0/0	0/1	0/0	1/1	0/0
Chromosome1-1722324	BND	PRECISE	0	0	0	0/0	0/0	0/0	0/0	0/0	0/0	0/0	0/0	0/0	0/0	0/0	0/0	0/1	0/0
Chromosome1-1722326	INS	PRECISE	5999	0	0	0/0	0/0	0/0	0/0	0/0	0/0	0/0	0/0	1/1	0/0	0/0	0/0	0/0	0/0
Chromosome1-1722327	INS	PRECISE	4923	0	0	0/0	0/0	0/0	0/0	0/0	0/0	0/0	0/0	0/0	0/0	0/0	0/1	0/0	0/0
Chromosome1-1722829	INS	PRECISE	238	0	0	0/0	0/0	0/0	0/0	0/0	0/0	0/0	0/0	0/0	0/0	0/0	0/0	0/0	0/1
Chromosome1-1722976	DEL	PRECISE	51	0	0	0/0	0/1	0/1	0/0	1/1	1/1	0/1	0/1	1/1	0/1	0/1	0/0	0/1	1/1
Chromosome1-1723126	INS	PRECISE	162	0	0.5	0/0	0/0	0/0	0/0	0/0	0/0	0/0	0/0	0/1	0/0	0/1	0/0	0/1	0/0
Chromosome1-1723519	DEL	PRECISE	1537	0	0	0/0	0/1	0/1	0/0	1/1	1/1	0/1	0/1	1/1	0/1	0/0	0/0	0/0	1/1
Chromosome1-1724117	DEL	PRECISE	54	0	0	0/0	0/0	0/0	0/0	0/1	0/1	0/0	0/0	0/1	0/0	0/0	0/0	0/0	0/0
Chromosome1-1727266	INS	PRECISE	62	0	0	0/0	0/0	0/0	0/0	0/1	0/1	0/0	0/0	1/1	0/0	0/0	0/0	0/0	0/0
Chromosome1-1727783	DEL	PRECISE	767	0.5	0.5	0/0	0/0	0/0	0/0	0/0	0/0	0/0	0/0	0/1	0/0	0/0	0/1	0/0	0/0
Chromosome1-1727885	DEL	PRECISE	951	0	0	0/0	0/0	0/0	0/0	0/0	0/0	0/0	0/0	0/1	0/0	0/1	0/1	0/1	0/0
Chromosome1-1728226	INS	PRECISE	63	0	0	0/0	0/0	0/0	0/0	0/0	0/0	0/0	0/0	0/0	0/0	0/0	0/1	0/0	0/0
Chromosome1-1728413	INS	PRECISE	446	0	0	0/0	0/0	0/0	0/1	0/0	0/0	0/0	0/0	0/0	0/0	0/0	0/0	0/0	0/0
Chromosome1-1728461	DEL	PRECISE	133	0	0	0/0	0/0	0/0	0/0	0/0	0/0	0/0	0/0	0/1	0/0	0/1	0/1	0/1	0/0
Chromosome1-1728686	INS	PRECISE	71	0	0	0/0	0/0	0/0	0/0	0/0	0/0	0/0	0/0	0/0	0/0	0/0	0/0	0/0	0/1
Chromosome1-1728927	INV	PRECISE	3162	0.577	0	0/0	0/0	0/0	0/0	0/0	0/0	0/0	0/0	0/1	0/0	0/1	0/0	0/1	0/0
Chromosome1-1729103	DEL	PRECISE	85	0	0	0/0	0/0	0/0	0/1	0/0	0/0	0/0	0/0	0/0	0/0	0/0	0/0	0/0	0/0
Chromosome1-1729216	BND	PRECISE	0	0	0	0/0	0/0	0/0	0/0	0/0	0/0	0/0	0/0	0/1	0/0	0/0	0/0	0/0	0/0
Chromosome1-1729223	INS	PRECISE	161	0	0.707	0/0	0/0	0/0	0/0	0/0	0/0	0/1	0/0	0/0	0/0	0/0	0/0	0/0	0/0
Chromosome1-1729248	BND	PRECISE	0	0	0	0/0	0/0	0/0	0/0	0/0	0/0	0/0	0/0	0/0	0/0	0/0	0/1	0/0	0/0
Chromosome1-1729628	BND	PRECISE	0	0	0	0/0	0/0	0/0	0/0	0/0	0/0	0/0	0/0	0/0	0/0	0/1	0/0	0/1	0/0
Chromosome1-1729863	DEL	PRECISE	1911	0	0	0/0	0/0	0/0	0/0	0/0	0/0	0/0	0/0	0/1	0/0	0/0	0/1	0/0	0/0
Chromosome1-1730379	INS	PRECISE	363	0	0.707	0/0	0/0	0/0	0/0	0/0	0/0	0/0	0/0	0/1	0/0	0/0	0/1	0/0	0/0
Chromosome1-1730561	DEL	PRECISE	328	0	0	0/0	0/0	0/0	0/0	0/0	0/0	0/0	0/0	0/0	0/0	0/0	0/0	0/0	0/1

Chromosome1-1730963	DEL	PRECISE	162	0	0	0/0	0/0	0/0	0/0	0/0	0/0	0/0	0/0	0/1	0/0	0/1	0/1	0/0	0/0
Chromosome1-1731002	DEL	PRECISE	5292	0	0	0/0	0/0	0/0	0/0	0/0	0/0	0/0	0/0	0/1	0/0	0/1	0/1	0/0	0/0
Chromosome1-1732283	INS	PRECISE	1939	0	0	0/0	0/0	0/0	0/0	0/0	0/0	0/0	0/0	0/0	0/0	0/0	0/0	0/0	0/1
Chromosome1-1733162	DEL	PRECISE	442	0	0	0/0	0/0	0/0	0/0	0/0	0/0	0/0	0/0	0/1	0/0	0/0	0/1	0/0	0/0
Chromosome1-1733283	INS	PRECISE	1009	0	0.707	0/0	0/0	0/0	0/0	0/0	0/0	0/0	0/0	0/0	0/0	0/1	0/0	0/1	0/0
Chromosome1-1734222	BND	PRECISE	0	0	0	0/0	0/0	0/0	0/0	0/0	0/0	0/0	0/0	0/1	0/0	0/1	0/0	0/1	0/0
Chromosome1-1734848	DEL	PRECISE	52	0	0	0/0	0/0	0/0	0/0	0/0	0/0	0/0	0/0	0/1	0/0	0/1	0/1	0/0	0/0
Chromosome1-1735052	DEL	PRECISE	206	0	0	0/0	0/0	0/0	0/0	0/0	0/0	0/0	0/0	0/1	0/0	0/1	0/1	0/1	0/0
Chromosome1-1735113	INS	PRECISE	554	0	0.408	0/0	0/1	0/1	0/0	0/1	0/1	0/0	0/0	0/0	0/1	0/0	0/0	0/0	0/1
Chromosome1-1736233	DEL	PRECISE	83	0.5	0.5	0/0	0/0	0/0	0/0	0/0	0/0	0/0	0/0	0/1	0/0	0/1	0/1	0/1	0/0
Chromosome1-1736352	DEL	PRECISE	99	0	0	0/0	0/0	0/0	1/1	0/1	1/1	0/0	0/0	0/1	0/0	0/0	0/0	0/0	0/0
Chromosome1-1737067	INS	PRECISE	998	0	0.816	0/0	0/0	0/0	0/0	0/0	0/0	0/0	0/0	0/1	0/0	0/1	0/1	0/1	0/0
Chromosome1-1737449	INS	PRECISE	1538	0	0	0/0	0/0	0/0	0/0	0/0	0/0	0/0	0/0	0/0	0/0	0/0	0/0	0/0	0/1
Chromosome1-1738367	DEL	PRECISE	484	0	0	0/0	0/1	0/1	1/1	1/1	1/1	0/1	0/1	1/1	0/1	0/1	0/1	0/1	1/1
Chromosome1-1739464	DEL	PRECISE	88	0	0	0/0	0/0	0/0	0/1	0/0	0/0	0/0	0/0	0/0	0/0	0/0	0/0	0/0	0/0
Chromosome1-1739572	BND	PRECISE	0	0	0	0/0	0/0	0/0	0/0	0/0	0/0	0/0	0/0	0/0	0/0	0/0	0/1	0/0	0/0
Chromosome1-1740463	BND	PRECISE	0	0	0	0/0	0/0	0/0	0/0	0/0	0/1	0/0	0/0	0/0	0/0	0/0	0/0	0/0	0/0
Chromosome1-1741014	DEL	PRECISE	379	0.267	0.267	0/1	0/1	1/1	1/1	1/1	1/1	0/1	0/1	1/1	0/1	0/1	0/1	0/1	1/1
Chromosome1-1741752	INS	PRECISE	3691	0	0	0/0	0/0	0/0	1/1	0/0	0/0	0/0	0/0	0/0	0/0	0/0	0/0	0/0	0/0
Chromosome1-1742476	INS	PRECISE	1118	0	0.5	0/0	0/0	0/0	0/0	0/0	0/0	0/0	0/0	0/1	0/0	1/1	0/0	0/1	0/0
Chromosome1-1743291	INS	PRECISE	120	0	0	0/0	0/0	0/0	0/1	0/0	0/0	0/0	0/0	0/0	0/0	0/0	0/0	0/0	0/0
Chromosome1-1743296	DEL	PRECISE	54	0	0	0/0	0/0	0/0	0/0	0/1	0/1	0/0	0/0	0/1	0/0	0/0	0/0	0/0	0/1
Chromosome1-1743603	DEL	PRECISE	1549	0.516	0	0/0	0/1	0/1	0/0	0/1	0/1	0/0	0/0	0/1	0/1	0/1	0/1	0/1	1/1
Chromosome1-1746114	INS	PRECISE	1377	0	0	0/0	0/0	0/0	0/0	0/0	0/0	0/1	0/1	0/0	0/0	0/0	0/0	0/0	0/0
Chromosome1-1746434	INS	PRECISE	162	0	0.5	0/0	0/0	0/0	0/0	0/0	0/0	0/0	0/0	0/0	0/0	1/1	0/1	1/1	0/0
Chromosome1-1746820	INS	PRECISE	744	0	0.957	0/0	0/0	0/0	0/0	0/0	0/0	0/0	0/0	0/1	0/0	1/1	0/1	0/1	0/0
Chromosome1-1747214	BND	PRECISE	0	0	0	0/0	0/0	0/0	0/0	0/0	0/0	0/0	0/0	0/0	0/0	1/1	0/0	0/1	0/0
Chromosome1-1747783	DEL	PRECISE	18676	0	0	0/0	0/0	0/0	0/0	0/0	0/0	0/0	0/0	0/1	0/0	1/1	1/1	1/1	0/0
Chromosome1-1748087	INS	PRECISE	1133	0	0	0/0	0/0	0/0	0/0	0/0	0/0	0/0	0/0	0/0	0/0	0/0	0/0	0/0	0/1
Chromosome1-1748686	INS	PRECISE	4731	0	0	0/0	0/0	0/0	0/0	0/0	0/0	0/0	0/0	0/0	0/0	0/0	0/0	0/0	1/1
Chromosome1-1748781	INS	PRECISE	53	0	0	0/0	0/1	0/1	0/0	0/1	0/1	0/0	0/0	0/0	0/1	0/0	0/0	0/0	1/1
Chromosome1-1749882	DEL	PRECISE	626	0	0	0/0	0/1	0/1	0/0	0/1	0/1	0/0	0/0	0/0	0/1	0/0	0/0	0/0	1/1
Chromosome1-1750289	INS	PRECISE	3755	0	0.816	0/0	0/0	0/0	0/0	0/0	0/0	0/0	0/0	0/1	0/0	1/1	1/1	0/1	0/0
Chromosome1-1751398	DEL	PRECISE	70	0	0	0/0	0/0	0/0	0/0	0/0	0/0	0/1	0/1	0/0	0/0	0/0	0/0	0/0	0/0
Chromosome1-1751809	INS	PRECISE	131	0	0.516	0/0	0/1	0/1	0/0	0/1	0/1	0/0	0/0	0/0	0/1	0/0	0/0	0/0	0/1
Chromosome1-1751810	DEL	PRECISE	1370	0	0	0/0	0/0	0/0	0/0	0/0	0/0	0/0	0/0	1/1	0/0	0/1	0/1	0/1	0/0
Chromosome1-1751987	DEL	PRECISE	732	0	0	0/0	0/0	0/0	0/0	0/0	0/0	0/0	0/0	1/1	0/0	0/1	0/1	0/1	0/0
Chromosome1-1752110	INS	PRECISE	132	0	0	0/0	0/0	0/0	0/0	0/0	0/0	0/1	0/1	0/0	0/0	0/0	0/0	0/0	0/0
Chromosome1-1752268	BND	PRECISE	0	0	0	0/0	0/0	0/0	0/0	0/0	0/0	0/0	0/0	0/0	0/0	0/1	0/0	0/0	0/0
Chromosome1-1752569	DEL	PRECISE	623	0.316	0	0/0	0/1	0/1	0/0	0/1	0/1	0/0	0/0	0/1	0/1	0/1	0/1	0/1	1/1
Chromosome1-1752818	DEL	PRECISE	61	0	0	0/0	0/0	0/0	0/0	0/0	0/0	0/0	0/0	0/1	0/0	0/1	0/1	0/1	0/0
Chromosome1-1753094	DEL	PRECISE	120	0	0	0/0	0/0	0/0	0/0	0/0	0/0	0/0	0/0	0/0	0/0	0/0	0/0	0/0	0/1
Chromosome1-1753626	DEL	PRECISE	5728	0	0	0/0	0/0	0/0	0/0	0/0	0/0	0/0	0/0	0/1	0/0	0/1	0/1	0/1	0/0
Chromosome1-1754200	INS	PRECISE	76	0	0	0/0	0/0	0/0	0/0	0/0	0/0	0/0	0/0	0/0	0/0	0/0	0/0	0/0	0/1
Chromosome1-1754939	DEL	PRECISE	214	0	0	0/0	0/0	0/0	0/0	0/0	0/0	0/0	0/0	0/0	0/0	0/0	0/0	0/0	1/1
Chromosome1-1755136	BND	PRECISE	0	0	0	0/0	0/0	0/0	0/0	0/0	0/0	0/0	0/0	0/0	0/0	0/0	0/1	0/1	0/0
Chromosome1-1755235	INS	PRECISE	1015	0	0.408	0/0	0/1	0/1	0/0	0/1	0/1	0/0	0/0	0/0	0/1	0/0	0/0	0/0	1/1
Chromosome1-1756638	DEL	PRECISE	92	0	0	0/0	0/0	0/0	0/0	0/0	0/0	0/0	0/0	0/0	0/0	0/0	0/0	0/0	0/1
Chromosome1-1757715	DEL	PRECISE	213	0	0	0/0	0/1	0/1	0/0	0/1	0/1	0/0	0/0	0/1	0/1	0/1	0/1	0/1	1/1
Chromosome1-1757923	INS	PRECISE	279	0	0	0/0	0/1	0/1	0/0	0/1	0/1	0/0	0/0	0/0	0/1	0/0	0/0	0/0	1/1
Chromosome1-1758747	INS	PRECISE	120	0	0.5	0/0	0/0	0/0	0/0	0/0	0/0	0/0	0/0	0/1	0/0	0/1	0/1	0/1	0/0
Chromosome1-1760605	DEL	PRECISE	3840	0	0	0/0	0/0	0/0	0/0	0/0	0/0	0/0	0/0	0/0	0/0	0/0	0/0	0/0	0/1
Chromosome1-1761089	INS	PRECISE	9503	0	0	0/0	0/0	0/0	0/0	0/0	0/0	0/0	0/0	0/0	0/0	0/0	0/0	0/0	0/1
Chromosome1-1761089	BND	PRECISE	0	0	0	0/0	0/0	0/0	0/0	0/0	0/0	0/0	0/0	0/0	0/0	0/0	0/0	0/0	0/1
Chromosome1-1761136	BND	PRECISE	0	0	0	0/0	0/0	0/0	0/0	0/0	0/1	0/0	0/0	0/0	0/0	0/0	0/0	0/0	0/0
Chromosome1-1761563	INS	PRECISE	1551	0	0	0/0	0/0	0/0	0/0	0/0	0/0	0/0	0/0	0/0	0/0	0/0	0/0	0/1	0/0
Chromosome1-1762089	INS	PRECISE	5555	0	0	0/0	0/0	0/0	0/0	0/0	0/0	0/0	0/0	0/0	0/0	0/0	0/0	0/0	1/1
Chromosome1-1762325	BND	PRECISE	0	0	0	0/0	0/0	0/0	0/0	0/0	0/0	0/0	0/0	0/0	0/0	0/0	0/1	0/0	0/0
Chromosome1-1762404	INS	PRECISE	133	0	0	0/0	0/0	0/0	0/0	0/0	0/0	0/0	0/0	0/0	0/0	0/1	0/0	0/1	0/0
Chromosome1-1762878	DEL	PRECISE	138	0	0	0/0	0/1	0/1	0/0	0/1	0/1	0/0	0/0	0/1	0/1	0/1	1/1	0/1	0/1
Chromosome1-1762951	INS	PRECISE	1647	0	0	0/0	0/0	0/0	0/1	0/0	0/0	0/0	0/0	0/0	0/0	0/0	0/0	0/0	0/0
Chromosome1-1763259	DEL	PRECISE	1574	0	0	0/0	0/0	0/0	0/0	0/0	0/0	0/0	0/0	0/1	0/0	0/1	0/1	0/1	0/0
Chromosome1-1763313	DEL	PRECISE	112	0	0	0/0	0/1	0/1	0/1	0/1	0/1	0/0	0/0	0/0	0/1	0/0	0/0	0/0	1/1
Chromosome1-1763466	DEL	PRECISE	226	0	0	0/0	0/1	0/1	0/1	0/1	0/1	0/0	0/0	0/1	0/1	0/1	0/1	0/1	1/1
Chromosome1-1763474	INS	PRECISE	192	0	0	0/0	0/0	0/0	0/0	0/1	0/1	0/1	0/1	0/1	0/0	0/0	0/0	0/0	0/0
Chromosome1-1765620	DEL	PRECISE	225	0	0	0/0	0/0	0/0	0/0	0/0	0/0	0/0	0/0	0/1	0/0	0/1	0/1	0/1	0/0
Chromosome1-1766790	DEL	PRECISE	86	0	0	0/0	0/0	0/0	0/0	0/0	0/0	0/0	0/0	0/1	0/0	0/1	0/1	0/1	0/0
Chromosome1-1767007	DEL	PRECISE	1610	0	0	0/0	0/0	0/0	0/0	0/0	0/0	0/0	0/0	0/1	0/0	0/1	0/1	0/1	0/0
Chromosome1-1767458	DEL	PRECISE	91	0	0	0/0	0/0	0/0	0/0	0/0	0/0	0/0	0/0	0/1	0/0	0/1	0/1	0/1	0/0
Chromosome1-1767990	DEL	PRECISE	444	0	0	0/0	0/0	0/0	0/0	0/0	0/0	0/0	0/0	0/1	0/0	0/1	0/1	0/1	0/0
Chromosome1-1768560	DEL	PRECISE	593	0	0	0/0	0/0	0/0	0/0	0/0	0/0	0/0	0/0	0/1	0/0	0/1	0/1	0/1	0/0
Chromosome1-1768967	BND	PRECISE	0	0	0	0/0	0/0	0/0	0/0	0/0	0/0	0/0	0/0	0/0	0/0	0/0	0/1	0/0	0/0
Chromosome1-1770428	DEL	PRECISE	1371	0	0	0/0	0/0	0/0	0/0	0/0	0/0	0/0	0/0	0/1	0/0	1/1	1/1	1/1	0/0

Chromosome1-1772192	INS	PRECISE	78	0	0	0/0	0/0	0/0	0/0	0/0	0/0	0/0	0/0	0/1	0/0	0/0	1/1	0/0	0/0
Chromosome1-1772366	INS	PRECISE	548	0	0	0/0	0/0	0/0	0/0	0/0	0/0	0/0	0/0	0/1	0/0	0/0	0/0	0/0	0/0
Chromosome1-1772653	INS	PRECISE	130	0.949	0.316	0/0	0/1	0/1	0/0	0/1	0/1	0/0	0/0	0/1	0/1	0/1	0/1	0/1	0/1
Chromosome1-1772763	INS	PRECISE	150	0	0	0/0	0/0	0/0	0/0	0/0	0/0	0/0	0/0	0/0	0/0	0/0	0/0	0/0	0/1
Chromosome1-1772988	INS	PRECISE	57	0	0	0/0	0/0	0/0	0/0	0/0	0/0	0/0	0/0	0/1	0/0	0/1	0/1	0/1	0/0
Chromosome1-1773078	DEL	PRECISE	483	0	0	0/0	0/0	0/0	0/0	0/0	0/0	0/0	0/0	0/1	0/0	0/1	0/1	0/1	0/0
Chromosome1-1773177	BND	PRECISE	0	0	0	0/0	0/0	0/0	0/0	0/0	0/0	0/0	0/0	0/0	0/1	0/0	0/0	0/0	0/0
Chromosome1-1773217	INS	PRECISE	102	0	0.483	0/0	0/1	0/1	0/0	0/1	1/1	0/0	0/0	0/1	0/1	0/1	0/1	0/1	0/1
Chromosome1-1773518	INS	PRECISE	159	0	0	0/0	0/0	0/0	0/0	0/0	0/0	0/0	0/0	0/0	0/0	0/0	0/0	0/0	0/1
Chromosome1-1774229	DEL	PRECISE	4899	0	0	0/0	0/0	0/0	0/0	0/0	0/0	0/0	0/0	0/1	0/0	0/1	0/1	0/1	0/0
Chromosome1-1774232	DEL	PRECISE	60	0	0	0/0	0/0	0/0	0/0	0/0	0/0	0/0	0/0	0/0	0/0	0/0	0/0	0/0	0/1
Chromosome1-1774756	INS	PRECISE	2599	0	0.753	0/0	1/1	1/1	0/0	1/1	0/1	0/0	0/0	0/0	0/1	0/0	0/0	0/0	0/1
Chromosome1-1774769	DEL	PRECISE	376	0	0	0/0	0/0	0/0	0/0	0/0	0/0	0/0	0/0	0/1	0/0	0/1	0/1	0/0	0/0
Chromosome1-1776103	DEL	PRECISE	644	0	0	0/0	0/0	0/0	0/0	0/0	0/0	0/0	0/0	0/1	0/0	0/1	0/0	0/1	0/0
Chromosome1-1776356	INS	PRECISE	7012	0	0	0/0	0/0	0/0	0/0	0/0	0/0	0/0	0/0	0/0	0/0	0/0	0/0	0/0	0/1
Chromosome1-1776357	BND	PRECISE	0	0	0	0/0	0/0	0/0	0/0	0/0	0/0	0/0	0/0	0/0	0/0	0/0	0/0	0/0	0/1
Chromosome1-1777343	DEL	PRECISE	71	0	0	0/0	0/0	0/0	0/0	0/1	1/1	0/0	0/0	0/1	0/0	0/0	0/0	0/0	0/0
Chromosome1-1777757	INS	PRECISE	451	0	0	0/0	0/0	0/0	0/0	0/0	0/0	0/0	0/0	0/0	0/0	0/0	0/1	0/0	0/0
Chromosome1-1778550	DEL	PRECISE	211	0	0	0/0	0/1	0/1	0/0	0/1	0/1	0/0	0/0	0/1	0/1	0/1	0/1	0/1	1/1
Chromosome1-1778840	INS	PRECISE	137	0	0.408	0/0	0/1	0/1	0/0	0/1	0/1	0/0	0/0	0/0	0/1	0/0	0/0	0/0	0/1
Chromosome1-1778883	DEL	PRECISE	537	0	0	0/0	0/0	0/0	0/0	0/0	0/0	0/0	0/0	0/0	0/0	0/0	0/0	0/0	0/1
Chromosome1-1779124	DEL	PRECISE	153	0	0	0/0	0/1	0/1	0/0	0/1	0/1	0/0	0/0	0/0	0/1	0/0	0/0	0/0	0/1
Chromosome1-1779634	DEL	PRECISE	237	0	0	0/0	0/1	0/1	0/0	0/1	0/1	0/0	0/0	0/0	0/1	0/0	0/0	0/0	0/1
Chromosome1-1780716	INS	PRECISE	2871	0	0	0/0	0/0	0/0	0/1	0/0	0/0	0/0	0/0	0/0	0/0	0/0	0/0	0/0	0/0
Chromosome1-1780716	INS	PRECISE	13631	0	0	0/0	0/0	0/0	0/0	0/1	0/0	0/0	0/0	0/0	0/0	0/0	0/0	0/0	0/0
Chromosome1-1781023	DEL	PRECISE	69	0	0	0/0	0/0	0/0	0/0	0/0	0/0	0/1	0/0	0/0	0/0	0/0	0/0	0/0	0/0
Chromosome1-1781420	DEL	PRECISE	712	0	0	0/0	0/0	0/0	0/0	0/1	0/1	0/0	0/0	1/1	0/0	0/0	0/0	0/0	0/0
Chromosome1-1784660	INS	PRECISE	53	0	0	0/0	0/0	0/0	0/0	0/0	0/0	0/1	0/0	0/0	0/0	0/0	0/1	0/0	0/0
Chromosome1-1784901	DEL	PRECISE	134	0	0	0/0	0/0	0/0	0/0	0/0	0/0	0/0	0/0	0/0	0/0	0/0	0/1	0/0	0/0
Chromosome1-1785626	INS	PRECISE	124	0	0.354	0/0	0/1	0/1	1/1	1/1	1/1	0/0	0/0	1/1	0/0	0/0	0/0	0/0	1/1
Chromosome1-1786176	DEL	PRECISE	5250	0	0	0/0	0/0	0/0	0/0	0/0	0/0	0/1	0/0	0/1	0/0	0/0	0/1	0/0	0/1
Chromosome1-1786323	INS	PRECISE	12565	0	0	0/0	0/0	0/1	0/0	0/0	0/0	0/0	0/0	0/0	0/0	0/0	0/0	0/0	0/0
Chromosome1-1787248	DEL	PRECISE	70	0	0	0/0	0/0	0/0	0/0	0/0	0/0	0/1	0/0	0/1	0/0	0/0	0/0	0/0	0/1
Chromosome1-1787426	DEL	PRECISE	208	0	0	0/0	0/0	0/0	0/0	0/0	0/0	0/0	0/0	0/0	0/0	0/0	0/0	0/0	0/1
Chromosome1-1787454	INS	PRECISE	475	0	0.894	0/0	0/0	0/0	0/0	0/0	0/0	0/0	0/0	0/0	0/0	0/0	0/0	0/0	0/1
Chromosome1-1787654	DEL	PRECISE	1296	0	0	0/0	0/1	0/0	0/0	0/1	0/0	0/1	0/1	0/1	0/1	0/0	0/1	0/0	0/1
Chromosome1-1788236	DEL	PRECISE	5301	0	0	0/0	0/1	0/0	0/0	0/1	0/0	0/1	0/1	0/1	0/1	0/0	0/1	0/0	0/1
Chromosome1-1789704	DEL	PRECISE	698	0	0	0/0	0/1	0/1	1/1	1/1	1/1	0/0	0/0	1/1	0/1	0/0	0/0	0/0	1/1
Chromosome1-1789796	INS	PRECISE	52	0	0.516	0/0	0/1	0/1	0/0	0/1	0/1	0/0	0/0	0/0	0/1	0/0	0/0	0/0	1/1
Chromosome1-1792364	DEL	PRECISE	417	0	0	0/0	0/1	0/1	0/0	0/1	0/1	0/0	0/0	0/0	0/1	0/0	0/0	0/0	1/1
Chromosome1-1795075	DEL	PRECISE	9884	0	0	0/0	0/1	0/0	0/0	0/1	0/0	0/1	0/0	1/1	0/1	0/0	0/0	0/0	0/1
Chromosome1-1796154	INS	PRECISE	155	0	0	0/0	0/0	0/0	0/0	0/0	0/0	0/0	0/0	0/0	0/0	1/1	0/0	1/1	0/0
Chromosome1-1797199	INS	PRECISE	9606	0	0	0/0	0/0	0/0	1/1	0/0	0/0	0/0	0/0	0/0	0/0	0/0	0/0	0/0	0/0
Chromosome1-1797199	INS	PRECISE	17117	0	0	0/0	0/0	0/0	0/0	0/0	0/1	0/0	0/0	0/0	0/0	0/0	0/0	0/0	0/0
Chromosome1-1797200	BND	PRECISE	0	0	0	0/0	0/0	0/0	0/0	0/0	0/0	0/0	0/0	0/1	0/0	0/0	0/0	0/0	0/0
Chromosome1-1797909	INS	PRECISE	1365	0	0.957	0/0	0/0	0/0	1/1	0/1	0/1	0/0	0/0	1/1	0/0	0/0	0/0	0/0	0/0
Chromosome1-1800024	DEL	PRECISE	144	0	0	0/0	0/0	0/0	0/0	0/0	0/0	0/0	0/0	0/0	0/0	0/1	0/0	0/0	0/0
Chromosome1-1800130	DEL	PRECISE	2797	0	0	0/0	0/0	0/0	1/1	0/0	0/0	0/0	0/0	0/0	0/0	0/0	0/0	0/0	0/0
Chromosome1-1800258	DEL	PRECISE	807	0	0	0/0	0/0	0/0	0/0	0/0	0/0	1/1	1/1	0/0	0/0	0/0	0/0	0/0	0/0
Chromosome1-1801299	DEL	PRECISE	114	0	0	0/0	0/1	0/1	0/0	0/1	0/1	0/0	0/0	0/0	0/1	0/0	0/0	0/0	1/1
Chromosome1-1801619	BND	PRECISE	0	0	0	0/0	0/0	0/0	0/0	0/0	0/0	0/0	0/0	0/1	0/0	0/0	0/1	0/1	0/0
Chromosome1-1801918	DEL	PRECISE	4319	0	0	0/0	0/1	0/1	0/0	0/1	0/1	0/0	0/0	0/0	0/1	0/0	0/0	0/0	1/1
Chromosome1-1802020	DEL	PRECISE	175	0	0	0/0	0/0	0/0	0/0	0/0	0/0	0/0	0/0	0/1	0/0	0/1	0/1	0/0	0/0
Chromosome1-1802210	BND	PRECISE	0	0	0	0/0	0/0	0/0	0/0	0/0	0/0	0/0	0/0	0/1	0/0	0/0	0/0	0/0	0/0
Chromosome1-1802212	BND	PRECISE	0	0	0	0/0	0/0	0/0	0/0	0/0	0/0	0/0	0/0	0/0	0/0	0/0	1/1	0/0	0/0
Chromosome1-1802332	DEL	PRECISE	830	0	0	0/0	0/0	0/0	0/0	0/0	0/0	0/0	0/0	0/1	0/0	0/1	0/1	0/0	0/0
Chromosome1-1802457	BND	PRECISE	0	0	0	0/0	0/1	0/1	0/0	0/0	0/1	0/0	0/0	0/0	0/1	0/0	0/0	0/0	1/1
Chromosome1-1802993	INS	PRECISE	462	0	0	0/0	0/0	0/0	0/0	0/0	0/0	0/1	0/1	0/0	0/0	0/0	0/0	0/0	0/0
Chromosome1-1802993	INS	PRECISE	2247	0	0	0/0	0/0	0/0	0/1	0/0	0/0	0/0	0/0	0/0	0/0	0/0	0/0	0/0	0/0
Chromosome1-1803374	DEL	PRECISE	1104	0	0	0/0	0/0	0/0	0/0	0/0	0/0	0/0	0/0	0/0	0/0	0/1	0/1	0/1	0/0
Chromosome1-1803468	INS	PRECISE	4936	0	0	0/0	0/0	0/0	0/0	0/0	0/0	0/0	0/0	0/1	0/0	0/0	0/0	0/0	0/0
Chromosome1-1803835	BND	PRECISE	0	0	0	0/0	0/0	0/1	0/0	0/0	0/0	0/0	0/0	0/0	0/0	0/0	0/0	0/0	0/0
Chromosome1-1803835	BND	PRECISE	0	0	0	0/0	0/0	0/0	0/0	0/1	0/0	0/0	0/0	0/0	0/0	0/0	0/0	0/0	0/0
Chromosome1-1805402	BND	PRECISE	0	0	0	0/0	0/0	0/0	0/0	0/0	0/0	0/0	0/0	0/1	0/0	0/0	0/0	0/0	0/0
Chromosome1-1805781	BND	PRECISE	0	0	0	0/0	0/0	0/0	0/0	0/0	0/0	0/0	0/0	0/0	0/0	0/0	0/1	0/0	0/0
Chromosome1-1805781	BND	PRECISE	0	0	0	0/0	0/0	0/0	0/0	0/0	0/0	0/0	0/0	0/0	0/0	0/1	0/1	0/0	0/0
Chromosome1-1805782	BND	PRECISE	0	0	0	0/0	0/0	0/0	0/0	0/0	0/0	0/0	0/0	0/1	0/0	0/0	0/0	0/0	0/0
Chromosome1-1805782	BND	PRECISE	0	0	0	0/0	0/0	0/0	0/0	0/0	0/0	0/0	0/0	0/0	0/0	0/1	0/1	0/1	0/0
Chromosome1-1806204	BND	PRECISE	0	0	0	0/0	0/1	0/1	0/0	0/0	0/1	0/0	0/0	0/0	0/1	0/0	0/0	0/0	1/1
Chromosome1-1807023	INS	PRECISE	8444	0	0	0/0	0/0	0/0	0/0	0/0	0/0	0/0	0/0	0/0	0/0	0/0	0/0	0/0	0/0
Chromosome1-1807948	DEL	PRECISE	678	0	0	0/0	0/0	0/0	0/0	0/0	0/0	0/0	0/0	0/0	0/1	0/0	0/0	0/0	0/0
Chromosome1-1809048	INS	PRECISE	435	0	0.408	0/0	0/1	0/1	0/0	0/1	0/1	0/0	0/0	0/0	0/0	0/0	0/0	0/0	0/1
Chromosome1-1809412	DEL	PRECISE	324	0	0	0/0	0/1	0/1	0/0	0/1	0/1	0/0	0/0	0/0	0/0	0/0	0/0	0/0	0/1

Chromosome1-1809475	DEL	PRECISE	881	0	0	0/0	0/1	0/1	0/0	0/1	0/1	0/0	0/0	0/0	0/0	0/0	0/0	0/0	0/1
Chromosome1-1809562	INS	PRECISE	84	0	0	0/0	0/1	0/1	0/0	0/1	0/1	0/0	0/0	0/0	0/1	0/0	0/0	0/0	0/1
Chromosome1-1809706	DEL	PRECISE	667	0	0	0/0	0/1	0/1	0/0	0/1	0/1	0/0	0/0	0/0	0/0	0/0	0/0	0/0	0/1
Chromosome1-1810294	DEL	PRECISE	96	0	0	0/0	0/1	0/1	0/0	0/0	0/0	0/0	0/0	0/0	0/0	0/0	0/0	0/0	0/1
Chromosome1-1813361	INS	PRECISE	71	0.516	0	0/0	0/0	0/0	0/1	0/0	0/0	0/1	0/1	0/0	0/0	0/0	0/0	0/0	0/0
Chromosome1-1814503	BND	PRECISE	0	0	0	0/0	0/0	0/0	0/0	0/0	0/0	0/0	0/0	0/0	0/0	0/0	0/0	0/0	0/1
Chromosome1-1815073	DEL	PRECISE	50	0	0	0/0	0/0	0/0	0/1	0/0	0/0	0/0	0/0	0/0	0/0	0/0	0/0	0/0	0/0
Chromosome1-1815269	DEL	PRECISE	5443	0	0	0/0	0/1	0/1	1/1	1/1	1/1	0/1	0/0	1/1	0/1	0/1	0/1	0/1	1/1
Chromosome1-1815852	DEL	PRECISE	69	0	0	0/0	0/0	0/0	0/1	0/0	0/0	0/1	0/0	0/0	0/0	0/0	0/0	0/0	0/0
Chromosome1-1816173	BND	PRECISE	0	0	0	0/0	0/0	0/0	0/0	0/0	0/1	0/0	0/0	0/0	0/0	0/0	0/0	0/0	0/0
Chromosome1-1816182	INS	PRECISE	1367	0	0.577	0/0	0/0	0/0	0/0	0/1	0/1	0/0	0/0	0/1	0/0	0/0	0/0	0/0	0/0
Chromosome1-1817596	INS	PRECISE	381	0	0	0/0	0/0	0/0	0/1	0/0	0/0	0/0	0/0	0/0	0/0	0/0	0/0	0/0	0/0
Chromosome1-1817701	DEL	PRECISE	1130	0.5	0.5	0/0	0/0	0/0	0/0	0/0	0/0	0/0	0/0	0/1	0/0	0/1	0/1	0/1	0/0
Chromosome1-1818006	INS	PRECISE	131	0	0	0/0	0/0	0/0	0/0	0/0	0/0	0/0	0/0	0/0	0/0	0/0	0/0	0/0	0/1
Chromosome1-1818207	INS	PRECISE	880	0	0	0/0	0/0	0/0	0/0	0/0	0/0	0/0	0/0	0/1	0/0	0/0	0/0	0/0	0/0
Chromosome1-1818742	DEL	PRECISE	190	0	0	0/0	0/0	0/0	0/0	0/0	0/0	0/0	0/0	0/1	0/0	0/1	0/1	0/1	0/0
Chromosome1-1818910	INS	PRECISE	276	0	0.577	0/0	0/0	0/0	0/0	0/0	0/0	0/0	0/0	0/0	0/0	0/1	0/1	0/1	0/0
Chromosome1-1820024	INS	PRECISE	541	0	0	0/0	0/0	0/0	0/1	0/0	0/0	0/0	0/0	0/0	0/0	0/0	0/0	0/0	0/0
Chromosome1-1820244	INS	PRECISE	5528	0	0	0/0	0/0	0/0	0/0	0/0	0/0	0/0	0/0	0/0	0/0	0/0	1/1	0/0	0/0
Chromosome1-1820245	BND	PRECISE	0	0	0	0/0	0/0	0/0	0/0	0/0	0/0	0/0	0/0	0/0	0/0	0/0	0/1	0/0	0/0
Chromosome1-1820663	INS	PRECISE	58	0	0	0/0	0/0	0/0	0/0	0/0	0/0	0/0	0/0	0/1	0/0	0/1	0/1	0/1	0/0
Chromosome1-1821021	INS	PRECISE	54	0	0	0/0	0/0	0/0	0/0	0/0	0/0	0/0	0/0	0/1	0/0	0/0	0/0	0/0	0/0
Chromosome1-1821373	INS	PRECISE	68	0	0	0/0	0/0	0/0	0/0	0/0	0/0	0/0	0/0	0/1	0/0	0/0	0/0	0/0	0/0
Chromosome1-1821426	INS	PRECISE	5043	0	0	0/0	0/0	0/0	0/1	0/0	0/0	0/0	0/0	0/0	0/0	0/0	0/0	0/0	0/0
Chromosome1-1822500	INS	PRECISE	323	0	0.707	0/0	0/0	0/0	0/0	0/0	0/0	0/1	0/1	0/0	0/0	0/0	0/0	0/0	0/0
Chromosome1-1822805	INS	PRECISE	5006	0	0	0/0	0/0	0/0	0/0	0/0	0/0	0/0	0/0	0/0	0/0	0/0	0/0	0/0	1/1
Chromosome1-1823055	DEL	PRECISE	1795	0	0	0/0	0/0	0/0	0/0	0/0	0/0	0/0	0/0	0/0	0/0	0/0	0/0	0/0	0/1
Chromosome1-1824049	INS	PRECISE	477	0	0	0/0	0/0	0/0	0/0	0/0	0/0	0/0	0/0	0/0	0/0	0/0	0/0	0/0	0/1
Chromosome1-1824722	DEL	PRECISE	338	0	0	0/0	0/0	0/0	0/1	0/0	0/0	0/0	0/0	0/0	0/0	0/0	0/0	0/0	0/0
Chromosome1-1824759	DEL	PRECISE	2216	0	0	0/0	0/0	0/0	1/1	0/0	0/0	0/0	0/0	0/0	0/0	0/0	0/0	0/0	0/0
Chromosome1-1824825	DEL	PRECISE	71	0	0	0/0	0/0	0/0	0/0	0/0	0/0	0/1	0/0	0/0	0/0	0/0	0/0	0/0	0/0
Chromosome1-1825480	DEL	PRECISE	1797	0	0	0/0	0/0	0/0	0/1	0/0	0/0	0/0	0/0	0/0	0/0	0/0	0/0	0/0	0/0
Chromosome1-1826330	DUP	PRECISE	323	0	0	0/0	0/0	0/0	0/0	0/0	0/0	0/0	0/0	0/0	0/0	0/0	0/0	0/0	0/1
Chromosome1-1826390	DEL	PRECISE	688	0	0	0/0	0/1	0/1	0/0	0/1	0/1	0/0	0/0	0/1	0/1	0/1	0/1	0/1	1/1
Chromosome1-1827070	DEL	PRECISE	91	0	0	0/0	0/0	0/0	0/0	0/0	0/0	0/0	0/0	0/1	0/0	0/0	0/1	0/0	0/0
Chromosome1-1827134	DEL	PRECISE	3318	0	0	0/0	0/0	0/0	0/0	0/0	0/0	0/0	0/0	0/1	0/0	0/0	0/1	0/0	0/0
Chromosome1-1827286	DEL	PRECISE	1606	0	0	0/0	0/1	0/1	0/0	0/1	0/1	0/0	0/0	0/0	0/1	0/1	0/0	0/1	1/1
Chromosome1-1828119	BND	PRECISE	0	0	0	0/0	0/0	0/0	0/0	0/0	0/0	0/0	0/0	0/1	0/0	0/0	0/1	0/0	0/0
Chromosome1-1830484	INS	PRECISE	57	0	0	0/0	0/1	0/1	0/0	0/1	0/1	0/0	0/0	0/0	0/1	0/0	0/1	0/0	1/1
Chromosome1-1830815	DEL	PRECISE	631	0	0	0/0	0/0	0/0	1/1	0/0	0/0	0/1	0/1	0/0	0/0	0/0	0/0	0/0	0/0
Chromosome1-1830980	DEL	PRECISE	74	0	0	0/0	0/0	0/0	1/1	0/0	0/0	0/1	0/1	0/0	0/0	0/0	0/0	0/0	0/1
Chromosome1-1831156	BND	PRECISE	0	0	0	0/0	0/0	0/0	0/0	0/0	0/0	0/0	0/0	0/0	0/0	0/1	0/0	0/1	0/0
Chromosome1-1831326	DEL	PRECISE	388	0	0	0/0	0/0	0/0	1/1	0/0	0/0	0/1	0/1	0/0	0/0	0/0	0/0	0/0	0/0
Chromosome1-1831496	DEL	PRECISE	5339	0	0	0/0	0/0	0/0	1/1	0/0	0/0	0/1	0/1	0/0	0/0	0/0	0/0	0/0	0/0
Chromosome1-1832371	DEL	PRECISE	1218	0	0	0/0	0/0	0/0	0/0	0/0	0/0	0/0	0/0	0/0	0/0	0/0	0/1	0/1	0/1
Chromosome1-1832418	DEL	PRECISE	63	0	0	0/0	0/0	0/0	1/1	0/0	0/0	0/1	0/1	0/0	0/0	0/0	0/0	0/0	0/0
Chromosome1-1832585	INS	PRECISE	88	0	0	0/0	0/0	0/0	0/0	0/0	0/0	0/0	0/0	0/0	0/0	0/1	0/1	0/1	0/0
Chromosome1-1832586	INS	PRECISE	2836	0	0	0/0	0/0	0/0	0/0	0/0	0/0	0/0	0/0	0/0	0/0	0/0	0/0	0/0	0/1
Chromosome1-1832983	DEL	PRECISE	235	0	0	0/0	0/0	0/0	0/0	0/0	0/0	0/0	0/0	0/1	0/0	0/0	0/0	0/0	0/0
Chromosome1-1833347	INS	PRECISE	5959	0	0	0/0	0/0	0/0	0/1	0/0	0/0	0/0	0/0	0/0	0/0	0/0	0/0	0/0	0/0
Chromosome1-1833721	DEL	PRECISE	16676	0	0	0/0	0/0	0/0	0/0	0/0	0/0	0/1	1/1	0/0	0/0	0/0	0/0	0/0	0/0
Chromosome1-1834382	INS	PRECISE	6009	0	0	0/0	0/0	0/0	1/1	0/0	0/0	0/0	0/0	0/0	0/0	0/0	0/0	0/0	0/0
Chromosome1-1834580	DEL	PRECISE	292	0	0	0/0	0/0	0/0	0/0	0/0	0/0	0/0	0/0	0/1	0/0	0/1	0/0	0/1	0/0
Chromosome1-1834661	DEL	PRECISE	1339	0	0	0/0	0/0	0/0	0/0	0/0	0/0	0/0	0/0	0/1	0/0	0/1	0/0	0/1	0/0
Chromosome1-1835467	INS	PRECISE	999	0	0	0/0	0/0	0/0	0/0	0/0	0/0	0/0	0/0	1/1	0/0	0/0	0/0	0/0	0/0
Chromosome1-1835618	DEL	PRECISE	4534	0	0	0/0	0/0	0/0	0/0	0/0	0/0	0/1	0/1	1/1	0/0	0/1	0/0	0/1	0/0
Chromosome1-1835934	DEL	PRECISE	146	0	0	0/0	0/0	0/0	1/1	0/0	0/0	0/0	0/0	0/0	0/0	0/0	0/0	0/0	0/0
Chromosome1-1836330	BND	PRECISE	0	0	0	0/0	0/0	0/0	0/0	0/0	0/0	0/0	0/0	0/1	0/0	0/0	0/0	0/1	0/0
Chromosome1-1836762	INS	PRECISE	890	0	0	0/0	0/0	0/0	0/0	0/0	0/0	0/0	0/0	0/1	0/0	0/0	0/0	0/0	0/0
Chromosome1-1836937	DEL	PRECISE	120	0	0	0/0	0/0	0/0	0/0	0/0	0/0	0/0	0/0	0/0	0/0	0/0	0/0	0/0	0/1
Chromosome1-1837359	DEL	PRECISE	62	0	0	0/0	0/0	0/0	0/0	0/0	0/0	0/0	0/0	0/0	0/0	0/0	0/0	0/0	0/1
Chromosome1-1837627	INS	PRECISE	65	0	0	0/0	0/0	0/0	0/0	0/0	0/0	0/0	0/0	0/0	0/0	0/0	0/0	0/0	0/1
Chromosome1-1837862	INS	PRECISE	703	0	0	0/0	0/0	0/0	0/0	0/0	0/0	0/0	0/0	0/0	0/0	0/0	0/0	0/0	0/1
Chromosome1-1838223	DEL	PRECISE	316	0	0	0/0	0/0	0/0	0/0	0/0	0/0	0/0	0/0	0/1	0/0	0/1	0/1	0/1	0/0
Chromosome1-1838295	BND	PRECISE	0	0	0	0/0	0/0	0/0	0/0	0/0	0/0	0/0	0/1	0/0	0/0	0/0	0/0	0/0	0/0
Chromosome1-1840804	DEL	PRECISE	59	0	0	0/0	0/0	0/0	0/0	0/0	0/0	0/0	0/0	0/0	0/0	0/1	0/0	0/1	0/0
Chromosome1-1841427	INS	PRECISE	93	0	0	0/0	0/0	0/0	0/0	0/0	0/0	0/1	1/1	0/0	0/0	0/0	0/0	0/0	0/0
Chromosome1-1843047	INS	PRECISE	50	0	0.957	0/0	0/0	0/0	0/0	0/0	0/0	0/0	0/0	0/1	0/0	1/1	0/1	0/1	0/0
Chromosome1-1844102	INS	PRECISE	50	0	0	0/0	0/0	0/0	0/0	0/0	0/0	0/0	0/0	0/1	0/0	0/0	0/0	0/0	0/0
Chromosome1-1844116	BND	PRECISE	0	0	0	0/0	0/0	0/0	0/0	0/0	0/0	0/0	0/0	0/0	0/0	0/0	0/1	0/0	0/0
Chromosome1-1844328	DEL	PRECISE	812	0	0	0/0	0/0	0/0	0/0	0/0	0/0	0/0	0/0	0/0	0/0	0/1	0/1	0/1	0/0
Chromosome1-1844709	INS	PRECISE	83	0	0.535	0/0	0/1	0/1	0/1	0/1	0/1	0/0	0/0	0/0	1/1	0/0	0/0	0/0	0/1
Chromosome1-1845784	DEL	PRECISE	121	0	0	0/0	0/0	0/0	0/0	0/0	0/0	0/1	0/1	0/0	0/0	0/0	0/0	0/0	0/0

Chromosome1-1846345	DEL	PRECISE	63	0	0	0/0	0/0	0/0	0/0	0/0	0/0	0/0	0/0	0/0	0/0	0/1	0/0	0/1	0/0
Chromosome1-1846640	DEL	PRECISE	779	0	0	0/0	0/0	0/0	0/0	0/0	0/0	0/0	0/0	0/1	0/0	0/1	0/0	0/1	0/0
Chromosome1-1846696	INS	PRECISE	64	0	0	0/0	0/1	0/1	1/1	1/1	1/1	0/0	0/0	0/1	0/1	0/0	0/0	0/0	1/1
Chromosome1-1846973	INS	PRECISE	17506	0	0	0/0	0/0	0/0	0/0	0/0	0/0	0/1	0/0	0/0	0/0	0/0	0/0	0/0	0/0
Chromosome1-1847599	BND	PRECISE	0	0	0	0/0	0/0	0/0	0/0	0/0	0/0	0/0	0/0	0/1	0/0	0/0	0/0	0/0	0/0
Chromosome1-1849025	INS	PRECISE	722	0	0.577	0/0	0/0	0/0	0/0	0/1	0/1	0/0	0/0	0/1	0/0	0/0	0/0	0/0	0/0
Chromosome1-1850762	DEL	PRECISE	83	0	0	0/0	0/0	0/0	0/0	0/0	0/0	0/0	0/0	0/1	0/0	0/1	0/1	0/1	0/0
Chromosome1-1850972	DEL	PRECISE	117	0.5	0.5	0/0	0/0	0/0	0/0	0/0	0/0	0/0	0/0	0/1	0/0	0/1	0/1	0/1	0/0
Chromosome1-1854250	INS	PRECISE	132	0	0	0/0	0/0	0/0	0/0	0/1	0/1	0/0	0/0	1/1	0/0	0/0	0/0	0/0	0/0
Chromosome1-1854488	INS	PRECISE	7504	0	0.707	0/0	0/0	0/0	0/0	0/0	0/0	1/1	1/1	0/0	0/0	0/0	0/0	0/0	0/0
Chromosome1-1855019	BND	PRECISE	0	0	0	0/0	0/1	0/1	0/1	0/0	1/1	0/0	0/0	0/0	0/0	0/0	0/0	0/0	0/0
Chromosome1-1855636	INS	PRECISE	389	0	0.951	0/0	0/1	0/1	0/0	0/1	0/1	0/0	0/0	0/0	0/0	0/0	0/0	0/0	0/0
Chromosome1-1856339	DEL	PRECISE	469	0	0	0/0	0/1	0/1	0/1	0/1	0/1	0/0	0/0	0/0	0/1	1/1	0/0	0/1	0/1
Chromosome1-1856595	DEL	PRECISE	356	0	0	0/0	0/0	0/0	0/0	0/0	0/0	0/0	0/0	0/0	0/0	1/1	0/1	0/1	0/0
Chromosome1-1856889	INS	PRECISE	1570	0	0.707	0/0	0/0	0/0	0/0	0/0	0/0	0/1	0/1	0/0	0/0	0/0	0/0	0/0	0/0
Chromosome1-1857074	BND	PRECISE	0	0	0	0/0	0/0	0/0	0/0	0/0	0/0	0/0	0/0	0/0	0/0	0/1	0/0	0/0	0/0
Chromosome1-1857292	DEL	PRECISE	595	0	0	0/0	0/0	0/0	0/0	0/0	0/0	0/1	0/1	0/0	0/0	0/0	0/0	0/0	0/0
Chromosome1-1857555	DEL	PRECISE	130	0	0	0/0	0/0	0/0	0/0	0/0	0/0	0/0	0/0	0/1	0/0	0/1	0/1	0/1	0/0
Chromosome1-1858119	INS	PRECISE	934	0	0	0/0	0/0	0/0	0/0	0/0	0/0	0/0	0/0	0/0	0/0	0/0	0/0	0/0	0/1
Chromosome1-1858904	INS	PRECISE	5229	0	0	0/0	0/0	0/0	0/0	0/0	0/0	0/0	0/0	0/0	0/0	0/0	0/0	0/0	1/1
Chromosome1-1858963	INS	PRECISE	1103	0	0.707	0/0	0/0	0/0	0/0	0/0	0/0	0/0	0/0	0/0	0/0	0/1	0/0	0/1	0/0
Chromosome1-1860349	DEL	PRECISE	194	0	0	0/0	0/0	0/0	0/0	0/0	0/0	0/0	0/0	0/1	0/0	0/0	0/0	0/0	0/0
Chromosome1-1860369	INS	PRECISE	173	0	0	0/0	0/0	0/0	0/0	0/0	0/0	0/0	0/0	0/0	0/0	0/0	0/0	0/0	0/1
Chromosome1-1860758	DEL	PRECISE	513	0	0	0/0	0/0	0/0	0/1	0/1	0/1	0/1	0/1	1/1	0/0	1/1	0/1	0/1	0/1
Chromosome1-1860841	DEL	PRECISE	925	0	0	0/0	0/0	0/0	0/0	0/0	0/0	0/1	0/1	0/0	0/0	0/0	0/0	0/0	0/0
Chromosome1-1861060	DEL	PRECISE	545	0.816	0	0/0	0/0	0/0	0/0	0/0	0/0	0/1	0/1	0/1	0/0	1/1	0/1	0/1	0/0
Chromosome1-1861198	INS	PRECISE	696	0	0	0/0	0/0	0/0	0/0	0/0	0/0	0/0	0/0	0/0	0/0	0/0	0/0	0/0	0/1
Chromosome1-1862314	BND	PRECISE	0	0	0	0/0	0/0	0/0	0/0	0/0	0/0	0/0	0/0	0/0	0/0	0/0	0/0	0/0	0/1
Chromosome1-1862410	INS	PRECISE	2317	0	0	0/0	0/0	0/0	0/0	0/0	0/0	0/0	0/1	0/0	0/0	0/0	0/0	0/0	0/0
Chromosome1-1862495	DEL	PRECISE	12337	0	0	0/0	0/0	0/0	1/1	1/1	0/1	0/0	0/0	1/1	0/0	0/1	0/1	1/1	0/0
Chromosome1-1862704	INS	PRECISE	50	0	0.577	0/0	0/0	0/0	0/0	0/0	0/0	0/1	0/1	0/0	0/0	0/0	0/0	0/0	0/0
Chromosome1-1862751	DEL	PRECISE	512	0	0	0/0	0/0	0/0	0/0	0/0	0/0	0/1	0/1	0/0	0/0	0/0	0/0	0/0	0/0
Chromosome1-1863087	DEL	PRECISE	210	0	0	0/0	0/0	0/0	0/0	0/0	0/0	0/0	0/0	0/0	0/0	0/0	0/0	0/0	0/1
Chromosome1-1863332	INS	PRECISE	1244	0	0	0/0	0/0	0/0	0/0	0/0	0/0	0/0	0/0	0/0	0/0	0/0	0/0	0/0	0/1
Chromosome1-1863378	INS	PRECISE	4064	0	0	0/0	0/0	0/0	0/0	0/0	0/0	0/0	0/0	0/0	0/0	0/0	0/0	0/0	0/1
Chromosome1-1863878	INS	PRECISE	114	0	0.5	0/0	0/0	0/0	0/0	0/0	0/0	0/0	0/0	0/0	0/0	0/0	0/1	0/0	0/0
Chromosome1-1864007	DEL	PRECISE	68	0	0	0/0	0/0	0/0	0/1	0/1	0/1	1/1	0/1	1/1	0/0	0/1	0/1	0/1	0/1
Chromosome1-1864238	INS	PRECISE	343	0	0	0/0	0/0	0/0	0/0	0/0	0/0	0/0	0/0	0/0	0/0	0/0	0/0	0/0	0/1
Chromosome1-1864810	INS	PRECISE	53	0	0.408	0/0	0/0	0/0	0/0	0/0	0/0	0/0	0/1	0/1	0/0	0/1	0/1	0/1	0/0
Chromosome1-1865024	INS	PRECISE	53	0	0.707	0/0	0/0	0/0	0/0	0/0	0/0	0/0	0/0	0/0	0/0	0/1	0/0	0/1	0/0
Chromosome1-1866253	DEL	PRECISE	96	0	0	0/0	0/1	0/0	0/0	0/0	0/0	0/1	0/1	0/0	0/1	0/0	0/0	0/0	0/0
Chromosome1-1866679	DEL	PRECISE	469	0	0	0/0	0/1	0/0	0/0	0/0	0/0	1/1	1/1	0/0	0/1	0/0	0/0	0/0	0/1
Chromosome1-1867640	INS	PRECISE	80	0	0.378	0/0	0/1	0/1	0/1	0/1	0/1	0/0	0/0	0/0	0/1	0/0	0/0	0/0	0/1
Chromosome1-1869319	DEL	PRECISE	1426	0	0	0/0	0/0	0/0	0/0	0/0	0/0	0/0	0/0	0/0	0/0	0/1	0/0	0/0	0/0
Chromosome1-1869517	DEL	PRECISE	527	0	0	0/0	0/0	0/0	0/0	0/0	0/0	0/0	0/0	0/0	0/0	0/1	0/0	0/1	0/0
Chromosome1-1870489	DEL	PRECISE	57	0	0	0/0	0/0	0/0	0/1	0/1	0/1	0/0	0/0	1/1	0/0	0/0	0/0	0/0	0/0
Chromosome1-1870545	INS	PRECISE	11952	0	0	0/0	0/0	0/0	0/0	0/0	0/0	0/0	0/0	0/1	0/0	0/0	0/0	0/0	0/0
Chromosome1-1870545	BND	PRECISE	0	0	0	0/0	0/0	0/0	0/0	0/0	0/0	0/0	0/0	0/1	0/0	0/0	0/0	0/0	0/0
Chromosome1-1870790	INS	PRECISE	71	0	0.527	0/0	0/1	0/1	1/1	1/1	1/1	0/1	0/1	1/1	0/1	0/0	0/0	0/0	0/1
Chromosome1-1870959	BND	PRECISE	0	0	0	0/0	0/0	0/0	0/0	0/1	0/0	0/0	0/0	0/1	0/0	0/0	0/0	0/0	0/0
Chromosome1-1870960	BND	PRECISE	0	0	0	0/0	0/0	0/0	0/0	0/1	0/1	0/0	0/0	1/1	0/0	0/0	0/0	0/0	0/0
Chromosome1-1871469	DEL	PRECISE	64	0	0	0/0	0/0	0/0	0/0	0/0	0/0	0/0	0/0	0/0	0/0	0/1	0/0	0/1	0/0
Chromosome1-1872832	INS	PRECISE	722	0	0	0/0	0/0	0/0	0/1	0/0	0/0	0/0	0/0	0/0	0/0	0/0	0/0	0/0	0/0
Chromosome1-1872935	INS	PRECISE	5868	0	0	0/0	0/0	0/0	0/0	0/0	0/0	0/0	0/0	0/0	0/0	0/0	0/0	0/0	1/1
Chromosome1-1873297	INS	PRECISE	6043	0	0	0/0	0/0	0/0	0/0	0/0	0/0	0/0	0/0	0/0	0/0	0/0	0/0	0/0	0/1
Chromosome1-1873348	INS	PRECISE	407	0	0	0/0	0/0	0/0	0/0	0/0	0/0	0/0	0/0	0/0	0/0	0/0	0/1	0/0	0/0
Chromosome1-1873682	INS	PRECISE	5091	0	0	0/0	0/0	0/0	0/0	0/0	0/0	0/0	0/0	0/1	0/0	0/0	0/0	0/0	0/0
Chromosome1-1873863	DEL	PRECISE	124	0	0	0/0	0/0	0/0	0/0	0/1	0/0	0/0	0/0	0/1	0/0	0/0	0/0	0/0	0/0
Chromosome1-1874189	DEL	PRECISE	3886	0	0	0/0	0/1	0/1	1/1	1/1	1/1	0/0	0/0	0/0	0/1	0/0	0/0	0/0	1/1
Chromosome1-1876130	INV	PRECISE	6085391	0	0	0/0	0/0	0/0	0/1	0/1	0/1	0/0	0/0	0/0	0/0	0/0	0/0	0/0	0/0
Chromosome1-1877206	BND	PRECISE	0	0	0	0/0	0/0	0/0	0/0	0/0	0/0	0/0	0/0	0/0	0/0	0/0	0/0	0/0	0/1
Chromosome1-1877206	INV	PRECISE	6074632	0	0	0/1	0/1	0/0	0/1	0/0	0/1	0/1	0/1	0/0	0/1	0/1	0/1	0/1	0/0
Chromosome1-1877284	INS	PRECISE	575	0	0.441	0/0	0/1	0/1	1/1	1/1	1/1	0/1	0/1	0/0	0/1	0/0	0/0	0/0	1/1
Chromosome1-1877301	DEL	PRECISE	50	0	0	0/0	0/0	0/0	0/0	0/0	0/0	0/1	0/1	0/0	0/0	0/0	0/0	0/0	0/0
Chromosome1-1879419	BND	PRECISE	0	0	0	0/0	0/1	0/1	0/0	0/1	0/1	0/0	0/0	0/0	0/1	0/0	0/0	0/0	0/1
Chromosome1-1879638	DEL	PRECISE	72	0	0	0/0	0/0	0/0	0/0	0/0	0/0	0/0	0/0	0/1	0/0	0/0	0/1	0/0	0/0
Chromosome1-1880000	DEL	PRECISE	302	0	0	0/0	0/0	0/0	0/0	0/1	0/1	0/0	0/0	0/1	0/0	0/0	0/0	0/0	0/0
Chromosome1-1880024	INS	PRECISE	690	0	0	0/0	0/0	0/0	0/0	0/0	0/0	0/0	0/0	0/0	0/0	0/0	0/0	0/0	0/1
Chromosome1-1880058	INS	PRECISE	1379	0	0	0/0	0/0	0/0	0/0	0/0	0/0	0/0	0/0	0/0	0/0	0/0	0/0	0/0	0/1
Chromosome1-1880550	INS	PRECISE	5724	0	0	0/0	0/0	0/0	0/0	0/0	0/0	0/0	0/0	0/1	0/0	0/0	0/0	0/0	0/0
Chromosome1-1880572	INS	PRECISE	2356	0	0	0/0	0/0	0/1	0/1	0/0	0/0	0/0	0/0	0/0	0/0	0/0	0/0	0/0	0/1
Chromosome1-1880821	INS	PRECISE	5764	0	0	0/0	0/0	0/0	0/0	0/0	0/0	0/0	0/0	0/0	0/0	0/0	0/1	0/0	0/0
Chromosome1-1880904	DEL	PRECISE	3371	0	0	0/0	0/0	0/0	0/0	0/0	0/0	0/0	0/0	0/0	0/0	0/0	0/1	0/0	0/0

Chromosome1-1881533	INS	PRECISE	12859	0	0	0/0	0/0	0/0	0/0	0/0	0/1	0/0	0/0	0/0	0/0	0/0	0/0	0/0	0/0
Chromosome1-1881534	BND	PRECISE	0	0	0	0/0	0/0	0/0	0/0	0/0	0/1	0/0	0/0	0/0	0/0	0/0	0/0	0/0	0/0
Chromosome1-1882074	DEL	PRECISE	992	0	0	0/0	0/0	0/0	0/0	0/1	0/1	0/0	0/0	0/1	0/0	0/0	0/0	0/0	0/0
Chromosome1-1882083	INS	PRECISE	11167	0	0	0/0	0/0	0/0	0/0	0/0	0/0	0/0	0/0	0/0	0/0	0/0	0/1	0/0	0/0
Chromosome1-1883731	INS	PRECISE	662	0	0	0/0	0/0	0/0	0/0	0/0	0/0	0/0	0/0	0/1	0/0	0/0	0/0	0/0	0/0
Chromosome1-1883837	INS	PRECISE	95	0	0	0/0	0/0	0/0	0/0	0/0	0/0	0/0	0/0	0/0	0/0	0/1	0/1	0/1	0/0
Chromosome1-1885391	INS	PRECISE	379	0	0	0/0	0/0	0/0	0/1	0/0	0/0	0/0	0/0	0/0	0/0	0/0	0/0	0/0	0/0
Chromosome1-1885461	INS	PRECISE	4840	0	0	0/0	0/0	0/0	0/0	0/0	0/0	0/0	0/0	0/0	0/0	0/0	0/0	0/0	0/1
Chromosome1-1885822	INS	PRECISE	838	0	0	0/0	0/0	0/0	0/0	0/0	0/0	0/0	0/0	0/1	0/0	0/0	0/0	0/0	0/0
Chromosome1-1887073	INV	PRECISE	647	0	0	0/0	0/0	0/0	0/0	0/1	0/1	0/0	0/0	0/1	0/0	0/0	0/0	0/0	0/0
Chromosome1-1887489	INS	PRECISE	13506	0	0	0/0	0/0	0/0	0/0	0/0	0/0	0/0	0/0	0/0	0/0	0/0	0/0	0/0	0/0
Chromosome1-1887489	BND	PRECISE	0	0	0	0/0	0/0	0/0	0/0	0/0	0/1	0/0	0/0	0/0	0/0	0/0	0/0	0/0	0/0
Chromosome1-1887498	INS	PRECISE	51	0	0	0/0	0/0	0/0	0/0	0/0	0/0	0/0	0/0	1/1	0/0	0/1	0/1	0/1	0/0
Chromosome1-1888462	DEL	PRECISE	493	0	0	0/0	0/0	0/0	0/0	0/0	0/0	0/0	0/0	0/1	0/0	0/1	0/1	0/1	0/0
Chromosome1-1888591	INS	PRECISE	133	0	0	0/0	0/0	0/0	0/0	0/0	0/0	0/0	0/0	0/0	0/0	0/0	0/0	0/0	0/1
Chromosome1-1889161	DEL	PRECISE	66	0	0	0/0	0/0	0/0	0/0	0/0	0/0	0/0	0/0	0/1	0/0	0/0	0/0	0/0	0/0
Chromosome1-1889226	INS	PRECISE	201	0	0	0/0	0/0	0/0	0/0	0/0	0/0	0/0	0/0	0/1	0/0	0/0	0/0	0/0	0/0
Chromosome1-1890363	INS	PRECISE	216	0	0.577	0/0	0/0	0/0	0/0	0/0	0/0	0/0	0/0	0/1	0/0	0/1	0/1	0/0	0/0
Chromosome1-1890537	DEL	PRECISE	608	0	0	0/0	0/0	0/0	0/0	0/0	0/0	0/0	0/0	0/0	0/0	0/1	0/1	0/0	0/0
Chromosome1-1890796	DEL	PRECISE	827	0	0	0/0	0/0	0/0	0/0	0/0	0/0	0/0	0/0	0/1	0/0	0/1	0/1	0/0	0/1
Chromosome1-1891394	INS	PRECISE	3824	0	0	0/0	0/0	0/0	0/0	0/0	0/0	0/0	0/0	1/1	0/0	0/0	0/0	0/0	0/0
Chromosome1-1893901	INS	PRECISE	5764	0	0	0/0	0/0	0/0	0/0	0/0	0/0	0/0	0/0	1/1	0/0	0/0	0/0	0/0	0/0
Chromosome1-1894301	DEL	PRECISE	291	0	0	0/0	0/0	0/0	0/1	0/1	0/1	0/0	0/0	1/1	0/0	0/0	0/0	0/0	0/0
Chromosome1-1894394	DEL	PRECISE	2387	0	0	0/0	0/0	0/0	0/0	0/0	0/0	0/0	0/0	0/0	0/0	0/0	0/1	0/0	0/0
Chromosome1-1894638	DEL	PRECISE	580	0	0	0/0	0/0	0/0	0/0	0/0	0/0	0/0	0/0	0/0	0/0	0/0	0/1	0/0	0/0
Chromosome1-1894788	DEL	PRECISE	154	0	0	0/0	0/0	0/0	0/0	0/0	0/0	0/0	0/0	0/1	0/0	0/0	0/0	0/0	0/0
Chromosome1-1894821	INS	PRECISE	241	0	0	0/0	0/0	0/0	0/0	0/0	0/0	0/0	0/0	0/1	0/0	0/0	0/0	0/0	0/0
Chromosome1-1894921	DEL	PRECISE	10382	0	0	0/0	0/0	0/0	0/0	0/0	0/0	0/0	0/0	0/0	0/0	1/1	1/1	0/1	0/0
Chromosome1-1895218	INS	PRECISE	1166	0	0.782	0/0	0/1	0/1	0/1	0/1	0/1	0/1	0/1	0/0	0/1	0/0	0/0	0/0	0/1
Chromosome1-1896282	DEL	PRECISE	24196	0	0	0/0	0/1	1/1	1/1	0/1	1/1	1/1	1/1	0/0	1/1	0/0	0/0	0/0	0/1
Chromosome1-1898761	DEL	PRECISE	333	0	0	0/0	0/0	0/0	0/0	0/0	0/0	0/0	0/0	0/1	0/0	0/0	0/0	0/0	0/1
Chromosome1-1898911	DEL	PRECISE	5536	0	0	0/0	0/0	0/0	0/0	0/0	0/0	0/0	0/0	0/0	0/0	0/0	0/0	0/0	0/1
Chromosome1-1899714	BND	PRECISE	0	0	0	0/0	0/0	0/0	0/0	0/0	0/0	0/0	0/0	0/1	0/0	0/0	0/0	0/0	0/0
Chromosome1-1900106	INS	PRECISE	1344	0	0	0/0	0/0	0/0	0/0	0/0	0/0	0/0	0/0	0/1	0/0	0/0	0/0	0/0	0/0
Chromosome1-1900323	INS	PRECISE	62	0	0	0/0	0/1	0/1	1/1	1/1	1/1	0/1	0/1	1/1	0/1	0/1	0/1	0/0	1/1
Chromosome1-1901433	INS	PRECISE	832	0	0	0/0	0/0	0/0	0/0	0/0	0/0	0/0	0/0	0/1	0/0	0/0	0/0	0/0	0/0
Chromosome1-1901672	INS	PRECISE	242	0	0.577	0/0	0/0	0/0	0/0	0/0	0/0	0/0	0/0	0/0	0/0	0/1	0/1	0/1	0/0
Chromosome1-1902016	DEL	PRECISE	78	0	0	0/0	0/0	0/0	0/0	0/0	0/0	0/0	0/0	0/0	0/0	0/1	0/1	0/1	0/0
Chromosome1-1902973	INS	PRECISE	597	0	0	0/0	0/0	0/0	0/0	0/0	0/0	0/0	0/0	0/1	0/0	0/0	0/0	0/0	0/0
Chromosome1-1903386	INS	PRECISE	13688	0	0	0/0	0/0	0/0	0/0	0/0	0/0	0/0	0/0	0/0	0/0	0/0	0/0	0/0	0/0
Chromosome1-1903408	INS	PRECISE	11822	0	0	0/0	0/0	0/0	0/0	0/0	0/1	0/0	0/0	0/0	0/0	0/0	0/0	0/0	0/0
Chromosome1-1903423	INS	PRECISE	15378	0	0	0/0	0/0	0/0	0/0	0/0	0/0	0/0	0/0	0/1	0/0	0/0	0/0	0/0	0/0
Chromosome1-1903605	INS	PRECISE	13114	0	0	0/0	0/0	0/0	0/0	0/0	0/0	0/0	0/0	0/1	0/0	0/0	0/0	0/0	0/0
Chromosome1-1903749	DEL	PRECISE	633	0	0	0/0	0/1	0/1	1/1	1/1	1/1	0/1	0/1	1/1	0/1	0/0	0/0	0/0	1/1
Chromosome1-1904746	INS	PRECISE	120	0	0	0/0	0/0	0/0	0/0	0/0	0/0	0/0	0/0	0/1	0/0	0/0	0/0	0/0	0/0
Chromosome1-1904788	BND	PRECISE	0	0	0	0/0	0/0	0/0	0/0	0/0	0/0	0/0	0/0	0/0	0/0	0/1	0/1	0/1	0/0
Chromosome1-1904936	DEL	PRECISE	176	0	0	0/0	0/0	0/0	0/0	0/0	0/0	0/0	0/0	0/1	0/0	0/1	0/1	0/1	0/0
Chromosome1-1905336	INS	PRECISE	4017	0	0	0/0	0/0	0/0	0/0	0/0	0/0	0/0	0/0	1/1	0/0	0/0	0/0	0/0	0/0
Chromosome1-1905701	DEL	PRECISE	206	0	0	0/0	0/0	0/0	0/1	0/1	0/1	0/1	0/1	0/1	0/0	0/0	0/0	0/0	0/0
Chromosome1-1905825	BND	PRECISE	0	0	0	0/0	0/0	0/0	0/0	0/0	0/1	0/0	0/0	0/0	0/0	0/0	0/0	0/0	0/1
Chromosome1-1905825	BND	PRECISE	0	0	0	0/0	0/1	0/1	0/1	0/1	0/0	0/0	0/0	0/0	0/0	0/0	0/0	0/0	0/0
Chromosome1-1905825	BND	PRECISE	0	0	0	0/0	0/0	0/0	0/1	0/1	0/0	0/0	0/0	0/0	0/1	0/0	0/0	0/0	0/1
Chromosome1-1905912	DEL	PRECISE	211	0	0	0/0	0/0	0/0	0/0	0/0	0/0	0/0	0/0	0/1	0/0	0/1	0/1	0/1	0/0
Chromosome1-1906140	DEL	PRECISE	74	0	0	0/0	0/0	0/0	0/0	0/0	0/0	0/0	0/0	0/1	0/0	0/1	0/1	0/1	0/0
Chromosome1-1906244	INS	PRECISE	461	0	0.957	0/0	0/0	0/0	0/0	0/0	0/0	0/0	0/0	0/1	0/0	1/1	0/1	1/1	0/0
Chromosome1-1906326	DEL	PRECISE	454	0	0	0/0	0/0	0/0	0/0	0/0	0/0	0/0	0/0	0/0	0/0	0/0	0/1	0/1	0/0
Chromosome1-1906587	INS	PRECISE	575	0	0.577	0/0	0/0	0/0	0/0	0/0	0/0	0/0	0/0	0/1	0/0	0/0	0/1	0/1	0/0
Chromosome1-1906951	INS	PRECISE	179	0	0.577	0/0	0/0	0/0	0/0	0/0	0/0	0/0	0/0	0/1	0/0	0/0	0/1	0/1	0/0
Chromosome1-1908163	INS	PRECISE	57	0	0.577	0/0	0/0	0/0	0/0	0/0	0/0	0/0	0/0	0/1	0/0	0/1	0/1	0/1	0/0
Chromosome1-1908659	DEL	PRECISE	1552	0.376	0	0/0	0/1	0/1	1/1	1/1	1/1	0/1	0/1	1/1	0/1	0/1	0/1	0/1	1/1
Chromosome1-1909148	INS	PRECISE	152	0	0.577	0/0	0/0	0/0	0/0	0/0	0/0	0/0	0/0	0/1	0/0	0/1	0/1	0/1	0/0
Chromosome1-1909447	DEL	PRECISE	93	0	0	0/0	0/0	0/0	1/1	0/0	0/0	0/0	0/0	0/0	0/0	0/0	0/0	0/0	0/0
Chromosome1-1909731	INS	PRECISE	4576	0	0	0/0	0/0	0/0	1/1	0/0	0/0	0/0	0/0	0/0	0/0	0/0	0/0	0/0	0/0
Chromosome1-1910167	DEL	PRECISE	276	0	0	0/0	0/0	0/0	1/1	0/0	0/0	0/0	0/0	0/0	0/0	0/0	0/0	0/0	0/0
Chromosome1-1910379	DEL	PRECISE	658	0.707	0.707	0/0	0/0	0/0	1/1	0/0	0/0	0/0	0/0	0/0	0/0	0/0	0/0	0/0	1/1
Chromosome1-1910544	DEL	PRECISE	73	0	0	0/0	0/0	0/0	0/0	0/0	0/0	0/0	0/0	0/1	0/0	0/1	0/1	0/0	0/0
Chromosome1-1911328	DUP	PRECISE	6342	0	0	0/0	0/0	0/0	0/0	0/0	0/0	0/0	0/0	0/0	0/0	0/0	0/0	0/1	0/0
Chromosome1-1911328	BND	PRECISE	0	0	0	0/0	0/0	0/0	0/0	0/0	0/0	0/0	0/0	0/1	0/0	0/0	0/0	0/0	0/0
Chromosome1-1911950	BND	PRECISE	0	0	0	0/0	0/0	0/0	0/0	0/0	0/0	0/0	0/0	0/1	0/0	0/0	0/0	0/0	0/0
Chromosome1-1912019	INS	PRECISE	54	0	0	0/0	0/0	0/0	0/1	0/0	0/0	0/0	0/0	0/0	0/0	0/0	0/0	0/0	0/0
Chromosome1-1912967	INS	PRECISE	1735	0	0.577	0/0	0/0	0/0	0/0	0/0	0/0	0/0	0/0	0/0	0/0	0/1	0/1	0/1	0/0
Chromosome1-1913424	DEL	PRECISE	66	0	0	0/0	0/0	0/0	0/0	0/0	0/0	0/0	0/0	0/1	0/0	0/1	0/1	0/1	0/0
Chromosome1-1913589	DEL	PRECISE	1589	0	0	0/0	0/0	0/0	0/0	0/0	0/0	0/0	0/0	0/1	0/0	0/1	0/1	0/1	0/0

Chromosome1-1913645	DEL	PRECISE	396	0	0	0/0	0/0	0/0	0/0	0/0	0/0	0/0	0/0	0/0	0/0	0/0	0/0	0/0	0/1
Chromosome1-1914233	DEL	PRECISE	159	0	0	0/0	0/0	0/0	0/0	0/0	0/0	0/0	0/0	0/0	0/0	0/1	0/1	0/1	0/0
Chromosome1-1914235	INS	PRECISE	249	0	0	0/0	0/0	0/0	0/0	0/0	0/0	0/0	0/0	0/1	0/0	0/0	0/0	0/0	0/0
Chromosome1-1915508	INS	PRECISE	7167	0	0	0/0	0/0	0/0	0/0	0/0	0/0	0/0	0/0	0/0	0/0	0/0	0/0	0/0	1/1
Chromosome1-1916474	INS	PRECISE	4865	0	0	0/0	0/0	0/0	0/0	0/0	0/0	0/0	0/0	0/0	0/0	0/0	0/0	0/0	1/1
Chromosome1-1917154	DEL	PRECISE	51	0	0	0/0	0/0	0/0	0/0	0/0	0/0	0/0	0/0	0/1	0/0	0/1	0/1	1/1	0/0
Chromosome1-1917874	DEL	PRECISE	63	0	0	0/0	0/0	0/0	0/0	0/0	0/0	0/1	0/1	0/0	0/0	0/0	0/0	0/0	0/0
Chromosome1-1919089	DEL	PRECISE	1274	0	0	0/0	0/0	0/0	0/0	0/0	0/0	0/0	0/0	0/1	0/0	0/1	0/1	0/1	0/0
Chromosome1-1920286	DEL	PRECISE	368	0	0	0/0	0/0	0/0	0/1	0/0	0/0	0/0	0/1	0/0	0/0	0/0	0/0	0/0	0/0
Chromosome1-1920560	INS	PRECISE	998	0	0	0/0	0/0	0/0	0/0	0/0	0/0	0/0	0/0	0/1	0/0	0/0	0/0	0/0	0/0
Chromosome1-1920790	DEL	PRECISE	89	0	0	0/0	0/1	0/1	1/1	0/1	0/1	0/0	0/0	0/0	0/1	0/0	0/0	0/0	0/1
Chromosome1-1921229	DEL	PRECISE	5304	0	0	0/0	0/0	0/0	0/1	1/1	0/0	0/0	0/0	1/1	0/0	0/0	0/0	0/0	0/0
Chromosome1-1921230	DEL	PRECISE	6521	0	0	0/0	0/1	0/1	1/1	1/1	1/1	0/1	0/1	1/1	1/1	0/0	0/0	0/0	1/1
Chromosome1-1921779	DEL	PRECISE	1029	0	0	0/0	0/0	0/0	1/1	1/1	1/1	0/1	0/0	1/1	0/0	0/0	0/0	0/0	1/1
Chromosome1-1921911	INS	PRECISE	59	0	0.422	0/0	0/1	0/1	1/1	1/1	1/1	0/1	0/1	1/1	0/1	0/0	0/0	0/0	1/1
Chromosome1-1922333	DEL	PRECISE	87	0	0	0/0	0/0	0/0	0/1	0/0	0/0	0/0	0/0	0/0	0/0	0/0	0/0	0/0	0/0
Chromosome1-1922352	INS	PRECISE	1188	0	0	0/0	0/0	0/0	0/0	0/0	0/0	0/0	0/0	0/0	0/0	0/0	0/0	0/0	0/1
Chromosome1-1922620	DEL	PRECISE	420	0	0	0/0	0/0	0/0	0/1	0/0	0/0	0/0	0/0	0/0	0/0	0/0	0/0	0/0	0/0
Chromosome1-1922660	DEL	PRECISE	5401	0	0	0/0	0/0	0/0	0/0	0/0	0/0	0/0	0/0	0/1	0/0	0/1	0/0	0/0	0/0
Chromosome1-1923075	INS	PRECISE	154	0	0.316	0/0	0/1	0/1	0/1	1/1	1/1	0/1	0/1	1/1	0/1	0/0	0/0	0/0	0/1
Chromosome1-1923365	DEL	PRECISE	382	0	0	0/0	0/0	0/0	0/1	0/0	0/0	0/0	0/0	0/0	0/0	0/0	0/0	0/0	0/0
Chromosome1-1923584	DEL	PRECISE	567	0	0	0/0	0/0	0/0	0/0	0/0	0/0	0/0	0/0	0/0	0/0	0/0	0/0	0/0	0/1
Chromosome1-1923646	INS	PRECISE	5637	0	0	0/0	0/0	0/0	0/0	0/0	0/0	0/0	0/0	1/1	0/0	0/0	0/0	0/0	0/0
Chromosome1-1923662	INS	PRECISE	82	0	0	0/0	0/0	0/0	0/0	0/0	0/0	0/0	0/0	0/0	0/0	0/0	0/0	0/0	0/1
Chromosome1-1923805	DEL	PRECISE	56	0	0	0/0	0/0	0/0	0/0	0/0	0/0	0/1	0/1	0/0	0/0	0/0	0/0	0/0	0/0
Chromosome1-1923984	INS	PRECISE	5528	0	0	0/0	0/0	0/0	0/0	0/0	0/0	0/0	0/0	0/1	0/0	0/0	0/0	0/0	0/0
Chromosome1-1924055	DEL	PRECISE	64	0	0	0/0	0/0	0/0	0/0	0/0	0/0	0/0	0/0	0/0	0/0	0/0	0/0	0/0	0/1
Chromosome1-1924335	DEL	PRECISE	488	0	0	0/0	0/0	0/0	0/0	0/0	0/0	0/0	0/0	0/0	0/0	0/0	0/0	0/0	0/1
Chromosome1-1924565	DEL	PRECISE	73	0	0	0/0	0/0	0/0	0/0	0/0	0/0	0/0	0/0	0/0	0/0	0/0	0/1	0/0	0/0
Chromosome1-1924858	BND	PRECISE	0	0	0	0/0	0/0	0/0	0/0	0/0	0/0	0/0	0/0	0/0	0/0	0/0	0/1	0/1	0/0
Chromosome1-1925331	INS	PRECISE	9073	0	0	0/0	0/0	0/0	0/0	0/0	0/0	0/0	0/0	0/0	0/0	0/0	0/0	0/0	0/1
Chromosome1-1925390	INS	PRECISE	616	0	0.447	0/0	0/0	0/0	0/0	0/1	0/1	0/1	0/1	0/1	0/0	0/0	0/0	0/0	0/0
Chromosome1-1926127	INS	PRECISE	590	0	0.535	0/0	0/1	0/1	0/1	0/1	0/1	0/0	0/0	0/0	0/1	0/0	0/0	0/0	0/1
Chromosome1-1926751	DEL	PRECISE	662	0	0	0/0	0/0	0/0	0/0	0/0	0/0	0/0	0/0	0/1	0/0	0/0	0/0	0/0	0/0
Chromosome1-1926839	INS	PRECISE	159	0	0	0/0	0/0	0/0	0/0	0/0	0/0	0/0	0/0	0/0	0/0	0/0	0/0	0/0	0/1
Chromosome1-1927873	DEL	PRECISE	20546	0	0	0/0	0/0	0/0	0/0	0/0	0/0	0/0	0/0	0/1	0/0	0/0	0/0	0/0	0/0
Chromosome1-1931549	BND	PRECISE	0	0	0	0/0	0/0	0/0	0/0	0/0	0/0	0/0	0/0	0/0	0/0	0/1	0/0	0/1	0/0
Chromosome1-1931772	DEL	PRECISE	1017	0	0	0/0	0/0	0/0	0/0	0/0	0/0	0/0	0/0	0/0	0/0	0/0	0/0	0/0	0/1
Chromosome1-1931888	DEL	PRECISE	1139	0	0	0/0	0/0	0/0	0/1	0/1	0/1	0/0	0/0	1/1	0/0	0/0	0/0	0/0	0/1
Chromosome1-1931889	DEL	PRECISE	2922	0	0	0/0	0/0	0/0	0/0	0/0	0/0	1/1	0/0	0/0	0/0	0/0	0/0	0/0	0/0
Chromosome1-1932022	DEL	PRECISE	1587	0	0	0/0	0/0	0/0	0/1	0/1	0/1	0/0	0/0	1/1	0/0	0/0	0/0	0/0	0/1
Chromosome1-1932270	INS	PRECISE	5744	0	0	0/0	0/0	0/0	0/0	0/0	0/0	0/0	0/0	0/0	0/0	0/0	0/0	0/0	1/1
Chromosome1-1932548	DEL	PRECISE	4587	0	0	0/0	0/0	0/0	0/0	0/0	0/0	0/1	0/0	0/0	0/0	0/0	0/0	0/0	0/0
Chromosome1-1932589	INS	PRECISE	3553	0	0	0/0	0/0	0/0	0/0	0/0	0/0	1/1	0/0	0/0	0/0	0/0	0/0	0/0	0/0
Chromosome1-1932636	INS	PRECISE	123	0	0	0/0	0/0	0/0	0/1	0/1	0/1	0/0	0/0	0/1	0/0	0/0	0/0	0/0	0/0
Chromosome1-1932780	INS	PRECISE	77	0	0.855	0/1	0/1	0/0	0/1	0/1	0/1	1/1	1/1	1/1	0/1	1/1	1/1	1/1	0/1
Chromosome1-1932889	INS	PRECISE	3190	0	0	0/0	0/0	0/0	0/0	0/0	0/0	0/0	0/0	1/1	0/0	0/0	0/0	0/0	0/0
Chromosome1-1933234	DEL	PRECISE	2937	0	0	0/0	0/0	0/0	0/0	0/0	0/0	0/0	0/0	0/1	0/0	0/0	0/0	0/0	0/0
Chromosome1-1933308	DEL	PRECISE	156	0	0	0/0	0/0	0/0	0/0	0/0	0/0	0/1	0/0	0/0	0/0	0/0	0/0	0/0	0/0
Chromosome1-1933321	BND	PRECISE	0	0	0	0/0	0/0	0/0	0/0	0/0	0/0	0/0	0/0	0/0	0/0	0/1	0/1	0/0	0/0
Chromosome1-1934720	DEL	PRECISE	77	0	0	0/0	0/0	0/0	0/0	0/0	0/0	0/0	0/0	0/0	0/0	0/0	0/0	0/0	0/1
Chromosome1-1935117	INS	PRECISE	698	0	0	0/0	0/0	0/0	0/0	0/0	0/0	0/0	0/0	0/0	0/0	0/0	0/0	0/0	0/1
Chromosome1-1935312	DEL	PRECISE	1278	0.447	0.447	0/0	0/0	0/0	0/0	0/0	0/0	0/1	0/0	0/1	0/0	0/1	0/0	0/1	0/1
Chromosome1-1935802	DEL	PRECISE	190	0	0	0/0	0/0	0/0	0/0	0/0	0/0	0/0	0/0	0/1	0/0	0/0	0/0	0/0	0/0
Chromosome1-1935887	INS	PRECISE	238	0	0	0/0	0/0	0/0	0/0	0/0	0/0	0/0	0/0	0/1	0/0	0/0	0/0	0/0	0/0
Chromosome1-1936269	INS	PRECISE	910	0	0	0/0	0/0	0/0	0/0	0/0	0/0	0/1	0/0	0/0	0/0	0/0	0/0	0/0	0/0
Chromosome1-1936945	INS	PRECISE	427	0	0	0/0	0/0	0/0	0/0	0/0	0/0	0/0	0/0	0/0	0/0	0/0	0/0	0/0	0/1
Chromosome1-1937504	DEL	PRECISE	60	0	0	0/0	0/0	0/0	0/0	0/0	0/0	0/0	0/0	0/0	0/0	0/0	0/1	0/0	0/0
Chromosome1-1938727	DEL	PRECISE	66	0	0	0/0	0/0	0/0	0/1	0/0	0/0	0/1	0/0	0/0	0/0	0/0	0/0	0/0	0/0
Chromosome1-1938876	DEL	PRECISE	52	0	0	0/0	1/1	0/1	0/1	1/1	1/1	0/0	0/0	0/0	1/1	0/0	0/0	0/0	0/1
Chromosome1-1940075	INS	PRECISE	281	0	0	0/0	0/0	0/0	0/0	0/0	0/0	0/0	0/0	0/0	0/0	0/0	0/0	0/0	0/1
Chromosome1-1940355	DEL	PRECISE	62	0	0	0/0	0/0	0/0	0/0	0/0	0/0	0/0	0/0	0/1	0/0	0/0	0/0	0/0	0/1
Chromosome1-1940457	DEL	PRECISE	60	0	0	0/1	0/1	0/0	0/1	0/1	0/1	0/1	1/1	1/1	0/1	1/1	1/1	1/1	0/1
Chromosome1-1940781	BND	PRECISE	0	0	0	0/0	0/0	0/0	0/0	0/0	0/0	0/1	0/0	0/0	0/1	0/0	0/1	0/0	0/0
Chromosome1-1941327	INS	PRECISE	150	0	0	0/0	0/0	0/0	0/0	0/0	0/0	0/0	0/0	0/1	0/0	0/0	0/0	0/0	0/0
Chromosome1-1942385	INS	PRECISE	2857	0	0	0/0	0/0	0/1	0/0	0/0	0/0	0/0	0/0	0/0	0/0	0/0	0/0	0/0	0/0
Chromosome1-1942761	INS	PRECISE	158	0	0.579	0/1	1/1	0/1	1/1	1/1	1/1	1/1	1/1	1/1	1/1	1/1	1/1	1/1	1/1
Chromosome1-1942838	DEL	PRECISE	73	0	0	0/0	0/0	0/0	0/0	0/0	0/0	0/0	0/0	0/1	0/0	0/1	0/1	0/1	0/0
Chromosome1-1943292	INS	PRECISE	50	0	0	0/0	0/0	0/0	0/0	0/0	0/0	0/0	0/0	0/1	0/0	0/1	0/1	0/1	0/0
Chromosome1-1943900	DEL	PRECISE	10563	0.877	0.877	0/1	0/1	0/0	0/1	0/1	0/0	1/1	1/1	1/1	0/1	1/1	1/1	1/1	0/1
Chromosome1-1944285	DEL	PRECISE	69	0	0	0/0	0/1	0/1	0/1	0/1	1/1	0/0	0/0	0/0	0/1	0/0	0/0	0/0	1/1
Chromosome1-1944973	DEL	PRECISE	74	0	0	0/0	0/0	0/0	0/0	0/0	0/0	0/0	0/0	0/0	0/0	0/1	0/1	0/1	0/0
Chromosome1-1945222	DEL	PRECISE	851	0	0	0/0	0/0	0/0	0/0	0/0	0/0	0/0	0/0	0/0	0/0	0/0	0/0	0/0	0/1

Chromosome1-1945432	BND	PRECISE	0	0	0	0/0	0/0	0/0	0/0	0/0	0/0	0/1	0/0	0/0	0/0	0/0	0/0	0/0	0/0
Chromosome1-1945667	INS	PRECISE	2442	0	0	0/0	0/0	0/0	0/0	0/0	0/0	1/1	0/0	0/0	0/0	0/0	0/0	0/0	0/0
Chromosome1-1945755	DEL	PRECISE	314	0	0	0/1	0/1	0/0	0/1	0/1	0/1	1/1	1/1	1/1	0/1	0/1	0/1	0/1	0/0
Chromosome1-1945879	DEL	PRECISE	1043	0	0	0/0	0/0	0/0	0/0	0/0	0/0	0/0	0/0	0/0	0/0	0/1	0/1	0/1	0/0
Chromosome1-1946261	BND	PRECISE	0	0	0	0/1	0/1	0/0	0/0	1/1	0/1	1/1	1/1	1/1	0/1	0/1	0/1	0/1	0/0
Chromosome1-1946339	DEL	PRECISE	10431	0	0	1/1	0/1	0/0	0/1	0/1	1/1	1/1	1/1	1/1	0/1	1/1	1/1	1/1	0/0
Chromosome1-1946993	INS	PRECISE	242	0	0	0/0	0/0	0/0	0/0	0/0	0/0	0/0	0/0	0/0	0/0	0/0	0/0	0/0	0/1
Chromosome1-1947634	DEL	PRECISE	1348	0	0	0/0	0/0	0/0	0/0	0/0	0/0	0/0	0/0	0/1	0/0	0/0	0/0	0/0	0/0
Chromosome1-1948055	DEL	PRECISE	65	0	0	0/0	0/0	0/0	0/0	0/0	0/0	0/0	0/0	0/1	0/0	0/0	0/0	0/0	0/0
Chromosome1-1948681	INS	PRECISE	307	0	0.5	0/0	0/0	0/0	0/0	0/0	0/0	0/0	0/0	0/1	0/0	0/1	0/1	0/1	0/0
Chromosome1-1949588	DEL	PRECISE	1244	0	0	0/0	0/0	0/0	0/0	0/0	0/0	0/0	0/0	0/0	0/0	0/1	0/1	0/0	0/0
Chromosome1-1950251	DEL	PRECISE	3494	0	0	0/0	1/1	0/0	0/0	0/1	0/0	0/0	1/1	1/1	0/1	0/1	0/1	0/0	0/1
Chromosome1-1950728	DEL	PRECISE	1244	0	0	0/0	0/0	0/0	0/0	0/0	0/0	0/0	0/0	0/0	0/0	0/1	0/1	0/0	0/0
Chromosome1-1951181	DEL	PRECISE	279	0	0	0/1	0/0	0/1	0/0	0/1	1/1	0/0	0/0	0/0	0/0	0/0	0/0	0/0	0/0
Chromosome1-1951391	DEL	PRECISE	3494	0	0	0/0	1/1	0/0	0/0	0/1	0/0	0/0	1/1	1/1	0/1	0/1	0/1	0/0	0/1
Chromosome1-1951700	DEL	PRECISE	1113	0	0	0/1	0/0	0/1	0/0	0/1	1/1	0/0	0/0	0/0	0/0	0/0	0/0	0/0	0/0
Chromosome1-1955159	INS	PRECISE	211	0	0	0/0	0/0	0/0	0/0	0/0	0/0	0/1	0/0	0/0	0/0	0/0	0/0	0/0	0/0
Chromosome1-1955741	BND	PRECISE	0	0	0	0/0	0/0	0/0	0/0	0/0	0/0	0/1	0/0	0/0	0/0	0/0	0/0	0/0	0/0
Chromosome1-1956050	INS	PRECISE	90	0	0.577	0/0	0/0	0/0	0/0	0/0	0/0	0/0	0/0	0/1	0/0	0/1	0/1	0/1	0/0
Chromosome1-1956397	DEL	PRECISE	3330	0	0	0/0	0/0	0/0	0/0	0/0	0/0	1/1	0/0	0/0	0/0	0/0	0/0	0/0	0/0
Chromosome1-1956938	DEL	PRECISE	162	0	0	0/0	0/0	0/0	0/0	0/0	0/0	0/0	0/0	0/1	0/0	0/1	0/1	0/1	0/0
Chromosome1-1957004	INS	PRECISE	945	0	0	0/0	0/0	0/0	0/0	0/0	0/0	0/1	0/0	0/0	0/0	0/0	0/0	0/0	0/0
Chromosome1-1957110	INS	PRECISE	454	0.378	0.69	0/0	0/1	0/1	0/1	0/1	0/1	0/0	0/0	0/0	0/1	0/0	0/0	0/0	1/1
Chromosome1-1957223	DEL	PRECISE	214	0	0	0/0	0/1	0/1	0/1	0/1	0/1	0/0	0/0	0/0	0/1	0/0	0/0	0/0	0/1
Chromosome1-1958103	INS	PRECISE	415	0	0.577	0/0	0/0	0/0	0/0	0/0	0/0	0/0	0/0	0/0	0/0	0/0	0/0	0/1	0/0
Chromosome1-1958103	INS	PRECISE	1420	0	0	0/0	0/0	0/0	0/0	0/0	0/0	0/0	0/0	0/1	0/0	0/0	0/0	0/0	0/0
Chromosome1-1958268	DEL	PRECISE	745	0	0	0/0	0/1	0/1	0/1	0/1	0/0	0/0	0/0	0/0	0/0	0/0	0/0	0/0	0/1
Chromosome1-1958499	DEL	PRECISE	1373	0	0	0/0	0/0	0/0	0/0	0/0	0/0	0/0	0/0	0/1	0/0	0/0	0/0	0/0	0/0
Chromosome1-1958660	DEL	PRECISE	705	0	0	0/0	0/0	0/0	0/0	0/0	0/0	0/0	0/0	0/1	0/0	0/0	0/0	0/0	0/0
Chromosome1-1958744	DEL	PRECISE	379	0	0	0/1	1/1	0/1	1/1	1/1	1/1	0/1	1/1	1/1	1/1	1/1	1/1	1/1	1/1
Chromosome1-1959054	INS	PRECISE	1448	0	0	0/0	0/0	0/0	0/0	0/0	0/0	0/0	0/0	0/0	0/0	0/0	0/0	0/0	0/1
Chromosome1-1960231	DEL	PRECISE	237	0	0	0/1	1/1	0/1	1/1	1/1	1/1	1/1	1/1	1/1	1/1	1/1	1/1	1/1	1/1
Chromosome1-1960690	INS	PRECISE	2876	0	0	0/0	0/0	0/0	0/0	0/0	0/0	0/0	0/0	1/1	0/0	0/0	0/0	0/0	0/0
Chromosome1-1961110	DEL	PRECISE	56	0	0	0/0	0/0	0/0	0/0	0/0	0/0	0/0	0/0	0/0	0/0	0/0	0/0	0/0	0/1
Chromosome1-1961299	DEL	PRECISE	52	0	0	0/0	0/0	0/0	0/0	0/0	0/0	0/0	0/0	0/0	0/0	0/0	0/0	0/0	0/1
Chromosome1-1961796	DEL	PRECISE	122	0	0	0/0	0/0	0/0	0/0	0/0	0/0	0/0	0/0	0/0	0/0	0/0	0/0	0/0	0/1
Chromosome1-1961960	DEL	PRECISE	52	0	0	0/0	0/0	0/0	0/1	0/1	0/1	0/0	0/0	0/1	0/0	0/0	0/0	0/0	0/0
Chromosome1-1962688	INV	PRECISE	6279660	0	0	0/0	0/0	0/0	0/1	0/0	0/0	0/0	0/0	0/0	0/0	0/0	0/0	0/0	0/0
Chromosome1-1962739	INS	PRECISE	4888	0	0	0/0	0/0	0/0	0/0	0/0	0/0	0/1	0/0	0/0	0/0	0/0	0/0	0/0	0/0
Chromosome1-1966177	DEL	PRECISE	90	0	0	0/1	0/1	0/0	0/0	0/0	0/0	0/1	1/1	0/0	0/1	0/1	0/1	0/1	0/0
Chromosome1-1966588	INS	PRECISE	150	0	0	0/0	0/0	0/0	0/0	0/0	0/0	0/0	0/0	0/0	0/0	0/0	0/0	0/0	0/1
Chromosome1-1966847	DEL	PRECISE	2718	0	0	0/1	1/1	0/1	1/1	1/1	1/1	1/1	1/1	1/1	1/1	1/1	1/1	1/1	1/1
Chromosome1-1967516	INS	PRECISE	156	0	0	0/0	0/0	0/0	0/0	0/0	0/0	0/0	0/0	0/1	0/0	0/1	0/1	0/1	0/0
Chromosome1-1968314	DEL	PRECISE	51	0	0	0/1	1/1	0/1	1/1	1/1	1/1	1/1	1/1	1/1	1/1	1/1	1/1	1/1	1/1
Chromosome1-1968929	INS	PRECISE	1652	0	0	0/0	0/0	0/0	0/0	0/0	0/0	0/0	0/0	0/1	0/0	0/0	0/0	0/0	0/0
Chromosome1-1969172	DEL	PRECISE	73	0	0	0/1	0/1	0/0	0/1	0/1	0/1	1/1	1/1	0/1	0/1	0/1	0/1	0/1	0/1
Chromosome1-1970352	INS	PRECISE	8488	0	0	0/0	0/0	0/0	0/0	0/0	0/0	0/0	0/0	0/0	0/0	0/0	0/0	0/0	0/1
Chromosome1-1970352	BND	PRECISE	0	0	0	0/0	0/0	0/0	0/0	0/0	0/0	0/0	0/0	0/0	0/0	0/0	0/0	0/0	0/1
Chromosome1-1970525	INS	PRECISE	300	0	0.577	0/0	0/0	0/0	0/0	0/0	0/0	0/0	0/0	0/0	0/0	0/1	0/1	0/0	0/0
Chromosome1-1970589	INS	PRECISE	3241	0	0	0/0	0/0	0/0	0/0	0/0	0/0	0/0	0/0	0/0	0/0	1/1	1/1	0/0	0/0
Chromosome1-1970893	BND	PRECISE	0	0	0	0/0	0/1	0/1	0/0	0/1	0/0	0/0	0/0	0/0	0/0	0/0	0/0	0/0	0/0
Chromosome1-1970894	BND	PRECISE	0	0	0	0/0	0/1	0/0	0/0	0/0	0/1	0/0	0/0	0/0	0/1	0/0	0/0	0/0	0/1
Chromosome1-1970972	DEL	PRECISE	910	0	0	0/0	0/1	0/1	0/1	0/1	0/1	0/0	0/0	0/1	0/1	0/1	0/1	0/1	1/1
Chromosome1-1971979	BND	PRECISE	0	0	0	0/0	0/0	0/0	0/0	0/0	0/0	0/0	0/0	0/0	0/0	0/1	0/1	0/1	0/0
Chromosome1-1973017	INS	PRECISE	678	0	0	0/0	0/0	0/0	0/0	0/0	0/0	0/0	0/0	0/0	0/0	0/0	0/0	0/0	0/1
Chromosome1-1973676	INS	PRECISE	1333	0	0	0/0	0/0	0/0	0/0	0/0	0/0	0/0	0/0	0/0	0/0	0/0	0/0	0/0	0/1
Chromosome1-1973827	DEL	PRECISE	1471	0	0	0/0	0/0	0/0	0/0	0/0	0/0	0/0	0/0	0/1	0/0	0/1	0/1	0/1	0/0
Chromosome1-1974169	DEL	PRECISE	110	0	0	0/0	0/0	0/0	0/0	0/0	0/0	0/0	0/0	0/1	0/0	0/1	0/1	0/1	0/0
Chromosome1-1974423	INS	PRECISE	149	0	0.5	0/0	0/0	0/0	0/0	0/0	0/0	0/0	0/0	0/1	0/0	0/1	0/0	0/1	0/0
Chromosome1-1975841	INS	PRECISE	4555	0	0	0/0	0/0	0/0	0/0	0/0	0/0	0/0	0/0	0/0	0/0	0/0	0/0	0/0	1/1
Chromosome1-1976231	DEL	PRECISE	3230	0	0	0/0	0/0	0/0	0/0	0/0	0/0	0/0	0/0	0/0	0/0	0/0	0/0	0/0	0/1
Chromosome1-1976349	INS	PRECISE	11085	0	0	0/0	0/0	0/0	0/0	0/0	0/0	0/0	0/0	1/1	0/0	0/0	0/0	0/0	0/0
Chromosome1-1976383	INS	PRECISE	1304	0	0.951	0/0	0/1	0/1	0/1	0/1	0/1	0/0	0/0	0/0	1/1	0/0	0/0	0/0	0/1
Chromosome1-1977836	DEL	PRECISE	7307	0	0	1/1	0/0	0/1	0/0	0/0	0/0	0/0	0/0	1/1	0/0	0/0	1/1	0/0	0/0
Chromosome1-1979107	DEL	PRECISE	757	0	0	0/0	0/0	0/0	0/0	0/0	0/0	0/0	0/0	0/1	0/0	0/1	0/1	0/1	0/0
Chromosome1-1979381	DEL	PRECISE	113	0	0	0/0	0/0	0/0	0/0	0/0	0/0	0/0	0/0	0/1	0/0	0/1	0/1	0/1	0/0
Chromosome1-1979464	INS	PRECISE	200	0	0	0/0	0/0	0/0	0/0	0/0	0/0	0/0	0/0	0/0	0/0	0/0	0/1	0/0	0/0
Chromosome1-1979573	DEL	PRECISE	510	0	0	0/0	0/0	0/0	0/0	0/0	0/0	0/0	0/0	0/0	0/0	0/0	0/1	0/0	0/0
Chromosome1-1979670	DEL	PRECISE	51	0	0	0/0	0/0	0/0	0/0	0/0	0/0	0/0	0/0	0/1	0/0	0/1	0/1	0/1	0/1
Chromosome1-1979700	INS	PRECISE	994	0	0	0/0	0/0	0/0	0/0	0/0	0/0	0/0	0/0	0/0	0/0	0/0	0/0	0/0	0/1
Chromosome1-1980545	DEL	PRECISE	433	0	0	0/0	0/0	0/0	0/0	0/0	0/0	0/0	0/0	0/1	0/0	0/0	0/1	0/1	0/0
Chromosome1-1980589	INS	PRECISE	6034	0	0	0/0	0/0	0/0	1/1	0/0	0/0	0/0	0/0	0/0	0/0	0/0	0/0	0/0	0/0
Chromosome1-1980819	DEL	PRECISE	556	0	0	0/1	1/1	0/1	1/1	1/1	1/1	1/1	1/1	1/1	1/1	1/1	1/1	1/1	1/1

Chromosome1-1980960	DEL	PRECISE	190	0	0	0/1	0/1	0/0	0/0	0/1	0/1	1/1	1/1	1/1	0/1	1/1	1/1	1/1	0/0
Chromosome1-1981822	DEL	PRECISE	102	0	0	0/0	0/0	0/0	0/0	0/0	0/0	0/0	0/0	0/1	0/0	0/1	0/1	0/1	0/0
Chromosome1-1981900	INS	PRECISE	1086	0	0.5	0/0	0/0	0/0	0/0	0/0	0/0	0/0	0/0	0/1	0/0	0/1	0/1	0/1	0/0
Chromosome1-1981988	DEL	PRECISE	77	0	0	0/0	0/0	0/0	0/0	0/0	0/0	0/0	0/0	0/0	0/0	0/0	0/0	0/0	0/1
Chromosome1-1982477	INS	PRECISE	462	0	0.674	0/1	0/1	0/0	0/0	0/1	0/1	0/1	1/1	0/1	0/1	0/1	0/1	0/1	0/0
Chromosome1-1982605	INS	PRECISE	469	0	0	0/0	0/0	0/0	0/1	0/0	0/0	0/0	0/0	0/0	0/0	0/0	0/0	0/0	0/0
Chromosome1-1982962	DEL	PRECISE	218	0	0	0/0	0/0	0/0	0/0	0/0	0/0	1/1	0/0	0/0	0/0	0/0	0/0	0/0	0/0
Chromosome1-1983844	INS	PRECISE	5858	0	0	0/0	0/0	0/0	0/1	0/0	0/0	0/0	0/0	0/0	0/0	0/0	0/0	0/0	0/0
Chromosome1-1983844	BND	PRECISE	0	0	0	0/0	0/0	0/0	0/1	0/0	0/0	0/0	0/0	0/0	0/0	0/0	0/0	0/0	0/0
Chromosome1-1983890	DEL	PRECISE	211	0	0	0/0	0/0	0/0	0/0	0/0	0/0	0/0	0/0	0/0	0/0	0/0	0/1	0/0	0/0
Chromosome1-1984013	INS	PRECISE	975	0	0	0/0	0/0	0/0	0/0	0/0	0/0	0/0	0/0	0/0	0/0	0/0	0/1	0/0	0/0
Chromosome1-1984152	INS	PRECISE	11602	0	0	0/0	0/0	0/0	0/1	0/0	0/0	0/0	0/0	0/0	0/0	0/0	0/0	0/0	0/0
Chromosome1-1984152	BND	PRECISE	0	0	0	0/0	0/0	0/0	0/1	0/0	0/0	0/0	0/0	0/0	0/0	0/0	0/0	0/0	0/0
Chromosome1-1984247	DEL	PRECISE	14008	0	0	0/0	0/0	0/0	0/0	0/0	0/0	0/0	0/0	0/1	0/0	0/1	0/0	0/1	0/0
Chromosome1-1985303	DEL	PRECISE	159	0.289	0.289	0/1	0/1	0/0	0/1	0/1	0/1	0/1	1/1	0/1	0/1	0/1	1/1	0/1	0/0
Chromosome1-1985477	INS	PRECISE	57	0	0	0/0	0/0	0/0	0/0	0/0	0/0	0/1	0/0	0/0	0/0	0/0	0/0	0/0	0/0
Chromosome1-1985710	INS	PRECISE	147	0	0	0/0	0/0	0/0	0/0	0/0	0/0	0/1	0/0	0/0	0/0	0/0	0/0	0/0	0/0
Chromosome1-1985735	BND	PRECISE	0	0	0	0/0	0/0	0/0	0/0	0/0	0/0	0/0	0/0	0/0	0/0	0/0	0/1	0/0	0/0
Chromosome1-1985838	INS	PRECISE	1469	0	0	0/0	0/0	0/0	0/0	0/0	0/0	0/0	0/0	0/0	0/0	0/0	0/0	0/0	0/1
Chromosome1-1986239	BND	PRECISE	0	0	0	0/0	0/0	0/0	0/0	0/0	0/0	0/0	0/0	0/1	0/0	0/1	0/1	0/1	0/0
Chromosome1-1986536	DEL	PRECISE	263	0	0	0/1	1/1	0/1	1/1	1/1	1/1	1/1	1/1	1/1	1/1	1/1	1/1	1/1	1/1
Chromosome1-1986599	INS	PRECISE	213	0	0	0/0	0/0	0/0	0/0	0/0	0/0	0/1	0/0	0/0	0/0	0/0	0/0	0/0	0/0
Chromosome1-1986686	INS	PRECISE	1033	0	0	0/0	0/0	0/0	0/0	0/0	0/0	0/0	0/0	0/0	0/0	0/0	0/0	0/0	0/1
Chromosome1-1986942	INS	PRECISE	3036	0	0	0/0	0/0	0/0	0/0	0/0	0/0	1/1	0/0	0/0	0/0	0/0	0/0	0/0	0/0
Chromosome1-1987675	DEL	PRECISE	2301	0.5	0	0/0	0/0	0/0	0/0	0/0	0/0	0/0	0/0	0/1	0/0	0/1	0/1	0/1	0/0
Chromosome1-1988408	DEL	PRECISE	52	0	0	0/0	0/0	0/0	0/0	0/0	0/0	0/0	0/0	0/1	0/0	0/1	0/0	0/1	0/0
Chromosome1-1989289	INS	PRECISE	212	0	0.488	0/0	0/1	0/1	0/1	0/1	0/1	0/0	0/0	0/0	0/1	0/0	0/0	0/0	0/1
Chromosome1-1989972	DEL	PRECISE	67	0	0	0/0	0/0	0/0	0/0	0/0	0/0	0/0	0/0	0/0	0/0	0/0	0/1	0/0	0/0
Chromosome1-1990346	DEL	PRECISE	63	0	0	0/0	0/0	0/0	0/1	0/1	0/1	0/0	0/0	0/1	0/0	0/0	0/0	0/0	0/0
Chromosome1-1990546	INS	PRECISE	97	0	0.577	0/0	0/0	0/0	0/0	0/0	0/0	0/0	0/0	0/1	0/0	0/1	0/1	0/1	0/0
Chromosome1-1991583	INS	PRECISE	52	0	0	0/0	0/0	0/0	0/0	0/0	0/0	0/0	0/0	0/1	0/0	0/1	0/0	0/1	0/0
Chromosome1-1991750	DEL	PRECISE	56	0	0	0/0	0/0	0/0	0/1	0/0	0/0	0/0	0/0	0/0	0/0	0/0	0/0	0/0	0/0
Chromosome1-1991786	DEL	PRECISE	5188	0	0	0/1	1/1	0/1	1/1	1/1	1/1	1/1	1/1	1/1	1/1	1/1	1/1	1/1	1/1
Chromosome1-1992738	DEL	PRECISE	1352	0	0	0/0	0/0	0/0	0/0	0/0	0/0	0/0	0/0	0/1	0/0	0/1	0/1	0/1	0/0
Chromosome1-1993983	INS	PRECISE	207	0	0	0/0	0/0	0/0	0/0	0/0	0/0	0/1	0/0	0/0	0/0	0/0	0/0	0/0	0/0
Chromosome1-1995055	INS	PRECISE	1075	0	0	0/0	0/0	0/0	0/0	0/0	0/0	0/0	0/0	0/0	0/0	0/0	0/1	0/0	0/0
Chromosome1-1995869	INS	PRECISE	80	0	0	0/0	0/0	0/0	0/0	0/0	0/0	0/0	0/0	0/0	0/0	0/0	0/0	0/0	0/1
Chromosome1-1997041	DEL	PRECISE	73	0	0	0/0	0/0	0/0	0/0	0/0	0/0	0/0	0/0	0/1	0/0	0/1	0/0	0/0	0/0
Chromosome1-1998243	INV	PRECISE	4536359	0	0	0/0	0/0	0/0	0/0	0/0	0/0	0/0	0/0	0/0	0/0	0/0	0/0	0/0	0/1
Chromosome1-2000224	INS	PRECISE	96	0	0	0/0	0/0	0/0	0/0	0/0	0/0	0/0	0/0	0/0	0/0	0/1	1/1	0/1	0/0
Chromosome1-2000278	DEL	PRECISE	65	0	0	0/0	0/0	0/0	0/0	0/0	0/0	0/0	0/0	0/0	0/0	0/1	1/1	0/1	0/0
Chromosome1-2000733	INS	PRECISE	53	0	0	0/0	0/0	0/0	0/0	0/0	0/0	0/0	0/0	0/1	0/0	0/1	0/1	0/1	0/0
Chromosome1-2001062	DEL	PRECISE	95	0	0	0/0	0/0	0/0	0/0	0/0	0/0	0/0	0/0	0/0	0/0	0/1	0/0	0/0	0/0
Chromosome1-2001308	BND	PRECISE	0	0	0	0/0	0/0	0/0	0/0	0/0	0/0	0/1	0/0	0/0	0/0	0/0	0/0	0/0	0/0
Chromosome1-2003870	BND	PRECISE	0	0	0	0/0	0/0	0/0	0/0	0/0	0/0	0/0	0/0	0/1	0/0	0/1	0/0	0/1	0/0
Chromosome1-2003974	DEL	PRECISE	52	0	0	0/0	0/0	0/0	0/0	0/0	0/0	0/1	0/0	0/0	0/0	0/0	0/0	0/0	0/0
Chromosome1-2004264	BND	PRECISE	0	0.5	0	0/0	0/0	0/0	0/0	0/0	0/0	0/0	0/0	0/0	0/0	0/0	0/0	0/0	0/1
Chromosome1-2004917	INS	PRECISE	10704	0	0	0/0	0/0	0/0	0/0	0/0	0/0	0/0	0/0	0/0	0/0	0/0	0/1	0/0	0/0
Chromosome1-2006410	DEL	PRECISE	834	0	0	0/1	1/1	0/1	1/1	1/1	1/1	1/1	1/1	1/1	1/1	1/1	1/1	1/1	1/1
Chromosome1-2007065	DEL	PRECISE	933	0	0	0/0	0/0	0/0	0/0	0/0	0/0	0/0	0/0	0/1	0/0	0/1	0/1	0/1	0/0
Chromosome1-2007460	DEL	PRECISE	626	0	0	0/0	0/0	0/0	0/1	0/1	0/1	0/0	0/0	1/1	0/0	0/1	0/1	0/1	0/1
Chromosome1-2007997	INS	PRECISE	1539	0	0.9	0/0	0/1	0/1	0/1	0/1	0/1	0/0	0/0	0/0	0/1	0/0	0/0	0/0	0/1
Chromosome1-2008071	INS	PRECISE	721	0	0.577	0/0	0/0	0/0	0/0	0/0	0/0	0/0	0/0	0/1	0/0	0/0	0/0	0/1	0/0
Chromosome1-2008429	BND	PRECISE	0	0	0	0/0	0/1	0/0	0/1	0/0	0/1	0/0	0/0	0/0	0/0	0/0	0/0	0/0	0/1
Chromosome1-2009116	DEL	PRECISE	194	0	0	0/0	0/0	0/0	0/0	0/0	0/0	0/0	0/0	0/0	0/0	0/0	0/0	0/0	0/1
Chromosome1-2009314	DEL	PRECISE	364	0	0	0/0	0/0	0/0	0/0	0/0	0/0	0/0	0/0	1/1	0/0	0/1	0/1	0/1	0/0
Chromosome1-2010085	DEL	PRECISE	50	0	0	0/0	0/0	0/0	0/0	0/0	0/0	0/0	0/0	0/0	0/0	0/0	0/1	0/0	0/0
Chromosome1-2010517	INS	PRECISE	5573	0	0.707	0/0	0/0	0/0	0/0	0/0	0/0	1/1	0/0	0/0	0/0	0/0	0/0	0/0	0/1
Chromosome1-2011310	DEL	PRECISE	501	0	0	0/0	0/0	0/0	0/0	0/0	0/0	0/0	0/0	1/1	0/0	1/1	0/1	1/1	0/0
Chromosome1-2011717	DEL	PRECISE	89	0	0	0/0	0/1	0/1	0/1	0/1	0/1	0/0	0/0	0/0	0/1	0/0	0/0	0/0	0/1
Chromosome1-2011977	INS	PRECISE	132	0	0.577	0/0	0/0	0/0	0/0	0/1	0/1	0/0	0/0	0/1	0/0	0/0	0/0	0/0	0/0
Chromosome1-2013197	DEL	PRECISE	4534	0	0	0/0	0/0	0/0	0/0	0/0	0/0	0/0	0/0	0/0	0/0	0/0	0/0	0/0	0/0
Chromosome1-2013642	DEL	PRECISE	61	0	0	0/1	1/1	0/1	1/1	1/1	1/1	1/1	1/1	1/1	1/1	1/1	1/1	1/1	0/1
Chromosome1-2013936	DEL	PRECISE	363	0	0	0/1	1/1	0/1	1/1	1/1	1/1	1/1	1/1	1/1	1/1	1/1	1/1	1/1	1/1
Chromosome1-2016191	DEL	PRECISE	698	0	0	0/0	0/0	0/0	0/0	0/0	0/0	0/1	0/0	0/0	0/0	0/0	0/0	0/0	0/0
Chromosome1-2016591	DEL	PRECISE	943	0	0	0/0	0/0	0/0	0/0	0/0	0/0	0/0	0/0	0/0	0/0	0/0	0/0	0/0	0/1
Chromosome1-2017278	INS	PRECISE	142	0	0	0/0	0/0	0/0	0/0	0/0	0/0	0/0	0/0	0/0	0/0	0/0	0/1	0/0	0/0
Chromosome1-2017910	BND	PRECISE	0	0	0	0/0	0/0	0/0	0/0	0/0	0/0	0/0	0/0	0/0	0/0	0/0	0/1	0/0	0/0
Chromosome1-2017945	INS	PRECISE	1317	0	0	0/0	0/0	0/0	0/0	0/0	0/0	0/0	0/0	0/1	0/0	0/1	0/0	0/0	0/0
Chromosome1-2018026	DEL	PRECISE	102	0	0	0/0	0/0	0/0	0/0	0/0	0/0	0/0	0/0	0/1	0/0	0/1	0/0	0/0	0/0
Chromosome1-2020399	INS	PRECISE	2184	0	0	0/0	0/0	0/0	0/0	0/0	0/0	0/0	0/0	0/0	0/0	0/0	0/1	0/0	0/0
Chromosome1-2020636	DEL	PRECISE	54	0	0	0/0	0/1	0/1	0/1	0/1	0/1	0/0	0/0	0/0	0/1	0/0	0/0	0/0	0/1
Chromosome1-2021057	DEL	PRECISE	482	0	0	0/0	0/0	0/0	0/0	0/0	0/0	0/0	0/0	0/0	0/0	0/1	0/0	0/1	0/0

Chromosome1-2021214	DEL	PRECISE	1389	0	0	0/1	1/1	0/1	1/1	1/1	1/1	1/1	1/1	1/1	1/1	1/1	1/1	1/1	1/1
Chromosome1-2021672	INS	PRECISE	5774	0	0	0/0	0/0	0/0	0/0	0/0	0/0	0/0	0/0	0/0	0/0	0/0	0/1	0/0	0/0
Chromosome1-2021900	INS	PRECISE	1194	0	0	0/0	0/0	0/0	0/0	0/0	0/0	0/0	0/0	0/0	0/0	0/0	0/1	0/0	0/0
Chromosome1-2022370	INS	PRECISE	6008	0	0	0/0	0/0	0/0	0/0	0/0	0/0	0/0	0/0	0/0	0/0	0/0	0/1	0/0	0/0
Chromosome1-2022698	DEL	PRECISE	177	0	0	0/0	0/0	0/0	0/0	0/0	0/0	0/0	0/0	0/0	0/0	0/0	0/0	0/0	0/1
Chromosome1-2024446	INS	PRECISE	53	0	0.577	0/0	0/0	0/0	0/0	0/0	0/0	0/0	0/0	0/1	0/0	0/1	0/0	0/1	0/0
Chromosome1-2024902	INS	PRECISE	1392	0	0	0/0	0/0	0/0	0/0	0/0	0/0	0/1	0/0	0/0	0/0	0/0	0/0	0/0	0/0
Chromosome1-2025512	DEL	PRECISE	62	0	0	0/0	0/0	0/0	0/0	0/0	0/0	0/1	0/0	0/0	0/0	0/0	0/0	0/0	0/0
Chromosome1-2027612	DEL	PRECISE	3569	0	0	0/0	0/0	0/0	0/0	0/0	0/0	0/0	0/0	0/0	0/0	0/0	0/1	0/0	0/0
Chromosome1-2028006	INS	PRECISE	104	0	0	0/0	0/0	0/0	0/0	0/0	0/0	0/0	0/0	0/0	0/0	0/0	0/1	0/0	0/0
Chromosome1-2028241	DEL	PRECISE	1563	0	0	0/0	0/0	0/0	0/0	0/0	0/0	0/0	0/0	0/0	0/0	0/0	0/1	0/0	0/0
Chromosome1-2028435	INS	PRECISE	6913	0	0	0/0	0/0	0/0	0/0	0/0	0/0	0/0	0/0	0/0	0/0	0/0	0/1	0/0	0/0
Chromosome1-2028674	INS	PRECISE	1313	0	0	0/0	0/0	0/0	0/0	0/0	0/0	0/0	0/0	0/0	0/0	0/0	0/1	0/0	0/0
Chromosome1-2029514	DEL	PRECISE	12053	0	0	0/0	0/0	0/0	0/0	0/0	0/0	0/0	0/0	0/0	0/0	0/0	0/1	0/0	0/0
Chromosome1-2029560	DEL	PRECISE	55	0	0	0/0	0/0	0/0	0/0	0/0	0/0	0/1	0/0	0/0	0/0	0/0	0/0	0/0	0/0
Chromosome1-2029745	DEL	PRECISE	208	0	0	0/0	0/1	0/1	0/1	0/1	0/1	0/0	0/0	0/0	0/1	0/0	0/0	0/0	0/1
Chromosome1-2030955	INS	PRECISE	4555	0	0	0/0	0/0	0/0	0/0	0/0	0/0	0/0	0/0	0/0	0/0	0/0	0/1	0/0	0/0
Chromosome1-2031296	DEL	PRECISE	932	0	0	0/0	0/0	0/0	0/0	0/0	0/0	0/1	0/0	0/0	0/0	0/0	0/0	0/0	0/0
Chromosome1-2031490	DEL	PRECISE	186	0	0	0/0	0/0	0/0	0/0	0/0	0/0	0/0	0/0	0/0	0/0	0/0	0/1	0/0	0/0
Chromosome1-2031650	DEL	PRECISE	792	0	0	0/0	0/0	0/0	0/0	0/0	0/0	0/1	0/0	0/0	0/0	0/0	0/0	0/0	0/0
Chromosome1-2032347	DEL	PRECISE	58	0	0	0/1	0/1	0/0	0/1	0/1	0/1	0/1	1/1	1/1	0/1	1/1	1/1	1/1	0/0
Chromosome1-2032787	INS	PRECISE	761	0	0	0/0	0/0	0/0	0/0	0/0	0/0	0/1	0/0	0/0	0/0	0/0	0/0	0/0	0/0
Chromosome1-2033240	INS	PRECISE	219	0	0.5	0/0	0/0	0/0	0/0	0/0	0/0	0/0	0/0	0/1	0/0	0/1	0/1	0/1	0/0
Chromosome1-2033331	DEL	PRECISE	818	0	0	0/0	0/0	0/0	0/0	0/0	0/0	0/0	0/0	0/1	0/0	0/1	0/1	0/1	0/0
Chromosome1-2033483	DEL	PRECISE	5175	0	0	0/0	0/0	0/0	0/0	0/0	0/0	0/0	0/0	0/1	0/0	0/1	0/1	0/1	0/0
Chromosome1-2033609	DEL	PRECISE	95	0	0	0/0	0/0	0/0	0/0	0/0	0/0	0/0	0/0	0/0	0/0	0/0	0/0	0/0	0/1
Chromosome1-2033945	INS	PRECISE	165	0	0	0/1	1/1	0/1	1/1	1/1	1/1	1/1	1/1	0/1	1/1	0/1	0/1	0/1	1/1
Chromosome1-2034426	INS	PRECISE	1441	0	0	0/0	0/0	0/0	0/0	0/0	0/0	0/0	0/0	0/0	0/0	0/0	0/1	0/0	0/0
Chromosome1-2034752	INS	PRECISE	6276	0	0	0/0	0/0	0/0	0/0	0/0	0/0	0/0	0/0	0/0	0/0	0/0	0/0	0/0	1/1
Chromosome1-2034792	DEL	PRECISE	119	0	0	0/0	0/0	0/0	0/0	0/0	0/0	0/0	0/0	0/1	0/0	0/1	0/0	0/1	0/0
Chromosome1-2035171	DEL	PRECISE	55	0	0	0/1	1/1	0/1	1/1	1/1	1/1	1/1	1/1	1/1	1/1	1/1	1/1	1/1	1/1
Chromosome1-2035257	INS	PRECISE	1111	0	0	0/0	0/0	0/0	0/0	0/0	0/0	0/0	0/0	0/0	0/0	0/0	0/1	0/0	0/0
Chromosome1-2036115	BND	PRECISE	0	0	0	0/0	0/0	0/0	0/0	0/0	0/0	0/0	0/0	1/1	0/0	0/1	0/0	1/1	0/0
Chromosome1-2036524	BND	PRECISE	0	0	0	0/0	0/0	0/0	0/1	0/0	0/0	0/0	0/0	0/0	0/0	0/0	0/0	0/0	0/0
Chromosome1-2036524	BND	PRECISE	0	0	0	0/0	0/0	0/0	0/0	0/0	0/0	0/1	0/0	0/0	0/0	0/0	0/0	0/0	0/1
Chromosome1-2037401	INS	PRECISE	56	0	0	0/0	0/0	0/0	0/1	0/1	0/0	0/0	0/0	0/1	0/0	0/0	0/0	0/0	0/0
Chromosome1-2037488	BND	PRECISE	0	0	0	0/0	0/0	0/0	1/1	0/0	0/0	0/0	0/0	0/0	0/0	0/0	0/0	0/0	0/0
Chromosome1-2037882	DEL	PRECISE	644	0.354	0.354	0/0	0/1	0/1	0/1	0/1	0/1	0/1	0/0	0/0	0/1	0/0	0/0	0/0	1/1
Chromosome1-2037903	INS	PRECISE	50	0	0.289	0/1	0/1	0/0	0/1	0/1	0/1	0/1	1/1	1/1	0/1	1/1	1/1	1/1	0/0
Chromosome1-2038197	BND	PRECISE	0	0	0	0/0	0/0	0/0	0/0	0/0	0/0	0/0	0/0	0/1	0/0	0/1	0/1	0/0	0/0
Chromosome1-2038311	INS	PRECISE	7086	0	0	0/0	0/0	0/0	0/0	0/0	0/0	0/0	0/0	0/0	0/0	0/0	0/0	0/0	1/1
Chromosome1-2038311	BND	PRECISE	0	0	0	0/0	0/0	0/0	0/0	0/0	0/0	0/0	0/0	0/0	0/0	0/0	0/0	0/0	0/1
Chromosome1-2039117	BND	PRECISE	0	0	0	0/0	0/0	0/0	0/0	0/0	0/0	0/0	0/0	0/1	0/0	0/1	0/0	0/0	0/0
Chromosome1-2039224	DEL	PRECISE	4050	0.447	0.447	0/0	0/0	0/0	0/0	0/0	0/0	0/0	0/0	0/1	0/0	0/1	0/1	0/1	0/1
Chromosome1-2039468	INS	PRECISE	165	0	0	0/0	0/0	0/0	0/1	0/0	0/0	0/0	0/0	0/0	0/0	0/0	0/0	0/0	0/0
Chromosome1-2040323	INS	PRECISE	1201	0	0.957	0/0	0/0	0/0	0/1	0/1	0/1	0/0	0/0	0/1	0/0	0/0	0/0	0/0	0/0
Chromosome1-2042327	DEL	PRECISE	52	0	0	0/0	0/1	0/1	0/1	0/1	0/1	0/0	0/0	0/0	0/1	0/0	0/0	0/0	1/1
Chromosome1-2042435	DEL	PRECISE	624	0.535	0	0/1	1/1	0/1	1/1	1/1	1/1	1/1	1/1	1/1	1/1	1/1	1/1	1/1	1/1
Chromosome1-2042701	BND	PRECISE	0	0	0	0/0	0/0	0/0	0/0	0/0	0/0	0/0	0/0	0/0	0/0	0/0	0/0	0/0	0/1
Chromosome1-2043992	DEL	PRECISE	1462	0	0	0/0	0/0	0/0	0/0	0/0	0/0	0/0	0/0	0/1	0/0	0/1	0/1	0/0	0/0
Chromosome1-2044569	INS	PRECISE	3793	0	0	0/0	0/0	0/0	0/0	0/0	0/0	0/0	0/0	0/1	0/0	0/0	0/0	0/0	0/0
Chromosome1-2044986	BND	PRECISE	0	0	0	0/0	0/0	0/0	0/0	0/1	0/0	0/0	0/0	0/1	0/0	0/0	0/0	0/0	0/0
Chromosome1-2045036	DEL	PRECISE	4314	0.802	0.802	0/1	1/1	0/1	1/1	1/1	1/1	1/1	1/1	1/1	1/1	1/1	1/1	1/1	1/1
Chromosome1-2045716	DEL	PRECISE	51	0	0	0/1	1/1	0/1	0/1	0/1	0/1	1/1	1/1	0/0	1/1	0/1	0/1	0/1	0/1
Chromosome1-2046657	INS	PRECISE	5859	0	0	0/0	0/0	0/0	0/0	0/0	0/0	0/0	0/0	0/0	0/0	0/0	0/0	0/0	1/1
Chromosome1-2047391	INS	PRECISE	6575	0	0	0/0	0/0	0/0	0/0	0/0	0/0	0/0	0/0	0/0	0/0	0/0	0/0	0/0	0/1
Chromosome1-2047486	DEL	PRECISE	336	0	0	0/0	0/0	0/0	0/0	0/0	0/0	0/0	0/0	0/0	0/0	0/0	0/1	0/0	0/0
Chromosome1-2047676	DEL	PRECISE	79	0	0	0/0	0/0	0/0	0/0	0/0	0/0	0/1	0/0	0/0	0/0	0/0	0/0	0/0	0/0
Chromosome1-2047760	DEL	PRECISE	623	0	0	0/0	0/0	0/0	0/0	0/0	0/0	0/0	0/0	0/1	0/0	0/1	0/1	0/1	0/1
Chromosome1-2048062	DEL	PRECISE	65	0	0	0/0	0/0	0/0	0/0	0/0	0/0	0/0	0/0	0/1	0/0	0/1	0/1	0/1	0/0
Chromosome1-2048214	INS	PRECISE	1254	0	0	0/0	0/0	0/0	0/0	0/0	0/0	0/1	0/0	0/0	0/0	0/0	0/0	0/0	0/0
Chromosome1-2049217	DEL	PRECISE	243	0	0	0/1	0/1	0/0	0/0	0/0	0/0	1/1	1/1	0/0	0/1	0/1	0/1	0/1	0/0
Chromosome1-2049658	BND	PRECISE	0	0	0	0/0	0/0	0/0	0/0	0/0	0/0	0/1	0/0	0/0	0/0	0/0	0/0	0/0	0/0
Chromosome1-2049662	DUP	PRECISE	191579	0	0	0/0	0/0	0/0	0/1	0/1	0/1	0/0	0/0	0/1	0/0	0/0	0/0	0/0	0/0
Chromosome1-2049930	INS	PRECISE	482	0	0.577	0/0	0/0	0/0	0/0	0/0	0/0	0/0	0/0	1/1	0/0	0/1	0/1	0/1	0/0
Chromosome1-2051084	DEL	PRECISE	232	0	0	0/0	0/0	0/0	0/0	0/0	0/0	0/1	0/0	0/0	0/0	0/0	0/0	0/0	0/0
Chromosome1-2052415	DEL	PRECISE	8347	0	0	0/0	0/0	0/0	1/1	1/1	0/1	0/0	0/0	0/0	0/0	0/0	0/0	0/0	0/1
Chromosome1-2053302	DEL	PRECISE	3363	0	0	0/0	0/0	0/0	0/0	0/0	0/0	0/1	0/0	0/0	0/0	0/0	0/0	0/0	0/0
Chromosome1-2053541	INV	PRECISE	103485	0	0	0/0	0/0	0/0	0/0	0/0	0/0	0/0	0/0	0/1	0/0	0/1	0/1	0/0	0/0
Chromosome1-2054059	INS	PRECISE	72	0	0	0/0	0/0	0/0	0/0	0/0	0/0	0/0	0/0	0/0	0/0	0/0	0/0	0/0	0/1
Chromosome1-2054096	DEL	PRECISE	141	0	0	0/0	0/0	0/0	0/0	0/0	0/0	0/0	0/0	0/0	0/0	0/0	0/0	0/0	0/1
Chromosome1-2054506	INS	PRECISE	3232	0	0	0/0	0/0	0/0	0/0	0/0	0/0	0/1	0/0	0/0	0/0	0/0	0/0	0/0	0/0
Chromosome1-2054653	BND	PRECISE	0	0	0	0/0	0/0	0/0	0/0	0/0	0/0	0/0	0/0	0/0	0/0	0/0	0/0	0/1	0/0

Chromosome1-2054829	DEL	PRECISE	1004	0	0	0/0	0/0	0/0	0/0	0/0	0/0	0/1	0/0	0/0	0/0	0/0	0/0	0/0	0/0
Chromosome1-2054966	INS	PRECISE	162	0	0.376	0/1	1/1	0/1	1/1	0/1	0/1	0/1	1/1	0/0	1/1	0/1	0/1	0/0	0/1
Chromosome1-2054969	DEL	PRECISE	109	0	0	0/0	0/0	0/0	0/0	0/0	0/0	0/0	0/0	0/1	0/0	0/1	0/1	1/1	0/0
Chromosome1-2055751	DEL	PRECISE	57	0	0	0/1	1/1	0/1	0/1	0/1	0/1	0/1	1/1	0/0	1/1	0/1	0/1	0/1	0/1
Chromosome1-2055891	INS	PRECISE	1315	0	0	0/0	0/0	0/0	0/0	0/0	0/0	0/0	0/0	0/0	0/0	0/0	0/0	0/0	0/1
Chromosome1-2056150	DEL	PRECISE	3672	0	0	0/0	0/0	0/0	0/0	0/0	0/0	0/0	0/0	0/1	0/0	0/1	0/1	0/1	0/0
Chromosome1-2056610	INS	PRECISE	117	0	0	0/1	1/1	0/1	1/1	0/1	0/1	0/1	1/1	0/1	1/1	1/1	1/1	1/1	1/1
Chromosome1-2056689	INS	PRECISE	9981	0	0	0/0	0/0	0/0	0/0	0/0	0/0	0/0	0/0	0/0	0/0	0/0	0/0	0/0	0/1
Chromosome1-2056832	INS	PRECISE	684	0	0	0/0	0/0	0/0	0/0	0/0	0/0	0/0	0/0	0/0	0/0	0/0	0/0	0/0	0/1
Chromosome1-2057377	INS	PRECISE	9165	0	0	0/0	0/0	0/0	0/0	0/0	0/0	0/0	0/0	0/0	0/0	0/0	0/0	0/0	1/1
Chromosome1-2057634	DEL	PRECISE	2577	0	0	0/0	0/0	0/0	0/0	0/0	0/0	0/1	0/0	0/0	0/0	0/0	0/0	0/0	0/0
Chromosome1-2057960	INS	PRECISE	63	0	0	0/0	0/0	0/0	0/0	0/1	0/1	0/0	0/0	1/1	0/0	0/0	0/0	0/0	0/0
Chromosome1-2058026	DEL	PRECISE	1113	0	0	0/0	0/0	0/0	0/0	0/0	0/0	0/1	0/0	0/0	0/0	0/0	0/0	0/0	1/1
Chromosome1-2058081	INS	PRECISE	50	0	0	0/0	0/0	0/0	0/0	0/1	0/1	0/0	0/0	1/1	0/0	0/0	0/0	0/0	0/0
Chromosome1-2059105	DEL	PRECISE	1549	0.447	0	0/0	0/0	0/0	0/0	1/1	1/1	1/1	0/0	1/1	0/0	0/0	0/0	0/0	1/1
Chromosome1-2060470	DEL	PRECISE	66	0	0	0/0	0/0	0/0	0/0	0/0	0/0	0/0	0/0	0/0	0/0	0/0	0/0	0/0	1/1
Chromosome1-2061352	DEL	PRECISE	56	0	0	0/0	0/0	0/0	1/1	0/0	0/0	0/0	0/0	0/0	0/0	0/0	0/0	0/0	0/0
Chromosome1-2061435	DEL	PRECISE	455	0	0	0/0	0/0	0/0	1/1	0/0	0/0	0/0	0/0	0/0	0/0	0/0	0/0	0/0	0/0
Chromosome1-2062836	DEL	PRECISE	10541	0	0	0/0	0/0	0/0	0/0	0/1	0/1	0/1	0/0	0/1	0/0	0/0	0/0	0/0	1/1
Chromosome1-2062836	BND	PRECISE	0	0	0	0/0	0/0	0/0	0/0	0/0	0/0	0/1	0/0	0/0	0/0	0/0	0/0	0/0	0/0
Chromosome1-2062836	BND	PRECISE	0	0	0	0/0	0/1	0/0	0/1	0/1	0/1	0/0	0/0	0/1	0/0	0/0	0/0	0/0	0/0
Chromosome1-2062836	BND	PRECISE	0	0	0	0/0	0/1	0/0	0/0	0/0	0/0	0/0	0/0	0/0	0/0	0/1	0/0	0/0	0/0
Chromosome1-2062836	BND	PRECISE	0	0	0	0/0	0/1	0/0	0/0	0/0	0/0	0/0	0/0	0/0	0/0	0/0	0/0	0/0	0/0
Chromosome1-2062836	BND	PRECISE	0	0	0	0/0	0/0	0/0	0/0	0/0	0/1	0/0	0/0	0/0	0/0	0/0	0/0	0/0	0/0
Chromosome1-2062880	DEL	PRECISE	56	0	0	0/1	1/1	0/1	1/1	0/1	0/1	0/0	0/1	0/1	1/1	1/1	1/1	0/0	0/0
Chromosome1-2063890	BND	PRECISE	0	0	0	0/1	0/0	1/1	0/0	1/1	1/1	0/1	0/0	1/1	0/0	0/0	0/0	0/0	0/0
Chromosome1-2063890	BND	PRECISE	0	0	0	0/0	0/0	0/0	0/0	0/0	0/0	0/1	0/0	0/0	0/0	0/0	0/0	0/0	0/0
Chromosome1-2065784	BND	PRECISE	0	0	0	0/0	0/0	0/0	0/0	0/0	0/0	0/0	0/0	0/0	0/0	0/0	0/0	0/0	0/1
Chromosome1-2066145	DEL	PRECISE	134	0	0	0/0	0/0	0/0	0/0	0/0	0/0	0/0	0/0	0/0	0/0	0/0	0/0	0/0	0/1
Chromosome1-2067185	DEL	PRECISE	16984	0	0	0/0	0/0	0/0	1/1	0/0	0/0	0/1	0/0	0/1	0/0	0/0	0/0	0/0	0/0
Chromosome1-2068387	DEL	PRECISE	206	0	0	0/0	0/0	0/0	1/1	0/1	0/1	0/0	0/0	0/1	0/0	0/0	0/0	0/0	0/0
Chromosome1-2068512	INS	PRECISE	52	0	0	0/0	0/0	0/0	1/1	1/1	1/1	1/1	0/0	1/1	0/0	0/0	0/0	0/0	1/1
Chromosome1-2069581	BND	PRECISE	0	0	0	0/0	0/0	0/0	0/0	0/0	0/0	0/0	0/0	0/1	0/0	0/0	0/0	0/0	0/0
Chromosome1-2069581	BND	PRECISE	0	0	0	0/0	0/0	0/0	0/0	0/0	0/0	0/0	0/0	0/0	0/0	0/0	0/0	0/0	0/1
Chromosome1-2069978	INS	PRECISE	1484	0	0	0/0	0/0	0/0	0/0	0/0	0/0	0/1	0/0	0/0	0/0	0/0	0/0	0/0	0/0
Chromosome1-2070352	BND	PRECISE	0	0	0	0/0	0/0	0/0	0/0	0/0	0/0	0/1	0/0	0/0	0/0	0/0	0/0	0/0	0/0
Chromosome1-2070421	BND	PRECISE	0	0	0	0/0	0/0	0/0	0/0	0/1	0/0	0/0	0/0	1/1	0/0	0/0	0/1	0/0	0/1
Chromosome1-2071102	INS	PRECISE	170	0	0	0/0	0/0	0/0	0/0	0/0	0/0	0/1	0/0	0/0	0/0	0/0	0/0	0/0	0/0
Chromosome1-2071423	DEL	PRECISE	157	0	0	0/0	0/0	0/0	0/0	0/0	0/0	0/0	0/0	0/1	0/0	0/1	0/1	0/1	0/0
Chromosome1-2072000	DEL	PRECISE	52	0	0	0/0	0/0	0/0	0/0	0/0	0/0	0/0	0/0	0/0	0/0	0/0	0/0	0/0	0/1
Chromosome1-2073923	INS	PRECISE	2235	0	0	0/0	0/0	0/0	0/0	0/0	0/0	0/0	0/0	0/0	0/0	0/0	0/1	0/0	0/0
Chromosome1-2074052	INS	PRECISE	11187	0	0	0/0	0/0	0/0	0/0	0/0	0/0	0/0	0/0	0/0	0/0	0/0	1/1	0/0	0/0
Chromosome1-2074507	BND	PRECISE	0	0.894	0	0/0	0/1	0/0	0/0	0/1	0/0	0/0	0/0	0/0	0/0	0/0	0/0	0/0	0/0
Chromosome1-2074531	DEL	PRECISE	11928	0	0	0/0	0/0	0/0	0/0	0/0	0/0	0/0	0/0	0/1	0/0	1/1	0/1	0/1	0/0
Chromosome1-2075773	DEL	PRECISE	407	0	0	0/0	0/0	0/0	0/0	0/0	0/0	0/0	0/0	0/1	0/0	0/1	0/1	0/1	0/0
Chromosome1-2077054	BND	PRECISE	0	0	0	0/0	0/0	0/0	0/0	0/0	0/0	0/0	0/0	0/0	0/0	0/0	0/0	0/1	0/0
Chromosome1-2078461	INS	PRECISE	938	0	0	0/0	0/0	0/0	0/0	0/0	0/0	0/1	0/0	0/0	0/0	0/0	0/0	0/0	0/0
Chromosome1-2078461	DEL	PRECISE	942	0	0	0/1	1/1	0/1	0/1	1/1	1/1	0/1	1/1	1/1	1/1	1/1	1/1	1/1	1/1
Chromosome1-2079190	DEL	PRECISE	73	0	0	0/1	0/1	0/0	0/0	0/1	0/1	0/1	1/1	1/1	0/1	0/1	0/1	1/1	1/1
Chromosome1-2079345	INS	PRECISE	11243	0	0	0/0	0/0	0/0	0/0	0/0	0/0	0/0	0/0	0/0	0/0	0/0	0/1	0/0	0/0
Chromosome1-2079930	INS	PRECISE	1578	0.5	0.577	0/0	0/0	0/0	0/0	0/0	0/0	0/0	0/0	0/1	0/0	0/1	0/1	0/0	0/0
Chromosome1-2080062	INS	PRECISE	5072	0	0.957	0/0	0/0	0/0	0/0	0/0	0/0	0/0	0/0	0/1	0/0	0/1	0/1	0/0	0/0
Chromosome1-2080544	DEL	PRECISE	8373	0	0	0/0	0/0	0/0	0/0	0/0	0/0	0/0	0/0	0/0	0/0	0/0	0/0	0/0	0/1
Chromosome1-2080596	DEL	PRECISE	3893	0	0	0/1	0/1	0/0	0/0	0/1	0/1	0/1	1/1	1/1	0/1	1/1	1/1	1/1	0/0
Chromosome1-2081866	DEL	PRECISE	308	0	0	0/0	0/0	0/0	0/0	0/0	0/0	0/0	0/0	0/1	0/0	0/1	0/1	0/0	0/0
Chromosome1-2082013	INS	PRECISE	98	0	0.5	0/0	0/0	0/0	0/0	0/0	0/0	0/0	0/0	0/1	0/0	0/1	0/1	0/0	0/0
Chromosome1-2082381	DEL	PRECISE	1015	0	0	0/0	0/0	0/0	0/0	0/0	0/0	0/0	0/0	0/1	0/0	0/1	0/1	0/0	0/0
Chromosome1-2082902	DEL	PRECISE	4863	0.267	0.267	0/1	1/1	0/1	1/1	1/1	1/1	1/1	1/1	1/1	1/1	1/1	1/1	1/1	1/1
Chromosome1-2083459	INS	PRECISE	473	0	0	0/0	0/0	0/0	0/1	0/0	0/0	0/0	0/0	0/0	0/0	0/0	0/0	0/0	0/0
Chromosome1-2084223	DEL	PRECISE	5277	0	0	0/0	0/0	0/0	0/0	0/0	0/0	0/0	0/0	1/1	0/0	0/1	0/1	0/1	1/1
Chromosome1-2084974	INS	PRECISE	7431	0	0	0/0	0/0	0/0	0/0	0/0	0/0	0/0	0/0	0/0	0/0	0/0	0/0	0/0	0/1
Chromosome1-2085870	DEL	PRECISE	52	0	0	0/0	0/0	0/0	0/0	0/0	0/0	0/0	0/0	0/1	0/0	0/1	0/1	0/1	0/0
Chromosome1-2086182	DEL	PRECISE	134	0	0	0/0	0/0	0/0	0/0	0/0	0/0	0/0	0/0	0/1	0/0	0/1	0/1	0/1	0/0
Chromosome1-2086426	DEL	PRECISE	61	0	0	0/0	0/0	0/0	0/0	0/0	0/0	0/0	0/0	0/1	0/0	0/1	0/1	0/1	0/0
Chromosome1-2086676	DEL	PRECISE	5437	0	0	0/0	0/0	0/0	1/1	0/0	0/0	0/0	0/0	0/1	0/0	0/1	0/1	0/1	0/0
Chromosome1-2087111	INS	PRECISE	77	0	0	0/1	1/1	0/1	0/1	1/1	1/1	1/1	1/1	0/1	1/1	0/1	0/1	0/1	1/1
Chromosome1-2087245	INS	PRECISE	138	0	0	0/0	0/0	0/0	0/0	0/0	0/0	0/0	0/0	0/0	0/0	0/0	0/1	0/0	0/0
Chromosome1-2087309	DEL	PRECISE	10928	0	0	0/0	0/0	0/0	0/1	0/0	0/0	0/0	0/0	0/1	0/0	0/1	0/1	0/1	0/0
Chromosome1-2088687	INS	PRECISE	9705	0	0	0/0	0/0	0/0	1/1	0/0	0/0	0/0	0/0	0/0	0/0	0/0	0/0	0/0	0/0
Chromosome1-2088800	DEL	PRECISE	87	0	0	0/0	0/0	0/0	0/1	0/0	0/0	0/0	0/0	0/0	0/0	0/0	0/0	0/0	0/0
Chromosome1-2089109	INS	PRECISE	5085	0	0	0/0	0/0	0/0	1/1	0/0	0/0	0/0	0/0	0/0	0/0	0/0	0/0	0/0	0/0
Chromosome1-2089445	INS	PRECISE	5881	0	0	0/0	0/0	0/0	0/0	0/0	0/0	0/0	0/0	0/0	0/0	0/0	1/1	0/0	0/0
Chromosome1-2089503	INS	PRECISE	122	0	0	0/0	0/0	0/0	0/0	0/0	0/0	0/0	0/0	0/0	0/0	0/0	0/0	0/0	0/1

Chromosome1-2090233	INS	PRECISE	54	0	0	0/1	1/1	0/1	0/1	1/1	1/1	0/1	1/1	1/1	1/1	1/1	1/1	1/1	0/1
Chromosome1-2090348	INS	PRECISE	403	0	0	0/0	0/0	0/0	0/0	0/0	0/0	0/1	0/0	0/0	0/0	0/0	0/0	0/0	0/0
Chromosome1-2091693	DEL	PRECISE	61	0	0	0/0	0/0	0/0	0/1	0/0	0/0	0/0	0/0	0/0	0/0	0/0	0/0	0/0	0/0
Chromosome1-2092498	DEL	PRECISE	4367	0	0	0/0	0/0	0/0	1/1	0/0	0/0	0/0	0/0	0/0	0/0	0/0	0/0	0/0	0/0
Chromosome1-2093024	BND	PRECISE	0	0	0	0/0	0/0	0/0	0/0	0/1	0/1	0/0	0/0	0/1	0/0	0/0	0/0	0/0	0/0
Chromosome1-2093615	DEL	PRECISE	376	0	0	0/0	0/0	0/0	0/1	0/0	0/0	0/0	0/0	0/0	0/0	0/0	0/0	0/0	0/0
Chromosome1-2093794	DEL	PRECISE	84	0	0	0/0	0/0	0/0	0/1	0/1	0/1	0/0	0/0	0/1	0/1	0/0	0/0	0/0	0/0
Chromosome1-2094154	BND	PRECISE	0	0	0	0/0	0/0	0/1	0/0	0/0	0/0	0/0	0/0	0/0	0/0	0/0	0/0	0/0	0/0
Chromosome1-2094154	BND	PRECISE	0	0	0	0/0	0/0	0/1	0/0	0/0	0/0	0/0	0/0	0/0	0/0	0/0	0/0	0/0	0/0
Chromosome1-2094933	INS	PRECISE	1254	0	0	0/0	0/0	0/0	0/1	0/0	0/0	0/0	0/0	0/0	0/0	0/0	0/0	0/0	0/0
Chromosome1-2094955	INS	PRECISE	4875	0	0	0/0	0/0	0/0	0/0	0/0	0/0	0/0	0/0	0/0	0/0	0/0	0/0	0/0	1/1
Chromosome1-2095402	DEL	PRECISE	3342	0	0	0/0	0/0	0/0	0/0	0/0	0/1	0/0	0/0	0/0	0/0	0/0	0/0	0/0	0/1
Chromosome1-2095416	DEL	PRECISE	2160	0	0	0/0	0/0	0/0	0/0	0/0	0/0	0/0	0/0	0/0	0/0	0/0	0/0	0/0	0/1
Chromosome1-2095615	INS	PRECISE	667	0	0	0/0	0/0	0/0	1/1	0/0	0/0	0/0	0/0	0/0	0/0	0/0	0/0	0/0	0/0
Chromosome1-2095692	DEL	PRECISE	594	0	0	0/0	0/0	0/0	0/0	0/0	0/0	0/0	0/0	0/0	0/0	0/0	0/0	0/0	0/1
Chromosome1-2095738	INS	PRECISE	2048	0	0	0/0	0/0	0/0	0/0	0/0	0/0	0/0	0/0	0/0	0/0	0/0	0/1	0/0	0/0
Chromosome1-2096263	INS	PRECISE	4834	0	0	0/0	0/0	0/0	0/0	0/0	0/0	0/0	0/0	0/0	0/0	0/0	1/1	0/0	0/0
Chromosome1-2096396	INS	PRECISE	10692	0	0	0/0	0/0	0/0	0/0	0/0	0/0	0/0	0/0	0/0	0/0	0/0	0/0	0/0	0/1
Chromosome1-2096494	INS	PRECISE	800	0	0	0/0	0/0	0/0	0/0	0/0	0/0	0/0	0/0	0/0	0/0	0/0	0/1	0/0	0/0
Chromosome1-2097012	INS	PRECISE	900	0	0	0/0	0/0	0/0	0/0	0/0	0/0	0/0	0/0	0/0	0/0	0/0	0/0	0/0	0/1
Chromosome1-2098302	DEL	PRECISE	52	0	0	0/0	0/0	0/0	0/0	0/0	0/0	0/0	0/0	0/0	0/0	0/0	0/0	0/0	0/1
Chromosome1-2098847	DEL	PRECISE	6640	0	0	0/0	1/1	0/1	1/1	1/1	1/1	0/0	0/0	0/0	1/1	0/0	0/0	0/0	1/1
Chromosome1-2099760	BND	PRECISE	0	0	0	0/0	0/0	0/0	0/0	0/0	0/0	0/0	0/0	0/1	0/0	0/0	0/0	0/0	0/0
Chromosome1-2100335	DEL	PRECISE	184	0	0	0/0	0/0	0/0	0/0	0/0	0/0	0/0	0/0	0/0	0/0	0/0	0/0	0/1	0/0
Chromosome1-2100369	DEL	PRECISE	1587	0	0	0/0	0/0	0/0	0/0	0/0	0/0	0/0	0/0	0/0	0/0	0/0	0/0	0/0	0/1
Chromosome1-2100371	DEL	PRECISE	5718	0	0	0/0	0/0	0/0	0/0	0/0	0/0	0/0	0/0	0/0	0/0	0/0	0/0	0/0	0/1
Chromosome1-2100530	DEL	PRECISE	4123	0	0	0/0	0/0	0/0	0/0	0/0	0/0	0/0	0/0	0/0	0/0	0/0	0/0	0/0	0/1
Chromosome1-2100690	DEL	PRECISE	181	0	0	0/0	0/0	0/1	0/0	0/1	0/0	0/0	0/0	0/0	0/0	0/0	0/0	0/0	0/0
Chromosome1-2102037	DEL	PRECISE	106	0	0	0/0	0/0	0/0	0/0	0/0	0/0	0/0	0/0	0/0	0/0	0/0	0/0	0/0	0/1
Chromosome1-2102258	INS	PRECISE	1029	0	0.957	0/0	0/0	0/0	0/0	0/0	0/0	0/0	0/0	1/1	0/0	1/1	1/1	1/1	0/0
Chromosome1-2102305	DEL	PRECISE	359	0	0	0/0	0/0	0/0	0/0	0/0	0/0	0/0	0/0	0/0	0/0	0/0	0/0	0/0	0/1
Chromosome1-2102861	INS	PRECISE	85	0	0.488	0/0	0/1	0/1	0/1	0/1	0/1	0/0	0/0	0/0	0/1	0/0	0/0	0/0	0/1
Chromosome1-2102879	DEL	PRECISE	5862	0.905	0.905	0/0	0/1	0/1	0/1	0/1	0/1	0/0	0/0	1/1	0/1	1/1	1/1	1/1	1/1
Chromosome1-2104457	INS	PRECISE	60	0	0	0/0	0/0	0/0	0/0	0/0	0/0	0/0	0/0	0/0	0/0	0/0	0/0	0/0	0/1
Chromosome1-2105848	INV	PRECISE	1650776	0	0	0/0	0/0	0/0	0/0	0/0	0/0	0/0	0/0	0/0	0/0	0/0	0/1	0/0	0/0
Chromosome1-2106091	DEL	PRECISE	106	0	0	0/0	0/0	0/0	0/0	0/0	0/1	0/0	0/0	0/0	0/0	0/0	1/1	0/0	0/0
Chromosome1-2108280	DEL	PRECISE	2077	0	0	0/0	1/1	0/1	1/1	1/1	1/1	0/0	0/0	0/0	1/1	0/0	0/0	0/0	1/1
Chromosome1-2108642	DEL	PRECISE	73	0	0	0/0	0/0	0/0	0/0	0/0	0/0	0/0	0/0	0/1	0/0	0/0	0/1	0/0	0/0
Chromosome1-2109143	DEL	PRECISE	109	0	0	0/0	0/0	0/0	0/0	0/0	0/0	0/0	0/0	0/0	0/0	0/1	0/0	1/1	0/0
Chromosome1-2109303	DEL	PRECISE	448	0	0	0/0	0/0	0/0	0/0	0/0	0/0	0/0	0/0	0/0	0/0	0/1	0/0	0/0	0/0
Chromosome1-2109779	BND	PRECISE	0	0	0	0/0	0/0	0/0	0/0	0/0	0/0	0/0	0/0	0/0	0/0	0/0	0/0	0/0	0/1
Chromosome1-2110704	DEL	PRECISE	692	0	0	0/0	0/0	0/0	0/0	0/0	0/0	0/0	0/0	1/1	0/0	1/1	1/1	1/1	0/0
Chromosome1-2113541	INS	PRECISE	51	0.866	0	0/0	0/1	0/0	0/0	0/1	0/1	1/1	1/1	1/1	0/0	1/1	0/1	1/1	0/0
Chromosome1-2113848	INS	PRECISE	706	0	0	0/0	0/0	0/0	0/0	0/0	0/0	0/0	0/0	0/0	0/0	0/0	0/0	0/0	0/1
Chromosome1-2113971	INS	PRECISE	4636	0	0	0/0	0/0	0/0	0/0	0/0	0/0	0/0	0/0	0/0	0/0	0/0	0/0	0/0	0/1
Chromosome1-2114505	INS	PRECISE	227	0	0.488	0/0	0/0	0/0	0/1	0/0	0/0	0/0	0/0	0/0	0/0	0/0	0/0	0/0	0/1
Chromosome1-2114505	INS	PRECISE	5061	0	0	0/0	0/0	0/0	0/0	0/0	0/0	0/0	0/0	0/0	0/0	0/0	1/1	0/0	0/0
Chromosome1-2114627	INS	PRECISE	1696	0	0	0/0	0/0	0/0	0/1	0/0	0/0	0/0	0/0	0/0	0/0	0/0	0/0	0/0	0/0
Chromosome1-2114826	INS	PRECISE	802	0	0	0/0	0/0	0/0	0/0	0/0	0/0	0/0	0/0	0/0	0/0	0/0	0/1	0/0	0/0
Chromosome1-2115318	DEL	PRECISE	86	0.289	0.289	0/1	0/1	0/0	0/0	0/1	0/1	1/1	1/1	1/1	0/1	1/1	1/1	1/1	0/0
Chromosome1-2115376	DEL	PRECISE	1373	0	0	0/0	0/0	0/0	0/0	0/0	0/0	0/0	0/0	0/0	0/0	0/0	0/0	0/0	0/1
Chromosome1-2115554	INS	PRECISE	875	0	0	0/0	0/0	0/0	0/0	0/0	0/0	0/1	0/0	0/0	0/0	0/0	0/0	0/0	0/0
Chromosome1-2117532	DEL	PRECISE	621	0.5	0.5	0/0	0/0	0/0	0/0	0/0	0/0	0/0	0/0	0/1	0/0	0/1	0/1	0/1	0/0
Chromosome1-2117958	INS	PRECISE	1868	0	0	0/0	0/0	0/0	0/0	0/0	0/0	0/0	0/0	0/0	0/0	0/0	0/1	0/0	0/0
Chromosome1-2120288	INS	PRECISE	227	0	0.378	0/0	0/1	0/1	0/1	0/1	0/1	0/0	0/0	0/0	0/1	0/0	0/0	0/0	0/1
Chromosome1-2120443	INS	PRECISE	70	0	0	0/0	0/1	0/1	0/1	0/1	0/1	0/0	0/0	0/1	0/1	0/1	0/1	0/1	1/1
Chromosome1-2120775	DEL	PRECISE	64	0	0	0/0	0/0	0/0	0/0	0/0	0/0	0/0	0/0	0/1	0/0	0/1	0/1	0/1	0/0
Chromosome1-2121559	INS	PRECISE	106	0	0	0/0	0/0	0/0	0/0	0/0	0/0	0/1	0/0	0/0	0/0	0/0	0/0	0/0	0/0
Chromosome1-2122125	INS	PRECISE	211	0	0.488	0/0	0/1	0/0	0/0	0/1	0/1	0/0	0/0	0/0	0/1	0/0	0/0	0/0	0/1
Chromosome1-2122629	BND	PRECISE	0	0	0	0/0	0/0	0/0	0/0	0/0	0/0	0/0	0/0	0/1	0/0	0/0	0/0	0/0	0/0
Chromosome1-2123192	INS	PRECISE	212	0	0	0/0	0/0	0/0	0/0	0/0	0/0	0/0	0/0	0/0	0/0	0/0	0/0	0/0	0/1
Chromosome1-2123444	INS	PRECISE	12040	0	0	0/0	0/0	0/0	0/0	0/0	0/0	0/0	0/0	0/0	0/0	0/0	0/0	0/0	0/1
Chromosome1-2123444	BND	PRECISE	0	0	0	0/0	0/0	0/0	0/0	0/0	0/0	0/0	0/0	0/0	0/0	0/0	0/0	0/0	0/1
Chromosome1-2123616	INS	PRECISE	12582	0	0	0/0	0/1	0/0	0/0	0/0	0/0	0/0	0/0	0/0	0/0	0/0	0/0	0/0	0/0
Chromosome1-2124088	DEL	PRECISE	72	0	0	0/0	0/0	0/0	0/0	0/0	0/0	0/0	0/0	0/0	0/0	0/0	0/0	0/0	0/1
Chromosome1-2124911	BND	PRECISE	0	0	0	0/0	0/0	0/0	0/0	0/0	0/0	0/1	0/0	0/0	0/0	0/0	0/0	0/0	0/0
Chromosome1-2124912	BND	PRECISE	0	0	0	0/0	0/0	0/0	0/0	0/0	0/0	0/1	0/0	0/0	0/0	0/0	0/0	0/0	0/0
Chromosome1-2125110	INS	PRECISE	1253	0	0	0/0	0/0	0/0	0/0	0/0	0/0	0/0	0/0	0/0	0/0	0/0	0/0	0/0	0/1
Chromosome1-2125356	INS	PRECISE	162	0	0.378	0/0	0/1	0/1	0/1	0/1	0/1	0/0	0/0	0/0	0/1	0/0	0/0	0/0	0/1
Chromosome1-2125608	INS	PRECISE	905	0	0.916	0/1	0/1	0/0	0/0	0/0	0/0	0/1	1/1	0/0	0/1	0/1	0/1	0/1	0/0
Chromosome1-2125828	INS	PRECISE	87	0	0.577	0/0	0/0	0/0	0/0	0/0	0/0	0/0	0/0	0/1	0/0	0/1	0/1	0/1	0/0
Chromosome1-2126612	DEL	PRECISE	132	0	0	0/0	0/1	0/1	0/1	0/1	0/1	0/1	0/0	0/1	0/1	0/1	0/1	0/1	1/1
Chromosome1-2126667	DEL	PRECISE	5790	0.447	0	0/0	0/0	0/0	0/0	0/0	0/0	0/0	0/0	0/1	0/0	0/1	0/1	1/1	0/1

Chromosome1-2127458	INS	PRECISE	5246	0	0	0/0	0/0	0/0	0/0	0/0	0/0	0/0	0/0	0/0	0/0	0/0	0/0	0/0	1/1
Chromosome1-2127507	DEL	PRECISE	81	0	0	0/0	0/1	0/1	0/1	0/1	0/0	0/0	0/0	0/0	0/1	0/0	0/0	0/0	0/1
Chromosome1-2127738	DEL	PRECISE	3522	0	0	0/0	0/0	0/0	0/0	0/0	0/0	0/0	0/0	0/0	0/0	0/1	0/1	0/1	0/1
Chromosome1-2128072	INS	PRECISE	53	0	0.426	0/1	1/1	0/1	1/1	1/1	1/1	1/1	1/1	1/1	1/1	0/1	0/1	0/1	0/1
Chromosome1-2128791	INS	PRECISE	1200	0	0.816	0/0	0/0	0/0	0/0	0/0	0/0	0/0	0/0	0/1	0/0	0/1	0/1	0/1	0/0
Chromosome1-2128890	INS	PRECISE	276	0	0	0/0	0/0	0/0	0/0	0/0	0/0	0/0	0/0	0/0	0/0	0/0	0/0	0/0	0/1
Chromosome1-2130089	INS	PRECISE	1203	0	0	0/0	0/0	0/0	0/1	0/1	0/1	1/1	0/0	0/1	0/0	0/0	0/0	0/0	0/0
Chromosome1-2130484	DEL	PRECISE	478	0	0.378	0/0	0/1	0/1	0/1	0/1	0/1	0/0	0/0	0/0	0/1	0/0	0/0	0/0	0/1
Chromosome1-2130803	DEL	PRECISE	81	0	0	0/0	0/0	0/0	0/0	0/0	0/0	0/0	0/0	0/1	0/0	0/0	0/1	0/1	0/0
Chromosome1-2133034	INS	PRECISE	84	0	0.378	0/0	0/1	0/1	0/1	0/1	0/1	0/0	0/0	0/0	0/1	0/0	0/0	0/0	0/1
Chromosome1-2133079	BND	PRECISE	0	0	0	0/0	0/0	0/0	0/0	0/0	0/0	0/0	1/1	0/0	0/0	0/1	0/0	0/0	0/0
Chromosome1-2133080	BND	PRECISE	0	0	0	0/1	0/1	0/0	0/0	0/0	0/0	0/0	0/1	0/0	0/0	0/1	0/1	0/0	0/0
Chromosome1-2133562	INS	PRECISE	146	0	0	0/0	0/0	0/0	0/0	0/0	0/0	0/0	0/0	0/0	0/0	0/0	0/0	0/0	0/1
Chromosome1-2133857	INS	PRECISE	59	0	0	0/0	0/0	0/0	0/0	0/0	0/0	0/0	0/0	1/1	0/0	0/0	0/0	0/0	0/0
Chromosome1-2135112	BND	PRECISE	0	0.5	0	0/1	1/1	0/0	1/1	0/0	0/0	0/1	1/1	0/0	1/1	1/1	1/1	1/1	0/0
Chromosome1-2135112	BND	PRECISE	0	0	0	0/0	0/0	0/0	0/0	0/0	0/0	0/0	0/1	0/0	0/0	0/0	0/0	0/1	0/0
Chromosome1-2135564	DEL	PRECISE	92	0	0	0/0	0/0	0/0	0/0	1/1	1/1	0/0	0/0	1/1	0/0	0/0	0/0	0/0	0/0
Chromosome1-2136762	INS	PRECISE	63	0	0	0/0	0/0	0/0	0/0	0/0	0/0	0/0	0/0	0/1	0/0	0/0	0/0	0/0	0/0
Chromosome1-2137477	DEL	PRECISE	67	0.333	0.333	0/1	1/1	0/0	0/1	0/0	0/0	0/1	1/1	0/0	0/1	0/1	1/1	0/1	0/0
Chromosome1-2137714	INS	PRECISE	1047	0	0	0/0	0/0	0/0	0/0	0/0	0/0	0/0	0/0	0/0	0/0	0/0	0/0	0/0	0/1
Chromosome1-2138284	INS	PRECISE	3470	0	0	0/0	0/0	0/0	0/0	0/0	0/0	0/0	0/0	0/1	0/0	0/0	0/0	0/0	0/0
Chromosome1-2140221	INS	PRECISE	50	0	0	0/1	1/1	0/0	1/1	1/1	1/1	1/1	1/1	1/1	1/1	1/1	1/1	1/1	1/1
Chromosome1-2141331	DEL	PRECISE	272	0	0	0/0	0/0	0/0	0/0	0/0	0/0	0/0	0/0	0/0	0/0	0/1	0/1	0/1	0/0
Chromosome1-2141547	BND	PRECISE	0	0	0	0/0	0/0	0/0	0/1	0/0	0/0	0/0	0/0	0/0	0/0	0/0	0/0	0/0	0/0
Chromosome1-2142745	DEL	PRECISE	53	0.378	0.378	0/0	0/1	0/1	0/1	0/1	0/1	0/0	0/0	0/0	0/1	0/0	0/0	0/0	0/1
Chromosome1-2142973	DEL	PRECISE	1986	0	0	0/0	0/0	0/0	0/0	0/1	0/1	0/0	0/0	0/1	0/0	0/0	0/0	0/0	0/0
Chromosome1-2143413	DEL	PRECISE	50	0	0	0/0	0/1	0/1	0/1	0/1	0/1	0/0	0/0	0/0	0/1	0/0	0/0	0/0	0/1
Chromosome1-2143586	BND	PRECISE	0	0	0	0/0	0/0	0/0	0/0	0/0	0/0	0/0	0/0	0/1	0/0	0/0	0/0	0/0	0/0
Chromosome1-2143586	BND	PRECISE	0	0	0	0/0	0/0	0/0	0/0	0/0	0/1	0/0	0/0	0/1	0/0	0/0	0/0	0/0	0/0
Chromosome1-2143790	DEL	PRECISE	4843	0	0	0/0	0/1	0/1	0/1	0/1	0/1	0/0	0/0	0/0	0/1	0/0	0/0	0/0	0/1
Chromosome1-2143804	INS	PRECISE	72	0	0	0/0	0/0	0/0	0/0	0/0	0/0	0/1	0/0	0/0	0/0	0/0	0/0	0/0	0/0
Chromosome1-2144656	DEL	PRECISE	324	0	0	0/0	0/1	0/1	0/1	0/1	0/1	0/0	0/0	0/0	0/1	0/0	0/0	0/0	0/1
Chromosome1-2145538	INS	PRECISE	975	0	0.787	0/0	0/1	0/1	0/1	0/1	1/1	0/0	0/0	0/0	1/1	0/0	0/0	0/0	1/1
Chromosome1-2146129	DEL	PRECISE	4018007	0	0	0/0	0/0	0/0	1/1	0/1	0/0	0/0	0/0	0/0	0/1	0/0	0/0	0/0	0/1
Chromosome1-2146411	INS	PRECISE	5023	0	0	0/0	0/0	0/0	0/0	0/0	0/0	0/0	0/0	0/0	0/0	0/0	0/0	0/0	1/1
Chromosome1-2146589	DEL	PRECISE	1998	0	0	0/0	0/1	0/1	0/1	1/1	1/1	0/0	0/0	1/1	0/1	0/0	0/0	0/0	1/1
Chromosome1-2146886	DEL	PRECISE	1196	0	0	0/0	0/1	0/1	0/1	1/1	1/1	0/0	0/0	1/1	0/1	0/0	0/0	0/0	1/1
Chromosome1-2147030	INS	PRECISE	5265	0	0	0/0	0/0	0/0	0/0	0/0	0/0	0/0	0/0	0/0	0/0	0/0	0/0	0/0	0/1
Chromosome1-2147150	DEL	PRECISE	276	0	0	0/0	0/1	0/1	0/1	1/1	1/1	0/0	0/0	1/1	0/1	0/0	0/0	0/0	1/1
Chromosome1-2147638	INS	PRECISE	51	0	0	0/1	1/1	0/1	1/1	1/1	0/1	1/1	1/1	1/1	1/1	1/1	1/1	1/1	1/1
Chromosome1-2148122	BND	PRECISE	0	0	0	0/0	1/1	0/1	1/1	0/1	0/1	0/1	1/1	0/0	1/1	1/1	0/1	0/1	0/1
Chromosome1-2148299	INS	PRECISE	137	0	0	0/0	0/0	0/0	0/0	0/0	0/0	0/0	0/0	0/0	0/0	0/0	0/1	0/1	0/0
Chromosome1-2149037	DEL	PRECISE	92147	0	0	0/0	1/1	1/1	1/1	0/1	1/1	0/0	0/0	0/0	1/1	0/0	0/0	0/0	1/1
Chromosome1-2151245	DEL	PRECISE	53	0	0	0/0	0/0	0/0	0/0	0/0	0/0	0/0	0/0	0/0	0/0	0/0	0/0	0/0	1/1
Chromosome1-2151937	DEL	PRECISE	62	0	0	0/1	1/1	0/0	1/1	0/0	0/0	1/1	1/1	0/0	1/1	1/1	1/1	1/1	0/0
Chromosome1-2152570	INS	PRECISE	58	0	0	0/1	1/1	0/0	1/1	1/1	1/1	1/1	1/1	1/1	1/1	1/1	1/1	1/1	1/1
Chromosome1-2155010	DEL	PRECISE	97	0	0	0/0	0/0	0/0	0/0	0/0	0/0	0/0	0/0	0/0	0/0	0/0	0/0	0/0	1/1
Chromosome1-2155032	INS	PRECISE	230	0	0.5	0/0	0/0	0/0	0/0	0/0	0/0	0/0	0/0	0/1	0/0	0/1	0/0	0/1	0/0
Chromosome1-2156053	BND	PRECISE	0	0	0	0/0	0/0	0/0	0/0	1/1	0/1	0/0	0/0	0/1	0/0	0/0	0/0	0/0	0/0
Chromosome1-2156159	DEL	PRECISE	533	0	0	0/0	0/0	0/0	0/0	1/1	1/1	0/0	0/0	0/1	0/0	0/0	0/0	0/0	1/1
Chromosome1-2156212	INS	PRECISE	508	0	0.707	0/1	1/1	0/0	1/1	0/0	0/0	1/1	1/1	0/0	1/1	0/1	0/1	0/1	0/0
Chromosome1-2156360	INS	PRECISE	14326	0	0	0/0	0/0	0/0	0/0	0/0	0/0	0/0	0/0	0/0	0/0	0/0	0/0	0/0	0/0
Chromosome1-2156438	DEL	PRECISE	59	0	0	0/0	0/0	0/0	0/0	0/0	0/0	0/0	0/0	0/0	0/0	0/1	0/1	0/1	0/0
Chromosome1-2156987	DEL	PRECISE	323	0	0	0/1	1/1	0/0	1/1	1/1	1/1	1/1	1/1	1/1	1/1	1/1	1/1	1/1	1/1
Chromosome1-2157109	BND	PRECISE	0	0	0	0/0	0/0	0/0	0/0	0/0	0/0	0/0	0/0	0/0	0/0	0/0	0/0	0/1	0/0
Chromosome1-2157578	INS	PRECISE	71	0	0.577	0/0	0/0	0/0	0/0	0/0	0/0	0/0	0/0	0/0	0/0	0/1	0/1	0/0	0/0
Chromosome1-2157736	BND	PRECISE	0	0	0	0/0	0/0	0/0	0/0	0/0	0/0	0/0	0/0	0/0	0/0	0/0	0/0	0/0	1/1
Chromosome1-2157737	BND	PRECISE	0	0	0	0/0	0/0	0/0	0/0	0/0	0/0	0/0	0/0	0/0	0/0	0/1	0/0	0/1	0/0
Chromosome1-2157737	BND	PRECISE	0	0	0	0/0	0/0	0/0	0/0	0/0	0/0	0/0	0/0	0/1	0/0	0/0	0/0	0/0	0/0
Chromosome1-2157737	BND	PRECISE	0	0	0	0/0	0/0	0/0	0/0	0/0	0/0	0/0	0/0	0/0	0/0	0/0	0/0	0/0	1/1
Chromosome1-2157738	INS	PRECISE	16717	0	0	0/0	0/0	0/0	0/0	0/0	0/0	0/0	0/0	0/1	0/0	0/0	0/0	0/0	0/0
Chromosome1-2158102	DEL	PRECISE	84	0	0	0/0	0/0	0/0	0/0	0/0	0/0	0/0	0/0	0/1	0/0	0/1	0/1	0/1	0/0
Chromosome1-2160598	BND	PRECISE	0	0	0	0/0	0/0	0/0	0/0	0/1	0/0	0/0	0/0	0/0	0/0	0/0	0/0	0/0	0/0
Chromosome1-2160685	INS	PRECISE	97	0	0.816	0/0	0/0	0/0	0/0	0/0	0/0	0/0	0/0	0/1	0/0	1/1	1/1	1/1	0/0
Chromosome1-2160898	INS	PRECISE	1185	0	0.577	0/0	1/1	0/1	1/1	0/1	0/1	0/0	0/0	0/0	1/1	0/0	0/0	0/0	1/1
Chromosome1-2161278	INS	PRECISE	869	0	0	0/0	0/0	0/0	0/0	0/0	0/0	0/1	0/0	0/0	0/0	0/0	0/0	0/0	0/0
Chromosome1-2161460	INS	PRECISE	178	0	0.707	0/0	0/0	0/0	0/0	0/0	0/0	0/1	0/0	0/0	0/0	0/0	0/0	0/0	0/1
Chromosome1-2163090	DEL	PRECISE	57	0	0	0/0	0/0	0/0	0/0	0/0	0/0	0/0	0/0	0/1	0/0	0/0	0/0	0/0	0/0
Chromosome1-2163216	INS	PRECISE	63	0	0	0/0	0/0	0/0	0/0	0/0	0/0	0/0	0/0	0/1	0/0	0/0	0/0	0/0	0/0
Chromosome1-2163557	DEL	PRECISE	86	0	0	0/0	0/1	0/1	0/1	0/1	1/1	0/1	0/0	0/0	0/1	0/0	0/0	0/0	0/1
Chromosome1-2163735	DEL	PRECISE	165	0	0	0/1	0/1	0/0	0/1	0/0	0/0	0/1	1/1	0/0	0/1	0/1	0/1	0/1	0/0
Chromosome1-2164062	INS	PRECISE	899	0	0	0/0	0/0	0/0	0/0	0/0	0/0	0/0	0/0	0/0	0/0	0/0	0/0	0/0	0/1
Chromosome1-2164343	INS	PRECISE	161	0	0.707	0/0	0/0	0/0	0/0	0/0	0/0	0/0	0/0	0/0	0/0	0/0	0/0	0/1	0/0

Chromosome1-2164696	INS	PRECISE	127	0	0	0/0	0/0	0/0	0/1	0/0	0/0	0/0	0/0	0/0	0/0	0/0	0/0	0/0	0/0
Chromosome1-2165090	DEL	PRECISE	279	0	0	0/0	0/0	0/0	0/0	0/0	0/0	0/0	0/0	0/0	0/0	0/0	0/0	0/0	0/1
Chromosome1-2165329	DEL	PRECISE	1304	0	0	0/0	0/0	0/0	0/0	0/0	0/0	0/0	0/0	0/1	0/0	0/1	0/1	0/1	0/0
Chromosome1-2165409	INS	PRECISE	66	0	0	0/0	0/0	0/0	0/0	0/0	0/0	0/0	0/0	0/0	0/0	0/0	0/0	0/0	0/1
Chromosome1-2166341	DEL	PRECISE	220	0.267	0.267	0/1	1/1	0/1	1/1	1/1	1/1	1/1	1/1	0/1	1/1	1/1	1/1	1/1	0/1
Chromosome1-2166444	INS	PRECISE	49	0	0.378	0/0	0/1	0/1	0/0	0/1	0/1	0/0	0/0	0/0	0/1	0/0	0/0	0/0	0/1
Chromosome1-2166546	DEL	PRECISE	1122	0	0	0/0	0/0	0/0	0/0	0/0	0/0	0/0	0/0	0/1	0/0	0/1	0/0	0/1	0/0
Chromosome1-2166565	INS	PRECISE	1147	0	0	0/0	0/0	0/0	0/0	0/0	0/0	0/1	0/0	0/0	0/0	0/0	0/0	0/0	0/0
Chromosome1-2167887	INS	PRECISE	1215	0	0	0/0	0/0	0/0	0/0	0/0	0/0	1/1	0/0	0/0	0/0	0/0	0/0	0/0	0/0
Chromosome1-2167987	DEL	PRECISE	81	0	0	0/0	0/0	0/0	0/0	0/0	0/0	1/1	0/0	0/0	0/0	0/0	0/0	0/0	0/0
Chromosome1-2168095	DUP	PRECISE	1290	0	0	0/1	0/0	0/0	0/0	0/1	0/0	0/0	0/1	0/1	0/1	0/1	0/1	0/1	0/0
Chromosome1-2168115	INV	PRECISE	30029	0	0	0/0	0/0	0/0	0/0	0/1	0/1	0/0	0/0	0/0	0/1	0/0	0/0	0/0	0/0
Chromosome1-2168352	DEL	PRECISE	87	0	0	0/0	0/0	0/0	0/0	0/0	0/0	0/0	0/0	0/1	0/0	0/1	0/1	0/0	0/0
Chromosome1-2168717	DEL	PRECISE	82	0	0	0/0	0/0	0/0	0/0	0/0	0/0	0/0	0/0	0/1	0/0	0/1	0/1	0/0	0/0
Chromosome1-2169293	DEL	PRECISE	572	0	0	0/0	0/0	0/0	0/0	0/0	0/0	0/0	0/0	0/0	0/0	0/0	0/0	0/0	0/1
Chromosome1-2170932	INS	PRECISE	4981	0	0	0/0	0/0	0/0	0/0	0/0	0/0	0/0	0/0	0/0	0/0	0/0	0/0	0/0	0/1
Chromosome1-2171170	DEL	PRECISE	9282	0.316	0	0/0	0/0	0/0	0/0	0/1	0/1	0/0	0/0	0/0	0/1	0/0	0/0	0/0	0/0
Chromosome1-2171220	INS	PRECISE	493	0	0	0/0	0/0	0/0	0/0	0/0	0/0	0/0	0/0	0/0	0/0	0/0	0/0	0/0	0/1
Chromosome1-2171359	INS	PRECISE	505	0	0	0/0	0/0	0/0	0/0	0/0	0/0	0/0	0/0	0/0	0/0	0/0	0/0	0/0	0/1
Chromosome1-2171868	DEL	PRECISE	2015	0	0	0/0	0/0	0/0	0/0	0/0	0/0	0/0	0/0	0/0	0/0	0/0	0/0	0/0	0/1
Chromosome1-2172133	DEL	PRECISE	72	0	0	0/0	0/0	0/0	0/0	0/0	0/0	1/1	0/0	0/0	0/0	0/0	0/0	0/0	0/0
Chromosome1-2172194	INS	PRECISE	9986	0	0	0/0	0/0	0/0	0/0	0/0	0/0	1/1	0/0	0/0	0/0	0/0	0/0	0/0	0/0
Chromosome1-2172196	INS	PRECISE	69	0	0	0/0	0/0	0/0	0/0	0/0	0/0	0/0	0/0	0/0	0/0	0/0	0/0	0/0	0/1
Chromosome1-2172204	BND	PRECISE	0	0	0	0/0	0/0	0/0	0/0	0/0	0/0	0/1	0/0	0/0	0/0	0/0	0/0	0/0	0/0
Chromosome1-2172385	DEL	PRECISE	3846	0	0	0/0	0/0	0/0	0/0	0/0	0/0	1/1	0/0	0/0	0/0	0/0	0/0	0/0	0/0
Chromosome1-2172705	INS	PRECISE	11856	0	0	0/0	0/0	0/0	0/0	0/0	0/0	0/0	0/0	0/0	0/0	0/0	0/0	0/0	0/1
Chromosome1-2173423	INS	PRECISE	124	0	0	0/0	0/0	0/0	0/0	0/0	0/0	1/1	0/0	0/0	0/0	0/0	0/0	0/0	0/1
Chromosome1-2173823	DEL	PRECISE	641	0	0	0/0	0/0	0/0	0/0	0/0	0/0	1/1	0/0	0/0	0/0	0/0	0/0	0/0	0/1
Chromosome1-2175269	INV	PRECISE	248865	0	0	0/0	0/0	0/0	0/0	0/0	0/0	0/0	0/0	0/0	0/1	0/0	0/0	0/0	0/0
Chromosome1-2175690	INS	PRECISE	5463	0	0	0/0	0/0	0/0	0/0	0/0	0/0	0/0	0/0	0/0	0/0	0/0	0/0	0/0	0/1
Chromosome1-2175723	DEL	PRECISE	1117	0.5	0.5	0/0	0/0	0/0	0/0	0/0	0/0	0/0	0/0	1/1	0/0	1/1	1/1	1/1	0/0
Chromosome1-2175930	DEL	PRECISE	63	0	0	0/0	0/0	0/0	0/0	0/0	0/0	0/0	0/0	1/1	0/0	1/1	0/0	1/1	0/0
Chromosome1-2176122	INS	PRECISE	248	0	0.5	0/0	0/0	0/0	0/0	0/0	0/0	0/0	0/0	1/1	0/0	1/1	1/1	1/1	0/0
Chromosome1-2177047	INS	PRECISE	968	0	0	0/0	0/0	0/0	0/0	0/0	0/0	1/1	0/0	0/0	0/0	0/0	0/0	0/0	0/0
Chromosome1-2177092	INS	PRECISE	89	0	0.289	0/1	0/1	0/0	0/1	0/1	0/1	0/1	1/1	1/1	0/1	1/1	1/1	1/1	0/0
Chromosome1-2177320	INS	PRECISE	211	0	0.622	0/1	0/1	0/0	0/1	0/1	0/1	0/1	1/1	0/1	0/1	0/1	0/0	0/1	0/0
Chromosome1-2177545	DEL	PRECISE	382	0	0	0/1	0/1	0/0	0/1	0/1	0/1	1/1	1/1	1/1	0/1	1/1	1/1	1/1	0/1
Chromosome1-2177623	DEL	PRECISE	631	0	0	0/1	0/1	0/0	0/1	0/1	0/1	0/1	1/1	1/1	0/1	1/1	1/1	1/1	0/1
Chromosome1-2178553	INS	PRECISE	461	0	0	0/0	0/0	0/0	0/0	0/0	0/0	0/0	0/0	0/0	0/0	0/0	0/0	0/0	0/1
Chromosome1-2178584	DEL	PRECISE	169	0	0	0/0	0/0	0/0	0/0	0/0	0/0	0/1	0/0	0/0	0/0	0/0	0/0	0/0	0/0
Chromosome1-2178766	INS	PRECISE	308	0	0	0/0	0/0	0/0	0/0	0/0	0/0	0/0	0/0	0/1	0/0	0/1	0/0	0/1	0/0
Chromosome1-2180177	INS	PRECISE	197	0	0.707	0/0	0/0	0/0	0/0	0/0	0/0	0/0	0/0	0/1	0/0	0/0	0/1	0/0	0/0
Chromosome1-2180367	INS	PRECISE	11287	0	0	0/0	0/0	0/0	0/0	0/0	0/0	0/1	0/0	0/0	0/0	0/0	0/0	0/0	0/0
Chromosome1-2180399	INS	PRECISE	50	0	0	0/0	0/0	0/0	0/1	0/1	0/1	0/0	0/0	0/1	0/0	0/0	0/0	0/0	0/1
Chromosome1-2180439	DEL	PRECISE	75	0	0	0/0	0/1	0/1	0/1	0/1	0/1	0/0	0/0	0/0	0/1	0/0	0/0	0/0	0/1
Chromosome1-2180723	INS	PRECISE	220	0	0.707	0/0	0/0	0/0	0/0	0/0	0/0	0/0	0/0	0/1	0/0	0/0	0/1	0/0	0/0
Chromosome1-2180891	INS	PRECISE	1095	0	0	0/0	0/0	0/0	0/0	0/0	0/0	0/1	0/0	0/0	0/0	0/0	0/0	0/0	0/0
Chromosome1-2182211	INS	PRECISE	211	0	0	0/0	0/1	0/1	0/1	0/1	1/1	0/0	0/0	0/0	0/1	0/0	0/0	0/0	1/1
Chromosome1-2182447	DEL	PRECISE	916	0	0	0/0	0/0	0/0	0/0	0/0	0/0	0/0	0/0	0/0	0/0	0/0	0/0	0/1	0/0
Chromosome1-2182576	DEL	PRECISE	1445	0	0	0/1	1/1	0/1	1/1	1/1	1/1	1/1	1/1	1/1	1/1	1/1	1/1	1/1	1/1
Chromosome1-2182999	DEL	PRECISE	1757	0	0	0/0	0/0	0/0	0/0	0/0	0/0	0/0	0/0	0/0	0/0	0/1	0/0	0/1	0/0
Chromosome1-2183229	INS	PRECISE	5626	0	0	0/0	0/0	0/0	0/1	0/0	0/0	0/0	0/0	0/0	0/0	0/0	0/0	0/0	0/0
Chromosome1-2183499	DEL	PRECISE	235	0	0	0/1	0/1	0/0	0/0	0/0	0/0	0/0	1/1	0/0	0/1	0/1	0/1	0/1	0/0
Chromosome1-2183635	DEL	PRECISE	8009	0	0	0/0	0/0	0/0	0/0	0/0	0/0	0/0	0/0	0/1	0/0	0/0	0/0	0/0	0/0
Chromosome1-2184320	DEL	PRECISE	5033	0	0	0/0	0/0	0/0	0/0	0/0	0/0	0/0	0/0	0/0	0/0	0/0	0/0	0/0	0/1
Chromosome1-2184332	DEL	PRECISE	92	0	0	0/0	0/0	0/0	0/1	0/0	0/0	0/0	1/1	0/0	0/0	0/0	0/0	0/0	0/0
Chromosome1-2184433	INS	PRECISE	52	0	0.378	0/0	0/0	0/0	0/1	0/0	0/0	0/0	1/1	0/0	0/0	0/0	0/0	0/0	0/0
Chromosome1-2184602	DEL	PRECISE	1315	0	0	0/0	0/0	0/0	0/0	0/0	0/0	0/0	0/0	0/0	0/1	0/1	0/0	0/0	0/0
Chromosome1-2185549	INS	PRECISE	63	0	0	0/0	0/0	0/0	0/0	0/0	0/0	0/0	0/0	0/0	0/0	0/0	0/0	0/0	0/1
Chromosome1-2186074	DEL	PRECISE	3593	0	0	0/0	0/0	0/0	0/0	0/0	0/0	0/0	0/0	0/0	0/0	0/0	1/1	0/0	0/0
Chromosome1-2186153	DEL	PRECISE	1549	0	0	0/0	0/0	0/0	0/0	0/0	0/0	0/0	0/0	0/0	0/0	0/0	1/1	1/1	0/0
Chromosome1-2186347	DEL	PRECISE	54	0	0	0/1	0/1	0/0	0/1	0/0	0/0	0/1	1/1	0/0	0/1	0/1	1/1	0/1	0/0
Chromosome1-2186761	INS	PRECISE	150	0	0.707	0/0	0/0	0/0	0/0	0/0	0/0	0/0	0/0	0/0	0/0	0/1	0/0	0/0	0/0
Chromosome1-2187154	DEL	PRECISE	95	0	0	0/0	0/0	0/0	0/0	0/0	0/0	0/0	0/0	0/0	0/0	0/1	0/0	0/0	0/0
Chromosome1-2187264	DEL	PRECISE	1720	0	0	0/0	0/0	0/0	0/0	0/0	0/0	0/0	0/0	0/0	0/0	0/1	0/0	0/0	0/0
Chromosome1-2187271	DEL	PRECISE	52	0	0	0/0	0/0	0/0	0/0	0/0	0/0	0/0	0/0	0/1	0/0	0/1	0/1	0/1	0/0
Chromosome1-2187454	INS	PRECISE	9845	0	0	0/0	0/0	0/0	0/0	0/0	0/0	0/0	0/0	0/0	0/0	0/0	0/0	0/0	1/1
Chromosome1-2187911	DEL	PRECISE	79	0	0	0/0	0/0	0/0	0/0	0/0	0/0	0/0	0/0	0/0	0/0	0/1	0/0	0/1	0/0
Chromosome1-2188440	INS	PRECISE	4061	0	0	0/0	0/0	0/0	0/0	0/0	0/0	0/0	0/0	0/0	0/0	0/0	0/0	0/0	1/1
Chromosome1-2189689	DEL	PRECISE	249	0	0	0/1	0/1	0/0	0/1	0/0	0/0	0/1	1/1	0/0	0/1	1/1	1/1	1/1	0/0
Chromosome1-2190326	BND	PRECISE	0	0	0	0/0	0/0	0/0	0/0	0/0	0/0	0/0	0/0	0/0	0/0	0/1	0/0	0/1	0/0
Chromosome1-2190768	DEL	PRECISE	621	0	0	0/0	0/0	0/0	0/0	0/0	0/0	0/0	0/0	0/0	0/0	0/0	0/0	0/0	0/1
Chromosome1-2191121	DEL	PRECISE	1202	0	0	0/0	0/0	0/0	0/0	0/0	0/0	0/0	0/0	0/0	0/0	0/0	0/0	0/0	0/1

Chromosome1-2191244 DEL	PRECISE	8212	0	0	0/0	0/0	0/0	0/0	0/0	0/0	0/0	0/0	0/0	0/0	0/0	0/0	0/0	0/1
Chromosome1-2192184 DEL	PRECISE	392	0	0	0/0	0/0	0/0	0/0	0/0	0/0	0/0	0/0	0/0	0/0	0/0	0/0	0/0	0/1
Chromosome1-2192953 DEL	PRECISE	356	0	0	0/1	0/1	0/0	0/1	0/1	0/1	1/1	1/1	1/1	0/1	1/1	1/1	1/1	0/0
Chromosome1-2193718 INS	PRECISE	831	0	0	0/0	0/1	0/0	0/0	0/0	0/0	0/0	0/0	0/0	0/0	0/0	0/0	0/0	0/0
Chromosome1-2196126 DEL	PRECISE	150	0	0	0/1	0/1	0/0	0/1	0/1	0/1	1/1	1/1	1/1	0/1	1/1	1/1	1/1	0/0
Chromosome1-2196316 DEL	PRECISE	650	0	0	0/1	1/1	0/1	1/1	1/1	1/1	1/1	1/1	1/1	1/1	1/1	1/1	1/1	1/1
Chromosome1-2198345 DEL	PRECISE	62	0	0	0/1	0/1	0/0	0/1	0/1	0/1	1/1	1/1	1/1	0/1	1/1	1/1	1/1	0/0
Chromosome1-2198599 INS	PRECISE	134	0	0.378	0/0	0/1	0/1	0/1	0/1	0/1	0/0	0/0	0/0	0/1	0/0	0/0	0/0	1/1
Chromosome1-2199562 DEL	PRECISE	71	0	0	0/0	0/0	0/0	0/0	0/0	0/0	0/0	0/0	0/0	0/0	0/1	0/0	0/1	0/0
Chromosome1-2200202 INS	PRECISE	486	0	0.663	0/1	1/1	0/0	1/1	1/1	1/1	0/1	1/1	1/1	1/1	1/1	1/1	1/1	1/1
Chromosome1-2200317 DEL	PRECISE	356	0	0	0/0	0/0	0/0	0/0	0/0	0/0	0/0	0/0	0/0	0/0	0/0	0/0	0/0	0/1
Chromosome1-2201888 BND	PRECISE	0	0	0	0/0	0/0	0/0	0/0	0/0	0/0	0/0	0/0	1/1	0/0	0/0	0/1	0/1	0/0
Chromosome1-2202356 BND	PRECISE	0	0	0	0/0	0/0	0/0	0/0	0/0	0/0	0/0	0/0	0/0	0/0	0/1	0/0	0/0	0/0
Chromosome1-2204482 INS	PRECISE	151	0	0.707	0/0	0/0	0/0	0/0	0/0	0/0	0/0	0/0	0/0	0/0	0/1	0/0	0/1	0/0
Chromosome1-2204734 INS	PRECISE	66	0	0	0/0	0/0	0/0	0/0	0/0	0/0	0/1	0/0	0/0	0/0	0/0	0/0	0/0	0/0
Chromosome1-2204755 DEL	PRECISE	125	0	0	0/0	0/0	0/0	0/0	0/0	0/0	0/0	0/0	0/1	0/0	0/0	0/1	0/1	0/0
Chromosome1-2204950 DEL	PRECISE	62	0	0	0/0	0/0	0/0	0/0	0/0	0/0	0/0	0/0	0/1	0/0	0/0	0/1	0/1	0/0
Chromosome1-2205193 INS	PRECISE	4501	0	0	0/0	0/0	0/0	0/0	0/0	0/0	0/0	0/0	1/1	0/0	0/0	1/1	0/0	0/0
Chromosome1-2205253 DEL	PRECISE	402	0	0	0/0	0/0	0/0	0/0	0/0	0/0	0/0	0/0	1/1	0/0	0/0	1/1	0/0	0/0
Chromosome1-2205331 BND	PRECISE	0	0	0	0/0	0/0	0/0	0/0	0/1	0/0	0/0	0/0	0/0	0/0	0/0	0/0	0/0	0/0
Chromosome1-2205345 INS	PRECISE	722	0	0.707	0/0	0/0	0/0	0/0	0/0	0/0	0/0	0/0	0/1	0/0	0/0	0/1	0/0	0/0
Chromosome1-2207760 INS	PRECISE	5112	0	0	0/0	0/0	0/0	1/1	0/0	0/0	0/0	0/0	0/0	0/0	0/0	0/0	0/0	0/0
Chromosome1-2209321 INS	PRECISE	1340	0	0	0/0	0/0	0/0	0/1	0/0	0/0	0/0	0/0	0/0	0/0	0/0	0/0	0/0	0/0
Chromosome1-2209542 INS	PRECISE	54	0	0	0/0	0/0	0/0	0/1	0/0	0/0	0/0	0/0	0/0	0/0	0/0	0/0	0/0	0/0
Chromosome1-2209616 INS	PRECISE	12579	0	0	0/0	0/0	0/0	0/0	0/0	0/0	0/1	0/0	0/0	0/0	0/0	0/0	0/0	0/0
Chromosome1-2209616 BND	PRECISE	0	0	0	0/0	0/0	0/0	0/0	0/0	0/0	0/1	0/0	0/0	0/0	0/0	0/0	0/0	0/0
Chromosome1-2209707 DEL	PRECISE	188	0	0	0/0	0/0	0/0	0/0	0/0	0/0	0/0	0/0	0/1	0/0	0/1	0/0	0/1	0/0
Chromosome1-2209772 INS	PRECISE	211	0	0	0/0	0/0	0/0	0/1	0/0	0/0	0/0	0/0	0/0	0/0	0/0	0/0	0/0	0/0
Chromosome1-2210059 INS	PRECISE	132	0	0.707	0/0	0/0	0/0	0/0	0/0	0/0	0/0	0/0	0/0	0/0	0/1	0/0	0/1	0/0
Chromosome1-2210620 INS	PRECISE	125	0	0	0/0	0/0	0/0	0/0	0/0	0/0	0/1	0/0	0/0	0/0	0/0	0/0	0/0	0/0
Chromosome1-2210684 DEL	PRECISE	858	0	0	0/0	0/0	0/0	0/0	0/0	0/0	0/0	0/0	0/1	0/0	0/1	0/1	0/1	0/1
Chromosome1-2210717 DEL	PRECISE	58	0	0	0/0	0/0	0/0	0/0	0/0	0/0	0/0	0/0	0/0	0/0	0/0	0/0	0/0	0/1
Chromosome1-2210840 DEL	PRECISE	72	0	0	0/0	0/0	0/0	0/0	0/0	0/0	0/0	0/0	0/1	0/0	0/1	0/1	0/1	0/1
Chromosome1-2211333 INS	PRECISE	232	0.894	0.707	0/0	0/0	0/0	0/0	0/0	0/0	0/0	0/0	0/1	0/0	0/1	0/1	0/1	0/1
Chromosome1-2211597 DEL	PRECISE	5962	0	0	0/0	0/0	0/0	0/0	0/0	0/0	0/0	0/0	1/1	0/0	0/1	0/0	0/0	0/1
Chromosome1-2211889 INS	PRECISE	2422	0	0	0/0	0/0	0/0	0/0	0/0	0/0	0/1	0/0	0/0	0/0	0/0	0/0	0/0	0/0
Chromosome1-2212148 INS	PRECISE	12154	0	0	0/0	0/0	0/0	0/0	0/0	0/0	1/1	0/0	0/0	0/0	0/0	0/0	0/0	0/0
Chromosome1-2212270 DEL	PRECISE	82	0	0	0/0	0/0	0/0	0/0	0/0	0/0	0/1	0/0	0/0	0/0	0/0	0/0	0/0	0/0
Chromosome1-2213210 DUP	PRECISE	10101	0	0	0/0	0/0	0/0	0/0	0/0	0/0	0/0	0/0	0/0	0/0	0/0	0/0	0/0	0/1
Chromosome1-2213602 DEL	PRECISE	323	0	0	0/0	0/0	0/0	0/0	0/0	0/0	0/0	0/0	0/0	0/0	0/0	0/0	0/0	0/1
Chromosome1-2215013 INS	PRECISE	60	0	0	0/0	0/0	0/0	0/0	0/0	0/0	0/0	0/0	0/1	0/0	0/0	0/0	0/0	0/0
Chromosome1-2215107 BND	PRECISE	0	0	0	0/0	0/0	0/0	0/0	0/0	0/0	0/0	0/0	0/1	0/0	0/0	0/0	0/0	0/0
Chromosome1-2215423 DEL	PRECISE	109	0	0	0/1	0/1	0/0	0/0	0/0	0/0	0/1	1/1	0/0	0/1	1/1	1/1	1/1	1/1
Chromosome1-2216026 DUP	PRECISE	10762	0	0	0/0	0/0	0/0	0/0	0/0	0/0	0/0	0/0	0/1	0/0	0/1	0/0	0/1	0/1
Chromosome1-2216180 DEL	PRECISE	3391	0.832	0.832	0/1	0/1	0/0	0/1	0/1	0/1	1/1	1/1	1/1	0/1	1/1	1/1	1/1	1/1
Chromosome1-2217372 INS	PRECISE	233	0	0	0/0	0/0	0/0	0/0	0/0	0/0	0/1	0/0	0/0	0/0	0/0	0/0	0/0	0/0
Chromosome1-2217525 DEL	PRECISE	865	0.787	0.378	0/0	0/0	0/0	0/1	0/0	0/0	1/1	0/0	1/1	0/0	1/1	1/1	1/1	1/1
Chromosome1-2218136 INS	PRECISE	159	0	0	0/0	0/0	0/0	0/0	0/0	0/0	0/0	0/0	0/0	0/0	0/0	0/0	0/0	1/1
Chromosome1-2219324 INS	PRECISE	1000	0	0	0/0	0/0	0/0	0/0	0/0	0/0	0/0	0/0	0/0	0/0	0/0	0/1	0/0	0/0
Chromosome1-2219367 INS	PRECISE	67	0	0	0/0	0/0	0/0	0/0	0/0	0/0	0/0	0/0	0/0	0/0	0/0	0/1	0/0	0/0
Chromosome1-2220365 INS	PRECISE	139	0	0.577	0/0	0/0	0/0	0/0	0/1	0/1	0/0	0/0	0/1	0/0	0/0	0/0	0/0	0/0
Chromosome1-2220475 DEL	PRECISE	173	0.5	0.5	0/0	0/0	0/0	0/0	0/0	0/0	0/0	0/0	0/1	0/0	0/1	0/0	0/0	0/1
Chromosome1-2220791 DEL	PRECISE	663	0	0	0/0	0/0	0/0	0/1	0/0	0/0	0/0	0/0	0/1	0/0	0/1	0/1	0/0	0/1
Chromosome1-2220903 INS	PRECISE	137	0	0	0/0	0/0	0/0	0/1	0/0	0/0	0/0	0/0	0/0	0/0	0/0	0/0	0/0	0/0
Chromosome1-2220917 INS	PRECISE	5124	0	0	0/0	0/0	0/0	0/0	0/0	0/0	0/0	0/0	0/0	0/0	0/0	0/0	0/0	0/1
Chromosome1-2221055 INS	PRECISE	228	0	0	0/0	0/0	0/0	0/0	0/0	0/0	0/0	0/0	0/1	0/0	0/1	0/1	0/1	0/1
Chromosome1-2221230 INS	PRECISE	1681	0	0	0/0	0/0	0/0	0/0	0/0	0/0	0/0	0/0	0/0	0/0	0/0	0/0	0/0	0/1
Chromosome1-2221679 INS	PRECISE	241	0	0.5	0/0	0/0	0/0	0/0	0/0	0/0	0/0	0/0	0/0	0/0	0/1	0/0	0/1	0/1
Chromosome1-2221686 DEL	PRECISE	3556	0	0	0/0	0/0	0/0	0/0	0/0	0/0	0/0	0/0	0/0	0/0	0/0	0/0	0/0	1/1
Chromosome1-2222109 DEL	PRECISE	6533	0	0	0/0	0/0	0/0	0/0	0/0	0/0	0/0	0/0	0/0	0/0	0/0	0/0	0/0	0/1
Chromosome1-2222178 DEL	PRECISE	1372	0	0	0/0	0/0	0/0	0/0	0/0	0/0	0/0	0/0	0/1	0/0	0/1	0/1	0/0	0/0
Chromosome1-2222194 DEL	PRECISE	69	0	0	0/1	0/1	0/0	0/1	0/0	0/0	0/1	1/1	0/0	0/1	0/1	0/1	1/1	0/0
Chromosome1-2223918 DEL	PRECISE	50	0	0	0/0	0/0	0/0	0/0	0/0	0/0	0/0	0/0	0/0	0/0	0/0	0/0	0/0	0/1
Chromosome1-2224460 INS	PRECISE	1335	0	0	0/0	0/0	0/0	0/0	0/0	0/0	0/0	0/0	0/1	0/0	0/0	0/0	0/0	0/0
Chromosome1-2224709 INS	PRECISE	6390	0	0	0/0	0/0	0/0	0/0	0/0	0/0	1/1	0/0	0/0	0/0	0/0	0/0	0/0	0/0
Chromosome1-2224710 BND	PRECISE	0	0	0	0/0	0/0	0/0	0/0	0/0	0/0	0/1	0/0	0/0	0/0	0/0	0/0	0/0	0/0
Chromosome1-2224824 DEL	PRECISE	772	0	0	0/0	0/0	0/0	0/0	0/0	0/0	0/0	0/0	0/1	0/0	0/1	0/0	0/1	0/1
Chromosome1-2225326 INS	PRECISE	774	0	0	0/0	0/0	0/0	0/0	0/0	0/0	0/0	0/0	0/0	0/0	0/0	0/0	0/0	0/1
Chromosome1-2225755 INS	PRECISE	1166	0	0	0/0	0/0	0/0	0/0	0/0	0/0	0/0	0/0	1/1	0/0	0/0	0/0	0/0	0/0
Chromosome1-2225815 INS	PRECISE	2444	0	0	0/0	0/0	0/0	0/0	0/0	0/0	0/0	0/0	1/1	0/0	0/0	0/0	0/0	0/0
Chromosome1-2226714 INS	PRECISE	1078	0	0	0/0	0/0	0/0	0/0	0/0	0/0	0/1	0/0	0/0	0/0	0/0	0/0	0/0	0/0
Chromosome1-2227644 DEL	PRECISE	920	0	0	0/1	0/1	0/0	0/0	0/0	0/0	0/1	1/1	0/1	0/1	1/1	1/1	1/1	1/1
Chromosome1-2228001 DEL	PRECISE	251	0	0	0/1	0/1	0/0	0/0	0/0	0/0	0/1	1/1	0/0	0/1	0/1	0/1	0/1	0/0

Chromosome1-2228128	BND	PRECISE	0	0	0	0/0	0/0	0/0	0/0	0/0	0/0	0/1	0/0	0/0	0/0	0/0	0/1	0/1	0/0
Chromosome1-2228689	INS	PRECISE	242	0.843	0.516	0/1	0/1	0/0	0/0	0/0	0/0	0/1	1/1	0/0	0/1	1/1	1/1	1/1	0/1
Chromosome1-2228972	DEL	PRECISE	69	0	0	0/0	0/0	0/0	0/0	0/1	0/1	0/0	0/0	1/1	0/0	0/0	0/0	0/0	0/0
Chromosome1-2229268	DEL	PRECISE	3033	0	0	0/0	0/0	0/0	0/0	0/0	0/0	0/0	0/0	0/0	0/0	0/0	0/0	0/0	1/1
Chromosome1-2229367	INS	PRECISE	1334	0	0	0/0	0/0	0/0	0/0	0/1	0/0	0/0	0/0	1/1	0/0	0/0	0/0	0/0	0/0
Chromosome1-2229984	DEL	PRECISE	78	0	0	0/0	0/0	0/0	0/0	0/0	0/0	0/0	0/0	0/0	0/0	0/0	0/0	0/0	1/1
Chromosome1-2230693	DEL	PRECISE	68	0.408	0.408	0/0	1/1	0/1	0/1	0/1	0/1	0/0	0/0	0/0	1/1	0/0	0/0	0/0	0/0
Chromosome1-2230798	DEL	PRECISE	1519	0	0	0/0	1/1	0/1	0/1	0/1	0/1	0/0	0/0	0/0	1/1	0/0	0/0	0/0	0/0
Chromosome1-2231406	BND	PRECISE	0	0	0	0/0	0/0	0/0	0/0	0/0	0/0	0/1	0/1	0/0	0/0	0/0	0/1	0/0	0/0
Chromosome1-2232197	DEL	PRECISE	740	0	0	0/0	1/1	0/1	0/1	0/1	0/1	0/0	0/0	0/0	1/1	0/0	0/0	0/0	0/0
Chromosome1-2232509	DEL	PRECISE	734	0	0	0/0	0/0	0/0	0/0	0/1	0/1	0/0	0/0	1/1	0/0	0/0	0/0	0/0	0/0
Chromosome1-2233677	INS	PRECISE	2091	0	0	0/0	0/0	0/0	0/0	0/0	0/0	1/1	0/0	0/0	0/0	0/0	0/0	0/0	0/0
Chromosome1-2234245	DEL	PRECISE	212	0.707	0	0/1	0/1	0/0	0/0	0/0	0/0	0/1	1/1	0/0	0/1	0/1	1/1	0/1	0/0
Chromosome1-2234361	INS	PRECISE	104	0	0.408	0/0	0/1	0/1	0/1	0/1	0/1	0/0	0/0	0/0	0/1	0/0	0/0	0/0	0/0
Chromosome1-2234849	DEL	PRECISE	1804	0	0	0/0	0/0	0/0	0/0	0/1	0/1	0/0	0/0	1/1	0/0	0/0	0/0	0/0	0/0
Chromosome1-2235723	INS	PRECISE	64	0	0.289	0/1	0/1	0/0	0/1	0/1	0/1	1/1	1/1	1/1	0/1	1/1	1/1	1/1	0/0
Chromosome1-2235994	DEL	PRECISE	1373	0	0	0/1	0/1	0/0	0/1	0/0	0/0	1/1	1/1	0/0	0/1	1/1	1/1	1/1	0/0
Chromosome1-2237016	DEL	PRECISE	6094	0	0	0/1	0/1	0/0	0/1	0/0	0/0	1/1	1/1	0/0	0/0	0/1	1/1	1/1	0/0
Chromosome1-2237254	DEL	PRECISE	4034	0	0	0/0	0/0	0/0	0/0	0/1	0/1	0/0	0/0	1/1	0/0	0/0	0/0	0/0	0/0
Chromosome1-2237512	DEL	PRECISE	1307	0	0.5	0/0	0/0	0/0	0/0	0/0	0/0	0/0	0/0	0/0	0/0	0/1	0/0	0/0	0/0
Chromosome1-2237903	DEL	PRECISE	628	0	0	0/0	0/0	0/0	0/0	0/1	0/1	0/0	0/0	1/1	0/0	0/0	0/0	0/0	0/0
Chromosome1-2238081	DEL	PRECISE	2258	0	0	0/0	0/1	0/1	0/1	0/1	0/1	0/0	0/0	0/0	0/1	0/0	0/0	0/0	0/0
Chromosome1-2238868	DEL	PRECISE	313	0	0	0/1	0/1	0/0	0/1	0/0	0/0	1/1	1/1	0/0	0/1	1/1	1/1	1/1	0/0
Chromosome1-2239329	INS	PRECISE	1202	0	0.577	0/0	0/0	0/0	0/0	0/1	0/1	0/0	0/0	1/1	0/0	0/0	0/0	0/0	0/0
Chromosome1-2239700	DEL	PRECISE	327	0	0	0/1	0/1	0/0	0/1	0/0	0/0	1/1	1/1	0/0	0/1	1/1	1/1	1/1	0/0
Chromosome1-2239852	BND	PRECISE	0	0	0	0/0	0/0	0/0	0/0	0/0	0/0	1/1	0/1	0/0	0/0	0/0	0/1	0/1	0/0
Chromosome1-2240509	DEL	PRECISE	42902	0	0	0/0	0/0	0/0	0/0	0/1	1/1	0/0	0/0	0/0	0/0	0/0	0/0	0/0	0/0
Chromosome1-2241864	DEL	PRECISE	69	0	0	0/0	1/1	0/1	1/1	1/1	1/1	0/0	0/0	0/0	1/1	0/0	0/0	0/0	0/0
Chromosome1-2242484	DEL	PRECISE	54	0	0	0/0	1/1	0/1	1/1	1/1	1/1	0/0	0/0	0/0	0/1	0/0	0/0	0/0	0/0
Chromosome1-2243478	DEL	PRECISE	13564	0.926	0	0/1	0/1	0/0	0/0	0/0	0/0	0/1	1/1	0/0	0/1	1/1	1/1	0/1	0/0
Chromosome1-2243952	DEL	PRECISE	6611	0	0	0/0	0/0	0/0	0/0	0/0	0/0	0/0	0/0	0/0	0/0	0/0	0/0	0/0	0/1
Chromosome1-2245127	DEL	PRECISE	107	0	0	0/0	0/0	0/0	0/0	0/1	0/1	0/0	0/0	1/1	0/0	0/0	0/0	0/0	0/0
Chromosome1-2246196	INS	PRECISE	66	0	0	0/0	0/0	0/0	0/0	0/1	0/1	0/0	0/0	0/0	0/0	0/0	0/0	0/0	0/0
Chromosome1-2246712	DUP	PRECISE	4677731	0	0	0/0	0/0	0/0	0/0	0/0	0/0	0/1	0/0	0/0	0/0	0/0	0/0	0/0	0/0
Chromosome1-2246851	DEL	PRECISE	842	0	0	0/0	0/0	0/0	0/0	0/1	0/1	0/0	0/0	1/1	0/0	0/0	0/0	0/0	0/0
Chromosome1-2246945	INS	PRECISE	190	0	0	0/0	0/0	0/0	0/0	0/0	0/0	0/1	0/0	0/0	0/0	0/0	0/0	0/0	0/0
Chromosome1-2247073	INS	PRECISE	333	0	0.756	0/0	0/0	0/0	0/0	0/1	0/1	0/0	0/0	1/1	0/0	0/1	0/1	0/1	1/1
Chromosome1-2248028	BND	PRECISE	0	0	0	0/1	0/1	0/0	0/0	0/0	0/0	0/0	0/0	0/0	0/1	0/0	0/0	0/1	0/0
Chromosome1-2248380	INS	PRECISE	10349	0	0	0/0	0/0	0/0	0/0	0/0	0/0	0/0	0/0	0/0	0/0	0/0	0/0	0/0	0/0
Chromosome1-2248380	BND	PRECISE	0	0	0	0/0	0/0	0/0	0/0	0/0	0/0	0/0	0/0	0/0	0/0	0/0	0/0	0/0	0/1
Chromosome1-2248506	INS	PRECISE	1304	0	0.837	0/0	0/1	0/1	0/1	0/1	0/1	0/0	0/0	0/0	0/1	0/0	0/0	0/0	0/0
Chromosome1-2248910	INS	PRECISE	3506	0	0	0/0	0/0	0/0	0/0	0/0	0/0	0/0	0/0	0/0	0/0	0/0	0/0	0/0	1/1
Chromosome1-2249046	DEL	PRECISE	152	0.267	0	0/1	1/1	0/1	1/1	1/1	1/1	1/1	1/1	0/1	1/1	0/1	0/1	0/1	0/1
Chromosome1-2250046	INS	PRECISE	232	0	0.447	0/0	0/0	0/0	0/0	0/0	0/0	0/0	0/0	0/0	0/0	0/1	0/1	0/1	0/1
Chromosome1-2250352	INS	PRECISE	326	0	0.577	0/0	0/0	0/0	0/0	0/0	0/0	0/0	0/0	0/0	0/0	0/1	0/0	0/1	0/1
Chromosome1-2251109	INS	PRECISE	137	0	0	0/0	0/0	0/0	0/0	0/0	0/0	0/1	0/0	0/0	0/0	0/0	0/0	0/0	0/0
Chromosome1-2251247	DEL	PRECISE	53	0	0	0/0	0/0	0/0	0/0	0/0	0/0	0/0	0/0	0/1	0/0	0/1	0/1	0/1	0/1
Chromosome1-2251934	DEL	PRECISE	93	0	0	0/0	0/0	0/0	0/0	0/0	0/0	0/0	0/0	0/1	0/0	0/1	0/1	0/1	0/1
Chromosome1-2253200	DEL	PRECISE	270	0	0	0/0	0/0	0/0	0/0	0/0	0/0	0/0	0/0	0/1	0/0	0/1	0/1	0/1	0/1
Chromosome1-2253789	DEL	PRECISE	1066	0	0	0/0	0/0	0/0	0/0	0/0	0/0	0/0	0/0	0/1	0/0	0/1	0/1	0/1	0/1
Chromosome1-2254626	DEL	PRECISE	455	0	0	0/0	0/0	0/0	0/0	0/0	0/0	0/0	0/0	0/1	0/0	0/0	0/0	0/0	0/0
Chromosome1-2254712	DEL	PRECISE	80	0	0	0/0	0/0	0/0	0/0	0/0	0/0	0/0	0/0	0/1	0/0	0/0	0/0	0/0	0/0
Chromosome1-2255243	DEL	PRECISE	72	0	0	0/0	0/0	0/0	0/0	0/0	0/0	0/0	0/0	0/1	0/0	0/0	0/0	0/0	0/0
Chromosome1-2255468	DEL	PRECISE	656	0.354	0	0/1	0/1	0/0	0/0	0/0	0/0	1/1	1/1	0/0	0/1	1/1	1/1	1/1	0/0
Chromosome1-2255958	DEL	PRECISE	85	0	0	0/0	0/0	0/0	0/0	0/0	0/0	0/0	0/0	0/0	0/0	0/0	0/1	0/0	0/0
Chromosome1-2256374	BND	PRECISE	0	0	0	0/0	0/0	0/0	0/0	0/0	0/0	0/0	0/0	0/0	0/0	0/0	0/1	0/0	0/0
Chromosome1-2256696	INS	PRECISE	329	0	0	0/0	0/0	0/0	0/0	0/0	0/0	0/0	0/0	0/0	0/0	0/0	0/0	0/0	0/1
Chromosome1-2256915	DEL	PRECISE	1391	0	0	0/1	1/1	0/1	1/1	1/1	1/1	1/1	1/1	1/1	1/1	1/1	1/1	1/1	1/1
Chromosome1-2257107	DEL	PRECISE	56	0	0	0/0	0/0	0/0	0/0	0/0	0/0	0/0	0/0	0/0	0/0	0/1	0/0	0/1	0/0
Chromosome1-2257128	DEL	PRECISE	3043	0	0	0/0	0/0	0/0	0/0	0/0	0/0	0/0	0/0	0/0	0/0	0/1	0/0	0/1	0/0
Chromosome1-2257462	INS	PRECISE	988	0	0	0/0	0/0	0/0	0/1	0/0	0/0	0/0	0/0	0/0	0/0	0/0	0/0	0/0	0/0
Chromosome1-2257474	DEL	PRECISE	79	0	0	0/0	0/0	0/0	0/0	0/0	0/0	0/0	0/0	0/0	0/0	0/0	0/0	0/0	1/1
Chromosome1-2257693	INS	PRECISE	985	0	0	0/0	0/0	0/0	0/0	0/0	0/0	0/0	0/0	0/0	0/0	0/0	0/0	0/0	0/1
Chromosome1-2257957	INS	PRECISE	4706	0	0	0/0	0/0	0/0	1/1	0/0	0/0	0/0	0/0	0/0	0/0	0/0	0/0	0/0	0/0
Chromosome1-2258101	INS	PRECISE	329	0	0.289	0/1	1/1	0/1	0/1	0/1	0/1	0/1	1/1	0/0	1/1	0/1	1/1	0/1	0/0
Chromosome1-2258481	INS	PRECISE	140	0	0.447	0/0	0/1	0/1	0/0	0/1	0/1	0/0	0/0	0/0	0/1	0/0	0/0	0/0	0/0
Chromosome1-2258728	INS	PRECISE	92	0	0.354	0/1	0/1	0/0	0/0	0/0	0/0	0/1	1/1	0/0	0/1	0/1	1/1	0/1	0/0
Chromosome1-2258841	INS	PRECISE	645	0	0.577	0/0	0/0	0/0	0/0	0/0	0/0	0/0	0/0	0/0	0/0	0/1	0/0	0/1	0/1
Chromosome1-2258951	INS	PRECISE	1240	0	0.5	0/0	0/0	0/0	0/0	0/0	0/0	0/0	0/0	0/0	0/0	0/1	0/0	0/1	0/1
Chromosome1-2259227	INS	PRECISE	98	0	0	0/0	0/0	0/0	0/0	0/0	0/0	0/0	0/0	0/0	0/0	0/0	0/0	0/0	0/1
Chromosome1-2259452	DEL	PRECISE	1547	0	0	0/0	0/0	0/0	0/0	0/0	0/0	0/0	0/0	0/1	0/0	0/1	0/0	0/1	0/1
Chromosome1-2259706	INS	PRECISE	131	0	0	0/0	0/0	0/0	0/0	0/0	0/0	0/0	0/0	0/0	0/0	0/0	0/0	0/0	0/1
Chromosome1-2259782	INS	PRECISE	4711	0	0	0/0	0/0	0/0	0/0	0/0	0/0	0/0	0/0	0/0	0/0	0/0	0/0	0/0	1/1

Chromosome1-2260065	DEL	PRECISE	337	0	0	0/0	0/0	0/0	0/1	0/0	0/0	0/0	0/0	0/0	0/0	0/0	0/0	0/0	0/0
Chromosome1-2260152	DEL	PRECISE	628	0	0	0/0	0/0	0/0	0/0	0/0	0/0	0/0	0/0	0/1	0/0	0/0	0/0	0/0	0/0
Chromosome1-2260236	DEL	PRECISE	97	0	0	0/0	0/0	0/0	0/0	0/0	0/0	0/0	0/0	0/0	0/0	0/1	0/0	0/1	1/1
Chromosome1-2260279	INS	PRECISE	58	0	0	0/0	0/0	0/0	0/1	0/0	0/0	0/0	0/0	0/0	0/0	0/0	0/0	0/0	0/0
Chromosome1-2260281	DEL	PRECISE	1284	0	0	0/0	0/0	0/0	0/0	0/0	0/0	0/0	0/0	0/1	0/0	0/1	0/0	0/1	0/1
Chromosome1-2261309	DEL	PRECISE	977	0	0	0/0	0/0	0/0	0/0	0/0	0/0	0/1	0/0	0/0	0/0	0/0	0/0	0/0	0/0
Chromosome1-2262101	INS	PRECISE	328	0	0	0/0	0/0	0/0	0/1	0/0	0/0	0/0	0/0	0/0	0/0	0/0	0/0	0/0	0/0
Chromosome1-2262769	DEL	PRECISE	63	0	0	0/1	0/1	0/0	0/1	0/0	0/0	0/1	1/1	0/1	0/1	1/1	1/1	1/1	0/1
Chromosome1-2263407	DEL	PRECISE	703	0	0	0/0	0/0	0/0	0/0	0/0	0/0	0/0	0/0	0/1	0/0	0/0	0/1	0/0	0/0
Chromosome1-2264086	DEL	PRECISE	199	0	0	0/0	0/0	0/0	0/0	0/0	0/0	0/0	0/0	0/1	0/0	0/1	0/1	0/0	0/1
Chromosome1-2264377	DEL	PRECISE	58	0	0	0/0	0/0	0/0	0/0	0/0	0/0	0/0	0/0	0/0	0/0	0/1	0/0	0/0	0/1
Chromosome1-2264716	DEL	PRECISE	856	0	0	0/0	0/0	0/0	0/0	0/0	0/0	0/0	0/0	0/0	0/0	0/1	0/0	0/1	0/1
Chromosome1-2264758	DEL	PRECISE	52	0	0	0/0	0/0	0/0	0/0	0/0	0/0	0/0	0/0	0/1	0/0	0/0	0/1	0/0	0/0
Chromosome1-2265487	INS	PRECISE	429	0	0.577	0/0	0/0	0/0	0/1	0/0	0/0	0/1	0/0	0/0	0/0	0/0	0/0	0/0	0/1
Chromosome1-2265847	INS	PRECISE	86	0	0	0/1	1/1	0/1	1/1	1/1	1/1	1/1	1/1	1/1	1/1	1/1	1/1	1/1	1/1
Chromosome1-2265882	DEL	PRECISE	58	0.577	0.577	0/0	0/0	0/0	0/0	0/0	0/0	0/0	0/0	0/0	0/0	0/1	0/0	0/1	0/1
Chromosome1-2266203	DEL	PRECISE	55	0	0	0/0	0/0	0/0	0/0	0/0	0/0	0/0	0/0	0/0	0/0	0/1	0/0	0/1	0/0
Chromosome1-2266547	DEL	PRECISE	1696	0	0	0/1	1/1	0/1	1/1	1/1	1/1	1/1	1/1	1/1	1/1	1/1	1/1	1/1	1/1
Chromosome1-2267241	DEL	PRECISE	572	0	0	0/0	0/0	0/0	0/0	0/0	0/0	0/0	0/0	0/0	0/0	0/0	0/1	0/0	0/0
Chromosome1-2267384	DEL	PRECISE	177	0	0	0/0	0/0	0/0	0/0	0/0	0/0	0/0	0/0	0/0	0/0	0/0	0/1	0/0	0/0
Chromosome1-2268014	DEL	PRECISE	41327	0	0	0/1	0/1	0/0	0/1	0/1	0/0	0/0	0/0	0/0	0/0	0/0	0/0	0/0	0/0
Chromosome1-2268037	DEL	PRECISE	969	0	0	0/0	0/0	0/0	0/0	0/0	0/0	0/0	0/0	0/0	0/0	0/1	0/0	0/1	0/1
Chromosome1-2269761	DEL	PRECISE	870	0	0	0/0	0/0	0/0	0/0	0/0	0/0	0/0	0/0	0/0	0/0	0/1	0/0	0/1	0/1
Chromosome1-2269771	DUP	PRECISE	72350	0	0	0/1	0/1	0/0	1/1	0/1	0/1	0/0	0/1	0/1	0/1	0/1	0/1	0/1	0/0
Chromosome1-2270059	INS	PRECISE	6693	0	0	0/0	0/0	0/0	0/0	0/0	0/0	0/0	0/0	0/0	0/0	0/0	0/0	0/0	0/0
Chromosome1-2270192	INS	PRECISE	4732	0	0	0/0	0/0	0/0	1/1	0/0	0/0	0/0	0/0	0/0	0/0	0/0	0/0	0/0	0/0
Chromosome1-2270663	DEL	PRECISE	3023	0	0	0/0	0/0	0/0	0/0	0/0	0/0	0/0	0/0	0/0	0/0	0/1	0/0	0/1	0/1
Chromosome1-2270700	BND	PRECISE	0	0	0	0/0	0/0	0/0	0/0	0/1	0/1	0/0	0/0	0/0	0/0	0/0	0/0	0/0	0/0
Chromosome1-2271159	DEL	PRECISE	78	0	0	0/0	0/0	0/0	0/0	0/0	0/0	0/0	0/0	0/0	0/0	0/1	0/0	0/1	0/0
Chromosome1-2271733	INS	PRECISE	3445	0	0	0/0	0/0	0/0	0/0	0/0	0/0	0/0	0/0	0/0	0/0	0/0	0/0	0/0	1/1
Chromosome1-2271776	INS	PRECISE	159	0	0	0/0	0/0	0/0	0/0	0/0	0/0	0/0	0/0	0/0	0/0	0/0	0/0	0/0	0/1
Chromosome1-2271932	DEL	PRECISE	141	0	0	0/0	0/0	0/0	0/0	0/0	0/0	0/0	0/0	0/1	0/0	0/0	0/0	0/0	0/0
Chromosome1-2271973	BND	PRECISE	0	0	0	0/0	0/0	0/0	0/0	0/0	0/0	0/0	0/0	0/0	0/0	0/0	0/0	0/1	0/0
Chromosome1-2272354	INS	PRECISE	5446	0	0	0/0	0/0	0/0	0/0	0/0	0/0	0/0	0/0	0/0	0/0	0/0	0/1	0/0	0/0
Chromosome1-2272575	DEL	PRECISE	1507	0.316	0	0/1	0/1	0/0	0/0	0/1	0/1	0/0	0/1	0/1	0/1	0/1	1/1	0/1	0/0
Chromosome1-2272793	DEL	PRECISE	542	0	0	0/0	0/0	0/0	0/0	0/0	0/0	0/0	0/0	0/0	0/0	1/1	0/0	1/1	0/1
Chromosome1-2272889	INS	PRECISE	194	0	0.894	0/0	0/0	0/0	0/1	0/0	0/0	0/0	0/0	0/1	0/0	0/1	0/0	0/1	1/1
Chromosome1-2273052	INS	PRECISE	5645	0	0	0/0	0/0	0/0	1/1	0/0	0/0	0/0	0/0	0/0	0/0	0/0	0/0	0/0	0/0
Chromosome1-2273055	INS	PRECISE	8371	0	0	0/0	0/0	0/0	0/1	0/0	0/0	0/0	0/0	0/0	0/0	0/0	0/0	0/0	0/0
Chromosome1-2273066	INS	PRECISE	634	0	0.577	0/0	0/0	0/0	0/0	0/0	0/0	0/0	0/0	0/0	0/0	0/1	0/0	0/1	0/1
Chromosome1-2274066	DEL	PRECISE	171	0	0	0/1	0/1	0/0	0/1	0/1	0/1	0/0	0/1	0/1	0/1	1/1	1/1	0/1	0/0
Chromosome1-2274790	BND	PRECISE	0	0	0	0/0	0/0	0/0	0/0	0/0	0/0	0/0	0/0	0/0	0/0	0/0	0/1	0/0	0/0
Chromosome1-2274805	INS	PRECISE	8927	0	0	0/0	0/0	0/0	0/1	0/0	0/0	0/0	0/0	0/0	0/0	0/0	0/0	0/0	0/0
Chromosome1-2274821	INS	PRECISE	1411	0	0	0/0	0/0	0/0	0/0	0/0	0/0	0/0	0/0	0/1	0/0	0/0	0/0	0/0	0/0
Chromosome1-2275013	DEL	PRECISE	52	0	0	0/0	0/0	0/0	0/0	0/0	0/0	0/0	0/0	0/0	0/0	0/0	0/1	0/0	0/0
Chromosome1-2275132	DEL	PRECISE	1156	0.302	0.302	0/1	0/1	0/0	0/1	0/1	0/1	0/0	0/1	0/0	0/1	0/1	0/1	0/1	0/0
Chromosome1-2275283	INS	PRECISE	1007	0	0	0/0	0/0	0/0	0/0	0/0	0/0	0/0	0/0	0/1	0/0	0/0	0/0	0/0	0/0
Chromosome1-2275392	INS	PRECISE	3161	0	0	0/0	0/0	0/0	0/0	0/0	0/0	0/0	0/0	0/0	0/0	0/0	1/1	0/0	0/0
Chromosome1-2275691	DEL	PRECISE	1202	0	0	0/1	0/1	0/0	1/1	0/1	0/1	0/0	0/1	1/1	0/1	1/1	1/1	1/1	0/1
Chromosome1-2276355	DEL	PRECISE	66	0	0	0/0	0/0	0/0	0/0	0/0	0/0	0/0	0/0	0/1	0/0	0/0	0/0	0/0	0/0
Chromosome1-2277456	DEL	PRECISE	8093	0	0	0/0	0/0	0/0	0/0	0/0	0/0	0/0	0/0	0/1	0/0	0/0	0/0	0/0	0/0
Chromosome1-2278025	DEL	PRECISE	110	0	0	0/0	0/0	0/0	0/0	0/0	0/0	0/0	0/0	0/1	0/0	0/0	0/0	0/0	0/0
Chromosome1-2278578	DEL	PRECISE	64	0	0	0/0	0/0	0/0	0/0	0/0	0/0	0/0	0/0	0/0	0/0	0/1	0/0	0/0	0/0
Chromosome1-2279095	INS	PRECISE	5655	0	0	0/0	0/0	0/0	0/0	0/0	0/0	0/0	0/0	0/0	0/0	0/0	0/1	0/0	0/0
Chromosome1-2279226	DEL	PRECISE	2578	0	0	0/0	0/0	0/0	0/0	0/0	0/0	0/0	0/0	0/0	0/0	0/0	0/1	0/0	0/0
Chromosome1-2279487	DEL	PRECISE	2392	0	0	0/0	0/0	0/0	0/0	0/0	0/0	0/0	0/0	0/0	0/0	0/0	0/1	0/0	0/0
Chromosome1-2279733	DEL	PRECISE	1798	0	0	0/0	0/0	0/0	0/0	0/0	0/0	0/0	0/0	0/0	0/0	0/0	1/1	0/0	0/0
Chromosome1-2280166	INS	PRECISE	1414	0	0	0/0	0/0	0/0	0/0	0/0	0/0	0/0	0/0	0/0	0/0	0/0	0/0	0/0	1/1
Chromosome1-2280205	DEL	PRECISE	604	0	0	0/0	0/0	0/0	0/0	0/0	0/0	0/0	0/0	0/0	0/0	1/1	0/0	1/1	0/0
Chromosome1-2280397	DEL	PRECISE	2807	0	0	0/0	0/0	0/0	0/0	0/0	0/0	0/0	0/0	0/0	0/0	0/0	1/1	0/0	0/0
Chromosome1-2280798	INS	PRECISE	5955	0	0	0/0	0/0	0/0	0/0	0/0	0/0	0/0	0/0	1/1	0/0	0/0	0/0	0/0	0/0
Chromosome1-2281693	INS	PRECISE	87	0	0	0/0	1/1	0/1	0/0	1/1	1/1	1/1	1/1	1/1	1/1	0/0	0/0	0/0	1/1
Chromosome1-2283092	DEL	PRECISE	5023	0	0	0/0	0/0	0/0	0/0	0/0	0/0	0/0	0/0	0/0	0/0	0/0	0/0	0/0	0/1
Chromosome1-2283672	DEL	PRECISE	625	0	0	0/0	0/0	0/0	0/0	0/0	0/0	0/0	0/0	0/0	0/0	1/1	1/1	0/0	0/0
Chromosome1-2284009	DEL	PRECISE	154	0	0	0/0	1/1	0/1	0/0	1/1	1/1	1/1	1/1	1/1	1/1	1/1	1/1	0/0	1/1
Chromosome1-2284169	DEL	PRECISE	2079	0	0	0/0	0/0	0/0	0/0	0/0	0/0	0/0	0/0	0/0	0/0	0/0	0/0	0/0	0/1
Chromosome1-2284306	DEL	PRECISE	2493	0	0	0/0	0/0	0/0	0/0	0/0	0/0	0/0	0/0	0/0	0/0	1/1	0/0	0/0	0/0
Chromosome1-2284432	DEL	PRECISE	1165	0	0	0/0	0/0	0/0	0/0	0/0	0/0	0/0	0/0	1/1	0/0	0/1	1/1	0/0	1/1
Chromosome1-2284626	INS	PRECISE	287	0	0	0/0	0/0	0/0	0/0	0/0	0/0	0/0	0/0	0/0	0/0	0/0	0/0	0/0	0/1
Chromosome1-2284714	INS	PRECISE	6498	0	0	0/0	0/0	0/0	0/0	0/0	0/0	0/0	0/0	0/0	0/0	0/0	1/1	0/0	0/0
Chromosome1-2284974	INS	PRECISE	4020	0	0	0/0	0/0	0/0	0/0	0/0	0/0	0/0	0/0	0/0	0/0	0/0	0/0	0/0	0/1
Chromosome1-2285099	DEL	PRECISE	53	0	0	0/1	0/0	0/0	0/0	0/0	0/0	0/0	0/0	0/0	0/0	1/1	0/0	0/0	0/1
Chromosome1-2285140	INS	PRECISE	442	0	0	0/0	0/0	0/0	0/0	0/0	0/0	0/0	0/0	0/0	0/0	0/0	0/1	0/0	0/0

Chromosome1-2287164	INS	PRECISE	121	0	0	0/0	0/0	0/0	0/0	0/0	0/0	0/0	0/0	1/1	0/0	0/0	0/1	0/0	0/1
Chromosome1-2287339	DEL	PRECISE	438	0	0	0/0	0/0	0/0	0/0	0/0	0/0	0/0	0/0	0/0	0/0	1/1	0/1	1/1	1/1
Chromosome1-2287412	INS	PRECISE	298	0	0	0/0	0/0	0/0	0/0	0/0	0/0	0/0	0/0	0/0	0/0	0/0	0/1	0/0	0/0
Chromosome1-2287584	INS	PRECISE	51	0	0.5	0/0	0/0	0/0	0/0	0/0	0/0	0/0	0/0	0/0	0/0	0/1	0/1	1/1	1/1
Chromosome1-2287593	DEL	PRECISE	79	0	0	0/0	0/0	0/0	0/0	0/0	0/0	0/0	0/0	0/0	0/0	1/1	0/0	1/1	1/1
Chromosome1-2287775	INS	PRECISE	54	0	0	0/0	0/1	0/0	0/0	0/0	0/0	0/1	0/1	0/0	0/0	0/1	0/0	0/0	0/1
Chromosome1-2288039	DEL	PRECISE	867	0.447	0.447	0/0	0/1	0/0	0/0	0/0	0/0	0/0	0/0	0/0	0/0	0/1	0/0	0/0	0/1
Chromosome1-2288614	INS	PRECISE	54	0	0	0/0	0/1	0/0	0/0	0/0	0/0	0/1	0/1	0/0	0/0	0/1	0/0	0/0	0/1
Chromosome1-2288694	DEL	PRECISE	84	0	0	0/0	0/0	0/0	0/0	0/0	0/0	0/0	0/0	0/0	0/0	1/1	0/0	1/1	1/1
Chromosome1-2289146	DEL	PRECISE	12567	0	0	0/0	0/0	0/0	0/0	0/0	0/0	0/0	0/0	0/0	0/0	1/1	0/0	0/0	1/1
Chromosome1-2291726	BND	PRECISE	0	0	0	0/0	0/0	0/0	0/0	0/0	0/0	0/0	0/0	0/0	0/0	0/1	0/0	0/0	0/1
Chromosome1-2292695	INS	PRECISE	704	0	0	0/0	0/0	0/0	0/0	0/0	0/0	0/0	0/0	0/0	0/0	1/1	0/0	1/1	1/1
Chromosome1-2292904	DEL	PRECISE	1081	0	0	0/0	0/0	0/0	0/0	0/0	0/0	0/0	0/0	0/0	0/0	0/0	1/1	0/0	0/0
Chromosome1-2294018	DEL	PRECISE	20528	0	0	0/0	0/0	0/0	0/0	0/0	0/0	0/0	0/0	0/0	0/0	0/1	0/1	0/1	0/0
Chromosome1-2295011	DEL	PRECISE	4666	0	0	0/1	0/1	0/0	1/1	0/1	0/0	0/1	0/1	1/1	0/1	1/1	1/1	1/1	0/0
Chromosome1-2296571	INS	PRECISE	133	0	0	0/0	0/0	0/0	0/0	0/0	0/0	0/0	0/0	0/0	0/0	0/0	0/0	0/0	0/1
Chromosome1-2297554	DEL	PRECISE	1716	0	0	0/0	0/0	0/0	0/1	0/0	0/0	0/0	0/0	0/0	0/0	0/0	0/0	0/0	0/0
Chromosome1-2297638	DEL	PRECISE	11751	0	0	0/0	0/0	0/0	0/0	0/0	0/0	0/0	0/0	0/0	0/0	0/0	0/0	0/0	0/1
Chromosome1-2297714	DEL	PRECISE	2349	0	0	0/0	0/0	0/0	0/0	0/0	0/0	0/0	0/0	0/0	0/0	0/0	0/0	0/0	0/1
Chromosome1-2297812	DEL	PRECISE	59	0	0	0/0	0/0	0/0	0/0	0/1	0/0	0/0	0/0	0/1	0/0	0/0	0/0	0/0	0/0
Chromosome1-2297859	DEL	PRECISE	2590	0	0	0/0	0/0	0/0	0/0	0/0	0/0	0/1	0/0	0/0	0/0	0/0	0/0	0/0	0/0
Chromosome1-2298437	DEL	PRECISE	939	0	0	0/0	0/0	0/0	0/0	0/0	0/0	0/1	0/0	0/0	0/0	0/0	0/0	0/0	0/0
Chromosome1-2298575	INS	PRECISE	703	0	0	0/0	0/0	0/0	0/0	0/0	0/0	0/0	0/0	0/0	0/0	0/0	0/0	0/0	1/1
Chromosome1-2298594	DEL	PRECISE	64	0	0	0/0	0/0	0/0	0/0	0/1	0/1	0/0	0/0	0/1	0/0	0/0	0/0	0/0	0/0
Chromosome1-2298888	INS	PRECISE	2369	0	0	0/0	0/0	0/0	0/0	0/0	0/0	0/1	0/0	0/0	0/0	0/0	0/0	0/0	0/0
Chromosome1-2299230	DEL	PRECISE	237	0	0	0/0	0/0	0/0	0/0	0/0	0/0	0/0	0/0	0/0	0/0	0/1	0/0	0/0	1/1
Chromosome1-2299449	INS	PRECISE	50	0	0.447	0/0	0/0	0/0	0/0	0/0	0/0	0/0	0/0	0/1	0/0	0/1	0/0	0/0	1/1
Chromosome1-2299480	DEL	PRECISE	5902	0	0	0/0	0/0	0/0	0/1	0/1	0/1	0/1	0/1	0/1	0/0	0/1	0/1	0/1	1/1
Chromosome1-2299949	DEL	PRECISE	214	0	0	0/1	0/1	0/0	0/1	0/0	0/0	0/0	0/1	0/0	0/1	0/1	0/1	0/1	0/0
Chromosome1-2300520	DEL	PRECISE	1521	0	0	0/0	0/0	0/0	0/0	0/1	0/0	0/1	0/0	1/1	0/0	0/1	0/1	0/1	1/1
Chromosome1-2304723	BND	PRECISE	0	0	0	0/0	0/0	0/0	0/0	0/0	0/0	0/0	0/0	0/1	0/0	0/0	0/0	0/0	0/0
Chromosome1-2306026	DEL	PRECISE	61	0	0	0/0	0/0	0/0	0/0	0/0	0/0	0/0	0/0	1/1	0/1	0/0	0/0	0/0	0/0
Chromosome1-2306068	INS	PRECISE	124	0	0	0/0	0/0	0/0	0/0	0/0	0/0	0/0	0/0	1/1	0/1	0/0	0/0	0/0	0/0
Chromosome1-2306466	DEL	PRECISE	79	0	0	0/0	0/0	0/0	0/0	0/0	0/0	0/0	0/0	0/1	0/0	0/0	0/0	0/0	0/0
Chromosome1-2307569	INS	PRECISE	482	0	0	0/0	0/0	0/0	0/0	0/0	0/0	0/0	0/0	0/0	0/0	0/0	0/0	0/0	0/1
Chromosome1-2308155	DUP	PRECISE	2895	0	0	0/0	0/0	0/0	0/0	0/0	0/0	0/0	0/0	0/0	0/0	0/0	0/0	0/0	0/1
Chromosome1-2308272	INS	PRECISE	10850	0	0	0/0	0/0	0/0	0/0	0/0	0/0	0/0	0/0	0/1	0/0	0/0	0/0	0/0	0/0
Chromosome1-2308281	INS	PRECISE	8197	0	0	0/0	0/0	0/0	0/0	0/0	0/0	0/0	0/0	0/0	0/0	0/0	0/0	0/0	0/1
Chromosome1-2308312	INS	PRECISE	14780	0	0	0/0	0/0	0/0	0/0	0/0	0/0	0/0	0/0	0/0	0/0	0/0	0/0	0/0	1/1
Chromosome1-2308312	INS	PRECISE	5644	0	0	0/0	0/0	0/0	0/0	0/0	0/0	0/0	0/0	0/1	0/0	0/0	0/0	0/0	0/0
Chromosome1-2308316	BND	PRECISE	0	0.837	0	0/0	0/0	0/0	0/0	0/0	0/0	0/0	0/0	0/1	0/0	0/1	0/0	0/0	0/0
Chromosome1-2308364	INS	PRECISE	7691	0	0	0/0	0/0	0/0	0/0	0/0	0/0	1/1	0/0	0/0	0/0	0/0	0/0	0/0	0/0
Chromosome1-2308408	INS	PRECISE	607	0	0	0/0	0/0	0/0	0/0	0/0	0/0	0/1	0/0	0/0	0/0	0/0	0/0	0/0	0/0
Chromosome1-2308709	INS	PRECISE	308	0	0.518	0/1	1/1	0/0	1/1	0/0	0/0	0/0	1/1	0/0	1/1	1/1	0/1	0/1	0/0
Chromosome1-2308768	BND	PRECISE	0	0	0	0/0	0/0	0/0	0/0	0/1	1/1	0/0	0/0	0/1	0/0	0/0	0/0	0/0	0/0
Chromosome1-2310422	INS	PRECISE	2325	0	0	0/0	0/0	0/0	0/0	0/0	0/0	0/0	0/0	0/0	0/0	0/0	0/0	0/0	0/1
Chromosome1-2310452	INS	PRECISE	1204	0	0	0/0	0/0	0/0	0/0	0/0	0/0	0/0	0/0	0/0	0/0	0/0	0/1	0/0	0/0
Chromosome1-2310653	INS	PRECISE	473	0	0	0/0	0/0	0/0	0/0	0/0	0/0	0/1	0/0	0/0	0/0	0/0	0/0	0/0	0/0
Chromosome1-2311086	INS	PRECISE	426	0	0	0/0	0/0	0/0	0/0	0/0	0/0	0/0	0/0	0/0	0/0	0/0	0/1	0/0	0/0
Chromosome1-2311483	INS	PRECISE	71	0	0	0/0	0/0	0/0	0/0	0/0	0/0	0/0	0/0	0/1	0/0	0/1	0/1	0/1	0/1
Chromosome1-2311866	INS	PRECISE	89	0	0	0/0	0/0	0/0	0/0	0/0	0/0	0/0	0/0	0/1	0/0	0/1	0/0	0/1	0/1
Chromosome1-2312414	INS	PRECISE	137	0	0	0/0	0/0	0/0	0/0	0/0	0/0	0/0	0/0	0/0	0/0	0/1	0/0	0/1	0/1
Chromosome1-2313501	DEL	PRECISE	75	0	0	0/0	0/0	0/0	0/0	0/0	0/0	0/0	0/0	0/0	0/0	0/1	0/1	0/1	0/1
Chromosome1-2313911	INS	PRECISE	1000	0	0	0/0	0/0	0/0	0/1	0/0	0/0	0/0	0/0	0/0	0/0	0/0	0/0	0/0	0/0
Chromosome1-2313936	INS	PRECISE	104	0	0	0/0	0/0	0/0	0/0	0/0	0/0	0/0	0/0	0/1	0/0	0/1	0/1	0/1	1/1
Chromosome1-2314213	INS	PRECISE	51	0	0	0/0	0/0	0/0	0/0	0/0	0/0	0/0	0/0	0/1	0/0	0/1	0/1	0/1	0/1
Chromosome1-2314519	DEL	PRECISE	63	0.447	0.447	0/0	0/0	0/0	0/0	0/0	0/0	0/0	0/0	0/1	0/0	0/1	0/1	0/1	1/1
Chromosome1-2315104	INS	PRECISE	5056	0	0	0/0	0/0	0/0	0/0	0/0	0/0	0/0	0/0	0/0	0/0	0/0	0/0	0/0	0/1
Chromosome1-2315124	INS	PRECISE	50	0	0.378	0/0	0/0	0/0	0/0	0/1	0/1	0/0	0/0	0/1	0/0	0/1	0/1	0/1	1/1
Chromosome1-2315316	DEL	PRECISE	700	0	0	0/1	1/1	0/1	1/1	1/1	1/1	1/1	1/1	1/1	1/1	1/1	1/1	1/1	0/1
Chromosome1-2316104	BND	PRECISE	0	0	0	0/0	0/0	0/0	0/0	0/0	0/0	0/0	0/0	0/1	0/0	0/0	0/0	0/0	0/0
Chromosome1-2316104	INS	PRECISE	5534	0	0	0/0	0/0	0/0	0/0	0/0	0/0	0/0	0/0	0/0	0/0	0/0	0/1	0/0	0/0
Chromosome1-2316750	DEL	PRECISE	462	0	0	0/0	0/0	0/0	0/0	0/0	0/0	0/0	0/0	0/0	0/0	0/0	0/0	0/0	0/1
Chromosome1-2316825	INS	PRECISE	132	0	0	0/0	0/0	0/0	0/0	0/0	0/0	0/1	0/0	0/0	0/0	0/0	0/0	0/0	0/0
Chromosome1-2317758	INS	PRECISE	223	0	0.5	0/0	0/0	0/0	0/0	0/1	0/1	0/1	0/0	0/1	0/0	0/0	0/0	0/0	0/0
Chromosome1-2317907	DEL	PRECISE	218	0	0	0/0	0/0	0/0	0/0	0/0	0/0	0/0	0/0	0/1	0/0	0/1	0/1	0/1	1/1
Chromosome1-2318043	DEL	PRECISE	1098	0	0	0/1	1/1	0/1	1/1	1/1	1/1	0/1	1/1	0/1	1/1	0/1	0/1	1/1	0/0
Chromosome1-2318121	INS	PRECISE	600	0	0	0/0	0/0	0/0	0/0	0/0	0/0	0/1	0/0	0/0	0/0	0/0	0/0	0/0	0/0
Chromosome1-2318586	INS	PRECISE	52	0	0.837	0/0	0/0	0/0	0/0	0/0	0/0	0/0	0/0	0/1	0/0	0/1	0/1	0/0	1/1
Chromosome1-2318641	DEL	PRECISE	483	0	0	0/0	0/0	0/0	0/0	0/0	0/0	0/0	0/0	0/1	0/0	0/1	0/1	0/1	1/1
Chromosome1-2318664	INS	PRECISE	1556	0	0	0/0	0/0	0/0	0/0	0/0	0/0	0/1	0/0	0/0	0/0	0/0	0/0	0/0	0/0
Chromosome1-2318745	INS	PRECISE	276	0	0.63	0/1	1/1	0/1	1/1	1/1	1/1	0/1	1/1	0/1	1/1	0/1	0/1	0/1	0/0
Chromosome1-2318803	INS	PRECISE	1406	0	0.9	0/0	0/1	0/1	0/0	0/1	0/1	0/1	0/1	0/0	0/1	0/0	0/0	0/0	0/0

Chromosome1-2319158	DEL	PRECISE	52	0	0	0/0	0/0	0/0	0/0	0/0	0/0	0/0	0/0	0/0	0/0	0/1	0/0	0/0	1/1
Chromosome1-2319376	INS	PRECISE	304	0	0	0/0	0/0	0/0	0/1	0/0	0/0	0/0	0/0	0/0	0/0	0/0	0/0	0/0	0/0
Chromosome1-2319529	INS	PRECISE	51	0	0	0/0	0/0	0/0	0/0	0/0	0/0	0/0	0/0	0/0	0/0	0/0	0/0	0/0	0/1
Chromosome1-2321561	INS	PRECISE	1390	0	0.535	0/1	1/1	0/0	0/0	0/0	0/0	0/0	1/1	0/0	1/1	1/1	1/1	1/1	0/0
Chromosome1-2321743	DEL	PRECISE	1242705	0	0	0/0	0/1	0/1	1/1	1/1	0/1	0/1	0/0	0/1	0/0	0/1	0/0	0/1	0/0
Chromosome1-2322026	DEL	PRECISE	218	0	0	0/0	0/1	0/1	1/1	1/1	1/1	0/1	0/1	1/1	0/1	0/0	0/0	0/0	0/0
Chromosome1-2322110	INS	PRECISE	832	0	0	0/0	0/0	0/0	0/0	0/0	0/0	0/0	0/0	0/0	0/0	0/0	0/0	0/0	1/1
Chromosome1-2322653	DEL	PRECISE	54	0	0	0/1	0/1	0/0	0/0	0/0	0/0	0/0	0/1	0/0	0/1	0/1	0/1	0/1	0/0
Chromosome1-2323246	DEL	PRECISE	33244	0.316	0.316	0/0	0/1	0/0	0/0	0/0	0/0	0/0	0/0	0/0	0/1	0/0	0/0	0/0	0/0
Chromosome1-2323246	DEL	PRECISE	19378	0	0	0/0	0/0	0/0	0/1	0/0	0/0	0/0	0/0	0/0	0/0	0/0	0/0	0/0	0/0
Chromosome1-2325229	DEL	PRECISE	13417	0	0	0/0	0/0	0/0	0/1	0/0	0/0	0/0	0/0	0/0	0/0	0/0	0/0	0/0	0/0
Chromosome1-2327110	INS	PRECISE	69	0	0	0/0	0/0	0/0	0/0	0/0	0/0	0/0	0/0	0/0	0/0	0/0	0/1	0/0	0/0
Chromosome1-2327670	INS	PRECISE	98	0	0	0/0	0/0	0/0	0/0	0/0	0/0	0/0	0/0	0/0	0/0	0/0	0/0	0/0	0/1
Chromosome1-2328768	INS	PRECISE	1558	0	0	0/0	0/0	0/0	0/0	0/0	0/0	0/0	0/0	0/0	0/0	0/0	0/0	0/0	0/1
Chromosome1-2328838	DEL	PRECISE	178	0	0	0/0	0/1	0/1	0/1	1/1	1/1	1/1	0/1	0/1	0/1	0/0	0/0	0/0	0/1
Chromosome1-2329047	DEL	PRECISE	112	0	0	0/0	0/0	0/0	0/0	0/0	0/0	0/0	0/0	0/0	0/0	0/0	0/1	0/0	0/0
Chromosome1-2330685	INS	PRECISE	60	0	0.354	0/1	0/1	0/0	0/1	0/0	0/0	0/0	0/1	0/0	0/1	0/1	0/1	0/1	0/0
Chromosome1-2330752	DEL	PRECISE	80	0	0	0/1	1/1	0/1	1/1	1/1	1/1	1/1	1/1	0/1	1/1	0/1	0/1	0/1	0/0
Chromosome1-2331052	INS	PRECISE	7900	0	0	0/0	0/0	0/0	0/0	0/0	0/0	0/0	0/0	0/0	0/0	0/1	0/0	0/0	0/0
Chromosome1-2331148	INS	PRECISE	5014	0	0	0/0	0/0	0/0	0/0	0/0	0/0	0/0	0/0	0/0	0/0	0/0	1/1	0/0	0/0
Chromosome1-2331176	INS	PRECISE	8968	0	0	0/0	0/0	0/0	0/0	0/0	0/0	0/0	0/0	0/0	0/0	0/0	0/0	0/0	0/0
Chromosome1-2331195	INS	PRECISE	72	0	0	0/0	0/0	0/0	0/0	0/0	0/0	0/0	0/0	0/0	0/0	0/0	0/0	0/0	1/1
Chromosome1-2331391	INS	PRECISE	3383	0	0	0/0	0/0	0/0	0/1	0/0	0/0	0/0	0/0	0/0	0/0	0/0	0/0	0/0	0/0
Chromosome1-2331392	BND	PRECISE	0	0	0	0/0	0/1	0/0	0/1	0/1	0/0	0/0	0/1	0/1	0/0	0/0	0/0	0/0	0/0
Chromosome1-2333001	INS	PRECISE	709	0	0	0/0	0/0	0/0	0/0	0/0	0/0	0/0	0/0	0/1	0/0	0/0	0/0	0/0	0/0
Chromosome1-2333071	DEL	PRECISE	4974	0	0	0/1	1/1	0/1	1/1	1/1	1/1	1/1	1/1	1/1	1/1	1/1	1/1	1/1	1/1
Chromosome1-2333735	DEL	PRECISE	304	0	0	0/0	0/0	0/0	0/0	0/0	0/0	0/0	0/0	0/1	0/0	0/1	0/1	0/1	1/1
Chromosome1-2333922	INS	PRECISE	5174	0	0	0/0	0/0	0/0	0/0	0/0	0/0	0/0	0/0	0/1	0/0	0/0	0/0	0/0	0/0
Chromosome1-2334214	DEL	PRECISE	53	0	0	0/0	0/0	0/0	0/0	0/0	0/0	0/0	0/0	0/1	0/0	0/1	0/1	0/1	1/1
Chromosome1-2335872	INS	PRECISE	622	0	0.577	0/0	0/0	0/0	0/0	0/0	0/0	0/0	0/0	0/0	0/0	0/1	0/0	0/1	1/1
Chromosome1-2335994	DEL	PRECISE	918	0.363	0.363	0/1	1/1	0/1	1/1	1/1	1/1	1/1	1/1	1/1	1/1	1/1	1/1	1/1	1/1
Chromosome1-2336481	DEL	PRECISE	383	0	0	0/0	0/0	0/0	0/0	0/0	0/0	0/1	0/1	0/0	0/0	0/0	0/0	0/0	0/0
Chromosome1-2336561	DEL	PRECISE	162	0	0	0/0	0/0	0/0	0/0	0/0	0/0	0/0	0/0	0/1	0/0	0/0	0/0	0/0	0/0
Chromosome1-2336633	INS	PRECISE	177	0	0	0/0	0/0	0/0	0/1	1/1	1/1	0/0	0/0	0/1	0/0	0/0	0/0	0/0	0/0
Chromosome1-2336670	DEL	PRECISE	485	0	0	0/0	0/0	0/0	0/1	1/1	1/1	0/0	0/0	0/1	0/0	0/0	0/0	0/0	0/0
Chromosome1-2336736	DEL	PRECISE	57	0	0	0/0	0/0	0/0	0/0	0/0	0/0	0/0	0/0	0/0	0/0	0/0	0/0	0/0	0/1
Chromosome1-2336878	INS	PRECISE	75	0	0.333	0/0	0/0	0/0	0/0	0/0	0/0	0/1	1/1	0/0	0/1	1/1	0/0	1/1	0/1
Chromosome1-2337105	INS	PRECISE	3387	0	0	0/0	0/0	0/0	0/0	0/0	0/0	0/0	0/0	0/1	0/0	0/0	0/0	0/0	0/0
Chromosome1-2337119	DEL	PRECISE	7629	0	0	0/0	0/0	0/0	0/0	0/0	0/0	0/0	0/0	0/0	0/0	0/0	1/1	0/0	0/0
Chromosome1-2337194	INS	PRECISE	81	0	0.439	0/0	0/1	0/0	1/1	1/1	1/1	0/1	1/1	1/1	0/1	1/1	0/0	1/1	0/1
Chromosome1-2337526	DEL	PRECISE	167	0	0	0/0	1/1	0/1	1/1	1/1	1/1	1/1	0/0	1/1	1/1	0/0	0/0	0/0	0/0
Chromosome1-2338836	DEL	PRECISE	1197	0	0	0/0	0/0	0/0	0/0	0/0	0/0	0/0	0/0	0/0	0/0	0/0	1/1	0/0	0/0
Chromosome1-2339358	INS	PRECISE	1310	0	0	0/0	0/0	0/0	0/0	0/0	0/0	0/0	0/0	0/0	0/0	0/0	1/1	0/0	0/0
Chromosome1-2341123	DEL	PRECISE	1317	0	0	0/0	0/0	0/0	0/0	0/0	0/0	0/0	0/0	0/0	0/0	1/1	1/1	1/1	0/0
Chromosome1-2341402	DEL	PRECISE	188	0	0	0/0	0/0	0/0	0/0	0/0	0/0	0/0	0/0	0/0	0/0	1/1	1/1	1/1	0/0
Chromosome1-2341737	INS	PRECISE	2206	0	0.816	0/0	0/0	0/0	0/0	0/0	0/0	0/0	0/0	0/0	0/0	1/1	1/1	1/1	1/1
Chromosome1-2343780	INS	PRECISE	75	0	0	0/1	1/1	0/0	1/1	1/1	0/0	1/1	1/1	0/1	1/1	0/0	0/0	0/0	0/0
Chromosome1-2344250	DEL	PRECISE	7018	0	0	0/1	1/1	0/0	1/1	1/1	0/0	1/1	1/1	0/1	1/1	0/0	0/0	0/0	0/0
Chromosome1-2344372	INS	PRECISE	3872	0	0	0/0	0/0	0/0	0/0	0/0	0/0	0/0	0/0	0/1	0/0	0/0	0/0	0/0	0/0
Chromosome1-2344577	BND	PRECISE	0	0	0	0/0	0/0	0/0	0/0	0/0	0/0	0/0	0/0	0/0	0/0	0/1	0/0	0/1	0/0
Chromosome1-2344893	DEL	PRECISE	87848	0	0	0/0	0/0	0/0	0/0	0/0	0/0	0/0	0/0	0/0	0/0	0/0	0/0	0/0	1/1
Chromosome1-2345124	DEL	PRECISE	162	0	0	0/0	0/1	0/0	1/1	1/1	0/0	1/1	0/1	1/1	0/1	0/0	0/0	0/0	0/0
Chromosome1-2346924	DEL	PRECISE	1325	0.577	0.577	0/0	0/0	0/0	0/0	0/0	0/0	0/0	0/0	1/1	0/0	0/0	1/1	0/0	1/1
Chromosome1-2347200	DEL	PRECISE	189	0	0	0/0	0/0	0/0	0/0	0/0	0/0	0/0	0/0	1/1	0/0	0/0	1/1	0/0	1/1
Chromosome1-2347421	DEL	PRECISE	3102	0	0	0/0	0/0	0/0	0/0	0/0	0/0	0/0	0/0	1/1	0/0	0/0	0/0	0/0	0/0
Chromosome1-2347788	INS	PRECISE	55	0	0	0/0	0/0	0/0	0/0	0/0	0/0	0/0	0/0	1/1	0/0	0/0	1/1	0/0	1/1
Chromosome1-2347916	DEL	PRECISE	8239	0	0	0/0	0/0	0/0	0/0	0/0	0/0	0/0	0/0	1/1	0/0	0/0	0/0	0/0	0/0
Chromosome1-2349008	DEL	PRECISE	4802	0	0	0/0	0/0	0/0	0/0	0/0	0/0	0/0	0/0	1/1	0/0	0/0	0/0	0/0	0/0
Chromosome1-2349498	INS	PRECISE	103	0	0	0/0	0/0	0/0	0/0	0/0	0/0	0/0	0/0	1/1	0/0	0/0	0/0	0/0	0/0
Chromosome1-2350416	INS	PRECISE	143	0	0	0/0	0/0	0/0	0/0	0/0	0/0	0/0	0/0	1/1	0/0	0/0	0/0	0/0	0/0
Chromosome1-2350432	DEL	PRECISE	4778	0	0	0/0	0/0	0/0	0/0	0/0	0/0	0/0	0/0	1/1	0/0	0/0	0/0	0/0	0/0
Chromosome1-2352381	DEL	PRECISE	162	0	0	0/0	1/1	0/1	0/0	0/0	0/0	1/1	1/1	0/0	1/1	1/1	0/0	1/1	0/0
Chromosome1-2352522	DEL	PRECISE	143	0	0	0/0	1/1	0/1	0/0	0/0	0/0	1/1	1/1	0/0	1/1	1/1	0/0	1/1	0/0
Chromosome1-2353528	BND	PRECISE	0	0	0	0/0	0/0	0/0	0/0	0/0	0/0	0/0	0/0	0/0	0/1	0/0	0/1	0/0	0/0
Chromosome1-2353805	DEL	PRECISE	2045	0	0	0/0	0/0	0/0	0/0	0/0	0/0	0/0	0/0	0/0	0/0	0/1	1/1	0/1	0/0
Chromosome1-2354274	DEL	PRECISE	963	0	0	0/0	0/0	0/0	0/0	0/0	0/0	0/0	0/0	0/1	0/0	0/1	0/1	0/1	1/1
Chromosome1-2354567	INS	PRECISE	114	0	0	0/0	0/0	0/0	0/0	0/0	0/0	0/0	0/0	0/1	0/0	0/1	0/1	0/1	0/1
Chromosome1-2354634	DEL	PRECISE	1769	0	0	0/0	0/0	0/0	0/0	0/0	0/0	0/1	0/1	0/0	0/0	0/0	0/0	0/0	0/0
Chromosome1-2355099	DEL	PRECISE	962	0	0	0/0	0/0	0/0	0/0	0/0	0/0	0/1	0/1	0/0	0/0	0/0	0/0	0/0	0/0
Chromosome1-2355300	INS	PRECISE	75	0	0.378	0/1	0/1	0/0	0/0	0/0	0/0	0/0	0/0	0/0	0/1	0/1	0/1	0/1	0/0
Chromosome1-2355526	DEL	PRECISE	108	0	0	0/1	0/1	0/0	0/0	0/0	0/0	0/0	0/0	0/0	0/0	0/1	0/1	0/1	0/0
Chromosome1-2356084	DEL	PRECISE	73	0	0	0/0	0/0	0/0	0/0	0/0	0/0	0/0	0/0	0/0	0/0	0/1	0/0	0/1	0/1
Chromosome1-2356560	INS	PRECISE	6426	0	0	0/0	0/0	0/0	0/0	0/0	0/0	0/0	0/0	0/1	0/0	0/0	0/0	0/0	0/0

Chromosome1-2357260	DEL	PRECISE	56	0	0	0/0	0/0	0/0	0/0	0/0	0/0	0/0	0/0	0/1	0/0	0/1	0/0	0/1	1/1
Chromosome1-2357276	DEL	PRECISE	9419	0.548	0	0/0	0/0	0/0	0/0	0/0	0/0	0/0	0/0	0/1	0/0	0/1	0/1	0/1	1/1
Chromosome1-2357504	DEL	PRECISE	458	0	0	0/0	0/0	0/0	0/0	0/0	0/0	0/0	0/0	0/0	0/0	0/0	0/0	0/0	0/1
Chromosome1-2357840	INS	PRECISE	99	0	0.469	0/1	1/1	0/1	1/1	1/1	1/1	1/1	1/1	0/1	1/1	0/1	0/1	0/1	0/1
Chromosome1-2358094	DEL	PRECISE	62	0	0	0/0	0/0	0/0	0/0	0/0	0/0	0/0	0/0	0/0	0/0	0/0	0/0	0/0	0/1
Chromosome1-2358351	DEL	PRECISE	885	0	0	0/1	1/1	0/1	1/1	1/1	1/1	1/1	1/1	1/1	1/1	1/1	1/1	1/1	1/1
Chromosome1-2358616	DEL	PRECISE	8455	0	0	0/0	1/1	0/1	0/0	0/0	0/0	1/1	1/1	0/0	1/1	0/0	0/0	0/0	0/1
Chromosome1-2358643	DEL	PRECISE	6140	0	0	0/0	0/0	0/0	0/0	0/0	0/0	0/0	0/0	0/0	0/0	1/1	0/0	1/1	1/1
Chromosome1-2359549	DEL	PRECISE	137	0.756	0	0/1	0/0	0/0	0/0	0/0	0/0	0/0	0/1	0/0	0/1	0/1	0/1	0/1	0/0
Chromosome1-2361337	DEL	PRECISE	98	0.267	0.267	0/1	1/1	0/0	1/1	1/1	1/1	1/1	1/1	1/1	1/1	1/1	1/1	1/1	1/1
Chromosome1-2361470	BND	PRECISE	0	0	0	0/0	0/0	0/0	0/0	0/0	0/0	0/0	0/0	0/1	0/0	0/0	0/0	0/0	0/1
Chromosome1-2361497	INS	PRECISE	5841	0	0.707	0/0	1/1	0/1	0/0	0/0	0/0	0/0	0/0	0/0	0/0	0/0	0/0	0/0	0/0
Chromosome1-2363308	DEL	PRECISE	12059	0	0	0/1	1/1	0/1	1/1	1/1	1/1	1/1	1/1	0/1	1/1	0/1	1/1	1/1	1/1
Chromosome1-2363308	BND	PRECISE	0	0	0	0/0	0/0	0/0	0/0	0/1	0/0	0/1	0/0	0/0	0/0	0/0	0/0	0/0	0/0
Chromosome1-2363308	BND	PRECISE	0	0	0	0/0	0/0	0/0	0/0	0/0	0/0	0/0	0/0	0/0	0/0	0/1	0/0	0/0	0/0
Chromosome1-2364514	BND	PRECISE	0	0	0	0/0	0/0	0/0	0/0	0/1	0/0	0/0	0/0	0/1	0/0	1/1	0/0	0/0	0/0
Chromosome1-2364858	DEL	PRECISE	4496	0	0	0/1	1/1	0/1	1/1	1/1	1/1	1/1	1/1	1/1	1/1	1/1	1/1	1/1	1/1
Chromosome1-2365787	DEL	PRECISE	66	0	0	0/0	0/0	0/0	0/0	0/0	0/0	0/1	0/1	0/1	0/0	0/0	0/1	0/0	0/0
Chromosome1-2365866	DEL	PRECISE	288	0	0	0/0	0/0	0/0	0/0	0/0	0/0	0/0	0/0	0/0	0/0	0/0	0/0	0/0	1/1
Chromosome1-2367593	DEL	PRECISE	436	0	0	0/0	0/0	0/0	0/0	0/0	0/0	1/1	0/1	0/0	0/0	0/0	0/0	0/0	1/1
Chromosome1-2370226	DEL	PRECISE	98	0	0	0/0	0/1	0/1	0/0	0/0	0/0	0/1	0/0	0/0	0/1	0/0	0/0	0/0	0/0
Chromosome1-2371397	INS	PRECISE	6870	0	0	0/0	0/0	0/0	0/1	0/0	0/0	0/0	0/0	0/0	0/0	0/0	0/0	0/0	0/0
Chromosome1-2372026	DEL	PRECISE	190	0	0	0/1	0/1	0/0	1/1	1/1	1/1	0/0	0/1	1/1	0/1	0/1	1/1	0/1	0/0
Chromosome1-2372189	DEL	PRECISE	16118	0	0	0/0	0/0	0/0	0/0	0/1	0/0	0/0	0/0	0/1	0/0	0/0	0/1	0/1	0/0
Chromosome1-2372196	BND	PRECISE	0	0	0	0/0	0/1	0/0	0/0	0/0	0/0	0/0	0/0	0/0	0/1	0/0	0/0	0/0	0/0
Chromosome1-2372702	DEL	PRECISE	1229	0	0	0/0	0/0	0/0	0/0	0/0	0/0	0/0	0/0	0/0	0/0	0/1	0/0	0/1	0/1
Chromosome1-2373086	INS	PRECISE	171	0	0	0/0	0/1	0/1	0/0	0/0	0/0	0/1	0/0	0/0	0/1	0/0	0/0	0/0	0/0
Chromosome1-2373660	DEL	PRECISE	1409	0	0	0/0	0/0	0/0	0/1	0/0	0/1	0/0	0/0	0/0	0/0	0/1	0/0	0/0	0/0
Chromosome1-2374182	DEL	PRECISE	232	0	0	0/0	0/0	0/0	0/0	0/0	0/0	0/0	0/0	0/1	0/0	0/0	0/0	0/0	0/0
Chromosome1-2374220	DEL	PRECISE	1920	0	0	0/0	0/0	0/0	0/0	0/0	0/0	0/0	0/0	0/1	0/0	0/0	0/0	0/0	0/0
Chromosome1-2374437	DEL	PRECISE	923	0	0	0/0	0/0	0/0	0/0	0/0	0/0	0/0	0/0	0/1	0/0	0/0	0/0	0/0	0/0
Chromosome1-2374480	DEL	PRECISE	1516	0	0	0/0	0/0	0/0	0/1	0/0	0/0	0/1	0/0	0/0	0/0	0/0	0/0	0/1	0/0
Chromosome1-2374578	INS	PRECISE	1542	0	0	0/0	0/0	0/0	0/1	0/0	0/0	0/0	0/0	0/0	0/0	0/0	0/0	0/0	0/1
Chromosome1-2374788	DEL	PRECISE	60	0	0	0/0	0/0	0/0	0/0	0/0	0/0	0/0	0/0	0/1	0/0	0/1	0/0	0/1	0/1
Chromosome1-2375257	BND	PRECISE	0	0	0	0/0	0/0	0/0	0/1	0/0	0/0	0/0	0/0	0/0	0/0	0/0	0/0	0/0	0/0
Chromosome1-2375291	INS	PRECISE	380	0	0	0/1	1/1	0/1	1/1	1/1	1/1	1/1	1/1	1/1	1/1	0/1	1/1	0/1	0/0
Chromosome1-2376126	INS	PRECISE	12848	0	0	0/0	0/0	0/0	0/0	0/0	0/0	0/0	0/0	0/0	0/0	0/0	0/0	1/1	0/0
Chromosome1-2376231	INS	PRECISE	1022	0	0	0/0	0/0	0/0	0/0	0/0	0/0	0/0	0/0	0/0	0/0	0/0	0/1	0/0	0/0
Chromosome1-2376798	BND	PRECISE	0	0	0	0/0	0/0	0/1	0/0	0/0	0/0	0/1	0/1	0/0	0/0	0/0	0/0	0/0	0/0
Chromosome1-2377444	INS	PRECISE	1343	0	0.899	0/1	1/1	0/1	1/1	1/1	1/1	0/1	0/1	1/1	1/1	1/1	0/1	1/1	0/0
Chromosome1-2377619	DEL	PRECISE	52	0	0	0/0	0/0	0/0	0/0	0/0	0/0	0/0	0/0	0/0	0/0	0/0	0/0	0/0	1/1
Chromosome1-2377674	INS	PRECISE	2120	0	0	0/0	0/0	0/0	0/0	0/0	0/0	0/0	0/0	0/0	0/0	0/0	0/1	0/0	0/0
Chromosome1-2377836	DEL	PRECISE	119	0	0	0/0	0/0	0/0	0/0	0/0	0/0	0/0	0/0	0/0	0/0	0/0	0/1	0/0	0/0
Chromosome1-2377930	DEL	PRECISE	12670	0	0	0/1	1/1	0/1	1/1	1/1	1/1	0/1	0/1	1/1	1/1	1/1	1/1	1/1	0/0
Chromosome1-2378493	BND	PRECISE	0	0	0	0/0	0/0	0/0	0/0	0/0	0/0	0/1	0/1	0/0	0/0	0/0	0/0	0/0	0/0
Chromosome1-2379270	DEL	PRECISE	50	0	0	0/0	0/0	0/0	0/0	0/0	0/0	0/0	0/0	0/0	0/0	0/0	0/1	0/0	0/0
Chromosome1-2379375	BND	PRECISE	0	0	0	0/0	0/0	0/0	0/0	0/0	0/0	0/0	0/0	0/0	0/0	0/0	0/0	0/0	1/1
Chromosome1-2379678	INS	PRECISE	6283	0	0	0/0	0/0	0/0	0/0	0/0	0/0	0/0	0/0	0/0	0/0	0/0	0/0	0/0	1/1
Chromosome1-2379678	BND	PRECISE	0	0	0	0/0	0/0	0/0	0/0	0/0	0/0	0/0	0/0	0/0	0/0	0/0	0/0	0/0	1/1
Chromosome1-2380454	INS	PRECISE	533	0	0	0/0	0/0	0/0	0/0	0/0	0/0	0/0	0/0	0/0	0/0	0/0	0/1	0/0	0/0
Chromosome1-2380702	DEL	PRECISE	67	0	0	0/1	0/1	0/0	0/1	0/0	0/0	0/0	0/1	0/0	0/1	0/1	0/1	0/1	0/0
Chromosome1-2380925	INS	PRECISE	5414	0	0	0/0	0/0	0/0	0/0	0/0	0/0	0/0	0/0	1/1	0/0	0/0	0/0	0/0	0/0
Chromosome1-2381098	BND	PRECISE	0	0	0	0/0	0/0	0/0	0/0	0/0	0/0	0/0	0/0	0/0	0/0	0/1	0/0	0/0	0/0
Chromosome1-2381384	INS	PRECISE	380	0	0	0/0	0/0	0/0	0/0	0/0	0/0	0/0	0/0	0/1	0/0	0/0	0/0	0/0	0/0
Chromosome1-2381462	INS	PRECISE	4891	0	0	0/0	0/0	0/0	0/0	0/0	0/0	0/0	0/0	0/1	0/0	0/0	0/0	0/0	0/0
Chromosome1-2381685	INS	PRECISE	63	0	0	0/0	0/0	0/0	0/0	0/0	0/0	0/1	0/1	0/0	0/0	0/0	0/0	0/0	0/0
Chromosome1-2382075	INS	PRECISE	115	0	0.5	0/0	0/0	0/0	0/1	1/1	1/1	0/0	0/0	1/1	0/0	0/0	0/0	0/0	0/0
Chromosome1-2382774	BND	PRECISE	0	0	0	0/0	0/1	0/0	0/0	0/0	0/0	0/0	0/0	0/0	0/0	0/1	0/0	0/0	0/0
Chromosome1-2382774	BND	PRECISE	0	0	0	0/0	0/0	0/0	0/0	0/0	0/0	0/0	0/0	0/1	0/0	0/0	0/0	0/0	0/0
Chromosome1-2384548	INS	PRECISE	211	0	0	0/0	0/0	0/0	0/1	1/1	1/1	0/0	0/0	0/1	0/0	0/0	0/0	0/0	0/0
Chromosome1-2384651	INS	PRECISE	335	0	0.577	0/0	0/0	0/0	0/0	0/0	0/0	0/0	0/0	0/0	0/0	0/1	0/0	0/1	1/1
Chromosome1-2384742	DEL	PRECISE	662	0	0	0/0	0/0	0/0	0/0	0/0	0/0	0/0	0/0	0/1	0/0	0/1	0/1	0/1	0/1
Chromosome1-2384950	DEL	PRECISE	222	0.408	0.408	0/0	0/0	0/0	0/1	1/1	1/1	0/1	0/1	0/1	0/0	0/0	0/0	0/0	0/0
Chromosome1-2385198	INS	PRECISE	454	0.756	0.69	0/1	0/1	0/0	0/0	0/0	0/0	0/0	0/1	0/0	0/1	0/1	0/1	0/1	0/0
Chromosome1-2385331	DEL	PRECISE	994	0	0	0/0	0/0	0/0	0/0	0/0	0/0	0/0	0/0	0/1	0/0	0/1	0/1	0/1	0/1
Chromosome1-2386113	INS	PRECISE	471	0	0.548	0/0	0/0	0/0	0/0	0/0	0/0	0/0	0/0	0/1	0/0	0/1	0/1	0/1	0/1
Chromosome1-2386379	DEL	PRECISE	52	0	0	0/0	0/0	0/0	0/0	0/0	0/0	0/0	0/0	0/0	0/0	0/1	0/0	0/1	0/1
Chromosome1-2387295	INS	PRECISE	1016	0	0	0/0	0/1	0/1	0/0	0/0	0/0	0/1	0/0	0/0	0/1	0/0	0/0	0/0	0/0
Chromosome1-2388574	DEL	PRECISE	148	0	0	0/0	0/0	0/0	0/0	0/0	0/0	0/0	0/0	0/0	0/0	0/1	0/1	0/0	0/0
Chromosome1-2388992	DEL	PRECISE	53	0	0	0/0	0/0	0/0	0/0	0/0	0/0	0/0	0/0	0/0	0/0	0/1	0/1	0/0	0/1
Chromosome1-2389078	DEL	PRECISE	539	0	0	0/0	0/0	0/0	0/0	0/0	0/0	0/0	0/0	0/0	0/0	0/1	0/1	0/0	0/1
Chromosome1-2389199	DEL	PRECISE	215	0	0	0/0	0/0	0/0	0/0	0/0	0/0	0/0	0/0	0/0	0/0	0/1	0/1	0/0	0/1
Chromosome1-2389247	INS	PRECISE	625	0	0.76	0/1	1/1	0/1	1/1	1/1	1/1	1/1	1/1	1/1	1/1	0/1	0/1	1/1	0/0

Chromosome1-2389582	INS	PRECISE	1469	0	0	0/0	0/0	0/0	0/0	0/0	0/0	0/0	0/0	0/0	0/0	0/0	0/0	0/0	0/1
Chromosome1-2389943	DEL	PRECISE	1977	0	0	0/0	0/0	0/0	0/0	0/0	0/0	0/0	0/0	0/0	0/0	0/1	0/1	0/0	0/1
Chromosome1-2390160	DEL	PRECISE	140	0	0	0/0	0/0	0/0	0/0	0/0	0/0	0/0	0/0	0/0	0/0	0/1	0/1	0/0	0/1
Chromosome1-2390333	DEL	PRECISE	950	0	0	0/0	0/0	0/0	0/0	0/0	0/0	0/0	0/0	0/0	0/0	0/1	0/1	0/0	0/1
Chromosome1-2390872	INS	PRECISE	545	0	0.48	0/1	0/0	1/1	0/0	0/0	0/0	0/0	0/0	0/0	0/0	0/0	0/0	0/0	0/0
Chromosome1-2391284	INS	PRECISE	67	0	0.277	0/1	1/1	0/1	1/1	1/1	1/1	1/1	1/1	1/1	1/1	1/1	1/1	1/1	0/0
Chromosome1-2391660	DEL	PRECISE	131	0	0	0/0	0/0	0/0	0/0	0/0	0/0	0/0	0/0	0/0	0/0	0/0	0/0	0/0	0/1
Chromosome1-2391680	INS	PRECISE	353	0	0.707	0/0	0/0	0/0	0/0	0/0	0/0	0/1	0/1	0/0	0/0	0/0	0/0	0/0	0/0
Chromosome1-2392760	DEL	PRECISE	791	0	0	0/0	0/0	0/0	0/0	0/0	0/0	0/0	0/0	0/1	0/0	0/1	0/1	0/1	0/1
Chromosome1-2393055	INS	PRECISE	128	0	0.392	0/1	1/1	0/1	1/1	1/1	1/1	1/1	1/1	1/1	1/1	1/1	1/1	1/1	1/1
Chromosome1-2393672	DEL	PRECISE	73	0	0	0/0	0/0	0/0	0/0	0/0	0/0	0/0	0/0	0/1	0/0	0/1	0/0	0/1	0/1
Chromosome1-2394480	BND	PRECISE	0	0	0	0/1	0/0	0/0	0/0	0/1	0/0	0/0	0/0	0/0	0/0	0/0	0/0	0/0	0/0
Chromosome1-2395052	DEL	PRECISE	4629	0	0	0/0	0/0	0/0	0/0	0/0	0/0	0/0	0/0	0/0	0/0	0/0	0/0	0/0	0/1
Chromosome1-2395519	DEL	PRECISE	549	0	0	0/0	0/0	0/0	0/0	0/0	0/0	0/0	0/0	0/0	0/0	0/0	0/0	0/0	0/1
Chromosome1-2396789	INS	PRECISE	353	0	0.816	0/0	0/0	0/0	0/0	0/0	0/0	0/0	0/0	0/1	0/0	0/1	0/1	0/0	0/1
Chromosome1-2397020	INS	PRECISE	274	0	0.447	0/0	0/0	0/0	0/0	0/0	0/0	0/0	0/0	0/0	0/0	0/0	0/1	0/1	0/1
Chromosome1-2397423	DEL	PRECISE	9560	0.548	0.548	0/0	0/0	0/0	0/0	0/0	0/0	0/0	0/0	0/1	0/0	0/1	0/1	0/1	0/1
Chromosome1-2398629	DEL	PRECISE	17529	0	0	0/0	0/1	0/1	0/0	0/1	1/1	0/1	0/0	1/1	0/1	0/1	0/1	0/1	1/1
Chromosome1-2400489	BND	PRECISE	0	0	0	0/0	0/1	0/1	0/0	0/1	1/1	0/0	0/0	1/1	0/1	0/0	0/0	0/0	0/0
Chromosome1-2400700	DEL	PRECISE	58	0	0	0/1	0/1	0/0	1/1	0/1	0/1	1/1	1/1	0/0	0/1	0/1	0/1	0/1	0/0
Chromosome1-2400971	INS	PRECISE	75	0	0	0/1	1/1	0/1	1/1	1/1	1/1	1/1	1/1	1/1	1/1	1/1	1/1	1/1	1/1
Chromosome1-2402519	BND	PRECISE	0	0	0	0/1	0/0	0/0	0/1	0/0	0/0	0/0	0/0	0/0	0/0	0/0	0/0	0/1	0/0
Chromosome1-2404060	INS	PRECISE	377	0	0.707	0/0	0/0	0/0	0/0	0/0	0/0	0/0	0/0	0/1	0/0	0/1	0/1	0/1	0/1
Chromosome1-2404099	DEL	PRECISE	6444	0	0	0/0	0/0	0/0	0/0	0/0	0/0	0/0	0/0	0/1	0/0	0/1	0/1	0/1	1/1
Chromosome1-2404618	INS	PRECISE	12555	0	0	0/0	0/0	0/0	0/0	0/0	0/0	0/0	1/1	0/0	0/0	0/0	0/0	0/0	0/0
Chromosome1-2404618	BND	PRECISE	0	0	0	0/0	0/0	0/0	0/0	0/0	0/0	0/1	0/1	0/0	0/0	0/0	0/0	0/0	0/0
Chromosome1-2404706	INS	PRECISE	125	0	0	0/0	0/0	0/0	0/0	0/0	0/0	1/1	0/0	0/0	0/0	0/0	0/0	0/0	0/0
Chromosome1-2405647	DEL	PRECISE	873	0	0	0/0	0/0	0/0	0/0	0/0	0/0	0/0	0/0	0/1	0/0	1/1	1/1	0/1	1/1
Chromosome1-2406547	INS	PRECISE	50	0	0.447	0/0	0/0	0/0	0/0	0/0	0/0	0/0	0/0	0/1	0/0	0/1	1/1	0/1	0/1
Chromosome1-2406577	INS	PRECISE	10013	0	0	0/0	0/0	0/0	0/0	0/0	0/1	0/0	0/0	0/0	0/0	0/0	0/0	0/0	0/0
Chromosome1-2406795	INS	PRECISE	5769	0	0	0/0	0/0	0/0	0/0	0/0	0/0	0/0	0/0	0/0	0/0	0/0	0/0	0/0	1/1
Chromosome1-2406795	BND	PRECISE	0	0	0	0/0	0/0	0/0	0/0	0/0	0/0	0/0	0/0	0/0	0/0	0/0	0/0	0/0	0/1
Chromosome1-2406928	DEL	PRECISE	427	0	0	0/0	0/0	0/0	0/0	0/0	0/0	0/0	0/0	0/1	0/0	0/1	1/1	0/1	1/1
Chromosome1-2407157	DEL	PRECISE	90	0	0	0/0	0/0	0/0	0/0	0/0	0/0	0/0	0/0	0/1	0/0	0/1	1/1	0/1	0/1
Chromosome1-2407230	INS	PRECISE	952	0	0	0/0	0/0	0/0	0/0	0/0	0/0	0/0	0/0	0/0	0/0	0/0	0/0	0/0	0/1
Chromosome1-2407878	DEL	PRECISE	531	0	0	0/0	0/0	0/0	0/0	0/0	0/0	0/0	0/0	0/1	0/0	0/1	0/1	0/1	0/1
Chromosome1-2407927	INS	PRECISE	149	0	0	0/0	0/0	0/0	0/0	0/0	0/0	0/0	0/0	0/0	0/0	0/0	0/0	0/0	0/1
Chromosome1-2408107	INS	PRECISE	7734	0	0	0/0	0/0	0/0	0/0	0/0	0/0	0/0	0/0	0/0	0/0	0/0	0/0	0/0	0/1
Chromosome1-2408107	BND	PRECISE	0	0	0	0/0	0/0	0/0	0/0	0/0	0/0	0/0	0/0	0/0	0/0	0/0	0/0	0/0	0/1
Chromosome1-2408245	INS	PRECISE	299	0	0.535	0/0	0/1	0/1	0/0	0/1	0/1	0/1	0/0	0/1	0/1	0/0	0/0	0/0	0/0
Chromosome1-2409778	INS	PRECISE	858	0	0	0/0	0/0	0/0	0/0	0/0	0/0	0/0	0/0	0/0	0/0	0/0	0/0	0/0	0/1
Chromosome1-2410379	BND	PRECISE	0	0	0	0/0	0/0	0/0	0/0	0/0	0/0	0/0	0/0	0/1	0/0	0/1	1/1	0/1	1/1
Chromosome1-2410480	BND	PRECISE	0	0	0	0/0	0/0	0/0	0/0	0/0	0/0	0/0	0/0	0/0	0/0	0/0	1/1	0/1	0/0
Chromosome1-2410528	INV	PRECISE	674125	0	0	0/0	0/0	0/0	0/0	0/0	0/0	0/0	0/0	0/0	0/0	0/0	0/0	0/0	0/1
Chromosome1-2411057	DEL	PRECISE	210	0.751	0.376	0/1	0/1	0/0	1/1	0/1	0/1	0/1	1/1	0/0	0/1	1/1	1/1	1/1	1/1
Chromosome1-2411202	DEL	PRECISE	107	0	0	0/0	0/0	0/0	0/0	0/0	0/0	0/0	0/0	0/1	0/0	0/1	1/1	0/1	0/1
Chromosome1-2411333	INS	PRECISE	653	0	0.535	0/0	0/1	0/1	0/0	0/1	0/1	0/1	0/0	0/1	0/1	0/0	0/0	0/0	0/0
Chromosome1-2412271	DEL	PRECISE	76	0	0	0/0	0/0	0/0	0/0	0/0	0/0	0/0	0/0	0/1	0/0	1/1	0/1	0/1	0/1
Chromosome1-2413394	DEL	PRECISE	567	0	0	0/0	0/0	0/0	0/0	0/0	0/0	0/0	0/0	0/1	0/0	0/1	0/1	0/1	0/1
Chromosome1-2413785	DEL	PRECISE	5404	0	0	0/0	0/0	0/0	0/0	0/0	0/0	0/0	0/0	0/1	0/0	0/1	0/1	0/1	0/1
Chromosome1-2414902	INS	PRECISE	4821	0	0	0/0	0/0	0/0	0/0	0/0	0/0	1/1	1/1	0/0	0/0	0/0	0/0	0/0	0/0
Chromosome1-2415252	INS	PRECISE	2358	0	0	0/0	0/0	0/0	0/0	0/0	0/0	0/0	0/0	0/0	0/0	0/0	0/0	0/0	0/1
Chromosome1-2416866	DEL	PRECISE	602	0	0	0/0	0/0	0/0	0/0	0/0	0/0	0/0	0/0	0/1	0/0	0/1	0/1	0/1	1/1
Chromosome1-2417778	INS	PRECISE	81	0	0	0/0	0/0	0/0	0/0	0/0	0/0	0/0	0/0	0/0	0/0	0/1	0/1	0/1	1/1
Chromosome1-2417948	INS	PRECISE	211	0	0.5	0/1	0/1	0/0	0/1	0/0	0/0	0/1	1/1	0/0	0/1	0/1	0/1	0/1	0/0
Chromosome1-2418495	DEL	PRECISE	368	0	0	0/1	1/1	0/1	0/1	1/1	1/1	0/1	0/1	0/1	1/1	0/1	0/1	0/1	0/0
Chromosome1-2419500	INS	PRECISE	268	0	0	0/0	0/0	0/0	0/0	0/0	0/0	0/0	0/0	1/1	0/0	0/0	0/0	0/0	0/0
Chromosome1-2419726	BND	PRECISE	0	0	0	0/0	0/1	0/0	0/0	0/0	0/0	0/0	1/1	0/0	0/0	1/1	0/1	1/1	0/0
Chromosome1-2419760	INS	PRECISE	1390	0	0	0/0	0/0	0/0	0/0	0/0	0/0	0/1	1/1	0/0	0/0	0/0	0/0	0/0	0/0
Chromosome1-2419767	BND	PRECISE	0	0	0	0/0	0/0	0/0	0/0	0/0	0/0	0/0	0/0	0/1	0/0	1/1	0/0	0/0	0/1
Chromosome1-2420050	INS	PRECISE	1327	0	0.548	0/0	0/0	0/0	0/0	0/0	0/0	0/0	0/0	1/1	0/0	1/1	0/0	0/0	0/1
Chromosome1-2420685	DEL	PRECISE	50	0	0	0/0	0/0	0/0	0/1	0/1	0/1	0/0	0/0	0/0	0/0	0/0	0/0	0/0	0/0
Chromosome1-2420692	INS	PRECISE	7372	0	0	0/0	0/0	0/0	0/0	0/0	0/0	0/0	0/0	0/0	0/0	0/0	0/0	0/0	0/1
Chromosome1-2422800	INS	PRECISE	1374	0	0	0/0	0/0	0/0	0/0	0/0	0/0	0/0	0/0	0/1	0/0	0/0	0/0	0/0	0/0
Chromosome1-2423941	INS	PRECISE	1260	0	0	0/0	0/0	0/0	0/0	0/0	0/0	0/0	0/0	0/1	0/0	0/0	0/0	0/0	0/0
Chromosome1-2424058	DEL	PRECISE	686	0	0	0/0	0/0	0/0	0/0	0/0	0/0	0/0	0/0	0/0	0/0	0/1	0/1	0/1	0/1
Chromosome1-2424389	INS	PRECISE	138	0	0.5	0/0	0/0	0/0	0/0	0/0	0/0	0/0	0/0	0/0	0/0	0/1	0/1	0/1	0/1
Chromosome1-2424869	DEL	PRECISE	363	0.447	0.447	0/0	0/1	0/1	0/0	0/1	0/0	0/1	0/0	0/1	0/0	0/0	0/0	0/0	0/0
Chromosome1-2425150	DEL	PRECISE	122	0	0	0/0	0/0	0/0	0/0	0/0	0/0	0/1	0/1	0/0	0/0	0/0	0/0	0/0	0/0
Chromosome1-2425550	INS	PRECISE	150	0	0.505	0/1	1/1	0/0	1/1	0/1	1/1	0/1	1/1	0/0	1/1	1/1	1/1	1/1	0/0
Chromosome1-2426175	INS	PRECISE	151	0	0	0/0	0/0	0/0	0/0	0/0	0/0	0/0	0/0	0/1	0/0	0/0	0/0	0/0	0/0
Chromosome1-2426501	DEL	PRECISE	105	0	0	0/0	0/0	0/0	0/0	0/0	0/0	0/0	0/0	0/1	0/0	0/0	0/0	0/0	0/0
Chromosome1-2426951	DEL	PRECISE	66	0	0	0/0	0/0	0/0	0/0	0/0	0/0	0/0	0/0	0/0	0/0	0/1	0/1	0/1	0/1

Chromosome1-2427103	INS	PRECISE	5028	0	0	0/0	0/0	0/0	0/0	0/0	0/0	0/0	0/0	1/1	0/0	0/0	0/0	0/0	0/0
Chromosome1-2428975	INS	PRECISE	19414	0	0	0/0	0/0	0/0	0/0	0/0	0/0	0/0	0/0	0/0	0/0	0/0	0/0	0/0	0/0
Chromosome1-2429420	DEL	PRECISE	1163	0	0	0/1	1/1	0/1	1/1	1/1	1/1	1/1	1/1	1/1	1/1	1/1	1/1	1/1	1/1
Chromosome1-2430247	INS	PRECISE	53	0	0	0/0	0/0	0/0	0/0	0/0	0/0	0/0	0/0	0/0	0/0	0/0	0/0	0/0	0/1
Chromosome1-2431106	DEL	PRECISE	74	0	0	0/0	0/0	0/0	0/0	0/0	0/0	0/0	0/0	0/0	0/0	0/1	0/1	0/1	0/1
Chromosome1-2431337	DEL	PRECISE	331	0	0	0/0	0/0	0/0	0/0	0/0	0/0	0/1	0/1	0/1	0/0	0/1	0/1	0/1	0/1
Chromosome1-2432392	DEL	PRECISE	51	0	0	0/0	0/0	0/0	0/0	0/0	0/0	0/0	0/0	0/1	0/0	0/1	0/1	0/1	0/1
Chromosome1-2433079	DEL	PRECISE	1214	0	0	0/1	1/1	1/1	1/1	1/1	1/1	1/1	1/1	1/1	1/1	1/1	1/1	1/1	1/1
Chromosome1-2435011	INS	PRECISE	1768	0	0	0/0	0/0	0/1	0/0	0/0	0/0	0/0	0/0	0/0	0/0	0/0	0/0	0/0	0/0
Chromosome1-2436948	DEL	PRECISE	130	0	0	0/0	0/0	0/0	0/0	0/0	0/0	0/0	0/0	0/0	0/0	0/0	0/1	0/0	0/0
Chromosome1-2437045	DEL	PRECISE	452	0	0	0/0	0/0	0/0	0/0	0/0	0/0	0/0	0/0	0/0	0/0	0/1	0/0	0/1	0/1
Chromosome1-2437112	INS	PRECISE	160	0	0.577	0/0	0/0	0/0	0/0	0/0	0/0	0/0	0/0	0/0	0/0	0/1	0/0	0/1	0/1
Chromosome1-2437170	DEL	PRECISE	236	0	0	0/0	0/0	0/0	0/0	0/0	0/0	1/1	0/1	0/1	0/0	0/0	0/1	0/0	0/0
Chromosome1-2437336	DEL	PRECISE	613	0.289	0.289	0/1	0/1	0/0	0/1	0/1	0/1	1/1	1/1	0/0	0/1	0/1	0/1	0/1	0/1
Chromosome1-2439264	DEL	PRECISE	1292	0	0	0/0	0/0	0/0	0/0	0/0	0/0	0/0	0/0	0/1	0/0	0/0	0/0	0/0	0/0
Chromosome1-2439613	DEL	PRECISE	135	0	0	0/0	0/0	0/0	0/0	0/0	0/0	0/0	0/0	0/0	0/0	0/0	0/0	0/0	0/1
Chromosome1-2439673	DEL	PRECISE	591	0	0	0/1	0/1	0/0	1/1	0/1	0/1	0/0	0/1	0/1	0/1	1/1	1/1	1/1	0/1
Chromosome1-2439692	BND	PRECISE	0	0	0	0/0	0/0	0/0	0/0	0/0	0/0	0/1	0/1	0/0	0/0	0/0	0/0	0/0	0/0
Chromosome1-2442746	INS	PRECISE	3413	0	0	0/0	0/0	0/0	0/0	0/0	0/0	0/0	0/0	0/0	0/0	0/0	0/0	0/0	1/1
Chromosome1-2443056	DEL	PRECISE	211	0	0	0/0	0/0	0/0	0/0	0/0	0/0	0/0	0/0	0/1	0/0	0/1	0/1	0/1	0/1
Chromosome1-2444090	INS	PRECISE	9017	0	0	0/0	0/0	0/0	0/0	0/0	0/0	0/0	0/0	0/0	0/0	0/0	0/0	0/0	0/0
Chromosome1-2444172	INS	PRECISE	676	0	0	0/0	0/0	0/0	0/0	0/0	0/0	0/0	0/0	0/0	0/0	0/0	0/0	0/0	0/1
Chromosome1-2444383	DEL	PRECISE	308	0	0	0/0	0/0	0/0	0/0	0/0	0/0	0/0	0/0	0/0	0/1	0/1	0/1	0/1	0/1
Chromosome1-2444511	INS	PRECISE	1565	0	0	0/0	0/0	0/0	0/0	0/0	0/0	0/0	0/0	0/1	0/0	0/0	0/0	0/0	0/0
Chromosome1-2444878	DEL	PRECISE	680	0	0	0/0	0/0	0/0	0/0	0/0	0/0	0/0	0/0	0/1	0/1	0/1	0/1	0/1	0/1
Chromosome1-2446186	INS	PRECISE	1380	0	0	0/0	0/0	0/0	0/0	0/0	0/0	0/0	0/0	0/0	0/0	0/0	0/1	0/0	0/0
Chromosome1-2447240	INS	PRECISE	6301	0	0	0/0	0/0	0/0	0/0	0/0	0/0	0/0	0/0	0/0	0/0	0/0	1/1	0/0	0/0
Chromosome1-2447563	INS	PRECISE	125	0	0	0/0	0/0	0/0	0/1	0/1	0/1	0/0	0/0	0/0	0/0	0/0	0/0	0/0	0/0
Chromosome1-2447993	DEL	PRECISE	443	0	0	0/1	0/1	0/0	0/0	0/0	0/0	0/1	1/1	0/0	0/1	0/1	0/1	0/1	0/0
Chromosome1-2448460	INS	PRECISE	61	0	0.5	0/0	0/0	0/0	0/0	0/0	0/0	0/0	0/0	0/1	0/0	0/1	0/0	0/1	1/1
Chromosome1-2448549	INS	PRECISE	625	0	0.951	0/1	0/1	0/0	0/0	0/0	0/0	0/0	0/1	0/0	0/1	0/1	0/1	0/1	0/0
Chromosome1-2449986	INS	PRECISE	106	0	0	0/0	0/0	0/0	0/0	0/0	0/0	0/0	0/0	0/0	0/1	0/0	0/0	0/0	0/0
Chromosome1-2450524	DEL	PRECISE	1196	0	0	0/0	0/0	0/0	0/0	0/0	0/0	0/0	0/0	1/1	0/0	1/1	0/0	0/1	0/1
Chromosome1-2451313	INS	PRECISE	65	0	0.408	0/0	0/0	0/0	0/0	0/0	0/0	0/0	0/0	1/1	0/1	0/1	0/1	0/1	0/1
Chromosome1-2451344	DEL	PRECISE	176	0	0	0/0	0/0	0/0	0/0	0/0	0/0	0/0	0/0	1/1	0/1	0/1	0/1	0/1	0/1
Chromosome1-2451537	DEL	PRECISE	65	0	0	0/0	0/0	0/0	0/0	0/0	0/0	0/0	0/0	0/0	0/0	0/0	0/0	0/0	0/1
Chromosome1-2451877	INS	PRECISE	5965	0	0	0/0	0/0	0/0	0/0	0/0	0/0	0/0	0/0	0/0	0/0	0/0	0/0	0/0	1/1
Chromosome1-2453012	INS	PRECISE	56	0	0	0/0	0/0	0/0	0/0	0/0	0/0	0/0	0/0	0/1	0/1	0/1	0/0	0/1	0/1
Chromosome1-2453522	DEL	PRECISE	364	0	0	0/1	1/1	0/1	1/1	1/1	1/1	1/1	1/1	1/1	1/1	1/1	1/1	1/1	1/1
Chromosome1-2453619	INS	PRECISE	986	0	0	0/0	0/0	0/0	0/0	0/0	0/0	0/0	0/0	0/1	0/0	0/0	0/0	0/0	0/0
Chromosome1-2454277	DEL	PRECISE	1454	0	0	0/0	0/0	0/0	0/0	0/0	0/0	0/0	0/0	0/1	0/1	0/0	0/1	0/0	0/0
Chromosome1-2454434	INS	PRECISE	67	0	0	0/0	0/0	0/0	0/0	0/0	0/0	0/0	0/0	0/0	0/0	0/1	0/0	1/1	0/1
Chromosome1-2454570	DEL	PRECISE	89	0	0	0/0	0/0	0/0	0/0	0/0	0/0	0/0	0/0	0/1	0/1	0/1	0/1	0/1	0/1
Chromosome1-2455441	INS	PRECISE	137	0	0	0/0	0/0	0/0	0/0	0/0	0/0	0/0	0/0	0/0	0/0	0/0	0/0	0/0	0/1
Chromosome1-2456048	DEL	PRECISE	199	0	0	0/0	0/0	0/0	0/0	0/0	0/0	0/0	0/0	0/0	0/1	0/0	0/0	0/0	0/0
Chromosome1-2456191	INS	PRECISE	68	0	0	0/0	0/0	0/0	0/0	0/0	0/0	0/1	0/1	0/0	0/0	0/0	0/0	0/0	0/0
Chromosome1-2456648	INS	PRECISE	70	0	0	0/0	0/0	0/0	0/0	0/0	0/0	0/0	0/0	0/0	0/0	0/1	1/1	0/1	0/1
Chromosome1-2457721	DEL	PRECISE	743	0	0	0/0	0/0	0/0	0/0	0/0	0/0	0/0	0/0	1/1	1/1	1/1	1/1	1/1	0/1
Chromosome1-2457827	INS	PRECISE	64	0	0	0/0	0/0	0/0	0/0	0/0	0/0	0/0	0/0	0/0	0/0	0/0	0/0	0/0	0/1
Chromosome1-2457870	BND	PRECISE	0	0	0	0/1	0/1	0/0	0/0	0/1	0/0	0/0	0/1	0/0	0/0	0/0	0/0	0/0	0/0
Chromosome1-2457870	BND	PRECISE	0	0	0	0/0	0/0	0/0	0/0	0/0	0/0	0/0	0/0	0/0	0/1	0/0	0/0	0/1	0/0
Chromosome1-2458220	DEL	PRECISE	88	0	0	0/0	0/1	0/1	0/0	0/0	0/0	0/1	0/0	0/0	0/0	0/0	0/0	0/0	0/0
Chromosome1-2458568	INS	PRECISE	364	0	0	0/0	0/0	0/0	0/0	0/0	0/0	0/0	0/0	0/1	0/0	0/0	0/0	0/0	0/0
Chromosome1-2458758	INS	PRECISE	137	0	0.577	0/0	0/0	0/0	0/0	0/0	0/0	0/0	0/0	0/1	0/1	0/0	0/1	0/0	0/0
Chromosome1-2458978	DEL	PRECISE	157	0	0	0/0	0/0	0/0	0/0	0/0	0/0	0/0	0/0	0/1	0/1	0/1	0/1	0/1	0/1
Chromosome1-2460247	INS	PRECISE	847	0	0	0/0	0/0	0/0	0/0	0/0	0/0	0/0	0/0	0/0	0/0	0/0	0/1	0/0	0/0
Chromosome1-2461007	INS	PRECISE	5197	0	0	0/0	0/0	0/0	0/0	0/0	0/0	0/0	0/0	0/0	0/0	0/0	0/0	0/0	1/1
Chromosome1-2461402	DEL	PRECISE	806	0	0	0/0	0/0	0/0	0/0	0/1	0/1	0/0	0/0	0/0	0/0	0/0	0/0	0/0	0/0
Chromosome1-2462373	INS	PRECISE	10864	0	0	0/0	0/0	0/1	0/0	0/0	0/0	0/0	0/0	0/0	0/0	0/0	0/0	0/0	0/0
Chromosome1-2462374	BND	PRECISE	0	0	0	0/0	0/1	0/1	0/0	0/0	0/0	0/1	0/0	0/0	0/0	0/0	0/0	0/0	0/0
Chromosome1-2462479	DEL	PRECISE	54	0	0	0/0	0/0	0/0	0/0	0/0	0/0	0/0	0/0	0/0	0/0	0/0	0/0	0/0	0/1
Chromosome1-2463801	DEL	PRECISE	753	0	0	0/0	0/0	0/0	0/0	0/0	0/0	0/0	0/0	0/1	0/0	0/1	0/1	0/1	0/1
Chromosome1-2463810	INS	PRECISE	573	0	0	0/0	0/0	0/0	0/0	0/1	0/1	0/0	0/0	0/0	0/0	0/0	0/0	0/0	0/0
Chromosome1-2463919	INS	PRECISE	1536	0	0	0/0	0/0	0/0	0/0	0/0	0/0	0/1	0/1	0/0	0/0	0/0	0/0	0/0	0/0
Chromosome1-2464989	DEL	PRECISE	5250	0	0	0/0	0/0	0/0	0/0	0/0	0/0	0/0	0/0	0/1	0/0	0/0	0/0	0/0	0/0
Chromosome1-2465717	DEL	PRECISE	67	0	0	0/0	0/0	0/0	0/1	1/1	1/1	0/1	0/1	1/1	0/0	0/0	0/0	0/0	0/0
Chromosome1-2466445	INS	PRECISE	901	0	0	0/0	0/0	0/0	0/0	0/0	0/0	0/0	0/0	0/0	0/0	0/0	0/0	0/0	0/1
Chromosome1-2466470	INS	PRECISE	87	0	0	0/0	0/0	0/0	0/0	0/0	0/0	0/0	0/0	0/0	0/0	0/0	0/0	0/0	0/1
Chromosome1-2466511	DEL	PRECISE	724	0	0	0/0	0/0	0/0	0/0	0/0	0/0	0/0	0/0	0/1	0/1	0/1	0/1	0/1	0/1
Chromosome1-2467399	DEL	PRECISE	50	0	0	0/0	0/0	0/0	0/0	0/0	0/0	0/0	0/0	0/1	0/1	0/1	0/1	0/1	0/1
Chromosome1-2467527	INS	PRECISE	161	0	0	0/0	0/0	0/0	0/0	0/0	0/0	0/0	0/0	0/1	0/1	0/1	0/1	1/1	0/1
Chromosome1-2469820	DEL	PRECISE	142	0	0	0/0	0/0	0/0	0/0	0/0	0/0	0/0	0/0	0/0	0/1	0/0	0/0	0/0	0/0
Chromosome1-2470765	INS	PRECISE	780	0	0	0/0	0/0	0/0	0/0	0/0	0/0	0/0	0/0	0/1	0/0	0/0	0/0	0/0	0/0

Chromosome1-2470976	DEL	PRECISE	62	0	0	0/0	0/0	0/0	0/0	0/0	0/0	0/0	0/0	0/0	0/0	0/0	0/0	0/0	0/1
Chromosome1-2471005	BND	PRECISE	0	0	0	0/0	0/0	0/0	0/0	0/0	0/0	0/0	0/0	0/0	0/0	0/0	0/1	0/0	0/0
Chromosome1-2471013	INS	PRECISE	3917	0	0	0/0	0/0	0/0	0/0	0/0	0/0	0/0	0/0	0/0	0/0	0/0	0/0	0/0	1/1
Chromosome1-2471044	DEL	PRECISE	633	0	0	0/0	0/0	0/0	0/0	0/0	0/0	0/0	0/0	0/1	1/1	1/1	1/1	1/1	0/1
Chromosome1-2471219	DEL	PRECISE	245	0.577	0.577	0/0	0/0	0/1	0/0	0/0	0/0	0/1	0/0	0/0	0/0	0/0	0/0	0/0	0/0
Chromosome1-2471474	INS	PRECISE	685	0	0.641	0/1	0/1	0/0	0/1	0/0	0/0	0/0	0/1	0/0	0/0	0/1	0/1	0/1	0/0
Chromosome1-2471809	INS	PRECISE	5063	0	0	0/0	0/0	0/0	0/0	0/0	0/0	0/0	0/0	0/0	1/1	0/0	0/0	0/0	0/0
Chromosome1-2471809	BND	PRECISE	0	0	0	0/0	0/0	0/0	0/0	0/0	0/0	0/0	0/0	0/0	0/1	0/0	0/0	0/0	0/0
Chromosome1-2471862	INS	PRECISE	245	0	0	0/0	0/0	0/0	0/0	0/0	0/0	0/0	0/0	0/0	0/1	0/0	0/0	0/0	0/0
Chromosome1-2472438	DEL	PRECISE	1391	0	0	0/1	1/1	0/1	1/1	1/1	1/1	1/1	1/1	1/1	1/1	1/1	1/1	1/1	1/1
Chromosome1-2472664	INS	PRECISE	60	0	0	0/1	1/1	0/1	1/1	1/1	1/1	1/1	1/1	1/1	1/1	1/1	1/1	1/1	1/1
Chromosome1-2472865	DEL	PRECISE	882	0	0	0/0	0/0	0/0	0/0	0/0	0/0	0/0	0/0	0/1	0/1	0/0	0/1	0/1	0/1
Chromosome1-2473546	BND	PRECISE	0	0	0	0/0	0/0	0/0	0/0	0/0	0/0	0/0	0/0	0/0	0/0	0/0	0/0	0/1	0/0
Chromosome1-2474759	DEL	PRECISE	307	0	0	0/1	1/1	0/1	1/1	1/1	1/1	1/1	1/1	1/1	0/1	0/1	1/1	0/1	0/0
Chromosome1-2474903	DEL	PRECISE	63	0	0	0/0	0/0	0/0	0/0	0/0	0/0	0/0	0/0	0/0	0/1	0/1	0/0	0/1	0/1
Chromosome1-2474930	DEL	PRECISE	1442	0	0	0/0	0/0	0/0	0/0	0/0	0/0	0/0	0/0	0/1	0/0	0/0	0/0	0/0	0/0
Chromosome1-2475105	INS	PRECISE	211	0	0	0/0	0/0	0/0	0/0	0/0	0/0	0/0	0/0	0/0	0/1	0/0	0/0	0/0	0/0
Chromosome1-2475375	DEL	PRECISE	237	0	0	0/1	1/1	0/1	1/1	1/1	1/1	1/1	1/1	1/1	1/1	1/1	1/1	1/1	1/1
Chromosome1-2475504	BND	PRECISE	0	0	0	0/0	0/0	0/0	0/0	0/0	0/0	0/1	0/1	0/0	0/0	0/0	0/0	0/0	0/0
Chromosome1-2475705	INS	PRECISE	11296	0	0	0/0	0/0	0/0	0/0	0/0	0/0	0/0	0/0	0/0	0/0	0/0	0/0	0/0	0/1
Chromosome1-2475792	BND	PRECISE	0	0	0	0/0	0/0	0/0	0/0	0/1	0/0	0/0	0/0	0/0	0/0	0/0	0/0	0/0	0/0
Chromosome1-2475991	INS	PRECISE	14172	0	0	0/0	0/0	0/0	0/0	0/0	0/0	0/0	0/0	0/1	0/0	0/0	0/0	0/0	0/0
Chromosome1-2476302	INS	PRECISE	434	0	0.577	0/0	1/1	0/1	0/0	0/0	0/0	1/1	0/0	0/0	0/0	0/0	0/0	0/0	0/0
Chromosome1-2476656	DEL	PRECISE	1448	0	0	0/0	0/0	0/0	0/0	0/0	0/0	0/0	0/0	0/1	0/1	0/1	0/1	0/1	1/1
Chromosome1-2477159	INS	PRECISE	4122	0	0	0/0	0/0	0/0	0/0	0/0	0/0	0/0	0/0	0/0	0/1	0/0	0/0	0/0	0/0
Chromosome1-2477173	INS	PRECISE	1300	0	0.707	0/0	0/0	0/0	0/0	0/1	0/1	0/0	0/0	0/0	0/0	0/0	0/0	0/0	0/0
Chromosome1-2477587	INS	PRECISE	9689	0	0	0/0	0/0	0/0	0/0	0/0	0/0	0/0	0/0	0/0	0/0	0/0	0/0	0/0	1/1
Chromosome1-2477938	DEL	PRECISE	5992	0.267	0.267	0/1	1/1	0/1	1/1	1/1	1/1	1/1	1/1	1/1	1/1	1/1	1/1	1/1	1/1
Chromosome1-2478618	BND	PRECISE	0	0	0	0/0	0/0	0/0	0/0	0/0	0/0	0/0	0/0	0/1	0/0	0/0	0/0	0/0	0/0
Chromosome1-2478749	INS	PRECISE	4776	0	0	0/0	0/0	0/0	0/0	0/0	0/0	0/0	0/0	0/0	0/0	0/0	0/1	0/0	0/0
Chromosome1-2479219	INS	PRECISE	458	0	0	0/0	0/0	0/0	0/0	0/0	0/0	0/0	0/0	0/0	0/0	0/0	0/0	0/0	1/1
Chromosome1-2479586	DEL	PRECISE	51	0	0	0/0	0/0	0/0	0/0	0/0	0/0	0/0	0/0	1/1	0/1	0/1	0/1	0/1	0/1
Chromosome1-2479921	INS	PRECISE	623	0	0	0/0	0/1	0/1	0/0	0/0	0/0	0/1	0/0	0/0	0/0	0/0	0/0	0/0	0/0
Chromosome1-2479982	DEL	PRECISE	53	0	0	0/1	0/1	0/0	0/1	0/0	0/0	0/1	1/1	0/0	0/1	0/1	0/1	0/1	0/0
Chromosome1-2480088	INS	PRECISE	211	0	0	0/0	0/0	0/0	0/1	1/1	1/1	0/0	0/0	0/1	0/0	0/0	0/0	0/0	0/0
Chromosome1-2481196	INS	PRECISE	5572	0	0	0/0	0/0	0/0	0/0	0/0	0/0	0/0	0/0	0/0	1/1	0/0	0/0	0/0	0/0
Chromosome1-2481233	DEL	PRECISE	163	0	0	0/0	0/0	0/0	0/0	0/0	0/0	0/0	0/0	0/0	0/1	0/0	0/0	0/0	0/0
Chromosome1-2481867	INS	PRECISE	454	0	0.452	0/1	0/1	0/0	1/1	1/1	0/1	0/0	1/1	0/1	0/1	1/1	0/1	0/1	0/0
Chromosome1-2482780	DEL	PRECISE	986	0	0	0/0	0/0	0/0	0/0	0/0	0/0	0/0	0/0	0/1	0/1	0/0	0/1	0/1	0/1
Chromosome1-2482806	DEL	PRECISE	68	0	0	0/0	0/0	0/0	0/0	0/0	0/0	0/1	0/1	0/0	0/0	0/0	0/0	0/0	0/0
Chromosome1-2482921	DEL	PRECISE	641	0	0	0/0	0/0	0/0	0/0	0/0	0/0	0/0	0/0	0/1	0/1	0/1	0/1	0/1	0/1
Chromosome1-2483534	DEL	PRECISE	58	0	0	0/0	0/0	0/0	0/0	0/0	0/0	0/0	0/0	0/0	0/0	0/0	0/0	0/0	0/1
Chromosome1-2483711	BND	PRECISE	0	0	0	0/0	0/0	0/0	0/0	0/0	0/0	0/1	0/0	0/0	0/0	0/0	0/0	0/0	0/0
Chromosome1-2483758	INS	PRECISE	827	0	0	0/0	0/0	0/0	0/0	0/0	0/0	0/0	0/0	0/0	0/1	0/0	0/0	0/0	0/0
Chromosome1-2484163	DEL	PRECISE	64	0	0	0/0	0/0	0/0	0/0	0/0	0/0	0/1	0/0	0/0	0/0	0/0	0/0	0/0	0/0
Chromosome1-2484484	INS	PRECISE	7553	0	0	0/0	0/0	0/0	0/0	0/0	0/0	0/0	0/0	0/1	0/0	0/0	0/0	0/0	0/0
Chromosome1-2484484	BND	PRECISE	0	0	0	0/0	0/0	0/0	0/0	0/0	0/0	0/0	0/0	0/1	0/0	0/0	0/0	0/0	0/0
Chromosome1-2484540	INS	PRECISE	5443	0	0	0/0	0/0	0/0	0/0	0/0	0/0	0/0	0/0	0/0	0/1	0/0	0/0	0/0	0/0
Chromosome1-2484593	INS	PRECISE	489	0	0.518	0/0	0/1	0/1	0/0	1/1	0/1	0/1	0/1	0/1	0/0	0/0	0/0	0/0	0/1
Chromosome1-2485722	INS	PRECISE	404	0	0.548	0/0	0/0	0/0	0/0	0/0	0/0	0/0	0/0	1/1	1/1	1/1	1/1	0/0	1/1
Chromosome1-2486553	INS	PRECISE	51	0	0	0/0	0/0	0/0	0/0	0/0	0/0	0/1	0/0	0/0	0/0	0/0	0/0	0/0	0/0
Chromosome1-2487152	DEL	PRECISE	57	0	0	0/1	1/1	0/1	1/1	1/1	1/1	0/1	1/1	0/1	0/1	0/1	0/1	0/1	0/1
Chromosome1-2488514	INS	PRECISE	122	0	0	0/0	0/0	0/0	0/0	0/0	0/0	0/0	0/0	0/1	0/0	0/0	0/0	0/0	0/0
Chromosome1-2488578	DEL	PRECISE	101	0	0	0/0	0/0	0/0	0/0	0/0	0/0	0/0	0/0	0/0	0/0	0/0	0/1	0/0	0/0
Chromosome1-2489000	DEL	PRECISE	1650	0	0	0/0	0/0	0/0	0/0	0/0	0/0	0/0	0/0	0/0	0/0	0/0	0/0	0/1	0/0
Chromosome1-2489143	DEL	PRECISE	1341	0	0	0/0	0/0	0/0	0/0	0/0	0/0	0/1	0/0	0/0	0/0	0/0	0/0	0/0	0/0
Chromosome1-2489319	INS	PRECISE	58	0	0	0/1	1/1	0/1	1/1	1/1	1/1	1/1	1/1	1/1	1/1	1/1	1/1	1/1	1/1
Chromosome1-2489900	INS	PRECISE	72	0	0	0/0	0/0	0/0	0/0	0/0	0/0	0/1	0/0	0/0	0/0	0/0	0/0	0/0	0/0
Chromosome1-2490450	INS	PRECISE	229	0	0	0/0	0/0	0/0	0/0	0/0	0/0	0/0	0/0	0/0	0/1	0/0	0/0	0/0	0/0
Chromosome1-2490653	DEL	PRECISE	137	0	0	0/1	1/1	0/1	1/1	1/1	1/1	1/1	1/1	1/1	1/1	1/1	1/1	1/1	1/1
Chromosome1-2490889	INS	PRECISE	122	0	0.5	0/0	0/0	0/0	0/0	0/0	0/0	0/0	0/0	1/1	0/0	0/1	0/0	0/1	0/1
Chromosome1-2490970	DEL	PRECISE	235	0	0	0/0	0/0	0/0	0/0	0/0	0/0	0/0	0/0	1/1	0/1	0/1	0/1	0/1	0/1
Chromosome1-2491333	BND	PRECISE	0	0	0	0/0	0/0	0/0	0/0	0/0	0/1	0/0	0/0	0/0	0/0	0/0	0/0	0/0	0/0
Chromosome1-2491353	INS	PRECISE	5333	0	0	0/0	0/0	0/0	0/0	0/0	0/0	0/0	0/0	0/0	0/0	0/0	0/0	0/0	1/1
Chromosome1-2491458	DEL	PRECISE	751	0.408	0.408	0/0	0/0	0/0	0/0	0/0	0/0	0/0	0/0	1/1	0/1	0/1	0/1	0/1	0/1
Chromosome1-2491686	INS	PRECISE	53	0	0	0/0	0/0	0/0	0/0	0/0	0/0	0/0	0/0	0/1	0/1	0/1	0/1	0/1	0/1
Chromosome1-2491750	DEL	PRECISE	458	0	0	0/0	0/0	0/0	0/0	0/0	0/0	0/0	0/0	0/1	0/1	0/1	0/1	0/1	0/1
Chromosome1-2491864	DEL	PRECISE	113	0	0	0/0	0/0	0/0	0/0	0/0	0/0	0/0	0/0	0/1	0/1	0/1	0/1	0/1	0/1
Chromosome1-2492623	DEL	PRECISE	380	0	0	0/0	0/0	0/0	0/0	0/0	0/0	0/0	0/0	0/1	0/1	0/1	0/1	0/1	0/1
Chromosome1-2493315	DEL	PRECISE	194	0	0	0/0	0/0	0/0	0/0	0/0	0/0	0/0	0/0	0/0	0/0	0/1	0/0	0/1	0/1
Chromosome1-2493504	INS	PRECISE	269	0.548	0.816	0/0	0/0	0/0	0/0	0/0	0/0	0/0	0/0	0/1	0/1	0/1	0/1	0/1	0/1
Chromosome1-2493629	INS	PRECISE	177	0	0.816	0/0	0/0	0/0	0/0	0/0	0/0	0/0	0/0	0/1	0/0	0/1	0/1	0/0	0/1
Chromosome1-2494073	DEL	PRECISE	190	0	0	0/0	0/0	0/0	0/0	0/0	0/0	0/0	0/0	0/1	0/0	0/1	0/1	0/0	0/1

Chromosome1-2494557	INS	PRECISE	84	0	0.577	0/0	0/0	0/0	0/0	0/0	0/0	0/0	0/0	0/0	0/0	0/1	0/0	0/1	0/1
Chromosome1-2496011	DEL	PRECISE	129	0	0	0/0	0/0	0/0	0/0	0/0	0/0	0/1	0/1	0/1	0/0	0/0	0/1	0/0	0/0
Chromosome1-2496690	DEL	PRECISE	2955	0	0	0/0	0/0	0/0	0/0	0/0	0/0	0/1	0/0	0/0	0/0	0/0	0/0	0/0	0/0
Chromosome1-2496845	INS	PRECISE	72	0	0	0/1	1/1	0/1	1/1	1/1	1/1	0/1	1/1	1/1	1/1	1/1	1/1	1/1	0/1
Chromosome1-2497460	DEL	PRECISE	73	0	0	0/0	0/0	0/0	0/0	0/0	0/0	0/0	0/0	0/0	0/0	0/1	0/0	0/1	0/1
Chromosome1-2497910	INS	PRECISE	59	0	0	0/0	0/0	0/0	0/0	0/0	0/0	0/1	0/0	0/1	0/1	0/0	0/1	0/0	0/0
Chromosome1-2498061	INS	PRECISE	645	0	0	0/0	0/0	0/0	0/0	0/0	0/0	0/0	0/0	0/0	0/0	0/0	0/0	0/0	0/1
Chromosome1-2498143	INS	PRECISE	836	0	0.577	0/0	0/0	0/0	0/0	0/0	0/0	0/0	0/0	0/0	0/0	0/1	0/0	0/1	0/1
Chromosome1-2498412	INS	PRECISE	444	0	0	0/0	0/0	0/0	0/0	0/0	0/0	0/0	0/0	0/0	0/0	0/0	0/0	0/0	0/1
Chromosome1-2502041	DEL	PRECISE	767	0	0	0/0	0/0	0/0	0/0	0/0	0/0	0/0	0/0	0/1	0/1	1/1	0/1	0/1	0/1
Chromosome1-2502077	INS	PRECISE	54	0	0	0/0	0/0	0/0	0/0	0/0	0/0	0/1	0/1	0/0	0/0	0/0	0/0	0/0	0/0
Chromosome1-2502782	INS	PRECISE	4999	0	0	0/0	0/0	0/0	0/0	0/0	0/0	0/0	0/0	0/0	1/1	0/0	0/0	0/0	0/0
Chromosome1-2503157	DEL	PRECISE	129	0	0	0/1	1/1	0/1	1/1	1/1	1/1	1/1	1/1	1/1	1/1	1/1	1/1	1/1	1/1
Chromosome1-2503222	INS	PRECISE	680	0	0	0/0	0/0	0/0	0/1	0/0	0/0	0/0	0/0	0/0	0/0	0/0	0/0	0/0	0/0
Chromosome1-2504149	DEL	PRECISE	393	0	0	0/0	0/0	0/0	0/0	0/0	0/0	0/0	0/0	0/0	0/1	0/0	0/0	0/0	0/0
Chromosome1-2504282	INS	PRECISE	201	0	0	0/0	0/0	0/0	0/0	0/0	0/0	0/0	0/0	0/0	0/1	0/0	0/0	0/0	0/0
Chromosome1-2504462	INS	PRECISE	6010	0	0.707	0/0	0/0	0/0	0/0	0/0	0/0	1/1	1/1	0/0	0/0	0/0	0/0	0/0	0/0
Chromosome1-2504579	DEL	PRECISE	189	0.447	0.447	0/0	0/0	0/0	0/0	0/0	0/0	0/0	0/0	0/1	0/1	0/0	0/0	0/0	0/1
Chromosome1-2505261	DEL	PRECISE	523	0	0	0/0	0/0	0/0	0/1	0/1	0/1	0/0	0/1	0/0	0/0	0/0	0/0	0/0	0/0
Chromosome1-2506182	DEL	PRECISE	1195	0	0	0/0	0/0	0/0	0/0	0/0	0/0	0/0	0/0	0/0	0/0	0/0	0/1	0/0	0/0
Chromosome1-2506325	DEL	PRECISE	162	0	0	0/0	0/0	0/0	0/0	0/0	0/0	0/0	0/0	0/0	0/1	0/0	0/1	0/0	0/0
Chromosome1-2506664	INS	PRECISE	575	0	0	0/0	0/0	0/0	0/0	0/0	0/0	0/0	0/0	0/0	1/1	0/0	1/1	0/0	0/0
Chromosome1-2506760	INS	PRECISE	164	0	0.447	0/0	0/0	0/0	0/1	1/1	0/1	0/1	0/1	0/0	0/0	0/0	0/0	0/0	0/0
Chromosome1-2506785	DEL	PRECISE	521	0	0	0/1	1/1	0/1	1/1	1/1	1/1	1/1	1/1	1/1	1/1	1/1	1/1	1/1	1/1
Chromosome1-2507800	INS	PRECISE	363	0	0.756	0/1	0/1	0/0	0/1	0/0	0/0	0/1	1/1	0/0	0/1	0/1	0/1	0/0	0/0
Chromosome1-2508001	INS	PRECISE	627	0	0	0/0	0/0	0/0	0/0	0/0	0/0	0/1	0/0	0/0	0/0	0/0	0/0	0/0	0/0
Chromosome1-2509268	INS	PRECISE	158	0	0.641	0/1	0/1	0/0	1/1	0/1	0/1	0/1	1/1	0/1	1/1	1/1	1/1	0/1	1/1
Chromosome1-2510214	DEL	PRECISE	150	0	0	0/1	1/1	0/1	1/1	1/1	1/1	1/1	1/1	1/1	1/1	1/1	1/1	1/1	1/1
Chromosome1-2510925	INS	PRECISE	4653	0	0	0/0	0/0	0/0	0/0	0/0	0/0	1/1	0/0	0/0	0/0	0/0	0/0	0/0	0/0
Chromosome1-2511047	DEL	PRECISE	627	0	0	0/0	0/0	0/0	0/0	0/0	0/0	0/0	0/0	0/0	0/0	0/1	0/1	0/1	0/1
Chromosome1-2512276	DEL	PRECISE	5300	0	0	0/1	1/1	0/1	1/1	1/1	1/1	1/1	1/1	1/1	1/1	1/1	1/1	1/1	1/1
Chromosome1-2513302	INS	PRECISE	54	0	0	0/0	0/0	0/0	0/1	0/0	0/0	0/0	0/0	0/0	0/0	0/0	0/0	0/0	0/0
Chromosome1-2513918	INS	PRECISE	150	0	0	0/0	0/0	0/0	0/0	0/0	0/0	0/1	0/0	0/0	0/0	0/0	0/0	0/0	0/0
Chromosome1-2515161	INS	PRECISE	70	0	0	0/0	0/0	0/0	0/0	0/0	0/0	0/1	0/0	0/0	0/0	0/0	0/0	0/0	0/0
Chromosome1-2515237	DEL	PRECISE	5198	0	0	0/0	0/0	0/0	0/0	0/0	0/0	0/1	0/0	0/0	0/0	0/0	0/0	0/0	0/0
Chromosome1-2516755	INS	PRECISE	89	0	0.267	0/1	1/1	0/1	1/1	1/1	1/1	0/1	1/1	1/1	1/1	1/1	1/1	1/1	1/1
Chromosome1-2518325	INS	PRECISE	313	0	0	0/0	0/0	0/0	0/0	0/0	0/0	0/0	0/0	0/0	0/1	0/0	0/0	0/0	0/0
Chromosome1-2518596	INS	PRECISE	152	0	0	0/0	0/0	0/0	0/1	0/1	0/1	0/0	0/0	0/0	0/0	0/0	0/0	0/0	0/0
Chromosome1-2518666	INS	PRECISE	653	0	0	0/0	0/0	0/0	0/0	0/0	0/0	0/1	0/0	0/0	0/0	0/0	0/0	0/0	0/0
Chromosome1-2519121	INS	PRECISE	6154	0	0	0/0	0/0	0/0	0/0	0/0	0/0	0/0	0/0	0/0	0/0	0/0	0/0	0/0	1/1
Chromosome1-2519260	INS	PRECISE	3110	0	0	0/0	0/0	0/0	0/0	0/0	0/0	0/0	0/0	0/0	0/0	0/1	0/0	0/0	0/0
Chromosome1-2519309	INS	PRECISE	8858	0	0	0/0	0/0	0/0	0/0	0/0	0/0	0/0	0/0	0/0	0/0	0/1	0/0	0/0	0/0
Chromosome1-2519430	INS	PRECISE	711	0	0	0/0	0/0	0/0	0/0	0/0	0/0	0/0	0/0	0/1	0/0	0/0	0/0	0/0	0/0
Chromosome1-2519453	INS	PRECISE	2097	0	0	0/0	0/0	0/0	0/0	0/0	0/0	0/0	0/0	0/1	0/0	0/0	0/0	0/0	0/0
Chromosome1-2520538	DEL	PRECISE	70	0.816	0.816	0/0	0/0	0/0	0/0	0/0	0/0	0/0	0/0	0/1	0/0	0/1	0/1	0/0	0/1
Chromosome1-2520915	DEL	PRECISE	705	0.408	0	0/0	0/0	0/0	0/0	0/0	0/0	0/0	0/0	0/1	0/1	0/1	0/1	0/1	0/1
Chromosome1-2521911	DEL	PRECISE	379	0	0	0/1	1/1	0/1	1/1	1/1	1/1	0/1	1/1	1/1	0/1	1/1	0/1	0/1	0/0
Chromosome1-2522377	INS	PRECISE	343	0	0	0/0	0/0	0/0	0/0	0/0	0/0	0/0	0/0	0/0	0/0	0/0	0/0	0/0	1/1
Chromosome1-2522943	BND	PRECISE	0	0	0	0/0	0/0	0/0	0/0	0/0	0/0	0/0	0/0	0/0	0/0	0/0	0/0	0/1	0/0
Chromosome1-2522944	INS	PRECISE	4186	0	0	0/0	0/0	0/0	0/0	0/0	0/0	0/0	0/0	1/1	0/0	0/0	0/0	0/0	0/0
Chromosome1-2526851	DEL	PRECISE	84	0	0	0/0	0/0	0/0	0/1	0/0	0/0	1/1	0/1	0/1	0/0	0/0	0/1	0/0	0/0
Chromosome1-2527356	INS	PRECISE	92	0	0	0/0	0/0	0/0	0/0	0/0	0/0	0/0	0/0	0/0	0/1	0/0	0/0	0/0	0/0
Chromosome1-2527554	DEL	PRECISE	297	0	0	0/0	0/0	0/0	0/0	0/0	0/0	0/0	0/0	0/1	0/1	0/0	0/1	0/1	0/0
Chromosome1-2528024	DEL	PRECISE	126	0	0	0/0	0/0	0/0	0/0	0/0	0/0	0/1	0/0	0/0	0/0	0/0	0/0	0/0	0/0
Chromosome1-2528139	INS	PRECISE	625	0	0.823	0/1	0/1	0/0	0/1	0/1	0/1	0/1	1/1	0/0	0/1	1/1	0/1	0/0	0/0
Chromosome1-2529223	INS	PRECISE	86	0	0	0/0	0/0	0/0	0/0	0/0	0/0	0/0	0/0	0/1	0/1	0/1	0/1	0/1	0/1
Chromosome1-2529233	DEL	PRECISE	5239	0.277	0.277	0/0	0/1	0/1	0/1	0/1	0/1	1/1	0/1	1/1	0/1	0/1	0/1	1/1	1/1
Chromosome1-2529757	DEL	PRECISE	2910822	0	0	0/1	0/1	0/0	0/0	0/0	0/0	0/0	0/0	0/0	0/0	0/0	0/0	0/0	0/0
Chromosome1-2529888	DEL	PRECISE	55	0	0	0/1	0/1	0/0	0/1	0/1	0/1	0/0	0/1	0/0	0/1	0/1	0/1	0/0	0/0
Chromosome1-2530370	BND	PRECISE	0	0	0	0/0	0/0	0/0	0/0	0/0	0/0	0/0	0/0	0/0	0/0	0/0	0/0	0/1	1/1
Chromosome1-2530457	DEL	PRECISE	1792	0	0	0/0	0/0	0/0	0/0	0/0	0/0	0/0	0/0	0/0	0/1	0/0	0/0	0/0	0/0
Chromosome1-2530597	INS	PRECISE	19280	0	0	0/0	0/0	0/0	0/0	0/0	0/0	0/0	0/0	0/1	0/0	0/0	0/0	0/0	0/0
Chromosome1-2530806	DEL	PRECISE	62	0	0	0/0	0/0	0/0	0/0	0/0	0/0	0/1	0/1	0/0	0/0	0/0	0/0	0/0	0/1
Chromosome1-2531304	INS	PRECISE	7894	0	0	0/0	0/0	0/0	0/0	0/0	0/0	0/0	0/0	0/0	0/0	0/0	0/0	0/0	1/1
Chromosome1-2531536	INS	PRECISE	908	0.707	0.707	0/0	0/0	0/0	0/0	0/0	0/0	0/0	0/0	0/1	1/1	0/0	0/0	0/0	0/0
Chromosome1-2531598	INS	PRECISE	104	0	0	0/0	1/1	0/1	1/1	1/1	1/1	0/0	0/1	1/1	1/1	1/1	1/1	1/1	0/0
Chromosome1-2532194	DEL	PRECISE	839	0	0	0/0	0/0	0/0	0/0	0/0	0/0	0/0	0/0	0/0	1/1	0/0	0/0	0/0	0/0
Chromosome1-2532347	DEL	PRECISE	263	0	0	0/0	0/0	0/0	0/0	0/0	0/0	0/0	0/0	0/0	1/1	0/0	0/0	0/0	0/0
Chromosome1-2532546	INS	PRECISE	4121	0	0	0/0	0/0	0/0	0/0	0/0	0/0	0/0	0/0	0/0	0/0	0/0	0/0	0/0	1/1
Chromosome1-2532721	DEL	PRECISE	81	0.577	0.577	0/0	0/0	0/0	0/0	0/0	0/0	0/1	0/0	0/0	0/0	0/0	0/0	0/0	0/0
Chromosome1-2534374	INS	PRECISE	312	0	0	0/0	0/0	0/0	0/0	0/0	0/0	0/0	0/0	0/1	0/0	0/0	0/0	0/0	0/0
Chromosome1-2534447	DEL	PRECISE	155	0	0	0/0	0/0	0/0	0/0	0/0	0/0	0/0	0/0	0/1	0/0	0/0	0/0	0/0	0/0
Chromosome1-2536502	DEL	PRECISE	7595	0	0	0/0	0/0	0/0	0/0	0/0	0/0	1/1	0/0	0/0	0/0	0/0	0/0	0/0	0/0

Chromosome1-2537262	BND	PRECISE	0	0	0	0/0	0/0	0/1	1/1	0/0	1/1	0/0	0/0	0/0	0/0	0/0	0/0	0/0	0/0
Chromosome1-2537558	DUP	PRECISE	79284	0	0	0/0	0/0	0/0	0/0	0/0	0/0	0/0	0/0	0/0	0/1	0/0	0/0	0/0	0/0
Chromosome1-2537728	DEL	PRECISE	1536	0	0	0/0	1/1	0/0	1/1	1/1	1/1	0/0	1/1	1/1	0/1	0/0	0/0	0/0	0/0
Chromosome1-2537917	INS	PRECISE	683	0	0	0/0	0/0	0/0	0/0	0/0	0/0	0/0	0/0	0/0	0/1	0/0	0/0	0/0	0/0
Chromosome1-2540868	INS	PRECISE	1556	0	0.816	0/0	0/1	0/0	0/1	0/0	0/1	0/0	0/0	1/1	0/0	0/0	0/0	1/1	0/0
Chromosome1-2541026	DEL	PRECISE	2620	0	0	0/0	0/0	0/0	0/0	0/0	0/0	0/1	0/0	0/0	0/0	0/0	0/0	0/0	0/0
Chromosome1-2542070	INS	PRECISE	236	0	0	0/0	0/0	0/0	0/0	0/0	0/0	0/0	0/0	0/0	0/0	0/0	0/0	0/0	0/1
Chromosome1-2542598	DEL	PRECISE	1837	0	0	0/1	1/1	0/0	1/1	1/1	1/1	1/1	1/1	1/1	1/1	1/1	1/1	1/1	1/1
Chromosome1-2542787	DEL	PRECISE	4860	0	0	0/1	1/1	0/0	1/1	1/1	1/1	1/1	1/1	1/1	1/1	1/1	1/1	1/1	1/1
Chromosome1-2543300	DEL	PRECISE	1241	0	0	0/0	0/0	0/0	0/0	0/0	0/0	1/1	0/0	0/0	0/0	0/0	0/0	0/0	0/0
Chromosome1-2543410	DEL	PRECISE	59	0.577	0	0/0	0/0	0/0	0/0	0/0	0/0	0/0	0/0	0/0	0/0	0/1	0/0	0/1	0/1
Chromosome1-2543828	DEL	PRECISE	178	0	0	0/0	0/0	0/0	0/0	0/0	0/0	0/0	0/0	0/0	0/0	0/0	0/0	0/0	0/1
Chromosome1-2544151	DUP	PRECISE	23726	0	0	0/0	0/0	0/0	0/0	0/0	0/0	0/0	0/0	0/0	0/0	0/0	0/0	0/0	0/0
Chromosome1-2544466	DEL	PRECISE	1468	0	0	0/0	0/0	0/0	0/0	0/0	0/0	0/0	0/0	0/0	1/1	0/0	0/0	0/0	0/0
Chromosome1-2544838	DEL	PRECISE	2514	0	0	0/0	0/0	0/0	0/0	0/0	0/0	0/0	0/0	0/0	1/1	0/0	0/0	0/0	0/0
Chromosome1-2544913	BND	PRECISE	0	0	0	0/0	0/0	0/0	0/0	0/0	0/0	0/0	0/0	0/0	0/0	0/1	0/0	0/1	1/1
Chromosome1-2545191	DEL	PRECISE	57	0	0	0/0	0/1	0/0	0/1	0/1	0/1	0/0	0/1	0/1	0/0	0/1	0/1	0/1	0/0
Chromosome1-2545229	INS	PRECISE	1072	0	0	0/0	0/0	0/0	0/0	0/0	0/0	0/0	0/0	0/0	0/1	0/0	0/0	0/0	0/0
Chromosome1-2545331	INS	PRECISE	1696	0	0	0/0	0/0	0/0	0/0	0/0	0/0	0/0	0/0	0/0	0/1	0/0	0/0	0/0	0/0
Chromosome1-2545739	DEL	PRECISE	3527	0	0	0/1	1/1	0/0	1/1	1/1	1/1	1/1	1/1	1/1	0/1	0/1	1/1	1/1	0/0
Chromosome1-2545851	DEL	PRECISE	849	0	0	0/0	0/0	0/0	0/0	0/0	0/0	0/0	0/0	0/0	0/0	0/0	0/0	0/0	1/1
Chromosome1-2546416	INS	PRECISE	556	0	0	0/0	0/0	0/0	0/0	0/0	0/0	0/0	0/0	0/0	0/0	0/0	0/1	0/0	0/0
Chromosome1-2546667	DUP	PRECISE	3494950	0	0	0/0	0/0	0/0	0/1	0/0	0/1	0/0	0/1	0/0	0/0	0/0	0/0	0/0	0/0
Chromosome1-2547930	BND	PRECISE	0	0	0	0/0	0/0	0/0	0/0	0/0	0/0	0/0	0/0	0/0	0/0	0/0	0/0	0/0	0/1
Chromosome1-2547930	BND	PRECISE	0	0	0	0/0	0/0	0/0	0/0	0/0	0/0	0/0	0/0	0/0	0/0	0/0	0/0	0/1	0/0
Chromosome1-2557969	INS	PRECISE	1342	0	0.707	0/0	0/0	0/0	0/0	0/1	0/0	0/0	0/0	0/0	0/0	0/0	0/0	0/0	0/0
Chromosome1-2558003	INS	PRECISE	4001	0	0	0/0	0/0	0/0	0/0	0/0	0/0	0/0	0/0	0/1	0/0	0/0	0/0	0/0	0/0
Chromosome1-2560871	DEL	PRECISE	1382	0	0	0/0	0/0	0/0	0/0	0/0	0/0	0/0	0/0	0/0	1/1	0/0	0/0	0/0	0/0
Chromosome1-2561464	DEL	PRECISE	233	0	0	0/1	1/1	0/1	1/1	1/1	1/1	1/1	1/1	1/1	1/1	1/1	1/1	1/1	1/1
Chromosome1-2561898	INS	PRECISE	7387	0	0	0/0	0/0	0/0	0/0	0/0	0/0	1/1	0/0	0/0	0/0	0/0	0/0	0/0	0/0
Chromosome1-2562219	INS	PRECISE	1167	0	0	0/0	0/0	0/0	0/0	0/0	0/0	0/0	0/0	0/1	0/0	0/0	0/0	0/0	0/0
Chromosome1-2562364	DEL	PRECISE	1197	0	0	0/1	1/1	0/1	1/1	1/1	1/1	1/1	1/1	1/1	1/1	1/1	1/1	1/1	0/0
Chromosome1-2563726	DEL	PRECISE	513	0	0	0/0	0/0	0/0	0/0	0/0	0/0	0/0	0/0	0/1	0/0	0/0	0/0	0/0	0/0
Chromosome1-2564034	INS	PRECISE	464	0	0.506	0/1	1/1	0/1	1/1	1/1	1/1	1/1	1/1	0/1	0/1	1/1	1/1	1/1	0/0
Chromosome1-2564790	DEL	PRECISE	51	0	0	0/1	0/1	0/1	0/1	1/1	0/1	0/1	0/1	0/1	0/1	0/1	0/1	0/1	0/0
Chromosome1-2564963	DEL	PRECISE	1313	0	0	0/1	1/1	0/1	1/1	1/1	1/1	1/1	1/1	1/1	1/1	1/1	1/1	1/1	0/0
Chromosome1-2565290	INS	PRECISE	271	0	0.277	0/1	1/1	0/1	1/1	1/1	1/1	1/1	1/1	1/1	1/1	1/1	1/1	1/1	0/0
Chromosome1-2565520	DEL	PRECISE	13674	0	0	0/0	0/0	0/0	0/0	0/0	0/0	0/0	0/0	0/1	1/1	0/0	0/0	0/0	0/0
Chromosome1-2565776	BND	PRECISE	0	0	0	0/0	0/1	0/0	0/0	1/1	0/0	1/1	1/1	0/1	0/1	1/1	0/1	0/1	1/1
Chromosome1-2567383	DEL	PRECISE	5458	0	0	0/1	1/1	0/1	1/1	1/1	1/1	0/1	1/1	1/1	1/1	1/1	0/1	1/1	0/0
Chromosome1-2567424	INS	PRECISE	1548	0	0.577	0/0	0/0	0/0	0/0	0/0	0/0	0/0	0/0	0/0	0/0	1/1	0/0	1/1	1/1
Chromosome1-2567941	INS	PRECISE	211	0	0	0/0	0/0	0/0	0/0	0/0	0/0	0/1	0/0	0/0	0/0	0/0	0/0	0/0	0/1
Chromosome1-2568248	DEL	PRECISE	1241	0	0	0/1	1/1	0/1	1/1	1/1	1/1	0/1	1/1	1/1	1/1	1/1	1/1	1/1	1/1
Chromosome1-2568387	INS	PRECISE	88	0	0.426	0/1	1/1	0/1	1/1	1/1	1/1	0/1	1/1	1/1	1/1	1/1	1/1	1/1	1/1
Chromosome1-2569388	DEL	PRECISE	249	0	0	0/0	0/0	0/0	0/0	0/0	0/0	0/1	0/0	0/0	0/0	0/0	0/0	0/0	0/0
Chromosome1-2569780	DEL	PRECISE	54	0	0	0/1	0/1	0/0	0/0	0/0	0/0	0/0	0/1	0/0	0/1	0/1	0/1	0/0	0/1
Chromosome1-2570438	DEL	PRECISE	99	0	0	0/0	0/0	0/0	0/0	0/0	0/0	0/0	0/0	0/1	0/1	0/1	0/1	0/1	0/1
Chromosome1-2570916	INS	PRECISE	7495	0	0	0/0	0/0	0/0	0/0	0/0	0/0	0/0	0/0	0/0	0/0	0/0	1/1	0/0	0/0
Chromosome1-2570916	INS	PRECISE	6101	0	0	0/0	0/0	0/0	0/0	0/0	0/0	0/0	0/0	0/1	0/0	0/0	0/0	0/0	0/0
Chromosome1-2571029	INS	PRECISE	5148	0	0	0/0	0/0	0/0	0/1	0/0	0/0	0/0	0/0	0/0	0/0	0/0	0/0	0/0	0/0
Chromosome1-2571710	DEL	PRECISE	107	0	0	0/0	0/0	0/0	0/0	0/0	0/0	0/0	0/0	0/0	0/0	0/0	0/1	0/0	0/0
Chromosome1-2571800	BND	PRECISE	0	0	0	0/0	0/0	0/0	0/0	0/0	0/0	0/0	0/0	0/0	0/1	0/0	0/0	0/1	0/1
Chromosome1-2572501	BND	PRECISE	0	0.707	0	0/0	0/0	0/0	0/0	0/0	0/0	0/1	0/0	0/0	0/0	0/0	0/0	0/0	0/0
Chromosome1-2572734	INS	PRECISE	5499	0	0	0/0	0/0	0/0	0/0	0/0	0/0	0/0	0/0	0/0	0/0	0/0	0/0	0/0	1/1
Chromosome1-2573018	INS	PRECISE	3037	0	0	0/0	0/0	0/0	0/0	0/0	0/0	0/0	0/0	0/0	0/0	0/0	0/0	0/0	1/1
Chromosome1-2573044	INS	PRECISE	11961	0	0	0/0	0/0	0/0	0/0	0/0	0/0	0/1	0/0	0/0	0/0	0/0	0/0	0/0	0/0
Chromosome1-2573366	INS	PRECISE	622	0	0	0/0	0/0	0/0	0/0	0/0	0/0	0/1	0/0	0/0	0/0	0/0	0/0	0/0	0/0
Chromosome1-2573716	INS	PRECISE	711	0	0	0/0	0/0	0/0	0/0	0/0	0/0	0/0	0/0	0/0	0/1	0/0	0/0	0/0	0/0
Chromosome1-2574113	DEL	PRECISE	62	0	0	0/0	0/0	0/0	0/0	0/0	0/0	0/0	0/0	0/1	0/1	0/0	0/1	0/1	0/1
Chromosome1-2574570	INS	PRECISE	137	0	0	0/0	0/0	0/0	0/0	0/0	0/0	0/0	0/0	0/0	0/0	0/0	0/1	0/0	0/0
Chromosome1-2575482	INS	PRECISE	2366	0	0	0/0	0/0	0/0	0/0	0/0	0/0	0/0	0/0	0/0	0/0	0/0	0/0	0/0	0/1
Chromosome1-2576196	INS	PRECISE	55	0	0.408	0/0	0/0	0/0	0/0	0/0	0/0	0/0	0/0	0/1	0/1	0/1	0/1	0/1	0/1
Chromosome1-2576417	DEL	PRECISE	112	0	0	0/1	1/1	0/1	1/1	1/1	1/1	0/1	1/1	0/1	0/1	0/1	0/1	1/1	0/0
Chromosome1-2576810	INS	PRECISE	907	0	0.577	0/0	0/0	0/0	0/0	0/0	0/0	0/0	0/0	0/0	0/0	0/1	0/0	1/1	0/1
Chromosome1-2576954	DEL	PRECISE	1253	0	0	0/0	0/0	0/0	0/0	0/0	0/0	0/0	0/0	0/1	0/1	0/0	0/1	0/1	0/1
Chromosome1-2577533	INS	PRECISE	211	0	0.389	0/1	1/1	0/1	0/1	1/1	1/1	0/0	0/1	0/1	0/1	0/1	0/1	0/1	0/0
Chromosome1-2578623	INS	PRECISE	335	0	0.577	0/0	0/0	0/0	0/0	0/0	0/0	0/0	0/0	0/0	0/0	0/1	0/0	0/1	0/1
Chromosome1-2579284	INS	PRECISE	52	0	0.277	0/1	1/1	0/1	1/1	0/1	0/1	0/0	0/0	0/1	0/1	0/1	0/1	0/1	0/1
Chromosome1-2579473	DEL	PRECISE	62	0	0	0/0	0/0	0/0	0/0	0/0	0/0	0/0	0/0	0/1	0/1	0/1	0/1	0/1	0/1
Chromosome1-2579543	DEL	PRECISE	453	0	0	0/0	0/0	0/0	0/0	0/0	0/0	0/0	0/0	0/1	0/1	0/1	0/1	0/1	0/1
Chromosome1-2580007	DEL	PRECISE	59	0	0	0/0	0/0	0/0	0/0	0/1	0/1	0/0	0/0	0/0	0/0	0/0	0/0	0/0	0/0
Chromosome1-2580107	INS	PRECISE	150	0	0	0/0	0/0	0/0	0/0	0/0	0/0	0/1	1/1	0/0	0/0	0/0	0/0	0/0	0/0
Chromosome1-2580254	INS	PRECISE	626	0	0	0/0	0/0	0/0	0/0	0/0	0/0	0/1	0/0	0/0	0/0	0/0	0/0	0/0	0/0

Chromosome1-2580503	INS	PRECISE	126	0	0	0/0	0/0	0/0	0/0	0/0	0/0	0/0	0/0	0/0	0/0	0/0	0/0	0/0	0/1
Chromosome1-2582579	DEL	PRECISE	4961	0	0	0/1	1/1	0/1	1/1	1/1	1/1	1/1	1/1	1/1	1/1	1/1	1/1	1/1	1/1
Chromosome1-2583199	DEL	PRECISE	3954	0	0	0/1	1/1	0/1	1/1	1/1	1/1	1/1	1/1	1/1	1/1	1/1	1/1	1/1	1/1
Chromosome1-2583371	BND	PRECISE	0	0	0	0/1	0/1	0/1	0/0	0/0	0/1	0/0	0/1	0/0	0/0	0/0	0/0	0/0	0/0
Chromosome1-2584411	DEL	PRECISE	50	0	0	0/0	0/0	0/0	0/0	0/0	0/0	0/1	0/0	0/0	0/0	0/0	0/0	0/0	0/0
Chromosome1-2584455	INS	PRECISE	132	0	0	0/0	0/0	0/0	0/0	0/0	0/0	0/1	0/1	0/0	0/0	0/0	0/0	0/0	0/0
Chromosome1-2584693	INS	PRECISE	327	0	0.707	0/0	0/0	0/0	0/0	0/1	0/1	0/0	0/0	0/0	0/0	0/0	0/0	0/0	0/0
Chromosome1-2584923	INS	PRECISE	5835	0	0	0/0	0/0	1/1	0/0	0/0	0/0	0/0	0/0	0/0	0/0	0/0	0/0	0/0	0/0
Chromosome1-2584975	INS	PRECISE	677	0	0.577	0/0	0/0	0/0	0/0	0/0	0/0	0/0	0/0	0/0	0/0	0/0	0/0	1/1	0/1
Chromosome1-2585038	INS	PRECISE	1458	0	0	0/0	0/0	0/0	0/0	0/0	0/0	1/1	0/0	0/0	0/0	0/0	0/0	0/0	0/0
Chromosome1-2585406	INS	PRECISE	187	0	0	0/0	0/0	0/0	0/0	0/0	0/0	0/0	0/0	0/0	0/1	0/0	0/0	0/0	0/0
Chromosome1-2585523	INS	PRECISE	841	0	0	0/0	0/0	0/0	0/0	0/0	0/0	0/0	0/0	1/1	0/0	0/0	0/1	0/0	0/0
Chromosome1-2585882	INS	PRECISE	679	0	0.707	0/0	0/0	0/0	0/0	0/0	0/0	0/0	0/0	1/1	0/0	0/0	0/1	0/0	0/0
Chromosome1-2586000	INS	PRECISE	5143	0	0	0/0	0/0	0/0	0/0	0/0	0/0	0/0	0/0	0/0	1/1	0/0	0/0	0/0	0/0
Chromosome1-2586000	BND	PRECISE	0	0	0	0/0	0/0	0/0	0/0	0/0	0/0	0/0	0/0	0/0	0/1	0/0	0/0	0/0	0/0
Chromosome1-2586232	DEL	PRECISE	68	0	0	0/0	0/0	0/0	0/0	0/0	0/0	0/0	0/0	0/0	0/1	0/1	0/0	1/1	0/1
Chromosome1-2586697	DEL	PRECISE	61	0	0	0/1	1/1	0/1	1/1	0/1	0/1	1/1	1/1	1/1	1/1	1/1	1/1	1/1	1/1
Chromosome1-2586853	INS	PRECISE	662	0	0	0/0	0/0	0/0	0/0	0/0	0/0	0/0	0/0	0/0	0/0	0/0	0/0	0/0	1/1
Chromosome1-2587138	INS	PRECISE	89	0	0	0/0	0/0	0/0	0/0	0/0	0/0	0/1	0/0	0/0	0/0	0/0	0/0	0/0	0/0
Chromosome1-2587145	DEL	PRECISE	75	0	0	0/0	0/1	0/1	0/1	0/1	0/1	0/0	0/0	0/1	0/0	0/0	0/0	0/1	0/0
Chromosome1-2587147	INS	PRECISE	8178	0	0	0/0	0/0	0/0	1/1	0/0	0/0	0/0	0/0	0/0	0/0	0/0	0/0	0/0	0/0
Chromosome1-2587618	DEL	PRECISE	623	0	0	0/1	1/1	0/1	1/1	1/1	1/1	0/1	1/1	1/1	1/1	1/1	1/1	1/1	1/1
Chromosome1-2588441	INS	PRECISE	137	0	0	0/0	0/0	0/0	0/0	0/0	0/0	0/0	0/0	0/0	0/1	0/0	0/0	0/0	0/0
Chromosome1-2589726	DEL	PRECISE	150	0	0	0/1	1/1	0/1	1/1	1/1	1/1	1/1	1/1	1/1	1/1	1/1	1/1	1/1	1/1
Chromosome1-2589873	INS	PRECISE	1431	0	0.707	0/0	0/0	0/0	0/0	0/0	0/0	0/1	0/1	0/0	0/0	0/0	0/0	0/0	0/0
Chromosome1-2589923	INS	PRECISE	234	0	0	0/0	0/0	0/0	0/0	0/0	0/0	0/0	0/0	0/0	0/0	0/0	0/0	0/0	0/1
Chromosome1-2590002	INS	PRECISE	620	0	0	0/0	0/0	0/0	0/0	0/1	0/1	0/0	0/0	0/0	0/0	0/0	0/0	0/0	0/0
Chromosome1-2590636	BND	PRECISE	0	0	0	0/0	0/0	0/0	0/0	0/1	0/0	0/0	0/0	0/0	0/0	0/0	0/0	0/0	0/0
Chromosome1-2590653	DEL	PRECISE	3342	0	0	0/0	0/0	0/0	0/0	0/0	0/0	0/0	0/0	0/0	0/0	0/0	0/0	0/1	0/1
Chromosome1-2590653	DEL	PRECISE	4490	0.802	0.535	0/1	1/1	0/1	1/1	1/1	1/1	1/1	1/1	0/1	1/1	0/1	1/1	0/1	0/1
Chromosome1-2591363	DEL	PRECISE	60	0	0	0/0	0/0	0/0	0/0	0/0	0/0	0/0	0/0	0/1	0/0	0/1	0/1	0/1	0/1
Chromosome1-2593815	DEL	PRECISE	85	0	0	0/0	0/0	0/0	0/0	0/0	0/0	0/0	0/0	0/1	0/1	0/0	0/1	0/1	0/1
Chromosome1-2594917	DEL	PRECISE	67	0	0	0/0	0/0	0/0	0/0	0/0	0/0	0/0	0/0	0/0	0/0	0/0	0/0	0/0	1/1
Chromosome1-2596240	INS	PRECISE	56	0	0	0/0	0/0	0/0	0/0	0/0	0/0	0/0	0/0	0/0	0/0	0/0	0/0	0/0	0/1
Chromosome1-2598295	INS	PRECISE	150	0	0	0/0	0/0	0/0	0/0	0/0	0/0	0/0	0/0	0/0	0/0	0/0	0/0	0/0	0/1
Chromosome1-2598408	DEL	PRECISE	470	0	0	0/0	0/0	0/0	0/0	0/0	0/0	0/0	0/0	0/1	0/1	0/1	0/1	0/1	0/1
Chromosome1-2598834	INS	PRECISE	2144	0	0	0/0	0/0	0/0	0/0	0/0	0/0	0/1	0/0	0/0	0/0	0/0	0/0	0/0	0/0
Chromosome1-2599091	DEL	PRECISE	55	0	0	0/0	0/0	0/0	0/0	0/0	0/0	0/1	0/0	0/0	0/0	0/0	0/0	0/0	0/0
Chromosome1-2599565	DEL	PRECISE	61	0	0	0/0	0/0	0/0	0/0	0/0	0/0	0/0	0/0	0/1	0/1	0/1	0/1	0/1	0/1
Chromosome1-2601509	INS	PRECISE	334	0	0.69	0/0	0/1	0/1	0/1	0/1	0/1	0/0	0/0	0/1	0/0	0/0	0/0	1/1	0/0
Chromosome1-2601781	INS	PRECISE	137	0	0.707	0/0	0/0	0/0	0/0	0/0	0/0	0/0	0/0	0/1	0/1	0/0	0/0	0/0	0/0
Chromosome1-2601977	INS	PRECISE	60	0	0	0/0	0/0	0/0	0/0	0/0	0/0	0/0	0/0	0/1	0/1	0/0	0/0	0/0	0/0
Chromosome1-2602488	DEL	PRECISE	445	0	0	0/1	1/1	0/1	1/1	1/1	1/1	1/1	1/1	1/1	1/1	1/1	1/1	1/1	1/1
Chromosome1-2602994	INS	PRECISE	150	0	0	0/0	0/0	0/0	0/0	0/0	0/0	0/0	0/0	0/0	0/0	0/0	0/0	0/0	0/1
Chromosome1-2603074	INS	PRECISE	582	0	0	0/0	0/0	0/0	0/0	0/0	0/0	0/0	0/0	0/1	0/1	0/0	0/0	0/0	0/0
Chromosome1-2603511	INS	PRECISE	199	0	0.707	0/0	0/0	0/0	0/0	0/1	0/1	0/0	0/0	0/0	0/0	0/0	0/0	0/0	0/0
Chromosome1-2604639	INS	PRECISE	211	0	0	0/0	0/0	0/0	0/0	0/1	1/1	0/0	0/0	0/0	0/0	0/0	0/0	0/0	0/0
Chromosome1-2604730	DEL	PRECISE	728	0	0	0/0	0/0	0/0	0/0	0/0	0/0	0/0	0/0	0/0	0/0	0/0	0/1	0/0	0/0
Chromosome1-2604876	DEL	PRECISE	230	0	0	0/0	0/0	0/0	0/0	0/0	0/0	0/1	0/0	0/0	0/0	0/0	0/0	0/0	0/0
Chromosome1-2607408	INS	PRECISE	6725	0	0	0/0	0/0	0/0	0/0	0/0	0/0	0/0	0/0	0/0	0/0	0/0	0/0	0/0	1/1
Chromosome1-2607602	DEL	PRECISE	331	0	0	0/1	1/1	0/1	1/1	1/1	1/1	1/1	1/1	1/1	1/1	1/1	1/1	1/1	1/1
Chromosome1-2609221	INS	PRECISE	77	0	0	0/0	0/0	0/0	0/0	0/0	0/0	0/0	0/0	0/0	0/0	0/0	0/0	0/0	0/1
Chromosome1-2609381	INS	PRECISE	1374	0	0	0/0	0/0	0/0	0/0	0/0	0/0	0/0	0/0	0/1	0/0	0/0	0/0	0/0	0/0
Chromosome1-2609557	INS	PRECISE	1360	0	0	0/0	0/0	0/0	0/0	0/0	0/0	0/1	0/0	0/0	0/0	0/0	0/0	0/0	0/0
Chromosome1-2609597	INS	PRECISE	187	0	0.5	0/0	0/0	0/0	0/0	0/0	0/0	0/0	0/0	0/0	0/1	0/1	0/0	0/1	0/1
Chromosome1-2609746	DEL	PRECISE	211	0.267	0	0/1	1/1	0/1	1/1	1/1	1/1	1/1	1/1	1/1	1/1	1/1	1/1	1/1	1/1
Chromosome1-2609975	INS	PRECISE	828	0	0	0/0	0/0	0/0	0/0	0/0	0/0	0/1	0/0	0/0	0/0	0/0	0/0	0/0	0/0
Chromosome1-2610129	DEL	PRECISE	78	0	0	0/0	0/0	0/0	0/0	0/0	0/0	0/0	0/0	0/0	0/1	0/0	0/0	0/0	0/0
Chromosome1-2610325	INS	PRECISE	5070	0	0	0/0	0/0	0/0	1/1	0/0	0/0	0/0	0/0	0/0	0/0	0/0	0/0	0/0	0/0
Chromosome1-2610479	INS	PRECISE	239	0	0	0/0	0/0	0/0	0/0	0/1	0/1	0/0	0/0	0/0	0/0	0/0	0/0	0/0	0/0
Chromosome1-2610653	DEL	PRECISE	63	0	0	0/0	0/0	0/0	0/0	0/0	0/0	0/0	0/0	0/1	0/1	0/1	0/1	1/1	0/1
Chromosome1-2610861	INS	PRECISE	7241	0	0	0/0	0/0	0/0	0/0	0/0	0/0	0/0	0/0	0/0	0/0	0/0	0/0	0/0	0/1
Chromosome1-2610862	BND	PRECISE	0	0	0	0/0	0/0	0/0	0/0	0/0	0/0	0/0	0/0	0/0	0/0	0/0	0/0	0/0	0/1
Chromosome1-2610881	INS	PRECISE	1241	0	0.5	0/0	0/0	0/0	0/0	0/0	0/0	0/0	0/0	0/1	0/0	0/1	0/0	1/1	0/1
Chromosome1-2610886	INS	PRECISE	212	0	0	0/0	0/0	0/0	0/0	0/0	0/0	0/0	0/0	0/0	0/1	0/0	0/1	0/0	0/0
Chromosome1-2611553	DEL	PRECISE	66	0	0	0/1	1/1	0/1	1/1	0/1	0/1	0/0	0/1	0/1	0/1	0/1	0/1	0/1	0/0
Chromosome1-2612253	DEL	PRECISE	910	0.447	0.447	0/0	0/0	0/0	0/0	0/0	0/0	0/0	0/0	0/1	0/0	0/1	0/0	0/1	1/1
Chromosome1-2612506	DEL	PRECISE	59	0	0	0/0	0/0	0/0	0/0	0/0	0/0	0/1	0/0	0/0	0/0	0/0	0/0	0/0	0/0
Chromosome1-2612956	INS	PRECISE	10750	0	0	0/0	0/0	0/0	0/0	0/0	0/0	0/1	0/0	0/0	0/0	0/0	0/0	0/0	0/0
Chromosome1-2612957	BND	PRECISE	0	0	0	0/0	0/0	0/0	0/0	0/0	0/0	0/1	0/0	0/0	0/0	0/0	0/0	0/0	0/0
Chromosome1-2613130	INS	PRECISE	885	0	0	0/0	0/0	0/0	0/0	0/0	0/0	0/0	0/0	0/0	0/0	0/0	0/0	0/0	0/1
Chromosome1-2613445	INS	PRECISE	210	0	0.577	0/0	0/0	0/0	0/1	0/0	0/0	0/1	0/1	0/0	0/0	0/0	0/0	0/0	0/0
Chromosome1-2614172	DUP	PRECISE	404	0	0	0/0	0/0	0/0	0/0	0/0	0/0	0/0	0/0	0/0	0/0	0/1	0/0	0/0	0/0

Chromosome1-2614342	DEL	PRECISE	6132	0	0	0/1	1/1	0/1	1/1	0/1	0/1	0/1	1/1	1/1	1/1	1/1	1/1	1/1	1/1
Chromosome1-2614942	BND	PRECISE	0	0	0	0/0	0/0	0/0	0/0	0/0	0/0	0/0	0/0	0/0	0/0	0/0	0/0	0/0	0/1
Chromosome1-2615077	INS	PRECISE	796	0	0	0/0	0/0	0/0	0/0	0/0	0/0	0/0	0/0	0/0	0/1	0/0	0/0	0/0	0/0
Chromosome1-2615797	INS	PRECISE	96	0	0	0/0	0/0	0/0	0/0	0/0	0/0	0/0	0/0	0/1	0/0	0/1	0/1	0/1	0/1
Chromosome1-2616376	DEL	PRECISE	56	0	0	0/0	0/1	0/1	0/1	0/1	0/1	0/0	0/0	0/1	0/0	0/0	0/0	0/1	0/0
Chromosome1-2616688	INS	PRECISE	212	0	0.363	0/1	1/1	0/1	1/1	0/1	0/1	0/1	1/1	1/1	1/1	1/1	1/1	1/1	0/1
Chromosome1-2617602	INS	PRECISE	54	0	0.408	0/0	0/0	0/0	0/0	0/0	0/0	0/0	0/0	0/1	0/1	0/1	0/1	0/1	0/1
Chromosome1-2617926	INS	PRECISE	132	0	0	0/0	0/0	0/0	0/0	0/0	0/0	0/1	0/1	0/0	0/0	0/0	0/0	0/0	0/0
Chromosome1-2618569	INS	PRECISE	192	0	0	0/0	0/0	0/0	0/0	0/0	0/0	0/0	0/0	0/0	0/0	0/0	0/0	0/0	1/1
Chromosome1-2619076	INS	PRECISE	605	0	0.548	0/0	0/0	0/0	0/0	0/0	0/0	0/0	0/0	0/1	0/0	0/1	0/0	0/1	0/0
Chromosome1-2619439	INS	PRECISE	173	0	0	0/0	0/0	0/0	0/1	0/0	0/0	0/0	0/0	0/0	0/0	0/0	0/0	0/0	0/0
Chromosome1-2619971	DEL	PRECISE	364	0	0	0/0	0/0	0/0	0/0	0/0	0/0	0/0	0/0	0/0	0/0	0/0	0/0	0/0	0/1
Chromosome1-2620052	DEL	PRECISE	58	0	0	0/0	0/0	0/0	0/0	0/0	0/0	0/0	0/0	0/0	0/0	0/0	0/0	0/0	0/1
Chromosome1-2621092	INS	PRECISE	259	0	0	0/0	0/0	0/0	0/0	0/0	0/0	0/0	0/0	0/0	0/0	0/0	0/0	0/0	0/1
Chromosome1-2622104	INS	PRECISE	151	0	0	0/0	0/0	0/0	0/0	0/1	0/1	0/1	0/1	0/0	0/0	0/0	0/0	0/0	0/0
Chromosome1-2622661	INS	PRECISE	8405	0	0	0/0	0/0	0/0	0/0	0/0	0/0	0/0	0/0	1/1	0/0	0/0	0/0	0/0	0/0
Chromosome1-2622667	BND	PRECISE	0	0	0	0/0	0/0	0/0	0/0	0/1	1/1	0/1	0/1	0/0	0/0	0/0	0/0	0/0	0/0
Chromosome1-2623112	DEL	PRECISE	153	0	0	0/0	0/0	0/0	0/0	0/0	0/0	0/0	0/0	0/1	0/1	0/1	0/1	0/1	0/1
Chromosome1-2623297	DEL	PRECISE	86	0	0	0/1	1/1	0/1	0/1	0/1	0/1	0/1	0/1	0/1	0/1	0/1	0/1	0/1	0/0
Chromosome1-2623315	INS	PRECISE	79	0.302	0.505	0/0	0/0	0/0	0/1	0/1	0/1	0/1	0/1	0/1	0/1	0/1	0/1	0/1	1/1
Chromosome1-2624018	DEL	PRECISE	94	0	0	0/1	1/1	0/1	0/1	0/1	0/1	0/1	0/1	0/1	0/1	0/1	0/1	0/1	0/0
Chromosome1-2624245	INS	PRECISE	211	0	0.548	0/0	0/0	0/0	0/1	0/1	0/1	0/1	0/1	0/0	0/0	0/0	0/0	0/0	0/0
Chromosome1-2624334	DEL	PRECISE	52	0.548	0.548	0/0	0/0	0/0	0/1	0/1	0/1	0/1	0/1	0/0	0/0	0/0	0/0	0/0	0/0
Chromosome1-2624535	DEL	PRECISE	2896	0	0	0/0	0/0	0/0	0/0	0/0	0/0	0/0	0/0	0/1	0/1	0/1	0/1	0/1	0/1
Chromosome1-2624810	INS	PRECISE	132	0	0.447	0/1	0/1	0/0	0/0	0/0	0/0	0/0	0/0	0/0	0/1	0/1	0/1	0/0	0/0
Chromosome1-2626023	DEL	PRECISE	60	0	0	0/0	0/0	0/0	0/0	0/0	0/0	0/0	0/0	0/1	0/1	0/1	0/1	0/1	0/1
Chromosome1-2627163	INS	PRECISE	3887	0	0	0/0	0/0	0/0	0/0	0/0	0/0	0/0	0/0	0/0	0/0	0/0	0/0	0/0	1/1
Chromosome1-2628370	DEL	PRECISE	60	0	0	0/0	0/0	0/0	0/0	0/0	0/0	0/0	0/0	0/1	0/1	0/1	0/1	0/1	0/1
Chromosome1-2628394	INS	PRECISE	172	0	0.983	0/0	0/0	0/0	0/0	0/0	0/0	0/0	0/0	0/1	0/1	0/1	0/1	0/1	0/1
Chromosome1-2628884	INS	PRECISE	4908	0	0	0/0	0/0	0/0	0/0	0/0	0/0	0/0	0/0	0/0	0/0	0/0	0/0	0/0	1/1
Chromosome1-2630186	BND	PRECISE	0	0	0	0/0	0/0	0/0	0/0	0/0	0/0	0/0	0/1	0/0	0/0	0/0	0/0	0/0	0/0
Chromosome1-2630776	DEL	PRECISE	167	0	0	0/0	0/0	0/0	0/0	0/0	0/0	0/1	0/1	0/0	0/0	0/0	0/0	0/0	0/0
Chromosome1-2631167	BND	PRECISE	0	0	0	0/0	0/0	0/0	0/0	0/0	0/0	0/1	0/0	0/0	0/0	0/0	0/0	0/0	0/0
Chromosome1-2631291	DEL	PRECISE	95	0	0	0/0	0/0	0/0	0/0	0/0	0/0	0/0	0/0	0/1	0/0	0/0	0/0	0/1	0/1
Chromosome1-2631819	DEL	PRECISE	240	0	0	0/0	0/0	0/0	0/0	0/0	0/0	0/0	0/0	0/0	0/1	0/0	0/0	0/0	0/0
Chromosome1-2631824	INS	PRECISE	374	0	0	0/0	0/0	0/0	0/1	0/1	0/1	0/1	0/1	0/0	0/0	0/0	0/0	0/0	0/0
Chromosome1-2631947	DEL	PRECISE	237	0	0	0/0	0/0	0/0	0/0	0/0	0/0	0/0	0/0	0/0	0/0	0/0	0/0	0/1	0/1
Chromosome1-2632020	INS	PRECISE	2080	0	0	0/0	0/0	0/0	0/0	0/0	0/0	0/0	0/0	0/0	0/0	0/0	1/1	0/0	0/0
Chromosome1-2632180	DEL	PRECISE	1074	0	0	0/0	0/0	0/0	0/0	0/0	0/0	0/0	0/0	0/0	0/1	0/0	0/1	0/0	0/1
Chromosome1-2632915	DEL	PRECISE	211	0.447	0.447	0/0	0/0	0/0	0/0	0/0	0/0	0/0	0/0	0/1	0/1	0/0	0/1	0/0	1/1
Chromosome1-2633112	INS	PRECISE	1160	0	0	0/0	0/0	0/0	0/0	0/0	0/0	0/0	0/0	0/0	0/0	0/0	0/0	0/0	0/1
Chromosome1-2633175	DEL	PRECISE	110	0	0	0/0	0/0	0/0	0/0	0/0	0/0	0/1	0/1	0/0	0/0	0/0	0/0	0/0	0/0
Chromosome1-2634606	DEL	PRECISE	973	0	0	0/0	0/0	0/0	0/0	0/0	0/0	0/0	0/0	0/1	1/1	1/1	0/0	1/1	0/1
Chromosome1-2634664	BND	PRECISE	0	0	0	0/0	0/1	0/0	0/0	0/0	0/0	0/0	0/0	0/0	1/1	0/0	0/0	0/0	0/0
Chromosome1-2634664	BND	PRECISE	0	0	0	0/1	0/1	0/0	0/0	0/0	0/0	0/0	0/0	0/0	1/1	0/0	0/0	0/0	0/0
Chromosome1-2635048	INS	PRECISE	93	0	0.5	0/0	0/0	0/0	0/0	0/0	0/0	0/1	0/0	0/0	0/0	0/0	0/0	0/0	0/0
Chromosome1-2636180	INS	PRECISE	137	0	0.447	0/0	0/0	0/0	0/0	0/0	0/0	0/0	0/0	0/1	0/0	0/1	0/0	0/1	0/1
Chromosome1-2636374	INS	PRECISE	52	0	0	0/0	0/0	0/0	0/0	0/0	0/0	0/0	0/0	0/0	0/0	0/0	0/0	0/0	0/1
Chromosome1-2638070	INS	PRECISE	343	0	0	0/0	0/0	0/0	0/0	0/0	0/0	0/0	0/0	0/0	0/1	0/0	0/0	0/0	0/0
Chromosome1-2638376	DEL	PRECISE	53	0	0	0/0	0/1	0/1	1/1	1/1	1/1	0/1	0/1	0/1	0/0	0/1	0/0	0/1	0/1
Chromosome1-2638837	BND	PRECISE	0	0	0	0/0	0/0	0/0	0/0	0/0	0/0	0/0	0/0	0/0	0/0	0/0	0/0	0/0	0/1
Chromosome1-2638999	INS	PRECISE	161	0	0	0/0	0/0	0/0	0/0	0/0	0/0	0/0	0/0	0/1	0/0	0/1	0/0	0/1	0/1
Chromosome1-2639218	DEL	PRECISE	93	0	0	0/1	1/1	0/1	1/1	1/1	1/1	1/1	1/1	1/1	1/1	1/1	1/1	1/1	1/1
Chromosome1-2639318	DEL	PRECISE	1215	0	0	0/0	0/0	0/0	0/0	0/0	0/0	0/0	0/0	1/1	0/1	0/1	1/1	0/1	0/1
Chromosome1-2640382	DEL	PRECISE	653	0	0	0/1	1/1	0/1	1/1	1/1	1/1	1/1	1/1	1/1	1/1	1/1	1/1	1/1	1/1
Chromosome1-2640560	INS	PRECISE	2530	0	0	0/0	0/0	0/0	0/0	0/0	0/0	0/0	0/0	0/0	0/1	0/0	0/0	0/0	0/0
Chromosome1-2640772	DEL	PRECISE	150	0	0	0/0	0/0	0/0	0/0	0/0	0/0	0/0	0/0	0/1	0/1	0/1	0/1	0/1	1/1
Chromosome1-2640974	DEL	PRECISE	360	0	0	0/0	0/0	0/0	0/0	0/0	0/0	0/0	0/0	0/1	0/1	0/1	0/1	0/1	0/1
Chromosome1-2642729	INS	PRECISE	181	0	0	0/0	0/0	0/0	0/0	0/0	0/0	0/0	0/0	0/0	0/0	0/0	0/0	0/0	0/1
Chromosome1-2642929	DEL	PRECISE	58	0	0	0/1	1/1	0/1	1/1	1/1	1/1	1/1	1/1	0/1	0/1	0/1	0/1	0/1	0/1
Chromosome1-2644078	DEL	PRECISE	89	0	0	0/1	1/1	0/1	0/1	0/1	0/1	0/0	0/0	0/1	0/1	0/1	0/1	0/1	0/0
Chromosome1-2644321	INS	PRECISE	1016	0	0	0/0	0/0	0/0	0/0	0/0	0/0	0/0	0/0	0/0	0/0	0/0	0/0	0/0	0/1
Chromosome1-2644446	DEL	PRECISE	834	0	0	0/0	0/0	0/0	0/0	0/0	0/0	0/0	0/0	0/1	0/0	0/1	0/1	0/1	0/1
Chromosome1-2644638	INS	PRECISE	137	0	0	0/0	0/0	0/0	0/0	0/0	0/0	0/0	0/0	0/0	0/0	0/0	0/0	0/0	0/1
Chromosome1-2644943	DEL	PRECISE	335	0	0	0/0	0/0	0/0	0/1	0/1	0/1	0/1	0/1	0/1	0/0	0/0	0/1	0/1	0/1
Chromosome1-2645142	DEL	PRECISE	52	0	0	0/1	1/1	0/1	0/1	0/1	0/1	0/1	0/1	0/1	0/1	0/1	0/1	0/1	0/1
Chromosome1-2646681	INS	PRECISE	200	0	0	0/1	0/1	0/0	0/0	0/0	0/0	0/0	0/0	0/0	0/1	0/0	0/1	0/0	0/0
Chromosome1-2647539	DEL	PRECISE	309	0	0	0/0	0/0	0/0	0/0	0/0	0/0	0/0	0/0	0/1	0/1	0/1	0/1	0/1	0/1
Chromosome1-2647791	INS	PRECISE	334	0	0	0/0	0/0	0/0	0/0	0/0	0/0	0/0	0/0	0/0	0/0	0/0	1/1	0/0	0/0
Chromosome1-2648348	INS	PRECISE	60	0	0	0/0	0/0	0/0	0/0	0/0	0/0	0/0	0/0	0/0	0/0	0/0	0/1	0/0	0/0
Chromosome1-2648870	DEL	PRECISE	73	0	0	0/0	1/1	0/1	0/1	1/1	0/1	0/1	0/0	0/1	0/0	0/1	0/0	0/1	0/1
Chromosome1-2648940	INS	PRECISE	997	0.5	0.957	0/0	0/0	0/0	0/0	0/0	0/0	0/0	0/0	0/1	0/0	0/1	0/0	0/1	0/1
Chromosome1-2648972	INS	PRECISE	161	0	0	0/0	0/0	0/0	0/0	0/0	0/0	0/0	0/0	0/0	0/0	0/0	0/0	0/0	0/1

Chromosome1-2649407	INS	PRECISE	623	0	0.5	0/1	0/1	0/0	0/0	0/0	0/0	0/0	0/0	0/0	0/1	0/0	0/1	0/0	0/0
Chromosome1-2649552	BND	PRECISE	0	0	0	0/0	0/0	0/0	0/0	0/0	0/0	0/0	0/0	0/0	0/1	0/0	0/0	0/0	0/0
Chromosome1-2649621	DEL	PRECISE	99	0	0	0/0	0/0	0/0	0/0	0/0	0/0	0/1	0/1	0/0	0/0	0/0	0/0	0/0	0/0
Chromosome1-2649784	DEL	PRECISE	261	0	0	0/0	0/0	0/0	0/0	0/0	0/0	0/0	0/0	0/1	0/0	0/1	0/1	0/1	0/1
Chromosome1-2651541	INS	PRECISE	714	0	0	0/0	0/0	0/0	0/0	0/0	0/0	0/0	0/0	0/1	0/0	0/1	0/0	0/1	0/0
Chromosome1-2652147	INS	PRECISE	137	0	0	0/0	0/0	0/0	0/1	0/1	0/1	0/1	0/1	0/0	0/0	0/0	0/0	0/0	0/0
Chromosome1-2652886	DEL	PRECISE	150	0	0	0/1	0/1	0/0	0/1	0/1	0/1	0/1	1/1	0/1	1/1	0/1	1/1	0/1	1/1
Chromosome1-2653109	DEL	PRECISE	751	0	0	0/0	0/0	0/0	0/1	0/1	0/1	0/1	0/1	0/0	0/0	0/0	0/0	0/0	0/0
Chromosome1-2655233	DEL	PRECISE	77	0	0	0/0	0/0	0/0	0/0	0/0	0/0	0/0	0/0	0/0	0/0	0/0	1/1	0/0	0/0
Chromosome1-2657169	DEL	PRECISE	174	0	0	0/1	0/1	0/0	0/1	0/1	0/1	0/1	0/1	0/0	0/1	0/0	0/1	0/0	0/1
Chromosome1-2657320	DEL	PRECISE	56	0	0	0/0	0/0	0/0	0/1	0/0	0/0	0/0	0/0	0/0	0/0	0/0	0/0	0/0	0/0
Chromosome1-2657379	INS	PRECISE	146	0	0	0/0	0/0	0/0	0/0	0/0	0/0	0/0	0/0	0/0	0/0	0/0	0/0	0/0	0/1
Chromosome1-2657438	DEL	PRECISE	156	0	0	0/0	0/0	0/0	0/0	0/0	0/0	0/0	0/0	0/0	0/0	0/0	0/0	0/0	0/1
Chromosome1-2658304	INS	PRECISE	458	0	0	0/0	0/0	0/0	0/0	0/0	0/0	0/0	0/0	0/0	0/0	0/0	0/0	0/0	0/1
Chromosome1-2658647	DEL	PRECISE	94	0	0	0/0	0/0	0/0	0/1	0/0	0/0	0/0	0/0	0/0	0/0	0/0	0/0	0/0	0/0
Chromosome1-2658681	BND	PRECISE	0	0	0	0/0	0/0	0/0	0/0	0/0	0/0	0/0	0/0	0/1	0/1	0/1	0/1	0/0	0/0
Chromosome1-2658975	DEL	PRECISE	1234	0	0	0/0	0/0	0/0	1/1	0/1	0/1	0/1	0/1	0/0	0/0	0/0	0/0	0/0	0/0
Chromosome1-2659105	INS	PRECISE	1443	0	0	0/0	0/0	0/0	0/0	0/0	0/0	0/0	0/0	0/0	0/0	0/0	0/0	0/0	1/1
Chromosome1-2659301	DEL	PRECISE	7104	0	0	0/0	0/0	0/0	0/0	0/0	0/0	0/1	0/0	0/0	0/0	0/0	0/0	0/0	0/0
Chromosome1-2659677	DEL	PRECISE	88	0.447	0.447	0/0	0/0	0/0	1/1	0/1	0/1	0/1	1/1	0/0	0/0	0/0	0/0	0/0	0/0
Chromosome1-2660010	INS	PRECISE	1230	0	0.69	0/0	0/1	0/1	0/0	0/1	1/1	0/0	0/0	0/1	0/0	0/1	0/0	0/1	0/0
Chromosome1-2660020	DEL	PRECISE	1886	0	0	0/0	0/0	0/0	0/0	0/0	0/0	0/1	1/1	0/0	0/0	0/0	0/0	0/0	0/0
Chromosome1-2660054	BND	PRECISE	0	0	0	0/0	0/0	0/0	0/0	0/0	0/0	0/0	0/0	0/0	0/0	0/0	0/0	0/0	0/1
Chromosome1-2660909	DEL	PRECISE	87	0	0	0/0	0/0	0/0	0/0	0/0	0/0	0/0	0/0	0/1	0/0	0/0	0/0	0/1	0/1
Chromosome1-2662144	INS	PRECISE	360	0	0	0/0	0/0	0/0	0/0	0/0	0/0	0/0	0/0	0/0	0/0	0/0	0/0	0/0	0/1
Chromosome1-2662864	DEL	PRECISE	86	0	0	0/0	0/1	0/1	0/0	0/1	0/1	0/0	0/0	0/1	0/0	0/1	0/0	0/1	0/0
Chromosome1-2663061	DEL	PRECISE	421	0.302	0.302	0/0	0/0	0/0	1/1	0/1	0/1	0/1	0/1	0/1	0/1	0/1	0/1	0/1	0/1
Chromosome1-2664287	INS	PRECISE	122	0	0	0/0	0/0	0/0	0/0	0/0	0/0	0/0	0/0	0/0	0/0	0/0	0/0	0/0	0/1
Chromosome1-2664739	DEL	PRECISE	1169	0	0	0/1	1/1	0/1	1/1	1/1	1/1	1/1	1/1	1/1	1/1	1/1	1/1	1/1	1/1
Chromosome1-2665902	INS	PRECISE	848	0	0	0/0	0/0	0/0	0/0	0/0	0/0	0/0	0/0	0/0	0/0	0/0	0/0	0/0	0/1
Chromosome1-2665969	DEL	PRECISE	839	0	0	0/0	0/0	0/0	0/0	0/0	0/0	0/0	0/0	0/1	0/1	0/1	0/1	0/1	0/1
Chromosome1-2666157	INS	PRECISE	50	0	0	0/0	0/0	0/0	0/0	0/0	0/0	0/0	0/0	0/1	0/1	0/1	0/1	0/1	0/1
Chromosome1-2666330	INS	PRECISE	644	0	0.5	0/0	0/0	0/0	0/0	0/0	0/0	0/0	0/0	0/1	0/0	0/1	0/0	0/1	0/1
Chromosome1-2666921	DEL	PRECISE	181	0	0	0/0	0/0	0/0	0/0	0/0	0/0	0/0	0/0	0/0	0/0	0/0	0/0	0/0	0/1
Chromosome1-2667909	INS	PRECISE	549	0	0.816	0/1	0/1	0/0	0/0	0/0	0/0	0/0	0/0	0/0	0/1	0/0	0/0	0/0	0/0
Chromosome1-2668163	INS	PRECISE	1294	0	0.894	0/0	0/0	0/0	1/1	0/1	0/1	0/1	0/1	0/0	0/0	0/0	0/0	0/0	0/0
Chromosome1-2668433	DEL	PRECISE	56	0	0	0/0	0/0	0/0	0/0	0/0	0/0	0/1	0/0	0/0	0/0	0/0	0/0	0/0	0/0
Chromosome1-2669613	INS	PRECISE	1266	0	0	0/0	0/0	0/0	0/0	0/0	0/0	0/0	0/0	0/0	0/0	0/0	0/1	0/0	0/0
Chromosome1-2669653	INS	PRECISE	12542	0	0	0/0	0/0	0/0	0/0	0/0	0/0	0/0	0/0	0/0	0/0	0/0	1/1	0/0	0/0
Chromosome1-2669653	BND	PRECISE	0	0	0	0/0	0/1	0/0	0/0	0/0	0/0	0/0	0/0	0/0	0/0	0/0	0/0	0/0	0/0
Chromosome1-2669653	BND	PRECISE	0	0	0	0/1	0/0	0/0	0/0	0/0	0/0	0/0	0/0	0/0	0/0	0/0	0/0	0/0	0/0
Chromosome1-2669723	DEL	PRECISE	56	0	0	0/0	0/0	0/0	1/1	0/0	0/0	0/0	0/0	0/0	0/0	0/0	0/0	0/0	0/0
Chromosome1-2669855	DEL	PRECISE	137	0	0	0/0	0/0	0/0	0/0	0/0	0/0	0/0	0/0	0/1	0/1	0/1	0/1	0/1	1/1
Chromosome1-2670288	INS	PRECISE	1252	0	0	0/1	0/1	0/0	0/0	0/0	0/0	0/0	0/0	0/0	1/1	0/0	0/1	0/0	0/0
Chromosome1-2670597	INS	PRECISE	132	0	0	0/0	0/0	0/0	0/0	0/0	0/0	0/1	0/1	0/0	0/0	0/0	0/0	0/0	0/0
Chromosome1-2670645	DEL	PRECISE	363	0	0	0/1	1/1	0/1	1/1	1/1	1/1	1/1	1/1	1/1	1/1	1/1	1/1	1/1	1/1
Chromosome1-2670827	INS	PRECISE	1747	0	0	0/0	0/0	0/0	0/0	0/0	0/0	0/0	0/0	0/0	0/0	0/0	0/0	0/0	0/1
Chromosome1-2670899	DEL	PRECISE	276	0	0	0/1	0/1	0/0	0/0	0/0	0/0	0/0	0/0	0/1	1/1	0/1	1/1	0/1	0/1
Chromosome1-2671275	INS	PRECISE	1560	0	0	0/0	0/0	0/0	0/0	0/0	0/0	0/0	0/0	0/0	0/0	0/0	0/0	0/0	0/1
Chromosome1-2673535	DEL	PRECISE	701	0	0	0/0	0/0	0/0	0/0	0/0	0/0	0/0	0/0	0/1	0/1	0/1	0/1	0/1	0/1
Chromosome1-2674495	INS	PRECISE	105	0	0	0/0	0/0	0/0	0/0	0/0	0/0	0/0	0/0	0/0	0/1	0/0	0/0	0/0	0/0
Chromosome1-2674616	DEL	PRECISE	206	0	0	0/1	0/1	0/0	0/0	0/0	0/0	0/0	0/0	0/0	0/1	0/0	0/0	0/0	0/0
Chromosome1-2674736	INS	PRECISE	3252	0	0	0/0	0/0	0/0	0/0	0/0	0/0	0/0	0/0	0/0	0/0	0/0	1/1	0/0	0/0
Chromosome1-2675346	DEL	PRECISE	453	0	0	0/1	1/1	0/1	1/1	1/1	1/1	1/1	1/1	1/1	1/1	1/1	1/1	1/1	1/1
Chromosome1-2676100	DEL	PRECISE	148	0	0	0/0	0/0	0/0	0/0	0/0	0/0	0/0	0/0	0/1	0/1	0/1	1/1	0/1	0/1
Chromosome1-2676303	INS	PRECISE	812	0	0	0/0	0/0	0/0	0/0	0/0	0/0	0/0	0/0	0/0	0/0	0/0	0/1	0/0	0/0
Chromosome1-2676727	INS	PRECISE	55	0	0.5	0/0	0/0	0/0	0/0	0/0	0/0	0/0	0/0	0/1	0/0	0/1	0/0	0/1	0/1
Chromosome1-2676925	DEL	PRECISE	139	0	0	0/0	0/0	0/0	0/0	0/0	0/0	0/0	0/0	0/1	0/1	0/1	0/1	0/1	0/1
Chromosome1-2677771	DEL	PRECISE	265	0	0	0/0	0/0	0/0	1/1	0/1	0/1	0/1	0/1	0/0	0/0	0/0	0/0	0/0	0/0
Chromosome1-2678068	INV	PRECISE	15298	0	0.816	0/0	0/0	0/0	0/0	0/0	0/0	0/0	0/0	0/1	0/0	0/0	0/1	0/0	0/0
Chromosome1-2678189	INS	PRECISE	70	0	0	0/1	0/1	0/0	0/0	0/0	0/0	0/0	0/0	0/0	1/1	0/0	1/1	0/0	0/0
Chromosome1-2678547	DEL	PRECISE	107	0	0	0/0	0/0	0/0	0/0	0/0	0/0	0/0	0/0	0/0	0/0	0/0	0/0	0/0	1/1
Chromosome1-2678935	DEL	PRECISE	51	0	0	0/0	0/0	0/0	0/0	0/0	0/0	0/1	0/1	0/0	0/0	0/0	0/0	0/0	0/0
Chromosome1-2679544	INS	PRECISE	137	0	0	0/0	0/0	0/0	0/0	0/0	0/0	0/1	0/1	0/0	0/0	0/0	0/0	0/0	0/0
Chromosome1-2680764	DEL	PRECISE	52	0	0	0/0	0/0	0/0	0/0	0/0	0/0	0/0	0/0	0/0	0/0	0/0	1/1	0/0	0/0
Chromosome1-2681042	DEL	PRECISE	790	0	0	0/0	0/0	0/0	1/1	0/1	0/1	0/1	0/1	0/0	0/0	0/0	0/0	0/0	0/0
Chromosome1-2681083	DEL	PRECISE	153	0.816	0.408	0/0	0/0	0/0	0/0	0/0	0/0	0/0	0/0	0/1	0/0	0/1	0/1	0/1	0/1
Chromosome1-2681537	DEL	PRECISE	547	0.751	0.376	0/1	0/1	0/0	1/1	0/1	0/1	1/1	1/1	0/1	1/1	0/1	1/1	0/0	1/1
Chromosome1-2682249	BND	PRECISE	0	0	0	0/0	0/0	0/0	1/1	0/1	0/0	0/0	0/0	0/0	0/0	0/0	0/0	0/0	0/0
Chromosome1-2682250	BND	PRECISE	0	0	0	0/0	0/0	0/0	0/0	0/0	0/0	0/1	0/1	0/0	0/0	0/0	0/0	0/0	0/0
Chromosome1-2682259	INS	PRECISE	878	0	0	0/0	0/0	0/0	0/0	0/0	0/0	0/0	0/0	0/0	0/0	0/0	0/0	0/0	0/1
Chromosome1-2683464	DEL	PRECISE	799	0	0	0/0	0/0	0/0	0/0	0/0	0/0	0/0	0/0	1/1	0/0	1/1	0/0	0/1	1/1
Chromosome1-2684694	DEL	PRECISE	583	0	0	0/0	0/0	0/0	0/0	0/0	0/0	0/0	0/0	0/1	1/1	0/1	0/1	0/1	0/1

Chromosome1-2684825	DEL	PRECISE	366	0	0	0/1	1/1	0/1	1/1	1/1	1/1	1/1	1/1	1/1	1/1	1/1	1/1	1/1	1/1
Chromosome1-2685379	INS	PRECISE	871	0	0.577	0/1	0/1	0/0	0/0	0/0	0/0	0/0	0/0	0/0	0/1	0/0	1/1	0/0	0/0
Chromosome1-2685747	INS	PRECISE	731	0	0	0/0	0/0	0/0	0/0	0/0	0/0	0/0	0/0	0/0	0/0	0/0	0/1	0/0	0/0
Chromosome1-2686093	DEL	PRECISE	88	0	0	0/0	0/0	0/0	0/0	0/0	0/0	0/0	0/0	0/0	0/0	0/0	0/0	0/0	0/1
Chromosome1-2686606	DEL	PRECISE	53	0	0	0/0	0/0	0/0	0/0	0/0	0/0	0/0	0/0	0/1	0/1	0/1	0/1	0/1	0/1
Chromosome1-2687307	DEL	PRECISE	299	0	0	0/1	0/1	0/0	1/1	0/1	0/1	1/1	1/1	0/1	1/1	0/1	1/1	0/1	1/1
Chromosome1-2688184	INS	PRECISE	66	0	0.548	0/0	0/0	0/0	0/0	0/0	0/0	0/0	0/0	0/1	0/1	0/1	0/1	0/1	0/1
Chromosome1-2689749	INS	PRECISE	218	0	0.707	0/0	0/0	0/0	0/0	0/0	0/0	0/0	0/0	0/0	0/0	0/0	0/1	0/1	0/0
Chromosome1-2689844	INS	PRECISE	51	0	0.519	0/1	0/1	0/0	1/1	0/1	0/1	0/1	0/1	0/1	1/1	0/1	1/1	0/1	0/1
Chromosome1-2690015	INS	PRECISE	328	0	0	0/0	0/0	0/0	0/0	0/0	0/0	0/0	0/0	0/1	0/0	0/1	0/0	0/0	1/1
Chromosome1-2690103	DEL	PRECISE	56	0	0	0/0	0/0	0/0	0/0	0/0	0/0	0/0	0/0	0/0	0/0	0/0	0/1	0/1	0/0
Chromosome1-2690338	DEL	PRECISE	150	0	0	0/0	0/0	0/0	0/0	0/0	0/0	0/0	0/0	0/1	0/1	0/1	0/1	0/1	0/1
Chromosome1-2691206	DEL	PRECISE	59	0	0	0/0	0/0	0/0	0/0	0/0	0/0	0/0	0/0	0/1	0/1	0/1	0/1	0/1	0/1
Chromosome1-2691440	DEL	PRECISE	60	0	0	0/0	0/0	0/0	0/0	0/0	0/0	0/0	0/0	0/1	0/1	0/1	0/0	0/1	0/1
Chromosome1-2692592	DEL	PRECISE	286	0.548	0.408	0/0	0/0	0/0	0/0	0/0	0/0	0/0	0/0	0/1	0/1	0/1	0/1	0/1	0/1
Chromosome1-2692813	DEL	PRECISE	53	0	0	0/0	0/0	0/0	0/0	0/0	0/0	0/0	0/0	0/0	0/0	0/0	0/0	0/0	0/1
Chromosome1-2693855	DEL	PRECISE	104	0	0	0/0	0/0	0/0	0/0	0/0	0/0	0/0	0/0	0/0	0/0	0/0	0/0	0/0	0/1
Chromosome1-2694010	INS	PRECISE	230	0	0	0/0	0/0	0/0	0/1	0/0	0/0	0/0	0/0	0/0	0/0	0/0	0/0	0/0	0/0
Chromosome1-2694197	DEL	PRECISE	52	0	0	0/0	0/0	0/0	0/0	0/0	0/0	0/0	0/0	0/0	0/0	0/0	0/0	0/0	0/1
Chromosome1-2694286	DEL	PRECISE	420	0	0	0/0	0/0	0/0	0/0	0/0	0/0	0/0	0/0	0/1	0/0	0/1	0/1	0/1	0/1
Chromosome1-2698095	DEL	PRECISE	736	0	0	0/0	0/0	0/0	0/0	0/0	0/0	0/0	0/0	0/0	0/0	0/1	0/0	0/1	0/1
Chromosome1-2698361	DEL	PRECISE	125	0.408	0.408	0/0	0/0	0/0	0/0	0/0	0/0	0/0	0/0	0/0	0/0	0/1	0/0	0/1	0/1
Chromosome1-2698372	INS	PRECISE	138	0	0	0/0	0/0	0/0	0/0	0/0	0/0	0/0	0/0	0/0	0/0	0/0	0/0	0/0	0/1
Chromosome1-2698629	INS	PRECISE	265	0.707	0.707	0/0	0/0	0/0	0/1	0/0	0/0	0/0	0/0	0/0	0/0	0/0	0/0	0/0	1/1
Chromosome1-2699488	DEL	PRECISE	231	0.516	0.516	0/0	0/0	0/0	0/0	0/0	0/0	0/0	0/0	0/1	0/1	0/1	0/1	0/1	0/1
Chromosome1-2699586	DEL	PRECISE	594	0	0	0/0	0/0	0/0	0/0	0/0	0/0	0/0	0/0	0/1	0/1	0/1	0/1	0/1	0/1
Chromosome1-2700673	BND	PRECISE	0	0	0	0/0	0/0	0/0	0/0	0/0	0/0	0/0	0/0	0/0	0/0	0/1	0/1	0/0	0/1
Chromosome1-2701179	DEL	PRECISE	254	0	0	0/0	0/0	0/0	0/0	0/0	0/0	0/0	0/0	0/1	0/0	0/1	0/0	0/1	1/1
Chromosome1-2701885	DEL	PRECISE	70	0	0	0/0	0/0	0/0	0/1	0/0	0/0	0/0	0/0	0/0	0/0	0/0	0/0	0/0	0/0
Chromosome1-2701958	BND	PRECISE	0	0	0	0/0	0/0	0/0	0/0	0/0	0/0	0/0	0/0	0/0	0/0	0/0	0/0	0/0	0/1
Chromosome1-2701959	INS	PRECISE	5284	0	0	0/0	0/0	0/0	0/0	0/0	0/0	0/0	0/0	0/0	0/0	0/0	0/0	0/0	1/1
Chromosome1-2703100	DEL	PRECISE	1195	0	0	0/0	0/0	0/0	0/0	0/0	0/0	1/1	1/1	0/0	0/0	0/0	0/0	0/0	0/0
Chromosome1-2703250	DEL	PRECISE	103	0	0	0/0	0/0	0/0	0/0	0/0	0/0	0/0	0/0	0/0	0/0	0/0	0/0	0/0	1/1
Chromosome1-2704972	INS	PRECISE	67	0	0.277	0/1	0/1	0/0	0/1	0/1	0/1	0/1	0/1	0/1	1/1	0/1	1/1	0/1	0/1
Chromosome1-2704990	INS	PRECISE	1551	0	0.577	0/0	0/0	0/0	0/0	0/0	0/0	0/0	0/0	0/1	0/0	0/1	0/0	0/0	0/1
Chromosome1-2705217	INS	PRECISE	1279	0	0	0/0	0/0	0/0	0/0	0/0	0/0	0/0	0/0	0/0	0/1	0/0	0/0	0/0	0/0
Chromosome1-2705657	INS	PRECISE	132	0	0.354	0/0	0/1	0/1	0/1	0/1	0/1	0/0	0/0	0/1	0/0	0/1	0/0	0/0	0/0
Chromosome1-2706109	INS	PRECISE	211	0	0	0/0	0/0	0/0	0/0	0/0	0/0	0/0	0/0	0/0	0/1	0/0	0/0	0/0	0/0
Chromosome1-2706556	DEL	PRECISE	1202	0.277	0.277	0/1	0/1	0/0	0/1	0/1	0/1	0/1	0/1	0/1	1/1	0/1	1/1	0/1	1/1
Chromosome1-2707417	DEL	PRECISE	78	0	0	0/0	0/0	0/0	0/0	0/0	0/0	0/1	0/1	0/0	0/0	0/0	0/0	0/0	0/0
Chromosome1-2707749	DEL	PRECISE	331	0	0	0/0	0/1	0/1	0/1	0/1	0/1	0/0	0/0	1/1	0/0	1/1	0/0	1/1	0/0
Chromosome1-2708033	DEL	PRECISE	1027	0.577	0.577	0/0	0/0	0/0	0/0	0/0	0/0	0/0	0/0	0/0	0/1	0/0	0/1	1/1	0/0
Chromosome1-2708263	BND	PRECISE	0	0	0	0/0	0/0	0/0	0/0	0/0	0/0	0/0	0/0	0/1	0/0	0/1	0/0	0/0	0/1
Chromosome1-2710033	INS	PRECISE	8499	0	0	0/0	0/0	0/0	0/0	0/0	0/0	0/0	0/0	0/0	0/0	0/0	0/0	0/0	0/1
Chromosome1-2710532	DEL	PRECISE	345	0	0	0/0	0/0	0/0	0/0	0/0	0/0	0/0	0/0	0/1	0/0	0/1	0/1	0/1	0/1
Chromosome1-2711601	INS	PRECISE	1079	0	0	0/0	0/0	0/0	0/0	0/0	0/0	0/0	0/0	0/0	0/1	0/0	0/0	0/0	0/0
Chromosome1-2711997	INS	PRECISE	56	0	0	0/0	0/0	0/0	0/0	0/0	0/0	0/0	0/0	0/0	0/0	0/0	0/1	0/1	0/0
Chromosome1-2712370	INS	PRECISE	141	0	0	0/0	0/0	0/0	0/0	0/0	0/0	0/1	0/1	0/0	0/0	0/0	0/0	0/0	0/0
Chromosome1-2712371	BND	PRECISE	0	0	0	0/0	0/0	0/0	0/0	0/0	0/0	0/0	0/0	0/1	0/0	0/0	0/0	0/0	0/1
Chromosome1-2712470	INS	PRECISE	51	0	0	0/0	0/0	0/0	0/0	0/0	0/0	0/0	0/0	0/1	0/1	0/1	0/1	0/1	0/1
Chromosome1-2712746	INS	PRECISE	157	0	0	0/0	0/0	0/0	0/0	0/0	0/0	0/0	0/0	0/0	0/1	0/0	0/0	0/0	0/0
Chromosome1-2713234	INS	PRECISE	3758	0	0	0/0	0/0	0/0	0/0	0/0	0/0	0/0	0/0	0/0	0/1	0/0	0/0	0/0	0/0
Chromosome1-2713698	DEL	PRECISE	139	0	0	0/0	0/0	0/0	0/0	0/0	0/0	0/0	0/0	0/1	0/0	0/1	0/0	0/0	0/1
Chromosome1-2714086	INS	PRECISE	241	0	0	0/0	0/0	0/0	0/0	0/0	0/0	0/0	0/0	0/1	0/0	0/1	0/0	0/0	0/1
Chromosome1-2714770	DEL	PRECISE	1014	0	0	0/0	0/0	0/0	0/0	0/0	0/0	0/0	0/0	0/1	0/0	1/1	0/1	0/1	0/1
Chromosome1-2714896	DEL	PRECISE	876	0	0	0/1	0/1	0/0	0/1	0/1	0/0	1/1	1/1	0/1	1/1	1/1	1/1	0/0	1/1
Chromosome1-2715219	BND	PRECISE	0	0	0	0/0	0/0	0/0	0/0	0/0	0/0	0/1	1/1	0/0	0/0	0/0	0/0	0/0	0/0
Chromosome1-2715219	BND	PRECISE	0	0	0	0/0	0/0	0/0	0/0	0/0	0/0	0/1	0/1	0/0	0/0	0/0	0/0	0/0	0/0
Chromosome1-2715676	DEL	PRECISE	68	0.316	0.316	0/1	0/1	0/0	0/1	0/1	0/1	1/1	1/1	0/0	1/1	0/0	1/1	0/0	0/1
Chromosome1-2716144	DEL	PRECISE	85	0.316	0.316	0/1	0/1	0/0	0/1	0/1	0/1	1/1	1/1	0/0	1/1	0/0	1/1	0/0	0/1
Chromosome1-2716460	DEL	PRECISE	10558	0	0	0/0	0/0	0/0	0/0	0/0	0/0	0/0	0/0	0/1	0/0	0/0	0/0	0/0	0/0
Chromosome1-2716838	INS	PRECISE	66	0	0	0/0	0/0	0/0	0/0	0/0	0/0	0/1	0/1	0/0	0/0	0/0	0/0	0/0	0/0
Chromosome1-2716843	DEL	PRECISE	86	0	0	0/0	0/0	0/0	0/0	0/0	0/0	0/1	0/1	0/0	0/0	0/0	0/0	0/0	0/0
Chromosome1-2716974	INS	PRECISE	4003	0	0	0/0	0/0	0/0	0/0	0/0	0/0	0/0	0/0	0/0	0/1	0/0	0/0	0/0	0/0
Chromosome1-2717399	INS	PRECISE	5563	0	0	0/0	0/0	0/0	0/0	0/0	0/0	0/0	0/0	0/0	0/1	0/0	0/0	0/0	0/0
Chromosome1-2718629	DEL	PRECISE	873	0	0	0/0	0/0	0/0	0/0	0/0	0/0	0/0	0/0	0/0	0/0	0/0	0/0	0/0	1/1
Chromosome1-2718827	DEL	PRECISE	137	0	0	0/0	0/0	0/0	0/0	0/0	0/0	0/0	0/0	0/1	0/1	0/1	0/0	0/1	1/1
Chromosome1-2719631	DEL	PRECISE	3271	0	0	0/1	1/1	0/1	1/1	1/1	1/1	1/1	1/1	1/1	1/1	1/1	1/1	1/1	1/1
Chromosome1-2721979	INS	PRECISE	152	0	0	0/0	0/0	0/0	0/0	0/0	0/0	0/0	0/0	0/0	0/1	0/0	0/0	0/0	0/0
Chromosome1-2723953	DEL	PRECISE	163	0	0	0/0	0/0	0/0	0/0	0/0	0/0	0/0	0/0	0/0	0/1	0/0	0/1	0/1	0/0
Chromosome1-2724111	INS	PRECISE	322	0	0	0/0	0/0	0/0	0/0	0/0	0/0	0/0	0/0	0/0	0/1	0/0	0/0	0/0	0/0
Chromosome1-2725600	INS	PRECISE	51	0	0	0/1	1/1	0/1	1/1	1/1	1/1	1/1	1/1	1/1	1/1	1/1	1/1	1/1	1/1
Chromosome1-2725702	DEL	PRECISE	116	0	0	0/0	0/0	0/0	0/0	0/0	0/0	0/0	0/0	0/1	0/0	0/1	0/0	0/1	0/1

Chromosome1-2726546	DEL	PRECISE	91	0	0	0/0	0/0	0/0	0/0	0/0	0/0	0/0	0/0	0/1	0/0	0/1	0/0	0/1	0/1
Chromosome1-2728248	DEL	PRECISE	79	0	0	0/0	0/0	0/0	0/0	0/0	0/0	0/0	0/0	0/0	0/0	0/0	0/1	0/0	0/0
Chromosome1-2728506	DEL	PRECISE	88	0	0	0/0	0/0	0/0	0/0	0/0	0/0	0/0	0/0	1/1	0/1	0/0	0/0	0/1	0/1
Chromosome1-2729401	INS	PRECISE	1965	0	0	0/0	0/0	0/0	0/0	0/0	0/0	0/0	0/0	0/0	0/0	0/0	0/0	0/0	0/1
Chromosome1-2730199	DEL	PRECISE	154	0	0	0/0	0/0	0/0	0/0	0/0	0/0	0/0	0/0	0/1	0/0	0/0	0/1	0/0	0/0
Chromosome1-2731028	DEL	PRECISE	691	0	0	0/0	0/0	0/0	0/0	0/0	0/0	0/0	0/0	0/0	0/0	0/0	0/1	0/0	0/0
Chromosome1-2731469	INS	PRECISE	58	0	0	0/0	0/0	0/0	0/0	0/0	0/0	0/0	0/0	0/1	0/1	0/1	0/0	0/1	0/1
Chromosome1-2731582	INS	PRECISE	5841	0	0	0/0	0/0	0/0	0/0	0/1	0/0	0/0	0/0	0/0	0/0	0/0	0/0	0/0	0/0
Chromosome1-2732007	INS	PRECISE	1538	0	0	0/0	0/0	0/0	0/0	0/0	0/0	0/0	0/0	0/0	0/0	0/0	0/0	0/0	0/1
Chromosome1-2732082	INS	PRECISE	1113	0	0	0/0	0/0	0/0	0/0	0/0	0/0	0/0	0/0	0/0	0/0	0/0	0/1	0/0	0/0
Chromosome1-2733319	INS	PRECISE	211	0	0	0/0	0/0	0/0	0/0	0/0	0/0	0/0	0/0	0/0	0/0	0/0	0/0	0/0	0/1
Chromosome1-2733764	DEL	PRECISE	82	0	0	0/0	0/0	0/0	0/0	0/0	0/0	0/0	0/0	1/1	0/1	0/1	0/1	0/1	0/1
Chromosome1-2733836	INS	PRECISE	880	0	0	0/0	0/0	0/0	0/0	0/0	0/0	0/0	0/0	0/0	0/0	0/0	0/0	0/0	0/1
Chromosome1-2734526	INS	PRECISE	58	0	0.408	0/0	0/0	0/0	0/0	0/0	0/0	0/0	0/0	1/1	0/1	0/1	0/1	0/1	0/1
Chromosome1-2734943	DEL	PRECISE	66	0	0	0/0	0/0	0/0	0/0	0/0	0/0	0/0	0/0	0/0	0/0	0/0	0/0	0/0	0/1
Chromosome1-2735000	INS	PRECISE	217	0	0	0/0	0/0	0/0	0/0	0/0	0/0	0/0	0/0	0/0	0/1	0/0	0/0	0/0	0/0
Chromosome1-2735524	INS	PRECISE	7009	0	0	0/0	0/0	0/0	0/0	0/0	0/0	0/0	0/0	0/0	0/0	0/0	0/0	0/0	1/1
Chromosome1-2735550	DEL	PRECISE	1532	0	0	0/0	0/1	0/1	0/0	0/1	0/0	0/0	0/0	0/1	0/0	0/1	0/0	0/1	0/0
Chromosome1-2736691	DEL	PRECISE	444	0	0	0/1	0/1	0/0	1/1	0/1	0/1	1/1	1/1	0/1	1/1	0/1	1/1	0/1	1/1
Chromosome1-2736741	DEL	PRECISE	1856	0	0	0/0	0/0	0/0	0/0	0/0	0/0	0/0	0/0	0/1	0/1	0/1	0/0	0/1	0/1
Chromosome1-2736918	INS	PRECISE	567	0	0	0/0	0/0	0/0	0/0	0/0	0/0	0/0	0/0	0/0	0/0	0/0	0/0	0/0	0/1
Chromosome1-2736944	DEL	PRECISE	52	0	0	0/0	0/0	0/0	0/0	0/0	0/0	0/0	0/0	0/1	0/1	0/1	0/0	0/1	0/1
Chromosome1-2737097	INS	PRECISE	133	0	0	0/0	0/0	0/0	0/0	0/0	0/0	0/0	0/0	0/0	0/0	0/0	0/0	0/0	0/1
Chromosome1-2737472	INS	PRECISE	12023	0	0	0/0	0/0	0/0	0/0	0/0	0/0	0/0	0/0	0/0	0/0	0/0	0/0	0/0	0/1
Chromosome1-2737472	BND	PRECISE	0	0	0	0/0	0/0	0/0	0/0	0/0	0/0	0/0	0/0	0/0	0/0	0/0	0/0	0/0	0/1
Chromosome1-2738507	DEL	PRECISE	175	0	0	0/0	0/0	0/0	0/0	0/0	0/0	0/0	0/0	0/1	0/1	0/1	0/1	0/1	0/1
Chromosome1-2738768	DEL	PRECISE	223	0	0	0/0	0/0	0/0	0/0	0/0	0/0	0/0	0/0	0/1	0/0	0/1	0/1	0/1	0/1
Chromosome1-2738779	INS	PRECISE	130	0	0.333	0/1	0/1	0/0	1/1	0/1	0/1	0/1	0/1	0/0	1/1	0/0	0/1	0/0	0/0
Chromosome1-2739010	DEL	PRECISE	513	0	0	0/0	0/0	0/0	0/0	0/0	0/0	0/0	0/0	0/0	0/0	0/0	0/1	0/0	0/0
Chromosome1-2739115	DEL	PRECISE	357	0	0	0/0	0/0	0/0	0/0	0/0	0/0	0/0	0/0	0/0	0/0	0/0	0/1	0/0	0/0
Chromosome1-2739328	INS	PRECISE	169	0	0	0/0	0/0	0/0	0/0	0/0	0/0	0/0	0/0	0/0	0/0	0/0	0/1	0/0	0/0
Chromosome1-2739426	INS	PRECISE	66	0	0	0/0	0/0	0/0	0/0	0/0	0/0	0/0	0/0	0/1	0/0	0/1	0/0	0/1	0/1
Chromosome1-2739464	DEL	PRECISE	66	0	0	0/0	0/0	0/0	0/0	0/0	0/0	0/1	0/1	0/0	0/0	0/0	0/0	0/0	0/0
Chromosome1-2739923	INS	PRECISE	180	0	0	0/0	0/0	0/0	0/0	0/0	0/0	0/0	0/0	0/0	0/1	0/0	0/0	0/0	0/0
Chromosome1-2740451	INS	PRECISE	239	0	0	0/0	0/0	0/0	0/0	0/0	0/0	0/0	0/0	0/0	0/1	0/0	0/0	0/0	0/0
Chromosome1-2741658	INS	PRECISE	52	0	0	0/0	0/0	0/0	0/0	0/0	0/0	0/0	0/0	0/0	0/0	0/0	1/1	0/0	0/0
Chromosome1-2742259	DEL	PRECISE	86	0	0	0/0	0/0	0/0	0/0	0/0	0/0	0/0	0/0	0/0	0/1	0/0	0/1	0/0	0/0
Chromosome1-2742474	DEL	PRECISE	1167	0	0	0/0	0/0	0/0	0/0	0/0	0/0	0/0	0/0	0/0	0/0	0/0	0/1	0/0	0/0
Chromosome1-2742898	DEL	PRECISE	445	0	0	0/1	1/1	0/1	1/1	1/1	1/1	1/1	1/1	1/1	1/1	1/1	0/1	1/1	1/1
Chromosome1-2744738	INS	PRECISE	211	0	0.707	0/0	0/0	0/0	0/0	0/0	0/0	0/1	0/1	0/0	0/0	0/0	0/0	0/0	0/0
Chromosome1-2744862	INS	PRECISE	137	0	0	0/0	0/0	0/0	0/0	0/0	0/0	0/0	0/0	0/0	0/1	0/0	0/0	0/0	0/0
Chromosome1-2746052	INS	PRECISE	10776	0	0	0/0	0/0	0/0	0/0	0/0	0/0	0/1	0/0	0/0	0/0	0/0	0/0	0/0	0/0
Chromosome1-2746086	BND	PRECISE	0	0	0	0/0	0/0	0/0	0/0	0/0	0/0	0/1	0/1	0/0	0/0	0/0	0/0	0/0	0/0
Chromosome1-2746486	DEL	PRECISE	141	0	0	0/0	0/0	0/0	0/0	0/0	0/0	0/0	0/0	0/1	0/1	0/1	0/1	0/1	0/1
Chromosome1-2748557	BND	PRECISE	0	0	0	0/0	0/0	0/0	0/0	0/0	0/0	0/0	0/0	0/0	0/0	0/1	0/0	0/1	1/1
Chromosome1-2748641	INS	PRECISE	121	0	0	0/0	0/0	0/0	0/0	0/0	0/0	0/0	0/0	0/0	0/0	0/0	0/0	0/0	1/1
Chromosome1-2749275	DEL	PRECISE	117	0	0	0/0	0/0	0/0	0/0	0/0	0/0	0/0	0/0	0/1	0/1	0/1	0/1	0/1	0/1
Chromosome1-2749426	DEL	PRECISE	128	0	0	0/1	0/1	0/0	1/1	0/1	0/1	0/1	0/1	0/0	0/1	0/0	0/1	0/0	0/0
Chromosome1-2750274	INS	PRECISE	79	0	0	0/0	0/0	0/0	0/0	0/0	0/0	0/0	0/0	0/1	0/1	0/1	0/1	0/1	0/1
Chromosome1-2750510	DEL	PRECISE	1159	0	0	0/0	0/0	0/0	0/0	0/0	0/0	0/0	0/0	0/1	0/1	0/1	0/1	0/1	0/1
Chromosome1-2750643	INS	PRECISE	74	0	0	0/1	0/1	0/0	1/1	0/0	0/1	0/1	0/1	0/1	1/1	0/1	1/1	0/1	1/1
Chromosome1-2751427	DEL	PRECISE	57	0	0	0/0	0/0	0/0	0/0	0/0	0/0	0/1	0/1	0/0	0/0	0/0	0/0	0/0	0/1
Chromosome1-2751674	DEL	PRECISE	6464	0	0	0/0	0/0	0/0	0/0	0/0	0/0	0/0	0/0	0/1	0/1	0/1	0/1	0/0	0/1
Chromosome1-2753035	INS	PRECISE	141	0	0	0/0	0/0	0/0	0/0	0/0	0/0	0/0	0/0	0/0	0/0	0/0	0/1	0/0	0/0
Chromosome1-2755547	INS	PRECISE	59	0	0	0/1	0/1	0/0	1/1	0/1	0/1	0/1	0/1	0/0	0/1	0/0	0/1	0/0	0/1
Chromosome1-2756203	INS	PRECISE	582	0	0.527	0/1	0/1	0/0	1/1	0/1	0/1	0/1	0/1	0/0	0/1	0/0	0/0	0/0	0/0
Chromosome1-2756204	DEL	PRECISE	1055	0	0	0/0	0/0	0/0	0/0	0/0	0/0	0/0	0/0	0/0	0/1	0/0	1/1	0/1	0/0
Chromosome1-2756654	INS	PRECISE	63	0	0.577	0/0	0/0	0/0	0/0	0/0	0/0	0/0	0/0	0/1	0/0	0/1	0/0	0/0	0/1
Chromosome1-2757189	BND	PRECISE	0	0	0	0/0	0/0	0/0	0/0	0/0	0/0	0/0	0/0	0/0	0/1	0/0	0/0	0/0	0/0
Chromosome1-2757255	INS	PRECISE	63	0	0	0/1	0/1	0/0	1/1	0/1	0/1	1/1	1/1	0/0	1/1	0/0	1/1	0/0	1/1
Chromosome1-2757636	BND	PRECISE	0	0	0	0/0	0/0	0/0	0/1	0/0	0/0	0/0	0/0	0/0	0/0	0/0	0/0	0/0	0/0
Chromosome1-2758350	BND	PRECISE	0	0	0	0/0	0/1	0/0	0/0	0/1	0/1	0/0	0/0	0/1	0/0	0/1	0/0	0/1	0/0
Chromosome1-2758501	DEL	PRECISE	150	0	0	0/1	1/1	0/1	1/1	1/1	1/1	1/1	1/1	1/1	1/1	1/1	1/1	1/1	1/1
Chromosome1-2758744	DEL	PRECISE	50	0	0	0/0	0/0	0/0	0/0	0/0	0/0	0/1	0/1	0/0	0/0	0/0	0/0	0/0	0/0
Chromosome1-2759121	DEL	PRECISE	65	0	0	0/1	0/1	0/0	1/1	0/1	0/1	0/1	0/1	0/1	1/1	0/1	1/1	0/1	1/1
Chromosome1-2759389	DUP	PRECISE	20363	0	0	0/0	0/0	0/0	0/0	0/0	0/0	0/0	0/0	0/1	0/1	0/0	0/0	0/1	0/1
Chromosome1-2759610	INS	PRECISE	334	0	0	0/0	0/0	0/0	0/0	0/0	0/0	0/0	0/0	0/0	0/0	0/0	0/1	0/0	0/0
Chromosome1-2760919	INS	PRECISE	56	0	0.408	0/0	0/0	0/0	0/0	0/0	0/0	0/0	0/0	0/1	0/1	0/0	0/1	0/1	0/1
Chromosome1-2761003	INS	PRECISE	741	0	0	0/0	0/0	0/0	0/0	0/0	0/0	0/0	0/0	0/0	0/0	0/0	0/1	0/0	0/0
Chromosome1-2761305	INS	PRECISE	50	0	0	0/0	0/0	0/0	0/0	0/0	0/0	0/0	0/0	0/0	0/0	0/0	0/1	0/0	0/0
Chromosome1-2761458	INS	PRECISE	2241	0	0	0/0	0/0	0/0	0/0	0/0	0/0	0/0	0/0	0/0	0/0	0/0	0/1	0/0	0/0
Chromosome1-2762039	DEL	PRECISE	506	0	0	0/1	0/1	0/0	1/1	0/1	0/1	0/1	0/1	0/1	1/1	0/1	1/1	0/1	0/1
Chromosome1-2762938	INS	PRECISE	272	0	0	0/0	0/0	0/0	0/0	0/0	0/0	0/0	0/0	0/0	0/0	0/0	0/1	0/0	0/0

Chromosome1-2762969	INS	PRECISE	984	0	0	0/0	0/0	0/0	0/0	0/0	0/0	0/1	0/1	0/0	0/0	0/0	0/0	0/0	0/0
Chromosome1-2763454	INS	PRECISE	65	0	0	0/1	0/1	0/0	1/1	0/1	0/1	0/1	0/1	0/0	1/1	0/0	0/1	0/0	0/0
Chromosome1-2763844	DEL	PRECISE	470	0	0	0/0	0/0	0/0	0/0	0/0	0/0	1/1	0/1	0/0	0/0	0/0	0/0	0/0	0/0
Chromosome1-2764121	INS	PRECISE	662	0	0.866	0/1	0/1	0/0	1/1	0/1	0/1	0/0	0/1	0/0	0/1	0/0	0/1	0/0	0/0
Chromosome1-2764316	INS	PRECISE	5262	0	0	0/0	0/0	0/0	0/0	0/1	0/0	0/0	0/0	0/0	0/0	0/0	0/0	0/0	0/0
Chromosome1-2764726	INS	PRECISE	888	0	0.707	0/0	0/0	0/0	0/0	0/0	0/0	0/0	0/0	0/0	0/1	0/0	0/0	0/1	0/0
Chromosome1-2764994	INS	PRECISE	91	0	0.408	0/0	0/0	0/0	0/0	0/0	0/0	0/0	0/0	0/1	0/1	0/1	0/1	0/1	0/1
Chromosome1-2765313	INS	PRECISE	150	0	0.577	0/0	0/0	0/0	0/0	0/0	0/0	0/0	0/0	0/1	0/0	0/1	0/0	0/0	0/1
Chromosome1-2766266	DEL	PRECISE	400	0	0	0/0	0/0	0/0	0/0	0/0	0/0	0/0	0/0	0/0	0/0	0/1	0/0	0/0	0/1
Chromosome1-2766974	INS	PRECISE	352	0	0	0/0	0/0	0/0	0/0	0/0	0/0	1/1	1/1	0/0	0/0	0/0	0/0	0/0	0/0
Chromosome1-2767052	INS	PRECISE	621	0	0.577	0/0	0/0	0/0	0/0	0/0	0/0	0/0	0/0	0/1	0/1	0/1	0/0	0/0	0/1
Chromosome1-2767110	INS	PRECISE	133	0	0	0/0	0/0	0/0	0/0	0/0	0/0	0/0	0/0	0/1	0/0	0/1	0/0	0/0	0/1
Chromosome1-2767599	INS	PRECISE	6789	0	0	0/0	0/0	0/0	0/0	0/0	0/0	0/0	0/0	0/0	0/0	0/0	0/0	0/0	1/1
Chromosome1-2768264	INS	PRECISE	161	0	0	0/0	0/0	0/0	0/0	0/0	0/0	0/0	0/0	0/1	0/1	0/1	0/0	0/0	0/1
Chromosome1-2768911	DEL	PRECISE	886	0	0	0/0	0/0	0/0	0/0	0/0	0/0	0/0	0/0	0/0	1/1	0/0	0/0	1/1	1/1
Chromosome1-2769329	DEL	PRECISE	318	0	0	0/0	0/0	0/0	0/0	0/0	0/0	0/0	0/0	0/0	0/0	0/0	0/0	0/1	0/0
Chromosome1-2770367	DEL	PRECISE	757	0	0	0/0	0/0	0/0	0/0	0/0	0/0	0/0	0/0	0/0	0/0	0/0	0/0	0/1	0/0
Chromosome1-2770696	INS	PRECISE	123	0	0.957	0/0	0/0	0/0	0/0	0/0	0/0	0/0	0/0	0/1	0/1	0/1	0/0	0/0	0/1
Chromosome1-2770870	INS	PRECISE	89	0.289	0.289	0/1	0/1	0/0	1/1	0/0	0/1	0/1	0/1	0/1	1/1	0/1	1/1	0/1	1/1
Chromosome1-2771546	INS	PRECISE	128	0	0	0/0	0/0	0/0	0/0	0/0	0/0	0/0	0/0	0/0	0/0	0/0	0/1	0/0	0/0
Chromosome1-2772380	INS	PRECISE	178	0	0.488	0/0	0/1	0/1	0/0	1/1	1/1	0/0	0/0	0/1	0/0	0/1	0/0	0/1	0/0
Chromosome1-2773781	INS	PRECISE	168	0	0.378	0/0	0/1	0/1	0/0	1/1	1/1	0/0	0/0	0/1	0/0	0/1	0/0	0/1	0/0
Chromosome1-2774925	DEL	PRECISE	631	0	0	0/0	0/0	0/0	0/0	0/0	0/0	0/0	0/0	0/1	0/1	0/1	0/1	0/1	0/1
Chromosome1-2775138	BND	PRECISE	0	0	0	0/0	0/0	0/0	0/0	0/0	0/0	0/0	0/0	0/1	0/0	0/1	0/1	0/1	0/0
Chromosome1-2775298	INS	PRECISE	108	0	0.516	0/0	0/0	0/0	0/0	0/0	0/0	0/0	0/0	0/1	0/1	0/1	0/1	0/0	0/1
Chromosome1-2775890	INS	PRECISE	229	0	0.983	0/0	0/0	0/0	0/0	0/0	0/0	0/0	0/0	0/1	0/1	0/1	0/1	0/1	0/1
Chromosome1-2776383	INS	PRECISE	773	0	0	0/0	0/0	0/0	0/0	0/0	0/0	0/0	0/0	0/0	0/0	0/0	1/1	0/0	0/0
Chromosome1-2776646	INS	PRECISE	886	0	0	0/0	0/0	0/0	0/0	0/0	0/0	0/0	0/0	0/0	0/1	0/0	0/0	0/0	0/0
Chromosome1-2776861	INS	PRECISE	54	0	0	0/0	0/0	0/0	0/0	0/0	0/0	0/0	0/0	0/0	0/0	0/0	0/0	0/1	0/0
Chromosome1-2777102	DEL	PRECISE	56	0	0	0/0	0/0	0/0	0/0	0/0	0/0	0/1	0/0	0/0	0/0	0/0	0/0	0/0	0/0
Chromosome1-2779211	DEL	PRECISE	731	0	0	0/1	0/1	0/0	1/1	0/0	0/1	0/1	0/1	0/1	1/1	0/1	1/1	0/1	0/1
Chromosome1-2779463	DEL	PRECISE	61	0	0	0/0	0/0	0/0	0/0	0/0	0/0	0/0	0/0	0/1	0/1	0/1	0/1	0/1	0/1
Chromosome1-2780273	BND	PRECISE	0	0	0	0/0	0/0	0/0	0/0	0/0	0/0	0/0	0/0	0/1	0/1	0/1	0/1	0/1	0/0
Chromosome1-2780385	DEL	PRECISE	597	0	0	0/1	0/1	0/0	1/1	0/0	0/1	0/1	0/1	0/0	1/1	0/0	1/1	0/0	0/0
Chromosome1-2780500	BND	PRECISE	0	0.577	0	0/0	0/0	0/0	0/0	0/0	0/0	0/0	0/0	0/1	0/0	0/1	0/0	0/1	0/0
Chromosome1-2781347	DEL	PRECISE	134	0	0	0/0	0/0	0/0	0/0	0/0	0/0	0/0	0/0	0/1	0/0	0/1	0/0	0/0	0/1
Chromosome1-2782299	INS	PRECISE	655	0	0	0/0	0/0	0/0	0/0	0/0	0/0	0/0	0/0	0/0	0/0	0/0	0/0	0/0	0/1
Chromosome1-2782539	DEL	PRECISE	167	0	0	0/0	0/0	0/0	0/0	0/0	0/0	0/0	0/0	0/1	0/1	0/1	0/1	0/0	0/1
Chromosome1-2782637	INS	PRECISE	234	0	0	0/0	0/0	0/0	0/0	0/0	0/0	0/0	0/0	0/0	0/0	0/0	0/0	0/0	0/1
Chromosome1-2782763	INS	PRECISE	1300	0	0	0/0	0/0	0/0	0/0	0/0	0/0	0/0	0/0	0/0	0/1	0/0	0/0	0/0	0/0
Chromosome1-2783095	DEL	PRECISE	222	0	0	0/0	0/0	0/0	0/0	0/0	0/0	0/0	0/0	0/1	0/0	0/1	0/0	0/0	0/1
Chromosome1-2784140	DEL	PRECISE	106	0	0	0/1	0/1	0/0	1/1	0/0	0/1	0/1	0/1	0/0	1/1	0/0	1/1	0/0	0/0
Chromosome1-2784192	INS	PRECISE	122	0	0.816	0/0	0/0	0/0	0/0	0/0	0/0	0/0	0/0	0/1	0/1	0/1	0/1	0/1	0/1
Chromosome1-2784864	INS	PRECISE	1001	0	0	0/0	0/0	0/0	0/0	0/0	0/0	0/0	0/0	0/0	0/0	0/0	0/0	0/0	0/1
Chromosome1-2785557	INS	PRECISE	5625	0	0	0/0	0/0	0/0	0/0	0/0	0/0	0/0	0/0	0/0	0/0	0/0	0/0	0/0	1/1
Chromosome1-2785558	BND	PRECISE	0	0	0	0/0	0/0	0/0	0/0	0/0	0/0	0/0	0/0	0/0	0/0	0/0	0/0	0/0	0/1
Chromosome1-2785717	INS	PRECISE	1008	0	0.707	0/0	0/0	0/0	0/0	0/0	0/0	0/1	0/1	0/0	0/0	0/0	0/0	0/0	0/0
Chromosome1-2786189	DEL	PRECISE	165	0	0	0/0	0/0	0/0	0/0	0/0	0/0	0/0	0/0	0/1	0/1	0/1	0/1	0/1	0/1
Chromosome1-2786464	DEL	PRECISE	56	0	0	0/0	0/0	0/0	0/0	0/0	0/0	0/1	0/1	0/0	0/0	0/0	0/0	0/0	0/0
Chromosome1-2788123	BND	PRECISE	0	0	0	0/0	0/0	0/0	0/0	0/0	0/0	0/0	0/0	0/0	0/0	0/0	0/1	0/1	0/0
Chromosome1-2788231	INS	PRECISE	56	0	0	0/1	0/1	0/0	1/1	0/0	0/1	1/1	1/1	0/1	1/1	0/0	1/1	0/1	1/1
Chromosome1-2788328	INS	PRECISE	627	0	0.707	0/1	0/1	0/0	1/1	0/0	0/1	1/1	1/1	0/0	1/1	0/0	1/1	0/0	0/0
Chromosome1-2788358	DEL	PRECISE	74	0	0	0/0	0/0	0/0	0/0	0/0	0/0	0/0	0/0	0/1	0/0	0/0	0/0	0/0	0/1
Chromosome1-2788696	DEL	PRECISE	63	0	0	0/0	0/0	0/0	0/0	0/0	0/0	0/0	0/0	0/1	0/1	0/1	0/0	0/0	0/1
Chromosome1-2789734	INS	PRECISE	625	0	0.926	0/1	0/1	0/0	1/1	0/0	0/1	1/1	1/1	0/0	1/1	0/0	1/1	0/0	0/0
Chromosome1-2790531	INS	PRECISE	52	0	0	0/0	0/0	0/0	0/0	0/0	0/0	0/0	0/0	0/1	0/1	1/1	0/1	0/1	0/1
Chromosome1-2790957	INS	PRECISE	3556	0	0	0/0	0/0	0/0	0/0	0/0	0/0	0/0	0/0	0/0	0/0	0/0	0/0	0/0	1/1
Chromosome1-2791271	DEL	PRECISE	73	0	0	0/0	0/0	0/0	0/0	0/0	0/0	0/0	0/0	0/0	0/0	0/1	0/0	0/0	0/1
Chromosome1-2791610	DEL	PRECISE	68	0	0	0/0	0/0	0/0	0/0	0/0	0/0	0/0	0/0	0/0	0/0	0/1	0/0	0/0	0/1
Chromosome1-2792275	DEL	PRECISE	117	0	0	0/0	0/0	0/0	0/0	0/0	0/0	0/0	0/0	0/0	0/0	0/0	0/1	0/1	0/0
Chromosome1-2794512	DEL	PRECISE	201	0	0	0/0	0/0	0/0	0/0	0/0	0/0	0/0	0/0	0/1	0/1	0/1	0/1	0/1	0/1
Chromosome1-2794591	INS	PRECISE	1178	0	0.707	0/0	0/0	0/0	0/0	0/0	0/0	0/0	0/0	0/0	0/0	0/0	0/1	0/1	0/0
Chromosome1-2796295	DEL	PRECISE	95	0	0	0/0	0/1	0/1	0/0	1/1	0/1	0/0	0/0	0/1	0/0	0/1	0/0	0/1	0/0
Chromosome1-2798173	INS	PRECISE	54	0	0	0/0	0/0	0/0	0/0	0/0	0/0	0/0	0/0	0/1	0/1	0/1	0/1	0/1	0/1
Chromosome1-2798522	INS	PRECISE	55	0	0.408	0/0	0/0	0/0	0/0	0/0	0/0	0/0	0/0	0/1	0/1	0/1	0/1	0/1	0/1
Chromosome1-2799169	DEL	PRECISE	171	0	0	0/0	0/0	0/0	0/0	0/0	0/0	0/0	0/0	0/0	0/0	0/0	0/0	0/0	0/1
Chromosome1-2799214	DEL	PRECISE	743	0	0	0/0	0/0	0/0	0/0	0/0	0/0	0/0	0/0	0/0	0/0	0/0	0/0	0/0	0/1
Chromosome1-2799300	DEL	PRECISE	112	0	0	0/0	0/0	0/0	0/0	0/0	0/0	0/0	0/0	0/0	0/1	0/0	0/0	0/0	0/0
Chromosome1-2800673	INS	PRECISE	466	0	0	0/0	0/0	0/0	0/0	0/0	0/0	0/0	0/0	0/0	0/0	0/0	0/1	0/1	0/0
Chromosome1-2800725	DEL	PRECISE	106	0	0	0/0	0/0	0/0	0/0	0/0	0/0	0/0	0/0	0/1	0/0	0/1	0/0	0/0	0/1
Chromosome1-2800833	INS	PRECISE	990	0	0.707	0/0	0/0	0/0	0/0	0/0	0/0	0/0	0/0	0/0	0/0	0/0	0/1	0/1	0/0
Chromosome1-2800869	DEL	PRECISE	75	0	0	0/0	0/0	0/0	0/0	0/0	0/0	0/0	0/0	0/0	0/0	0/0	0/0	0/0	0/1
Chromosome1-2800942	INS	PRECISE	10750	0	0	0/0	0/0	0/0	0/0	0/1	0/0	0/0	0/0	0/0	0/0	0/0	0/0	0/0	0/0

Chromosome1-2803788	INS	PRECISE	1026	0	0	0/0	0/0	0/0	0/0	0/0	0/0	0/0	0/0	0/0	0/0	0/0	0/0	0/0	0/1
Chromosome1-2804089	INS	PRECISE	1140	0	0.577	0/0	0/0	0/0	0/0	0/0	0/0	0/0	0/0	0/1	0/0	0/1	0/0	0/0	0/1
Chromosome1-2805113	INS	PRECISE	1495	0	0	0/0	0/0	0/0	0/0	0/0	0/0	0/0	0/0	0/0	0/0	0/0	0/0	0/0	1/1
Chromosome1-2805353	DEL	PRECISE	133	0.835	0.518	0/1	0/1	0/0	0/0	0/0	0/0	0/0	0/0	0/1	1/1	0/1	1/1	0/1	1/1
Chromosome1-2805527	INS	PRECISE	56	0	0	0/0	0/0	0/0	0/0	0/0	0/0	0/0	0/0	0/0	0/0	0/0	0/1	0/0	0/0
Chromosome1-2806055	INS	PRECISE	108	0	0.548	0/0	0/0	0/0	0/0	0/0	0/0	0/0	0/0	0/1	0/1	0/1	0/1	0/1	0/1
Chromosome1-2806194	INS	PRECISE	193	0	0.753	0/0	0/0	0/0	0/0	0/0	0/0	0/0	0/0	0/1	0/1	0/1	0/1	0/1	0/1
Chromosome1-2806397	DEL	PRECISE	82	0	0	0/0	0/0	0/0	0/0	0/0	0/0	0/0	0/0	0/1	1/1	0/1	0/1	0/1	0/1
Chromosome1-2806604	INS	PRECISE	829	0	0	0/0	0/0	0/0	0/0	0/0	0/0	0/0	0/0	0/0	0/0	0/0	0/0	0/0	0/1
Chromosome1-2807901	DEL	PRECISE	388	0	0	0/0	0/0	0/0	0/0	0/0	0/0	0/0	0/0	0/1	0/1	0/1	0/0	0/0	0/1
Chromosome1-2810764	DEL	PRECISE	302	0	0	0/0	0/0	0/0	0/0	0/0	0/0	0/0	0/0	0/0	0/1	0/0	0/0	0/0	0/0
Chromosome1-2811230	DEL	PRECISE	57	0	0	0/0	0/0	0/0	0/0	0/0	0/0	0/0	0/0	0/0	0/0	0/0	0/0	0/0	0/1
Chromosome1-2812109	DEL	PRECISE	54	0	0	0/0	0/0	0/0	0/0	0/0	0/0	0/0	0/0	0/0	0/1	0/0	0/0	0/0	0/0
Chromosome1-2814216	DEL	PRECISE	76	0	0	0/1	0/1	0/0	0/0	0/0	0/0	0/0	0/0	0/0	0/0	0/0	0/0	0/0	0/0
Chromosome1-2814273	INS	PRECISE	70	0	0	0/0	0/0	0/0	0/0	0/0	0/0	0/0	0/0	0/0	0/0	0/0	0/0	0/0	0/1
Chromosome1-2815724	DEL	PRECISE	123	0	0	0/0	0/0	0/0	0/0	0/0	0/0	0/0	0/0	0/0	0/0	0/0	1/1	1/1	0/0
Chromosome1-2816089	DEL	PRECISE	69	0	0	0/0	0/0	0/0	0/0	0/0	0/0	0/0	0/0	0/1	0/1	0/1	1/1	1/1	0/1
Chromosome1-2816168	INS	PRECISE	65	0	0	0/0	0/0	0/0	0/0	0/0	0/0	0/0	0/0	0/1	0/1	0/1	1/1	1/1	0/1
Chromosome1-2816558	DEL	PRECISE	428	0.816	0.816	0/0	0/0	0/0	0/0	0/0	0/0	0/0	0/0	0/1	0/1	0/1	0/1	0/1	0/1
Chromosome1-2816634	DEL	PRECISE	52	0	0	0/0	0/0	0/0	0/0	0/0	0/0	0/0	0/0	0/0	0/0	0/0	0/1	0/1	0/0
Chromosome1-2816795	INS	PRECISE	652	0	0	0/0	0/0	0/0	0/0	0/0	0/0	0/0	0/0	0/0	0/0	0/0	0/0	0/0	0/1
Chromosome1-2816874	INS	PRECISE	970	0	0	0/1	0/1	0/0	0/0	0/0	0/0	0/0	0/0	0/0	0/0	0/0	0/0	0/0	0/0
Chromosome1-2816908	DEL	PRECISE	127	0	0	0/0	0/0	0/0	0/0	0/0	0/0	0/0	0/0	0/0	0/1	0/0	0/1	0/1	0/0
Chromosome1-2817052	INS	PRECISE	662	0	0.983	0/0	0/0	0/0	0/0	0/0	0/0	0/0	0/0	0/1	1/1	1/1	1/1	1/1	1/1
Chromosome1-2818258	INS	PRECISE	484	0	0	0/0	0/0	0/0	0/0	0/0	0/0	0/0	0/0	0/0	0/0	0/0	0/0	0/0	0/1
Chromosome1-2819047	BND	PRECISE	0	0	0	0/0	0/0	0/0	0/0	0/0	0/0	0/1	0/0	0/0	0/0	0/0	0/0	0/0	0/0
Chromosome1-2819394	DEL	PRECISE	1268	0	0	0/0	0/0	0/0	0/0	0/0	0/0	0/0	0/0	0/0	0/0	0/0	0/1	0/1	0/0
Chromosome1-2819465	INS	PRECISE	75	0	0	0/1	0/1	0/0	0/0	0/0	0/0	0/0	0/0	0/0	0/0	0/0	0/0	0/0	0/0
Chromosome1-2819536	BND	PRECISE	0	0	0	0/0	0/0	0/0	0/0	0/0	0/0	0/0	0/0	0/0	0/1	0/0	0/0	0/0	0/0
Chromosome1-2820072	DEL	PRECISE	6743	0	0	0/0	0/0	0/0	0/0	0/0	0/0	0/0	0/0	0/0	0/0	0/0	0/0	0/0	0/1
Chromosome1-2820120	DEL	PRECISE	2661	0	0	0/0	0/0	0/0	0/0	0/0	0/0	0/0	0/0	0/0	0/1	0/0	0/1	0/0	0/0
Chromosome1-2820616	DEL	PRECISE	1300	0	0	0/0	0/0	0/0	0/0	0/0	0/0	0/0	0/0	0/1	0/1	0/1	0/1	0/1	0/1
Chromosome1-2821035	INS	PRECISE	113	0	0.408	0/0	0/0	0/0	0/0	0/0	0/0	0/0	0/0	0/1	0/1	0/1	0/1	0/1	1/1
Chromosome1-2822312	INS	PRECISE	997	0	0.816	0/0	0/0	0/0	0/0	0/0	0/0	0/0	0/0	0/1	0/1	0/1	0/1	0/1	0/1
Chromosome1-2823500	INS	PRECISE	87	0	0.408	0/0	0/0	0/0	0/0	0/0	0/0	0/0	0/0	0/1	0/1	0/1	0/1	0/1	0/1
Chromosome1-2823778	DEL	PRECISE	91	0	0	0/0	0/0	0/0	0/0	0/0	0/0	0/0	0/0	0/1	0/1	0/1	0/1	0/1	0/1
Chromosome1-2823921	INS	PRECISE	170	0	0	0/1	0/1	0/0	0/0	0/0	0/0	0/0	0/0	0/0	0/0	0/0	0/0	0/0	0/0
Chromosome1-2824337	DEL	PRECISE	926	0	0	0/0	0/0	0/0	0/0	0/0	0/0	0/0	0/0	0/1	1/1	0/1	1/1	1/1	1/1
Chromosome1-2824610	INS	PRECISE	53	0	0.408	0/0	0/0	0/0	0/0	0/0	0/0	0/0	0/0	0/1	0/1	0/1	0/1	0/1	0/1
Chromosome1-2824718	INS	PRECISE	887	0	0	0/1	0/1	0/0	0/0	0/0	0/0	0/0	0/0	0/0	0/0	0/0	0/0	0/0	0/0
Chromosome1-2824791	DEL	PRECISE	69	0	0	0/0	0/0	0/0	0/0	0/0	0/0	0/0	0/0	0/0	0/1	0/0	0/0	0/0	0/0
Chromosome1-2824845	INS	PRECISE	155	0	0.408	0/0	0/0	0/0	0/0	0/0	0/0	0/0	0/0	0/1	0/1	0/1	0/1	0/1	0/1
Chromosome1-2825120	INS	PRECISE	966	0	0	0/0	0/0	0/0	0/0	0/0	0/0	0/0	0/0	0/0	0/1	0/0	0/0	0/0	0/0
Chromosome1-2825740	BND	PRECISE	0	0	0	0/0	0/0	0/0	0/0	0/0	0/0	0/0	0/0	0/0	0/0	0/0	0/0	0/1	0/0
Chromosome1-2825741	INS	PRECISE	6050	0	0	0/0	0/0	0/0	0/0	0/0	0/0	0/0	0/0	0/0	1/1	0/0	0/0	0/0	0/0
Chromosome1-2825776	INS	PRECISE	119	0	0	0/0	0/1	0/1	1/1	1/1	1/1	0/1	0/1	0/1	0/1	0/1	1/1	0/1	0/0
Chromosome1-2826322	DEL	PRECISE	1188	0	0	0/0	0/0	0/0	0/0	0/0	0/0	0/0	0/0	0/1	0/0	0/1	0/1	0/1	0/1
Chromosome1-2826784	INS	PRECISE	454	0	0.289	0/0	0/1	0/1	1/1	1/1	1/1	0/1	0/1	0/1	0/1	0/1	0/1	0/1	0/0
Chromosome1-2826896	DEL	PRECISE	5791	0	0	0/1	1/1	0/1	1/1	1/1	1/1	1/1	1/1	1/1	1/1	1/1	1/1	1/1	1/1
Chromosome1-2827801	DEL	PRECISE	63	0	0	0/0	0/0	0/0	0/0	0/0	0/0	0/0	0/0	0/1	0/1	0/1	0/0	0/0	0/1
Chromosome1-2828391	DEL	PRECISE	98	0	0	0/0	0/0	0/0	0/0	0/0	0/0	0/0	0/0	0/1	0/1	1/1	0/1	0/1	0/1
Chromosome1-2828645	INS	PRECISE	56	0	0.707	0/0	0/0	0/0	0/0	0/0	0/0	0/0	0/0	0/0	0/0	0/0	1/1	1/1	0/0
Chromosome1-2830445	DEL	PRECISE	1028	0	0	0/1	1/1	0/1	1/1	1/1	1/1	1/1	1/1	1/1	1/1	1/1	1/1	1/1	1/1
Chromosome1-2834126	INS	PRECISE	63	0	0.408	0/0	0/0	0/0	0/0	0/0	0/0	0/0	0/0	0/1	0/1	0/1	0/1	0/0	0/1
Chromosome1-2835663	DEL	PRECISE	180	0	0	0/0	0/0	0/0	0/0	0/0	0/0	0/0	0/0	0/1	0/1	0/1	0/1	0/1	0/1
Chromosome1-2836892	DEL	PRECISE	53	0	0	0/0	0/0	0/0	0/0	0/0	0/0	0/0	0/0	0/1	0/1	0/1	0/0	0/1	0/1
Chromosome1-2837200	INS	PRECISE	74	0	0	0/1	1/1	0/1	1/1	1/1	1/1	0/1	0/1	0/1	0/1	0/1	1/1	1/1	0/1
Chromosome1-2838049	DEL	PRECISE	1362	0	0	0/0	0/0	0/0	0/0	0/0	0/0	0/0	0/0	0/0	0/1	0/0	0/0	0/0	0/0
Chromosome1-2838073	INS	PRECISE	667	0	0	0/0	0/0	0/0	0/0	0/0	0/0	0/0	0/0	0/0	0/0	0/0	0/0	0/1	0/0
Chromosome1-2838190	INS	PRECISE	858	0	0.996	0/0	0/1	0/1	1/1	1/1	1/1	0/1	0/1	0/1	0/1	0/1	1/1	0/1	0/0
Chromosome1-2838251	DEL	PRECISE	107	0	0	0/0	0/0	0/0	0/0	0/0	0/0	0/0	0/0	0/0	0/0	0/0	0/0	0/1	0/0
Chromosome1-2838745	INS	PRECISE	54	0	0	0/0	0/0	0/0	0/0	0/0	0/0	0/0	0/0	0/0	0/1	0/0	0/0	0/0	0/0
Chromosome1-2839103	INS	PRECISE	322	0	0	0/0	0/0	0/0	0/0	0/0	0/0	0/0	0/0	0/0	0/1	0/0	0/0	0/0	0/0
Chromosome1-2839184	INS	PRECISE	602	0	0	0/0	0/0	0/0	0/0	0/0	0/0	0/0	0/0	0/0	0/1	0/0	0/0	0/0	0/0
Chromosome1-2839887	DEL	PRECISE	54	0	0	0/0	0/0	0/0	0/0	0/0	0/0	0/0	0/0	0/0	0/0	0/0	0/0	0/0	0/1
Chromosome1-2840150	INS	PRECISE	124	0	0	0/0	0/0	0/0	0/0	0/0	0/0	0/0	0/0	0/1	0/0	0/1	0/0	0/0	0/0
Chromosome1-2840580	DEL	PRECISE	60	0.816	0	0/0	0/0	0/0	0/0	0/0	0/0	0/0	0/0	0/1	0/1	1/1	0/1	0/1	1/1
Chromosome1-2840778	INS	PRECISE	63	0	0.408	0/0	0/0	0/0	0/0	0/0	0/0	0/0	0/0	0/1	0/1	0/1	0/1	0/1	0/1
Chromosome1-2841162	INS	PRECISE	149	0	0	0/0	0/0	0/0	0/0	0/0	0/0	0/0	0/0	0/1	0/1	1/1	0/1	0/1	0/1
Chromosome1-2841408	DEL	PRECISE	62	0	0	0/0	0/0	0/0	0/0	0/0	0/0	0/0	0/0	0/1	0/0	1/1	0/1	0/1	0/1
Chromosome1-2841659	DEL	PRECISE	904	0	0	0/1	1/1	0/1	1/1	1/1	1/1	0/1	0/1	1/1	1/1	1/1	1/1	1/1	1/1
Chromosome1-2841918	DEL	PRECISE	56	0	0	0/0	0/0	0/0	0/0	0/0	0/0	0/0	0/0	0/0	0/1	0/0	0/0	0/0	0/0
Chromosome1-2841988	INS	PRECISE	624	0	0	0/0	0/0	0/0	0/0	0/0	0/0	0/0	0/0	0/0	0/0	0/0	0/0	0/0	0/1

Chromosome1-2842271	DEL	PRECISE	243	0	0	0/0	0/1	0/1	0/0	1/1	0/1	0/0	0/0	0/1	0/1	0/0	0/1	1/1	0/0
Chromosome1-2842464	INS	PRECISE	54	0	0	0/0	0/0	0/0	0/0	0/0	0/0	0/0	0/0	0/0	0/0	0/0	0/0	0/0	0/1
Chromosome1-2842690	INS	PRECISE	50	0	0	0/0	0/1	0/1	0/0	1/1	0/1	0/0	0/0	0/1	0/1	0/1	1/1	1/1	0/0
Chromosome1-2842703	DEL	PRECISE	571	0	0	0/0	0/0	0/0	0/0	0/0	0/0	0/0	0/0	0/1	0/1	0/1	0/0	0/0	0/1
Chromosome1-2843008	INS	PRECISE	1010	0	0	0/0	0/0	0/0	0/0	0/0	0/0	0/0	0/0	0/0	0/0	0/0	0/0	0/0	0/1
Chromosome1-2843623	INS	PRECISE	54	0	0	0/0	0/0	0/0	0/0	0/0	0/0	0/0	0/0	0/1	0/0	0/1	0/0	0/0	0/1
Chromosome1-2843728	INS	PRECISE	368	0	0	0/1	0/1	0/0	0/0	0/0	0/0	0/0	0/0	0/0	0/0	0/0	0/0	0/0	0/0
Chromosome1-2845568	DEL	PRECISE	1304	0	0	0/1	0/1	0/0	0/0	0/0	0/0	0/0	0/0	0/0	0/0	0/0	0/0	0/0	0/0
Chromosome1-2845705	DEL	PRECISE	1570	0	0	0/1	0/1	0/0	0/0	0/0	0/0	0/0	0/0	0/0	0/0	0/0	0/0	0/0	0/0
Chromosome1-2845866	DEL	PRECISE	3762	0	0	0/1	0/1	0/0	0/0	0/0	0/0	0/0	0/0	0/0	0/0	0/0	0/0	0/0	0/0
Chromosome1-2846588	INS	PRECISE	4412	0	0.957	0/0	0/0	0/0	1/1	0/0	1/1	0/1	1/1	0/0	0/0	0/0	0/0	0/0	0/0
Chromosome1-2847137	DEL	PRECISE	99	0.408	0	0/0	0/0	0/0	0/0	0/0	0/0	0/0	0/0	0/1	0/1	0/1	0/1	0/1	0/1
Chromosome1-2847693	DEL	PRECISE	952	0	0	0/0	0/0	0/0	0/0	0/0	0/0	0/0	0/0	0/0	0/0	0/0	0/1	0/0	0/0
Chromosome1-2848638	DEL	PRECISE	560	0.289	0.289	0/0	0/1	0/1	1/1	1/1	1/1	1/1	0/1	0/1	0/1	0/1	0/1	0/1	0/0
Chromosome1-2848664	DEL	PRECISE	3511	0	0	0/0	0/0	0/0	0/0	0/0	0/0	0/0	0/0	0/1	0/1	0/1	0/1	0/1	0/1
Chromosome1-2849284	INS	PRECISE	111	0	0	0/0	0/0	0/0	0/0	0/0	0/0	0/0	0/0	0/1	0/0	0/1	0/0	0/0	0/1
Chromosome1-2849996	DEL	PRECISE	619	0	0	0/0	0/0	0/0	0/0	0/0	0/0	0/0	0/0	0/1	0/1	0/1	1/1	0/1	0/1
Chromosome1-2850295	DEL	PRECISE	166	0	0	0/0	0/0	0/0	0/0	0/0	0/0	0/0	0/0	0/0	0/0	0/0	1/1	0/1	0/0
Chromosome1-2850348	INS	PRECISE	150	0	0	0/0	0/0	0/0	0/0	0/0	0/0	0/0	0/0	0/0	0/0	0/0	0/0	0/0	0/1
Chromosome1-2850562	INS	PRECISE	363	0	0	0/0	0/0	0/0	0/0	0/0	0/0	0/0	0/0	0/0	0/0	0/0	0/0	0/0	0/1
Chromosome1-2850805	DEL	PRECISE	5031	0	0	0/0	0/0	0/0	0/0	0/0	0/0	0/0	0/0	0/1	0/0	0/1	0/1	0/0	0/0
Chromosome1-2850949	INS	PRECISE	117	0	0.363	0/1	1/1	0/1	1/1	1/1	1/1	1/1	1/1	0/0	1/1	0/1	0/1	1/1	1/1
Chromosome1-2851987	INS	PRECISE	924	0	0	0/0	0/0	0/0	0/0	0/0	0/0	0/0	0/0	0/0	0/0	0/0	0/0	0/0	0/1
Chromosome1-2852360	DEL	PRECISE	117	0	0	0/1	0/1	0/0	0/0	0/0	0/0	0/0	0/0	0/0	0/0	0/0	0/0	0/0	0/1
Chromosome1-2854234	INS	PRECISE	52	0	0	0/1	0/1	0/0	0/0	0/0	0/0	0/1	0/0	0/0	0/0	0/0	0/0	0/0	0/0
Chromosome1-2854245	DEL	PRECISE	203	0	0	0/0	0/0	0/0	0/0	0/0	0/0	0/0	0/0	0/1	0/0	0/0	0/0	0/0	0/0
Chromosome1-2854293	INS	PRECISE	825	0	0	0/0	0/0	0/0	0/0	0/0	0/0	0/1	0/0	0/0	0/0	0/0	0/0	0/0	0/0
Chromosome1-2854299	BND	PRECISE	0	0	0	0/0	0/0	0/0	0/0	0/0	0/0	0/0	0/0	0/0	0/0	0/0	0/0	0/0	0/1
Chromosome1-2854801	INS	PRECISE	1236	0	0	0/0	0/0	0/0	0/0	0/0	0/0	0/0	0/0	0/0	0/0	0/0	0/0	0/1	0/0
Chromosome1-2856658	INS	PRECISE	5097	0	0.707	1/1	1/1	0/0	0/0	0/0	0/0	0/0	0/0	0/0	0/0	0/0	0/0	0/0	0/0
Chromosome1-2857550	INS	PRECISE	1285	0	0.707	0/0	0/0	0/0	0/0	0/0	0/0	0/0	0/0	0/1	0/0	0/1	0/0	0/0	0/0
Chromosome1-2857636	DEL	PRECISE	103	0	0	0/0	0/0	0/0	0/0	0/0	0/0	0/0	0/0	0/1	0/1	0/1	0/1	0/1	0/1
Chromosome1-2857909	DEL	PRECISE	58	0	0	0/0	0/0	0/0	0/0	0/0	0/0	0/0	0/0	0/0	0/0	0/0	0/1	0/1	0/1
Chromosome1-2858475	DEL	PRECISE	1934	0	0	0/1	0/1	0/0	0/0	0/0	0/0	0/0	0/0	0/0	0/0	0/0	0/0	0/0	0/0
Chromosome1-2858670	DEL	PRECISE	6373	0	0	0/1	0/1	0/0	0/0	0/0	0/0	0/0	0/0	0/0	0/0	0/0	0/0	0/0	0/0
Chromosome1-2859609	INS	PRECISE	553	0	0	0/0	0/0	0/0	0/0	0/0	0/0	0/0	0/0	0/0	0/1	0/0	0/0	0/0	0/0
Chromosome1-2861379	INS	PRECISE	284	0	0	0/0	0/0	0/0	0/0	0/0	0/0	0/0	0/0	0/0	0/1	0/0	0/0	0/0	0/0
Chromosome1-2861477	INS	PRECISE	1401	0	0	0/0	0/0	0/0	0/0	0/0	0/0	0/0	0/0	0/0	1/1	0/0	0/0	0/0	0/0
Chromosome1-2862711	DEL	PRECISE	1074	0	0	0/0	0/0	0/0	0/0	0/0	0/0	0/0	0/0	0/1	0/1	0/1	0/1	0/1	0/1
Chromosome1-2862882	DEL	PRECISE	5534	0.535	0	0/1	1/1	0/1	1/1	1/1	1/1	1/1	1/1	1/1	1/1	1/1	1/1	1/1	1/1
Chromosome1-2863533	DEL	PRECISE	133	0	0	0/0	0/0	0/0	0/0	0/0	0/0	0/0	0/0	0/1	0/0	0/0	0/0	0/1	0/1
Chromosome1-2864008	INS	PRECISE	364	0	0.707	0/0	0/0	0/0	0/0	0/0	0/0	0/1	0/1	0/0	0/0	0/0	0/0	0/0	0/0
Chromosome1-2864697	INS	PRECISE	2808	0	0	0/0	0/0	0/0	0/0	0/0	0/0	0/0	0/0	0/0	0/0	0/0	0/0	0/0	1/1
Chromosome1-2865486	INS	PRECISE	111	0	0.516	0/0	0/0	0/0	0/0	0/0	0/0	0/0	0/0	0/1	0/1	0/1	0/1	0/1	1/1
Chromosome1-2865596	INS	PRECISE	594	0	0	0/0	0/0	0/0	0/0	0/1	0/0	0/0	0/0	0/0	0/0	0/0	0/0	0/0	0/0
Chromosome1-2866866	DEL	PRECISE	1406	0	0	0/0	0/0	0/0	1/1	0/0	0/1	0/1	0/1	0/0	0/0	0/0	0/0	0/0	0/0
Chromosome1-2867184	DEL	PRECISE	229	0	0	0/0	0/0	0/0	0/0	0/0	0/0	0/0	0/0	0/1	0/1	0/1	0/0	0/0	0/0
Chromosome1-2867425	BND	PRECISE	0	0.707	0	0/1	0/1	0/0	0/0	0/0	0/0	0/0	0/0	0/0	0/0	0/0	0/0	0/0	0/0
Chromosome1-2867828	DEL	PRECISE	50	0	0	0/0	0/0	0/0	0/0	0/0	0/0	0/0	0/0	0/0	0/0	0/0	0/0	0/0	0/1
Chromosome1-2868260	DEL	PRECISE	925	0	0	0/0	0/0	0/0	0/0	0/0	0/0	0/0	0/0	0/1	0/1	0/1	0/1	0/1	0/1
Chromosome1-2868467	BND	PRECISE	0	0	0	0/0	0/0	0/0	0/0	0/0	0/0	0/0	0/0	0/0	0/0	1/1	0/1	0/1	0/1
Chromosome1-2868717	DUP	PRECISE	10515	0	0	0/0	0/0	0/0	0/0	0/0	0/0	0/0	0/0	0/0	0/0	0/0	0/0	0/1	0/0
Chromosome1-2868763	BND	PRECISE	0	0	0	0/0	0/0	0/0	0/0	0/0	0/0	0/0	0/0	0/1	0/1	1/1	0/0	1/1	0/1
Chromosome1-2868874	DEL	PRECISE	60	0	0	0/0	0/0	0/0	0/0	0/0	0/0	0/0	0/0	0/1	0/1	0/0	0/0	0/1	0/0
Chromosome1-2869037	DEL	PRECISE	308	0	0	0/0	0/0	0/0	0/0	0/0	0/0	0/0	0/0	0/0	0/0	0/0	0/0	0/0	0/1
Chromosome1-2869191	DEL	PRECISE	97	0	0	0/0	0/0	0/0	0/0	0/0	0/0	0/0	0/0	0/1	0/1	0/1	0/1	0/1	0/1
Chromosome1-2870028	INS	PRECISE	59	0.408	0	0/0	0/0	0/0	0/0	0/0	0/0	0/0	0/0	0/1	0/1	0/1	0/1	1/1	0/1
Chromosome1-2870696	DEL	PRECISE	509	0	0	0/0	0/1	0/1	0/0	1/1	1/1	0/0	0/0	0/1	0/1	0/1	0/1	0/1	0/1
Chromosome1-2871754	DEL	PRECISE	641	0.707	0.707	0/1	1/1	0/0	0/0	0/0	0/0	0/0	0/0	0/0	0/0	0/0	0/0	0/0	0/0
Chromosome1-2871809	INS	PRECISE	1352	0	0.707	0/0	0/0	0/0	0/0	0/0	0/0	0/1	0/1	0/0	0/0	0/0	0/0	0/0	0/0
Chromosome1-2872682	DEL	PRECISE	82	0	0	0/1	0/1	0/0	0/0	0/0	0/0	0/0	0/0	0/0	0/0	0/0	0/0	0/0	1/1
Chromosome1-2873501	INS	PRECISE	153	0	0.577	0/0	0/0	0/0	0/0	0/0	0/0	0/0	0/0	0/0	0/0	0/1	0/1	0/0	0/1
Chromosome1-2873521	DEL	PRECISE	249	0	0	0/1	0/1	0/0	0/0	0/0	0/0	0/0	0/0	0/0	0/0	0/0	0/0	0/0	0/0
Chromosome1-2876421	DEL	PRECISE	83	0	0	0/0	0/0	0/0	0/0	0/0	0/0	0/0	0/0	0/0	0/0	0/0	0/0	0/0	0/1
Chromosome1-2876879	DEL	PRECISE	237	0	0	0/0	0/0	0/0	0/0	0/0	0/0	0/0	0/0	0/1	0/1	0/1	0/1	0/1	0/1
Chromosome1-2877968	INS	PRECISE	2853	0	0	0/0	0/0	0/0	0/0	0/1	0/0	0/0	0/0	0/0	0/0	0/0	0/0	0/0	0/0
Chromosome1-2878495	DEL	PRECISE	790	0	0	0/0	0/0	0/0	0/0	0/0	0/0	0/0	0/0	0/0	0/0	0/0	0/0	0/0	1/1
Chromosome1-2879358	INS	PRECISE	66	0	0.577	0/0	0/0	0/0	0/0	0/0	0/0	0/0	0/0	0/0	0/0	0/0	0/1	0/0	0/0
Chromosome1-2879616	DEL	PRECISE	852	0	0	0/0	0/0	0/0	0/0	0/0	0/0	0/0	0/0	0/0	0/0	0/1	0/1	0/1	1/1
Chromosome1-2879617	DEL	PRECISE	3225	0.447	0.894	0/0	0/0	0/0	0/0	0/0	0/0	0/0	0/0	0/0	0/0	0/0	0/1	0/0	1/1
Chromosome1-2880069	DEL	PRECISE	59	0	0	0/0	0/0	0/0	0/0	0/0	0/0	0/0	0/0	0/0	0/0	0/1	0/1	0/1	1/1
Chromosome1-2880835	INS	PRECISE	122	0	0	0/0	0/0	0/0	0/0	0/0	0/0	0/0	0/0	0/1	0/0	0/0	0/0	0/0	0/0
Chromosome1-2883821	DEL	PRECISE	270	0	0	0/1	1/1	0/1	1/1	0/1	1/1	0/0	0/1	0/0	0/1	0/1	0/1	0/1	0/0

Chromosome1-2884647	INS	PRECISE	161	0	0	0/0	0/0	0/0	1/1	0/0	0/1	0/0	0/1	0/0	0/0	0/0	0/0	0/0	0/0
Chromosome1-2884777	DEL	PRECISE	86	0	0	0/1	0/1	0/0	0/0	0/0	0/0	0/0	0/0	0/0	0/0	0/0	0/0	0/0	0/0
Chromosome1-2885388	DEL	PRECISE	514	0	0	0/1	0/1	0/0	0/0	0/0	0/0	0/0	0/0	0/1	0/1	0/1	0/1	0/1	0/1
Chromosome1-2885399	INS	PRECISE	1715	0	0	0/0	0/0	0/0	0/0	1/1	0/0	0/0	0/0	0/0	0/0	0/0	0/0	0/0	0/0
Chromosome1-2885453	DEL	PRECISE	1932	0	0	0/1	0/1	0/0	0/0	0/1	0/0	0/0	0/0	0/1	0/1	0/1	0/1	0/1	0/1
Chromosome1-2885699	DEL	PRECISE	122	0	0	0/1	0/1	0/0	0/0	0/0	0/0	0/0	0/0	0/1	0/1	0/1	0/1	0/1	0/1
Chromosome1-2885912	INS	PRECISE	530	0	0	0/0	0/0	0/0	0/0	0/0	0/0	0/0	0/0	0/0	0/0	0/0	0/0	0/0	0/1
Chromosome1-2886373	DEL	PRECISE	233	0	0	0/0	0/0	0/1	1/1	1/1	1/1	0/1	0/1	0/1	0/1	0/0	0/1	0/0	0/0
Chromosome1-2887015	DEL	PRECISE	876	0	0	0/1	0/1	0/1	1/1	1/1	1/1	1/1	1/1	1/1	1/1	0/0	1/1	0/1	1/1
Chromosome1-2888539	DEL	PRECISE	253	0	0	0/1	0/1	0/0	0/0	0/0	0/0	0/0	0/0	0/0	0/0	0/0	0/0	0/1	0/0
Chromosome1-2888881	DEL	PRECISE	492	0	0	0/0	0/0	0/0	0/0	0/0	0/0	0/0	0/0	0/1	0/0	0/0	0/0	0/0	0/0
Chromosome1-2889118	INS	PRECISE	623	0	0	0/0	0/0	0/0	0/0	0/0	0/0	0/0	0/0	0/0	0/0	0/0	0/1	0/0	0/0
Chromosome1-2889172	DEL	PRECISE	126	0	0	0/0	0/1	0/0	0/0	0/0	0/0	0/0	0/0	0/1	0/0	0/0	0/1	0/0	0/0
Chromosome1-2889208	INS	PRECISE	137	0	0	0/0	0/1	0/0	0/0	0/0	0/0	0/0	0/0	0/1	0/0	0/0	0/0	0/0	0/0
Chromosome1-2889371	INS	PRECISE	57	0	0.441	0/0	0/0	0/0	1/1	0/0	0/0	1/1	1/1	0/1	0/0	0/0	0/0	0/0	1/1
Chromosome1-2891327	INS	PRECISE	142	0	0	0/0	0/0	0/0	0/0	0/0	0/0	0/0	0/0	0/0	0/0	0/0	0/0	0/1	0/0
Chromosome1-2891441	INS	PRECISE	325	0	0	0/0	0/0	0/0	0/0	0/1	0/0	0/0	0/0	0/0	0/0	0/0	0/0	0/0	0/0
Chromosome1-2891847	DEL	PRECISE	706	0	0	0/1	1/1	0/0	0/0	0/0	0/0	0/0	0/0	0/0	0/0	0/0	0/0	0/0	0/0
Chromosome1-2892217	INS	PRECISE	1040	0	0.707	0/0	0/0	0/0	0/0	0/0	0/0	0/1	0/0	0/0	0/0	0/0	0/0	0/0	0/0
Chromosome1-2892242	INS	PRECISE	52	0	0	0/0	0/0	0/0	0/0	0/0	0/0	0/0	0/0	0/1	0/0	0/0	0/0	0/0	0/0
Chromosome1-2893513	INS	PRECISE	824	0	0.707	0/0	0/0	0/0	0/0	0/0	0/0	0/1	0/0	0/0	0/0	0/0	0/0	0/0	0/0
Chromosome1-2893530	INS	PRECISE	10962	0	0	0/0	0/0	0/0	0/0	0/0	0/0	0/0	0/0	0/0	0/0	0/0	0/0	1/1	0/0
Chromosome1-2893650	DEL	PRECISE	55	0	0	0/1	0/1	0/0	0/0	0/0	0/0	0/0	0/0	0/0	0/0	0/0	0/0	0/0	0/0
Chromosome1-2894371	INS	PRECISE	5644	0	0	0/0	0/0	0/0	0/0	1/1	0/0	0/0	0/0	0/0	0/0	0/0	0/0	0/0	0/0
Chromosome1-2894543	DEL	PRECISE	452	0	0	0/0	0/0	0/0	0/0	0/0	0/0	0/0	0/0	0/0	0/0	0/0	0/0	0/0	0/1
Chromosome1-2894820	INS	PRECISE	105	0	0.354	0/1	0/1	0/0	0/0	0/0	0/0	0/0	0/0	0/1	0/1	0/1	0/1	0/1	0/1
Chromosome1-2894868	INS	PRECISE	1899	0	0	0/0	0/0	0/0	0/0	0/0	0/0	0/0	0/0	0/0	0/0	0/0	0/1	0/0	0/0
Chromosome1-2895287	INS	PRECISE	636	0	0	0/0	0/0	0/0	0/0	0/0	0/0	0/0	0/0	0/0	0/0	0/1	0/0	0/1	0/1
Chromosome1-2895828	INS	PRECISE	137	0	0	0/0	0/0	0/0	0/0	0/0	0/0	0/1	0/1	0/0	0/0	0/0	0/0	0/0	0/0
Chromosome1-2896051	INS	PRECISE	5068	0	0	0/0	0/0	0/0	0/0	1/1	0/0	0/0	0/0	0/0	0/0	0/0	0/0	0/0	0/0
Chromosome1-2896084	INS	PRECISE	462	0	0.707	0/0	0/0	0/0	0/0	0/0	0/0	0/0	0/0	0/1	0/1	0/0	0/0	0/0	0/0
Chromosome1-2896327	INS	PRECISE	1200	0	0	0/0	0/0	0/0	0/0	0/1	0/0	0/0	0/0	0/0	0/0	0/0	0/0	0/0	0/0
Chromosome1-2897166	INS	PRECISE	362	0	0	0/1	0/1	0/0	0/0	0/0	0/0	0/0	0/0	0/0	0/0	0/0	0/0	0/0	0/0
Chromosome1-2897250	BND	PRECISE	0	0	0	0/0	0/0	0/0	0/0	0/1	0/0	0/0	0/0	0/0	0/0	0/0	0/0	0/0	0/0
Chromosome1-2897587	BND	PRECISE	0	0	0	0/0	0/0	0/0	0/0	0/0	0/0	0/0	0/0	0/1	0/0	0/0	0/0	0/0	0/0
Chromosome1-2897676	DEL	PRECISE	304	0	0	0/1	0/1	0/1	1/1	1/1	1/1	1/1	1/1	1/1	1/1	0/0	1/1	0/1	1/1
Chromosome1-2897741	INS	PRECISE	96	0	0	0/1	0/1	0/0	0/0	0/0	0/0	0/0	0/0	0/1	0/1	0/0	0/0	0/0	0/0
Chromosome1-2898299	DEL	PRECISE	137	0	0	0/1	0/1	0/1	1/1	1/1	1/1	1/1	1/1	1/1	1/1	0/1	1/1	0/1	1/1
Chromosome1-2898483	INS	PRECISE	150	0.976	0.787	0/1	0/1	0/0	0/0	0/0	0/0	0/0	0/0	0/1	0/1	0/1	0/0	0/1	0/1
Chromosome1-2899385	INS	PRECISE	71	0	0.333	0/1	0/1	0/0	0/0	0/1	0/0	0/0	0/0	0/1	0/1	0/1	0/1	0/1	0/1
Chromosome1-2899564	DEL	PRECISE	5380	0	0	0/0	0/0	0/0	0/0	0/0	0/0	0/0	0/0	0/0	0/0	0/0	0/0	0/0	0/1
Chromosome1-2900043	DEL	PRECISE	232	0	0	0/1	0/1	0/0	0/0	0/1	0/0	0/0	0/0	0/1	0/1	0/1	0/0	0/1	0/1
Chromosome1-2900434	INS	PRECISE	400	0	0	0/0	0/0	0/0	0/0	0/0	0/0	0/0	0/0	0/0	0/0	0/0	0/0	0/0	0/1
Chromosome1-2900766	DEL	PRECISE	1883	0	0	0/0	0/0	0/0	0/0	0/0	0/0	0/0	0/0	0/0	0/0	0/0	0/0	0/1	0/0
Chromosome1-2901122	DEL	PRECISE	73	0	0	0/0	0/0	0/0	0/0	0/0	0/0	0/0	0/0	0/0	0/1	0/0	0/1	0/1	0/0
Chromosome1-2901247	INS	PRECISE	1334	0	0	0/0	0/0	0/0	0/0	0/0	0/0	0/0	0/0	0/0	0/0	0/0	0/0	0/1	0/0
Chromosome1-2901899	DEL	PRECISE	193	0	0	0/0	0/0	0/0	0/0	0/0	0/0	0/0	0/0	0/0	0/0	0/1	0/0	0/0	0/1
Chromosome1-2902113	INS	PRECISE	688	0	0.707	0/0	0/0	0/0	0/0	0/0	0/0	0/1	0/1	0/0	0/0	0/0	0/0	0/0	0/0
Chromosome1-2902568	DEL	PRECISE	2882	0	0	0/0	0/0	0/0	0/0	0/0	0/0	0/0	0/0	0/0	0/0	0/1	0/0	0/0	0/0
Chromosome1-2903208	INS	PRECISE	178	0	0	0/0	0/0	0/0	0/0	0/0	0/0	0/0	0/0	0/0	0/0	0/0	0/1	0/0	0/0
Chromosome1-2905080	DEL	PRECISE	207	0	0	0/0	0/0	0/0	0/0	0/1	0/0	0/0	0/0	0/0	0/0	0/0	0/0	0/0	0/0
Chromosome1-2905140	INS	PRECISE	2442	0	0.707	0/0	0/0	0/0	0/0	0/0	0/0	0/1	0/1	0/0	0/0	0/0	0/0	0/0	0/0
Chromosome1-2905210	INS	PRECISE	74	0	0	0/1	0/0	0/0	0/0	0/0	0/0	0/0	0/0	0/1	0/1	0/1	0/1	0/1	0/1
Chromosome1-2905275	DEL	PRECISE	706	0	0	0/0	0/0	0/0	0/0	0/0	0/0	0/0	0/0	0/1	0/1	0/1	0/1	0/1	0/1
Chromosome1-2905931	INS	PRECISE	75	0	0	0/0	0/0	0/0	0/0	0/0	0/0	0/0	0/0	0/0	0/0	0/0	0/0	0/0	0/1
Chromosome1-2906211	INS	PRECISE	112	0	0	0/1	0/1	0/0	0/0	0/0	0/0	0/0	0/0	0/0	0/0	0/0	0/0	0/0	0/0
Chromosome1-2906437	DEL	PRECISE	78	0	0	0/1	0/1	0/0	0/0	0/0	0/0	0/0	0/0	0/0	0/0	0/0	0/0	0/0	0/0
Chromosome1-2906871	INS	PRECISE	926	0	0	0/0	0/0	0/0	0/0	0/0	0/0	0/0	0/0	0/0	0/0	0/0	0/1	0/0	0/0
Chromosome1-2907189	INS	PRECISE	5475	0	0	0/0	0/0	0/0	0/0	0/1	0/0	0/0	0/0	0/0	0/0	0/0	0/0	0/0	0/0
Chromosome1-2908045	INS	PRECISE	1128	0	0	0/1	0/1	0/0	0/0	0/0	0/0	0/0	0/0	0/0	0/0	0/0	0/0	0/0	0/0
Chromosome1-2908075	INS	PRECISE	1836	0	0	0/0	0/0	0/0	0/0	0/0	0/0	0/1	0/0	0/0	0/0	0/0	0/0	0/0	0/0
Chromosome1-2908241	DEL	PRECISE	454	0	0	0/0	0/0	0/0	0/0	0/0	0/0	0/0	0/0	0/0	0/0	0/0	0/0	0/0	0/1
Chromosome1-2909291	INS	PRECISE	841	0	0	0/0	0/0	0/0	0/0	0/1	0/0	0/0	0/0	0/0	0/0	0/0	0/0	0/0	0/0
Chromosome1-2910180	INS	PRECISE	211	0	0.354	0/1	0/1	0/0	0/0	0/0	0/0	0/0	0/0	0/1	0/1	0/1	0/1	0/1	1/1
Chromosome1-2910386	DEL	PRECISE	129	0	0	0/1	0/1	0/0	0/0	0/0	0/0	0/0	0/0	0/1	0/1	0/1	0/1	0/1	1/1
Chromosome1-2910510	INS	PRECISE	135	0	0	0/0	0/0	0/0	0/0	0/0	0/0	0/0	0/0	0/0	0/0	0/0	0/0	0/0	0/1
Chromosome1-2910533	DEL	PRECISE	85	0	0	0/0	0/0	0/0	0/0	0/0	0/0	0/0	0/0	0/0	0/0	0/0	0/0	0/1	0/0
Chromosome1-2910670	INS	PRECISE	212	0	0	0/0	0/0	0/0	0/0	0/0	0/0	0/0	0/0	0/0	0/0	0/0	0/0	0/0	0/1
Chromosome1-2910747	DEL	PRECISE	50	0	0	0/0	0/0	0/0	0/0	0/1	0/0	0/0	0/0	0/0	0/0	0/0	0/0	0/0	0/0
Chromosome1-2910977	INS	PRECISE	60	0	0	0/0	0/0	0/0	0/0	0/0	0/0	0/0	0/0	0/0	0/0	0/0	0/1	0/0	0/0
Chromosome1-2912287	DEL	PRECISE	75	0	0	0/0	0/0	0/0	0/0	0/0	0/0	0/0	0/0	0/0	0/0	0/1	0/0	0/0	0/1
Chromosome1-2913132	DEL	PRECISE	53	0	0	0/0	0/0	0/0	0/0	0/0	0/0	0/0	0/0	0/0	0/0	0/0	0/1	0/0	0/0
Chromosome1-2914163	DEL	PRECISE	1311	0	0	0/0	0/0	0/1	1/1	1/1	1/1	0/1	0/1	0/1	0/1	0/0	0/1	0/0	0/0

Chromosome1-2914301	DEL	PRECISE	84	0	0	0/0	0/0	0/1	0/0	0/1	0/1	0/0	0/0	0/1	0/1	0/0	0/1	0/0	0/0
Chromosome1-2914726	INS	PRECISE	623	0	0.816	0/0	0/0	0/1	0/0	0/1	0/1	0/0	0/0	0/1	0/1	0/0	0/1	0/0	0/0
Chromosome1-2914772	INS	PRECISE	50	0	0	0/0	0/0	0/0	0/0	0/0	0/0	0/0	0/0	0/0	0/0	0/0	0/1	0/1	0/0
Chromosome1-2914811	DEL	PRECISE	54	0	0	0/1	0/1	0/0	0/0	0/0	0/0	0/0	0/0	0/0	0/1	0/0	0/1	0/1	0/0
Chromosome1-2915108	INS	PRECISE	5495	0	0	0/0	0/0	0/0	1/1	0/0	0/0	0/0	0/0	0/0	0/0	0/0	0/0	0/0	0/0
Chromosome1-2916600	DEL	PRECISE	392	0	0	0/0	0/0	0/0	0/0	0/0	0/0	0/0	0/0	0/0	0/0	0/1	0/0	0/0	0/1
Chromosome1-2916861	DEL	PRECISE	196	0	0	0/0	0/0	0/0	0/0	0/0	0/0	0/0	0/0	0/1	0/0	0/0	0/0	0/0	0/0
Chromosome1-2917049	INS	PRECISE	54	0	0.333	0/1	0/1	0/0	0/0	0/1	0/0	0/0	0/0	0/1	0/0	0/1	0/0	0/1	1/1
Chromosome1-2917369	DEL	PRECISE	677	0	0	0/1	0/1	0/0	0/0	0/1	0/0	0/0	0/0	0/1	0/0	0/1	0/1	0/1	1/1
Chromosome1-2918785	INS	PRECISE	122	0	0	0/0	0/0	0/0	0/0	0/0	0/0	0/0	0/0	0/0	0/0	0/0	0/0	0/1	0/0
Chromosome1-2918987	DEL	PRECISE	86	0	0	0/0	0/0	0/0	0/0	0/0	0/0	0/0	0/0	0/0	0/1	0/0	0/0	0/0	0/0
Chromosome1-2919091	DEL	PRECISE	118	0	0	0/0	0/0	0/0	0/0	0/0	0/0	0/0	0/0	0/0	0/0	0/0	0/1	0/0	0/0
Chromosome1-2919588	INS	PRECISE	761	0	0	0/0	0/0	0/0	0/0	0/0	0/0	0/0	0/0	0/0	0/0	0/0	0/1	0/0	0/0
Chromosome1-2919792	DEL	PRECISE	118	0	0	0/1	0/1	0/0	0/0	0/0	0/0	0/0	0/0	0/1	0/0	0/1	0/0	0/0	0/1
Chromosome1-2919911	DEL	PRECISE	1034	0	0	0/0	0/0	0/0	0/0	0/0	0/0	0/0	0/0	0/0	1/1	0/0	0/0	0/0	0/1
Chromosome1-2920599	DEL	PRECISE	63	0	0	0/0	0/0	0/0	0/0	0/0	0/0	0/0	0/0	0/0	0/0	0/0	0/0	0/1	0/0
Chromosome1-2920695	INS	PRECISE	5304	0	0	0/0	0/0	0/0	0/0	0/0	0/0	0/0	0/0	0/0	0/0	0/0	0/0	0/0	1/1
Chromosome1-2920732	INS	PRECISE	7020	0	0	0/0	0/0	0/0	0/0	0/0	0/0	0/0	0/0	0/0	0/1	0/0	0/0	0/0	0/0
Chromosome1-2920866	INS	PRECISE	713	0	0	0/0	0/0	0/0	0/0	0/0	0/0	0/0	0/0	0/0	0/1	0/0	0/0	0/0	0/0
Chromosome1-2921023	DEL	PRECISE	80	0	0	0/0	0/0	0/0	0/0	0/0	0/0	0/0	0/0	0/0	0/1	0/0	0/0	0/0	0/1
Chromosome1-2921034	INS	PRECISE	7005	0	0	0/0	0/0	0/0	0/0	0/0	0/0	0/0	0/0	0/0	0/0	0/0	0/0	1/1	0/0
Chromosome1-2921034	BND	PRECISE	0	0	0	0/0	0/0	0/0	0/0	0/0	0/0	0/0	0/0	0/0	0/0	0/0	0/0	0/1	0/0
Chromosome1-2921186	DEL	PRECISE	56	0	0	0/1	0/1	0/0	0/0	0/0	0/0	0/0	0/0	0/0	0/0	0/1	0/0	0/0	0/1
Chromosome1-2921532	INS	PRECISE	60	0	0.707	0/0	0/0	0/0	0/0	0/1	0/0	0/0	0/0	0/0	0/0	0/0	0/0	0/0	0/1
Chromosome1-2921820	INS	PRECISE	5508	0	0	0/0	0/0	0/0	1/1	0/0	0/0	0/0	0/0	0/0	0/0	0/0	0/0	0/0	0/0
Chromosome1-2921992	DEL	PRECISE	2563	0	0	0/0	0/0	0/0	0/0	0/0	0/0	0/0	0/0	0/0	0/1	0/0	0/0	0/0	0/0
Chromosome1-2922045	DEL	PRECISE	5754	0	0	0/0	0/0	0/0	0/0	0/0	0/0	0/0	0/0	0/0	0/1	0/0	0/1	0/1	0/0
Chromosome1-2922176	DEL	PRECISE	873	0	0	0/0	0/0	0/0	0/0	0/0	0/0	0/0	0/0	0/0	0/0	0/0	0/0	0/0	0/1
Chromosome1-2922465	INS	PRECISE	5004	0	0	0/0	0/0	0/0	0/0	0/0	0/0	0/0	0/0	0/0	0/0	0/0	0/0	0/0	0/1
Chromosome1-2922753	DEL	PRECISE	153	0	0	0/0	0/0	0/0	0/0	0/0	0/0	0/0	0/0	0/1	0/0	0/0	0/0	0/0	0/0
Chromosome1-2922812	DEL	PRECISE	1148	0	0	0/0	0/0	0/0	0/0	0/0	0/0	0/0	0/0	0/0	0/0	0/0	0/0	0/0	0/1
Chromosome1-2922869	INS	PRECISE	4527	0	0	0/0	0/0	0/0	0/0	0/0	0/0	0/0	0/0	1/1	0/0	0/0	0/0	0/0	0/0
Chromosome1-2923165	INS	PRECISE	5430	0	0	0/0	0/0	0/0	0/0	0/0	0/0	0/0	0/0	0/0	0/0	0/0	0/0	0/0	0/1
Chromosome1-2923415	DEL	PRECISE	1015	0.926	0	0/1	0/1	0/0	0/0	0/0	0/0	0/0	0/0	0/1	0/1	0/1	0/1	0/1	1/1
Chromosome1-2924689	DEL	PRECISE	200	0	0	0/1	0/1	0/0	0/0	0/0	0/0	0/0	0/0	0/1	0/1	0/1	0/1	0/1	0/1
Chromosome1-2924818	DEL	PRECISE	151	0.376	0	0/1	0/1	0/1	0/0	0/1	0/1	0/1	0/1	1/1	1/1	0/1	1/1	0/1	0/1
Chromosome1-2926005	INS	PRECISE	97	0	0	0/0	0/0	0/1	1/1	0/1	1/1	1/1	1/1	1/1	0/1	0/1	1/1	0/0	0/1
Chromosome1-2926151	INS	PRECISE	353	0	0	0/0	0/0	0/0	0/0	0/1	0/0	0/0	0/0	0/0	0/0	0/0	0/0	0/0	0/0
Chromosome1-2926727	DEL	PRECISE	591	0	0	0/1	0/0	0/0	0/0	0/0	0/0	0/0	0/0	0/0	0/1	0/1	0/1	0/0	0/1
Chromosome1-2927554	INS	PRECISE	441	0	0	0/0	0/0	0/0	0/0	0/0	0/0	0/0	0/0	0/0	0/0	0/0	0/0	0/0	0/1
Chromosome1-2927647	DEL	PRECISE	239	0	0	0/1	0/1	0/0	0/0	0/0	0/0	0/0	0/0	0/1	0/1	0/1	0/1	0/0	0/1
Chromosome1-2927775	DEL	PRECISE	139	0	0	0/0	0/0	0/0	0/0	0/0	0/0	0/0	0/0	0/1	0/0	0/0	0/0	0/0	0/0
Chromosome1-2928325	INS	PRECISE	791	0	0	0/0	0/0	0/0	0/0	0/0	0/0	0/0	0/0	0/0	0/0	0/0	0/0	0/0	0/1
Chromosome1-2928467	INS	PRECISE	326	0	0	0/0	0/0	0/0	0/0	0/0	0/0	0/0	0/0	0/0	0/0	0/0	0/0	0/0	1/1
Chromosome1-2928920	INS	PRECISE	1298	0	0	0/0	0/0	0/0	0/0	0/0	0/0	0/0	0/0	0/0	0/0	0/0	0/0	0/1	0/0
Chromosome1-2929486	DEL	PRECISE	69	0	0	0/0	0/0	0/0	0/0	0/0	0/0	0/0	0/0	0/1	0/0	0/1	0/1	0/1	0/1
Chromosome1-2929898	INS	PRECISE	58	0	0	0/1	0/1	0/0	0/0	0/0	0/0	0/0	0/0	0/1	0/1	0/1	0/1	0/1	0/1
Chromosome1-2930895	DEL	PRECISE	150	0	0	0/1	0/1	0/0	0/0	0/0	0/0	0/0	0/0	0/1	0/1	0/1	1/1	0/1	0/1
Chromosome1-2931081	DEL	PRECISE	103	0	0	0/1	0/1	0/0	0/0	0/0	0/0	0/0	0/0	0/1	0/1	0/1	1/1	0/1	0/1
Chromosome1-2931203	INS	PRECISE	3442	0	0.577	0/0	0/0	0/0	1/1	0/0	1/1	1/1	1/1	0/0	0/0	0/0	0/0	0/0	0/0
Chromosome1-2931431	INS	PRECISE	211	0	0	0/0	0/0	0/0	0/0	0/0	0/0	0/1	0/0	0/0	0/0	0/0	0/0	0/0	0/0
Chromosome1-2932065	INS	PRECISE	2214	0	0	0/0	0/0	0/0	0/0	0/0	0/0	0/0	0/0	0/0	0/0	0/0	0/0	0/0	1/1
Chromosome1-2932397	DEL	PRECISE	67	0.447	0.447	0/1	0/1	0/0	0/0	0/0	0/0	0/0	0/0	0/1	0/1	0/0	0/0	0/1	0/0
Chromosome1-2933491	INS	PRECISE	784	0	0	0/0	0/0	0/0	0/0	0/0	0/0	0/0	0/0	0/0	0/1	0/0	0/0	0/0	0/0
Chromosome1-2933601	INS	PRECISE	1381	0	0	0/0	0/0	0/0	0/0	0/1	0/0	0/0	0/0	0/0	0/0	0/0	0/0	0/0	0/0
Chromosome1-2933955	INS	PRECISE	137	0	0	0/0	0/0	0/0	0/0	0/0	0/0	0/1	0/0	0/0	0/0	0/0	0/0	0/0	0/0
Chromosome1-2934502	INS	PRECISE	84	0	0	0/0	0/0	0/0	0/0	0/1	0/0	0/0	0/0	0/0	0/0	0/0	0/0	0/0	0/0
Chromosome1-2934612	INS	PRECISE	239	0	0	0/0	0/0	0/0	0/0	0/0	0/0	0/0	0/0	0/0	0/0	0/0	0/0	0/1	0/0
Chromosome1-2934642	DEL	PRECISE	491	0.354	0.707	0/1	0/1	0/0	0/0	0/0	0/0	0/0	0/0	0/1	0/1	0/1	0/1	0/1	0/1
Chromosome1-2935702	DEL	PRECISE	130	0	0	0/0	0/0	0/0	0/0	0/0	0/0	0/0	0/0	0/0	0/1	0/0	0/0	0/0	0/1
Chromosome1-2935820	DEL	PRECISE	859	0	0	0/0	0/0	0/0	0/0	0/0	0/0	0/1	0/0	0/0	0/0	0/0	0/0	0/0	0/0
Chromosome1-2935995	DEL	PRECISE	157	0	0	0/0	0/0	0/0	0/0	0/1	0/0	0/0	0/0	0/0	0/0	0/0	0/0	0/0	0/1
Chromosome1-2935996	INS	PRECISE	75	0	0.354	0/1	0/1	0/0	0/0	0/0	0/0	0/0	0/0	0/1	0/1	0/0	0/1	0/1	0/1
Chromosome1-2936883	DEL	PRECISE	76	0	0	0/1	0/1	0/0	0/0	0/0	0/0	0/0	0/0	0/1	0/1	0/0	0/1	0/1	0/1
Chromosome1-2937087	BND	PRECISE	0	0	0	0/0	0/0	0/0	0/0	0/0	0/0	0/0	0/0	0/0	0/0	0/1	0/0	0/1	0/1
Chromosome1-2938783	INS	PRECISE	3280	0	0	0/0	0/0	0/0	0/0	0/1	0/0	0/0	0/0	0/0	0/0	0/0	0/0	0/0	0/0
Chromosome1-2938946	INS	PRECISE	689	0	0	0/0	0/0	0/0	0/0	0/0	0/0	0/0	0/0	0/0	0/1	0/0	0/0	0/0	0/0
Chromosome1-2939095	INS	PRECISE	232	0	0	0/0	0/0	0/0	0/0	0/0	0/0	0/0	0/0	0/0	0/0	0/0	0/1	0/0	0/0
Chromosome1-2939121	INS	PRECISE	1285	0	0	0/0	0/0	0/0	0/0	0/0	0/0	0/0	0/0	1/1	0/0	0/0	0/0	0/0	0/0
Chromosome1-2939374	INS	PRECISE	161	0	0.441	0/0	0/0	0/1	1/1	0/1	1/1	0/1	1/1	0/1	0/1	0/0	0/1	0/0	0/0
Chromosome1-2940375	INS	PRECISE	1064	0	0	0/0	0/0	0/0	0/0	0/0	0/0	0/0	0/0	0/1	0/0	0/0	0/0	0/0	0/0
Chromosome1-2940425	INS	PRECISE	100	0	0	0/1	0/1	0/0	0/0	0/0	0/0	0/0	0/0	0/1	0/1	0/1	0/1	0/1	0/1
Chromosome1-2942160	DEL	PRECISE	98	0	0	0/0	0/0	0/0	0/0	0/0	0/0	0/0	0/0	0/0	0/0	0/0	0/0	0/0	0/1

Chromosome1-2942265	INS	PRECISE	51	0	0	0/0	0/0	0/0	0/0	0/0	0/0	0/0	0/0	0/0	0/0	0/0	0/0	0/1	0/0
Chromosome1-2943501	INS	PRECISE	120	0	0.744	0/1	0/1	0/0	0/0	0/0	0/0	0/0	0/0	0/1	0/1	0/1	0/1	0/1	0/1
Chromosome1-2943781	DEL	PRECISE	55	0	0	0/1	1/1	0/0	0/0	0/0	0/0	0/0	0/0	0/1	0/1	0/1	1/1	0/1	0/1
Chromosome1-2945075	DEL	PRECISE	116	0	0	0/1	0/1	0/0	0/0	0/0	0/0	0/0	0/0	0/0	0/0	0/0	0/0	0/0	0/0
Chromosome1-2945774	DEL	PRECISE	189	0	0	0/0	0/0	0/0	0/0	0/1	0/0	0/0	0/0	0/0	0/0	0/0	0/0	0/0	0/0
Chromosome1-2946325	DEL	PRECISE	8865	0	0	0/1	0/1	0/0	0/0	0/1	0/0	1/1	0/0	0/1	1/1	0/1	0/1	0/1	1/1
Chromosome1-2947335	DEL	PRECISE	108	0	0	0/0	0/0	0/0	0/0	0/0	0/0	0/0	0/0	0/0	0/0	0/0	0/1	0/0	0/0
Chromosome1-2947896	INS	PRECISE	2086	0	0	0/0	0/0	0/0	0/0	0/0	0/0	0/0	0/0	0/0	0/0	0/0	0/0	0/0	0/1
Chromosome1-2948096	INS	PRECISE	76	0	0	0/0	0/0	0/0	0/0	0/0	0/0	0/0	0/0	0/0	0/1	0/0	0/1	0/1	0/1
Chromosome1-2948182	DEL	PRECISE	51	0	0	0/0	0/0	0/0	0/0	0/0	0/0	0/0	0/0	0/0	0/0	0/0	0/0	0/0	0/1
Chromosome1-2949346	DEL	PRECISE	150	0.926	0.463	0/1	0/1	0/0	0/0	0/0	0/0	0/0	0/0	0/0	0/1	0/1	0/0	0/1	0/1
Chromosome1-2949773	DEL	PRECISE	72	0	0	0/0	0/0	0/0	0/0	0/0	0/0	0/1	0/0	0/0	0/0	0/0	0/0	0/0	0/0
Chromosome1-2950848	INS	PRECISE	327	0	0	0/0	0/0	0/0	0/0	0/0	0/0	0/0	0/0	0/0	0/0	0/0	0/0	0/1	0/0
Chromosome1-2951416	INS	PRECISE	1080	0	0	0/0	0/0	0/0	0/0	0/0	0/0	0/0	0/0	0/0	0/0	0/0	0/0	0/1	0/0
Chromosome1-2951433	INS	PRECISE	2077	0	0	0/0	0/0	0/0	0/0	0/1	0/0	0/0	0/0	0/0	0/0	0/0	0/0	0/0	0/0
Chromosome1-2951435	DEL	PRECISE	54	0	0	0/0	0/0	0/0	0/0	0/0	0/0	0/0	0/0	0/0	0/0	0/0	0/0	0/0	0/1
Chromosome1-2951554	DEL	PRECISE	50	0	0	0/0	0/0	0/0	1/1	0/0	0/1	1/1	0/1	0/0	0/0	0/0	0/0	0/0	0/0
Chromosome1-2951757	DEL	PRECISE	72	0	0	0/0	0/0	0/0	1/1	0/0	0/1	0/1	0/1	0/0	0/0	0/0	0/0	0/0	0/0
Chromosome1-2952095	DEL	PRECISE	441	0	0	0/0	0/0	0/0	0/0	0/0	0/0	0/1	0/0	0/0	0/0	0/0	0/0	0/0	0/0
Chromosome1-2952196	INS	PRECISE	347	0	0	0/0	0/0	0/0	0/0	0/0	0/0	0/0	0/0	0/0	0/0	0/0	0/0	0/0	0/1
Chromosome1-2953034	DEL	PRECISE	1778	0	0	0/0	0/0	0/0	0/0	0/1	0/0	0/0	0/0	0/1	0/1	0/1	0/1	0/1	1/1
Chromosome1-2953071	DEL	PRECISE	730	0	0	0/0	0/0	0/0	0/0	0/0	0/0	0/1	0/0	0/0	0/0	0/0	0/0	0/0	0/0
Chromosome1-2953148	INS	PRECISE	124	0	0.441	0/0	0/0	0/1	1/1	0/1	1/1	0/1	1/1	0/1	0/1	0/0	0/1	0/0	0/0
Chromosome1-2954385	DEL	PRECISE	50	0	0	0/0	0/0	0/0	0/0	0/0	0/0	0/0	0/0	0/0	0/0	0/0	0/0	0/0	0/1
Chromosome1-2954666	INS	PRECISE	220	0	0	0/0	0/0	0/0	0/0	0/0	0/0	0/0	0/0	0/0	0/0	0/0	0/0	0/0	0/1
Chromosome1-2955531	BND	PRECISE	0	0	0	0/0	0/0	0/0	0/0	0/1	0/0	0/0	0/0	0/0	0/0	0/0	0/0	0/0	0/0
Chromosome1-2955953	INS	PRECISE	51	0	0.577	0/0	0/0	0/0	0/0	0/0	0/0	0/0	0/0	0/1	0/0	0/1	0/0	0/0	0/1
Chromosome1-2956701	INS	PRECISE	109	0	0	0/0	0/0	0/0	0/0	0/0	0/0	0/0	0/0	0/1	0/0	0/1	0/0	0/0	0/1
Chromosome1-2956856	DEL	PRECISE	92	0	0	0/0	0/0	0/0	0/0	0/0	0/0	0/0	0/0	0/0	0/0	0/0	0/0	0/0	0/1
Chromosome1-2956981	INS	PRECISE	130	0	0.354	0/0	0/0	0/0	0/0	0/0	0/0	0/0	0/0	0/1	0/0	0/1	0/0	0/1	1/1
Chromosome1-2957019	INS	PRECISE	6119	0	0	0/0	0/0	0/0	0/0	0/0	0/0	0/1	0/0	0/0	0/0	0/0	0/0	0/0	0/0
Chromosome1-2957091	DEL	PRECISE	4484	0	0	0/0	0/0	0/0	0/0	0/0	0/0	0/0	0/0	1/1	0/0	1/1	0/1	0/0	0/0
Chromosome1-2959572	DEL	PRECISE	521	0	0	0/0	0/0	0/0	0/0	0/0	0/0	0/1	0/0	0/0	0/0	0/0	0/0	0/0	0/1
Chromosome1-2959775	DEL	PRECISE	1607	0	0	0/0	0/0	0/0	0/0	0/1	0/0	0/1	0/0	0/0	0/0	0/0	0/0	0/0	0/0
Chromosome1-2962302	INS	PRECISE	6433	0	0	0/0	0/0	0/0	0/0	0/0	0/0	0/0	0/0	0/0	0/0	0/0	0/0	0/0	0/1
Chromosome1-2962605	DEL	PRECISE	747	0	0	0/1	0/1	0/0	0/0	0/1	0/0	0/0	0/0	0/1	0/1	0/1	0/1	0/1	1/1
Chromosome1-2963101	DEL	PRECISE	419	0.707	0	0/1	0/1	0/0	0/0	0/0	0/0	0/0	0/0	0/1	0/1	0/1	0/1	0/1	1/1
Chromosome1-2963850	DEL	PRECISE	825	0.333	0.333	0/1	1/1	0/0	0/0	0/1	0/0	0/0	0/0	0/1	0/1	0/1	0/1	1/1	1/1
Chromosome1-2964322	INS	PRECISE	85	0	0	0/0	0/0	0/0	0/0	0/0	0/0	0/0	0/0	1/1	0/0	0/1	0/0	0/0	1/1
Chromosome1-2964803	INS	PRECISE	795	0	0	0/0	0/0	0/0	0/0	0/1	0/0	0/0	0/0	0/0	0/0	0/0	0/0	0/0	0/0
Chromosome1-2964880	INS	PRECISE	455	0	0.707	0/0	0/0	0/0	0/0	0/0	0/0	0/1	0/0	0/0	0/0	0/0	0/0	0/0	0/1
Chromosome1-2965025	DEL	PRECISE	326	0	0	1/1	0/1	0/0	0/0	0/0	0/0	0/0	0/0	0/1	0/1	0/1	0/1	1/1	0/1
Chromosome1-2965059	INS	PRECISE	211	0	0	0/0	0/0	0/0	0/0	0/1	0/0	0/0	0/0	0/0	0/0	0/0	0/0	0/0	0/0
Chromosome1-2965926	DEL	PRECISE	1237	0	0	0/0	0/0	0/0	0/0	0/1	0/0	0/0	0/0	0/0	0/1	0/1	0/0	0/0	1/1
Chromosome1-2966142	INS	PRECISE	1321	0.756	0.577	0/0	0/0	0/1	0/0	0/1	0/1	0/0	0/1	1/1	0/1	0/0	1/1	0/0	0/0
Chromosome1-2966195	INS	PRECISE	55	0	0	0/0	0/0	0/0	0/0	0/1	0/0	0/0	0/0	0/0	0/0	0/0	0/0	0/0	0/0
Chromosome1-2966727	INS	PRECISE	1944	0	0	0/0	0/0	0/0	0/0	0/1	0/0	0/0	0/0	0/0	0/0	0/0	0/0	0/0	0/0
Chromosome1-2966961	INS	PRECISE	1968	0	0	0/0	0/0	0/0	0/0	0/0	0/0	0/0	0/0	0/0	0/0	0/0	0/0	0/0	1/1
Chromosome1-2967900	INS	PRECISE	62	0	0	0/0	0/0	0/0	0/0	0/1	0/0	0/0	0/0	0/0	0/0	0/0	0/0	0/0	0/0
Chromosome1-2968012	INS	PRECISE	501	0	0	0/0	0/0	0/0	0/0	0/1	0/0	0/0	0/0	0/0	0/0	0/0	0/0	0/0	0/0
Chromosome1-2968094	INS	PRECISE	238	0	0.5	0/0	0/0	0/0	0/0	0/0	0/0	0/0	0/0	0/1	0/0	0/1	0/1	0/0	0/1
Chromosome1-2968119	INS	PRECISE	3907	0	0	0/0	0/0	0/0	0/0	0/1	0/0	0/0	0/0	0/0	0/0	0/0	0/0	0/0	0/0
Chromosome1-2968321	INS	PRECISE	49	0	0.354	0/1	0/1	0/0	0/0	0/0	0/0	0/0	0/0	0/1	0/1	0/1	0/1	0/1	0/1
Chromosome1-2969273	INS	PRECISE	4327	0	0	0/0	0/0	0/0	0/0	0/0	0/0	0/0	0/0	0/0	0/0	0/0	0/0	0/0	1/1
Chromosome1-2970037	INS	PRECISE	1679	0	0.707	1/1	0/1	0/0	0/0	0/0	0/0	0/0	0/0	0/0	0/0	0/0	0/0	0/0	0/0
Chromosome1-2970043	INS	PRECISE	133	0	0	0/0	0/0	0/0	0/0	0/0	0/0	0/0	0/0	0/0	0/1	0/0	0/0	0/0	0/0
Chromosome1-2970451	INS	PRECISE	138	0	0	0/0	0/0	0/0	0/0	0/0	0/0	0/0	0/0	0/0	0/0	0/0	0/0	0/1	0/0
Chromosome1-2970985	INS	PRECISE	50	0	0	0/0	0/0	0/0	0/0	0/0	0/0	0/0	0/0	0/0	0/0	0/0	0/0	0/1	0/0
Chromosome1-2972151	INS	PRECISE	365	0	0	0/0	0/0	0/0	0/0	0/0	0/0	0/0	0/0	0/0	0/0	0/0	0/1	0/0	0/0
Chromosome1-2972480	INS	PRECISE	1225	0	0	0/0	0/0	0/0	0/0	0/0	0/0	0/0	0/0	0/0	0/0	0/0	0/1	0/0	0/0
Chromosome1-2972724	INS	PRECISE	285	0	0.5	0/0	0/0	0/0	0/0	0/0	0/0	0/0	0/0	0/1	0/1	0/1	0/0	0/0	0/1
Chromosome1-2973514	DEL	PRECISE	87	0	0	0/1	0/1	0/0	0/0	0/0	0/0	0/0	0/0	0/1	0/1	0/1	0/1	0/1	0/1
Chromosome1-2973522	INS	PRECISE	1512	0	0	0/0	0/0	0/0	0/0	0/0	0/0	0/0	0/0	0/0	0/0	0/0	0/0	0/0	0/1
Chromosome1-2974735	DEL	PRECISE	979	0	0	0/0	0/0	0/0	0/0	0/0	0/0	0/0	0/0	0/0	0/0	0/0	0/0	0/0	0/1
Chromosome1-2974781	INS	PRECISE	1030	0	0.707	0/1	0/1	0/0	0/0	0/0	0/0	0/0	0/0	0/0	0/0	0/0	0/0	0/0	0/0
Chromosome1-2974917	DEL	PRECISE	57	0	0	0/0	0/0	0/0	0/0	0/0	0/0	0/0	0/0	0/0	0/0	0/0	0/0	0/0	0/1
Chromosome1-2975333	DEL	PRECISE	534	0	0	0/0	0/0	0/0	0/0	0/0	0/0	0/0	0/0	0/0	0/0	0/0	0/0	0/0	0/1
Chromosome1-2976738	INS	PRECISE	137	0	0	0/0	0/0	0/0	0/0	0/1	0/0	0/0	0/0	0/0	0/0	0/0	0/0	0/0	0/0
Chromosome1-2976745	DEL	PRECISE	569	0	0	0/0	0/0	0/0	0/0	0/0	0/0	0/0	0/0	0/0	0/0	0/0	0/0	0/1	0/0
Chromosome1-2978328	BND	PRECISE	0	0	0	0/0	0/0	0/0	0/0	0/0	0/0	0/0	0/0	0/0	0/0	0/0	0/1	0/0	0/0
Chromosome1-2978722	DEL	PRECISE	368	0	0	0/0	0/0	0/0	0/0	0/0	0/0	0/0	0/0	0/0	0/0	0/0	0/0	0/1	0/0
Chromosome1-2978835	INS	PRECISE	56	0	0	0/0	0/0	0/0	0/0	0/0	0/0	0/0	0/0	0/0	0/0	0/0	0/0	0/0	0/1
Chromosome1-2979755	DEL	PRECISE	80	0	0	0/1	0/1	0/0	0/0	0/0	0/0	0/0	0/0	0/1	0/1	0/1	0/1	0/1	0/1

Chromosome1-2981515 INS	PRECISE	189	0	0	0/0	0/0	0/0	0/0	0/0	0/0	0/0	0/0	0/0	0/1	0/0	0/0	0/0	0/0
Chromosome1-2981944 DEL	PRECISE	152	0	0	0/0	0/0	0/0	0/0	0/0	0/0	0/0	0/0	0/0	0/1	0/0	0/0	0/0	0/0
Chromosome1-2982436 DEL	PRECISE	72	0	0	0/1	0/1	0/0	0/0	0/0	0/0	0/0	0/0	0/1	0/1	0/1	0/1	0/1	0/1
Chromosome1-2982700 INS	PRECISE	163	0	0	0/0	0/0	0/0	0/0	0/0	0/0	0/0	0/0	0/0	0/0	0/0	0/0	0/0	0/1
Chromosome1-2983250 INS	PRECISE	1351	0	0	0/0	0/0	0/0	0/0	0/0	0/0	0/0	0/0	0/0	0/0	0/0	0/0	1/1	0/0
Chromosome1-2983344 INS	PRECISE	1046	0	0	0/0	0/0	0/0	0/0	0/0	0/0	0/0	0/0	0/0	0/0	0/0	0/0	0/0	0/1
Chromosome1-2983775 DEL	PRECISE	174	0	0	0/0	0/0	0/0	0/0	0/0	0/0	0/0	0/0	0/0	0/1	0/0	0/0	0/0	0/0
Chromosome1-2984233 INS	PRECISE	67	0	0.463	0/1	0/1	0/0	0/0	0/0	0/0	0/0	0/0	0/1	0/1	0/1	0/1	0/1	0/1
Chromosome1-2984569 DEL	PRECISE	56	0	0	0/0	0/0	0/0	0/0	0/0	0/0	0/0	0/0	0/0	0/0	0/0	0/1	0/0	0/0
Chromosome1-2985221 INS	PRECISE	60	0	0	0/0	0/0	0/0	0/0	0/0	0/0	0/0	0/0	0/1	0/0	0/1	0/0	0/0	0/1
Chromosome1-2985489 INS	PRECISE	348	0	0	0/0	0/0	0/0	0/0	0/1	0/0	0/0	0/0	0/0	0/0	0/0	0/0	0/0	0/0
Chromosome1-2987923 INS	PRECISE	79	0	0	0/0	0/0	0/0	0/0	0/0	0/0	0/0	0/0	0/0	0/0	0/0	0/0	0/1	0/0
Chromosome1-2988226 DEL	PRECISE	52	0	0	0/0	0/0	0/0	0/0	0/1	0/0	0/0	0/0	0/0	0/0	0/0	0/0	0/0	0/0
Chromosome1-2989743 DEL	PRECISE	624	0	0	0/0	0/0	0/1	1/1	1/1	1/1	1/1	1/1	1/1	1/1	0/0	1/1	0/0	1/1
Chromosome1-2989844 DEL	PRECISE	329	0	0	0/0	0/0	0/1	1/1	1/1	1/1	1/1	1/1	1/1	1/1	0/0	1/1	0/0	1/1
Chromosome1-2990711 DEL	PRECISE	58	0	0	0/0	0/0	0/0	0/0	0/0	0/0	0/0	0/0	0/1	0/0	0/1	0/0	0/1	0/1
Chromosome1-2991331 INS	PRECISE	1154	0	0.527	0/0	0/0	0/1	1/1	0/1	1/1	0/1	1/1	0/1	0/1	0/0	1/1	0/0	0/0
Chromosome1-2991392 INS	PRECISE	163	0	0	0/0	0/0	0/0	0/0	0/0	0/0	0/0	0/0	0/0	0/0	0/0	0/0	0/1	0/0
Chromosome1-2991619 INS	PRECISE	1115	0	0	0/0	0/0	0/0	0/0	0/0	0/0	0/0	0/0	0/0	0/0	0/0	0/0	0/0	0/1
Chromosome1-2991824 DEL	PRECISE	626	0	0	0/1	0/0	0/1	1/1	1/1	1/1	1/1	1/1	1/1	1/1	0/0	1/1	0/0	1/1
Chromosome1-2992412 INS	PRECISE	2188	0	0	0/0	0/0	0/0	0/0	0/0	0/0	0/0	0/0	0/0	0/0	0/0	0/0	0/0	1/1
Chromosome1-2992648 DEL	PRECISE	102	0	0	0/0	0/0	0/0	0/0	0/1	0/0	0/0	0/0	0/0	0/0	0/0	0/0	0/0	0/0
Chromosome1-2993950 INS	PRECISE	54	0.354	0	0/1	0/1	0/0	0/0	0/0	0/0	0/1	0/0	0/1	0/0	0/1	0/1	0/1	1/1
Chromosome1-2994102 DEL	PRECISE	123	0	0	0/1	0/1	0/0	0/0	0/0	0/0	0/0	0/0	0/1	0/0	0/1	0/1	0/1	0/1
Chromosome1-2994468 INS	PRECISE	672	0	0	0/0	0/0	0/0	0/0	0/0	0/0	0/1	0/0	0/0	0/0	0/0	0/0	0/0	0/0
Chromosome1-2994486 INS	PRECISE	1802	0	0	0/0	0/0	0/0	0/0	0/0	0/0	0/1	0/0	0/0	0/0	0/0	0/0	0/0	0/0
Chromosome1-2994527 DEL	PRECISE	4667	0	0	0/0	0/0	0/0	0/0	0/0	0/0	0/0	0/0	0/1	0/0	0/1	0/0	0/1	0/1
Chromosome1-2994562 INS	PRECISE	278	0	0	0/1	0/1	0/0	0/0	0/0	0/0	0/0	0/0	0/0	0/0	0/0	0/0	0/0	0/0
Chromosome1-2994702 DEL	PRECISE	1987	0	0	0/0	0/0	0/0	0/0	0/0	0/0	0/0	0/0	0/0	0/1	0/0	0/1	0/0	0/0
Chromosome1-2994950 DEL	PRECISE	601	0	0	0/0	0/0	0/0	0/0	0/0	0/0	0/1	0/0	0/0	0/0	0/0	0/0	0/0	0/0
Chromosome1-2995131 BND	PRECISE	0	0	0	0/0	0/0	0/0	0/0	0/0	0/0	0/0	0/0	0/0	0/1	0/0	0/0	0/0	0/0
Chromosome1-2996607 DEL	PRECISE	129	0	0	0/1	0/1	0/0	0/0	0/0	0/0	0/0	0/0	0/1	0/1	0/1	0/1	0/1	0/1
Chromosome1-2996715 DEL	PRECISE	132	0	0	0/1	0/1	0/0	0/0	0/0	0/0	0/0	0/0	0/1	0/1	0/1	0/1	0/1	0/1
Chromosome1-2997418 INS	PRECISE	1476	0	0	0/0	0/0	0/0	0/0	0/0	0/0	0/0	0/0	0/0	0/0	0/0	0/0	0/0	0/1
Chromosome1-2997975 INS	PRECISE	90	0	0	0/0	0/0	0/0	0/0	0/0	0/0	0/0	0/0	0/0	0/0	0/0	0/0	0/1	0/0
Chromosome1-2998708 DEL	PRECISE	301	0	0	0/0	0/0	0/0	0/0	0/0	0/0	0/0	0/0	0/0	0/1	0/0	0/1	0/0	0/0
Chromosome1-2998840 DEL	PRECISE	346	0	0	0/0	0/0	0/0	0/0	0/1	0/0	0/1	0/0	0/0	0/0	0/0	0/0	0/0	0/0
Chromosome1-2999170 DEL	PRECISE	486	0	0	0/1	0/1	0/0	0/0	0/0	0/0	0/0	0/0	0/1	0/1	0/0	0/0	0/0	0/1
Chromosome1-2999433 INS	PRECISE	190	0	0	0/0	0/0	0/0	0/0	0/0	0/0	0/0	0/0	0/0	0/0	0/0	0/0	0/0	0/1
Chromosome1-2999487 INS	PRECISE	659	0	0	0/0	0/0	0/0	0/0	0/0	0/0	0/0	0/0	0/0	0/0	0/0	0/0	0/1	0/0
Chromosome1-2999872 DUP	PRECISE	8693	0	0	0/0	0/0	0/0	0/0	0/0	0/0	0/0	0/0	0/0	0/0	0/0	0/0	0/1	0/0
Chromosome1-3000493 INS	PRECISE	364	0	0	0/0	0/0	0/0	0/0	0/0	0/0	0/0	0/0	0/0	0/0	0/0	0/1	0/0	0/0
Chromosome1-3000742 INS	PRECISE	775	0	0	0/0	0/0	0/0	0/0	0/1	0/0	0/0	0/0	0/0	0/0	0/0	0/0	0/0	0/0
Chromosome1-3001694 DEL	PRECISE	329	0	0	0/1	0/1	0/1	1/1	1/1	1/1	1/1	1/1	1/1	1/1	0/1	1/1	0/1	1/1
Chromosome1-3002870 DEL	PRECISE	586	0	0	0/0	0/0	0/0	0/0	0/1	0/0	0/0	0/0	0/0	0/0	0/0	0/0	0/0	0/0
Chromosome1-3003743 INS	PRECISE	8060	0	0	0/0	0/0	0/0	0/0	0/0	0/0	0/0	0/0	0/0	0/0	0/0	0/0	0/1	0/0
Chromosome1-3004412 INS	PRECISE	211	0	0	0/0	0/0	0/0	0/0	0/0	0/0	0/1	0/0	0/0	0/0	0/0	0/0	0/0	0/0
Chromosome1-3004557 INS	PRECISE	148	0	0	0/0	0/0	0/0	0/0	0/0	0/0	0/0	0/0	0/0	0/0	0/0	0/0	0/0	0/1
Chromosome1-3004856 DEL	PRECISE	106	0	0	0/1	0/1	0/0	0/0	0/0	0/0	0/0	0/0	0/0	0/0	0/0	0/0	0/0	0/0
Chromosome1-3005085 DEL	PRECISE	50	0	0	0/1	0/1	0/0	0/0	0/0	0/0	0/0	0/0	0/1	0/1	0/1	0/1	0/1	0/1
Chromosome1-3005639 DEL	PRECISE	51	0	0	0/0	0/0	0/0	0/0	0/0	0/0	0/0	0/0	0/0	0/0	0/0	0/0	0/0	0/1
Chromosome1-3006008 INS	PRECISE	161	0	0.354	0/1	0/1	0/0	0/0	0/0	0/0	0/0	0/0	0/1	0/1	0/1	0/1	0/1	0/1
Chromosome1-3006060 INS	PRECISE	1103	0	0	0/0	0/0	0/0	0/0	0/0	0/0	0/1	0/0	0/0	0/0	0/0	0/0	0/0	0/0
Chromosome1-3006353 INS	PRECISE	1016	0	0	0/0	0/0	0/0	0/0	0/0	0/0	0/0	0/0	0/0	0/0	0/0	0/0	0/0	0/1
Chromosome1-3006628 DEL	PRECISE	177	0	0	0/1	0/1	0/0	0/0	0/0	0/0	0/0	0/0	0/1	0/1	0/0	0/1	0/1	0/1
Chromosome1-3006993 DEL	PRECISE	324	0	0	0/0	0/0	0/0	0/0	0/1	0/0	0/0	0/0	0/0	0/0	0/0	0/0	0/0	0/0
Chromosome1-3007602 DEL	PRECISE	128	0	0	0/0	0/0	0/0	0/0	0/0	0/0	0/0	0/0	0/0	0/0	0/0	0/1	0/0	0/0
Chromosome1-3007643 INS	PRECISE	258	0	0	0/0	0/0	0/0	0/0	0/1	0/0	0/0	0/0	0/0	0/0	0/0	0/0	0/0	0/0
Chromosome1-3008140 INS	PRECISE	1352	0	0	0/0	0/0	0/0	0/0	0/1	0/0	0/0	0/0	0/0	0/0	0/0	0/0	0/0	0/0
Chromosome1-3008418 INS	PRECISE	53	0	0	0/0	0/0	0/0	0/0	0/0	0/0	0/0	0/0	0/0	0/1	0/0	0/0	0/0	0/0
Chromosome1-3009094 INS	PRECISE	1025	0	0	0/0	0/0	0/0	0/0	0/0	0/0	0/0	0/0	0/0	0/0	0/0	0/0	0/0	0/1
Chromosome1-3011785 DEL	PRECISE	102	0	0	0/0	0/0	0/0	1/1	0/0	0/1	0/1	1/1	0/0	0/0	0/0	0/0	0/0	0/0
Chromosome1-3012273 DEL	PRECISE	86	0	0	0/0	0/0	0/0	0/0	0/1	0/0	0/0	0/0	0/0	0/0	0/0	0/0	0/0	0/0
Chromosome1-3012458 INS	PRECISE	6126	0	0	0/0	0/0	0/0	0/0	0/0	0/0	0/0	0/0	0/0	0/0	0/0	0/0	1/1	0/0
Chromosome1-3013581 INS	PRECISE	57	0	0	0/1	0/1	0/1	1/1	1/1	1/1	0/0	1/1	1/1	1/1	0/1	1/1	0/1	1/1
Chromosome1-3013817 INS	PRECISE	4015	0	0	0/0	0/0	0/0	0/0	0/0	0/0	0/0	0/0	0/0	0/0	0/0	1/1	0/0	0/0
Chromosome1-3014377 INS	PRECISE	3380	0	0	0/0	0/0	0/0	0/0	1/1	0/0	0/0	0/0	0/0	0/0	0/0	0/0	0/0	0/0
Chromosome1-3016170 DEL	PRECISE	934	0	0	0/0	0/0	0/0	0/0	0/0	0/0	0/0	0/0	0/0	0/0	0/0	0/0	0/1	0/0
Chromosome1-3016298 INS	PRECISE	7253	0	0	0/0	0/0	0/0	0/0	0/0	0/0	0/0	0/0	0/0	0/0	0/0	0/0	0/0	1/1
Chromosome1-3016365 INS	PRECISE	161	0	0	0/0	0/0	0/0	0/0	0/1	0/0	0/0	0/0	0/0	0/0	0/0	0/0	0/0	0/0
Chromosome1-3016760 INS	PRECISE	203	0	0	0/0	0/0	0/0	0/0	0/0	0/0	0/0	0/0	0/0	0/0	0/0	0/0	0/0	1/1
Chromosome1-3016986 DEL	PRECISE	472	0.378	0.378	0/0	0/0	0/1	0/0	0/1	0/1	0/0	0/1	1/1	1/1	0/0	1/1	0/0	0/0
Chromosome1-3017750 INS	PRECISE	80	0	0.463	0/1	0/1	0/0	0/0	0/0	0/0	0/0	0/0	0/1	0/1	0/0	0/1	0/1	0/1

Chromosome1-3020253	DEL	PRECISE	53	0	0	0/1	0/1	0/0	0/0	0/0	0/0	0/0	0/0	0/1	0/1	0/1	1/1	0/1	0/1
Chromosome1-3020412	DEL	PRECISE	995	0	0	0/1	0/1	0/1	1/1	1/1	1/1	0/1	1/1	1/1	1/1	0/1	1/1	0/1	1/1
Chromosome1-3020957	DEL	PRECISE	54	0	0	0/1	0/1	0/0	0/0	0/0	0/0	0/0	0/0	0/0	0/0	0/0	0/0	0/0	0/0
Chromosome1-3021766	INS	PRECISE	1148	0	0.744	0/1	0/1	0/0	0/0	0/0	0/0	0/0	0/0	0/1	0/1	0/1	0/1	1/1	0/1
Chromosome1-3022067	INS	PRECISE	56	0	0	0/0	0/0	0/0	0/0	0/1	0/0	0/0	0/0	0/0	0/0	0/0	0/0	0/0	0/0
Chromosome1-3022183	DEL	PRECISE	1263	0	0	0/0	0/0	0/0	0/0	0/0	0/0	0/0	0/0	0/1	0/1	0/1	0/1	0/1	0/1
Chromosome1-3022204	INS	PRECISE	622	0	0.707	0/1	0/1	0/0	0/0	0/0	0/0	0/0	0/0	0/0	0/0	0/0	0/0	0/0	0/0
Chromosome1-3023724	DEL	PRECISE	140	0	0	0/0	0/0	0/0	0/0	0/0	0/0	0/0	0/0	0/0	0/0	0/0	0/0	0/0	0/1
Chromosome1-3024257	DEL	PRECISE	289	0	0	0/1	0/1	0/0	0/0	0/0	0/0	0/0	0/0	0/0	0/0	0/0	0/0	0/0	0/0
Chromosome1-3024656	INS	PRECISE	879	0	0	0/0	0/0	0/0	0/0	0/1	0/0	0/0	0/0	0/0	0/0	0/0	0/0	0/0	0/0
Chromosome1-3024730	INS	PRECISE	379	0	0.641	0/1	0/1	0/0	0/0	0/0	0/0	0/0	0/0	0/1	0/0	0/1	0/1	0/1	0/1
Chromosome1-3025318	INS	PRECISE	1825	0	0	0/0	0/0	0/0	0/0	0/1	0/0	0/0	0/0	0/0	0/0	0/0	0/0	0/0	0/0
Chromosome1-3025361	INS	PRECISE	3279	0	0	0/0	0/0	0/0	0/0	0/0	0/0	0/0	0/0	0/0	0/0	0/0	0/0	0/1	0/0
Chromosome1-3025828	INS	PRECISE	139	0	0.707	0/0	0/0	0/0	0/0	0/1	0/0	0/0	0/0	0/0	0/0	0/0	0/0	0/0	1/1
Chromosome1-3025828	DEL	PRECISE	530	0	0	1/1	1/1	0/0	0/0	0/0	0/0	0/0	0/0	0/1	0/1	0/1	0/1	0/1	1/1
Chromosome1-3026884	INS	PRECISE	182	0	0	0/0	0/0	0/0	0/0	0/0	0/0	0/0	0/0	0/0	0/0	0/0	0/0	0/0	0/1
Chromosome1-3027583	DEL	PRECISE	288	0	0	0/1	0/1	0/0	0/0	0/0	0/0	0/0	0/0	0/1	0/1	0/1	0/1	0/1	1/1
Chromosome1-3027890	INS	PRECISE	462	0	0.916	0/1	0/1	0/0	0/0	0/0	0/0	0/0	0/0	0/1	0/1	0/1	0/1	0/1	1/1
Chromosome1-3028426	INS	PRECISE	57	0	0	0/0	0/0	0/0	0/0	0/0	0/0	0/0	0/0	0/0	0/0	0/0	0/0	0/1	0/0
Chromosome1-3028428	DEL	PRECISE	88	0	0	0/1	0/1	0/0	0/0	0/0	0/0	0/0	0/0	0/1	0/1	0/1	0/1	0/0	0/1
Chromosome1-3028790	DEL	PRECISE	146	0	0	0/0	0/0	0/0	0/0	0/0	0/0	0/0	0/0	0/0	0/1	0/0	0/1	0/0	0/0
Chromosome1-3028818	INS	PRECISE	1167	0	0	0/0	0/0	0/0	0/0	0/1	0/0	0/0	0/0	0/0	0/0	0/0	0/0	0/0	0/0
Chromosome1-3029043	INS	PRECISE	10992	0	0	0/0	0/0	0/0	0/0	0/1	0/0	0/0	0/0	0/0	0/0	0/0	0/0	0/0	0/0
Chromosome1-3029043	BND	PRECISE	0	0	0	0/0	0/0	0/0	0/0	0/1	0/0	0/0	0/0	0/0	0/0	0/0	0/0	0/0	0/0
Chromosome1-3029062	DEL	PRECISE	840	0	0	0/0	0/0	0/0	1/1	0/0	0/1	0/1	0/0	0/0	0/0	0/0	0/0	0/0	0/0
Chromosome1-3029286	INS	PRECISE	2587	0	0.707	1/1	1/1	0/0	0/0	0/0	0/0	0/0	0/0	0/0	0/0	0/0	0/0	0/0	0/0
Chromosome1-3029704	INS	PRECISE	53	0	0	0/0	0/0	0/0	1/1	0/0	0/1	0/1	0/1	0/0	0/0	0/0	0/0	0/0	0/0
Chromosome1-3030026	INS	PRECISE	105	0	0.5	0/0	0/0	0/0	1/1	0/0	0/1	0/1	0/1	0/0	0/0	0/0	0/0	0/0	0/0
Chromosome1-3031168	DEL	PRECISE	76	0	0	0/1	0/1	0/0	0/0	0/0	0/0	0/0	0/0	0/1	0/1	0/1	0/1	0/1	0/1
Chromosome1-3031258	INS	PRECISE	1377	0	0	0/1	0/1	0/0	0/0	0/0	0/0	0/0	0/0	0/0	0/0	0/0	0/0	0/0	0/0
Chromosome1-3031582	DEL	PRECISE	52	0	0	0/0	0/0	0/0	0/0	0/0	0/0	0/0	0/0	0/0	0/1	0/0	0/1	0/0	0/0
Chromosome1-3031959	INS	PRECISE	939	0	0	0/0	0/0	0/0	0/0	0/0	0/0	0/0	0/0	0/0	0/0	0/0	0/0	0/0	0/1
Chromosome1-3033440	DEL	PRECISE	131	0.667	0	0/1	0/1	0/0	0/0	0/1	0/0	0/0	0/0	0/1	0/1	0/1	0/1	0/1	1/1
Chromosome1-3033553	DEL	PRECISE	535	0	0	0/0	0/0	0/0	0/0	0/0	0/0	0/0	0/0	0/0	0/1	0/0	0/1	0/0	0/0
Chromosome1-3034080	DEL	PRECISE	72	0	0	0/0	0/0	0/0	0/0	0/1	0/0	0/0	0/0	0/0	0/0	0/0	0/0	0/0	0/0
Chromosome1-3035043	INS	PRECISE	278	0	0.756	0/1	0/1	0/0	0/0	0/0	0/0	0/0	0/0	0/1	0/1	0/1	0/0	0/1	0/1
Chromosome1-3035303	INS	PRECISE	100	0	0	0/0	0/0	0/0	0/0	0/1	0/0	0/0	0/0	0/0	0/0	0/0	0/0	0/0	0/0
Chromosome1-3035704	INS	PRECISE	150	0	0	0/0	0/0	0/0	0/0	0/1	0/0	0/0	0/0	0/0	0/0	0/0	0/0	0/0	0/0
Chromosome1-3036025	DEL	PRECISE	139	0	0	0/0	0/0	0/0	0/0	0/0	0/0	0/0	0/0	0/0	0/1	0/0	0/1	0/0	0/0
Chromosome1-3036684	INS	PRECISE	9176	0	0	0/0	0/0	0/0	0/0	0/0	0/0	0/0	0/0	0/0	0/0	0/0	0/0	0/0	1/1
Chromosome1-3036685	BND	PRECISE	0	0	0	0/0	0/0	0/0	0/0	0/0	0/0	0/0	0/0	0/0	0/0	0/0	0/0	0/0	0/1
Chromosome1-3036821	INS	PRECISE	1139	0	0	0/0	0/0	0/0	0/0	0/0	0/0	0/0	0/0	0/0	1/1	0/0	1/1	0/0	0/0
Chromosome1-3037009	INS	PRECISE	134	0	0	0/0	0/0	0/0	0/0	0/0	0/0	0/0	0/0	0/0	0/0	0/0	0/0	0/0	1/1
Chromosome1-3037557	DEL	PRECISE	831	0	0	0/1	0/1	0/0	0/0	0/0	0/0	0/0	0/0	0/1	0/1	0/1	0/1	0/1	0/1
Chromosome1-3037594	DEL	PRECISE	105	0	0	0/0	0/0	0/0	0/0	0/1	0/0	0/0	0/0	0/0	0/0	0/0	0/0	0/0	0/0
Chromosome1-3038022	INS	PRECISE	63	0	0	0/1	0/1	0/0	0/0	0/0	0/0	0/0	0/0	0/1	0/1	0/1	0/1	0/1	0/1
Chromosome1-3039137	INS	PRECISE	201	0	0.378	0/0	0/1	0/0	0/0	0/0	0/0	0/0	0/0	0/1	0/1	0/1	0/0	0/1	0/1
Chromosome1-3039314	DEL	PRECISE	65	0	0	0/0	0/0	0/0	0/0	0/0	0/0	0/0	0/0	0/1	0/0	0/1	0/1	0/1	0/1
Chromosome1-3040090	DEL	PRECISE	854	0	0	0/0	0/0	0/0	0/0	0/0	0/0	0/0	0/0	0/0	0/0	0/0	0/0	0/0	0/1
Chromosome1-3040489	INS	PRECISE	50	0	0.354	0/1	0/1	0/0	0/0	0/0	0/0	0/0	0/0	0/1	0/1	0/1	0/1	0/1	0/1
Chromosome1-3040820	INS	PRECISE	5847	0	0	0/0	0/0	0/0	0/0	1/1	0/0	0/0	0/0	0/0	0/0	0/0	0/0	0/0	0/0
Chromosome1-3042267	DEL	PRECISE	70	0	0	0/1	0/1	0/0	0/0	0/0	0/0	0/0	0/0	0/0	0/0	0/0	0/1	0/0	0/0
Chromosome1-3042334	DEL	PRECISE	5314	0	0	0/1	1/1	0/0	0/0	0/0	0/0	0/0	0/0	0/1	0/0	1/1	0/0	1/1	0/1
Chromosome1-3042777	DEL	PRECISE	2148	0	0	0/0	0/0	0/0	0/0	0/1	0/0	0/0	0/0	0/0	0/0	0/0	0/0	0/0	0/0
Chromosome1-3043019	DEL	PRECISE	337	0	0	0/1	0/1	0/0	0/0	0/0	0/0	0/0	0/0	0/1	0/0	0/1	0/0	1/1	0/1
Chromosome1-3044082	INS	PRECISE	212	0	0	0/0	0/0	0/0	0/0	0/0	0/0	0/0	0/0	0/0	0/0	0/0	0/0	0/0	0/1
Chromosome1-3044415	INS	PRECISE	206	0	0	0/0	0/0	0/0	0/0	0/1	0/0	0/0	0/0	0/0	0/0	0/0	0/0	0/0	0/0
Chromosome1-3045504	DEL	PRECISE	89	0	0	0/0	0/0	0/0	0/0	0/1	0/0	0/0	0/0	0/0	0/0	0/0	0/0	0/0	0/0
Chromosome1-3045957	INS	PRECISE	137	0	0	0/0	0/0	0/0	0/0	0/0	0/0	0/0	0/0	0/0	0/0	0/0	0/0	0/0	1/1
Chromosome1-3046005	INS	PRECISE	861	0	0	0/0	0/0	0/0	0/0	0/0	0/0	0/0	0/0	0/0	0/0	0/0	0/0	0/0	1/1
Chromosome1-3046083	INS	PRECISE	233	0.976	0.976	0/1	0/0	0/0	0/0	0/0	0/0	0/0	0/0	0/1	0/1	0/1	0/1	0/1	0/0
Chromosome1-3046290	INS	PRECISE	56	0	0	0/0	0/0	0/0	0/0	0/1	0/0	0/0	0/0	0/0	0/0	0/0	0/0	0/0	0/0
Chromosome1-3047381	INS	PRECISE	1298	0	0	0/0	0/0	0/0	0/0	0/0	0/0	0/0	0/0	0/0	0/0	0/0	0/0	0/0	0/1
Chromosome1-3047858	DEL	PRECISE	63	0	0	0/0	0/0	0/0	0/0	0/0	0/0	0/0	0/0	0/1	0/0	0/1	0/0	0/1	0/1
Chromosome1-3048342	DEL	PRECISE	904	0	0	0/0	0/0	0/0	0/0	0/0	0/0	0/0	0/0	0/0	0/0	0/0	0/0	0/0	0/1
Chromosome1-3048692	DEL	PRECISE	55	0	0	0/0	0/0	0/0	0/0	0/0	0/0	0/0	0/0	0/0	0/1	0/0	0/0	0/0	0/0
Chromosome1-3049268	DEL	PRECISE	117	0.447	0.447	0/0	0/0	0/0	0/0	0/0	0/0	0/0	0/0	0/0	0/0	0/1	0/0	0/1	0/1
Chromosome1-3049286	INS	PRECISE	137	0	0	0/1	0/1	0/0	0/0	0/0	0/0	0/0	0/0	0/0	0/0	0/0	0/1	0/0	0/0
Chromosome1-3049317	BND	PRECISE	0	0	0	0/0	0/0	0/0	0/0	0/0	0/0	0/0	0/0	0/1	0/1	0/1	0/0	0/0	0/0
Chromosome1-3050687	DEL	PRECISE	156	0	0	0/1	0/0	0/0	0/0	0/0	0/0	0/0	0/0	0/0	0/0	0/0	0/0	0/0	0/0
Chromosome1-3051056	INS	PRECISE	711	0	0.548	0/0	0/0	0/0	1/1	0/0	1/1	0/1	0/1	0/0	0/0	0/0	0/0	0/0	0/1
Chromosome1-3051058	DEL	PRECISE	336	0	0	0/1	0/1	0/0	0/0	0/1	0/0	0/0	0/0	0/1	0/0	0/0	0/0	0/1	0/1
Chromosome1-3051879	DEL	PRECISE	2382	0	0	0/0	0/0	0/0	0/0	0/0	0/0	0/0	0/0	0/0	0/1	0/0	0/0	0/0	0/0

Chromosome1-3051985	BND	PRECISE	0	0	0	0/0	0/0	0/0	0/1	0/0	0/0	0/0	0/1	0/0	0/0	0/0	0/0	0/0	0/0
Chromosome1-3052063	INS	PRECISE	1167	0	0	0/0	0/0	0/0	0/0	0/1	0/0	0/0	0/0	0/0	0/0	0/0	0/0	0/0	0/0
Chromosome1-3052119	DEL	PRECISE	376	0	0	0/0	0/0	0/0	0/0	0/0	0/0	0/0	0/0	0/0	0/1	0/0	0/0	0/0	0/0
Chromosome1-3052568	DEL	PRECISE	65	0	0	0/0	0/0	0/0	1/1	0/0	0/1	0/1	0/1	0/0	0/0	0/0	0/0	0/0	0/0
Chromosome1-3052875	DEL	PRECISE	82	0	0	0/0	0/0	0/0	1/1	0/0	0/1	0/1	0/1	0/0	0/0	0/0	0/0	0/0	0/1
Chromosome1-3053584	DEL	PRECISE	3741	0	0	0/0	0/0	0/0	0/0	0/1	0/0	0/0	0/0	0/0	0/0	0/0	0/0	0/0	0/0
Chromosome1-3054139	DEL	PRECISE	109	0	0	0/0	0/0	0/0	0/0	0/1	0/0	0/0	0/0	0/0	0/0	0/0	0/0	0/0	0/0
Chromosome1-3054595	DEL	PRECISE	189	0	0	0/0	0/0	0/0	1/1	0/0	0/1	0/1	0/0	0/0	0/0	0/0	0/0	0/0	0/1
Chromosome1-3056033	DEL	PRECISE	182	0	0	0/0	0/0	0/0	1/1	0/0	0/0	0/0	0/0	0/0	0/0	0/0	0/0	0/0	0/0
Chromosome2-54756	DEL	PRECISE	124	0.756	0.378	0/0	1/1	0/1	0/1	0/0	0/0	0/1	1/1	0/0	0/0	1/1	0/0	0/0	1/1
Chromosome2-54924	BND	PRECISE	0	0	0	0/0	0/0	0/0	0/0	0/0	0/1	0/0	0/0	0/1	0/0	0/0	0/0	0/0	0/0
Chromosome2-57435	DEL	PRECISE	403	0	0	0/0	0/0	0/0	0/0	0/0	0/0	0/0	0/0	0/0	0/0	0/0	0/0	0/0	1/1
Chromosome2-65118	DEL	PRECISE	173	0	0	0/0	0/0	0/0	0/1	0/0	0/0	0/0	0/0	0/0	0/0	0/0	0/0	0/0	0/0
Chromosome2-77567	DEL	PRECISE	3449	0	0	0/0	0/0	0/0	0/1	0/0	0/0	0/1	0/0	0/0	0/0	0/0	0/0	0/0	0/0
Chromosome2-80710	DEL	PRECISE	2087	0	0	0/0	0/0	0/0	0/0	0/0	0/0	0/0	0/0	0/0	0/0	0/0	0/0	0/0	0/1
Chromosome2-85794	DEL	PRECISE	1786	0	0	0/0	0/0	0/0	0/0	0/0	0/0	0/0	0/0	0/0	0/0	0/1	0/1	0/1	0/0
Chromosome2-97699	INS	PRECISE	67	0	0	0/0	0/0	0/0	0/0	0/1	0/0	0/0	0/0	0/0	0/0	0/0	0/0	0/0	0/0
Chromosome2-129700	DEL	PRECISE	2619	0	0	0/0	0/0	0/0	0/0	0/0	0/0	0/0	0/0	0/0	0/0	1/1	1/1	1/1	0/0
Chromosome2-130916	INS	PRECISE	4615	0	0	0/0	0/0	0/0	0/0	0/0	0/0	0/0	0/0	0/0	0/0	0/0	0/0	0/0	1/1
Chromosome2-142406	INS	PRECISE	827	0	0	0/0	0/0	0/0	0/0	0/0	0/0	0/0	0/0	0/0	0/0	0/0	0/0	0/1	0/0
Chromosome2-147637	INS	PRECISE	1454	0	0	0/0	0/0	0/0	0/0	0/0	0/0	0/0	0/0	0/0	0/0	0/0	0/0	1/1	0/0
Chromosome2-149477	INS	PRECISE	1007	0	0	0/0	0/0	0/0	0/0	0/0	0/0	0/0	0/0	0/0	0/0	0/0	0/0	1/1	0/0
Chromosome2-153106	DEL	PRECISE	450	0	0	0/0	0/0	0/0	0/0	0/0	0/0	0/0	0/0	0/0	0/0	0/0	0/0	0/1	0/0
Chromosome2-156376	INS	PRECISE	5198	0	0.707	0/0	0/0	0/0	0/0	0/0	0/0	0/0	0/0	0/0	0/0	1/1	1/1	0/0	0/0
Chromosome2-158700	INS	PRECISE	65	0	0	0/0	0/0	0/0	0/0	0/0	0/0	0/0	0/0	0/0	0/0	0/1	0/1	0/1	0/0
Chromosome2-173336	INS	PRECISE	52	0	0	0/0	0/0	0/0	0/0	0/0	0/0	0/0	0/0	0/0	0/0	0/1	0/1	0/1	0/0
Chromosome2-185200	DEL	PRECISE	227	0	0	0/0	0/0	0/0	0/0	0/0	0/0	0/0	0/0	0/0	0/0	0/1	0/1	0/1	0/0
Chromosome2-186243	DEL	PRECISE	74	0	0	0/0	0/0	0/0	0/0	0/1	0/0	0/0	0/0	0/0	0/0	0/0	0/0	0/1	0/0
Chromosome2-206757	INS	PRECISE	56	0	0	0/0	0/0	0/0	0/0	0/0	0/0	0/0	0/0	0/0	0/0	0/0	0/0	0/1	0/0
Chromosome2-209185	INS	PRECISE	8836	0	0	0/0	0/0	0/0	0/0	0/0	0/0	0/0	0/0	0/0	0/0	0/0	0/0	0/0	0/0
Chromosome2-209450	DEL	PRECISE	220	0	0	0/0	0/0	0/0	0/0	0/0	0/0	0/0	0/0	0/0	0/0	0/1	0/1	0/0	0/0
Chromosome2-215086	DEL	PRECISE	72	0	0	0/0	0/0	0/0	0/0	0/0	0/0	0/0	0/0	0/0	0/0	0/0	0/0	0/0	0/1
Chromosome2-215176	INS	PRECISE	2182	0	0	0/0	0/0	0/0	0/0	0/0	0/0	0/0	0/0	0/0	0/0	0/0	0/0	0/1	0/0
Chromosome2-219833	INS	PRECISE	241	0	0.577	0/0	0/0	0/0	0/0	0/0	0/0	0/0	0/0	0/0	0/0	0/1	0/1	0/1	0/0
Chromosome2-220743	DEL	PRECISE	151	0	0	0/0	0/0	0/0	0/0	0/0	0/0	0/0	0/0	0/0	0/0	0/1	0/1	0/1	0/0
Chromosome2-234726	DEL	PRECISE	430	0	0	0/0	0/0	0/0	0/0	0/0	0/0	0/0	0/0	0/0	0/0	0/1	0/1	0/1	0/0
Chromosome2-237912	INS	PRECISE	52	0	0	0/0	0/0	0/0	0/0	0/0	0/0	0/0	0/0	0/0	0/0	0/1	0/1	0/0	0/0
Chromosome2-239370	DEL	PRECISE	372	0	0	0/0	0/0	0/0	0/0	1/1	0/0	0/0	0/0	0/0	0/0	0/1	0/1	0/1	0/0
Chromosome2-245704	DEL	PRECISE	150	0	0	0/0	0/0	0/0	0/0	0/0	0/0	0/0	0/0	0/0	0/0	0/1	0/1	0/0	0/0
Chromosome2-247390	DUP	PRECISE	8502	0	0	0/0	0/0	0/0	0/0	0/1	0/0	0/0	0/0	0/0	0/0	0/0	0/0	0/0	0/0
Chromosome2-250130	INV	PRECISE	1608	0	0	0/0	0/0	0/0	0/0	0/1	0/0	0/0	0/0	0/0	0/0	0/0	0/0	0/0	0/0
Chromosome2-250382	DEL	PRECISE	1544	0	0	1/1	0/0	0/1	1/1	0/1	0/1	1/1	0/0	0/0	0/0	0/0	0/0	0/0	0/0
Chromosome2-250836	DEL	PRECISE	903	0	0	0/0	0/0	0/0	0/0	1/1	0/0	0/0	0/0	0/0	0/0	0/0	0/0	0/0	0/0
Chromosome2-253134	INS	PRECISE	7888	0	0	0/0	0/0	0/0	0/0	0/1	0/0	0/0	0/0	0/0	0/0	0/0	0/0	0/0	0/0
Chromosome2-255951	INS	PRECISE	669	0	0	0/0	0/0	0/0	0/0	0/0	0/0	0/0	0/0	0/0	0/0	0/0	0/0	0/1	0/0
Chromosome2-256762	DEL	PRECISE	970	0	0	0/0	0/0	0/0	0/0	0/0	0/0	0/0	0/0	0/0	0/0	0/0	0/0	0/0	0/1
Chromosome2-257480	DEL	PRECISE	324	0	0	0/0	0/0	0/0	0/1	0/0	0/0	0/0	0/0	0/0	0/0	0/0	0/0	0/0	0/0
Chromosome2-262973	INS	PRECISE	211	0	0.707	0/0	0/0	0/0	0/0	0/0	0/0	0/0	0/0	0/0	0/0	0/1	0/1	0/0	0/0
Chromosome2-269513	INS	PRECISE	96	0	0	0/0	0/0	0/0	0/0	0/0	0/0	0/0	0/0	0/0	0/0	0/1	0/1	0/0	0/0
Chromosome2-275157	INS	PRECISE	399	0	0	0/0	0/0	0/0	0/0	0/0	0/0	0/0	0/0	0/0	0/0	0/0	0/0	0/1	0/0
Chromosome2-280610	DEL	PRECISE	149	0	0	0/0	0/0	0/0	0/0	0/0	0/0	0/0	0/0	0/0	0/0	0/0	0/0	0/1	0/0
Chromosome2-285833	INS	PRECISE	6137	0	0	0/0	0/0	0/0	0/0	0/0	0/0	0/0	0/0	0/0	0/0	0/0	0/0	0/0	0/1
Chromosome2-287614	INS	PRECISE	5373	0	0	0/0	0/0	0/0	1/1	0/0	0/0	0/0	0/0	0/0	0/0	0/0	0/0	0/0	0/0
Chromosome2-287619	BND	PRECISE	0	0	0	0/0	0/0	0/0	0/1	0/0	0/0	0/0	0/0	0/0	0/0	0/0	0/0	0/0	0/0
Chromosome2-298409	INS	PRECISE	879	0	0	0/0	0/0	0/0	0/0	0/0	0/0	1/1	0/0	0/0	0/0	0/0	0/0	0/0	0/0
Chromosome2-302761	INS	PRECISE	150	0	0	0/0	0/0	0/0	0/0	0/1	0/0	0/0	0/0	0/0	0/0	0/0	0/0	0/0	0/0
Chromosome2-317583	INS	PRECISE	372	0	0	0/0	0/0	0/0	0/0	0/0	0/0	0/0	0/0	0/0	0/0	0/0	0/0	0/0	0/1
Chromosome2-318717	DEL	PRECISE	99	0	0	0/1	0/0	0/1	0/1	0/0	0/0	0/0	0/0	0/0	0/0	0/0	0/0	0/0	0/0
Chromosome2-322036	INS	PRECISE	316	0	0.707	0/0	0/0	0/0	0/0	0/0	0/0	0/0	0/0	0/0	0/0	0/1	0/1	0/0	0/0
Chromosome2-354351	DEL	PRECISE	73	0	0	0/0	0/0	0/0	0/0	0/0	0/0	0/0	0/0	0/0	0/0	0/1	0/1	0/1	0/0
Chromosome2-356128	DEL	PRECISE	380	0	0	0/0	0/0	0/0	0/0	0/0	0/0	0/0	0/0	0/0	0/0	0/0	0/0	0/0	0/1
Chromosome2-358452	INS	PRECISE	888	0	0	0/0	0/0	0/0	0/0	0/0	0/0	0/0	0/0	0/0	0/0	0/0	0/0	0/1	0/0
Chromosome2-361465	INS	PRECISE	135	0	0	0/0	0/0	0/0	0/0	0/0	0/0	0/0	0/0	0/0	0/0	0/0	0/0	0/0	0/1
Chromosome2-365831	INS	PRECISE	66	0	0	0/0	0/0	0/0	0/0	0/0	0/0	0/0	0/0	0/0	0/0	0/0	0/0	0/1	0/0
Chromosome2-378973	INS	PRECISE	198	0	0.707	0/0	0/0	0/0	0/0	0/0	0/0	0/0	0/0	0/0	0/0	0/1	0/1	0/0	0/0
Chromosome2-380482	INS	PRECISE	935	0	0	0/0	0/0	0/0	0/0	0/0	0/0	0/0	0/0	0/0	0/0	0/0	0/0	0/1	0/0
Chromosome2-393233	INS	PRECISE	9551	0	0	0/0	0/0	0/0	0/0	0/0	0/0	0/0	0/0	0/0	0/0	0/0	0/0	0/1	0/0
Chromosome2-400448	DEL	PRECISE	61	0	0	0/0	0/0	0/1	1/1	1/1	0/1	0/1	0/0	0/0	0/0	0/1	0/1	0/1	0/1
Chromosome2-403527	DEL	PRECISE	995	0	0	0/0	0/0	0/1	1/1	1/1	0/1	0/1	0/0	0/0	0/0	0/1	0/1	0/1	0/1
Chromosome2-419866	INS	PRECISE	82	0	0	0/0	0/0	0/0	0/0	0/0	0/0	0/0	0/0	0/0	0/0	0/1	0/1	0/1	0/0
Chromosome2-423404	DEL	PRECISE	243	0.894	0.447	0/0	0/0	0/1	1/1	0/1	0/1	0/1	0/0	0/0	0/0	0/0	0/0	0/0	0/0
Chromosome2-431012	DEL	PRECISE	137	0	0	0/0	0/0	0/0	0/0	0/0	0/0	0/0	0/0	0/0	0/0	0/1	0/1	0/0	0/0
Chromosome2-434031	DEL	PRECISE	157	0	0	0/0	0/0	0/0	0/0	0/0	0/0	0/0	0/0	0/0	0/0	0/0	0/1	0/1	0/0

Chromosome2-438026	INS	PRECISE	1553	0	0	0/0	0/0	0/0	0/1	0/0	0/0	0/0	0/0	0/0	0/0	0/0	0/0	0/0	0/0
Chromosome2-438528	INS	PRECISE	997	0	0.707	0/0	0/0	0/1	0/0	0/1	0/0	0/0	0/0	0/0	0/0	0/0	0/0	0/0	0/0
Chromosome2-449033	DEL	PRECISE	174	0	0	0/0	0/0	0/0	0/0	0/0	0/0	0/0	0/0	0/0	0/0	0/1	0/1	0/1	0/0
Chromosome2-450173	INS	PRECISE	454	0	0	0/0	0/0	0/0	0/0	0/0	0/0	0/0	0/0	0/0	0/0	0/1	0/1	0/1	0/0
Chromosome2-450792	DEL	PRECISE	91	0	0	0/0	0/0	0/0	0/0	0/0	0/0	0/0	0/0	0/0	0/0	0/0	0/1	0/0	0/0
Chromosome2-451550	INS	PRECISE	52	0	0	0/0	0/0	0/0	0/0	0/1	0/0	0/0	0/0	0/0	0/0	0/0	0/0	0/0	0/0
Chromosome2-464584	INS	PRECISE	466	0	0	0/0	0/0	0/0	0/0	0/1	0/0	0/0	0/0	0/0	0/0	0/0	0/0	0/0	0/0
Chromosome2-467472	DEL	PRECISE	1390	0	0	0/0	0/0	0/1	1/1	1/1	0/1	0/1	0/0	0/0	0/0	0/0	0/0	0/0	0/1
Chromosome2-469754	INS	PRECISE	4757	0	0	0/0	0/0	0/0	0/0	0/0	0/0	0/0	0/0	0/0	0/0	0/0	1/1	0/0	0/0
Chromosome2-471061	DEL	PRECISE	557	0	0	0/0	0/0	0/0	0/0	0/1	0/0	0/0	0/0	0/0	0/0	0/1	0/1	0/1	0/1
Chromosome2-474762	INS	PRECISE	2893	0	0	0/0	0/0	1/1	0/0	0/0	0/0	0/0	0/0	0/0	0/0	0/0	0/0	0/0	0/0
Chromosome2-482277	DEL	PRECISE	493	0	0	0/0	0/0	0/0	0/0	0/0	0/0	0/0	0/0	0/0	0/0	0/0	0/1	0/0	0/0
Chromosome2-484217	INS	PRECISE	364	0	0	0/0	0/0	0/0	0/0	0/0	0/0	0/0	0/0	0/0	0/0	0/0	0/1	0/0	0/0
Chromosome2-494452	DEL	PRECISE	77	0	0	0/0	0/0	0/0	0/0	0/1	0/0	0/0	0/0	0/0	0/0	0/0	0/0	0/0	0/0
Chromosome2-502537	INS	PRECISE	629	0	0	0/0	0/0	0/0	0/0	0/0	0/0	0/0	0/0	0/0	0/0	0/0	0/0	0/0	0/1
Chromosome2-513721	BND	PRECISE	0	0	0	0/0	0/0	0/0	0/0	0/0	0/0	0/0	0/0	0/0	0/0	0/1	0/1	0/1	0/0
Chromosome2-522634	DEL	PRECISE	70	0	0	0/0	0/0	0/0	0/0	0/0	0/0	0/0	0/0	0/0	0/0	0/1	0/1	0/1	0/0
Chromosome2-523753	INS	PRECISE	150	0	0	0/0	0/0	0/0	0/0	0/0	0/0	0/0	0/0	0/0	0/0	0/0	0/0	0/0	0/1
Chromosome2-531266	DUP	PRECISE	23172	0	0	0/0	0/0	0/0	0/0	0/0	0/0	0/0	0/0	0/0	0/0	0/0	0/0	0/0	0/1
Chromosome2-532547	DEL	PRECISE	1108	0	0	0/0	0/0	0/0	0/0	0/0	0/0	0/0	0/0	0/0	0/0	0/1	0/1	0/1	0/0
Chromosome2-545356	DEL	PRECISE	18094	0	0	0/0	0/0	0/0	0/0	0/1	0/1	0/0	0/0	0/0	0/0	0/0	0/0	0/0	0/0
Chromosome2-545805	DEL	PRECISE	2471	0	0	0/0	0/0	0/0	0/0	0/0	0/0	0/0	0/0	0/0	0/0	0/0	0/0	0/0	0/1
Chromosome2-547317	DEL	PRECISE	1170	0	0	0/0	0/0	0/0	0/0	0/1	0/0	0/0	0/0	0/0	0/0	0/0	0/0	0/0	0/0
Chromosome2-556992	DEL	PRECISE	1187	0	0	0/0	0/0	0/1	0/0	0/1	0/0	1/1	0/0	0/0	0/0	0/0	0/0	0/0	0/0
Chromosome2-559504	DEL	PRECISE	2027	0	0	0/0	0/0	0/0	0/0	0/0	0/0	0/1	0/0	0/0	0/0	0/0	0/0	0/0	0/0
Chromosome2-561546	DEL	PRECISE	5562	0	0	0/0	0/0	0/1	0/0	0/0	0/0	0/1	0/0	0/0	0/0	0/0	0/0	0/0	0/0
Chromosome2-562106	DEL	PRECISE	2415	0	0	0/0	0/0	0/0	0/0	0/1	0/0	0/1	0/0	0/0	0/0	0/0	0/0	0/0	0/0
Chromosome2-571870	DEL	PRECISE	1170	0	0	0/0	0/0	0/0	0/0	0/1	0/0	0/0	0/0	0/0	0/0	0/0	0/0	0/0	0/0
Chromosome2-572882	DEL	PRECISE	7851	0	0	0/0	0/0	0/1	0/0	0/0	0/0	0/0	0/0	0/0	0/0	0/0	0/0	0/0	0/0
Chromosome2-574090	DEL	PRECISE	150	0	0	0/0	0/0	0/0	0/0	0/0	0/0	0/0	0/0	0/0	0/0	0/1	0/0	0/1	0/0
Chromosome2-579488	DEL	PRECISE	67	0	0.577	0/0	0/0	0/0	0/0	0/0	0/0	0/0	0/0	0/0	0/0	0/1	0/0	0/1	0/0
Chromosome2-579672	DEL	PRECISE	8147	0	0	0/0	0/0	0/0	0/0	0/0	0/0	0/0	0/0	0/0	0/0	0/1	0/0	0/1	0/0
Chromosome2-584720	INS	PRECISE	876	0	0	0/0	0/0	0/0	0/0	0/1	0/0	0/0	0/0	0/0	0/0	0/0	0/0	0/0	0/0
Chromosome2-585803	INS	PRECISE	676	0	0	0/0	0/0	0/0	0/0	0/0	0/0	0/0	0/0	0/0	0/0	0/0	0/0	0/0	0/1
Chromosome2-593166	DEL	PRECISE	655	0.577	0.577	0/0	0/0	0/0	0/0	0/0	0/0	0/0	0/0	0/0	0/0	0/1	0/1	0/1	0/0
Chromosome2-599309	DEL	PRECISE	15040125	0	0	0/0	0/0	0/0	0/0	0/0	0/0	0/0	0/0	0/0	0/0	0/0	0/0	0/0	1/1
Chromosome2-599530	INS	PRECISE	90	0	0.707	0/0	0/0	0/0	0/0	0/0	0/0	0/0	0/0	0/0	0/0	0/1	0/0	0/1	0/0
Chromosome2-607719	DEL	PRECISE	80	0	0	0/0	0/0	0/0	0/0	0/0	0/0	0/0	0/0	0/0	0/0	0/0	0/1	0/0	0/0
Chromosome2-609706	DEL	PRECISE	62	0	0	0/0	0/0	0/0	0/0	0/0	0/0	0/0	0/0	0/0	0/0	0/1	0/0	0/1	0/0
Chromosome2-622399	INS	PRECISE	137	0	0	0/0	0/0	0/0	0/0	0/0	0/0	0/0	0/0	0/0	0/0	0/0	0/1	0/0	0/0
Chromosome2-624181	INS	PRECISE	67	0	0	0/0	0/0	0/0	0/0	0/0	0/0	0/0	0/0	0/0	0/0	0/0	1/1	0/0	0/0
Chromosome2-630675	INS	PRECISE	90	0	0	0/0	0/0	0/0	0/0	0/0	0/0	0/0	0/0	0/0	0/0	0/1	0/0	0/1	0/0
Chromosome2-631997	DEL	PRECISE	80	0	0	0/0	0/0	0/0	0/0	0/1	0/1	0/1	0/0	0/0	0/0	0/0	0/0	0/0	0/0
Chromosome2-633210	INS	PRECISE	107	0	0	0/0	0/0	0/0	0/0	0/0	0/0	0/0	0/0	0/0	0/0	0/0	0/0	0/0	0/1
Chromosome2-635934	DEL	PRECISE	130	0	0	0/0	0/0	0/0	0/0	0/0	0/0	0/0	0/0	0/0	0/0	0/1	0/0	0/0	0/0
Chromosome2-635953	INS	PRECISE	54	0	0	0/0	0/0	0/0	0/0	0/0	0/0	0/0	0/0	0/0	0/0	0/0	0/0	0/0	0/1
Chromosome2-648122	INS	PRECISE	232	0	0.756	0/0	0/0	0/0	0/0	0/1	0/1	0/1	0/0	0/0	0/0	0/0	0/1	0/1	0/1
Chromosome2-648745	INS	PRECISE	1054	0	0	0/0	0/0	0/0	0/0	0/0	0/0	0/0	0/0	0/0	0/0	0/0	0/0	0/0	0/1
Chromosome2-656879	INS	PRECISE	53	0	0	0/0	0/0	0/0	0/0	0/1	0/1	0/0	0/0	0/0	0/0	0/0	0/0	0/0	0/0
Chromosome2-661803	INS	PRECISE	231	0	0.577	0/0	0/0	0/0	0/0	0/0	0/0	0/0	0/0	0/0	0/0	0/1	0/1	0/1	0/0
Chromosome2-664836	DEL	PRECISE	836	0	0	0/0	0/0	0/0	0/0	0/0	0/0	0/0	0/0	0/0	0/0	0/1	0/1	0/1	0/0
Chromosome2-671203	INS	PRECISE	86	0	0	0/0	0/0	0/0	0/0	0/0	0/0	0/0	0/0	0/0	0/0	0/0	0/0	0/0	0/1
Chromosome2-671367	DEL	PRECISE	696	0	0	0/0	0/0	0/0	0/0	0/0	0/0	0/0	0/0	0/0	0/0	0/0	0/0	0/0	0/1
Chromosome2-679949	INS	PRECISE	439	0	0	0/0	0/0	0/0	1/1	0/0	0/0	0/0	0/0	0/0	0/0	0/0	0/0	0/0	0/0
Chromosome2-689418	INS	PRECISE	138	0	0.5	0/0	0/0	0/0	0/0	0/0	0/0	0/0	0/0	0/0	0/0	0/1	0/1	0/1	0/1
Chromosome2-690367	INS	PRECISE	2832	0	0.707	0/0	0/0	0/0	0/0	0/0	0/0	0/0	0/0	0/0	0/0	0/1	0/0	0/1	0/0
Chromosome2-691658	INS	PRECISE	59	0	0	0/0	0/0	0/0	0/0	0/0	0/0	0/0	0/0	0/0	0/0	0/0	0/0	0/0	0/1
Chromosome2-701875	INS	PRECISE	741	0	0	0/0	0/0	0/0	0/0	0/0	0/0	0/0	0/0	0/0	0/0	0/0	0/0	0/1	0/0
Chromosome2-703247	INS	PRECISE	120	0	0.354	0/0	0/0	0/0	0/1	1/1	0/1	0/1	0/0	0/0	0/0	0/0	0/1	0/1	0/1
Chromosome2-704284	DEL	PRECISE	236	0	0	0/0	0/0	0/0	0/0	0/0	0/0	0/0	0/0	0/0	0/0	0/0	0/1	0/1	0/0
Chromosome2-710929	DEL	PRECISE	208	0	0	0/0	0/0	0/0	0/1	0/0	0/0	0/0	0/0	0/0	0/0	0/0	0/0	0/0	0/0
Chromosome2-714201	DEL	PRECISE	94	0	0	0/0	0/0	0/0	0/0	0/0	0/0	0/0	0/0	0/0	0/0	0/1	0/0	0/1	0/0
Chromosome2-718839	INS	PRECISE	653	0	0.707	0/0	0/0	0/0	0/0	0/0	0/0	0/0	0/0	0/0	0/0	0/1	0/1	0/0	0/0
Chromosome2-721979	INS	PRECISE	68	0	0.577	0/0	0/0	0/0	0/0	0/0	0/0	0/0	0/0	0/0	0/0	0/1	0/1	0/0	0/0
Chromosome2-724605	INS	PRECISE	995	0	0	0/0	0/0	0/0	0/0	0/0	0/0	0/0	0/0	0/0	0/0	0/1	0/0	0/0	0/0
Chromosome2-744857	INS	PRECISE	265	0	0	0/0	0/0	0/0	0/0	0/0	0/0	0/0	0/0	0/0	0/0	0/1	0/1	0/1	0/0
Chromosome2-762609	INS	PRECISE	1575	0	0.577	0/0	0/0	0/0	0/0	0/0	0/0	0/0	0/0	0/0	0/0	0/1	0/1	0/1	0/0
Chromosome2-763649	DEL	PRECISE	257	0	0	0/0	0/0	0/0	0/0	0/0	0/0	0/0	0/0	0/0	0/0	0/1	0/1	0/1	0/0
Chromosome2-769644	INS	PRECISE	775	0	0.577	0/0	0/0	0/0	0/0	0/0	0/0	0/0	0/0	0/0	0/0	0/1	0/1	0/1	0/0
Chromosome2-771657	INS	PRECISE	2829	0	0	0/0	0/0	0/0	0/0	0/0	0/0	0/0	0/0	0/0	0/0	0/1	0/0	0/0	0/0
Chromosome2-778827	INS	PRECISE	8927	0	0	0/0	0/0	0/0	0/0	0/0	0/0	0/0	0/0	0/0	0/0	0/0	0/0	0/0	0/1
Chromosome2-778833	BND	PRECISE	0	0	0	0/0	0/0	0/0	0/0	0/0	0/0	0/0	0/0	0/0	0/0	0/0	0/0	0/0	0/1
Chromosome2-779095	DEL	PRECISE	2547	0	0	0/0	0/0	0/0	0/1	0/0	0/0	0/0	0/0	0/0	0/0	0/0	0/0	0/0	0/0

Chromosome2-781561	INS	PRECISE	5319	0	0	0/0	0/0	1/1	0/0	0/0	0/0	0/0	0/0	0/0	0/0	0/0	0/0	0/0	0/0
Chromosome2-784236	INS	PRECISE	666	0	0	0/0	0/0	0/0	0/1	0/0	0/0	0/0	0/0	0/0	0/0	0/0	0/0	0/0	0/0
Chromosome2-787166	DEL	PRECISE	178	0	0	0/0	0/0	0/0	0/0	0/0	0/0	0/0	0/0	0/0	0/0	0/0	0/1	0/0	0/0
Chromosome2-789759	DEL	PRECISE	73	0	0	0/0	0/0	0/0	0/1	0/0	0/0	0/0	0/0	0/0	0/0	0/0	0/0	0/0	0/0
Chromosome2-793287	DEL	PRECISE	962	0	0	0/0	0/0	0/0	0/0	0/0	0/0	0/0	0/0	0/0	0/0	0/0	0/1	0/0	0/0
Chromosome2-795650	DEL	PRECISE	4718	0	0	0/0	0/0	0/0	0/0	0/0	0/0	0/0	0/0	0/0	0/0	0/1	0/0	0/0	0/0
Chromosome2-799601	INS	PRECISE	12531	0	0	0/0	0/0	0/0	1/1	0/0	0/0	0/0	0/0	0/0	0/0	0/0	0/0	0/0	0/0
Chromosome2-804290	DEL	PRECISE	3288	0	0	0/0	0/0	0/0	0/0	0/0	0/0	0/0	0/0	0/0	0/0	0/1	0/1	0/0	0/0
Chromosome2-812302	INS	PRECISE	2678	0	0	0/0	0/0	0/0	0/0	0/1	0/0	0/0	0/0	0/0	0/0	0/0	0/0	0/0	0/0
Chromosome2-812615	INS	PRECISE	1846	0	0	0/0	0/0	0/1	0/0	0/0	0/0	0/0	0/0	0/0	0/0	0/0	0/0	0/0	0/0
Chromosome2-813921	DEL	PRECISE	327	0	0	0/0	0/0	0/0	0/0	0/0	0/0	0/0	0/0	0/0	0/0	0/1	0/1	0/0	0/0
Chromosome2-821725	INS	PRECISE	12465	0	0	0/0	0/0	0/0	0/0	0/1	0/0	0/0	0/0	0/0	0/0	0/0	0/0	0/0	0/0
Chromosome2-826931	INS	PRECISE	7615	0	0	0/0	0/0	1/1	0/0	0/0	0/0	0/0	0/0	0/0	0/0	0/0	0/0	0/0	0/0
Chromosome2-832398	INS	PRECISE	6090	0	0	0/0	0/0	0/0	0/0	0/0	0/0	0/0	0/0	0/0	0/0	0/0	0/0	0/0	0/1
Chromosome2-835061	INS	PRECISE	754	0	0	0/0	0/0	0/0	0/0	0/0	0/0	0/0	0/0	0/0	0/0	0/0	0/0	0/1	0/0
Chromosome2-849980	DEL	PRECISE	54923	0	0	0/0	0/0	0/0	0/0	0/0	0/0	0/0	0/0	0/0	0/0	0/0	0/0	1/1	0/0
Chromosome2-851628	INS	PRECISE	664	0	0	0/0	0/0	0/0	0/0	0/0	0/0	0/0	0/0	0/0	0/0	0/0	0/0	0/0	0/1
Chromosome2-857345	INS	PRECISE	707	0	0	0/0	0/0	0/0	0/0	0/1	0/0	0/0	0/0	0/0	0/0	0/0	0/0	0/0	0/0
Chromosome2-857351	BND	PRECISE	0	0	0	0/0	0/0	0/1	0/0	0/0	0/0	0/0	0/0	0/0	0/0	0/0	0/0	0/0	0/0
Chromosome2-865083	INS	PRECISE	1199	0	0.957	0/0	0/0	0/0	0/1	0/1	0/1	0/1	0/0	0/0	0/0	0/0	0/0	0/0	0/0
Chromosome2-874814	INS	PRECISE	1198	0	0	0/0	0/0	0/1	0/0	0/0	0/0	0/0	0/0	0/0	0/0	0/0	0/0	0/0	0/0
Chromosome2-879122	INS	PRECISE	1201	0	0	0/0	0/0	0/1	0/0	0/0	0/0	0/0	0/0	0/0	0/0	0/0	0/0	0/0	0/0
Chromosome2-880120	DEL	PRECISE	5417	0	0	0/0	0/0	0/1	0/1	1/1	0/1	0/1	0/0	0/0	0/0	0/0	0/0	0/0	0/0
Chromosome2-887020	DEL	PRECISE	70	0	0	0/0	0/0	0/0	0/0	0/0	0/0	0/0	0/0	0/0	0/0	0/0	0/0	0/0	0/1
Chromosome2-887290	DEL	PRECISE	3553	0	0	0/0	0/0	0/0	0/0	0/0	0/0	0/0	0/0	0/0	0/0	0/0	0/0	0/0	0/1
Chromosome2-899885	DEL	PRECISE	16914	0	0	0/0	0/0	0/0	0/0	0/0	0/0	0/0	0/0	0/0	0/0	0/0	0/0	0/0	0/1
Chromosome2-906474	INS	PRECISE	242	0	0	0/0	0/0	0/0	0/0	0/0	0/0	0/0	0/0	0/0	0/0	0/0	0/0	0/1	0/0
Chromosome2-906689	INS	PRECISE	4861	0	0	0/0	0/0	0/0	0/0	0/0	0/0	0/1	0/0	0/0	0/0	0/0	0/0	0/0	0/0
Chromosome2-910633	INS	PRECISE	5349	0	0	0/0	0/0	0/1	0/0	0/0	0/0	0/0	0/0	0/0	0/0	0/0	0/0	0/0	0/0
Chromosome2-915914	INS	PRECISE	3761	0	0	0/0	0/0	0/0	0/0	1/1	0/0	0/0	0/0	0/0	0/0	0/0	0/0	0/0	0/0
Chromosome2-922911	DEL	PRECISE	1978	0	0	0/0	0/0	0/0	0/1	1/1	0/1	0/1	0/0	0/0	0/0	0/0	0/1	0/0	0/1
Chromosome2-925539	DEL	PRECISE	1057	0	0	0/0	0/0	0/0	0/0	0/0	0/0	0/0	0/0	0/0	0/0	0/1	0/0	0/1	0/0
Chromosome2-928801	INS	PRECISE	58	0	0	0/0	0/0	0/0	0/0	0/0	0/0	0/0	0/0	0/0	0/0	0/0	0/0	0/1	0/0
Chromosome2-931819	DEL	PRECISE	168	0	0	0/0	0/0	0/0	0/0	0/1	0/0	0/0	0/0	0/0	0/0	0/1	1/1	0/1	0/0
Chromosome2-939520	INS	PRECISE	2990	0	0	0/0	0/0	0/1	0/0	0/0	0/0	0/0	0/0	0/0	0/0	0/0	0/0	0/0	0/0
Chromosome2-943456	INV	PRECISE	4280684	0	0	0/0	0/0	0/0	0/0	0/0	0/0	0/0	0/0	0/0	0/0	0/0	0/0	0/0	0/1
Chromosome2-946357	INS	PRECISE	12593	0	0	0/0	0/0	0/1	0/0	0/0	0/0	0/0	0/0	0/0	0/0	0/0	0/0	0/0	0/0
Chromosome2-951154	INS	PRECISE	3678	0	0	0/0	0/0	0/0	0/0	0/0	0/0	0/0	0/0	0/0	0/0	0/1	0/0	0/0	0/0
Chromosome2-956418	INS	PRECISE	814	0	0.816	0/0	0/0	0/0	1/1	0/1	0/1	0/1	0/0	0/0	0/0	0/0	0/0	0/0	0/0
Chromosome2-965604	INS	PRECISE	77	0	0	0/0	0/0	0/0	0/0	0/0	0/0	0/0	0/0	0/0	0/0	0/0	0/0	0/0	0/1
Chromosome2-968826	DEL	PRECISE	2658	0	0	0/0	0/0	0/0	1/1	1/1	0/1	0/0	0/0	0/0	0/0	0/0	0/0	0/0	0/0
Chromosome2-971100	DEL	PRECISE	55	0	0	0/0	0/0	0/0	0/0	0/0	0/0	0/1	0/0	0/0	0/0	0/0	0/0	0/0	0/0
Chromosome2-992326	DEL	PRECISE	129	0	0	0/0	0/0	0/0	0/0	1/1	0/0	0/0	0/0	0/0	0/0	0/0	0/0	0/0	0/0
Chromosome2-1011072	INS	PRECISE	1179	0	0	0/0	0/0	0/0	0/0	0/0	0/0	0/0	0/0	0/0	0/0	0/0	0/0	0/0	0/1
Chromosome2-1012687	DEL	PRECISE	380	0	0	0/0	0/0	0/0	0/0	0/0	0/0	0/0	0/0	0/0	0/0	0/0	0/0	0/0	0/1
Chromosome2-1014807	INS	PRECISE	61	0	0	0/0	0/0	0/0	0/0	0/0	0/0	0/0	0/0	0/0	0/0	0/1	0/0	0/0	0/0
Chromosome2-1016087	DEL	PRECISE	579	0	0	0/0	0/0	0/0	0/0	0/0	0/0	0/0	0/0	0/0	0/0	0/0	0/1	0/0	0/0
Chromosome2-1023909	DEL	PRECISE	5356	0	0	0/0	0/0	0/0	1/1	0/0	0/0	0/0	0/0	0/0	0/0	0/0	0/0	0/0	0/1
Chromosome2-1031381	DEL	PRECISE	70	0	0	0/0	0/0	0/0	1/1	0/0	0/0	0/0	0/0	0/0	0/0	0/0	0/0	0/0	0/0
Chromosome2-1035095	DEL	PRECISE	57	0	0	0/0	0/0	0/1	1/1	0/0	0/0	0/1	0/0	0/0	0/0	0/0	0/0	0/0	0/1
Chromosome2-1037320	DEL	PRECISE	214	0	0	0/0	0/0	0/1	1/1	0/0	0/0	0/1	0/0	0/0	0/0	0/0	0/0	0/0	0/1
Chromosome2-1042102	INS	PRECISE	1362	0	0	0/0	0/0	0/0	0/0	0/0	0/0	0/0	0/0	0/0	0/0	0/0	0/0	0/0	1/1
Chromosome2-1042350	INS	PRECISE	275	0	0	0/0	0/0	0/0	0/0	0/0	0/0	0/0	0/0	0/0	0/0	0/0	0/0	0/1	0/0
Chromosome2-1046110	INS	PRECISE	5094	0	0	0/0	0/0	0/0	0/0	0/1	0/0	0/0	0/0	0/0	0/0	0/0	0/0	0/0	0/0
Chromosome2-1046866	INS	PRECISE	67	0	0	0/0	0/0	0/1	0/1	0/1	0/1	0/1	0/0	0/0	0/0	0/1	0/0	0/1	0/1
Chromosome2-1046994	DEL	PRECISE	571	0	0	0/0	0/0	0/0	0/0	0/0	0/0	0/1	0/0	0/0	0/0	0/0	0/0	0/0	0/0
Chromosome2-1051328	INS	PRECISE	69	0	0	0/0	0/0	0/0	0/0	0/0	0/0	0/0	0/0	0/0	0/0	0/0	0/1	0/0	0/0
Chromosome2-1051728	DEL	PRECISE	5831	0	0	0/0	0/0	0/0	0/0	0/0	0/0	0/0	0/0	0/0	0/0	0/0	0/1	0/0	0/0
Chromosome2-1058433	DEL	PRECISE	598	0	0	0/0	0/0	0/0	0/0	0/0	0/0	0/1	0/0	0/0	0/0	0/0	0/0	0/0	0/0
Chromosome2-1064080	DEL	PRECISE	80	0	0	0/0	0/0	0/0	0/0	0/0	0/0	0/0	0/0	0/0	0/0	0/1	0/0	0/0	0/0
Chromosome2-1064643	INS	PRECISE	3941	0	0	0/0	0/0	0/0	0/0	0/0	0/0	0/0	0/0	0/0	0/0	0/0	1/1	0/0	0/0
Chromosome2-1068475	DEL	PRECISE	54	0	0	0/0	0/0	0/0	0/0	0/0	0/0	0/0	0/0	0/0	0/0	0/1	0/1	0/0	0/0
Chromosome2-1076660	DEL	PRECISE	127	0.577	0.577	0/0	0/0	0/0	0/0	0/0	0/0	0/0	0/0	0/0	0/0	0/1	0/1	0/0	0/0
Chromosome2-1078544	INS	PRECISE	87	0	0	0/0	0/0	0/0	0/0	0/0	0/0	0/1	0/0	0/0	0/0	0/0	0/0	0/0	0/0
Chromosome2-1083762	DEL	PRECISE	128	0	0	0/0	0/0	0/0	0/0	0/0	0/0	0/0	0/0	0/0	0/0	0/0	0/1	0/0	0/0
Chromosome2-1087761	DEL	PRECISE	59	0	0	0/0	0/0	0/0	0/0	0/0	0/0	0/0	0/0	0/0	0/0	0/1	0/0	0/0	0/0
Chromosome2-1088850	INS	PRECISE	81	0	0	0/0	0/0	0/0	0/0	0/0	0/0	0/0	0/0	0/0	0/0	0/0	0/1	0/0	0/0
Chromosome2-1090155	DEL	PRECISE	177	0	0	0/0	0/0	0/0	0/1	0/0	0/0	0/1	0/0	0/0	0/0	0/0	0/0	0/0	0/1
Chromosome2-1097523	DEL	PRECISE	61	0	0	0/0	0/0	0/0	0/1	0/1	0/1	0/1	0/0	0/0	0/0	0/0	0/0	0/0	0/0
Chromosome2-1099341	INS	PRECISE	189	0	0	0/0	0/0	0/0	0/0	0/0	0/0	0/0	0/0	0/0	0/0	0/0	0/0	0/1	0/0
Chromosome2-1103113	INS	PRECISE	369	0	0	0/0	0/0	0/0	0/0	0/0	0/0	0/0	0/0	0/0	0/0	0/1	0/0	0/1	0/0
Chromosome2-1111222	DEL	PRECISE	132	0.518	0.354	0/0	0/0	0/0	1/1	1/1	0/1	0/1	0/0	0/0	0/0	0/1	0/1	0/1	0/1
Chromosome2-1118608	INS	PRECISE	161	0	0	0/0	0/0	0/0	0/0	0/1	0/0	0/0	0/0	0/0	0/0	0/0	0/0	0/0	0/0

Chromosome2-1118755	DEL	PRECISE	137	0	0	0/0	0/0	0/0	0/0	0/1	0/0	0/0	0/0	0/0	0/0	0/1	0/1	0/1	0/1
Chromosome2-1120288	DEL	PRECISE	675	0	0	0/0	0/0	0/0	0/0	0/1	0/0	0/0	0/0	0/0	0/0	0/1	0/1	0/1	0/0
Chromosome2-1121630	DEL	PRECISE	56	0	0	0/0	0/0	0/0	0/0	0/0	0/0	0/0	0/0	0/0	0/0	0/1	0/1	0/0	0/0
Chromosome2-1123857	INS	PRECISE	736	0	0	0/0	0/0	0/0	0/0	0/0	0/0	0/0	0/0	0/0	0/0	0/0	0/0	0/1	0/0
Chromosome2-1123936	DEL	PRECISE	2378	0	0	0/0	0/0	0/0	0/0	0/0	0/0	0/0	0/0	0/0	0/0	0/1	0/1	0/0	0/0
Chromosome2-1128293	DEL	PRECISE	903	0	0	0/0	0/0	0/0	0/0	0/0	0/0	0/1	0/0	0/0	0/0	0/0	0/0	0/0	0/0
Chromosome2-1134488	INS	PRECISE	81	0	0	0/0	0/0	0/0	0/0	0/0	0/0	0/0	0/0	0/0	0/0	0/0	0/0	0/0	0/1
Chromosome2-1135555	DEL	PRECISE	174	0	0	0/0	0/0	0/0	0/0	0/0	0/0	0/0	0/0	0/0	0/0	0/1	0/1	0/1	0/0
Chromosome2-1138739	INS	PRECISE	181	0	0.577	0/0	0/0	0/0	0/0	0/0	0/0	0/0	0/0	0/0	0/0	0/1	0/1	0/1	0/0
Chromosome2-1140588	DEL	PRECISE	105	0	0	0/0	0/0	0/0	0/0	0/0	0/0	0/0	0/0	0/0	0/0	0/0	0/1	0/0	0/0
Chromosome2-1142286	INS	PRECISE	623	0	0	0/0	0/0	0/0	0/0	0/0	0/0	0/0	0/0	0/0	0/0	0/0	0/0	0/0	0/1
Chromosome2-1147587	INS	PRECISE	50	0	0	0/0	0/0	0/0	0/0	1/1	0/1	0/0	0/0	0/0	0/0	0/0	0/0	0/0	0/0
Chromosome2-1150008	INS	PRECISE	166	0	0	0/0	0/0	0/0	0/0	0/0	0/0	0/0	0/0	0/0	0/0	0/1	0/0	0/1	0/0
Chromosome2-1152466	BND	PRECISE	0	0	0	0/0	0/0	0/0	0/0	0/1	0/1	0/0	0/0	0/0	0/0	0/0	0/0	0/0	0/0
Chromosome2-1155923	DEL	PRECISE	885	0	0	0/0	0/0	0/1	1/1	1/1	0/0	0/1	0/0	0/0	0/0	0/0	0/0	0/0	0/1
Chromosome2-1162323	INS	PRECISE	10779	0	0	0/0	0/0	0/0	0/1	0/0	0/0	0/0	0/0	0/0	0/0	0/0	0/0	0/0	0/0
Chromosome2-1162861	INS	PRECISE	211	0	0	0/0	0/0	0/0	0/1	0/0	0/0	0/0	0/0	0/0	0/0	0/0	0/0	0/0	0/0
Chromosome2-1172411	INS	PRECISE	1469	0	0.707	0/0	0/0	0/0	0/0	0/0	0/0	0/0	0/0	0/0	0/0	0/1	0/0	0/1	0/0
Chromosome2-1172863	INS	PRECISE	4449	0	0	0/0	0/0	0/0	0/0	0/1	0/1	0/0	0/0	0/0	0/0	0/0	0/0	0/0	0/0
Chromosome2-1176892	INS	PRECISE	5595	0	0	0/0	0/0	0/0	0/0	0/0	0/0	0/0	0/0	0/0	0/0	0/0	1/1	0/0	0/0
Chromosome2-1177389	DEL	PRECISE	132	0	0	0/0	0/0	0/0	0/1	0/0	0/0	0/0	0/0	0/0	0/0	0/0	0/0	0/0	0/0
Chromosome2-1180029	DEL	PRECISE	1587	0	0	0/0	0/0	0/0	0/0	0/0	0/0	0/0	0/0	0/0	0/0	0/1	0/0	0/1	0/0
Chromosome2-1182689	INS	PRECISE	622	0	0	0/0	0/0	0/0	0/0	0/0	0/0	0/0	0/0	0/0	0/0	0/0	0/0	0/0	0/1
Chromosome2-1194659	INS	PRECISE	1312	0	0	0/0	0/0	0/0	0/1	0/0	0/0	0/0	0/0	0/0	0/0	0/0	0/0	0/0	0/0
Chromosome2-1202791	INS	PRECISE	61	0	0.354	0/0	0/0	0/0	1/1	1/1	0/1	0/1	0/0	0/0	0/0	0/1	0/1	0/1	0/1
Chromosome2-1204449	DEL	PRECISE	1004	0	0	0/0	0/0	0/0	0/0	0/0	0/0	0/0	0/0	0/0	0/0	0/1	0/1	0/1	0/0
Chromosome2-1208665	DEL	PRECISE	222	0	0	0/0	0/0	0/0	0/0	0/0	0/0	0/0	0/0	0/0	0/0	0/1	0/1	0/1	0/0
Chromosome2-1210255	INS	PRECISE	3048	0	0	0/0	0/0	0/0	0/0	0/0	0/0	0/0	0/0	0/0	0/0	0/0	0/1	0/0	0/0
Chromosome2-1210416	INS	PRECISE	893	0	0	0/0	0/0	0/0	0/0	0/0	0/0	0/0	0/0	0/0	0/0	0/0	0/1	0/0	0/0
Chromosome2-1223648	INS	PRECISE	1195	0	0.577	0/0	0/0	0/0	0/0	0/0	0/0	0/0	0/0	0/0	0/0	0/1	1/1	0/1	0/0
Chromosome2-1227745	INS	PRECISE	1243	0	0	0/0	0/0	0/0	0/0	0/0	0/0	0/0	0/0	0/0	0/0	0/0	0/0	0/0	0/1
Chromosome2-1239630	DEL	PRECISE	96	0	0	0/0	0/0	0/0	0/0	0/0	0/0	0/0	0/0	0/0	0/0	0/0	0/0	0/1	0/0
Chromosome2-1243873	DEL	PRECISE	225	0	0	0/0	0/0	0/0	0/0	0/0	0/0	0/0	0/0	0/0	0/0	0/1	0/0	0/1	0/0
Chromosome2-1244694	INS	PRECISE	4924	0	0	0/0	0/0	0/0	0/0	0/0	0/0	0/0	0/0	0/0	0/0	0/0	0/0	0/0	1/1
Chromosome2-1246876	DEL	PRECISE	686	0	0	0/0	0/0	0/0	0/0	0/0	0/0	0/0	0/0	0/0	0/0	0/1	0/1	0/1	0/0
Chromosome2-1255205	DEL	PRECISE	178	0	0	0/0	0/0	0/0	0/0	0/0	0/0	0/0	0/0	0/0	0/0	0/0	0/1	0/0	0/0
Chromosome2-1263659	INS	PRECISE	125	0	0	0/0	0/0	0/0	0/0	0/0	0/0	0/0	0/0	0/0	0/0	0/1	0/0	0/1	0/0
Chromosome2-1267255	INS	PRECISE	61	0	0	0/0	0/0	0/0	0/0	0/0	0/0	0/0	0/0	0/0	0/0	0/0	0/1	0/0	0/0
Chromosome2-1271955	INS	PRECISE	77	0	0	0/0	0/0	0/0	0/0	0/0	0/0	0/0	0/0	0/0	0/0	0/0	0/1	0/0	0/0
Chromosome2-1284906	DEL	PRECISE	74	0	0	0/0	0/0	0/0	0/0	0/0	0/0	0/0	0/0	0/0	0/0	0/1	0/0	0/1	0/0
Chromosome2-1289461	INS	PRECISE	120	0	0	0/0	0/0	0/0	1/1	0/0	0/0	0/1	0/0	0/0	0/0	0/0	0/0	0/0	0/0
Chromosome2-1291218	INS	PRECISE	150	0	0	0/0	0/0	0/0	0/1	0/0	0/0	0/0	0/0	0/0	0/0	0/0	0/0	0/0	0/0
Chromosome2-1303099	INS	PRECISE	107	0	0	0/0	0/0	0/0	0/0	0/1	0/0	0/0	0/0	0/0	0/0	0/0	0/0	0/0	0/0
Chromosome2-1312876	INS	PRECISE	307	0	0.707	0/0	0/0	0/0	0/0	0/0	0/0	0/0	0/0	0/0	0/0	0/1	0/0	0/1	0/0
Chromosome2-1315681	DEL	PRECISE	1292	0	0	0/0	0/0	0/0	0/0	0/0	0/0	0/0	0/0	0/0	0/0	0/1	0/1	0/1	0/1
Chromosome2-1316349	INS	PRECISE	272	0	0.5	0/0	0/0	0/0	1/1	0/1	0/1	0/1	0/0	0/0	0/0	0/0	0/0	0/0	0/0
Chromosome2-1331442	DEL	PRECISE	633	0	0	0/0	0/0	0/0	0/0	0/1	0/0	0/0	0/0	0/0	0/0	0/1	0/1	0/1	0/0
Chromosome2-1333304	DEL	PRECISE	244	0	0	0/0	0/0	0/0	0/0	0/0	0/0	0/0	0/0	0/0	0/0	0/0	0/1	0/0	0/0
Chromosome2-1338091	DEL	PRECISE	62	0	0	0/0	0/0	0/0	0/1	0/1	0/1	0/0	0/0	0/0	0/0	0/0	0/0	0/0	0/0
Chromosome2-1338957	INS	PRECISE	6585	0	0	0/0	0/0	0/0	0/0	0/1	0/0	0/0	0/0	0/0	0/0	0/0	0/0	0/0	0/0
Chromosome2-1346402	INS	PRECISE	56	0	0	0/0	0/0	0/0	0/0	0/0	0/0	0/0	0/0	0/0	0/0	0/0	0/0	0/0	0/1
Chromosome2-1347825	INS	PRECISE	2670	0	0	0/0	0/0	0/0	0/0	0/0	0/0	0/0	0/0	0/0	0/0	0/0	0/0	1/1	0/0
Chromosome2-1355234	INS	PRECISE	164	0	0	0/0	0/0	0/0	0/0	0/1	0/0	0/0	0/0	0/0	0/0	0/0	0/0	0/0	0/0
Chromosome2-1355357	DEL	PRECISE	147	0	0	0/0	0/0	0/0	0/0	0/0	0/0	0/0	0/0	0/0	0/0	0/0	0/1	0/1	0/0
Chromosome2-1363557	DEL	PRECISE	361	0	0	0/0	0/0	0/0	1/1	1/1	0/1	0/1	0/0	0/0	0/0	0/1	0/1	0/1	0/1
Chromosome2-1375486	DEL	PRECISE	58	0	0	0/0	0/0	0/0	0/0	0/0	0/0	0/0	0/0	0/0	0/0	0/0	0/0	0/0	0/1
Chromosome2-1376450	INS	PRECISE	364	0	0	0/0	0/0	0/0	0/0	0/1	0/0	0/0	0/0	0/0	0/0	0/0	0/0	0/0	0/0
Chromosome2-1396033	BND	PRECISE	0	0	0	0/0	0/0	0/0	0/0	0/1	0/0	0/0	0/0	0/0	0/0	0/0	0/0	0/0	0/0
Chromosome2-1404005	INS	PRECISE	6443	0	0	0/0	0/0	0/1	0/0	0/0	0/0	0/0	0/0	0/0	0/0	0/0	0/0	0/0	0/0
Chromosome2-1404011	BND	PRECISE	0	0	0	0/0	0/0	0/1	0/0	0/0	0/0	0/0	0/0	0/0	0/0	0/0	0/0	0/0	0/0
Chromosome2-1406046	DEL	PRECISE	150	0	0	0/0	0/0	0/0	1/1	1/1	0/1	0/1	0/0	0/0	0/0	0/1	0/1	0/0	0/0
Chromosome2-1408574	DEL	PRECISE	122	0	0	0/0	0/0	0/0	0/0	0/0	0/0	0/0	0/0	0/0	0/0	0/0	0/0	0/1	0/0
Chromosome2-1410773	INS	PRECISE	1249	0	0	0/0	0/0	0/0	0/0	0/0	0/0	0/0	0/0	0/0	0/0	0/0	0/0	0/1	0/0
Chromosome2-1411941	INS	PRECISE	1070	0	0	0/0	0/0	0/0	0/0	0/0	0/0	0/0	0/0	0/0	0/0	0/0	0/0	0/0	0/1
Chromosome2-1417170	INS	PRECISE	81	0	0	0/0	0/0	0/0	0/0	0/0	0/0	0/0	0/0	0/0	0/0	0/0	0/0	0/0	0/1
Chromosome2-1426336	DEL	PRECISE	98	0	0	0/0	0/0	0/0	0/0	0/1	0/0	0/0	0/0	0/0	0/0	0/0	0/0	0/1	0/0
Chromosome2-1427193	INS	PRECISE	1256	0	0	0/0	0/0	0/0	0/0	0/0	0/0	0/0	0/0	0/0	0/0	0/0	0/0	0/0	1/1
Chromosome2-1429822	INS	PRECISE	74	0.707	0.707	0/0	0/0	0/0	0/0	0/0	0/0	0/0	0/0	0/0	0/0	0/0	0/0	0/1	0/0
Chromosome2-1443949	DEL	PRECISE	115	0	0	0/0	0/0	0/0	0/0	0/0	0/0	0/0	0/0	0/0	0/0	0/0	0/0	0/0	0/1
Chromosome2-1463855	DEL	PRECISE	1075	0	0	0/0	0/0	0/0	0/0	0/0	0/0	0/0	0/0	0/0	0/0	0/0	0/0	0/0	0/1
Chromosome2-1465120	DEL	PRECISE	166	0	0	0/0	0/0	0/0	0/0	0/0	0/0	0/0	0/0	0/0	0/0	0/0	0/0	0/0	0/1
Chromosome2-1468195	DEL	PRECISE	95	0	0	0/0	0/0	0/0	0/0	0/0	0/0	0/0	0/0	0/0	0/0	0/1	0/0	0/1	0/0
Chromosome2-1471086	DEL	PRECISE	251	0	0	0/0	0/0	0/0	0/0	0/0	0/0	0/0	0/0	0/0	0/0	0/1	0/1	0/1	0/1

Chromosome2-1476689 INS	PRECISE	14601	0	0	0/0	0/0	0/0	0/0	0/0	0/0	0/0	0/0	0/0	0/0	0/1	0/0	0/0	0/0
Chromosome2-1477845 DEL	PRECISE	16108	0	0	0/0	0/0	0/0	0/0	0/0	0/0	0/0	0/0	0/0	0/0	0/0	0/0	0/0	0/1
Chromosome2-1477955 INS	PRECISE	93	0	0	0/0	0/0	0/0	0/0	0/0	0/0	0/0	0/0	0/0	0/0	0/1	0/0	0/0	0/0
Chromosome2-1479155 INS	PRECISE	8243	0	0	0/0	0/0	0/0	1/1	0/0	0/0	0/0	0/0	0/0	0/0	0/0	0/0	0/0	0/0
Chromosome2-1479584 DEL	PRECISE	73	0	0	0/0	0/0	0/0	0/0	0/0	0/0	0/0	0/0	0/0	0/0	0/1	0/1	0/1	0/0
Chromosome2-1481028 DEL	PRECISE	295	0	0	0/0	0/0	0/0	0/0	0/0	0/0	0/0	0/0	0/0	0/0	0/0	0/0	0/1	0/0
Chromosome2-1483151 DEL	PRECISE	6120	0.354	0.354	0/0	0/0	0/1	1/1	1/1	0/1	0/1	0/0	0/0	0/0	0/1	0/1	0/1	0/0
Chromosome2-1493252 DEL	PRECISE	50	0	0	0/0	0/0	0/0	0/0	0/1	0/0	0/0	0/0	0/0	0/0	0/0	0/0	0/0	0/0
Chromosome2-1493626 INS	PRECISE	4504	0	0	0/0	0/0	0/0	0/0	0/1	0/0	0/0	0/0	0/0	0/0	0/0	0/0	0/0	0/0
Chromosome2-1494106 BND	PRECISE	0	0	0	0/0	0/0	0/0	0/0	0/0	0/0	0/1	0/0	0/0	0/0	0/0	0/0	0/0	0/0
Chromosome2-1500886 DEL	PRECISE	4278	0	0	0/0	0/0	0/1	0/1	1/1	0/0	0/0	0/0	0/0	0/0	0/0	0/0	0/0	0/0
Chromosome2-1501450 INS	PRECISE	5096	0	0	0/0	0/0	0/0	0/0	0/0	0/0	0/0	0/0	0/0	0/0	1/1	0/0	0/0	0/0
Chromosome2-1502962 DEL	PRECISE	87	0	0	0/0	0/0	0/0	0/0	0/0	0/0	0/0	0/0	0/0	0/0	0/1	0/0	0/0	0/0
Chromosome2-1504665 DEL	PRECISE	248	0	0	0/0	0/0	0/0	0/0	0/0	0/0	0/0	0/0	0/0	0/0	0/1	0/0	0/0	0/0
Chromosome2-1510105 DEL	PRECISE	181	0	0	0/0	0/0	0/0	0/1	0/0	0/0	0/0	0/0	0/0	0/0	0/0	0/0	0/0	0/0
Chromosome2-1516915 DEL	PRECISE	54	0	0	0/0	0/0	0/0	0/0	0/0	0/0	0/0	0/0	0/0	0/0	0/1	0/0	0/0	0/0
Chromosome2-1518367 INS	PRECISE	74	0	0	0/0	0/0	0/0	0/0	0/0	0/0	0/0	0/0	0/0	0/0	0/1	0/0	0/0	0/0
Chromosome2-1521532 BND	PRECISE	0	0	0	0/0	0/0	0/0	0/0	0/1	0/0	0/1	0/0	0/0	0/0	0/0	0/0	0/0	0/0
Chromosome2-1522418 DEL	PRECISE	4954	0	0	0/0	0/0	0/0	0/0	0/0	0/0	0/0	0/0	0/0	0/0	0/1	0/1	0/0	0/0
Chromosome2-1525526 DEL	PRECISE	1420	0	0	0/0	0/0	0/0	0/0	0/1	0/0	0/0	0/0	0/0	0/0	0/0	0/0	0/0	0/0
Chromosome2-1534011 DEL	PRECISE	635	0	0	0/0	0/0	0/0	0/0	0/0	0/0	0/0	0/0	0/0	0/0	0/0	0/1	0/0	0/0
Chromosome2-1539373 DEL	PRECISE	66	0	0	0/0	0/0	0/0	0/0	0/0	0/0	0/0	0/0	0/0	0/0	0/0	0/1	0/0	0/0
Chromosome2-1542627 DEL	PRECISE	272	0	0	0/0	0/0	0/0	0/1	0/0	0/0	0/0	0/0	0/0	0/0	0/0	0/0	0/0	0/0
Chromosome2-1543422 DEL	PRECISE	512	0	0	0/0	0/0	0/0	0/0	0/0	0/0	0/0	0/0	0/0	0/0	0/0	0/0	0/0	0/1
Chromosome2-1547558 DEL	PRECISE	2785	0	0	0/0	0/0	0/0	0/0	0/0	0/0	0/0	0/0	0/0	0/0	0/1	0/1	0/0	0/0
Chromosome2-1547669 DEL	PRECISE	275	0	0	0/0	0/0	0/1	0/1	1/1	0/1	0/1	0/0	0/0	0/0	0/0	0/0	0/0	0/0
Chromosome2-1558138 DEL	PRECISE	73	0	0	0/0	0/0	0/0	0/0	0/1	0/1	0/1	0/0	0/0	0/0	0/0	0/0	0/0	0/0
Chromosome2-1559922 DEL	PRECISE	34985	0	0	0/0	0/0	0/0	0/0	0/0	0/0	0/0	0/0	0/0	0/0	0/1	1/1	1/1	0/0
Chromosome2-1561593 DEL	PRECISE	1408	0	0	0/0	0/0	0/0	0/0	0/0	0/0	0/0	0/0	0/0	0/0	0/1	0/0	0/0	0/0
Chromosome2-1566076 INS	PRECISE	1084	0	0	0/0	0/0	0/0	0/0	0/0	0/0	0/0	0/0	0/0	0/0	1/1	0/0	0/0	0/0
Chromosome2-1571850 INS	PRECISE	8676	0	0	0/0	0/0	0/0	0/0	0/0	0/0	0/0	0/0	0/0	0/0	0/0	0/0	0/0	0/1
Chromosome2-1594839 INS	PRECISE	401	0	0	0/0	0/0	0/0	0/0	0/0	0/0	0/0	0/0	0/0	0/0	0/0	0/0	0/0	0/1
Chromosome2-1595632 INS	PRECISE	162	0	0	0/0	0/0	0/0	0/0	0/0	0/0	0/0	0/0	0/0	0/0	0/0	0/0	0/0	0/1
Chromosome2-1597321 DEL	PRECISE	1338	0	0	0/0	0/0	0/0	0/0	0/0	0/0	0/0	0/0	0/0	0/0	0/1	0/0	0/1	0/1
Chromosome2-1602712 INS	PRECISE	52	0	0	0/0	0/0	0/1	1/1	1/1	0/1	0/1	0/0	0/0	0/0	0/0	0/0	0/0	0/0
Chromosome2-1620590 DEL	PRECISE	91	0	0	0/0	0/0	0/0	0/0	0/0	0/0	0/0	0/0	0/0	0/0	0/1	0/0	0/1	0/0
Chromosome2-1623105 DEL	PRECISE	263	0	0	0/0	0/0	0/0	0/0	0/0	0/0	0/0	0/0	0/0	0/0	0/1	0/0	0/0	0/0
Chromosome2-1629163 INS	PRECISE	336	0	0	0/0	0/0	0/0	0/0	0/0	0/0	0/0	0/0	0/0	0/0	0/0	0/1	0/0	0/0
Chromosome2-1633697 INS	PRECISE	66	0	0	0/0	0/0	0/0	0/0	0/0	0/0	0/0	0/0	0/0	0/0	0/0	0/0	0/0	0/1
Chromosome2-1635682 DEL	PRECISE	182	0	0	0/0	0/0	0/0	0/0	0/0	0/0	0/0	0/0	0/0	0/0	0/1	0/0	0/1	0/0
Chromosome2-1639557 DEL	PRECISE	57	0	0	0/0	0/0	0/0	0/0	0/0	0/0	0/0	0/0	0/0	0/0	0/1	0/0	0/1	0/0
Chromosome2-1642394 INS	PRECISE	2854	0	0	0/0	0/0	0/0	1/1	0/0	0/0	0/0	0/0	0/0	0/0	0/0	0/0	0/0	0/0
Chromosome2-1642524 DEL	PRECISE	211	0	0	0/0	0/0	0/0	1/1	0/1	0/0	0/0	0/0	0/0	0/0	0/1	0/0	0/1	0/1
Chromosome2-1643672 INS	PRECISE	8240	0	0	0/0	0/0	0/0	0/0	0/0	0/0	0/0	0/0	0/0	0/0	0/0	0/0	0/0	0/0
Chromosome2-1643672 BND	PRECISE	0	0	0	0/0	0/0	0/0	0/1	0/0	0/0	0/0	0/0	0/0	0/0	0/0	0/0	0/0	0/0
Chromosome2-1652777 INS	PRECISE	178	0	0	0/0	0/0	0/0	0/0	0/0	0/0	0/0	0/0	0/0	0/0	0/0	0/1	0/0	0/0
Chromosome2-1653633 DEL	PRECISE	718	0	0	0/0	0/0	0/0	0/0	0/0	0/0	0/0	0/0	0/0	0/0	0/1	0/1	0/1	0/0
Chromosome2-1660115 DEL	PRECISE	83	0	0	0/0	0/0	0/0	0/0	0/0	0/0	0/0	0/0	0/0	0/0	0/1	0/1	0/1	0/0
Chromosome2-1661792 INS	PRECISE	3548	0	0	0/0	0/0	0/0	0/0	0/0	0/0	0/0	0/0	0/0	0/0	0/0	0/0	0/0	0/1
Chromosome2-1663338 INS	PRECISE	656	0	0	0/0	0/0	0/0	0/0	0/1	0/0	0/0	0/0	0/0	0/0	0/0	0/0	0/0	0/0
Chromosome2-1667072 DEL	PRECISE	71	0	0	0/0	0/0	0/0	0/0	0/0	0/0	0/0	0/0	0/0	0/0	0/1	0/1	0/1	0/0
Chromosome2-1668941 DEL	PRECISE	5592	0	0	0/0	0/0	0/0	0/0	0/0	0/0	0/0	0/0	0/0	0/0	0/0	0/1	0/0	0/0
Chromosome2-1681890 INS	PRECISE	69	0	0	0/0	0/0	0/0	0/0	0/0	0/0	0/0	0/0	0/0	0/0	0/0	0/0	0/0	0/1
Chromosome2-1691441 INS	PRECISE	150	0	0	0/0	0/0	0/0	0/0	0/0	0/0	0/0	0/0	0/0	0/0	0/0	0/0	0/0	0/1
Chromosome2-1691616 DEL	PRECISE	57	0	0	0/0	0/0	0/0	0/0	0/1	0/0	0/0	0/0	0/0	0/0	0/0	0/0	0/0	0/0
Chromosome2-1698276 INS	PRECISE	1010	0	0	0/0	0/0	0/0	0/0	0/0	0/0	0/0	0/0	0/0	0/0	0/0	0/0	0/0	0/1
Chromosome2-1699119 DEL	PRECISE	1282	0.577	0	0/0	0/0	0/0	0/0	0/0	0/0	0/0	0/0	0/0	0/0	0/1	0/1	0/1	0/0
Chromosome2-1699763 DEL	PRECISE	63	0	0	0/0	0/0	0/0	0/0	0/1	0/0	0/0	0/0	0/0	0/0	0/0	0/0	0/0	0/0
Chromosome2-1708067 DEL	PRECISE	3467	0	0	0/0	0/0	0/0	0/0	0/1	0/0	0/0	0/0	0/0	0/0	0/0	0/0	0/0	0/0
Chromosome2-1716863 DEL	PRECISE	441	0	0	0/0	0/0	0/0	0/0	0/0	0/0	0/0	0/0	0/0	0/0	0/1	0/1	0/0	0/0
Chromosome2-1719154 INS	PRECISE	6381	0	0	0/0	0/0	0/0	0/0	0/0	0/0	0/0	0/0	0/0	0/0	1/1	0/0	0/0	0/0
Chromosome2-1719265 INS	PRECISE	5430	0	0	0/0	0/0	0/0	0/0	0/0	0/0	0/0	0/0	0/0	0/0	0/0	0/1	0/0	0/0
Chromosome2-1720181 INS	PRECISE	1199	0	0	0/0	0/0	0/0	0/0	0/0	0/0	0/0	0/0	0/0	0/0	0/1	0/0	0/0	0/0
Chromosome2-1721870 INS	PRECISE	8052	0	0	0/0	0/0	0/0	0/0	0/0	0/0	0/0	0/0	0/0	0/0	1/1	0/0	0/0	0/0
Chromosome2-1726996 INS	PRECISE	6944	0	0	0/0	0/0	0/0	1/1	0/0	0/0	0/0	0/0	0/0	0/0	0/0	0/0	0/0	0/0
Chromosome2-1728895 INS	PRECISE	998	0	0	0/0	0/0	0/0	0/0	0/0	0/0	0/1	0/0	0/0	0/0	0/0	0/0	0/0	0/0
Chromosome2-1730228 INS	PRECISE	5402	0	0	0/0	0/0	0/0	0/0	1/1	0/0	0/0	0/0	0/0	0/0	0/0	0/0	0/0	0/0
Chromosome2-1731900 DEL	PRECISE	1456	0	0	0/0	0/0	0/1	0/0	0/0	0/0	0/0	0/0	0/0	0/0	0/1	0/1	0/0	0/0
Chromosome2-1734045 INS	PRECISE	61	0	0	0/0	0/0	0/1	0/0	0/0	0/0	0/0	0/0	0/0	0/0	0/0	0/0	0/0	0/0
Chromosome2-1735693 DEL	PRECISE	436	0	0	0/0	0/0	0/1	0/0	0/0	0/0	0/0	0/0	0/0	0/0	0/1	0/1	0/0	0/0
Chromosome2-1737542 DEL	PRECISE	50	0	0	0/0	0/0	0/1	0/0	0/0	0/0	0/0	0/0	0/0	0/0	0/0	0/0	0/0	0/0
Chromosome2-1737758 INS	PRECISE	73	0	0	0/0	0/0	0/0	0/0	0/0	0/0	0/0	0/0	0/0	0/0	0/1	0/0	0/0	0/0
Chromosome2-1751168 INS	PRECISE	610	0	0	0/0	0/0	0/0	0/0	0/0	0/0	0/0	0/0	0/0	0/0	0/0	0/0	1/1	0/0

Chromosome2-1754274 DEL	PRECISE	10188	0	0	0/0	0/0	0/0	0/0	0/0	0/0	0/0	0/0	0/0	0/0	0/0	0/1	0/0	0/0
Chromosome2-1755823 DEL	PRECISE	9111	0	0	0/0	0/0	0/0	0/0	0/0	0/0	0/0	0/0	0/0	0/0	0/0	0/0	1/1	0/0
Chromosome2-1758583 INS	PRECISE	3236	0	0	0/0	0/0	0/0	0/0	0/0	0/0	0/0	0/0	0/0	0/0	0/0	0/0	0/0	1/1
Chromosome2-1763879 DEL	PRECISE	151	0	0	0/0	0/0	0/0	0/0	0/0	0/0	1/1	0/0	0/0	0/0	0/0	0/0	0/0	0/0
Chromosome2-1764213 INS	PRECISE	12757	0	0	0/0	0/0	0/0	0/0	0/0	0/0	0/0	0/0	0/0	0/0	0/0	1/1	0/0	0/0
Chromosome2-1764213 INS	PRECISE	10744	0	0	0/0	0/0	0/0	0/0	0/0	0/0	1/1	0/0	0/0	0/0	0/0	0/0	0/0	0/0
Chromosome2-1764213 INS	PRECISE	8101	0	0	0/0	0/0	0/0	0/1	0/0	0/0	0/0	0/0	0/0	0/0	0/0	0/0	0/0	0/0
Chromosome2-1764839 DEL	PRECISE	19762	0	0	0/0	0/0	0/0	0/0	0/0	0/0	0/0	0/0	0/0	0/0	0/0	0/1	0/0	0/0
Chromosome2-1765102 INS	PRECISE	59	0	0.5	0/0	0/0	0/1	0/1	0/0	0/0	0/1	0/0	0/0	0/0	0/0	0/1	0/0	0/0
Chromosome2-1765917 INS	PRECISE	415	0	0.577	0/0	0/0	0/0	0/1	1/1	0/1	0/0	0/0	0/0	0/0	0/0	0/0	0/0	0/0
Chromosome2-1766083 DEL	PRECISE	774	0	0	0/0	0/0	0/0	0/0	0/0	0/0	0/0	0/0	0/0	0/0	0/0	0/0	0/1	0/1
Chromosome2-1768550 DEL	PRECISE	1442	0	0	0/0	0/0	0/0	0/0	0/0	0/0	0/0	0/0	0/0	0/0	0/0	0/1	0/0	0/0
Chromosome2-1777634 INS	PRECISE	12816	0	0	0/0	0/0	0/0	0/0	0/0	0/0	0/0	0/0	0/0	0/0	0/0	1/1	0/0	0/0
Chromosome2-1777914 INS	PRECISE	6241	0	0	0/0	0/0	0/0	0/1	1/1	0/0	0/0	0/0	0/0	0/0	0/0	0/0	0/0	0/0
Chromosome2-1779608 INS	PRECISE	5266	0	0	0/0	0/0	0/0	0/0	0/0	0/0	0/0	0/0	0/0	0/0	0/0	0/0	0/0	0/1
Chromosome2-1779806 DEL	PRECISE	63	0	0	0/0	0/0	0/0	0/1	1/1	0/0	0/0	0/0	0/0	0/0	0/0	0/0	0/0	0/0
Chromosome2-1783080 DEL	PRECISE	108	0	0	0/0	0/0	0/0	0/1	1/1	0/0	0/0	0/0	0/0	0/0	0/1	0/0	0/0	0/0
Chromosome2-1791518 INS	PRECISE	324	0	0	0/0	0/0	0/0	0/0	0/0	0/0	0/0	0/0	0/0	0/0	0/0	0/1	0/0	0/0
Chromosome2-1793161 DEL	PRECISE	2319	0	0	0/0	0/0	0/0	0/0	0/1	0/0	0/0	0/0	0/0	0/0	0/0	0/0	0/0	0/0
Chromosome2-1807711 DEL	PRECISE	331	0	0	0/0	0/0	0/0	1/1	1/1	0/0	0/0	0/0	0/0	0/0	0/0	0/0	0/0	0/1
Chromosome2-1807724 INV	PRECISE	331	0	0	0/0	0/0	0/1	0/1	0/1	0/1	0/1	0/0	0/0	0/0	0/0	0/0	0/0	0/0
Chromosome2-1828118 INS	PRECISE	211	0	0	0/0	0/0	0/0	0/0	0/1	0/1	0/1	0/0	0/0	0/0	0/0	0/0	0/0	0/0
Chromosome2-1834636 DEL	PRECISE	59	0	0	0/0	0/0	0/0	0/0	1/1	0/0	0/0	0/0	0/0	0/0	0/0	0/0	0/0	0/0
Chromosome2-1835677 DEL	PRECISE	340	0	0	0/0	0/0	0/0	0/0	0/0	0/0	0/0	0/0	0/0	0/0	0/1	0/0	0/0	0/0
Chromosome2-1846086 DEL	PRECISE	68	0	0	0/0	0/0	0/0	0/0	0/1	0/0	0/0	0/0	0/0	0/0	0/0	0/0	0/0	0/0
Chromosome2-1855854 INS	PRECISE	1810	0	0	0/0	0/0	0/0	0/0	0/1	0/0	0/0	0/0	0/0	0/0	0/0	0/0	0/0	0/0
Chromosome2-1862624 DEL	PRECISE	5255	0.463	0.463	0/0	0/0	0/1	1/1	1/1	0/1	0/0	0/0	0/0	0/0	0/1	0/1	0/0	0/1
Chromosome2-1886143 DEL	PRECISE	10706	0	0	0/0	0/0	0/0	1/1	0/0	0/0	0/0	0/0	0/0	0/0	0/0	0/0	0/0	0/0
Chromosome2-1904226 DEL	PRECISE	211	0	0	0/0	0/0	0/0	1/1	0/0	0/0	0/0	0/0	0/0	0/0	0/0	0/0	0/0	0/0
Chromosome2-1919547 INS	PRECISE	12469	0	0	0/0	0/0	0/0	0/0	0/0	0/0	0/0	0/0	0/0	0/1	0/0	0/0	0/0	0/0
Chromosome2-1934464 INS	PRECISE	90	0	0	0/0	0/0	0/0	1/1	0/0	0/0	0/0	0/0	0/0	0/0	0/0	0/0	0/0	0/0
Chromosome2-1941631 BND	PRECISE	0	0	0	0/0	0/0	0/0	0/1	0/0	0/0	0/0	0/0	0/0	0/0	0/0	0/0	0/0	0/0
Chromosome2-1948979 DEL	PRECISE	1012	0	0	0/0	0/0	0/0	1/1	0/0	0/0	0/0	0/0	0/0	0/0	0/0	0/0	0/0	0/0
Chromosome2-1951285 DEL	PRECISE	390	0	0	0/0	0/0	0/0	0/0	0/0	0/0	0/0	0/0	0/0	0/0	0/1	0/1	0/1	0/0
Chromosome2-1952199 INS	PRECISE	60	0.816	0.516	0/0	0/0	0/0	0/0	0/0	0/0	0/0	0/0	0/0	0/0	0/1	0/1	0/1	0/0
Chromosome2-1952228 DEL	PRECISE	90	0	0	0/0	0/0	0/0	0/0	1/1	0/0	0/0	0/0	0/0	0/0	0/0	0/0	0/0	0/0
Chromosome2-1958815 DEL	PRECISE	12524	0	0	0/0	0/0	0/0	1/1	0/0	0/0	0/0	0/0	0/0	0/0	0/0	0/0	0/0	0/0
Chromosome2-1976552 DEL	PRECISE	155	0	0	0/0	0/0	0/1	0/0	1/1	0/1	0/0	0/0	0/0	0/0	0/0	0/0	0/0	0/1
Chromosome2-1981583 INS	PRECISE	106	0	0	0/0	0/0	0/0	0/0	0/1	0/0	0/0	0/0	0/0	0/0	0/0	0/0	0/0	0/0
Chromosome2-1982953 DEL	PRECISE	219	0	0	0/0	0/0	0/0	0/0	0/1	0/0	0/0	0/0	0/0	0/0	0/0	0/0	0/0	0/0
Chromosome2-1987185 INS	PRECISE	77	0	0.535	0/0	0/0	0/1	0/0	1/1	0/1	0/0	0/0	0/0	0/0	0/1	0/1	0/1	0/1
Chromosome2-1987874 DEL	PRECISE	868	0	0	0/0	0/0	0/0	0/0	0/1	0/0	0/0	0/0	0/0	0/0	0/0	0/0	0/0	0/0
Chromosome2-1988854 INS	PRECISE	74	0	0	0/0	0/0	0/0	0/0	0/1	0/0	0/0	0/0	0/0	0/0	0/0	0/0	0/0	0/0
Chromosome2-1991108 INS	PRECISE	11241	0	0	0/0	0/0	0/1	0/0	0/0	0/0	0/0	0/0	0/0	0/0	0/0	0/0	0/0	0/0
Chromosome2-1995394 DEL	PRECISE	249	0	0	0/0	0/0	0/1	0/0	1/1	0/1	0/0	0/0	0/0	0/0	0/1	0/1	0/1	0/1
Chromosome2-2020730 DEL	PRECISE	8328	0	0	0/0	0/0	0/0	0/0	0/0	0/0	0/1	0/0	0/0	0/0	0/0	0/0	0/0	0/0
Chromosome2-2037182 DEL	PRECISE	654	0	0	0/0	0/0	0/0	1/1	0/0	0/0	0/1	0/0	0/0	0/0	0/0	0/0	0/0	0/0
Chromosome2-2039764 DEL	PRECISE	108	0	0	0/0	0/0	0/0	0/0	0/0	0/0	0/1	0/0	0/0	0/0	0/0	0/0	0/0	0/0
Chromosome2-2043074 INS	PRECISE	74	0	0	0/0	0/0	0/0	1/1	0/0	0/0	1/1	0/0	0/0	0/0	0/0	0/0	0/0	0/0
Chromosome2-2069916 DEL	PRECISE	5230	0	0	0/0	0/0	0/0	1/1	0/0	0/0	0/1	0/0	0/0	0/0	0/0	0/0	0/0	0/0
Chromosome2-2078254 INS	PRECISE	7876	0	0	0/0	0/0	0/0	1/1	0/0	0/0	0/0	0/0	0/0	0/0	0/0	0/0	0/0	0/0
Chromosome2-2078260 BND	PRECISE	0	0	0	0/0	0/0	0/0	0/1	0/0	0/0	0/0	0/0	0/0	0/0	0/0	0/0	0/0	0/0
Chromosome2-2078260 BND	PRECISE	0	0	0	0/0	0/0	0/0	0/1	0/0	0/0	0/0	0/0	0/0	0/0	0/0	0/0	0/0	0/0
Chromosome2-2088508 INS	PRECISE	7474	0	0	0/0	0/0	0/0	1/1	0/0	0/0	0/0	0/0	0/0	0/0	0/0	0/0	0/0	0/0
Chromosome2-2088508 INS	PRECISE	1204	0	0	0/0	0/0	0/0	0/0	0/0	0/0	0/1	0/0	0/0	0/0	0/0	0/0	0/0	0/0
Chromosome2-2088515 BND	PRECISE	0	0	0	0/0	0/0	0/0	0/1	0/0	0/0	0/0	0/0	0/0	0/0	0/0	0/0	0/0	0/0
Chromosome2-2096853 DEL	PRECISE	1617	0	0	0/0	0/0	0/0	0/0	1/1	0/0	0/1	0/0	0/0	0/0	0/0	0/0	0/0	0/0
Chromosome2-2100332 INS	PRECISE	2139	0	0	0/0	0/0	0/0	0/0	0/0	0/0	0/0	0/0	0/0	0/0	0/0	0/0	0/0	0/1
Chromosome2-2101939 DEL	PRECISE	460	0	0	0/0	0/0	0/0	0/0	0/0	0/0	0/0	0/0	0/0	0/0	0/1	0/1	0/1	0/0
Chromosome2-2104287 DEL	PRECISE	131	0	0	0/0	0/0	0/0	0/0	0/0	0/0	0/0	0/0	0/0	0/0	0/1	0/1	0/1	0/0
Chromosome2-2111365 INS	PRECISE	207	0	0	0/0	0/0	0/0	0/0	0/0	0/0	0/0	0/0	0/0	0/0	0/0	0/1	0/0	0/0
Chromosome2-2112790 BND	PRECISE	0	0.577	0	0/0	0/0	0/0	0/0	0/1	0/0	0/1	0/0	0/0	0/0	0/0	0/0	0/0	0/0
Chromosome2-2113741 INS	PRECISE	307	0	0	0/0	0/0	0/0	0/1	0/0	0/0	0/1	0/0	0/0	0/0	0/0	0/0	0/0	0/0
Chromosome2-2119147 DEL	PRECISE	3976	0	0	0/0	0/0	0/0	0/1	0/0	0/0	0/1	0/0	0/0	0/0	0/0	0/0	0/0	0/0
Chromosome2-2123700 DEL	PRECISE	264	0	0	0/0	0/0	0/1	0/1	0/1	0/1	0/1	0/0	0/0	0/0	0/0	0/0	0/0	0/0
Chromosome2-2123744 DEL	PRECISE	1493	0	0	0/0	0/0	0/0	0/0	0/0	0/0	0/0	0/0	0/0	0/0	0/0	0/1	0/0	0/0
Chromosome2-2125340 DEL	PRECISE	9879	0	0	0/0	0/0	0/0	0/0	0/0	0/0	0/0	0/0	0/0	0/0	0/0	0/1	0/0	0/0
Chromosome2-2126763 DEL	PRECISE	5796	0	0	0/0	0/0	0/1	0/1	1/1	0/1	0/0	0/0	0/0	0/0	0/1	0/0	0/1	0/1
Chromosome2-2132559 INS	PRECISE	2948	0	0	0/0	0/0	0/0	0/0	0/0	0/0	0/0	0/0	0/0	0/0	0/0	0/0	0/0	0/0
Chromosome2-2134027 INS	PRECISE	4853	0	0	0/0	0/0	0/0	0/0	0/0	0/0	0/0	0/0	0/0	0/0	0/0	0/0	0/0	0/1
Chromosome2-2135664 DEL	PRECISE	52	0	0	0/0	0/0	0/0	0/0	0/0	0/0	0/0	0/0	0/0	0/0	0/0	0/1	0/0	0/0
Chromosome2-2136422 BND	PRECISE	0	0	0	0/0	0/0	0/0	0/1	0/0	0/0	0/0	0/0	0/0	0/0	0/0	0/0	0/0	0/0
Chromosome2-2139591 BND	PRECISE	0	0	0	0/0	0/0	0/0	0/1	0/1	0/0	0/1	0/0	0/0	0/0	0/0	0/0	0/0	0/0

Chromosome2-2141652 DEL	PRECISE	646	0	0	0/0	0/0	0/0	0/0	0/1	0/0	0/0	0/0	0/0	0/0	0/0	0/0	0/0	0/0
Chromosome2-2144484 DEL	PRECISE	802	0	0	0/0	0/0	0/0	0/1	0/1	0/0	0/0	0/0	0/0	0/0	0/0	0/0	0/0	0/0
Chromosome2-2145781 INS	PRECISE	55	0	0	0/0	0/0	0/0	0/1	0/0	0/0	0/0	0/0	0/0	0/0	0/0	0/0	0/0	0/0
Chromosome2-2147498 DEL	PRECISE	1164	0	0	0/0	0/0	0/0	0/0	0/1	0/0	0/0	0/0	0/0	0/0	0/0	0/0	0/0	0/0
Chromosome2-2151562 INS	PRECISE	5442	0	0	0/0	0/0	0/0	1/1	0/0	0/0	0/0	0/0	0/0	0/0	0/0	0/0	0/0	0/0
Chromosome2-2153236 DEL	PRECISE	232	0	0	0/0	0/0	0/1	0/1	0/1	0/1	0/1	0/0	0/0	0/0	0/0	0/0	0/0	0/0
Chromosome2-2154310 INS	PRECISE	88	0	0	0/0	0/0	0/0	0/0	0/0	0/0	0/0	0/0	0/0	0/0	0/1	0/0	0/0	0/0
Chromosome2-2162884 DEL	PRECISE	556	0	0	0/0	0/0	0/0	0/0	0/0	0/0	0/0	0/0	0/0	0/0	0/1	0/1	0/1	0/0
Chromosome2-2167622 INS	PRECISE	65	0	0	0/0	0/0	0/0	0/0	0/1	0/1	0/1	0/0	0/0	0/0	0/0	0/0	0/0	0/1
Chromosome2-2171038 INS	PRECISE	10792	0	0	0/0	0/0	0/0	0/0	0/0	0/0	0/1	0/0	0/0	0/0	0/0	0/0	0/0	0/0
Chromosome2-2174786 DEL	PRECISE	324	0	0	0/0	0/0	0/0	0/1	0/0	0/0	0/1	0/0	0/0	0/0	0/0	0/0	0/0	0/0
Chromosome2-2178468 INS	PRECISE	487	0	0.707	0/0	0/0	0/0	0/0	0/0	0/0	0/0	0/0	0/0	0/0	0/1	0/0	0/1	0/0
Chromosome2-2181231 DEL	PRECISE	1099	0	0	0/0	0/0	0/1	0/0	0/1	0/0	0/0	0/0	0/0	0/0	0/0	0/0	0/0	0/0
Chromosome2-2186938 INS	PRECISE	137	0	0	0/0	0/0	0/0	0/1	0/0	0/0	0/0	0/0	0/0	0/0	0/0	0/0	0/0	0/0
Chromosome2-2194619 DEL	PRECISE	197	0	0	0/0	0/0	0/0	0/0	0/0	0/0	0/0	0/0	0/0	0/0	0/1	0/0	0/0	0/0
Chromosome2-2195476 INS	PRECISE	552	0	0	0/0	0/0	0/0	0/0	0/0	0/0	0/0	0/0	0/0	0/0	0/0	0/1	0/0	0/0
Chromosome2-2196986 INS	PRECISE	5746	0	0	0/0	0/0	0/0	0/0	0/1	0/0	0/0	0/0	0/0	0/0	0/0	0/0	0/0	0/0
Chromosome2-2212091 DEL	PRECISE	167	0	0	0/0	0/0	0/0	0/0	0/0	0/0	0/0	0/0	0/0	0/0	0/1	0/1	0/1	0/1
Chromosome2-2217790 INS	PRECISE	103	0	0.577	0/0	0/0	0/0	0/0	0/0	0/0	0/0	0/0	0/0	0/0	0/1	0/1	0/1	0/1
Chromosome2-2219656 INS	PRECISE	5151	0	0	0/0	0/0	0/0	0/0	0/0	0/0	0/0	0/0	0/0	0/0	0/0	0/1	0/0	0/0
Chromosome2-2220782 DEL	PRECISE	55	0	0	0/0	0/0	0/0	0/0	0/0	0/0	0/0	0/0	0/0	0/0	0/0	0/0	0/0	0/1
Chromosome2-2221392 INS	PRECISE	720	0	0	0/0	0/0	0/0	0/0	0/0	0/0	0/0	0/0	0/0	0/0	0/0	0/0	0/0	0/1
Chromosome2-2231808 INS	PRECISE	673	0	0	0/0	0/0	0/0	0/1	0/0	0/0	0/0	0/0	0/0	0/0	0/0	0/0	0/0	0/0
Chromosome2-2232233 DEL	PRECISE	231	0	0	0/0	0/0	0/0	0/0	0/0	0/0	0/0	0/0	0/0	0/0	0/1	0/0	0/1	0/0
Chromosome2-2232464 INS	PRECISE	2636	0	0	0/0	0/0	0/0	0/0	0/0	0/0	0/0	0/0	0/0	0/0	0/0	0/0	0/0	0/0
Chromosome2-2234859 INS	PRECISE	183	0	0.463	0/0	0/0	0/0	0/0	0/0	0/0	0/0	0/0	0/0	0/0	0/0	0/1	0/0	0/0
Chromosome2-2235865 INS	PRECISE	58	0	0	0/0	0/0	0/0	0/0	0/0	0/0	0/0	0/0	0/0	0/0	0/0	0/1	0/0	0/0
Chromosome2-2239235 DEL	PRECISE	1242	0	0	0/0	0/0	0/0	0/0	0/0	0/0	0/0	0/0	0/0	0/0	0/1	0/1	0/1	0/0
Chromosome2-2239807 INS	PRECISE	64	0	0	0/0	0/0	0/0	0/0	0/0	0/0	0/0	0/0	0/0	0/0	0/0	0/0	0/0	0/1
Chromosome2-2240886 DEL	PRECISE	59	0	0	0/0	0/0	0/0	0/0	0/0	0/0	0/0	0/0	0/0	0/0	0/1	0/1	0/1	0/0
Chromosome2-2242528 DEL	PRECISE	406	0	0	0/0	0/0	0/0	0/1	0/0	0/0	0/0	0/0	0/0	0/0	0/0	0/0	0/0	0/0
Chromosome2-2244474 DEL	PRECISE	3724	0	0	0/0	0/0	0/1	0/1	0/1	0/1	0/1	0/0	0/0	0/0	0/0	0/0	0/0	0/0
Chromosome2-2250562 DEL	PRECISE	144	0	0	0/0	0/0	0/0	0/1	0/1	0/1	0/1	0/0	0/0	0/0	0/0	0/0	0/0	0/0
Chromosome2-2250854 INS	PRECISE	5406	0	0	0/0	0/0	0/0	0/0	0/0	0/0	0/1	0/0	0/0	0/0	0/0	0/0	0/0	0/0
Chromosome2-2265628 DEL	PRECISE	688	0	0	0/0	0/0	0/0	0/0	0/0	0/0	0/0	0/0	0/0	0/0	0/1	0/1	0/1	0/1
Chromosome2-2269051 DEL	PRECISE	54	0	0	0/0	0/0	0/0	0/0	0/0	0/0	0/0	0/0	0/0	0/0	0/1	0/1	0/0	0/0
Chromosome2-2277992 DEL	PRECISE	368	0	0	0/0	0/0	0/0	0/0	0/0	0/0	0/0	0/0	0/0	0/0	0/0	0/1	0/1	0/0
Chromosome2-2282530 DEL	PRECISE	60	0	0	0/0	0/0	0/0	0/0	0/0	0/0	0/0	0/0	0/0	0/0	0/0	0/0	0/0	0/1
Chromosome2-2291252 DEL	PRECISE	52	0	0	0/0	0/0	0/0	0/0	0/1	0/1	0/1	0/0	0/0	0/0	0/0	0/0	0/0	0/0
Chromosome2-2300860 INS	PRECISE	210	0	0.577	0/0	0/0	0/0	0/0	0/0	0/0	0/0	0/0	0/0	0/0	0/1	0/1	1/1	0/0
Chromosome2-2303001 INS	PRECISE	2180	0	0	0/0	0/0	0/1	0/0	0/0	0/0	0/0	0/0	0/0	0/0	0/0	0/0	0/0	0/0
Chromosome2-2312832 DEL	PRECISE	164	0	0	0/0	0/0	0/0	0/1	0/0	0/0	0/1	0/0	0/0	0/0	0/1	0/0	0/1	0/1
Chromosome2-2317709 INS	PRECISE	624	0	0	0/0	0/0	0/1	0/0	0/1	0/0	0/0	0/0	0/0	0/0	0/0	0/0	0/0	0/0
Chromosome2-2322302 INS	PRECISE	344	0	0.577	0/0	0/0	0/1	0/1	0/1	0/0	0/0	0/0	0/0	0/0	0/0	0/0	0/0	0/0
Chromosome2-2323605 INS	PRECISE	3798	0	0.707	0/0	0/0	0/1	0/0	0/1	0/0	0/0	0/0	0/0	0/0	0/0	0/0	0/0	0/0
Chromosome2-2323638 INS	PRECISE	82	0	0	0/0	0/0	0/1	0/1	0/1	0/0	0/0	0/0	0/0	0/0	0/0	0/0	0/0	0/0
Chromosome2-2331722 INS	PRECISE	314	0	0	0/0	0/0	0/0	0/1	0/0	0/0	0/0	0/0	0/0	0/0	0/0	0/0	0/0	0/0
Chromosome2-2335056 INS	PRECISE	5451	0	0	0/0	0/0	0/0	0/0	0/0	0/0	0/0	0/0	0/0	0/0	0/0	0/0	0/0	1/1
Chromosome2-2337380 INS	PRECISE	52	0	0	0/0	0/0	0/0	0/0	0/0	0/0	0/0	0/0	0/0	0/0	0/0	0/1	0/0	0/0
Chromosome2-2337940 INS	PRECISE	650	0	0	0/0	0/0	0/0	0/0	0/0	0/0	0/0	0/0	0/0	0/0	0/0	0/0	0/0	0/1
Chromosome2-2341512 INS	PRECISE	723	0	0.707	0/0	0/0	0/0	0/0	0/0	0/0	0/0	0/0	0/0	0/0	0/1	0/0	0/1	0/0
Chromosome2-2342837 INS	PRECISE	5000	0	0.707	0/0	0/0	1/1	0/0	1/1	0/0	0/0	0/0	0/0	0/0	0/0	0/0	0/0	0/0
Chromosome2-2342837 BND	PRECISE	0	0	0	0/0	0/0	0/0	0/0	0/1	0/0	0/0	0/0	0/0	0/0	0/0	0/0	0/0	0/0
Chromosome2-2344846 INS	PRECISE	1585	0	0	0/0	0/0	0/0	0/0	0/0	0/0	0/0	0/0	0/0	0/0	0/0	0/0	0/0	0/1
Chromosome2-2359284 DUP	PRECISE	3564	0	0	0/0	0/0	0/0	0/0	0/1	0/0	0/0	0/0	0/0	0/0	0/0	0/0	0/0	0/0
Chromosome2-2359379 INS	PRECISE	252	0	0	0/0	0/0	0/0	0/0	0/0	0/0	0/0	0/0	0/0	0/0	0/1	0/0	0/1	0/0
Chromosome2-2383569 DEL	PRECISE	3056	0	0	0/0	0/0	0/0	0/0	0/0	0/0	0/0	0/0	0/0	0/0	0/0	0/1	0/0	0/0
Chromosome2-2386625 DEL	PRECISE	23673	0	0	0/0	0/0	0/0	1/1	1/1	1/1	0/1	0/0	0/0	0/0	0/0	0/0	0/0	0/0
Chromosome2-2411394 INS	PRECISE	167	0	0	0/0	0/0	0/0	0/1	1/1	0/1	0/0	0/0	0/0	0/0	0/0	0/0	0/0	0/0
Chromosome2-2417648 DEL	PRECISE	55	0	0	0/0	0/0	0/0	0/1	0/0	0/0	0/0	0/0	0/0	0/0	0/0	0/0	0/0	0/0
Chromosome2-2418450 DEL	PRECISE	1900	0	0	0/0	0/0	0/0	0/0	0/0	0/0	0/0	0/0	0/0	0/0	0/0	1/1	0/0	0/0
Chromosome2-2421250 INS	PRECISE	812	0	0	0/0	0/0	0/0	0/0	0/0	0/0	0/0	0/0	0/0	0/0	0/0	0/1	0/0	0/0
Chromosome2-2424577 DEL	PRECISE	69	0	0	0/0	0/0	0/0	0/1	0/0	0/0	0/1	0/0	0/0	0/0	0/0	0/1	0/0	0/0
Chromosome2-2427256 INS	PRECISE	132	0	0	0/0	0/0	0/0	0/0	0/0	0/0	0/1	0/0	0/0	0/0	0/0	0/0	0/0	0/0
Chromosome2-2440968 INS	PRECISE	132	0	0	0/0	0/0	0/0	0/0	0/0	0/0	0/0	0/0	0/0	0/0	0/0	0/1	0/0	0/0
Chromosome2-2448673 DEL	PRECISE	1021	0	0	0/0	0/0	0/1	0/0	0/1	0/0	0/0	0/0	0/0	0/0	0/1	0/0	0/0	0/0
Chromosome2-2451484 DEL	PRECISE	1594	0	0	0/0	0/0	0/0	0/0	0/0	0/0	0/0	0/0	0/0	0/0	0/1	0/0	0/0	0/0
Chromosome2-2451751 DEL	PRECISE	91	0	0	0/0	0/0	0/0	0/0	0/0	0/0	0/0	0/0	0/0	0/0	0/1	0/1	0/1	0/0
Chromosome2-2456658 DEL	PRECISE	80	0	0	0/0	0/0	0/0	0/0	0/0	0/0	0/0	0/0	0/0	0/0	0/0	0/1	0/0	0/0
Chromosome2-2462630 BND	PRECISE	0	0	0	0/0	0/0	0/0	0/0	0/0	0/0	0/0	0/0	0/0	0/0	1/1	0/0	0/0	0/0
Chromosome2-2473673 INS	PRECISE	2156	0	0	0/0	0/0	0/0	0/0	0/1	0/0	0/0	0/0	0/0	0/0	0/0	0/0	0/0	0/0
Chromosome2-2475739 DEL	PRECISE	1365	0	0	0/0	0/0	0/1	0/0	0/1	0/0	0/0	0/0	0/0	0/0	0/1	0/1	0/1	0/1
Chromosome2-2477349 DEL	PRECISE	2110	0	0	0/0	0/0	0/1	0/0	0/1	0/0	0/0	0/0	0/0	0/0	0/1	0/1	0/1	0/1

Chromosome2-2481868 INS	PRECISE	247	0	0.707	0/0	0/0	0/0	0/0	0/0	0/0	0/0	0/0	0/0	0/0	0/0	0/1	0/1	0/0
Chromosome2-2486016 DEL	PRECISE	107	0	0	0/0	0/0	0/0	0/0	0/0	0/0	0/0	0/0	0/0	0/0	0/1	0/1	0/1	0/0
Chromosome2-2486613 INS	PRECISE	122	0	0	0/0	0/0	0/0	0/1	0/0	0/0	0/0	0/0	0/0	0/0	0/0	0/0	0/0	0/0
Chromosome2-2491217 INS	PRECISE	7129	0	0	0/0	0/0	0/0	0/0	0/0	0/0	0/0	0/0	0/0	0/0	0/0	1/1	0/0	0/0
Chromosome2-2491444 DEL	PRECISE	61	0	0	0/0	0/0	0/0	0/0	0/0	0/0	0/0	0/0	0/0	0/0	0/1	0/0	0/1	0/0
Chromosome2-2492247 DEL	PRECISE	289	0	0	0/0	0/0	0/0	0/0	0/0	0/0	0/0	0/0	0/0	0/0	0/1	0/0	0/1	0/0
Chromosome2-2500899 DEL	PRECISE	52	0	0	0/0	0/0	0/0	0/0	0/0	0/0	0/0	0/0	0/0	0/0	0/1	0/1	0/1	0/0
Chromosome2-2508799 INS	PRECISE	69	0	0	0/0	0/0	0/0	0/0	0/0	0/0	0/1	0/0	0/0	0/0	0/0	0/0	0/0	0/0
Chromosome2-2519884 INS	PRECISE	239	0	0.577	0/0	0/0	0/1	0/0	1/1	0/1	0/1	0/0	0/0	0/0	0/0	0/0	0/0	0/0
Chromosome2-2522778 INS	PRECISE	860	0	0	0/0	0/0	0/0	0/0	0/0	0/0	0/0	0/0	0/0	0/0	0/0	0/0	0/1	0/0
Chromosome2-2525439 DEL	PRECISE	321	0	0	0/0	0/0	0/0	0/1	0/0	0/0	0/0	0/0	0/0	0/0	0/0	0/0	0/0	0/0
Chromosome2-2525537 INS	PRECISE	57	0	0	0/0	0/0	0/0	0/0	0/0	0/0	0/0	0/0	0/0	0/0	0/1	0/0	0/1	0/0
Chromosome2-2527663 DEL	PRECISE	782	0	0	0/0	0/0	0/0	0/0	0/0	0/0	0/0	0/0	0/0	0/0	0/0	0/1	0/1	0/0
Chromosome2-2528565 DEL	PRECISE	1882	0	0	0/0	0/0	0/0	0/0	0/0	0/0	0/0	0/0	0/0	0/0	0/0	0/1	0/1	0/0
Chromosome2-2529423 INS	PRECISE	1551	0	0	0/0	0/0	0/1	0/0	0/1	0/0	0/0	0/0	0/0	0/0	0/0	0/0	0/0	0/0
Chromosome2-2533852 INS	PRECISE	5313	0	0	0/0	0/0	0/0	0/0	0/0	0/0	0/1	0/0	0/0	0/0	0/0	0/0	0/0	0/0
Chromosome2-2534333 INS	PRECISE	693	0	0	0/0	0/0	0/0	0/0	0/0	0/0	0/0	0/0	0/0	0/0	0/0	0/1	0/0	0/0
Chromosome2-2539947 DEL	PRECISE	134	0	0	0/0	0/0	0/0	0/0	0/0	0/0	0/0	0/0	0/0	0/0	0/0	0/1	0/1	0/0
Chromosome2-2541357 INS	PRECISE	2009	0	0	0/0	0/0	0/0	0/0	0/0	0/0	0/0	0/0	0/0	0/0	0/0	0/1	0/0	0/0
Chromosome2-2544865 DEL	PRECISE	137	0	0	0/0	0/0	0/0	0/1	0/1	0/1	0/0	0/0	0/0	0/0	0/1	0/1	0/1	0/1
Chromosome2-2547780 INS	PRECISE	1741	0	0	0/0	0/0	0/0	0/0	0/0	0/0	0/0	0/0	0/0	0/0	0/1	0/0	0/0	0/0
Chromosome2-2547780 INS	PRECISE	3070	0	0	0/0	0/0	0/1	0/0	0/0	0/0	0/0	0/0	0/0	0/0	0/0	0/0	0/0	0/0
Chromosome2-2556904 DEL	PRECISE	2056	0	0	0/0	0/0	0/1	0/0	0/0	0/0	0/0	0/0	0/0	0/0	0/0	0/0	0/0	0/0
Chromosome2-2564410 INS	PRECISE	148	0	0	0/0	0/0	0/1	0/0	0/0	0/0	0/0	0/0	0/0	0/0	0/0	0/0	0/0	0/0
Chromosome2-2580569 DEL	PRECISE	78	0	0	0/0	0/0	0/0	0/0	0/0	0/0	0/0	0/0	0/0	0/0	0/1	0/1	0/1	0/0
Chromosome2-2582195 INS	PRECISE	208	0	0	0/0	0/0	0/0	0/0	0/0	0/0	0/0	0/0	0/0	0/0	0/0	0/0	0/0	0/1
Chromosome2-2582835 DEL	PRECISE	67	0	0	0/0	0/0	0/0	0/0	0/0	0/0	0/0	0/0	0/0	0/0	0/0	0/1	0/0	0/0
Chromosome2-2589981 DEL	PRECISE	70	0	0	0/0	0/0	0/0	0/0	0/1	0/1	0/0	0/0	0/0	0/0	0/0	0/0	0/0	0/0
Chromosome2-2599389 INS	PRECISE	4031	0	0	0/0	0/0	0/0	0/1	0/0	0/0	0/0	0/0	0/0	0/0	0/0	0/0	0/0	0/0
Chromosome2-2599397 BND	PRECISE	0	0	0	0/0	0/0	0/0	0/1	0/0	0/0	0/0	0/0	0/0	0/0	0/0	0/0	0/0	0/0
Chromosome2-2600039 INS	PRECISE	8430	0	0	0/0	0/0	0/0	1/1	0/0	0/0	0/0	0/0	0/0	0/0	0/0	0/0	0/0	0/0
Chromosome2-2600503 INS	PRECISE	7660	0	0	0/0	0/0	0/0	0/0	0/0	0/0	0/0	0/0	0/0	0/0	0/1	0/0	0/0	0/0
Chromosome2-2600675 DEL	PRECISE	8914	0	0	0/0	0/0	0/1	0/1	0/1	0/0	0/0	0/0	0/0	0/0	0/0	0/0	0/0	0/0
Chromosome2-2607621 INS	PRECISE	276	0	0	0/0	0/0	0/0	0/0	0/0	0/0	0/0	0/0	0/0	0/0	0/1	0/0	0/0	0/0
Chromosome2-2609688 DEL	PRECISE	608	0	0	0/0	0/0	0/1	0/1	0/1	0/0	0/0	0/0	0/0	0/0	0/0	0/0	0/0	0/0
Chromosome2-2614034 INS	PRECISE	3817	0	0	0/0	0/0	0/0	0/0	0/0	0/0	0/0	0/0	0/0	0/0	0/0	0/1	0/0	0/0
Chromosome2-2620148 INS	PRECISE	631	0	0	0/0	0/0	0/0	0/1	0/0	0/0	0/0	0/0	0/0	0/0	0/0	0/0	0/0	0/0
Chromosome2-2623368 INS	PRECISE	1410	0	0	0/0	0/0	0/0	0/1	0/0	0/0	0/0	0/0	0/0	0/0	0/0	0/0	0/0	0/0
Chromosome2-2625760 INS	PRECISE	2126	0	0	0/0	0/0	0/0	0/0	0/0	0/0	0/0	0/0	0/0	0/0	0/1	0/0	0/0	0/0
Chromosome2-2626474 INS	PRECISE	3850	0	0	0/0	0/0	0/0	0/1	0/0	0/0	0/0	0/0	0/0	0/0	0/0	0/0	0/0	0/0
Chromosome2-2626993 BND	PRECISE	0	0	0	0/0	0/0	0/0	0/0	0/0	0/0	0/0	0/0	0/0	0/0	0/1	0/0	0/0	0/0
Chromosome2-2630690 DEL	PRECISE	82	0	0	0/0	0/0	0/0	0/1	0/1	0/1	0/1	0/0	0/0	0/0	0/1	0/0	0/0	0/1
Chromosome2-2633549 INS	PRECISE	352	0	0	0/0	0/0	0/0	0/1	0/0	0/0	0/0	0/0	0/0	0/0	0/0	0/0	0/0	0/0
Chromosome2-2636802 DEL	PRECISE	299	0	0	0/0	0/0	0/0	0/0	0/0	0/0	0/0	0/0	0/0	0/0	0/1	0/0	0/0	0/0
Chromosome2-2642626 INS	PRECISE	702	0	0	0/0	0/0	0/0	0/0	0/0	0/0	0/0	0/0	0/0	0/0	0/0	0/0	0/0	0/1
Chromosome2-2646062 DEL	PRECISE	52	0	0	0/0	0/0	0/0	0/0	0/0	0/0	0/0	0/0	0/0	0/0	0/0	0/0	0/1	0/0
Chromosome2-2650020 INS	PRECISE	52	0	0.577	0/0	0/0	0/0	0/0	0/0	0/0	0/0	0/0	0/0	0/0	0/0	0/1	0/1	0/1
Chromosome2-2650510 INS	PRECISE	1262	0	0	0/0	0/0	0/0	0/0	0/0	0/0	0/0	0/0	0/0	0/0	0/0	0/0	0/1	0/0
Chromosome2-2652201 INS	PRECISE	1726	0	0	0/0	0/0	0/0	0/0	0/0	0/0	0/0	0/0	0/0	0/0	0/0	0/0	0/0	0/1
Chromosome2-2654190 INS	PRECISE	718	0	0	0/0	0/0	0/0	0/0	0/0	0/0	0/0	0/0	0/0	0/0	0/0	0/1	0/0	0/0
Chromosome2-2654220 INS	PRECISE	16020	0	0	0/0	0/0	0/0	0/1	0/0	0/0	0/0	0/0	0/0	0/0	0/0	0/0	0/0	0/0
Chromosome2-2662390 DEL	PRECISE	804	0	0	0/0	0/0	0/0	0/1	0/0	0/0	0/0	0/0	0/0	0/0	0/0	0/0	0/0	0/0
Chromosome2-2671580 DEL	PRECISE	55	0	0	0/0	0/0	0/0	0/0	0/0	0/0	0/0	0/0	0/0	0/0	0/0	0/0	0/0	0/1
Chromosome2-2674351 DEL	PRECISE	657	0	0	0/0	0/0	0/1	0/1	1/1	0/1	0/0	0/0	0/0	0/0	0/0	0/0	0/0	0/0
Chromosome2-2676676 INS	PRECISE	5044	0	0.707	0/0	0/0	1/1	0/0	0/1	0/0	0/0	0/0	0/0	0/0	0/0	0/0	0/0	0/0
Chromosome2-2677760 DEL	PRECISE	132	0	0	0/0	0/0	0/1	0/0	0/1	0/0	0/0	0/0	0/0	0/0	0/0	0/0	0/0	0/0
Chromosome2-2683679 DEL	PRECISE	1149	0	0	0/0	0/0	0/1	0/0	0/0	0/0	0/0	0/0	0/0	0/0	0/0	0/0	0/0	0/0
Chromosome2-2686711 INS	PRECISE	2505	0	0	0/0	0/0	0/0	0/0	0/0	0/0	0/0	0/0	0/0	0/0	0/0	0/0	0/0	0/1
Chromosome2-2689255 DEL	PRECISE	3858	0	0	0/0	0/0	0/1	0/0	0/1	0/0	0/0	0/0	0/0	0/0	0/0	0/0	0/0	0/0
Chromosome2-2694374 DEL	PRECISE	71	0	0	0/0	0/0	0/0	0/0	0/0	0/1	0/0	0/0	0/0	0/0	0/0	0/0	0/0	0/1
Chromosome2-2695605 DEL	PRECISE	5143	0	0	0/0	0/0	0/1	1/1	1/1	0/1	0/0	0/0	0/0	0/0	0/0	0/0	0/0	0/1
Chromosome2-2704495 INS	PRECISE	2559	0	0	0/0	0/0	0/0	0/0	0/0	0/0	0/0	0/0	0/0	0/0	0/1	0/0	0/0	0/0
Chromosome2-2706381 INS	PRECISE	5452	0	0	0/0	0/0	0/0	0/0	0/0	0/0	0/0	0/0	0/0	0/0	0/0	0/0	0/0	1/1
Chromosome2-2708935 INS	PRECISE	9388	0	0	0/0	0/0	0/0	0/1	0/0	0/0	0/0	0/0	0/0	0/0	0/0	0/0	0/0	0/0
Chromosome2-2711368 INS	PRECISE	6577	0	0	0/0	0/0	0/0	0/1	0/0	0/0	0/0	0/0	0/0	0/0	0/0	0/0	0/0	0/0
Chromosome2-2713157 INS	PRECISE	3138	0	0	0/0	0/0	0/0	0/0	0/0	0/0	0/1	0/0	0/0	0/0	0/0	0/0	0/0	0/0
Chromosome2-2713216 INS	PRECISE	6079	0	0	0/0	0/0	0/0	0/0	0/0	0/0	0/0	0/0	0/0	0/0	0/0	0/0	0/0	0/1
Chromosome2-2715060 INS	PRECISE	1610	0	0	0/0	0/0	0/0	0/0	0/0	0/0	0/0	0/0	0/0	0/0	0/0	0/0	0/0	0/1
Chromosome2-2716303 DEL	PRECISE	243	0	0	0/0	0/0	0/1	0/0	0/0	0/0	0/0	0/0	0/0	0/0	0/0	0/0	0/0	0/0
Chromosome2-2717592 DEL	PRECISE	53	0	0	0/0	0/0	0/1	0/0	0/1	0/0	0/0	0/0	0/0	0/0	0/0	0/0	0/0	0/0
Chromosome2-2719726 INS	PRECISE	1599	0	0.707	0/0	0/0	0/1	0/0	0/1	0/0	0/0	0/0	0/0	0/0	0/0	0/0	0/0	0/0
Chromosome2-2733228 DEL	PRECISE	5918	0	0	0/0	0/0	0/1	0/0	0/1	0/0	0/0	0/0	0/0	0/0	0/0	0/0	0/0	0/0
Chromosome2-2735234 DEL	PRECISE	788	0	0	0/0	0/0	0/0	0/0	0/0	0/0	0/0	0/0	0/0	0/0	0/0	0/0	0/0	0/1

Chromosome2-2752794 INS	PRECISE	165	0	0.5	0/0	0/0	0/0	0/0	0/1	0/0	0/0	0/0	0/0	0/0	0/0	0/0	0/0	0/0
Chromosome2-2753107 DEL	PRECISE	144	0	0	0/0	0/0	0/0	1/1	0/1	0/0	0/0	0/0	0/0	0/0	0/0	0/0	0/0	0/0
Chromosome2-2756591 INS	PRECISE	344	0	0	0/0	0/0	0/0	0/0	0/0	0/0	0/0	0/0	0/0	0/0	0/0	0/1	0/0	0/0
Chromosome2-2763604 DEL	PRECISE	288	0	0	0/0	0/0	0/1	1/1	0/1	0/0	0/0	0/0	0/0	0/0	0/0	0/1	0/0	0/0
Chromosome2-2764014 INS	PRECISE	139	0.488	0.756	0/0	0/0	0/1	1/1	0/1	0/0	0/1	0/0	0/0	0/0	0/0	0/1	0/0	0/1
Chromosome2-2766537 DEL	PRECISE	1252	0	0	0/0	0/0	0/0	0/0	0/0	0/0	0/0	0/0	0/0	0/0	1/1	0/0	0/1	0/0
Chromosome2-2772010 DEL	PRECISE	191	0	0	0/0	0/0	0/1	1/1	1/1	0/1	0/1	0/0	0/0	0/0	0/1	0/1	0/1	0/1
Chromosome2-2786118 DEL	PRECISE	3176	0	0	0/0	0/0	0/1	0/1	0/1	0/0	0/0	0/0	0/0	0/0	0/1	0/1	0/1	0/1
Chromosome2-2786941 DEL	PRECISE	2354	0	0	0/0	0/0	0/0	0/1	0/1	0/1	0/0	0/0	0/0	0/0	0/0	0/0	0/0	0/0
Chromosome2-2800635 INS	PRECISE	149	0	0	0/0	0/0	0/0	0/0	0/0	0/0	0/0	0/0	0/0	0/0	1/1	0/0	0/0	0/0
Chromosome2-2800963 DEL	PRECISE	922	0	0	0/0	0/0	0/0	0/0	0/0	0/0	0/0	0/0	0/0	0/0	0/1	0/0	0/0	0/0
Chromosome2-2801821 DEL	PRECISE	319	0	0	0/0	0/0	0/1	0/0	0/1	0/0	0/0	0/0	0/0	0/0	0/0	0/0	0/0	0/0
Chromosome2-2803342 DEL	PRECISE	78	0	0	0/0	0/0	0/0	0/0	0/0	0/0	0/0	0/0	0/0	0/0	0/0	0/0	0/1	0/0
Chromosome2-2807670 DEL	PRECISE	77	0	0	0/0	0/0	0/1	0/0	0/1	0/0	0/0	0/0	0/0	0/0	0/0	0/0	0/0	0/0
Chromosome2-2814097 INS	PRECISE	56	0	0	0/0	0/0	0/0	0/0	0/0	0/0	0/0	0/0	0/0	0/0	0/0	0/0	0/1	0/0
Chromosome2-2821592 DEL	PRECISE	348	0	0	0/0	0/0	0/1	0/0	0/1	0/0	0/0	0/0	0/0	0/0	0/0	0/0	0/1	0/0
Chromosome2-2829313 DEL	PRECISE	102	0	0	0/0	0/0	0/0	0/0	0/0	0/0	0/0	0/0	0/0	0/0	0/1	0/1	0/1	0/0
Chromosome2-2836342 DEL	PRECISE	135	0	0	0/0	0/0	0/0	0/0	0/0	0/0	0/0	0/0	0/0	0/0	0/0	0/0	0/1	0/0
Chromosome2-2842581 DEL	PRECISE	154	0	0	0/0	0/0	0/1	0/0	0/1	0/0	0/0	0/0	0/0	0/0	0/0	0/0	0/0	0/0
Chromosome2-2846366 INS	PRECISE	3693	0	0	0/0	0/0	0/0	0/0	0/0	0/0	0/0	0/0	0/0	0/0	0/0	0/1	0/0	0/0
Chromosome2-2848146 DEL	PRECISE	53	0	0	0/0	0/0	0/0	0/0	0/0	0/0	0/0	0/0	0/0	0/0	0/0	0/1	0/0	0/0
Chromosome2-2848672 INS	PRECISE	6692	0	0	0/0	0/0	0/0	0/0	0/0	0/0	0/0	0/0	0/0	0/0	0/0	0/1	0/0	0/0
Chromosome2-2857559 DEL	PRECISE	234	0	0	0/0	0/0	0/1	0/1	1/1	0/1	0/1	0/0	0/0	0/0	0/1	0/1	0/1	0/1
Chromosome2-2862220 DEL	PRECISE	80	0	0	0/0	0/0	0/1	0/1	0/1	0/0	0/0	0/0	0/0	0/0	0/0	0/0	0/0	0/0
Chromosome2-2863788 INS	PRECISE	522	0	0	0/0	0/0	0/0	0/0	0/0	0/0	0/0	0/0	0/0	0/0	0/0	0/1	0/0	0/0
Chromosome2-2863930 INS	PRECISE	2735	0	0	0/0	0/0	0/0	0/0	0/0	0/0	0/0	0/0	0/0	0/0	0/0	0/0	1/1	0/0
Chromosome2-2864800 DEL	PRECISE	233	0	0	0/0	0/0	0/0	0/0	0/1	0/1	0/0	0/0	0/0	0/0	0/0	0/0	0/0	0/0
Chromosome2-2865566 INS	PRECISE	1081	0	0	0/0	0/0	0/0	0/0	0/0	0/0	0/0	0/0	0/0	0/0	0/1	0/0	0/0	0/0
Chromosome2-2866116 DEL	PRECISE	70	0	0	0/0	0/0	0/0	0/1	0/0	0/0	0/0	0/0	0/0	0/0	0/0	0/0	0/0	0/0
Chromosome2-2873222 DEL	PRECISE	662	0	0	0/0	0/0	0/0	0/0	0/0	0/0	0/0	0/0	0/0	0/0	0/0	0/1	0/0	0/0
Chromosome2-2877296 DEL	PRECISE	75	0	0	0/0	0/0	0/0	0/0	0/0	0/0	0/0	0/0	0/0	0/0	0/1	0/0	0/0	0/0
Chromosome2-2878580 DEL	PRECISE	349	0	0	0/0	0/0	0/1	0/1	0/1	0/0	0/1	0/0	0/0	0/0	0/1	0/1	0/1	0/1
Chromosome2-2879536 BND	PRECISE	0	0	0	0/0	0/0	0/0	0/0	0/0	0/0	0/0	0/0	0/0	0/0	0/0	0/0	0/0	1/1
Chromosome2-2879897 BND	PRECISE	0	0	0	0/0	0/0	0/0	0/0	0/0	0/0	0/1	0/0	0/0	0/0	0/0	0/0	0/0	0/0
Chromosome2-2881704 INS	PRECISE	145	0	0	0/0	0/0	0/0	0/0	0/0	0/0	0/0	0/0	0/0	0/0	0/1	0/0	0/0	0/0
Chromosome2-2882656 INV	PRECISE	27494206	0	0	0/0	0/0	0/0	0/0	0/0	0/0	0/0	0/0	0/0	0/0	0/0	0/0	0/0	0/1
Chromosome2-2886532 DEL	PRECISE	137	0	0	0/0	0/0	0/0	0/0	0/0	0/0	0/1	0/0	0/0	0/0	0/0	0/0	0/1	0/0
Chromosome2-2887176 DEL	PRECISE	946	0	0	0/0	0/0	0/0	0/0	0/0	0/0	0/1	0/0	0/0	0/0	0/0	0/0	0/0	0/0
Chromosome2-2887801 INS	PRECISE	204	0	0	0/0	0/0	0/0	0/0	0/0	0/0	0/0	0/0	0/0	0/0	0/0	0/1	0/0	0/0
Chromosome2-2893132 DEL	PRECISE	5459	0	0	0/0	0/0	0/0	0/0	0/0	0/0	0/0	0/0	0/0	0/0	1/1	0/0	1/1	0/0
Chromosome2-2896633 INS	PRECISE	1478	0	0	0/0	0/0	0/0	0/0	0/0	0/0	0/0	0/0	0/0	0/0	0/0	0/1	0/0	0/0
Chromosome2-2903626 INS	PRECISE	575	0	0	0/0	0/0	0/0	0/0	0/0	0/0	0/0	0/0	0/0	0/0	0/0	1/1	0/0	0/0
Chromosome2-2904970 DEL	PRECISE	107	0	0	0/0	0/0	0/0	0/0	0/0	0/0	0/0	0/0	0/0	0/0	0/0	0/0	0/1	0/0
Chromosome2-2906641 INS	PRECISE	844	0	0	0/0	0/0	0/0	0/0	0/0	0/0	0/0	0/0	0/0	0/0	0/1	0/0	0/0	0/0
Chromosome2-2912961 DEL	PRECISE	57	0	0	0/0	0/0	0/0	0/1	0/0	0/0	0/0	0/0	0/0	0/0	0/0	0/0	0/0	0/0
Chromosome2-2914614 INS	PRECISE	193	0	0	0/0	0/0	0/0	0/0	0/0	0/0	0/0	0/0	0/0	0/0	0/0	0/0	0/0	0/1
Chromosome2-2930308 INS	PRECISE	250	0	0	0/0	0/0	0/0	0/0	0/0	0/0	0/0	0/0	0/0	0/0	0/0	0/0	0/0	0/1
Chromosome2-2930814 DEL	PRECISE	466	0	0	0/0	0/0	0/0	0/1	0/0	0/0	0/0	0/0	0/0	0/0	0/0	0/0	0/0	0/0
Chromosome2-2954877 INS	PRECISE	55	0	0	0/0	0/0	0/0	0/0	0/0	0/0	0/0	0/0	0/0	0/0	0/0	0/0	0/1	0/0
Chromosome2-2955376 DEL	PRECISE	150	0	0	0/0	0/0	0/0	0/0	0/0	0/0	0/0	0/0	0/0	0/0	0/0	0/0	0/1	0/1
Chromosome2-2957594 INS	PRECISE	160	0	0.707	0/0	0/0	0/0	0/0	0/0	0/0	0/0	0/0	0/0	0/0	0/0	0/0	0/1	0/1
Chromosome2-2958888 INS	PRECISE	117	0	0	0/0	0/0	0/1	0/0	0/1	0/0	0/1	0/0	0/0	0/0	0/0	0/0	0/1	0/1
Chromosome2-2964737 INS	PRECISE	258	0	0	0/0	0/0	0/0	0/0	0/0	0/0	0/0	0/0	0/0	0/0	0/0	0/0	0/0	0/1
Chromosome2-2965769 DEL	PRECISE	360	0	0	0/0	0/0	0/1	0/0	1/1	0/1	0/0	0/1	0/0	0/0	0/0	0/0	0/0	0/0
Chromosome2-2966571 DEL	PRECISE	60	0	0.527	0/0	0/0	0/1	0/1	1/1	0/1	0/0	0/1	0/0	0/0	0/0	0/1	0/1	0/1
Chromosome2-2981050 INS	PRECISE	74	0	0	0/0	0/0	0/0	0/0	0/0	0/0	0/0	0/0	0/0	0/0	0/1	0/1	1/1	0/0
Chromosome2-2982734 DEL	PRECISE	60	0	0	0/0	0/0	0/0	0/0	0/0	0/0	0/0	0/0	0/0	0/0	0/0	0/1	0/0	0/0
Chromosome2-2983698 INS	PRECISE	5415	0	0	0/0	0/0	0/0	0/0	0/0	0/0	0/0	0/0	0/0	0/0	0/0	0/0	0/0	1/1
Chromosome2-2984741 DEL	PRECISE	201	0	0	0/0	0/0	0/0	0/0	0/0	0/0	0/0	0/0	0/0	0/0	0/0	0/0	0/0	1/1
Chromosome2-2987914 DEL	PRECISE	828	0	0	0/0	0/0	0/1	0/1	1/1	0/1	0/0	1/1	0/0	0/0	0/1	0/1	0/1	1/1
Chromosome2-2993828 DEL	PRECISE	50	0	0	0/0	0/0	0/0	0/0	0/0	0/0	0/0	0/0	0/0	0/0	0/0	0/0	0/1	0/0
Chromosome2-2995383 INS	PRECISE	2010	0	0	0/0	0/0	0/0	0/0	0/0	0/0	0/0	0/0	0/0	0/0	0/0	0/0	0/1	0/0
Chromosome2-3003816 DEL	PRECISE	53	0	0	0/0	0/0	0/1	0/1	0/1	0/0	0/0	0/0	0/0	0/0	0/0	0/0	0/0	0/1
Chromosome2-3005491 INS	PRECISE	427	0	0	0/0	0/0	0/0	0/0	0/0	0/0	0/0	0/0	0/0	0/0	0/1	0/0	0/0	0/0
Chromosome2-3007312 BND	PRECISE	0	0.577	0	0/0	0/0	0/0	0/0	0/0	0/0	0/0	0/0	0/0	0/0	0/0	0/1	0/0	0/0
Chromosome2-3007313 BND	PRECISE	0	0	0	0/0	0/0	0/0	0/0	0/0	0/0	0/0	0/0	0/0	0/0	0/1	0/0	0/0	0/0
Chromosome2-3008681 DEL	PRECISE	182	0	0	0/0	0/0	0/0	0/0	0/0	0/0	0/0	0/0	0/0	0/0	0/1	0/1	0/1	0/0
Chromosome2-3020779 INS	PRECISE	10769	0	0	0/0	0/0	0/0	0/0	0/0	0/0	0/0	0/0	0/0	0/0	0/1	0/0	0/0	0/0
Chromosome2-3024652 DEL	PRECISE	3820	0	0	0/0	0/0	0/0	0/0	0/0	0/0	0/0	0/0	0/0	0/0	0/1	0/1	0/1	0/0
Chromosome2-3029032 INS	PRECISE	77	0	0	0/0	0/0	0/0	0/0	0/0	0/0	0/0	0/0	0/0	0/0	0/1	0/1	0/1	0/0
Chromosome2-3035655 DEL	PRECISE	203	0	0	0/0	0/0	0/0	0/0	0/0	0/0	0/0	0/0	0/0	0/0	0/1	0/1	0/1	0/0
Chromosome2-3037100 DEL	PRECISE	143	0	0	0/0	0/0	0/0	0/0	0/0	0/0	0/0	0/0	0/0	0/0	0/0	0/0	0/1	0/0
Chromosome2-3045941 DEL	PRECISE	234	0	0	0/0	0/0	0/0	0/0	0/0	0/0	0/0	0/0	0/0	0/0	0/0	0/0	0/0	0/1

Chromosome2-3067314	INS	PRECISE	556	0	0	0/0	0/0	0/0	0/0	0/0	0/0	0/0	0/0	0/0	0/0	0/0	0/0	0/0	0/1
Chromosome2-3074344	INS	PRECISE	704	0	0	0/0	0/0	0/0	0/0	0/0	0/0	0/0	0/0	0/0	0/0	0/1	0/0	0/0	0/0
Chromosome2-3079871	DEL	PRECISE	90	0	0	0/0	0/0	0/0	0/0	0/0	0/0	0/0	0/0	0/0	0/0	0/0	0/0	0/1	0/0
Chromosome2-3082480	INS	PRECISE	56	0	0	0/0	0/0	0/0	0/0	0/0	0/0	0/0	0/0	0/0	0/0	0/0	0/0	0/1	0/0
Chromosome2-3092025	INS	PRECISE	5375	0	0	0/0	0/0	0/0	0/0	0/0	0/0	1/1	0/0	0/0	0/0	0/0	0/0	0/0	0/0
Chromosome2-3093170	INS	PRECISE	130	0	0	0/0	0/0	0/0	0/0	0/1	0/1	0/0	0/1	0/0	0/0	0/0	0/0	0/0	0/0
Chromosome2-3105233	DEL	PRECISE	234	0	0	0/0	0/0	0/0	0/0	0/0	0/0	0/0	0/0	0/0	0/0	0/1	0/1	0/1	0/0
Chromosome2-3106182	INS	PRECISE	616	0	0	0/0	0/0	0/0	0/1	0/0	0/0	0/0	0/0	0/0	0/0	0/0	0/0	0/0	0/0
Chromosome2-3107454	INS	PRECISE	655	0	0	0/0	0/0	0/0	0/0	0/0	0/0	0/1	0/0	0/0	0/0	0/0	0/0	0/0	0/0
Chromosome2-3114530	DEL	PRECISE	1002	0	0	0/0	0/0	0/0	0/0	0/0	0/0	0/0	0/0	0/0	0/0	0/1	0/1	0/1	0/0
Chromosome2-3116207	INS	PRECISE	174	0	0	0/0	0/0	0/0	0/0	0/0	0/0	0/0	0/0	0/0	0/0	0/1	0/0	0/0	0/0
Chromosome2-3127437	INS	PRECISE	517	0	0	0/0	0/0	0/0	0/0	0/0	0/0	0/0	0/0	0/0	0/0	0/0	0/0	0/1	0/0
Chromosome2-3127452	DEL	PRECISE	211	0	0	0/0	0/0	0/1	0/1	0/1	0/0	0/0	0/0	0/0	0/0	0/0	0/0	0/0	0/0
Chromosome2-3131041	INS	PRECISE	748	0	0	0/0	0/0	0/0	0/0	0/0	0/0	0/0	0/0	0/0	0/0	0/0	0/0	0/1	0/0
Chromosome2-3136812	INS	PRECISE	5221	0	0	0/0	0/0	0/0	0/0	0/0	0/0	0/0	0/0	0/0	0/0	0/1	0/0	0/0	0/0
Chromosome2-3145804	INS	PRECISE	80	0	0	0/0	0/0	0/0	0/0	0/0	0/0	0/0	0/0	0/0	0/0	0/0	0/1	0/0	0/0
Chromosome2-3150689	DEL	PRECISE	90	0	0	0/0	0/0	0/0	0/0	0/0	0/0	0/0	0/0	0/0	0/0	0/0	0/0	0/1	0/0
Chromosome2-3157255	DEL	PRECISE	666	0	0	0/0	0/0	0/0	0/0	0/0	0/0	0/0	0/0	0/0	0/0	0/1	0/1	0/1	0/0
Chromosome2-3160029	DEL	PRECISE	426	0	0	0/0	0/0	0/1	0/0	0/1	0/0	0/0	0/0	0/0	0/0	0/1	0/1	0/1	0/1
Chromosome2-3161655	INS	PRECISE	433	0	0.707	0/0	0/0	0/1	0/0	0/1	0/0	0/0	0/0	0/0	0/0	0/0	0/0	0/0	0/0
Chromosome2-3168996	DEL	PRECISE	87	0	0	0/0	0/0	0/1	0/0	0/1	0/0	0/0	0/0	0/0	0/0	0/0	0/0	0/0	0/0
Chromosome2-3171655	DEL	PRECISE	57	0.5	0.5	0/0	0/0	0/0	0/0	0/1	0/1	0/1	0/1	0/0	0/0	0/0	0/0	0/0	0/0
Chromosome2-3174606	INS	PRECISE	1515	0	0	0/0	0/0	0/0	0/0	0/0	0/0	0/0	0/0	0/0	0/0	0/0	0/1	0/0	0/0
Chromosome2-3195151	DEL	PRECISE	2033	0	0	0/0	0/0	0/0	0/0	1/1	0/1	0/0	1/1	0/0	0/0	0/0	0/0	0/0	0/0
Chromosome2-3205028	INS	PRECISE	437	0	0.378	0/0	0/0	0/1	1/1	0/1	0/0	0/1	0/0	0/0	0/0	0/0	0/1	0/1	0/1
Chromosome2-3212386	DEL	PRECISE	150	0	0	0/0	0/0	0/0	0/1	0/0	0/0	0/0	0/0	0/0	0/0	0/0	0/0	0/0	0/0
Chromosome2-3218274	BND	PRECISE	0	0	0	0/0	0/0	0/0	0/0	0/1	0/0	0/0	0/0	0/0	0/0	0/0	0/0	0/0	0/0
Chromosome2-3234285	INS	PRECISE	211	0	0	0/0	0/0	0/0	0/0	0/0	0/0	0/0	0/0	0/0	0/0	0/0	0/0	0/0	0/1
Chromosome2-3238734	INS	PRECISE	2989	0	0	0/0	0/0	0/0	0/0	0/0	0/0	0/0	0/0	0/0	0/0	0/0	0/0	0/0	1/1
Chromosome2-3239682	DEL	PRECISE	246	0	0	0/0	0/0	0/0	0/1	0/0	0/0	0/0	0/0	0/0	0/0	0/0	0/0	0/0	0/0
Chromosome2-3242764	DUP	PRECISE	6344	0	0	0/0	0/0	0/0	0/1	0/0	0/0	0/1	0/0	0/0	0/0	0/0	0/1	0/0	0/0
Chromosome2-3250711	INS	PRECISE	1204	0	0	0/0	0/0	0/0	0/0	0/0	0/0	0/1	0/0	0/0	0/0	0/0	0/0	0/0	0/0
Chromosome2-3259181	INS	PRECISE	13094	0	0	0/0	0/0	0/0	0/0	0/0	0/0	0/0	0/0	0/0	0/0	0/1	0/0	0/0	0/0
Chromosome2-3259184	INS	PRECISE	11412	0	0	0/0	0/0	0/0	0/0	0/0	0/0	0/0	0/0	0/0	0/0	0/0	0/0	0/1	0/0
Chromosome2-3259189	BND	PRECISE	0	0	0	0/0	0/0	0/0	0/0	0/0	0/0	0/0	0/0	0/0	0/0	1/1	0/0	0/1	0/0
Chromosome2-3259189	BND	PRECISE	0	0	0	0/0	0/0	0/0	0/0	0/0	0/0	0/0	0/0	0/0	0/0	0/0	0/1	0/1	0/0
Chromosome2-3260254	INS	PRECISE	436	0	0.447	0/0	0/0	0/0	1/1	0/1	0/0	0/1	0/1	0/0	0/0	0/0	0/0	0/0	0/0
Chromosome2-3260837	DEL	PRECISE	8421	0	0	0/0	0/0	0/0	1/1	0/0	0/0	0/1	0/0	0/0	0/0	0/0	0/0	0/0	0/0
Chromosome2-3268673	INV	PRECISE	31192458	0	0	0/0	0/0	0/0	0/1	0/0	0/0	0/0	0/0	0/0	0/0	0/0	0/0	0/0	0/0
Chromosome2-3276579	INS	PRECISE	1299	0	0	0/0	0/0	0/0	0/0	0/0	0/0	0/0	0/0	0/0	0/0	0/0	0/0	0/1	0/0
Chromosome2-3278339	DEL	PRECISE	67	0	0	0/0	0/0	0/0	0/0	0/0	0/0	0/0	0/0	0/0	0/0	0/1	0/1	0/1	0/0
Chromosome2-3282725	DEL	PRECISE	423	0	0	0/0	0/0	0/0	0/0	0/0	0/0	0/0	0/0	0/0	0/0	0/1	0/1	0/1	0/1
Chromosome2-3288932	INS	PRECISE	1747	0	0	0/0	0/0	0/0	0/0	0/0	0/0	0/0	0/0	0/0	0/0	0/1	0/0	0/0	0/0
Chromosome2-3292631	DEL	PRECISE	660	0	0	0/0	0/0	0/0	0/0	0/0	0/0	0/0	0/0	0/0	0/0	0/0	0/1	0/0	0/0
Chromosome2-3297208	INS	PRECISE	455	0	0	0/0	0/0	0/0	0/0	0/0	0/0	0/0	0/0	0/0	0/0	0/0	0/0	0/0	0/1
Chromosome2-3300999	INS	PRECISE	100	0	0	0/0	0/0	0/1	0/0	0/1	0/0	0/0	0/0	0/0	0/0	0/0	0/0	0/0	0/0
Chromosome2-3305862	INS	PRECISE	7037	0	0	0/0	0/0	0/0	0/0	0/0	0/0	0/0	0/0	0/0	0/0	0/0	0/0	0/0	1/1
Chromosome2-3305869	BND	PRECISE	0	0	0	0/0	0/0	0/0	0/0	0/0	0/0	0/0	0/0	0/0	0/0	0/0	0/0	0/0	0/1
Chromosome2-3306075	BND	PRECISE	0	0	0	0/0	0/0	0/0	0/0	0/0	0/0	0/0	0/0	0/0	0/0	0/1	0/0	0/0	0/0
Chromosome2-3306214	INS	PRECISE	6368	0	0	0/0	0/0	0/0	0/0	0/0	0/0	0/0	0/0	0/0	0/0	0/0	0/0	0/0	0/0
Chromosome2-3306354	INS	PRECISE	78	0	0	0/0	0/0	0/0	0/0	0/0	0/0	0/0	0/0	0/0	0/0	1/1	0/0	0/0	0/0
Chromosome2-3308434	INS	PRECISE	1360	0	0	0/0	0/0	0/0	0/0	0/0	0/0	0/0	0/0	0/0	0/0	0/1	0/0	0/0	0/0
Chromosome2-3310306	INS	PRECISE	81	0	0	0/0	0/0	0/0	0/0	0/0	0/0	0/0	0/0	0/0	0/0	0/0	0/1	0/0	0/0
Chromosome2-3311527	INS	PRECISE	5938	0	0	0/0	0/0	0/0	0/0	0/0	0/0	0/0	0/0	0/0	0/0	0/0	1/1	0/0	0/0
Chromosome2-3311532	BND	PRECISE	0	0	0	0/0	0/0	0/0	0/0	0/0	0/0	0/0	0/0	0/0	0/0	0/0	0/1	0/0	0/0
Chromosome2-3311616	INS	PRECISE	453	0	0	0/0	0/0	0/1	0/0	0/1	0/0	0/0	0/0	0/0	0/0	0/0	0/0	0/0	0/0
Chromosome2-3314943	DEL	PRECISE	65	0	0	0/0	0/0	0/0	0/0	0/0	0/0	0/0	0/0	0/0	0/0	0/1	0/1	0/1	0/0
Chromosome2-3321797	INS	PRECISE	326	0	0	0/0	0/0	0/0	0/0	0/0	0/0	0/0	0/0	0/0	0/0	0/1	0/0	0/0	0/0
Chromosome2-3326661	INS	PRECISE	1302	0	0	0/0	0/0	0/0	0/0	0/0	0/0	0/0	0/0	0/0	0/0	0/0	0/1	0/0	0/0
Chromosome2-3339478	DEL	PRECISE	1867	0	0	0/0	0/0	0/1	0/0	0/1	0/0	0/0	0/0	0/0	0/0	0/1	0/0	0/1	0/1
Chromosome2-3343733	INS	PRECISE	972	0	0	0/0	0/0	0/0	0/0	0/0	0/0	0/0	0/0	0/0	0/0	0/0	0/1	0/0	0/0
Chromosome2-3351344	DEL	PRECISE	202	0	0	0/0	0/0	0/0	0/1	0/1	0/1	0/1	0/1	0/0	0/0	0/1	0/1	0/1	0/0
Chromosome2-3353521	DEL	PRECISE	50	0	0	0/0	0/0	0/0	0/0	0/0	0/0	0/0	0/0	0/0	0/0	0/1	0/1	0/1	0/0
Chromosome2-3354665	INS	PRECISE	1120	0	0	0/0	0/0	0/0	0/0	0/1	0/1	0/0	0/1	0/0	0/0	0/0	0/0	0/0	0/0
Chromosome2-3359002	DEL	PRECISE	3413	0	0	0/0	0/0	0/0	0/1	0/1	0/1	0/0	0/1	0/0	0/0	0/0	0/0	0/0	0/0
Chromosome2-3361258	DEL	PRECISE	1388	0	0	0/0	0/0	0/1	0/0	0/1	0/0	0/0	0/0	0/0	0/0	0/1	0/1	0/1	0/0
Chromosome2-3362525	DEL	PRECISE	914	0	0	0/0	0/0	0/0	0/1	0/1	0/1	0/0	0/1	0/0	0/0	0/0	0/0	0/0	0/0
Chromosome2-3365690	INS	PRECISE	75	0	0.577	0/0	0/0	0/0	0/0	0/0	0/0	0/0	0/0	0/0	0/0	0/1	0/1	0/1	0/0
Chromosome2-3377706	INS	PRECISE	9940	0	0	0/0	0/0	0/0	0/0	0/0	0/0	0/0	0/0	0/0	0/0	0/0	0/0	1/1	0/0
Chromosome2-3381792	DEL	PRECISE	79	0	0	0/0	0/0	0/0	0/0	0/1	1/1	0/1	0/1	0/0	0/0	0/0	0/0	0/0	0/0
Chromosome2-3413803	INS	PRECISE	180	0	0	0/0	0/0	0/0	0/0	0/1	0/1	0/1	0/1	0/0	0/0	0/0	0/0	0/0	0/0
Chromosome2-3413837	DEL	PRECISE	82	0	0	0/0	0/0	0/0	0/0	0/0	0/0	0/0	0/0	0/0	0/0	0/0	0/0	0/1	0/0
Chromosome2-3421250	INS	PRECISE	786	0	0	0/0	0/0	0/0	0/0	0/0	0/0	0/0	0/0	0/0	0/0	0/0	0/1	0/0	0/0

Chromosome2-3421676	INS	PRECISE	50	0	0	0/0	0/0	0/0	0/0	0/0	0/0	0/0	0/0	0/0	0/0	0/0	0/0	0/1	0/0
Chromosome2-3423168	DEL	PRECISE	5270	0	0	0/0	0/0	0/0	0/0	0/0	0/0	0/0	0/0	0/0	0/0	0/0	0/0	0/1	0/0
Chromosome2-3430571	INS	PRECISE	5742	0	0	0/0	0/0	0/0	0/0	0/0	0/0	0/0	0/0	0/0	0/0	1/1	0/0	0/0	0/0
Chromosome2-3437570	INS	PRECISE	444	0	0.707	0/0	0/0	0/1	0/0	0/1	0/0	0/0	0/0	0/0	0/0	0/0	0/0	0/0	0/0
Chromosome2-3437638	DEL	PRECISE	667	0	0	0/0	0/0	0/0	0/0	0/0	0/0	0/0	0/0	0/0	0/0	0/1	0/0	0/0	0/0
Chromosome2-3438280	INS	PRECISE	50	0	0	0/0	0/0	0/0	0/0	0/0	0/0	0/0	0/0	0/0	0/0	0/0	0/0	0/0	0/1
Chromosome2-3453594	INS	PRECISE	112	0	0	0/0	0/0	0/0	0/0	0/0	0/0	0/0	0/0	0/0	0/0	0/0	0/0	0/1	0/0
Chromosome2-3461145	DEL	PRECISE	126	0	0	0/0	0/0	0/0	0/1	0/0	0/0	0/0	0/0	0/0	0/0	0/0	0/0	0/0	0/0
Chromosome2-3462064	INS	PRECISE	10809	0	0	0/0	0/0	0/0	0/0	0/0	0/0	0/0	0/0	0/0	0/0	0/0	0/0	0/0	0/0
Chromosome2-3465474	DEL	PRECISE	485	0	0	0/0	0/0	0/0	0/0	0/0	0/0	0/0	0/0	0/0	0/0	1/1	0/1	0/0	0/0
Chromosome2-3465773	INS	PRECISE	9870	0	0	0/0	0/0	0/0	0/1	0/0	0/0	0/0	0/0	0/0	0/0	0/0	0/0	0/0	0/0
Chromosome2-3466531	DEL	PRECISE	319	0	0	0/0	0/0	0/0	0/0	0/0	0/0	0/0	0/0	0/0	0/0	0/1	0/1	0/0	0/0
Chromosome2-3474603	BND	PRECISE	0	0	0	0/0	0/0	0/0	0/0	0/0	0/0	0/0	0/0	0/0	0/0	0/1	0/0	0/0	0/0
Chromosome2-3477452	INS	PRECISE	9922	0	0	0/0	0/0	0/0	0/0	0/0	0/0	0/0	0/0	0/0	0/0	0/1	0/0	0/0	0/0
Chromosome2-3484981	INS	PRECISE	3154	0	0	0/0	0/0	0/0	0/0	0/0	0/0	0/0	0/0	0/0	0/0	0/0	0/0	0/1	0/0
Chromosome2-3486407	INS	PRECISE	321	0	0	0/0	0/0	0/0	0/0	0/0	0/0	0/0	0/0	0/0	0/0	0/0	0/1	0/0	0/0
Chromosome2-3489181	DEL	PRECISE	4365	0	0	0/0	0/0	0/0	0/0	0/0	0/0	0/0	0/0	0/0	0/0	0/0	0/1	0/0	0/0
Chromosome2-3492208	DEL	PRECISE	1338	0	0	0/0	0/0	0/0	0/0	0/0	0/0	0/0	0/0	0/0	0/0	0/0	0/1	0/0	0/1
Chromosome2-3493721	INS	PRECISE	165	0	0	0/0	0/0	0/1	0/0	1/1	0/0	0/0	0/0	0/0	0/0	0/0	0/0	0/0	0/0
Chromosome2-3495416	DEL	PRECISE	353	0	0	0/0	0/0	0/0	0/0	0/0	0/0	0/0	0/0	0/0	0/0	0/0	0/1	0/0	0/0
Chromosome2-3496044	DEL	PRECISE	1357	0	0	0/0	0/0	0/0	0/0	0/0	0/0	0/0	0/0	0/0	0/0	0/0	0/1	0/0	0/0
Chromosome2-3501411	INS	PRECISE	700	0	0	0/0	0/0	0/0	0/0	0/0	0/0	0/0	0/0	0/0	0/0	0/0	0/0	0/0	0/1
Chromosome2-3507360	DEL	PRECISE	1293	0	0	0/0	0/0	0/0	0/0	0/0	0/0	0/0	0/0	0/0	0/0	0/0	1/1	0/0	0/0
Chromosome2-3508743	INS	PRECISE	1263	0	0	0/0	0/0	0/0	0/0	0/0	0/0	0/0	0/0	0/0	0/0	0/0	0/0	0/0	0/1
Chromosome2-3510661	INS	PRECISE	3523	0	0	0/0	0/0	0/0	0/0	0/0	0/0	0/0	0/0	0/0	0/0	0/0	0/0	0/0	0/1
Chromosome2-3512357	INS	PRECISE	519	0	0	0/0	0/0	0/0	0/0	0/0	0/0	0/0	0/0	0/0	0/0	0/0	0/0	0/0	0/1
Chromosome2-3513421	DEL	PRECISE	3050	0	0	0/0	0/0	0/0	0/0	0/0	0/0	0/0	0/0	0/0	0/0	0/0	0/0	0/0	0/1
Chromosome2-3513562	INS	PRECISE	127	0	0	0/0	0/0	0/0	0/0	0/0	0/0	0/0	0/0	0/0	0/0	0/1	0/1	0/1	0/0
Chromosome2-3516361	INS	PRECISE	3085	0	0	0/0	0/0	0/0	0/0	0/0	0/0	0/0	0/0	0/0	0/0	0/0	1/1	0/0	0/0
Chromosome2-3518443	INS	PRECISE	5329	0	0	0/0	0/0	0/0	0/0	0/0	0/0	0/0	0/0	0/0	0/0	0/1	0/0	0/0	0/0
Chromosome2-3523969	DEL	PRECISE	90	0	0	0/0	0/0	0/0	0/0	0/0	0/0	0/0	0/0	0/0	0/0	0/0	0/1	0/0	0/0
Chromosome2-3524450	INS	PRECISE	73	0	0	0/0	0/0	0/0	0/0	0/0	0/0	0/0	0/0	0/0	0/0	0/1	0/0	0/0	0/0
Chromosome2-3529126	INS	PRECISE	248	0	0	0/0	0/0	0/1	0/0	1/1	0/0	0/0	0/0	0/0	0/0	0/0	0/0	0/0	0/0
Chromosome2-3535067	INS	PRECISE	118	0	0.577	0/0	0/0	0/0	0/1	0/0	0/0	1/1	0/0	0/0	0/0	0/0	0/0	0/0	0/0
Chromosome2-3542372	DEL	PRECISE	1898	0	0	0/0	0/0	0/0	0/1	0/0	0/0	0/0	0/0	0/0	0/0	0/0	0/0	0/0	0/0
Chromosome2-3545265	INS	PRECISE	5016	0	0	0/0	0/0	0/0	0/0	0/0	0/0	0/0	0/0	0/0	0/0	0/0	0/0	0/0	0/1
Chromosome2-3546108	DEL	PRECISE	9602	0	0	0/0	0/0	0/0	0/0	0/0	0/0	0/0	0/0	0/0	0/0	0/1	0/0	0/0	0/0
Chromosome2-3547235	INS	PRECISE	1201	0	0.816	0/0	0/0	0/0	0/0	0/1	0/1	0/1	1/1	0/0	0/0	0/0	0/0	0/0	0/0
Chromosome2-3549299	DEL	PRECISE	6019	0	0	0/0	0/0	0/1	1/1	1/1	0/1	0/1	1/1	0/0	0/0	0/0	0/1	0/1	0/0
Chromosome2-3555792	DEL	PRECISE	77	0	0	0/0	0/0	0/1	0/0	0/1	0/0	0/0	0/0	0/0	0/0	0/0	0/0	0/0	0/0
Chromosome2-3559321	DEL	PRECISE	53	0	0	0/0	0/0	0/0	0/0	0/0	0/0	0/1	0/0	0/0	0/0	0/0	0/0	0/0	0/0
Chromosome2-3576055	DEL	PRECISE	51	0	0	0/0	0/0	0/0	0/0	0/0	0/0	0/0	0/0	0/0	0/0	0/0	0/0	0/0	0/1
Chromosome2-3582427	DEL	PRECISE	52	0	0	0/0	0/0	0/0	0/0	0/0	0/0	0/0	0/0	0/0	0/0	0/0	0/0	0/0	0/1
Chromosome2-3583401	INS	PRECISE	71	0	0	0/0	0/0	0/0	0/0	0/0	0/0	0/0	0/0	0/0	0/0	0/0	0/0	0/0	0/1
Chromosome2-3591719	DEL	PRECISE	1012	0	0	0/0	0/0	0/1	0/1	1/1	0/1	1/1	1/1	0/0	0/0	0/1	0/1	0/0	0/0
Chromosome2-3593017	DEL	PRECISE	744	0	0	0/0	0/0	0/1	0/1	1/1	0/1	1/1	1/1	0/0	0/0	0/0	0/0	0/0	0/0
Chromosome2-3594828	INS	PRECISE	1337	0	0	0/0	0/0	0/0	0/0	0/0	0/0	0/0	0/0	0/0	0/0	0/0	0/0	0/0	0/1
Chromosome2-3615913	INS	PRECISE	401	0	0	0/0	0/0	0/0	0/0	0/0	0/0	0/0	0/0	0/0	0/0	0/0	0/0	0/0	0/1
Chromosome2-3616866	INS	PRECISE	150	0	0.5	0/0	0/0	0/0	0/0	0/0	0/0	0/0	0/0	0/0	0/1	0/1	1/1	0/1	0/0
Chromosome2-3620357	DEL	PRECISE	70	0	0	0/0	0/0	0/0	0/0	0/0	0/0	0/0	0/0	0/0	0/1	0/1	1/1	0/1	0/0
Chromosome2-3629684	DEL	PRECISE	174	0	0	0/0	0/0	0/1	0/0	0/1	0/0	0/0	0/0	0/0	0/0	0/1	0/0	0/0	0/1
Chromosome2-3630110	INS	PRECISE	11294	0	0	0/0	0/0	0/0	0/0	0/0	0/0	0/0	0/0	0/0	0/0	0/1	0/0	0/0	0/0
Chromosome2-3630111	BND	PRECISE	0	0	0	0/0	0/0	0/0	0/0	0/0	0/0	0/0	0/0	0/0	0/0	0/0	0/0	0/1	0/0
Chromosome2-3632527	INS	PRECISE	362	0	0	0/0	0/0	0/1	0/0	0/1	0/0	0/0	0/0	0/0	0/0	0/0	0/0	0/0	0/0
Chromosome2-3632806	DEL	PRECISE	74	0.882	0	0/0	0/0	0/1	0/0	0/1	0/1	0/1	0/1	0/0	0/0	0/0	0/1	0/0	0/0
Chromosome2-3635445	INS	PRECISE	11240	0	0	0/0	0/0	0/0	0/0	0/0	0/0	0/0	0/0	0/0	0/0	0/0	0/1	0/0	0/0
Chromosome2-3645921	DEL	PRECISE	303	0	0	0/0	0/0	0/1	0/0	0/1	0/0	0/0	0/0	0/0	0/1	0/1	1/1	0/1	0/0
Chromosome2-3647816	INS	PRECISE	356	0	0.707	0/0	0/0	0/0	0/0	0/0	0/0	0/0	0/0	0/0	0/1	0/0	1/1	0/0	0/0
Chromosome2-3674463	INS	PRECISE	117	0	0	0/0	0/0	0/0	0/0	0/0	0/0	0/0	0/0	0/0	0/0	0/0	0/0	0/0	0/1
Chromosome2-3674597	DEL	PRECISE	780	0	0	0/0	0/0	0/1	0/0	0/1	0/0	0/0	0/0	0/0	0/0	0/0	0/0	0/0	0/0
Chromosome2-3689417	INS	PRECISE	84	0	0	0/0	0/0	0/1	0/0	0/1	0/0	0/1	0/0	0/0	0/1	0/1	1/1	0/1	0/1
Chromosome2-3702232	DEL	PRECISE	185	0	0	0/0	0/0	0/0	0/0	0/0	0/0	0/0	0/0	0/0	0/0	0/0	0/0	0/1	0/0
Chromosome2-3704151	DEL	PRECISE	2053	0	0	0/0	0/0	0/0	0/0	0/0	0/0	0/0	0/0	0/0	0/1	0/0	0/0	0/0	0/0
Chromosome2-3714970	INS	PRECISE	624	0	0	0/0	0/0	0/0	0/1	0/0	0/0	0/0	0/0	0/0	0/0	0/0	0/0	0/0	0/0
Chromosome2-3720032	DEL	PRECISE	263	0	0	0/0	0/0	0/1	0/0	0/1	0/0	0/0	0/0	0/0	0/0	0/0	0/0	0/0	0/0
Chromosome2-3722125	DEL	PRECISE	832	0	0	0/0	0/0	0/1	0/0	0/1	0/0	0/0	0/0	0/0	0/0	0/0	0/0	0/0	0/0
Chromosome2-3723200	INS	PRECISE	253	0	0.707	0/0	0/0	0/0	0/0	0/0	0/0	0/0	0/0	0/0	0/1	0/0	1/1	0/0	0/0
Chromosome2-3728447	BND	PRECISE	0	0	0	0/0	0/0	0/0	0/0	0/0	0/0	0/0	0/0	0/0	0/0	0/0	0/1	0/0	0/0
Chromosome2-3729946	INS	PRECISE	1440	0	0	0/0	0/0	0/0	0/0	0/0	0/0	0/0	0/0	0/0	0/0	0/0	0/1	0/0	0/0
Chromosome2-3732125	INS	PRECISE	330	0	0.548	0/0	0/0	0/0	0/1	0/1	0/1	0/1	0/1	0/0	0/0	0/0	0/0	0/0	0/0
Chromosome2-3732840	DEL	PRECISE	157	0	0	0/0	0/0	0/0	0/0	0/0	0/0	0/0	0/0	0/0	0/0	0/0	0/1	0/0	0/0
Chromosome2-3735869	DEL	PRECISE	1197	0	0	0/0	0/0	0/0	1/1	1/1	0/1	1/1	1/1	0/0	0/0	0/0	0/0	0/0	0/0
Chromosome2-3738461	DEL	PRECISE	70	0	0	0/0	0/0	0/0	0/0	0/0	0/0	0/1	0/0	0/0	0/0	0/0	0/0	0/0	0/0

Chromosome2-3755513	DEL	PRECISE	1182	0	0	0/0	0/0	0/0	1/1	1/1	0/1	1/1	1/1	0/0	0/0	0/0	0/0	0/0	0/0
Chromosome2-3769364	DEL	PRECISE	963	0	0	0/0	0/0	0/1	1/1	0/1	0/0	0/1	0/0	0/0	0/1	0/1	1/1	0/1	0/1
Chromosome2-3774507	DEL	PRECISE	190	0	0	0/0	0/0	0/1	1/1	0/1	0/0	0/1	0/0	0/0	0/0	0/0	0/0	0/0	0/1
Chromosome2-3775646	DEL	PRECISE	63	0	0	0/0	0/0	0/0	0/0	0/0	0/0	0/0	0/0	0/0	0/1	0/1	1/1	0/1	0/0
Chromosome2-3784934	DEL	PRECISE	394	0	0	0/0	0/0	0/0	0/0	0/0	0/0	0/0	0/0	0/0	0/0	0/1	0/0	0/1	0/0
Chromosome2-3787826	DEL	PRECISE	1549	0	0	0/0	0/0	0/0	0/0	0/0	0/0	0/0	0/0	0/0	0/1	0/1	1/1	0/1	0/0
Chromosome2-3793130	INS	PRECISE	55	0	0.577	0/0	0/0	0/0	0/0	0/0	0/0	0/0	0/0	0/0	0/0	0/1	0/1	0/1	0/0
Chromosome2-3794244	INS	PRECISE	1739	0	0.707	0/0	0/0	0/0	0/0	0/0	0/0	0/0	0/0	0/0	0/1	0/0	0/1	0/0	0/0
Chromosome2-3794451	DEL	PRECISE	62	0	0	0/0	0/0	0/0	0/0	0/0	0/0	0/0	0/0	0/0	0/0	0/1	0/0	0/1	0/0
Chromosome2-3799209	DEL	PRECISE	5478	0	0	0/0	0/0	0/0	0/0	0/0	0/0	0/0	0/0	0/0	0/0	1/1	0/0	0/1	0/0
Chromosome2-3811704	DEL	PRECISE	263	0	0	0/0	0/0	0/0	0/0	0/0	0/0	0/0	0/0	0/0	0/1	0/0	0/1	0/0	0/0
Chromosome2-3817178	DEL	PRECISE	1200	0	0	0/0	0/0	0/0	0/0	0/0	0/0	0/0	0/0	0/0	0/1	0/1	1/1	0/1	0/1
Chromosome2-3830033	DEL	PRECISE	474	0	0	0/0	0/0	0/0	0/0	0/0	0/0	0/0	0/0	0/0	1/1	0/0	0/0	0/0	0/0
Chromosome2-3831299	INS	PRECISE	336	0	0	0/0	0/0	0/0	0/0	0/0	0/0	0/0	0/0	0/0	1/1	0/0	0/0	0/0	0/0
Chromosome2-3832287	DEL	PRECISE	690	0	0	0/0	0/0	0/0	0/0	0/0	0/0	0/0	0/0	0/0	0/1	0/0	1/1	0/0	0/0
Chromosome2-3847127	DEL	PRECISE	664	0	0	0/0	0/0	0/0	0/0	0/0	0/0	0/0	0/0	0/0	0/0	0/0	0/0	0/0	0/1
Chromosome2-3847633	DEL	PRECISE	139	0	0	0/0	0/0	0/0	0/0	0/0	0/0	0/1	0/0	0/0	0/0	0/0	0/0	0/0	0/0
Chromosome2-3849939	DEL	PRECISE	79	0	0	0/0	0/0	0/0	0/0	0/0	0/0	0/0	0/0	0/0	0/1	0/0	0/1	0/0	0/0
Chromosome2-3853619	DEL	PRECISE	290	0	0	0/0	0/0	0/0	0/0	0/0	0/0	0/0	0/0	0/0	0/1	0/0	1/1	0/0	0/0
Chromosome2-3854991	INS	PRECISE	148	0	0	0/0	0/0	0/0	0/0	0/0	0/0	0/0	0/0	0/0	0/1	0/1	1/1	0/0	0/0
Chromosome2-3858699	DEL	PRECISE	117	0	0	0/0	0/0	0/0	0/0	0/0	0/0	0/0	0/0	0/0	0/0	0/1	0/1	0/1	0/0
Chromosome2-3861826	INS	PRECISE	55	0	0.707	0/0	0/0	0/0	0/0	0/0	0/0	0/0	0/0	0/0	0/1	0/0	0/1	0/0	0/0
Chromosome2-3878940	INS	PRECISE	144	0	0	0/0	0/0	0/0	0/0	0/0	0/0	0/1	0/0	0/0	0/0	0/0	0/0	0/0	0/0
Chromosome2-3885776	INS	PRECISE	52	0	0	0/0	0/0	0/0	0/0	0/0	0/0	0/0	0/0	0/0	0/1	0/1	1/1	0/1	0/0
Chromosome2-3887155	INS	PRECISE	150	0	0	0/0	0/0	0/0	0/0	0/0	0/0	0/0	0/0	0/0	0/0	0/0	0/0	0/0	0/1
Chromosome2-3887216	DEL	PRECISE	93	0	0	0/0	0/0	0/0	0/0	0/0	0/0	0/0	0/0	0/0	0/1	0/1	1/1	0/1	0/0
Chromosome2-3900664	DEL	PRECISE	72	0	0	0/0	0/0	0/0	0/1	0/1	0/1	1/1	1/1	0/0	0/0	0/0	0/0	0/0	0/1
Chromosome2-3903551	INS	PRECISE	10343	0	0	0/0	0/0	0/0	0/0	0/0	0/0	1/1	0/0	0/0	0/0	0/0	0/0	0/0	0/0
Chromosome2-3903551	BND	PRECISE	0	0	0	0/0	0/0	0/0	0/0	0/1	0/0	0/1	0/1	0/0	0/0	0/0	0/0	0/0	0/1
Chromosome2-3911712	DEL	PRECISE	731	0	0	0/0	0/0	0/0	0/1	0/1	0/1	0/1	1/1	0/1	0/0	0/1	0/0	0/1	0/0
Chromosome2-3912399	INS	PRECISE	10260	0	0	0/0	0/0	0/0	0/0	0/0	0/0	0/0	0/0	0/0	0/0	0/0	0/0	0/0	0/1
Chromosome2-3913901	DEL	PRECISE	494	0	0	0/0	0/0	0/0	0/0	0/0	0/0	0/0	0/0	0/0	0/0	0/1	0/0	0/1	0/0
Chromosome2-3913973	INS	PRECISE	866	0	0.707	0/0	0/0	0/0	0/0	0/0	0/0	0/0	0/0	0/0	0/1	0/0	1/1	0/0	0/0
Chromosome2-3940287	DEL	PRECISE	122	0	0	0/0	0/0	0/1	0/0	1/1	0/0	0/0	0/0	0/0	0/0	0/0	0/0	0/0	0/0
Chromosome2-3944383	DEL	PRECISE	60	0	0	0/0	0/0	0/0	0/0	0/0	0/0	0/0	0/0	0/0	0/0	0/1	0/0	0/1	0/0
Chromosome2-3951155	DEL	PRECISE	74	0	0	0/0	0/0	0/0	0/0	0/0	0/0	0/0	0/0	0/0	0/0	0/1	0/0	0/1	0/0
Chromosome2-3954572	INV	PRECISE	23678296	0	0.5	0/0	0/0	0/0	0/0	0/0	0/1	0/1	0/0	0/0	0/0	0/0	0/0	0/0	0/0
Chromosome2-3979794	INS	PRECISE	705	0	0	0/0	0/0	0/0	0/0	0/0	0/0	0/0	0/0	0/0	0/0	0/0	1/1	0/0	0/0
Chromosome2-3980438	DEL	PRECISE	649	0	0	0/0	0/0	0/0	0/0	0/0	0/0	0/0	0/0	0/0	0/0	0/0	0/1	0/0	0/0
Chromosome2-3987641	INS	PRECISE	54	0	0	0/0	0/0	0/0	0/0	0/0	0/0	0/0	0/0	0/0	0/0	0/0	0/0	0/0	0/1
Chromosome2-3987747	DEL	PRECISE	61	0	0	0/0	0/0	0/0	0/0	0/0	0/0	0/0	0/0	0/0	0/0	0/0	0/1	0/0	0/0
Chromosome2-3990486	DEL	PRECISE	1002	0	0	0/0	0/0	0/0	0/1	0/0	0/0	0/0	0/0	0/0	0/0	0/0	0/0	0/0	0/0
Chromosome2-3993395	INS	PRECISE	12457	0	0	0/0	0/0	0/0	0/0	0/0	0/0	0/0	0/0	0/0	0/0	0/0	0/1	0/0	0/0
Chromosome2-3993401	BND	PRECISE	0	0	0	0/0	0/0	0/0	0/0	0/0	0/0	0/0	0/0	0/0	0/0	0/0	0/1	0/0	0/0
Chromosome2-3995749	DUP	PRECISE	34670	0	0	0/0	0/0	0/1	0/0	0/1	0/0	0/0	0/0	0/0	0/0	0/0	0/0	0/0	0/0
Chromosome2-3997641	BND	PRECISE	0	0	0	0/0	0/0	0/0	0/0	0/1	0/0	0/0	0/0	0/0	0/0	0/0	0/0	0/0	0/0
Chromosome2-3997986	INS	PRECISE	3580	0	0.707	0/0	0/0	0/0	0/0	0/0	0/0	0/0	0/0	0/0	1/1	0/0	1/1	0/0	0/0
Chromosome2-4003693	DEL	PRECISE	54	0	0	0/0	0/0	0/1	0/0	0/1	0/0	0/0	0/0	0/0	0/0	0/0	0/0	0/0	0/0
Chromosome2-4011317	INS	PRECISE	52	0	0	0/0	0/0	0/0	0/1	0/0	0/0	0/0	0/0	0/0	0/0	0/0	0/0	0/0	0/0
Chromosome2-4018785	DEL	PRECISE	57	0	0	0/0	0/0	0/0	0/0	0/0	0/0	0/0	0/0	0/0	0/0	0/0	0/1	0/0	0/0
Chromosome2-4018911	BND	PRECISE	0	0	0	0/0	0/0	0/1	0/0	0/1	0/0	0/0	0/0	0/0	0/0	0/0	0/0	0/0	0/0
Chromosome2-4026242	INS	PRECISE	59	0	0.577	0/0	0/0	0/0	0/0	0/0	0/0	0/0	0/0	0/0	0/1	0/0	0/1	0/0	0/0
Chromosome2-4027546	INS	PRECISE	7485	0	0	0/0	0/0	0/0	0/0	0/0	0/0	0/0	0/0	0/0	0/0	0/0	0/0	0/0	0/1
Chromosome2-4027553	BND	PRECISE	0	0	0	0/0	0/0	0/0	0/0	0/0	0/0	0/0	0/0	0/0	0/0	0/0	0/0	0/0	0/1
Chromosome2-4031635	INS	PRECISE	124	0	0.548	0/0	0/0	0/0	0/0	0/1	0/1	1/1	1/1	0/1	0/0	0/0	0/0	0/0	0/0
Chromosome2-4032497	DEL	PRECISE	482	0	0	0/0	0/0	0/1	0/0	0/0	0/0	0/0	0/0	0/0	0/0	0/0	0/0	0/0	0/1
Chromosome2-4050998	INS	PRECISE	68	0	0	0/0	0/0	0/1	0/0	0/1	0/0	0/0	0/0	0/0	0/0	0/0	0/0	0/0	0/0
Chromosome2-4052095	DEL	PRECISE	13499	0	0	0/0	0/0	0/0	0/0	0/0	0/0	0/0	0/0	0/0	0/0	0/1	0/1	0/1	0/0
Chromosome2-4057149	INS	PRECISE	1366	0	0.447	0/0	0/0	0/0	0/0	0/1	0/1	1/1	1/1	0/1	0/0	0/0	0/0	0/0	0/0
Chromosome2-4060546	DEL	PRECISE	1285	0	0	0/0	0/0	0/0	0/0	0/0	0/0	0/0	0/0	0/0	0/0	0/0	1/1	0/0	0/0
Chromosome2-4062534	INS	PRECISE	78	0	0	0/0	0/0	0/0	0/0	0/0	0/0	0/0	0/0	0/0	0/0	0/0	1/1	0/0	0/0
Chromosome2-4065411	DEL	PRECISE	2031	0	0	0/0	0/0	0/0	0/0	0/0	0/0	0/0	0/0	0/0	0/0	0/0	0/1	0/0	0/0
Chromosome2-4067492	DEL	PRECISE	899	0	0	0/0	0/0	0/0	0/0	0/0	0/0	0/0	0/0	0/0	0/0	0/0	0/1	0/0	0/0
Chromosome2-4069199	INS	PRECISE	710	0	0	0/0	0/0	0/0	0/0	0/0	0/0	0/0	0/0	0/0	0/1	0/1	1/1	0/1	0/0
Chromosome2-4071211	INS	PRECISE	365	0	0	0/0	0/0	0/0	0/0	0/0	0/0	0/0	0/0	0/0	0/0	0/0	0/1	0/0	0/0
Chromosome2-4074566	INS	PRECISE	54	0	0	0/0	0/0	0/0	0/0	0/0	0/0	0/0	0/0	0/0	0/1	0/0	0/1	0/0	0/0
Chromosome2-4077470	INS	PRECISE	5549	0	0	0/0	0/0	0/0	0/0	0/0	0/0	0/0	0/0	0/0	0/0	0/0	1/1	0/0	0/0
Chromosome2-4091767	DEL	PRECISE	291	0	0	0/0	0/0	0/0	0/1	0/1	0/1	1/1	1/1	0/1	0/1	0/1	1/1	0/1	0/0
Chromosome2-4110245	INS	PRECISE	56	0	0.707	0/0	0/0	0/1	0/0	0/1	0/0	0/0	0/0	0/0	0/0	0/0	0/0	0/0	0/0
Chromosome2-4110320	DEL	PRECISE	55	0	0	0/0	0/0	0/0	0/0	0/0	0/0	0/0	0/0	0/0	0/1	0/0	0/1	0/0	0/0
Chromosome2-4110721	DEL	PRECISE	971	0	0	0/0	0/0	0/1	0/0	0/1	0/0	0/0	0/0	0/0	0/1	0/1	1/1	0/1	0/0
Chromosome2-4111750	INS	PRECISE	159	0	0	0/0	0/0	0/0	0/0	0/0	0/0	0/0	0/0	0/0	0/0	0/0	0/1	0/0	0/0
Chromosome2-4116262	DEL	PRECISE	58	0	0	0/0	0/0	0/0	0/0	0/0	0/0	0/0	0/0	0/0	0/0	0/0	0/1	0/0	0/0

Chromosome2-4126600	INS	PRECISE	71	0	0.5	0/0	0/0	0/0	0/0	0/0	0/0	0/0	0/0	0/0	0/1	0/1	1/1	0/1	0/0
Chromosome2-4130584	INS	PRECISE	71	0	0	0/0	0/0	0/0	0/0	0/0	0/0	0/0	0/0	0/0	0/0	0/0	0/0	0/0	0/1
Chromosome2-4135972	DUP	PRECISE	58	0	0	0/0	0/0	0/0	0/0	0/0	0/0	0/0	0/0	0/0	0/0	0/0	0/1	0/0	0/0
Chromosome2-4136238	INS	PRECISE	104	0	0	0/0	0/0	0/0	0/0	0/0	0/0	0/0	0/0	0/0	0/1	0/1	1/1	0/1	0/0
Chromosome2-4139861	DEL	PRECISE	51	0	0	0/0	0/0	0/0	0/0	0/0	0/0	0/0	0/0	0/0	0/1	0/1	1/1	0/1	0/0
Chromosome2-4145835	DEL	PRECISE	124	0	0	0/0	0/0	0/0	0/0	0/0	0/0	0/0	0/0	0/0	0/1	0/1	1/1	0/1	0/0
Chromosome2-4149229	INS	PRECISE	132	0	0	0/0	0/0	0/1	0/0	0/1	0/0	0/0	0/0	0/0	0/0	0/0	0/0	0/0	0/0
Chromosome2-4151681	INS	PRECISE	137	0	0.548	0/0	0/0	0/0	0/0	0/1	0/1	1/1	1/1	0/1	0/0	0/0	0/0	0/0	0/0
Chromosome2-4153312	DEL	PRECISE	234	0	0	0/0	0/0	0/0	0/0	0/0	0/0	0/1	0/0	0/0	0/0	0/0	0/0	0/0	0/0
Chromosome2-4155216	INS	PRECISE	150	0	0	0/0	0/0	0/1	0/0	0/1	0/0	0/0	0/0	0/0	0/0	0/0	0/0	0/0	0/0
Chromosome2-4162426	DEL	PRECISE	252	0	0	0/0	0/0	0/0	0/0	0/0	0/0	0/0	0/0	0/0	0/1	0/1	1/1	0/1	0/0
Chromosome2-4170811	DEL	PRECISE	53	0	0	0/0	0/0	0/1	0/0	0/1	0/0	0/0	0/0	0/0	0/0	0/0	0/0	0/0	0/0
Chromosome2-4171684	DEL	PRECISE	975	0	0	0/0	0/0	0/0	0/0	0/0	0/0	0/0	0/0	0/0	0/0	0/0	0/0	0/0	0/1
Chromosome2-4172108	INS	PRECISE	65	0	0.5	0/0	0/0	0/0	0/0	0/0	0/0	0/0	0/0	0/0	0/1	0/1	1/1	0/1	0/0
Chromosome2-4179354	DEL	PRECISE	72	0	0	0/0	0/0	0/0	0/0	0/0	0/0	0/0	0/0	0/0	0/0	0/0	0/0	0/0	0/1
Chromosome2-4180220	DEL	PRECISE	1195	0	0	0/0	0/0	0/0	0/0	0/0	0/0	0/0	0/0	0/0	0/1	0/1	1/1	0/1	0/0
Chromosome2-4182007	INS	PRECISE	211	0	0	0/0	0/0	0/0	0/1	0/0	0/0	0/0	0/0	0/0	0/0	0/0	0/0	0/0	0/0
Chromosome2-4184797	INS	PRECISE	657	0	0	0/0	0/0	0/0	0/1	0/0	0/0	0/0	0/0	0/0	0/0	0/0	0/0	0/0	0/0
Chromosome2-4194844	DEL	PRECISE	51	0	0	0/0	0/0	0/0	0/0	0/0	0/0	0/0	0/0	0/0	0/1	0/1	1/1	0/1	0/0
Chromosome2-4196966	INS	PRECISE	125	0	0	0/0	0/0	0/0	0/0	0/0	0/0	0/0	0/0	0/0	0/0	0/0	0/0	0/0	0/1
Chromosome2-4198816	INS	PRECISE	873	0	0	0/0	0/0	0/0	0/0	0/0	0/0	0/0	0/0	0/0	0/0	0/0	0/0	0/0	0/1
Chromosome2-4213105	DEL	PRECISE	63	0	0	0/0	0/0	0/0	0/0	0/0	0/0	0/0	0/0	0/0	0/1	0/1	1/1	0/1	0/0
Chromosome2-4216891	DEL	PRECISE	936	0	0	0/0	0/0	0/0	0/0	0/0	0/0	0/0	0/0	0/0	0/1	0/0	1/1	0/1	0/0
Chromosome2-4223365	DEL	PRECISE	71	0	0	0/0	0/0	0/0	0/0	0/0	0/0	0/0	0/0	0/0	0/0	0/0	0/0	0/0	0/1
Chromosome2-4227009	DEL	PRECISE	89	0	0	0/0	0/0	0/0	0/0	0/0	0/0	0/0	0/0	0/0	0/1	0/1	1/1	0/1	0/0
Chromosome2-4229632	DEL	PRECISE	60	0	0	0/0	0/0	0/0	0/0	0/1	0/1	0/1	1/1	0/1	0/0	0/0	0/0	0/0	0/0
Chromosome2-4234757	INS	PRECISE	140	0	0	0/0	0/0	0/0	0/0	0/0	0/0	0/0	0/0	0/0	0/0	0/0	0/1	0/0	0/0
Chromosome2-4242944	DEL	PRECISE	61	0	0	0/0	0/0	0/0	0/0	0/0	0/0	0/0	0/0	0/0	0/1	0/0	1/1	0/0	0/0
Chromosome2-4244839	INS	PRECISE	239	0	0.707	0/0	0/0	0/1	0/0	0/1	0/0	0/0	0/0	0/0	0/0	0/0	0/0	0/0	0/0
Chromosome2-4245888	DEL	PRECISE	80	0	0	0/0	0/0	0/0	0/0	0/0	0/0	0/0	0/0	0/0	0/0	0/1	0/0	0/1	0/0
Chromosome2-4256853	DEL	PRECISE	86	0	0	0/0	0/0	0/0	0/0	0/0	0/0	0/0	0/0	0/0	0/0	0/0	0/0	0/0	0/1
Chromosome2-4257879	INS	PRECISE	211	0	0	0/0	0/0	0/0	0/0	0/0	0/0	0/0	0/0	0/0	0/0	0/0	0/0	0/0	0/1
Chromosome2-4260917	DEL	PRECISE	56	0	0	0/0	0/0	0/1	0/0	0/1	0/0	0/0	0/0	0/0	0/1	0/1	1/1	0/1	0/1
Chromosome2-4265618	INS	PRECISE	211	0	0	0/0	0/0	0/0	0/0	0/0	0/0	0/0	0/0	0/0	0/0	0/1	0/0	0/1	0/0
Chromosome2-4271223	DEL	PRECISE	152	0	0	0/0	0/0	0/1	0/1	0/1	0/0	0/1	0/0	0/0	0/0	0/0	0/0	0/0	0/0
Chromosome2-4281229	DEL	PRECISE	135	0	0	0/0	0/0	0/1	0/0	0/1	0/0	0/0	0/0	0/0	0/0	0/0	0/0	0/0	0/0
Chromosome2-4283182	INS	PRECISE	54	0	0	0/0	0/0	0/0	0/0	0/0	0/0	0/0	0/0	0/0	0/1	0/1	1/1	0/1	0/0
Chromosome2-4286445	DEL	PRECISE	50	0	0	0/0	0/0	0/1	0/0	0/1	0/0	0/0	0/0	0/0	0/0	0/0	0/0	0/0	0/0
Chromosome2-4288795	INS	PRECISE	95	0	0	0/0	0/0	0/0	0/0	0/0	0/0	0/0	0/0	0/0	0/1	0/0	1/1	0/0	0/0
Chromosome2-4291543	DEL	PRECISE	52	0	0	0/0	0/0	0/0	0/0	0/0	0/0	0/0	0/0	0/0	0/1	0/0	1/1	0/0	0/0
Chromosome2-4292421	DEL	PRECISE	722	0	0	0/0	0/0	0/0	0/1	0/0	0/0	0/1	0/0	0/0	0/0	0/0	0/0	0/0	0/0
Chromosome2-4296508	DEL	PRECISE	106	0	0	0/0	0/0	0/0	0/1	0/0	0/0	0/1	0/0	0/0	0/0	0/0	0/0	0/0	0/0
Chromosome2-4298984	DEL	PRECISE	125	0	0	0/0	0/0	0/0	0/0	0/1	0/1	0/1	1/1	0/1	0/0	0/0	0/0	0/0	0/0
Chromosome2-4304508	INS	PRECISE	2578	0	0	0/0	0/0	0/0	0/0	0/0	0/0	0/0	0/0	0/0	0/0	0/0	0/0	0/0	0/1
Chromosome2-4306478	INS	PRECISE	149	0	0	0/0	0/0	0/0	0/0	0/0	0/0	0/0	0/0	0/0	0/0	0/0	0/0	0/0	0/1
Chromosome2-4308008	INS	PRECISE	445	0	0	0/1	0/0	0/0	0/0	0/0	0/0	0/0	0/0	0/0	0/0	0/0	0/0	0/0	0/0
Chromosome2-4317154	INS	PRECISE	57	0	0	0/0	0/0	0/0	0/0	0/0	0/0	0/0	0/0	0/0	0/0	0/0	0/0	0/0	0/1
Chromosome2-4324477	INS	PRECISE	426	0	0.707	0/0	0/0	0/0	0/0	0/0	0/0	0/0	0/0	0/0	0/1	0/0	1/1	0/0	0/0
Chromosome2-4330358	DEL	PRECISE	150	0	0	0/0	0/0	0/0	0/0	0/0	0/0	0/0	0/0	0/0	0/1	0/0	0/1	0/0	0/1
Chromosome2-4331845	DEL	PRECISE	75	0	0	0/0	0/0	0/0	0/0	0/0	0/0	0/0	0/0	0/0	0/1	0/0	0/1	0/0	0/0
Chromosome2-4333309	DEL	PRECISE	168	0	0	0/0	0/0	0/0	0/0	0/0	0/0	0/0	0/0	0/0	0/0	0/0	0/0	0/0	0/1
Chromosome2-4343056	INS	PRECISE	775	0	0	0/0	0/0	0/0	0/0	0/0	0/0	0/0	0/0	0/0	0/0	0/0	1/1	0/0	0/0
Chromosome2-4356819	INS	PRECISE	2696	0	0	0/0	0/0	0/0	0/0	0/0	0/0	0/0	0/0	0/0	0/0	0/0	0/0	0/0	1/1
Chromosome2-4363118	DEL	PRECISE	877	0	0	0/0	0/0	0/0	0/0	0/1	0/1	1/1	1/1	0/1	0/1	0/1	1/1	0/1	0/1
Chromosome2-4364109	INS	PRECISE	2403	0	0	0/0	0/0	0/0	0/1	0/0	0/0	0/0	0/0	0/0	0/0	0/0	0/0	0/0	0/0
Chromosome2-4368860	DEL	PRECISE	54	0	0	0/0	0/0	0/0	0/0	0/0	0/0	0/0	0/0	0/0	0/1	0/0	1/1	0/0	0/0
Chromosome2-4390299	DEL	PRECISE	3948	0	0	0/0	0/0	0/0	0/0	0/0	0/0	0/0	0/0	0/0	0/1	0/0	0/1	0/0	0/0
Chromosome2-4401881	DEL	PRECISE	590	0	0	0/0	0/0	0/0	0/0	0/0	0/0	0/0	0/0	0/0	0/0	0/1	0/1	0/1	0/0
Chromosome2-4405419	DEL	PRECISE	422	0.577	0.577	0/0	0/0	0/0	0/0	0/0	0/0	0/0	0/0	0/0	0/0	0/1	0/1	0/0	0/0
Chromosome2-4406201	DEL	PRECISE	50	0	0	0/0	0/0	0/1	0/1	1/1	0/1	1/1	1/1	0/1	0/1	0/1	1/1	0/1	0/1
Chromosome2-4411235	DEL	PRECISE	702	0	0	0/0	0/0	0/0	0/0	0/0	0/0	0/0	0/0	0/0	0/0	0/1	0/1	0/0	0/0
Chromosome2-4415511	BND	PRECISE	0	0	0	0/0	0/0	0/0	0/0	0/0	1/1	0/0	0/1	0/1	0/0	0/0	0/0	0/0	0/0
Chromosome2-4423113	DEL	PRECISE	2453	0	0	0/0	0/0	0/1	0/0	0/1	0/0	0/0	0/0	0/0	0/0	0/0	0/0	0/0	0/0
Chromosome2-4427005	DEL	PRECISE	68	0	0	0/0	0/0	0/0	0/1	0/0	0/0	0/1	0/0	0/0	0/0	0/0	0/0	0/0	0/0
Chromosome2-4427120	INS	PRECISE	243	0	0.707	0/0	0/0	0/1	0/0	0/1	0/0	0/0	0/0	0/0	0/0	0/0	0/0	0/0	0/0
Chromosome2-4427591	INS	PRECISE	7263	0	0	0/0	0/0	0/1	0/0	0/0	0/0	0/0	0/0	0/0	0/0	0/0	0/0	0/0	0/0
Chromosome2-4434857	DEL	PRECISE	70	0	0	0/0	0/0	0/1	0/0	0/1	0/0	0/0	0/0	0/0	0/0	0/0	0/0	0/0	0/0
Chromosome2-4437466	INS	PRECISE	65	0	0	0/0	0/0	0/0	0/0	0/0	0/0	0/0	0/0	0/0	0/0	0/0	1/1	0/0	0/0
Chromosome2-4438672	DEL	PRECISE	1224	0	0	0/0	0/0	0/0	0/0	0/0	0/0	0/0	0/0	0/0	0/1	0/0	0/0	0/1	0/0
Chromosome2-4440343	INS	PRECISE	56	0	0	0/0	0/0	0/0	0/1	0/0	0/0	0/0	0/0	0/0	0/0	0/0	0/0	0/0	0/0
Chromosome2-4441995	DEL	PRECISE	353	0.378	0.378	0/0	0/0	0/1	1/1	1/1	0/1	1/1	1/1	0/1	0/0	0/0	0/0	0/0	0/0
Chromosome2-4443497	DEL	PRECISE	413	0.707	0.707	0/0	0/0	0/0	0/0	0/0	0/0	0/0	0/0	0/0	0/0	0/0	1/1	0/0	0/0
Chromosome2-4446158	DEL	PRECISE	411	0	0	0/0	0/0	0/0	0/0	0/0	0/0	0/0	0/0	0/0	0/0	0/0	1/1	0/0	0/0

Chromosome2-4448811 BND	PRECISE	0	0	0	0/0	0/0	0/0	0/0	0/0	0/0	0/0	0/0	0/0	0/1	0/0	0/0	0/0	0/0
Chromosome2-4451997 INV	PRECISE	38876167	0	0	0/0	0/0	0/0	0/0	0/0	0/0	0/0	0/0	0/0	0/0	0/0	0/1	0/0	0/0
Chromosome2-4461703 DEL	PRECISE	624	0	0	0/0	0/0	0/1	0/0	0/1	0/0	0/0	0/0	0/0	0/0	0/0	0/0	0/0	0/0
Chromosome2-4463152 INS	PRECISE	4741	0	0	0/0	0/0	0/0	0/1	0/0	0/0	0/0	0/0	0/0	0/0	0/0	0/0	0/0	0/0
Chromosome2-4467926 INS	PRECISE	116	0	0	0/0	0/0	0/0	0/0	0/0	0/0	0/0	0/0	0/0	0/0	0/0	0/0	0/0	0/1
Chromosome2-4483563 INS	PRECISE	1364	0	0.707	0/0	0/0	0/0	0/0	0/0	0/0	0/0	0/0	0/0	0/1	0/0	0/1	0/0	0/0
Chromosome2-4483563 INS	PRECISE	12392	0	0	0/0	0/0	0/0	0/0	0/0	0/0	0/0	0/0	0/0	0/0	0/0	0/0	0/1	0/0
Chromosome2-4492890 INS	PRECISE	944	0	0	0/0	0/0	1/1	0/0	0/1	0/0	0/0	0/0	0/0	0/0	0/0	0/0	0/0	0/0
Chromosome2-4505470 DEL	PRECISE	113	0	0	0/0	0/0	0/0	0/0	0/0	0/0	0/0	0/0	0/0	0/0	0/1	0/0	0/1	0/0
Chromosome2-4509639 INS	PRECISE	66	0	0.707	0/0	0/0	0/0	0/0	0/0	0/0	0/0	0/0	0/0	0/0	0/1	0/0	0/1	0/0
Chromosome2-4511837 DEL	PRECISE	59	0	0	0/0	0/0	0/0	0/1	0/0	0/0	0/0	0/0	0/0	0/0	0/0	0/0	0/0	0/0
Chromosome2-4512925 DEL	PRECISE	92	0.302	0.302	0/0	0/0	0/0	0/1	0/1	0/1	1/1	1/1	0/1	0/1	0/1	1/1	0/1	0/1
Chromosome2-4515227 INS	PRECISE	347	0	0.707	0/0	0/0	0/0	0/0	0/0	0/0	0/0	0/0	0/0	0/0	0/1	0/0	0/1	0/0
Chromosome2-4525506 INS	PRECISE	87	0	0	0/0	0/0	0/0	0/0	0/0	0/0	0/0	0/0	0/0	0/0	0/0	0/0	0/0	0/1
Chromosome2-4525632 DEL	PRECISE	312	0	0	0/0	0/0	0/0	0/0	0/0	0/0	0/0	0/0	0/0	0/0	0/0	0/0	0/0	0/1
Chromosome2-4526721 INS	PRECISE	75	0	0	0/0	0/0	0/0	0/0	0/0	0/0	0/1	0/0	0/0	0/0	0/0	0/0	0/0	0/0
Chromosome2-4530391 INS	PRECISE	55	0	0	0/0	0/0	0/0	0/0	0/0	0/0	0/0	0/0	0/0	0/1	0/1	1/1	0/1	0/1
Chromosome2-4536318 DEL	PRECISE	117	0	0	0/0	0/0	0/0	0/0	0/0	0/0	0/1	0/0	0/0	0/0	0/0	0/0	0/0	0/0
Chromosome2-4543923 DEL	PRECISE	54	0	0	0/0	0/0	0/0	0/0	0/0	0/0	0/0	0/0	0/0	0/1	0/0	0/1	0/0	0/0
Chromosome2-4550785 INS	PRECISE	162	0	0	0/0	0/0	0/0	0/0	0/0	0/0	0/0	0/0	0/0	0/1	0/1	1/1	0/1	0/0
Chromosome2-4552123 DEL	PRECISE	745	0	0	0/0	0/0	0/1	1/1	1/1	0/1	1/1	1/1	0/1	0/0	0/1	1/1	0/0	0/1
Chromosome2-4578762 DEL	PRECISE	81	0	0	0/0	0/0	0/0	0/0	0/0	0/0	0/0	0/0	0/0	0/1	0/1	0/1	0/1	0/0
Chromosome2-4579261 INS	PRECISE	328	0	0	0/0	0/0	0/0	0/0	0/0	0/0	0/0	0/0	0/0	0/0	0/0	0/0	0/0	0/1
Chromosome2-4579487 INS	PRECISE	1926	0	0	0/0	0/0	0/0	0/0	0/0	0/0	0/0	0/0	0/0	0/0	0/0	0/1	0/0	0/0
Chromosome2-4582137 BND	PRECISE	0	0	0	0/0	0/0	0/0	0/0	0/0	0/0	0/0	0/0	0/0	1/1	0/0	0/0	0/0	0/0
Chromosome2-4582645 INS	PRECISE	457	0	0	0/0	0/0	0/0	0/0	0/0	0/0	0/0	0/0	0/0	0/0	0/0	0/0	0/0	0/1
Chromosome2-4586733 DEL	PRECISE	123	0	0	0/0	0/0	0/0	0/0	0/0	0/0	0/1	0/0	0/0	0/0	0/0	0/0	0/0	0/0
Chromosome2-4589574 DEL	PRECISE	413	0	0	0/0	0/0	0/0	0/0	0/0	0/0	0/0	0/0	0/0	0/0	0/1	0/0	0/0	0/0
Chromosome2-4594829 INS	PRECISE	75	0	0	0/0	0/0	0/0	0/0	0/0	0/0	0/0	0/0	0/0	0/1	0/0	1/1	0/0	0/0
Chromosome2-4602938 DEL	PRECISE	90	0	0	0/0	0/0	0/0	1/1	0/0	0/0	0/0	0/0	0/0	0/0	0/0	0/0	0/0	0/0
Chromosome2-4611979 INS	PRECISE	426	0	0	0/0	0/0	0/0	1/1	0/0	0/0	0/0	0/0	0/0	0/0	0/0	0/0	0/0	0/0
Chromosome2-4616347 INS	PRECISE	119	0	0.753	0/1	0/1	0/1	0/0	0/0	0/1	0/0	0/0	0/1	0/1	0/0	0/0	0/0	0/0
Chromosome2-4625184 INS	PRECISE	400	0	0.488	0/0	0/0	0/1	1/1	1/1	0/1	0/1	1/1	0/1	0/0	0/0	0/0	0/0	0/0
Chromosome2-4628327 DEL	PRECISE	886	0	0	0/0	0/0	0/0	0/0	0/0	0/0	0/0	0/0	0/0	0/1	0/1	1/1	0/1	0/0
Chromosome2-4629873 INS	PRECISE	787	0	0	0/0	0/0	0/0	0/1	0/0	0/0	0/0	0/0	0/0	0/0	0/0	0/0	0/0	0/0
Chromosome2-4631178 DEL	PRECISE	53	0	0	0/0	0/0	0/0	0/0	0/0	0/0	0/0	0/0	0/0	0/0	0/1	0/1	0/1	0/0
Chromosome2-4638127 INS	PRECISE	686	0.577	0.957	0/0	0/0	0/0	0/0	0/0	0/0	0/0	0/0	0/0	0/1	0/1	1/1	0/1	0/0
Chromosome2-4648571 INS	PRECISE	139	0	0	0/0	0/0	0/0	0/0	0/0	0/0	0/0	0/0	0/0	0/0	0/0	0/1	0/0	0/0
Chromosome2-4649757 DEL	PRECISE	615	0	0	0/0	0/0	0/0	0/0	0/0	0/0	0/0	0/0	0/0	0/1	0/0	1/1	0/0	0/0
Chromosome2-4652551 DEL	PRECISE	271	0	0	0/0	0/0	0/0	0/0	0/0	0/0	0/0	0/0	0/0	0/1	0/1	1/1	0/1	0/0
Chromosome2-4654304 DEL	PRECISE	104	0	0	0/0	0/0	0/0	0/0	0/0	0/0	0/0	0/0	0/0	0/1	0/1	1/1	0/1	0/0
Chromosome2-4654518 BND	PRECISE	0	0	0	0/0	0/0	0/1	0/1	0/0	0/0	0/0	0/0	0/0	0/0	0/0	0/0	0/0	0/0
Chromosome2-4660536 INS	PRECISE	410	0	0.5	0/0	0/0	0/0	0/0	0/0	0/0	0/0	0/0	0/0	0/1	0/1	1/1	0/1	0/0
Chromosome2-4666393 DEL	PRECISE	62	0	0	0/0	0/0	0/1	0/1	0/0	0/0	0/0	0/0	0/0	0/0	0/0	0/0	0/0	0/0
Chromosome2-4672992 DEL	PRECISE	1249	0	0	0/0	0/0	0/0	0/0	1/1	0/0	0/1	1/1	0/1	0/0	0/0	0/0	0/0	0/0
Chromosome2-4673274 DEL	PRECISE	211	0	0	0/0	0/0	0/0	0/0	0/0	0/0	0/0	0/0	0/0	0/1	1/1	1/1	0/1	0/0
Chromosome2-4673863 INS	PRECISE	55	0	0	0/0	0/0	0/0	0/0	0/0	0/0	0/0	0/0	0/0	0/0	0/0	0/1	0/0	0/0
Chromosome2-4684888 DEL	PRECISE	7989	0	0	0/0	0/0	0/0	0/0	0/0	0/0	0/0	0/0	0/0	0/0	0/0	1/1	0/0	0/0
Chromosome2-4685265 DEL	PRECISE	9510432	0	0	0/0	0/0	0/0	0/0	0/1	0/0	0/1	1/1	0/1	0/0	0/0	0/0	0/0	0/0
Chromosome2-4686371 INS	PRECISE	424	0	0.707	0/0	0/0	0/1	0/1	0/0	0/0	0/0	0/0	0/0	0/0	0/0	0/0	0/0	0/0
Chromosome2-4688996 DEL	PRECISE	734	0	0	0/0	0/0	0/1	0/1	0/0	0/0	1/1	0/0	0/0	0/0	0/0	0/0	0/0	0/0
Chromosome2-4699027 DUP	PRECISE	9521063	0	0	0/0	0/0	0/0	0/0	1/1	0/1	1/1	0/1	0/1	0/0	0/0	0/0	0/0	0/0
Chromosome2-4707459 INS	PRECISE	119	0	0.354	0/0	0/0	0/1	1/1	0/1	0/0	0/0	0/0	0/0	0/1	0/1	1/1	0/1	0/1
Chromosome2-4713280 INS	PRECISE	654	0	0	0/0	0/0	0/0	0/0	0/0	0/0	0/0	0/0	0/0	0/0	0/0	0/0	0/0	0/1
Chromosome2-4727882 INS	PRECISE	454	0	0	0/0	0/0	0/0	0/0	0/0	0/0	0/1	0/0	0/0	0/0	0/0	0/0	0/0	0/0
Chromosome2-4733022 INS	PRECISE	94	0	0.389	0/0	0/0	0/1	1/1	1/1	0/1	0/1	1/1	0/1	0/1	0/1	1/1	0/1	0/0
Chromosome2-4767039 INS	PRECISE	851	0	0	0/0	0/0	0/0	0/1	0/0	0/0	0/0	0/0	0/0	0/0	0/0	0/0	0/0	0/0
Chromosome2-4772320 DEL	PRECISE	77	0	0	0/0	0/0	0/0	0/0	0/0	0/0	0/1	0/0	0/0	0/0	0/0	0/0	0/0	0/1
Chromosome2-4792659 INS	PRECISE	96	0	0	0/0	0/0	0/0	0/0	0/0	0/0	0/0	0/0	0/0	0/1	0/1	1/1	0/1	0/0
Chromosome2-4794444 INS	PRECISE	60	0	0	0/0	0/0	0/0	0/0	0/0	0/0	0/0	0/0	0/0	0/0	0/0	0/1	0/0	0/0
Chromosome2-4796896 DEL	PRECISE	50	0	0	0/0	0/0	0/1	0/1	1/1	0/1	1/1	1/1	0/1	0/0	0/1	0/1	0/1	0/0
Chromosome2-4804076 INS	PRECISE	68	0	0.5	0/0	0/0	0/0	0/0	0/0	0/0	0/0	0/0	0/0	0/1	0/1	1/1	0/1	0/0
Chromosome2-4806542 INS	PRECISE	75	0	0	0/0	0/0	0/0	0/0	0/0	0/0	0/0	0/0	0/0	0/1	0/1	1/1	0/1	0/0
Chromosome2-4807476 INS	PRECISE	3497	0	0	0/0	0/0	0/0	1/1	0/0	0/0	0/0	0/0	0/0	0/0	0/0	0/0	0/0	0/0
Chromosome2-4809569 DEL	PRECISE	4226	0	0	0/0	0/0	0/0	0/0	0/0	0/0	0/0	0/0	0/0	0/0	0/0	0/0	0/0	0/1
Chromosome2-4823078 DEL	PRECISE	135	0	0	0/0	0/0	0/0	0/0	0/0	0/0	0/0	0/0	0/0	0/1	0/1	1/1	0/1	0/0
Chromosome2-4825151 INS	PRECISE	124	0	0.707	0/0	0/0	0/0	0/0	0/0	0/0	0/0	0/0	0/0	0/1	0/0	0/1	0/0	0/0
Chromosome2-4826771 DEL	PRECISE	52	0	0	0/0	0/0	0/0	0/0	0/0	0/0	0/0	0/0	0/0	0/0	0/0	0/0	0/0	0/1
Chromosome2-4827165 DEL	PRECISE	7148	0	0	0/0	0/0	0/0	0/0	0/0	0/0	0/0	0/0	0/0	1/1	0/0	1/1	0/0	0/0
Chromosome2-4831182 DEL	PRECISE	61	0	0	0/0	0/0	0/1	0/1	0/1	0/0	0/0	0/0	0/0	0/0	0/0	0/0	0/0	0/0
Chromosome2-4836224 INS	PRECISE	347	0	0	0/0	0/0	0/0	0/0	0/0	0/0	0/0	0/0	0/0	0/1	0/0	0/1	0/0	0/0
Chromosome2-4845911 INS	PRECISE	63	0	0	0/0	0/0	0/0	0/0	0/0	0/0	0/0	0/0	0/0	0/1	0/1	1/1	0/1	0/0
Chromosome2-4861511 INS	PRECISE	350	0	0	0/0	0/0	0/0	0/0	0/0	0/0	0/0	0/0	0/0	0/0	0/0	0/0	0/0	0/1

Chromosome2-4862422	INS	PRECISE	982	0	0	0/0	0/0	0/0	0/0	0/0	0/0	0/0	0/0	0/0	0/0	0/0	0/0	0/0	0/1
Chromosome2-4865618	DEL	PRECISE	56	0	0	0/0	0/0	0/1	0/1	0/1	0/0	0/0	0/0	0/0	0/0	0/0	0/0	0/0	0/0
Chromosome2-4867590	BND	PRECISE	0	0	0	0/0	0/0	0/0	0/0	0/1	0/0	0/0	0/0	0/0	0/0	0/0	0/0	0/0	0/0
Chromosome2-4868697	DEL	PRECISE	93	0	0	0/0	0/0	0/0	0/0	0/0	0/0	0/0	0/0	0/0	0/0	0/0	1/1	0/1	0/0
Chromosome2-4869757	DEL	PRECISE	2992	0.996	0.996	0/0	0/0	0/0	0/1	1/1	0/1	0/0	1/1	0/1	0/0	0/1	1/1	0/1	0/1
Chromosome2-4874434	DEL	PRECISE	14269	0	0	0/0	0/0	0/0	0/1	0/0	0/0	0/1	0/0	0/1	0/1	0/1	1/1	0/1	0/1
Chromosome2-4889057	INS	PRECISE	162	0	0	0/0	0/0	0/0	0/0	0/0	0/0	0/0	0/0	0/0	0/1	0/0	0/1	0/0	0/0
Chromosome2-4889195	BND	PRECISE	0	0	0	0/0	0/0	0/0	0/0	0/1	0/0	0/1	0/1	0/0	0/0	0/0	0/0	0/0	0/0
Chromosome2-4907524	INS	PRECISE	623	0	0.707	0/0	0/0	0/1	0/1	1/1	0/1	0/1	1/1	0/1	0/0	0/0	1/1	0/0	0/0
Chromosome2-4914011	INS	PRECISE	5214	0	0	0/0	0/0	0/0	0/0	0/0	0/0	1/1	0/0	0/0	0/0	0/0	0/0	0/0	0/0
Chromosome2-4914015	BND	PRECISE	0	0	0	0/0	0/0	0/0	0/0	0/0	0/0	0/1	0/0	0/0	0/0	0/0	0/0	0/0	0/0
Chromosome2-4916462	DEL	PRECISE	85	0	0	0/0	0/0	0/0	0/0	0/0	0/0	0/0	0/0	0/0	0/0	1/1	0/0	1/1	0/0
Chromosome2-4917034	INS	PRECISE	132	0	0	0/0	0/0	0/0	0/0	0/1	1/1	0/1	1/1	1/1	0/0	0/0	0/0	0/0	0/0
Chromosome2-4930276	DEL	PRECISE	88	0	0	0/0	0/0	0/0	0/0	0/0	0/0	0/0	0/0	0/0	0/0	0/0	0/0	0/0	0/1
Chromosome2-4932687	DEL	PRECISE	748	0	0	0/0	0/0	0/0	0/0	0/0	0/0	0/0	0/0	0/0	0/1	0/1	1/1	0/1	0/0
Chromosome2-4933890	INS	PRECISE	7439	0	0	0/0	0/0	0/0	0/0	0/0	0/0	1/1	0/0	0/0	0/0	0/0	0/0	0/0	0/0
Chromosome2-4940859	DEL	PRECISE	1141	0	0	0/0	0/0	0/0	0/0	0/0	0/0	0/0	0/0	0/0	0/1	0/1	1/1	0/0	0/0
Chromosome2-4940911	INS	PRECISE	623	0	0.837	0/0	0/0	0/0	0/0	0/0	0/1	0/0	1/1	0/1	0/0	0/0	0/0	0/0	0/0
Chromosome2-4944979	DEL	PRECISE	5229	0.548	0.548	0/0	0/0	0/1	0/1	0/1	0/0	0/0	0/0	0/0	0/0	0/1	0/1	0/0	0/0
Chromosome2-4945782	INS	PRECISE	1286	0	0	0/0	0/0	0/0	0/0	0/0	0/0	0/0	0/0	0/0	1/1	0/0	1/1	0/0	0/0
Chromosome2-4946528	DEL	PRECISE	369	0	0	0/0	0/0	0/0	0/0	0/0	0/0	0/1	0/0	0/0	0/0	0/0	0/0	0/0	0/0
Chromosome2-4947311	INS	PRECISE	205	0	0	0/0	0/0	0/0	0/0	0/0	0/0	0/0	0/0	0/0	0/0	0/0	0/0	0/0	0/1
Chromosome2-4948418	DEL	PRECISE	986	0	0	0/0	0/0	0/0	0/0	0/0	0/0	0/0	0/0	0/0	1/1	0/0	1/1	0/0	0/0
Chromosome2-4950331	INS	PRECISE	622	0	0	0/0	0/0	0/0	0/0	0/0	0/0	0/0	0/0	0/0	0/1	0/0	1/1	0/0	0/0
Chromosome2-4950342	DEL	PRECISE	377	0	0	0/0	0/0	0/1	0/1	0/1	0/0	0/0	0/0	0/0	0/0	0/1	0/0	0/0	0/0
Chromosome2-4955158	INS	PRECISE	1814	0	0	0/0	0/0	0/0	0/0	0/0	0/0	0/1	0/0	0/0	0/0	0/0	0/0	0/0	0/0
Chromosome2-4957378	DEL	PRECISE	70	0	0	0/0	0/0	0/0	0/0	0/0	0/0	0/0	0/0	0/0	0/0	0/0	0/0	0/0	0/1
Chromosome2-4958646	INS	PRECISE	67	0	0	0/0	0/0	0/0	0/0	0/1	0/1	0/1	1/1	0/1	0/0	0/0	0/0	0/0	0/0
Chromosome2-4965236	DEL	PRECISE	340	0	0	0/0	0/0	0/0	0/0	0/0	0/0	0/0	0/0	0/0	0/0	0/0	0/0	0/1	0/0
Chromosome2-4966423	INS	PRECISE	1851	0	0	0/0	0/0	0/0	0/0	0/0	0/0	0/0	0/0	0/0	0/0	0/0	0/0	0/1	0/0
Chromosome2-4971849	INS	PRECISE	64	0	0	0/0	0/0	0/0	0/0	0/0	0/0	0/0	0/0	0/0	0/1	0/1	1/1	0/1	0/0
Chromosome2-4981660	INV	PRECISE	37779249	0	0	0/0	0/0	0/0	0/0	0/0	0/0	0/0	0/0	0/0	0/0	0/0	0/0	0/0	0/1
Chromosome2-4987153	INS	PRECISE	205	0	0	0/0	0/0	0/0	0/0	0/0	0/0	0/0	0/0	0/0	0/1	0/0	0/1	0/0	0/0
Chromosome2-4988835	INS	PRECISE	229	0	0.516	0/0	0/0	0/0	0/1	0/1	0/1	1/1	1/1	0/1	0/0	0/0	0/0	0/0	0/0
Chromosome2-4989576	DEL	PRECISE	149	0	0	0/0	0/0	0/0	0/0	0/0	0/0	0/0	0/0	0/0	0/1	0/1	1/1	0/1	0/0
Chromosome2-4999986	INS	PRECISE	426	0	0.5	0/0	0/0	0/0	0/0	0/0	0/0	0/0	0/0	0/0	0/1	0/1	1/1	0/1	0/0
Chromosome2-5003210	INS	PRECISE	991	0	0	0/0	0/0	0/0	0/0	0/0	0/0	0/0	0/0	0/0	0/0	0/0	0/0	0/0	0/1
Chromosome2-5004019	DEL	PRECISE	155	0	0	0/0	0/0	0/0	0/0	0/0	0/0	0/0	0/0	0/0	0/0	0/1	0/0	0/1	0/0
Chromosome2-5013041	DEL	PRECISE	124	0	0	0/0	0/0	0/0	0/0	0/0	0/0	0/0	0/0	0/0	0/1	0/1	1/1	0/1	0/0
Chromosome2-5022505	DEL	PRECISE	679	0	0	0/0	0/0	0/0	0/1	0/1	0/0	0/1	1/1	0/1	0/0	0/0	0/0	0/0	0/0
Chromosome2-5024578	INS	PRECISE	61	0	0	0/0	0/0	0/0	0/1	0/1	0/1	0/1	1/1	0/1	0/1	0/1	1/1	0/1	0/0
Chromosome2-5026788	INS	PRECISE	1262	0	0	0/0	0/0	0/0	0/0	0/0	0/0	0/0	0/0	0/0	0/0	0/0	0/0	0/0	0/1
Chromosome2-5031490	BND	PRECISE	0	0	0	0/0	0/0	0/0	0/0	0/0	0/0	0/1	0/0	0/0	0/0	0/0	0/0	0/0	0/0
Chromosome2-5040864	INS	PRECISE	9444	0	0	0/0	0/0	0/0	0/0	0/0	0/0	1/1	0/0	0/0	0/0	0/0	0/0	0/0	0/0
Chromosome2-5045313	DUP	PRECISE	15954	0	0	0/0	0/0	0/0	0/0	0/0	0/0	0/0	0/0	0/0	0/0	0/0	0/0	0/0	0/1
Chromosome2-5048874	DEL	PRECISE	582	0	0	0/0	0/0	0/0	0/0	0/0	0/0	0/0	0/0	0/0	0/0	0/0	1/1	0/0	0/0
Chromosome2-5048875	DEL	PRECISE	5327	0	0	0/0	0/0	0/0	0/0	0/0	0/0	0/0	0/0	0/0	0/0	0/0	0/0	0/0	0/1
Chromosome2-5050673	DEL	PRECISE	1438	0	0	0/0	0/0	0/1	0/0	0/1	0/0	0/0	0/0	0/0	0/0	0/0	0/0	0/0	0/0
Chromosome2-5054222	DEL	PRECISE	1145	0	0	0/0	0/0	0/0	0/0	0/0	0/0	0/0	0/0	0/0	0/0	0/0	0/0	0/0	0/1
Chromosome2-5054246	INS	PRECISE	434	0	0	0/0	0/0	0/1	0/0	0/1	0/0	0/0	0/0	0/0	0/0	0/0	0/0	0/0	0/0
Chromosome2-5057929	INS	PRECISE	71	0	0	0/0	0/0	0/0	0/0	0/0	0/0	0/0	0/0	0/0	0/0	0/0	0/0	0/0	0/1
Chromosome2-5059726	DEL	PRECISE	121	0	0	0/0	0/0	0/0	0/0	0/0	0/0	0/0	0/0	0/0	0/0	0/0	0/1	0/0	0/0
Chromosome2-5059924	DEL	PRECISE	1788	0	0	0/0	0/0	0/0	0/0	0/0	0/0	0/0	0/0	0/0	0/1	0/1	0/1	0/1	0/0
Chromosome2-5061709	INS	PRECISE	3889	0	0	0/0	0/0	0/0	0/0	0/0	0/0	0/0	0/0	0/0	0/0	0/0	0/1	0/0	0/0
Chromosome2-5078275	INS	PRECISE	161	0	0	0/0	0/0	0/0	0/1	0/0	0/0	0/0	0/0	0/0	0/0	0/0	0/0	0/0	0/0
Chromosome2-5082801	INS	PRECISE	8960	0	0	0/0	0/0	0/0	0/0	0/0	0/0	0/0	0/0	0/0	0/0	0/0	0/0	0/0	0/1
Chromosome2-5082801	BND	PRECISE	0	0	0	0/0	0/0	0/0	0/0	0/0	0/0	0/0	0/0	0/0	0/0	0/0	0/0	0/0	0/1
Chromosome2-5092780	INS	PRECISE	1084	0	0	0/0	0/0	0/1	0/0	0/1	0/0	0/0	0/0	0/0	0/0	0/0	0/0	0/0	0/0
Chromosome2-5095324	DEL	PRECISE	121	0	0	0/0	0/0	0/0	0/0	0/0	0/0	0/0	0/0	0/0	0/0	0/1	1/1	0/0	0/0
Chromosome2-5103870	DEL	PRECISE	925	0	0	0/0	0/0	0/0	0/0	0/0	0/0	0/0	0/0	0/0	0/0	0/0	1/1	0/0	0/0
Chromosome2-5129523	INS	PRECISE	1125	0	0.707	0/0	0/0	0/0	0/0	0/0	0/0	0/1	0/0	0/0	0/0	0/0	0/0	0/0	0/1
Chromosome2-5138919	DEL	PRECISE	427	0	0	0/0	0/0	0/0	0/0	0/0	0/0	0/0	0/0	0/0	0/1	0/1	1/1	0/1	0/0
Chromosome2-5146499	DUP	PRECISE	123	0	0	0/0	0/0	0/0	0/0	0/0	0/0	0/0	0/0	0/0	0/0	0/0	0/1	0/0	0/0
Chromosome2-5155807	BND	PRECISE	0	0	0	0/0	0/0	0/0	0/0	0/0	0/0	0/0	0/0	0/0	0/1	0/0	0/0	0/0	0/0
Chromosome2-5167520	DEL	PRECISE	268	0	0	0/0	0/0	0/0	0/1	0/0	0/0	0/0	0/0	0/0	0/0	0/0	0/0	0/0	0/0
Chromosome2-5174647	INS	PRECISE	136	0	0.707	0/0	0/0	0/0	0/0	0/0	0/0	0/0	0/0	0/0	0/0	0/0	0/0	0/0	0/1
Chromosome2-5179305	DEL	PRECISE	21999189	0	0	0/0	0/0	0/0	0/0	0/0	0/0	0/0	0/0	0/1	0/0	0/0	1/1	0/0	0/0
Chromosome2-5192043	INS	PRECISE	7227	0	0	0/0	0/0	0/0	0/0	0/0	0/0	0/0	0/0	0/0	0/0	0/0	0/0	0/0	1/1
Chromosome2-5193272	INS	PRECISE	10882	0	0	0/0	0/0	0/0	1/1	0/0	0/0	0/0	0/0	0/0	0/0	0/0	0/0	0/0	0/0
Chromosome2-5195508	DEL	PRECISE	155	0.5	0.5	0/0	0/0	0/0	0/0	0/0	0/0	0/0	0/0	0/0	0/1	0/0	1/1	0/1	0/0
Chromosome2-5199612	BND	PRECISE	0	0	0	0/0	0/0	0/0	0/0	0/1	0/0	1/1	0/1	0/0	0/0	0/0	0/0	0/0	0/0
Chromosome2-5210618	INS	PRECISE	55	0	0.5	0/0	0/0	0/0	0/0	0/0	0/0	0/0	0/0	0/0	0/1	0/1	1/1	0/1	0/0
Chromosome2-5214255	DEL	PRECISE	1284	0	0	0/0	0/0	0/0	0/0	0/0	0/0	0/0	0/0	0/0	0/1	0/1	1/1	0/1	0/0

Chromosome2-5214818	BND	PRECISE	0	0	0	0/0	0/0	0/0	0/0	0/0	0/0	0/1	0/1	0/1	0/0	0/0	0/0	0/0	0/0
Chromosome2-5220105	DEL	PRECISE	211	0	0	0/0	0/0	0/0	0/0	0/0	0/0	0/0	0/0	0/0	0/1	0/1	1/1	0/1	0/0
Chromosome2-5225335	BND	PRECISE	0	0	0	0/0	0/0	0/1	0/0	0/0	0/0	0/0	0/0	0/0	0/0	0/0	0/0	0/0	0/0
Chromosome2-5228079	INS	PRECISE	83	0	0.707	0/0	0/0	0/0	0/0	0/0	0/0	0/0	0/0	0/0	0/0	0/1	0/1	0/0	0/0
Chromosome2-5229175	DEL	PRECISE	51	0	0	0/0	0/0	0/0	0/0	0/0	0/0	0/0	0/0	0/0	0/0	0/1	1/1	0/0	0/0
Chromosome2-5234414	DEL	PRECISE	615	0	0	0/0	0/0	0/0	0/0	0/0	0/0	0/0	0/0	0/0	0/1	0/1	1/1	0/1	0/0
Chromosome2-5241667	DEL	PRECISE	122	0	0	0/0	0/0	0/0	0/0	0/0	0/0	0/0	0/0	0/0	0/1	0/1	1/1	0/1	0/0
Chromosome2-5246372	DEL	PRECISE	2470	0	0	0/0	0/0	0/0	0/0	0/0	0/0	0/0	0/0	0/0	0/1	0/1	1/1	0/1	0/0
Chromosome2-5254098	DEL	PRECISE	170	0	0	0/0	0/0	0/0	0/0	0/0	0/0	0/0	0/0	0/0	0/1	0/0	0/1	0/0	0/0
Chromosome2-5256627	DEL	PRECISE	55	0	0	0/0	0/0	0/0	0/0	0/0	0/0	0/0	0/0	0/0	0/0	0/0	0/1	0/0	0/0
Chromosome2-5274528	INS	PRECISE	1068	0	0	0/0	0/0	0/1	0/0	0/0	0/0	0/0	0/0	0/0	0/0	0/0	0/0	0/0	0/0
Chromosome2-5276752	DEL	PRECISE	297	0	0	0/0	0/0	0/0	0/0	0/0	0/0	0/0	0/0	0/0	0/0	0/0	0/0	0/0	0/1
Chromosome2-5282812	DEL	PRECISE	71	0	0	0/0	0/0	0/0	0/1	0/0	0/0	0/0	0/0	0/0	0/0	0/0	0/0	0/0	0/0
Chromosome2-5285110	DEL	PRECISE	98	0	0	0/0	0/0	0/0	0/0	0/0	0/0	0/0	0/0	0/0	0/0	0/1	0/1	0/0	0/0
Chromosome2-5286524	DEL	PRECISE	878	0	0	0/0	0/0	0/0	0/0	0/0	0/0	0/0	0/0	0/0	0/0	0/0	0/0	0/0	0/1
Chromosome2-5290330	INS	PRECISE	170	0	0	0/0	0/0	0/0	0/0	0/0	0/0	0/0	0/0	0/0	0/0	0/0	0/0	0/0	0/1
Chromosome2-5296429	DEL	PRECISE	672	0	0	0/0	0/0	0/0	0/0	0/0	0/0	0/0	0/0	0/0	0/1	0/1	1/1	0/1	0/0
Chromosome2-5296436	INS	PRECISE	62	0	0	0/0	0/0	0/0	0/1	0/0	0/0	0/0	0/0	0/0	0/0	0/0	0/0	0/0	0/0
Chromosome2-5303856	DEL	PRECISE	877	0	0	0/0	0/0	0/0	0/0	0/0	0/0	0/0	0/0	0/0	0/1	0/1	1/1	0/1	0/0
Chromosome2-5305498	INS	PRECISE	137	0.577	0	0/0	0/0	0/1	0/1	0/1	0/0	0/0	0/0	0/0	0/0	0/0	0/0	0/0	0/0
Chromosome2-5306824	DEL	PRECISE	265	0	0	0/0	0/0	0/0	0/1	0/0	0/0	0/0	0/0	0/0	0/0	0/0	0/0	0/0	0/0
Chromosome2-5315694	INS	PRECISE	2733	0	0	0/0	0/0	0/0	0/0	0/0	0/0	0/0	0/0	0/0	0/0	0/0	1/1	0/0	0/0
Chromosome2-5329305	INS	PRECISE	148	0	0	0/0	0/0	0/1	0/1	0/0	0/0	0/0	0/0	0/0	0/0	0/0	0/0	0/0	0/0
Chromosome2-5334422	INS	PRECISE	64	0	0	0/0	0/0	0/0	0/0	0/0	0/0	0/0	0/0	0/0	0/0	0/0	0/1	0/0	0/0
Chromosome2-5336580	INS	PRECISE	53	0	0	0/0	0/0	0/0	0/0	0/0	0/0	0/0	0/0	0/0	0/0	0/0	0/1	0/0	0/0
Chromosome2-5338133	DEL	PRECISE	203	0	0	0/0	0/0	0/0	0/0	0/0	0/0	0/0	0/0	0/0	0/0	0/0	1/1	0/0	0/0
Chromosome2-5338507	INS	PRECISE	226	0	0	0/0	0/0	0/0	0/0	0/0	0/0	0/0	0/0	0/0	0/0	0/0	0/0	0/0	0/1
Chromosome2-5345979	INS	PRECISE	66	0.894	0.447	0/0	0/0	0/0	0/0	0/0	0/0	0/0	0/0	0/0	0/0	0/1	1/1	0/1	0/0
Chromosome2-5346643	DEL	PRECISE	995	0	0	0/0	0/0	0/1	1/1	1/1	0/1	1/1	1/1	0/1	0/0	0/1	0/0	0/1	0/0
Chromosome2-5347638	BND	PRECISE	0	0	0	0/0	0/0	0/0	0/0	0/0	0/0	0/0	0/0	0/0	0/0	0/0	0/1	0/0	0/0
Chromosome2-5348430	INS	PRECISE	211	0	0	0/0	0/0	0/1	0/1	0/0	0/0	0/0	0/0	0/0	0/0	0/0	0/0	0/0	0/0
Chromosome2-5349405	BND	PRECISE	0	0	0	0/0	0/0	0/0	0/0	0/0	0/0	0/0	0/0	0/0	0/0	0/0	0/0	0/0	0/1
Chromosome2-5358962	INS	PRECISE	879	0	0	0/0	0/0	0/0	0/0	0/1	0/0	0/0	0/0	0/0	0/0	0/0	0/0	0/0	0/0
Chromosome2-5366440	INS	PRECISE	151	0	0	0/0	0/0	0/0	0/0	0/0	0/0	0/0	0/0	0/0	0/0	0/0	0/0	0/0	0/1
Chromosome2-5368037	INS	PRECISE	10816	0	0	0/0	0/0	0/0	0/1	0/0	0/0	0/0	0/0	0/0	0/0	0/0	0/0	0/0	0/0
Chromosome2-5369631	INS	PRECISE	711	0	0	0/0	0/0	0/0	0/0	0/0	0/0	0/0	0/0	0/0	0/0	1/1	0/0	1/1	0/0
Chromosome2-5373221	INS	PRECISE	876	0	0.577	0/0	0/0	0/1	0/1	0/0	0/0	0/0	0/0	0/0	0/0	0/0	0/0	0/0	0/0
Chromosome2-5374293	INS	PRECISE	867	0	0	0/0	0/0	0/0	0/0	0/0	0/0	0/0	0/0	0/0	1/1	0/0	0/0	0/0	0/0
Chromosome2-5376202	DEL	PRECISE	1551	0	0	0/0	0/0	0/1	1/1	1/1	0/1	1/1	1/1	0/1	0/1	0/1	1/1	0/1	0/1
Chromosome2-5378368	DEL	PRECISE	1673	0	0	0/0	0/0	0/0	0/0	0/0	0/0	0/0	0/0	0/0	0/1	0/1	1/1	0/1	0/0
Chromosome2-5380244	DEL	PRECISE	76	0	0	0/0	0/0	0/1	1/1	0/0	0/0	0/0	0/0	0/0	0/0	0/0	0/0	0/0	0/0
Chromosome2-5395841	DEL	PRECISE	1186	0	0	0/0	0/0	0/0	0/0	0/0	0/0	0/0	0/0	0/0	1/1	1/1	0/0	1/1	0/0
Chromosome2-5397369	DEL	PRECISE	133	0	0	0/0	0/0	0/1	0/1	1/1	0/1	1/1	1/1	0/1	0/0	0/0	0/0	0/0	0/1
Chromosome2-5399288	DEL	PRECISE	1013	0	0	0/0	0/0	0/0	0/0	0/1	0/0	0/0	0/0	0/0	0/0	0/0	0/0	0/0	0/0
Chromosome2-5399872	DEL	PRECISE	87	0	0	0/0	0/0	0/0	0/0	0/0	0/0	0/0	0/0	0/0	0/0	0/1	0/0	0/0	0/0
Chromosome2-5402312	INS	PRECISE	56	0.778	0.289	0/0	0/0	0/1	0/1	1/1	0/1	1/1	1/1	0/1	0/0	0/1	1/1	0/0	0/0
Chromosome2-5410181	DEL	PRECISE	383	0	0	0/0	0/0	0/0	0/0	0/0	0/0	0/0	0/0	0/0	1/1	0/1	1/1	0/1	0/0
Chromosome2-5418451	INS	PRECISE	183	0	0.5	0/0	0/0	0/0	0/0	0/0	0/0	0/0	0/0	0/0	0/1	0/1	1/1	0/1	0/0
Chromosome2-5420791	DEL	PRECISE	127	0	0	0/0	0/0	0/0	0/0	0/0	0/0	0/0	0/0	0/0	0/0	0/1	0/0	0/1	0/0
Chromosome2-5421270	DEL	PRECISE	1011	0	0	0/0	0/0	0/0	0/0	0/0	0/0	0/0	0/0	0/0	0/0	0/1	0/0	0/1	0/0
Chromosome2-5422069	DEL	PRECISE	131	0	0	0/0	0/0	0/0	0/0	0/0	0/0	0/0	0/0	0/0	0/0	0/0	0/0	0/0	0/1
Chromosome2-5423397	INS	PRECISE	282	0	0.707	0/0	0/0	0/0	0/0	0/0	0/0	0/0	0/0	0/0	0/1	0/0	1/1	0/0	0/0
Chromosome2-5423957	DEL	PRECISE	265	0	0	0/0	0/0	0/0	0/0	0/1	0/1	0/1	1/1	0/1	0/0	0/0	0/0	0/0	0/0
Chromosome2-5424579	INS	PRECISE	1139	0	0	0/0	0/0	0/0	0/1	0/0	0/0	0/0	0/0	0/0	0/0	0/0	0/0	0/0	0/0
Chromosome2-5426969	INS	PRECISE	327	0	0.5	0/0	0/0	0/0	0/0	0/0	0/0	0/0	0/0	0/0	0/1	0/1	1/1	0/1	0/0
Chromosome2-5427433	DEL	PRECISE	160	0	0	0/0	0/0	0/1	1/1	0/1	0/0	0/1	0/0	0/0	0/1	0/1	1/1	0/1	0/1
Chromosome2-5431293	INS	PRECISE	10848	0	0	0/0	0/0	0/0	0/0	0/0	0/0	0/0	0/0	0/0	0/0	0/0	0/0	0/0	0/1
Chromosome2-5438691	INS	PRECISE	78	0	0	0/0	0/0	0/0	0/0	0/0	0/0	0/0	0/0	0/0	0/1	0/1	1/1	0/1	0/0
Chromosome2-5449098	DEL	PRECISE	128	0	0	0/0	0/0	0/0	0/0	0/0	0/0	0/0	0/0	0/0	0/1	0/1	1/1	0/1	0/0
Chromosome2-5451766	DEL	PRECISE	90	0	0	0/0	0/0	0/0	0/0	0/0	0/0	0/0	0/0	0/0	0/1	0/0	0/1	0/0	0/0
Chromosome2-5467669	DEL	PRECISE	279	0	0	0/0	0/0	0/0	0/0	0/0	0/0	0/0	0/0	0/0	0/0	0/0	0/1	0/0	0/0
Chromosome2-5472185	DEL	PRECISE	420	0	0	0/0	0/0	0/0	0/0	0/0	0/0	0/0	0/0	0/0	0/1	0/1	1/1	0/1	0/0
Chromosome2-5479272	INS	PRECISE	59	0	0	0/0	0/0	0/1	0/1	0/0	0/0	0/0	0/0	0/0	0/0	0/0	0/0	0/0	0/0
Chromosome2-5481776	DEL	PRECISE	618	0	0	0/0	0/0	0/0	0/0	0/0	0/0	0/0	0/0	0/0	0/0	0/1	1/1	0/1	0/0
Chromosome2-5485773	DEL	PRECISE	580	0	0	0/0	0/0	0/0	0/0	0/0	0/0	0/0	0/0	0/0	0/1	0/1	1/1	0/1	0/0
Chromosome2-5487409	DEL	PRECISE	171	0	0	0/0	0/0	0/0	0/0	0/0	0/0	0/0	0/0	0/0	0/1	0/1	1/1	0/1	0/0
Chromosome2-5511679	DEL	PRECISE	352	0	0	0/0	0/0	0/0	0/0	0/0	0/0	0/0	0/0	0/0	0/1	0/1	1/1	0/1	0/0
Chromosome2-5512057	INS	PRECISE	326	0	0.894	0/0	0/0	0/0	0/0	0/1	0/1	0/1	1/1	0/1	0/0	0/0	0/0	0/0	0/0
Chromosome2-5513719	INS	PRECISE	57	0	0.707	0/0	0/0	0/0	0/0	0/0	0/0	0/0	0/0	0/0	0/0	0/1	0/0	0/1	0/0
Chromosome2-5519589	DEL	PRECISE	805	0	0	0/0	0/0	0/0	0/0	0/0	0/0	0/0	0/0	0/0	0/1	0/1	1/1	0/1	1/1
Chromosome2-5519849	DEL	PRECISE	55	0	0	0/0	0/0	0/0	0/0	0/1	0/0	0/0	0/0	0/0	0/0	0/0	0/0	0/0	0/0
Chromosome2-5527215	DEL	PRECISE	5398	0.354	0.354	0/0	0/0	0/1	1/1	1/1	0/1	1/1	1/1	0/0	0/0	0/0	0/0	0/0	0/1
Chromosome2-5532702	INS	PRECISE	93	0	0	0/0	0/0	0/0	0/0	0/1	0/0	0/0	0/0	0/0	0/0	0/0	0/0	0/0	0/0

Chromosome2-5537132	DEL	PRECISE	56	0	0	0/0	0/0	0/0	0/0	0/0	0/0	0/0	0/0	0/0	0/1	0/1	1/1	0/1	0/0
Chromosome2-5540511	DEL	PRECISE	6293	0	0	0/0	0/0	0/0	0/0	0/1	0/0	0/0	0/0	0/0	0/0	0/0	0/0	0/0	0/0
Chromosome2-5546856	INS	PRECISE	1376	0	0	0/0	0/0	0/0	0/0	0/0	0/0	0/0	0/0	0/0	0/0	0/0	0/1	0/0	0/0
Chromosome2-5552353	DEL	PRECISE	1083	0	0	0/0	0/0	0/0	0/0	0/1	0/0	0/0	0/0	0/0	0/1	0/1	1/1	0/1	0/1
Chromosome2-5558705	INS	PRECISE	10543	0	0	0/0	0/0	0/0	0/0	0/0	0/0	0/0	0/0	0/0	0/0	0/0	0/0	0/0	0/0
Chromosome2-5559436	DEL	PRECISE	308	0	0	0/0	0/0	0/0	0/0	0/1	0/0	0/1	1/1	0/1	0/0	0/0	0/0	0/0	0/0
Chromosome2-5568151	BND	PRECISE	0	0.447	0	0/0	0/0	0/0	0/0	0/0	0/0	0/1	0/1	0/0	0/0	0/0	0/0	0/0	0/0
Chromosome2-5569726	DEL	PRECISE	171	0.5	0	0/0	0/0	0/0	0/0	0/0	0/0	0/0	0/0	0/0	0/1	0/0	0/0	0/1	0/0
Chromosome2-5570101	INS	PRECISE	276	0	0.707	0/0	0/0	0/0	0/0	0/0	0/0	0/0	0/0	0/0	0/0	0/0	0/0	0/1	0/0
Chromosome2-5572643	DEL	PRECISE	74	0	0	0/0	0/0	0/0	0/0	0/0	0/0	0/0	0/0	0/0	0/1	0/1	0/1	0/1	0/0
Chromosome2-5579464	DEL	PRECISE	79	0	0	0/0	0/0	0/0	0/0	0/1	0/1	0/0	0/0	0/0	0/0	0/0	0/0	0/0	0/0
Chromosome2-5582581	DEL	PRECISE	131	0	0	0/0	0/0	0/0	0/0	0/0	0/0	0/0	0/0	0/0	0/0	0/0	1/1	0/1	0/0
Chromosome2-5583502	DEL	PRECISE	1006	0	0	0/0	0/0	0/0	0/0	0/0	0/0	0/0	0/0	0/0	0/1	0/1	1/1	0/1	0/1
Chromosome2-5596770	DEL	PRECISE	159	0	0	0/0	0/0	0/1	0/1	1/1	1/1	0/1	1/1	0/1	0/0	0/0	0/0	0/0	0/1
Chromosome2-5597918	INS	PRECISE	81	0	0	0/0	0/0	0/0	0/0	0/1	0/1	0/0	0/0	0/0	0/0	0/0	0/0	0/0	0/0
Chromosome2-5603510	DEL	PRECISE	84	0	0	0/0	0/0	0/0	0/0	0/0	0/0	0/0	0/0	0/0	0/0	0/1	1/1	0/1	0/0
Chromosome2-5609214	DEL	PRECISE	294	0	0	0/0	0/0	0/0	0/0	1/1	1/1	0/0	1/1	0/0	0/1	0/1	1/1	0/1	0/1
Chromosome2-5610176	INS	PRECISE	869	0	0	0/0	0/0	0/0	0/0	0/0	0/0	0/0	0/0	0/0	0/0	0/0	0/0	0/0	0/1
Chromosome2-5618279	DEL	PRECISE	261	0	0	0/0	0/0	0/0	0/0	0/0	0/0	0/0	0/0	0/0	0/0	0/0	0/1	0/0	0/0
Chromosome2-5625929	BND	PRECISE	0	0	0	0/0	0/0	0/0	0/0	0/1	0/0	0/0	0/0	0/0	0/0	0/0	0/0	0/0	0/0
Chromosome2-5626564	INS	PRECISE	50	0	0	0/0	0/0	0/0	0/0	0/0	0/0	0/0	0/0	0/0	0/1	0/1	1/1	0/1	0/0
Chromosome2-5633795	DEL	PRECISE	325	0	0	0/0	0/0	0/0	0/0	0/0	0/0	0/0	0/0	0/0	0/1	0/1	1/1	0/1	0/0
Chromosome2-5634643	INS	PRECISE	121	0	0	0/0	0/0	0/0	0/0	0/0	0/0	0/0	0/0	0/0	0/0	0/0	0/0	0/0	0/1
Chromosome2-5635386	DEL	PRECISE	103	0	0	0/0	0/0	0/0	0/0	0/0	0/0	0/0	0/0	0/0	0/1	0/1	1/1	0/1	0/0
Chromosome2-5644815	DEL	PRECISE	61	0	0	0/0	0/0	0/0	0/0	0/0	0/0	0/1	0/0	0/0	0/0	0/1	1/1	0/1	0/1
Chromosome2-5647939	DEL	PRECISE	82	0	0	0/0	0/0	0/0	1/1	0/1	0/1	1/1	1/1	0/1	0/0	0/0	0/0	0/0	0/0
Chromosome2-5665675	DEL	PRECISE	61	0	0	0/0	0/0	0/0	0/0	0/0	0/0	1/1	0/0	0/0	0/0	0/0	0/0	0/0	0/1
Chromosome2-5684802	DEL	PRECISE	890	0	0	0/0	0/0	0/0	0/0	0/0	0/0	0/0	0/0	0/0	0/1	0/1	1/1	0/1	0/0
Chromosome2-5689377	INS	PRECISE	201	0	0.816	0/0	0/0	0/0	0/0	0/0	0/0	0/0	0/0	0/0	1/1	0/1	1/1	0/1	0/0
Chromosome2-5708893	DEL	PRECISE	890	0	0	0/0	0/0	0/0	0/0	0/0	0/0	0/0	0/0	0/0	0/0	1/1	1/1	1/1	0/0
Chromosome2-5710582	INS	PRECISE	5694	0	0	0/0	0/0	0/0	0/0	0/0	0/0	0/0	0/0	0/0	0/0	0/1	0/0	0/0	0/0
Chromosome2-5716081	DEL	PRECISE	303	0.5	0.5	0/0	0/0	0/0	0/0	0/0	0/0	0/0	0/0	0/0	0/0	0/1	1/1	0/1	0/0
Chromosome2-5720935	DEL	PRECISE	882	0	0	0/0	0/0	0/0	0/0	0/0	0/0	0/0	0/0	0/0	0/1	0/1	0/1	0/0	0/0
Chromosome2-5722919	BND	PRECISE	0	0	0	0/0	0/0	0/0	0/0	0/1	0/1	0/0	0/0	0/1	0/0	0/0	0/0	0/0	0/0
Chromosome2-5725502	INS	PRECISE	201	0	0.5	0/0	0/0	0/0	0/0	0/0	0/0	0/0	0/0	0/0	0/1	0/1	0/1	0/1	0/0
Chromosome2-5734607	DEL	PRECISE	7310	0	0	0/0	0/0	0/0	0/0	0/0	0/0	0/0	0/0	0/0	0/0	0/0	0/0	0/0	0/1
Chromosome2-5737515	DUP	PRECISE	19142	0	0	0/0	0/0	0/1	0/1	0/0	0/0	0/0	0/0	0/0	0/0	0/0	0/0	0/0	0/0
Chromosome2-5748180	DEL	PRECISE	77	0	0	0/0	0/0	0/0	0/0	0/0	0/0	0/0	0/0	0/0	0/0	0/0	0/1	0/0	0/0
Chromosome2-5755370	BND	PRECISE	0	0	0	0/0	0/0	0/1	0/1	0/0	0/0	0/0	0/0	0/0	0/0	0/0	0/0	0/0	0/0
Chromosome2-5756289	DEL	PRECISE	52	0	0	0/0	0/0	0/0	0/0	0/0	0/0	0/0	0/0	0/0	0/1	0/1	1/1	0/1	0/0
Chromosome2-5758345	INS	PRECISE	5285	0	0	0/0	0/0	0/0	1/1	0/0	0/0	0/0	0/0	0/0	0/0	0/0	0/0	0/0	0/0
Chromosome2-5758473	INS	PRECISE	126	0	0	0/0	0/0	0/0	0/0	0/0	0/0	0/0	0/0	0/0	0/1	0/0	1/1	0/0	0/0
Chromosome2-5763708	INS	PRECISE	57	0	0	0/0	0/0	0/1	0/1	0/0	0/0	0/0	0/0	0/0	0/0	0/0	0/0	0/0	0/0
Chromosome2-5771004	DEL	PRECISE	1288	0	0	0/0	0/0	0/0	0/0	0/0	0/0	0/0	0/0	0/0	0/1	0/1	1/1	0/1	0/0
Chromosome2-5773991	INS	PRECISE	132	0	0	0/0	0/0	0/0	0/0	0/0	0/0	0/0	0/0	0/0	0/1	0/0	0/1	0/0	0/0
Chromosome2-5779282	INS	PRECISE	704	0	0	0/0	0/0	0/0	0/0	0/0	0/0	0/0	0/0	0/0	0/0	0/0	0/0	0/0	0/1
Chromosome2-5787755	INS	PRECISE	2398	0	0	0/0	0/0	0/0	0/1	0/0	0/0	0/0	0/0	0/0	0/0	0/0	0/0	0/0	0/0
Chromosome2-5792641	INS	PRECISE	890	0	0	0/0	0/0	0/0	0/0	0/1	0/0	0/0	0/0	0/0	0/0	0/0	0/0	0/0	0/0
Chromosome2-5793198	DEL	PRECISE	159	0	0	0/0	0/0	0/1	0/1	0/0	0/0	0/0	0/0	0/0	0/1	0/1	1/1	0/1	0/1
Chromosome2-5794141	DEL	PRECISE	628	0	0	0/0	0/0	0/1	0/1	0/0	0/0	0/0	0/0	0/0	0/0	0/0	0/0	0/0	0/0
Chromosome2-5798899	BND	PRECISE	0	0	0	0/0	0/0	0/1	0/1	0/0	0/0	0/0	0/0	0/0	0/0	0/0	0/0	0/0	0/0
Chromosome2-5799711	DEL	PRECISE	84	0	0	0/0	0/0	0/0	0/0	0/0	0/0	0/0	0/0	0/0	0/1	0/1	1/1	0/1	0/0
Chromosome2-5801510	INS	PRECISE	137	0	0.707	0/0	0/0	0/0	0/0	0/0	0/0	0/0	0/0	0/0	0/1	0/0	0/1	0/0	0/0
Chromosome2-5808072	INS	PRECISE	69	0	0	0/0	0/0	0/0	0/0	0/0	0/0	0/0	0/0	0/0	0/1	0/1	1/1	0/1	0/0
Chromosome2-5809504	DEL	PRECISE	91	0	0	0/0	0/0	0/0	0/0	0/0	0/0	0/0	0/0	0/0	0/0	0/1	1/1	0/1	0/0
Chromosome2-5810582	DEL	PRECISE	2252	0	0	0/0	0/0	0/0	0/0	0/0	0/0	0/0	0/0	0/0	0/0	0/0	0/0	0/0	0/1
Chromosome2-5811828	DEL	PRECISE	399	0	0	0/0	0/0	0/0	0/0	0/1	0/1	1/1	1/1	0/1	0/0	0/0	0/0	0/0	0/0
Chromosome2-5825514	DEL	PRECISE	7391	0	0	0/0	0/0	0/0	0/0	0/0	0/0	0/0	0/0	0/0	0/1	0/0	1/1	0/0	0/0
Chromosome2-5826172	INS	PRECISE	90	0	0.354	0/0	0/0	0/1	0/1	1/1	1/1	1/1	1/1	0/1	0/0	0/0	0/0	0/0	0/1
Chromosome2-5828423	DEL	PRECISE	50	0	0	0/0	0/0	0/0	0/0	0/1	0/1	1/1	1/1	0/1	0/0	0/0	0/0	0/0	0/0
Chromosome2-5839489	DEL	PRECISE	255	0	0	0/0	0/0	0/0	0/0	0/0	0/0	0/0	0/0	0/0	0/0	0/0	1/1	0/0	0/1
Chromosome2-5840942	DEL	PRECISE	154	0	0	0/0	0/0	0/0	0/0	0/0	0/0	0/0	0/0	0/0	0/0	0/0	0/0	0/0	0/1
Chromosome2-5842104	DEL	PRECISE	93	0	0	0/0	0/0	0/0	0/0	0/0	0/0	0/0	0/0	0/0	0/0	0/0	0/0	0/0	0/1
Chromosome2-5844668	DEL	PRECISE	426	0	0	0/0	0/0	0/1	0/1	1/1	1/1	1/1	1/1	0/1	0/1	0/1	1/1	0/1	0/1
Chromosome2-5852544	DEL	PRECISE	944	0	0	0/0	0/0	0/1	0/0	0/1	0/1	0/0	0/0	0/0	0/1	0/1	1/1	0/1	0/1
Chromosome2-5854883	INS	PRECISE	3989	0	0.707	0/0	0/0	0/0	0/0	0/0	0/0	0/0	0/0	0/0	0/0	1/1	0/0	1/1	0/0
Chromosome2-5860734	INS	PRECISE	2562	0	0	0/0	0/0	0/0	0/0	0/0	0/0	0/0	0/0	0/0	0/0	1/1	0/0	1/1	0/0
Chromosome2-5864364	DEL	PRECISE	55	0	0	0/0	0/0	0/0	0/0	0/0	0/0	0/0	0/0	0/0	0/0	0/1	0/1	0/1	0/0
Chromosome2-5867631	DEL	PRECISE	80	0	0	0/0	0/0	0/0	0/0	0/0	0/0	0/0	0/0	0/0	0/1	0/0	1/1	0/0	0/0
Chromosome2-5878745	DEL	PRECISE	1368	0	0	0/0	0/0	0/1	0/1	0/0	0/0	0/0	0/0	0/0	0/0	0/0	0/0	0/0	0/1
Chromosome2-5879192	DEL	PRECISE	778	0	0	0/0	0/0	0/0	0/0	0/0	0/0	0/0	0/0	0/0	0/1	0/1	1/1	0/1	0/0
Chromosome2-5880495	INS	PRECISE	82	0	0.707	0/0	0/0	0/0	0/0	0/0	0/0	0/0	0/0	0/0	0/1	0/0	1/1	0/0	0/0
Chromosome2-5881139	DEL	PRECISE	83	0	0	0/0	0/0	0/0	0/0	0/0	0/0	0/0	0/0	0/0	0/0	0/0	0/0	0/0	0/1

Chromosome2-5883721	INS	PRECISE	363	0	0	0/0	0/0	0/0	0/0	0/0	0/0	0/0	0/0	0/0	0/1	0/0	1/1	0/0	0/0
Chromosome2-5889216	BND	PRECISE	0	0	0	0/0	0/0	0/0	0/0	0/1	0/1	0/0	0/0	0/0	0/0	0/0	0/0	0/0	0/0
Chromosome2-5889226	INS	PRECISE	19071	0	0	0/0	0/0	0/0	0/0	0/1	0/0	0/0	0/0	0/0	0/0	0/0	0/0	0/0	0/0
Chromosome2-5895230	INS	PRECISE	5624	0	0	0/0	0/0	0/0	1/1	0/0	0/0	0/0	0/0	0/0	0/0	0/0	0/0	0/0	0/0
Chromosome2-5900588	DEL	PRECISE	284	0	0	0/0	0/0	0/0	0/0	0/0	0/0	0/0	0/0	0/0	0/1	0/0	1/1	0/0	0/0
Chromosome2-5907719	DEL	PRECISE	56	0	0	0/0	0/0	0/0	0/0	0/0	0/0	0/1	0/0	0/0	0/0	0/0	0/0	0/0	0/0
Chromosome2-5931479	DEL	PRECISE	81	0	0	0/0	0/0	0/1	0/1	0/1	0/1	0/1	1/1	0/1	0/0	0/0	0/0	0/0	0/0
Chromosome2-5941653	INS	PRECISE	90	0	0	0/0	0/0	0/0	0/0	0/0	0/0	0/0	0/0	0/0	0/1	0/1	1/1	1/1	0/0
Chromosome2-5958928	INS	PRECISE	53	0	0	0/0	0/0	0/0	0/0	0/0	0/0	0/0	0/0	0/0	0/1	0/1	1/1	1/1	0/0
Chromosome2-5961166	DEL	PRECISE	1080	0	0	0/0	0/0	0/1	0/1	0/1	0/1	0/0	0/0	0/0	0/1	0/1	1/1	1/1	0/1
Chromosome2-5998249	DEL	PRECISE	66	0	0	0/0	0/0	0/1	0/1	0/1	0/1	0/0	0/0	0/0	0/0	0/0	0/0	0/0	0/0
Chromosome2-5999856	INS	PRECISE	1006	0	0	0/0	0/0	0/0	0/0	0/0	0/0	0/0	0/0	0/0	0/0	0/1	0/0	0/1	0/0
Chromosome2-6001046	INS	PRECISE	77	0	0.408	0/0	0/0	0/0	0/1	0/1	0/1	1/1	1/1	0/1	0/0	0/0	0/0	0/0	0/0
Chromosome2-6002215	INS	PRECISE	877	0	0.707	0/0	0/0	0/0	0/0	0/0	0/0	0/0	0/0	0/0	0/0	0/1	0/0	0/0	0/0
Chromosome2-6010760	DEL	PRECISE	54	0	0	0/0	0/0	0/0	0/0	0/0	0/0	0/0	0/0	0/0	0/1	0/1	1/1	0/1	0/0
Chromosome2-6012388	INS	PRECISE	58	0	0	0/0	0/0	0/0	0/0	0/0	0/0	0/0	0/0	0/0	0/0	0/0	0/0	0/0	0/1
Chromosome2-6018414	DEL	PRECISE	64	0	0	0/0	0/0	0/0	0/0	0/0	0/0	0/0	0/0	0/0	0/1	0/0	1/1	0/0	0/0
Chromosome2-6022204	DEL	PRECISE	454	0	0	0/0	0/0	0/1	1/1	1/1	1/1	1/1	1/1	0/1	0/1	0/1	1/1	0/1	0/1
Chromosome2-6037132	DEL	PRECISE	8186	0	0	0/0	0/0	0/0	0/0	0/1	0/1	0/1	1/1	0/1	0/0	0/0	0/0	0/0	0/0
Chromosome2-6041101	DEL	PRECISE	1046	0	0	0/0	0/0	0/1	1/1	0/1	0/1	0/1	0/0	0/0	0/1	0/1	1/1	0/1	0/1
Chromosome2-6043469	DEL	PRECISE	182	0	0	0/0	0/0	0/0	0/0	0/0	0/0	0/0	0/0	0/0	0/0	0/0	0/0	0/0	0/1
Chromosome2-6046967	INS	PRECISE	1907	0	0.707	0/0	0/0	0/0	0/0	0/0	0/0	0/0	0/0	0/0	0/0	0/1	0/0	0/1	0/0
Chromosome2-6049521	DUP	PRECISE	9350	0	0	0/0	0/0	0/1	0/0	0/0	0/0	0/0	0/0	0/0	0/0	0/0	0/0	0/0	0/0
Chromosome2-6050502	INS	PRECISE	3448	0	0	0/0	0/0	0/0	0/0	0/0	0/0	0/0	0/0	0/0	0/0	1/1	0/0	1/1	0/0
Chromosome2-6050502	INS	PRECISE	4948	0	0	0/0	0/0	0/0	0/0	0/0	0/0	0/0	0/0	0/0	0/0	0/0	0/0	0/0	0/1
Chromosome2-6054987	INS	PRECISE	2053	0	0	0/0	0/0	0/0	0/0	0/0	0/0	0/0	0/0	0/0	0/0	0/0	0/0	0/0	0/1
Chromosome2-6058027	DEL	PRECISE	68	0	0	0/0	0/0	0/0	0/0	0/0	0/0	0/0	0/0	0/0	0/1	0/0	1/1	0/0	0/0
Chromosome2-6061682	INS	PRECISE	996	0	0	0/0	0/0	0/0	0/1	0/0	0/0	0/0	0/0	0/0	0/0	0/0	0/0	0/0	0/0
Chromosome2-6062209	INS	PRECISE	104	0	0	0/0	0/0	0/0	0/0	0/0	0/0	0/0	0/0	0/0	0/1	0/0	1/1	0/0	0/0
Chromosome2-6066627	INS	PRECISE	531	0	0.835	0/0	0/0	0/1	1/1	1/1	1/1	1/1	1/1	0/1	0/0	0/0	0/0	0/0	0/1
Chromosome2-6067508	DEL	PRECISE	80	0	0	0/0	0/0	0/0	0/0	0/0	0/0	0/1	0/0	0/0	0/0	0/0	0/0	0/0	0/0
Chromosome2-6068702	INS	PRECISE	6290	0	0	0/0	0/0	0/0	0/1	0/0	0/0	0/0	0/0	0/0	0/0	0/0	0/0	0/0	0/0
Chromosome2-6077494	INS	PRECISE	71	0	0	0/0	0/0	0/0	0/0	0/0	0/0	0/1	0/0	0/0	0/0	0/0	0/0	0/0	0/0
Chromosome2-6080113	DEL	PRECISE	69	0	0	0/0	0/0	0/0	0/0	0/0	0/0	0/0	0/0	0/0	0/1	0/0	1/1	0/0	0/0
Chromosome2-6082080	DEL	PRECISE	1076	0	0	0/0	0/0	0/1	0/1	0/1	0/1	0/0	0/0	0/0	0/0	0/0	0/0	0/0	0/0
Chromosome2-6082514	INS	PRECISE	211	0	0	0/0	0/0	0/0	0/0	0/1	0/1	1/1	1/1	0/1	0/0	0/0	0/0	0/0	0/0
Chromosome2-6086867	INS	PRECISE	117	0	0	0/0	0/0	0/0	0/0	0/0	0/0	0/0	0/0	0/0	0/0	0/1	0/0	0/1	0/0
Chromosome2-6095649	DEL	PRECISE	277	0	0	0/0	0/0	0/0	0/0	0/0	0/0	0/0	0/0	0/0	0/0	0/1	0/0	0/1	0/0
Chromosome2-6099249	INS	PRECISE	56	0	0	0/0	0/0	0/0	0/0	0/0	0/0	0/0	0/0	0/0	0/1	0/0	1/1	0/0	0/0
Chromosome2-6106772	DEL	PRECISE	61	0	0	0/0	0/0	0/0	0/1	0/0	0/0	0/0	0/0	0/0	0/0	0/0	0/0	0/0	0/0
Chromosome2-6115408	INS	PRECISE	105	0	0.5	0/0	0/0	0/0	0/0	0/0	0/0	0/0	0/0	0/0	1/1	0/1	1/1	0/1	0/0
Chromosome2-6123300	INS	PRECISE	752	0	0.5	0/0	0/0	0/0	0/0	0/0	0/0	0/0	0/0	0/0	0/1	0/1	1/1	0/1	0/0
Chromosome2-6131299	BND	PRECISE	0	0	0	0/0	0/0	0/0	0/0	0/0	0/0	0/0	0/0	0/0	0/0	0/0	0/0	0/0	0/1
Chromosome2-6136341	BND	PRECISE	0	0	0	0/0	0/0	0/0	0/0	0/0	0/0	0/0	0/0	0/0	0/0	0/1	0/1	0/0	0/0
Chromosome2-6136348	BND	PRECISE	0	0	0	0/0	0/0	0/0	0/0	0/0	0/0	0/0	0/0	0/0	0/1	0/1	1/1	0/1	0/0
Chromosome2-6139938	DEL	PRECISE	150	0	0	0/0	0/0	0/0	0/0	0/1	0/1	0/0	0/0	0/0	0/0	0/0	0/0	0/0	0/0
Chromosome2-6142802	INS	PRECISE	97	0	0	0/0	0/0	0/0	0/0	0/1	0/1	0/0	0/0	0/0	0/0	0/0	0/0	0/0	0/0
Chromosome2-6144990	INS	PRECISE	54	0	0.707	0/0	0/0	0/1	1/1	0/1	0/1	0/0	0/0	0/0	0/0	0/0	0/0	0/0	0/1
Chromosome2-6145648	DEL	PRECISE	60	0	0	0/0	0/0	0/0	1/1	0/0	0/0	0/0	0/0	0/0	0/0	0/0	0/0	0/0	0/1
Chromosome2-6145689	DEL	PRECISE	36253	0	0	0/0	0/0	0/0	0/1	0/1	0/1	0/0	0/0	0/0	0/0	0/0	0/0	0/0	0/0
Chromosome2-6152484	DEL	PRECISE	161	0	0	0/0	0/0	0/0	0/0	0/0	0/0	0/0	0/0	0/0	0/1	0/1	1/1	0/1	0/0
Chromosome2-6155839	INS	PRECISE	214	0	0	0/0	0/0	0/0	0/0	0/0	0/0	0/0	0/0	0/0	0/1	0/1	1/1	0/1	0/0
Chromosome2-6157100	DEL	PRECISE	13462	0	0	0/0	0/0	0/0	0/0	0/0	0/0	0/0	0/0	0/0	0/1	0/1	1/1	0/1	0/0
Chromosome2-6177088	DEL	PRECISE	1112	0	0	0/0	0/0	0/0	1/1	1/1	1/1	1/1	1/1	0/1	0/0	0/0	0/0	0/0	0/1
Chromosome2-6204964	INS	PRECISE	5415	0	0	0/0	0/0	0/0	1/1	0/0	0/0	0/0	0/0	0/0	0/0	0/0	0/0	0/0	0/0
Chromosome2-6204971	BND	PRECISE	0	0	0	0/0	0/0	0/0	0/1	0/0	0/0	0/0	0/0	0/0	0/0	0/0	0/0	0/0	0/0
Chromosome2-6206429	DEL	PRECISE	63	0	0	0/0	0/0	0/0	0/1	0/0	0/0	0/0	0/0	0/0	0/0	0/0	0/0	0/0	0/0
Chromosome2-6222730	DEL	PRECISE	186	0	0	0/0	0/0	0/0	0/0	0/0	0/0	0/0	0/0	0/0	0/0	0/1	1/1	0/1	0/0
Chromosome2-6224219	DEL	PRECISE	188	0	0	0/0	0/0	0/0	0/0	0/0	0/0	0/0	0/0	0/0	0/0	0/0	0/0	0/0	0/1
Chromosome2-6224724	INS	PRECISE	359	0	0.447	0/0	0/0	0/0	0/0	0/1	0/1	1/1	1/1	0/1	0/0	0/0	0/0	0/0	0/0
Chromosome2-6228641	INS	PRECISE	1025	0	0	0/0	0/0	0/0	0/0	0/0	0/0	0/1	0/0	0/0	0/0	0/0	0/0	0/0	0/0
Chromosome2-6238150	INS	PRECISE	462	0	0.707	0/0	0/0	0/0	0/0	0/0	0/0	0/0	0/0	0/0	0/1	0/0	0/1	0/0	0/0
Chromosome2-6241860	INS	PRECISE	642	0	0	0/0	0/0	0/0	0/0	0/1	1/1	0/0	0/0	0/0	0/0	0/0	0/0	0/0	0/0
Chromosome2-6242923	DEL	PRECISE	63	0	0	0/0	0/0	0/0	0/0	0/0	0/0	0/0	0/0	0/0	0/0	0/1	1/1	0/1	0/0
Chromosome2-6244411	INS	PRECISE	536	0	0.548	0/0	0/0	0/0	0/0	1/1	0/1	1/1	0/0	1/1	0/0	0/0	0/0	0/0	0/0
Chromosome2-6244815	DEL	PRECISE	198	0	0	0/0	0/0	0/0	0/0	0/1	0/1	0/1	1/1	0/1	0/0	0/0	0/0	0/0	0/0
Chromosome2-6245469	INS	PRECISE	334	0	0.707	0/0	0/0	0/0	0/0	0/0	0/0	0/0	0/0	0/0	0/0	0/0	0/1	0/0	0/0
Chromosome2-6245688	DEL	PRECISE	50	0	0	0/0	0/0	0/1	0/1	1/1	1/1	1/1	1/1	0/1	0/0	0/0	0/0	0/0	0/1
Chromosome2-6246666	INS	PRECISE	139	0	0.707	0/0	0/0	0/0	0/0	0/0	0/0	0/0	0/0	0/0	0/1	0/0	0/1	0/0	0/0
Chromosome2-6248290	DEL	PRECISE	285	0	0	0/0	0/0	0/0	0/0	0/0	0/0	0/0	0/0	0/0	0/1	0/0	0/1	0/0	0/0
Chromosome2-6256285	INS	PRECISE	1987	0	0	0/0	0/0	0/0	0/0	0/0	0/0	0/0	0/0	0/0	0/0	0/0	0/0	0/0	0/1
Chromosome2-6256434	INS	PRECISE	53	0	0	0/0	0/0	0/0	0/0	0/1	0/1	0/1	1/1	0/1	0/0	0/0	0/0	0/0	0/0
Chromosome2-6262801	DEL	PRECISE	168	0	0	0/0	0/0	0/0	0/0	0/0	0/0	0/0	0/0	0/0	0/0	0/1	1/1	0/1	0/0

Chromosome2-6264139	INS	PRECISE	51	0	0	0/0	0/0	0/0	0/0	1/1	1/1	0/1	1/1	0/1	0/0	0/0	0/0	0/0	0/0
Chromosome2-6268181	INS	PRECISE	878	0	0.447	0/0	0/0	0/0	0/0	0/1	0/1	1/1	1/1	0/1	0/0	0/0	0/0	0/0	0/0
Chromosome2-6275337	DEL	PRECISE	140	0.5	0.5	0/0	0/0	0/0	0/0	0/0	0/0	0/0	0/0	0/0	0/0	0/1	1/1	0/1	0/0
Chromosome2-6282708	INS	PRECISE	51	0	0	0/0	0/0	0/1	0/1	1/1	1/1	1/1	1/1	0/1	0/1	0/1	1/1	0/1	0/1
Chromosome2-6284277	DEL	PRECISE	216	0	0	0/0	0/0	0/0	0/0	0/0	0/0	0/0	0/0	0/0	0/1	0/1	1/1	0/1	0/0
Chromosome2-6285798	INS	PRECISE	126	0	0.5	0/0	0/0	0/0	0/0	0/0	0/0	0/0	0/0	0/0	0/1	0/1	1/1	0/1	0/0
Chromosome2-6290835	DEL	PRECISE	171	0	0	0/0	0/0	0/0	0/0	0/0	0/0	0/0	0/0	0/0	0/0	0/0	0/0	0/0	0/1
Chromosome2-6291814	DEL	PRECISE	632	0	0	0/0	0/0	0/0	0/0	0/0	0/0	0/0	0/0	0/0	0/1	0/1	1/1	0/1	0/0
Chromosome2-6293209	INS	PRECISE	132	0	0	0/0	0/0	0/0	0/0	0/0	0/0	0/0	0/0	0/0	0/1	0/1	1/1	0/1	0/0
Chromosome2-6297196	INS	PRECISE	63	0	0	0/0	0/0	0/0	0/0	0/0	0/0	0/0	0/0	0/0	0/1	0/1	1/1	0/1	0/0
Chromosome2-6304316	DEL	PRECISE	283	0	0	0/0	0/0	0/0	0/0	0/0	0/0	0/0	0/0	0/0	0/1	0/1	1/1	1/1	0/0
Chromosome2-6319246	INS	PRECISE	1359	0	0	0/0	0/0	0/0	0/0	0/0	0/0	0/0	0/0	0/0	0/0	0/0	0/0	0/0	0/1
Chromosome2-6321506	DEL	PRECISE	78	0	0	0/0	0/0	0/0	0/0	0/0	0/0	0/0	0/0	0/0	0/1	0/1	1/1	0/0	0/0
Chromosome2-6329146	DEL	PRECISE	538	0	0	0/0	0/0	0/1	0/1	0/0	0/1	0/0	0/0	0/0	0/0	0/0	0/0	0/0	0/1
Chromosome2-6330802	INS	PRECISE	628	0	0	0/0	0/0	0/1	0/1	0/0	0/0	0/0	0/0	0/0	0/0	0/0	0/0	0/0	0/0
Chromosome2-6333752	DEL	PRECISE	89	0	0	0/0	0/0	0/0	0/0	0/1	0/1	0/0	0/0	0/0	0/0	0/0	0/0	0/0	0/0
Chromosome2-6352899	DEL	PRECISE	1117	0	0	0/0	0/0	0/0	0/0	0/0	0/0	0/0	0/0	0/0	0/0	1/1	1/1	0/0	0/0
Chromosome2-6358315	DEL	PRECISE	330	0	0	0/0	0/0	0/1	0/1	0/0	0/0	0/0	0/0	0/0	0/0	0/0	0/0	0/0	0/1
Chromosome2-6367518	INS	PRECISE	124	0	0	0/0	0/0	0/0	0/0	0/1	0/1	0/0	0/0	0/0	0/0	0/0	0/0	0/0	0/0
Chromosome2-6370206	INS	PRECISE	125	0	0	0/0	0/0	0/0	0/0	0/0	0/0	0/0	0/0	0/0	0/1	0/0	0/1	0/0	0/0
Chromosome2-6374440	INS	PRECISE	6088	0	0	0/0	0/0	0/0	0/0	0/0	0/0	1/1	0/0	0/0	0/0	0/0	0/0	0/0	0/0
Chromosome2-6385560	DEL	PRECISE	133	0	0	0/0	0/0	0/1	0/1	1/1	1/1	1/1	1/1	0/1	0/0	0/0	0/1	0/1	0/0
Chromosome2-6387841	DUP	PRECISE	211	0	0	0/0	0/0	0/0	0/0	0/0	0/0	0/0	0/0	0/0	0/0	0/0	0/1	0/1	0/0
Chromosome2-6406482	INS	PRECISE	3036	0	0	0/0	0/0	0/0	0/0	0/1	0/1	0/0	0/0	0/0	0/0	0/0	0/0	0/0	0/0
Chromosome2-6427463	INS	PRECISE	58	0	0	0/0	0/0	0/0	0/0	0/0	0/0	0/1	0/0	0/0	0/0	0/0	0/0	0/0	0/0
Chromosome2-6431579	INS	PRECISE	56	0	0.408	0/0	0/0	0/1	0/1	0/1	0/1	0/0	0/0	0/0	0/0	0/0	0/0	0/0	0/1
Chromosome2-6454441	DEL	PRECISE	5792	0	0	0/0	0/0	0/0	0/0	0/0	0/0	0/0	0/0	0/0	0/1	0/1	1/1	0/1	0/0
Chromosome2-6458452	BND	PRECISE	0	0.894	0	0/0	0/0	0/0	0/0	0/0	0/0	0/1	0/1	0/0	0/0	0/0	0/0	0/0	0/0
Chromosome2-6458457	BND	PRECISE	0	0	0	0/0	0/0	0/0	0/0	0/0	0/0	0/1	0/1	0/0	0/0	0/0	0/0	0/0	0/0
Chromosome2-6462845	DEL	PRECISE	148	0	0	0/0	0/0	0/0	0/0	0/0	0/0	0/0	0/0	0/0	0/1	0/1	1/1	0/1	0/0
Chromosome2-6469210	INS	PRECISE	125	0	0	0/0	0/0	0/0	0/0	0/0	0/0	0/0	0/0	0/0	0/0	0/0	0/0	0/0	0/1
Chromosome2-6473790	BND	PRECISE	0	0.577	0	0/0	0/0	0/0	0/0	0/0	0/0	0/0	0/0	0/0	0/0	0/1	1/1	0/1	0/0
Chromosome2-6475968	INS	PRECISE	557	0	0	0/0	0/0	0/0	0/0	0/0	0/0	0/0	0/0	0/0	0/0	0/0	0/0	0/0	0/1
Chromosome2-6476154	DEL	PRECISE	62	0	0	0/0	0/0	0/0	0/0	0/0	0/0	0/0	0/0	0/0	0/1	0/1	1/1	1/1	0/0
Chromosome2-6477161	INS	PRECISE	1982	0	0.707	0/0	0/0	0/1	0/1	0/0	0/0	0/0	0/0	0/0	0/0	0/0	0/0	0/0	0/0
Chromosome2-6481454	DEL	PRECISE	876	0	0	0/0	0/0	0/1	0/1	0/1	0/1	0/0	0/0	0/0	0/1	0/1	1/1	0/1	0/1
Chromosome2-6481470	INS	PRECISE	109	0.447	0.447	0/0	0/0	0/0	0/0	0/1	0/1	0/1	1/1	0/1	0/0	0/0	0/0	0/0	0/0
Chromosome2-6482470	DEL	PRECISE	1821	0	0	0/0	0/0	0/1	0/1	0/1	0/1	0/0	0/0	0/0	0/1	0/1	1/1	0/1	0/1
Chromosome2-6489932	INS	PRECISE	4968	0	0	0/0	0/0	0/0	0/0	0/0	0/0	1/1	0/0	0/0	0/0	0/0	0/0	0/0	0/0
Chromosome2-6494875	DEL	PRECISE	6283	0	0	0/0	0/0	0/0	0/0	0/0	0/0	0/0	0/0	0/0	0/1	0/1	1/1	1/1	0/0
Chromosome2-6496479	DEL	PRECISE	2264	0	0	0/0	0/0	0/1	0/1	1/1	0/1	0/0	0/0	0/0	0/0	0/0	0/0	0/0	0/1
Chromosome2-6516433	DEL	PRECISE	406	0	0	0/0	0/0	0/0	0/0	0/1	0/1	1/1	1/1	0/1	0/0	0/0	0/0	0/0	0/0
Chromosome2-6518230	INS	PRECISE	325	0	0	0/0	0/0	0/0	0/0	0/0	0/0	0/0	0/0	0/0	0/0	0/0	0/0	0/0	0/1
Chromosome2-6525798	INS	PRECISE	100	0	0.5	0/0	0/0	0/0	0/0	0/0	0/0	0/0	0/0	0/0	0/1	0/1	1/1	0/1	0/0
Chromosome2-6527071	DEL	PRECISE	621	0	0	0/0	0/0	0/0	0/0	0/0	0/0	0/0	0/0	0/0	0/1	0/1	1/1	0/1	0/0
Chromosome2-6529282	DEL	PRECISE	8069	0	0	0/0	0/0	0/0	0/0	0/1	0/1	0/0	0/0	0/0	0/0	0/0	0/0	0/0	0/0
Chromosome2-6530106	BND	PRECISE	0	0	0	0/0	0/0	0/0	0/0	0/0	0/0	0/0	0/1	0/0	0/0	0/0	0/0	0/0	0/0
Chromosome2-6531824	INS	PRECISE	7057	0	0	0/0	0/0	0/0	0/0	0/0	0/0	1/1	0/0	0/0	0/0	0/0	0/0	0/0	0/0
Chromosome2-6532417	INS	PRECISE	5047	0	0.707	0/0	0/0	1/1	1/1	0/0	0/0	0/0	0/0	0/0	0/0	0/0	0/0	0/0	0/0
Chromosome2-6538677	DEL	PRECISE	58	0	0	0/0	0/0	0/0	0/0	0/0	0/0	0/0	0/0	0/0	0/1	0/1	1/1	0/1	0/0
Chromosome2-6538713	INS	PRECISE	4926	0	0	0/0	0/0	0/0	0/0	0/0	0/0	0/0	0/0	0/0	0/0	0/0	0/0	0/0	1/1
Chromosome2-6542870	INS	PRECISE	90	0	0.492	0/0	0/0	0/1	1/1	1/1	1/1	1/1	1/1	0/1	0/1	0/1	1/1	0/1	0/1
Chromosome2-6545595	INS	PRECISE	57	0	0	0/0	0/0	0/0	0/0	0/0	0/0	0/0	0/0	0/0	0/1	0/0	0/1	0/0	0/0
Chromosome2-6558532	DEL	PRECISE	1105	0	0	0/0	0/0	0/1	0/1	1/1	1/1	1/1	1/1	0/1	0/0	0/0	0/0	0/0	0/0
Chromosome2-6560386	DEL	PRECISE	454	0	0	0/0	0/0	0/0	0/0	0/0	0/0	0/0	0/0	0/0	0/1	0/0	0/0	0/0	0/0
Chromosome2-6565132	DEL	PRECISE	822	0	0	0/0	0/0	0/0	0/0	0/0	0/0	0/0	0/0	0/0	0/1	0/0	0/0	0/0	0/1
Chromosome2-6569420	DEL	PRECISE	891	0	0	0/0	0/0	0/0	0/0	0/0	0/0	0/0	0/0	0/0	0/1	0/1	1/1	0/1	0/0
Chromosome2-6573673	INS	PRECISE	1491	0	0	0/0	0/0	0/0	0/0	0/0	0/0	0/0	0/0	0/0	0/0	0/0	0/0	0/0	0/1
Chromosome2-6574499	INS	PRECISE	3311	0	0	0/0	0/0	0/0	0/0	0/0	0/0	0/0	0/0	0/0	0/0	0/0	0/0	0/0	0/0
Chromosome2-6591892	INS	PRECISE	125	0	0	0/0	0/0	0/1	0/1	0/1	0/1	0/0	0/0	0/0	0/0	0/0	0/0	0/0	0/0
Chromosome2-6599444	DEL	PRECISE	840	0	0	0/0	0/0	0/1	0/1	1/1	1/1	1/1	1/1	0/1	0/1	0/1	1/1	0/1	0/1
Chromosome2-6609734	INS	PRECISE	291	0	0.707	0/0	0/0	0/0	0/0	0/0	0/0	0/0	0/0	0/0	1/1	0/0	1/1	0/0	0/0
Chromosome2-6610127	DEL	PRECISE	888	0	0	0/0	0/0	0/0	0/0	0/0	0/0	0/0	0/0	0/0	0/1	0/1	1/1	0/1	0/0
Chromosome2-6614195	DEL	PRECISE	6047	0	0	0/0	0/0	0/0	0/0	0/0	0/0	0/0	0/0	0/0	0/0	0/0	0/0	0/0	0/1
Chromosome2-6616430	INV	PRECISE	430	0	0	0/0	0/0	0/0	0/0	0/0	0/0	0/1	0/0	0/0	0/0	0/0	0/0	0/0	0/0
Chromosome2-6616825	DEL	PRECISE	3903	0	0	0/0	0/0	0/1	0/1	0/1	0/1	0/0	0/0	0/0	0/1	0/1	1/1	0/1	0/0
Chromosome2-6635414	INS	PRECISE	64	0	0	0/0	0/0	0/0	0/0	0/0	0/0	0/0	0/0	0/0	0/0	0/0	0/0	0/0	0/1
Chromosome2-6636471	INS	PRECISE	1980	0	0	0/0	0/0	0/0	0/0	0/0	0/0	0/0	0/0	0/0	0/0	0/0	0/1	0/0	0/0
Chromosome2-6655197	DEL	PRECISE	5678	0	0	0/0	0/0	0/0	0/0	0/0	0/0	0/0	0/0	0/0	0/1	0/1	1/1	0/1	0/0
Chromosome2-6657450	DEL	PRECISE	911	0	0	0/0	0/0	0/1	0/1	0/1	0/1	0/0	0/0	0/0	0/0	0/0	0/0	0/0	0/1
Chromosome2-6670720	INS	PRECISE	4727	0	0	0/0	0/0	0/0	0/0	0/0	0/0	0/0	0/0	0/0	0/0	0/0	0/0	0/0	1/1
Chromosome2-6670725	BND	PRECISE	0	0	0	0/0	0/0	0/0	0/0	0/0	0/0	0/0	0/0	0/0	0/0	0/0	0/0	0/0	0/1
Chromosome2-6677136	DEL	PRECISE	76	0	0	0/0	0/0	0/1	0/1	0/1	0/1	0/0	0/0	0/0	0/0	0/0	0/0	0/0	0/0

Chromosome2-6677170	INS	PRECISE	122	0	0.5	0/0	0/0	0/0	0/0	0/0	0/0	0/0	0/0	0/0	0/1	0/1	1/1	0/1	0/0
Chromosome2-6704445	DEL	PRECISE	1062	0.707	0.707	0/0	0/0	0/0	0/0	0/0	0/0	0/0	0/0	0/0	0/1	0/0	0/1	0/0	0/0
Chromosome2-6706796	INS	PRECISE	646	0	0	0/0	0/0	0/0	0/0	0/0	0/0	0/1	0/0	0/0	0/0	0/0	0/0	0/0	0/0
Chromosome2-6707354	DEL	PRECISE	1352	0	0	0/0	0/0	0/0	0/0	0/0	0/0	0/0	0/0	0/0	0/1	0/0	1/1	0/0	0/0
Chromosome2-6707688	INS	PRECISE	80	0	0.577	0/0	0/0	0/0	0/0	0/0	0/0	0/0	0/0	0/0	0/0	0/1	1/1	0/1	0/0
Chromosome2-6718398	DEL	PRECISE	10524	0	0	0/0	0/0	0/0	0/0	0/0	0/0	0/0	0/0	0/0	0/0	0/1	1/1	1/1	0/0
Chromosome2-6726444	DEL	PRECISE	63	0	0	0/0	0/0	0/0	0/0	0/0	0/0	0/0	0/0	0/0	0/0	0/0	0/1	0/0	0/0
Chromosome2-6736361	BND	PRECISE	0	0	0	0/0	0/0	0/0	0/0	0/0	0/0	0/0	0/0	0/0	0/0	0/1	0/1	0/0	0/0
Chromosome2-6749618	INS	PRECISE	53	0	0	0/0	0/0	0/0	0/0	0/0	0/0	0/0	0/0	0/0	0/1	0/1	1/1	0/1	0/0
Chromosome2-6751434	INS	PRECISE	892	0	0.707	0/0	0/0	0/0	0/0	0/0	0/0	0/0	0/0	0/0	0/1	0/0	1/1	0/0	0/0
Chromosome2-6761783	INS	PRECISE	846	0	0	0/0	0/0	0/0	0/0	0/0	0/0	0/0	0/0	0/0	0/0	0/0	0/1	0/0	0/0
Chromosome2-6765396	DEL	PRECISE	250	0	0	0/0	0/0	0/0	0/0	0/0	0/0	0/0	0/0	0/0	0/1	0/1	1/1	0/1	0/0
Chromosome2-6768360	DEL	PRECISE	97	0	0	0/0	0/0	0/0	0/0	0/1	0/1	0/0	0/0	0/0	0/1	0/1	1/1	0/1	0/0
Chromosome2-6770366	DEL	PRECISE	692	0	0	0/0	0/0	0/0	0/0	0/0	0/0	0/0	0/0	0/0	0/1	0/1	1/1	0/1	0/0
Chromosome2-6777088	INS	PRECISE	366	0	0	0/0	0/0	0/0	0/0	0/0	0/0	0/0	0/0	0/0	0/0	0/0	0/0	0/0	0/1
Chromosome2-6780164	INS	PRECISE	1367	0	0.707	0/0	0/0	0/1	0/1	0/0	0/0	0/0	0/0	0/0	0/0	0/0	0/0	0/0	0/0
Chromosome2-6782176	INS	PRECISE	150	0	0	0/0	0/0	0/0	0/0	0/0	0/0	0/0	0/0	0/0	0/0	0/0	0/0	0/0	0/1
Chromosome2-6784557	DEL	PRECISE	555	0	0	0/0	0/0	0/0	0/0	0/0	0/0	0/0	0/0	0/0	0/1	0/0	1/1	0/1	0/0
Chromosome2-6784788	INS	PRECISE	82	0	0	0/0	0/0	0/0	0/0	0/0	0/0	0/1	0/0	0/0	0/0	0/0	0/0	0/0	0/0
Chromosome2-6805417	INS	PRECISE	126	0	0	0/0	0/0	0/0	0/0	0/0	0/0	0/0	0/0	0/0	0/1	0/1	1/1	0/1	0/0
Chromosome2-6805938	DEL	PRECISE	129	0	0	0/0	0/0	0/0	0/0	0/0	0/0	0/0	0/0	0/0	0/0	0/0	0/1	0/0	0/0
Chromosome2-6808409	DEL	PRECISE	976	0	0	0/0	0/0	0/0	1/1	0/1	0/1	0/1	0/0	0/0	0/0	0/0	0/0	0/0	0/0
Chromosome2-6811488	DEL	PRECISE	85	0	0	0/0	0/0	0/0	0/0	0/0	0/0	0/0	0/0	0/0	0/1	0/1	1/1	0/1	0/0
Chromosome2-6823494	INS	PRECISE	1194	0	0.816	0/0	0/0	0/0	0/0	0/0	0/0	0/0	0/0	0/0	0/0	0/1	1/1	0/1	0/0
Chromosome2-6830394	DEL	PRECISE	8127	0	0	0/0	0/0	0/1	0/1	0/1	0/1	0/0	0/0	0/0	0/1	0/1	1/1	0/1	0/0
Chromosome2-6852458	INS	PRECISE	2258	0	0.707	0/0	0/0	0/0	0/0	0/0	0/0	0/0	0/0	0/0	0/0	0/1	0/0	0/1	0/0
Chromosome2-6857656	INS	PRECISE	1874	0	0.707	0/0	0/0	0/1	0/1	0/0	0/0	0/0	0/0	0/0	0/0	0/0	0/0	0/0	0/0
Chromosome2-6859681	INS	PRECISE	12396	0	0	0/0	0/0	0/0	0/0	0/0	0/0	0/0	0/0	0/0	0/0	0/0	0/0	0/0	0/0
Chromosome2-6859681	BND	PRECISE	0	0	0	0/0	0/0	0/0	0/0	0/0	0/0	0/0	0/0	0/0	0/1	0/0	0/0	0/0	0/0
Chromosome2-6864462	INS	PRECISE	67	0	0.5	0/0	0/0	0/0	0/0	0/0	0/0	0/0	0/0	0/0	0/1	0/1	1/1	0/1	0/0
Chromosome2-6864546	DEL	PRECISE	71	0	0	0/0	0/0	0/0	0/0	0/0	0/0	0/0	0/0	0/0	0/1	0/1	1/1	0/1	0/0
Chromosome2-6872352	INS	PRECISE	654	0	0	0/0	0/0	0/0	0/1	0/0	0/0	0/0	0/0	0/0	0/0	0/0	0/0	0/0	0/0
Chromosome2-6872944	INS	PRECISE	111	0	0	0/0	0/0	0/0	0/0	0/0	0/0	0/0	0/0	0/0	0/1	0/1	1/1	0/1	0/0
Chromosome2-6882438	BND	PRECISE	0	0	0	0/0	0/0	0/0	0/0	0/0	0/0	0/0	0/0	0/0	0/1	0/0	1/1	0/0	0/0
Chromosome2-6883453	DEL	PRECISE	50	0	0	0/0	0/0	0/0	0/0	0/0	0/0	0/0	0/0	0/0	0/0	0/1	1/1	0/1	0/0
Chromosome2-6893628	INS	PRECISE	1392	0	0	0/0	0/0	0/0	0/0	0/0	0/0	0/0	0/0	0/0	0/0	0/0	0/0	0/0	0/1
Chromosome2-6901152	DEL	PRECISE	86	0	0	0/0	0/0	0/0	0/0	0/0	0/0	0/0	0/0	0/0	0/1	0/1	1/1	0/1	0/0
Chromosome2-6903012	DEL	PRECISE	695	0	0	0/0	0/0	0/1	0/1	0/0	0/0	0/0	0/0	0/0	0/0	0/0	0/0	0/0	0/0
Chromosome2-6911420	DEL	PRECISE	161	0	0	0/0	0/0	0/0	0/0	0/0	0/0	0/0	0/0	0/0	0/1	0/1	1/1	0/1	0/0
Chromosome2-6914375	INS	PRECISE	218	0	0	0/0	0/0	0/0	0/0	0/0	0/0	0/0	0/0	0/0	0/0	0/0	0/0	0/0	0/1
Chromosome2-6920753	INS	PRECISE	1158	0	0.707	0/0	0/0	0/1	0/1	0/0	0/0	0/0	0/0	0/0	0/0	0/0	0/0	0/0	0/0
Chromosome2-6931972	DEL	PRECISE	5198	0	0	0/0	0/0	0/0	0/0	0/0	0/0	0/0	0/0	0/0	1/1	1/1	0/0	0/1	0/0
Chromosome2-6940834	DEL	PRECISE	390	0	0	0/0	0/0	0/0	0/0	0/0	0/0	0/0	0/0	0/0	0/0	0/1	1/1	0/0	0/0
Chromosome2-6941523	INS	PRECISE	72	0	0	0/0	0/0	0/0	0/0	0/0	0/0	0/0	0/0	0/0	0/0	0/0	0/0	0/0	0/1
Chromosome2-6945133	DEL	PRECISE	81	0	0	0/0	0/0	0/0	0/0	0/0	0/0	0/1	0/0	0/0	0/0	0/0	0/0	0/0	0/0
Chromosome2-6958136	INS	PRECISE	204	0	0.707	0/0	0/0	0/0	0/0	0/0	0/0	0/0	0/0	0/0	0/0	0/1	0/0	0/1	0/0
Chromosome2-6963442	BND	PRECISE	0	0	0	0/0	0/0	0/0	0/1	0/1	0/1	0/0	0/0	0/0	0/0	0/0	0/1	0/0	0/0
Chromosome2-6963443	INV	PRECISE	27047333	0	0	0/0	0/0	0/0	0/0	0/0	0/0	0/0	0/0	0/0	0/0	0/0	0/0	0/1	0/0
Chromosome2-6974019	BND	PRECISE	0	0	0	0/0	0/1	0/1	0/1	0/1	0/1	0/1	0/0	0/0	0/0	0/0	0/0	0/0	0/0
Chromosome2-6974019	BND	PRECISE	0	0	0	0/0	0/0	0/0	0/0	0/0	0/0	0/0	0/0	0/0	0/0	0/0	0/0	0/0	0/1
Chromosome2-6974019	INV	PRECISE	27036593	0	0	0/0	0/0	0/0	0/0	0/0	0/0	0/0	0/0	0/0	0/0	0/0	0/0	0/1	0/0
Chromosome2-6983496	INS	PRECISE	1087	0	0.787	0/0	0/0	0/1	1/1	1/1	1/1	1/1	1/1	0/1	0/0	0/0	0/0	0/0	0/0
Chromosome2-6986141	DEL	PRECISE	321	0	0	0/0	0/0	0/0	0/1	0/1	0/1	1/1	1/1	0/1	0/0	0/0	0/0	0/0	0/0
Chromosome2-6987647	DEL	PRECISE	537	0	0	0/0	0/0	0/1	1/1	1/1	1/1	1/1	1/1	0/1	0/0	0/0	0/0	0/0	0/0
Chromosome2-6992994	DEL	PRECISE	6285	0	0	0/0	0/0	0/1	0/1	0/0	0/0	0/0	0/0	0/0	0/0	0/0	0/0	0/0	0/0
Chromosome2-7009650	DEL	PRECISE	109	0	0	0/0	0/0	0/0	0/0	0/1	0/1	0/1	1/1	0/1	0/0	0/0	0/0	0/0	0/0
Chromosome2-7012775	INS	PRECISE	1271	0	0.707	0/0	0/0	0/0	0/0	0/1	0/1	0/0	0/0	0/0	0/0	0/0	0/0	0/0	0/0
Chromosome2-7014831	DEL	PRECISE	155	0	0	0/0	0/0	0/0	1/1	0/1	0/1	1/1	1/1	0/1	0/0	0/0	0/0	0/0	0/0
Chromosome2-7015861	BND	PRECISE	0	0	0	0/0	0/0	0/0	0/1	0/0	0/0	0/0	0/0	0/0	0/0	0/0	0/0	0/0	0/0
Chromosome2-7016474	DEL	PRECISE	13991	0	0	0/0	0/0	0/0	1/1	0/0	0/0	0/0	0/0	0/0	0/0	0/0	0/0	0/0	0/0
Chromosome2-7016475	DEL	PRECISE	17511	0	0	0/0	0/0	0/0	0/1	0/0	0/0	0/0	0/0	0/0	0/0	0/0	0/0	0/0	0/0
Chromosome2-7016868	DEL	PRECISE	554	0	0	0/0	0/0	0/0	0/0	0/0	0/0	0/0	0/0	0/0	0/1	0/1	1/1	0/1	0/0
Chromosome2-7021012	DEL	PRECISE	102	0	0	0/0	0/0	0/0	0/0	0/0	0/0	0/0	0/0	0/0	0/1	0/0	1/1	0/0	0/0
Chromosome2-7029157	INS	PRECISE	1284	0	0.894	0/0	0/0	0/0	0/0	1/1	0/1	1/1	1/1	0/1	0/0	0/0	0/0	0/0	0/0
Chromosome2-7029851	DEL	PRECISE	255	0	0	0/0	0/0	0/0	0/0	0/0	0/0	0/0	0/0	0/0	0/0	0/1	0/0	0/1	0/0
Chromosome2-7030483	DEL	PRECISE	3503	0	0	0/0	0/0	0/0	1/1	0/0	0/0	0/0	0/0	0/0	0/0	0/0	0/0	0/0	0/0
Chromosome2-7031503	DEL	PRECISE	3552	0	0	0/0	0/0	0/0	0/0	0/0	0/0	0/0	0/0	0/0	0/0	0/0	0/1	0/0	0/0
Chromosome2-7031600	INS	PRECISE	2110	0	0.707	0/0	0/0	0/0	0/0	0/1	0/1	0/0	0/0	0/0	0/0	0/0	0/0	0/0	0/0
Chromosome2-7033516	INS	PRECISE	68	0	0	0/0	0/0	0/0	0/0	0/0	0/0	0/0	0/0	0/0	0/1	0/1	1/1	0/1	0/0
Chromosome2-7035914	DEL	PRECISE	2700	0	0	0/0	0/0	0/0	0/0	0/1	0/1	0/1	1/1	0/1	0/0	0/0	0/0	0/0	0/0
Chromosome2-7036772	BND	PRECISE	0	0	0	0/0	0/0	0/0	0/1	0/0	0/0	0/0	0/0	0/0	0/0	0/0	0/0	0/0	0/0
Chromosome2-7050368	INS	PRECISE	149	0	0	0/0	0/0	0/0	0/0	0/0	0/0	0/0	0/0	0/0	0/1	0/1	1/1	0/1	0/0
Chromosome2-7057820	DEL	PRECISE	652	0	0	0/0	0/0	0/1	0/1	0/1	0/1	0/1	0/0	0/0	0/0	0/0	0/0	0/0	0/0

Chromosome2-7058321	DEL	PRECISE	63	0	0	0/0	0/0	0/0	0/0	0/0	0/0	0/0	0/0	0/0	0/1	0/1	1/1	0/1	0/0
Chromosome2-7061465	DEL	PRECISE	5483	0	0	0/0	0/0	0/0	0/1	0/0	0/0	0/1	0/0	0/0	0/1	0/1	1/1	0/1	0/0
Chromosome2-7067748	DEL	PRECISE	405	0.949	0	0/0	0/0	0/0	0/1	0/0	0/0	0/1	0/0	0/0	0/1	0/1	1/1	0/1	0/0
Chromosome2-7069361	DEL	PRECISE	1020	0	0	0/0	0/0	0/0	1/1	1/1	1/1	1/1	1/1	0/1	0/1	0/1	1/1	0/1	0/0
Chromosome2-7094119	DEL	PRECISE	156	0	0	0/0	0/0	0/0	0/0	0/0	0/0	0/0	0/0	0/0	0/1	0/1	1/1	0/1	0/0
Chromosome2-7097067	INS	PRECISE	252	0	0.577	0/0	0/0	0/0	0/1	0/1	0/1	0/0	0/0	0/0	0/0	0/0	0/0	0/0	0/0
Chromosome2-7097994	DEL	PRECISE	445	0	0	0/0	0/0	0/0	0/0	0/0	0/0	0/1	0/0	0/0	0/1	0/1	1/1	0/1	0/1
Chromosome2-7101122	DEL	PRECISE	504	0	0	0/0	0/0	0/0	0/1	0/0	0/0	0/0	0/0	0/0	0/0	0/0	0/0	0/0	0/0
Chromosome2-7103043	DEL	PRECISE	126	0	0	0/0	0/0	0/0	0/0	0/1	0/1	0/0	0/0	0/0	0/0	0/0	0/0	0/0	0/0
Chromosome2-7106014	DEL	PRECISE	1744	0	0	0/0	0/0	0/1	1/1	1/1	1/1	1/1	1/1	0/1	0/1	0/0	1/1	0/0	0/1
Chromosome2-7109662	DEL	PRECISE	62	0	0	0/0	0/0	0/1	0/1	0/0	0/0	0/0	0/0	0/0	0/0	0/0	0/0	0/0	0/0
Chromosome2-7117193	DEL	PRECISE	841	0	0	0/0	0/0	0/1	1/1	1/1	1/1	0/1	1/1	0/1	0/1	0/1	1/1	0/1	0/1
Chromosome2-7123793	INS	PRECISE	89	0	0.518	0/0	0/0	0/1	1/1	1/1	1/1	1/1	1/1	1/1	0/0	0/0	0/0	0/0	0/1
Chromosome2-7124757	DEL	PRECISE	150	0	0	0/0	0/0	0/0	0/0	0/0	0/0	0/0	0/0	0/0	0/0	0/0	0/0	0/1	0/0
Chromosome2-7134227	DEL	PRECISE	958	0	0	0/0	0/0	0/0	0/0	0/0	0/0	0/0	0/0	0/0	0/1	0/1	1/1	1/1	0/0
Chromosome2-7149247	DEL	PRECISE	217	0	0	0/0	0/0	0/0	0/0	0/0	0/0	0/0	0/0	0/0	0/1	0/1	1/1	0/1	0/0
Chromosome2-7157206	INS	PRECISE	97	0	0.707	0/0	0/0	0/0	0/0	0/1	0/1	0/0	0/0	0/0	0/0	0/0	0/0	0/0	0/0
Chromosome2-7159247	INS	PRECISE	251	0	0.577	0/0	0/0	0/0	0/0	0/0	0/0	0/0	0/0	0/0	0/0	0/0	1/1	0/1	0/0
Chromosome2-7160181	DEL	PRECISE	1313	0	0	0/0	0/0	0/0	0/0	0/0	0/0	0/0	0/0	0/0	1/1	1/1	1/1	1/1	0/0
Chromosome2-7164958	INS	PRECISE	755	0	0.816	0/0	0/0	0/0	0/0	0/0	0/0	0/0	0/0	0/0	1/1	1/1	0/0	1/1	0/0
Chromosome2-7166170	DEL	PRECISE	12511	0.492	0	0/0	0/0	0/1	0/1	1/1	1/1	1/1	1/1	0/1	0/1	0/1	1/1	0/1	0/1
Chromosome2-7189827	INS	PRECISE	245	0	0	0/0	0/0	0/0	0/0	0/0	0/0	0/0	0/0	0/0	0/0	0/0	0/1	0/0	0/0
Chromosome2-7193732	INS	PRECISE	995	0	0	0/0	0/0	0/0	0/0	0/0	0/0	1/1	0/0	0/0	0/0	0/0	0/0	0/0	0/0
Chromosome2-7196862	INS	PRECISE	134	0	0.707	0/0	0/0	0/0	0/0	0/0	0/0	0/0	0/0	0/0	0/0	0/1	0/0	0/1	0/0
Chromosome2-7197797	INS	PRECISE	516	0	0.707	0/0	0/0	0/1	0/1	0/0	0/0	0/0	0/0	0/0	0/0	0/0	0/0	0/0	0/0
Chromosome2-7228095	DEL	PRECISE	190	0	0	0/0	0/0	0/0	0/0	0/0	0/0	0/0	0/0	0/0	0/1	0/0	1/1	0/0	0/0
Chromosome2-7239528	DEL	PRECISE	696	0	0	0/0	0/0	0/1	0/1	0/0	0/0	0/0	0/0	0/0	0/0	0/0	0/0	0/0	0/0
Chromosome2-7246866	DEL	PRECISE	355	0	0	0/0	0/0	0/1	0/1	0/1	1/1	0/0	0/0	0/0	0/0	0/0	0/0	0/0	0/0
Chromosome2-7254861	DEL	PRECISE	373	0	0	0/0	0/0	0/0	0/0	0/0	0/0	0/0	0/0	0/0	1/1	0/0	1/1	0/0	0/0
Chromosome2-7255807	INS	PRECISE	66	0	0	0/0	0/0	0/0	0/0	0/0	0/0	0/0	0/0	0/0	0/0	0/0	0/0	0/0	0/1
Chromosome2-7259661	INS	PRECISE	9001	0	0	0/0	0/0	0/0	0/0	0/0	0/0	0/0	0/0	0/0	0/0	0/0	0/0	0/0	1/1
Chromosome2-7264084	DEL	PRECISE	543	0	0	0/0	0/0	0/0	0/0	0/0	0/0	0/0	0/0	0/0	0/0	0/1	1/1	0/1	0/0
Chromosome2-7268170	DUP	PRECISE	72	0	0	0/0	0/0	0/0	0/0	0/0	0/0	0/0	0/0	0/0	0/0	0/1	0/0	0/1	0/0
Chromosome2-7269006	INS	PRECISE	843	0	0	0/0	0/0	0/0	0/0	0/0	0/0	0/1	0/0	0/0	0/0	0/0	0/0	0/0	0/0
Chromosome2-7269727	INS	PRECISE	282	0	0.816	0/0	0/0	0/0	0/0	0/0	0/0	0/0	0/0	0/0	0/0	0/1	1/1	0/1	0/0
Chromosome2-7273378	INS	PRECISE	697	0	0.707	0/0	0/0	0/0	0/0	0/1	0/1	0/0	0/0	0/0	0/0	0/0	0/0	0/0	0/0
Chromosome2-7278459	INS	PRECISE	102	0	0.389	0/0	0/0	0/1	1/1	1/1	1/1	1/1	1/1	0/1	0/1	0/1	1/1	0/1	0/1
Chromosome2-7286459	DEL	PRECISE	313	0	0	0/0	0/0	0/1	1/1	1/1	1/1	0/0	0/0	0/0	0/1	0/1	1/1	0/1	0/1
Chromosome2-7289359	BND	PRECISE	0	0.707	0	0/0	0/0	0/0	0/1	0/0	0/0	0/0	0/0	0/0	0/0	0/0	0/0	0/0	0/0
Chromosome2-7296865	INS	PRECISE	1365	0	0	0/0	0/0	0/0	0/1	0/0	0/0	0/0	0/0	0/0	0/0	0/0	0/0	0/0	0/0
Chromosome2-7302703	INS	PRECISE	5353	0	0	0/0	0/0	0/0	0/1	0/0	0/0	0/0	0/0	0/0	0/0	0/0	0/0	0/0	0/0
Chromosome2-7312920	DEL	PRECISE	444	0	0	0/0	0/0	0/0	0/0	0/0	0/0	0/0	0/0	0/0	0/1	0/1	1/1	0/1	0/0
Chromosome2-7314861	INS	PRECISE	2757	0	0	0/0	0/0	0/0	0/0	0/0	0/0	0/0	0/0	0/0	0/0	0/0	0/0	0/0	1/1
Chromosome2-7333537	INS	PRECISE	204	0	0	0/0	0/0	0/0	0/0	0/0	0/0	0/0	0/0	0/0	0/1	0/1	0/1	0/1	0/0
Chromosome2-7335722	INS	PRECISE	137	0	0	0/0	0/0	0/0	0/0	0/0	0/0	0/0	0/0	0/0	0/0	0/0	0/1	0/0	0/0
Chromosome2-7340792	INS	PRECISE	66	0	0	0/0	0/0	0/0	0/0	0/0	0/1	0/0	0/0	0/0	0/0	0/0	0/0	0/0	0/0
Chromosome2-7341942	INS	PRECISE	144	0	0	0/0	0/0	0/0	0/0	0/0	0/0	0/0	0/0	0/0	0/1	0/1	1/1	0/1	0/0
Chromosome2-7352809	DEL	PRECISE	10173	0	0	0/0	0/0	0/0	0/0	1/1	1/1	1/1	1/1	0/1	0/1	0/1	1/1	0/1	0/0
Chromosome2-7370557	INS	PRECISE	61	0	0	0/0	0/0	0/0	0/0	0/0	0/0	0/0	0/0	0/0	0/0	0/0	0/0	0/0	0/1
Chromosome2-7371396	BND	PRECISE	0	0	0	0/0	0/0	0/0	0/0	0/0	0/1	0/0	0/1	0/1	0/0	0/0	0/0	0/0	0/0
Chromosome2-7386665	BND	PRECISE	0	0	0	0/0	0/0	0/0	0/0	0/1	0/0	0/0	0/0	0/0	0/0	0/0	0/0	0/0	0/0
Chromosome2-7392787	INS	PRECISE	137	0	0	0/0	0/0	0/0	0/0	0/1	0/1	0/0	0/0	0/0	0/0	0/0	0/0	0/0	0/0
Chromosome2-7400375	DEL	PRECISE	80	0	0	0/0	0/0	0/0	0/0	0/0	0/0	0/0	0/0	0/0	0/0	0/0	0/0	0/0	0/1
Chromosome2-7407731	DEL	PRECISE	159	0	0	0/0	0/0	0/1	0/1	0/0	0/0	0/0	0/0	0/0	0/0	0/0	0/0	0/0	0/0
Chromosome2-7410620	DEL	PRECISE	21818	0	0	0/0	0/0	0/0	0/0	0/0	0/0	0/0	0/0	0/0	1/1	1/1	0/0	1/1	0/0
Chromosome2-7410887	DEL	PRECISE	5816	0	0	0/0	0/0	0/0	0/0	0/0	0/0	0/0	0/0	0/0	0/0	0/0	0/0	0/0	0/1
Chromosome2-7417959	DEL	PRECISE	608	0	0	0/0	0/0	0/0	0/0	0/0	0/0	0/0	0/0	0/0	0/0	0/0	0/0	0/0	0/1
Chromosome2-7424617	DEL	PRECISE	6474	0	0	0/0	0/0	0/0	0/0	0/0	0/0	0/0	0/0	0/0	0/0	0/0	0/0	0/0	0/1
Chromosome2-7427661	INS	PRECISE	425	0	0	0/0	0/0	0/0	0/0	0/0	0/0	0/1	0/0	0/0	0/0	0/0	0/0	0/0	0/0
Chromosome2-7434301	DEL	PRECISE	52	0	0	0/0	0/0	0/0	0/0	0/0	0/0	0/0	0/0	0/0	0/0	0/0	1/1	0/0	0/0
Chromosome2-7441840	INS	PRECISE	5572	0	0	0/0	0/0	0/0	0/0	0/0	0/0	0/0	0/0	0/0	0/0	0/0	1/1	0/0	0/0
Chromosome2-7442449	INS	PRECISE	6250	0	0	0/0	0/0	0/0	0/0	0/0	0/0	0/1	0/0	0/0	0/0	0/0	0/0	0/0	0/0
Chromosome2-7442457	BND	PRECISE	0	0	0	0/0	0/0	0/0	0/0	0/0	0/0	0/1	0/0	0/0	0/0	0/0	0/0	0/0	0/0
Chromosome2-7442838	DEL	PRECISE	656	0	0	0/0	0/0	0/0	0/0	0/0	0/0	0/0	0/0	0/0	0/1	0/0	1/1	0/1	0/0
Chromosome2-7457456	DEL	PRECISE	702	0	0	0/0	0/0	0/1	1/1	1/1	1/1	1/1	1/1	0/1	0/1	0/1	1/1	0/0	0/1
Chromosome2-7461236	INS	PRECISE	724	0	0.577	0/0	0/0	0/0	0/0	0/0	0/0	0/0	0/0	0/0	0/1	0/1	1/1	0/0	0/0
Chromosome2-7461287	DEL	PRECISE	66	0	0	0/0	0/0	0/0	0/0	0/1	0/1	0/0	0/0	0/0	0/0	0/0	0/0	0/0	0/0
Chromosome2-7474076	INS	PRECISE	76	0	0	0/0	0/0	0/0	0/0	0/0	0/0	0/0	0/0	0/0	0/0	0/0	0/0	0/0	0/1
Chromosome2-7477134	INS	PRECISE	445	0	0	0/0	0/0	0/0	0/0	0/0	0/0	0/0	0/0	0/0	0/0	0/0	0/0	0/0	0/1
Chromosome2-7480379	INS	PRECISE	348	0	0.707	0/0	0/0	0/0	0/0	0/1	0/1	0/0	0/0	0/0	0/0	0/0	0/0	0/0	0/0
Chromosome2-7481217	INS	PRECISE	1024	0	0.5	0/0	0/0	0/0	0/0	0/0	0/0	0/0	0/0	0/0	0/1	0/1	1/1	0/1	0/0
Chromosome2-7483464	INS	PRECISE	567	0.5	0.577	0/0	0/0	0/0	0/0	0/0	0/0	0/0	0/0	0/0	0/1	0/1	1/1	0/1	0/0
Chromosome2-7497938	DEL	PRECISE	625	0	0	0/0	0/0	0/0	0/0	0/0	0/0	0/0	0/0	0/0	0/1	0/1	1/1	0/1	0/0

Chromosome2-7503357	INS	PRECISE	731	0	0.577	0/0	0/0	0/0	0/0	0/0	0/0	0/0	0/0	0/0	0/0	0/1	0/1	1/1	0/0
Chromosome2-7504477	DEL	PRECISE	81	0	0	0/0	0/0	0/0	0/0	0/0	0/0	0/1	0/0	0/0	0/0	0/0	0/0	0/0	0/0
Chromosome2-7515712	INS	PRECISE	829	0	0	0/0	0/0	0/1	0/0	0/0	0/0	0/0	0/0	0/0	0/0	0/0	0/0	0/0	0/0
Chromosome2-7526790	DEL	PRECISE	1007	0	0	0/0	0/0	0/0	0/0	0/0	0/0	0/0	0/0	0/0	0/0	0/0	1/1	0/0	0/0
Chromosome2-7539604	DEL	PRECISE	4565	0	0	0/0	0/0	0/0	0/0	0/0	0/0	0/0	0/0	0/0	0/0	0/1	0/1	0/0	0/0
Chromosome2-7540762	DEL	PRECISE	699	0	0	0/0	0/0	0/0	0/0	0/0	0/0	0/0	0/0	0/0	0/0	0/0	0/1	0/0	0/0
Chromosome2-7544193	DEL	PRECISE	1744	0	0	0/0	0/0	0/0	0/0	0/0	0/0	0/0	0/0	0/0	0/0	0/1	0/1	0/0	0/0
Chromosome2-7544328	DEL	PRECISE	7100	0	0	0/0	0/0	0/1	0/1	0/0	0/0	0/0	0/0	0/0	0/0	0/0	0/0	0/0	0/0
Chromosome2-7546543	DEL	PRECISE	477	0	0	0/1	0/1	0/0	0/0	0/1	0/1	1/1	1/1	1/1	0/0	0/1	0/0	0/1	0/1
Chromosome2-7547342	DEL	PRECISE	180	0	0	0/0	0/0	0/0	0/0	0/0	0/0	0/0	0/0	0/0	0/0	0/0	0/1	0/0	0/0
Chromosome2-7550783	DEL	PRECISE	334	0	0	0/0	0/0	0/0	0/0	0/0	0/0	0/0	0/0	0/0	0/0	0/0	0/0	0/0	0/1
Chromosome2-7552412	INS	PRECISE	66	0	0	0/0	0/0	0/0	0/0	0/1	0/1	0/0	0/0	0/0	0/0	0/0	0/0	0/0	0/0
Chromosome2-7556617	INS	PRECISE	1329	0	0	0/0	0/0	0/0	0/0	0/0	0/0	0/0	0/0	0/0	0/0	0/0	0/1	0/0	0/0
Chromosome2-7571650	DEL	PRECISE	445	0	0	0/0	0/0	0/1	0/1	0/1	0/1	0/0	0/0	0/0	0/1	0/1	1/1	0/1	0/1
Chromosome2-7573622	INS	PRECISE	813	0	0	0/0	0/0	0/0	0/0	0/0	0/0	0/0	0/0	0/0	0/0	0/0	0/0	0/0	0/1
Chromosome2-7576638	INS	PRECISE	127	0	0.707	0/0	0/0	0/0	0/0	0/0	0/0	0/0	0/0	0/0	0/1	0/0	1/1	0/0	0/0
Chromosome2-7576935	DEL	PRECISE	725	0	0	0/0	0/0	0/0	0/0	0/0	0/0	0/0	0/0	0/0	0/1	0/0	1/1	0/0	0/0
Chromosome2-7579280	INS	PRECISE	5564	0	0	0/0	0/0	0/0	0/0	0/0	0/0	0/0	0/0	0/0	0/0	0/0	0/0	0/0	1/1
Chromosome2-7597213	DEL	PRECISE	132	0	0	0/1	0/1	0/1	1/1	1/1	1/1	1/1	1/1	1/1	0/1	1/1	1/1	1/1	1/1
Chromosome2-7600593	INS	PRECISE	1115	0	0	0/0	0/0	0/0	0/0	0/0	0/0	0/0	0/0	0/0	0/0	0/0	1/1	0/0	0/0
Chromosome2-7601436	INS	PRECISE	252	0	0	0/0	0/0	0/0	0/0	0/0	0/0	0/0	0/0	0/0	0/0	0/0	0/0	0/0	0/1
Chromosome2-7604408	DEL	PRECISE	18727	0	0	0/0	0/0	0/0	0/0	0/1	0/1	0/0	0/0	0/0	0/0	0/0	0/0	0/0	0/0
Chromosome2-7614160	INS	PRECISE	957	0	0	0/0	0/0	0/1	0/1	0/0	0/0	0/0	0/0	0/0	0/0	0/0	0/0	0/0	0/0
Chromosome2-7621047	DEL	PRECISE	211	0	0	0/0	0/0	0/1	0/1	0/0	0/0	0/0	0/0	0/0	0/1	0/1	1/1	0/1	0/1
Chromosome2-7628283	BND	PRECISE	0	0	0	0/0	0/0	0/0	0/0	0/0	0/0	0/0	0/0	0/0	0/0	0/0	0/0	0/0	0/1
Chromosome2-7630573	DEL	PRECISE	423	0	0	0/0	0/0	0/0	0/0	0/0	0/0	0/1	0/0	0/0	0/0	0/0	0/0	0/0	0/0
Chromosome2-7632606	INS	PRECISE	5265	0	0	0/0	0/0	0/0	0/0	0/0	0/0	0/0	0/0	0/0	0/0	0/0	0/0	0/0	1/1
Chromosome2-7637423	INS	PRECISE	1349	0	0	0/0	0/0	0/0	0/0	0/0	0/0	0/0	0/0	0/0	0/0	0/0	0/0	0/0	0/1
Chromosome2-7639678	INS	PRECISE	12362	0	0	0/0	0/0	0/0	0/0	0/0	0/0	0/1	0/0	0/0	0/0	0/0	0/0	0/0	0/0
Chromosome2-7641798	DEL	PRECISE	1341	0	0	0/0	0/0	0/0	0/0	0/0	0/0	0/1	0/0	0/0	0/1	0/1	1/1	0/1	0/1
Chromosome2-7643530	INS	PRECISE	5791	0	0	0/0	0/0	0/0	0/0	0/0	0/0	0/0	0/0	0/0	0/0	0/0	0/0	0/0	1/1
Chromosome2-7653414	DEL	PRECISE	57	0	0	0/0	0/0	0/0	0/0	0/0	0/0	0/0	0/0	0/0	0/1	0/1	1/1	0/1	0/0
Chromosome2-7653740	INS	PRECISE	261	0	0.5	0/0	0/0	0/0	0/0	0/0	0/0	0/0	0/0	0/0	0/1	0/1	1/1	0/1	0/0
Chromosome2-7664973	BND	PRECISE	0	0	0	0/0	0/0	0/0	0/0	0/0	0/0	0/0	0/0	0/0	0/1	0/0	0/0	0/0	0/0
Chromosome2-7668086	INS	PRECISE	102	0	0.577	0/0	0/0	0/0	0/0	0/0	0/0	0/0	0/0	0/0	0/1	0/1	1/1	0/1	0/0
Chromosome2-7668584	INS	PRECISE	11783	0	0	0/0	0/0	0/0	0/0	0/0	0/0	0/1	0/0	0/0	0/0	0/0	0/0	0/0	0/0
Chromosome2-7672474	INS	PRECISE	1335	0	0.577	0/0	0/0	0/0	0/0	0/0	0/0	0/0	0/0	0/0	0/1	0/1	0/1	0/0	0/0
Chromosome2-7685086	DEL	PRECISE	278	0	0	0/0	0/0	0/0	0/1	0/0	0/0	0/1	0/0	0/0	0/1	0/1	1/1	0/1	0/0
Chromosome2-7691664	DEL	PRECISE	147	0	0	0/1	0/1	0/0	0/0	0/0	0/0	0/0	0/0	0/1	0/1	1/1	1/1	1/1	0/1
Chromosome2-7693215	INS	PRECISE	1263	0	0.707	0/0	0/0	0/1	0/1	0/0	0/0	0/0	0/0	0/0	0/0	0/0	0/0	0/0	0/0
Chromosome2-7698677	INS	PRECISE	6028	0	0	0/0	0/0	0/0	1/1	0/0	0/0	0/0	0/0	0/0	0/0	0/0	0/0	0/0	0/0
Chromosome2-7698693	BND	PRECISE	0	0	0	0/0	0/0	0/0	0/1	0/0	0/0	0/0	0/0	0/0	0/0	0/0	0/0	0/0	0/0
Chromosome2-7700018	INS	PRECISE	1365	0	0.816	0/1	0/1	0/0	0/0	0/0	0/0	0/0	0/0	0/1	0/0	0/1	0/0	0/1	0/1
Chromosome2-7711619	BND	PRECISE	0	0	0	0/0	0/0	0/0	0/0	0/0	0/0	0/0	0/0	0/0	0/0	0/1	0/1	0/1	0/0
Chromosome2-7719931	DEL	PRECISE	173	0	0	0/0	0/0	0/0	0/1	0/0	0/0	0/0	0/0	0/0	0/0	0/0	0/0	0/0	0/0
Chromosome2-7723167	DEL	PRECISE	167	0	0	0/0	0/0	0/0	0/1	0/0	0/0	0/1	0/0	0/0	0/0	0/0	0/0	0/0	0/0
Chromosome2-7725781	DEL	PRECISE	743	0	0	0/1	0/0	0/1	1/1	1/1	1/1	1/1	1/1	1/1	0/1	1/1	1/1	1/1	0/1
Chromosome2-7730720	DEL	PRECISE	58	0	0	0/1	0/1	0/0	0/0	0/0	0/0	0/0	0/0	0/1	0/1	1/1	0/1	1/1	0/1
Chromosome2-7732306	DEL	PRECISE	68	0	0	0/0	0/0	0/0	0/0	0/0	0/0	0/0	0/0	0/0	0/0	0/0	0/0	0/0	0/1
Chromosome2-7735273	INS	PRECISE	53	0	0	0/0	0/0	0/0	0/0	0/0	0/0	0/0	0/0	0/0	0/0	0/0	0/1	0/0	0/0
Chromosome2-7740764	DEL	PRECISE	286	0	0	0/1	0/1	0/0	0/0	0/0	0/0	0/0	0/0	0/1	0/1	1/1	1/1	1/1	0/1
Chromosome2-7747140	DEL	PRECISE	87	0	0	0/0	0/0	0/0	0/0	0/0	0/0	0/0	0/0	0/0	0/1	1/1	0/1	1/1	0/0
Chromosome2-7764386	INS	PRECISE	741	0	0.707	0/0	0/0	0/1	0/1	0/0	0/0	0/0	0/0	0/0	0/0	0/0	0/0	0/0	0/0
Chromosome2-7764859	DEL	PRECISE	58	0	0	0/1	0/1	0/0	0/0	0/1	0/1	0/0	0/0	0/1	0/0	0/1	0/1	0/1	1/1
Chromosome2-7776835	DEL	PRECISE	372	0	0	0/0	0/0	0/0	0/0	0/1	0/1	0/0	0/0	0/0	0/0	0/0	0/0	0/0	0/0
Chromosome2-7779290	INS	PRECISE	93	0	0	0/1	0/1	0/0	0/0	0/0	0/0	0/0	0/0	0/1	0/0	1/1	0/1	1/1	0/1
Chromosome2-7786436	DEL	PRECISE	1323	0.378	0.378	0/0	0/0	0/0	0/0	0/0	0/0	0/0	0/0	0/0	0/1	1/1	1/1	1/1	0/1
Chromosome2-7791093	DEL	PRECISE	522	0	0	0/0	0/0	0/0	0/0	0/0	0/0	0/0	0/0	0/0	0/1	0/0	1/1	0/0	0/0
Chromosome2-7791892	DEL	PRECISE	1222	0	0	0/0	0/0	0/0	0/0	0/0	0/0	0/0	0/0	0/0	0/1	0/0	1/1	0/0	0/0
Chromosome2-7794595	DEL	PRECISE	196	0	0	0/0	0/0	0/0	0/0	0/0	0/0	0/0	0/0	0/0	0/1	0/0	1/1	0/0	0/0
Chromosome2-7796206	INS	PRECISE	57	0	0.354	0/1	0/1	0/0	0/0	0/0	0/0	0/0	0/0	0/0	0/1	1/1	1/1	1/1	0/0
Chromosome2-7809207	DEL	PRECISE	97	0	0	0/0	0/0	0/0	0/0	0/1	0/1	1/1	1/1	0/1	0/0	0/0	0/0	0/0	0/0
Chromosome2-7811033	DEL	PRECISE	6071	0	0	0/0	0/0	0/0	0/0	0/0	0/0	0/0	0/0	0/0	0/0	0/0	0/0	0/0	0/1
Chromosome2-7815150	DEL	PRECISE	1339	0	0	0/1	0/1	0/0	0/0	0/1	0/1	0/0	0/0	0/1	0/0	1/1	1/1	1/1	0/1
Chromosome2-7819802	INS	PRECISE	52	0	0	0/0	0/0	0/0	0/0	0/0	0/0	0/0	0/0	0/0	0/0	0/1	0/0	0/1	0/0
Chromosome2-7823976	INS	PRECISE	82	0	0	0/1	0/1	0/0	0/0	0/0	0/0	0/0	0/0	0/1	0/1	0/1	1/1	0/1	0/1
Chromosome2-7829280	INS	PRECISE	113	0	0	0/0	0/0	0/0	0/0	0/0	0/0	0/0	0/0	0/0	0/0	0/1	0/0	0/1	0/0
Chromosome2-7831820	INS	PRECISE	5377	0	0	0/0	0/0	0/0	0/0	0/0	0/0	0/0	0/0	0/0	0/0	0/1	0/0	0/1	0/0
Chromosome2-7831826	BND	PRECISE	0	0	0	0/0	0/0	0/0	0/0	0/0	0/0	0/0	0/0	0/0	0/0	0/0	0/0	0/1	0/0
Chromosome2-7832009	DEL	PRECISE	52	0	0	0/0	0/0	0/0	0/0	0/0	0/0	0/0	0/0	0/0	0/0	0/1	0/0	0/1	0/0
Chromosome2-7833087	DEL	PRECISE	196	0	0	0/0	0/0	0/1	0/0	0/0	0/0	0/0	0/0	0/0	0/0	0/0	0/0	0/0	0/1
Chromosome2-7834549	INS	PRECISE	1318	0	0.707	0/0	0/0	0/0	0/0	0/0	0/0	0/0	0/0	0/0	0/0	0/0	1/1	0/0	0/0
Chromosome2-7836576	DEL	PRECISE	667	0	0	0/0	0/0	0/0	0/0	0/0	0/0	0/0	0/0	0/0	0/0	0/0	1/1	0/0	0/0

Chromosome2-7838768 BND	PRECISE	0	0	0	0/0	0/0	0/0	0/0	0/0	0/0	0/0	0/0	0/0	0/0	0/0	0/1	0/0	0/0
Chromosome2-7854980 INS	PRECISE	1868	0	0	0/0	0/0	0/0	0/1	0/0	0/0	0/0	0/0	0/0	0/0	0/0	0/0	0/0	0/0
Chromosome2-7875949 DEL	PRECISE	3709	0.289	0.289	0/1	0/1	0/1	0/1	0/1	0/1	0/0	0/0	0/1	0/1	1/1	1/1	1/1	1/1
Chromosome2-7891526 DEL	PRECISE	541	0	0	0/1	0/1	0/1	0/1	0/1	0/1	0/0	0/0	0/1	0/1	1/1	1/1	1/1	1/1
Chromosome2-7893767 INS	PRECISE	12818	0	0	0/0	0/0	0/0	0/0	0/1	0/0	0/0	0/0	0/0	0/0	0/0	0/0	0/0	0/0
Chromosome2-7893773 BND	PRECISE	0	0	0	0/0	0/0	0/0	0/0	0/1	0/1	0/0	0/0	0/0	0/0	0/0	0/0	0/0	0/0
Chromosome2-7893773 BND	PRECISE	0	0	0	0/0	0/0	0/0	0/0	0/0	0/1	0/0	0/0	0/0	0/0	0/0	0/0	0/0	0/0
Chromosome2-7897262 DEL	PRECISE	58	0	0	0/0	0/0	0/0	0/0	0/1	0/1	0/0	0/0	0/0	0/0	0/0	0/0	0/0	0/0
Chromosome2-7897343 INS	PRECISE	150	0	0.756	0/1	0/1	0/0	0/0	0/0	0/0	0/0	0/0	0/1	0/1	0/1	0/1	0/1	0/1
Chromosome2-7897385 INS	PRECISE	5531	0	0	0/0	0/0	0/0	0/0	0/0	0/0	1/1	0/0	0/0	0/0	0/0	0/0	0/0	0/0
Chromosome2-7901635 INS	PRECISE	59	0	0.707	0/0	0/0	0/0	0/0	0/1	0/1	0/0	0/0	0/0	0/0	0/0	0/0	0/0	0/0
Chromosome2-7908385 INS	PRECISE	239	0	0	0/0	0/0	0/0	0/0	0/0	0/0	0/1	0/0	0/0	0/0	0/0	0/0	0/0	0/0
Chromosome2-7909011 INS	PRECISE	7132	0	0.707	0/0	0/0	0/0	0/0	1/1	1/1	0/0	0/0	0/0	0/0	0/0	0/0	0/0	0/0
Chromosome2-7914724 INS	PRECISE	82	0	0	0/0	0/0	0/0	0/0	0/0	0/0	0/1	0/0	0/0	0/0	0/0	0/0	0/0	0/0
Chromosome2-7915533 INV	PRECISE	374	0	0	0/0	0/0	0/0	0/0	0/1	0/1	0/0	0/0	0/0	0/0	0/0	0/0	0/0	0/0
Chromosome2-7916350 INS	PRECISE	62	0	0	0/0	0/0	0/0	0/0	0/0	0/0	0/0	0/0	0/0	0/0	0/0	0/0	0/0	1/1
Chromosome2-7916947 INS	PRECISE	12580	0	0	0/0	0/0	0/0	0/0	0/0	0/1	0/0	0/0	0/0	0/0	0/0	0/0	0/0	0/0
Chromosome2-7921343 DEL	PRECISE	98	0	0	0/0	0/0	0/0	0/0	0/1	0/1	1/1	1/1	1/1	0/0	0/0	0/0	0/0	0/0
Chromosome2-7921903 INS	PRECISE	1195	0	0	0/0	0/0	0/0	0/0	0/0	0/0	0/0	0/0	0/0	0/0	0/0	0/0	0/0	1/1
Chromosome2-7924445 DEL	PRECISE	58	0	0	0/0	0/0	0/0	0/0	0/1	0/1	0/0	0/0	0/0	0/0	0/0	0/0	0/0	0/0
Chromosome2-7924526 INS	PRECISE	150	0	0.463	0/0	0/0	0/0	0/0	0/0	0/0	0/0	0/0	0/0	0/0	0/1	0/1	0/1	0/1
Chromosome2-7926868 INS	PRECISE	138	0	0.354	0/0	0/0	0/0	0/0	0/0	0/0	0/0	0/0	0/0	0/0	1/1	1/1	1/1	0/1
Chromosome2-7932894 INS	PRECISE	3261	0	0	0/0	0/0	0/1	0/0	0/0	0/0	0/0	0/0	0/0	0/0	0/0	0/0	0/0	0/0
Chromosome2-7932894 BND	PRECISE	0	0	0	0/0	0/0	0/0	0/1	0/0	0/1	0/0	0/0	0/0	0/0	0/0	0/1	0/0	0/0
Chromosome2-7945967 DEL	PRECISE	813	0	0	0/0	0/0	0/0	0/0	0/0	0/0	0/0	0/0	0/0	0/0	0/0	0/0	0/0	0/1
Chromosome2-7950424 DEL	PRECISE	64	0	0	0/0	0/0	0/0	0/0	0/0	0/0	0/0	0/0	0/0	0/0	0/0	0/0	0/0	0/1
Chromosome2-7953774 DEL	PRECISE	396	0	0	0/0	0/0	0/0	0/0	0/0	0/0	0/0	0/0	0/0	0/0	1/1	1/1	1/1	0/0
Chromosome2-7954294 INS	PRECISE	53	0	0	1/1	0/1	0/1	0/1	0/1	0/1	0/0	0/0	1/1	1/1	1/1	1/1	1/1	1/1
Chromosome2-7954363 INS	PRECISE	10299	0	0	0/0	0/0	0/0	0/0	0/0	0/0	0/0	0/0	0/0	0/0	0/0	1/1	0/0	0/0
Chromosome2-7955426 DEL	PRECISE	64	0	0	0/1	0/1	0/0	0/0	0/0	0/0	0/0	0/0	0/1	0/1	1/1	0/1	1/1	0/1
Chromosome2-7969678 BND	PRECISE	0	0	0	0/1	0/0	0/0	0/0	0/0	0/0	0/0	0/0	0/0	0/0	0/0	0/0	0/0	0/0
Chromosome2-7971027 INS	PRECISE	1220	0	0.707	0/0	0/0	0/1	0/1	0/0	0/0	0/0	0/0	0/0	0/0	0/0	0/0	0/0	0/0
Chromosome2-7972758 DEL	PRECISE	103	0	0	0/1	0/1	0/0	0/0	0/0	0/0	0/0	0/0	0/1	0/0	0/0	0/0	0/1	0/1
Chromosome2-7983996 BND	PRECISE	0	0	0	0/0	0/0	0/1	0/1	0/0	0/0	0/0	0/0	0/0	0/0	0/0	0/0	0/0	0/0
Chromosome2-7984001 BND	PRECISE	0	0	0	0/0	0/0	0/0	0/1	0/0	0/0	0/0	0/0	0/0	0/0	0/0	0/0	0/0	0/0
Chromosome2-7986602 DEL	PRECISE	66	0	0	0/0	0/0	0/0	0/0	0/0	0/0	0/0	0/0	0/0	1/1	0/0	1/1	0/0	0/0
Chromosome2-7987809 INS	PRECISE	624	0	0	0/0	0/0	0/0	0/0	0/0	0/0	0/0	0/0	0/0	1/1	0/0	0/1	0/0	0/0
Chromosome2-7991533 DEL	PRECISE	1309	0	0	0/0	0/0	0/0	0/1	0/1	0/1	0/1	1/1	1/1	0/0	0/0	0/0	0/0	0/0
Chromosome2-7994708 DEL	PRECISE	7861688	0	0	0/0	0/0	0/0	0/0	0/0	0/0	0/0	0/0	0/0	0/0	1/1	0/0	0/0	0/0
Chromosome2-7995439 INS	PRECISE	4873	0	0	0/0	0/0	0/0	0/0	0/0	0/0	0/0	0/0	0/0	0/0	0/0	1/1	1/1	0/0
Chromosome2-7996135 INS	PRECISE	468	0	0	0/0	0/0	0/0	0/0	0/0	0/0	0/0	0/0	0/0	0/0	0/0	0/0	0/0	0/1
Chromosome2-7997638 DEL	PRECISE	244	0	0	0/0	0/0	0/0	0/0	0/1	0/1	0/0	0/0	0/0	0/0	0/0	0/0	0/0	0/1
Chromosome2-8010928 INS	PRECISE	179	0	0.991	0/1	0/1	0/0	0/0	0/0	0/0	0/0	0/0	0/1	0/1	1/1	1/1	1/1	0/1
Chromosome2-8014096 DEL	PRECISE	2229	0	0	0/0	0/0	0/0	0/0	0/0	0/0	0/0	0/0	0/0	0/0	0/0	0/1	0/0	0/0
Chromosome2-8014145 INV	PRECISE	2181	0	0	0/0	0/0	0/0	0/0	0/0	0/0	0/0	0/0	0/0	0/1	0/0	0/1	0/0	0/0
Chromosome2-8015473 DEL	PRECISE	458	0	0	0/1	0/1	0/1	0/1	0/0	0/0	0/0	0/0	0/1	0/0	1/1	0/1	1/1	1/1
Chromosome2-8017254 INS	PRECISE	139	0	0.422	0/1	0/1	0/0	0/0	0/1	0/1	0/0	0/0	0/1	0/0	1/1	1/1	1/1	1/1
Chromosome2-8018822 INS	PRECISE	450	0	0.641	0/0	0/0	0/1	0/1	1/1	1/1	1/1	1/1	0/1	0/0	0/0	0/0	0/0	0/1
Chromosome2-8018822 INS	PRECISE	9913	0	0	0/0	0/0	0/0	0/0	0/0	0/0	0/0	0/0	0/0	0/0	0/1	0/0	0/0	0/0
Chromosome2-8023690 INS	PRECISE	589	0	0	0/0	0/0	0/0	0/0	0/0	0/0	0/0	0/0	0/0	0/1	0/0	0/1	0/0	0/0
Chromosome2-8029689 INS	PRECISE	56	0	0.378	0/1	0/1	0/0	0/0	0/0	0/0	0/0	0/0	0/1	0/0	1/1	0/1	1/1	0/1
Chromosome2-8042392 BND	PRECISE	0	0	0	0/0	0/0	0/0	0/0	0/0	0/0	0/0	0/0	0/0	0/0	0/0	0/0	0/0	0/1
Chromosome2-8043081 INS	PRECISE	56	0	0	0/0	0/0	0/0	0/0	0/0	0/0	0/0	0/0	0/0	0/1	0/1	1/1	0/1	0/0
Chromosome2-8044448 DEL	PRECISE	1301	0	0	0/0	0/0	0/0	0/0	0/0	0/0	0/0	0/0	0/0	0/1	0/1	1/1	0/1	0/0
Chromosome2-8046862 INS	PRECISE	1921	0	0	0/0	0/0	0/0	0/0	0/0	0/0	0/0	0/0	0/0	0/0	0/0	0/0	0/0	0/1
Chromosome2-8051136 INS	PRECISE	326	0	0.707	0/0	0/0	0/0	0/1	0/0	0/0	0/1	0/0	0/0	0/0	0/0	0/0	0/0	0/0
Chromosome2-8060695 DEL	PRECISE	5990	0	0	0/1	0/1	0/1	1/1	1/1	1/1	1/1	1/1	1/1	0/1	1/1	1/1	1/1	1/1
Chromosome2-8068148 BND	PRECISE	0	0	0	0/0	0/0	0/0	0/0	0/0	0/0	0/0	0/0	0/0	0/1	0/0	0/0	0/0	0/0
Chromosome2-8081617 DEL	PRECISE	340	0	0	0/0	0/0	0/0	0/1	0/0	0/0	0/0	0/0	0/0	0/0	0/0	0/0	0/0	0/0
Chromosome2-8081978 INS	PRECISE	1974	0	0	0/0	0/0	0/0	0/0	0/0	0/0	0/0	0/0	0/0	0/0	0/0	0/0	0/0	0/1
Chromosome2-8082186 INS	PRECISE	880	0	0.707	0/0	0/0	1/1	1/1	0/0	0/0	0/0	0/0	0/0	0/0	0/0	0/0	0/0	0/0
Chromosome2-8082332 DEL	PRECISE	1023	0	0	0/0	0/0	0/0	0/1	0/1	0/1	0/0	0/0	0/0	0/0	0/0	0/0	0/0	0/0
Chromosome2-8083820 DEL	PRECISE	635	0	0	0/0	0/0	0/0	0/0	0/1	0/1	0/0	0/0	0/0	0/0	0/0	0/0	0/0	0/0
Chromosome2-8084947 DEL	PRECISE	187	0	0	0/0	0/0	0/0	0/0	0/0	0/0	0/1	0/0	0/0	0/0	0/0	0/0	0/0	0/0
Chromosome2-8091483 DEL	PRECISE	2220	0	0	0/1	0/1	0/0	0/0	0/0	0/0	0/0	0/0	0/1	0/0	0/1	0/0	0/1	0/1
Chromosome2-8092353 DEL	PRECISE	1158	0	0	0/0	0/0	0/0	0/0	0/0	0/0	0/0	0/0	0/0	0/1	0/1	1/1	0/1	0/0
Chromosome2-8094116 INS	PRECISE	56	0	0	0/1	0/1	0/0	0/0	0/0	0/0	0/0	0/0	0/1	0/1	1/1	1/1	1/1	0/1
Chromosome2-8099511 DEL	PRECISE	80	0	0	0/0	0/0	0/1	0/1	0/0	0/0	0/0	0/0	0/0	0/0	0/0	0/0	0/0	0/0
Chromosome2-8104323 DEL	PRECISE	794	0.816	0.816	0/1	0/0	0/0	0/0	0/0	0/0	0/0	0/0	0/0	0/0	1/1	0/0	1/1	0/1
Chromosome2-8104842 DEL	PRECISE	62	0	0	0/0	0/0	0/0	0/0	0/1	0/0	0/0	0/0	0/0	0/0	0/0	0/0	0/0	0/0
Chromosome2-8107647 INS	PRECISE	599	0	0.577	0/0	0/0	0/0	0/0	0/0	0/0	0/0	0/0	0/0	1/1	0/0	0/0	0/0	0/0
Chromosome2-8117785 DEL	PRECISE	341	0	0	0/0	0/0	0/0	0/0	0/0	0/0	0/0	0/0	0/1	0/0	0/0	1/1	0/0	0/0
Chromosome2-8117981 INS	PRECISE	80	0	0	0/0	0/0	0/1	0/1	0/0	0/0	0/0	0/0	0/0	0/0	0/0	0/0	0/0	0/0

Chromosome2-8122665	DEL	PRECISE	36393	0	0	0/0	0/0	0/0	0/0	0/0	0/0	0/0	0/0	0/1	0/0	0/0	0/0	0/0	0/1
Chromosome2-8126308	INS	PRECISE	6075	0	0	0/0	0/0	0/0	1/1	0/0	0/0	0/0	0/0	0/0	0/0	0/0	0/0	0/0	0/0
Chromosome2-8135608	INS	PRECISE	13229	0	0	0/0	0/0	0/0	0/0	0/0	0/0	0/0	0/0	0/0	0/0	0/0	0/0	0/0	0/1
Chromosome2-8150336	DEL	PRECISE	91	0	0	0/0	0/0	0/0	0/0	0/0	0/0	0/0	0/0	0/0	0/0	0/0	0/0	0/0	1/1
Chromosome2-8151626	INS	PRECISE	1458	0	0	0/0	0/0	0/0	0/1	0/0	0/0	0/0	0/0	0/0	0/0	0/0	0/0	0/0	0/0
Chromosome2-8154702	BND	PRECISE	0	0	0	0/0	0/0	0/0	0/1	0/0	0/0	0/0	0/0	0/0	0/0	0/0	0/0	0/0	0/0
Chromosome2-8162564	INS	PRECISE	2287	0	0	0/0	0/0	0/0	0/1	0/0	0/0	0/0	0/0	0/0	0/0	0/0	0/0	0/0	0/0
Chromosome2-8166490	DEL	PRECISE	6096	0	0	0/0	0/0	0/1	1/1	0/1	0/1	0/0	0/0	0/0	0/0	0/0	0/0	0/0	1/1
Chromosome2-8186927	INS	PRECISE	860	0	0	0/0	0/0	0/0	0/1	0/0	0/0	0/0	0/0	0/0	0/0	0/0	0/0	0/0	0/0
Chromosome2-8192101	DEL	PRECISE	54	0	0	0/1	0/1	0/0	0/0	0/0	0/0	0/0	0/0	0/1	0/1	1/1	1/1	1/1	0/1
Chromosome2-8203603	DEL	PRECISE	203	0	0	0/0	0/0	0/0	0/0	0/0	0/0	0/0	0/0	0/0	0/0	0/0	0/1	0/1	0/0
Chromosome2-8218481	INS	PRECISE	1213	0	0	0/0	0/0	0/0	0/0	0/0	0/0	0/0	0/0	0/0	0/0	0/0	0/0	0/0	1/1
Chromosome2-8228148	DEL	PRECISE	1032	0	0	0/1	0/1	0/0	0/0	0/0	0/0	0/0	0/0	0/1	0/1	1/1	1/1	1/1	0/1
Chromosome2-8229277	DEL	PRECISE	146	0.408	0.408	0/1	0/1	0/0	0/0	0/0	0/0	0/0	0/0	0/1	0/0	0/1	0/0	0/1	0/1
Chromosome2-8230014	DEL	PRECISE	711	0	0	0/1	0/1	0/0	0/0	0/0	0/0	0/0	0/0	0/1	0/1	1/1	1/1	1/1	0/1
Chromosome2-8231257	DEL	PRECISE	624	0	0	0/0	0/0	0/0	0/0	0/1	0/1	0/0	0/0	0/0	0/0	0/0	0/0	0/0	0/0
Chromosome2-8241254	INS	PRECISE	226	0	0	0/0	0/0	0/0	0/0	0/0	0/0	0/1	0/0	0/0	0/0	0/0	0/0	0/0	0/0
Chromosome2-8256085	DEL	PRECISE	1388	0	0	0/0	0/0	0/1	0/1	1/1	1/1	0/1	1/1	1/1	0/0	0/0	0/0	0/0	1/1
Chromosome2-8267348	INS	PRECISE	162	0	0	0/0	0/0	0/0	0/0	0/0	0/0	0/0	0/0	0/0	0/0	0/0	0/0	0/0	1/1
Chromosome2-8270901	DEL	PRECISE	75	0.354	0.354	0/1	0/1	0/0	0/0	0/0	0/0	0/0	0/0	0/0	0/1	1/1	1/1	1/1	0/0
Chromosome2-8275249	INS	PRECISE	211	0	0.516	0/1	0/1	0/0	0/0	0/0	0/0	0/0	0/0	0/1	0/0	0/0	0/0	1/1	0/1
Chromosome2-8277989	INS	PRECISE	67	0	0	0/0	0/0	0/0	0/0	0/0	0/0	0/0	0/0	0/0	0/0	1/1	0/0	0/0	0/0
Chromosome2-8293745	INS	PRECISE	189	0	0	0/0	0/0	0/0	0/1	0/0	0/0	0/0	0/0	0/0	0/0	0/0	0/0	0/0	0/0
Chromosome2-8298036	INS	PRECISE	176	0	0	0/0	0/0	0/0	0/0	0/0	0/0	0/0	0/0	0/0	0/1	0/0	0/1	0/0	0/0
Chromosome2-8305210	INS	PRECISE	197	0	0	0/0	0/0	0/0	0/0	0/0	0/0	0/0	0/0	0/0	0/1	0/1	1/1	0/1	0/0
Chromosome2-8313544	INS	PRECISE	1267	0	0	0/0	0/0	0/0	0/0	0/0	0/0	0/0	0/0	0/0	0/0	0/0	0/0	0/0	1/1
Chromosome2-8314374	DEL	PRECISE	57	0	0	0/0	0/0	0/0	0/0	0/1	0/1	0/1	1/1	1/1	0/1	0/0	0/0	0/0	0/0
Chromosome2-8317789	INS	PRECISE	195	0	0.463	0/1	0/1	0/0	0/0	0/0	0/0	0/0	0/0	0/1	0/1	1/1	1/1	1/1	0/1
Chromosome2-8321200	INS	PRECISE	789	0	0	0/0	0/0	0/0	0/0	0/0	0/0	0/0	0/0	0/0	0/0	0/1	0/0	0/0	0/0
Chromosome2-8326208	INS	PRECISE	75	0	0	0/1	0/1	0/0	0/0	0/0	0/0	0/0	0/0	0/1	1/1	1/1	1/1	1/1	0/1
Chromosome2-8327597	DEL	PRECISE	2220	0	0	0/1	0/1	0/0	0/0	0/0	0/0	0/0	0/0	0/1	0/1	1/1	1/1	1/1	1/1
Chromosome2-8329499	INS	PRECISE	249	0	0.926	0/0	0/0	0/1	0/1	0/1	0/0	1/1	1/1	0/1	0/1	0/0	0/0	0/0	0/0
Chromosome2-8357755	BND	PRECISE	0	0	0	0/0	0/0	0/0	0/0	0/0	0/0	0/0	0/0	0/0	0/0	0/0	0/0	0/0	0/1
Chromosome2-8358373	DEL	PRECISE	212	0	0	0/0	0/0	0/0	0/0	0/0	0/0	0/0	0/0	0/0	0/0	0/0	0/0	0/0	0/1
Chromosome2-8367997	DEL	PRECISE	635	0	0	0/0	0/0	0/1	0/1	0/0	0/0	0/0	0/0	0/0	0/0	0/0	0/0	0/0	0/0
Chromosome2-8375759	DEL	PRECISE	82	0	0	0/0	0/0	0/0	0/0	0/1	0/1	0/0	0/0	0/0	0/0	0/0	0/0	0/0	0/0
Chromosome2-8377692	BND	PRECISE	0	0	0	0/0	0/0	0/0	0/0	0/0	0/0	0/0	0/0	0/0	0/1	0/0	0/1	0/0	0/0
Chromosome2-8382690	INS	PRECISE	783	0	0.707	0/0	0/0	0/0	0/0	0/0	0/0	0/0	0/0	0/0	0/0	0/0	0/1	0/0	0/0
Chromosome2-8385074	INS	PRECISE	81	0	0.641	0/1	0/1	0/0	0/0	0/0	0/0	0/0	0/0	0/1	0/0	1/1	1/1	1/1	0/1
Chromosome2-8387163	INS	PRECISE	56	0	0	0/0	0/0	0/0	0/1	0/0	0/0	0/0	0/0	0/0	0/0	0/0	0/0	0/0	0/0
Chromosome2-8391085	INS	PRECISE	133	0	0	0/0	0/0	0/0	0/0	0/0	0/0	0/0	0/0	0/0	0/0	0/0	0/0	0/0	1/1
Chromosome2-8391773	INS	PRECISE	531	0	0.916	0/1	0/1	0/0	0/0	0/0	0/0	0/0	0/0	0/1	0/0	1/1	1/1	1/1	0/0
Chromosome2-8393324	BND	PRECISE	0	0	0	0/0	0/0	0/0	0/0	0/0	0/0	0/0	0/0	0/0	0/0	0/1	0/1	0/1	0/0
Chromosome2-8393939	INS	PRECISE	551	0	0.707	0/0	0/0	0/1	0/1	0/0	0/0	0/0	0/0	0/0	0/0	0/0	0/0	0/0	0/0
Chromosome2-8394378	DEL	PRECISE	52	0	0	0/0	0/0	0/0	0/0	0/0	0/0	0/0	0/0	0/0	0/0	0/0	0/0	0/0	0/1
Chromosome2-8399915	DEL	PRECISE	81	0	0	0/1	0/1	0/0	0/0	0/0	0/0	0/0	0/0	0/0	0/0	1/1	1/1	1/1	0/1
Chromosome2-8400295	INS	PRECISE	383	0	0.518	0/1	1/1	0/0	0/0	0/0	0/0	0/0	0/0	0/0	1/1	0/0	1/1	0/0	0/1
Chromosome2-8400748	DEL	PRECISE	957	0	0	0/1	0/1	0/0	0/0	0/0	0/0	0/0	0/0	0/0	0/1	1/1	1/1	1/1	1/1
Chromosome2-8406284	INS	PRECISE	57	0	0	0/1	0/1	0/0	0/0	0/0	0/0	0/0	0/0	0/0	0/1	1/1	1/1	1/1	0/1
Chromosome2-8414612	INS	PRECISE	3667	0	0.707	0/0	0/0	0/0	0/0	0/0	0/0	0/0	0/0	0/0	0/0	0/0	1/1	1/1	0/0
Chromosome2-8414612	BND	PRECISE	0	0	0	0/0	0/0	0/0	0/0	0/0	0/0	0/0	0/0	0/0	0/0	0/0	0/1	0/1	0/0
Chromosome2-8414812	DEL	PRECISE	96	0	0	0/0	0/0	0/0	0/0	0/0	0/0	0/1	0/0	0/0	0/0	0/0	0/0	0/0	0/0
Chromosome2-8416187	INS	PRECISE	71	0	0	0/1	0/1	0/1	0/1	0/1	0/1	0/1	0/0	0/1	0/1	1/1	1/1	1/1	1/1
Chromosome2-8417309	DEL	PRECISE	2224	0	0	0/1	0/1	0/0	0/0	0/0	0/0	0/0	0/0	0/1	0/1	1/1	1/1	1/1	0/1
Chromosome2-8425344	DEL	PRECISE	55	0	0	0/0	0/0	0/0	0/0	0/1	0/1	0/0	0/0	0/0	0/0	0/0	0/0	0/0	0/0
Chromosome2-8434864	DEL	PRECISE	5128	0	0	0/1	0/1	0/0	0/0	0/0	0/0	0/0	0/0	0/1	0/1	1/1	1/1	1/1	0/1
Chromosome2-8434869	INV	PRECISE	5123	0	0	0/0	0/0	0/0	0/0	0/1	0/1	0/0	0/0	0/0	0/0	0/0	0/0	0/0	0/0
Chromosome2-8444222	DEL	PRECISE	5097	0	0	0/0	0/0	0/1	0/1	1/1	1/1	1/1	1/1	0/1	0/1	0/0	0/0	0/0	0/1
Chromosome2-8454704	INS	PRECISE	210	0	0.632	0/0	0/0	0/0	0/0	0/1	0/1	0/1	1/1	0/1	0/1	0/0	0/0	0/0	0/0
Chromosome2-8466032	INS	PRECISE	2377	0	0	0/0	0/0	0/0	0/0	0/0	0/0	0/0	0/0	0/0	0/0	0/0	0/0	0/0	1/1
Chromosome2-8467555	INS	PRECISE	729	0	0	0/0	0/0	0/0	0/0	0/0	0/0	0/0	0/0	0/0	0/0	0/0	0/0	0/0	1/1
Chromosome2-8471078	BND	PRECISE	0	0	0	0/0	0/0	0/0	0/0	0/0	0/0	0/0	0/0	0/0	0/0	0/1	0/0	0/0	0/0
Chromosome2-8471086	BND	PRECISE	0	0	0	0/0	0/1	0/0	0/0	0/0	0/0	0/0	0/0	0/0	0/0	0/0	0/0	0/0	0/0
Chromosome2-8473346	DEL	PRECISE	1525	0	0	0/0	0/0	0/1	0/1	0/0	0/0	0/0	0/0	0/0	0/0	0/0	0/0	0/0	0/0
Chromosome2-8477179	INS	PRECISE	121	0	0	0/0	0/0	0/0	0/0	0/0	0/0	0/0	0/0	0/0	0/0	0/0	0/0	0/0	0/1
Chromosome2-8490022	DEL	PRECISE	102	0	0	0/0	0/0	0/0	0/0	0/1	0/1	0/0	0/0	0/0	0/0	0/0	0/0	0/0	0/0
Chromosome2-8494542	DEL	PRECISE	83	0.707	0.707	0/0	0/0	0/0	0/0	0/0	0/0	0/0	0/0	0/0	0/1	0/0	0/1	0/0	0/0
Chromosome2-8496983	INS	PRECISE	55	0	0	0/0	0/0	0/0	0/0	0/0	0/0	0/0	0/0	0/0	0/0	0/0	0/0	0/0	0/1
Chromosome2-8502474	DEL	PRECISE	1390	0	0	0/0	0/0	0/1	0/1	0/0	0/0	0/0	0/0	0/0	0/0	0/0	0/0	0/0	0/0
Chromosome2-8505803	INS	PRECISE	60	0	0	0/0	0/0	0/0	0/0	0/1	0/1	0/0	0/0	0/0	0/1	0/1	1/1	0/1	0/1
Chromosome2-8519737	INS	PRECISE	452	0	0.577	0/0	0/0	0/0	0/0	0/0	0/0	0/0	0/0	0/0	0/0	0/1	0/1	0/1	0/0
Chromosome2-8527098	INS	PRECISE	462	0	0.69	0/1	0/0	0/0	0/0	0/0	0/0	0/0	0/0	0/1	0/1	0/0	0/0	0/0	0/0
Chromosome2-8536719	DEL	PRECISE	49259	0	0	0/0	0/0	0/0	0/0	0/0	0/0	0/0	0/0	0/0	0/1	1/1	0/1	0/0	0/0

Chromosome2-8537556 INS	PRECISE	4791	0	0	0/0	0/0	0/0	0/0	0/0	0/0	0/0	0/0	0/0	0/0	0/0	0/0	0/0	1/1
Chromosome2-8543567 DEL	PRECISE	2269	0	0	0/0	0/0	0/1	0/1	0/0	0/0	0/0	0/0	0/0	0/0	0/0	0/0	0/0	0/0
Chromosome2-8549157 INS	PRECISE	12894	0	0	0/0	0/0	0/0	0/1	0/0	0/0	0/0	0/0	0/0	0/0	0/0	0/0	0/0	0/0
Chromosome2-8552449 DEL	PRECISE	1016	0	0	0/0	0/0	0/0	1/1	1/1	1/1	0/1	1/1	1/1	1/1	0/0	0/0	0/0	1/1
Chromosome2-8563373 BND	PRECISE	0	0	0	0/0	0/0	0/0	0/0	0/0	0/0	0/0	0/1	0/0	0/0	0/0	0/0	0/0	0/0
Chromosome2-8573133 DEL	PRECISE	58	0	0	0/0	0/0	0/0	0/0	0/0	0/0	0/1	0/0	0/0	0/0	0/0	0/0	0/0	0/0
Chromosome2-8579988 DEL	PRECISE	5655	0	0	0/1	0/1	0/0	0/0	0/0	0/0	0/0	0/0	0/0	0/1	1/1	1/1	1/1	0/1
Chromosome2-8580649 DEL	PRECISE	4522	0	0	0/0	0/0	0/1	0/1	0/0	0/0	0/0	0/0	0/0	0/0	0/0	0/0	0/0	0/0
Chromosome2-8598680 DEL	PRECISE	357	0	0	0/0	0/0	0/1	0/1	0/0	0/0	0/0	0/0	0/0	0/0	0/0	0/0	0/0	0/0
Chromosome2-8599003 INS	PRECISE	79	0	0.463	0/0	0/0	0/0	0/1	1/1	1/1	0/1	1/1	1/1	0/1	0/0	0/0	0/0	0/1
Chromosome2-8602174 INS	PRECISE	54	0	0	0/0	0/0	0/0	0/0	0/0	0/0	0/0	0/0	0/0	0/0	0/0	0/1	0/1	0/0
Chromosome2-8603230 DEL	PRECISE	8024	0.463	0.463	0/1	0/1	0/0	0/0	0/0	0/0	0/0	0/0	0/1	0/1	0/0	1/1	1/1	0/1
Chromosome2-8613213 INS	PRECISE	454	0	0.787	0/0	0/0	0/0	0/1	1/1	1/1	0/1	1/1	0/1	0/1	0/0	0/0	0/0	0/0
Chromosome2-8621734 INS	PRECISE	106	0	0	0/1	0/1	0/0	0/0	0/0	0/0	0/0	0/0	0/1	0/1	1/1	1/1	1/1	0/1
Chromosome2-8628812 DEL	PRECISE	237	0	0	0/1	0/1	0/0	0/0	0/0	0/0	0/0	0/0	0/1	0/0	1/1	1/1	1/1	1/1
Chromosome2-8631599 DEL	PRECISE	688	0	0	0/1	0/1	0/1	0/1	0/0	0/0	0/0	0/0	0/1	0/0	1/1	1/1	1/1	1/1
Chromosome2-8633765 DEL	PRECISE	10365	0	0	0/0	0/0	0/0	0/0	0/0	0/0	0/1	0/0	0/0	0/0	0/0	0/0	0/0	0/0
Chromosome2-8635744 DEL	PRECISE	129	0	0	0/0	0/0	0/0	0/0	0/0	0/0	0/0	0/0	0/0	0/0	0/1	0/0	0/0	0/0
Chromosome2-8638680 INS	PRECISE	1520	0	0	0/0	0/0	0/0	0/1	0/0	0/0	0/0	0/0	0/0	0/0	0/0	0/0	0/0	0/0
Chromosome2-8639248 DEL	PRECISE	731	0	0	0/1	0/1	0/0	0/0	0/0	0/0	0/0	0/0	0/1	0/0	1/1	0/0	0/1	0/1
Chromosome2-8639597 INS	PRECISE	3727	0	0	0/0	0/0	0/0	0/0	0/0	0/0	0/0	0/0	0/0	0/0	0/0	0/0	0/0	1/1
Chromosome2-8641121 DEL	PRECISE	1519	0	0	0/1	0/1	0/0	0/0	0/0	0/0	0/0	0/0	0/1	0/0	1/1	0/0	0/1	0/1
Chromosome2-8641398 INS	PRECISE	63	0	0	0/0	0/0	0/0	0/0	0/1	0/1	0/0	0/0	0/0	0/0	0/0	0/0	0/0	0/0
Chromosome2-8642562 INS	PRECISE	59	0	0	0/0	0/0	0/1	0/1	0/1	0/1	0/0	0/0	0/0	0/0	0/0	0/0	0/0	0/1
Chromosome2-8642995 DEL	PRECISE	728	0.408	0.408	0/1	0/1	0/0	0/0	0/0	0/0	0/0	0/0	0/1	0/0	1/1	0/0	0/1	0/1
Chromosome2-8643907 INS	PRECISE	187	0	0	0/1	0/1	0/0	0/0	0/0	0/0	0/0	0/0	0/1	0/0	1/1	0/0	0/1	0/1
Chromosome2-8647145 INS	PRECISE	10730	0	0	0/0	0/0	0/0	0/0	0/0	0/0	0/0	0/0	0/0	0/0	0/0	0/0	0/0	0/1
Chromosome2-8648290 INS	PRECISE	256	0	0	0/0	0/0	0/0	0/0	0/0	0/0	0/0	0/0	0/0	0/0	0/0	0/0	0/0	0/1
Chromosome2-8651127 DEL	PRECISE	1099	0	0	0/1	0/1	0/0	0/0	0/1	0/1	0/0	0/0	0/1	1/1	1/1	1/1	1/1	1/1
Chromosome2-8651159 INS	PRECISE	57	0	0	0/0	0/0	0/1	0/1	0/0	0/0	0/0	0/0	0/0	0/0	0/0	0/0	0/0	0/0
Chromosome2-8652171 INS	PRECISE	58	0	0	0/0	0/0	0/0	0/1	0/0	0/0	0/0	0/0	0/0	0/0	0/0	0/0	0/0	0/0
Chromosome2-8660985 INS	PRECISE	5505	0	0.707	0/0	0/0	0/1	1/1	0/0	0/0	0/0	0/0	0/0	0/0	0/0	0/0	0/0	0/0
Chromosome2-8660985 BND	PRECISE	0	0	0	0/0	0/0	0/1	0/1	0/0	0/0	0/0	0/0	0/0	0/0	0/0	0/0	0/0	0/0
Chromosome2-8665458 DEL	PRECISE	150	0	0	0/0	0/0	0/0	0/0	0/1	0/1	0/0	0/0	0/0	0/0	0/0	0/0	0/0	0/0
Chromosome2-8665731 INS	PRECISE	5935	0	0	0/0	0/0	0/0	0/0	0/0	0/0	0/0	0/0	0/0	0/0	0/0	0/0	0/0	1/1
Chromosome2-8667659 DUP	PRECISE	13441139	0	0	0/1	0/1	0/0	0/0	0/0	0/0	0/0	0/0	0/1	0/0	0/1	0/0	0/1	0/1
Chromosome2-8675866 DEL	PRECISE	133	0	0	0/1	0/1	0/0	0/0	0/0	0/0	0/0	0/0	0/1	0/1	1/1	1/1	1/1	0/1
Chromosome2-8681030 INS	PRECISE	1791	0	0	0/0	0/0	0/0	0/0	0/0	0/0	0/0	0/0	0/0	0/1	0/0	1/1	0/1	0/0
Chromosome2-8692346 DEL	PRECISE	137	0	0	0/1	0/1	0/0	0/0	0/1	0/1	0/0	0/0	0/1	0/1	1/1	1/1	1/1	0/1
Chromosome2-8697202 INS	PRECISE	51	0	0	0/1	0/1	0/0	0/0	0/0	0/0	0/0	0/0	1/1	0/1	1/1	1/1	1/1	0/1
Chromosome2-8701080 DEL	PRECISE	102	0	0	0/1	0/1	0/0	0/0	0/0	0/0	0/0	0/0	0/1	0/0	1/1	0/0	0/1	0/1
Chromosome2-8706982 BND	PRECISE	0	0	0	0/0	0/0	0/0	0/0	0/0	0/0	0/0	0/0	0/0	0/0	1/1	0/0	1/1	0/0
Chromosome2-8718357 DEL	PRECISE	174	0	0	0/1	0/1	0/0	0/0	0/0	0/0	0/0	0/0	0/1	0/1	1/1	1/1	1/1	0/1
Chromosome2-8718688 DEL	PRECISE	858	0	0	0/1	0/1	0/1	0/1	0/1	0/1	0/0	0/0	0/1	0/1	1/1	1/1	1/1	1/1
Chromosome2-8721398 INS	PRECISE	77	0	0	0/1	0/1	0/0	0/0	0/0	0/0	0/0	0/0	0/1	0/1	1/1	1/1	1/1	0/1
Chromosome2-8740389 DEL	PRECISE	1190	0	0	0/1	0/1	0/0	0/0	0/0	0/0	0/0	0/0	0/1	0/0	1/1	0/0	1/1	0/1
Chromosome2-8747344 BND	PRECISE	0	0	0	0/0	0/0	0/0	0/0	0/0	0/0	0/0	0/0	0/0	0/1	0/0	0/1	0/0	0/0
Chromosome2-8750836 INS	PRECISE	624	0	0.707	0/0	0/0	0/0	0/0	0/1	0/1	0/0	0/0	0/0	0/0	0/0	0/0	0/0	0/0
Chromosome2-8751353 DEL	PRECISE	488	0	0	0/1	0/1	0/0	0/0	0/0	0/0	0/0	0/0	0/1	1/1	0/1	1/1	1/1	0/1
Chromosome2-8753620 DEL	PRECISE	7270	0.354	0.354	0/0	0/0	0/0	0/0	0/0	0/0	0/0	0/0	0/0	0/1	1/1	1/1	1/1	0/1
Chromosome2-8767050 INS	PRECISE	829	0	0	0/0	0/0	0/1	0/0	0/0	0/0	0/0	0/0	0/0	0/0	0/0	0/0	0/0	0/0
Chromosome2-8767844 INS	PRECISE	617	0	0	0/0	0/0	0/0	1/1	0/0	0/0	0/0	0/0	0/0	0/0	0/0	0/0	0/0	0/0
Chromosome2-8767977 DEL	PRECISE	211	0	0	0/1	0/1	0/1	1/1	1/1	1/1	1/1	1/1	1/1	1/1	1/1	1/1	1/1	1/1
Chromosome2-8786615 DEL	PRECISE	144	0	0	0/1	0/1	0/0	0/0	0/0	0/0	0/0	0/0	0/1	0/0	1/1	0/0	1/1	0/1
Chromosome2-8789061 DEL	PRECISE	873	0.516	0	0/1	0/1	0/1	0/0	0/1	0/1	0/0	0/0	0/1	0/0	1/1	0/0	1/1	1/1
Chromosome2-8793436 INS	PRECISE	5097	0	0	0/0	0/0	0/0	0/0	0/0	0/0	0/0	0/0	0/0	0/0	0/0	0/0	0/0	1/1
Chromosome2-8793619 INS	PRECISE	51	0	0	0/1	0/1	0/0	0/0	0/0	0/0	0/0	0/0	0/1	0/1	1/1	1/1	1/1	0/1
Chromosome2-8794646 INS	PRECISE	144	0	0	0/0	0/0	0/0	0/0	0/0	0/0	0/0	0/0	0/0	0/0	0/0	0/0	0/0	0/1
Chromosome2-8799327 DEL	PRECISE	10830	0	0	0/1	0/1	0/0	0/0	0/0	0/0	0/0	0/0	0/1	0/1	1/1	1/1	1/1	0/1
Chromosome2-8820248 DEL	PRECISE	667	0	0	0/0	0/0	0/0	1/1	0/1	0/1	0/1	1/1	0/1	0/1	0/0	0/0	0/0	0/0
Chromosome2-8826406 INS	PRECISE	982	0	0	0/0	0/0	0/0	0/0	0/0	0/0	0/1	0/0	0/0	0/0	0/0	0/0	0/0	0/0
Chromosome2-8829054 DEL	PRECISE	55	0	0	0/0	0/0	0/1	0/0	0/0	0/0	0/0	0/0	0/0	0/0	0/0	0/0	0/0	0/0
Chromosome2-8833969 INS	PRECISE	211	0	0	0/1	0/1	0/0	0/0	0/0	0/0	0/0	0/0	0/1	0/1	1/1	1/1	1/1	0/1
Chromosome2-8836697 BND	PRECISE	0	0	0	0/0	0/0	0/0	0/0	0/0	0/0	0/0	0/0	0/1	0/0	0/0	0/0	0/0	0/0
Chromosome2-8836706 BND	PRECISE	0	0	0	0/0	0/1	0/0	0/0	0/0	0/0	0/0	0/0	0/0	0/0	0/1	0/1	0/0	0/1
Chromosome2-8848593 DEL	PRECISE	211	0	0	0/1	0/1	0/0	0/0	0/0	0/0	0/0	0/0	1/1	0/1	1/1	1/1	1/1	1/1
Chromosome2-8850751 DEL	PRECISE	66	0	0	0/1	0/1	0/0	0/0	0/0	0/0	0/0	0/0	1/1	0/1	1/1	1/1	1/1	1/1
Chromosome2-8852215 INS	PRECISE	1426	0	0	0/0	0/0	0/0	0/0	0/0	0/0	0/0	0/0	0/0	0/0	1/1	0/0	0/0	0/0
Chromosome2-8852609 DEL	PRECISE	244	0	0	0/1	0/1	0/0	0/0	0/0	0/0	0/0	0/0	0/1	0/1	1/1	0/1	1/1	1/1
Chromosome2-8852613 DEL	PRECISE	3000	0.577	0.577	0/0	0/0	0/0	0/0	0/0	0/0	0/0	0/0	0/0	0/0	0/0	0/1	0/0	0/0
Chromosome2-8852895 DEL	PRECISE	4059	0	0.463	0/1	0/1	0/0	0/0	0/0	0/0	0/0	0/0	1/1	0/1	1/1	1/1	1/1	1/1
Chromosome2-8855616 INS	PRECISE	64	0	0	0/0	0/0	0/1	1/1	1/1	1/1	1/1	1/1	0/0	0/1	0/0	0/0	0/0	0/0
Chromosome2-8855666 DEL	PRECISE	1294	0	0	0/0	0/0	0/0	0/0	0/0	0/0	0/0	0/0	0/0	0/0	0/0	0/1	0/0	0/0

Chromosome2-8862649	DEL	PRECISE	221	0	0	0/1	0/1	0/0	0/0	0/0	0/0	0/0	0/0	0/1	0/1	1/1	1/1	1/1	1/1
Chromosome2-8866818	INS	PRECISE	395	0	0	0/0	0/0	0/0	0/0	0/0	0/0	0/1	0/0	0/0	0/0	0/0	0/0	0/0	0/0
Chromosome2-8866818	INS	PRECISE	5413	0	0	0/0	0/0	0/0	1/1	0/0	0/0	0/0	0/0	0/0	0/0	0/0	0/0	0/0	0/0
Chromosome2-8869017	DEL	PRECISE	78	0	0	0/1	0/1	0/0	0/0	0/0	0/0	0/0	0/0	0/1	0/0	1/1	1/1	1/1	0/1
Chromosome2-8881147	INS	PRECISE	63	0	0.389	0/1	0/0	0/0	1/1	1/1	1/1	1/1	1/1	1/1	1/1	1/1	1/1	1/1	0/0
Chromosome2-8883231	DEL	PRECISE	148	0	0	0/0	0/0	0/0	0/0	0/0	0/0	0/0	0/0	0/0	0/1	0/1	0/1	0/0	0/0
Chromosome2-8888093	DEL	PRECISE	150	0	0	0/0	0/0	0/1	0/0	0/0	0/0	0/0	0/0	0/0	0/0	0/0	0/0	0/0	0/0
Chromosome2-8891818	INS	PRECISE	75	0	0	0/0	0/0	0/0	0/0	0/0	0/0	0/0	0/0	0/0	0/1	0/0	1/1	0/1	0/0
Chromosome2-8896816	INS	PRECISE	161	0	0.354	0/1	0/1	0/0	0/0	0/0	0/0	0/0	0/0	0/1	0/1	1/1	0/1	0/1	0/1
Chromosome2-8900134	INS	PRECISE	150	0.926	0.518	0/1	0/1	0/0	0/0	0/0	0/0	0/0	0/0	0/1	0/0	1/1	1/1	1/1	0/1
Chromosome2-8900959	DEL	PRECISE	54	0	0	0/0	0/0	0/1	0/0	0/0	0/0	0/0	0/0	0/0	0/0	0/0	0/0	0/0	0/0
Chromosome2-8908137	DEL	PRECISE	77	0	0	0/0	0/0	0/0	1/1	1/1	1/1	1/1	1/1	1/1	1/1	0/0	0/0	0/0	0/0
Chromosome2-8914555	DEL	PRECISE	12561	0	0	0/0	0/0	0/0	0/0	0/0	0/0	0/0	0/0	0/0	0/0	0/0	0/0	0/0	1/1
Chromosome2-8927116	BND	PRECISE	0	0	0	0/0	0/0	0/1	0/0	0/0	0/0	0/0	0/0	0/0	0/0	0/0	0/0	0/0	0/0
Chromosome2-8934525	INV	PRECISE	12003	0	0	0/1	0/1	0/0	0/0	0/0	0/0	0/0	0/0	0/1	0/1	1/1	1/1	1/1	0/1
Chromosome2-8947307	INS	PRECISE	1863	0	0	0/0	0/0	0/0	0/0	0/0	0/0	0/0	0/0	0/0	0/0	0/0	0/0	0/0	1/1
Chromosome2-8948470	INS	PRECISE	11816	0	0	0/0	0/0	0/0	0/0	0/0	0/0	0/1	0/0	0/0	0/0	0/0	0/0	0/0	0/0
Chromosome2-8948895	DEL	PRECISE	492	0	0	0/0	0/0	0/0	0/0	0/0	0/0	0/0	0/0	0/0	0/0	0/0	0/0	0/0	0/1
Chromosome2-8949394	INS	PRECISE	133	0	0.378	0/0	0/0	0/0	1/1	0/1	0/1	1/1	1/1	0/1	0/1	0/0	0/0	0/0	0/0
Chromosome2-8950011	DEL	PRECISE	718	0	0	0/1	0/1	0/0	1/1	0/1	0/1	1/1	1/1	1/1	1/1	1/1	1/1	1/1	1/1
Chromosome2-8954677	BND	PRECISE	0	0	0	0/0	0/0	0/0	0/1	0/0	0/0	0/0	0/0	0/0	0/0	0/0	0/0	0/0	0/0
Chromosome2-8954859	INS	PRECISE	910	0	0	0/0	0/0	0/0	0/0	0/0	0/0	0/0	0/0	0/0	0/0	0/1	0/0	0/0	0/0
Chromosome2-8960272	DEL	PRECISE	1006	0	0	0/1	0/1	0/1	1/1	0/1	0/1	1/1	1/1	1/1	1/1	1/1	1/1	1/1	1/1
Chromosome2-8967476	INS	PRECISE	955	0	0	0/0	0/0	0/0	0/0	0/0	0/0	0/1	0/0	0/0	0/0	0/0	0/0	0/0	0/0
Chromosome2-8970950	INS	PRECISE	701	0	0.707	0/0	0/0	0/0	0/0	0/1	0/1	0/0	0/0	0/0	0/0	0/0	0/0	0/0	0/0
Chromosome2-8974532	DEL	PRECISE	56	0	0	0/1	0/0	0/0	0/0	0/0	0/0	0/0	0/0	0/1	0/1	0/1	1/1	1/1	0/1
Chromosome2-8977454	INS	PRECISE	2663	0	0	0/0	0/0	0/0	0/0	0/0	0/0	0/0	0/0	0/0	0/0	0/1	0/0	0/0	0/0
Chromosome2-8980921	DEL	PRECISE	2404	0.577	0.577	0/0	0/0	0/0	0/0	0/0	0/0	0/0	0/0	0/0	0/0	0/0	0/1	0/1	0/0
Chromosome2-8987541	DEL	PRECISE	2233	0	0	0/0	0/0	0/0	0/0	0/0	0/0	0/0	0/0	0/0	0/0	0/0	0/0	0/0	0/0
Chromosome2-8989526	DEL	PRECISE	132	0	0	0/0	0/0	0/1	0/0	0/0	0/0	0/0	0/0	0/0	0/0	0/0	0/0	0/0	0/0
Chromosome2-8990019	INS	PRECISE	330	0	0	0/0	0/0	0/1	0/1	0/1	0/1	1/1	0/0	0/0	0/0	0/0	0/0	0/0	0/1
Chromosome2-8995623	DEL	PRECISE	1546	0.756	0.756	0/0	0/1	0/0	0/0	0/0	0/0	0/0	0/0	0/0	0/0	0/1	0/0	0/1	0/1
Chromosome2-8996565	INS	PRECISE	5062	0	0	0/0	0/0	0/0	0/0	0/0	0/0	0/0	0/0	0/0	0/0	0/0	0/0	0/0	1/1
Chromosome2-8997361	INS	PRECISE	6819	0	0	0/0	0/0	0/1	0/0	0/0	0/0	0/0	0/0	0/0	0/0	0/0	0/0	0/0	0/0
Chromosome2-8997797	DEL	PRECISE	68	0	0	0/0	0/0	0/1	0/0	0/0	0/0	0/0	0/0	0/0	0/0	0/0	0/0	0/0	0/0
Chromosome2-8997809	INS	PRECISE	1484	0	0	0/0	0/0	0/1	0/0	0/0	0/0	0/0	0/0	0/0	0/0	0/0	0/0	0/0	0/0
Chromosome2-8997885	INS	PRECISE	161	0	0	0/0	0/1	0/0	0/0	0/0	0/0	0/0	0/0	0/0	0/0	0/1	0/0	0/0	0/0
Chromosome2-9001301	INS	PRECISE	347	0	0.535	0/1	0/1	0/0	0/0	0/0	0/0	0/0	0/0	0/1	0/0	0/1	0/1	0/1	0/1
Chromosome2-9008068	DEL	PRECISE	633	0	0	0/0	0/0	0/0	0/1	0/1	0/1	0/1	1/1	0/1	0/1	0/0	0/0	0/0	0/0
Chromosome2-9008381	INS	PRECISE	1410	0	0	0/0	0/0	0/0	0/0	0/0	0/0	0/0	0/0	0/0	0/0	0/0	0/0	0/0	0/1
Chromosome2-9011539	DEL	PRECISE	103	0	0	0/1	0/1	0/0	0/0	0/0	0/0	0/0	0/0	0/1	0/0	0/1	0/1	1/1	0/1
Chromosome2-9013397	DEL	PRECISE	80	0	0	0/0	0/0	0/0	0/0	0/0	0/0	0/0	0/0	0/0	0/1	0/1	0/1	0/0	0/0
Chromosome2-9014730	INS	PRECISE	59	0.5	0	0/1	0/1	0/1	0/0	0/0	0/0	0/0	0/0	0/1	0/1	1/1	1/1	1/1	1/1
Chromosome2-9015067	DEL	PRECISE	336	0	0	0/0	0/0	0/0	0/0	0/1	0/1	0/0	0/0	0/0	0/0	0/0	0/0	0/0	0/0
Chromosome2-9018034	INS	PRECISE	87	0	0.707	0/0	0/0	0/0	0/0	0/0	0/0	0/0	0/0	0/0	0/1	0/0	0/1	0/0	0/0
Chromosome2-9020602	BND	PRECISE	0	0	0	0/0	0/0	0/0	0/0	0/0	0/0	0/0	0/0	0/0	0/0	0/1	0/0	0/0	0/0
Chromosome2-9028964	DEL	PRECISE	51748	0	0	0/0	0/1	0/0	0/0	0/0	0/0	0/0	0/0	0/0	0/1	0/1	1/1	0/1	0/0
Chromosome2-9029887	INS	PRECISE	146	0	0.354	0/0	0/0	0/0	1/1	0/1	0/0	1/1	1/1	0/1	0/1	0/0	0/0	0/0	0/0
Chromosome2-9041753	INS	PRECISE	59	0	0	0/0	0/0	0/0	0/1	1/1	1/1	1/1	1/1	0/1	1/1	0/0	0/0	0/0	0/0
Chromosome2-9041765	DEL	PRECISE	95	0	0	0/0	0/0	0/0	0/0	0/0	0/0	0/0	0/0	0/1	0/0	1/1	0/0	1/1	0/0
Chromosome2-9048631	DEL	PRECISE	187	0	0	0/0	0/0	0/0	0/1	0/0	0/0	0/0	0/0	0/0	0/0	0/0	0/0	0/0	0/0
Chromosome2-9065510	DEL	PRECISE	66	0	0	0/0	0/0	0/0	1/1	1/1	1/1	1/1	1/1	1/1	1/1	0/0	0/0	0/0	1/1
Chromosome2-9070031	INS	PRECISE	464	0	0	0/0	0/0	0/0	0/0	0/0	0/0	0/0	0/0	0/0	0/0	0/0	0/0	0/0	1/1
Chromosome2-9080564	INS	PRECISE	1058	0	0	0/0	0/0	0/0	0/0	0/0	0/0	0/0	0/0	0/0	0/0	0/0	0/0	0/0	1/1
Chromosome2-9084257	DEL	PRECISE	2570	0	0	0/0	0/0	0/0	0/1	0/0	0/0	0/0	0/0	0/0	0/0	0/0	0/0	0/0	0/0
Chromosome2-9086135	DEL	PRECISE	50	0.667	0.333	0/1	0/1	0/1	0/0	0/0	0/0	0/0	0/0	0/1	0/1	1/1	1/1	1/1	0/0
Chromosome2-9091692	DEL	PRECISE	95	0	0	0/1	0/1	0/0	0/0	0/0	0/0	0/0	0/0	0/1	0/1	1/1	1/1	1/1	0/1
Chromosome2-9097379	INS	PRECISE	58	0	0	0/0	0/0	0/0	0/0	0/0	0/0	0/1	0/0	0/0	0/0	0/0	0/0	0/0	0/0
Chromosome2-9099778	INS	PRECISE	120	0	0	0/0	0/0	0/0	0/0	0/0	0/0	0/0	0/0	0/0	0/1	0/0	0/0	0/0	0/0
Chromosome2-9103854	INS	PRECISE	1634	0	0.707	0/0	0/0	0/0	0/0	0/0	0/0	0/0	0/0	0/0	0/1	0/0	0/1	0/0	0/0
Chromosome2-9110715	BND	PRECISE	0	0	0	0/0	0/0	0/0	0/0	0/0	0/0	0/0	0/0	0/1	0/0	0/1	0/1	1/1	0/0
Chromosome2-9111523	INS	PRECISE	1490	0	0.707	0/0	0/0	0/0	0/0	0/0	0/0	0/0	0/0	0/0	0/1	0/0	1/1	0/0	0/0
Chromosome2-9111754	INS	PRECISE	77	0	0.5	0/0	0/0	0/0	0/0	0/0	0/0	0/0	0/0	0/0	0/1	1/1	1/1	0/0	0/0
Chromosome2-9111755	BND	PRECISE	0	0.408	0	0/0	0/0	0/0	0/0	0/0	0/0	0/0	0/0	0/1	0/0	1/1	0/1	0/0	0/0
Chromosome2-9118112	INS	PRECISE	87	0	0	0/0	0/0	0/0	0/1	0/0	0/0	0/0	0/0	0/0	0/0	0/0	0/0	0/0	0/0
Chromosome2-9119104	DEL	PRECISE	81	0	0	0/0	0/0	0/1	0/0	0/0	0/0	0/0	0/0	0/0	0/0	0/0	0/0	0/0	0/0
Chromosome2-9127009	INS	PRECISE	107	0	0	0/0	0/0	0/1	0/0	0/0	0/0	0/0	0/0	0/0	0/0	0/0	0/0	0/0	0/0
Chromosome2-9129718	DEL	PRECISE	52	0	0	0/0	0/0	0/0	0/1	0/1	0/1	0/0	1/1	0/1	0/1	0/0	0/0	0/0	0/0
Chromosome2-9134116	INS	PRECISE	524	0	0	0/0	0/0	0/1	0/0	0/0	0/0	0/0	0/0	0/0	0/0	0/0	0/0	0/0	0/0
Chromosome2-9134457	INS	PRECISE	2368	0	0	0/0	0/0	0/0	0/0	0/0	0/0	0/0	0/0	0/0	0/0	0/0	0/0	0/0	0/1
Chromosome2-9155774	BND	PRECISE	0	0.707	0	0/1	0/0	0/0	0/0	0/0	0/0	0/0	0/0	1/1	0/0	0/0	0/0	0/0	1/1
Chromosome2-9155774	BND	PRECISE	0	0.756	0	0/1	0/0	0/0	0/0	0/0	0/0	0/0	0/0	0/0	0/0	0/0	0/0	0/0	0/1
Chromosome2-9174810	DEL	PRECISE	197	0	0	0/1	0/1	0/0	0/0	0/0	0/0	0/0	0/0	0/1	0/1	1/1	1/1	1/1	0/1

Chromosome2-9175560 INS	PRECISE	623	0	0.707	0/0	0/0	0/0	0/0	0/0	0/0	0/0	0/0	1/1	0/0	0/0	0/1	0/0	0/0
Chromosome2-9176628 INS	PRECISE	640	0	0.991	0/1	0/1	0/0	0/0	0/0	0/0	0/0	0/0	0/1	0/1	1/1	1/1	1/1	0/1
Chromosome2-9184493 INS	PRECISE	995	0	0	0/0	0/0	0/0	0/1	0/0	0/0	0/0	0/0	0/0	0/0	0/0	0/0	0/0	0/0
Chromosome2-9189866 DEL	PRECISE	129	0	0	0/0	0/0	0/0	0/0	0/0	0/0	0/0	0/0	0/0	0/0	0/0	0/0	0/0	1/1
Chromosome2-9192037 INS	PRECISE	5956	0	0	0/0	0/0	0/0	0/0	0/0	0/0	0/0	0/0	0/0	0/0	0/0	0/0	0/0	1/1
Chromosome2-9193333 INS	PRECISE	11033	0	0	0/0	0/0	0/0	0/0	0/0	0/0	1/1	0/0	0/0	0/0	0/0	0/0	0/0	0/0
Chromosome2-9193333 BND	PRECISE	0	0	0	0/0	0/0	0/0	0/0	0/0	0/0	0/1	0/0	0/0	0/0	0/0	0/0	0/0	0/0
Chromosome2-9193696 INS	PRECISE	3621	0	0	0/0	0/0	0/0	0/0	0/0	0/0	0/0	0/0	0/0	0/0	1/1	0/0	0/0	0/0
Chromosome2-9195192 DEL	PRECISE	1244	0	0	0/0	0/0	0/0	0/0	0/0	0/0	0/0	0/0	0/0	0/0	0/0	0/0	0/0	0/1
Chromosome2-9198839 DEL	PRECISE	679	0	0	0/0	0/0	0/0	0/0	0/0	0/0	0/0	0/0	0/1	1/1	1/1	1/1	1/1	0/0
Chromosome2-9199669 INS	PRECISE	4266	0	0	0/0	0/0	0/0	0/0	0/0	0/0	0/0	0/0	0/0	0/0	0/0	0/0	0/0	1/1
Chromosome2-9199945 INS	PRECISE	329	0	0	0/0	0/0	0/0	0/0	0/0	0/0	0/0	0/0	0/0	0/0	0/0	0/0	0/0	0/1
Chromosome2-9200345 DEL	PRECISE	447	0	0	0/1	0/1	0/0	0/0	0/0	0/0	0/0	0/0	0/1	1/1	1/1	1/1	1/1	0/0
Chromosome2-9202405 DEL	PRECISE	718	0	0	0/0	0/0	0/0	0/0	0/0	0/0	0/0	0/0	0/0	0/0	0/0	0/0	0/0	1/1
Chromosome2-9208703 INS	PRECISE	12358	0	0	0/0	0/0	0/1	0/0	0/0	0/0	0/0	0/0	0/0	0/0	0/0	0/0	0/0	0/0
Chromosome2-9208708 BND	PRECISE	0	0	0	0/0	0/0	1/1	0/0	0/0	0/0	0/0	0/0	0/0	0/0	0/0	0/0	0/0	0/0
Chromosome2-9208873 DEL	PRECISE	795	0	0	0/1	0/1	0/0	0/0	0/0	0/0	0/0	0/0	0/1	0/1	1/1	1/1	1/1	0/1
Chromosome2-9210643 INS	PRECISE	158	0	0	0/0	0/0	0/1	0/0	0/0	0/0	0/0	0/0	0/0	0/0	0/0	0/0	0/0	0/0
Chromosome2-9211387 DEL	PRECISE	189	0	0	0/1	0/1	0/0	0/0	0/0	0/0	0/0	0/0	0/1	0/1	1/1	1/1	1/1	0/1
Chromosome2-9214562 INS	PRECISE	1456	0	0	0/0	0/0	0/1	0/0	0/0	0/0	0/0	0/0	0/0	0/0	0/0	0/0	0/0	0/0
Chromosome2-9216137 BND	PRECISE	0	0	0	0/1	0/1	0/0	0/0	0/0	0/0	0/0	0/0	0/1	0/1	0/1	0/1	0/1	0/1
Chromosome2-9223845 DEL	PRECISE	66	0	0	0/0	0/0	0/0	0/0	0/0	0/0	0/0	0/0	0/0	0/0	0/0	0/0	0/0	0/1
Chromosome2-9225604 INS	PRECISE	234	0	0.354	0/1	0/1	0/0	0/0	0/0	0/0	0/0	0/0	0/1	0/1	1/1	1/1	1/1	0/1
Chromosome2-9237695 DEL	PRECISE	6141	0	0	1/1	0/1	0/0	0/0	0/0	0/0	0/0	0/0	1/1	0/1	1/1	0/0	1/1	1/1
Chromosome2-9238194 DEL	PRECISE	83	0	0	0/0	0/0	0/0	0/0	0/0	0/0	0/0	0/0	0/0	0/0	0/0	0/0	0/0	1/1
Chromosome2-9238214 INS	PRECISE	942	0	0	0/0	0/0	0/0	0/0	0/0	0/0	0/1	0/0	0/0	0/0	0/0	0/0	0/0	0/0
Chromosome2-9242203 INS	PRECISE	192	0	0	0/0	0/0	0/0	0/0	0/0	0/0	0/1	0/0	0/0	0/0	0/0	0/0	0/0	0/0
Chromosome2-9245621 DEL	PRECISE	68	0	0	0/0	0/0	0/0	0/0	0/0	0/0	0/1	0/0	0/0	0/0	0/0	0/0	0/0	0/0
Chromosome2-9249321 INS	PRECISE	182	0	0	0/1	0/1	0/0	0/0	0/0	0/0	0/0	0/0	0/1	0/1	1/1	1/1	1/1	0/1
Chromosome2-9251253 DEL	PRECISE	624	0.577	0	0/1	0/1	0/1	0/0	0/1	0/1	0/1	0/0	1/1	0/1	1/1	1/1	1/1	1/1
Chromosome2-9257706 INS	PRECISE	883	0	0	0/0	0/0	0/0	0/0	0/0	0/0	0/0	0/0	0/0	0/0	0/0	0/0	0/0	0/1
Chromosome2-9268829 DEL	PRECISE	89	0.5	0.5	0/0	0/0	0/0	0/0	0/0	0/0	0/0	0/0	0/0	0/0	1/1	1/1	1/1	0/0
Chromosome2-9269261 DEL	PRECISE	1302	0	0	0/0	0/0	0/0	0/0	0/0	0/0	0/0	0/0	0/0	0/0	1/1	1/1	1/1	0/0
Chromosome2-9270851 DEL	PRECISE	485	0	0	0/0	0/0	0/0	0/0	0/0	0/0	0/0	0/0	0/0	0/0	1/1	1/1	1/1	0/0
Chromosome2-9272231 BND	PRECISE	0	0	0	0/0	0/0	0/0	0/0	0/0	0/0	0/0	0/0	0/0	0/0	0/0	0/0	0/0	0/1
Chromosome2-9272234 DEL	PRECISE	56	0	0	0/0	0/0	0/0	0/0	0/0	0/0	0/0	0/0	0/0	0/0	0/1	0/0	0/0	0/0
Chromosome2-9275938 INS	PRECISE	74	0	0.744	0/1	0/1	0/0	0/0	0/0	0/0	0/0	0/0	0/1	0/1	1/1	1/1	1/1	0/1
Chromosome2-9282389 INS	PRECISE	198	0	0	0/1	0/1	0/0	0/0	0/0	0/0	0/0	0/0	0/1	0/1	1/1	1/1	1/1	0/1
Chromosome2-9286119 INS	PRECISE	101	0	0.354	0/1	0/1	0/0	0/0	0/0	0/0	0/0	0/0	0/1	0/1	1/1	1/1	1/1	0/1
Chromosome2-9286635 DEL	PRECISE	3159	0	0	0/1	0/1	0/0	0/0	0/0	0/0	0/0	0/0	0/1	1/1	1/1	1/1	1/1	0/1
Chromosome2-9290946 DEL	PRECISE	51	0	0	0/0	0/0	0/0	0/0	0/1	0/1	0/0	0/0	0/0	0/0	0/0	0/0	0/0	0/0
Chromosome2-9291517 DEL	PRECISE	651	0	0	0/0	0/0	0/0	0/0	0/0	0/0	0/0	0/0	0/0	0/0	0/0	0/0	0/0	0/1
Chromosome2-9295426 INS	PRECISE	123	0	0	0/1	0/1	0/0	0/0	0/0	0/0	0/0	0/0	0/1	1/1	1/1	1/1	1/1	0/1
Chromosome2-9300568 DEL	PRECISE	55	0	0	0/0	0/1	0/0	0/0	0/0	0/0	0/0	0/0	0/0	0/1	1/1	0/1	0/0	0/0
Chromosome2-9303329 DEL	PRECISE	465	0	0	0/0	0/1	0/0	0/0	0/1	0/1	0/0	0/0	0/1	0/1	1/1	1/1	1/1	1/1
Chromosome2-9326603 INS	PRECISE	1863	0	0	0/0	0/0	0/1	0/0	0/0	0/0	0/0	0/0	0/0	0/0	0/0	0/0	0/0	0/0
Chromosome2-9326686 DEL	PRECISE	146	0	0	0/0	0/0	0/0	0/0	0/0	0/0	0/0	0/0	0/0	0/0	0/0	1/1	1/1	0/0
Chromosome2-9334976 DEL	PRECISE	12813	0	0	0/0	0/0	0/1	0/0	0/1	0/1	0/0	0/0	0/0	0/0	0/0	0/0	0/0	1/1
Chromosome2-9354101 DEL	PRECISE	85	0	0	0/0	0/0	0/1	0/1	0/0	0/0	0/1	0/0	0/0	0/0	0/0	0/0	0/0	1/1
Chromosome2-9357484 INS	PRECISE	127	0	0	0/0	0/0	0/0	0/0	0/0	0/0	0/0	0/0	0/0	0/0	0/0	0/0	0/0	1/1
Chromosome2-9358179 DEL	PRECISE	433	0	0	0/0	0/0	0/0	0/1	0/1	0/0	0/0	0/0	0/0	0/0	0/0	0/0	0/0	0/0
Chromosome2-9359964 DEL	PRECISE	625	0	0	0/0	0/0	0/1	0/1	0/1	0/1	0/1	0/0	0/0	0/0	0/0	0/0	0/0	1/1
Chromosome2-9366069 INS	PRECISE	133	0	0	0/0	0/0	0/0	0/0	0/0	0/0	0/1	0/0	0/0	0/0	0/0	0/0	0/0	0/0
Chromosome2-9380610 DEL	PRECISE	1103	0	0	0/0	0/0	0/0	0/0	0/0	0/0	0/1	0/0	0/0	0/0	0/0	0/0	0/0	0/0
Chromosome2-9381623 DEL	PRECISE	430	0	0	0/0	0/0	0/0	0/0	0/1	0/1	0/0	0/0	0/0	0/0	0/0	0/0	0/0	0/0
Chromosome2-9382242 DEL	PRECISE	12298	0	0	0/0	0/0	0/1	0/1	0/1	0/1	0/1	0/0	0/0	0/0	0/0	0/0	0/0	0/0
Chromosome2-9385085 DEL	PRECISE	242	0	0	0/0	0/0	0/0	0/0	0/0	0/0	0/0	0/0	0/0	0/0	0/0	0/0	0/0	1/1
Chromosome2-9397165 INS	PRECISE	341	0	0	0/0	0/0	0/1	0/0	0/0	0/0	0/0	0/0	0/0	0/0	0/0	0/0	0/0	0/0
Chromosome2-9414369 DEL	PRECISE	1552	0	0	0/0	0/0	0/1	0/0	0/1	0/1	0/0	0/0	0/0	0/0	0/0	0/0	0/0	1/1
Chromosome2-9419740 BND	PRECISE	0	0	0	0/0	0/0	0/0	0/0	0/0	0/0	0/0	0/0	0/0	0/0	0/0	0/0	0/0	0/1
Chromosome2-9428316 DEL	PRECISE	1942	0	0	0/0	0/0	0/0	0/0	0/0	0/0	0/0	0/0	0/0	0/0	0/0	0/0	0/0	1/1
Chromosome2-9454296 INS	PRECISE	628	0	0	0/0	0/0	0/1	0/0	0/0	0/0	0/0	0/0	0/0	0/0	0/0	0/0	0/0	0/0
Chromosome2-9455725 INS	PRECISE	962	0	0	0/0	0/0	0/1	0/0	0/0	0/0	0/0	0/0	0/0	0/0	0/0	0/0	0/0	0/0
Chromosome2-9479909 DEL	PRECISE	173	0	0	0/0	0/0	0/1	0/0	0/0	0/0	0/0	0/0	0/0	0/0	0/0	0/0	0/0	0/0
Chromosome2-9513547 INS	PRECISE	79	0	0	0/0	0/0	0/0	0/1	0/0	0/0	0/0	0/0	0/0	0/0	0/0	0/0	0/0	0/0
Chromosome2-9520345 DEL	PRECISE	8561	0	0	0/0	0/0	0/0	0/0	0/1	0/1	0/0	0/0	0/0	0/0	0/0	0/0	0/0	0/0
Chromosome2-9534415 DEL	PRECISE	1389	0	0	0/0	0/0	0/1	0/1	0/1	0/1	0/1	0/0	0/0	0/0	0/0	0/0	0/0	1/1
Chromosome2-9540291 DEL	PRECISE	63	0	0	0/0	0/0	0/0	0/1	0/0	0/0	0/0	0/0	0/0	0/0	0/0	0/0	0/0	0/0
Chromosome2-9541391 DEL	PRECISE	677	0	0	0/0	0/0	0/1	0/0	0/0	0/0	0/0	0/0	0/0	0/0	0/0	0/0	0/0	0/0
Chromosome2-9544504 INS	PRECISE	1781	0	0	0/0	0/0	0/0	0/0	0/0	0/0	0/0	0/0	0/0	0/0	0/0	0/0	0/0	1/1
Chromosome2-9577539 DEL	PRECISE	136	0	0	0/0	0/0	0/1	0/1	0/1	0/0	0/1	0/0	0/0	0/0	0/0	0/0	0/0	1/1
Chromosome2-9580379 INS	PRECISE	477	0	0	0/0	0/0	0/0	0/0	0/0	0/0	0/0	0/0	0/0	0/0	0/0	0/0	0/0	1/1
Chromosome2-9613918 DEL	PRECISE	175	0	0	0/0	0/0	0/1	0/0	0/0	0/0	0/0	0/0	0/0	0/0	0/0	0/0	0/0	0/0

Chromosome2-9635800	DEL	PRECISE	96	0	0	0/0	0/0	0/0	0/1	0/1	0/1	0/0	0/0	0/0	0/0	0/0	0/0	0/0	0/0
Chromosome2-9644297	DEL	PRECISE	521	0	0	0/0	0/0	0/0	0/1	0/0	0/0	0/0	0/0	0/0	0/0	0/0	0/0	0/0	0/0
Chromosome2-9645774	DEL	PRECISE	129	0	0	0/0	0/0	0/0	0/1	0/1	0/1	0/0	0/0	0/0	0/0	0/0	0/0	0/0	0/0
Chromosome2-9650175	INS	PRECISE	122	0	0	0/0	0/0	0/0	0/1	0/0	0/0	0/0	0/0	0/0	0/0	0/0	0/0	0/0	0/0
Chromosome2-9652685	INS	PRECISE	52	0	0	0/0	0/0	0/1	0/0	0/0	0/0	0/0	0/0	0/0	0/0	0/0	0/0	0/0	0/0
Chromosome2-9663302	DEL	PRECISE	625	0	0	0/0	0/0	0/0	0/0	0/0	0/0	0/0	0/0	0/0	0/0	0/0	0/0	0/0	1/1
Chromosome2-9698602	DEL	PRECISE	375	0	0	0/0	0/0	0/0	0/0	0/0	0/0	0/0	0/0	0/0	0/0	0/0	0/0	0/0	1/1
Chromosome2-9699147	INS	PRECISE	651	0	0	0/0	0/0	0/0	0/1	0/0	0/0	0/0	0/0	0/0	0/0	0/0	0/0	0/0	0/0
Chromosome2-9706217	INS	PRECISE	783	0	0	0/0	0/0	0/1	0/0	0/0	0/0	0/0	0/0	0/0	0/0	0/0	0/0	0/0	0/0
Chromosome2-9720803	INS	PRECISE	370	0	0.707	0/0	0/0	0/0	0/1	0/0	0/0	0/1	0/0	0/0	0/0	0/0	0/0	0/0	0/0
Chromosome2-9727244	DEL	PRECISE	643	0	0	0/0	0/0	0/1	0/1	0/1	0/1	0/1	0/0	0/0	0/0	0/0	0/0	0/0	1/1
Chromosome2-9731621	DEL	PRECISE	120	0	0	0/0	0/0	0/1	0/0	0/0	0/0	0/0	0/0	0/0	0/0	0/0	0/0	0/0	0/0
Chromosome2-9738190	DEL	PRECISE	63	0	0	0/0	0/0	0/1	0/0	0/0	0/0	0/0	0/0	0/0	0/0	0/0	0/0	0/0	0/0
Chromosome2-9745066	INS	PRECISE	5559	0	0	0/0	0/0	0/0	0/0	0/0	0/0	0/0	0/0	0/0	0/0	0/0	0/0	0/0	1/1
Chromosome2-9745072	BND	PRECISE	0	0	0	0/0	0/0	0/0	0/0	0/0	0/0	0/0	0/0	0/0	0/0	0/0	0/0	0/0	0/1
Chromosome2-9746915	INS	PRECISE	85	0	0	0/0	0/0	0/0	0/0	0/1	0/1	0/0	0/0	0/0	0/0	0/0	0/0	0/0	0/0
Chromosome2-9751017	DEL	PRECISE	91	0	0	0/0	0/0	0/1	0/0	0/0	0/0	0/0	0/0	0/0	0/0	0/0	0/0	0/0	0/0
Chromosome2-9754907	DEL	PRECISE	121	0	0	0/0	0/0	0/1	0/1	0/1	0/1	0/0	0/0	0/0	0/0	0/0	0/0	0/0	1/1
Chromosome2-9759091	INS	PRECISE	1127	0	0	0/0	0/0	0/0	0/0	0/0	0/0	0/0	0/0	0/0	0/0	0/0	0/0	0/0	0/1
Chromosome2-9766528	DEL	PRECISE	66	0.603	0.302	0/1	0/0	0/0	0/1	0/1	0/1	0/1	0/1	0/1	0/0	0/0	0/0	0/0	0/1
Chromosome2-9766823	BND	PRECISE	0	0	0	0/1	0/0	0/1	0/0	0/1	0/0	0/0	0/0	0/0	0/0	0/0	0/0	0/0	0/1
Chromosome2-9766823	BND	PRECISE	0	0	0	0/0	0/0	0/0	0/0	0/0	0/0	0/0	0/1	0/1	0/0	0/0	0/0	0/0	0/0
Chromosome2-9766823	BND	PRECISE	0	0	0	0/0	0/0	0/0	0/0	0/0	0/0	0/0	0/0	0/0	0/0	0/0	0/0	0/0	0/1
Chromosome2-9769780	BND	PRECISE	0	0	0	0/0	0/0	0/1	0/0	0/0	0/0	0/0	0/0	0/0	0/0	0/0	0/0	0/0	0/0
Chromosome2-9774346	DEL	PRECISE	61	0	0	0/0	0/0	0/0	0/0	0/1	0/1	0/0	0/0	0/0	0/0	0/0	0/0	0/0	0/0
Chromosome2-9775655	INS	PRECISE	7993	0	0	0/0	0/0	1/1	0/0	0/0	0/0	0/0	0/0	0/0	0/0	0/0	0/0	0/0	0/0
Chromosome2-9775655	BND	PRECISE	0	0	0	0/0	0/0	0/1	0/0	0/0	0/0	0/0	0/0	0/0	0/0	0/0	0/0	0/0	0/0
Chromosome2-9776523	INS	PRECISE	137	0	0	0/0	0/0	0/0	0/0	0/1	0/1	0/0	0/0	0/0	0/0	0/0	0/0	0/0	0/0
Chromosome2-9798101	INS	PRECISE	59	0	0	0/0	0/0	0/1	0/1	0/1	0/1	0/1	0/0	0/0	0/0	0/0	0/0	0/0	1/1
Chromosome2-9800618	INS	PRECISE	214	0	0	0/0	0/0	0/0	0/1	0/0	0/0	0/0	0/0	0/0	0/0	0/0	0/0	0/0	0/0
Chromosome2-9805750	DEL	PRECISE	3574	0	0	0/0	0/0	0/1	0/1	0/1	0/1	0/0	0/0	0/0	0/0	0/0	0/0	0/0	1/1
Chromosome2-9806773	INS	PRECISE	3655	0	0	0/0	0/0	0/0	0/0	0/0	0/0	1/1	0/0	0/0	0/0	0/0	0/0	0/0	0/0
Chromosome2-9811759	DEL	PRECISE	69	0	0	0/0	0/0	0/1	0/0	0/0	0/0	0/0	0/0	0/0	0/0	0/0	0/0	0/0	0/0
Chromosome2-9817290	DEL	PRECISE	51	0	0	0/0	0/0	0/1	0/0	0/1	0/1	0/0	0/0	0/0	0/0	0/0	0/0	0/0	0/0
Chromosome2-9818711	INS	PRECISE	2221	0	0	0/0	0/0	0/1	0/0	0/0	0/0	0/0	0/0	0/0	0/0	0/0	0/0	0/0	0/0
Chromosome2-9820070	DEL	PRECISE	132	0	0	0/0	0/0	0/1	0/0	0/0	0/0	0/0	0/0	0/0	0/0	0/0	0/0	0/0	0/0
Chromosome2-9823336	INS	PRECISE	5074	0	0	0/0	0/0	1/1	0/0	0/0	0/0	0/0	0/0	0/0	0/0	0/0	0/0	0/0	0/0
Chromosome2-9823923	DEL	PRECISE	124	0	0	0/0	0/0	0/0	0/0	0/0	0/0	0/0	0/0	0/0	0/0	0/0	0/0	0/0	1/1
Chromosome2-9846897	DEL	PRECISE	542	0	0	0/0	0/0	0/1	0/1	0/0	0/0	0/0	0/0	0/0	0/0	0/0	0/0	0/0	0/0
Chromosome2-9848644	INS	PRECISE	62	0	0	0/0	0/0	0/1	0/1	0/0	0/0	0/0	0/0	0/0	0/0	0/0	0/0	0/0	0/0
Chromosome2-9849532	DEL	PRECISE	728	0	0	0/0	0/0	0/0	0/0	0/1	0/0	0/0	0/0	0/0	0/0	0/0	0/0	0/0	1/1
Chromosome2-9863806	DEL	PRECISE	2112	0	0	0/0	0/0	0/1	0/0	0/1	0/1	0/0	0/0	0/0	0/0	0/0	0/0	0/0	1/1
Chromosome2-9867632	DEL	PRECISE	381	0	0	0/0	0/0	0/1	0/0	0/0	0/0	0/0	0/0	0/0	0/0	0/0	0/0	0/0	1/1
Chromosome2-9877113	DEL	PRECISE	4383	0	0	0/0	0/0	0/0	0/1	0/0	0/0	0/0	0/0	0/0	0/0	0/0	0/0	0/0	0/0
Chromosome2-9879310	INS	PRECISE	878	0	0.707	0/0	0/0	0/0	0/0	0/1	0/1	0/0	0/0	0/0	0/0	0/0	0/0	0/0	0/0
Chromosome2-9888381	DEL	PRECISE	78	0	0	0/0	0/0	0/0	0/0	0/0	0/0	0/0	0/0	0/0	0/0	0/0	0/0	0/0	1/1
Chromosome2-9893628	DEL	PRECISE	122	0	0	0/0	0/0	0/0	0/1	0/0	0/0	0/0	0/0	0/0	0/0	0/0	0/0	0/0	0/0
Chromosome2-9894287	INS	PRECISE	1630	0	0	0/0	0/0	0/0	0/0	0/0	0/0	0/0	0/0	0/0	0/0	0/0	0/0	0/0	1/1
Chromosome2-9898227	DEL	PRECISE	156	0	0	0/0	0/0	0/0	0/0	0/0	0/0	0/0	0/0	0/0	0/0	0/0	0/0	0/0	1/1
Chromosome2-9907458	DEL	PRECISE	71	0	0	0/0	0/0	0/0	0/0	0/1	0/1	0/0	0/0	0/0	0/0	0/0	0/0	0/0	0/0
Chromosome2-9914637	INS	PRECISE	1750	0	0	0/0	0/0	0/0	0/1	0/0	0/0	0/0	0/0	0/0	0/0	0/0	0/0	0/0	0/0
Chromosome2-9916629	INS	PRECISE	67	0	0	0/0	0/0	0/0	0/0	0/0	0/0	0/0	0/0	0/0	0/0	0/0	0/0	0/0	1/1
Chromosome2-9917438	INS	PRECISE	2216	0	0	0/0	0/0	0/0	0/0	0/0	0/0	0/0	0/0	0/0	0/0	0/0	0/0	0/0	1/1
Chromosome2-9924531	INS	PRECISE	1186	0	0	0/0	0/0	0/0	0/1	0/0	0/0	0/0	0/0	0/0	0/0	0/0	0/0	0/0	0/0
Chromosome2-9925109	INS	PRECISE	134	0	0	0/0	0/0	0/1	0/0	0/0	0/0	0/0	0/0	0/0	0/0	0/0	0/0	0/0	0/0
Chromosome2-9926105	INS	PRECISE	6044	0	0	0/0	0/0	0/0	0/0	0/0	0/0	0/0	0/0	0/0	0/0	0/0	0/0	0/0	1/1
Chromosome2-9931430	INS	PRECISE	62	0	0	0/0	0/0	0/0	0/0	0/0	0/0	0/0	0/0	0/0	0/0	0/0	0/0	0/0	1/1
Chromosome2-9941996	DEL	PRECISE	1878	0	0	0/0	0/0	0/1	0/1	0/1	0/1	0/1	0/0	0/0	0/0	0/0	0/0	0/0	1/1
Chromosome2-9951849	DEL	PRECISE	382	0	0	0/0	0/0	0/1	0/0	0/0	0/0	0/0	0/0	0/0	0/0	0/0	0/0	0/0	0/0
Chromosome2-9953012	DEL	PRECISE	73	0	0	0/0	0/0	0/1	0/0	0/1	0/1	0/0	0/0	0/0	0/0	0/0	0/0	0/0	0/0
Chromosome2-9963899	INS	PRECISE	135	0	0	0/0	0/0	0/0	0/0	0/1	0/1	0/0	0/0	0/0	0/0	0/0	0/0	0/0	0/0
Chromosome2-9970075	INS	PRECISE	71	0	0	0/0	0/0	0/0	0/1	0/0	0/0	0/1	0/0	0/0	0/0	0/0	0/0	0/0	0/0
Chromosome2-9974973	DEL	PRECISE	1004	0	0	0/0	0/0	0/0	0/0	0/0	0/0	0/0	0/0	0/0	0/0	0/0	0/0	0/0	1/1
Chromosome2-9977569	INS	PRECISE	425	0	0	0/0	0/0	0/1	0/0	0/0	0/0	0/0	0/0	0/0	0/0	0/0	0/0	0/0	0/0
Chromosome2-9985257	DEL	PRECISE	1391	0	0	0/0	0/0	0/0	0/0	0/1	0/1	0/0	0/0	0/0	0/1	1/1	0/0	0/0	1/1
Chromosome2-1000744	INS	PRECISE	5502	0	0	0/0	0/0	0/0	0/0	0/0	0/0	0/0	0/0	0/0	0/0	0/0	0/0	0/0	1/1
Chromosome2-1000744	INS	PRECISE	10416	0	0	0/0	0/0	1/1	0/0	0/0	0/0	0/0	0/0	0/0	0/0	0/0	0/0	0/0	0/0
Chromosome2-1001299	DEL	PRECISE	122	0	0	0/0	0/0	0/0	0/0	0/0	0/0	0/0	0/0	0/0	0/0	0/0	0/0	0/0	1/1
Chromosome2-1001318	INS	PRECISE	58	0	0	0/0	0/0	0/1	0/0	0/0	0/0	0/0	0/0	0/0	0/0	0/0	0/0	0/0	1/1
Chromosome2-1001619	INS	PRECISE	63	0	0	0/0	0/0	0/0	0/0	0/1	0/1	0/0	0/0	0/0	0/0	0/0	0/0	0/0	0/0
Chromosome2-1002745	DEL	PRECISE	885	0	0	0/0	0/0	0/1	0/0	0/1	0/1	0/1	0/0	0/0	0/0	0/0	0/0	0/0	0/0
Chromosome2-1003853	DEL	PRECISE	229	0	0	0/0	0/0	0/0	0/0	0/0	0/0	0/0	0/0	0/0	0/0	0/0	0/0	0/0	1/1
Chromosome2-1004024	DEL	PRECISE	5212	0	0	0/0	0/0	0/1	0/1	0/1	0/1	0/0	0/0	0/0	0/0	0/0	0/0	0/0	1/1

Chromosome2-1004888	INS	PRECISE	5426	0	0	0/0	0/0	0/0	0/1	0/0	0/0	0/0	0/0	0/0	0/0	0/0	0/0	0/0	0/0
Chromosome2-1005099	BND	PRECISE	0	0	0	0/0	0/0	0/0	0/0	0/0	0/0	0/0	0/0	0/0	0/0	0/0	0/0	0/0	0/1
Chromosome2-1006179	DEL	PRECISE	891	0	0	0/0	0/0	0/1	0/0	0/0	0/0	0/0	0/0	0/0	0/0	0/0	0/0	0/0	0/0
Chromosome2-1007724	INS	PRECISE	59	0	0	0/0	0/0	0/1	0/0	0/0	0/0	0/0	0/0	0/0	0/0	0/0	0/0	0/0	0/0
Chromosome2-1007935	INS	PRECISE	3533	0	0	0/0	0/0	0/1	0/0	0/0	0/0	0/0	0/0	0/0	0/0	0/0	0/0	0/0	0/0
Chromosome2-1008263	DEL	PRECISE	5342	0	0	0/0	0/0	0/1	0/1	0/0	0/0	0/0	0/0	0/0	0/0	0/0	0/0	0/0	1/1
Chromosome2-1009257	INS	PRECISE	59	0	0	0/0	0/0	0/1	0/0	0/0	0/0	0/0	0/0	0/0	0/0	0/0	0/0	0/0	0/0
Chromosome2-1009677	DEL	PRECISE	3203	0	0	0/0	0/0	0/0	0/1	0/0	0/0	0/0	0/0	0/0	0/0	0/0	0/0	0/0	1/1
Chromosome2-1010018	INS	PRECISE	64	0	0	0/0	0/0	0/1	0/1	0/0	0/0	0/0	0/0	0/0	0/0	0/0	0/0	0/0	1/1
Chromosome2-1010642	DEL	PRECISE	421	0	0	0/0	0/0	0/1	0/0	0/0	0/0	0/0	0/0	0/0	0/0	0/0	0/0	0/0	0/0
Chromosome2-1010855	INS	PRECISE	8119	0	0	0/0	0/0	0/1	0/0	0/0	0/0	0/0	0/0	0/0	0/0	0/0	0/0	0/0	0/0
Chromosome2-1011191	INS	PRECISE	952	0	0	0/0	0/0	0/1	0/0	0/0	0/0	0/0	0/0	0/0	0/0	0/0	0/0	0/0	0/0
Chromosome2-1011461	INS	PRECISE	9607	0	0	0/0	0/0	0/0	0/0	0/0	0/0	0/0	0/0	0/0	0/0	0/0	0/0	0/0	1/1
Chromosome2-1011500	DEL	PRECISE	59	0	0	0/0	0/0	0/0	0/1	0/0	0/0	0/0	0/0	0/0	0/0	0/0	0/0	0/0	0/0
Chromosome2-1011593	INS	PRECISE	1403	0	0	0/0	0/0	0/0	0/1	0/0	0/0	0/0	0/0	0/0	0/0	0/0	0/0	0/0	0/0
Chromosome2-1013030	INS	PRECISE	5402	0	0	0/0	0/0	1/1	0/0	0/0	0/0	0/0	0/0	0/0	0/0	0/0	0/0	0/0	0/0
Chromosome2-1013386	DEL	PRECISE	642	0	0	0/0	0/0	0/1	0/1	0/0	0/0	0/0	0/0	0/0	0/0	0/0	0/0	0/0	1/1
Chromosome2-1013799	DEL	PRECISE	488	0	0	0/0	0/0	0/1	0/0	0/0	0/0	0/0	0/0	0/0	0/0	0/0	0/0	0/0	0/0
Chromosome2-1015389	INS	PRECISE	2603	0	0	0/0	0/0	0/0	0/0	0/0	0/0	0/0	0/0	0/1	0/0	0/0	0/0	0/0	0/0
Chromosome2-1016126	DEL	PRECISE	273	0	0	0/0	0/0	0/0	0/0	0/0	0/0	0/0	0/0	0/0	0/0	0/0	0/0	0/0	1/1
Chromosome2-1016335	INS	PRECISE	870	0	0	0/0	0/0	0/0	0/0	0/0	0/0	0/0	0/0	0/0	0/0	0/0	0/0	0/0	1/1
Chromosome2-1016448	DEL	PRECISE	959	0	0	0/0	0/0	0/1	0/0	0/1	0/1	0/1	0/0	0/1	0/0	0/0	1/1	1/1	1/1
Chromosome2-1017906	DEL	PRECISE	869	0	0	0/0	0/0	0/1	0/1	0/0	0/0	0/0	0/0	0/0	0/0	0/0	0/0	0/0	0/0
Chromosome2-1018053	DEL	PRECISE	175	0	0	0/0	0/0	0/1	0/1	0/0	0/0	0/0	0/0	0/0	0/0	0/0	0/0	0/0	0/0
Chromosome2-1018055	INS	PRECISE	129	0	0	0/0	0/0	0/0	0/0	0/0	0/0	0/1	0/0	0/0	0/0	0/0	0/0	0/0	0/0
Chromosome2-1019372	INS	PRECISE	1549	0	0	0/0	0/0	0/0	0/0	0/0	0/0	0/1	0/0	0/0	0/0	0/0	0/0	0/0	0/0
Chromosome2-1019384	INS	PRECISE	10089	0	0	0/0	0/0	0/0	0/0	0/0	0/0	0/0	0/0	0/0	0/0	0/0	0/0	0/0	1/1
Chromosome2-1019385	BND	PRECISE	0	0	0	0/0	0/0	0/0	0/0	0/0	0/0	0/0	0/0	0/0	0/0	0/0	0/0	0/0	0/1
Chromosome2-1020726	DEL	PRECISE	53	0	0	0/0	0/0	0/1	0/0	0/0	0/0	0/0	0/0	0/0	0/0	0/0	0/0	0/0	0/0
Chromosome2-1020992	DEL	PRECISE	122	0	0	0/0	0/0	0/0	0/0	0/1	0/1	0/0	0/0	0/0	0/0	0/0	0/0	0/0	0/0
Chromosome2-1022382	DEL	PRECISE	60	0	0	0/0	0/0	0/0	0/0	0/0	0/0	0/0	0/0	0/0	0/0	0/0	0/0	0/0	1/1
Chromosome2-1023336	INS	PRECISE	54	0	0	0/0	0/0	0/1	0/0	0/0	0/0	0/0	0/0	0/0	0/0	0/0	0/0	0/0	0/0
Chromosome2-1023780	INS	PRECISE	867	0	0	0/0	0/0	0/1	0/0	0/0	0/0	0/0	0/0	0/0	0/0	0/0	0/0	0/0	0/0
Chromosome2-1024124	INS	PRECISE	658	0	0	0/0	0/0	0/0	0/0	0/0	0/0	0/1	0/0	0/0	0/0	0/0	0/0	0/0	0/0
Chromosome2-1025176	INS	PRECISE	138	0	0	0/0	0/0	0/0	0/0	0/0	0/0	0/0	0/0	0/0	0/0	0/0	0/0	0/0	1/1
Chromosome2-1025310	DEL	PRECISE	3049	0	0	0/0	0/0	0/0	0/0	0/0	0/0	0/0	0/0	0/0	0/0	0/0	0/0	0/0	1/1
Chromosome2-1026941	INS	PRECISE	212	0	0	0/0	0/0	0/0	0/0	0/0	0/0	0/0	0/0	0/0	0/0	0/0	0/0	0/0	1/1
Chromosome2-1027836	INS	PRECISE	477	0	0	0/0	0/0	0/0	0/0	0/0	0/0	0/1	0/0	0/0	0/0	0/0	0/0	0/0	0/0
Chromosome2-1028167	INS	PRECISE	6001	0	0	0/0	0/0	0/0	0/1	0/0	0/0	0/0	0/0	0/0	0/0	0/0	0/0	0/0	0/0
Chromosome2-1028462	INS	PRECISE	6581	0	0.707	0/0	0/0	0/0	0/0	0/1	0/1	0/0	0/0	0/0	0/0	0/0	0/0	0/0	0/0
Chromosome2-1028814	DEL	PRECISE	307	0	0	0/0	0/0	0/0	0/0	0/0	0/0	0/1	0/0	0/0	0/0	0/0	0/0	0/0	0/0
Chromosome2-1029004	INS	PRECISE	448	0	0	0/0	0/0	0/0	0/0	0/0	0/0	0/1	0/0	0/0	0/0	0/0	0/0	0/0	0/0
Chromosome2-1030090	INS	PRECISE	623	0	0	0/0	0/0	0/0	0/1	0/0	0/0	0/0	0/0	0/0	0/0	0/0	0/0	0/0	0/0
Chromosome2-1030289	INS	PRECISE	7036	0	0	0/0	0/0	0/1	0/0	0/0	0/0	0/0	0/0	0/0	0/0	0/0	0/0	0/0	0/0
Chromosome2-1030290	BND	PRECISE	0	0	0	0/0	0/0	0/0	0/0	0/1	0/1	0/0	0/0	0/0	0/0	0/0	0/0	0/0	0/0
Chromosome2-1030494	INS	PRECISE	131	0	0	0/0	0/0	0/0	0/0	0/0	0/0	0/0	0/0	0/0	0/0	0/0	0/0	0/0	1/1
Chromosome2-1031199	DEL	PRECISE	99	0	0	0/0	0/0	0/0	0/1	0/1	0/1	0/0	0/0	0/0	0/0	0/0	0/0	0/0	0/0
Chromosome2-1031818	INS	PRECISE	925	0	0	0/0	0/0	0/1	0/0	0/0	0/0	0/0	0/0	0/0	0/0	0/0	0/0	0/0	0/0
Chromosome2-1032011	DEL	PRECISE	70	0	0	0/0	0/0	0/0	0/0	0/1	0/1	0/0	0/0	0/0	0/0	0/0	0/0	0/0	0/0
Chromosome2-1033520	INS	PRECISE	995	0	0	0/0	0/0	0/0	0/0	0/0	0/0	0/0	0/0	0/0	0/0	0/0	0/0	0/0	1/1
Chromosome2-1033759	INS	PRECISE	6547	0	0	0/0	0/0	0/1	0/0	0/0	0/0	0/0	0/0	0/0	0/0	0/0	0/0	0/0	0/0
Chromosome2-1033844	INS	PRECISE	890	0	0	0/0	0/0	0/1	0/0	0/0	0/0	0/0	0/0	0/0	0/0	0/0	0/0	0/0	0/0
Chromosome2-1034119	INS	PRECISE	86	0	0	0/0	0/0	0/0	0/1	0/1	0/1	0/1	0/0	0/0	0/0	0/0	0/0	0/0	1/1
Chromosome2-1034301	DEL	PRECISE	5291	0	0	0/0	0/0	0/1	0/0	0/0	0/0	0/0	0/0	0/0	0/0	0/0	0/0	0/0	0/0
Chromosome2-1035123	INS	PRECISE	1352	0	0	0/0	0/0	0/1	0/0	0/0	0/0	0/0	0/0	0/0	0/0	0/0	0/0	0/0	0/0
Chromosome2-1035230	INS	PRECISE	6101	0	0	0/0	0/0	0/0	1/1	0/0	0/0	0/0	0/0	0/0	0/0	0/0	0/0	0/0	0/0
Chromosome2-1036244	DEL	PRECISE	624	0	0	0/0	0/0	0/1	0/0	0/1	1/1	0/0	0/0	0/0	0/0	0/0	0/0	0/0	1/1
Chromosome2-1036994	INS	PRECISE	739	0	0	0/0	0/0	0/0	0/0	0/0	0/0	0/0	0/0	0/0	0/0	0/0	0/0	0/0	1/1
Chromosome2-1037077	DEL	PRECISE	51	0	0	0/0	0/0	0/0	0/0	0/0	0/0	0/0	0/0	0/0	0/0	0/0	0/0	0/0	1/1
Chromosome2-1037257	INS	PRECISE	120	0	0	0/0	0/0	0/0	0/0	0/0	0/0	0/0	0/0	0/0	0/0	0/0	0/0	0/0	1/1
Chromosome2-1038160	DEL	PRECISE	6313	0	0	0/0	0/0	0/0	0/0	0/0	0/0	0/0	0/0	0/0	0/0	1/1	1/1	1/1	0/0
Chromosome2-1040267	INS	PRECISE	1403	0	0	0/0	0/0	0/0	0/0	0/0	0/0	0/0	0/0	0/0	0/0	0/0	0/0	0/0	1/1
Chromosome2-1040404	INS	PRECISE	213	0	0.577	0/0	0/0	0/0	0/0	0/0	0/0	0/0	0/0	0/1	0/0	1/1	1/1	1/1	0/0
Chromosome2-1041206	DEL	PRECISE	568	0	0	0/0	0/0	0/0	0/0	0/0	0/0	0/0	0/0	0/0	0/0	0/0	0/0	0/0	1/1
Chromosome2-1041636	DEL	PRECISE	69	0	0	0/0	0/0	0/1	0/0	0/1	0/1	0/0	0/0	0/0	0/0	0/0	0/0	0/0	0/0
Chromosome2-1041696	DEL	PRECISE	1424	0	0	0/0	0/0	0/1	0/0	0/1	0/1	0/0	0/0	0/1	0/0	1/1	1/1	1/1	1/1
Chromosome2-1041840	DEL	PRECISE	87	0	0	0/0	0/0	0/0	0/0	0/0	0/0	0/1	0/0	0/0	0/0	0/0	0/0	0/0	0/0
Chromosome2-1042025	DEL	PRECISE	415	0	0	0/0	0/0	0/0	0/0	0/0	0/0	0/0	0/0	0/0	0/0	1/1	0/0	0/0	0/0
Chromosome2-1042473	DEL	PRECISE	2051	0	0	0/0	0/0	0/0	0/0	0/0	0/0	0/0	0/0	0/1	0/0	1/1	1/1	1/1	0/0
Chromosome2-1043065	DEL	PRECISE	10476603	0	0	0/0	0/0	0/0	0/0	0/0	0/0	0/0	0/0	1/1	0/0	1/1	0/1	0/1	0/1
Chromosome2-1045603	INS	PRECISE	329	0	0.5	0/0	0/0	0/0	0/0	0/0	0/0	0/0	0/0	0/1	0/0	1/1	1/1	1/1	0/0
Chromosome2-1045760	INS	PRECISE	377	0	0	0/0	0/0	0/0	0/0	0/0	0/0	0/0	0/0	0/0	0/0	0/0	0/0	0/0	1/1
Chromosome2-1047023	DEL	PRECISE	70	0	0	0/0	0/0	0/1	0/0	0/0	0/0	0/0	0/0	0/0	0/0	0/0	0/0	0/0	0/0

Chromosome2-1048291	INS	PRECISE	1650	0	0	0/0	0/0	0/0	0/0	0/0	0/0	0/0	0/0	0/0	0/0	0/0	0/0	0/0	1/1
Chromosome2-1048409	DEL	PRECISE	10135	0	0	0/0	0/0	0/1	0/0	0/1	0/1	0/0	0/0	0/0	0/0	0/0	0/0	0/0	0/0
Chromosome2-1048453	INS	PRECISE	132	0	0	0/0	0/0	0/0	0/1	0/0	0/0	0/0	0/0	0/0	0/0	0/0	0/0	0/0	0/0
Chromosome2-1049864	BND	PRECISE	0	0	0	0/0	0/0	0/1	0/0	0/0	0/0	0/0	0/0	0/0	0/0	0/0	0/0	0/0	0/0
Chromosome2-1049940	INS	PRECISE	828	0	0	0/0	1/1	0/0	0/0	0/0	0/0	0/0	0/0	0/0	0/0	0/0	0/0	0/0	0/0
Chromosome2-1050056	INS	PRECISE	7395	0	0	0/0	0/0	1/1	0/0	0/0	0/0	0/0	0/0	0/0	0/0	0/0	0/0	0/0	0/0
Chromosome2-1051176	INS	PRECISE	4364	0	0.707	0/0	0/0	0/0	0/0	0/1	1/1	0/0	0/0	0/0	0/0	0/0	0/0	0/0	0/0
Chromosome2-1051352	DEL	PRECISE	426	0	0	0/0	0/0	0/1	0/0	0/1	0/1	0/0	0/0	0/0	0/0	0/0	0/0	0/0	1/1
Chromosome2-1051844	INS	PRECISE	6824	0	0	0/0	0/0	0/0	0/0	0/0	0/0	0/0	0/0	0/0	0/0	0/0	0/0	0/0	1/1
Chromosome2-1051844	BND	PRECISE	0	0	0	0/0	0/0	0/0	0/0	0/0	0/0	0/0	0/0	0/0	0/0	0/0	0/0	0/0	0/1
Chromosome2-1052356	DEL	PRECISE	52	0	0	0/0	0/0	0/0	0/0	0/0	0/0	0/0	0/0	0/0	0/0	0/0	0/0	0/0	1/1
Chromosome2-1052411	DEL	PRECISE	1747	0	0	0/0	0/0	0/0	0/0	0/1	0/1	0/0	0/0	0/0	0/0	0/0	0/0	0/0	0/0
Chromosome2-1052538	DEL	PRECISE	157	0	0	0/0	0/0	0/0	0/0	0/0	0/0	0/0	0/0	0/0	0/0	0/0	0/0	0/0	1/1
Chromosome2-1052597	INS	PRECISE	1365	0	0	0/0	0/0	0/0	0/0	0/0	0/0	0/1	0/0	0/0	0/0	0/0	0/0	0/0	0/0
Chromosome2-1052597	INS	PRECISE	12455	0	0	0/0	0/0	0/0	0/1	0/0	0/0	0/0	0/0	0/0	0/0	0/0	0/0	0/0	0/0
Chromosome2-1053648	DEL	PRECISE	1558	0	0	0/0	0/0	0/0	0/0	0/0	0/0	0/1	0/0	0/0	0/0	0/0	0/0	0/0	0/0
Chromosome2-1054740	INS	PRECISE	1463	0	0	0/0	0/0	0/0	0/0	0/0	0/0	0/0	0/0	0/0	0/0	0/0	0/0	0/0	1/1
Chromosome2-1055245	DEL	PRECISE	161	0	0	0/0	0/0	0/0	0/0	0/1	0/1	0/0	0/0	0/0	0/0	0/0	0/0	0/0	0/0
Chromosome2-1055560	DEL	PRECISE	415	0	0	0/0	0/0	0/1	0/0	0/1	0/1	0/0	0/0	0/0	0/0	0/0	0/0	0/0	1/1
Chromosome2-1055900	DEL	PRECISE	62	0	0	0/0	0/0	0/0	0/1	0/0	0/0	0/0	0/0	0/0	0/0	0/0	0/0	0/0	0/0
Chromosome2-1057074	INS	PRECISE	55	0	0	0/0	0/0	0/1	0/0	0/0	0/0	0/0	0/0	0/0	0/0	0/0	0/0	0/0	0/0
Chromosome2-1057096	INS	PRECISE	8221	0	0	0/0	0/0	0/0	1/1	0/0	0/0	0/0	0/0	0/0	0/0	0/0	0/0	0/0	0/0
Chromosome2-1057618	INS	PRECISE	292	0	0	0/0	0/0	0/1	0/0	0/0	0/0	0/0	0/0	0/0	0/0	0/0	0/0	0/0	0/0
Chromosome2-1059747	INS	PRECISE	661	0	0	0/0	0/0	0/0	0/0	0/0	0/0	0/0	0/0	0/0	0/0	0/0	0/0	0/0	1/1
Chromosome2-1060106	INS	PRECISE	2511	0.707	0.707	0/0	0/0	0/0	0/0	0/0	0/0	1/1	0/0	0/0	0/0	0/0	0/0	0/0	1/1
Chromosome2-1060552	INS	PRECISE	366	0	0	0/0	0/0	0/0	0/1	0/0	0/0	0/0	0/0	0/0	0/0	0/0	0/0	0/0	0/0
Chromosome2-1061427	INS	PRECISE	648	0	0	0/0	0/0	0/0	1/1	0/0	0/0	0/0	0/0	0/0	0/0	0/0	0/0	0/0	0/0
Chromosome2-1061673	DEL	PRECISE	741	0	0	0/0	0/0	1/1	0/0	1/1	1/1	0/0	0/0	0/0	0/0	0/0	0/0	0/0	0/0
Chromosome2-1061757	DEL	PRECISE	371	0	0	0/0	0/0	0/0	0/0	0/0	0/0	0/1	0/0	0/0	0/0	0/0	0/0	0/0	0/0
Chromosome2-1062365	DEL	PRECISE	885	0	0	0/0	0/0	0/1	1/1	0/1	0/1	0/1	0/0	0/0	0/0	0/0	0/0	0/0	1/1
Chromosome2-1062727	INS	PRECISE	1017	0	0	0/0	0/0	0/0	1/1	0/0	0/0	0/0	0/0	0/0	0/0	0/0	0/0	0/0	0/0
Chromosome2-1062790	DEL	PRECISE	66	0	0	0/0	0/0	0/0	0/0	0/1	0/1	0/0	0/0	0/0	0/0	0/0	0/0	0/0	0/0
Chromosome2-1063225	INS	PRECISE	861	0	0	0/0	0/0	0/0	0/1	0/0	0/0	0/0	0/0	0/0	0/0	0/0	0/0	0/0	0/0
Chromosome2-1063772	DEL	PRECISE	805	0	0	0/0	0/0	0/1	0/1	0/1	0/1	0/0	0/0	0/0	0/0	0/0	0/0	0/0	1/1
Chromosome2-1064245	INS	PRECISE	62	0	0	0/0	0/0	0/1	0/0	0/0	0/0	0/0	0/0	0/0	0/0	0/0	0/0	0/0	0/0
Chromosome2-1064384	DEL	PRECISE	82	0	0	0/0	0/0	0/1	0/1	0/1	0/1	0/0	0/0	0/0	0/0	0/0	0/0	0/0	1/1
Chromosome2-1064917	INS	PRECISE	52	0	0	0/0	0/0	0/0	0/1	0/0	0/0	0/0	0/0	0/0	0/0	0/0	0/0	0/0	0/0
Chromosome2-1065814	INS	PRECISE	8425	0	0	0/0	0/0	0/0	0/0	1/1	0/0	0/0	0/0	0/0	0/0	0/0	0/0	0/0	0/0
Chromosome2-1067146	DEL	PRECISE	356	0	0	0/0	0/0	0/0	0/0	0/0	0/0	0/0	0/0	0/0	0/0	0/0	1/1	0/0	0/0
Chromosome2-1068845	DEL	PRECISE	445	0	0	0/0	0/0	0/1	0/0	0/0	0/0	0/0	0/0	0/0	0/0	0/0	0/0	0/0	0/0
Chromosome2-1069450	DEL	PRECISE	715	0	0	0/0	0/0	0/0	0/0	0/1	0/1	0/0	0/0	0/0	0/0	0/0	0/0	0/0	0/0
Chromosome2-1070814	DEL	PRECISE	6242	0.408	0.408	0/0	0/0	0/1	0/0	0/1	0/1	0/0	0/0	0/0	0/1	0/0	1/1	0/0	1/1
Chromosome2-1071169	DEL	PRECISE	136	0	0	0/0	0/0	0/0	0/1	0/0	0/0	0/0	0/0	0/0	0/0	0/0	0/0	0/0	0/0
Chromosome2-1071986	INS	PRECISE	1498	0	0	0/0	0/0	0/0	0/0	0/0	0/0	0/0	0/0	0/0	0/0	0/0	0/0	0/0	1/1
Chromosome2-1073202	INS	PRECISE	4677	0	0	0/0	0/0	1/1	0/0	0/0	0/0	0/0	0/0	0/0	0/0	0/0	0/0	0/0	0/0
Chromosome2-1073202	BND	PRECISE	0	0	0	0/0	0/0	0/1	0/0	0/0	0/0	0/0	0/0	0/0	0/0	0/0	0/0	0/0	0/0
Chromosome2-1073306	INV	PRECISE	2910	0	0	0/0	0/0	0/1	0/0	0/1	0/1	0/0	0/0	0/0	0/1	0/0	1/1	0/0	1/1
Chromosome2-1073690	DEL	PRECISE	4894	0	0	0/0	0/0	0/1	0/0	0/1	0/1	0/0	0/0	0/0	0/1	0/0	1/1	0/0	1/1
Chromosome2-1074890	INS	PRECISE	53	0	0	0/0	0/0	0/1	0/0	0/1	0/1	0/0	0/0	0/0	0/1	1/1	1/1	0/0	1/1
Chromosome2-1075476	DEL	PRECISE	72	0	0	0/0	0/0	0/0	0/0	0/0	0/0	0/0	0/0	0/0	0/0	0/0	0/0	0/0	1/1
Chromosome2-1075615	INS	PRECISE	194	0	0	0/0	0/0	0/0	0/0	0/0	0/0	0/0	0/0	0/0	0/0	0/0	0/0	0/0	1/1
Chromosome2-1075638	DEL	PRECISE	50	0	0	0/0	0/0	0/0	0/0	0/0	0/0	0/0	0/0	0/0	0/1	0/0	0/1	0/0	0/0
Chromosome2-1075697	INS	PRECISE	4294	0	0	0/0	0/0	0/0	0/0	0/0	0/0	0/0	0/0	0/0	0/0	0/0	0/0	0/0	1/1
Chromosome2-1075928	INS	PRECISE	6294	0	0	0/0	0/0	0/0	0/0	0/0	0/0	0/0	0/0	0/0	0/0	0/0	0/0	0/0	1/1
Chromosome2-1076551	DEL	PRECISE	57	0	0	0/0	0/0	0/0	0/0	0/0	0/0	0/0	0/0	0/0	0/0	0/0	0/0	0/0	1/1
Chromosome2-1076778	INS	PRECISE	1277	0	0	0/0	0/0	0/0	0/0	0/0	0/0	0/0	0/0	0/0	0/0	0/0	0/0	0/0	1/1
Chromosome2-1076931	DEL	PRECISE	622	0	0	0/0	0/0	0/0	0/0	0/0	0/0	0/0	0/0	0/1	0/0	1/1	1/1	1/1	0/0
Chromosome2-1077022	DEL	PRECISE	69	0	0	0/0	0/0	0/0	0/0	0/0	0/0	0/0	0/0	0/0	0/0	1/1	0/0	0/0	0/0
Chromosome2-1077035	DEL	PRECISE	993	0	0	0/0	0/0	0/0	0/0	0/0	0/0	0/0	0/0	0/0	0/0	1/1	0/0	0/0	0/0
Chromosome2-1078578	DEL	PRECISE	689	0	0	0/0	0/0	0/0	0/0	0/0	0/0	0/0	0/0	0/0	0/0	1/1	0/0	0/0	0/0
Chromosome2-1079356	INS	PRECISE	7224	0	0	0/0	0/0	0/0	0/0	0/0	0/0	0/0	0/0	0/0	0/0	0/0	0/0	0/0	1/1
Chromosome2-1080176	DEL	PRECISE	120	0	0	0/0	0/0	0/1	0/0	0/0	0/0	0/0	0/0	0/0	0/0	0/0	0/0	0/0	0/0
Chromosome2-1080989	DEL	PRECISE	1182	0.894	0.894	0/0	0/0	0/0	0/0	0/0	0/1	0/0	0/0	0/0	0/0	0/0	0/0	0/0	1/1
Chromosome2-1081576	DUP	PRECISE	28792906	0	0	0/0	0/0	0/0	0/0	0/0	0/0	0/0	0/0	0/0	0/0	0/0	0/0	0/1	0/0
Chromosome2-1082556	DEL	PRECISE	287	0	0	0/0	0/0	0/1	0/1	0/0	0/1	0/0	0/0	0/0	0/0	0/0	0/0	0/0	1/1
Chromosome2-1083027	INS	PRECISE	6866	0	0	0/0	0/0	0/0	1/1	0/0	0/0	0/0	0/0	0/0	0/0	0/0	0/0	0/0	0/0
Chromosome2-1083107	INS	PRECISE	121	0	0	0/0	0/0	0/0	0/0	0/0	0/0	0/0	0/0	0/0	0/0	0/0	0/0	0/0	1/1
Chromosome2-1083897	INS	PRECISE	854	0	0	0/0	0/0	0/0	0/0	0/0	0/0	0/0	0/0	0/0	0/0	0/0	0/0	0/0	1/1
Chromosome2-1084186	INS	PRECISE	13317	0	0	0/0	0/0	1/1	0/0	0/0	0/0	0/0	0/0	0/0	0/0	0/0	0/0	0/0	0/0
Chromosome2-1084186	INS	PRECISE	3219	0	0	0/0	0/0	0/1	0/0	0/0	0/0	0/0	0/0	0/0	0/0	0/0	0/0	0/0	0/0
Chromosome2-1084614	DEL	PRECISE	1375	0	0	0/0	0/0	0/0	0/0	0/0	0/0	0/0	0/0	0/0	0/0	0/0	0/0	0/0	1/1
Chromosome2-1084785	INS	PRECISE	12290	0	0	0/0	0/0	0/0	0/0	0/0	0/0	1/1	0/0	0/0	0/0	0/0	0/0	0/0	0/0
Chromosome2-1084785	BND	PRECISE	0	0	0	0/0	0/0	0/0	0/0	0/0	0/0	0/1	0/0	0/0	0/0	0/0	0/0	0/0	0/0

Chromosome2-1084948	INS	PRECISE	1112	0	0	0/0	0/0	0/0	0/0	0/0	0/0	0/0	0/0	0/0	0/0	0/0	0/0	0/0	1/1
Chromosome2-1085931	INS	PRECISE	549	0	0	0/0	0/0	0/0	1/1	0/0	0/0	0/0	0/0	0/0	0/0	0/0	0/0	0/0	0/0
Chromosome2-1086067	INS	PRECISE	150	0	0	0/0	0/0	0/1	0/0	0/0	0/0	0/0	0/0	0/0	0/0	0/0	0/0	0/0	0/0
Chromosome2-1086991	DEL	PRECISE	51	0	0	0/0	0/0	0/0	0/0	0/0	0/0	0/0	0/0	0/0	0/0	0/0	0/0	0/0	1/1
Chromosome2-1088641	INS	PRECISE	2467	0	0	0/0	0/0	0/0	0/0	0/0	0/0	0/0	0/0	0/0	0/0	0/0	0/0	0/0	1/1
Chromosome2-1089461	INS	PRECISE	135	0	0	0/0	0/0	0/0	0/0	0/0	0/0	0/1	0/0	0/0	0/0	0/0	0/0	0/0	0/0
Chromosome2-1089668	DEL	PRECISE	173	0	0	0/0	0/0	0/0	0/0	0/0	0/0	0/0	0/0	0/0	0/0	0/0	0/0	0/0	1/1
Chromosome2-1089762	DEL	PRECISE	532	0	0	0/0	0/0	0/0	0/0	0/0	0/0	0/0	0/0	0/0	0/0	0/0	0/0	0/0	1/1
Chromosome2-1090001	DEL	PRECISE	2767	0	0	0/0	0/0	0/0	0/0	0/0	0/0	0/0	0/0	0/0	0/0	0/0	0/0	0/0	1/1
Chromosome2-1090759	DEL	PRECISE	168	0	0	0/0	0/0	0/0	0/0	0/0	0/0	0/0	0/0	0/0	0/0	0/0	0/0	0/0	1/1
Chromosome2-1091117	INS	PRECISE	54	0	0	0/0	0/0	0/0	0/0	0/0	0/0	0/0	0/0	0/0	0/0	0/0	0/0	0/0	1/1
Chromosome2-1091726	DEL	PRECISE	11667	0	0	0/0	0/0	0/1	0/1	0/1	0/1	0/1	0/0	0/0	0/0	0/0	0/0	0/0	1/1
Chromosome2-1093025	DEL	PRECISE	80	0	0	0/0	0/0	0/0	0/0	0/0	0/0	0/0	0/0	0/0	0/0	0/0	0/0	0/0	1/1
Chromosome2-1093576	INS	PRECISE	62	0	0	0/0	0/0	0/0	0/0	0/0	0/0	0/1	0/0	0/0	0/0	0/0	0/0	0/0	0/0
Chromosome2-1095791	INS	PRECISE	211	0	0	0/0	0/0	0/0	0/1	0/0	0/0	0/0	0/0	0/0	0/0	0/0	0/0	0/0	0/0
Chromosome2-1096007	DEL	PRECISE	58	0	0	0/0	0/0	0/0	0/0	0/1	0/1	0/0	0/0	0/0	0/0	0/0	0/0	0/0	0/0
Chromosome2-1096640	INS	PRECISE	66	0	0	0/0	0/0	0/0	0/0	0/0	0/0	0/0	0/0	0/0	0/0	0/0	0/0	0/0	1/1
Chromosome2-1097270	INS	PRECISE	1036	0	0	0/0	0/0	0/1	0/0	0/0	0/0	0/0	0/0	0/0	0/0	0/0	0/0	0/0	0/0
Chromosome2-1097316	DEL	PRECISE	187	0	0	0/0	0/0	0/0	0/0	0/0	0/0	0/0	0/0	0/0	0/0	0/0	0/0	0/0	1/1
Chromosome2-1097559	INS	PRECISE	652	0	0	0/0	0/0	0/1	0/0	0/0	0/0	0/0	0/0	0/0	0/0	0/0	0/0	0/0	0/0
Chromosome2-1098070	INS	PRECISE	95	0	0	0/0	0/0	0/1	0/0	0/0	0/0	0/0	0/0	0/0	0/0	0/0	0/0	0/0	0/0
Chromosome2-1098543	DEL	PRECISE	1664	0	0	0/0	0/0	0/0	0/0	0/0	0/0	0/0	0/0	0/0	0/0	0/0	0/0	0/0	1/1
Chromosome2-1100635	INS	PRECISE	2282	0	0	0/0	0/0	0/0	0/0	0/0	0/0	0/1	0/0	0/0	0/0	0/0	0/0	0/0	0/0
Chromosome2-1101620	DEL	PRECISE	657	0	0	0/0	0/0	0/1	0/1	0/1	0/1	0/1	0/0	0/0	0/0	0/0	0/0	0/0	1/1
Chromosome2-1102121	INS	PRECISE	6715	0	0	0/0	0/0	0/0	0/0	0/0	0/0	1/1	0/0	0/0	0/0	0/0	0/0	0/0	0/0
Chromosome2-1102783	DEL	PRECISE	1340	0	0	0/0	0/0	0/1	0/0	0/1	0/1	0/0	0/0	0/0	0/0	0/0	0/0	0/0	1/1
Chromosome2-1103286	DEL	PRECISE	324	0	0	0/0	0/0	0/1	0/0	0/0	0/0	0/0	0/0	0/0	0/0	0/0	0/0	0/0	0/0
Chromosome2-1103353	DEL	PRECISE	5433	0	0	0/0	0/0	0/0	0/1	0/0	0/0	0/0	0/0	0/0	0/0	0/0	0/0	0/0	0/0
Chromosome2-1103748	BND	PRECISE	0	0	0	0/0	0/0	0/0	0/0	0/1	1/1	0/0	0/0	0/0	0/0	0/0	0/0	0/0	0/1
Chromosome2-1103785	INS	PRECISE	247	0	0.707	0/0	0/0	0/0	0/0	0/1	0/1	0/0	0/0	0/0	0/0	0/0	0/0	0/0	0/0
Chromosome2-1104333	INS	PRECISE	102	0	0	0/0	0/0	0/1	0/0	0/0	0/0	0/0	0/0	0/0	0/0	0/0	0/0	0/0	0/0
Chromosome2-1104603	INS	PRECISE	4005	0	0	0/0	0/0	0/0	0/0	0/0	0/0	0/0	0/0	0/0	0/0	0/0	0/0	0/0	1/1
Chromosome2-1104647	DEL	PRECISE	151	0	0	0/0	0/0	0/0	0/0	0/1	0/1	0/0	0/0	0/0	0/0	0/0	0/0	0/0	0/0
Chromosome2-1104734	INS	PRECISE	629	0	0	0/0	0/0	0/1	0/0	0/0	0/0	0/0	0/0	0/0	0/0	0/0	0/0	0/0	0/0
Chromosome2-1106658	DEL	PRECISE	285	0	0	0/0	0/0	0/0	0/0	0/0	0/0	0/0	0/0	0/0	0/0	0/0	0/0	0/0	0/1
Chromosome2-1106875	DEL	PRECISE	190	0	0	0/0	0/0	0/0	0/0	0/0	0/0	0/0	0/0	0/0	0/0	0/0	0/0	0/0	0/1
Chromosome2-1106959	INS	PRECISE	148	0	0	0/0	0/0	0/0	0/0	0/0	0/0	0/0	0/0	0/0	0/0	0/0	0/0	0/0	0/1
Chromosome2-1108206	DEL	PRECISE	61	0	0	0/0	0/0	0/0	0/0	0/0	0/0	0/0	0/0	0/0	0/0	0/0	0/0	0/0	1/1
Chromosome2-1109447	DEL	PRECISE	1656	0	0	0/0	0/0	0/1	0/0	0/0	0/0	0/0	0/0	0/0	0/0	0/0	0/0	0/0	0/0
Chromosome2-1109525	DEL	PRECISE	151	0	0	0/0	0/0	0/0	0/0	0/1	0/1	0/0	0/0	0/0	0/0	0/0	0/0	0/0	1/1
Chromosome2-1109801	DEL	PRECISE	98	0	0	0/0	0/0	0/0	0/0	0/1	0/1	0/0	0/0	0/0	0/0	0/0	0/0	0/0	0/0
Chromosome2-1110767	DEL	PRECISE	110	0.577	0.577	0/0	0/0	0/0	0/0	0/1	0/1	0/0	0/0	0/0	0/0	0/0	0/0	0/0	1/1
Chromosome2-1110835	INS	PRECISE	72	0	0	0/0	0/0	0/0	0/0	0/0	0/0	0/0	0/0	0/0	0/0	0/0	0/0	0/0	1/1
Chromosome2-1111423	INS	PRECISE	322	0	0.707	0/0	0/0	0/0	0/0	0/1	0/1	0/0	0/0	0/0	0/0	0/0	0/0	0/0	0/0
Chromosome2-1111620	DEL	PRECISE	434	0	0	0/0	0/0	0/1	0/1	0/0	0/0	0/0	0/0	0/0	0/0	0/0	0/0	0/0	0/0
Chromosome2-1112249	DEL	PRECISE	13181	0	0	0/0	0/0	0/0	0/0	0/1	0/1	0/0	0/0	0/0	0/0	0/0	0/0	0/0	0/0
Chromosome2-1112748	DEL	PRECISE	1112	0	0	0/0	0/0	0/1	0/1	0/0	0/0	0/0	0/0	0/0	0/0	0/0	0/0	0/0	1/1
Chromosome2-1112869	INS	PRECISE	56	0	0	0/0	0/0	0/1	0/1	0/0	0/0	0/0	0/0	0/0	0/0	0/0	0/0	0/0	0/0
Chromosome2-1113503	DEL	PRECISE	4721	0.707	0.707	0/0	0/0	0/0	0/1	0/0	0/0	0/0	0/0	0/0	0/0	0/0	0/0	0/0	0/0
Chromosome2-1113951	DEL	PRECISE	2964	0	0	0/0	0/0	0/0	0/0	0/1	0/1	0/0	0/0	0/0	0/0	0/0	0/0	0/0	0/0
Chromosome2-1113999	DEL	PRECISE	2167	0	0	0/0	0/0	0/0	0/1	0/0	0/0	0/0	0/0	0/0	0/0	0/0	0/0	0/0	0/0
Chromosome2-1114627	DEL	PRECISE	94	0.707	0.707	0/0	0/0	0/0	0/1	0/0	0/0	0/0	0/0	0/0	0/0	0/0	0/0	0/0	0/0
Chromosome2-1114875	DEL	PRECISE	1496	0.707	0	0/0	0/0	0/0	0/1	0/0	0/0	0/0	0/0	0/0	0/0	0/0	0/0	0/0	0/0
Chromosome2-1115905	BND	PRECISE	0	0	0	0/0	0/0	0/0	0/0	0/0	0/0	0/1	0/0	0/0	0/0	0/0	0/0	0/0	0/0
Chromosome2-1116279	INS	PRECISE	61	0	0.707	0/0	0/0	0/0	0/0	0/1	0/1	0/0	0/0	0/0	0/0	0/0	0/0	0/0	0/0
Chromosome2-1116710	DEL	PRECISE	2654	0	0	0/0	0/0	0/0	0/0	0/0	0/0	0/0	0/0	0/0	0/0	0/0	0/0	0/0	1/1
Chromosome2-1117055	DEL	PRECISE	338	0	0	0/0	0/0	0/1	0/1	0/0	0/0	0/0	0/0	0/0	0/0	0/0	0/0	0/0	0/0
Chromosome2-1117161	INS	PRECISE	179	0	0.707	0/0	0/0	0/1	0/1	0/0	0/0	0/0	0/0	0/0	0/0	0/0	0/0	0/0	0/0
Chromosome2-1118050	INS	PRECISE	62	0	0.408	0/0	0/0	0/1	0/1	0/1	0/1	0/1	0/0	0/0	0/0	0/0	0/0	0/0	1/1
Chromosome2-1118160	INS	PRECISE	2019	0	0.707	0/0	0/0	0/1	0/1	0/0	0/0	0/0	0/0	0/0	0/0	0/0	0/0	0/0	0/0
Chromosome2-1118444	INS	PRECISE	4516	0	0	0/0	0/0	0/0	0/0	0/0	0/0	0/0	0/0	0/0	0/0	0/0	0/0	0/0	1/1
Chromosome2-1118568	INS	PRECISE	1065	0	0	0/0	0/0	0/1	0/0	0/0	0/0	0/0	0/0	0/0	0/0	0/0	0/0	0/0	0/0
Chromosome2-1119150	INS	PRECISE	1362	0	0	0/0	0/0	0/0	0/1	0/0	0/0	0/0	0/0	0/0	0/0	0/0	0/0	0/0	0/0
Chromosome2-1119200	DEL	PRECISE	98	0	0	0/0	0/0	0/0	0/1	0/0	0/0	0/0	0/0	0/0	0/0	0/0	0/0	0/0	0/0
Chromosome2-1119396	INS	PRECISE	639	0	0	0/0	0/0	0/0	0/1	0/0	0/0	0/0	0/0	0/0	0/0	0/0	0/0	0/0	0/0
Chromosome2-1119878	INS	PRECISE	1802	0	0	0/0	0/0	0/0	0/0	0/0	0/0	0/0	0/0	0/0	0/0	0/0	0/0	0/0	1/1
Chromosome2-1120071	INS	PRECISE	153	0	0	0/0	0/0	0/0	0/0	0/0	0/0	0/1	0/0	0/0	0/0	0/0	0/0	0/0	1/1
Chromosome2-1120786	DEL	PRECISE	12393	0	0	0/0	0/0	0/0	0/0	1/1	1/1	0/0	0/0	0/0	0/0	0/0	0/0	0/0	1/1
Chromosome2-1122767	DEL	PRECISE	62	0	0	0/0	0/0	0/0	0/0	0/1	0/1	0/0	0/0	0/0	0/0	0/0	0/0	0/0	1/1
Chromosome2-1123656	INS	PRECISE	345	0	0	0/0	0/0	0/0	0/0	0/1	0/1	0/0	0/0	0/0	0/0	0/0	0/0	0/0	0/0
Chromosome2-1123740	INS	PRECISE	1122	0	0.707	0/0	0/0	0/0	0/0	1/1	0/1	0/0	0/0	0/0	0/0	0/0	0/0	0/0	0/0
Chromosome2-1123785	DEL	PRECISE	355	0	0	0/0	0/0	0/0	0/0	0/1	0/1	0/0	0/0	0/0	0/0	0/0	0/0	0/0	0/0
Chromosome2-1126159	DEL	PRECISE	24301	0	0	0/0	0/0	0/0	0/0	0/0	0/0	0/0	0/0	0/0	0/0	0/0	0/0	0/0	1/1

Chromosome2-1127565	DEL	PRECISE	1514	0	0	0/0	0/0	0/1	0/0	0/0	0/0	0/0	0/0	0/0	0/1	0/0	0/0	0/0	1/1
Chromosome2-1129648	DEL	PRECISE	239	0	0	0/0	0/0	0/0	0/0	0/0	0/0	0/0	0/0	0/0	0/0	0/0	0/0	0/0	1/1
Chromosome2-1129652	DEL	PRECISE	3063	0	0	0/0	0/0	0/0	0/0	0/1	0/1	0/0	0/0	0/0	0/0	0/0	0/0	0/0	0/0
Chromosome2-1131220	DEL	PRECISE	1549	0	0	0/0	0/0	0/0	0/0	0/1	0/1	0/0	0/0	0/0	0/0	0/0	0/0	0/0	0/0
Chromosome2-1131893	INS	PRECISE	1539	0	0	0/0	0/0	0/0	0/0	0/0	0/0	0/0	0/0	0/0	0/0	0/0	0/0	0/0	1/1
Chromosome2-1131916	INS	PRECISE	444	0	0	0/0	0/0	0/0	0/0	0/0	0/0	0/0	0/0	0/0	0/0	0/0	0/0	0/0	1/1
Chromosome2-1132543	DEL	PRECISE	115	0	0	0/0	0/0	0/0	0/0	0/1	0/1	0/0	0/0	0/0	0/0	0/0	0/0	0/0	1/1
Chromosome2-1133698	DEL	PRECISE	33576	0	0	0/0	0/0	0/0	0/0	0/1	1/1	0/0	0/0	0/0	0/0	0/0	0/0	0/0	0/0
Chromosome2-1133884	DEL	PRECISE	111	0	0	0/0	0/0	0/0	0/0	0/0	0/0	0/0	0/0	0/0	0/0	0/0	0/0	0/0	1/1
Chromosome2-1133989	DEL	PRECISE	11058	0	0	0/0	0/0	0/0	0/0	0/0	0/0	0/0	0/0	0/0	0/0	0/0	0/0	0/0	1/1
Chromosome2-1137093	INS	PRECISE	5819	0	0	0/0	0/0	0/0	0/0	0/0	0/0	0/0	0/0	0/0	0/0	0/0	0/0	0/0	1/1
Chromosome2-1138219	INS	PRECISE	2912	0	0.707	0/0	0/0	0/0	0/0	0/1	0/1	0/0	0/0	0/0	0/0	0/0	0/0	0/0	0/0
Chromosome2-1138764	INS	PRECISE	12526	0	0	0/0	0/0	0/0	0/0	0/1	0/0	0/0	0/0	0/0	0/0	0/0	0/0	0/0	0/0
Chromosome2-1141454	INS	PRECISE	1937	0	0	0/0	0/0	0/0	0/0	0/1	0/1	0/0	0/0	0/0	0/0	0/0	0/0	0/0	0/0
Chromosome2-1141920	DEL	PRECISE	869	0	0	0/0	0/0	0/0	0/0	0/1	0/1	0/0	0/0	0/0	0/0	0/0	0/0	0/0	0/0
Chromosome2-1143680	INS	PRECISE	3323	0	0	0/0	0/0	0/1	0/0	0/0	0/0	0/0	0/0	0/0	0/0	0/0	0/0	0/0	0/0
Chromosome2-1143680	INS	PRECISE	4786	0	0	0/0	0/0	0/0	0/1	0/0	0/0	0/0	0/0	0/0	0/0	0/0	0/0	0/0	0/0
Chromosome2-1144370	INS	PRECISE	1297	0	0	0/0	0/0	0/1	0/0	0/0	0/0	0/0	0/0	0/0	0/0	0/0	0/0	0/0	0/0
Chromosome2-1145505	INS	PRECISE	160	0	0	0/0	0/0	0/0	0/0	0/0	0/0	0/1	0/0	0/0	0/0	0/0	0/0	0/0	0/0
Chromosome2-1147236	INS	PRECISE	211	0	0.707	0/0	0/0	0/1	0/1	0/0	0/0	0/0	0/0	0/0	0/0	0/0	0/0	0/0	0/0
Chromosome2-1147619	DEL	PRECISE	112	0	0	0/0	0/0	0/1	0/1	0/1	0/1	0/1	0/0	0/0	0/0	0/0	0/0	0/0	1/1
Chromosome2-1147985	INS	PRECISE	114	0	0	0/0	0/0	0/1	0/0	0/0	0/0	0/0	0/0	0/0	0/0	0/0	0/0	0/0	0/0
Chromosome2-1149871	INS	PRECISE	5046	0	0	0/0	0/0	0/0	0/1	0/0	0/0	0/0	0/0	0/0	0/0	0/0	0/0	0/0	0/0
Chromosome2-1149983	INS	PRECISE	1185	0	0	0/0	0/0	0/0	0/0	0/0	0/0	0/0	0/0	0/0	0/0	0/0	0/0	0/0	1/1
Chromosome2-1150120	DEL	PRECISE	56	0	0	0/0	0/0	0/1	0/0	0/1	0/1	0/0	0/0	0/0	0/0	0/0	0/0	0/0	0/0
Chromosome2-1150845	INS	PRECISE	132	0	0	0/0	0/0	0/1	0/0	0/1	0/1	0/0	0/0	0/0	0/0	0/0	0/0	0/0	1/1
Chromosome2-1151838	DEL	PRECISE	57	0	0	0/0	0/0	0/0	0/0	0/0	0/0	0/0	0/0	0/0	0/0	0/0	0/0	0/0	1/1
Chromosome2-1153587	DEL	PRECISE	113	0	0	0/0	0/0	0/0	0/0	0/0	0/0	0/0	0/0	0/0	0/0	0/0	0/0	0/0	1/1
Chromosome2-1155369	DEL	PRECISE	524	0	0	0/0	0/0	0/0	0/0	0/0	0/0	0/0	0/0	0/0	0/0	0/0	0/0	0/0	1/1
Chromosome2-1155829	DEL	PRECISE	340	0	0	0/0	0/0	0/0	0/0	0/1	0/1	0/0	0/0	0/0	0/0	0/0	0/0	0/0	0/0
Chromosome2-1157264	DEL	PRECISE	53	0.707	0.707	0/0	0/0	0/0	0/0	0/1	0/1	0/0	0/0	0/0	0/0	0/0	0/0	0/0	0/0
Chromosome2-1157774	INS	PRECISE	69	0	0	0/0	0/0	0/1	0/0	0/1	0/1	0/1	0/0	0/0	0/0	0/0	0/0	0/0	1/1
Chromosome2-1158057	INS	PRECISE	507	0	0	0/0	0/0	0/0	0/0	0/0	0/0	0/0	0/0	0/0	0/0	0/0	0/0	0/0	1/1
Chromosome2-1158785	DEL	PRECISE	2167	0	0	0/0	0/0	0/1	0/0	0/0	0/0	0/0	0/0	0/0	0/0	0/0	0/0	0/0	0/0
Chromosome2-1160321	DEL	PRECISE	57	0	0	0/0	0/0	0/0	0/0	0/0	0/0	0/1	0/0	0/0	0/0	0/0	0/0	0/0	0/0
Chromosome2-1160463	DEL	PRECISE	84	0	0	0/0	0/0	0/0	0/0	0/0	0/0	0/0	0/0	0/1	0/1	0/0	1/1	1/1	0/0
Chromosome2-1160591	INS	PRECISE	1574	0	0	0/0	0/0	0/1	0/0	0/0	0/0	0/0	0/0	0/0	0/0	0/0	0/0	0/0	0/0
Chromosome2-1160863	DEL	PRECISE	137	0	0	0/0	0/0	0/0	0/0	0/0	0/0	0/0	0/0	0/0	0/1	0/0	0/1	0/0	0/0
Chromosome2-1161065	INS	PRECISE	197	0	0.674	0/0	0/0	0/1	0/1	0/1	0/1	0/1	0/0	0/1	0/1	1/1	1/1	1/1	1/1
Chromosome2-1161878	INS	PRECISE	554	0	0	0/0	0/0	0/0	0/0	0/0	0/0	0/1	0/0	0/0	0/0	0/0	0/0	0/0	0/0
Chromosome2-1162902	INS	PRECISE	1295	0	0	0/0	0/0	0/1	0/0	0/0	0/0	0/0	0/0	0/0	0/0	0/0	0/0	0/0	0/0
Chromosome2-1164471	INV	PRECISE	49639	0	0	0/0	0/0	0/0	0/0	0/1	0/0	0/0	0/0	0/0	0/0	0/0	0/0	0/0	0/0
Chromosome2-1165307	INS	PRECISE	211	0	0	0/0	0/0	0/0	0/0	0/0	0/0	0/0	0/0	0/0	0/0	0/0	0/0	0/0	1/1
Chromosome2-1165404	DEL	PRECISE	4597	0	0	0/0	0/0	0/0	0/1	0/0	0/0	0/0	0/0	0/0	0/0	0/0	0/0	0/0	0/0
Chromosome2-1166219	DEL	PRECISE	61	0.548	0	0/0	0/1	0/1	0/0	0/0	0/0	0/0	0/0	0/1	0/0	0/1	0/0	1/1	0/0
Chromosome2-1166339	INS	PRECISE	6148	0	0	0/0	0/0	0/0	0/0	0/0	0/0	1/1	0/0	0/0	0/0	0/0	0/0	0/0	0/0
Chromosome2-1166339	BND	PRECISE	0	0	0	0/0	0/0	0/0	0/0	0/0	0/0	0/1	0/0	0/0	0/0	0/0	0/0	0/0	0/0
Chromosome2-1166407	INS	PRECISE	1204	0	0	0/0	0/1	0/1	0/0	0/0	0/0	0/0	0/0	0/0	0/0	0/0	0/0	0/0	0/0
Chromosome2-1166793	DEL	PRECISE	69	0	0	0/0	0/0	0/1	0/0	0/0	0/0	0/0	0/0	0/0	0/0	0/0	0/0	0/0	0/0
Chromosome2-1166850	INS	PRECISE	72	0	0	0/0	0/0	0/0	0/0	0/0	0/0	0/0	0/0	0/0	0/0	0/0	0/0	0/0	1/1
Chromosome2-1170119	INS	PRECISE	50	0	0	0/0	0/0	0/1	0/0	0/0	0/0	0/0	0/0	0/0	0/0	0/0	0/0	0/0	1/1
Chromosome2-1170266	DEL	PRECISE	9881	0	0	0/0	0/0	0/1	0/0	0/0	0/1	0/0	0/0	0/0	0/0	0/0	0/0	0/0	0/0
Chromosome2-1170307	DEL	PRECISE	115	0	0	0/0	0/0	0/0	0/0	0/0	0/0	0/1	0/0	0/0	0/0	0/0	0/0	0/0	0/0
Chromosome2-1170602	INS	PRECISE	57	0	0	0/0	0/0	0/0	0/1	0/0	0/0	0/1	0/0	0/0	0/0	0/0	0/0	0/0	0/0
Chromosome2-1171319	INS	PRECISE	10185	0	0	0/0	0/0	0/0	0/0	0/0	0/0	0/1	0/0	0/0	0/0	0/0	0/0	0/0	0/0
Chromosome2-1172963	DEL	PRECISE	50	0	0	0/0	0/0	0/0	0/0	0/0	0/0	0/0	0/0	0/0	0/0	1/1	1/1	1/1	0/0
Chromosome2-1174079	INS	PRECISE	141	0	0	0/0	0/0	0/0	0/0	0/0	0/0	0/0	0/0	0/0	0/0	0/0	0/0	0/0	1/1
Chromosome2-1175628	INS	PRECISE	579	0	0.707	0/0	0/0	0/0	0/0	0/1	0/1	0/0	0/0	0/0	0/0	0/0	0/0	0/0	0/0
Chromosome2-1176957	DEL	PRECISE	122	0	0	0/0	0/0	0/0	0/0	0/1	0/1	0/0	0/0	0/0	0/0	0/0	0/0	0/0	0/0
Chromosome2-1177241	DEL	PRECISE	161	0	0	0/0	0/0	0/0	0/0	0/1	0/1	0/0	0/0	0/0	0/0	0/0	0/0	0/0	0/0
Chromosome2-1177850	DEL	PRECISE	293	0	0	0/0	0/0	0/0	0/0	0/0	0/0	0/0	0/0	0/0	0/0	0/0	0/0	0/0	1/1
Chromosome2-1179238	DEL	PRECISE	459	0	0	0/0	0/0	0/0	0/1	0/0	0/0	0/0	0/0	0/0	0/0	0/0	0/0	0/0	0/0
Chromosome2-1180412	DEL	PRECISE	6058	0	0	0/0	0/0	0/0	0/0	0/0	0/0	0/0	0/0	0/0	0/0	0/0	0/0	0/0	1/1
Chromosome2-1181099	INS	PRECISE	84	0	0	0/0	0/0	0/0	0/0	0/0	0/0	0/0	0/0	0/0	0/0	0/0	0/0	0/0	1/1
Chromosome2-1181136	DEL	PRECISE	207	0	0	0/0	0/0	0/0	0/0	0/0	0/0	0/0	0/0	0/0	0/0	0/0	0/0	0/0	1/1
Chromosome2-1181589	INS	PRECISE	533	0	0	0/0	0/0	0/0	0/0	0/0	0/0	0/0	0/0	0/0	0/0	0/0	0/0	0/0	1/1
Chromosome2-1182182	DEL	PRECISE	1474	0	0	0/0	0/0	0/1	0/0	0/1	0/1	0/0	0/0	0/0	0/0	0/0	0/0	0/0	1/1
Chromosome2-1182598	INS	PRECISE	57	0	0	0/0	0/0	0/1	0/0	0/1	0/1	0/0	0/0	0/0	0/0	0/0	0/0	0/0	1/1
Chromosome2-1187637	DEL	PRECISE	1574	0	0	0/0	0/0	0/0	0/0	0/0	0/0	0/0	0/0	0/0	0/0	0/0	0/0	0/0	1/1
Chromosome2-1188494	DEL	PRECISE	311	0	0	0/0	0/0	0/1	0/0	0/0	0/0	0/0	0/0	0/0	0/0	0/0	0/0	0/0	0/0
Chromosome2-1188542	DEL	PRECISE	2015	0	0	0/0	0/0	0/0	0/0	0/1	0/1	0/0	0/0	0/0	0/0	0/0	0/0	0/0	0/0
Chromosome2-1188553	INS	PRECISE	388	0	0	0/0	0/0	0/0	0/0	0/0	0/0	0/0	0/0	0/0	0/0	0/0	0/0	0/0	1/1
Chromosome2-1189417	DEL	PRECISE	1510	0	0	0/0	0/0	0/0	0/1	0/0	0/0	0/0	0/0	0/0	0/0	0/0	0/0	0/0	0/0

Chromosome2-1190590	DEL	PRECISE	3556	0	0	0/0	0/0	0/1	0/1	0/1	0/1	0/0	0/0	0/0	0/0	0/0	0/0	0/0	1/1
Chromosome2-1190973	DEL	PRECISE	588	0	0	0/0	0/0	0/0	0/1	0/0	0/0	0/0	0/0	0/0	0/0	0/0	0/0	0/0	0/0
Chromosome2-1191131	INS	PRECISE	5277	0	0	0/0	0/0	0/0	0/0	0/0	0/0	0/0	0/0	0/0	0/0	0/0	0/0	0/0	1/1
Chromosome2-1192342	DEL	PRECISE	843	0	0	0/0	0/0	0/1	0/0	0/1	0/1	0/0	0/0	0/0	0/0	0/0	0/0	0/0	1/1
Chromosome2-1192497	DEL	PRECISE	512	0	0	0/0	0/0	0/0	0/0	0/0	0/0	0/0	0/0	0/0	0/0	0/0	0/0	0/0	1/1
Chromosome2-1192563	DEL	PRECISE	865	0	0	0/0	0/0	0/0	0/1	0/0	0/0	0/0	0/0	0/0	0/0	0/0	0/0	0/0	0/0
Chromosome2-1192652	DEL	PRECISE	210	0	0	0/0	0/0	0/0	0/1	0/0	0/0	0/0	0/0	0/0	0/0	0/0	0/0	0/0	0/0
Chromosome2-1193701	DEL	PRECISE	1189	0	0	0/1	0/1	0/1	0/1	0/1	0/1	0/1	0/1	0/1	0/1	1/1	1/1	0/0	0/1
Chromosome2-1193815	DEL	PRECISE	1122	0	0	0/0	0/0	0/0	0/0	0/0	0/0	0/0	0/0	0/0	0/0	0/0	0/0	0/0	0/1
Chromosome2-1194233	DEL	PRECISE	1041	0	0	0/1	0/1	0/1	0/1	0/1	0/1	0/1	0/1	0/1	0/1	0/0	0/1	0/0	0/0
Chromosome2-1194602	BND	PRECISE	0	0	0	0/0	0/1	0/0	0/0	0/0	0/0	0/0	0/1	0/0	0/0	0/0	0/0	0/0	0/0
Chromosome2-1194664	INS	PRECISE	863	0	0	0/0	0/0	0/0	0/0	0/0	0/0	0/0	0/0	0/0	0/0	0/0	0/0	0/0	1/1
Chromosome2-1195005	DEL	PRECISE	1295	0	0	0/0	0/0	0/1	0/0	0/0	0/0	0/0	0/0	0/0	0/0	0/0	0/0	0/0	1/1
Chromosome2-1195571	INS	PRECISE	86	0	0	0/0	0/0	0/0	0/0	0/0	0/0	0/0	0/0	0/0	0/0	0/0	0/0	0/0	1/1
Chromosome2-1195763	INS	PRECISE	131	0	0	0/0	0/0	0/0	0/0	0/0	0/0	0/0	0/0	0/0	0/0	0/0	0/0	0/0	1/1
Chromosome2-1195821	DEL	PRECISE	372	0	0	0/0	0/0	0/1	0/0	0/1	0/1	0/0	0/0	0/0	0/0	0/0	0/0	0/0	1/1
Chromosome2-1196048	DEL	PRECISE	39760	0	0	0/0	0/0	0/1	0/0	0/0	0/0	0/0	0/0	0/0	0/0	0/0	0/0	0/0	0/0
Chromosome2-1196313	DEL	PRECISE	79	0	0	0/0	0/0	0/0	0/0	0/1	0/1	0/0	0/0	0/0	0/0	0/0	0/0	0/0	0/0
Chromosome2-1198897	INS	PRECISE	887	0	0	0/0	0/0	0/0	0/0	0/0	0/0	0/0	0/0	0/0	0/0	0/0	0/0	0/0	1/1
Chromosome2-1199954	INS	PRECISE	10285	0	0	0/0	0/0	0/0	0/0	0/0	0/0	0/0	0/0	0/0	0/0	0/0	0/0	0/0	1/1
Chromosome2-1201838	DEL	PRECISE	55	0	0	0/0	0/0	0/1	0/0	0/0	0/0	0/0	0/0	0/0	0/0	0/0	0/0	0/0	0/0
Chromosome2-1202001	INS	PRECISE	1458	0	0	0/0	0/0	0/0	0/0	0/0	0/0	0/0	0/0	0/0	0/0	0/0	0/0	0/0	1/1
Chromosome2-1202593	DEL	PRECISE	380	0	0	0/0	0/0	0/0	0/1	0/0	0/0	0/0	0/0	0/0	0/0	0/0	0/0	0/0	0/0
Chromosome2-1203055	DEL	PRECISE	538	0	0	0/0	0/0	0/0	0/0	0/0	0/0	0/0	0/0	0/0	0/0	0/0	0/0	0/0	1/1
Chromosome2-1203661	INS	PRECISE	713	0	0	0/0	0/0	0/0	0/0	0/0	0/0	0/0	0/0	0/0	0/0	0/0	0/0	0/0	1/1
Chromosome2-1204047	DEL	PRECISE	150	0	0	0/0	0/0	0/1	0/0	0/1	0/0	0/0	0/0	0/0	0/0	0/0	0/0	0/0	0/0
Chromosome2-1204554	INS	PRECISE	3574	0	0	0/0	0/0	0/0	0/0	0/0	0/0	0/0	0/0	0/0	0/0	0/0	0/0	0/0	1/1
Chromosome2-1204632	INS	PRECISE	137	0	0	0/0	0/0	0/0	0/0	0/0	0/0	0/1	0/0	0/0	0/0	0/0	0/0	0/0	0/0
Chromosome2-1205009	DEL	PRECISE	2195	0	0	0/0	0/0	0/0	0/1	0/0	0/0	0/0	0/0	0/0	0/0	0/0	0/0	0/0	0/0
Chromosome2-1205025	DEL	PRECISE	107	0	0	0/0	0/0	0/1	0/0	0/1	0/0	0/0	0/0	0/0	0/0	0/0	0/0	0/0	0/0
Chromosome2-1205547	INS	PRECISE	9769	0	0	0/0	0/0	0/0	0/0	0/0	0/1	0/0	0/0	0/0	0/0	0/0	0/0	0/0	0/0
Chromosome2-1206798	INS	PRECISE	78	0	0	0/0	0/0	0/0	0/0	0/0	0/0	0/0	0/0	0/0	0/0	0/0	0/0	0/0	1/1
Chromosome2-1207666	INS	PRECISE	837	0	0	0/0	0/0	0/0	0/1	0/0	0/0	0/0	0/0	0/0	0/0	0/0	0/0	0/0	0/0
Chromosome2-1207954	INS	PRECISE	218	0	0	0/0	0/0	0/1	0/0	0/0	0/0	0/0	0/0	0/0	0/0	0/0	0/0	0/0	0/0
Chromosome2-1208156	INS	PRECISE	627	0	0	0/0	0/0	0/0	0/0	0/0	0/0	0/0	0/0	0/0	0/0	0/0	0/0	0/0	1/1
Chromosome2-1209317	INV	PRECISE	19830169	0	0	0/0	0/0	0/0	0/0	0/0	0/0	0/0	0/0	0/0	0/0	0/0	0/0	0/0	0/1
Chromosome2-1209432	INS	PRECISE	184	0	0	0/0	0/0	0/0	0/0	0/0	0/0	0/0	0/0	0/0	0/0	0/0	0/0	0/0	1/1
Chromosome2-1210057	INS	PRECISE	166	0	0	0/0	0/0	0/0	0/0	0/1	0/1	0/0	0/0	0/0	0/0	0/0	0/0	0/0	0/0
Chromosome2-1210100	DEL	PRECISE	97	0	0	0/0	0/0	0/0	0/0	0/0	0/0	0/0	0/0	0/0	0/0	0/0	1/1	0/0	0/0
Chromosome2-1210230	DEL	PRECISE	230	0	0	0/0	0/0	0/0	0/0	0/0	0/0	0/0	0/0	0/0	0/0	0/0	1/1	0/0	0/0
Chromosome2-1210552	INS	PRECISE	826	0	0.707	0/0	0/0	0/0	0/0	0/1	0/1	0/0	0/0	0/0	0/0	0/0	0/0	0/0	0/0
Chromosome2-1211178	INS	PRECISE	194	0	0	0/0	0/0	0/1	0/0	0/0	0/0	0/0	0/0	0/0	0/0	0/0	0/0	0/0	0/0
Chromosome2-1211478	DEL	PRECISE	556	0	0	0/0	0/0	0/1	0/0	0/1	0/1	0/0	0/0	0/0	0/0	0/0	0/0	0/0	1/1
Chromosome2-1213592	INS	PRECISE	4141	0	0.707	0/0	0/0	0/0	0/0	1/1	1/1	0/0	0/0	0/0	0/0	0/0	0/0	0/0	0/0
Chromosome2-1215031	DEL	PRECISE	6792	0	0	0/0	0/0	0/0	0/0	0/0	0/0	0/0	0/0	0/0	0/0	0/0	0/0	0/0	1/1
Chromosome2-1216259	INS	PRECISE	560	0	0	0/0	0/0	0/0	0/0	0/0	0/0	0/1	0/0	0/0	0/0	0/0	0/0	0/0	0/0
Chromosome2-1217280	DEL	PRECISE	88	0	0	0/0	0/0	0/1	0/0	0/0	0/0	0/0	0/0	0/0	0/0	0/0	0/0	0/0	0/0
Chromosome2-1217429	INS	PRECISE	1416	0	0	0/0	0/0	0/0	0/0	0/0	0/0	0/1	0/0	0/0	0/0	0/0	0/0	0/0	0/0
Chromosome2-1219060	INS	PRECISE	1393	0	0	0/0	0/0	0/1	0/0	0/0	0/0	0/0	0/0	0/0	0/0	0/0	0/0	0/0	0/0
Chromosome2-1219274	DEL	PRECISE	69	0	0	0/0	0/0	0/1	0/0	0/0	0/0	0/0	0/0	0/0	0/0	0/0	0/0	0/0	0/0
Chromosome2-1220569	INS	PRECISE	991	0	0	0/0	0/0	0/0	0/0	0/0	0/0	0/0	0/0	0/0	0/0	0/0	0/0	0/0	1/1
Chromosome2-1220717	INS	PRECISE	59	0	0	0/0	0/0	0/0	0/0	0/0	0/0	0/0	0/0	0/0	0/0	0/0	0/0	0/0	1/1
Chromosome2-1221507	DEL	PRECISE	480	0	0	0/0	0/0	0/1	0/0	0/0	0/0	0/1	0/0	0/0	0/0	0/0	0/0	0/0	0/0
Chromosome2-1221860	INS	PRECISE	1362	0	0	0/0	0/0	0/0	0/0	0/0	0/0	0/1	0/0	0/0	0/0	0/0	0/0	0/0	0/0
Chromosome2-1221860	INS	PRECISE	11227	0	0	0/0	0/0	0/0	0/0	0/0	0/0	0/0	0/0	0/0	0/0	0/0	0/0	0/0	1/1
Chromosome2-1222748	DEL	PRECISE	74	0	0	0/0	0/0	0/0	0/0	0/0	0/0	0/1	0/0	0/0	0/0	0/0	0/0	0/0	0/0
Chromosome2-1222971	INS	PRECISE	196	0	0	0/0	0/0	0/0	0/0	0/0	0/0	0/1	0/0	0/0	0/0	0/0	0/0	0/0	0/0
Chromosome2-1223622	BND	PRECISE	0	0	0	0/0	0/1	0/0	0/0	0/0	0/0	0/0	0/0	0/0	0/0	0/1	0/1	0/0	0/0
Chromosome2-1223731	DEL	PRECISE	360	0	0	0/0	0/1	0/0	0/0	0/0	0/0	0/0	0/0	0/0	0/0	1/1	1/1	0/0	0/0
Chromosome2-1223926	BND	PRECISE	0	0	0	0/0	0/0	0/0	0/0	0/0	0/0	0/0	0/0	0/0	0/0	0/0	0/1	0/0	0/0
Chromosome2-1224131	INS	PRECISE	12464	0	0	0/0	0/0	0/0	0/0	0/0	0/0	0/1	0/0	0/0	0/0	0/0	0/0	0/0	0/0
Chromosome2-1224165	DEL	PRECISE	117	0	0	0/0	0/0	0/0	0/0	0/0	0/0	0/0	0/0	0/0	0/0	0/1	0/0	0/0	0/0
Chromosome2-1224359	INS	PRECISE	4492	0	0	0/0	0/0	1/1	0/0	0/0	0/0	0/0	0/0	0/0	0/0	0/0	0/0	0/0	0/0
Chromosome2-1224473	INS	PRECISE	1369	0	0	0/0	0/0	0/0	0/0	0/0	0/0	0/0	0/0	0/0	0/0	0/1	0/0	0/0	0/0
Chromosome2-1224650	DEL	PRECISE	642	0	0	0/0	0/0	0/0	0/0	0/0	0/0	0/0	0/0	0/0	0/0	0/0	0/1	0/0	0/0
Chromosome2-1224850	BND	PRECISE	0	0	0	0/0	0/1	0/0	0/0	0/0	0/0	0/0	0/0	0/0	0/0	0/0	0/0	0/0	0/0
Chromosome2-1225606	INS	PRECISE	12589	0	0	0/0	0/0	0/1	0/0	0/0	0/0	0/0	0/0	0/0	0/0	0/0	0/0	0/0	0/0
Chromosome2-1226812	INS	PRECISE	2989	0	0	0/0	0/0	1/1	0/0	0/0	0/0	0/0	0/0	0/0	0/0	0/0	0/0	0/0	0/0
Chromosome2-1226907	INS	PRECISE	11006	0	0	0/0	0/0	0/0	0/0	0/0	0/0	1/1	0/0	0/0	0/0	0/0	0/0	0/0	0/0
Chromosome2-1226907	BND	PRECISE	0	0	0	0/0	0/0	0/0	0/0	0/0	0/0	0/1	0/0	0/0	0/0	0/0	0/0	0/0	0/0
Chromosome2-1227394	INS	PRECISE	7331	0	0	0/0	0/0	1/1	0/0	0/0	0/0	0/0	0/0	0/0	0/0	0/0	0/0	0/0	0/0
Chromosome2-1227894	INS	PRECISE	58	0	0	0/0	0/0	0/0	0/0	0/1	0/1	0/0	0/0	0/0	0/0	0/0	0/0	0/0	0/0
Chromosome2-1228049	INS	PRECISE	5895	0	0	0/0	0/0	0/0	0/0	0/0	0/0	0/0	0/0	0/0	0/0	0/0	0/0	0/0	1/1

Chromosome2-1228051	BND	PRECISE	0	0	0	0/0	0/0	0/0	0/0	0/0	0/0	0/0	0/0	0/0	0/0	0/0	0/0	0/0	0/1
Chromosome2-1228179	INS	PRECISE	1079	0	0	0/0	0/0	0/0	0/0	0/0	0/0	0/0	0/0	0/0	0/0	0/0	0/0	0/0	1/1
Chromosome2-1228382	DEL	PRECISE	61	0	0	0/0	0/0	0/0	0/0	0/1	0/1	0/0	0/0	0/0	0/0	0/0	0/0	0/0	0/0
Chromosome2-1228586	DEL	PRECISE	68	0	0	0/0	0/0	0/0	0/0	0/0	0/0	0/0	0/0	0/0	0/0	0/0	0/0	0/0	1/1
Chromosome2-1229777	DEL	PRECISE	2394	0	0	0/0	1/1	0/0	0/0	0/1	0/0	0/1	0/1	1/1	0/1	0/0	0/0	0/0	1/1
Chromosome2-1230127	INS	PRECISE	491	0	0.441	0/0	0/1	0/0	0/0	0/1	0/0	0/1	0/1	0/1	0/1	1/1	1/1	0/0	0/1
Chromosome2-1230346	INS	PRECISE	7243	0	0	0/0	0/0	0/0	0/0	0/0	0/0	0/0	0/0	0/0	0/0	0/0	0/0	0/0	0/1
Chromosome2-1230976	DEL	PRECISE	54	0	0	0/0	0/1	0/0	0/0	0/1	0/0	0/1	0/1	0/1	0/1	0/0	0/1	0/0	0/1
Chromosome2-1231127	BND	PRECISE	0	0	0	0/0	0/1	0/0	0/0	0/1	0/0	0/1	0/1	0/1	0/1	0/0	0/0	0/0	1/1
Chromosome2-1232938	INS	PRECISE	888	0	0	0/0	0/0	0/0	0/0	0/0	0/0	0/0	0/0	0/0	0/0	0/0	0/0	0/0	1/1
Chromosome2-1233694	INS	PRECISE	427	0	0	0/0	0/0	0/0	0/0	0/0	0/0	0/0	0/0	0/0	0/0	0/0	0/0	0/0	1/1
Chromosome2-1234325	INS	PRECISE	635	0	0	0/0	0/0	0/0	0/0	0/0	0/0	0/1	0/0	0/0	0/0	0/0	0/0	0/0	0/0
Chromosome2-1235153	DEL	PRECISE	245	0	0	0/0	0/0	0/0	0/0	0/0	0/0	0/1	0/0	0/0	0/0	0/0	0/0	0/0	0/0
Chromosome2-1236469	DEL	PRECISE	53	0	0	0/0	0/0	0/0	0/0	0/1	0/1	0/0	0/0	0/0	0/0	0/0	0/0	0/0	0/0
Chromosome2-1236632	INS	PRECISE	306	0	0	0/0	0/0	0/0	0/0	0/0	0/0	0/1	0/0	0/0	0/0	0/0	0/0	0/0	0/0
Chromosome2-1237220	DEL	PRECISE	12474	0	0	0/0	0/0	0/1	0/0	0/1	0/1	0/1	0/0	0/0	0/0	0/0	0/0	0/0	0/0
Chromosome2-1238868	INS	PRECISE	58	0	0	0/0	0/0	0/0	0/0	0/0	0/0	0/1	0/0	0/0	0/0	0/0	0/0	0/0	0/0
Chromosome2-1239081	INS	PRECISE	363	0	0	0/0	0/0	0/0	0/0	0/0	0/0	0/1	0/0	0/0	0/0	0/0	0/0	0/0	0/0
Chromosome2-1239895	DEL	PRECISE	66	0	0	0/0	0/0	0/0	0/0	0/1	0/1	0/0	0/0	0/0	0/0	0/0	0/0	0/0	0/0
Chromosome2-1240116	INS	PRECISE	104	0	0	0/0	0/0	0/0	0/0	0/0	0/0	0/1	0/0	0/0	0/0	0/0	0/0	0/0	0/0
Chromosome2-1241009	INS	PRECISE	140	0	0	0/0	0/0	0/0	0/0	0/1	0/0	0/0	0/0	0/0	0/0	0/0	0/0	0/0	0/0
Chromosome2-1242097	DEL	PRECISE	187	0.577	0.577	0/0	0/0	0/1	0/0	0/1	0/1	0/0	0/0	0/0	0/0	0/0	0/0	0/0	0/0
Chromosome2-1245365	DUP	PRECISE	2889	0	0	0/0	0/0	0/0	0/1	0/0	0/0	0/1	0/0	0/0	0/0	0/0	0/0	0/0	0/0
Chromosome2-1245582	INS	PRECISE	5784	0	0	0/0	0/0	0/0	0/0	0/0	0/0	0/0	0/1	0/0	0/0	0/0	0/0	0/0	0/0
Chromosome2-1247915	INS	PRECISE	1352	0	0	0/0	0/0	0/0	0/0	0/0	0/0	0/0	0/0	0/0	0/0	0/0	0/0	0/0	1/1
Chromosome2-1250139	DEL	PRECISE	162	0	0	0/0	0/0	0/1	0/0	0/1	0/1	0/0	0/0	0/0	0/0	0/0	0/0	0/0	0/0
Chromosome2-1250313	INS	PRECISE	158	0	0	0/0	0/0	0/1	0/0	0/1	0/1	0/0	0/0	0/0	0/0	0/0	0/0	0/0	0/0
Chromosome2-1251821	INS	PRECISE	506	0	0.707	0/0	0/0	0/0	0/0	0/1	0/1	0/0	0/0	0/0	0/0	0/0	0/0	0/0	0/0
Chromosome2-1252632	DEL	PRECISE	7340	0	0	0/0	0/0	0/1	0/0	0/0	0/0	0/0	0/0	0/0	0/0	0/0	0/0	0/0	0/0
Chromosome2-1252743	INS	PRECISE	1414	0	0	0/0	0/0	0/0	0/0	0/0	0/0	0/0	0/0	0/0	0/0	0/0	0/0	0/0	1/1
Chromosome2-1252753	DEL	PRECISE	5454	0	0	0/0	0/0	0/0	0/0	0/1	0/1	0/0	0/0	0/0	0/0	0/0	0/0	0/0	0/0
Chromosome2-1252959	INS	PRECISE	53	0	0	0/0	0/0	0/0	0/0	0/0	0/0	0/0	0/0	0/0	0/0	0/0	0/0	0/0	1/1
Chromosome2-1253239	INS	PRECISE	133	0	0	0/0	0/0	0/0	0/0	0/0	0/0	0/0	0/0	0/0	0/0	0/0	0/0	0/0	1/1
Chromosome2-1254060	INS	PRECISE	132	0	0	0/0	0/0	0/0	0/0	0/0	0/0	0/1	0/0	0/0	0/0	0/0	0/0	0/0	0/0
Chromosome2-1254297	DEL	PRECISE	287	0	0	0/0	0/0	0/1	0/0	0/1	0/1	0/0	0/0	0/0	0/0	0/0	0/0	0/0	0/0
Chromosome2-1254590	DEL	PRECISE	70	0	0	0/0	0/0	0/1	0/0	0/0	0/0	0/0	0/0	0/0	0/0	0/0	0/0	0/0	0/0
Chromosome2-1254592	DEL	PRECISE	1751	0	0	0/0	0/0	0/0	0/0	0/0	0/0	0/0	0/0	0/0	0/0	0/0	0/0	0/0	1/1
Chromosome2-1257330	INS	PRECISE	133	0	0	0/0	0/0	0/0	0/0	0/0	0/0	0/0	0/0	0/0	0/0	0/0	0/0	0/0	1/1
Chromosome2-1257840	DEL	PRECISE	10830	0	0	0/0	0/0	0/1	0/0	0/0	0/0	0/0	0/0	0/0	0/0	0/0	0/0	0/0	0/0
Chromosome2-1258178	DEL	PRECISE	56	0	0	0/0	0/0	0/0	0/0	0/0	0/0	0/0	0/0	0/0	0/0	0/0	0/0	0/0	1/1
Chromosome2-1261118	DEL	PRECISE	65	0	0	0/0	0/0	0/0	0/0	0/0	0/0	0/0	0/0	0/0	0/0	0/0	0/0	0/0	1/1
Chromosome2-1261505	DEL	PRECISE	62	0	0	0/0	0/0	0/0	0/0	0/1	0/1	0/0	0/0	0/0	0/0	0/0	0/0	0/0	0/0
Chromosome2-1261925	DEL	PRECISE	504	0	0	0/0	0/0	0/1	0/0	0/1	0/1	0/0	0/0	0/0	0/0	0/0	0/0	0/0	0/0
Chromosome2-1262002	DEL	PRECISE	62	0	0	0/0	0/0	0/1	0/0	0/1	0/1	0/0	0/0	0/0	0/0	0/0	0/0	0/0	0/0
Chromosome2-1263045	INS	PRECISE	9999	0	0	0/0	0/0	0/0	0/0	0/0	0/0	0/1	0/0	0/0	0/0	0/0	0/0	0/0	0/0
Chromosome2-1263960	DEL	PRECISE	932	0	0	0/0	0/0	0/1	0/0	0/1	0/1	0/0	0/0	0/0	0/0	0/0	0/0	0/0	0/0
Chromosome2-1264668	INS	PRECISE	1270	0	0.707	0/0	0/0	0/0	0/0	0/1	0/1	0/0	0/0	0/0	0/0	0/0	0/0	0/0	0/0
Chromosome2-1265119	DEL	PRECISE	102	0	0	0/0	0/0	0/0	0/0	0/0	0/0	0/0	0/0	0/0	0/0	0/0	0/0	0/0	1/1
Chromosome2-1265234	INS	PRECISE	3450	0	0	0/0	0/0	0/0	0/0	0/0	0/0	0/0	0/0	0/0	0/0	0/0	0/0	0/0	1/1
Chromosome2-1265917	DEL	PRECISE	11422	0	0	0/0	0/0	0/1	0/0	0/1	0/1	0/0	0/0	0/0	0/0	0/0	0/0	0/0	0/0
Chromosome2-1266187	DEL	PRECISE	5178	0	0	0/0	0/0	0/0	0/0	0/0	0/0	0/0	0/0	0/0	0/0	0/0	0/0	0/0	1/1
Chromosome2-1267136	INS	PRECISE	1389	0	0	0/0	0/0	0/0	0/0	0/0	0/0	0/0	0/0	0/0	0/0	0/0	0/0	0/0	1/1
Chromosome2-1267149	DEL	PRECISE	115	0	0	0/0	0/0	0/0	0/0	0/0	0/0	0/0	0/0	0/0	0/0	0/0	0/0	0/0	1/1
Chromosome2-1267370	DEL	PRECISE	98	0	0	0/0	0/0	0/0	0/0	0/1	0/1	0/0	0/0	0/0	0/0	0/0	0/0	0/0	0/0
Chromosome2-1269317	INS	PRECISE	55	0	0	0/0	0/0	0/1	0/0	0/1	0/1	0/0	0/0	0/0	0/0	0/0	0/0	0/0	1/1
Chromosome2-1269750	INS	PRECISE	735	0	0	0/0	0/0	0/0	0/0	0/0	0/0	0/1	0/0	0/0	0/0	0/0	0/0	0/0	0/0
Chromosome2-1270447	DEL	PRECISE	9892	0	0	0/0	0/0	0/1	0/0	0/1	0/1	0/0	0/0	0/0	0/0	0/0	0/0	0/0	0/0
Chromosome2-1271766	DEL	PRECISE	1032	0	0	0/0	0/0	0/0	0/0	0/0	0/0	0/0	0/0	0/0	0/0	0/0	1/1	0/0	0/0
Chromosome2-1272092	INS	PRECISE	7413	0	0	0/0	0/0	0/1	0/0	0/0	0/0	0/0	0/0	0/0	0/0	0/0	0/0	0/0	0/0
Chromosome2-1272524	INS	PRECISE	1110	0	0	0/0	0/0	0/1	0/0	0/0	0/0	0/0	0/0	0/0	0/0	0/0	0/0	0/0	0/0
Chromosome2-1272582	INS	PRECISE	63	0	0	0/0	0/0	0/0	0/0	0/0	0/0	0/0	0/0	0/0	0/0	0/0	1/1	0/0	1/1
Chromosome2-1273875	BND	PRECISE	0	0	0	0/0	0/0	0/0	0/1	0/0	0/0	0/0	0/0	0/0	0/0	0/0	0/0	0/0	0/0
Chromosome2-1273917	INS	PRECISE	3531	0	0	0/0	0/0	1/1	0/0	0/0	0/0	0/0	0/0	0/0	0/0	0/0	0/0	0/0	0/0
Chromosome2-1274065	DEL	PRECISE	657	0	0	0/0	0/0	0/1	0/1	0/1	0/1	0/1	0/1	0/0	0/0	0/0	0/0	0/0	1/1
Chromosome2-1275078	INS	PRECISE	710	0	0	0/0	0/0	0/1	0/0	0/0	0/0	0/0	0/0	0/0	0/0	0/0	0/0	0/0	0/0
Chromosome2-1275836	DEL	PRECISE	626	0	0	0/0	0/0	0/1	0/0	0/0	0/0	0/0	0/0	0/0	0/0	0/0	0/0	0/0	1/1
Chromosome2-1276069	DEL	PRECISE	264	0	0	0/0	0/0	0/1	0/0	0/0	0/0	0/0	0/0	0/0	0/0	0/0	0/0	0/0	1/1
Chromosome2-1277597	INS	PRECISE	973	0	0	0/0	0/0	0/0	0/0	0/0	0/0	0/0	0/0	0/0	0/0	0/0	0/0	0/0	1/1
Chromosome2-1277734	INS	PRECISE	3629	0	0	0/0	0/0	1/1	0/0	0/0	0/0	0/0	0/0	0/0	0/0	0/0	0/0	0/0	0/0
Chromosome2-1278493	INS	PRECISE	91	0	0	0/0	0/0	0/0	0/0	0/0	0/0	0/0	0/0	0/0	0/0	0/0	0/0	0/0	1/1
Chromosome2-1278682	DEL	PRECISE	1591	0.577	0.577	0/0	0/0	1/1	0/0	0/1	0/1	0/0	0/0	0/0	0/0	0/0	0/0	0/0	0/0
Chromosome2-1278682	BND	PRECISE	0	0	0	0/0	0/0	0/0	0/0	0/1	0/1	0/0	0/1	0/0	1/1	0/0	0/0	0/0	0/0
Chromosome2-1278841	DEL	PRECISE	21248572	0	0	0/0	0/0	0/0	0/1	0/1	0/1	0/1	0/1	0/0	1/1	0/0	0/0	0/0	0/0

Chromosome2-1279218	DEL	PRECISE	286	0	0	0/0	0/0	0/1	0/0	0/0	0/0	0/0	0/0	0/0	0/0	0/0	0/0	0/0	0/0
Chromosome2-1279421	BND	PRECISE	0	0	0	0/0	0/0	0/1	0/0	0/0	0/1	0/0	0/0	0/0	0/0	0/0	0/0	0/0	0/0
Chromosome2-1279601	BND	PRECISE	0	0	0	0/0	0/0	0/0	0/0	0/0	0/0	0/0	0/0	0/0	0/0	0/0	0/0	0/0	1/1
Chromosome2-1279601	DEL	PRECISE	21356	0	0	0/0	0/0	1/1	0/0	0/1	0/1	0/0	0/0	0/0	0/0	0/0	0/0	0/0	0/1
Chromosome2-1280196	DEL	PRECISE	838	0	0	0/0	0/0	0/0	0/0	0/0	0/0	0/0	0/0	0/0	0/0	0/0	0/0	0/0	1/1
Chromosome2-1281817	INS	PRECISE	123	0	0	0/0	0/0	0/0	0/0	0/0	0/0	0/0	0/0	0/0	0/0	0/0	0/0	0/0	1/1
Chromosome2-1283274	DEL	PRECISE	85	0	0	0/0	0/0	0/1	0/0	0/1	0/1	0/0	0/0	0/0	0/0	0/0	0/0	0/0	1/1
Chromosome2-1283475	INS	PRECISE	106	0	0	0/0	0/0	0/1	0/0	0/1	0/1	0/0	0/0	0/0	0/0	0/0	0/0	0/0	0/0
Chromosome2-1283535	INS	PRECISE	11701	0	0	0/0	0/0	1/1	0/0	0/0	0/0	0/0	0/0	0/0	0/0	0/0	0/0	0/0	0/0
Chromosome2-1283708	DEL	PRECISE	467	0	0	0/0	0/0	0/0	0/0	0/0	0/0	0/0	0/0	0/0	0/0	0/0	0/0	0/0	1/1
Chromosome2-1286150	INS	PRECISE	102	0	0	0/0	0/0	0/1	0/0	0/1	0/1	0/0	0/0	0/0	0/0	0/0	0/0	0/0	0/0
Chromosome2-1286883	DEL	PRECISE	1362	0	0	0/0	0/0	0/1	0/1	0/1	0/1	0/1	0/1	0/0	0/0	0/0	0/0	0/0	1/1
Chromosome2-1287365	INS	PRECISE	577	0	0	0/0	0/0	0/0	0/0	0/0	0/0	0/1	0/0	0/0	0/0	0/0	0/0	0/0	0/0
Chromosome2-1287799	INS	PRECISE	12578	0	0	0/0	0/0	0/0	0/0	0/0	0/1	0/0	0/0	0/0	0/0	0/0	0/0	0/0	0/0
Chromosome2-1289148	DEL	PRECISE	61	0	0	0/0	0/0	0/0	0/0	0/1	0/0	0/0	0/0	0/0	0/0	0/0	0/0	0/0	0/0
Chromosome2-1289479	INS	PRECISE	196	0	0.577	0/0	0/0	0/0	0/1	0/0	0/0	0/1	0/1	0/0	0/0	0/0	0/0	0/0	0/0
Chromosome2-1289874	DEL	PRECISE	1106	0	0	0/0	0/0	0/1	0/0	0/1	0/1	0/0	0/0	0/0	0/0	0/0	0/0	0/0	1/1
Chromosome2-1290418	INS	PRECISE	1866	0	0	0/0	0/0	0/0	0/0	0/0	0/0	0/1	0/0	0/0	0/0	0/0	0/0	0/0	0/0
Chromosome2-1290630	DEL	PRECISE	132	0	0	0/0	0/0	0/1	0/1	0/1	0/1	1/1	0/1	0/0	0/0	0/0	0/0	0/0	1/1
Chromosome2-1291444	INS	PRECISE	136	0	0	0/0	0/0	0/0	0/0	0/0	0/0	0/0	0/0	0/0	0/0	0/0	0/0	0/0	1/1
Chromosome2-1292945	DEL	PRECISE	141	0	0	0/0	0/0	0/0	0/0	0/0	0/0	0/1	0/1	0/0	0/0	0/0	0/0	0/0	0/0
Chromosome2-1294557	BND	PRECISE	0	0	0	0/0	0/1	0/0	0/0	0/0	0/0	0/0	0/0	0/0	0/0	0/0	0/0	0/0	0/0
Chromosome2-1294902	INS	PRECISE	8965	0	0	0/0	0/0	0/1	0/0	0/0	0/0	0/0	0/0	0/0	0/0	0/0	0/0	0/0	0/0
Chromosome2-1294903	BND	PRECISE	0	0	0	0/0	0/0	0/1	0/0	0/0	0/0	0/0	0/0	0/0	0/0	0/0	0/0	0/0	0/0
Chromosome2-1298116	DEL	PRECISE	139	0	0	0/0	0/0	0/0	0/1	0/0	0/0	0/0	0/0	0/0	0/0	0/0	0/0	0/0	0/0
Chromosome2-1298655	DEL	PRECISE	51	0	0	0/0	0/0	0/0	0/0	0/0	0/0	0/0	0/0	0/0	0/0	0/0	0/0	0/0	1/1
Chromosome2-1299717	INS	PRECISE	626	0	0.707	0/0	0/0	0/0	0/0	0/1	0/1	0/0	0/0	0/0	0/0	0/0	0/0	0/0	0/0
Chromosome2-1299795	INS	PRECISE	132	0	0	0/0	0/0	0/0	0/0	0/0	0/0	0/0	0/0	0/0	0/0	0/0	0/0	0/0	1/1
Chromosome2-1301155	INS	PRECISE	2422	0	0	0/0	0/0	0/0	0/0	0/0	0/0	0/0	0/0	0/0	0/0	0/0	0/0	0/0	1/1
Chromosome2-1301371	INS	PRECISE	9692	0	0	0/0	0/0	1/1	0/0	0/0	0/0	0/0	0/0	0/0	0/0	0/0	0/0	0/0	0/0
Chromosome2-1301371	BND	PRECISE	0	0	0	0/0	0/0	0/1	0/0	0/0	0/0	0/0	0/0	0/0	0/0	0/0	0/0	0/0	0/0
Chromosome2-1301412	INS	PRECISE	5972	0	0	0/0	0/0	0/0	0/0	0/1	1/1	0/0	0/0	0/0	0/0	0/0	0/0	0/0	0/0
Chromosome2-1302104	DEL	PRECISE	6891	0	0	0/0	0/0	0/0	0/0	0/0	0/0	0/0	0/0	0/0	0/0	0/0	0/0	0/0	1/1
Chromosome2-1302459	DEL	PRECISE	1372	0	0	0/0	0/0	0/0	0/0	0/1	0/0	0/0	0/0	0/0	0/0	0/0	0/0	0/0	0/0
Chromosome2-1303046	DEL	PRECISE	77	0	0	0/0	0/0	0/0	0/0	0/0	0/0	0/0	0/0	0/0	0/0	0/0	0/0	0/0	1/1
Chromosome2-1304418	BND	PRECISE	0	0	0	0/0	0/0	0/0	0/0	0/0	0/0	0/1	0/0	0/0	0/0	0/0	0/0	0/0	0/0
Chromosome2-1304682	INS	PRECISE	925	0	0.577	0/0	0/0	0/0	0/0	0/0	0/0	0/0	0/0	0/0	0/1	1/1	1/1	0/0	0/0
Chromosome2-1304912	INS	PRECISE	58	0	0	0/0	0/0	0/1	0/0	0/0	0/0	0/0	0/0	0/0	0/0	0/0	0/0	0/0	0/0
Chromosome2-1305319	DEL	PRECISE	6522	0	0	0/0	0/0	0/0	0/0	0/0	0/0	0/0	0/0	0/0	0/0	0/0	0/0	0/0	1/1
Chromosome2-1306044	BND	PRECISE	0	0	0	0/0	0/0	0/0	0/0	0/0	0/0	0/0	0/0	0/0	0/0	0/0	0/0	0/0	0/1
Chromosome2-1306131	DEL	PRECISE	1445	0	0	0/0	0/0	0/0	0/0	0/0	0/0	0/0	0/0	0/0	0/0	1/1	0/0	0/0	1/1
Chromosome2-1306255	INS	PRECISE	136	0	0	0/0	0/0	0/1	0/0	0/0	0/0	0/0	0/0	0/0	0/0	0/0	0/0	0/0	0/0
Chromosome2-1309559	DEL	PRECISE	58	0	0	0/0	0/0	0/1	0/0	0/0	0/0	0/0	0/0	0/0	0/0	0/0	0/0	0/0	0/0
Chromosome2-1309856	DEL	PRECISE	190	0	0	0/0	0/0	0/1	0/0	0/0	0/0	0/0	0/0	0/0	0/0	0/0	0/0	0/0	0/0
Chromosome2-1310901	INS	PRECISE	52	0	0.354	0/0	0/0	0/1	0/1	0/1	0/1	0/1	0/1	0/0	0/0	0/0	0/0	0/0	1/1
Chromosome2-1314146	INS	PRECISE	1391	0	0	0/0	0/0	0/1	0/0	0/0	0/0	0/0	0/0	0/0	0/0	0/0	0/0	0/0	0/0
Chromosome2-1314166	INS	PRECISE	65	0	0	0/0	0/0	0/1	0/0	0/1	0/1	0/0	0/0	0/0	0/0	0/0	0/0	0/0	0/0
Chromosome2-1316641	DEL	PRECISE	60	0	0	0/0	0/0	0/1	0/0	0/1	0/1	0/0	0/0	0/0	0/0	0/0	0/0	0/0	0/0
Chromosome2-1319505	INS	PRECISE	100	0	0	0/0	0/0	0/1	0/1	0/1	0/1	0/1	0/1	0/0	0/0	0/0	0/0	0/0	1/1
Chromosome2-1319795	DEL	PRECISE	50	0	0	0/0	0/0	0/1	0/0	0/1	0/1	0/0	0/0	0/0	0/0	0/0	0/0	0/0	0/0
Chromosome2-1319864	INS	PRECISE	160	0	0	0/0	0/0	0/0	0/0	0/1	0/1	0/0	0/0	0/0	0/0	0/0	0/0	0/0	0/0
Chromosome2-1320759	INS	PRECISE	627	0	0	0/0	0/0	0/1	0/0	0/0	0/0	0/0	0/0	0/0	0/0	0/0	0/0	0/0	0/0
Chromosome2-1321043	INS	PRECISE	959	0	0	0/0	0/0	0/0	0/0	0/0	0/0	0/0	0/0	0/0	0/0	0/0	0/0	0/0	1/1
Chromosome2-1322170	DEL	PRECISE	1063	0	0	0/0	0/0	0/1	0/0	0/0	0/0	0/0	0/0	0/0	0/0	0/0	0/0	0/0	0/0
Chromosome2-1323765	INS	PRECISE	58	0	0	0/0	0/0	0/1	0/0	0/0	0/0	0/0	0/0	0/0	0/0	0/0	0/0	0/0	0/0
Chromosome2-1324094	DEL	PRECISE	8587	0	0	0/0	0/0	0/1	0/0	0/0	0/0	0/0	0/0	0/0	0/0	0/0	0/0	0/0	0/0
Chromosome2-1328285	INS	PRECISE	2238	0	0	0/0	0/0	0/0	0/0	0/0	0/0	0/0	0/0	0/0	0/0	0/0	0/0	0/0	1/1
Chromosome2-1330013	DEL	PRECISE	12356	0	0	0/0	0/0	0/1	1/1	0/1	0/1	1/1	0/1	0/0	0/0	0/0	0/0	0/0	1/1
Chromosome2-1332475	DEL	PRECISE	1459	0	0	0/0	0/0	0/0	0/0	0/1	0/1	0/0	0/0	0/0	0/0	0/0	0/0	0/0	0/0
Chromosome2-1333389	DEL	PRECISE	72	0	0	0/0	0/0	0/0	0/0	0/1	0/1	0/0	0/0	0/0	0/0	0/0	0/0	0/0	0/0
Chromosome2-1335129	INS	PRECISE	121	0	0	0/0	0/0	0/1	0/1	0/1	0/1	1/1	0/1	0/0	0/0	0/0	0/0	0/0	1/1
Chromosome2-1335464	DEL	PRECISE	164	0	0	0/0	0/0	0/0	0/0	0/0	0/0	0/0	0/0	0/0	0/0	0/0	0/0	0/0	1/1
Chromosome2-1335815	INS	PRECISE	514	0	0.577	0/0	0/0	0/1	0/0	0/1	0/1	0/0	0/0	0/0	0/0	0/0	0/0	0/0	0/0
Chromosome2-1336100	DEL	PRECISE	110	0	0	0/0	0/0	0/1	0/0	0/1	0/1	0/0	0/0	0/0	0/0	0/0	0/0	0/0	0/0
Chromosome2-1336292	DEL	PRECISE	219	0	0	0/0	0/0	0/1	0/0	0/0	0/0	0/0	0/0	0/0	0/0	0/0	0/0	0/0	0/0
Chromosome2-1341172	DEL	PRECISE	66	0	0	0/0	0/0	0/0	0/0	0/0	0/0	0/1	0/1	0/0	0/0	0/0	0/0	0/0	0/0
Chromosome2-1342825	DEL	PRECISE	411	0	0	0/0	0/0	0/1	0/0	0/1	0/1	0/0	0/0	0/0	0/0	0/0	0/0	0/0	0/0
Chromosome2-1342847	INS	PRECISE	4881	0	0	0/0	0/0	0/0	0/0	0/0	0/0	0/0	0/0	0/0	0/0	0/0	0/0	0/0	1/1
Chromosome2-1345481	INS	PRECISE	62	0	0.5	0/0	0/0	0/1	0/0	0/1	0/1	0/0	0/0	0/0	0/0	0/0	0/0	0/0	1/1
Chromosome2-1349666	INS	PRECISE	1368	0	0	0/0	0/0	0/1	0/0	0/0	0/0	0/0	0/0	0/0	0/0	0/0	0/0	0/0	0/0
Chromosome2-1350791	DEL	PRECISE	345	0	0	0/0	0/0	0/1	0/0	0/1	0/1	0/0	0/0	0/0	0/0	0/0	0/0	0/0	0/0
Chromosome2-1351638	DEL	PRECISE	64	0	0	0/0	0/0	0/0	0/0	0/0	0/0	0/0	0/0	0/0	0/0	0/0	0/0	0/0	1/1
Chromosome2-1352374	DEL	PRECISE	12553	0	0	0/0	0/0	0/1	0/1	0/1	0/1	1/1	0/1	0/0	0/0	0/0	0/0	0/0	1/1

Chromosome2-1352375 BND	PRECISE	0	0	0	0/0	0/0	0/0	0/0	0/0	0/0	0/0	0/0	0/0	0/0	0/1	0/0	0/0	0/0
Chromosome2-1353630 BND	PRECISE	0	0	0	0/0	0/0	0/1	0/0	0/0	0/0	0/0	0/1	0/0	0/0	0/0	0/0	0/0	0/0
Chromosome2-1354069 INS	PRECISE	1857	0	0.577	0/0	0/0	0/1	0/0	0/1	0/1	0/0	0/0	0/0	0/0	0/0	0/0	0/0	0/0
Chromosome2-1355239 DEL	PRECISE	57	0	0	0/0	0/0	0/0	0/0	0/1	0/1	0/0	0/0	0/0	0/0	0/0	0/0	0/0	0/0
Chromosome2-1357462 DEL	PRECISE	91	0.378	0.378	0/0	0/0	0/1	0/1	0/1	0/1	1/1	0/1	0/0	0/0	0/0	0/0	0/0	1/1
Chromosome2-1359606 DEL	PRECISE	50	0	0	0/0	0/0	0/0	0/0	0/0	0/0	0/0	0/0	0/0	0/0	0/0	0/0	0/0	1/1
Chromosome2-1360526 DEL	PRECISE	51	0	0	0/0	0/0	0/0	0/0	0/0	0/0	0/0	0/0	0/0	0/0	0/0	0/0	0/0	1/1
Chromosome2-1360611 INS	PRECISE	495	0	0	0/0	0/0	0/0	0/0	0/0	0/0	0/0	0/0	0/0	0/0	0/0	0/0	0/0	1/1
Chromosome2-1360930 DEL	PRECISE	12364	0	0	0/0	0/0	0/1	0/0	0/1	0/0	0/0	0/0	0/0	0/0	0/0	0/0	0/0	1/1
Chromosome2-1365412 DEL	PRECISE	502	0	0	0/0	0/0	0/1	0/0	0/0	0/0	0/0	0/0	0/0	0/0	0/0	0/0	0/0	0/0
Chromosome2-1365824 INS	PRECISE	477	0	0	0/0	0/0	0/0	0/0	0/0	0/0	0/0	0/0	0/0	0/0	0/0	0/0	0/0	1/1
Chromosome2-1367070 DEL	PRECISE	85	0	0	0/0	0/0	0/1	0/0	0/0	0/0	0/0	0/0	0/0	0/0	0/0	0/0	0/0	0/0
Chromosome2-1367070 INS	PRECISE	2085	0	0	0/0	0/0	0/1	0/0	0/0	0/0	0/0	0/0	0/0	0/0	0/0	0/0	0/0	0/0
Chromosome2-1371204 INS	PRECISE	12541	0	0	0/0	0/0	0/1	0/0	0/0	0/0	0/0	0/0	0/0	0/0	0/0	0/0	0/0	0/0
Chromosome2-1371888 DEL	PRECISE	128	0	0	0/0	0/0	0/0	0/0	0/1	0/1	0/0	0/0	0/0	0/0	0/0	0/0	0/0	0/0
Chromosome2-1372408 DEL	PRECISE	98	0	0	0/0	0/0	0/1	0/0	0/1	0/1	0/0	0/0	0/0	0/0	0/0	0/0	0/0	0/0
Chromosome2-1372909 DEL	PRECISE	598	0	0	0/0	0/0	0/0	0/0	0/0	0/0	0/1	0/0	0/0	0/0	0/0	0/0	0/0	0/0
Chromosome2-1373645 DEL	PRECISE	14439	0	0	0/0	0/0	0/0	0/0	0/0	0/0	0/1	0/0	0/0	0/0	0/0	0/0	0/0	0/0
Chromosome2-1378447 INS	PRECISE	1372	0	0	0/0	0/0	0/0	0/0	0/0	0/0	0/0	0/0	0/0	0/0	0/0	0/0	0/0	1/1
Chromosome2-1378552 DUP	PRECISE	17158292	0	0	0/0	0/0	0/0	0/0	0/0	0/0	0/0	0/0	0/0	0/0	0/0	0/1	0/0	0/0
Chromosome2-1379692 DEL	PRECISE	1370	0	0	0/0	0/0	0/0	0/0	0/0	0/0	0/0	0/0	0/0	0/0	0/0	0/0	0/0	1/1
Chromosome2-1381557 DEL	PRECISE	963	0	0	0/0	0/0	0/1	0/0	0/1	1/1	0/0	0/0	0/0	0/0	0/0	0/0	0/0	1/1
Chromosome2-1382436 DEL	PRECISE	1525	0	0	0/0	0/0	0/0	0/0	0/0	0/0	0/0	0/0	0/0	0/0	0/0	0/0	0/0	1/1
Chromosome2-1383419 DEL	PRECISE	66	0	0	0/0	0/0	0/1	0/0	0/1	0/1	0/0	0/0	0/0	0/0	0/0	0/0	0/0	0/0
Chromosome2-1384255 DEL	PRECISE	51	0	0	0/0	0/0	0/1	0/0	0/1	0/1	0/0	0/0	0/0	0/0	0/0	0/0	0/0	0/0
Chromosome2-1385350 DEL	PRECISE	58	0	0	0/0	0/0	0/1	0/0	0/1	0/1	0/0	0/0	0/0	0/0	0/0	0/0	0/0	0/0
Chromosome2-1385909 INS	PRECISE	1360	0	0	0/0	0/0	0/1	0/0	0/0	0/0	0/0	0/0	0/0	0/0	0/0	0/0	0/0	0/0
Chromosome2-1386383 INS	PRECISE	276	0	0	0/0	0/0	0/1	0/0	0/1	0/1	0/0	0/0	0/0	0/0	0/0	0/0	0/0	0/0
Chromosome2-1387658 DEL	PRECISE	7612	0	0	0/0	0/0	0/0	0/0	0/0	0/0	0/1	0/0	0/0	0/0	0/0	0/0	0/0	0/0
Chromosome2-1390016 INS	PRECISE	467	0	0	0/0	0/0	0/1	0/0	0/0	0/0	0/0	0/0	0/0	0/0	0/0	0/0	0/0	0/0
Chromosome2-1390409 BND	PRECISE	0	0	0	0/0	0/0	0/0	0/0	0/0	0/0	0/0	0/0	0/0	0/0	0/0	0/0	0/0	0/1
Chromosome2-1390529 INS	PRECISE	1376	0	0	0/0	0/0	0/0	0/0	0/0	0/0	0/0	0/0	0/0	0/0	0/0	0/0	0/0	1/1
Chromosome2-1391162 INS	PRECISE	50	0	0	0/0	0/0	0/1	0/0	0/1	0/1	0/0	0/0	0/0	0/0	0/0	0/0	0/0	0/0
Chromosome2-1391246 DEL	PRECISE	9226	0	0	0/0	0/0	0/1	0/0	0/1	1/1	0/0	0/0	0/0	0/0	0/0	0/0	0/0	0/0
Chromosome2-1392163 INS	PRECISE	1949	0	0	0/0	0/0	0/0	0/0	0/0	0/0	0/0	0/0	0/0	0/0	0/0	0/0	0/0	1/1
Chromosome2-1395400 DEL	PRECISE	1388	0	0	0/0	0/0	0/1	0/1	0/1	0/1	1/1	0/1	0/0	0/0	0/0	0/0	0/0	1/1
Chromosome2-1395913 DEL	PRECISE	161	0	0	0/0	0/0	0/1	0/0	0/0	0/0	0/0	0/0	0/0	0/0	0/0	0/0	0/0	0/0
Chromosome2-1396034 DEL	PRECISE	51	0	0	0/0	0/0	0/0	0/0	0/1	0/1	0/0	0/0	0/0	0/0	0/0	0/0	0/0	0/0
Chromosome2-1396434 INS	PRECISE	301	0	0.5	0/0	0/0	0/1	0/0	0/1	0/1	0/0	0/0	0/0	0/0	0/0	0/0	0/0	1/1
Chromosome2-1396481 INS	PRECISE	7528	0	0	0/0	0/0	0/0	0/0	0/1	0/0	0/0	0/0	0/0	0/0	0/0	0/0	0/0	0/0
Chromosome2-1396935 DEL	PRECISE	5447	0	0	0/0	0/0	0/0	0/0	0/0	0/0	0/0	0/0	0/0	0/0	0/0	0/0	0/0	1/1
Chromosome2-1401634 INS	PRECISE	70	0	0	0/0	0/0	0/1	0/1	0/1	0/1	1/1	0/1	0/0	0/0	0/0	0/0	0/0	1/1
Chromosome2-1405317 DEL	PRECISE	2313	0	0	0/0	0/0	0/0	0/0	0/0	0/0	0/0	0/0	0/0	0/0	0/0	0/0	0/0	1/1
Chromosome2-1408847 INS	PRECISE	62	0	0	0/0	0/0	0/0	0/0	0/0	0/0	0/0	0/0	0/1	0/0	0/0	1/1	1/1	0/0
Chromosome2-1409474 DEL	PRECISE	1656	0	0	0/0	0/0	0/0	0/0	0/0	0/0	0/0	0/0	0/0	0/0	0/0	0/0	0/0	1/1
Chromosome2-1410831 INS	PRECISE	1118	0	0	0/0	0/0	0/0	0/0	0/0	0/0	0/0	0/0	0/0	0/0	0/0	0/0	0/0	1/1
Chromosome2-1411232 DEL	PRECISE	2207	0	0	0/0	0/0	0/0	0/0	0/0	0/0	0/0	0/0	0/0	0/0	0/0	0/0	0/0	1/1
Chromosome2-1411645 INS	PRECISE	106	0	0.707	0/0	0/0	0/0	0/0	0/0	0/0	0/0	0/0	0/1	0/0	0/0	0/0	1/1	0/0
Chromosome2-1411819 DEL	PRECISE	56	0	0	0/0	0/0	0/0	0/0	0/0	0/0	0/0	0/0	0/1	0/0	0/0	0/0	1/1	0/0
Chromosome2-1412371 DEL	PRECISE	245	0	0	0/0	0/0	0/0	0/0	0/0	0/0	0/0	0/0	0/0	0/0	0/0	0/0	0/0	1/1
Chromosome2-1412976 DEL	PRECISE	71	0	0	0/0	0/0	0/0	0/0	0/0	0/0	0/0	0/0	0/0	0/0	0/0	0/0	0/0	1/1
Chromosome2-1418345 INS	PRECISE	82	0	0	0/0	0/0	0/1	0/0	0/0	0/0	0/0	0/0	0/0	0/0	0/0	0/0	0/0	0/0
Chromosome2-1418683 DEL	PRECISE	57	0	0	0/0	0/0	0/1	0/0	0/0	0/1	0/0	0/0	0/0	0/0	0/0	0/0	0/0	0/0
Chromosome2-1419569 BND	PRECISE	0	0	0	0/0	0/0	0/0	0/0	0/0	0/0	0/1	0/1	0/0	0/0	0/0	0/0	0/0	0/0
Chromosome2-1419922 INS	PRECISE	159	0	0	0/0	0/0	0/0	0/0	0/0	0/0	0/1	0/1	0/0	0/0	0/0	0/0	0/0	0/0
Chromosome2-1420671 INS	PRECISE	111	0	0	0/0	0/0	0/0	0/0	0/0	0/0	0/1	0/1	0/0	0/0	0/0	0/0	0/0	0/0
Chromosome2-1420807 INS	PRECISE	69	0	0	0/0	0/0	0/0	0/0	0/0	0/0	0/0	0/1	0/0	0/0	0/0	0/0	0/0	0/0
Chromosome2-1421440 DEL	PRECISE	51	0	0	0/0	0/0	0/0	0/0	0/0	0/0	0/0	0/1	0/0	0/0	0/0	0/0	0/0	0/0
Chromosome2-1422008 BND	PRECISE	0	0	0	0/0	0/0	0/0	0/0	0/0	0/0	0/1	0/0	0/0	0/0	0/0	0/0	0/0	0/0
Chromosome2-1422008 BND	PRECISE	0	0	0	0/0	0/0	0/0	0/0	0/0	0/0	0/1	0/0	0/0	0/0	0/0	0/0	0/0	0/0
Chromosome2-1422008 BND	PRECISE	0	0	0	0/0	0/0	0/0	0/0	0/0	0/0	0/1	0/1	0/0	0/0	0/0	0/0	0/0	0/0
Chromosome2-1422009 BND	PRECISE	0	0	0	0/0	0/0	0/1	0/0	0/0	0/0	0/0	0/0	0/0	0/0	0/0	0/0	0/0	0/0
Chromosome2-1423455 INS	PRECISE	63	0	0	0/0	0/0	0/0	0/0	0/0	0/0	0/0	0/0	0/0	0/0	0/0	0/0	0/0	1/1
Chromosome2-1424460 DEL	PRECISE	12467	0	0	0/0	0/0	0/0	0/0	0/0	0/0	0/0	0/0	0/0	0/0	0/0	0/0	0/0	1/1
Chromosome2-1426575 DEL	PRECISE	12561	0	0	0/0	0/0	0/0	0/1	0/0	0/0	1/1	0/1	0/0	0/0	0/0	0/0	0/0	1/1
Chromosome2-1428859 DEL	PRECISE	5419	0	0	0/0	0/0	0/0	0/0	0/1	0/1	0/0	0/0	0/0	0/0	0/0	0/0	0/0	0/0
Chromosome2-1429130 INS	PRECISE	176	0	0	0/0	0/0	0/0	0/0	0/0	0/0	0/0	0/0	0/0	0/0	0/0	0/0	0/0	1/1
Chromosome2-1429335 DEL	PRECISE	183	0	0	0/0	0/0	0/0	0/0	0/0	0/0	0/1	0/0	0/0	0/0	0/0	0/0	0/0	0/0
Chromosome2-1429564 DEL	PRECISE	52	0	0	0/0	0/0	0/1	0/0	0/0	0/0	0/0	0/0	0/0	0/0	0/0	0/0	0/0	0/0
Chromosome2-1431137 DEL	PRECISE	644	0	0	0/0	0/0	0/0	0/0	0/0	0/0	0/0	0/0	0/0	0/0	0/0	0/0	1/1	0/0
Chromosome2-1431535 INS	PRECISE	1379	0	0	0/0	0/0	0/0	0/0	0/0	0/0	0/0	0/0	0/0	0/0	0/0	0/0	1/1	0/0
Chromosome2-1431698 INS	PRECISE	12078	0	0	0/0	0/0	0/0	0/0	0/0	0/1	0/0	0/0	0/0	0/0	0/0	0/0	0/0	0/0
Chromosome2-1431949 BND	PRECISE	0	0	0	0/0	0/0	0/0	0/0	0/1	0/0	0/0	0/0	0/0	0/0	0/0	0/0	0/0	0/0

Chromosome2-1431950 BND	PRECISE	0	0	0	0/0	0/1	0/0	0/0	0/0	0/0	0/0	0/0	0/0	0/1	0/0	0/0	0/0	0/1
Chromosome2-1432090 DEL	PRECISE	171	0	0	0/0	0/0	0/1	0/0	0/0	0/0	0/0	0/0	0/0	0/0	0/0	0/0	0/0	0/0
Chromosome2-1432408 INS	PRECISE	861	0	0	0/0	0/0	0/0	0/0	0/0	0/0	0/0	0/0	0/0	0/0	0/0	0/0	0/0	1/1
Chromosome2-1432877 INS	PRECISE	9590	0	0	0/0	0/0	0/0	1/1	0/0	0/0	0/0	0/0	0/0	0/0	0/0	0/0	0/0	0/0
Chromosome2-1434144 INS	PRECISE	159	0	0	0/0	0/0	0/0	0/0	0/0	0/0	0/0	0/0	0/0	0/0	0/0	0/0	0/0	1/1
Chromosome2-1437858 INV	PRECISE	122934	0	0	0/0	0/0	0/0	0/0	0/1	0/1	0/0	0/0	0/0	0/0	0/0	0/0	0/0	0/0
Chromosome2-1439265 INV	PRECISE	108869	0	0	0/0	0/0	0/0	0/0	0/1	0/1	0/0	0/0	0/0	0/0	0/0	0/0	0/0	0/0
Chromosome2-1440772 INS	PRECISE	63	0	0	0/0	0/0	0/1	0/0	0/1	0/1	0/0	0/0	0/0	0/0	0/0	0/0	0/0	1/1
Chromosome2-1441745 INS	PRECISE	533	0	0.5	0/0	0/0	0/1	0/0	0/1	0/1	0/0	0/0	0/0	0/0	0/0	0/0	0/0	1/1
Chromosome2-1442781 DEL	PRECISE	2206	0	0	0/0	0/0	0/1	0/0	0/0	0/0	0/0	0/0	0/0	0/0	0/0	0/0	0/0	0/0
Chromosome2-1443154 DEL	PRECISE	172	0	0	0/0	0/0	0/0	0/0	0/0	0/0	0/0	0/0	0/0	0/0	0/0	0/0	0/0	1/1
Chromosome2-1443539 DEL	PRECISE	2666	0	0	0/0	0/0	0/1	0/0	0/1	0/1	0/0	0/0	0/0	0/0	0/0	0/0	0/0	0/0
Chromosome2-1443996 DEL	PRECISE	7874	0	0	0/0	0/0	0/0	0/0	0/0	0/0	0/0	0/0	0/0	0/0	0/0	0/0	0/0	1/1
Chromosome2-1447324 DEL	PRECISE	51	0	0	0/0	0/0	0/0	0/1	0/0	0/0	1/1	0/1	0/0	0/0	0/0	0/0	0/0	0/0
Chromosome2-1455463 INS	PRECISE	12982	0	0	0/0	0/0	1/1	0/0	0/0	0/0	0/0	0/0	0/0	0/0	0/0	0/0	0/0	0/0
Chromosome2-1457274 INS	PRECISE	276	0	0	0/0	0/0	0/1	0/0	0/0	0/0	0/0	0/0	0/0	0/0	0/0	0/0	0/0	0/0
Chromosome2-1459302 DEL	PRECISE	137	0	0	0/0	0/0	0/0	0/0	0/1	0/1	0/0	0/0	0/0	0/0	0/0	0/0	0/0	0/0
Chromosome2-1460074 DEL	PRECISE	79	0	0	0/0	0/0	0/1	0/0	0/1	1/1	0/0	0/0	0/0	0/0	0/0	0/0	0/0	1/1
Chromosome2-1460383 DEL	PRECISE	57	0	0	0/0	0/0	0/0	0/1	0/0	0/0	1/1	0/1	0/0	0/0	0/0	0/0	0/0	0/0
Chromosome2-1461106 INS	PRECISE	10325	0	0	0/0	0/0	0/0	0/0	0/0	0/0	0/0	0/0	0/0	0/0	0/0	0/0	0/0	1/1
Chromosome2-1461106 BND	PRECISE	0	0	0	0/0	0/0	0/0	0/0	0/0	0/0	0/0	0/0	0/0	0/0	0/0	0/0	0/0	0/1
Chromosome2-1461754 INS	PRECISE	80	0	0	0/0	0/0	0/1	0/0	0/1	0/1	0/0	0/0	0/0	0/0	0/0	0/0	0/0	1/1
Chromosome2-1463480 DEL	PRECISE	59	0	0	0/0	0/0	0/0	0/1	0/0	0/0	0/1	0/1	0/0	0/0	0/0	0/0	0/0	0/0
Chromosome2-1463925 INS	PRECISE	3772	0	0	0/0	0/0	0/0	0/0	0/0	0/1	0/0	0/0	0/0	0/0	0/0	0/0	0/0	0/0
Chromosome2-1464046 INS	PRECISE	1375	0	0	0/0	0/0	0/0	0/0	0/0	0/0	0/0	0/0	0/0	0/0	0/0	0/0	0/0	1/1
Chromosome2-1467279 INS	PRECISE	1364	0	0	0/0	0/0	0/0	0/0	0/0	0/0	0/0	0/0	0/0	0/0	0/0	0/0	0/0	1/1
Chromosome2-1469420 INS	PRECISE	6661	0	0	0/0	0/0	1/1	0/0	0/0	0/0	0/0	0/0	0/0	0/0	0/0	0/0	0/0	0/0
Chromosome2-1471865 DEL	PRECISE	1033	0	0	0/0	0/0	0/0	0/0	0/0	0/0	0/0	0/0	0/0	0/0	0/0	0/0	0/0	1/1
Chromosome2-1472553 DEL	PRECISE	17080	0	0	0/0	0/0	0/0	0/0	0/0	0/0	0/0	0/0	0/0	0/0	0/0	0/0	0/0	1/1
Chromosome2-1473599 DEL	PRECISE	72	0	0	0/0	0/0	0/1	0/0	0/1	0/1	0/1	0/0	0/0	0/0	0/0	0/0	0/0	0/0
Chromosome2-1473713 DEL	PRECISE	402	0	0	0/0	0/0	0/1	0/0	0/0	0/0	0/0	0/0	0/0	0/0	0/0	0/0	0/0	0/0
Chromosome2-1474666 DEL	PRECISE	6933	0	0	0/0	0/0	0/1	0/0	0/1	0/1	0/0	0/0	0/0	0/0	0/0	0/0	0/0	1/1
Chromosome2-1474667 BND	PRECISE	0	0	0	0/1	0/0	0/0	0/0	0/0	0/0	0/0	0/0	0/0	0/0	0/0	0/0	0/0	0/0
Chromosome2-1475359 BND	PRECISE	0	0	0	0/1	0/0	0/0	0/0	0/0	0/0	0/0	0/0	0/0	0/0	0/0	0/0	0/0	0/0
Chromosome2-1475576 INS	PRECISE	12769	0	0	0/0	0/0	0/0	0/0	0/0	0/0	0/1	0/0	0/0	0/0	0/0	0/0	0/0	0/0
Chromosome2-1475877 INS	PRECISE	62	0	0	0/0	0/0	0/1	0/0	0/1	0/1	0/0	0/0	0/0	0/0	0/0	0/0	0/0	0/0
Chromosome2-1477644 INS	PRECISE	1791	0	0	0/0	0/0	0/1	0/0	0/0	0/0	0/0	0/0	0/0	0/0	0/0	0/0	0/0	0/0
Chromosome2-1478007 DEL	PRECISE	136	0	0	0/0	0/0	0/0	0/0	0/0	0/0	0/0	0/0	0/0	0/0	0/0	0/0	0/0	1/1
Chromosome2-1478399 INS	PRECISE	1985	0	0	0/0	0/0	0/0	0/0	0/0	0/0	0/0	0/0	0/0	0/0	0/0	0/0	0/0	1/1
Chromosome2-1480934 INS	PRECISE	9768	0	0	0/0	0/0	0/0	0/0	0/0	0/0	0/0	0/0	0/0	0/0	0/0	0/0	0/0	1/1
Chromosome2-1480934 BND	PRECISE	0	0	0	0/0	0/0	0/0	0/0	0/0	0/0	0/0	0/0	0/0	0/0	0/0	0/0	0/0	1/1
Chromosome2-1480934 BND	PRECISE	0	0	0	0/0	0/0	0/0	0/0	0/0	0/0	0/0	0/0	0/0	0/0	0/0	0/0	0/0	1/1
Chromosome2-1481196 INS	PRECISE	852	0	0	0/0	0/0	0/0	0/0	0/0	0/0	0/0	0/0	0/0	0/0	0/0	0/0	0/0	1/1
Chromosome2-1482128 DEL	PRECISE	5203	0	0	0/0	0/0	0/0	0/0	0/0	0/0	0/0	0/0	0/0	0/0	0/0	0/0	0/0	1/1
Chromosome2-1482863 INS	PRECISE	325	0	0	0/0	0/0	0/1	0/0	0/0	0/0	0/0	0/0	0/0	0/0	0/0	0/0	0/0	0/0
Chromosome2-1484508 DEL	PRECISE	927	0	0	0/0	0/0	0/0	0/0	0/0	0/0	0/0	0/0	0/0	0/0	0/0	0/0	0/0	0/1
Chromosome2-1486418 BND	PRECISE	0	0	0	0/0	0/0	0/0	0/0	0/1	0/0	0/0	0/0	0/0	0/0	0/0	0/0	0/0	0/0
Chromosome2-1488256 INS	PRECISE	54	0	0	0/0	0/0	0/1	0/0	0/1	0/1	0/0	0/0	0/0	0/0	0/0	0/0	0/0	0/0
Chromosome2-1491460 INV	PRECISE	1101	0	0	0/0	0/0	0/0	0/1	0/0	0/0	0/0	0/0	0/0	0/0	0/0	0/0	0/0	0/0
Chromosome2-1491745 DEL	PRECISE	483	0	0	0/0	0/0	0/0	0/1	0/0	0/0	0/0	0/0	0/0	0/0	0/0	0/0	0/0	0/0
Chromosome2-1492585 DEL	PRECISE	1551	0	0	0/0	0/0	0/0	0/0	1/1	0/1	0/0	0/0	0/0	0/0	0/0	0/0	0/0	0/0
Chromosome2-1492916 DEL	PRECISE	332	0	0	0/0	0/0	0/0	0/0	0/1	0/1	0/0	0/0	0/0	0/0	0/0	0/0	0/0	0/0
Chromosome2-1493107 INS	PRECISE	1021	0	0.577	0/0	0/0	0/1	0/0	0/1	0/1	0/0	0/0	0/0	0/0	0/0	0/0	0/0	0/0
Chromosome2-1493697 DEL	PRECISE	387	0	0	0/0	0/0	0/1	0/0	0/1	0/1	0/0	0/0	0/0	0/0	0/0	0/0	0/0	0/0
Chromosome2-1493860 DEL	PRECISE	65	0	0	0/0	0/0	0/0	0/0	0/0	0/0	0/0	0/0	0/0	0/0	0/0	0/0	0/0	1/1
Chromosome2-1493896 INS	PRECISE	178	0	0	0/0	0/0	0/1	0/0	0/1	0/1	0/0	0/0	0/0	0/0	0/0	0/0	0/0	0/0
Chromosome2-1494038 DEL	PRECISE	140	0	0	0/0	0/0	0/0	0/0	0/0	0/0	0/0	0/0	0/0	0/0	0/0	0/0	0/0	1/1
Chromosome2-1494676 BND	PRECISE	0	0	0	0/0	0/0	0/0	0/0	0/0	0/1	0/0	0/0	0/0	0/0	0/0	0/0	0/0	0/0
Chromosome2-1494730 DEL	PRECISE	74	0	0	0/0	0/0	0/1	0/0	0/1	0/1	0/0	0/0	0/0	0/0	0/0	0/0	0/0	1/1
Chromosome2-1495036 DEL	PRECISE	74	0	0	0/0	0/0	0/0	0/0	0/1	1/1	0/0	0/0	0/0	0/0	0/0	0/0	0/0	0/0
Chromosome2-1495282 INV	PRECISE	10869002	0	0	0/0	0/0	0/0	0/0	0/0	0/0	0/0	0/0	0/0	0/0	0/0	0/1	0/0	0/0
Chromosome2-1495528 DEL	PRECISE	71	0	0	0/0	0/0	0/1	0/0	0/0	0/0	0/0	0/0	0/0	0/0	0/0	0/0	0/0	0/0
Chromosome2-1495668 INS	PRECISE	104	0	0	0/0	0/0	0/0	0/0	0/0	0/0	0/0	0/0	0/0	0/1	0/0	0/0	0/0	0/0
Chromosome2-1497646 DEL	PRECISE	57	0	0	0/0	0/0	0/1	0/0	0/1	0/1	0/0	0/0	0/0	0/0	0/0	0/0	0/0	1/1
Chromosome2-1497759 INS	PRECISE	68	0	0	0/0	0/0	0/0	0/0	0/0	0/0	0/0	0/0	0/0	0/0	0/0	0/0	0/0	1/1
Chromosome2-1497989 INS	PRECISE	7763	0	0	0/0	0/0	0/1	0/0	0/0	0/0	0/0	0/0	0/0	0/0	0/0	0/0	0/0	0/0
Chromosome2-1498583 INS	PRECISE	8715	0	0	0/0	0/0	1/1	0/0	0/0	0/0	0/0	0/0	0/0	0/0	0/0	0/0	0/0	0/0
Chromosome2-1499675 DEL	PRECISE	469	0	0	0/0	0/0	0/1	0/0	0/1	0/1	0/0	0/0	0/0	0/0	0/0	0/0	0/0	1/1
Chromosome2-1499812 INS	PRECISE	65	0	0	0/0	0/0	0/0	0/0	0/0	0/0	0/0	0/0	0/0	0/0	0/0	0/0	0/0	1/1
Chromosome2-1500651 INS	PRECISE	343	0	0.577	0/0	0/0	0/1	0/0	0/1	0/1	0/0	0/0	0/0	0/0	0/0	0/0	0/0	0/0
Chromosome2-1500897 DEL	PRECISE	1919	0	0	0/0	0/0	0/0	0/0	0/0	0/0	0/0	0/0	0/0	0/0	0/0	0/0	0/0	1/1
Chromosome2-1501517 DEL	PRECISE	6825	0	0	0/0	0/0	0/1	0/1	0/1	0/1	1/1	0/1	0/0	0/0	0/0	0/0	0/0	1/1
Chromosome2-1502352 DEL	PRECISE	820	0	0	0/0	0/0	0/1	0/0	0/0	0/0	0/0	0/0	0/0	0/0	0/0	0/0	0/0	0/0

Chromosome2-1507633	INS	PRECISE	1017	0	0.577	0/0	0/0	0/1	0/0	0/1	0/1	0/0	0/0	0/0	0/0	0/0	0/0	0/0	0/0
Chromosome2-1508123	INS	PRECISE	75	0	0	0/0	0/0	0/1	0/0	0/1	0/1	0/0	0/0	0/0	0/0	0/0	0/0	0/0	0/0
Chromosome2-1509598	DEL	PRECISE	483	0	0	0/0	0/0	0/0	0/0	0/0	0/0	0/0	0/0	0/1	0/0	0/0	1/1	0/0	0/0
Chromosome2-1510161	DEL	PRECISE	129	0	0	0/0	0/0	0/0	0/0	0/0	0/0	0/0	0/1	0/0	0/0	0/0	0/0	0/0	0/0
Chromosome2-1510519	DEL	PRECISE	50	0	0	0/0	0/0	0/1	0/0	0/1	0/1	0/0	0/0	0/0	0/0	0/0	0/0	0/0	0/0
Chromosome2-1510531	DEL	PRECISE	1364	0	0	0/0	0/0	0/1	0/0	0/1	0/1	0/0	0/0	0/0	0/0	0/0	0/0	0/0	0/0
Chromosome2-1511325	DEL	PRECISE	334	0	0	0/0	0/0	0/0	0/0	0/0	0/0	0/0	0/0	0/0	0/0	0/0	0/0	0/0	1/1
Chromosome2-1511485	DEL	PRECISE	57	0	0	0/0	0/0	0/1	0/0	0/1	0/1	0/0	0/0	0/0	0/0	0/0	0/0	0/0	0/0
Chromosome2-1512662	DEL	PRECISE	288	0	0	0/0	0/0	0/0	0/0	0/0	0/0	0/0	0/0	0/0	0/0	0/0	0/0	0/0	1/1
Chromosome2-1512896	DEL	PRECISE	1015	0	0	0/0	0/0	0/0	0/0	0/0	0/0	0/0	0/0	0/0	0/0	0/0	0/0	0/0	1/1
Chromosome2-1512896	INV	PRECISE	1024	0	0	0/0	0/0	0/1	0/0	0/1	1/1	0/0	0/0	0/0	0/0	0/0	0/0	0/0	0/0
Chromosome2-1513243	BND	PRECISE	0	0	0	0/0	0/0	0/0	0/0	0/0	0/0	0/0	0/0	0/0	0/0	0/0	0/0	0/0	1/1
Chromosome2-1513531	DEL	PRECISE	57	0	0	0/0	0/0	0/0	0/0	0/0	0/0	0/0	0/0	0/0	0/0	0/0	0/0	0/0	1/1
Chromosome2-1513534	INS	PRECISE	96	0	0	0/0	0/0	0/0	0/1	0/0	0/0	1/1	0/1	0/0	0/0	0/0	0/0	0/0	0/0
Chromosome2-1513726	INS	PRECISE	1600	0	0.707	0/0	0/0	0/0	0/0	0/1	0/1	0/0	0/0	0/0	0/0	0/0	0/0	0/0	0/0
Chromosome2-1513788	DEL	PRECISE	4548	0	0	0/0	0/0	0/0	0/0	0/0	0/0	0/0	0/0	0/0	0/0	0/0	0/0	0/0	1/1
Chromosome2-1514404	INS	PRECISE	91	0	0	0/0	0/0	0/1	0/0	0/1	0/1	0/0	0/0	0/0	0/0	0/0	0/0	0/0	0/0
Chromosome2-1515053	INS	PRECISE	886	0	0	0/0	0/0	0/0	0/0	0/0	0/0	0/0	0/0	0/0	0/0	0/0	0/0	0/0	1/1
Chromosome2-1515230	INS	PRECISE	1014	0	0	0/0	0/0	0/0	0/0	0/0	0/0	0/0	0/0	0/0	0/0	0/0	0/0	0/0	1/1
Chromosome2-1516316	DEL	PRECISE	70	0	0	0/0	0/0	0/0	0/0	0/0	0/0	0/1	0/0	0/0	0/0	0/0	0/0	0/0	0/0
Chromosome2-1517202	BND	PRECISE	0	0	0	0/0	0/1	0/0	0/0	0/0	0/1	0/0	0/0	0/0	0/0	0/0	0/0	0/0	0/0
Chromosome2-1517202	BND	PRECISE	0	0	0	0/0	0/0	0/0	0/0	0/0	0/0	0/1	0/0	0/0	0/0	0/0	0/0	0/0	0/0
Chromosome2-1518182	DEL	PRECISE	59	0	0	0/0	0/0	0/0	0/0	0/0	0/0	0/0	0/0	0/0	0/0	0/0	0/0	0/0	0/1
Chromosome2-1518270	BND	PRECISE	0	0	0	0/0	0/0	0/0	0/0	0/0	0/0	0/0	0/0	0/0	0/0	0/0	0/0	0/0	0/1
Chromosome2-1519911	DEL	PRECISE	4198	0	0	0/0	0/0	0/1	0/0	0/1	0/1	0/0	0/0	0/0	0/0	0/0	0/0	0/0	0/0
Chromosome2-1521050	DEL	PRECISE	4781	0	0	0/0	0/0	0/0	0/0	0/0	0/0	0/0	0/0	0/0	0/0	0/0	0/0	0/0	1/1
Chromosome2-1521409	DEL	PRECISE	571	0	0	0/0	0/0	0/0	0/0	0/0	0/0	0/1	0/0	0/0	0/0	0/0	0/0	0/0	0/0
Chromosome2-1521917	DEL	PRECISE	3608	0.577	0.577	0/0	0/0	0/1	0/0	0/1	0/1	0/0	0/0	0/0	0/0	0/0	0/0	0/0	0/0
Chromosome2-1524308	INS	PRECISE	113	0	0	0/0	0/0	0/1	0/0	0/1	0/0	0/0	0/0	0/0	0/0	0/0	0/0	0/0	1/1
Chromosome2-1524317	DEL	PRECISE	199	0	0	0/0	0/0	0/0	0/1	0/0	0/0	0/1	0/1	0/0	0/0	0/0	0/0	0/0	0/0
Chromosome2-1524683	DEL	PRECISE	74	0	0	0/0	0/0	0/0	0/0	0/1	0/0	0/0	0/0	0/0	0/0	0/0	0/0	0/0	0/0
Chromosome2-1525449	DEL	PRECISE	494	0	0	0/0	0/0	0/0	0/1	0/0	0/0	1/1	0/1	0/0	0/0	0/0	0/0	0/0	0/0
Chromosome2-1526235	INS	PRECISE	2527	0	0.707	0/0	0/0	0/0	0/0	1/1	0/1	0/0	0/0	0/0	0/0	0/0	0/0	0/0	0/0
Chromosome2-1527436	DEL	PRECISE	1785	0	0	0/0	0/0	0/0	0/0	0/0	0/0	0/0	0/0	0/0	0/0	0/0	0/0	0/0	1/1
Chromosome2-1528896	DEL	PRECISE	78	0	0	0/0	0/0	0/0	0/0	0/0	0/0	0/0	0/0	0/0	0/0	0/0	0/0	0/0	1/1
Chromosome2-1529128	DEL	PRECISE	5310	0	0	0/0	0/0	0/1	0/0	0/1	0/1	0/0	0/0	0/0	0/0	0/0	0/0	0/0	0/0
Chromosome2-1530194	INS	PRECISE	75	0	0	0/0	0/0	0/0	0/0	0/1	0/1	0/0	0/0	0/0	0/0	0/0	0/0	0/0	0/0
Chromosome2-1531127	DEL	PRECISE	12634	0	0	0/0	0/0	0/0	0/1	0/0	0/0	1/1	0/1	0/0	0/0	0/0	0/0	0/0	0/0
Chromosome2-1531203	INS	PRECISE	6994	0	0	0/0	0/0	0/1	0/0	0/0	0/0	0/0	0/0	0/0	0/0	0/0	0/0	0/0	0/0
Chromosome2-1531686	INS	PRECISE	4559	0	0	0/0	0/0	0/0	0/1	0/0	0/0	0/0	0/0	0/0	0/0	0/0	0/0	0/0	0/0
Chromosome2-1531740	INS	PRECISE	135	0	0	0/0	0/0	0/1	0/0	0/1	0/1	0/0	0/0	0/0	0/0	0/0	0/0	0/0	1/1
Chromosome2-1531833	DEL	PRECISE	2862	0	0	0/0	0/0	0/0	0/0	0/0	0/0	0/0	0/0	0/0	0/0	0/0	0/0	0/0	1/1
Chromosome2-1532647	INS	PRECISE	1624	0	0	0/0	0/0	0/0	0/0	0/0	0/0	0/0	0/0	0/0	0/0	0/0	0/0	0/0	1/1
Chromosome2-1533652	DEL	PRECISE	55	0	0	0/0	0/0	0/1	0/0	0/0	0/0	0/0	0/0	0/0	0/0	0/0	0/0	0/0	0/0
Chromosome2-1533815	DEL	PRECISE	96	0	0	0/0	0/0	0/1	0/0	0/1	0/1	0/0	0/0	0/0	0/0	0/0	0/0	0/0	0/0
Chromosome2-1534234	DEL	PRECISE	858	0	0	0/0	0/0	0/1	0/0	0/0	0/0	0/0	0/0	0/0	0/0	0/0	0/0	0/0	0/0
Chromosome2-1535040	DEL	PRECISE	75	0	0	0/0	0/0	0/1	0/0	0/1	0/1	0/0	0/0	0/0	0/0	0/0	0/0	0/0	0/0
Chromosome2-1536357	DEL	PRECISE	110	0	0	0/0	0/0	0/0	0/0	0/0	0/0	0/1	0/0	0/0	0/0	0/0	0/0	0/0	0/0
Chromosome2-1537658	INS	PRECISE	345	0	0	0/0	0/0	0/1	0/0	0/1	0/1	0/0	0/0	0/0	0/0	0/0	0/0	0/0	0/0
Chromosome2-1538115	BND	PRECISE	0	0	0	0/0	0/0	0/0	0/0	0/0	0/0	0/0	0/0	0/0	0/0	0/0	1/1	0/0	0/1
Chromosome2-1539116	BND	PRECISE	0	0	0	0/0	0/0	0/0	0/0	0/0	0/0	0/1	0/0	0/0	0/0	0/0	0/0	0/0	0/0
Chromosome2-1539204	DEL	PRECISE	963	0	0	0/0	0/0	0/1	0/1	0/1	0/1	0/1	0/0	0/0	0/0	0/0	0/0	0/0	1/1
Chromosome2-1539367	DEL	PRECISE	918	0	0	0/0	0/0	0/1	0/0	0/1	0/1	0/0	0/0	0/0	0/0	0/0	0/0	0/0	0/0
Chromosome2-1540087	INS	PRECISE	413	0	0	0/0	0/0	0/1	0/0	0/0	0/0	0/0	0/0	0/0	0/0	0/0	0/0	0/0	0/0
Chromosome2-1540251	DEL	PRECISE	80	0	0	0/0	0/0	0/1	0/0	0/0	0/0	0/0	0/0	0/0	0/0	0/0	0/0	0/0	0/0
Chromosome2-1541282	BND	PRECISE	0	0	0	0/0	0/0	0/0	0/1	0/0	0/0	0/1	0/0	0/0	0/0	0/0	0/0	0/0	0/0
Chromosome2-1541799	INS	PRECISE	55	0	0	0/0	0/0	0/0	0/0	0/0	0/0	0/0	0/0	0/0	0/0	0/0	0/0	0/0	1/1
Chromosome2-1542324	DEL	PRECISE	4884	0	0	0/0	0/0	0/0	0/1	0/0	0/0	1/1	0/1	0/0	0/0	0/0	0/0	0/0	0/0
Chromosome2-1542815	DEL	PRECISE	3838	0	0	0/0	0/0	0/0	0/1	0/0	0/0	1/1	0/1	0/0	0/0	0/0	0/0	0/0	0/0
Chromosome2-1544651	DEL	PRECISE	3724	0	0	0/0	0/0	0/1	0/0	0/1	0/1	0/0	0/0	0/0	0/0	0/0	0/0	0/0	0/0
Chromosome2-1545028	DEL	PRECISE	13991	0	0	0/0	0/0	0/1	0/0	0/1	0/1	0/0	0/0	0/0	0/0	0/0	0/0	0/0	0/0
Chromosome2-1545237	INS	PRECISE	6852	0	0.707	0/0	0/0	0/0	0/0	0/0	0/0	0/1	1/1	0/0	0/0	0/0	0/0	0/0	0/0
Chromosome2-1545357	INS	PRECISE	8057	0	0	0/0	0/0	0/0	0/0	0/0	0/0	0/0	0/0	0/0	0/1	0/0	0/0	0/0	0/0
Chromosome2-1545884	INS	PRECISE	349	0	0	0/0	0/0	0/0	0/0	0/0	0/0	0/0	0/0	0/0	0/0	0/0	0/0	0/0	1/1
Chromosome2-1546430	DEL	PRECISE	12344	0	0	0/0	0/0	0/1	0/0	0/1	0/1	0/0	0/0	0/0	0/0	0/0	0/0	0/0	0/0
Chromosome2-1546443	DEL	PRECISE	363	0	0	0/0	0/0	0/0	0/0	0/0	0/0	0/0	0/0	0/0	0/0	0/0	0/0	0/0	1/1
Chromosome2-1546624	INS	PRECISE	3100	0	0.707	0/0	0/0	0/0	0/0	0/0	0/0	0/0	0/0	0/0	0/0	0/0	0/0	0/0	0/0
Chromosome2-1550047	INS	PRECISE	386	0	0.957	0/0	0/0	0/1	0/0	0/1	0/1	0/0	0/0	0/0	0/0	0/0	0/0	0/0	1/1
Chromosome2-1550109	DEL	PRECISE	3332	0	0	0/0	0/0	0/0	0/0	0/0	0/0	0/0	0/0	0/0	0/0	0/0	0/0	0/0	1/1
Chromosome2-1551012	DEL	PRECISE	6802	0	0	0/0	0/0	0/1	0/0	0/1	0/1	0/0	0/0	0/0	0/0	0/0	0/0	0/0	1/1
Chromosome2-1551358	INV	PRECISE	23267	0	0	0/0	0/0	0/0	0/0	0/0	0/0	0/0	0/0	0/0	0/0	0/0	0/0	0/0	0/1
Chromosome2-1554226	DEL	PRECISE	5332	0	0	0/0	0/0	0/0	0/0	0/0	0/0	0/0	0/1	0/0	0/0	0/0	0/0	0/0	1/1
Chromosome2-1554257	BND	PRECISE	0	0	0	0/0	0/0	0/0	0/0	0/1	0/0	0/0	0/0	0/0	0/0	0/0	0/0	0/0	0/0

Chromosome2-1556219	DEL	PRECISE	62	0	0	0/0	0/0	0/0	0/0	0/1	0/1	0/0	0/0	0/0	0/0	0/0	0/0	0/0	0/0
Chromosome2-1556222	INS	PRECISE	62	0	0	0/0	0/0	0/0	0/0	0/0	0/0	0/0	0/0	0/0	0/0	0/0	0/0	0/0	1/1
Chromosome2-1557208	DUP	PRECISE	1740	0	0	0/0	0/0	0/0	0/0	0/0	0/0	0/0	0/0	0/0	0/0	0/0	0/0	0/0	0/1
Chromosome2-1558230	DEL	PRECISE	130	0	0	0/0	0/0	0/1	0/0	0/1	0/1	0/0	0/0	0/0	0/0	0/0	0/0	0/0	1/1
Chromosome2-1558260	INS	PRECISE	161	0	0	0/0	0/0	0/0	0/0	0/0	0/0	0/0	0/0	0/0	0/0	0/0	0/0	0/0	1/1
Chromosome2-1558632	DEL	PRECISE	417	0	0	0/0	0/0	0/0	0/0	0/0	0/0	0/0	0/0	0/0	0/0	0/0	0/0	0/0	1/1
Chromosome2-1560207	DEL	PRECISE	58	0	0	0/0	0/0	0/0	0/0	0/0	0/0	0/0	0/0	0/0	0/0	0/0	0/0	0/0	1/1
Chromosome2-1560365	INS	PRECISE	94	0	0	0/0	0/0	0/1	0/0	0/1	0/1	0/0	0/0	0/0	0/0	0/0	0/0	0/0	1/1
Chromosome2-1560507	DEL	PRECISE	143	0	0	0/0	0/0	0/0	0/0	0/0	0/0	0/0	0/0	0/0	0/0	0/0	0/0	0/0	1/1
Chromosome2-1560579	INS	PRECISE	6957	0	0	0/0	0/0	0/0	0/0	0/0	0/0	0/0	0/0	0/0	0/0	0/0	0/0	0/0	1/1
Chromosome2-1562550	INS	PRECISE	1332	0	0.5	0/0	0/0	0/1	0/0	0/1	0/1	0/0	0/0	0/0	0/0	0/0	0/0	0/0	1/1
Chromosome2-1563943	DEL	PRECISE	10327	0	0	0/0	0/0	0/1	0/0	0/1	0/1	0/0	0/0	0/0	0/0	0/0	0/0	0/0	1/1
Chromosome2-1566439	INS	PRECISE	1015	0	0.707	0/0	0/0	0/1	0/0	0/1	0/0	0/0	0/0	0/0	0/0	0/0	0/0	0/0	0/0
Chromosome2-1567088	INS	PRECISE	85	0	0	0/0	0/0	0/0	0/0	0/0	0/0	0/0	0/0	0/0	0/0	0/0	0/0	0/0	1/1
Chromosome2-1568489	DEL	PRECISE	827	0.966	0.316	0/0	0/1	0/0	0/0	0/1	0/0	0/1	0/0	0/0	0/0	0/1	0/1	0/1	0/1
Chromosome2-1571714	DEL	PRECISE	11399	0	0	0/0	0/0	0/0	0/0	0/0	0/0	0/1	0/0	0/0	0/0	0/0	0/0	0/0	0/0
Chromosome2-1571716	BND	PRECISE	0	0	0	0/0	0/0	0/0	0/0	0/0	0/0	0/1	0/0	0/0	0/0	0/0	0/0	0/0	0/0
Chromosome2-1572816	DEL	PRECISE	171	0	0	0/0	0/0	0/1	0/0	0/1	0/1	0/0	0/0	0/0	0/0	0/0	0/0	0/0	0/0
Chromosome2-1573013	INS	PRECISE	51	0	0	0/0	0/0	0/0	0/1	0/0	0/0	1/1	0/1	0/0	0/0	0/0	0/0	0/0	0/0
Chromosome2-1574573	DEL	PRECISE	225	0	0	0/0	0/0	0/0	0/1	0/0	0/0	1/1	0/1	0/0	0/0	0/0	0/0	0/0	0/0
Chromosome2-1574662	DEL	PRECISE	484	0	0	0/0	0/0	0/0	0/0	0/0	0/0	0/0	0/0	0/0	0/0	0/0	0/0	0/0	1/1
Chromosome2-1574731	DEL	PRECISE	64	0	0	0/0	0/0	0/1	0/0	0/1	0/1	0/0	0/0	0/0	0/0	0/0	0/0	0/0	0/0
Chromosome2-1575791	DEL	PRECISE	20347	0	0	0/0	0/0	0/0	0/0	0/0	0/0	0/0	0/0	0/0	0/0	0/0	0/0	0/0	1/1
Chromosome2-1576302	INS	PRECISE	87	0	0	0/0	0/0	0/0	0/0	0/1	0/1	0/0	0/0	0/0	0/0	0/0	0/0	0/0	0/0
Chromosome2-1577405	DUP	PRECISE	598	0	0	0/0	0/0	0/0	0/0	0/0	0/0	0/1	0/0	0/0	0/0	0/0	0/0	0/0	0/0
Chromosome2-1577686	INV	PRECISE	2698	0	0	0/0	0/0	0/1	0/0	0/0	0/0	0/0	0/0	0/0	0/0	0/0	0/0	0/0	0/0
Chromosome2-1577963	DEL	PRECISE	543	0	0	0/0	0/0	0/0	0/0	0/0	0/0	0/0	0/0	0/0	0/0	0/0	0/0	0/0	1/1
Chromosome2-1578944	INS	PRECISE	50	0	0	0/0	0/0	0/0	0/0	0/0	0/0	0/0	0/0	0/0	0/0	0/0	0/0	0/0	1/1
Chromosome2-1579492	DEL	PRECISE	62	0	0	0/0	0/0	0/0	0/0	0/1	0/1	0/0	0/0	0/0	0/0	0/0	0/0	0/0	0/0
Chromosome2-1579679	INS	PRECISE	7351	0	0	0/0	0/0	0/1	0/0	0/0	0/0	0/0	0/0	0/0	0/0	0/0	0/0	0/0	0/0
Chromosome2-1580165	DEL	PRECISE	62	0	0	0/0	0/0	0/0	0/0	0/0	0/0	0/0	0/0	0/0	0/0	0/0	0/0	0/0	1/1
Chromosome2-1581519	DEL	PRECISE	55	0	0	0/0	0/0	0/0	0/0	0/1	0/1	0/0	0/0	0/0	0/0	0/0	0/0	0/0	0/0
Chromosome2-1582066	DEL	PRECISE	1002	0	0	0/0	0/0	0/1	0/0	0/0	0/0	0/0	0/0	0/0	0/0	0/0	0/0	0/0	0/0
Chromosome2-1582394	INS	PRECISE	1371	0	0	0/0	0/0	0/0	0/1	0/0	0/0	0/0	0/0	0/0	0/0	0/0	0/0	0/0	0/0
Chromosome2-1583688	INS	PRECISE	7220	0	0	0/0	0/0	0/1	0/0	0/0	0/0	0/0	0/0	0/0	0/0	0/0	0/0	0/0	0/0
Chromosome2-1584776	INS	PRECISE	1016	0	0	0/0	0/0	0/0	0/0	0/0	0/0	0/0	0/0	0/0	0/0	0/0	0/0	0/0	1/1
Chromosome2-1584897	DEL	PRECISE	51	0	0	0/0	0/0	0/1	0/0	0/0	0/0	0/0	0/0	0/0	0/0	0/0	0/0	0/0	0/0
Chromosome2-1586752	INS	PRECISE	335	0	0.535	0/0	0/0	0/1	0/1	0/1	0/1	1/1	0/1	0/0	0/0	0/0	0/0	0/0	1/1
Chromosome2-1589066	INS	PRECISE	7192	0	0	0/0	0/0	0/0	0/0	0/0	0/0	0/0	0/0	0/0	0/0	0/0	0/0	0/0	0/0
Chromosome2-1590043	DEL	PRECISE	726	0	0	0/0	0/0	0/1	0/0	0/1	0/1	0/0	0/0	0/0	0/0	0/0	0/0	0/0	0/0
Chromosome2-1591109	INS	PRECISE	356	0	0	0/0	0/0	0/0	0/0	0/0	0/0	0/1	0/0	0/0	0/0	0/0	0/0	0/0	0/0
Chromosome2-1592001	DEL	PRECISE	1547	0	0	0/0	0/0	0/1	0/0	0/1	0/1	0/0	0/0	0/0	0/0	0/0	0/0	0/0	1/1
Chromosome2-1593052	DEL	PRECISE	625	0	0	0/0	0/0	0/1	0/0	0/1	0/1	0/0	0/0	0/0	0/0	0/0	0/0	0/0	1/1
Chromosome2-1595432	INS	PRECISE	66	0	0	0/0	0/0	0/0	0/0	0/0	0/0	0/0	0/0	0/0	0/0	0/0	0/0	0/0	1/1
Chromosome2-1596226	DEL	PRECISE	6296	0	0	0/0	0/0	0/1	0/0	0/1	0/1	0/0	0/0	0/0	0/0	0/0	0/0	0/0	1/1
Chromosome2-1596826	DEL	PRECISE	60	0	0	0/0	0/0	0/0	0/0	0/0	0/0	0/0	0/0	0/0	0/0	1/1	0/0	1/1	0/1
Chromosome2-1598258	INS	PRECISE	5959	0	0	0/0	0/0	0/0	0/0	0/0	0/0	0/0	0/0	0/0	0/0	0/0	0/0	0/0	1/1
Chromosome2-1599105	INS	PRECISE	53	0	0	0/0	0/0	0/0	0/0	0/0	0/0	0/0	0/0	0/0	0/0	0/0	0/0	0/0	1/1
Chromosome2-1599180	DEL	PRECISE	55	0	0	0/0	0/0	0/1	0/0	0/1	0/1	0/0	0/0	0/0	0/0	0/0	0/0	0/0	0/0
Chromosome2-1601561	DEL	PRECISE	99	0	0	0/0	0/0	0/0	0/0	0/1	0/1	0/0	0/0	0/0	0/0	0/0	0/0	0/0	0/0
Chromosome2-1602386	DEL	PRECISE	275	0	0	0/0	0/0	0/0	0/1	0/0	0/0	1/1	0/1	0/0	0/0	0/0	0/0	0/0	0/0
Chromosome2-1602540	INS	PRECISE	113	0	0	0/0	0/0	0/1	0/0	0/1	0/1	0/0	0/0	0/0	0/0	0/0	0/0	0/0	0/0
Chromosome2-1602653	DEL	PRECISE	1717	0	0	0/0	0/0	0/1	0/0	0/1	0/1	0/0	0/0	0/0	0/0	0/0	0/0	0/0	0/0
Chromosome2-1603036	INS	PRECISE	5742	0	0	0/0	0/0	0/0	0/0	0/0	0/0	0/0	0/0	0/0	0/0	0/0	0/0	0/0	1/1
Chromosome2-1604601	DEL	PRECISE	117	0	0	0/0	0/0	0/0	0/0	0/0	0/0	0/0	0/0	0/0	0/1	0/0	1/1	0/0	0/0
Chromosome2-1605705	BND	PRECISE	0	0	0	0/0	0/0	0/0	0/0	0/0	0/0	0/0	0/0	0/1	0/0	0/0	0/0	0/0	0/0
Chromosome2-1606597	DEL	PRECISE	14112	0	0	0/0	0/0	0/0	0/0	0/0	0/0	0/0	0/0	0/0	0/0	0/0	0/0	0/0	1/1
Chromosome2-1608215	DEL	PRECISE	63	0	0	0/0	0/0	0/1	0/0	0/1	0/0	0/0	0/0	0/0	0/0	0/0	0/0	0/0	0/0
Chromosome2-1608333	INS	PRECISE	293	0	0.577	0/0	0/0	0/1	0/0	0/1	0/1	0/0	0/0	0/0	0/0	0/0	0/0	0/0	0/0
Chromosome2-1611393	DEL	PRECISE	201	0	0	0/0	0/0	0/0	0/1	0/0	0/0	1/1	0/1	0/0	0/0	0/0	0/0	0/0	0/0
Chromosome2-1612831	BND	PRECISE	0	0	0	0/0	0/0	0/1	0/0	0/0	0/0	0/0	0/0	0/0	0/0	0/0	0/0	0/0	0/0
Chromosome2-1613193	INS	PRECISE	4293	0	0	0/0	0/0	0/0	0/0	0/0	0/0	0/0	0/0	0/0	0/0	0/0	0/0	0/0	1/1
Chromosome2-1613269	DEL	PRECISE	3674	0	0	0/0	0/0	0/1	0/0	0/1	0/1	0/0	0/0	0/0	0/0	0/0	0/0	0/0	1/1
Chromosome2-1613675	INS	PRECISE	160	0	0	0/0	0/0	0/0	0/0	0/0	0/0	0/0	0/0	0/0	0/0	0/0	0/0	0/0	1/1
Chromosome2-1614183	INS	PRECISE	54	0	0	0/0	0/0	0/0	0/0	0/0	0/0	0/0	0/0	0/0	0/0	0/0	0/0	0/0	1/1
Chromosome2-1615135	DEL	PRECISE	7653	0	0	0/0	0/0	0/1	0/0	0/0	0/1	0/0	0/0	0/0	0/0	0/0	0/0	0/0	0/0
Chromosome2-1616865	INS	PRECISE	339	0	0	0/0	0/0	0/1	0/0	0/1	0/1	0/0	0/0	0/0	0/0	0/0	0/0	0/0	0/0
Chromosome2-1620354	INS	PRECISE	968	0	0	0/0	0/0	1/1	0/0	0/0	0/0	0/0	0/0	0/0	0/0	0/0	0/0	0/0	0/0
Chromosome2-1620506	DEL	PRECISE	1055	0	0	0/0	0/0	0/1	0/0	0/1	0/1	0/0	0/0	0/0	0/0	0/0	0/0	0/0	1/1
Chromosome2-1620943	INS	PRECISE	1105	0	0	0/0	0/0	0/0	0/0	0/0	0/0	0/0	0/0	0/0	0/0	0/0	0/0	0/0	1/1
Chromosome2-1621188	INS	PRECISE	1440	0	0	0/0	0/0	0/0	0/0	0/0	0/0	0/0	0/0	0/0	0/0	0/0	0/0	0/0	1/1
Chromosome2-1623627	DEL	PRECISE	698	0	0	0/0	0/0	0/1	0/0	0/1	0/1	0/0	0/0	0/0	0/0	0/0	0/0	0/0	0/0
Chromosome2-1623925	INS	PRECISE	181	0	0	0/0	0/0	0/1	0/1	0/1	0/1	1/1	0/1	0/0	0/0	0/0	0/0	0/0	1/1

Chromosome2-1624186	DEL	PRECISE	1640	0	0	0/0	0/0	0/1	0/0	0/1	0/1	0/0	0/0	0/0	0/0	0/0	0/0	0/0	0/0
Chromosome2-1624428	INS	PRECISE	261	0	0	0/0	0/0	0/1	0/0	0/0	0/0	0/0	0/0	0/0	0/0	0/0	0/0	0/0	0/0
Chromosome2-1626662	INS	PRECISE	132	0	0	0/0	0/0	0/0	0/0	0/0	0/0	0/0	0/0	0/0	0/0	0/0	0/0	0/0	1/1
Chromosome2-1627104	INS	PRECISE	85	0	0	0/0	0/0	0/1	0/0	0/1	0/1	0/0	0/0	0/0	0/0	0/0	0/0	0/0	0/0
Chromosome2-1627263	DEL	PRECISE	687	0	0	0/0	0/0	0/1	0/0	0/1	0/0	0/0	0/0	0/0	0/0	0/0	0/0	0/0	1/1
Chromosome2-1627875	INS	PRECISE	2181	0	0	0/0	0/0	0/1	0/0	0/0	0/0	0/0	0/0	0/0	0/0	0/0	0/0	0/0	0/0
Chromosome2-1627876	BND	PRECISE	0	0	0	0/0	0/0	0/0	0/0	0/1	0/0	0/0	0/0	0/0	0/0	0/0	0/0	0/0	0/0
Chromosome2-1627989	INS	PRECISE	4829	0	0	0/0	0/0	1/1	0/0	0/0	0/0	0/0	0/0	0/0	0/0	0/0	0/0	0/0	0/0
Chromosome2-1628521	DEL	PRECISE	83	0	0	0/0	0/0	0/1	0/0	0/1	0/1	0/0	0/0	0/0	0/0	0/0	0/0	0/0	0/0
Chromosome2-1631517	INS	PRECISE	137	0	0	0/0	0/0	0/0	0/0	0/0	0/0	0/0	0/0	0/0	0/0	0/0	0/0	0/0	1/1
Chromosome2-1631531	DEL	PRECISE	211	0	0	0/0	0/0	0/0	0/0	0/0	0/0	0/1	0/0	0/0	0/0	0/0	0/0	0/0	1/1
Chromosome2-1632598	DEL	PRECISE	996	0	0	0/0	0/0	0/0	0/0	0/0	0/0	0/0	0/0	0/0	0/0	0/0	0/0	0/0	1/1
Chromosome2-1632869	INS	PRECISE	517	0	0	0/0	0/0	0/1	0/0	0/0	0/0	0/0	0/0	0/0	0/0	0/0	0/0	0/0	1/1
Chromosome2-1634167	DEL	PRECISE	3615	0	0	0/0	0/1	0/0	0/0	0/0	0/0	0/0	0/0	0/0	0/0	0/0	0/0	0/0	0/0
Chromosome2-1634977	DEL	PRECISE	252	0.378	0	0/0	0/0	0/1	1/1	0/0	0/1	1/1	0/1	0/0	0/0	0/0	0/0	0/0	1/1
Chromosome2-1635697	INS	PRECISE	896	0	0.707	0/0	0/0	0/1	0/0	0/0	0/0	0/0	0/0	0/0	0/0	0/0	0/0	0/0	0/0
Chromosome2-1635751	DEL	PRECISE	51	0	0	0/0	0/0	0/1	0/0	0/0	0/0	0/0	0/0	0/0	0/0	0/0	0/0	0/0	0/0
Chromosome2-1636292	INS	PRECISE	58	0	0	0/0	0/0	0/1	0/0	0/0	0/0	0/0	0/0	0/0	0/0	0/0	0/0	0/0	0/0
Chromosome2-1637667	DEL	PRECISE	12535	0	0	0/0	0/0	0/1	0/0	0/1	0/0	0/0	0/0	0/0	0/0	0/0	0/0	0/0	1/1
Chromosome2-1638982	DEL	PRECISE	56	0	0	0/0	0/0	0/1	0/0	0/0	0/0	0/0	0/0	0/0	0/0	0/0	0/0	0/0	0/0
Chromosome2-1639653	INS	PRECISE	9914	0	0	0/0	0/0	0/0	0/0	0/0	0/0	0/0	0/0	0/0	0/0	0/0	0/0	0/0	1/1
Chromosome2-1639653	BND	PRECISE	0	0	0	0/0	0/0	0/0	0/0	0/0	0/0	0/0	0/0	0/0	0/0	0/0	0/0	0/0	0/1
Chromosome2-1640875	DEL	PRECISE	252	0	0	0/0	0/0	0/0	0/0	0/1	0/0	0/0	0/0	0/0	0/0	0/0	0/0	0/0	0/0
Chromosome2-1641172	INS	PRECISE	53	0	0	0/0	0/0	0/0	0/0	0/0	0/0	0/0	0/0	0/0	0/0	0/0	0/0	0/0	1/1
Chromosome2-1641496	INS	PRECISE	1354	0	0	0/0	0/0	0/1	0/0	0/0	0/0	0/0	0/0	0/0	0/0	0/0	0/0	0/0	0/0
Chromosome2-1642922	INS	PRECISE	741	0	0	0/0	0/0	0/0	0/0	0/1	0/0	0/0	0/0	0/0	0/0	0/0	0/0	0/0	0/0
Chromosome2-1643544	INS	PRECISE	970	0	0	0/0	0/0	0/1	0/0	0/0	0/0	0/0	0/0	0/0	0/0	0/0	0/0	0/0	0/0
Chromosome2-1643662	INS	PRECISE	566	0	0	0/0	0/0	0/0	0/0	0/0	0/0	0/1	0/0	0/0	0/0	0/0	0/0	0/0	0/0
Chromosome2-1643759	DEL	PRECISE	475	0	0	0/0	0/0	0/1	0/1	0/1	0/1	1/1	0/1	0/0	0/0	0/0	0/0	0/0	1/1
Chromosome2-1644183	INS	PRECISE	8695	0	0	0/0	0/0	0/1	0/0	0/0	0/0	0/0	0/0	0/0	0/0	0/0	0/0	0/0	0/0
Chromosome2-1644724	DEL	PRECISE	4356	0	0	0/0	0/0	0/0	0/0	0/0	0/0	0/0	0/0	0/0	0/0	0/0	0/0	0/0	1/1
Chromosome2-1645426	DEL	PRECISE	5306	0	0	0/0	0/0	0/0	0/0	0/0	0/0	0/0	0/0	0/0	0/0	0/0	0/0	0/0	1/1
Chromosome2-1645895	DEL	PRECISE	10597	0	0	0/0	0/0	0/0	0/1	0/0	0/1	1/1	0/1	0/0	0/0	0/0	0/0	0/0	0/0
Chromosome2-1646602	INS	PRECISE	54	0	0.707	0/0	0/0	0/0	0/0	0/0	0/0	0/0	0/0	0/0	0/1	0/0	1/1	0/0	0/0
Chromosome2-1647640	INS	PRECISE	149	0	0	0/0	0/0	0/0	0/0	0/0	0/0	0/0	0/0	0/0	0/1	0/0	1/1	0/0	0/0
Chromosome2-1647656	DEL	PRECISE	133	0	0	0/0	0/0	0/1	0/0	0/1	0/0	0/0	0/0	0/0	0/0	0/0	0/0	0/0	0/0
Chromosome2-1648468	BND	PRECISE	0	0	0	0/0	0/0	0/0	0/0	0/0	0/0	0/0	0/0	0/0	0/0	0/0	0/1	0/1	0/1
Chromosome2-1648468	BND	PRECISE	0	0	0	0/0	0/0	0/0	0/0	0/0	0/0	0/0	0/1	0/0	0/0	0/1	0/0	0/0	0/0
Chromosome2-1648545	DEL	PRECISE	11539	0	0	0/0	0/0	0/0	0/1	0/0	0/1	0/0	0/1	0/0	0/0	0/0	0/0	0/0	0/0
Chromosome2-1649612	INS	PRECISE	1035	0	0	0/0	0/0	0/0	0/0	0/0	0/0	0/0	0/0	0/0	0/0	0/0	0/0	0/1	0/0
Chromosome2-1649719	DEL	PRECISE	26478863	0	0	0/0	0/0	0/0	0/0	0/0	0/0	0/0	0/0	0/0	0/0	0/0	0/0	0/0	1/1
Chromosome2-1649719	BND	PRECISE	0	0	0	0/0	0/0	0/0	0/0	0/0	0/0	0/0	0/1	0/0	1/1	0/0	0/0	0/0	0/0
Chromosome2-1649719	BND	PRECISE	0	0	0	0/0	0/0	0/0	0/0	0/0	0/0	0/0	0/1	0/0	0/0	0/0	0/0	0/0	0/0
Chromosome2-1649719	BND	PRECISE	0	0	0	0/0	0/0	0/0	0/0	0/0	0/0	0/0	0/0	0/0	0/0	0/0	0/0	0/0	0/1
Chromosome2-1649719	BND	PRECISE	0	0	0	0/0	0/0	0/0	0/0	0/0	0/0	0/0	0/0	0/0	0/1	0/0	0/0	0/0	0/1
Chromosome2-1650474	DEL	PRECISE	53	0	0	0/0	0/0	0/1	0/0	0/0	0/0	0/0	0/0	0/0	0/0	0/0	0/0	0/0	0/0
Chromosome2-1650691	BND	PRECISE	0	0	0	0/0	0/0	0/0	0/0	0/1	0/0	0/0	0/0	0/0	0/0	0/0	0/0	0/0	0/0
Chromosome2-1652142	INS	PRECISE	99	0	0	0/0	0/0	0/1	0/0	0/0	0/0	0/0	0/0	0/0	0/0	0/0	0/0	0/0	0/0
Chromosome2-1652431	DEL	PRECISE	707	0	0	0/0	0/0	0/1	0/0	0/0	0/1	1/1	0/1	0/0	0/0	0/0	0/0	0/0	1/1
Chromosome2-1653087	DEL	PRECISE	625	0	0	0/0	0/0	0/1	0/0	0/0	0/0	0/0	0/0	0/0	0/0	0/0	0/0	0/0	1/1
Chromosome2-1653211	DEL	PRECISE	135	0	0	0/0	0/0	0/0	0/1	0/0	0/0	0/1	0/0	0/0	0/0	0/0	0/0	0/0	0/0
Chromosome2-1653480	DEL	PRECISE	67	0	0	0/0	0/0	0/0	0/0	0/0	0/0	0/0	0/0	0/0	0/0	0/0	0/0	0/0	1/1
Chromosome2-1653966	INS	PRECISE	136	0	0	0/0	0/0	0/0	0/0	0/0	0/0	0/0	0/0	0/0	0/0	0/0	0/0	0/0	1/1
Chromosome2-1654033	DEL	PRECISE	807	0	0	0/0	0/0	0/0	0/1	0/0	0/0	0/0	0/0	0/0	0/0	0/0	0/0	0/0	0/0
Chromosome2-1654153	DEL	PRECISE	707	0	0	0/0	0/0	0/1	0/0	0/1	0/0	0/0	0/0	0/0	0/0	0/0	0/0	0/0	1/1
Chromosome2-1654534	INS	PRECISE	180	0	0	0/0	0/0	0/0	0/0	0/1	0/0	0/0	0/0	0/0	0/0	0/0	0/0	0/0	0/0
Chromosome2-1655568	INS	PRECISE	12152	0	0	0/0	0/0	0/1	0/0	0/0	0/0	0/0	0/0	0/0	0/0	0/0	0/0	0/0	0/0
Chromosome2-1655569	BND	PRECISE	0	0	0	0/0	0/0	0/1	0/0	0/0	0/0	0/0	0/0	0/0	0/0	0/0	0/0	0/0	0/0
Chromosome2-1657155	INS	PRECISE	6404	0	0	0/0	0/0	0/1	0/0	0/0	0/0	0/0	0/0	0/0	0/0	0/0	0/0	0/0	0/0
Chromosome2-1658775	INS	PRECISE	358	0	0	0/0	0/0	0/0	0/0	0/1	0/0	0/0	0/0	0/0	0/0	0/0	0/0	0/0	0/0
Chromosome2-1658867	DEL	PRECISE	1022	0	0	0/0	0/0	0/0	0/0	0/0	0/0	0/0	0/0	0/0	0/0	0/0	0/0	0/0	1/1
Chromosome2-1659391	INS	PRECISE	446	0	0	0/0	0/0	0/0	0/0	0/0	0/0	0/1	0/0	0/0	0/0	0/0	0/0	0/0	0/0
Chromosome2-1659642	DEL	PRECISE	5126	0	0	0/0	0/0	0/0	0/0	0/0	0/0	0/0	0/0	0/0	0/0	0/0	0/0	0/0	1/1
Chromosome2-1660539	DEL	PRECISE	26759	0	0	0/0	0/0	1/1	0/0	0/0	0/0	0/0	0/0	0/0	0/0	0/0	0/0	0/0	0/0
Chromosome2-1662334	INS	PRECISE	270	0	0	0/0	0/0	0/0	0/0	0/0	0/0	0/0	0/0	0/0	0/0	0/0	0/0	0/0	1/1
Chromosome2-1662616	INS	PRECISE	5962	0	0	0/0	0/0	0/0	0/0	0/0	0/0	0/0	0/0	0/0	0/0	0/0	0/0	0/0	1/1
Chromosome2-1662618	BND	PRECISE	0	0	0	0/0	0/0	0/0	0/0	0/0	0/0	0/0	0/0	0/0	0/0	0/0	0/0	0/0	1/1
Chromosome2-1663293	DEL	PRECISE	61	0	0	0/0	0/0	0/1	0/0	0/0	0/0	0/0	0/0	0/0	0/0	0/0	0/0	0/0	0/0
Chromosome2-1664190	INS	PRECISE	67	0	0	0/0	0/0	0/1	0/0	0/0	0/0	0/0	0/0	0/0	0/0	0/0	0/0	0/0	1/1
Chromosome2-1666407	DEL	PRECISE	336	0	0	0/0	0/0	0/0	0/1	0/0	0/1	1/1	0/1	0/0	0/0	0/0	0/0	0/0	0/0
Chromosome2-1667638	DEL	PRECISE	561	0	0	0/0	0/0	0/0	0/0	0/0	0/0	0/0	0/0	0/0	0/0	1/1	0/0	0/0	0/0
Chromosome2-1667867	INS	PRECISE	7603	0	0	0/0	0/0	0/0	0/0	1/1	0/0	0/0	0/0	0/0	0/0	0/0	0/0	0/0	0/0
Chromosome2-1667874	INS	PRECISE	68	0	0	0/0	0/0	0/0	0/0	0/0	0/0	0/0	0/0	0/0	0/0	1/1	0/0	0/0	0/0

Chromosome2-1667926	DEL	PRECISE	66	0	0	0/0	0/0	0/0	0/0	0/1	0/0	0/0	0/0	0/0	0/0	0/0	0/0	0/0	0/0
Chromosome2-1668613	DEL	PRECISE	1182	0	0	0/0	0/0	0/0	0/0	0/0	0/0	0/0	0/0	0/0	0/0	1/1	0/0	0/0	0/0
Chromosome2-1668665	INS	PRECISE	12551	0	0	0/0	0/0	0/0	0/0	0/1	0/0	0/0	0/0	0/0	0/0	0/0	0/0	0/0	0/0
Chromosome2-1669420	DEL	PRECISE	75	0	0	0/0	0/0	0/0	0/0	0/0	0/0	0/0	0/0	0/0	0/0	1/1	0/0	0/0	0/0
Chromosome2-1669474	INS	PRECISE	1251	0	0	0/0	0/0	0/0	0/0	0/0	0/0	0/0	0/0	0/0	0/0	0/0	0/0	0/0	1/1
Chromosome2-1671693	INS	PRECISE	2361	0	0	0/0	0/0	0/0	0/0	0/0	0/0	0/0	0/0	0/0	0/0	0/0	0/0	0/0	1/1
Chromosome2-1672183	DEL	PRECISE	2656	0	0	0/0	0/0	0/0	0/0	0/0	0/0	0/0	0/0	0/0	0/0	0/0	0/0	0/0	1/1
Chromosome2-1672617	DEL	PRECISE	1399	0	0	0/0	0/0	0/1	0/0	0/0	0/0	0/0	0/0	0/0	0/0	0/0	0/0	0/0	1/1
Chromosome2-1675216	DEL	PRECISE	1552	0	0	0/0	0/0	0/0	0/0	0/0	0/0	0/0	0/0	0/0	0/0	0/0	0/0	0/0	1/1
Chromosome2-1675656	INS	PRECISE	251	0	0	0/0	0/0	0/1	0/0	0/0	0/0	0/0	0/0	0/0	0/0	0/0	0/0	0/0	0/0
Chromosome2-1676168	DEL	PRECISE	641	0	0	0/0	0/0	0/1	0/0	0/1	0/0	0/0	0/0	0/0	0/0	0/0	0/0	0/0	0/0
Chromosome2-1676854	DEL	PRECISE	51	0	0	0/0	0/0	0/1	0/0	0/1	0/0	0/0	0/0	0/0	0/0	0/0	0/0	0/0	0/0
Chromosome2-1677382	INS	PRECISE	1314	0	0	0/0	0/0	1/1	0/0	0/0	0/0	0/0	0/0	0/0	0/0	0/0	0/0	0/0	0/0
Chromosome2-1678080	DEL	PRECISE	1139	0	0	0/0	0/0	0/0	0/0	0/0	0/0	0/0	0/0	0/0	0/0	0/0	0/0	0/0	1/1
Chromosome2-1678226	INS	PRECISE	55	0	0	0/0	0/0	0/1	0/0	0/1	0/0	0/0	0/0	0/0	0/0	0/0	0/0	0/0	0/0
Chromosome2-1678769	DEL	PRECISE	683	0	0	0/0	0/0	0/1	0/0	0/1	0/0	0/0	0/0	0/0	0/0	1/1	0/0	0/0	1/1
Chromosome2-1678931	INS	PRECISE	7103	0	0	0/0	0/0	0/0	0/0	0/1	0/0	0/0	0/0	0/0	0/0	0/0	0/0	0/0	0/0
Chromosome2-1678932	BND	PRECISE	0	0	0	0/0	0/0	0/0	0/0	0/1	0/0	0/0	0/0	0/0	0/0	0/0	0/0	0/0	0/0
Chromosome2-1679682	DEL	PRECISE	62	0	0	0/0	0/0	0/0	0/0	0/0	0/0	0/0	0/0	0/0	0/0	1/1	0/0	0/0	0/0
Chromosome2-1680014	DEL	PRECISE	237	0	0	0/0	0/0	0/1	0/0	0/0	0/0	0/0	0/0	0/0	0/0	0/0	0/0	0/0	0/0
Chromosome2-1680930	DEL	PRECISE	132	0	0	0/0	0/0	0/1	0/0	0/0	0/0	0/0	0/0	0/0	0/0	0/0	0/0	0/0	1/1
Chromosome2-1681455	DEL	PRECISE	621	0	0	0/0	0/0	0/0	0/0	0/0	0/0	0/0	0/0	0/0	0/0	0/0	0/0	0/0	1/1
Chromosome2-1683837	DEL	PRECISE	3909	0	0	0/0	0/0	0/1	0/0	0/0	0/0	0/0	0/0	0/0	0/0	0/0	0/0	0/0	0/0
Chromosome2-1684453	INS	PRECISE	708	0	0	0/0	0/0	0/0	0/0	0/1	0/0	0/0	0/0	0/0	0/0	0/0	0/0	0/0	0/0
Chromosome2-1684614	DEL	PRECISE	208	0	0	0/0	0/0	0/0	0/0	0/1	0/0	0/0	0/0	0/0	0/0	0/0	0/0	0/0	0/0
Chromosome2-1689721	INS	PRECISE	1538	0	0	0/0	0/0	0/1	0/0	0/0	0/0	0/0	0/0	0/0	0/0	0/0	0/0	0/0	0/0
Chromosome2-1689863	DEL	PRECISE	1371	0	0	0/0	0/0	0/1	0/0	0/0	0/0	0/0	0/0	0/0	0/0	0/0	0/0	0/0	1/1
Chromosome2-1691348	DEL	PRECISE	99	0	0	0/0	0/0	0/1	0/0	0/0	0/0	0/0	0/0	0/0	0/0	0/0	0/0	0/0	1/1
Chromosome2-1692910	DEL	PRECISE	6964	0	0	0/0	0/0	0/0	0/0	0/0	0/0	0/0	0/0	0/0	0/0	0/0	0/0	0/0	1/1
Chromosome2-1693306	DEL	PRECISE	1604	0	0	0/0	0/0	0/1	0/0	0/0	0/0	0/0	0/0	0/0	0/0	0/0	0/0	0/0	0/0
Chromosome2-1696338	DEL	PRECISE	401	0	0	0/0	0/0	0/0	0/0	0/0	0/0	0/0	0/0	0/0	0/0	0/0	0/0	0/0	1/1
Chromosome2-1702775	DEL	PRECISE	63	0	0	0/0	0/0	0/1	0/0	0/0	0/0	0/0	0/0	0/0	0/0	0/0	0/0	0/0	0/0
Chromosome2-1706318	BND	PRECISE	0	0	0	0/0	0/0	0/0	0/0	0/0	0/0	0/1	0/0	0/0	0/0	0/0	0/0	0/0	0/0
Chromosome2-1706687	DEL	PRECISE	19829	0	0	0/0	0/0	0/0	0/0	0/0	0/0	0/0	0/0	0/0	0/0	0/0	0/0	0/0	1/1
Chromosome2-1706806	DEL	PRECISE	12564	0	0	0/0	0/0	0/1	0/0	0/0	0/0	0/0	0/0	0/0	0/0	0/0	0/0	0/0	0/0
Chromosome2-1707786	INS	PRECISE	55	0.447	0.447	0/0	0/0	0/0	0/1	0/1	0/0	0/0	0/0	0/0	0/1	0/0	0/0	0/0	0/0
Chromosome2-1707869	INS	PRECISE	10876	0	0	0/0	0/0	0/0	0/0	0/0	0/0	0/0	0/0	0/0	0/0	0/0	0/0	0/0	0/0
Chromosome2-1707973	INS	PRECISE	52	0	0.894	0/0	0/0	0/0	0/0	0/1	0/0	0/0	0/0	0/0	0/1	0/0	0/0	0/0	0/0
Chromosome2-1708530	INS	PRECISE	6737	0	0.707	0/0	0/0	0/0	0/0	0/0	0/0	0/0	0/0	0/1	0/0	0/0	0/0	0/0	0/0
Chromosome2-1708609	DEL	PRECISE	671	0	0	0/0	0/0	0/0	0/0	0/0	0/0	0/0	0/0	0/0	0/1	0/0	0/1	0/0	0/0
Chromosome2-1710461	DEL	PRECISE	4613	0	0	0/0	0/0	0/0	0/0	0/0	0/0	0/0	0/0	0/1	0/0	1/1	1/1	1/1	0/0
Chromosome2-1711969	INS	PRECISE	100	0	0	0/0	0/0	0/1	0/0	0/0	0/0	0/0	0/0	0/0	0/0	1/1	0/0	0/0	1/1
Chromosome2-1712180	INS	PRECISE	127	0	0	0/0	0/0	0/1	0/0	0/0	0/0	0/0	0/0	0/0	0/0	0/0	0/0	0/0	1/1
Chromosome2-1712684	DEL	PRECISE	227	0	0	0/0	0/0	0/0	0/0	0/0	0/0	0/0	0/0	0/0	0/0	0/0	0/0	0/0	1/1
Chromosome2-1712921	DEL	PRECISE	203	0	0	0/0	0/0	0/0	0/0	0/0	0/0	0/0	0/0	0/0	0/0	0/0	0/0	0/0	1/1
Chromosome2-1719159	DEL	PRECISE	74	0	0	0/0	0/0	0/0	0/0	0/0	0/0	0/0	0/0	0/0	0/0	0/0	0/0	0/0	1/1
Chromosome2-1720410	DEL	PRECISE	65572	0	0	0/0	0/0	0/0	0/0	0/0	0/0	0/0	0/0	0/0	0/0	0/0	0/0	0/0	1/1
Chromosome2-1720539	DEL	PRECISE	78	0	0	0/0	0/0	0/1	0/0	0/0	0/0	0/0	0/0	0/0	0/0	0/0	0/0	0/0	0/0
Chromosome2-1722075	DEL	PRECISE	1392	0	0	0/0	0/0	0/1	0/0	0/0	0/0	0/0	0/0	0/0	0/0	0/0	0/0	0/0	0/0
Chromosome2-1724289	DEL	PRECISE	301	0	0	0/0	0/0	0/1	0/0	0/0	0/0	0/0	0/0	0/0	0/0	0/0	0/0	0/0	0/0
Chromosome2-1724665	INS	PRECISE	684	0	0.837	0/0	0/0	0/1	0/1	0/0	0/1	1/1	0/1	0/0	0/0	0/0	0/0	0/0	0/0
Chromosome2-1725347	INS	PRECISE	5471	0	0	0/0	0/0	1/1	0/0	0/0	0/0	0/0	0/0	0/0	0/0	0/0	0/0	0/0	0/0
Chromosome2-1726510	DEL	PRECISE	417	0	0	0/0	0/0	0/0	0/0	0/0	0/0	0/0	0/0	0/0	0/0	0/0	0/0	0/0	1/1
Chromosome2-1726777	DEL	PRECISE	4198	0	0	0/0	0/0	0/0	0/1	0/0	0/0	0/0	0/0	0/0	0/0	0/0	0/0	0/0	0/0
Chromosome2-1728067	DEL	PRECISE	847	0	0	0/0	0/0	0/0	0/0	0/0	0/0	0/1	0/0	0/0	0/0	0/0	0/0	0/0	0/0
Chromosome2-1728252	DEL	PRECISE	121	0	0	0/0	0/0	0/0	0/0	0/0	0/0	0/0	0/0	0/0	0/0	0/0	0/0	0/0	0/1
Chromosome2-1731209	DEL	PRECISE	2177	0	0	0/0	0/0	0/0	0/0	0/0	0/0	0/1	0/0	0/0	0/0	0/0	0/0	0/0	0/0
Chromosome2-1732612	INS	PRECISE	437	0	0	0/0	0/0	0/0	0/1	0/0	0/0	0/0	0/0	0/0	0/0	0/0	0/0	0/0	0/0
Chromosome2-1733271	DEL	PRECISE	1087	0	0	0/0	0/0	0/0	0/1	0/0	0/0	0/0	0/0	0/0	0/0	0/0	0/0	0/0	0/0
Chromosome2-1736983	DEL	PRECISE	3630	0	0	0/0	0/0	0/0	0/1	0/0	0/0	0/0	0/0	0/0	0/0	0/0	0/0	0/0	0/0
Chromosome2-1740995	DEL	PRECISE	761	0	0	0/0	0/0	0/1	0/0	0/0	0/0	0/0	0/0	0/0	0/0	0/0	0/0	0/0	0/1
Chromosome2-1741087	DEL	PRECISE	3825	0	0	0/0	0/0	0/1	0/0	0/0	0/0	0/0	0/0	0/0	0/0	0/0	0/0	0/0	0/1
Chromosome2-1741501	DEL	PRECISE	3964	0	0	0/0	0/0	0/0	0/0	0/0	0/0	0/0	0/0	0/0	0/0	0/0	1/1	0/0	0/0
Chromosome2-1741522	DEL	PRECISE	283	0	0	0/0	0/0	0/0	0/0	0/0	0/0	0/0	0/0	0/0	0/1	0/0	1/1	0/0	0/0
Chromosome2-1742523	DEL	PRECISE	5440	0	0	0/0	0/0	0/0	0/0	0/0	0/0	0/0	0/0	0/0	0/0	0/0	0/0	0/0	0/1
Chromosome2-1742741	INV	PRECISE	23942850	0	0	0/0	0/0	0/0	0/0	0/0	0/0	0/1	0/0	0/0	0/0	0/0	0/0	0/0	0/0
Chromosome2-1743071	DEL	PRECISE	2668	0	0	0/0	0/0	0/0	0/0	0/0	0/0	0/0	0/0	0/0	0/0	0/0	0/0	0/0	0/1
Chromosome2-1743785	DEL	PRECISE	72	0	0	0/0	0/0	0/0	0/0	0/0	0/0	0/0	0/0	0/0	0/0	0/0	0/0	0/0	0/1
Chromosome2-1743801	DEL	PRECISE	3228	0	0	0/0	0/0	0/0	0/0	0/0	0/0	0/0	0/0	0/0	0/0	0/0	0/0	0/0	0/1
Chromosome2-1749972	DEL	PRECISE	122	0	0	0/0	0/0	0/0	0/0	0/0	0/0	0/0	0/0	0/0	0/0	0/0	0/0	0/0	1/1
Chromosome2-1750379	INS	PRECISE	256	0	0	0/0	0/0	0/0	0/0	0/0	0/0	0/0	0/0	0/0	0/0	0/0	0/0	0/0	1/1
Chromosome2-1750513	DEL	PRECISE	521	0	0	0/0	0/0	0/0	0/0	0/0	0/0	0/0	0/0	0/0	0/0	0/0	0/0	0/0	1/1
Chromosome2-1772208	DEL	PRECISE	806	0	0	0/0	0/0	0/0	0/0	0/0	0/0	0/0	0/0	0/0	0/0	0/0	0/0	0/0	1/1

Chromosome2-1772293	DEL	PRECISE	2929	0	0	0/0	0/0	0/0	0/0	0/0	0/0	0/0	0/0	0/0	0/0	0/0	0/0	0/0	1/1
Chromosome2-1772588	DEL	PRECISE	1233	0	0	0/0	0/0	0/0	0/0	0/0	0/0	0/0	0/0	0/0	0/0	0/0	0/0	0/0	1/1
Chromosome2-1772719	DEL	PRECISE	1352	0	0	0/0	0/0	0/0	0/0	0/0	0/0	0/0	0/0	0/0	0/0	0/0	0/0	0/0	1/1
Chromosome2-1772864	DEL	PRECISE	758	0	0	0/0	0/0	0/0	0/0	0/0	0/0	0/0	0/0	0/0	0/0	0/0	0/0	0/0	1/1
Chromosome2-1773773	DEL	PRECISE	480	0	0	0/0	0/0	0/1	0/0	0/0	0/0	0/0	0/0	0/0	0/0	0/0	0/0	0/0	1/1
Chromosome2-1776801	DEL	PRECISE	53	0	0	0/0	0/0	0/0	0/0	0/0	0/0	0/0	0/0	0/0	0/0	1/1	0/0	0/0	0/0
Chromosome2-1776844	INS	PRECISE	53	0	0	0/0	0/0	0/1	0/0	0/0	0/0	0/0	0/0	0/0	0/0	0/0	0/0	0/0	0/0
Chromosome2-1777553	DEL	PRECISE	88	0	0	0/0	0/0	0/1	0/0	0/0	0/0	0/0	0/0	0/0	0/0	0/0	0/0	0/0	1/1
Chromosome2-1780176	BND	PRECISE	0	0	0	0/0	0/0	0/1	0/0	0/0	0/0	0/0	0/0	0/0	0/0	0/0	0/0	0/0	0/0
Chromosome2-1780661	INS	PRECISE	3848	0	0	0/0	0/0	0/1	0/0	0/0	0/0	0/0	0/0	0/0	0/0	0/0	0/0	0/0	0/0
Chromosome2-1780784	DEL	PRECISE	12431	0.488	0.378	0/0	0/0	0/1	0/0	0/0	0/0	0/0	0/0	0/0	0/1	1/1	1/1	1/1	1/1
Chromosome2-1782808	DEL	PRECISE	1233	0	0	0/0	0/0	0/0	0/0	0/0	0/0	0/1	0/0	0/0	0/0	0/0	0/0	0/0	0/0
Chromosome2-1783268	INS	PRECISE	55	0	0	0/0	0/0	0/0	0/0	0/0	0/0	0/0	0/0	0/0	0/1	0/0	0/1	0/0	0/0
Chromosome2-1783275	INS	PRECISE	5945	0	0	0/0	0/0	0/0	0/0	0/0	0/0	0/0	0/0	0/0	0/0	1/1	0/0	0/0	0/0
Chromosome2-1783303	DEL	PRECISE	50	0	0	0/0	0/0	0/0	0/0	0/1	0/0	0/1	0/0	0/0	0/0	0/0	0/0	0/0	0/0
Chromosome2-1784026	INS	PRECISE	11195	0	0	0/0	0/0	0/0	0/0	0/0	0/0	0/0	0/0	0/0	0/0	0/0	0/1	0/0	0/0
Chromosome2-1785164	DEL	PRECISE	12365	0	0	0/0	0/0	0/0	0/0	0/0	0/0	0/0	0/0	0/1	1/1	1/1	1/1	0/0	0/0
Chromosome2-1786778	INS	PRECISE	230	0	0.5	0/0	0/0	0/1	0/0	0/1	0/0	0/1	0/0	0/1	0/1	1/1	1/1	1/1	1/1
Chromosome2-1786953	DEL	PRECISE	1199	0	0	0/0	0/0	0/0	0/0	0/1	0/0	0/1	0/0	0/0	0/0	0/0	0/0	0/0	0/0
Chromosome2-1787127	DEL	PRECISE	54	0	0	0/0	0/0	0/0	0/0	0/0	0/0	0/0	0/0	0/0	0/1	0/0	0/1	0/0	0/0
Chromosome2-1787593	BND	PRECISE	0	0	0	0/0	0/0	0/0	0/0	0/0	0/0	0/0	0/0	0/0	0/1	0/0	0/0	0/0	0/0
Chromosome2-1787693	INS	PRECISE	3938	0	0	0/0	0/0	0/0	0/0	0/0	0/0	1/1	0/0	0/0	0/0	0/0	0/0	0/0	0/0
Chromosome2-1789262	INS	PRECISE	5521	0	0	0/0	0/0	0/0	0/0	0/0	0/0	0/0	0/0	0/0	0/0	0/0	0/0	0/0	1/1
Chromosome2-1790174	INS	PRECISE	54	0	0	0/0	0/0	0/0	0/0	0/1	0/0	0/0	0/0	0/0	0/0	0/0	0/0	0/0	0/0
Chromosome2-1790576	INS	PRECISE	931	0	0	0/0	0/0	0/0	0/0	0/0	0/0	0/0	0/0	0/0	0/0	0/0	0/0	0/0	1/1
Chromosome2-1790696	DEL	PRECISE	50	0	0	0/0	0/0	0/1	0/0	0/0	0/0	0/0	0/0	0/0	0/0	0/0	0/0	0/0	0/0
Chromosome2-1790872	DEL	PRECISE	1460	0	0	0/0	0/0	0/1	0/0	0/0	0/0	0/0	0/0	0/0	0/0	0/0	0/0	0/0	0/0
Chromosome2-1790982	DEL	PRECISE	70	0	0	0/0	0/0	0/0	0/0	0/0	0/0	0/1	0/0	0/0	0/0	0/0	0/0	0/0	0/0
Chromosome2-1791087	INS	PRECISE	570	0	0	0/0	0/0	0/0	0/0	0/0	0/0	0/1	0/0	0/0	0/0	0/0	0/0	0/0	0/0
Chromosome2-1791328	DEL	PRECISE	99	0	0	0/0	0/0	0/0	0/0	0/1	0/0	0/0	0/0	0/0	0/0	0/0	0/0	0/0	0/0
Chromosome2-1791964	DEL	PRECISE	480	0	0	0/0	0/0	0/1	0/0	0/0	0/0	0/0	0/0	0/0	0/0	0/0	0/0	0/0	0/0
Chromosome2-1792865	INS	PRECISE	146	0	0	0/0	0/0	0/0	0/0	0/1	0/0	0/0	0/0	0/0	0/0	0/0	0/0	0/0	0/0
Chromosome2-1793041	INS	PRECISE	962	0	0	0/0	0/0	0/0	0/0	0/1	0/0	0/0	0/0	0/0	0/0	0/0	0/0	0/0	0/0
Chromosome2-1793531	DEL	PRECISE	9923	0	0	0/0	0/0	0/0	0/0	0/1	0/1	0/0	0/0	1/1	0/0	0/0	0/0	0/0	0/0
Chromosome2-1795514	INS	PRECISE	236	0	0	0/0	0/0	0/0	0/0	0/0	0/0	0/0	0/0	0/0	0/0	0/0	0/0	0/0	1/1
Chromosome2-1795558	INS	PRECISE	10874	0	0	0/0	0/0	0/0	0/0	0/0	0/0	0/1	0/0	0/0	0/0	0/0	0/0	0/0	0/0
Chromosome2-1797738	INS	PRECISE	92	0	0	0/0	0/0	0/0	0/0	0/0	0/0	0/1	0/0	0/0	0/0	0/0	0/0	0/0	0/0
Chromosome2-1798754	INS	PRECISE	12573	0	0.707	0/0	0/0	0/0	0/0	0/0	0/0	0/1	0/1	0/0	0/0	0/0	0/0	0/0	0/0
Chromosome2-1798821	DEL	PRECISE	530	0	0	0/0	0/0	0/0	0/0	0/0	0/0	0/0	0/0	0/0	0/0	0/0	0/0	0/0	1/1
Chromosome2-1799376	DEL	PRECISE	378	0	0	0/0	0/0	0/0	0/0	0/0	0/0	0/1	0/0	0/0	0/0	0/0	0/0	0/0	0/0
Chromosome2-1799709	DEL	PRECISE	67	0.707	0	0/0	0/0	0/0	0/1	0/0	0/0	0/1	0/0	0/0	0/0	0/0	0/0	0/0	0/0
Chromosome2-1800507	DEL	PRECISE	108	0	0	0/0	0/0	0/1	0/0	0/0	0/0	0/0	0/0	0/0	0/0	0/0	0/0	0/0	0/0
Chromosome2-1800935	INS	PRECISE	888	0	0	0/0	0/0	0/0	0/0	0/0	0/0	0/0	0/0	0/0	0/0	0/0	0/0	0/0	1/1
Chromosome2-1801635	INS	PRECISE	1240	0	0	0/0	0/0	0/0	0/0	0/1	0/0	0/0	0/0	0/0	0/0	0/0	0/0	0/0	0/0
Chromosome2-1802443	INS	PRECISE	2463	0	0	0/0	0/0	0/0	0/0	0/0	0/0	0/1	0/0	0/0	0/0	0/0	0/0	0/0	0/0
Chromosome2-1805136	INS	PRECISE	277	0	0.983	0/0	0/0	0/1	0/1	0/0	0/1	0/1	0/1	0/0	0/0	0/0	0/0	0/0	1/1
Chromosome2-1805419	INS	PRECISE	8266	0	0	0/0	0/0	0/0	0/0	0/0	0/0	1/1	0/0	0/0	0/0	0/0	0/0	0/0	0/0
Chromosome2-1805921	DEL	PRECISE	251	0	0	0/0	0/0	0/1	0/0	0/0	0/0	0/0	0/0	0/0	0/0	0/0	0/0	0/0	0/0
Chromosome2-1805993	INS	PRECISE	331	0	0.707	0/0	0/0	0/0	0/0	0/1	0/1	0/0	0/0	0/0	0/0	0/0	0/0	0/0	0/0
Chromosome2-1806068	INS	PRECISE	625	0	0	0/0	0/0	0/0	0/0	0/1	0/0	0/0	0/0	0/0	0/0	0/0	0/0	0/0	0/0
Chromosome2-1806325	BND	PRECISE	0	0	0	0/0	0/0	0/1	0/0	0/0	0/0	0/0	0/0	0/0	0/0	0/0	0/0	0/0	0/0
Chromosome2-1806326	BND	PRECISE	0	0	0	0/0	0/0	0/1	0/0	0/0	0/0	0/0	0/0	0/0	0/0	0/0	0/0	0/0	0/0
Chromosome2-1806671	INS	PRECISE	57	0	0	0/0	0/0	0/0	0/0	0/0	0/0	0/0	0/0	0/0	0/0	0/0	0/0	0/0	1/1
Chromosome2-1806934	INS	PRECISE	150	0	0	0/0	0/0	0/0	0/1	0/0	0/0	0/1	0/0	0/0	0/0	0/0	0/0	0/0	0/0
Chromosome2-1808714	DEL	PRECISE	896	0	0	0/0	0/0	0/0	0/1	0/0	0/1	0/1	0/1	0/0	0/0	0/0	0/0	0/0	0/0
Chromosome2-1808773	INS	PRECISE	1375	0	0	0/0	0/0	0/0	0/0	0/1	0/0	0/0	0/0	0/0	0/0	0/0	0/0	0/0	0/0
Chromosome2-1809818	INS	PRECISE	995	0	0	0/0	0/0	0/0	0/0	0/1	0/0	0/0	0/0	0/0	0/0	0/0	0/0	0/0	0/0
Chromosome2-1811270	INS	PRECISE	830	0	0	0/0	0/0	0/1	0/0	0/0	0/0	0/0	0/0	0/0	0/0	0/0	0/0	0/0	0/0
Chromosome2-1811444	INS	PRECISE	560	0	0	0/0	0/0	0/1	0/0	0/0	0/0	0/0	0/0	0/0	0/0	0/0	0/0	0/0	0/0
Chromosome2-1811589	DEL	PRECISE	61	0	0	0/0	0/0	0/0	0/1	0/0	0/1	0/1	0/1	0/0	0/0	0/0	0/0	0/0	0/0
Chromosome2-1812205	DEL	PRECISE	173	0	0	0/0	0/0	0/0	0/0	0/0	0/0	0/0	0/0	0/0	0/0	0/0	0/0	0/0	1/1
Chromosome2-1812373	DEL	PRECISE	51	0	0	0/0	0/0	0/0	0/0	0/0	0/0	0/0	0/0	0/0	0/0	0/0	1/1	0/0	0/0
Chromosome2-1812972	INS	PRECISE	51	0	0	0/0	0/0	0/1	0/0	0/1	0/1	1/1	0/1	0/0	0/0	1/1	1/1	0/0	1/1
Chromosome2-1813433	DEL	PRECISE	519	0	0	0/0	0/0	0/0	0/0	0/0	0/0	0/0	0/0	0/0	0/0	0/0	1/1	0/0	0/0
Chromosome2-1814405	INS	PRECISE	380	0	0.753	0/0	0/0	0/1	0/1	0/1	0/1	1/1	0/1	0/0	0/0	0/0	0/0	0/0	0/0
Chromosome2-1815038	INS	PRECISE	1096	0	0	0/0	0/0	0/0	0/0	0/0	0/0	1/1	0/0	0/0	0/0	0/0	0/0	0/0	0/0
Chromosome2-1815650	DEL	PRECISE	1870	0	0	0/0	0/0	0/1	0/0	0/0	0/0	0/0	0/0	0/0	0/0	0/0	0/0	0/0	0/0
Chromosome2-1815835	INS	PRECISE	473	0	0	0/0	0/0	0/0	0/0	0/1	0/0	0/0	0/0	0/0	0/0	0/0	0/0	0/0	0/0
Chromosome2-1815836	BND	PRECISE	0	0	0	0/0	0/0	0/0	0/0	0/0	0/0	0/0	0/0	0/0	0/0	0/0	0/0	0/0	0/1
Chromosome2-1818581	BND	PRECISE	0	0	0	0/0	0/0	0/0	0/0	0/0	0/0	0/0	0/0	0/0	0/0	0/0	0/0	0/0	0/1
Chromosome2-1819862	INS	PRECISE	3304	0	0	0/0	0/0	0/0	0/0	0/0	0/0	1/1	0/0	0/0	0/0	0/0	0/0	0/0	0/0
Chromosome2-1819956	INS	PRECISE	322	0	0	0/0	0/0	0/1	0/0	0/0	0/0	0/0	0/0	0/0	0/0	0/0	0/0	0/0	0/0
Chromosome2-1820036	INS	PRECISE	882	0	0	0/0	0/0	0/0	0/0	0/0	0/0	0/0	0/0	0/0	0/0	0/0	0/0	0/0	1/1

Chromosome2-1820583	INS	PRECISE	3259	0	0	0/0	0/0	0/0	0/0	0/0	0/0	0/0	0/0	0/0	0/0	0/0	0/0	0/0	1/1
Chromosome2-1821262	DEL	PRECISE	190	0	0	0/0	0/0	0/1	0/0	0/0	0/0	0/0	0/0	0/0	0/0	0/0	0/0	0/0	0/0
Chromosome2-1821391	DEL	PRECISE	996	0	0	0/0	0/0	0/1	0/0	0/1	0/1	1/1	0/0	0/0	0/0	0/0	0/0	0/0	0/0
Chromosome2-1821598	DEL	PRECISE	53	0	0	0/0	0/0	0/1	0/0	0/0	0/0	0/0	0/0	0/0	0/0	0/0	0/0	0/0	0/0
Chromosome2-1821601	INS	PRECISE	94	0	0	0/0	0/0	0/0	0/0	0/1	0/0	0/0	0/0	0/0	0/0	0/0	0/0	0/0	0/0
Chromosome2-1822399	DEL	PRECISE	615	0	0	0/0	0/0	0/0	0/0	0/0	0/0	0/0	0/0	0/0	0/0	0/0	0/0	0/0	1/1
Chromosome2-1823024	DEL	PRECISE	2659	0	0	0/0	0/0	0/0	0/0	0/0	0/0	0/1	0/0	0/0	0/0	0/0	0/0	0/0	0/0
Chromosome2-1823975	DEL	PRECISE	59	0	0	0/0	0/0	0/0	0/1	0/0	0/1	0/1	0/1	0/0	0/0	0/0	0/0	0/0	0/0
Chromosome2-1824399	INS	PRECISE	537	0	0	0/0	0/0	0/0	0/0	0/0	0/0	0/0	0/0	0/0	0/0	0/0	0/0	0/0	1/1
Chromosome2-1824823	DEL	PRECISE	121	0	0	0/0	0/0	0/0	0/0	0/0	0/0	0/0	0/0	0/0	0/0	0/0	0/0	0/0	1/1
Chromosome2-1825857	INS	PRECISE	82	0	0	0/0	0/0	0/1	0/1	0/1	0/1	0/1	0/1	0/0	0/0	0/0	0/0	0/0	1/1
Chromosome2-1826670	INS	PRECISE	8279	0	0	0/0	0/0	0/0	0/0	0/0	0/0	0/1	0/0	0/0	0/0	0/0	0/0	0/0	0/0
Chromosome2-1826975	DEL	PRECISE	3265	0	0	0/0	0/0	0/0	0/0	0/1	0/0	0/0	0/0	0/0	0/0	0/0	0/0	0/0	1/1
Chromosome2-1827975	DEL	PRECISE	96	0	0	0/0	0/0	0/0	0/0	0/0	0/0	0/0	0/0	0/0	0/0	0/0	0/0	0/0	1/1
Chromosome2-1828003	INS	PRECISE	977	0	0	0/0	0/0	0/0	0/0	0/0	0/0	0/0	0/0	0/0	0/0	0/0	0/0	0/0	1/1
Chromosome2-1828441	INS	PRECISE	817	0	0	0/0	0/0	0/1	0/0	0/0	0/0	0/0	0/0	0/0	0/0	0/0	0/0	0/0	0/0
Chromosome2-1828806	DEL	PRECISE	2657	0	0	0/0	0/0	0/0	0/0	0/0	0/0	0/0	0/0	0/0	0/0	0/0	0/0	0/0	1/1
Chromosome2-1829400	INS	PRECISE	704	0	0	0/0	0/0	0/1	0/0	0/0	0/0	0/0	0/0	0/0	0/0	0/0	0/0	0/0	0/0
Chromosome2-1829589	DEL	PRECISE	9991	0	0	0/0	0/0	0/1	0/0	0/0	0/0	0/0	0/0	0/0	0/0	0/0	0/0	0/0	0/0
Chromosome2-1830260	INS	PRECISE	62	0	0	0/0	0/0	0/0	0/0	0/1	0/0	0/0	0/0	0/0	0/0	0/0	0/0	0/0	0/0
Chromosome2-1831719	DEL	PRECISE	5628	0	0	0/0	0/0	0/0	0/0	0/0	0/0	0/1	0/0	0/0	0/0	0/0	0/0	0/0	0/0
Chromosome2-1832073	DEL	PRECISE	183	0	0	0/0	0/0	0/1	0/0	0/0	0/0	0/0	0/0	0/0	0/0	0/0	0/0	0/0	0/0
Chromosome2-1832117	INS	PRECISE	474	0	0	0/0	0/0	0/1	0/0	0/0	0/0	0/0	0/0	0/0	0/0	0/0	0/0	0/0	0/0
Chromosome2-1832729	DEL	PRECISE	3493	0	0	0/0	0/0	0/1	0/0	0/0	0/0	0/0	0/0	0/0	0/0	0/0	0/0	0/0	0/0
Chromosome2-1833202	DEL	PRECISE	61	0	0	0/0	0/0	0/0	0/1	0/0	0/1	0/1	0/1	0/0	0/0	0/0	0/0	0/0	0/0
Chromosome2-1833412	DEL	PRECISE	12423	0	0	0/0	0/0	0/1	0/0	0/0	0/0	0/0	0/0	0/0	0/0	0/0	0/0	0/0	0/1
Chromosome2-1833810	DEL	PRECISE	69	0	0	0/1	0/0	0/0	0/0	0/0	0/0	0/0	0/0	0/0	0/0	0/0	0/0	0/0	0/0
Chromosome2-1834654	BND	PRECISE	0	0	0	0/0	0/0	0/0	0/1	0/0	1/1	0/0	0/1	0/1	0/1	0/0	0/0	0/0	0/1
Chromosome2-1835518	DEL	PRECISE	1389	0	0	0/0	0/0	0/1	0/1	0/1	0/1	1/1	0/1	0/0	0/0	0/0	0/0	0/0	1/1
Chromosome2-1835878	INS	PRECISE	445	0	0.837	0/0	0/0	0/0	0/1	0/0	0/1	0/1	0/1	0/0	0/0	0/0	0/0	0/0	0/0
Chromosome2-1836155	DEL	PRECISE	344	0	0	0/0	0/0	0/1	0/0	0/0	0/0	0/0	0/0	0/0	0/0	0/0	0/0	0/0	0/0
Chromosome2-1836174	INS	PRECISE	624	0	0	0/0	0/0	0/0	0/0	0/1	0/0	0/0	0/0	0/0	0/0	0/0	0/0	0/0	0/0
Chromosome2-1836188	DEL	PRECISE	1281	0	0	0/0	0/0	0/0	0/0	0/0	0/0	0/0	0/0	0/0	0/0	0/0	0/0	0/0	1/1
Chromosome2-1836403	DEL	PRECISE	64	0	0	0/0	0/0	0/0	0/0	0/1	0/0	0/0	0/0	0/0	0/0	0/0	0/0	0/0	0/0
Chromosome2-1837926	DEL	PRECISE	291	0	0	0/0	0/0	0/0	0/0	0/1	0/0	0/0	0/0	0/0	0/0	0/0	0/0	0/0	1/1
Chromosome2-1839047	DEL	PRECISE	271	0	0	0/0	0/0	0/0	0/0	0/0	0/0	0/1	0/0	0/0	0/0	0/0	0/0	0/0	0/0
Chromosome2-1839181	INS	PRECISE	72	0	0	0/0	0/0	0/0	0/0	0/0	0/0	0/1	0/0	0/0	0/0	0/0	0/0	0/0	0/0
Chromosome2-1839784	DEL	PRECISE	13837	0	0	0/0	0/0	0/0	0/0	0/0	0/0	0/0	0/0	0/0	0/0	0/0	0/0	0/0	0/1
Chromosome2-1839818	BND	PRECISE	0	0	0	0/0	0/0	0/0	0/0	0/0	0/0	0/0	0/0	0/0	0/0	0/0	0/0	0/0	0/1
Chromosome2-1841046	BND	PRECISE	0	0	0	0/0	0/1	0/0	0/0	0/0	0/0	0/0	0/1	0/1	0/0	0/0	0/0	0/0	0/0
Chromosome2-1841211	DEL	PRECISE	5148	0	0	0/0	0/0	0/1	0/0	0/1	0/0	0/1	0/0	0/0	0/0	0/0	0/0	0/0	1/1
Chromosome2-1841726	DEL	PRECISE	272	0	0	0/0	0/0	0/1	0/0	0/1	0/0	0/1	0/0	0/0	0/0	0/0	0/0	0/0	1/1
Chromosome2-1842771	INS	PRECISE	1258	0	0	0/0	0/0	0/1	0/0	0/0	0/0	0/0	0/0	0/0	0/0	0/0	0/0	0/0	0/0
Chromosome2-1842850	DEL	PRECISE	63	0	0	0/0	0/0	0/1	0/0	0/0	0/0	0/0	0/0	0/0	0/0	0/0	0/0	0/0	0/0
Chromosome2-1843039	DEL	PRECISE	5261	0	0	0/0	0/0	0/0	0/0	0/0	0/0	0/1	0/0	0/0	0/0	0/0	0/0	0/0	0/0
Chromosome2-1843080	DEL	PRECISE	171	0	0	0/0	0/0	0/1	0/0	0/0	0/0	0/0	0/0	0/0	0/0	0/0	0/0	0/0	0/0
Chromosome2-1843354	INS	PRECISE	63	0	0	0/0	0/0	0/0	0/0	0/1	0/0	0/0	0/0	0/0	0/0	0/0	0/0	0/0	1/1
Chromosome2-1843458	DEL	PRECISE	276	0	0	0/0	0/0	0/1	0/0	0/0	0/0	0/0	0/0	0/0	0/0	0/0	0/0	0/0	0/0
Chromosome2-1843616	INS	PRECISE	3140	0	0	0/0	0/0	1/1	0/0	0/0	0/0	0/0	0/0	0/0	0/0	0/0	0/0	0/0	0/0
Chromosome2-1844017	DEL	PRECISE	699	0	0	0/0	0/0	0/1	0/0	0/1	0/0	0/0	0/0	0/0	0/0	0/0	0/0	0/0	1/1
Chromosome2-1845122	DEL	PRECISE	4276	0	0	0/0	0/0	0/1	0/0	0/0	0/0	0/0	0/0	0/0	0/0	0/0	0/0	0/0	0/0
Chromosome2-1845552	DEL	PRECISE	1563	0	0	0/0	0/0	0/1	0/0	0/0	0/0	0/0	0/0	0/0	0/0	0/0	0/0	0/0	0/0
Chromosome2-1846584	INS	PRECISE	165	0	0	0/0	0/0	0/0	0/0	0/0	0/0	0/0	0/0	0/0	0/0	0/0	0/0	0/0	1/1
Chromosome2-1848594	DEL	PRECISE	12512	0	0	0/0	0/0	0/1	0/0	0/1	0/0	0/0	0/0	0/0	0/0	0/0	0/0	0/0	1/1
Chromosome2-1849877	INS	PRECISE	12745	0	0	0/0	0/0	0/0	0/0	0/0	0/0	0/0	0/1	0/0	0/0	0/0	0/0	0/0	0/0
Chromosome2-1849898	DEL	PRECISE	4164	0	0	0/0	0/0	0/0	0/0	0/0	0/0	0/0	0/0	0/0	0/0	0/0	0/0	0/0	1/1
Chromosome2-1852671	DEL	PRECISE	10917	0	0	0/0	0/0	0/0	0/0	0/1	0/0	0/0	0/0	0/0	0/0	0/0	0/0	0/0	0/0
Chromosome2-1854217	INS	PRECISE	5786	0	0	0/0	0/0	0/0	0/0	1/1	0/0	0/0	0/0	0/0	0/0	0/0	0/0	0/0	0/0
Chromosome2-1854217	BND	PRECISE	0	0	0	0/0	0/0	0/0	0/0	0/1	0/0	0/0	0/0	0/0	0/0	0/0	0/0	0/0	0/0
Chromosome2-1854409	DEL	PRECISE	651	0	0	0/0	0/0	0/1	0/0	0/1	0/0	0/0	0/0	0/0	0/0	0/0	0/0	0/0	1/1
Chromosome2-1855480	INS	PRECISE	84	0	0	0/0	0/0	0/0	0/0	0/1	0/0	0/0	0/0	0/0	0/0	0/0	0/0	0/0	0/0
Chromosome2-1855728	DEL	PRECISE	65	0	0	0/0	0/0	0/1	0/0	0/0	0/0	0/0	0/0	0/0	0/0	0/0	0/0	0/0	0/0
Chromosome2-1856043	DEL	PRECISE	72	0	0	0/0	0/0	0/0	0/0	0/0	0/0	0/0	0/0	0/0	0/0	0/0	0/0	0/0	1/1
Chromosome2-1856371	DEL	PRECISE	843	0	0	0/0	0/0	0/1	0/0	0/0	0/0	0/0	0/0	0/0	0/0	0/0	0/0	0/0	1/1
Chromosome2-1856800	DEL	PRECISE	2439	0	0	0/0	0/0	0/0	0/0	0/0	0/0	0/0	0/0	0/0	0/0	0/0	0/0	0/0	1/1
Chromosome2-1857095	DEL	PRECISE	18423	0	0	0/0	0/0	0/0	0/0	0/0	0/0	0/0	0/0	0/0	0/0	0/0	0/0	0/0	0/1
Chromosome2-1857322	DEL	PRECISE	12439	0	0	0/0	0/0	0/1	0/0	0/0	0/0	0/1	0/0	0/0	0/0	0/0	0/0	0/0	0/0
Chromosome2-1858566	BND	PRECISE	0	0	0	0/0	0/0	0/0	0/0	0/1	0/0	0/0	0/0	0/0	0/0	0/0	0/0	0/0	0/0
Chromosome2-1858698	INS	PRECISE	562	0	0	0/0	0/0	0/0	0/0	0/0	0/0	0/1	0/0	0/0	0/0	0/0	0/0	0/0	0/0
Chromosome2-1859180	INS	PRECISE	1054	0	0	0/0	0/0	0/0	0/0	0/0	0/0	0/1	0/0	0/0	0/0	0/0	0/0	0/0	0/0
Chromosome2-1859731	DEL	PRECISE	17473	0	0	0/0	0/0	0/0	0/0	0/0	0/0	0/0	0/0	0/0	0/0	0/0	0/0	0/0	1/1
Chromosome2-1859966	INS	PRECISE	11807	0	0	0/0	0/0	0/0	0/1	0/0	0/0	0/0	0/0	0/0	0/0	0/0	0/0	0/0	0/0
Chromosome2-1861749	INS	PRECISE	5364	0	0	0/0	0/0	0/1	0/0	0/0	0/0	0/0	0/0	0/0	0/0	0/0	0/0	0/0	0/0

Chromosome2-1861985	DEL	PRECISE	1105	0	0	0/0	0/0	0/0	0/0	0/1	0/0	0/0	0/0	0/0	0/0	0/0	0/0	0/0	1/1
Chromosome2-1862527	INS	PRECISE	750	0	0	0/0	0/0	0/1	0/0	0/0	0/0	0/0	0/0	0/0	0/0	0/0	0/0	0/0	0/0
Chromosome2-1862634	DEL	PRECISE	100	0	0	0/0	0/0	0/0	0/0	0/0	0/0	0/0	0/0	0/0	0/0	0/0	0/0	0/0	1/1
Chromosome2-1863234	DEL	PRECISE	1191	0	0	0/0	0/0	0/1	0/1	0/1	0/1	1/1	0/1	0/0	0/0	0/0	0/0	0/0	1/1
Chromosome2-1863595	DEL	PRECISE	78	0	0	0/0	0/0	0/1	0/0	0/0	0/0	0/0	0/0	0/0	0/0	0/0	0/0	0/0	1/1
Chromosome2-1865700	INS	PRECISE	5502	0	0	0/0	0/0	0/0	0/0	1/1	0/0	0/0	0/0	0/0	0/0	0/0	0/0	0/0	0/0
Chromosome2-1865818	INS	PRECISE	666	0	0	0/0	0/0	0/0	0/0	0/0	0/0	0/0	0/0	0/0	0/0	0/0	0/0	0/0	1/1
Chromosome2-1866054	DEL	PRECISE	886	0	0	0/0	0/0	0/1	0/1	0/1	0/1	1/1	0/1	0/0	0/0	0/0	0/0	0/0	1/1
Chromosome2-1867725	INS	PRECISE	1404	0	0	0/0	0/0	0/0	0/0	0/0	0/0	0/0	0/0	0/0	0/0	0/0	0/0	0/0	1/1
Chromosome2-1867872	INS	PRECISE	648	0	0.577	0/0	0/0	0/0	0/1	0/0	1/1	1/1	0/1	0/0	0/0	0/0	0/0	0/0	0/0
Chromosome2-1868298	DEL	PRECISE	70	0	0	0/0	0/0	0/1	0/0	0/0	0/0	0/0	0/0	0/0	0/0	0/0	0/0	0/0	0/0
Chromosome2-1868368	INS	PRECISE	4739	0	0	0/0	0/0	1/1	0/0	0/0	0/0	0/0	0/0	0/0	0/0	0/0	0/0	0/0	0/0
Chromosome2-1868958	DEL	PRECISE	68	0	0	0/0	0/0	0/1	0/0	0/0	0/0	0/0	0/0	0/0	0/0	0/0	0/0	0/0	0/0
Chromosome2-1869491	INS	PRECISE	137	0	0	0/0	0/0	0/1	0/0	0/0	0/0	0/0	0/0	0/0	0/0	0/0	0/0	0/0	0/0
Chromosome2-1869592	DEL	PRECISE	882	0.488	0.488	0/0	0/0	0/1	0/1	0/1	0/1	1/1	0/0	0/0	0/1	0/0	1/1	0/0	0/0
Chromosome2-1870987	DEL	PRECISE	294	0	0	0/0	0/0	0/0	0/0	0/0	0/0	0/0	0/0	0/0	0/1	0/0	1/1	0/0	0/0
Chromosome2-1871107	DEL	PRECISE	602	0	0	0/0	0/0	0/0	0/0	0/1	0/0	0/0	0/0	0/0	0/0	0/0	0/0	0/0	0/0
Chromosome2-1871236	INS	PRECISE	1555	0	0	0/0	0/0	0/1	0/0	0/0	0/0	0/0	0/0	0/0	0/0	0/0	0/0	0/0	0/0
Chromosome2-1871494	BND	PRECISE	0	0	0	0/0	0/0	0/0	0/0	0/0	0/1	0/1	0/1	0/0	0/0	0/0	0/0	0/0	0/0
Chromosome2-1872055	DEL	PRECISE	105	0	0	0/0	0/0	0/0	0/0	0/0	0/0	0/0	0/0	0/0	0/0	0/0	1/1	0/0	0/0
Chromosome2-1872569	DEL	PRECISE	2001	0	0	0/0	0/0	0/0	0/0	0/0	0/0	0/0	0/0	0/0	0/0	0/0	0/0	0/0	0/1
Chromosome2-1872756	DEL	PRECISE	52	0	0	0/1	0/0	0/0	0/0	0/0	0/0	0/0	0/0	0/0	0/0	0/0	0/0	0/0	0/0
Chromosome2-1873414	DEL	PRECISE	2001	0	0	0/0	0/0	0/0	0/0	0/0	0/0	0/0	0/0	0/0	0/0	0/0	0/0	0/0	0/1
Chromosome2-1873602	DEL	PRECISE	52	0	0	0/1	0/0	0/0	0/0	0/0	0/0	0/0	0/0	0/0	0/0	0/0	0/0	0/0	0/0
Chromosome2-1874260	DEL	PRECISE	2001	0	0	0/0	0/0	0/0	0/0	0/0	0/0	0/0	0/0	0/0	0/0	0/0	0/0	0/0	0/1
Chromosome2-1875880	DEL	PRECISE	63	0	0	0/0	0/0	0/1	0/1	0/0	0/1	1/1	0/1	0/0	0/0	0/0	0/0	0/0	0/0
Chromosome2-1875977	BND	PRECISE	0	0	0	0/0	0/0	0/0	0/0	0/0	0/0	0/1	0/0	0/0	0/0	0/0	0/0	0/0	0/0
Chromosome2-1875977	BND	PRECISE	0	0	0	0/0	0/0	0/0	0/0	0/0	0/0	0/1	0/0	0/0	0/0	0/0	0/0	0/0	0/0
Chromosome2-1877318	INS	PRECISE	153	0	0	0/0	0/0	0/0	0/0	0/0	0/0	0/0	0/0	0/0	0/0	0/0	0/0	0/0	1/1
Chromosome2-1879132	DEL	PRECISE	1077	0	0	0/0	0/0	0/1	0/1	0/0	0/1	1/1	0/1	0/0	0/0	0/0	0/0	0/0	0/0
Chromosome2-1879190	DEL	PRECISE	106	0	0	0/0	0/0	0/0	0/0	0/0	0/0	0/0	0/0	0/0	0/0	0/0	0/0	0/0	1/1
Chromosome2-1881138	DEL	PRECISE	68	0	0	0/0	0/0	0/0	0/0	0/0	0/0	0/0	0/0	0/0	0/0	0/0	0/0	0/0	1/1
Chromosome2-1881501	INS	PRECISE	60	0	0	0/0	0/0	0/0	0/0	0/0	0/0	0/0	0/0	0/0	0/0	0/0	0/0	0/0	1/1
Chromosome2-1881720	INS	PRECISE	1390	0	0	0/0	0/0	0/0	0/0	0/1	0/0	0/0	0/0	0/0	0/0	0/0	0/0	0/0	0/0
Chromosome2-1881785	DEL	PRECISE	50	0	0	0/0	0/0	0/0	0/0	0/0	0/0	0/0	0/0	0/0	0/0	0/0	0/0	0/0	1/1
Chromosome2-1882775	INS	PRECISE	141	0	0	0/0	0/0	0/0	0/0	0/1	0/0	0/0	0/0	0/0	0/0	0/0	0/0	0/0	0/0
Chromosome2-1883107	INS	PRECISE	56	0	0	0/0	0/0	0/0	0/0	0/1	0/0	0/0	0/0	0/0	0/0	0/0	0/0	0/0	0/0
Chromosome2-1884311	DEL	PRECISE	27162	0	0	0/0	0/0	0/0	0/0	1/1	0/0	0/0	0/0	0/0	0/0	0/0	0/0	0/0	0/0
Chromosome2-1887162	DEL	PRECISE	2605	0	0	0/0	0/0	0/0	0/0	0/0	0/0	0/0	0/0	0/0	0/0	0/0	0/0	0/0	1/1
Chromosome2-1887365	INS	PRECISE	4169	0	0	0/0	0/0	0/1	0/0	0/0	0/0	0/0	0/0	0/0	0/0	0/0	0/0	0/0	0/0
Chromosome2-1887598	DEL	PRECISE	65	0	0	0/0	0/0	0/1	0/0	0/0	0/0	0/0	0/0	0/0	0/0	0/0	0/0	0/0	0/0
Chromosome2-1889250	DEL	PRECISE	689	0	0	0/0	0/0	0/1	0/1	0/1	0/1	1/1	0/1	0/0	0/0	0/0	0/0	0/0	1/1
Chromosome2-1889362	INS	PRECISE	149	0	0	0/0	0/0	0/1	0/0	0/0	0/0	0/0	0/0	0/0	0/0	0/0	0/0	0/0	0/0
Chromosome2-1890463	BND	PRECISE	0	0	0	0/0	0/0	0/1	0/0	0/0	0/0	0/0	0/0	0/0	0/0	0/0	0/0	0/0	0/0
Chromosome2-1890785	INS	PRECISE	51	0	0	0/0	0/0	0/0	0/1	0/0	0/1	1/1	0/1	0/0	0/0	0/0	0/0	0/0	0/0
Chromosome2-1890867	INS	PRECISE	11111	0	0	0/0	0/0	0/0	0/0	0/0	0/0	0/0	0/0	0/0	0/0	0/0	0/0	0/0	0/1
Chromosome2-1892317	INS	PRECISE	5282	0	0	0/0	0/0	1/1	0/0	0/0	0/0	0/0	0/0	0/0	0/0	0/0	0/0	0/0	0/0
Chromosome2-1892317	INS	PRECISE	14545	0	0	0/0	0/0	0/0	0/0	0/1	0/0	0/0	0/0	0/0	0/0	0/0	0/0	0/0	0/0
Chromosome2-1892725	DEL	PRECISE	631	0	0	0/0	0/0	0/0	0/0	0/0	0/0	0/0	0/0	0/0	0/0	0/0	0/0	0/0	1/1
Chromosome2-1893767	INS	PRECISE	13551	0	0	0/0	0/0	0/0	0/0	0/0	0/0	0/0	0/0	0/1	0/0	0/0	0/0	0/0	0/0
Chromosome2-1893823	INS	PRECISE	2799	0	0	0/0	0/0	0/0	0/0	0/0	0/0	0/0	0/0	0/0	0/0	0/0	0/0	0/0	1/1
Chromosome2-1894568	DEL	PRECISE	84	0	0	0/0	0/0	0/0	0/1	0/0	0/1	0/1	0/1	0/0	0/0	0/0	0/0	0/0	0/0
Chromosome2-1895558	DEL	PRECISE	372	0	0	0/0	0/0	0/0	0/0	0/0	0/0	0/1	0/0	0/0	0/0	0/0	0/0	0/0	0/0
Chromosome2-1896480	INS	PRECISE	74	0	0	0/0	0/0	0/1	0/0	0/0	0/0	0/0	0/0	0/0	0/0	0/0	0/0	0/0	0/0
Chromosome2-1897421	INS	PRECISE	66	0	0	0/0	0/0	0/1	0/0	0/0	0/0	0/0	0/0	0/0	0/0	0/0	0/0	0/0	0/0
Chromosome2-1898989	INS	PRECISE	12490	0	0	0/0	0/0	0/0	0/0	0/1	0/0	0/0	0/0	0/0	0/0	0/0	0/0	0/0	0/0
Chromosome2-1898989	BND	PRECISE	0	0	0	0/0	0/0	0/0	0/0	0/1	0/0	0/0	0/0	0/0	0/0	0/0	0/0	0/0	0/0
Chromosome2-1898989	BND	PRECISE	0	0	0	0/0	0/0	0/0	0/0	0/1	0/0	0/0	0/0	0/0	0/0	0/0	0/0	0/0	0/0
Chromosome2-1899817	INS	PRECISE	357	0	0	0/0	0/0	0/0	0/0	0/0	0/0	0/0	0/0	0/0	0/0	0/0	0/0	0/0	1/1
Chromosome2-1899965	DEL	PRECISE	92	0	0	0/0	0/0	0/0	0/0	0/0	0/0	0/0	0/0	0/0	0/0	0/0	0/0	0/0	1/1
Chromosome2-1900815	DEL	PRECISE	182	0	0	0/0	0/0	0/1	0/0	0/1	0/0	0/0	0/0	0/0	0/0	0/0	0/0	0/0	0/0
Chromosome2-1901894	INS	PRECISE	62	0	0	0/0	0/0	0/1	0/0	0/1	0/0	0/0	0/0	0/0	0/0	0/0	0/0	0/0	0/0
Chromosome2-1902294	INS	PRECISE	3932	0	0	0/0	0/0	1/1	0/0	0/0	0/0	0/0	0/0	0/0	0/0	0/0	0/0	0/0	0/0
Chromosome2-1902392	INS	PRECISE	55	0	0	0/0	0/0	0/0	0/1	0/0	0/1	0/1	0/1	0/0	0/0	0/0	0/0	0/0	0/0
Chromosome2-1902777	DEL	PRECISE	437	0	0	0/0	0/0	0/1	0/0	0/1	0/0	0/0	0/0	0/0	0/0	0/0	0/0	0/0	0/0
Chromosome2-1903522	INS	PRECISE	2465	0	0	0/0	0/0	0/0	0/0	0/0	0/0	0/0	0/0	0/0	0/0	0/0	0/0	0/0	1/1
Chromosome2-1904070	DEL	PRECISE	86	0	0	0/0	0/0	0/0	0/0	0/1	0/0	0/0	0/0	0/0	0/0	0/0	0/0	0/0	0/0
Chromosome2-1908824	INS	PRECISE	480	0	0	0/0	0/0	0/0	0/0	0/1	0/0	0/0	0/0	0/0	0/0	0/0	0/0	0/0	0/0
Chromosome2-1909264	INS	PRECISE	6177	0	0	0/0	0/0	1/1	0/0	0/0	0/0	0/0	0/0	0/0	0/0	0/0	0/0	0/0	0/0
Chromosome2-1909701	DEL	PRECISE	104	0	0	0/0	0/0	0/1	0/0	0/0	0/0	0/0	0/0	0/0	0/0	0/0	0/0	0/0	0/0
Chromosome2-1912001	DEL	PRECISE	143	0	0	0/0	0/0	0/1	0/0	0/1	0/0	0/0	0/0	0/0	0/0	0/0	0/0	0/0	1/1
Chromosome2-1912199	INS	PRECISE	220	0	0	0/0	0/0	0/0	0/0	0/0	0/0	0/0	0/0	0/0	0/0	0/0	0/0	0/0	1/1
Chromosome2-1912929	BND	PRECISE	0	0	0	0/0	0/0	0/0	0/0	0/0	0/0	0/0	0/0	0/0	0/0	0/0	0/0	0/0	0/1

Chromosome2-1914339	DEL	PRECISE	1335	0	0	0/0	0/0	0/0	0/0	0/0	0/0	0/0	0/0	0/0	0/1	0/0	1/1	0/0	0/0
Chromosome2-1914340	DUP	PRECISE	19454557	0	0	0/0	0/0	0/0	0/0	0/0	0/0	0/0	0/1	0/1	0/0	0/0	0/1	0/0	0/0
Chromosome2-1916546	DEL	PRECISE	2930	0	0	0/0	0/0	0/0	0/0	0/0	0/0	0/1	0/0	0/0	0/0	0/0	0/0	0/0	0/0
Chromosome2-1916842	DEL	PRECISE	5312	0	0	0/0	0/0	0/0	0/0	0/0	0/0	0/1	0/0	0/0	0/0	0/0	0/0	0/0	0/0
Chromosome2-1917220	DEL	PRECISE	111	0	0	0/0	0/0	0/0	0/0	0/1	0/0	0/0	0/0	0/0	0/0	0/0	0/0	0/0	0/0
Chromosome2-1918719	DEL	PRECISE	57	0	0	0/0	0/0	0/0	0/0	0/1	0/0	0/0	0/0	0/0	0/0	0/0	0/0	0/0	0/0
Chromosome2-1919440	INS	PRECISE	673	0	0	0/0	0/0	0/0	0/0	0/1	0/0	0/0	0/0	0/0	0/0	0/0	0/0	0/0	0/0
Chromosome2-1919515	DEL	PRECISE	57	0	0	0/0	0/0	0/0	0/0	0/0	0/0	0/1	0/0	0/0	0/0	0/0	0/0	0/0	0/0
Chromosome2-1919754	INS	PRECISE	112	0	0	0/0	0/0	0/0	0/0	0/0	0/0	0/1	0/0	0/0	0/0	0/0	0/0	0/0	0/0
Chromosome2-1921359	INS	PRECISE	747	0	0	0/0	0/0	0/0	0/0	0/0	0/0	0/0	0/0	0/0	0/0	0/0	0/0	0/0	1/1
Chromosome2-1921793	INS	PRECISE	852	0	0	0/0	0/0	0/0	0/0	0/1	0/0	0/0	0/0	0/0	0/0	0/0	0/0	0/0	0/0
Chromosome2-1922767	INS	PRECISE	724	0	0	0/0	0/0	0/0	0/0	0/0	0/0	0/1	0/0	0/0	0/0	0/0	0/0	0/0	0/0
Chromosome2-1924954	INS	PRECISE	85	0	0	0/0	0/0	0/1	0/0	0/0	0/0	1/1	0/0	0/0	0/0	0/0	0/0	0/0	1/1
Chromosome2-1924969	INS	PRECISE	2814	0	0	0/0	0/0	0/0	0/0	1/1	0/0	0/0	0/0	0/0	0/0	0/0	0/0	0/0	0/0
Chromosome2-1925030	INS	PRECISE	411	0	0	0/0	0/0	0/0	0/0	0/1	0/0	0/0	0/0	0/0	0/0	0/0	0/0	0/0	0/0
Chromosome2-1925405	INS	PRECISE	885	0	0	0/0	0/0	0/1	0/0	0/0	0/0	0/0	0/0	0/0	0/0	0/0	0/0	0/0	0/0
Chromosome2-1926179	INS	PRECISE	55	0	0	0/0	0/0	0/1	0/0	0/0	0/0	0/0	0/0	0/0	0/0	0/0	0/0	0/0	0/0
Chromosome2-1927302	DEL	PRECISE	95	0	0	0/0	0/0	0/0	0/0	0/0	0/0	0/0	0/0	0/0	0/0	0/0	0/0	0/0	1/1
Chromosome2-1929624	DEL	PRECISE	125	0	0	0/0	0/0	0/1	0/0	0/0	0/0	0/0	0/0	0/0	0/0	0/0	0/0	0/0	0/0
Chromosome2-1930235	DEL	PRECISE	62	0	0	0/0	0/0	0/1	0/0	0/0	0/0	0/0	0/0	0/0	0/0	0/0	0/0	0/0	0/0
Chromosome2-1931181	DEL	PRECISE	590	0	0	0/0	0/0	0/1	0/1	0/1	0/1	1/1	0/1	0/0	0/0	0/0	0/0	0/0	1/1
Chromosome2-1932436	DEL	PRECISE	828	0.816	0.816	0/0	0/0	0/1	0/0	0/1	0/1	0/1	0/1	0/0	0/0	0/0	0/0	0/0	1/1
Chromosome2-1932709	INS	PRECISE	888	0	0	0/0	0/0	0/0	0/1	0/0	0/0	0/0	0/0	0/0	0/0	0/0	0/0	0/0	0/0
Chromosome2-1934379	INS	PRECISE	13075	0	0	0/0	0/0	0/1	0/0	0/0	0/0	0/0	0/0	0/0	0/0	0/0	0/0	0/0	0/0
Chromosome2-1934380	BND	PRECISE	0	0	0	0/0	0/0	0/1	0/0	0/0	0/0	0/0	0/0	0/0	0/0	0/0	0/0	0/0	0/0
Chromosome2-1935832	INS	PRECISE	646	0	0	0/0	0/0	0/0	0/0	0/0	0/0	0/1	0/0	0/0	0/0	0/0	0/0	0/0	0/0
Chromosome2-1935918	INS	PRECISE	331	0	0	0/0	0/0	0/0	0/1	0/0	0/0	0/0	0/0	0/0	0/0	0/0	0/0	0/0	0/0
Chromosome2-1936422	DEL	PRECISE	1352	0	0	0/0	0/0	0/1	0/0	0/0	0/0	0/0	0/0	0/0	0/0	0/0	0/0	0/0	0/0
Chromosome2-1936755	DEL	PRECISE	670	0	0	0/0	0/0	0/1	0/0	0/0	0/0	0/0	0/0	0/0	0/0	0/0	0/0	0/0	0/0
Chromosome2-1936855	INS	PRECISE	2220	0	0	0/0	0/0	0/0	0/0	0/0	0/0	0/0	0/0	0/0	0/0	0/0	0/0	0/0	1/1
Chromosome2-1936937	DEL	PRECISE	132	0	0	0/0	0/0	0/1	0/0	0/0	0/0	0/0	0/0	0/0	0/0	0/0	0/0	0/0	0/0
Chromosome2-1937040	INS	PRECISE	53	0	0	0/0	0/0	0/1	0/0	0/0	0/0	0/0	0/0	0/0	0/0	0/0	0/0	0/0	0/0
Chromosome2-1937496	INS	PRECISE	178	0	0	0/0	0/0	0/0	0/1	0/0	0/0	0/0	0/0	0/0	0/0	0/0	0/0	0/0	0/0
Chromosome2-1937571	INS	PRECISE	2692	0	0	0/0	0/0	0/0	0/0	1/1	0/0	0/0	0/0	0/0	0/0	0/0	0/0	0/0	0/0
Chromosome2-1938007	INS	PRECISE	240	0	0	0/0	0/0	0/1	0/0	0/0	0/0	0/0	0/0	0/0	0/0	0/0	0/0	0/0	0/0
Chromosome2-1938828	DEL	PRECISE	5287	0	0	0/0	0/0	0/0	0/0	0/0	0/1	0/0	0/0	0/0	0/0	0/0	0/0	0/0	0/0
Chromosome2-1938971	DEL	PRECISE	83	0	0	0/0	0/0	0/0	0/0	1/1	0/0	0/0	0/0	0/0	0/0	0/0	0/0	0/0	0/0
Chromosome2-1939657	BND	PRECISE	0	0	0	0/0	0/0	0/0	0/0	0/1	0/1	0/1	0/1	0/0	0/0	0/0	0/0	0/0	0/0
Chromosome2-1940023	DEL	PRECISE	81	0.577	0.577	0/0	0/0	0/0	0/0	0/0	0/1	0/1	0/1	0/0	0/0	0/0	0/0	0/0	0/0
Chromosome2-1940120	INS	PRECISE	1030	0	0	0/0	0/0	0/0	0/0	0/0	0/0	0/0	0/0	0/0	0/0	0/0	0/0	0/0	1/1
Chromosome2-1940253	DEL	PRECISE	654	0	0	0/0	0/0	0/1	0/0	0/0	0/0	0/0	0/0	0/0	0/0	0/0	0/0	0/0	0/0
Chromosome2-1940478	DEL	PRECISE	56	0	0	0/0	0/0	0/1	0/0	0/0	0/0	0/0	0/0	0/0	0/0	0/0	0/0	0/0	0/0
Chromosome2-1940539	INS	PRECISE	339	0	0	0/0	0/0	0/1	0/0	0/1	0/1	1/1	0/1	0/0	0/0	0/0	0/0	0/0	0/0
Chromosome2-1941486	INS	PRECISE	6552	0	0	0/0	0/0	0/0	0/0	0/0	0/0	0/0	0/0	0/0	0/0	0/0	0/0	0/0	1/1
Chromosome2-1941486	DEL	PRECISE	218	0	0	0/0	0/0	0/0	0/0	0/0	0/0	0/0	0/0	0/0	0/0	0/0	0/0	0/0	0/1
Chromosome2-1942867	DEL	PRECISE	63	0	0	0/0	0/0	0/0	0/0	0/0	0/0	0/0	0/0	0/0	0/0	0/0	0/0	0/0	1/1
Chromosome2-1943820	INS	PRECISE	264	0	0	0/0	0/0	0/0	0/0	0/0	0/0	0/0	0/0	0/0	0/0	0/0	0/0	0/0	1/1
Chromosome2-1944724	DEL	PRECISE	1474	0	0	0/0	0/0	0/0	0/0	0/1	0/0	0/0	0/0	0/0	0/0	0/0	0/0	0/0	0/0
Chromosome2-1945034	INS	PRECISE	340	0	0.707	0/0	0/0	0/0	0/0	0/1	0/0	0/1	0/0	0/0	0/0	0/0	0/0	0/0	0/0
Chromosome2-1945269	DEL	PRECISE	10472	0	0	0/0	0/0	0/0	0/0	0/1	0/0	0/0	0/0	0/0	0/0	0/0	0/0	0/0	0/0
Chromosome2-1945868	DEL	PRECISE	82	0	0	0/0	0/0	0/0	0/0	0/0	0/0	0/1	0/0	0/0	0/0	0/0	0/0	0/0	0/0
Chromosome2-1945897	DEL	PRECISE	2172	0	0	0/0	0/0	0/0	0/0	0/0	0/0	0/1	0/0	0/0	0/0	0/0	0/0	0/0	0/0
Chromosome2-1946078	INS	PRECISE	12521	0	0	0/0	0/0	0/1	0/0	0/0	0/0	0/0	0/0	0/0	0/0	0/0	0/0	0/0	0/0
Chromosome2-1946078	BND	PRECISE	0	0	0	0/0	0/0	0/1	0/0	0/0	0/0	0/0	0/0	0/0	0/0	0/0	0/0	0/0	0/0
Chromosome2-1946177	DEL	PRECISE	55	0	0	0/0	0/0	0/0	0/0	0/0	0/0	0/1	0/0	0/0	0/0	0/0	0/0	0/0	0/0
Chromosome2-1946277	DEL	PRECISE	81	0	0	0/0	0/0	0/0	0/1	0/0	0/0	0/0	0/0	0/0	0/0	0/0	0/0	0/0	0/0
Chromosome2-1946302	INS	PRECISE	3610	0	0	0/0	0/0	0/0	0/0	0/1	0/0	0/0	0/0	0/0	0/0	0/0	0/0	0/0	0/0
Chromosome2-1946594	DEL	PRECISE	124	0	0	0/0	0/0	0/0	0/0	0/0	0/1	1/1	0/1	0/0	0/0	0/0	0/0	0/0	0/0
Chromosome2-1946735	DEL	PRECISE	50	0	0	0/0	0/0	0/0	0/1	0/0	0/0	0/0	0/0	0/0	0/0	0/0	0/0	0/0	0/0
Chromosome2-1948632	DEL	PRECISE	290	0	0	0/0	0/0	0/0	0/0	0/1	0/0	0/0	0/0	0/0	0/0	0/0	0/0	0/0	0/0
Chromosome2-1949235	INS	PRECISE	133	0	0	0/0	0/0	0/0	0/0	0/0	0/0	0/0	0/0	0/0	0/0	0/0	0/0	0/0	1/1
Chromosome2-1951066	INS	PRECISE	211	0	0	0/0	0/0	0/0	0/1	0/0	0/0	0/0	0/0	0/0	0/0	0/0	0/0	0/0	0/0
Chromosome2-1951822	INS	PRECISE	1132	0	0	0/0	0/0	0/0	0/1	0/0	0/0	0/0	0/0	0/0	0/0	0/0	0/0	0/0	0/0
Chromosome2-1952935	DEL	PRECISE	443	0	0	0/0	0/0	0/0	0/0	0/1	0/0	0/0	0/0	0/0	0/0	0/0	0/0	0/0	1/1
Chromosome2-1953137	BND	PRECISE	0	0.756	0	0/0	0/1	0/1	0/1	0/0	0/1	0/0	0/0	0/0	0/1	0/0	0/0	0/0	0/1
Chromosome2-1954391	BND	PRECISE	0	0	0	0/0	0/1	0/1	0/1	0/1	0/1	0/0	0/0	0/0	0/1	0/0	0/0	0/0	0/1
Chromosome2-1954391	BND	PRECISE	0	0	0	0/0	0/0	0/0	0/0	0/0	0/1	0/0	0/0	0/0	0/0	0/0	0/0	0/0	0/0
Chromosome2-1954798	DEL	PRECISE	101	0	0	0/0	0/0	0/0	0/0	0/0	0/0	0/0	0/0	0/0	0/0	0/0	0/0	0/0	1/1
Chromosome2-1955077	INS	PRECISE	59	0	0	0/0	0/0	0/0	0/0	0/0	0/0	0/1	0/0	0/0	0/0	0/0	0/0	0/0	0/0
Chromosome2-1956065	DEL	PRECISE	829	0	0	0/0	1/1	1/1	1/1	1/1	1/1	1/1	1/1	1/1	1/1	0/0	0/0	0/0	0/0
Chromosome2-1956276	INS	PRECISE	146	0	0.408	0/0	0/0	0/1	0/1	0/1	0/1	0/1	0/1	0/0	0/0	0/0	0/0	0/0	0/0
Chromosome2-1956337	DEL	PRECISE	179	0	0	0/0	0/0	0/1	0/1	0/1	0/1	0/1	0/1	0/0	0/0	0/0	0/0	0/0	0/0
Chromosome2-1958914	DEL	PRECISE	89	0	0	0/0	0/0	0/0	0/0	0/0	0/0	0/0	0/0	0/0	0/0	0/0	0/0	0/0	1/1

Chromosome2-1959477	INS	PRECISE	1834	0	0	0/0	0/0	0/1	0/0	0/0	0/0	0/0	0/0	0/0	0/0	0/0	0/0	0/0	0/0
Chromosome2-1960913	INS	PRECISE	162	0	0	0/0	0/0	0/0	0/0	0/0	0/0	0/0	0/0	0/0	0/0	0/0	0/0	0/0	1/1
Chromosome2-1961179	INS	PRECISE	987	0	0	0/0	0/0	0/1	0/0	0/0	0/0	0/0	0/0	0/0	0/0	0/0	0/0	0/0	0/0
Chromosome2-1961699	INS	PRECISE	485	0	0.707	0/0	0/0	0/1	0/0	0/0	0/0	0/0	0/0	0/0	0/0	0/0	0/0	0/0	1/1
Chromosome2-1961917	DEL	PRECISE	3684	0	0	0/0	0/0	0/1	0/0	0/1	0/0	0/0	0/0	0/0	0/0	0/0	0/0	0/0	1/1
Chromosome2-1962510	INS	PRECISE	473	0	0	0/0	0/0	0/0	0/0	0/0	0/0	0/0	0/0	0/0	0/0	0/0	0/0	0/0	1/1
Chromosome2-1964179	DEL	PRECISE	112	0	0	0/0	0/0	0/0	0/0	0/0	0/0	0/0	0/0	0/0	0/0	0/0	0/0	0/0	1/1
Chromosome2-1967015	INS	PRECISE	211	0	0	0/0	0/0	0/0	0/0	0/0	0/0	0/0	0/0	0/0	0/0	0/0	0/0	0/0	1/1
Chromosome2-1967124	DEL	PRECISE	105	0	0	0/0	0/0	0/0	0/0	0/0	0/0	0/0	0/0	0/0	0/0	0/0	0/0	0/0	1/1
Chromosome2-1967549	INS	PRECISE	1374	0	0	0/0	0/0	0/0	0/0	0/1	0/0	0/0	0/0	0/0	0/0	0/0	0/0	0/0	1/1
Chromosome2-1968669	INS	PRECISE	192	0	0.707	0/0	0/0	0/0	0/0	0/1	0/0	0/0	0/0	0/0	0/0	0/0	0/0	0/0	1/1
Chromosome2-1969105	DEL	PRECISE	662	0	0	0/0	0/0	0/1	0/0	0/1	0/0	0/0	0/0	0/0	0/0	0/0	0/0	0/0	1/1
Chromosome2-1970914	INS	PRECISE	62	0	0	0/0	0/0	0/1	0/0	0/0	0/0	0/0	0/0	0/0	0/0	0/0	0/0	0/0	0/0
Chromosome2-1971057	DEL	PRECISE	89	0	0	0/0	0/0	0/1	0/0	0/0	0/0	0/0	0/0	0/0	0/0	0/0	0/0	0/0	0/0
Chromosome2-1971433	INS	PRECISE	212	0	0	0/0	0/0	0/0	0/1	0/0	0/1	1/1	0/1	0/0	0/0	0/0	0/0	0/0	0/0
Chromosome2-1972689	DEL	PRECISE	962	0	0	0/0	0/0	0/1	0/0	0/1	0/0	0/0	0/0	0/0	0/0	0/0	0/0	0/0	0/0
Chromosome2-1973897	INS	PRECISE	5519	0	0	0/0	0/0	0/0	0/0	0/0	0/0	0/0	0/0	0/0	0/0	0/0	0/0	0/0	1/1
Chromosome2-1975478	INS	PRECISE	211	0	0	0/0	0/0	0/1	0/0	0/0	0/0	0/0	0/0	0/0	0/0	0/0	0/0	0/0	0/0
Chromosome2-1975590	INS	PRECISE	667	0	0	0/0	0/0	0/1	0/0	0/0	0/0	0/0	0/0	0/0	0/0	0/0	0/0	0/0	0/0
Chromosome2-1976372	INS	PRECISE	150	0	0	0/0	0/0	0/0	0/0	0/0	0/0	0/0	0/0	0/0	0/0	0/0	0/0	0/0	1/1
Chromosome2-1976549	INS	PRECISE	150	0	0	0/0	0/0	0/0	0/0	0/0	0/0	0/0	0/0	0/0	0/0	0/0	0/0	0/0	1/1
Chromosome2-1976885	INS	PRECISE	312	0	0	0/0	0/0	0/0	0/0	0/1	0/0	0/0	0/0	0/0	0/0	0/0	0/0	0/0	0/0
Chromosome2-1976903	DEL	PRECISE	67	0	0	0/0	0/0	0/0	0/0	0/1	0/0	0/0	0/0	0/0	0/0	0/0	0/0	0/0	0/0
Chromosome2-1977015	INS	PRECISE	137	0	0	0/0	0/0	0/0	0/0	0/1	0/0	0/0	0/0	0/0	0/0	0/0	0/0	0/0	0/0
Chromosome2-1977347	DEL	PRECISE	1374	0	0	0/0	0/0	1/1	0/0	0/0	0/0	0/0	0/0	0/0	0/0	0/0	0/0	0/0	0/0
Chromosome2-1977527	DEL	PRECISE	81	0	0	0/0	0/0	0/1	0/0	0/0	0/0	0/0	0/0	0/0	0/0	0/0	0/0	0/0	1/1
Chromosome2-1977709	DEL	PRECISE	53	0	0	0/0	0/0	0/1	0/0	0/0	0/0	0/0	0/0	0/0	0/0	0/0	0/0	0/0	1/1
Chromosome2-1979190	DEL	PRECISE	500	0	0	0/0	0/0	0/1	0/0	0/0	0/0	0/0	0/0	0/0	0/0	0/0	0/0	0/0	0/0
Chromosome2-1980378	DEL	PRECISE	326	0	0	0/0	0/0	0/1	0/0	0/0	0/0	0/0	0/0	0/0	0/0	0/0	0/0	0/0	1/1
Chromosome2-1981097	INS	PRECISE	7930	0	0	0/0	0/0	0/0	0/1	0/0	0/0	0/0	0/0	0/0	0/0	0/0	0/0	0/0	0/0
Chromosome2-1981097	BND	PRECISE	0	0	0	0/0	0/0	0/0	0/1	0/0	0/0	0/0	0/0	0/0	0/0	0/0	0/0	0/0	0/0
Chromosome2-1981243	INS	PRECISE	997	0	0	0/0	0/0	0/0	0/1	0/0	0/0	0/0	0/0	0/0	0/0	0/0	0/0	0/0	0/0
Chromosome2-1985185	DEL	PRECISE	1617	0	0	0/0	0/0	0/0	0/0	0/0	0/0	0/0	0/0	1/1	0/0	0/0	0/1	0/1	0/0
Chromosome2-1985218	DEL	PRECISE	253	0	0	0/0	0/0	0/0	0/0	0/0	0/0	0/0	0/0	0/0	1/1	0/0	0/1	0/0	0/0
Chromosome2-1986049	DEL	PRECISE	198	0	0	0/0	0/0	0/0	0/0	0/0	0/0	0/0	0/0	0/0	0/0	0/0	0/0	0/0	0/1
Chromosome2-1986061	INS	PRECISE	399	0	0.577	0/0	0/0	0/0	0/0	0/0	0/0	0/0	0/0	1/1	0/0	0/0	0/1	0/1	0/0
Chromosome2-1987147	DEL	PRECISE	68	0	0	0/0	0/0	0/0	0/0	0/0	0/0	0/0	0/0	0/0	0/0	0/0	0/0	0/0	0/1
Chromosome2-1988096	INS	PRECISE	132	0	0	0/0	0/0	0/0	0/0	0/0	0/0	0/0	0/0	0/0	1/1	0/0	0/1	0/0	0/0
Chromosome2-1988403	DEL	PRECISE	187	0	0	0/0	0/0	0/0	0/0	0/0	0/0	0/0	0/0	0/0	0/0	0/1	0/0	0/0	0/0
Chromosome2-1989412	DEL	PRECISE	689	0	0	0/0	0/0	0/0	0/0	0/0	0/0	0/0	0/0	1/1	1/1	0/1	1/1	0/1	0/0
Chromosome2-1993016	INS	PRECISE	1335	0	0.577	0/0	0/0	0/0	0/0	0/0	0/0	0/0	0/0	1/1	0/0	0/0	0/1	0/1	0/0
Chromosome2-1993768	INS	PRECISE	1967	0	0.707	0/0	0/0	0/0	0/0	0/0	0/0	0/0	0/0	0/0	1/1	0/0	0/1	0/0	0/0
Chromosome2-1996246	INS	PRECISE	276	0	0.577	0/0	0/0	0/0	0/0	0/0	0/0	0/0	0/0	1/1	0/0	0/0	0/1	0/1	0/0
Chromosome2-1998727	INS	PRECISE	1184	0	0	0/0	0/0	0/0	0/0	0/0	0/0	0/0	0/0	0/0	0/0	0/1	0/0	0/0	0/0
Chromosome2-1999093	INS	PRECISE	53	0	0	0/0	0/0	0/0	0/0	0/0	0/0	0/0	0/0	1/1	1/1	0/1	1/1	0/1	0/0
Chromosome2-2000196	DEL	PRECISE	162	0	0	0/0	0/0	0/0	0/0	0/0	0/0	0/0	0/0	1/1	1/1	0/0	1/1	0/1	0/0
Chromosome2-2000307	DEL	PRECISE	77	0	0	0/0	0/0	0/0	0/0	0/0	0/0	0/0	0/0	1/1	1/1	0/1	1/1	0/1	0/0
Chromosome2-2003242	DEL	PRECISE	349	0	0	0/0	0/0	0/0	0/0	0/0	0/0	0/0	0/0	0/0	0/0	0/1	0/0	0/0	0/0
Chromosome2-2004465	DEL	PRECISE	70	0	0	0/0	0/0	0/0	0/0	0/0	0/0	0/0	0/0	1/1	0/0	0/1	0/1	0/1	0/0
Chromosome2-2006141	INS	PRECISE	60	0	0	0/0	0/0	0/0	0/0	0/0	0/0	0/0	0/0	0/0	1/1	0/0	0/1	0/0	0/0
Chromosome2-2006622	INS	PRECISE	1298	0	0	0/0	0/0	0/0	0/0	0/0	0/0	0/0	0/0	0/0	0/0	0/1	0/0	0/0	0/0
Chromosome2-2009725	DEL	PRECISE	6979	0	0	0/0	0/0	0/0	0/0	0/0	0/0	0/0	0/0	1/1	0/0	0/0	0/1	0/1	0/0
Chromosome2-2009726	DUP	PRECISE	8429275	0	0	0/0	0/0	0/0	0/0	0/0	0/0	0/0	0/0	0/0	0/1	0/0	0/0	0/0	0/0
Chromosome2-2009791	DEL	PRECISE	120	0	0	0/0	0/0	0/0	1/1	0/0	0/0	0/0	0/0	0/0	0/0	0/0	0/0	0/0	0/0
Chromosome2-2010374	DEL	PRECISE	75	0	0	0/0	0/0	1/1	0/0	1/1	0/0	0/0	0/0	0/0	0/0	0/0	0/0	0/0	0/0
Chromosome2-2010633	INS	PRECISE	72	0	0.577	0/0	0/0	0/0	0/0	0/0	0/0	0/0	0/0	1/1	0/0	0/0	0/1	0/1	0/0
Chromosome2-2011189	INS	PRECISE	54	0	0.577	0/0	0/0	0/0	0/0	0/0	0/0	0/0	0/0	1/1	0/0	0/0	0/1	0/1	0/0
Chromosome2-2012224	INS	PRECISE	315	0	0.707	0/0	0/0	0/0	0/0	0/0	0/0	0/0	0/0	0/0	1/1	0/0	0/1	0/0	0/0
Chromosome2-2012427	DEL	PRECISE	132	0	0	0/0	0/0	0/0	0/0	0/0	0/0	0/0	0/0	1/1	1/1	0/1	1/1	0/1	0/0
Chromosome2-2013998	DEL	PRECISE	117	0.447	0.447	0/0	0/0	0/0	0/0	0/0	0/0	0/0	0/0	1/1	1/1	0/1	1/1	0/1	0/0
Chromosome2-2014029	DEL	PRECISE	10889	0	0	0/0	0/0	0/0	0/0	0/0	0/0	0/0	0/0	0/1	0/0	0/1	0/1	0/0	0/0
Chromosome2-2016971	DEL	PRECISE	701	0	0	0/0	0/0	0/0	0/0	0/0	0/0	0/0	0/0	1/1	0/0	0/1	0/1	0/1	0/0
Chromosome2-2020074	INV	PRECISE	1026681	0	0	0/0	0/0	0/0	0/0	0/0	0/0	0/0	0/0	1/1	0/0	0/0	0/0	0/0	0/0
Chromosome2-2020088	DEL	PRECISE	65	0	0	0/0	0/0	0/0	0/0	0/0	0/0	0/0	0/0	1/1	0/0	0/0	0/1	0/1	0/0
Chromosome2-2020156	DEL	PRECISE	1528	0	0	0/0	0/0	0/0	0/0	0/0	0/0	0/0	0/0	0/0	1/1	0/0	0/1	0/0	0/0
Chromosome2-2023193	INS	PRECISE	55	0	0	0/0	0/0	0/0	1/1	0/0	0/0	0/0	0/0	0/0	1/1	0/1	0/1	0/0	0/0
Chromosome2-2024151	DEL	PRECISE	59	0	0	0/0	0/0	0/0	0/0	0/0	0/0	0/0	0/0	0/0	1/1	0/0	0/1	0/0	0/0
Chromosome2-2024775	DEL	PRECISE	4616	0	0	0/0	0/0	0/0	0/0	0/0	0/0	0/0	0/0	1/1	1/1	0/1	1/1	0/1	0/0
Chromosome2-2026475	DEL	PRECISE	4645	0	0	0/0	0/0	0/0	0/0	0/0	0/0	0/0	0/0	0/0	0/0	0/1	0/0	0/0	0/0
Chromosome2-2026699	INV	PRECISE	4782463	0	0	0/0	0/0	0/0	0/0	0/0	0/0	0/0	0/0	0/0	0/1	0/0	0/0	0/0	0/0
Chromosome2-2027857	INS	PRECISE	677	0	0	0/0	0/0	0/0	0/0	0/0	0/0	0/0	0/0	0/0	0/0	1/1	0/0	0/0	0/0
Chromosome2-2027901	DEL	PRECISE	557	0	0	0/0	0/0	0/0	0/0	0/0	0/0	0/0	0/0	1/1	1/1	0/1	1/1	0/1	0/0
Chromosome2-2028362	DEL	PRECISE	1032	0	0	0/0	0/0	0/0	0/0	0/0	0/0	0/0	0/0	1/1	1/1	0/0	1/1	0/1	0/0

Chromosome2-2028610 DEL	PRECISE	406	0	0	0/0	0/0	0/0	0/0	0/0	0/0	0/0	0/0	0/0	0/0	0/1	0/0	0/0	0/0
Chromosome2-2029154 DEL	PRECISE	350	0.447	0.447	0/0	0/0	0/0	0/0	0/0	0/0	0/0	0/0	1/1	1/1	0/1	1/1	0/1	0/0
Chromosome2-2029784 DEL	PRECISE	1206	0	0	0/0	0/0	0/0	0/0	0/0	0/0	0/0	0/0	0/0	1/1	0/1	1/1	0/0	0/0
Chromosome2-2030076 DEL	PRECISE	91	0	0	0/0	0/0	0/0	0/0	0/0	0/0	0/0	0/0	0/0	1/1	0/0	1/1	0/0	0/0
Chromosome2-2030139 DEL	PRECISE	996	0	0	0/0	0/0	0/0	0/0	0/0	0/0	0/0	0/0	1/1	1/1	0/1	1/1	0/1	0/0
Chromosome2-2030386 DEL	PRECISE	1165	0	0	0/0	0/0	0/0	0/0	0/0	0/0	0/0	0/0	1/1	1/1	0/1	1/1	0/1	0/0
Chromosome2-2030698 DEL	PRECISE	827	0	0	0/0	0/0	0/0	0/0	0/0	0/0	0/0	0/0	1/1	1/1	0/1	1/1	0/1	0/0
Chromosome2-2031089 INS	PRECISE	161	0	0.577	0/0	0/0	0/0	0/0	0/0	0/0	0/0	0/0	1/1	0/0	0/0	0/1	0/1	0/0
Chromosome2-2031957 DEL	PRECISE	54	0	0	0/0	0/0	0/0	0/0	0/0	0/0	0/0	0/0	0/0	0/0	0/1	0/0	0/0	0/0
Chromosome2-2034273 DEL	PRECISE	50	0	0	0/0	0/0	0/0	0/0	0/0	0/0	0/0	0/0	0/0	1/1	0/1	0/1	0/0	0/0
Chromosome2-2035987 DEL	PRECISE	699	0	0	0/0	0/0	0/0	0/0	0/0	0/0	0/0	0/0	1/1	1/1	0/1	1/1	0/1	0/0
Chromosome2-2037277 INS	PRECISE	51	0	0	0/0	0/0	0/0	0/0	0/0	0/0	0/0	0/0	1/1	0/0	0/0	0/1	0/1	0/0
Chromosome2-2037403 INS	PRECISE	953	0	0.577	0/0	0/0	0/0	0/0	0/0	0/0	0/0	0/0	0/0	1/1	0/1	0/1	0/0	0/0
Chromosome2-2038610 DEL	PRECISE	132	0	0	0/0	0/0	0/0	0/0	0/0	0/0	0/0	0/0	1/1	1/1	0/1	1/1	0/1	0/0
Chromosome2-2041092 DEL	PRECISE	150	0	0	0/0	0/0	0/0	0/0	0/0	0/0	0/0	0/0	0/0	1/1	0/1	0/1	0/0	0/0
Chromosome2-2041204 DEL	PRECISE	114	0	0	0/0	0/0	0/0	0/0	0/0	0/0	0/0	0/0	1/1	1/1	0/1	1/1	0/1	0/0
Chromosome2-2042098 INV	PRECISE	13976418	0	0	0/0	0/0	0/0	0/0	0/0	0/0	0/0	0/0	0/0	0/0	0/0	0/1	0/0	0/0
Chromosome2-2043577 DEL	PRECISE	294	0	0	0/0	0/0	0/0	0/0	0/0	0/0	0/0	0/0	1/1	1/1	0/1	1/1	0/1	0/0
Chromosome2-2048806 DEL	PRECISE	344	0	0	0/0	0/0	0/0	0/0	0/0	0/0	0/0	0/0	1/1	1/1	0/0	1/1	0/1	0/0
Chromosome2-2048960 INS	PRECISE	108	0	0	0/0	0/0	0/0	0/0	0/0	0/0	0/0	0/0	1/1	0/0	0/0	0/1	0/1	0/0
Chromosome2-2049118 DEL	PRECISE	211	0	0	0/0	0/0	0/0	0/0	0/0	0/0	0/0	0/0	1/1	1/1	0/0	1/1	0/1	0/0
Chromosome2-2049555 INS	PRECISE	184	0	0	0/0	0/0	0/0	0/0	0/0	0/0	0/0	0/0	1/1	1/1	0/0	1/1	1/1	0/0
Chromosome2-2049786 DEL	PRECISE	33458	0	0	0/0	0/0	0/0	0/0	0/0	0/0	0/0	0/0	0/0	0/0	0/0	0/1	0/0	0/0
Chromosome2-2050033 DEL	PRECISE	59	0	0	0/0	0/0	0/0	0/0	0/0	0/0	0/0	0/0	1/1	0/0	0/0	1/1	0/1	0/0
Chromosome2-2053625 DEL	PRECISE	1551	0	0	0/0	0/0	0/0	0/0	0/0	0/0	0/0	0/0	1/1	1/1	0/1	1/1	0/1	0/0
Chromosome2-2054395 DEL	PRECISE	12353	0	0	0/0	0/0	0/0	0/0	0/0	0/0	0/0	0/0	0/0	1/1	0/1	0/1	0/0	0/0
Chromosome2-2055757 DEL	PRECISE	1550	0	0	0/0	0/0	0/0	0/0	0/0	0/0	0/0	0/0	1/1	1/1	0/1	1/1	0/1	0/0
Chromosome2-2056070 DEL	PRECISE	1284	0	0	0/0	0/0	0/0	0/0	0/0	0/0	0/0	0/0	0/0	1/1	0/1	0/1	0/0	0/0
Chromosome2-2058045 INS	PRECISE	6077	0	0	0/0	0/0	0/0	0/0	0/0	0/0	1/1	0/0	0/0	0/0	0/0	0/0	0/0	0/0
Chromosome2-2059510 INS	PRECISE	3414	0	0	0/0	0/0	0/0	0/0	0/0	0/0	1/1	0/0	0/0	0/0	0/0	0/0	0/0	0/0
Chromosome2-2060407 DEL	PRECISE	349	0	0	0/0	0/0	0/0	0/0	0/0	0/0	1/1	0/0	0/0	0/0	0/0	0/0	0/0	0/0
Chromosome2-2061079 DEL	PRECISE	7950	0	0	0/0	0/0	0/0	0/0	0/0	0/0	1/1	0/0	0/0	0/0	0/0	0/0	0/0	0/0
Chromosome2-2061928 DEL	PRECISE	947	0	0	0/0	0/0	0/0	0/0	0/0	0/0	1/1	0/0	1/1	0/0	0/0	0/1	0/1	0/0
Chromosome2-2062442 DEL	PRECISE	17745	0	0	0/0	0/0	0/0	0/0	0/0	0/0	1/1	0/0	0/0	0/0	0/0	0/0	0/0	0/0
Chromosome2-2065013 INS	PRECISE	76	0	0	0/0	0/0	0/0	0/0	0/0	0/0	1/1	0/0	0/0	0/0	0/0	0/0	0/0	0/0
Chromosome2-2065284 INS	PRECISE	2209	0	0	0/0	0/0	0/0	0/0	0/0	0/0	1/1	0/0	0/0	0/0	0/0	0/0	0/0	0/0
Chromosome2-2065823 DEL	PRECISE	218	0	0	0/0	0/0	0/0	0/0	0/0	0/0	1/1	0/0	0/0	0/0	0/0	0/0	0/0	0/0
Chromosome2-2067807 DEL	PRECISE	201	0	0	0/0	0/0	0/0	0/0	0/0	0/0	0/0	0/0	0/0	1/1	0/1	0/1	0/0	0/0
Chromosome2-2069999 DEL	PRECISE	94	0	0	0/0	0/0	0/0	0/0	0/0	0/0	0/0	0/0	0/1	0/0	0/0	0/0	0/1	0/0
Chromosome2-2072065 INS	PRECISE	339	0	0.577	0/0	0/0	0/0	0/0	0/0	0/0	0/0	0/0	1/1	0/0	0/0	0/1	0/1	0/0
Chromosome2-2072598 DEL	PRECISE	2248	0	0	0/0	0/0	0/0	0/0	0/0	0/0	0/0	0/0	1/1	0/0	0/0	1/1	0/1	0/0
Chromosome2-2073335 DEL	PRECISE	437	0	0	0/0	0/0	0/0	0/0	0/0	0/0	0/0	0/0	1/1	0/0	0/0	1/1	0/1	0/0
Chromosome2-2076648 DEL	PRECISE	51	0.548	0.548	0/0	0/0	0/0	0/0	0/0	0/0	0/0	0/0	1/1	1/1	0/1	1/1	0/1	0/0
Chromosome2-2078827 DEL	PRECISE	225	0	0	0/0	0/0	0/0	0/0	0/0	0/0	0/0	0/0	1/1	0/0	0/0	0/1	0/1	0/0
Chromosome2-2080585 DEL	PRECISE	804	0	0	0/0	0/0	0/0	0/0	0/0	0/0	0/0	0/0	1/1	1/1	0/1	1/1	0/1	0/0
Chromosome2-2080980 DEL	PRECISE	133	0.447	0.447	0/0	0/0	0/0	0/0	0/0	0/0	0/0	0/0	1/1	1/1	0/1	1/1	0/1	0/0
Chromosome2-2081767 DEL	PRECISE	160	0	0	0/0	0/0	0/0	0/0	0/0	0/0	0/0	0/0	1/1	1/1	0/1	1/1	0/1	0/0
Chromosome2-2082780 DEL	PRECISE	52	0	0	0/0	0/0	0/0	0/0	0/0	0/0	0/0	0/0	0/0	0/0	0/1	0/0	0/0	0/0
Chromosome2-2083393 INS	PRECISE	1188	0	0	0/0	0/0	0/0	0/0	0/0	0/0	0/0	0/0	0/0	0/0	0/1	0/0	0/0	0/0
Chromosome2-2083467 INS	PRECISE	587	0	0	0/0	0/0	0/0	0/0	0/0	0/0	0/0	0/0	0/0	0/0	0/1	0/0	0/0	0/0
Chromosome2-2084576 INS	PRECISE	67	0	0	0/0	0/0	0/0	0/0	0/0	0/0	0/0	0/0	1/1	1/1	0/0	1/1	0/1	0/0
Chromosome2-2085600 INS	PRECISE	2398	0	0.577	0/0	0/0	0/0	0/0	0/0	0/0	0/0	0/0	0/0	0/0	0/0	0/1	1/1	0/0
Chromosome2-2085743 DEL	PRECISE	3413	0.5	0.5	0/0	0/0	0/0	0/0	0/0	0/0	0/0	0/0	1/1	0/0	0/1	0/1	0/0	0/0
Chromosome2-2086472 DEL	PRECISE	334	0	0	0/0	0/0	0/0	0/0	0/0	0/0	0/0	0/0	1/1	0/0	0/1	0/1	0/0	0/0
Chromosome2-2086855 DEL	PRECISE	57	0	0	0/0	0/0	0/0	0/0	0/0	0/0	0/0	0/0	1/1	0/0	0/1	0/1	0/1	0/0
Chromosome2-2091806 DEL	PRECISE	871	0	0	0/0	0/0	0/0	0/0	0/0	0/0	0/0	0/0	1/1	0/0	0/1	0/1	0/1	0/0
Chromosome2-2092333 DEL	PRECISE	357	0	0	0/0	0/0	0/0	0/0	0/0	0/0	0/0	0/0	1/1	0/0	0/1	0/1	0/1	0/0
Chromosome2-2093457 DEL	PRECISE	4194	0	0	0/0	0/0	0/0	0/0	0/0	0/0	0/0	0/0	1/1	1/1	0/1	1/1	0/1	0/0
Chromosome2-2096478 DEL	PRECISE	82	0	0	0/0	0/0	0/0	0/0	0/0	0/0	0/0	0/0	1/1	0/0	0/0	0/1	0/1	0/0
Chromosome2-2096823 INS	PRECISE	770	0	0.577	0/0	0/0	0/0	0/0	0/0	0/0	0/0	0/0	1/1	0/0	0/0	0/1	0/1	0/0
Chromosome2-2097310 INS	PRECISE	15423	0	0	0/0	0/0	0/0	0/0	0/0	0/0	0/0	0/0	0/0	0/0	0/1	0/0	0/0	0/0
Chromosome2-2097698 INS	PRECISE	68	0	0	0/0	0/0	0/0	0/0	0/0	0/0	0/0	0/0	1/1	0/0	0/0	0/1	0/1	0/0
Chromosome2-2098125 DEL	PRECISE	1328	0	0	0/0	0/0	0/0	0/0	0/0	0/0	0/0	0/0	1/1	0/0	0/0	0/1	0/1	0/0
Chromosome2-2098553 INS	PRECISE	124	0	0.577	0/0	0/0	0/0	0/0	0/0	0/0	0/0	0/0	1/1	0/0	0/0	0/1	0/1	0/0
Chromosome2-2098644 DEL	PRECISE	3666	0	0	0/0	0/0	0/0	0/0	0/0	0/0	0/0	0/0	0/0	0/0	0/1	0/0	0/0	0/0
Chromosome2-2099008 INS	PRECISE	57	0	0.577	0/0	0/0	0/0	0/0	0/0	0/0	0/0	0/0	1/1	0/0	0/0	0/1	0/1	0/0
Chromosome2-2099815 INS	PRECISE	1105	0	0	0/0	0/0	0/0	0/0	0/0	0/0	0/0	0/0	0/0	0/0	0/1	0/0	0/0	0/0
Chromosome2-2101699 DEL	PRECISE	229	0	0	0/0	0/0	0/0	0/0	0/0	0/0	0/0	0/0	0/0	0/0	0/1	0/0	0/0	0/0
Chromosome2-2103141 INS	PRECISE	1224	0	0.707	0/0	0/0	0/0	0/0	0/0	0/0	0/0	0/0	0/0	1/1	0/0	0/1	0/0	0/0
Chromosome2-2103360 INS	PRECISE	423	0	0	0/0	0/0	0/0	0/0	0/0	0/0	0/0	0/0	1/1	0/0	0/0	0/0	0/1	0/0
Chromosome2-2103885 DEL	PRECISE	1095	0	0	0/0	0/0	0/0	0/0	0/0	0/0	0/0	0/0	1/1	0/0	0/0	0/0	0/1	0/0
Chromosome2-2104483 INS	PRECISE	362	0	0.577	0/0	0/0	0/0	0/0	0/0	0/0	0/0	0/0	1/1	0/0	0/0	0/1	0/1	0/0
Chromosome2-2106719 DEL	PRECISE	355	0	0	0/0	0/0	0/0	0/0	0/0	0/0	0/0	0/0	1/1	0/0	0/0	0/1	0/1	0/0

Chromosome2-2107564 DEL	PRECISE	51	0	0	0/0	0/0	0/0	0/0	0/0	0/0	0/0	0/0	0/0	1/1	0/0	0/1	0/0	0/0
Chromosome2-2107877 INS	PRECISE	1125	0	0.707	0/0	0/0	0/0	0/0	0/0	0/0	0/0	0/0	0/0	1/1	0/0	0/1	0/0	0/0
Chromosome2-2107885 DEL	PRECISE	53	0	0	0/0	0/0	0/0	0/0	0/0	0/0	0/0	0/0	1/1	0/0	0/0	0/1	0/1	0/0
Chromosome2-2107976 INS	PRECISE	177	0	0.5	0/0	0/0	0/0	0/0	0/0	0/0	0/0	0/0	1/1	1/1	0/0	1/1	0/1	0/0
Chromosome2-2108251 DEL	PRECISE	3582142	0	0	0/0	0/0	0/0	0/0	0/0	0/0	0/0	0/0	1/1	0/0	0/0	1/1	0/1	0/0
Chromosome2-2108461 INS	PRECISE	146	0	0	0/0	0/0	0/0	0/0	0/0	0/0	0/0	0/0	1/1	0/0	0/0	0/1	0/1	0/0
Chromosome2-2109576 DEL	PRECISE	198	0	0	0/0	0/0	0/0	0/0	0/0	0/0	0/0	0/0	0/0	0/0	0/0	0/0	0/1	0/0
Chromosome2-2112745 DEL	PRECISE	57	0	0	0/0	0/0	0/0	0/0	0/1	0/0	0/0	0/0	0/1	0/0	0/0	0/0	0/0	0/0
Chromosome2-2114080 INS	PRECISE	952	0	0	0/0	0/0	0/0	0/1	0/0	0/0	0/0	0/0	0/0	0/0	0/0	0/0	0/0	0/0
Chromosome2-2114131 DEL	PRECISE	2711	0	0	0/0	0/0	0/0	0/0	0/0	0/0	0/0	0/0	0/1	0/0	0/0	1/1	0/1	0/0
Chromosome2-2114614 INS	PRECISE	1670	0	0.707	0/0	0/0	0/0	0/0	0/0	0/0	0/0	0/0	0/0	0/0	0/0	1/1	0/0	0/0
Chromosome2-2114614 INS	PRECISE	15094	0	0	0/0	0/0	0/0	0/0	0/0	0/0	0/0	0/0	0/0	0/0	0/0	1/1	0/0	0/0
Chromosome2-2115163 DEL	PRECISE	88	0	0	0/0	0/0	0/0	0/0	0/0	0/0	0/0	0/0	1/1	0/0	0/0	1/1	0/1	0/0
Chromosome2-2115292 DEL	PRECISE	51	0	0	0/0	0/0	0/0	0/0	0/0	0/0	0/0	0/0	0/0	1/1	0/0	0/0	0/0	0/0
Chromosome2-2115395 DEL	PRECISE	4350	0	0	0/0	0/0	0/0	0/0	0/0	0/0	0/0	0/0	0/0	1/1	0/0	0/0	0/0	0/0
Chromosome2-2116263 INS	PRECISE	58	0	0	0/0	0/0	0/0	0/0	0/0	0/0	0/0	0/0	0/0	0/0	0/1	0/0	0/0	0/0
Chromosome2-2116320 DEL	PRECISE	150	0	0	0/0	0/0	0/0	0/0	0/0	0/0	0/0	0/0	0/0	1/1	0/0	1/1	0/1	0/0
Chromosome2-2116929 DEL	PRECISE	5960	0.707	0.707	0/0	0/0	0/0	0/0	0/0	0/0	0/0	0/0	0/0	0/0	0/1	0/0	0/0	0/0
Chromosome2-2117644 BND	PRECISE	0	0	0	0/0	0/0	0/0	0/0	0/0	0/0	0/0	0/0	0/1	0/0	0/0	0/0	0/1	0/0
Chromosome2-2119053 DEL	PRECISE	259	0	0	0/0	0/0	0/0	0/0	1/1	1/1	0/0	0/0	0/0	0/0	0/0	0/0	0/0	0/0
Chromosome2-2120191 BND	PRECISE	0	0	0	0/0	0/0	0/0	0/0	0/0	0/0	0/0	0/0	0/0	0/0	0/1	0/0	0/0	0/0
Chromosome2-2120387 BND	PRECISE	0	0	0	0/0	0/0	0/0	0/0	0/0	0/0	0/0	0/0	0/1	0/0	0/0	0/0	0/1	0/0
Chromosome2-2120644 DEL	PRECISE	161	0	0	0/0	0/0	0/0	0/0	1/1	1/1	0/0	0/0	1/1	1/1	0/1	1/1	0/1	0/0
Chromosome2-2120725 INS	PRECISE	137	0	0	0/0	0/0	0/0	0/0	0/0	0/0	0/0	0/0	0/0	0/0	0/1	0/0	0/0	0/0
Chromosome2-2121792 DEL	PRECISE	318	0	0	0/0	0/0	0/0	0/0	0/0	0/0	0/0	0/0	0/0	1/1	0/0	0/1	0/0	0/0
Chromosome2-2122051 DEL	PRECISE	171	0	0	0/0	0/0	0/0	0/0	0/0	0/0	0/0	0/0	1/1	0/0	0/0	1/1	0/1	0/0
Chromosome2-2122117 DEL	PRECISE	4507	0	0	0/0	0/0	0/0	0/0	0/0	0/0	0/0	0/0	1/1	0/0	0/0	1/1	0/1	0/0
Chromosome2-2123076 INS	PRECISE	1352	0	0	0/0	0/0	0/0	0/0	0/0	0/0	0/0	0/0	0/0	0/0	0/1	0/0	0/0	0/0
Chromosome2-2123527 INS	PRECISE	503	0	0	0/0	0/0	0/0	0/0	0/0	0/0	0/0	0/0	0/0	0/0	0/1	0/0	0/0	0/0
Chromosome2-2124980 DEL	PRECISE	66	0	0	0/0	0/0	0/0	0/0	0/0	0/0	0/0	0/0	1/1	1/1	0/0	1/1	0/1	0/0
Chromosome2-2125034 INS	PRECISE	240	0	0.707	0/0	0/0	0/0	0/0	0/0	0/0	0/0	0/0	0/0	1/1	0/0	0/1	0/0	0/0
Chromosome2-2126706 INS	PRECISE	62	0	0	0/0	0/0	0/0	0/0	0/0	0/0	0/0	0/0	1/1	0/0	0/0	0/1	0/1	0/0
Chromosome2-2129398 DEL	PRECISE	52	0	0	0/0	0/0	0/0	0/0	0/0	0/0	0/0	0/0	1/1	1/1	0/1	1/1	0/1	0/0
Chromosome2-2129736 DEL	PRECISE	363	0	0	0/0	0/0	0/0	0/0	0/0	0/0	0/0	0/0	1/1	1/1	0/1	1/1	0/1	0/0
Chromosome2-2133651 DEL	PRECISE	364	0	0	0/0	0/0	0/0	0/0	0/0	0/0	0/0	0/0	1/1	1/1	0/1	1/1	0/1	0/0
Chromosome2-2133823 INS	PRECISE	53	0	0	0/0	0/0	0/0	0/0	0/0	0/0	0/0	0/0	0/0	1/1	0/1	0/1	0/0	0/0
Chromosome2-2134621 DEL	PRECISE	691	0	0	0/0	0/0	0/0	0/0	0/0	0/0	0/0	0/0	1/1	1/1	0/1	1/1	0/1	0/0
Chromosome2-2135109 DEL	PRECISE	166	0	0	0/0	0/0	0/0	0/0	0/0	0/0	0/0	0/0	1/1	0/0	0/0	0/1	0/1	0/0
Chromosome2-2135438 DEL	PRECISE	773	0	0	0/0	0/0	0/0	0/0	0/0	0/0	0/0	0/0	1/1	1/1	0/1	1/1	0/1	0/0
Chromosome2-2136080 INS	PRECISE	161	0	0	0/0	0/0	0/0	0/0	0/0	0/0	0/0	0/0	0/0	0/0	0/1	0/0	0/0	0/0
Chromosome2-2136358 INS	PRECISE	788	0	0.957	0/0	0/0	0/0	0/0	0/0	0/0	0/0	0/0	1/1	0/0	0/1	0/1	0/1	0/0
Chromosome2-2137420 INS	PRECISE	279	0	0	0/0	0/0	0/0	0/0	0/0	0/0	0/0	0/0	0/0	0/0	0/1	0/0	0/0	0/0
Chromosome2-2138138 INS	PRECISE	946	0	0.707	0/0	0/0	0/0	0/0	0/0	0/0	0/0	0/0	0/0	1/1	0/0	0/1	0/0	0/0
Chromosome2-2140975 DEL	PRECISE	10856	0	0	0/0	0/0	0/0	0/0	0/0	0/0	0/0	0/0	1/1	1/1	0/1	1/1	0/1	0/0
Chromosome2-2142610 DEL	PRECISE	12555	0	0	0/0	0/0	0/0	0/0	0/0	0/0	0/0	0/0	1/1	1/1	0/1	1/1	0/1	0/0
Chromosome2-2143866 BND	PRECISE	0	0	0	0/0	0/0	0/0	0/0	0/0	0/0	0/0	0/0	0/0	0/1	0/0	0/0	0/0	0/0
Chromosome2-2145426 DEL	PRECISE	614	0	0	0/0	0/0	0/0	0/0	0/0	0/0	0/0	0/0	0/0	1/1	0/1	1/1	0/0	0/0
Chromosome2-2146858 DUP	PRECISE	7940	0	0	0/0	0/1	0/0	0/0	0/0	0/0	0/0	0/0	0/0	0/0	0/0	0/0	0/0	0/0
Chromosome2-2147039 INS	PRECISE	1047	0	0	0/0	0/0	0/0	0/0	0/0	0/0	0/0	0/0	0/0	0/1	0/0	0/0	0/0	0/0
Chromosome2-2148868 DEL	PRECISE	1499	0	0	0/0	0/0	1/1	1/1	0/0	0/0	0/0	0/0	0/0	0/0	0/0	0/0	0/0	0/0
Chromosome2-2149562 DEL	PRECISE	5464	0	0	0/0	0/0	1/1	1/1	0/0	0/0	0/0	0/0	0/0	0/0	0/0	0/0	0/0	0/0
Chromosome2-2150446 INS	PRECISE	1345	0	0	0/0	0/0	0/0	0/0	0/0	0/0	0/0	0/0	1/1	0/0	0/0	0/1	0/1	0/0
Chromosome2-2151245 INS	PRECISE	12424	0	0	0/0	0/0	0/0	0/0	0/0	0/0	0/0	0/0	0/0	0/0	0/0	0/1	0/0	0/0
Chromosome2-2154767 DEL	PRECISE	2893	0	0	0/0	0/0	1/1	1/1	0/0	0/0	0/0	0/0	0/0	0/0	0/0	0/0	0/0	0/0
Chromosome2-2155268 DEL	PRECISE	95	0	0	0/0	0/0	1/1	1/1	0/0	0/0	0/0	0/0	0/0	0/0	0/0	0/0	0/0	0/0
Chromosome2-2155745 INS	PRECISE	1359	0	0	0/0	0/0	0/0	0/0	0/0	0/0	0/0	0/0	0/0	0/0	0/0	1/1	0/0	0/0
Chromosome2-2156045 DEL	PRECISE	396	0	0	0/0	0/0	1/1	1/1	0/0	0/0	0/0	0/0	0/0	0/0	0/0	0/0	0/0	0/0
Chromosome2-2156571 DEL	PRECISE	13101	0	0	0/0	0/0	1/1	0/0	0/0	0/0	0/0	0/0	0/0	0/0	0/0	0/0	0/0	0/0
Chromosome2-2156736 DEL	PRECISE	87	0	0	0/0	0/0	0/0	0/0	0/0	0/0	0/0	0/0	0/0	1/1	0/1	0/1	0/0	0/0
Chromosome2-2156797 DEL	PRECISE	11195	0	0	0/0	0/0	0/0	0/0	0/0	0/0	0/0	0/0	0/0	0/0	0/1	0/0	0/0	0/0
Chromosome2-2157541 DEL	PRECISE	426	0	0	0/0	0/0	0/0	0/0	0/0	0/0	0/0	0/0	1/1	1/1	0/0	1/1	0/1	0/0
Chromosome2-2157745 DEL	PRECISE	75	0	0	0/0	0/0	0/0	0/0	0/0	0/0	0/0	0/0	0/0	1/1	0/0	0/1	0/0	0/0
Chromosome2-2158638 DEL	PRECISE	1268	0	0	0/0	0/0	0/0	0/0	0/0	0/0	0/0	0/0	0/0	1/1	0/1	0/1	0/0	0/0
Chromosome2-2158909 BND	PRECISE	0	0	0	0/0	0/0	0/0	0/0	0/0	0/0	0/0	0/0	0/1	0/0	0/0	1/1	0/1	0/0
Chromosome2-2160809 DEL	PRECISE	2608	0	0	0/0	0/0	0/0	0/0	0/0	0/0	0/0	0/0	0/0	1/1	0/1	1/1	0/0	0/0
Chromosome2-2161325 BND	PRECISE	0	0	0	0/0	0/0	0/0	0/0	0/0	0/0	0/0	0/0	0/0	0/0	0/0	0/1	0/0	0/0
Chromosome2-2161626 DEL	PRECISE	4608	0	0	0/0	0/0	0/0	0/0	0/0	0/0	0/0	0/0	1/1	1/1	0/1	1/1	0/1	0/0
Chromosome2-2162935 DEL	PRECISE	715	0	0	0/0	0/0	0/0	0/0	0/0	0/0	0/0	0/0	1/1	0/0	0/0	0/1	0/1	0/0
Chromosome2-2163516 DEL	PRECISE	7121	0	0	0/0	0/0	0/0	0/0	0/0	0/0	0/0	0/0	0/0	1/1	0/0	0/1	0/0	0/0
Chromosome2-2164897 BND	PRECISE	0	0	0	0/0	0/0	0/0	0/0	0/0	0/0	0/0	0/0	0/0	0/1	0/0	0/1	0/0	0/0
Chromosome2-2166151 DEL	PRECISE	11583	0	0	0/0	0/0	0/0	0/0	0/0	0/0	0/0	0/0	0/0	1/1	0/1	0/1	0/0	0/0
Chromosome2-2166567 DEL	PRECISE	418	0	0	0/0	0/0	1/1	0/0	0/0	0/0	0/0	0/0	0/0	0/0	0/0	0/0	0/0	0/0
Chromosome2-2166940 DEL	PRECISE	1295	0	0	0/0	0/0	1/1	1/1	1/1	0/0	0/0	0/0	0/0	0/0	0/0	0/0	0/0	0/0

Chromosome2-2167893 INS	PRECISE	419	0	0.951	0/0	0/0	1/1	1/1	0/0	0/0	0/0	0/0	1/1	1/1	0/1	1/1	0/1	0/0
Chromosome2-2167961 DEL	PRECISE	62	0	0	0/0	0/0	1/1	1/1	0/0	0/0	0/0	0/0	0/0	0/0	0/0	0/0	0/0	0/0
Chromosome2-2168091 DEL	PRECISE	1019707	0	0	0/0	0/0	1/1	0/0	0/0	0/0	0/0	0/0	0/0	0/0	0/0	0/0	0/0	0/0
Chromosome2-2169213 DEL	PRECISE	2280	0	0	0/0	0/0	1/1	1/1	0/0	0/0	0/0	0/0	0/0	0/0	0/0	0/0	0/0	0/0
Chromosome2-2170701 DEL	PRECISE	291	0	0	0/0	0/0	0/0	1/1	0/0	0/0	0/0	0/0	0/0	0/0	0/0	0/0	0/0	0/0
Chromosome2-2172307 DEL	PRECISE	108	0	0	0/0	0/0	0/0	0/0	0/0	0/0	0/0	0/0	0/0	1/1	0/1	0/1	0/0	0/0
Chromosome2-2172322 DEL	PRECISE	1362	0	0	0/0	0/0	0/0	0/0	0/0	0/0	0/0	0/0	1/1	0/0	0/0	0/1	0/1	0/0
Chromosome2-2174205 DEL	PRECISE	1647	0	0	0/0	0/0	1/1	1/1	0/0	0/0	0/0	0/0	0/0	0/0	0/0	0/0	0/0	0/0
Chromosome2-2175914 BND	PRECISE	0	0	0	0/0	0/0	0/0	0/0	0/0	0/0	0/0	0/0	0/0	0/1	0/0	0/1	0/0	0/0
Chromosome2-2176318 INS	PRECISE	1350	0	0.577	0/0	0/0	0/0	0/0	0/0	0/0	0/0	0/0	0/0	1/1	0/1	0/1	0/0	0/0
Chromosome2-2177081 DEL	PRECISE	1617	0	0	0/0	0/0	1/1	1/1	0/0	0/0	0/0	0/0	0/0	0/0	0/0	0/0	0/0	0/0
Chromosome2-2178645 INS	PRECISE	1364	0	0	0/0	0/0	0/0	1/1	0/0	0/0	0/0	0/0	0/0	0/0	0/0	0/0	0/0	0/0
Chromosome2-2179881 INS	PRECISE	5419	0	0	0/0	0/0	0/0	1/1	0/0	0/0	0/0	0/0	0/0	0/0	0/0	0/0	0/0	0/0
Chromosome2-2179881 INS	PRECISE	395	0	0	0/0	0/0	1/1	0/0	0/0	0/0	0/0	0/0	0/0	0/0	0/0	0/0	0/0	0/0
Chromosome2-2180127 DEL	PRECISE	62	0	0	0/0	0/0	1/1	0/0	0/0	0/0	0/0	0/0	0/0	0/0	0/0	0/0	0/0	0/0
Chromosome2-2180193 DEL	PRECISE	475	0	0	0/0	0/0	0/0	0/0	0/0	0/0	0/0	0/0	1/1	0/0	0/0	0/1	0/1	0/0
Chromosome2-2181192 DEL	PRECISE	70	0	0	0/0	0/0	1/1	1/1	0/0	0/0	0/0	0/0	0/0	0/0	0/0	0/0	0/0	0/0
Chromosome2-2181721 BND	PRECISE	0	0	0	0/0	0/0	0/1	0/0	0/0	0/0	0/0	0/0	0/0	0/0	0/0	0/0	0/0	0/0
Chromosome2-2183712 DEL	PRECISE	201	0	0	0/0	0/0	1/1	1/1	0/0	0/0	0/0	0/0	1/1	1/1	0/1	1/1	0/1	0/0
Chromosome2-2183914 BND	PRECISE	0	0	0	0/0	0/0	1/1	0/1	0/0	0/0	0/0	0/0	0/0	0/0	0/0	0/0	0/0	0/0
Chromosome2-2183914 BND	PRECISE	0	0.577	0	0/0	0/0	0/1	0/1	0/0	0/0	0/0	0/0	0/0	0/0	0/0	0/0	0/0	0/0
Chromosome2-2184142 DEL	PRECISE	64	0	0	0/0	0/0	0/0	0/0	0/0	0/0	0/0	0/0	1/1	1/1	0/1	1/1	0/1	0/0
Chromosome2-2184214 INS	PRECISE	882	0	0	0/0	0/0	1/1	1/1	0/0	0/0	0/0	0/0	0/0	0/0	0/0	0/0	0/0	0/0
Chromosome2-2186278 INS	PRECISE	54	0	0	0/0	0/0	0/0	0/0	0/0	0/0	0/0	0/0	1/1	1/1	1/1	1/1	0/1	0/0
Chromosome2-2188901 INS	PRECISE	12109	0	0	0/0	0/0	0/0	0/0	0/0	0/0	0/0	0/0	0/0	0/0	0/0	0/1	0/0	0/0
Chromosome2-2192637 DEL	PRECISE	51	0	0	0/0	0/0	0/0	0/0	0/0	0/0	0/0	0/0	1/1	0/0	0/0	0/1	0/1	0/0
Chromosome2-2192844 INS	PRECISE	7461	0	0	0/0	0/0	0/0	0/0	0/0	0/0	0/0	0/0	0/0	0/0	0/0	0/0	0/0	0/0
Chromosome2-2192908 DEL	PRECISE	119	0	0	0/0	0/0	0/0	0/0	0/0	0/0	0/0	0/0	1/1	0/0	0/0	0/1	0/1	0/0
Chromosome2-2193103 BND	PRECISE	0	0	0	0/0	0/0	0/0	0/0	0/0	0/0	0/0	0/0	0/1	0/0	0/0	0/0	0/1	0/0
Chromosome2-2193168 DEL	PRECISE	236	0	0	0/0	0/0	0/0	0/0	0/0	0/0	0/0	0/0	1/1	0/0	0/1	0/1	0/1	0/0
Chromosome2-2193303 INS	PRECISE	162	0	0	0/0	0/0	0/0	0/0	0/0	0/0	0/0	0/0	0/0	0/0	0/1	0/0	0/0	0/0
Chromosome2-2194081 DEL	PRECISE	150	0	0	0/0	0/0	0/0	0/0	0/0	0/0	0/0	0/0	1/1	1/1	0/0	1/1	0/1	0/0
Chromosome2-2194247 INS	PRECISE	132	0	0	0/0	0/0	0/0	0/0	0/0	0/0	0/0	0/0	0/0	0/0	0/1	0/0	0/0	0/0
Chromosome2-2194249 INS	PRECISE	1201	0	0.707	0/0	0/0	0/0	0/0	0/0	0/0	0/0	0/0	0/0	1/1	0/0	0/1	0/0	0/0
Chromosome2-2196973 INS	PRECISE	236	0	0	0/0	0/0	0/0	0/0	0/0	0/0	0/0	0/0	0/0	0/0	0/1	0/0	0/0	0/0
Chromosome2-2198036 INS	PRECISE	1331	0	0.707	0/0	0/0	0/0	0/0	0/0	0/0	0/0	0/0	0/0	1/1	0/0	0/1	0/0	0/0
Chromosome2-2198053 DEL	PRECISE	6761	0	0	0/0	0/0	0/0	0/0	0/0	0/0	0/0	0/0	1/1	1/1	0/1	1/1	0/1	0/0
Chromosome2-2199041 INS	PRECISE	125	0	0	0/0	0/0	0/0	0/0	0/0	0/0	0/0	0/0	1/1	1/1	0/1	1/1	0/1	0/0
Chromosome2-2199351 INS	PRECISE	180	0	0.5	0/0	0/0	0/0	0/0	0/0	0/0	0/0	0/0	1/1	1/1	0/0	1/1	0/1	0/0
Chromosome2-2199351 INS	PRECISE	9070	0	0	0/0	0/0	0/0	0/0	0/0	0/0	0/0	0/0	0/0	0/0	1/1	0/0	0/0	0/0
Chromosome2-2199732 DEL	PRECISE	149	0	0	0/0	0/0	0/0	0/0	0/0	0/0	0/0	0/0	0/0	0/0	0/1	0/0	0/0	0/0
Chromosome2-2200063 INS	PRECISE	58	0	0	0/0	0/0	0/0	0/0	0/0	0/0	0/0	0/0	0/0	1/1	0/0	0/1	0/0	0/0
Chromosome2-2200794 DEL	PRECISE	172	0	0	0/0	0/0	0/0	0/0	0/0	0/0	0/0	0/0	0/0	0/0	0/1	0/0	0/0	0/0
Chromosome2-2202591 INS	PRECISE	134	0	0	0/0	0/0	0/0	0/0	0/0	0/0	0/0	0/0	1/1	0/0	0/0	0/1	0/1	0/0
Chromosome2-2203851 DEL	PRECISE	936	0	0	0/0	0/0	0/0	0/0	0/0	0/0	0/0	0/0	1/1	0/0	0/0	0/1	0/1	0/0
Chromosome2-2204342 INS	PRECISE	113	0.535	0	0/0	0/0	0/0	1/1	1/1	1/1	0/0	1/1	1/1	0/0	0/1	0/1	0/1	0/0
Chromosome2-2204689 DEL	PRECISE	9740	0	0	0/0	0/0	0/0	0/0	0/0	0/0	0/0	0/0	0/0	1/1	0/1	1/1	0/0	0/0
Chromosome2-2207560 DEL	PRECISE	147	0	0	0/0	0/0	0/0	0/0	0/0	0/0	0/0	0/1	0/0	0/0	0/0	0/0	0/1	0/0
Chromosome2-2207616 DEL	PRECISE	2794	0	0	0/0	0/0	0/0	0/0	0/0	0/0	0/0	0/0	0/0	0/0	0/0	0/0	0/1	0/0
Chromosome2-2208018 DEL	PRECISE	495	0	0	0/0	0/0	0/0	0/0	1/1	0/0	1/1	0/1	0/0	0/1	0/0	0/1	0/0	0/0
Chromosome2-2208113 DEL	PRECISE	1705	0	0	0/0	0/0	0/0	0/0	1/1	0/0	1/1	0/1	0/0	1/1	0/0	0/1	0/0	0/0
Chromosome2-2208294 DEL	PRECISE	2113	0.894	0.894	0/0	0/0	0/0	0/0	1/1	0/0	1/1	0/1	0/0	1/1	0/0	0/0	0/0	0/0
Chromosome2-2208390 BND	PRECISE	0	0	0	0/0	0/0	0/0	0/0	0/1	0/0	0/1	0/0	0/0	0/1	0/0	0/0	0/0	0/0
Chromosome2-2208393 DEL	PRECISE	294	0	0	0/0	0/0	0/0	0/0	0/0	0/0	0/0	0/1	0/1	0/0	0/0	0/0	0/0	0/0
Chromosome2-2209125 INS	PRECISE	3723	0	0	0/0	0/0	0/0	0/0	0/0	0/0	0/0	0/0	0/0	0/0	0/0	1/1	0/0	0/0
Chromosome2-2210938 DEL	PRECISE	168	0	0	0/0	0/0	0/0	0/0	0/0	0/0	0/0	0/0	0/0	0/0	0/1	0/0	0/0	0/0
Chromosome2-2211198 DEL	PRECISE	1311	0	0	0/0	0/0	0/0	0/0	0/0	0/0	0/0	0/0	1/1	0/0	0/0	0/1	0/1	0/0
Chromosome2-2211294 INS	PRECISE	1625	0	0	0/0	0/0	0/0	0/0	0/0	0/0	0/0	0/0	0/0	0/0	0/1	0/0	0/0	0/0
Chromosome2-2211701 INS	PRECISE	70	0	0.5	0/0	0/0	0/0	0/0	0/0	0/0	0/0	0/0	1/1	0/0	0/1	0/1	0/1	0/0
Chromosome2-2211901 DEL	PRECISE	1346	0	0	0/0	0/0	0/0	0/0	0/0	0/0	0/0	0/0	0/0	0/0	0/1	0/0	0/0	0/0
Chromosome2-2213525 INS	PRECISE	54	0	0	0/0	0/0	0/0	0/0	0/0	0/0	0/0	0/0	0/0	0/0	0/1	0/0	0/0	0/0
Chromosome2-2215790 INS	PRECISE	149	0	0	0/0	0/0	0/0	0/0	0/0	0/0	0/0	0/0	0/0	1/1	0/0	0/1	0/0	0/0
Chromosome2-2217306 INS	PRECISE	633	0	0	0/0	0/0	0/0	0/0	0/0	0/0	0/0	0/0	0/0	0/0	0/1	0/0	0/0	0/0
Chromosome2-2218127 DEL	PRECISE	66	0	0	0/0	0/0	0/0	0/0	0/0	0/0	0/0	0/0	0/0	0/0	0/1	0/0	0/0	0/0
Chromosome2-2219585 DEL	PRECISE	439	0	0	0/0	0/0	0/0	0/0	0/0	0/0	0/0	0/0	1/1	0/0	0/0	1/1	0/1	0/0
Chromosome2-2221269 DEL	PRECISE	69	0	0	0/0	0/0	0/0	0/0	0/0	0/0	0/0	0/0	1/1	0/0	0/0	0/1	0/1	0/0
Chromosome2-2223148 BND	PRECISE	0	0	0	0/0	0/0	0/0	0/0	0/0	0/0	0/0	0/0	0/0	0/0	0/0	0/1	0/0	0/0
Chromosome2-2223994 DEL	PRECISE	236	0	0	0/0	0/0	0/0	0/0	0/0	0/0	0/0	0/0	0/0	1/1	0/1	0/1	0/0	0/0
Chromosome2-2224963 DEL	PRECISE	860	0.577	0.577	0/0	0/0	0/0	0/0	0/0	0/0	0/0	0/0	0/0	1/1	0/1	0/1	0/0	0/0
Chromosome2-2225249 INS	PRECISE	707	0	0	0/0	0/0	0/0	0/0	0/0	0/0	0/0	0/0	0/0	0/0	0/1	0/0	0/0	0/0
Chromosome2-2225770 INS	PRECISE	3590	0	0	0/0	0/0	0/0	0/0	0/0	0/0	0/0	0/0	0/0	0/0	1/1	0/0	0/0	0/0
Chromosome2-2227692 DEL	PRECISE	58	0	0	0/0	0/0	0/0	0/0	0/0	0/0	0/0	0/0	0/0	1/1	0/0	0/1	0/0	0/0
Chromosome2-2228202 DEL	PRECISE	2028	0	0	0/0	0/0	0/0	0/0	0/0	0/0	0/0	0/0	0/0	1/1	0/0	0/1	0/0	0/0

Chromosome2-2228263	INS	PRECISE	1481	0	0	0/0	0/0	0/0	0/0	0/0	0/0	0/0	0/0	0/0	0/0	0/1	0/0	0/0	0/0
Chromosome2-2229478	DEL	PRECISE	6297	0.577	0	0/0	0/0	0/0	0/0	0/0	0/0	0/0	0/0	1/1	0/0	0/0	0/1	1/1	0/0
Chromosome2-2229623	INS	PRECISE	111	0	0	0/0	0/0	0/0	0/0	0/0	0/0	0/0	0/0	0/0	0/0	0/1	0/0	0/0	0/0
Chromosome2-2229720	DEL	PRECISE	50	0	0	0/0	0/0	0/0	0/0	0/0	0/0	0/0	0/0	0/0	1/1	0/0	0/1	0/0	0/0
Chromosome2-2230227	BND	PRECISE	0	0	0	0/0	0/0	0/0	0/0	0/0	0/0	0/0	0/0	0/0	0/0	0/1	0/0	0/0	0/0
Chromosome2-2230769	DEL	PRECISE	55	0	0	0/0	0/0	0/0	0/0	0/0	0/0	0/0	0/0	0/0	0/0	0/1	0/0	0/0	0/0
Chromosome2-2230833	INS	PRECISE	54	0	0	0/0	0/0	0/0	0/0	0/0	0/0	0/0	0/0	1/1	1/1	0/1	1/1	0/1	0/0
Chromosome2-2231171	INS	PRECISE	2611	0	0	0/0	0/0	0/0	0/0	0/0	0/0	0/0	0/0	0/0	0/0	1/1	0/0	0/0	0/0
Chromosome2-2232296	INS	PRECISE	852	0	0	0/0	0/0	0/0	0/0	0/0	0/0	0/0	0/0	0/0	0/0	0/1	0/0	0/0	0/0
Chromosome2-2232776	DEL	PRECISE	1871	0	0	0/0	0/0	0/0	0/0	0/0	0/0	0/0	0/0	0/0	0/0	0/1	0/0	0/0	0/0
Chromosome2-2233217	INS	PRECISE	12418	0	0	0/0	0/0	0/0	0/0	0/0	0/0	0/0	0/0	0/0	0/0	0/1	0/0	0/0	0/0
Chromosome2-2233218	BND	PRECISE	0	0	0	0/0	0/0	0/0	0/0	0/0	0/0	0/0	0/0	0/0	0/0	0/1	0/0	0/0	0/0
Chromosome2-2235178	INS	PRECISE	104	0	0	0/0	0/0	0/0	0/0	0/0	0/0	0/0	0/0	0/0	1/1	0/0	0/1	0/0	0/0
Chromosome2-2236182	BND	PRECISE	0	0	0	0/0	0/0	0/0	0/0	0/0	0/0	0/0	0/0	0/0	0/0	0/1	0/0	0/0	0/0
Chromosome2-2236187	INS	PRECISE	119	0	0.707	0/0	0/0	0/0	0/0	0/0	0/0	0/0	0/0	0/0	1/1	0/0	0/1	0/0	0/0
Chromosome2-2236216	DEL	PRECISE	50	0	0	0/0	0/0	0/0	0/0	0/0	0/0	0/0	0/0	1/1	0/0	0/0	0/1	0/1	0/0
Chromosome2-2236721	DEL	PRECISE	3758	0	0	0/0	0/0	0/0	0/0	0/0	0/0	0/0	0/0	0/0	0/0	0/1	0/0	0/0	0/0
Chromosome2-2237703	DEL	PRECISE	2611	0	0	0/0	0/0	0/0	0/0	0/0	0/0	0/0	0/0	0/0	0/0	0/1	0/0	0/0	0/0
Chromosome2-2237942	DEL	PRECISE	108	0	0	0/0	0/0	0/0	0/0	0/0	0/0	0/0	0/0	1/1	1/1	0/0	1/1	0/0	0/0
Chromosome2-2238412	INS	PRECISE	137	0	0	0/0	0/0	0/0	0/0	0/0	0/0	0/0	0/0	0/0	0/0	0/1	0/0	0/0	0/0
Chromosome2-2239715	INS	PRECISE	622	0	0	0/0	0/0	0/0	0/0	0/0	0/0	0/0	0/0	0/0	1/1	0/0	0/1	0/0	0/0
Chromosome2-2240003	BND	PRECISE	0	0	0	0/0	0/0	0/0	0/0	0/0	0/0	0/0	0/0	0/0	0/0	0/0	0/1	0/0	0/0
Chromosome2-2240376	DEL	PRECISE	54	0	0	0/0	0/1	0/0	0/0	0/0	0/0	0/0	0/0	0/0	0/0	0/0	0/0	0/0	0/0
Chromosome2-2240484	DUP	PRECISE	694131	0	0	0/0	0/0	0/0	0/0	0/0	0/0	0/0	0/0	0/0	0/0	0/0	0/1	0/0	0/0
Chromosome2-2241460	INS	PRECISE	72	0	0	0/0	0/0	0/0	0/0	0/0	0/0	0/0	0/0	1/1	0/0	0/0	1/1	0/1	0/0
Chromosome2-2245505	DEL	PRECISE	68	0	0	0/0	0/0	0/0	0/0	0/0	0/0	0/0	0/0	0/0	1/1	0/0	0/1	0/0	0/0
Chromosome2-2246080	INS	PRECISE	162	0	0	0/0	0/0	0/0	0/0	0/0	0/0	0/0	0/0	1/1	0/0	0/0	0/1	0/0	0/0
Chromosome2-2247693	INS	PRECISE	364	0	0	0/0	0/0	0/0	0/0	0/0	0/0	0/0	0/0	0/0	1/1	0/0	0/0	0/0	0/0
Chromosome2-2247767	INS	PRECISE	757	0	0	0/0	0/0	0/0	0/0	0/0	0/0	0/0	0/0	0/0	0/0	0/1	0/0	0/0	0/0
Chromosome2-2248007	INS	PRECISE	127	0	0	0/0	0/0	0/0	0/0	0/0	0/0	0/0	0/0	1/1	1/1	0/1	1/1	0/1	0/0
Chromosome2-2248068	DEL	PRECISE	306	0	0	0/0	0/0	0/0	0/0	0/0	0/0	0/0	0/0	0/0	1/1	0/1	0/0	0/0	0/0
Chromosome2-2248447	INS	PRECISE	69	0	0	0/0	0/0	0/0	0/0	0/0	0/0	0/0	0/0	1/1	0/0	0/1	1/1	1/1	0/0
Chromosome2-2249893	INS	PRECISE	56	0	0	0/0	0/0	0/0	0/0	0/0	0/0	0/0	0/0	0/0	0/0	0/1	0/0	0/0	0/0
Chromosome2-2250121	DEL	PRECISE	1460	0	0	0/0	0/0	0/0	0/0	0/0	0/0	0/0	0/0	0/0	1/1	0/1	0/1	0/0	0/0
Chromosome2-2250815	DEL	PRECISE	849	0	0	0/0	0/0	0/0	0/0	0/0	0/0	0/0	0/0	1/1	1/1	0/1	1/1	0/1	0/0
Chromosome2-2252212	DEL	PRECISE	85	0	0	0/0	0/0	0/0	0/0	0/0	0/0	0/0	0/0	0/0	0/0	0/1	0/0	0/0	0/0
Chromosome2-2252233	BND	PRECISE	0	0	0	0/0	0/0	0/0	0/0	0/0	0/0	0/0	0/0	0/0	0/1	0/0	0/0	0/0	0/0
Chromosome2-2252335	INS	PRECISE	59	0	0	0/0	0/0	0/0	0/0	0/0	0/0	0/0	0/0	0/0	0/0	0/1	0/0	0/0	0/0
Chromosome2-2253513	INS	PRECISE	1350	0	0.707	0/0	0/0	0/0	0/0	0/0	0/0	0/0	0/0	0/0	1/1	0/0	0/1	0/0	0/0
Chromosome2-2255411	INS	PRECISE	8874	0	0	0/0	0/0	0/0	0/0	0/0	0/0	0/0	0/0	0/0	0/0	0/0	0/0	0/0	0/1
Chromosome2-2257710	DEL	PRECISE	106	0	0	0/0	0/0	0/0	0/0	1/1	0/0	0/0	0/0	0/0	0/0	0/0	0/0	0/0	0/0
Chromosome2-2258115	INS	PRECISE	152	0	0	0/0	0/0	0/0	0/0	0/0	0/0	0/0	0/0	1/1	0/0	0/1	1/1	0/1	0/0
Chromosome2-2258351	DEL	PRECISE	29904	0	0	0/0	0/0	0/0	0/0	0/0	0/0	0/0	0/0	0/0	0/0	0/0	0/1	0/0	0/0
Chromosome2-2260091	DEL	PRECISE	206	0	0	0/0	0/0	0/0	0/0	0/0	0/0	0/0	0/0	0/0	0/0	0/1	0/0	0/0	0/0
Chromosome2-2260240	INS	PRECISE	595	0	0	0/0	0/0	0/0	0/0	0/0	0/0	0/0	0/0	0/0	0/0	0/1	0/0	0/0	0/0
Chromosome2-2260559	INS	PRECISE	59	0	0.577	0/0	0/0	0/0	0/0	0/0	0/0	0/0	0/0	1/1	0/0	0/0	1/1	0/1	0/0
Chromosome2-2263065	INS	PRECISE	2381	0	0	0/0	0/0	0/0	0/0	0/0	0/0	0/0	0/0	0/0	0/0	0/1	0/0	0/0	0/0
Chromosome2-2263551	INS	PRECISE	58	0	0	0/0	0/0	0/0	0/0	0/0	0/0	0/0	0/0	1/1	1/1	0/1	1/1	0/1	0/0
Chromosome2-2264451	INS	PRECISE	50	0	0	0/0	0/0	0/0	0/0	0/0	0/0	0/0	0/0	0/0	1/1	0/0	0/1	0/0	0/0
Chromosome2-2265229	INS	PRECISE	426	0	0	0/0	0/0	0/0	0/0	0/0	0/0	0/0	0/0	0/0	1/1	0/0	0/1	0/0	0/0
Chromosome2-2265692	DEL	PRECISE	232	0	0	0/0	0/0	0/0	0/0	0/0	0/0	0/0	0/0	0/0	1/1	0/0	0/1	0/0	0/0
Chromosome2-2266574	DEL	PRECISE	144	0	0	0/0	0/0	0/0	1/1	1/1	0/0	0/0	0/0	0/0	0/0	0/0	0/0	0/0	0/0
Chromosome2-2267065	INS	PRECISE	72	0	0	0/0	0/0	0/0	0/0	0/0	0/0	0/0	0/0	0/0	1/1	0/0	0/1	0/0	0/0
Chromosome2-2268713	BND	PRECISE	0	0	0	0/0	0/0	0/0	0/0	0/0	0/0	0/0	0/0	0/0	0/1	0/0	0/1	0/0	0/0
Chromosome2-2269407	DEL	PRECISE	51	0	0	0/0	0/0	0/0	0/0	0/0	0/0	0/0	0/0	0/0	1/1	0/0	0/1	0/0	0/0
Chromosome2-2269862	DEL	PRECISE	60	0	0	0/0	0/0	0/0	0/0	0/0	0/0	0/0	0/0	0/0	1/1	0/0	0/1	0/0	0/0
Chromosome2-2270061	BND	PRECISE	0	0	0	0/0	0/0	0/0	0/0	0/0	0/0	0/0	0/1	0/0	0/0	0/0	0/0	0/0	0/1
Chromosome2-2270174	DEL	PRECISE	54	0	0	0/0	0/0	0/0	0/0	0/0	0/0	0/0	0/0	0/0	0/0	0/0	0/0	0/1	0/0
Chromosome2-2270424	DEL	PRECISE	349	0	0	0/0	0/0	0/0	0/0	0/0	0/0	0/0	0/0	0/0	0/1	0/0	0/1	0/0	0/0
Chromosome2-2270526	DEL	PRECISE	103	0	0	0/0	0/0	0/1	0/1	0/0	0/0	0/0	0/0	0/0	0/0	0/0	0/0	0/0	0/0
Chromosome2-2271282	BND	PRECISE	0	0	0	0/0	0/0	0/0	0/0	0/0	0/0	0/0	0/0	0/1	0/0	0/1	0/1	0/0	0/0
Chromosome2-2271282	BND	PRECISE	0	0	0	0/0	0/0	0/0	0/0	0/0	0/0	0/0	0/0	0/0	0/0	0/0	0/0	0/0	0/1
Chromosome2-2272901	INS	PRECISE	1368	0	0.577	0/0	0/0	1/1	0/0	1/1	1/1	0/0	0/0	0/0	0/0	0/0	0/0	0/0	0/0
Chromosome2-2272917	INS	PRECISE	161	0	0	0/0	0/0	0/0	0/0	1/1	1/1	0/0	0/0	0/0	0/0	0/0	0/0	0/0	0/0
Chromosome2-2273383	INS	PRECISE	1003	0	0.957	0/0	0/0	0/0	0/0	0/0	0/0	0/0	0/0	1/1	0/0	0/1	0/1	0/1	0/0
Chromosome2-2274081	BND	PRECISE	0	0	0	0/0	0/0	0/1	0/0	0/0	0/0	0/0	0/0	0/0	0/0	0/0	0/0	0/0	0/0
Chromosome2-2274370	DEL	PRECISE	877	0	0	0/0	0/0	1/1	0/0	1/1	1/1	0/0	0/0	0/0	0/0	0/0	0/0	0/0	0/0
Chromosome2-2275008	INS	PRECISE	297	0	0.577	0/0	0/0	1/1	0/0	1/1	1/1	0/0	0/0	0/0	0/0	0/0	0/0	0/0	0/0
Chromosome2-2275529	DEL	PRECISE	110	0	0	0/0	0/0	0/0	0/0	0/0	0/0	0/0	0/0	0/0	1/1	0/0	0/1	0/0	0/0
Chromosome2-2278011	DEL	PRECISE	12418	0	0	0/0	0/0	0/0	0/0	0/0	0/0	0/0	0/0	1/1	1/1	1/1	1/1	0/1	0/0
Chromosome2-2279625	BND	PRECISE	0	0	0	0/0	0/0	0/0	0/0	0/0	0/0	0/0	0/0	0/0	0/0	0/1	0/0	0/0	0/0
Chromosome2-2281394	DEL	PRECISE	105	0	0	0/0	0/0	1/1	0/0	1/1	1/1	0/0	0/0	0/0	0/0	0/0	0/0	0/0	0/0
Chromosome2-2282097	DEL	PRECISE	8297	0	0	0/0	0/0	0/0	0/0	0/0	0/0	0/0	0/0	0/0	1/1	0/0	0/1	0/0	0/0

Chromosome2-2282519 DEL	PRECISE	7399	0	0	0/0	0/0	1/1	0/0	1/1	1/1	0/0	0/0	0/0	0/0	0/0	0/0	0/0	0/0
Chromosome2-2283728 INS	PRECISE	695	0	0.577	0/0	0/0	1/1	0/0	1/1	1/1	0/0	0/0	0/0	0/0	0/0	0/0	0/0	0/0
Chromosome2-2284259 INS	PRECISE	116	0	0.707	0/0	0/0	0/0	0/0	0/0	0/0	0/0	0/0	0/0	1/1	0/0	0/1	0/0	0/0
Chromosome2-2285322 DEL	PRECISE	54	0	0	0/0	0/0	0/0	0/0	0/0	0/0	0/0	0/0	0/0	1/1	0/0	0/1	0/0	0/0
Chromosome2-2286166 DEL	PRECISE	1598	0	0	0/0	0/0	0/0	0/0	0/0	0/0	0/0	0/0	0/0	1/1	0/0	0/1	0/0	0/0
Chromosome2-2287758 INS	PRECISE	84	0	0	0/0	0/0	0/0	0/0	0/0	0/0	0/0	0/0	0/0	0/0	0/1	0/0	0/0	0/0
Chromosome2-2287861 INS	PRECISE	12522	0	0	0/0	0/0	0/0	0/0	0/0	0/0	0/0	0/0	0/0	1/1	0/0	0/0	0/0	0/0
Chromosome2-2288465 DEL	PRECISE	686	0	0	0/0	0/0	0/0	0/0	0/0	0/0	0/0	0/0	0/0	1/1	0/0	0/1	0/0	0/0
Chromosome2-2288479 DEL	PRECISE	8087	0	0	0/0	0/0	0/0	0/0	0/0	0/0	0/0	0/0	0/0	0/0	0/1	0/0	0/0	0/0
Chromosome2-2288801 BND	PRECISE	0	0	0	0/0	0/0	0/1	0/0	0/1	0/0	0/0	0/0	0/0	0/0	0/0	0/0	0/0	0/0
Chromosome2-2288801 BND	PRECISE	0	0	0	0/0	0/0	0/1	0/0	0/0	0/0	0/0	0/0	0/0	0/0	0/0	0/0	0/0	0/0
Chromosome2-2289124 INS	PRECISE	55	0	0.577	0/0	0/0	1/1	0/0	1/1	1/1	0/0	0/0	0/0	0/0	0/0	0/0	0/0	0/0
Chromosome2-2289793 INS	PRECISE	67	0	0	0/0	0/0	1/1	0/0	1/1	1/1	0/0	0/0	0/0	0/0	0/0	0/0	0/0	0/0
Chromosome2-2289922 DEL	PRECISE	67	0	0	0/0	0/0	1/1	0/0	1/1	1/1	0/0	0/0	0/0	0/0	0/0	0/0	0/0	0/0
Chromosome2-2290311 BND	PRECISE	0	0	0	0/0	0/0	1/1	0/0	0/1	1/1	0/0	0/0	0/0	0/0	0/0	0/0	0/0	0/0
Chromosome2-2291382 INS	PRECISE	66	0	0	0/0	0/0	0/0	0/0	0/0	0/0	0/0	0/0	0/0	1/1	0/0	0/1	0/0	0/0
Chromosome2-2291569 INS	PRECISE	71	0	0	0/0	0/0	1/1	0/0	1/1	1/1	0/0	0/0	0/0	1/1	0/0	0/1	0/0	0/0
Chromosome2-2293394 DEL	PRECISE	368	0	0	0/0	0/0	1/1	0/0	0/0	0/0	0/0	0/0	0/0	0/0	0/0	0/0	0/0	0/0
Chromosome2-2293713 INS	PRECISE	1027	0	0	0/0	0/0	0/0	0/0	0/0	0/0	0/0	0/0	0/0	1/1	0/0	0/1	0/0	0/0
Chromosome2-2293777 DEL	PRECISE	4800	0.707	0.707	0/0	0/0	0/0	0/0	0/0	0/0	0/0	0/0	0/0	1/1	0/0	0/1	0/0	0/0
Chromosome2-2295237 DEL	PRECISE	38532	0	0	0/0	0/0	0/0	0/0	0/0	0/0	0/0	0/0	0/0	0/0	1/1	1/1	1/1	0/0
Chromosome2-2298004 DEL	PRECISE	10637	0	0	0/0	0/0	0/0	0/0	0/0	0/0	0/0	0/0	0/0	0/1	0/0	1/1	0/0	0/0
Chromosome2-2299768 DEL	PRECISE	430	0	0	0/0	0/0	0/0	0/0	0/0	0/0	0/0	0/0	0/0	1/1	0/0	0/1	0/0	0/0
Chromosome2-2300412 DEL	PRECISE	52	0	0	0/0	0/0	0/0	0/0	0/0	0/0	0/0	0/0	0/0	1/1	0/0	0/1	0/0	0/0
Chromosome2-2302160 DEL	PRECISE	88	0	0	0/0	0/0	0/0	0/0	0/0	0/0	0/0	0/0	0/0	1/1	0/0	0/1	0/0	0/0
Chromosome2-2302558 DEL	PRECISE	75	0	0	0/0	0/0	0/0	0/0	0/0	0/0	0/0	0/0	1/1	0/0	0/0	0/1	0/1	0/0
Chromosome2-2305167 DEL	PRECISE	406	0	0	0/0	0/0	0/0	0/0	0/0	0/0	0/0	0/0	1/1	0/0	0/0	0/1	0/1	0/0
Chromosome2-2305372 DEL	PRECISE	774	0	0	0/0	0/0	0/0	0/0	0/0	0/0	0/0	0/0	0/0	1/1	0/0	0/1	0/0	0/0
Chromosome2-2306514 DEL	PRECISE	7120	0	0	0/0	0/0	0/0	0/0	0/0	0/0	0/0	0/0	0/0	1/1	0/0	0/1	0/0	0/0
Chromosome2-2307303 INS	PRECISE	66	0	0	0/0	0/0	0/0	0/0	0/0	0/0	0/0	0/0	0/0	1/1	0/0	0/1	0/0	0/0
Chromosome2-2307572 INS	PRECISE	84	0	0	0/0	0/0	0/0	0/0	0/0	0/0	0/0	0/0	1/1	0/0	0/0	0/1	0/1	0/0
Chromosome2-2309072 DEL	PRECISE	2066	0	0	0/0	0/0	0/0	0/0	0/0	0/0	0/0	0/0	0/0	1/1	0/0	0/1	0/0	0/0
Chromosome2-2311133 DEL	PRECISE	949	0	0	0/0	0/0	0/0	0/0	0/0	0/0	0/0	0/0	0/0	0/0	0/0	0/1	0/1	0/0
Chromosome2-2311358 DEL	PRECISE	78	0	0	0/0	0/0	0/0	0/0	0/0	0/0	0/0	0/0	0/0	0/0	0/0	0/1	0/1	0/0
Chromosome2-2311634 DEL	PRECISE	1364	0	0	0/0	0/0	0/0	0/0	0/0	0/0	0/0	0/0	0/0	1/1	0/0	0/1	0/0	0/0
Chromosome2-2315307 BND	PRECISE	0	0	0	0/0	0/0	0/0	0/0	0/0	0/0	0/0	0/0	0/0	0/0	0/0	0/1	0/0	0/0
Chromosome2-2315614 DEL	PRECISE	4114	0.707	0.707	0/0	0/0	0/0	0/0	0/0	0/0	0/0	0/0	0/0	1/1	0/0	0/1	0/0	0/0
Chromosome2-2318886 INS	PRECISE	313	0	0	0/0	0/0	0/0	0/0	0/0	0/0	0/0	0/0	0/0	1/1	0/0	0/1	0/0	0/0
Chromosome2-2320424 INS	PRECISE	132	0	0	0/0	0/0	0/0	0/0	0/0	0/0	0/0	0/0	0/0	1/1	0/0	0/1	0/0	0/0
Chromosome2-2320880 DEL	PRECISE	2444	0	0	0/0	0/0	0/0	0/0	0/0	0/0	0/0	0/0	0/0	1/1	0/0	0/1	0/0	0/0
Chromosome2-2322558 INS	PRECISE	148	0	0	0/0	0/0	0/0	0/0	0/0	0/0	0/0	0/0	0/0	1/1	0/0	0/1	0/0	0/0
Chromosome2-2322813 DEL	PRECISE	745	0	0	0/0	0/0	0/0	0/0	0/0	0/0	0/0	0/0	0/0	1/1	0/0	0/1	0/0	0/0
Chromosome2-2323198 INS	PRECISE	1549	0	0.707	0/0	0/0	0/0	0/0	0/0	0/0	0/0	0/0	0/0	1/1	0/0	0/1	0/0	0/0
Chromosome2-2326512 DEL	PRECISE	12579	0	0	0/0	0/0	0/0	0/0	0/0	0/0	0/0	0/0	1/1	0/0	0/1	1/1	0/1	0/0
Chromosome2-2329339 DEL	PRECISE	159	0	0	0/0	0/0	0/0	0/0	0/0	0/0	0/0	0/0	0/0	0/0	0/1	0/0	0/0	0/0
Chromosome2-2331583 INS	PRECISE	121	0	0	0/0	0/0	0/0	0/0	0/0	0/0	0/0	0/0	1/1	0/0	0/1	0/1	0/1	0/0
Chromosome2-2332861 INS	PRECISE	1332	0	0.707	0/0	0/0	0/0	0/0	0/0	0/0	0/0	0/0	0/0	1/1	0/0	0/1	0/0	0/0
Chromosome2-2333925 DEL	PRECISE	72	0	0	0/0	0/0	0/0	0/0	0/0	0/0	0/0	0/0	0/0	1/1	0/0	0/1	0/0	0/0
Chromosome2-2335498 DEL	PRECISE	1059	0	0	0/0	0/0	0/0	0/0	0/0	0/0	0/0	0/0	0/0	1/1	0/0	0/1	0/0	0/0
Chromosome2-2335988 DEL	PRECISE	1262	0	0	0/0	0/0	0/0	0/0	0/0	0/0	0/0	0/0	0/0	1/1	0/0	0/1	0/0	0/0
Chromosome2-2336025 INV	PRECISE	272	0	0	0/0	0/0	0/0	0/0	0/0	0/0	0/0	0/0	1/1	0/0	0/1	0/1	0/1	0/0
Chromosome2-2336282 DEL	PRECISE	3982	0	0	0/0	0/0	0/0	0/0	0/0	0/0	0/0	0/0	0/0	0/0	0/0	0/0	0/1	0/0
Chromosome2-2336935 INS	PRECISE	116	0	0	0/0	0/0	0/0	0/0	0/0	0/0	0/0	0/0	0/0	0/0	0/0	0/0	0/1	0/0
Chromosome2-2337609 DEL	PRECISE	4691	0	0	0/0	0/0	0/0	0/0	0/0	0/0	0/0	0/0	0/0	1/1	0/0	0/1	0/0	0/0
Chromosome2-2337672 INS	PRECISE	62	0	0	0/0	0/0	0/0	0/0	0/0	0/0	0/0	0/0	0/0	0/0	0/0	0/0	0/0	0/1
Chromosome2-2338157 DEL	PRECISE	104	0	0	0/0	0/0	0/0	0/0	0/0	0/0	0/0	0/0	0/0	1/1	0/0	0/1	0/0	0/0
Chromosome2-2338176 INS	PRECISE	115	0	0	0/0	0/0	0/0	0/0	0/0	0/0	0/0	0/0	0/0	1/1	0/0	0/1	0/0	0/0
Chromosome2-2339109 INS	PRECISE	75	0	0.707	0/0	0/0	0/0	0/0	0/0	0/0	0/0	0/0	0/0	1/1	0/0	0/1	0/0	0/0
Chromosome2-2339493 INS	PRECISE	406	0	0.707	0/0	0/0	0/0	0/0	0/0	0/0	0/0	0/0	0/0	1/1	0/0	0/1	0/0	0/0
Chromosome2-2339797 INS	PRECISE	61	0	0	0/0	0/0	0/0	0/0	0/0	0/0	0/0	0/0	0/0	1/1	0/0	0/1	0/0	0/0
Chromosome2-2342134 DEL	PRECISE	61	0	0	0/0	0/0	0/0	0/0	0/0	0/0	0/0	0/0	1/1	1/1	0/1	1/1	0/1	0/0
Chromosome2-2344343 INS	PRECISE	122	0	0	0/0	0/0	0/0	0/0	0/0	0/0	0/0	0/0	0/0	1/1	0/0	0/1	0/0	0/0
Chromosome2-2344531 INS	PRECISE	1346	0	0.707	0/0	0/0	0/0	0/0	0/0	0/0	0/0	0/0	0/0	1/1	0/0	0/1	0/0	0/0
Chromosome2-2346379 INS	PRECISE	8051	0	0	0/0	0/0	0/0	0/0	0/0	0/0	0/0	0/0	0/0	1/1	0/0	1/1	0/0	0/0
Chromosome2-2346443 INS	PRECISE	1364	0	0.707	0/0	0/0	0/0	0/0	0/0	0/0	0/0	0/0	0/0	1/1	0/0	0/1	0/0	0/0
Chromosome2-2346968 INS	PRECISE	1371	0	0.707	0/0	0/0	0/0	0/0	0/0	0/0	0/0	0/0	0/0	1/1	0/0	0/1	0/0	0/0
Chromosome2-2347680 INS	PRECISE	58	0	0.408	0/0	0/0	0/0	0/0	1/1	0/0	0/0	0/0	1/1	1/1	0/1	1/1	0/1	0/0
Chromosome2-2348075 DEL	PRECISE	275	0	0	0/0	0/0	0/0	0/0	1/1	0/0	0/0	0/0	1/1	1/1	0/0	1/1	0/1	0/0
Chromosome2-2348820 DEL	PRECISE	1114	0	0	0/0	0/0	0/0	0/0	0/0	0/0	0/0	0/0	0/0	1/1	0/0	0/1	0/0	0/0
Chromosome2-2349542 DEL	PRECISE	6736	0	0	0/0	0/0	0/0	0/0	0/0	0/0	0/0	0/0	1/1	0/0	0/0	0/0	0/0	0/0
Chromosome2-2349776 INS	PRECISE	159	0.5	0.5	0/0	0/0	0/0	0/0	0/0	0/0	0/0	0/0	1/1	1/1	0/0	1/1	0/0	0/0
Chromosome2-2352230 DEL	PRECISE	106	0	0	0/0	0/0	0/0	0/0	0/0	0/0	0/0	0/0	1/1	0/0	0/0	0/1	0/1	0/0
Chromosome2-2353311 INS	PRECISE	1330	0	0.577	0/0	0/0	0/0	0/0	0/0	0/0	0/0	0/0	1/1	0/0	0/0	0/1	0/1	0/0

Chromosome2-2353351 DEL	PRECISE	1335	0	0	0/0	0/0	0/0	0/0	0/0	0/0	0/0	0/0	0/0	1/1	0/0	0/1	0/0	0/0
Chromosome2-2353584 INS	PRECISE	63	0	0.707	0/0	0/0	0/0	0/0	0/0	0/0	0/0	0/0	0/0	1/1	0/0	0/1	0/0	0/0
Chromosome2-2354646 DEL	PRECISE	50	0	0	0/0	0/0	0/0	0/0	0/0	0/0	0/0	0/0	1/1	1/1	0/1	1/1	0/1	0/0
Chromosome2-2354988 DEL	PRECISE	368	0	0	0/0	0/0	0/0	0/0	0/0	0/0	0/0	0/0	0/1	0/0	0/0	0/1	0/1	0/0
Chromosome2-2355169 DEL	PRECISE	2084	0	0	0/0	0/1	0/0	0/0	1/1	0/0	1/1	1/1	0/1	0/0	0/0	0/0	0/0	0/1
Chromosome2-2356842 INS	PRECISE	1051	0	0.577	0/0	0/0	0/0	0/0	0/0	0/0	0/0	0/0	1/1	0/0	0/0	1/1	0/1	0/0
Chromosome2-2357024 INS	PRECISE	1010	0	0	0/0	0/0	0/0	0/0	0/0	0/0	0/0	0/0	0/0	0/0	0/1	0/0	0/0	0/0
Chromosome2-2357839 INS	PRECISE	13210	0	0	0/0	0/0	0/0	0/0	0/0	0/0	0/0	0/0	0/0	0/0	0/1	0/0	0/0	0/0
Chromosome2-2357840 BND	PRECISE	0	0	0	0/0	0/0	0/0	0/0	0/0	0/0	0/0	0/0	0/0	0/0	0/1	0/0	0/0	0/0
Chromosome2-2358112 INS	PRECISE	364	0	0.577	0/0	0/0	0/0	0/0	0/0	0/0	0/0	0/0	1/1	0/0	0/0	0/1	0/1	0/0
Chromosome2-2359347 INS	PRECISE	363	0	0.707	0/0	0/0	0/0	0/0	0/0	0/0	0/0	0/0	0/0	1/1	0/0	0/1	0/0	0/0
Chromosome2-2360109 INS	PRECISE	879	0	0	0/0	0/0	0/0	0/0	0/0	0/0	0/0	0/0	0/0	1/1	0/0	0/1	0/0	0/0
Chromosome2-2360366 INS	PRECISE	321	0	0.5	0/0	0/0	0/0	0/0	0/0	0/0	0/0	0/0	1/1	0/0	0/1	0/1	0/1	0/0
Chromosome2-2360469 INS	PRECISE	161	0	0	0/0	0/0	0/0	0/0	0/0	0/0	0/0	0/0	0/0	1/1	0/0	0/1	0/0	0/0
Chromosome2-2364249 INS	PRECISE	341	0	0	0/0	0/0	0/0	0/0	0/0	0/0	0/0	0/0	0/0	1/1	0/0	0/1	0/0	0/0
Chromosome2-2364527 INS	PRECISE	85	0	0	0/0	0/0	0/0	0/0	0/0	0/0	0/0	0/0	1/1	1/1	0/1	1/1	1/1	0/0
Chromosome2-2367588 INS	PRECISE	653	0	0.707	0/0	0/0	0/0	0/0	0/0	0/0	0/0	0/0	0/0	1/1	0/0	0/1	0/0	0/0
Chromosome2-2367950 DEL	PRECISE	144	0	0	0/0	0/0	0/0	0/0	0/0	0/0	0/0	0/0	0/0	1/1	0/0	0/1	0/0	0/0
Chromosome2-2369484 INS	PRECISE	144	0	0	0/0	0/0	0/0	0/0	0/0	0/0	0/0	0/0	0/0	1/1	0/0	0/1	0/0	0/0
Chromosome2-2371085 BND	PRECISE	0	0	0	0/0	0/0	0/0	0/0	0/0	0/0	0/0	0/0	0/1	0/0	0/0	0/0	0/0	0/0
Chromosome2-2371217 DEL	PRECISE	12402	0	0	0/0	0/0	0/0	0/0	0/0	0/0	0/0	0/0	0/0	1/1	0/1	0/1	0/0	0/0
Chromosome2-2371506 DEL	PRECISE	76	0.577	0.577	0/0	0/0	0/0	0/0	0/0	0/0	0/0	0/0	1/1	0/0	0/0	0/1	0/1	0/0
Chromosome2-2372907 DEL	PRECISE	8382	0	0	0/0	0/0	0/0	0/0	0/0	0/0	0/0	0/0	0/0	1/1	0/0	0/1	0/0	0/0
Chromosome2-2374632 INS	PRECISE	360	0	0.707	0/0	0/0	0/0	0/0	0/0	0/0	0/0	0/0	0/0	1/1	0/0	0/1	0/0	0/0
Chromosome2-2374682 DEL	PRECISE	4517	0	0	0/0	0/0	0/0	0/0	0/0	0/0	0/0	0/0	1/1	0/0	0/0	0/1	0/1	0/0
Chromosome2-2376744 INS	PRECISE	1012	0	0.707	0/0	0/0	0/0	0/0	0/0	0/0	0/0	0/0	0/0	1/1	0/0	0/1	0/0	0/0
Chromosome2-2378294 INS	PRECISE	1368	0	0	0/0	0/0	0/0	0/0	0/0	0/0	0/0	0/0	0/0	0/0	0/1	0/0	0/0	0/0
Chromosome2-2379760 DEL	PRECISE	54	0	0	0/0	0/0	0/0	0/0	0/0	0/0	0/0	0/0	0/0	1/1	0/0	0/1	0/0	0/0
Chromosome2-2380642 DEL	PRECISE	159	0	0	0/0	0/0	0/0	0/0	0/0	0/0	0/0	0/0	0/0	1/1	0/0	0/1	0/0	0/0
Chromosome2-2381395 INS	PRECISE	1544	0	0.707	0/0	0/0	0/0	0/0	0/0	0/0	0/0	0/0	0/0	0/0	0/0	1/1	0/0	0/0
Chromosome2-2381489 DEL	PRECISE	862	0	0	0/0	0/0	0/0	0/0	0/0	0/0	0/0	0/0	0/0	1/1	0/0	0/0	0/0	0/0
Chromosome2-2383942 DEL	PRECISE	10024	0	0	0/0	0/0	0/0	0/0	0/0	0/0	0/0	0/0	0/0	1/1	0/0	0/1	0/0	0/0
Chromosome2-2385470 DEL	PRECISE	23457	0	0	0/0	0/0	0/0	0/0	0/0	0/0	0/0	0/0	0/0	1/1	0/0	0/1	0/0	0/0
Chromosome2-2386576 BND	PRECISE	0	0	0	0/0	0/0	0/0	0/0	0/0	0/0	0/0	0/0	0/0	0/0	0/0	0/1	0/0	0/0
Chromosome2-2386709 DEL	PRECISE	901	0	0	0/0	0/0	0/0	0/0	0/0	0/0	0/0	0/1	0/0	0/0	0/0	0/1	0/0	0/0
Chromosome2-2389599 INS	PRECISE	58	0	0	0/0	0/0	0/0	0/0	0/0	0/0	0/0	0/0	0/0	1/1	0/0	0/1	0/0	0/0
Chromosome2-2395586 INS	PRECISE	134	0	0	0/0	0/0	0/0	0/0	0/0	0/0	0/0	0/0	0/0	1/1	0/0	0/1	0/0	0/0
Chromosome2-2395991 DEL	PRECISE	197	0	0	0/0	0/0	0/0	0/0	0/0	0/0	0/0	0/0	0/0	1/1	0/0	0/1	0/0	0/0
Chromosome2-2396800 DEL	PRECISE	756	0	0	0/0	0/0	0/0	0/0	0/0	0/0	0/0	0/0	0/0	1/1	0/0	1/1	0/0	0/0
Chromosome2-2397208 DEL	PRECISE	9563	0	0	0/0	0/0	0/0	0/0	0/0	0/0	0/0	0/0	0/0	0/0	0/0	1/1	0/0	0/0
Chromosome2-2397226 INS	PRECISE	8203	0	0	0/0	0/0	0/0	0/0	0/0	0/0	0/0	0/0	0/0	0/0	0/0	0/0	0/1	0/0
Chromosome2-2397352 INS	PRECISE	8205	0	0	0/0	0/0	0/0	0/0	0/0	0/0	0/0	0/0	0/1	0/0	0/0	0/0	0/0	0/0
Chromosome2-2397481 INS	PRECISE	8219	0	0	0/0	0/0	0/0	0/0	0/0	0/0	0/0	0/0	0/1	0/0	0/0	0/0	0/0	0/0
Chromosome2-2397931 INS	PRECISE	8198	0	0	0/0	0/0	0/0	0/0	0/0	0/0	0/0	0/0	0/0	0/0	0/0	0/0	0/1	0/0
Chromosome2-2398244 DEL	PRECISE	356	0	0	0/0	0/0	0/0	0/0	0/0	0/0	0/0	0/0	0/0	1/1	0/0	0/1	0/0	0/0
Chromosome2-2398280 BND	PRECISE	0	0	0	0/0	0/0	0/0	0/0	0/0	0/0	0/0	0/0	0/0	0/1	0/0	0/0	0/0	0/0
Chromosome2-2398818 DEL	PRECISE	362	0	0	0/0	0/0	0/0	0/0	0/0	0/0	0/0	0/0	0/0	1/1	0/0	0/1	0/0	0/0
Chromosome2-2399346 DEL	PRECISE	61	0	0	0/0	0/0	0/0	0/0	0/0	0/0	0/0	0/0	0/0	1/1	0/0	0/1	0/0	0/0
Chromosome2-2399527 INS	PRECISE	103	0	0	0/0	0/0	0/0	0/0	0/0	0/0	0/0	0/0	0/0	1/1	0/0	0/1	0/0	0/0
Chromosome2-2399669 INS	PRECISE	56	0	0	0/0	0/0	0/0	0/0	0/0	0/0	0/0	0/0	0/0	1/1	0/0	0/1	0/0	0/0
Chromosome2-2399882 DEL	PRECISE	12035	0	0	0/0	0/0	0/0	0/0	0/0	0/0	0/0	0/0	0/0	1/1	0/0	0/1	0/0	0/0
Chromosome2-2403505 INS	PRECISE	59	0	0.707	0/0	0/0	0/0	0/0	0/0	0/0	0/0	0/0	0/0	1/1	0/0	0/1	0/0	0/0
Chromosome2-2403973 DEL	PRECISE	90	0	0	0/0	0/0	0/0	0/0	0/0	0/0	0/0	0/0	0/0	1/1	0/0	0/1	0/0	0/0
Chromosome2-2404905 DEL	PRECISE	1908	0	0	0/0	0/0	0/0	0/0	0/0	0/0	0/0	0/0	0/0	1/1	0/0	0/1	0/0	0/0
Chromosome2-2405863 DEL	PRECISE	400	0	0	0/0	0/0	0/0	1/1	1/1	0/0	1/1	1/1	0/1	0/0	0/0	0/0	0/0	0/0
Chromosome2-2406643 BND	PRECISE	0	0	0	0/0	0/0	0/0	0/0	0/0	0/0	0/0	0/0	0/0	1/1	0/0	0/1	0/0	0/0
Chromosome2-2408005 INS	PRECISE	52	0	0	0/0	0/0	0/0	0/0	0/0	0/0	0/0	0/0	0/0	1/1	0/0	0/1	0/0	0/0
Chromosome2-2411273 DEL	PRECISE	982	0	0	0/0	0/0	0/0	0/0	0/0	0/0	0/0	0/0	0/0	1/1	0/0	0/1	0/0	0/0
Chromosome2-2414528 DEL	PRECISE	4364	0	0	0/0	0/0	0/0	0/0	0/0	0/0	0/0	0/0	0/0	0/0	0/1	0/0	0/0	0/0
Chromosome2-2417056 INS	PRECISE	688	0	0	0/0	0/0	0/0	0/0	0/0	0/0	0/0	0/0	0/0	1/1	0/0	0/1	0/0	0/0
Chromosome2-2420460 DEL	PRECISE	9913	0	0	0/0	0/0	0/0	0/0	0/0	0/0	0/0	0/0	0/0	1/1	0/0	0/1	0/0	0/0
Chromosome2-2422056 INS	PRECISE	877	0	0	0/0	0/0	0/0	0/0	0/0	0/0	0/0	0/0	0/0	0/0	0/1	0/0	0/0	0/0
Chromosome2-2427872 DEL	PRECISE	197	0	0	0/0	0/0	0/0	1/1	0/0	0/0	0/0	0/0	0/0	0/0	0/0	0/0	0/0	0/0
Chromosome2-2428574 INS	PRECISE	12470	0	0	0/0	0/0	0/0	0/0	0/0	0/0	0/0	0/0	0/0	0/0	0/0	0/1	0/0	0/0
Chromosome2-2429971 INS	PRECISE	1357	0	0	0/0	0/0	0/0	0/0	0/0	0/0	0/0	0/0	0/0	0/0	0/1	0/0	0/0	0/0
Chromosome2-2430236 DEL	PRECISE	12193	0	0	0/0	0/0	1/1	0/0	0/1	0/0	0/0	0/0	0/0	0/0	0/0	0/0	0/0	0/0
Chromosome2-2430921 INS	PRECISE	970	0	0	0/0	0/0	0/0	0/0	0/0	0/0	0/0	0/0	0/0	0/0	0/0	0/1	0/0	0/0
Chromosome2-2431646 DEL	PRECISE	204	0	0	0/0	0/0	1/1	0/0	1/1	0/0	0/0	0/0	0/0	0/0	0/0	0/0	0/0	0/0
Chromosome2-2432843 DEL	PRECISE	60	0	0	0/0	0/0	0/0	0/0	0/0	0/0	0/0	0/0	0/0	1/1	0/0	0/0	0/0	0/0
Chromosome2-2436400 INS	PRECISE	12529	0	0	0/0	0/0	0/0	0/0	0/0	0/0	0/0	0/0	0/0	0/0	0/0	1/1	0/0	0/0
Chromosome2-2436400 BND	PRECISE	0	0	0	0/0	0/0	0/0	0/0	0/0	0/0	0/0	0/0	0/0	0/1	0/0	0/0	0/0	0/0
Chromosome2-2436400 BND	PRECISE	0	0	0	0/0	0/0	0/0	0/0	0/0	0/0	0/0	0/0	0/0	0/1	0/0	0/0	0/0	0/0
Chromosome2-2436400 BND	PRECISE	0	0	0	0/0	0/0	0/0	0/0	0/0	0/0	0/0	0/0	0/0	0/1	0/0	0/0	0/0	0/0

Chromosome2-2443598	DEL	PRECISE	51	0	0	0/0	0/0	1/1	0/0	0/0	0/0	0/0	0/0	0/0	0/0	0/0	0/0	0/0	0/0
Chromosome2-2443941	DEL	PRECISE	334	0	0	0/0	0/0	1/1	0/0	0/0	0/0	0/0	0/0	0/0	0/0	0/0	0/0	0/0	0/0
Chromosome2-2448196	INS	PRECISE	117	0	0	0/0	0/0	0/0	0/0	0/0	0/0	0/0	0/0	0/0	0/1	0/0	0/0	0/0	0/1
Chromosome2-2448585	INS	PRECISE	61	0	0.707	0/0	0/0	0/0	0/0	0/0	0/0	0/0	0/0	0/0	1/1	0/0	0/1	0/0	0/0
Chromosome2-2453077	DEL	PRECISE	50	0	0	0/0	0/0	0/0	0/0	0/0	0/0	0/0	0/0	0/0	1/1	0/0	0/1	0/0	0/0
Chromosome2-2453465	BND	PRECISE	0	0	0	0/0	0/1	0/0	0/0	0/0	0/0	0/0	0/0	0/0	0/0	0/0	0/0	0/0	0/0
Chromosome2-2453986	DEL	PRECISE	1319	0	0	0/0	0/1	0/0	0/0	0/0	0/0	0/0	0/0	0/1	0/0	0/0	0/0	0/1	0/0
Chromosome2-2454548	INS	PRECISE	210	0	0.837	0/0	0/1	0/0	0/0	0/0	0/0	0/0	0/0	0/1	0/0	0/1	0/0	0/1	0/0
Chromosome2-2455292	DEL	PRECISE	179	0	0	0/0	0/0	0/0	0/0	0/0	0/0	0/0	0/0	0/0	1/1	0/0	0/1	0/0	0/0
Chromosome2-2456916	INS	PRECISE	121	0	0.707	0/0	0/0	0/0	0/0	0/0	0/0	0/0	0/0	0/0	1/1	0/0	0/1	0/0	0/0
Chromosome2-2458315	INS	PRECISE	153	0	0.707	0/0	0/0	0/0	0/0	0/0	0/0	0/0	0/0	0/0	1/1	0/0	0/1	0/0	0/0
Chromosome2-2459660	INS	PRECISE	63	0	0	0/0	0/0	0/0	0/0	0/0	0/0	0/0	0/0	0/0	1/1	0/0	0/1	0/0	0/0
Chromosome2-2462818	DEL	PRECISE	513	0	0	0/0	0/0	0/0	0/0	0/0	0/0	0/0	0/0	1/1	1/1	0/1	1/1	0/1	0/0
Chromosome2-2467202	DEL	PRECISE	1579	0	0	0/0	0/0	0/0	0/0	0/0	0/0	0/0	0/0	0/0	0/0	0/0	0/1	0/0	0/0
Chromosome2-2468365	INS	PRECISE	11718	0	0	0/0	0/0	0/0	0/0	0/0	0/0	0/0	0/0	0/0	0/0	0/0	0/1	0/0	0/0
Chromosome2-2468544	INS	PRECISE	805	0	0	0/0	0/0	0/0	0/0	0/0	0/0	0/0	0/0	0/1	0/0	0/0	0/0	0/0	0/0
Chromosome2-2468880	DEL	PRECISE	1941	0	0	0/0	0/0	0/0	0/0	0/0	0/0	0/0	0/0	0/0	1/1	0/0	0/1	0/0	0/0
Chromosome2-2469558	DEL	PRECISE	52	0	0	0/0	0/0	0/0	0/0	0/0	0/0	0/0	0/0	0/0	1/1	0/0	0/1	0/0	0/0
Chromosome2-2470284	DEL	PRECISE	50	0	0	0/0	0/0	0/0	0/0	0/0	0/0	0/0	0/0	0/0	1/1	0/0	0/1	0/0	0/0
Chromosome2-2470779	DEL	PRECISE	60	0	0	0/0	0/0	0/0	0/0	0/0	0/0	0/0	0/0	0/1	0/0	0/0	0/0	0/1	0/0
Chromosome2-2471105	DEL	PRECISE	173	0.707	0.707	0/0	0/0	0/0	0/0	0/0	0/0	0/0	0/0	0/0	1/1	0/0	0/1	0/0	0/0
Chromosome2-2471606	BND	PRECISE	0	0	0	0/0	0/0	0/0	0/0	0/0	0/0	0/0	0/0	0/1	0/0	0/0	0/0	0/0	0/0
Chromosome2-2475328	BND	PRECISE	0	0	0	0/0	0/0	0/0	0/0	0/0	0/0	0/0	0/0	1/1	0/0	0/1	0/1	0/0	0/0
Chromosome2-2476648	INS	PRECISE	150	0	0	0/0	0/0	0/0	0/0	0/0	0/0	0/0	0/0	0/0	1/1	0/0	0/1	0/0	0/0
Chromosome2-2477680	DEL	PRECISE	1280	0.577	0	0/0	0/0	1/1	0/0	1/1	0/0	0/0	0/0	0/0	0/0	0/0	0/0	0/0	0/0
Chromosome2-2483071	DEL	PRECISE	2787	0	0	0/0	0/0	0/0	0/0	0/0	0/0	0/0	0/0	0/0	1/1	0/0	0/1	0/0	0/0
Chromosome2-2483098	DEL	PRECISE	51	0	0	0/0	0/1	1/1	0/0	0/1	1/1	0/0	1/1	0/1	0/0	0/0	0/0	0/0	0/0
Chromosome2-2483165	INS	PRECISE	105	0	0	0/0	0/1	1/1	1/1	0/1	1/1	0/0	1/1	0/1	0/0	0/0	0/0	0/0	0/0
Chromosome2-2483896	DEL	PRECISE	10584	0.577	0.577	0/0	0/0	0/0	0/0	0/0	0/0	0/0	0/0	0/1	0/0	0/0	0/1	0/1	0/0
Chromosome2-2483973	DEL	PRECISE	503	0	0	0/0	0/0	0/0	0/0	0/0	0/0	0/0	0/0	0/0	0/0	0/0	0/0	0/0	0/1
Chromosome2-2484097	DEL	PRECISE	3184	0	0	0/0	0/0	1/1	0/0	1/1	1/1	0/0	0/0	0/0	0/1	0/0	0/0	0/0	0/0
Chromosome2-2484975	BND	PRECISE	0	0	0	0/0	0/0	0/0	0/0	0/0	0/0	0/0	0/0	0/1	0/0	0/1	0/1	0/0	0/0
Chromosome2-2486196	DEL	PRECISE	68	0	0	0/0	0/0	0/0	0/0	0/0	0/0	0/0	0/0	0/0	1/1	0/0	0/1	0/0	0/0
Chromosome2-2487332	DEL	PRECISE	91	0	0	0/0	0/0	0/0	0/0	0/0	0/0	0/0	0/0	0/0	1/1	0/0	0/1	0/0	0/0
Chromosome2-2489760	DEL	PRECISE	317	0	0	0/0	0/0	0/0	0/0	0/0	0/0	1/1	0/0	0/0	0/0	0/0	0/0	0/0	0/0
Chromosome2-2489850	DEL	PRECISE	554	0	0	0/0	0/0	0/0	0/0	0/0	0/0	1/1	0/0	0/0	0/0	0/0	0/0	0/0	0/0
Chromosome2-2491285	INS	PRECISE	162	0	0	0/0	0/0	0/0	0/0	0/0	0/0	0/0	0/0	0/0	1/1	0/0	0/1	0/0	0/0
Chromosome2-2491428	DEL	PRECISE	50	0	0	0/0	0/0	0/0	0/0	0/0	0/0	0/0	0/0	0/0	1/1	0/0	0/1	0/0	0/0
Chromosome2-2492106	INS	PRECISE	5159	0	0.707	0/0	0/0	0/0	0/0	0/0	0/0	0/0	0/0	0/0	0/0	0/0	1/1	0/0	0/0
Chromosome2-2492106	BND	PRECISE	0	0	0	0/0	0/0	0/0	0/0	0/0	0/0	0/0	0/0	0/0	0/1	0/0	0/1	0/0	0/0
Chromosome2-2492217	DEL	PRECISE	3257	0	0	0/0	0/0	0/0	0/0	0/0	0/0	0/0	0/0	0/0	1/1	0/0	0/1	0/0	0/0
Chromosome2-2493531	INS	PRECISE	346	0	0	0/0	0/0	0/0	0/0	0/0	0/0	0/0	0/0	0/0	0/0	0/1	0/0	0/0	0/0
Chromosome2-2498133	DEL	PRECISE	106	0	0	0/0	0/0	0/0	0/0	0/0	0/0	0/0	0/0	0/0	1/1	0/0	0/1	0/0	0/0
Chromosome2-2499525	BND	PRECISE	0	0	0	0/0	0/0	0/0	0/0	0/0	0/0	0/0	0/0	0/0	0/1	0/0	0/1	0/0	0/0
Chromosome2-2509695	DEL	PRECISE	602	0	0	0/0	0/0	0/0	0/0	0/0	0/0	0/0	0/0	0/0	1/1	0/0	0/1	0/0	0/0
Chromosome2-2512175	DUP	PRECISE	11718	0	0	0/0	0/0	0/0	0/0	0/0	0/0	0/0	0/0	0/0	0/1	0/0	0/0	0/0	0/0
Chromosome2-2516270	DEL	PRECISE	846	0	0	0/0	0/0	0/0	0/0	0/0	0/0	0/0	0/0	0/1	0/0	0/0	0/1	0/1	0/0
Chromosome2-2516604	DEL	PRECISE	12504	0	0	0/0	0/0	0/0	0/0	0/0	0/0	0/0	0/0	1/1	1/1	0/1	1/1	0/1	0/0
Chromosome2-2517855	BND	PRECISE	0	0	0	0/0	0/0	0/0	0/0	0/0	0/0	0/0	0/0	0/0	0/1	0/0	0/0	0/0	0/0
Chromosome2-2518211	DEL	PRECISE	57	0	0	0/0	0/0	0/0	0/0	0/0	0/0	0/0	0/0	0/1	0/0	0/0	0/0	0/0	0/0
Chromosome2-2520033	INS	PRECISE	103	0	0	0/0	0/0	0/0	0/0	0/0	0/0	0/0	0/0	0/0	0/1	0/0	0/0	0/0	0/0
Chromosome2-2520083	DEL	PRECISE	145	0	0	0/0	0/0	0/0	0/0	0/0	0/0	0/0	0/0	0/0	0/1	0/0	0/0	0/0	0/0
Chromosome2-2520236	DEL	PRECISE	976279	0	0	0/0	0/0	0/0	0/0	0/0	0/0	0/0	0/0	0/0	0/1	0/0	0/0	0/0	0/0
Chromosome2-2520238	INV	PRECISE	308291	0	0	0/0	0/0	0/0	0/0	0/0	0/0	0/0	0/0	0/0	0/0	0/1	0/1	0/1	0/0
Chromosome2-2520377	INS	PRECISE	1021	0	0	0/0	0/0	0/0	0/0	0/0	0/0	0/0	0/0	0/0	1/1	0/0	0/1	0/0	0/0
Chromosome2-2520956	INS	PRECISE	2636	0	0	0/0	0/0	0/0	0/0	0/0	0/0	0/0	0/0	0/0	0/0	0/0	0/0	0/1	0/0
Chromosome2-2521603	BND	PRECISE	0	0	0	0/0	0/0	0/0	0/0	0/0	0/0	0/0	0/0	0/0	0/0	0/0	0/0	0/1	0/0
Chromosome2-2522957	DEL	PRECISE	1333	0	0	0/0	0/0	0/0	0/0	0/0	0/0	0/0	0/0	0/0	1/1	0/0	0/1	0/0	0/0
Chromosome2-2527498	INS	PRECISE	95	0	0	0/0	0/0	0/0	0/0	0/0	0/0	0/0	0/0	0/0	1/1	0/0	0/1	0/0	0/0
Chromosome2-2529265	INS	PRECISE	1021	0	0	0/0	0/0	0/0	0/0	0/0	0/0	0/0	0/0	0/0	1/1	0/0	0/1	0/0	0/0
Chromosome2-2529394	BND	PRECISE	0	0	0	0/0	0/0	0/0	0/1	0/0	0/1	0/1	0/1	0/0	0/0	0/0	0/0	0/0	0/0
Chromosome2-2529418	DEL	PRECISE	8692	0	0	0/0	0/0	0/0	0/0	0/0	0/0	0/0	0/0	0/1	0/0	0/0	0/1	0/1	0/0
Chromosome2-2529528	DEL	PRECISE	75	0	0	0/1	0/0	1/1	0/0	0/0	0/1	0/1	1/1	0/0	0/0	0/0	0/0	0/0	0/0
Chromosome2-2531584	DEL	PRECISE	7278	0	0	0/0	0/0	0/0	0/0	0/0	0/0	0/0	0/0	0/0	0/0	0/0	0/0	0/1	0/0
Chromosome2-2531786	INS	PRECISE	327	0	0	0/0	0/0	0/0	0/0	0/0	0/0	0/0	0/0	0/0	0/0	0/1	0/0	0/0	0/0
Chromosome2-2532312	DEL	PRECISE	14722	0	0	0/0	0/0	0/0	0/0	0/0	0/0	0/0	0/0	0/0	0/0	0/0	1/1	0/0	0/0
Chromosome2-2532580	DEL	PRECISE	5825	0	0	0/0	0/0	0/0	0/0	0/0	0/0	0/0	0/0	0/0	0/0	0/1	0/0	0/0	0/0
Chromosome2-2532792	DEL	PRECISE	213	0	0	0/0	0/0	0/0	0/0	0/0	0/0	0/0	0/0	1/1	0/0	0/0	1/1	0/1	0/0
Chromosome2-2533365	DEL	PRECISE	4334	0	0	0/0	0/0	0/0	0/0	0/0	0/0	0/0	0/0	0/0	0/0	0/1	0/0	0/0	0/0
Chromosome2-2533458	INS	PRECISE	156	0	0	0/0	0/0	0/0	0/0	0/0	0/0	0/0	0/0	1/1	0/0	0/0	1/1	0/1	0/0
Chromosome2-2533852	INS	PRECISE	700	0	0	0/0	0/0	0/0	0/0	0/0	0/0	0/0	0/0	0/0	1/1	0/0	0/1	0/0	0/0
Chromosome2-2536067	DEL	PRECISE	1032	0	0	0/0	0/0	0/0	0/0	0/0	0/0	0/0	0/0	0/0	0/0	0/1	0/0	0/0	0/0
Chromosome2-2536405	INS	PRECISE	75	0	0	0/0	0/0	0/0	0/0	0/0	0/0	0/0	0/0	0/0	1/1	0/0	0/1	0/0	0/0

Chromosome2-2536506 INS	PRECISE	833	0	0	0/0	0/0	0/0	0/0	0/0	0/0	0/0	0/0	0/1	0/0	0/0	0/0	0/0	0/0
Chromosome2-2536507 DEL	PRECISE	12424	0	0	0/0	0/0	0/0	0/0	0/0	0/0	0/0	0/0	0/0	1/1	0/0	0/1	0/0	0/0
Chromosome2-2537159 INS	PRECISE	64	0	0.577	0/0	0/0	0/0	0/0	0/0	0/0	0/0	0/0	0/1	0/0	0/0	0/1	0/1	0/0
Chromosome2-2537868 DEL	PRECISE	186	0	0	0/0	0/0	0/0	0/0	0/0	0/0	0/0	0/0	0/0	1/1	0/0	0/1	0/0	0/0
Chromosome2-2538943 DEL	PRECISE	1021	0	0	0/0	0/0	0/0	0/0	0/0	0/0	0/0	0/0	0/0	1/1	0/0	0/1	0/0	0/0
Chromosome2-2539820 DEL	PRECISE	9644	0	0	0/0	0/0	0/0	0/0	0/0	0/0	0/0	0/0	0/0	1/1	0/0	0/1	0/0	0/0
Chromosome2-2541608 DEL	PRECISE	74	0	0	0/0	0/0	0/0	0/0	0/0	0/0	0/0	0/0	0/0	1/1	0/0	0/1	0/0	0/0
Chromosome2-2542005 DEL	PRECISE	1020	0	0	0/0	0/0	0/0	0/0	0/0	0/0	0/0	0/0	0/0	1/1	0/0	0/1	0/0	0/0
Chromosome2-2542369 DEL	PRECISE	58	0	0	0/0	0/0	0/0	0/0	0/0	0/0	0/0	0/0	0/0	0/0	0/1	0/0	0/0	0/0
Chromosome2-2547538 DEL	PRECISE	639	0	0	0/0	0/0	0/0	0/0	0/0	0/0	0/0	0/0	0/0	1/1	0/0	0/1	0/0	0/0
Chromosome2-2549325 INS	PRECISE	104	0	0.447	0/0	0/0	0/0	0/0	0/0	0/0	0/0	0/0	1/1	1/1	0/1	1/1	0/1	0/0
Chromosome2-2549996 DEL	PRECISE	5699	0	0	0/0	0/0	0/0	0/0	0/0	0/0	0/0	0/0	0/0	0/1	0/0	0/1	0/0	0/0
Chromosome2-2550744 INS	PRECISE	8331	0	0	0/0	0/0	0/0	0/0	0/0	0/0	0/0	0/0	0/0	0/0	0/1	0/0	0/0	0/0
Chromosome2-2550747 DEL	PRECISE	483	0	0	0/0	0/0	0/0	0/0	0/0	0/0	0/0	0/0	0/0	0/0	0/1	0/0	0/0	0/0
Chromosome2-2551338 INS	PRECISE	16628	0	0	0/0	0/0	0/0	0/0	0/0	0/0	0/0	0/0	0/1	0/0	0/0	0/0	0/0	0/0
Chromosome2-2551640 DEL	PRECISE	1173	0	0	0/0	0/0	0/0	0/0	0/0	0/0	0/0	0/0	0/0	1/1	0/0	0/1	0/0	0/0
Chromosome2-2552166 INS	PRECISE	334	0	0	0/0	0/0	0/0	0/0	0/0	0/0	0/0	0/0	0/0	0/0	0/0	0/0	0/1	0/0
Chromosome2-2552638 INS	PRECISE	1607	0	0	0/0	0/0	0/0	0/0	0/0	0/0	0/0	0/0	0/0	0/0	0/0	0/0	0/1	0/0
Chromosome2-2552652 DEL	PRECISE	64382	0	0	0/0	0/0	0/0	0/0	0/0	0/0	0/0	0/0	1/1	0/0	1/1	1/1	0/1	0/0
Chromosome2-2552775 INS	PRECISE	277	0	0	0/0	0/0	0/0	0/0	0/0	0/0	0/0	0/0	0/0	0/0	0/0	0/0	0/1	0/0
Chromosome2-2553066 DEL	PRECISE	1842	0	0	0/0	0/0	0/0	0/0	0/0	0/0	0/0	0/0	0/0	0/0	0/1	0/0	0/0	0/0
Chromosome2-2554803 INS	PRECISE	7033	0	0	0/0	0/1	0/0	0/0	0/0	0/0	0/0	0/0	0/0	0/0	0/0	0/0	0/0	0/0
Chromosome2-2555576 DEL	PRECISE	4963	0	0	0/0	0/0	0/0	0/0	0/0	0/0	0/0	0/0	0/0	0/0	0/0	0/0	0/1	0/0
Chromosome2-2556078 DEL	PRECISE	1857	0	0	0/0	0/0	0/0	0/0	0/0	0/0	0/0	0/0	0/0	0/0	0/0	0/0	0/1	0/0
Chromosome2-2556267 DEL	PRECISE	540	0	0	0/0	0/0	0/0	0/0	0/0	0/0	0/0	0/0	0/0	0/0	0/0	0/0	0/1	0/0
Chromosome2-2556532 DEL	PRECISE	870	0	0	0/0	0/0	0/0	0/0	0/0	0/0	0/0	0/0	0/0	0/0	0/0	0/0	0/1	0/0
Chromosome2-2557446 INS	PRECISE	3625	0	0	0/0	0/0	0/0	0/0	0/0	0/0	0/0	0/0	0/0	0/0	0/0	0/0	0/1	0/0
Chromosome2-2559359 INV	PRECISE	734311	0	0	0/0	0/0	0/0	0/0	0/0	0/0	0/0	0/0	0/0	0/0	0/0	0/1	0/0	0/0
Chromosome2-2561049 INS	PRECISE	9497	0	0	0/0	0/0	0/0	0/0	0/0	0/0	0/0	0/0	0/0	0/0	1/1	0/0	0/0	0/0
Chromosome2-2561051 BND	PRECISE	0	0	0	0/0	0/0	0/0	0/0	0/0	0/0	0/0	0/0	0/0	0/0	0/1	0/0	0/0	0/0
Chromosome2-2562115 DEL	PRECISE	63	0	0	0/0	0/0	0/0	0/0	0/0	0/0	0/0	0/0	1/1	0/0	0/1	1/1	0/1	0/0
Chromosome2-2564393 INS	PRECISE	7000	0	0.707	0/0	0/0	0/0	0/0	0/0	0/0	0/0	0/0	0/0	1/1	0/0	0/0	0/0	0/0
Chromosome2-2564599 INS	PRECISE	9193	0	0	0/0	0/0	0/0	0/0	0/0	0/0	0/0	0/0	0/0	0/0	0/0	0/0	0/0	0/0
Chromosome2-2565007 DEL	PRECISE	201	0	0	0/0	0/0	0/0	0/0	0/0	0/0	0/0	0/0	1/1	0/0	0/0	0/1	0/1	0/0
Chromosome2-2565595 DEL	PRECISE	89	0	0	0/0	0/0	0/0	0/0	0/0	0/0	0/0	0/0	0/0	1/1	0/0	0/1	0/0	0/0
Chromosome2-2566024 INS	PRECISE	2756	0	0.707	0/0	0/0	0/0	0/0	0/0	0/0	0/0	0/0	0/0	1/1	0/0	0/1	0/0	0/0
Chromosome2-2566844 INS	PRECISE	73	0	0	0/0	0/0	0/0	0/0	0/0	0/0	0/0	0/0	0/0	1/1	0/0	0/1	0/0	0/0
Chromosome2-2569497 INS	PRECISE	9890	0.707	0.707	0/0	0/0	0/0	0/0	0/0	0/0	0/0	0/0	0/0	1/1	0/0	1/1	0/0	0/0
Chromosome2-2569498 BND	PRECISE	0	0	0	0/0	0/0	0/0	0/0	0/0	0/0	0/0	0/0	0/0	0/1	0/0	0/0	0/0	0/0
Chromosome2-2573961 DEL	PRECISE	17151	0	0	0/0	0/0	0/0	0/0	0/0	0/0	0/0	0/0	1/1	0/0	0/1	0/1	0/1	0/0
Chromosome2-2574906 DEL	PRECISE	72	0	0	0/0	0/0	0/0	0/0	0/0	0/0	0/0	0/0	0/0	0/1	0/0	0/1	0/0	0/0
Chromosome2-2575995 DEL	PRECISE	5322	0	0	0/0	0/0	0/0	0/0	0/0	0/0	0/0	0/0	0/0	0/0	0/1	0/0	0/0	0/0
Chromosome2-2577255 DEL	PRECISE	80	0.577	0.577	0/0	0/0	0/0	0/0	0/0	0/0	0/0	0/0	1/1	0/0	0/0	0/1	0/1	0/0
Chromosome2-2577955 DEL	PRECISE	11460	0.707	0.707	0/0	0/0	0/0	0/0	0/0	0/0	0/0	0/0	0/0	0/1	0/0	0/1	0/0	0/0
Chromosome2-2577955 DEL	PRECISE	4170	0	0	0/0	0/0	0/0	0/0	0/0	0/0	0/0	0/0	0/0	0/1	0/0	0/0	0/0	0/0
Chromosome2-2578109 DEL	PRECISE	23042	0	0	0/0	0/0	0/0	0/0	0/0	0/0	0/0	0/0	1/1	0/0	0/0	0/1	0/1	0/0
Chromosome2-2582184 DEL	PRECISE	11363	0	0	0/0	0/0	0/0	0/0	0/0	0/0	0/0	0/0	0/0	0/0	0/0	0/0	0/1	0/0
Chromosome2-2582395 DEL	PRECISE	81	0	0	0/0	0/0	0/0	0/0	0/0	0/0	0/0	0/0	0/0	1/1	0/0	1/1	0/0	0/0
Chromosome2-2582628 DEL	PRECISE	400	0	0	0/0	0/0	0/0	0/0	0/0	0/0	0/0	0/0	0/0	1/1	0/1	1/1	0/0	0/0
Chromosome2-2584641 DEL	PRECISE	276	0	0	0/0	0/0	0/0	0/0	0/0	0/0	0/0	0/0	0/0	0/0	0/1	1/1	0/0	0/0
Chromosome2-2584711 DEL	PRECISE	609	0	0	0/0	0/0	0/0	0/0	0/0	0/0	0/0	0/0	0/0	0/0	0/0	0/1	0/0	0/0
Chromosome2-2585149 INS	PRECISE	7315	0	0	0/0	0/0	0/0	0/0	0/0	0/0	0/0	0/0	0/0	0/0	0/0	0/0	0/0	0/0
Chromosome2-2585279 INS	PRECISE	7274	0	0	0/0	0/0	0/0	0/0	0/0	0/0	0/0	0/0	0/0	0/0	0/0	0/0	0/0	0/0
Chromosome2-2586347 DEL	PRECISE	1286	0	0	0/0	0/0	0/0	0/0	0/0	0/0	0/0	0/0	0/0	1/1	0/0	0/1	0/0	0/0
Chromosome2-2586499 DEL	PRECISE	247	0	0	0/0	0/0	0/0	0/0	0/0	0/0	0/0	0/0	1/1	0/0	0/0	0/1	0/1	0/0
Chromosome2-2586663 INS	PRECISE	56	0	0	0/0	0/0	0/0	0/0	0/0	0/0	0/0	0/0	1/1	1/1	0/1	1/1	0/1	0/0
Chromosome2-2587427 BND	PRECISE	0	0	0	0/0	0/0	0/0	0/0	0/0	0/0	0/0	0/0	0/0	0/0	0/0	0/1	0/0	0/0
Chromosome2-2587518 INS	PRECISE	55	0	0	0/0	0/0	0/0	0/0	0/0	0/0	0/0	0/0	0/1	0/0	0/1	0/1	0/1	0/0
Chromosome2-2587880 DEL	PRECISE	1127	0	0	0/0	0/0	0/0	0/0	0/0	0/0	0/0	0/0	0/1	0/0	0/1	0/1	0/1	0/0
Chromosome2-2588376 BND	PRECISE	0	0	0	0/0	0/0	0/0	0/0	0/0	0/0	0/0	0/0	0/1	0/0	0/0	0/0	0/1	0/0
Chromosome2-2588965 DEL	PRECISE	54	0	0	0/0	0/0	0/0	0/0	0/0	0/0	0/0	0/0	0/0	1/1	0/0	0/1	0/0	0/0
Chromosome2-2589116 DEL	PRECISE	62	0	0	0/0	0/0	0/0	0/0	0/0	0/0	0/0	0/0	1/1	0/0	0/0	0/1	0/1	0/0
Chromosome2-2592428 INS	PRECISE	137	0	0	0/0	0/0	0/0	0/0	0/0	0/0	0/0	0/0	0/0	1/1	0/0	0/1	0/0	0/0
Chromosome2-2593198 INS	PRECISE	139	0	0	0/0	0/0	0/0	0/0	0/0	0/0	0/0	0/0	0/0	1/1	0/0	0/1	0/0	0/0
Chromosome2-2596049 DEL	PRECISE	76	0	0	0/0	0/0	0/0	0/0	0/0	0/0	0/0	0/0	0/0	1/1	0/0	0/1	0/0	0/0
Chromosome2-2599410 BND	PRECISE	0	0	0	0/0	0/0	0/0	0/0	0/0	0/0	0/0	0/0	0/0	0/0	0/1	0/0	0/0	0/0
Chromosome2-2599704 DEL	PRECISE	4120	0	0	0/0	0/0	0/0	0/0	0/0	0/0	0/0	0/0	0/0	1/1	0/0	0/1	0/0	0/0
Chromosome2-2601025 INS	PRECISE	101	0	0	0/0	0/0	0/0	0/0	0/0	0/0	0/0	0/0	0/0	1/1	0/0	0/1	0/0	0/0
Chromosome2-2604666 DEL	PRECISE	752	0	0	0/0	0/0	0/0	0/0	0/0	0/0	0/0	0/0	0/0	1/1	0/0	0/1	0/0	0/0
Chromosome2-2604979 BND	PRECISE	0	0	0	0/0	0/0	0/0	0/0	0/0	0/0	0/0	0/0	0/0	0/0	0/0	0/1	0/0	0/0
Chromosome2-2605418 DEL	PRECISE	86	0	0	0/0	0/0	0/0	0/0	0/0	0/0	0/0	0/0	0/0	1/1	0/0	0/1	0/0	0/0
Chromosome2-2605712 DEL	PRECISE	11956	0.707	0.707	0/0	0/0	0/0	0/0	0/0	0/0	0/0	0/0	0/0	1/1	0/0	0/1	0/0	0/0
Chromosome2-2607254 DEL	PRECISE	59	0	0	0/0	0/0	0/0	0/0	0/0	0/0	0/0	0/0	0/0	1/1	0/0	0/1	0/0	0/0

Chromosome2-2609247 DEL	PRECISE	6093	0.577	0.577	0/0	0/0	0/0	0/0	0/0	0/0	0/0	0/0	1/1	0/0	0/0	1/1	0/1	0/0
Chromosome2-2609610 DEL	PRECISE	213	0	0	0/0	0/0	0/0	0/0	0/0	0/0	0/0	0/0	0/0	1/1	0/1	0/1	0/0	0/0
Chromosome2-2609910 DEL	PRECISE	14973	0	0	0/0	0/0	0/0	0/0	0/0	0/0	0/0	0/0	1/1	1/1	0/1	1/1	0/1	0/0
Chromosome2-2611822 DEL	PRECISE	4189	0	0	0/0	0/0	0/0	0/0	0/0	0/0	0/0	0/0	0/0	0/0	0/1	0/0	0/0	0/0
Chromosome2-2613392 DEL	PRECISE	181	0	0	0/0	0/0	0/0	0/0	0/0	0/0	0/0	0/0	0/0	0/0	0/1	0/0	0/0	0/0
Chromosome2-2615617 INS	PRECISE	12738	0	0	0/0	0/0	0/0	0/0	0/0	0/0	0/0	0/0	0/0	0/0	1/1	0/0	0/0	0/0
Chromosome2-2615617 BND	PRECISE	0	0	0	0/0	0/0	0/0	0/0	0/0	0/0	0/0	0/0	0/0	0/0	0/1	0/0	0/0	0/0
Chromosome2-2615618 BND	PRECISE	0	0	0	0/0	0/0	0/0	0/0	0/0	0/0	0/0	0/0	0/0	0/0	0/1	0/0	0/0	0/0
Chromosome2-2615990 DEL	PRECISE	469	0	0	0/0	0/0	0/0	0/0	0/0	0/0	0/0	0/0	1/1	1/1	0/1	1/1	0/1	0/0
Chromosome2-2616216 INS	PRECISE	50	0	0	0/0	0/0	0/0	0/0	0/0	0/0	0/0	0/0	1/1	1/1	0/1	1/1	0/1	0/0
Chromosome2-2620397 DEL	PRECISE	455	0	0	0/0	0/0	0/0	0/0	0/0	0/0	0/0	0/0	0/1	0/0	0/0	0/1	0/1	0/0
Chromosome2-2620445 DEL	PRECISE	2039	0	0	0/0	0/0	0/0	0/0	0/0	0/0	0/0	0/0	1/1	0/0	0/0	0/1	0/1	0/0
Chromosome2-2621197 DEL	PRECISE	2219	0	0	0/0	0/0	0/0	0/0	0/0	0/0	0/0	0/0	0/0	0/0	0/0	0/1	0/0	0/0
Chromosome2-2621425 DEL	PRECISE	583	0	0	0/0	0/0	0/0	0/0	0/0	0/0	0/0	0/0	0/0	0/0	0/0	0/1	0/0	0/0
Chromosome2-2621743 DEL	PRECISE	4986	0	0	0/0	0/0	0/0	0/0	0/0	0/0	0/0	0/0	0/1	1/1	0/1	1/1	0/1	0/0
Chromosome2-2625743 INS	PRECISE	62	0	0	0/0	0/0	0/0	0/0	0/0	0/0	0/0	0/0	1/1	0/0	0/0	0/1	0/1	0/0
Chromosome2-2625748 DEL	PRECISE	124	0	0	0/0	0/0	0/0	0/0	0/0	0/0	0/0	0/0	0/0	1/1	0/1	0/1	0/0	0/0
Chromosome2-2626646 INS	PRECISE	65	0	0	0/0	0/0	0/0	0/0	0/0	0/0	0/0	0/0	0/0	1/1	0/0	0/1	0/0	0/0
Chromosome2-2627518 INS	PRECISE	312	0	0	0/0	0/0	0/0	0/0	0/0	0/0	0/0	0/0	0/0	1/1	0/0	0/1	0/0	0/0
Chromosome2-2630681 INS	PRECISE	2691	0	0.707	0/0	0/0	0/0	0/0	0/0	0/0	0/0	0/0	0/0	1/1	0/0	1/1	0/0	0/0
Chromosome2-2631270 INS	PRECISE	7000	0	0.707	0/0	0/0	0/0	0/0	0/0	0/0	0/0	0/0	0/0	1/1	0/0	1/1	0/0	0/0
Chromosome2-2631270 DEL	PRECISE	8024	0	0	0/0	0/0	0/0	0/0	0/0	0/0	0/0	0/0	0/0	0/1	0/0	0/0	0/0	0/0
Chromosome2-2633912 DEL	PRECISE	80	0	0	0/0	0/0	0/0	0/0	0/0	0/0	0/0	0/0	0/0	1/1	0/0	0/1	0/0	0/0
Chromosome2-2634503 DEL	PRECISE	63	0.707	0.707	0/0	0/0	0/0	0/0	0/0	0/0	0/0	0/0	0/0	1/1	0/0	0/1	0/0	0/0
Chromosome2-2635112 INS	PRECISE	200	0	0	0/0	0/0	0/0	0/0	0/0	0/0	0/0	0/0	0/0	1/1	0/0	0/1	0/0	0/0
Chromosome2-2635198 DEL	PRECISE	7253	0	0	0/0	0/0	0/0	0/0	0/0	0/0	0/0	0/0	1/1	1/1	0/0	1/1	0/1	0/0
Chromosome2-2635199 INS	PRECISE	1250	0	0	0/0	0/0	0/0	0/0	0/0	0/0	0/0	0/0	0/0	0/0	0/1	0/0	0/0	0/0
Chromosome2-2636683 INS	PRECISE	217	0	0	0/0	0/0	0/0	0/0	0/0	0/0	0/0	0/0	0/0	1/1	0/0	0/1	0/0	0/0
Chromosome2-2637032 DEL	PRECISE	1923	0	0	0/0	0/0	0/0	0/0	0/0	0/0	0/0	0/0	0/0	1/1	0/0	0/1	0/0	0/0
Chromosome2-2644182 INS	PRECISE	586	0	0	0/0	0/0	0/0	0/0	0/0	0/0	0/0	0/0	0/0	1/1	0/0	0/1	0/0	0/0
Chromosome2-2645983 INS	PRECISE	53	0	0	0/0	0/0	0/0	0/0	0/0	0/0	0/0	0/0	1/1	1/1	0/1	1/1	0/1	0/0
Chromosome2-2646543 INS	PRECISE	70	0	0	0/0	0/0	0/0	0/0	0/0	0/0	0/0	0/0	0/0	1/1	0/0	1/1	0/0	0/0
Chromosome2-2646834 INS	PRECISE	230	0	0.707	0/0	0/0	0/0	0/0	0/0	0/0	0/0	0/0	0/0	1/1	0/0	1/1	0/0	0/0
Chromosome2-2649317 DEL	PRECISE	72	0	0	0/0	0/0	0/0	0/0	0/0	0/0	0/0	0/0	0/0	1/1	0/0	0/1	0/0	0/0
Chromosome2-2649447 DEL	PRECISE	1020	0	0	0/0	0/0	0/0	0/0	0/0	0/0	0/0	0/0	0/0	1/1	0/0	0/1	0/0	0/0
Chromosome2-2651220 DEL	PRECISE	58	0	0	0/0	0/0	0/0	0/0	0/0	0/0	0/0	0/0	0/0	1/1	0/0	0/1	0/0	0/0
Chromosome2-2652353 DEL	PRECISE	5373	0	0	0/0	0/0	0/0	0/0	0/0	0/0	0/0	0/0	0/0	1/1	0/0	0/1	0/0	0/0
Chromosome2-2653139 DEL	PRECISE	7459	0	0	0/0	0/0	0/0	0/0	0/0	0/0	0/0	0/0	0/0	1/1	0/0	0/1	0/0	0/0
Chromosome2-2653160 INS	PRECISE	277	0	0	0/0	0/0	0/0	0/0	0/0	0/0	0/0	0/0	0/0	0/0	0/1	0/0	0/0	0/0
Chromosome2-2654456 DEL	PRECISE	1410	0	0	0/0	0/0	0/0	0/0	0/0	0/0	0/0	0/0	0/0	1/1	0/0	0/1	0/0	0/0
Chromosome2-2655763 DEL	PRECISE	22610	0	0	0/0	0/0	0/0	0/0	0/0	0/0	0/0	0/0	0/0	1/1	0/0	0/1	0/0	0/0
Chromosome2-2655763 DEL	PRECISE	3522	0	0	0/0	0/0	0/0	0/0	0/0	0/0	0/0	0/0	0/0	0/1	0/0	0/0	0/0	0/0
Chromosome2-2655802 BND	PRECISE	0	0	0	0/0	0/0	0/0	0/0	0/0	0/0	0/0	0/0	0/1	0/0	0/1	0/1	0/0	0/0
Chromosome2-2659866 INS	PRECISE	85	0	0	0/0	0/0	0/0	0/0	0/0	0/0	0/0	0/0	0/0	1/1	0/0	0/1	0/0	0/0
Chromosome2-2660092 INS	PRECISE	53	0	0	0/0	0/0	0/0	0/0	0/0	0/0	0/0	0/0	1/1	1/1	0/1	1/1	0/1	0/0
Chromosome2-2660593 INS	PRECISE	2322	0	0	0/0	0/0	0/0	0/0	0/0	0/0	0/0	0/0	0/0	0/0	0/1	0/0	0/0	0/0
Chromosome2-2664749 BND	PRECISE	0	0	0	0/0	0/1	0/0	0/0	0/0	0/0	0/0	0/0	0/0	0/0	0/0	0/0	0/0	0/1
Chromosome2-2667464 INS	PRECISE	2968	0	0	0/0	0/0	0/0	0/0	0/0	0/0	0/0	0/0	0/0	0/0	1/1	0/0	0/0	0/0
Chromosome2-2668622 DEL	PRECISE	698	0	0	0/0	0/0	0/0	0/0	0/0	0/0	0/0	0/0	1/1	1/1	0/1	1/1	0/1	0/0
Chromosome2-2669997 INS	PRECISE	99	0	0	0/0	0/0	0/0	0/0	0/0	0/0	0/0	0/0	0/0	0/0	0/1	0/0	0/0	0/0
Chromosome2-2675557 INS	PRECISE	482	0	0	0/0	0/0	0/0	0/0	0/0	0/0	0/0	0/0	0/0	1/1	0/0	0/1	0/0	0/0
Chromosome2-2675855 DEL	PRECISE	52	0	0	0/0	0/0	0/0	0/0	0/0	0/0	0/0	0/0	0/0	0/0	0/0	0/0	0/0	0/1
Chromosome2-2676191 DEL	PRECISE	71	0	0	0/0	0/0	0/0	0/0	0/0	0/0	0/0	0/0	0/0	0/0	0/0	0/0	0/0	0/1
Chromosome2-2679813 INS	PRECISE	211	0	0.707	0/0	0/0	0/0	0/0	0/0	0/0	0/0	0/0	0/0	1/1	0/0	0/1	0/0	0/0
Chromosome2-2680557 DEL	PRECISE	324	0	0	0/0	0/0	0/0	0/0	0/0	0/0	0/0	0/0	0/0	1/1	0/0	0/1	0/0	0/0
Chromosome2-2686849 DEL	PRECISE	92	0	0	0/0	0/0	0/0	0/0	0/0	0/0	0/0	0/0	0/0	1/1	0/0	0/1	0/0	0/0
Chromosome2-2688823 DEL	PRECISE	56	0	0	0/0	0/0	0/0	0/0	0/0	0/0	0/0	0/0	1/1	0/0	0/0	0/1	0/1	0/0
Chromosome2-2691450 INS	PRECISE	605	0	0.577	0/0	0/0	0/0	0/0	0/0	0/0	0/0	0/0	1/1	0/0	0/0	0/1	0/1	0/0
Chromosome2-2691891 DEL	PRECISE	5455	0	0	0/0	0/0	0/0	0/0	0/0	0/0	0/0	0/0	1/1	1/1	0/1	1/1	0/1	0/0
Chromosome2-2692591 DEL	PRECISE	276	0	0	0/0	0/0	0/0	0/0	0/0	0/0	0/0	0/0	0/0	1/1	0/0	0/1	0/0	0/0
Chromosome2-2693902 DEL	PRECISE	1394	0	0	0/0	0/0	0/0	0/0	0/0	0/0	0/0	0/0	0/0	1/1	0/0	0/1	0/0	0/0
Chromosome2-2695370 DEL	PRECISE	1405	0	0	0/0	0/0	0/0	0/0	0/0	0/0	0/0	0/0	0/0	1/1	0/0	0/1	0/0	0/0
Chromosome2-2698622 DEL	PRECISE	2219	0	0	0/0	0/0	0/0	0/0	0/0	0/0	0/0	0/0	0/0	0/1	0/0	0/1	0/0	0/0
Chromosome2-2698775 DEL	PRECISE	222	0.354	0.354	0/0	0/0	0/0	0/0	0/1	0/0	0/1	0/0	0/0	0/0	0/0	0/0	0/0	0/0
Chromosome2-2698850 DEL	PRECISE	1055	0	0	0/0	0/0	0/0	0/0	0/0	0/0	0/0	0/0	0/0	0/1	0/0	0/1	0/0	0/0
Chromosome2-2698958 DEL	PRECISE	930	0	0	0/0	0/0	0/0	0/0	0/0	0/0	0/0	0/0	0/0	0/1	0/0	0/1	0/0	0/0
Chromosome2-2699058 DEL	PRECISE	1335	0	0	0/0	0/0	0/0	0/0	0/0	0/0	0/0	0/0	0/0	0/1	0/0	0/1	0/0	0/0
Chromosome2-2702416 DEL	PRECISE	866	0	0	0/0	0/0	0/0	0/0	0/0	0/0	0/0	0/0	0/0	0/0	0/1	0/0	0/0	0/0
Chromosome2-2703162 DEL	PRECISE	834	0	0	0/0	0/0	0/0	0/0	0/0	0/0	0/0	0/0	0/0	1/1	0/0	0/1	0/0	0/0
Chromosome2-2703599 INS	PRECISE	1957	0	0	0/0	0/0	0/0	0/0	0/0	0/0	0/0	0/0	0/0	0/0	0/1	0/0	0/0	0/0
Chromosome2-2706322 BND	PRECISE	0	0	0	0/0	0/0	0/0	0/0	0/0	0/0	0/0	0/0	0/0	0/1	0/0	0/0	0/0	0/0
Chromosome2-2706322 BND	PRECISE	0	0	0	0/0	0/0	0/0	0/0	0/0	0/0	0/0	0/0	0/0	1/1	0/0	0/0	0/0	0/0
Chromosome2-2707514 INS	PRECISE	55	0	0.707	0/0	0/0	0/0	0/0	0/0	0/0	0/0	0/0	0/0	1/1	0/0	0/1	0/0	0/0

Chromosome2-2709365	DEL	PRECISE	71	0	0	0/0	0/0	0/0	0/0	0/0	0/0	0/0	0/0	0/0	0/0	0/1	0/0	0/0	0/0
Chromosome2-2709528	INS	PRECISE	1303	0	0.707	0/0	0/0	0/0	0/0	0/0	0/0	0/0	0/0	0/0	1/1	0/0	0/1	0/0	0/0
Chromosome2-2709790	INS	PRECISE	371	0	0	0/0	0/0	0/0	0/0	0/0	0/0	0/0	0/0	0/0	0/0	0/1	0/0	0/0	0/0
Chromosome2-2710404	INS	PRECISE	1603	0	0	0/0	0/0	0/0	0/0	0/0	0/0	0/0	0/0	0/0	0/0	0/1	0/0	0/0	0/0
Chromosome2-2710812	INS	PRECISE	3564	0	0	0/0	0/0	0/0	0/0	0/0	0/0	0/0	0/0	0/0	0/0	1/1	0/0	0/0	0/0
Chromosome2-2712255	DEL	PRECISE	132	0.447	0.447	0/0	0/0	0/0	0/0	0/0	0/0	0/0	0/0	1/1	1/1	0/1	1/1	0/0	0/0
Chromosome2-2712670	INS	PRECISE	612	0	0.707	0/0	0/0	0/0	0/0	0/0	0/0	0/0	0/0	0/0	1/1	0/0	0/1	0/0	0/0
Chromosome2-2712768	DEL	PRECISE	308	0	0	0/0	0/0	0/0	0/0	0/0	0/0	0/0	0/0	0/0	1/1	0/0	0/1	0/0	0/0
Chromosome2-2713067	DEL	PRECISE	1228	0	0	0/0	0/0	0/0	0/0	0/0	0/0	0/0	0/0	1/1	1/1	0/1	1/1	0/1	0/0
Chromosome2-2713298	DEL	PRECISE	712	0	0	0/0	0/0	0/0	0/0	0/0	0/0	0/0	0/0	0/0	0/0	0/1	0/0	0/0	0/0
Chromosome2-2713459	INS	PRECISE	131	0	0	0/0	0/0	0/0	0/0	0/0	0/0	0/0	0/0	0/0	0/0	0/1	0/0	0/0	0/0
Chromosome2-2713747	DEL	PRECISE	1448	0	0	0/0	0/0	0/0	0/0	0/0	0/0	0/0	0/0	1/1	1/1	0/0	1/1	0/1	0/0
Chromosome2-2714044	INS	PRECISE	278	0	0.577	0/0	0/0	0/0	0/0	0/0	0/0	0/0	0/0	0/0	1/1	0/1	0/1	0/0	0/0
Chromosome2-2714121	DEL	PRECISE	866	0	0	0/0	0/0	0/0	0/0	0/0	0/0	0/0	0/0	1/1	0/0	0/0	0/1	0/1	0/0
Chromosome2-2714226	DEL	PRECISE	161	0	0	0/0	0/0	0/0	0/0	0/0	0/0	0/0	0/0	1/1	1/1	0/1	1/1	0/1	0/0
Chromosome2-2714746	DEL	PRECISE	75	0	0	0/0	0/0	0/0	0/0	0/0	0/0	0/0	0/0	0/0	1/1	0/0	0/1	0/0	0/0
Chromosome2-2715332	INS	PRECISE	1364	0	0	0/0	0/0	0/0	0/0	0/0	0/0	0/0	0/0	0/0	0/0	0/1	0/0	0/0	0/0
Chromosome2-2715784	INS	PRECISE	57	0	0	0/0	0/0	0/0	0/0	0/0	0/0	0/0	0/0	1/1	1/1	0/0	1/1	0/1	0/0
Chromosome2-2716519	INS	PRECISE	60	0.707	0	0/0	0/0	0/0	0/0	0/0	0/0	0/0	0/0	0/0	1/1	0/0	0/1	0/0	0/0
Chromosome2-2718540	DEL	PRECISE	211	0	0	0/0	0/0	0/0	0/0	0/0	0/0	0/0	0/0	1/1	1/1	1/1	1/1	0/1	0/0
Chromosome2-2718998	INS	PRECISE	121	0	0	0/0	0/0	0/0	0/0	0/0	0/0	0/0	0/0	0/0	0/0	0/1	0/0	0/0	0/0
Chromosome2-2719325	DEL	PRECISE	146	0	0	0/0	0/0	0/0	0/0	0/0	0/0	0/0	0/0	0/0	1/1	0/0	0/1	0/0	0/0
Chromosome2-2720648	INS	PRECISE	755	0	0.707	0/0	0/0	0/0	0/0	0/0	0/0	0/0	0/0	0/0	1/1	0/0	0/1	0/0	0/0
Chromosome2-2721231	INS	PRECISE	100	0	0	0/0	0/0	0/0	0/0	0/0	0/0	0/0	0/0	1/1	1/1	0/1	1/1	0/1	0/0
Chromosome2-2722141	DEL	PRECISE	234	0	0	0/0	0/0	0/0	0/0	0/0	0/0	0/0	0/0	1/1	0/0	0/0	0/1	0/1	0/0
Chromosome2-2722790	DEL	PRECISE	1227	0	0	0/0	0/0	0/0	0/0	0/0	0/0	0/0	0/0	0/0	1/1	0/1	0/1	0/0	0/0
Chromosome2-2724802	INS	PRECISE	5440	0	0	0/0	0/0	0/0	0/0	0/0	0/0	0/0	0/0	0/0	0/0	0/1	0/0	0/0	0/0
Chromosome2-2725830	DEL	PRECISE	1354	0	0	0/0	0/0	0/0	0/0	0/0	0/0	0/0	0/0	0/0	1/1	0/0	0/1	0/0	0/0
Chromosome2-2726147	DEL	PRECISE	12310	0	0	0/0	0/0	0/0	0/0	0/0	0/0	0/0	0/0	1/1	1/1	0/0	1/1	0/1	0/0
Chromosome2-2728368	DEL	PRECISE	5237	0	0	0/0	0/0	0/0	0/0	0/0	0/0	0/0	0/0	0/0	1/1	0/0	0/1	0/0	0/0
Chromosome2-2728987	DEL	PRECISE	75	0	0	0/0	0/0	0/0	0/0	0/0	0/0	0/0	0/0	0/0	1/1	0/1	0/1	0/0	0/0
Chromosome2-2731563	DEL	PRECISE	61	0	0	0/0	0/0	0/0	0/0	0/0	0/0	0/0	0/0	0/0	0/0	0/1	0/0	0/0	0/0
Chromosome2-2732223	INS	PRECISE	50	0	0	0/0	0/0	0/0	0/0	0/0	0/0	0/0	0/0	1/1	0/0	0/0	0/1	0/1	0/0
Chromosome2-2734687	DEL	PRECISE	1470	0	0	0/0	0/0	0/0	0/0	0/0	0/0	0/0	0/0	0/0	1/1	0/1	0/1	0/0	0/0
Chromosome2-2735351	DEL	PRECISE	10657	0	0	0/0	0/0	0/0	0/0	0/0	0/0	0/0	0/0	0/1	1/1	0/1	1/1	0/1	0/0
Chromosome2-2735351	BND	PRECISE	0	0	0	0/0	0/0	0/0	0/0	0/0	0/0	0/0	0/0	0/1	0/0	0/0	0/0	0/0	0/0
Chromosome2-2735547	BND	PRECISE	0	0	0	0/0	0/0	0/0	0/0	0/0	0/0	0/0	0/0	0/0	0/1	0/0	0/0	0/0	0/0
Chromosome2-2736087	INS	PRECISE	156	0	0.535	0/0	0/0	1/1	1/1	1/1	0/0	0/0	0/0	1/1	0/1	0/1	0/1	0/0	0/0
Chromosome2-2738053	INS	PRECISE	6106	0	0	0/0	0/0	0/0	0/0	0/0	0/0	0/0	0/0	0/0	0/0	0/0	0/1	0/0	0/0
Chromosome2-2738157	INS	PRECISE	4972	0	0	0/0	0/0	0/0	0/0	0/0	0/0	0/0	0/0	0/0	0/0	0/0	0/1	0/0	0/0
Chromosome2-2738902	BND	PRECISE	0	0	0	0/0	0/0	0/0	0/0	0/0	0/0	0/0	0/0	0/1	0/0	0/0	0/1	0/0	0/0
Chromosome2-2739928	INS	PRECISE	778	0	0.707	0/0	0/0	0/0	0/0	0/0	0/0	0/0	0/0	0/0	1/1	0/0	0/1	0/0	0/0
Chromosome2-2741690	DEL	PRECISE	1302	0	0	0/0	0/0	0/0	0/0	0/0	0/0	0/0	0/0	0/0	1/1	0/1	1/1	0/0	0/0
Chromosome2-2742123	INS	PRECISE	1241	0	0.577	0/0	0/0	0/0	0/0	0/0	0/0	0/0	0/0	1/1	0/0	0/0	0/1	0/1	0/0
Chromosome2-2742691	DEL	PRECISE	328	0	0	0/0	0/0	0/0	0/0	0/0	0/0	0/0	0/0	0/0	1/1	0/0	0/1	0/0	0/0
Chromosome2-2742691	INS	PRECISE	5309	0	0	0/0	0/0	0/0	0/0	0/0	0/0	0/0	0/0	0/0	0/0	0/1	0/0	0/0	0/0
Chromosome2-2742779	INS	PRECISE	623	0	0	0/0	0/0	0/0	0/0	0/0	0/0	0/0	0/0	0/0	1/1	0/0	0/1	0/0	0/0
Chromosome2-2745145	INS	PRECISE	365	0	0	0/0	0/0	0/0	0/0	0/0	0/0	0/0	0/0	0/0	0/0	0/1	0/0	0/0	0/0
Chromosome2-2745258	INS	PRECISE	1942	0	0	0/0	0/0	0/0	0/0	0/0	0/0	0/0	0/0	0/0	0/0	0/1	0/0	0/0	0/0
Chromosome2-2745549	INS	PRECISE	64	0	0	0/0	0/0	0/0	0/0	0/0	0/0	0/0	0/0	0/0	0/0	0/1	0/0	0/0	0/0
Chromosome2-2746192	INS	PRECISE	7616	0	0	0/0	0/0	0/0	0/0	0/0	0/0	0/0	0/0	0/0	0/0	1/1	0/0	0/0	0/0
Chromosome2-2746383	BND	PRECISE	0	0	0	0/0	0/0	0/0	0/0	1/1	0/0	0/0	0/0	0/0	0/0	0/0	0/0	0/0	0/0
Chromosome2-2747279	INS	PRECISE	6281	0	0	0/0	0/0	0/0	0/0	0/0	0/0	0/0	0/0	0/0	0/0	0/0	0/0	0/0	0/1
Chromosome2-2748568	DEL	PRECISE	1016	0	0	0/0	0/0	0/0	0/0	0/0	0/0	0/0	0/0	1/1	1/1	0/1	1/1	0/1	0/0
Chromosome2-2748689	DEL	PRECISE	326	0	0	0/0	0/0	0/0	0/0	0/0	0/0	0/0	0/0	1/1	1/1	0/1	1/1	0/1	0/0
Chromosome2-2748811	BND	PRECISE	0	0	0	0/0	0/0	0/0	0/0	0/0	0/0	0/0	0/0	0/1	0/0	0/0	0/1	0/0	0/0
Chromosome2-2749613	DEL	PRECISE	101	0	0	0/0	0/0	0/0	0/0	0/0	0/0	0/0	0/0	1/1	0/0	0/0	0/1	0/1	0/0
Chromosome2-2749961	DEL	PRECISE	8689	0	0	0/0	0/0	0/0	0/0	0/0	0/0	0/0	0/0	0/0	1/1	0/0	0/1	0/0	0/0
Chromosome2-2749982	DEL	PRECISE	500	0	0	0/0	0/0	0/0	0/0	0/0	0/0	0/0	0/0	1/1	0/0	0/0	0/1	0/1	0/0
Chromosome2-2750585	INS	PRECISE	5729	0	0	0/0	0/0	0/0	0/0	0/0	0/0	0/0	0/0	0/0	0/0	0/1	0/0	0/0	0/0
Chromosome2-2751027	DEL	PRECISE	1338	0	0	0/0	0/0	0/0	0/0	0/0	0/0	0/0	0/0	1/1	1/1	0/1	1/1	0/1	0/0
Chromosome2-2751161	DEL	PRECISE	3138825	0	0	0/0	0/0	0/0	0/0	0/0	0/0	0/0	0/0	0/0	0/0	0/1	0/0	0/0	0/0
Chromosome2-2751840	DEL	PRECISE	46066	0	0	0/0	0/0	0/0	0/0	0/0	0/0	0/0	0/0	0/0	0/0	0/0	1/1	0/0	0/0
Chromosome2-2752265	DEL	PRECISE	128	0	0	0/0	0/0	0/0	0/0	0/0	0/0	0/0	0/0	1/1	0/0	0/1	1/1	0/1	0/0
Chromosome2-2753582	INS	PRECISE	1673	0	0	0/0	0/0	1/1	0/0	0/0	0/0	0/0	0/0	0/0	0/0	0/0	0/0	0/0	0/0
Chromosome2-2754413	DEL	PRECISE	300	0	0	0/0	0/0	1/1	0/0	0/0	0/0	0/0	0/0	0/0	0/0	0/0	0/0	0/0	0/0
Chromosome2-2754563	DEL	PRECISE	121	0	0	0/0	0/0	0/0	0/0	0/0	0/0	0/0	0/0	0/0	0/0	0/1	0/0	0/0	0/0
Chromosome2-2755029	DEL	PRECISE	55	0	0	0/0	0/0	0/0	0/0	0/0	0/0	0/0	0/0	0/0	0/0	0/1	0/0	0/0	0/0
Chromosome2-2755593	INS	PRECISE	1289	0	0.577	0/0	0/0	0/0	0/0	0/0	0/0	0/0	0/0	1/1	0/0	0/0	1/1	0/1	0/0
Chromosome2-2756652	DEL	PRECISE	3636	0	0	0/0	0/0	0/0	0/0	0/0	0/0	0/0	0/0	0/0	0/0	0/1	0/0	0/0	0/0
Chromosome2-2756863	DEL	PRECISE	8222	0	0	0/0	0/0	0/0	0/0	0/0	0/0	0/0	0/0	0/0	1/1	0/0	1/1	0/0	0/0
Chromosome2-2757685	INV	PRECISE	3935	0	0	0/0	0/0	0/0	0/0	0/0	0/0	0/0	0/0	0/0	0/0	0/0	0/1	0/0	0/0
Chromosome2-2758721	DEL	PRECISE	360	0	0	0/0	0/0	0/0	0/0	0/0	0/0	0/0	0/0	0/0	0/0	0/1	0/0	0/0	0/0

Chromosome2-2758777	INS	PRECISE	12536	0	0	0/0	0/0	0/0	0/0	0/0	0/0	0/0	0/0	0/0	0/0	1/1	0/0	0/0	0/0
Chromosome2-2761207	INS	PRECISE	1408	0	0	0/0	0/0	1/1	0/0	0/0	0/0	0/0	0/0	0/0	0/0	0/0	0/0	0/0	0/0
Chromosome2-2761638	INS	PRECISE	211	0	0	0/0	0/0	0/0	0/0	0/0	0/0	0/0	0/0	1/1	0/0	0/0	0/1	0/1	0/0
Chromosome2-2761802	DEL	PRECISE	54	0	0	0/0	0/0	0/0	0/0	0/0	0/0	0/0	0/0	0/0	0/0	0/1	0/0	0/0	0/0
Chromosome2-2762081	INS	PRECISE	3788	0	0.577	0/0	0/0	0/0	0/0	0/0	0/0	0/0	0/0	1/1	0/0	0/0	1/1	1/1	0/0
Chromosome2-2762433	INS	PRECISE	456	0	0	0/0	0/0	0/0	0/0	0/0	0/0	0/0	0/0	0/0	1/1	0/0	0/1	0/0	0/0
Chromosome2-2763199	DEL	PRECISE	84	0.408	0.408	0/0	0/0	0/0	0/0	1/1	0/0	1/1	1/1	0/1	0/0	0/0	0/0	0/0	0/0
Chromosome2-2764573	DEL	PRECISE	74	0	0	0/0	0/0	0/0	0/0	0/0	0/0	0/0	0/0	1/1	0/0	0/0	0/1	0/1	0/0
Chromosome2-2764957	INS	PRECISE	55	0	0	0/0	0/0	0/0	0/0	0/0	0/0	0/0	0/0	1/1	1/1	0/1	1/1	0/1	0/0
Chromosome2-2767184	DEL	PRECISE	101	0	0	0/0	0/0	0/0	0/0	0/0	0/0	0/0	0/0	0/0	0/0	0/0	0/1	0/0	0/0
Chromosome2-2767309	DEL	PRECISE	85	0	0	0/0	0/0	0/0	0/0	0/0	0/0	0/0	0/0	0/0	0/0	0/0	0/1	0/0	0/0
Chromosome2-2767317	DEL	PRECISE	12438	0	0	0/0	0/0	0/0	0/0	0/0	0/0	0/0	0/0	1/1	0/1	0/1	1/1	0/1	0/0
Chromosome2-2768561	DEL	PRECISE	613	0.707	0.707	0/0	0/0	0/0	0/0	0/0	0/0	0/0	0/0	0/1	0/1	0/0	0/0	0/0	0/0
Chromosome2-2768561	INS	PRECISE	1546	0	0	0/0	0/0	0/0	0/0	0/0	0/0	0/0	0/0	0/0	0/0	0/0	0/1	0/0	0/0
Chromosome2-2768945	DUP	PRECISE	3459630	0	0	0/0	0/0	0/0	0/0	0/0	0/0	0/0	0/0	0/1	0/1	0/0	1/1	0/1	0/1
Chromosome2-2769605	DEL	PRECISE	694	0	0	0/0	0/0	0/0	0/0	0/0	0/0	0/0	0/0	1/1	0/1	0/0	0/1	0/1	0/0
Chromosome2-2769958	DEL	PRECISE	115	0.5	0.5	0/0	0/0	0/0	0/0	0/0	0/0	0/0	0/0	1/1	0/1	0/0	0/1	0/1	0/0
Chromosome2-2770414	INS	PRECISE	67	0	0	0/0	0/0	0/0	0/0	0/0	0/0	0/0	0/0	0/0	0/0	0/0	0/1	0/0	0/0
Chromosome2-2771075	DEL	PRECISE	754	0	0	0/0	0/0	0/0	0/0	0/0	0/0	0/0	0/0	1/1	0/0	0/0	0/1	0/1	0/0
Chromosome2-2771627	INS	PRECISE	63	0.976	0	0/0	0/0	1/1	0/0	0/0	0/0	0/0	0/0	1/1	1/1	0/1	1/1	0/1	0/1
Chromosome2-2771941	DEL	PRECISE	85	0	0	0/0	0/0	0/0	0/0	0/0	0/0	0/0	0/0	0/0	0/0	0/1	0/0	0/0	0/0
Chromosome2-2772515	DEL	PRECISE	8129	0	0	0/0	0/0	0/0	0/0	0/0	0/0	0/0	0/0	0/0	0/0	0/1	0/0	0/0	0/0
Chromosome2-2773327	INS	PRECISE	13556	0	0	0/0	0/0	0/0	0/0	0/0	0/0	0/0	0/0	0/0	0/0	0/1	0/0	0/0	0/0
Chromosome2-2773414	INS	PRECISE	996	0	0	0/0	0/0	0/0	0/0	0/0	0/0	0/0	0/0	0/0	0/0	0/1	0/0	0/0	0/0
Chromosome2-2774298	DEL	PRECISE	1529	0	0	0/0	0/0	0/0	0/0	0/0	0/0	0/0	0/0	1/1	1/1	0/1	1/1	0/1	0/0
Chromosome2-2774613	INS	PRECISE	115	0	0.516	0/0	0/0	0/0	0/0	0/0	0/0	0/0	0/0	1/1	1/1	0/0	1/1	0/1	0/1
Chromosome2-2775284	INS	PRECISE	111	0.667	0.333	0/0	0/0	0/0	1/1	1/1	0/0	0/0	0/0	1/1	1/1	0/1	1/1	0/1	0/1
Chromosome2-2775688	DEL	PRECISE	19699	0.707	0.707	0/0	0/0	0/0	0/0	0/0	0/0	0/0	0/0	0/0	1/1	0/0	0/1	0/0	0/0
Chromosome2-2775722	DEL	PRECISE	952	0	0	0/0	0/0	0/0	0/0	0/0	0/0	0/0	0/0	0/0	0/0	0/1	0/0	0/0	0/0
Chromosome2-2776520	INS	PRECISE	849	0	0.957	0/0	0/0	0/0	0/0	0/0	0/0	0/0	0/0	1/1	0/0	0/1	0/1	0/1	0/0
Chromosome2-2777217	INS	PRECISE	58	0	0	0/0	0/0	0/0	0/0	0/0	0/0	0/0	0/0	1/1	0/0	0/0	0/1	0/1	0/0
Chromosome2-2777710	DEL	PRECISE	57	0	0	0/0	0/0	0/0	0/0	0/0	0/0	0/0	0/0	1/1	0/0	0/0	0/1	0/1	0/0
Chromosome2-2777790	INS	PRECISE	83	0	0	0/0	0/0	0/0	0/0	0/0	0/0	0/0	0/0	0/0	0/0	0/1	0/0	0/0	0/0
Chromosome2-2777828	DEL	PRECISE	98	0	0	0/0	0/0	0/0	0/0	0/0	0/0	0/0	0/0	0/0	0/0	0/1	0/0	0/0	0/0
Chromosome2-2778015	DEL	PRECISE	2694	0	0	0/0	0/0	0/0	0/0	0/0	0/0	0/0	0/0	0/0	1/1	0/0	0/1	0/0	0/0
Chromosome2-2778103	DEL	PRECISE	113	0	0	0/0	0/0	0/0	0/0	0/0	0/0	0/0	0/0	1/1	0/0	0/0	0/1	0/1	0/0
Chromosome2-2778499	BND	PRECISE	0	0	0	0/0	0/0	0/0	0/0	0/0	0/0	0/0	0/0	0/0	0/0	0/0	0/0	0/0	0/1
Chromosome2-2778917	INS	PRECISE	5212	0	0	0/0	0/0	0/0	0/0	0/0	0/0	0/0	0/0	0/0	0/0	0/0	0/0	0/0	0/1
Chromosome2-2780725	DEL	PRECISE	12358	0.548	0	0/0	0/0	0/0	0/0	0/0	0/0	0/0	0/0	1/1	0/1	0/1	1/1	0/1	0/0
Chromosome2-2780725	BND	PRECISE	0	0	0	0/0	0/0	0/0	0/0	1/1	0/0	1/1	1/1	0/1	0/1	0/0	0/1	0/0	0/1
Chromosome2-2781990	DEL	PRECISE	53	0	0	0/0	0/0	0/0	0/0	0/0	0/0	0/0	0/0	1/1	0/0	0/0	0/1	0/1	0/0
Chromosome2-2782956	DEL	PRECISE	420	0	0	0/0	0/0	0/0	0/0	0/0	0/0	0/0	0/0	1/1	0/0	0/0	0/1	0/1	0/0
Chromosome2-2783048	DEL	PRECISE	79	0	0	0/0	0/0	0/0	0/0	0/0	0/0	0/0	0/0	1/1	0/0	0/0	0/1	0/1	0/0
Chromosome2-2783101	BND	PRECISE	0	0	0	0/0	0/0	0/0	0/1	0/0	0/0	0/0	0/0	0/0	0/0	0/0	0/0	0/0	0/0
Chromosome2-2783277	DEL	PRECISE	87	0	0	0/0	0/0	0/0	0/1	0/0	0/0	0/0	0/0	0/0	0/0	0/0	0/0	0/0	0/0
Chromosome2-2783511	INS	PRECISE	216	0	0	0/0	0/0	0/0	0/1	0/0	0/0	0/0	0/0	0/0	0/1	0/1	0/0	0/0	0/0
Chromosome2-2784086	DEL	PRECISE	66	0	0	0/0	0/0	0/0	0/1	0/0	0/0	0/0	0/0	0/0	0/1	0/1	0/0	0/0	0/0
Chromosome2-2784390	INS	PRECISE	10168	0	0	0/0	0/0	0/0	0/0	0/0	0/0	0/0	0/0	0/0	0/0	0/0	0/1	0/0	0/0
Chromosome2-2784391	BND	PRECISE	0	0	0	0/0	0/0	0/0	0/0	0/0	0/0	0/0	0/0	1/1	0/0	0/0	0/1	0/0	0/0
Chromosome2-2784909	DEL	PRECISE	24911	0	0	0/0	0/0	0/0	0/0	0/0	0/0	0/0	0/0	0/0	1/1	0/0	1/1	0/0	0/0
Chromosome2-2788497	DEL	PRECISE	2562	0	0	0/0	0/0	0/0	0/0	0/0	0/0	0/0	0/0	0/0	0/0	0/1	0/0	0/0	0/0
Chromosome2-2788927	INS	PRECISE	5021	0	0	0/0	0/0	0/0	0/0	0/0	0/0	0/0	0/0	0/0	0/0	0/1	0/0	0/0	0/0
Chromosome2-2788927	BND	PRECISE	0	0	0	0/0	0/0	0/0	0/0	0/0	0/0	0/0	0/0	0/0	0/0	0/1	0/0	0/0	0/0
Chromosome2-2789389	INS	PRECISE	623	0	0	0/0	0/0	0/0	0/0	0/0	0/0	0/0	0/0	0/0	0/0	0/1	0/0	0/0	0/0
Chromosome2-2789815	INS	PRECISE	12425	0	0	0/0	0/0	0/0	0/0	0/0	0/0	0/0	0/0	0/0	0/0	0/0	0/0	0/1	0/0
Chromosome2-2789860	DEL	PRECISE	261	0	0	0/0	0/0	0/0	0/0	0/0	0/0	0/0	0/0	1/1	0/0	0/0	0/1	0/1	0/0
Chromosome2-2791058	INS	PRECISE	55	0	0	0/0	0/0	0/0	0/0	0/0	0/0	0/0	0/0	0/0	0/0	0/1	0/0	0/0	0/0
Chromosome2-2791240	DEL	PRECISE	60	0	0	0/0	0/0	0/0	0/0	0/0	0/0	0/0	0/0	0/0	1/1	0/0	0/1	0/0	0/0
Chromosome2-2791706	INS	PRECISE	74	0	0.577	0/0	0/0	0/0	0/0	0/0	0/0	0/0	0/0	1/1	0/0	0/0	0/1	0/1	0/0
Chromosome2-2793114	DEL	PRECISE	9848	0.577	0	0/0	0/0	0/0	0/0	0/0	0/0	0/0	0/0	1/1	0/0	0/0	0/1	0/1	0/0
Chromosome2-2794188	INS	PRECISE	12461	0	0	0/0	0/0	0/0	0/0	0/0	0/0	0/0	0/0	0/0	0/0	0/1	0/0	0/0	0/0
Chromosome2-2794859	INS	PRECISE	1354	0	0.577	0/0	0/0	0/0	0/0	0/0	0/0	0/0	0/0	1/1	0/0	0/0	0/1	0/1	0/0
Chromosome2-2795912	DEL	PRECISE	51	0	0	0/0	0/0	0/0	0/0	0/0	0/0	0/0	0/0	0/0	1/1	0/0	0/1	0/0	0/0
Chromosome2-2796049	DEL	PRECISE	153	0	0	0/0	0/0	0/0	0/0	0/0	0/0	0/0	0/0	0/0	0/0	0/1	0/0	0/0	0/0
Chromosome2-2796514	DEL	PRECISE	95	0	0	0/0	0/0	0/0	0/0	0/0	0/0	0/0	0/0	0/0	1/1	0/0	0/1	0/0	0/0
Chromosome2-2798647	INS	PRECISE	9579	0	0	0/0	0/0	0/0	0/0	0/0	0/0	0/0	0/0	0/0	0/0	0/0	0/1	0/0	0/0
Chromosome2-2798648	BND	PRECISE	0	0	0	0/0	0/0	0/0	0/0	0/0	0/0	0/0	0/0	0/0	0/0	0/0	1/1	0/1	0/0
Chromosome2-2798706	DEL	PRECISE	10615	0	0.707	0/0	0/0	0/0	0/0	0/0	0/0	0/0	0/0	0/0	0/0	0/0	0/0	0/0	0/1
Chromosome2-2799513	INS	PRECISE	245	0	0.5	0/0	0/0	0/0	0/0	0/0	0/0	0/0	0/0	0/0	1/1	0/1	0/1	0/0	0/0
Chromosome2-2799803	DEL	PRECISE	152	0	0	0/0	0/0	0/0	0/0	0/0	0/0	0/0	0/0	0/0	1/1	0/0	0/1	0/0	0/0
Chromosome2-2799914	BND	PRECISE	0	0	0	0/0	0/0	0/0	0/0	0/0	0/0	0/0	0/0	0/0	0/0	0/0	0/0	0/0	0/1
Chromosome2-2799915	DEL	PRECISE	12832	0	0	0/0	0/0	0/0	0/0	0/0	0/0	0/0	0/0	1/1	0/0	0/0	0/1	0/1	0/0
Chromosome2-2800453	DEL	PRECISE	7264	0	0	0/0	0/0	0/0	0/0	0/0	0/0	0/0	0/0	0/0	0/0	0/1	0/0	0/0	0/0

Chromosome2-2802191 DEL	PRECISE	56	0	0	0/0	0/0	0/0	0/0	0/0	0/0	0/0	0/0	1/1	0/0	0/0	0/1	0/1	0/0
Chromosome2-2804801 DEL	PRECISE	60	0	0	0/0	0/0	0/0	0/0	0/0	0/0	0/0	0/0	0/0	1/1	0/0	0/1	0/0	0/0
Chromosome2-2805957 INS	PRECISE	7795	0	0	0/0	0/0	0/0	0/0	0/0	0/0	0/0	0/0	0/0	0/0	0/0	0/1	0/0	0/0
Chromosome2-2806536 DEL	PRECISE	297	0	0	0/0	0/0	0/0	0/0	0/0	0/0	0/0	0/0	0/0	1/1	0/1	0/1	0/0	0/0
Chromosome2-2807342 DEL	PRECISE	78	0	0	0/0	0/0	0/0	0/0	0/0	0/0	0/0	0/0	0/0	1/1	0/1	0/1	0/0	0/0
Chromosome2-2812412 INS	PRECISE	1389	0	0	0/0	0/0	0/0	0/0	0/0	0/0	0/0	0/0	0/0	0/0	0/1	0/0	0/0	0/0
Chromosome2-2813987 INS	PRECISE	5223	0	0	0/0	0/0	0/0	0/0	0/0	0/0	0/0	0/0	0/0	0/0	1/1	0/0	0/0	0/0
Chromosome2-2814390 DEL	PRECISE	201	0	0	0/0	0/0	0/0	0/0	0/0	0/0	0/0	0/0	0/0	1/1	0/0	0/1	0/0	0/0
Chromosome2-2814409 DEL	PRECISE	1936	0.5	0.5	0/0	0/0	0/0	0/0	0/0	0/0	0/0	0/0	1/1	0/0	0/1	0/1	0/1	0/0
Chromosome2-2814421 INS	PRECISE	1365	0	0	0/0	0/0	0/0	0/0	0/0	0/0	0/0	0/0	0/0	1/1	0/0	0/1	0/0	0/0
Chromosome2-2814603 INS	PRECISE	194	0	0	0/0	0/0	0/0	0/0	0/0	0/0	0/0	0/0	0/0	1/1	0/0	0/1	0/0	0/0
Chromosome2-2815474 DEL	PRECISE	3567	0	0	0/0	0/0	0/0	0/0	0/0	0/0	0/0	0/0	0/0	0/0	0/1	0/0	0/0	0/0
Chromosome2-2816417 DEL	PRECISE	228	0	0	0/0	0/0	0/0	0/0	0/0	0/0	0/0	0/0	0/0	0/0	0/1	0/0	0/0	0/0
Chromosome2-2816604 INS	PRECISE	12311	0	0	0/0	0/0	0/0	0/0	0/0	0/0	0/0	0/0	0/0	0/0	1/1	0/0	0/0	0/0
Chromosome2-2817242 INS	PRECISE	50	0	0	0/0	0/0	0/0	0/0	0/0	0/0	0/0	0/0	0/0	0/0	0/1	0/0	0/0	0/0
Chromosome2-2817963 INS	PRECISE	150	0	0.577	0/0	0/0	0/0	0/0	0/0	0/0	0/0	0/0	1/1	0/0	0/0	0/1	0/1	0/0
Chromosome2-2818065 DEL	PRECISE	637	0	0	0/0	0/0	0/0	0/0	0/0	0/0	0/0	0/0	1/1	0/0	0/0	0/1	0/1	0/0
Chromosome2-2818880 INS	PRECISE	235	0	0.577	0/0	0/0	0/0	0/0	0/0	0/0	0/0	0/0	1/1	0/0	0/0	0/1	0/1	0/0
Chromosome2-2819229 INS	PRECISE	252	0	0.707	0/0	0/0	0/0	0/0	0/0	0/0	0/0	0/0	1/1	1/1	0/1	1/1	0/1	0/0
Chromosome2-2819476 DEL	PRECISE	53	0	0	0/0	0/0	0/0	0/0	0/0	0/0	0/0	0/0	1/1	0/0	0/0	1/1	0/1	0/0
Chromosome2-2819673 DEL	PRECISE	4106	0	0	0/0	0/0	0/0	0/0	0/0	0/0	0/0	0/0	1/1	0/0	0/0	1/1	0/1	0/0
Chromosome2-2820632 DEL	PRECISE	622	0	0	0/0	0/0	0/0	0/0	0/0	0/0	0/0	0/0	1/1	1/1	0/1	1/1	0/1	0/0
Chromosome2-2820946 DEL	PRECISE	14811	0	0	0/0	0/0	0/0	0/0	0/0	0/0	0/0	0/0	0/0	1/1	0/0	0/0	0/0	0/0
Chromosome2-2821163 INS	PRECISE	974	0	0	0/0	0/0	0/0	0/0	0/0	0/0	0/0	0/0	0/0	0/0	0/1	0/0	0/0	0/0
Chromosome2-2821224 DEL	PRECISE	7372	0	0	0/0	0/0	0/0	0/0	0/0	0/0	0/0	0/0	1/1	0/0	0/1	1/1	0/1	0/0
Chromosome2-2822904 DEL	PRECISE	161	0	0	0/0	0/0	0/0	0/0	0/0	0/0	0/0	0/0	0/0	1/1	0/0	0/0	0/0	0/0
Chromosome2-2823180 INS	PRECISE	6451	0	0	0/0	0/0	0/0	0/0	0/0	0/0	0/0	0/0	0/0	0/0	1/1	0/0	0/0	0/0
Chromosome2-2823455 BND	PRECISE	0	0	0	0/0	0/0	0/0	0/0	0/0	0/0	0/0	0/0	0/0	0/0	0/1	0/0	0/0	0/0
Chromosome2-2824461 DEL	PRECISE	474	0	0	0/0	0/0	0/0	0/0	0/0	0/0	0/0	0/0	0/0	0/0	0/1	0/0	0/0	0/0
Chromosome2-2824537 INS	PRECISE	5049	0	0	0/0	0/0	0/0	0/0	0/0	0/0	0/0	0/0	0/0	0/0	1/1	0/0	0/0	0/0
Chromosome2-2825736 INS	PRECISE	1368	0	0	0/0	0/0	0/0	0/0	0/0	0/0	0/0	0/0	0/0	0/0	0/1	0/0	0/0	0/0
Chromosome2-2825794 DEL	PRECISE	5260	0	0	0/0	0/0	0/0	0/0	0/0	0/0	0/0	0/0	1/1	0/0	0/0	0/1	0/0	0/0
Chromosome2-2826337 DEL	PRECISE	2291	0	0	0/0	0/0	0/0	0/0	0/0	0/0	0/0	0/0	1/1	0/0	0/0	1/1	0/0	0/0
Chromosome2-2826575 DEL	PRECISE	211	0	0	0/0	0/0	0/0	0/0	0/0	0/0	0/0	0/0	1/1	0/0	0/0	1/1	0/0	0/0
Chromosome2-2826603 DEL	PRECISE	1070	0	0	0/0	0/0	0/0	0/0	0/0	0/0	0/0	0/0	1/1	0/0	0/0	1/1	0/0	0/0
Chromosome2-2826717 DEL	PRECISE	159	0	0	0/0	0/0	0/0	0/0	0/0	0/0	0/0	0/0	1/1	0/0	0/0	0/1	0/0	0/0
Chromosome2-2826739 DEL	PRECISE	3058	0	0	0/0	0/0	0/0	0/0	0/0	0/0	0/0	0/0	1/1	0/0	0/0	0/1	0/0	0/0
Chromosome2-2831560 INS	PRECISE	77	0	0	0/0	0/0	0/0	0/0	0/0	0/0	0/0	0/0	0/0	0/0	0/1	0/0	0/0	0/0
Chromosome2-2832116 DEL	PRECISE	677	0	0	0/0	0/0	0/0	0/0	0/0	0/0	0/0	0/0	1/1	0/0	0/0	1/1	0/1	0/0
Chromosome2-2833477 DEL	PRECISE	61	0	0	0/0	0/0	0/0	0/0	0/0	0/0	0/0	0/0	0/1	0/0	0/0	0/1	0/0	0/0
Chromosome2-2833783 DEL	PRECISE	5291	0	0	0/0	0/0	0/0	0/0	0/0	0/0	0/0	0/0	0/1	0/0	0/0	0/1	0/0	0/0
Chromosome2-2834134 INS	PRECISE	12495	0	0	0/0	0/0	0/0	0/0	0/0	0/0	0/0	0/0	0/0	0/0	0/0	0/0	0/0	0/0
Chromosome2-2838388 DEL	PRECISE	133	0	0	0/0	0/0	0/0	0/0	0/0	0/0	0/0	0/0	1/1	0/0	0/0	0/1	0/1	0/0
Chromosome2-2838712 INS	PRECISE	623	0	0	0/0	0/0	0/0	0/0	0/0	0/0	0/0	0/0	0/0	0/0	0/1	0/0	0/0	0/0
Chromosome2-2839630 INS	PRECISE	1376	0	0.577	0/0	0/0	0/0	0/0	0/0	0/0	0/0	0/0	1/1	0/0	0/0	0/1	0/1	0/0
Chromosome2-2843973 DEL	PRECISE	1213	0	0	0/0	0/0	0/0	0/0	0/0	0/0	0/0	0/0	1/1	0/0	0/0	0/1	0/1	0/0
Chromosome2-2844913 INS	PRECISE	137	0	0	0/0	0/0	0/0	0/0	0/0	0/0	0/0	0/0	0/0	1/1	0/0	0/1	0/0	0/0
Chromosome2-2844939 INS	PRECISE	1550	0	0.707	0/0	0/0	0/0	0/0	0/0	0/0	0/0	0/0	0/0	1/1	0/0	0/1	0/0	0/0
Chromosome2-2845895 INS	PRECISE	393	0	0.577	0/0	0/0	0/0	0/0	0/0	0/0	0/0	0/0	1/1	0/0	0/0	0/1	0/1	0/0
Chromosome2-2849143 INS	PRECISE	183	0	0.577	0/0	0/0	0/0	0/0	0/0	0/0	0/0	0/0	1/1	0/0	0/0	0/1	0/1	0/0
Chromosome2-2850382 DEL	PRECISE	183	0	0	0/0	0/0	0/0	0/0	0/0	0/0	0/0	0/0	1/1	0/0	0/0	0/1	0/0	0/0
Chromosome2-2852001 INS	PRECISE	1367	0	0	0/0	0/0	0/0	0/0	0/0	0/0	0/0	0/0	0/0	0/0	0/1	0/0	0/0	0/0
Chromosome2-2853147 DEL	PRECISE	1417	0	0	0/0	0/0	0/0	0/0	0/0	0/0	0/0	0/0	0/0	1/1	0/1	0/1	0/0	0/0
Chromosome2-2854579 DUP	PRECISE	697998	0	0	0/0	0/0	0/0	0/0	0/0	0/0	0/0	0/0	0/0	0/1	0/0	0/0	0/0	0/0
Chromosome2-2855880 INS	PRECISE	211	0	0	0/0	0/0	0/0	0/0	0/0	0/0	0/0	0/0	1/1	0/0	0/0	0/1	0/0	0/0
Chromosome2-2856504 INS	PRECISE	535	0	0	0/0	0/0	0/0	0/0	0/0	0/0	1/1	0/0	0/0	0/0	0/0	0/0	0/0	0/0
Chromosome2-2857044 DEL	PRECISE	122	0	0	0/0	0/0	0/0	0/0	0/0	0/0	0/0	0/0	0/0	0/0	0/1	0/0	0/0	0/0
Chromosome2-2857987 INS	PRECISE	626	0	0.577	0/0	0/0	0/0	0/0	0/0	0/0	0/0	0/0	1/1	0/0	0/0	0/1	0/1	0/0
Chromosome2-2858558 INS	PRECISE	76	0	0.707	0/0	0/0	0/0	0/0	0/0	0/0	0/0	0/0	0/0	1/1	0/0	0/1	0/0	0/0
Chromosome2-2858796 DEL	PRECISE	1356	0	0	0/0	0/0	0/0	0/0	0/0	0/0	0/0	0/0	1/1	0/0	0/0	0/1	0/1	0/0
Chromosome2-2860806 INS	PRECISE	888	0	0	0/0	0/0	0/0	0/0	0/0	0/0	0/0	0/0	1/1	0/0	0/0	0/1	0/1	0/0
Chromosome2-2862647 INS	PRECISE	3444	0	0	0/0	0/0	0/0	0/0	0/0	0/0	0/0	0/0	0/0	0/0	0/1	0/0	0/0	0/0
Chromosome2-2863701 INS	PRECISE	360	0	0.707	0/0	0/0	0/0	0/0	0/0	0/0	0/0	0/0	0/0	1/1	0/0	0/1	0/0	0/0
Chromosome2-2863922 DEL	PRECISE	70	0	0	0/0	0/0	0/0	0/0	0/0	0/0	0/0	0/0	0/0	1/1	0/0	0/1	0/0	0/0
Chromosome2-2864063 INS	PRECISE	1493	0	0	0/0	0/0	0/0	0/0	0/0	0/0	0/0	0/0	0/0	0/0	0/1	0/0	0/0	0/0
Chromosome2-2865910 DEL	PRECISE	79	0	0	0/0	0/0	0/0	0/0	0/0	0/0	0/0	0/0	1/1	0/0	0/0	0/1	0/1	0/0
Chromosome2-2865923 DEL	PRECISE	4106	0	0	0/0	0/0	0/0	0/0	0/0	0/0	0/0	0/0	1/1	0/0	0/0	0/1	0/1	0/0
Chromosome2-2868758 DEL	PRECISE	91	0	0	0/0	0/0	0/0	0/0	0/0	0/0	0/0	0/0	1/1	1/1	0/0	1/1	0/1	0/0
Chromosome2-2869512 DEL	PRECISE	50	0	0	0/0	0/0	0/0	0/0	0/0	0/0	0/0	0/0	1/1	0/0	0/0	1/1	0/0	0/0
Chromosome2-2871984 DEL	PRECISE	848	0	0	0/0	0/0	0/0	0/0	0/0	0/0	0/0	0/0	1/1	0/0	0/0	0/1	0/1	0/0
Chromosome2-2872059 DEL	PRECISE	3404	0	0	0/0	0/0	0/0	0/0	0/0	0/0	0/0	0/0	0/0	0/0	0/1	0/0	0/0	0/0
Chromosome2-2873064 INS	PRECISE	875	0	0	0/0	0/0	0/0	0/0	0/0	0/0	0/0	0/0	0/0	0/0	0/1	0/0	0/0	0/0
Chromosome2-2874373 INS	PRECISE	161	0	0	0/0	0/0	0/0	0/0	0/0	0/0	0/0	0/0	0/0	1/1	0/0	0/1	0/0	0/0

Chromosome2-2874828	DEL	PRECISE	239	0	0	0/0	0/0	0/0	0/0	0/0	0/0	0/0	0/0	0/0	1/1	0/0	0/1	0/0	0/0
Chromosome2-2875396	INS	PRECISE	600	0	0.577	0/0	0/0	0/0	0/0	0/0	0/0	0/0	0/0	1/1	0/0	0/0	0/1	0/1	0/0
Chromosome2-2876194	DEL	PRECISE	1551	0	0	0/0	0/0	0/0	0/0	0/0	0/0	0/0	0/0	1/1	1/1	0/1	1/1	0/1	0/0
Chromosome2-2877128	BND	PRECISE	0	0	0	0/0	0/0	0/0	0/0	0/0	0/0	0/1	0/0	0/0	0/0	0/0	0/0	0/0	0/0
Chromosome2-2878080	INS	PRECISE	56	0	0	0/0	0/0	0/0	0/0	0/0	0/0	0/0	0/0	0/0	1/1	0/0	0/1	0/0	0/0
Chromosome2-2879460	DEL	PRECISE	54	0	0	0/0	0/0	0/0	0/0	0/0	1/1	1/1	1/1	0/0	0/0	0/0	0/0	0/0	0/0
Chromosome2-2879627	DEL	PRECISE	150	0	0	0/0	0/0	1/1	1/1	1/1	1/1	1/1	1/1	0/0	0/0	0/0	0/0	0/0	0/0
Chromosome2-2879631	INS	PRECISE	76	0	0	0/0	0/0	0/0	0/0	0/0	0/0	0/0	0/0	0/0	1/1	0/0	0/1	0/0	0/0
Chromosome2-2879891	INS	PRECISE	826	0	0.957	0/0	0/0	0/0	0/0	0/0	0/0	0/0	0/0	1/1	1/1	0/0	1/1	0/1	0/0
Chromosome2-2879910	INS	PRECISE	12964	0	0	0/0	0/0	0/0	0/0	0/0	0/0	0/0	0/0	0/0	0/0	0/0	0/0	0/0	0/0
Chromosome2-2880446	DEL	PRECISE	9735	0	0	0/0	0/0	0/0	0/0	0/0	0/0	0/0	0/0	0/0	0/0	0/1	0/0	0/0	0/0
Chromosome2-2881155	DEL	PRECISE	193	0	0	0/0	0/0	1/1	0/0	0/0	0/0	0/0	0/0	0/0	0/0	0/0	0/0	0/0	0/0
Chromosome2-2881284	INS	PRECISE	991	0	0	0/0	0/0	0/0	0/0	0/0	0/0	0/0	0/0	0/0	0/0	0/0	0/0	0/0	0/1
Chromosome2-2881391	DEL	PRECISE	57	0	0	0/0	0/0	1/1	0/0	0/0	0/0	0/0	0/0	0/0	0/0	0/0	0/0	0/0	0/0
Chromosome2-2882976	INS	PRECISE	4959	0	0	0/0	0/0	0/0	0/0	0/0	0/0	0/0	0/0	0/0	0/0	1/1	0/0	0/0	0/0
Chromosome2-2883143	INS	PRECISE	55	0	0	0/0	0/0	0/0	0/0	0/0	0/0	0/0	0/0	0/0	0/0	0/1	0/0	0/0	0/0
Chromosome2-2883275	INS	PRECISE	278	0	0	0/0	0/0	0/0	0/0	0/0	0/0	0/0	0/0	0/0	1/1	0/0	0/1	0/0	0/0
Chromosome2-2886174	DEL	PRECISE	323	0	0	0/0	0/0	0/0	0/0	0/0	0/0	0/0	0/0	1/1	1/1	0/0	1/1	0/1	0/0
Chromosome2-2887094	INS	PRECISE	10364	0	0	0/0	0/0	0/0	0/0	0/0	0/0	0/0	0/0	0/0	0/0	0/1	0/0	0/0	0/0
Chromosome2-2887095	BND	PRECISE	0	0	0	0/0	0/0	0/0	0/0	0/0	0/0	0/0	0/0	0/0	0/0	0/1	0/0	0/0	0/0
Chromosome2-2887416	DEL	PRECISE	211	0.577	0.577	0/0	0/0	0/0	0/0	0/0	0/0	0/0	0/0	1/1	0/0	0/0	0/1	0/1	0/0
Chromosome2-2887521	INS	PRECISE	12290	0	0	0/0	0/0	0/0	0/0	0/0	0/0	0/0	0/0	0/0	0/0	0/0	0/1	0/0	0/0
Chromosome2-2888088	BND	PRECISE	0	0	0	0/0	0/0	0/0	0/0	0/0	0/0	0/0	0/0	0/1	0/0	0/0	0/0	0/0	0/0
Chromosome2-2888500	INS	PRECISE	303	0	0.577	0/0	0/0	0/0	0/0	0/0	0/0	0/0	0/0	1/1	0/0	0/0	0/1	0/1	0/0
Chromosome2-2888687	DEL	PRECISE	8594	0	0	0/0	0/0	0/0	0/0	0/0	0/0	0/0	0/0	1/1	0/0	0/0	0/1	0/1	0/0
Chromosome2-2891236	INS	PRECISE	58	0	0	0/0	0/0	0/0	0/0	0/0	0/0	0/0	0/0	0/1	0/0	0/0	0/1	0/1	0/0
Chromosome2-2891245	DEL	PRECISE	154	0	0	0/0	0/0	0/0	0/0	0/0	0/0	0/0	0/0	0/0	0/0	0/1	0/0	0/0	0/0
Chromosome2-2891861	DEL	PRECISE	1480	0	0	0/0	0/0	0/0	0/0	0/0	0/0	0/0	0/0	0/0	1/1	0/1	0/1	0/0	0/0
Chromosome2-2892768	DEL	PRECISE	26098	0	0	0/0	0/0	0/0	0/0	0/0	0/0	0/0	0/0	0/0	0/0	0/1	0/0	0/0	0/0
Chromosome2-2892773	INS	PRECISE	1193	0	0	0/0	0/0	0/0	0/0	0/0	0/0	0/0	0/0	1/1	0/0	0/0	0/1	1/1	0/0
Chromosome2-2894791	DEL	PRECISE	94	0	0	0/0	0/0	0/0	0/0	0/0	0/0	1/1	1/1	1/1	0/0	0/0	0/1	0/0	0/0
Chromosome2-2895099	DEL	PRECISE	50	0	0	0/0	0/0	0/0	0/0	0/0	0/0	0/0	0/0	1/1	0/0	0/0	0/1	0/1	0/0
Chromosome2-2896134	BND	PRECISE	0	0	0	0/0	0/0	0/0	0/0	0/0	0/0	0/0	0/0	0/0	0/0	0/0	0/1	0/0	0/0
Chromosome2-2896278	BND	PRECISE	0	0	0	0/0	0/0	0/0	0/0	0/0	0/0	0/0	0/0	0/1	0/0	0/0	0/0	0/1	0/0
Chromosome2-2896494	DEL	PRECISE	635	0	0	0/0	0/0	1/1	0/0	0/0	0/0	0/0	0/0	1/1	1/1	0/1	1/1	0/1	0/0
Chromosome2-2897781	INS	PRECISE	6161	0	0	0/0	0/0	0/0	0/0	0/0	0/0	0/0	0/0	0/0	0/0	1/1	0/0	0/0	0/0
Chromosome2-2897799	INS	PRECISE	75	0	0	0/0	0/0	0/0	0/0	0/0	0/0	0/0	0/0	0/0	0/0	0/1	0/0	0/0	0/0
Chromosome2-2908573	BND	PRECISE	0	0	0	0/0	0/0	0/0	0/0	0/0	0/0	0/0	0/0	0/0	0/0	0/1	0/0	0/0	0/0
Chromosome2-2909481	DEL	PRECISE	2166	0.5	0.5	0/0	0/0	0/0	0/0	0/0	0/0	0/0	0/0	1/1	1/1	0/0	1/1	0/1	0/0
Chromosome2-2910494	BND	PRECISE	0	0	0	0/1	0/0	0/0	0/1	0/0	0/0	0/0	0/0	0/0	0/0	0/0	0/0	0/0	0/0
Chromosome2-2912158	DEL	PRECISE	9960	0	0	0/0	0/0	0/0	0/0	0/0	0/0	0/0	0/0	0/0	0/0	0/1	0/0	0/0	0/0
Chromosome2-2912323	BND	PRECISE	0	0	0	0/0	0/0	0/0	0/0	0/0	0/0	0/0	0/0	0/0	0/1	0/0	0/1	0/0	0/0
Chromosome2-2912323	BND	PRECISE	0	0	0	0/0	0/0	0/0	0/0	0/0	0/0	0/0	0/0	0/1	0/0	0/0	0/0	0/1	0/0
Chromosome2-2919827	BND	PRECISE	0	0	0	0/0	0/0	0/0	0/0	0/0	0/0	0/0	0/0	0/1	0/0	0/0	1/1	0/0	0/0
Chromosome2-2920137	DEL	PRECISE	56	0	0	0/0	0/0	0/0	0/0	0/0	0/0	0/0	0/0	1/1	1/1	0/0	1/1	0/0	0/0
Chromosome2-2921133	INS	PRECISE	213	0	0	0/0	0/0	0/0	0/0	0/0	0/0	0/0	0/0	0/0	0/0	0/0	0/0	0/0	0/1
Chromosome2-2921266	INS	PRECISE	54	0	0	0/0	0/0	0/0	0/0	0/0	0/0	0/0	0/0	0/0	0/0	0/0	0/0	0/0	0/1
Chromosome2-2922944	BND	PRECISE	0	0	0	0/0	0/0	0/0	0/0	0/0	0/0	0/0	0/0	0/0	1/1	0/0	0/1	0/0	0/0
Chromosome2-2923341	DEL	PRECISE	88	0	0	0/0	0/0	0/0	0/0	0/0	0/0	0/0	0/0	1/1	0/0	0/0	0/1	0/1	0/0
Chromosome2-2923679	DEL	PRECISE	7001	0	0	0/0	0/0	0/0	0/0	0/0	0/0	0/0	0/0	0/0	1/1	0/0	0/0	0/0	0/0
Chromosome2-2923679	DUP	PRECISE	3619474	0	0	0/0	0/0	0/0	0/0	0/0	0/0	0/0	0/0	0/1	0/0	0/0	0/0	0/0	0/0
Chromosome2-2923935	DEL	PRECISE	107	0	0	0/0	0/0	0/0	0/0	0/0	0/0	1/1	1/1	0/0	0/0	0/0	0/0	0/0	0/0
Chromosome2-2926018	BND	PRECISE	0	0	0	0/0	0/0	0/0	0/0	0/0	0/0	0/0	0/0	1/1	0/0	0/0	0/1	0/0	0/0
Chromosome2-2927292	DEL	PRECISE	81	0	0	0/0	0/0	0/0	0/0	0/0	0/0	0/0	0/0	0/0	1/1	0/0	0/1	0/0	0/0
Chromosome2-2928937	DEL	PRECISE	61	0	0	0/0	0/0	1/1	0/0	0/0	0/0	0/0	0/0	0/0	0/0	0/0	0/0	0/0	0/0
Chromosome2-2929141	DEL	PRECISE	66	0	0	0/0	0/0	1/1	0/0	0/0	0/0	0/0	0/0	0/0	0/0	0/0	0/0	0/0	0/0
Chromosome2-2929638	INS	PRECISE	1089	0	0	0/0	0/0	1/1	0/0	0/0	0/0	0/0	0/0	0/0	0/0	0/0	0/0	0/0	0/0
Chromosome2-2929707	INS	PRECISE	776	0	0	0/0	0/0	1/1	0/0	0/0	0/0	0/0	0/0	0/0	0/0	0/0	0/0	0/0	0/0
Chromosome2-2929877	INS	PRECISE	5361	0	0	0/0	0/0	1/1	0/0	0/0	0/0	0/0	0/0	0/0	0/0	0/0	0/0	0/0	0/0
Chromosome2-2929950	INS	PRECISE	235	0	0	0/0	0/0	1/1	0/0	0/0	0/0	0/0	0/0	0/0	0/0	0/0	0/0	0/0	0/0
Chromosome2-2930010	INV	PRECISE	3914995	0	0	0/0	0/0	0/0	0/0	0/0	0/0	0/0	0/0	0/1	0/1	0/0	0/1	0/0	0/0
Chromosome2-2930061	INS	PRECISE	92	0	0	0/0	0/0	0/0	0/0	0/0	0/0	0/0	0/0	1/1	0/0	0/0	0/1	0/1	0/0
Chromosome2-2930569	INS	PRECISE	160	0	0.5	0/0	0/0	0/0	0/0	0/0	0/0	0/0	0/0	1/1	0/0	0/1	1/1	0/1	0/0
Chromosome2-2931342	DEL	PRECISE	1121	0	0	0/0	0/0	1/1	0/0	0/0	0/0	0/0	0/0	0/0	0/0	0/1	0/0	0/0	0/0
Chromosome2-2932526	INS	PRECISE	6834	0	0	0/0	0/0	0/0	0/0	0/0	0/0	0/0	0/0	0/0	0/0	0/0	0/0	0/1	0/0
Chromosome2-2932596	DUP	PRECISE	1721	0	0	0/0	0/0	0/0	0/0	0/0	0/0	0/0	0/0	0/1	0/0	0/0	0/0	0/0	0/0
Chromosome2-2932646	DEL	PRECISE	94	0	0	0/0	0/0	1/1	0/0	0/0	0/0	0/0	0/0	0/0	0/0	0/0	0/0	0/0	0/0
Chromosome2-2933124	INS	PRECISE	498	0	0.707	0/0	0/0	0/0	0/0	0/0	0/0	0/0	0/0	0/0	1/1	0/0	1/1	0/0	0/0
Chromosome2-2933430	DEL	PRECISE	325	0	0	0/0	0/0	0/0	0/0	0/0	0/0	0/0	0/0	0/0	1/1	0/0	0/1	0/0	0/0
Chromosome2-2933546	INS	PRECISE	12442	0	0	0/0	0/0	0/0	0/0	0/0	0/0	0/0	0/0	0/0	0/0	0/0	0/0	0/1	0/0
Chromosome2-2933546	BND	PRECISE	0	0	0	0/0	0/0	0/0	0/0	0/0	0/0	0/0	0/0	0/1	0/0	0/0	0/1	0/0	0/0
Chromosome2-2934304	INS	PRECISE	52	0	0	0/0	0/0	1/1	0/0	0/0	0/0	0/0	0/0	0/0	0/0	0/0	0/0	0/0	0/0
Chromosome2-2934328	DEL	PRECISE	1298	0	0	0/0	0/0	1/1	0/0	0/0	0/0	0/0	0/0	1/1	1/1	0/0	1/1	0/0	0/0

Chromosome2-2934550	DEL	PRECISE	9115	0	0	0/0	0/0	1/1	0/0	0/0	0/0	0/0	0/0	0/0	0/0	0/0	0/0	0/0	0/0
Chromosome2-2934602	DEL	PRECISE	54	0	0	0/0	0/0	0/0	0/0	0/0	0/0	0/0	0/0	1/1	0/0	0/0	0/1	0/0	0/0
Chromosome2-2935167	DEL	PRECISE	2009	0	0	0/0	0/0	0/0	0/0	0/0	0/0	0/0	0/0	1/1	1/1	0/0	1/1	0/0	0/0
Chromosome2-2935652	BND	PRECISE	0	0	0	0/0	0/0	0/0	0/0	0/0	0/0	0/0	0/0	0/0	0/0	0/1	0/0	0/0	0/0
Chromosome2-2936802	INS	PRECISE	76	0	0.577	0/0	0/0	0/0	0/0	0/0	0/0	0/0	0/0	1/1	0/0	0/0	0/1	0/1	0/0
Chromosome2-2938954	INS	PRECISE	654	0	0	0/0	0/0	0/0	0/0	0/0	0/0	0/1	0/0	0/0	0/0	0/0	0/0	0/0	0/0
Chromosome2-2939813	INS	PRECISE	153	0	0	0/0	0/0	0/0	1/1	0/0	1/1	1/1	1/1	0/0	0/0	0/0	0/0	0/0	0/0
Chromosome2-2940062	DEL	PRECISE	294	0	0	0/0	0/0	0/0	0/0	0/0	0/0	0/0	0/0	0/0	1/1	0/0	0/0	0/0	0/0
Chromosome2-2940337	DEL	PRECISE	556	0	0	0/0	0/0	0/0	0/0	0/0	0/0	0/0	0/0	0/0	0/0	0/1	0/0	0/0	0/0
Chromosome2-2942604	INS	PRECISE	231	0	0.447	0/0	0/0	0/0	0/0	0/0	0/0	0/0	0/0	1/1	1/1	0/1	1/1	0/1	0/0
Chromosome2-2943891	INS	PRECISE	1362	0	0	0/0	0/0	0/0	0/0	0/0	0/0	0/0	0/0	0/0	1/1	0/0	0/1	0/0	0/0
Chromosome2-2945010	BND	PRECISE	0	0	0	0/0	0/0	0/0	0/0	0/0	0/0	0/0	0/0	0/0	0/0	0/0	0/1	0/0	0/0
Chromosome2-2948539	INS	PRECISE	3557	0	0	0/0	0/0	0/0	0/0	0/0	0/0	0/0	0/0	0/0	1/1	0/0	0/1	0/0	0/0
Chromosome2-2949071	DEL	PRECISE	261	0	0	0/0	0/0	0/0	0/0	0/0	0/0	0/0	0/0	0/0	1/1	0/0	0/1	0/0	0/0
Chromosome2-2949824	INS	PRECISE	79	0	0.577	0/0	0/0	0/0	0/0	0/0	0/0	0/0	0/0	0/0	1/1	0/1	0/1	0/0	0/0
Chromosome2-2949889	DEL	PRECISE	60	0	0	0/0	0/0	0/0	0/0	0/0	0/0	0/0	0/0	0/0	1/1	0/1	0/1	0/0	0/0
Chromosome2-2952352	INS	PRECISE	877	0	0	0/0	0/0	0/0	0/0	0/0	0/0	0/0	0/0	0/0	1/1	0/0	0/1	0/0	0/0
Chromosome2-2952807	DEL	PRECISE	177	0	0	0/0	0/0	0/0	0/0	0/0	0/0	0/0	0/0	0/0	1/1	0/0	0/1	0/0	0/0
Chromosome2-2953178	DEL	PRECISE	1385	0	0	0/0	0/0	0/0	0/0	0/0	0/0	0/0	0/0	0/0	1/1	0/1	1/1	0/0	0/0
Chromosome2-2955791	INS	PRECISE	72	0	0	0/0	0/0	0/0	0/0	0/0	0/0	0/0	0/0	0/0	0/0	0/0	0/0	0/1	0/0
Chromosome2-2956775	DEL	PRECISE	1171	0	0	0/0	0/0	0/0	0/0	0/0	0/0	0/0	0/0	1/1	1/1	0/1	1/1	0/1	0/0
Chromosome2-2957862	DEL	PRECISE	12132	0	0	0/0	0/0	0/0	0/0	0/0	0/0	0/0	0/0	1/1	0/0	0/0	1/1	0/1	0/0
Chromosome2-2960429	INS	PRECISE	68	0	0	0/0	0/0	0/0	0/0	0/0	0/0	0/0	0/0	1/1	1/1	0/1	1/1	0/1	0/0
Chromosome2-2961687	INS	PRECISE	867	0	0	0/0	0/0	0/0	0/0	0/0	0/0	0/0	0/0	0/0	1/1	0/0	1/1	0/0	0/0
Chromosome2-2963129	BND	PRECISE	0	0	0	0/0	0/0	0/1	0/0	0/0	0/0	0/0	0/0	0/0	0/0	0/0	0/0	0/0	0/0
Chromosome2-2963590	INS	PRECISE	451	0	0.577	0/0	0/0	0/0	0/0	0/0	0/0	0/0	0/0	0/0	1/1	0/1	0/1	0/0	0/0
Chromosome2-2964090	INS	PRECISE	541	0	0	0/0	0/0	0/0	0/0	0/0	0/0	0/0	0/0	0/0	0/0	0/1	0/0	0/0	0/0
Chromosome2-2964282	DEL	PRECISE	370	0	0	0/0	0/0	0/0	0/0	0/0	0/0	0/0	0/0	1/1	0/0	0/0	0/1	0/1	0/0
Chromosome2-2965420	BND	PRECISE	0	0	0	0/0	0/0	0/0	0/0	0/0	0/0	0/0	0/0	0/0	0/0	0/0	0/1	0/0	0/0
Chromosome2-2966877	DEL	PRECISE	108	0	0	0/0	0/0	0/0	0/0	0/0	0/0	0/0	0/0	0/0	1/1	0/0	0/1	0/0	0/0
Chromosome2-2967464	INS	PRECISE	623	0	0	0/0	0/0	0/0	0/0	0/0	0/0	0/0	0/0	0/0	0/0	0/1	0/0	0/0	0/0
Chromosome2-2968108	DEL	PRECISE	1272	0	0	0/0	0/0	0/0	0/0	0/0	0/0	0/0	0/0	1/1	1/1	0/1	1/1	0/1	0/0
Chromosome2-2969353	INS	PRECISE	370	0	0.408	0/0	0/0	0/0	0/0	0/0	0/0	1/1	0/0	1/1	1/1	0/1	1/1	0/1	0/0
Chromosome2-2970629	INS	PRECISE	6686	0	0	0/0	0/0	0/0	0/0	0/0	0/0	0/0	0/0	0/0	1/1	0/0	1/1	0/0	0/0
Chromosome2-2970629	BND	PRECISE	0	0	0	0/0	0/0	0/0	0/0	0/0	0/0	0/0	0/0	0/0	0/1	0/0	0/1	0/0	0/0
Chromosome2-2970630	BND	PRECISE	0	0	0	0/0	0/0	0/0	0/0	0/0	0/0	0/0	0/0	0/0	0/1	0/0	0/1	0/0	0/0
Chromosome2-2975441	DEL	PRECISE	70	0	0	0/0	0/0	0/0	0/0	0/0	0/0	0/0	0/0	0/0	1/1	0/0	0/1	0/0	0/0
Chromosome2-2981124	DEL	PRECISE	50	0	0	0/0	0/0	0/0	0/0	0/0	0/0	0/0	0/0	1/1	0/0	0/0	0/1	0/1	0/0
Chromosome2-2981331	DEL	PRECISE	996	0.894	0	0/0	0/0	0/0	0/0	0/0	0/0	0/0	0/0	1/1	1/1	0/1	1/1	0/1	0/0
Chromosome2-2983127	DEL	PRECISE	746	0	0	0/0	0/0	0/0	0/0	0/0	0/0	0/0	0/0	1/1	0/0	0/1	1/1	0/1	0/0
Chromosome2-2983499	DEL	PRECISE	341	0	0	0/0	0/0	0/0	0/0	0/0	0/0	0/0	0/0	1/1	1/1	0/1	1/1	0/1	0/0
Chromosome2-2984441	DEL	PRECISE	127	0	0	0/0	0/0	0/0	0/0	0/0	0/0	0/0	0/0	1/1	0/0	0/1	0/1	0/1	0/0
Chromosome2-2984546	INS	PRECISE	59	0	0	0/0	0/0	0/0	0/0	0/0	0/0	0/0	0/0	1/1	1/1	0/1	1/1	0/1	0/0
Chromosome2-2985609	BND	PRECISE	0	0	0	0/0	0/0	0/0	0/0	0/0	0/0	0/0	0/0	0/1	0/0	0/0	0/0	0/0	0/0
Chromosome2-2986162	DEL	PRECISE	1342	0	0	0/0	0/0	0/0	0/0	0/0	0/0	0/0	0/0	1/1	1/1	0/0	1/1	0/1	0/0
Chromosome2-2987243	DEL	PRECISE	226	0	0	0/0	0/0	0/0	0/0	0/0	0/0	0/0	0/0	0/0	0/0	0/1	0/0	0/0	0/0
Chromosome2-2989771	DEL	PRECISE	62	0.894	0.894	0/0	0/0	0/0	0/0	0/0	0/0	0/0	0/0	1/1	1/1	0/1	1/1	0/0	0/0
Chromosome2-2993572	BND	PRECISE	0	0	0	0/0	0/0	0/0	0/0	0/0	0/0	0/0	0/0	1/1	0/1	0/0	1/1	0/1	0/0
Chromosome2-2995498	INS	PRECISE	476	0	0.707	0/0	0/0	0/0	0/0	0/0	0/0	0/0	0/0	0/0	1/1	0/0	0/0	0/0	0/0
Chromosome2-2996765	DEL	PRECISE	1017	0	0	0/0	0/0	0/0	0/0	0/0	0/0	0/0	0/0	1/1	1/1	0/1	1/1	1/1	0/0
Chromosome2-2997616	INS	PRECISE	201	0	0	0/0	0/0	0/0	0/0	0/0	0/0	0/0	0/0	1/1	0/0	0/0	0/1	1/1	0/0
Chromosome2-3000217	DEL	PRECISE	355	0	0	0/0	0/0	0/0	0/0	0/0	0/0	0/0	0/0	1/1	1/1	0/0	1/1	0/1	0/0
Chromosome2-3000419	INS	PRECISE	135	0	0.577	0/0	0/0	0/0	0/0	0/0	0/0	0/0	0/0	0/0	1/1	0/1	0/1	0/0	0/0
Chromosome2-3003123	INS	PRECISE	417	0	0	0/0	0/0	0/0	0/1	0/0	0/0	0/0	0/0	0/0	0/0	0/0	0/0	0/0	0/0
Chromosome2-3003483	BND	PRECISE	0	0	0	0/1	0/0	0/0	0/0	0/0	0/0	0/0	0/0	0/0	0/0	0/0	0/0	0/0	0/0
Chromosome2-3004274	INS	PRECISE	2140	0	0.707	0/0	0/0	0/0	0/0	0/0	0/0	0/0	0/0	0/0	1/1	0/0	0/1	0/0	0/0
Chromosome2-3004824	DEL	PRECISE	121	0	0	0/0	0/0	0/0	0/0	0/0	0/0	0/0	0/0	1/1	1/1	0/1	1/1	0/1	0/0
Chromosome2-3008316	DEL	PRECISE	51	0	0	0/0	0/0	0/0	0/0	0/0	0/0	0/0	0/0	0/0	0/0	0/1	0/0	0/0	0/0
Chromosome2-3008402	INS	PRECISE	1555	0	0	0/0	0/0	0/0	0/0	0/0	0/0	0/0	0/0	0/0	0/0	0/1	0/0	0/0	0/0
Chromosome2-3008798	INS	PRECISE	4608	0	0	0/0	0/0	1/1	0/0	0/0	0/0	0/0	0/0	0/0	0/0	0/0	0/0	0/0	0/0
Chromosome2-3008872	BND	PRECISE	0	0	0	0/0	0/0	0/1	0/0	0/0	0/0	0/1	0/1	0/0	0/0	0/0	0/0	0/0	0/0
Chromosome2-3009067	DEL	PRECISE	8305	0	0	0/0	0/0	1/1	0/0	0/0	0/1	0/1	0/1	0/0	0/0	0/0	0/0	0/0	0/0
Chromosome2-3009225	DEL	PRECISE	284	0	0	0/0	0/0	0/1	0/0	0/0	0/1	0/1	0/1	0/0	0/0	0/0	0/0	0/0	0/0
Chromosome2-3009949	INS	PRECISE	5421	0	0	0/0	0/0	0/0	0/0	1/1	0/0	0/0	0/0	0/0	0/0	0/0	0/0	0/0	0/0
Chromosome2-3009949	BND	PRECISE	0	0	0	0/0	0/0	0/0	0/0	0/1	0/0	0/0	0/0	0/0	0/0	0/0	0/0	0/0	0/0
Chromosome2-3010050	INS	PRECISE	55	0	0	0/0	0/0	0/0	0/0	0/0	0/0	0/0	0/0	0/0	1/1	0/0	0/1	0/0	0/0
Chromosome2-3010439	DEL	PRECISE	174	0	0	0/0	0/0	0/1	0/0	0/0	0/0	0/0	0/0	0/0	0/0	0/0	0/0	0/0	0/0
Chromosome2-3010535	DEL	PRECISE	8085	0	0	0/0	0/0	0/0	0/0	1/1	0/0	0/0	0/0	0/0	1/1	0/1	0/1	0/0	0/0
Chromosome2-3011789	DEL	PRECISE	456	0	0	0/0	0/0	0/0	0/0	1/1	0/0	0/0	0/0	0/0	0/0	0/0	0/0	0/0	0/0
Chromosome2-3011876	DEL	PRECISE	7721	0	0	0/0	0/0	0/0	0/0	1/1	0/0	0/0	0/0	0/0	0/0	0/0	0/0	0/0	0/0
Chromosome2-3011980	DEL	PRECISE	3578	0	0	0/0	0/0	0/0	0/0	0/0	0/0	0/0	0/0	0/0	0/0	0/1	0/0	0/0	0/0
Chromosome2-3012345	DEL	PRECISE	2680	0	0	0/0	0/0	0/0	0/0	0/0	0/0	0/0	0/0	0/0	0/0	0/1	0/0	0/0	0/0
Chromosome2-3014069	DEL	PRECISE	160	0	0	0/0	0/0	0/0	0/0	0/0	0/0	0/0	0/0	0/0	0/0	0/1	0/0	0/0	0/0

Chromosome2-3014488	DEL	PRECISE	51	0	0	0/0	0/0	0/0	0/0	0/0	0/0	0/0	0/0	0/0	1/1	0/0	0/1	0/0	0/0
Chromosome2-3015432	DEL	PRECISE	286	0	0	0/0	0/0	0/0	0/0	0/0	0/0	0/0	0/0	0/0	0/0	0/0	0/0	0/0	0/1
Chromosome2-3017477	INS	PRECISE	610	0	0.577	0/0	0/0	0/0	0/0	0/0	0/0	0/0	0/0	0/1	0/0	0/1	0/1	0/0	0/0
Chromosome2-3017598	BND	PRECISE	0	0	0	0/0	0/0	0/0	0/0	0/0	0/0	0/0	0/0	0/1	0/0	0/0	0/1	0/0	0/0
Chromosome2-3017659	DEL	PRECISE	3788	0	0	0/0	0/0	0/0	0/0	0/0	0/0	0/0	0/0	0/0	1/1	0/0	0/1	0/0	0/0
Chromosome2-3017690	INS	PRECISE	427	0	0	0/0	0/0	0/0	0/0	0/0	0/0	0/0	0/0	0/0	0/0	0/1	0/0	0/0	0/0
Chromosome2-3018423	DEL	PRECISE	476	0.707	0	0/0	0/0	0/0	0/0	0/0	0/0	0/0	0/0	0/0	1/1	0/0	0/1	0/0	0/0
Chromosome2-3018827	DEL	PRECISE	53	0	0	0/0	0/0	0/0	0/0	0/0	0/0	0/0	0/0	0/0	1/1	0/0	0/1	0/0	0/0
Chromosome2-3020273	INS	PRECISE	989	0	0	0/0	0/0	0/0	0/0	0/0	0/0	0/0	0/0	0/0	0/0	0/1	0/0	0/0	0/0
Chromosome2-3021382	DEL	PRECISE	56	0	0	0/0	0/0	0/0	0/0	0/0	0/0	0/0	0/0	0/0	0/0	0/1	0/0	0/0	0/0
Chromosome2-3022508	DEL	PRECISE	96	0	0	0/0	0/0	0/0	0/0	0/0	0/0	0/0	0/0	0/0	1/1	0/0	0/1	0/0	0/0
Chromosome2-3023813	DEL	PRECISE	733	0	0	0/0	0/0	0/0	0/0	0/0	1/1	1/1	1/1	1/1	1/1	0/1	1/1	0/0	0/0
Chromosome2-3023911	DEL	PRECISE	79	0	0	0/0	0/0	0/0	0/0	0/0	0/0	0/0	0/0	0/0	1/1	0/0	0/0	0/0	0/0
Chromosome2-3024226	DEL	PRECISE	211	0	0	0/0	0/0	1/1	0/0	0/0	1/1	1/1	1/1	0/0	0/0	0/0	0/0	0/0	0/1
Chromosome2-3024839	DEL	PRECISE	56	0	0	0/0	0/0	0/0	0/0	0/0	0/0	0/0	0/0	1/1	0/0	0/0	1/1	0/0	0/0
Chromosome2-3026000	INS	PRECISE	149	0	0	0/0	0/0	0/0	0/0	0/0	0/0	0/0	0/0	0/0	0/0	0/1	0/0	0/0	0/0
Chromosome2-3026609	DEL	PRECISE	68	0	0	0/0	0/0	0/0	0/0	1/1	0/0	0/0	0/0	0/0	0/0	0/0	0/0	0/0	0/0
Chromosome2-3027906	DEL	PRECISE	915	0.488	0	0/0	0/0	0/0	0/0	1/1	0/0	0/0	0/0	1/1	1/1	0/1	1/1	1/1	0/1
Chromosome2-3028060	DEL	PRECISE	89	0	0	0/0	0/0	0/0	0/0	0/0	0/0	0/0	0/0	0/0	0/0	0/0	0/0	0/0	0/1
Chromosome2-3028122	DEL	PRECISE	5882	0	0	0/0	0/0	0/0	0/0	1/1	0/0	0/0	0/0	0/0	0/0	0/0	0/0	0/0	0/1
Chromosome2-3029117	DEL	PRECISE	686	0	0	0/0	0/0	0/0	0/0	0/0	0/0	0/0	0/0	0/0	0/0	0/0	0/0	0/0	0/1
Chromosome2-3029254	DEL	PRECISE	606	0	0	0/0	0/0	0/0	0/0	0/0	0/0	0/0	0/0	0/0	0/0	0/0	0/0	0/0	0/1
Chromosome2-3032103	DUP	PRECISE	12201742	0	0	0/0	0/1	0/0	0/0	0/1	0/0	0/1	0/0	0/0	0/1	0/1	0/0	0/1	0/0
Chromosome2-3034289	DEL	PRECISE	110	0	0	0/0	0/0	0/0	0/0	0/0	0/0	0/0	0/0	0/0	0/0	0/0	0/0	0/0	0/1
Chromosome2-3034832	INS	PRECISE	864	0	0	0/0	0/0	0/0	0/0	0/0	0/0	0/0	0/0	0/0	0/0	0/0	0/0	0/0	0/1
Chromosome2-3035068	DEL	PRECISE	228	0	0	0/0	0/0	0/0	0/0	0/0	0/0	0/0	0/0	0/0	0/0	0/0	0/0	0/0	0/1
Chromosome2-3037427	DEL	PRECISE	732	0	0	0/0	0/0	0/0	0/0	0/0	0/0	0/0	0/0	1/1	1/1	0/1	1/1	0/1	0/0
Chromosome2-3037772	INS	PRECISE	1320	0	0	0/0	0/0	0/0	0/0	0/0	0/0	0/0	0/0	0/0	0/0	0/1	0/0	0/0	0/0
Chromosome2-3038304	DEL	PRECISE	524	0	0	0/0	0/0	0/0	0/0	0/0	0/0	0/0	0/0	0/0	1/1	0/0	0/1	0/0	0/0
Chromosome2-3039232	BND	PRECISE	0	0	0	0/0	0/0	0/0	0/0	0/0	0/0	0/0	0/0	0/0	0/0	0/1	0/0	0/0	0/0
Chromosome2-3040339	INS	PRECISE	66	0	0	0/0	0/0	0/0	0/0	0/0	0/0	0/0	0/0	1/1	1/1	0/1	1/1	0/1	0/0
Chromosome2-3041036	DEL	PRECISE	96	0	0	0/0	0/0	0/0	0/0	0/0	0/0	0/0	0/0	1/1	1/1	0/0	1/1	0/1	0/0
Chromosome2-3041036	INS	PRECISE	1184	0	0	0/0	0/0	0/0	0/0	0/0	0/0	0/0	0/0	0/0	0/0	0/1	0/0	0/0	0/0
Chromosome2-3042522	INS	PRECISE	96	0	0.548	0/0	0/0	0/0	0/0	0/0	0/0	0/0	0/0	1/1	1/1	0/1	1/1	0/1	0/0
Chromosome2-3042818	DEL	PRECISE	1479	0	0	0/0	0/0	0/0	0/0	0/0	0/0	0/0	0/0	1/1	1/1	0/0	1/1	0/1	0/0
Chromosome2-3043166	DEL	PRECISE	13288	0.447	0.447	0/0	0/0	0/0	0/0	0/0	0/0	0/0	0/0	0/1	0/1	0/1	0/1	0/1	0/0
Chromosome2-3043166	BND	PRECISE	0	0	0	0/0	0/1	0/0	0/0	0/0	0/0	0/0	0/0	0/0	0/0	0/0	0/0	0/1	0/0
Chromosome2-3043166	BND	PRECISE	0	0	0	0/0	0/0	0/0	0/1	0/0	0/0	0/0	0/0	0/0	0/0	0/0	0/0	0/0	0/0
Chromosome2-3043179	INV	PRECISE	9462243	0	0	0/0	0/0	0/0	0/0	0/0	0/0	0/0	0/0	0/1	0/0	0/0	0/1	0/0	0/0
Chromosome2-3043179	BND	PRECISE	0	0	0	0/0	0/0	0/0	0/0	0/0	0/0	0/0	0/0	0/0	0/1	0/0	0/0	0/0	0/0
Chromosome2-3044389	INS	PRECISE	407	0	0.707	0/0	0/0	0/0	0/0	0/0	0/0	0/0	0/0	0/1	0/0	0/0	0/0	0/0	0/0
Chromosome2-3044494	BND	PRECISE	0	0	0	0/0	0/0	0/0	0/0	0/0	0/0	0/0	0/0	0/0	0/1	0/0	0/0	0/0	0/0
Chromosome2-3044495	BND	PRECISE	0	0	0	0/0	0/1	0/0	0/0	0/0	0/0	0/0	0/0	0/0	0/0	0/0	0/0	0/1	0/0
Chromosome2-3046498	INS	PRECISE	235	0	0	0/0	0/0	0/0	0/0	0/0	0/0	0/0	0/0	0/0	1/1	0/0	0/1	0/0	0/0
Chromosome2-3046982	BND	PRECISE	0	0	0	0/0	0/0	0/0	0/0	0/0	0/0	0/0	0/0	0/0	0/1	0/0	0/0	0/0	0/0
Chromosome2-3046984	BND	PRECISE	0	0	0	0/0	0/0	0/0	0/0	0/0	0/0	0/0	0/0	0/0	0/1	0/0	0/1	0/0	0/0
Chromosome2-3047156	INS	PRECISE	58	0	0	0/0	0/0	0/0	0/0	0/0	0/0	0/0	0/0	0/0	1/1	0/0	0/1	0/0	0/0
Chromosome2-3047494	DEL	PRECISE	697	0	0	0/0	0/0	0/0	0/0	0/0	0/0	0/0	0/0	0/0	0/0	0/1	0/0	0/0	0/0
Chromosome2-3048218	DEL	PRECISE	353	0	0	0/0	0/0	0/0	0/0	1/1	0/0	1/1	0/0	0/0	0/0	0/0	0/0	0/0	0/0
Chromosome2-3049606	DEL	PRECISE	1013	0	0	0/0	1/1	0/0	0/0	0/0	0/0	0/0	0/0	1/1	1/1	1/1	1/1	1/1	1/1
Chromosome2-3050039	INS	PRECISE	50	0.976	0.756	0/0	1/1	0/0	0/0	0/0	0/0	0/0	0/0	1/1	1/1	1/1	1/1	1/1	1/1
Chromosome2-3054159	DEL	PRECISE	7938	0	0	0/0	1/1	0/0	0/0	0/0	0/0	0/0	0/0	1/1	1/1	1/1	1/1	0/0	0/0
Chromosome2-3054760	DEL	PRECISE	6060	0	0	0/0	0/0	0/0	0/0	0/0	0/0	0/0	0/0	0/0	0/0	0/0	0/0	0/1	0/1
Chromosome2-3057286	INS	PRECISE	1375	0	0	0/0	1/1	0/0	0/0	0/0	0/0	0/0	0/0	1/1	1/1	1/1	1/1	1/1	1/1
Chromosome2-3060754	DEL	PRECISE	241	0	0	0/0	0/0	0/0	0/0	0/0	0/0	0/0	0/0	0/0	0/0	1/1	1/1	1/1	0/0
Chromosome2-3061105	INS	PRECISE	10042	0	0	0/0	0/0	0/0	0/0	0/1	0/0	0/0	0/0	0/0	0/0	0/0	0/0	0/0	0/0
Chromosome2-3062770	DEL	PRECISE	1354	0	0	0/0	0/1	0/1	0/0	0/1	0/1	0/1	0/1	0/1	0/1	1/1	1/1	1/1	0/0
Chromosome2-3062990	DEL	PRECISE	120	0	0	0/0	0/0	0/1	0/0	0/0	0/0	0/0	0/0	0/0	0/0	0/0	0/0	0/0	0/0
Chromosome2-3063272	DEL	PRECISE	23777	0	0	0/0	1/1	0/0	0/0	0/0	0/0	0/0	0/0	0/0	0/0	1/1	0/0	1/1	1/1
Chromosome2-3063499	DEL	PRECISE	2684	0	0	0/0	0/0	0/0	0/0	0/0	0/0	0/0	0/0	0/0	0/0	0/0	0/0	0/0	1/1
Chromosome2-3064220	DEL	PRECISE	605	0	0	0/0	0/0	0/0	0/0	0/0	0/0	0/1	0/0	0/0	0/0	0/0	0/0	0/0	0/0
Chromosome2-3064384	DEL	PRECISE	64	0	0	0/0	0/0	0/0	0/0	0/0	0/0	0/1	0/0	0/0	0/0	0/0	0/0	0/0	0/0
Chromosome2-3064589	INS	PRECISE	6608	0	0	0/0	0/0	0/0	0/0	0/0	0/0	0/0	0/0	0/0	0/0	0/0	0/0	0/0	1/1
Chromosome2-3065174	INS	PRECISE	108	0	0.894	0/0	0/0	0/0	0/0	0/0	0/0	0/0	0/0	0/1	0/0	1/1	1/1	1/1	0/0
Chromosome2-3065425	DEL	PRECISE	995	0	0	0/0	0/0	0/1	0/1	0/1	0/1	1/1	0/1	0/1	0/1	0/0	1/1	1/1	1/1
Chromosome2-3065549	DEL	PRECISE	333	0	0	0/0	0/0	0/1	0/0	0/1	0/1	0/1	0/1	0/1	0/1	0/0	1/1	1/1	0/0
Chromosome2-3065676	DEL	PRECISE	117	0	0	0/0	0/0	0/0	0/0	0/0	0/0	0/0	0/0	0/1	0/0	0/0	0/1	1/1	0/0
Chromosome2-3067604	INS	PRECISE	147	0	0	0/0	0/0	0/1	0/0	0/0	0/0	0/0	0/0	0/0	0/0	0/0	0/0	0/0	0/0
Chromosome2-3068504	DEL	PRECISE	88	0	0	0/0	0/0	0/0	0/0	0/0	0/0	0/0	0/0	0/0	0/0	0/1	0/0	0/0	0/0
Chromosome2-3069688	DEL	PRECISE	75	0	0	0/0	0/0	0/0	0/0	0/1	0/0	0/0	0/0	0/0	0/0	0/0	0/0	0/0	0/0
Chromosome2-3069802	DEL	PRECISE	5770	0	0	0/0	0/0	0/0	0/0	0/0	0/0	0/0	0/0	0/0	0/0	0/0	0/0	0/0	0/1
Chromosome2-3070022	INS	PRECISE	1368	0	0	0/0	0/0	0/0	0/1	0/0	0/0	0/0	0/0	0/0	0/0	0/0	0/0	0/0	0/0
Chromosome2-3070048	DEL	PRECISE	90	0	0	0/0	0/0	0/0	0/1	0/0	0/0	0/1	0/0	0/0	0/0	0/0	0/0	0/0	0/0

Chromosome2-3070128	INS	PRECISE	66	0	0	0/0	0/1	0/1	0/0	0/0	0/0	0/0	0/0	0/1	0/1	1/1	1/1	1/1	0/1
Chromosome2-3071402	INS	PRECISE	1015	0	0	0/0	0/0	0/1	0/0	0/0	0/0	0/0	0/0	0/0	0/0	0/0	0/0	0/0	0/0
Chromosome2-3072473	DEL	PRECISE	360	0	0	0/0	0/0	0/0	0/1	0/0	0/1	0/1	0/0	0/0	0/0	0/0	0/0	0/0	0/0
Chromosome2-3072639	DEL	PRECISE	1028	0	0	0/0	0/0	0/0	0/1	0/0	0/1	0/1	0/0	0/0	0/0	0/0	0/0	0/0	0/0
Chromosome2-3074116	INS	PRECISE	65	0	0	0/0	0/1	0/1	0/1	0/1	0/1	1/1	0/1	0/1	0/1	1/1	1/1	1/1	1/1
Chromosome2-3074423	DEL	PRECISE	59	0	0	0/0	0/0	0/0	0/0	0/0	0/0	0/0	0/0	0/0	0/0	0/0	0/0	0/0	0/1
Chromosome2-3074521	INS	PRECISE	858	0	0	0/0	0/0	0/0	0/0	0/1	0/0	0/0	0/0	0/0	0/0	0/0	0/0	0/0	0/0
Chromosome2-3074581	INS	PRECISE	160	0	0	0/0	0/0	0/0	0/0	0/0	0/0	0/0	0/0	0/1	0/0	0/1	0/1	0/1	0/0
Chromosome2-3075104	BND	PRECISE	0	0	0	0/0	0/0	0/0	0/0	0/0	0/0	0/0	0/0	0/0	0/1	0/0	0/0	0/0	0/0
Chromosome2-3075511	INS	PRECISE	1273	0	0	0/0	0/0	0/0	0/0	0/0	0/0	0/0	0/0	0/0	0/0	0/0	0/0	0/0	0/1
Chromosome2-3077479	DEL	PRECISE	51	0	0	0/0	0/0	0/0	0/0	0/0	0/0	0/0	0/0	0/1	0/1	0/0	1/1	0/1	0/0
Chromosome2-3077655	INS	PRECISE	1394	0	0.5	0/0	0/1	0/0	0/0	0/0	0/0	0/0	0/0	0/0	0/0	0/1	0/0	0/1	0/1
Chromosome2-3078676	INS	PRECISE	2237	0	0	0/0	0/0	0/0	0/0	0/0	0/0	0/0	0/0	0/0	0/0	0/1	0/0	0/0	0/0
Chromosome2-3078957	INS	PRECISE	85	0	0	0/0	0/1	0/1	0/0	0/1	0/0	1/1	0/1	0/1	0/1	1/1	1/1	1/1	1/1
Chromosome2-3079076	DEL	PRECISE	5021	0	0	0/0	0/0	0/1	0/0	0/0	0/0	0/0	0/0	0/0	0/0	0/0	0/0	0/0	0/0
Chromosome2-3079313	DEL	PRECISE	74	0.447	0.447	0/0	0/0	0/0	0/1	0/0	0/1	1/1	0/1	0/0	0/0	0/0	0/0	0/0	1/1
Chromosome2-3081420	INS	PRECISE	150	0	0	0/0	0/0	0/0	0/0	0/0	0/0	0/1	0/1	0/0	0/0	0/0	0/0	0/0	0/0
Chromosome2-3082393	DEL	PRECISE	64	0	0	0/0	0/0	0/0	0/0	0/0	0/0	0/0	0/0	0/0	0/0	0/1	0/0	0/1	0/1
Chromosome2-3083392	INS	PRECISE	180	0	0	0/0	0/0	0/0	0/0	0/0	0/0	0/0	0/0	0/0	0/0	0/0	0/0	0/0	1/1
Chromosome2-3083413	DEL	PRECISE	1342	0.787	0.787	0/0	0/1	0/0	0/0	0/0	0/0	0/0	0/0	0/1	0/1	1/1	1/1	1/1	0/0
Chromosome2-3083653	INS	PRECISE	9258	0	0	0/0	0/0	0/0	0/0	0/0	0/0	0/0	0/0	0/0	0/0	0/0	0/0	0/0	1/1
Chromosome2-3083776	DEL	PRECISE	1174	0	0	0/0	0/0	0/0	0/0	0/0	1/1	1/1	0/1	0/0	0/0	0/0	0/0	0/0	0/0
Chromosome2-3083811	INS	PRECISE	56	0	0.787	0/0	0/1	0/0	0/0	0/0	0/0	0/0	0/0	0/1	0/1	1/1	1/1	1/1	0/0
Chromosome2-3083945	INS	PRECISE	4490	0	0	0/0	0/0	1/1	0/0	0/0	0/0	0/0	0/0	0/0	0/0	0/0	0/0	0/0	0/0
Chromosome2-3084208	DEL	PRECISE	50	0	0	0/0	0/0	0/0	0/0	0/0	1/1	0/1	0/0	0/0	0/0	0/0	0/0	0/0	0/0
Chromosome2-3084368	DEL	PRECISE	701	0	0	0/0	0/1	0/1	0/0	0/1	0/0	0/0	0/0	0/1	0/1	1/1	1/1	1/1	1/1
Chromosome2-3084842	INS	PRECISE	92	0	0.354	0/0	0/1	0/0	0/1	0/0	0/0	0/0	0/0	0/1	0/1	1/1	1/1	1/1	0/1
Chromosome2-3085387	INS	PRECISE	377	0	0	0/0	0/0	0/1	0/0	0/0	0/0	0/0	0/0	0/0	0/0	0/0	0/0	0/0	0/0
Chromosome2-3086105	INS	PRECISE	65	0	0.378	0/0	0/1	0/0	0/0	0/0	0/0	0/0	0/0	0/0	0/1	1/1	1/1	1/1	0/1
Chromosome2-3086856	DEL	PRECISE	148	0	0	0/0	0/0	0/0	0/0	0/0	0/0	0/0	0/0	0/1	0/1	0/0	1/1	1/1	0/0
Chromosome2-3088018	DEL	PRECISE	51	0	0	0/0	0/0	0/0	0/0	0/0	0/0	0/0	0/0	0/0	0/0	0/1	0/0	0/0	0/0
Chromosome2-3088748	INS	PRECISE	1115	0	0	0/0	0/0	0/0	0/0	0/0	0/0	0/0	0/0	0/0	0/0	0/1	0/0	0/0	0/0
Chromosome2-3088896	BND	PRECISE	0	0	0	0/0	0/0	0/0	0/1	0/0	0/0	0/0	0/0	0/0	0/0	0/0	0/0	0/0	0/0
Chromosome2-3088896	BND	PRECISE	0	0	0	0/0	0/0	0/0	0/1	0/0	0/0	0/0	0/0	0/0	0/0	0/0	0/0	0/0	0/0
Chromosome2-3088980	INS	PRECISE	959	0	0	0/0	0/0	0/0	0/0	0/0	0/0	0/0	0/0	0/0	0/0	1/1	0/0	0/0	0/0
Chromosome2-3089008	DEL	PRECISE	186	0	0	0/0	0/0	0/0	0/0	0/0	0/0	0/0	0/0	0/0	0/0	1/1	0/0	0/0	0/0
Chromosome2-3090148	DEL	PRECISE	625	0	0	0/0	0/0	0/0	1/1	0/0	0/0	0/0	0/0	0/0	0/0	0/0	0/0	0/0	0/0
Chromosome2-3090749	DEL	PRECISE	1351	0	0	0/0	0/1	0/0	0/0	0/0	0/0	0/0	0/0	0/1	0/1	1/1	1/1	1/1	0/1
Chromosome2-3091730	INS	PRECISE	139	0	0	0/0	0/0	0/0	0/0	0/0	0/0	0/0	0/0	0/0	0/0	0/1	0/0	0/0	0/0
Chromosome2-3091830	DEL	PRECISE	55	0	0	0/0	0/0	0/0	0/0	0/0	0/0	0/0	0/0	0/0	0/0	0/0	0/0	0/0	0/1
Chromosome2-3092102	INS	PRECISE	62	0	0	0/0	0/0	0/0	0/0	0/1	0/0	0/0	0/0	0/0	0/0	0/0	0/0	0/0	0/0
Chromosome2-3092132	INS	PRECISE	1298	0	0.756	0/0	0/1	0/0	0/0	0/0	0/0	0/0	0/0	0/1	0/1	1/1	1/1	1/1	0/1
Chromosome2-3092304	INS	PRECISE	123	0	0.5	0/0	0/0	0/0	0/0	0/1	0/0	0/1	0/1	0/0	0/0	0/0	0/0	0/0	0/1
Chromosome2-3093117	BND	PRECISE	0	0	0	0/0	0/0	0/0	0/0	0/0	0/0	0/0	0/0	0/0	0/1	0/0	0/1	0/0	0/0
Chromosome2-3093164	INS	PRECISE	84	0	0	0/0	0/0	0/0	0/0	0/0	0/0	0/0	0/0	0/0	0/0	0/0	0/0	0/0	0/1
Chromosome2-3093342	DEL	PRECISE	784	0	0	0/0	0/1	0/0	0/0	0/0	0/0	0/0	0/0	0/1	0/1	1/1	1/1	1/1	0/1
Chromosome2-3094086	INS	PRECISE	2484	0	0	0/0	0/0	0/0	0/0	0/1	0/0	0/0	0/0	0/0	0/0	0/0	0/0	0/0	0/0
Chromosome2-3094264	DEL	PRECISE	55	0	0	0/0	0/1	0/0	0/0	0/0	0/0	0/0	0/0	0/1	0/1	1/1	1/1	1/1	1/1
Chromosome2-3094672	BND	PRECISE	0	0	0	0/0	0/0	0/0	0/0	0/0	0/0	0/0	0/0	0/0	0/0	0/1	0/0	0/0	0/0
Chromosome2-3095172	DEL	PRECISE	54	0	0	0/0	0/1	0/0	0/0	0/0	0/0	0/0	0/0	0/1	0/1	1/1	1/1	1/1	0/1
Chromosome2-3095412	INS	PRECISE	1082	0	0	0/0	0/0	0/1	0/0	0/0	0/0	0/0	0/0	0/0	0/0	0/0	0/0	0/0	0/0
Chromosome2-3095801	DEL	PRECISE	52	0	0	0/0	0/0	0/1	0/0	0/0	0/0	0/0	0/0	0/0	0/0	0/0	0/0	0/0	0/0
Chromosome2-3096901	INS	PRECISE	4941	0	0	0/0	0/0	0/0	0/0	0/0	0/0	1/1	1/1	0/0	0/0	0/0	0/0	0/0	0/0
Chromosome2-3099420	DEL	PRECISE	70	0	0	0/0	0/1	0/0	0/0	0/0	0/0	0/0	0/0	0/0	0/0	1/1	0/0	0/1	0/1
Chromosome2-3100358	INS	PRECISE	995	0	0	0/0	0/0	0/0	0/0	0/1	0/0	0/0	0/0	0/0	0/0	0/0	0/0	0/0	0/0
Chromosome2-3100640	INS	PRECISE	528	0	0	0/0	0/0	0/1	0/0	0/0	0/0	0/0	0/0	0/0	0/0	0/0	0/0	0/0	0/0
Chromosome2-3101612	INS	PRECISE	107	0	0	0/0	0/1	0/0	0/0	0/0	0/0	0/0	0/0	0/1	0/1	1/1	1/1	1/1	0/1
Chromosome2-3101904	INS	PRECISE	5307	0	0	0/0	0/0	0/0	0/0	0/1	0/0	0/0	0/0	0/0	0/0	0/0	0/0	0/0	0/0
Chromosome2-3101920	INS	PRECISE	211	0	0.378	0/0	0/1	0/0	0/0	0/0	0/0	0/0	0/0	0/1	0/1	1/1	1/1	1/1	0/1
Chromosome2-3101971	DEL	PRECISE	147	0	0	0/0	0/0	0/0	0/0	0/0	0/1	0/1	0/0	0/0	0/0	0/0	0/0	0/0	0/0
Chromosome2-3102057	INS	PRECISE	76	0	0	0/0	0/1	0/0	0/0	0/0	0/0	0/0	0/0	0/1	0/1	1/1	1/1	1/1	0/1
Chromosome2-3103331	INS	PRECISE	629	0	0	0/0	0/0	0/1	0/0	0/0	0/0	0/0	0/0	0/0	0/0	0/0	0/0	0/0	0/0
Chromosome2-3103509	DEL	PRECISE	620	0	0	0/0	1/1	0/0	0/0	0/0	0/0	0/0	0/0	0/0	0/0	0/0	0/0	0/0	0/1
Chromosome2-3103728	DEL	PRECISE	619	0	0	0/0	1/1	0/0	0/0	0/0	0/0	0/0	0/0	0/1	0/0	1/1	1/1	1/1	1/1
Chromosome2-3104244	INS	PRECISE	5000	0	0	0/0	0/0	0/0	0/1	0/0	0/0	0/0	0/0	0/0	0/0	0/0	0/0	0/0	0/0
Chromosome2-3104298	INS	PRECISE	848	0	0	0/0	0/0	0/1	0/0	0/0	0/0	0/0	0/0	0/0	0/0	0/0	0/0	0/0	0/0
Chromosome2-3106204	INS	PRECISE	1472	0	0	0/0	0/0	0/0	0/0	0/1	0/0	0/0	0/0	0/0	0/0	0/0	0/0	0/0	0/0
Chromosome2-3108149	BND	PRECISE	0	0	0	0/0	0/0	0/0	0/1	0/0	0/0	0/0	0/0	0/0	0/0	0/0	0/0	0/0	0/0
Chromosome2-3108346	INS	PRECISE	146	0	0	0/0	0/0	0/1	0/0	0/0	0/0	0/0	0/0	0/0	0/0	0/0	0/0	0/0	0/0
Chromosome2-3108430	DEL	PRECISE	1259	0	0	0/0	0/1	0/0	0/0	0/0	0/0	0/0	0/0	0/0	0/0	0/1	0/0	0/1	0/1
Chromosome2-3110252	INS	PRECISE	96	0	0.378	0/0	0/1	0/0	0/0	0/0	0/0	0/0	0/0	0/1	0/1	1/1	1/1	1/1	0/1
Chromosome2-3111342	DEL	PRECISE	747	0	0	0/0	0/0	0/0	0/0	0/1	0/0	0/0	0/0	0/0	0/0	1/1	0/0	0/0	0/0
Chromosome2-3111579	DEL	PRECISE	10353	0	0	0/0	0/0	0/0	0/1	0/0	0/0	0/0	0/0	0/0	0/0	0/0	0/0	0/0	0/0

Chromosome2-3111945	DEL	PRECISE	1329	0	0	0/0	0/1	0/0	0/0	0/0	0/0	0/0	0/0	0/1	0/1	1/1	1/1	1/1	0/1
Chromosome2-3112055	DEL	PRECISE	5799	0	0	0/0	0/0	0/0	0/0	0/1	0/0	0/0	0/0	0/0	0/0	0/0	0/0	0/0	0/0
Chromosome2-3112143	INV	PRECISE	5626391	0	0	0/0	0/0	0/0	0/0	0/0	0/0	0/0	0/1	0/0	0/0	0/0	0/1	0/0	0/0
Chromosome2-3112692	DEL	PRECISE	19855	0	0	0/0	0/1	0/0	0/0	0/0	0/0	0/0	0/0	1/1	1/1	1/1	1/1	0/0	1/1
Chromosome2-3112811	INS	PRECISE	161	0	0	0/0	0/0	0/0	0/0	0/0	0/0	0/1	0/1	0/0	0/0	0/0	0/0	0/0	0/0
Chromosome2-3112840	DEL	PRECISE	17488	0	0	0/0	0/0	0/0	0/0	0/1	0/0	0/0	0/0	0/0	0/0	0/0	0/0	0/0	0/0
Chromosome2-3114169	INS	PRECISE	1554	0	0	0/0	0/0	0/0	0/1	0/0	0/0	0/0	0/0	0/0	0/0	0/0	0/0	0/0	0/0
Chromosome2-3114908	BND	PRECISE	0	0	0	0/0	0/0	0/0	0/0	0/0	0/0	0/0	0/0	0/1	0/1	0/0	0/0	0/0	0/0
Chromosome2-3115722	INS	PRECISE	1212	0	0.976	0/0	0/1	0/0	0/0	0/0	0/0	0/0	0/0	0/1	0/1	1/1	1/1	1/1	0/1
Chromosome2-3116069	INS	PRECISE	477	0	0	0/0	0/0	0/0	0/0	0/0	0/0	0/0	0/0	0/0	0/0	0/0	0/0	0/0	1/1
Chromosome2-3116668	DEL	PRECISE	9900	0	0	0/0	0/1	0/0	0/0	0/0	0/0	0/0	0/0	1/1	1/1	0/0	0/0	0/0	0/1
Chromosome2-3116742	DEL	PRECISE	1010	0	0	0/0	0/0	0/0	0/0	0/1	0/0	0/0	0/0	0/0	0/0	0/0	0/0	0/0	0/0
Chromosome2-3117269	INS	PRECISE	12649	0	0	0/0	0/0	0/0	1/1	0/0	0/0	0/0	0/0	0/0	0/0	0/0	0/0	0/0	0/0
Chromosome2-3117941	INS	PRECISE	3791	0	0	0/0	0/0	0/0	0/0	1/1	0/0	0/0	0/0	0/0	0/0	0/0	0/0	0/0	0/0
Chromosome2-3118214	INS	PRECISE	5593	0	0	0/0	0/0	0/0	0/0	1/1	0/0	0/0	0/0	0/0	0/0	0/0	0/0	0/0	0/0
Chromosome2-3118215	BND	PRECISE	0	0	0	0/0	0/0	0/0	0/0	0/1	0/0	0/0	0/0	0/0	0/0	0/0	0/0	0/0	0/0
Chromosome2-3118388	DEL	PRECISE	1024	0.832	0.277	0/0	0/1	0/1	0/1	0/1	0/1	1/1	0/1	0/1	0/1	1/1	1/1	1/1	1/1
Chromosome2-3118507	INS	PRECISE	887	0	0	0/0	0/0	0/1	0/0	0/0	0/0	0/0	0/0	0/0	0/0	0/0	0/0	0/0	0/0
Chromosome2-3118539	DEL	PRECISE	9954	0	0	0/0	0/1	0/1	0/0	0/1	0/0	0/0	0/0	0/1	0/1	1/1	1/1	1/1	0/1
Chromosome2-3120026	DEL	PRECISE	994	0	0	0/0	0/0	0/0	0/0	0/0	0/0	0/0	0/0	0/0	0/0	0/0	0/0	0/0	0/1
Chromosome2-3120152	INS	PRECISE	64	0	0	0/0	0/0	0/0	0/0	0/0	0/0	0/0	0/0	0/0	0/0	0/1	0/0	0/0	0/0
Chromosome2-3120597	DEL	PRECISE	2220	0	0	0/0	0/1	0/0	0/0	0/0	0/0	0/0	0/0	0/1	0/1	1/1	0/1	1/1	0/1
Chromosome2-3120813	DEL	PRECISE	13347	0	0	0/0	0/0	0/0	0/0	0/1	0/0	0/0	0/0	0/0	0/0	0/0	0/0	0/0	0/0
Chromosome2-3121089	DEL	PRECISE	5240	0.354	0.354	0/0	0/1	0/1	0/0	0/0	0/0	0/0	0/0	0/1	0/1	1/1	1/1	1/1	0/1
Chromosome2-3121966	DEL	PRECISE	56	0	0	0/0	0/1	0/0	0/0	0/0	0/0	0/0	0/0	0/1	0/1	0/1	1/1	1/1	0/1
Chromosome2-3122152	INS	PRECISE	79	0	0	0/0	0/1	0/1	0/0	0/0	0/0	0/0	0/0	0/1	0/1	1/1	1/1	1/1	0/1
Chromosome2-3123898	INS	PRECISE	435	0	0	0/0	0/0	0/0	0/0	0/1	0/0	0/0	0/0	0/0	0/0	0/0	0/0	0/0	0/0
Chromosome2-3124162	DEL	PRECISE	237	0	0	0/0	0/1	0/0	0/0	0/0	0/0	0/0	0/0	0/1	0/1	1/1	1/1	1/1	0/1
Chromosome2-3124373	DEL	PRECISE	4169	0	0	0/0	0/1	0/0	0/0	0/0	0/0	0/0	0/0	0/1	0/1	1/1	1/1	1/1	0/1
Chromosome2-3124480	DEL	PRECISE	2424	0	0	0/0	0/0	0/0	0/0	0/0	0/0	0/1	0/1	0/0	0/0	0/0	0/0	0/0	0/0
Chromosome2-3124599	DEL	PRECISE	887	0	0	0/0	0/0	0/0	0/1	0/0	0/0	0/0	0/0	0/0	0/0	0/0	0/0	0/0	0/0
Chromosome2-3125020	DEL	PRECISE	22869	0	0	0/0	0/0	0/1	0/0	0/0	0/0	0/0	0/0	0/0	0/0	0/0	0/0	0/0	0/0
Chromosome2-3125855	INS	PRECISE	216	0	0.69	0/0	0/1	0/0	0/0	0/0	0/0	0/0	0/0	0/1	0/1	1/1	1/1	1/1	0/1
Chromosome2-3125969	DEL	PRECISE	206	0	0	0/0	0/0	0/0	0/0	0/0	0/0	0/0	0/0	0/1	0/1	0/0	1/1	0/1	0/0
Chromosome2-3127145	INS	PRECISE	1355	0	0	0/0	0/0	0/0	0/0	0/0	0/0	0/0	0/0	0/0	0/0	1/1	0/0	0/0	0/0
Chromosome2-3127198	DEL	PRECISE	1459	0	0	0/0	0/0	0/0	0/0	0/0	0/0	0/0	0/0	0/0	0/0	0/1	0/0	0/0	0/0
Chromosome2-3127658	INS	PRECISE	133	0	0	0/0	0/0	0/0	0/0	0/0	0/0	0/0	0/0	0/0	0/0	0/1	0/0	0/0	0/0
Chromosome2-3127846	INS	PRECISE	79	0	0	0/0	0/0	0/0	0/0	0/0	0/0	0/0	0/0	0/0	0/0	0/0	0/0	0/0	0/1
Chromosome2-3127893	DEL	PRECISE	200	0	0	0/0	0/1	0/1	0/0	0/0	0/0	0/0	0/0	0/0	0/0	1/1	1/1	1/1	0/1
Chromosome2-3128650	INS	PRECISE	1395	0	0	0/0	0/0	0/0	0/0	0/0	0/0	0/0	0/0	0/0	0/0	0/1	0/0	0/0	0/0
Chromosome2-3129643	INS	PRECISE	54	0	0	0/0	0/0	0/0	0/0	0/0	0/0	0/0	0/0	0/0	0/0	0/1	0/0	0/0	0/0
Chromosome2-3130162	INS	PRECISE	161	0	0	0/0	0/0	0/1	0/0	0/0	0/0	0/0	0/0	0/0	0/0	0/0	0/0	0/0	0/0
Chromosome2-3130574	DEL	PRECISE	59	0	0	0/0	0/1	0/0	0/0	0/0	0/0	0/0	0/0	0/0	0/0	1/1	0/0	0/1	1/1
Chromosome2-3131763	INS	PRECISE	7793	0	0	0/0	0/0	1/1	0/0	0/0	0/0	0/0	0/0	0/0	0/0	0/0	0/0	0/0	0/0
Chromosome2-3132005	DEL	PRECISE	2519	0	0	0/0	0/0	0/0	0/0	0/0	0/0	0/0	0/0	0/0	0/0	0/0	0/0	0/0	1/1
Chromosome2-3132193	DEL	PRECISE	72	0	0	0/0	0/0	0/0	0/0	0/0	0/0	0/1	0/1	0/0	0/0	0/0	0/0	0/0	0/0
Chromosome2-3132262	DEL	PRECISE	1111	0	0	0/0	0/0	0/0	0/0	0/0	0/0	0/0	0/0	0/0	0/0	0/0	0/0	0/0	1/1
Chromosome2-3133902	DEL	PRECISE	1408	0	0	0/1	0/1	1/1	1/1	1/1	1/1	1/1	1/1	1/1	1/1	1/1	1/1	1/1	1/1
Chromosome2-3134191	INS	PRECISE	493	0	0	0/0	0/0	0/0	0/0	0/1	0/0	0/0	0/0	0/0	0/0	0/0	0/0	0/0	0/0
Chromosome2-3134557	DEL	PRECISE	134	0	0	0/0	0/1	0/1	0/1	0/1	0/1	1/1	0/1	0/1	0/1	1/1	1/1	1/1	1/1
Chromosome2-3134902	INS	PRECISE	1394	0	0	0/0	0/0	0/1	0/0	0/0	0/0	0/0	0/0	0/0	0/0	0/0	0/0	0/0	0/0
Chromosome2-3134957	INS	PRECISE	426	0	0.577	0/0	0/1	0/0	0/0	0/0	0/0	0/0	0/0	0/1	0/1	0/1	1/1	1/1	0/1
Chromosome2-3135146	DEL	PRECISE	669	0	0	0/0	0/1	0/0	0/0	0/0	0/0	0/0	0/0	0/1	0/1	1/1	1/1	1/1	0/1
Chromosome2-3135232	DEL	PRECISE	72	0	0	0/0	0/0	0/0	0/0	0/1	0/1	0/0	0/1	0/1	0/1	0/0	0/0	0/0	0/0
Chromosome2-3135679	INS	PRECISE	848	0	0	0/0	0/0	0/0	0/0	0/0	0/0	0/0	0/0	0/0	0/0	0/0	0/0	0/0	0/1
Chromosome2-3135931	INS	PRECISE	130	0	0	0/0	0/0	0/0	0/1	1/1	0/1	0/0	0/1	0/1	0/1	0/0	0/0	0/0	0/0
Chromosome2-3136311	INS	PRECISE	10499	0	0	0/0	0/0	0/0	0/0	0/0	0/0	0/0	0/0	0/0	0/0	0/0	0/0	0/0	1/1
Chromosome2-3136513	DEL	PRECISE	52	0	0	0/0	0/1	0/0	0/0	0/0	0/0	0/0	0/0	0/1	0/1	0/1	1/1	1/1	0/1
Chromosome2-3136733	DEL	PRECISE	862	0	0	0/0	0/0	0/0	0/0	0/0	0/0	0/1	0/1	0/0	0/0	0/0	0/0	0/0	0/1
Chromosome2-3136886	DEL	PRECISE	355	0	0	0/0	0/1	0/1	0/1	0/1	0/1	0/1	0/0	0/1	0/1	1/1	1/1	1/1	1/1
Chromosome2-3137032	INS	PRECISE	1407	0	0	0/0	0/0	0/0	0/1	0/0	0/0	0/0	0/0	0/0	0/0	0/0	0/0	0/0	0/0
Chromosome2-3137327	DEL	PRECISE	383	0	0	0/0	0/1	0/0	0/0	0/0	0/0	0/0	0/0	0/0	0/0	0/1	0/0	0/1	0/1
Chromosome2-3137741	DEL	PRECISE	152	0	0	0/0	0/1	0/0	0/0	0/0	0/0	0/0	0/0	0/1	0/1	1/1	1/1	1/1	0/1
Chromosome2-3138451	INS	PRECISE	290	0	0.378	0/0	0/1	0/0	0/0	0/0	0/0	0/0	0/0	0/1	0/1	1/1	1/1	1/1	0/1
Chromosome2-3138539	INS	PRECISE	56	0	0	0/0	0/0	0/0	0/1	0/0	0/0	0/0	0/0	0/0	0/0	0/0	0/0	0/0	0/0
Chromosome2-3138958	INS	PRECISE	6532	0	0	0/0	0/0	0/0	0/0	0/0	0/0	0/0	0/0	0/0	0/0	0/0	0/0	0/0	1/1
Chromosome2-3139253	BND	PRECISE	0	0	0	0/0	0/0	0/0	0/0	0/1	0/0	0/0	0/0	0/0	0/0	0/0	0/0	0/0	0/0
Chromosome2-3140105	BND	PRECISE	0	0	0	0/0	0/0	0/1	0/0	0/1	0/1	0/0	0/0	0/0	0/0	0/0	0/0	0/0	0/0
Chromosome2-3140200	DEL	PRECISE	55	0	0	0/0	0/0	0/0	0/1	0/1	0/1	0/1	1/1	0/1	0/1	0/0	0/0	0/0	0/0
Chromosome2-3140262	INS	PRECISE	700	0	0	0/0	0/0	0/1	0/0	0/0	0/0	0/0	0/0	0/0	0/0	0/0	0/0	0/0	0/0
Chromosome2-3140470	INS	PRECISE	230	0	0.378	0/0	0/1	0/0	0/0	0/0	0/0	0/0	0/0	0/1	0/1	1/1	1/1	1/1	0/1
Chromosome2-3140707	DEL	PRECISE	678	0.667	0.667	0/0	0/1	0/1	0/0	0/1	0/0	0/0	0/0	0/1	0/1	1/1	1/1	1/1	0/1
Chromosome2-3140855	INS	PRECISE	677	0	0	0/0	0/0	0/0	0/0	0/0	0/0	0/0	0/0	0/0	0/0	0/0	0/0	0/0	0/1

Chromosome2-3141496	DEL	PRECISE	997	0	0	0/0	0/1	0/0	0/0	0/0	0/0	0/0	0/0	0/1	0/1	1/1	1/1	1/1	0/1
Chromosome2-3141596	DEL	PRECISE	568	0	0	0/0	0/0	0/1	0/0	0/1	0/0	0/0	0/0	0/0	0/0	0/0	0/0	0/0	0/0
Chromosome2-3141642	INS	PRECISE	80	0	0	0/0	0/1	0/0	0/0	0/0	0/0	0/0	0/0	0/1	0/1	1/1	1/1	1/1	0/1
Chromosome2-3142019	INS	PRECISE	366	0	0.488	0/0	0/1	0/0	0/0	0/0	0/0	0/0	0/0	0/1	0/1	1/1	1/1	0/1	0/1
Chromosome2-3142823	DEL	PRECISE	116	0	0	0/0	0/1	0/0	0/0	0/0	0/0	0/0	0/0	0/1	0/1	1/1	1/1	0/1	0/1
Chromosome2-3143124	DEL	PRECISE	628	0	0	0/0	0/0	0/0	0/0	0/0	0/0	0/0	0/0	0/0	0/0	0/0	0/0	0/1	0/1
Chromosome2-3143315	DEL	PRECISE	101	0	0	0/0	0/1	0/0	0/0	0/0	0/0	0/0	0/0	0/1	0/1	1/1	1/1	0/1	0/1
Chromosome2-3143437	INS	PRECISE	3573	0	0	0/0	0/0	0/0	0/0	0/0	0/0	0/0	0/0	0/0	0/0	1/1	0/0	0/0	0/0
Chromosome2-3143850	DEL	PRECISE	77	0	0	0/0	0/0	0/1	0/0	0/0	0/0	0/0	0/0	0/0	0/0	0/0	0/0	0/0	0/0
Chromosome2-3144522	INS	PRECISE	12057	0	0	0/0	0/1	0/0	0/0	0/0	0/0	0/0	0/0	0/0	0/0	0/0	0/0	0/0	0/0
Chromosome2-3144529	DEL	PRECISE	290	0.316	0.316	0/0	0/0	0/1	0/1	0/1	0/1	0/0	0/1	1/1	1/1	0/0	1/1	1/1	1/1
Chromosome2-3144551	BND	PRECISE	0	0	0	0/0	0/0	0/0	0/0	0/0	0/0	0/0	0/0	0/0	0/0	0/1	0/1	0/0	0/0
Chromosome2-3144637	INS	PRECISE	480	0	0.577	0/0	0/0	0/0	0/1	0/0	0/1	0/1	0/0	0/0	0/0	0/0	0/0	0/0	0/0
Chromosome2-3145734	INS	PRECISE	9279	0	0	0/0	0/0	0/0	0/0	0/0	0/0	0/0	0/0	0/0	0/0	0/0	0/0	0/0	0/0
Chromosome2-3146367	DEL	PRECISE	93	0	0	0/0	0/0	0/0	0/0	0/0	0/0	0/0	0/0	0/1	0/0	0/1	0/1	1/1	0/0
Chromosome2-3146476	INS	PRECISE	573	0	0	0/0	0/0	0/0	0/0	0/0	0/0	0/1	0/1	0/0	0/0	0/0	0/0	0/0	0/0
Chromosome2-3146520	DEL	PRECISE	2593	0	0	0/0	0/0	0/0	0/0	0/0	0/0	0/0	0/0	0/0	0/0	0/1	0/0	0/0	0/0
Chromosome2-3146528	DEL	PRECISE	1188	0	0	0/0	0/1	0/0	0/0	0/0	0/0	0/0	0/0	0/1	0/1	0/1	1/1	0/1	0/1
Chromosome2-3148179	DEL	PRECISE	50	0	0	0/0	0/1	0/0	0/0	0/0	0/0	0/0	0/0	0/1	0/1	1/1	1/1	0/1	0/1
Chromosome2-3149384	INS	PRECISE	679	0	0	0/0	0/0	0/1	0/0	0/0	0/0	0/0	0/0	0/0	0/0	0/0	0/0	0/0	0/0
Chromosome2-3151164	DEL	PRECISE	80	0	0	0/0	0/1	0/0	0/0	0/0	0/0	0/0	0/0	0/1	0/1	1/1	1/1	0/0	0/1
Chromosome2-3151246	INS	PRECISE	233	0	0	0/0	0/0	0/1	0/0	0/0	0/0	0/0	0/0	0/0	0/0	0/0	0/0	0/0	0/0
Chromosome2-3151384	INS	PRECISE	1973	0	0	0/0	0/0	0/1	0/0	0/0	0/0	0/0	0/0	0/0	0/0	0/0	0/0	0/0	0/0
Chromosome2-3151565	INS	PRECISE	326	0	0	0/0	0/0	0/0	0/1	0/0	0/0	0/0	0/0	0/0	0/0	0/0	0/0	0/0	0/0
Chromosome2-3152417	DEL	PRECISE	305	0	0	0/0	0/0	0/0	0/0	0/0	0/0	0/0	0/0	0/1	0/0	0/0	0/1	0/1	0/0
Chromosome2-3152423	BND	PRECISE	0	0	0	0/0	0/0	0/0	0/0	0/0	0/0	0/0	0/0	0/0	0/0	1/1	0/0	0/0	0/1
Chromosome2-3153200	INS	PRECISE	1345	0	0	0/0	0/0	0/0	0/1	0/0	0/1	0/1	0/0	0/0	0/0	0/0	0/0	0/0	0/0
Chromosome2-3154081	DEL	PRECISE	2990	0	0	0/0	0/0	0/0	0/0	0/0	0/0	0/0	0/0	0/0	0/0	0/0	0/0	1/1	1/1
Chromosome2-3154117	INS	PRECISE	349	0	0.951	0/0	0/0	0/0	1/1	0/1	1/1	0/1	0/1	1/1	1/1	0/0	0/0	0/0	0/0
Chromosome2-3154176	INS	PRECISE	2532	0	0.707	0/0	0/0	0/0	0/0	0/0	0/0	1/1	1/1	0/0	0/0	0/0	0/0	0/0	0/0
Chromosome2-3154407	DEL	PRECISE	68	0	0	0/0	0/0	0/0	0/0	0/0	0/0	0/0	0/0	0/0	0/0	0/0	0/0	1/1	0/0
Chromosome2-3154703	DEL	PRECISE	884	0	0	0/0	0/0	0/0	0/1	1/1	0/1	0/0	0/1	0/1	0/1	0/0	0/0	0/0	0/0
Chromosome2-3154728	DEL	PRECISE	98	0	0	0/0	0/0	0/0	0/1	0/0	0/1	0/1	0/0	0/0	0/0	0/0	0/0	0/0	0/0
Chromosome2-3154924	BND	PRECISE	0	0	0	0/0	0/0	0/1	0/0	0/0	0/0	0/0	0/0	0/0	0/0	0/0	0/0	0/0	0/0
Chromosome2-3157104	INS	PRECISE	137	0	0	0/0	0/0	0/0	0/0	0/0	0/0	0/0	0/0	0/1	0/0	0/0	0/1	0/1	0/0
Chromosome2-3157140	DEL	PRECISE	52	0	0	0/0	0/0	0/0	0/0	0/0	0/0	0/0	0/0	0/1	0/0	0/0	0/1	0/1	0/0
Chromosome2-3157789	INS	PRECISE	211	0	0	0/0	0/0	0/0	0/0	0/1	0/0	0/0	0/0	0/0	0/0	0/0	0/0	0/0	0/0
Chromosome2-3157895	DEL	PRECISE	213	0	0	0/0	0/1	0/1	0/1	0/1	0/1	0/1	0/0	0/1	0/1	1/1	1/1	1/1	1/1
Chromosome2-3159472	INS	PRECISE	74	0	0.707	0/0	0/0	0/0	0/0	0/0	0/0	0/0	0/0	0/0	0/1	0/0	0/1	0/0	0/0
Chromosome2-3159558	DEL	PRECISE	61	0	0	0/0	0/0	0/0	0/0	0/0	0/0	0/0	0/0	0/0	0/0	0/0	0/0	0/1	1/1
Chromosome2-3159617	INS	PRECISE	1680	0	0	0/0	0/0	0/1	0/0	0/0	0/0	0/0	0/0	0/0	0/0	0/0	0/0	0/0	0/0
Chromosome2-3160083	DEL	PRECISE	285	0.577	0.577	0/0	0/0	0/0	0/0	0/0	0/0	0/0	0/0	0/1	0/0	0/0	0/0	0/1	0/0
Chromosome2-3160464	DEL	PRECISE	3102	0	0	0/0	0/1	0/0	0/0	0/0	0/0	0/0	0/0	0/0	0/1	1/1	1/1	0/0	0/1
Chromosome2-3160716	INS	PRECISE	615	0	0	0/0	0/0	0/1	0/0	0/0	0/0	0/0	0/0	0/0	0/0	0/0	0/0	0/0	0/0
Chromosome2-3160965	INS	PRECISE	4531	0	0	0/0	0/0	0/0	0/0	1/1	0/0	0/0	0/0	0/0	0/0	0/0	0/0	0/0	0/0
Chromosome2-3161001	DEL	PRECISE	54	0	0	0/0	0/1	0/0	0/0	0/0	0/0	0/0	0/0	0/0	0/1	0/1	1/1	0/0	0/1
Chromosome2-3161329	INS	PRECISE	5593	0	0	0/0	0/0	0/0	0/0	0/0	0/0	0/0	0/0	0/0	0/0	0/0	0/0	1/1	0/0
Chromosome2-3161490	INS	PRECISE	120	0	0.577	0/0	0/0	0/0	1/1	0/0	0/0	0/1	0/1	0/0	0/0	0/0	0/0	0/0	0/0
Chromosome2-3161566	INS	PRECISE	361	0	0	0/0	0/0	0/0	0/0	0/1	0/0	0/0	0/0	0/0	0/0	0/0	0/0	0/0	0/0
Chromosome2-3161671	INS	PRECISE	202	0	0	0/0	0/0	0/0	0/0	0/1	0/0	0/0	0/0	0/0	0/0	0/0	0/0	0/0	0/0
Chromosome2-3162066	DEL	PRECISE	254	0	0	0/0	0/0	0/0	0/0	0/0	0/0	0/0	0/0	0/0	0/0	0/0	0/0	0/0	0/1
Chromosome2-3162324	INS	PRECISE	9981	0	0	0/0	0/0	0/0	0/0	0/0	0/0	0/0	0/0	0/0	0/0	0/0	0/0	0/0	0/1
Chromosome2-3162325	BND	PRECISE	0	0	0	0/0	0/0	0/0	0/0	0/0	0/0	0/0	0/0	0/0	0/0	0/0	0/0	0/0	0/1
Chromosome2-3162336	DEL	PRECISE	372	0	0	0/0	0/0	0/0	0/0	0/0	0/0	0/0	0/0	0/0	0/1	0/0	0/1	0/0	0/0
Chromosome2-3162538	INV	PRECISE	927599	0	0.707	0/0	0/0	0/0	0/0	0/0	0/0	0/0	0/0	0/0	0/0	0/0	0/0	0/1	0/0
Chromosome2-3163551	DEL	PRECISE	965	0	0	0/0	0/0	0/0	0/0	0/0	0/0	0/1	0/1	0/0	0/0	0/0	0/0	0/0	0/0
Chromosome2-3163558	BND	PRECISE	0	0	0	0/0	0/0	0/0	0/0	0/0	0/0	0/0	0/0	0/0	0/0	0/0	0/0	0/1	0/0
Chromosome2-3163561	BND	PRECISE	0	0	0	0/0	0/0	0/0	0/0	0/0	0/0	0/0	0/0	0/0	0/0	0/0	0/0	0/0	0/1
Chromosome2-3163585	BND	PRECISE	0	0	0	0/0	0/0	0/0	0/0	0/0	0/0	0/0	0/0	0/0	0/0	0/1	0/0	0/0	0/1
Chromosome2-3163806	INS	PRECISE	426	0	0.707	0/0	0/0	0/0	0/0	0/0	0/0	0/0	0/0	0/0	0/1	0/0	0/1	0/0	0/0
Chromosome2-3166235	DEL	PRECISE	69	0	0	0/0	0/0	0/0	0/0	0/0	0/0	0/0	0/0	0/0	0/0	0/0	0/0	0/1	0/1
Chromosome2-3166261	DEL	PRECISE	1254	0	0	0/0	0/0	0/0	0/0	0/1	0/0	0/0	0/0	0/0	0/0	0/0	0/0	0/0	0/0
Chromosome2-3166786	INS	PRECISE	898	0	0	0/0	0/0	0/0	0/0	0/0	0/0	0/0	0/0	0/0	0/0	0/1	0/0	0/0	0/0
Chromosome2-3168170	DEL	PRECISE	56	0	0	0/0	0/0	0/0	0/0	0/0	0/1	0/1	0/0	0/0	0/0	0/0	0/0	0/0	1/1
Chromosome2-3169127	BND	PRECISE	0	0	0	0/0	0/0	0/1	0/0	0/0	0/0	0/0	0/0	0/0	0/0	0/0	0/0	0/0	0/0
Chromosome2-3169973	BND	PRECISE	0	0	0	0/0	0/0	0/0	0/0	0/0	0/1	0/1	0/0	0/0	0/0	0/0	0/0	0/0	0/0
Chromosome2-3170367	DEL	PRECISE	111	0	0	0/0	0/0	0/0	0/0	0/1	0/1	1/1	0/0	0/0	0/0	0/0	0/0	0/0	0/0
Chromosome2-3171668	DEL	PRECISE	1550	0	0	0/0	0/0	0/0	0/0	0/0	0/0	0/0	0/0	0/0	0/0	1/1	0/0	0/0	0/0
Chromosome2-3172285	INS	PRECISE	5911	0	0	0/0	0/0	0/0	0/0	0/0	0/0	0/0	0/0	0/0	0/0	0/0	0/0	0/0	0/0
Chromosome2-3172817	DEL	PRECISE	3133	0	0	0/0	0/0	0/0	0/0	0/0	0/0	0/0	0/0	0/0	0/0	0/0	0/0	0/0	0/1
Chromosome2-3172823	DEL	PRECISE	1457	0	0	0/0	0/1	0/0	0/0	0/0	0/0	0/0	0/0	0/1	0/1	1/1	1/1	0/1	0/1
Chromosome2-3173058	DEL	PRECISE	51	0	0	0/0	0/0	0/0	0/0	0/0	0/0	0/0	0/0	0/0	0/0	0/1	0/0	0/0	0/0
Chromosome2-3173640	INS	PRECISE	53	0	0	0/0	0/0	0/0	0/0	0/0	0/0	0/0	0/0	0/0	0/0	0/0	0/0	0/0	0/1

Chromosome2-3173885	DEL	PRECISE	969	0	0	0/0	0/0	0/0	0/0	0/0	0/0	0/0	0/0	0/0	0/0	0/0	0/0	0/0	0/1
Chromosome2-3173966	DEL	PRECISE	2995	0	0	0/0	0/0	0/0	0/0	0/0	0/0	0/0	0/0	0/0	0/0	0/0	0/0	0/0	0/1
Chromosome2-3174269	DEL	PRECISE	1259	0	0	0/0	0/0	0/0	0/0	0/0	0/0	0/0	0/0	0/0	0/0	0/0	0/0	0/0	0/1
Chromosome2-3174483	DEL	PRECISE	473	0	0	0/0	0/0	0/0	0/0	0/0	0/0	0/0	0/0	0/0	0/0	0/0	0/0	0/0	0/1
Chromosome2-3175238	DEL	PRECISE	136	0.408	0.408	0/0	0/0	0/0	0/0	0/1	0/1	0/0	0/0	0/0	0/1	0/0	1/1	0/0	0/0
Chromosome2-3175942	BND	PRECISE	0	0	0	0/0	0/0	0/0	0/0	0/1	1/1	0/0	0/1	0/0	0/0	0/0	0/0	0/0	0/0
Chromosome2-3176020	DEL	PRECISE	113	0	0	0/0	0/0	0/0	0/0	0/0	0/0	0/0	0/0	0/0	0/0	0/0	0/0	1/1	0/0
Chromosome2-3176501	DEL	PRECISE	173032	0	0	0/0	0/0	0/0	1/1	0/0	0/0	0/0	0/0	0/0	0/0	0/0	0/0	0/0	0/0
Chromosome2-3176613	DEL	PRECISE	4759	0	0	0/0	0/0	0/0	0/0	0/0	0/0	0/0	0/0	0/0	0/0	0/0	0/0	1/1	0/0
Chromosome2-3177326	DUP	PRECISE	154384	0	0	0/0	0/0	0/0	0/0	0/1	0/1	0/0	0/0	0/1	0/1	0/0	0/0	0/0	0/0
Chromosome2-3178395	DEL	PRECISE	134	0	0	0/0	0/0	0/0	0/0	0/0	0/0	0/0	0/0	0/0	0/0	1/1	0/0	0/0	0/0
Chromosome2-3178526	DEL	PRECISE	741	0	0	0/0	0/0	0/0	0/0	0/0	0/0	0/0	0/0	0/0	0/0	1/1	0/0	0/0	0/0
Chromosome2-3178901	BND	PRECISE	0	0	0	0/0	0/0	0/0	0/0	0/0	0/0	0/0	0/0	0/0	0/0	0/1	0/0	0/0	0/0
Chromosome2-3179823	DEL	PRECISE	908	0	0	0/0	0/0	0/0	0/0	0/0	0/0	0/0	0/0	0/0	0/0	0/0	0/0	0/0	0/1
Chromosome2-3179925	BND	PRECISE	0	0	0	0/0	0/0	0/0	0/1	0/0	0/0	0/1	0/0	0/0	0/0	0/0	0/0	0/0	0/0
Chromosome2-3180109	DEL	PRECISE	5025	0	0	0/0	0/0	0/1	1/1	0/1	0/0	1/1	0/1	0/0	0/0	0/0	0/0	0/0	1/1
Chromosome2-3180733	DEL	PRECISE	75	0	0	0/0	0/0	0/0	0/0	0/0	0/0	0/0	0/0	0/0	0/0	0/0	0/0	0/0	0/1
Chromosome2-3181253	BND	PRECISE	0	0	0	0/0	0/0	0/0	0/0	0/0	0/0	0/0	0/0	0/0	0/0	0/0	0/0	0/0	0/1
Chromosome2-3183369	INS	PRECISE	11186	0	0	0/0	0/0	0/0	0/0	0/0	0/0	0/0	0/0	0/0	0/0	0/0	0/0	0/0	0/0
Chromosome2-3183924	DEL	PRECISE	283	0	0	0/0	0/0	0/0	0/0	0/0	0/1	1/1	0/0	0/0	0/0	0/0	0/0	0/0	0/0
Chromosome2-3184410	INS	PRECISE	53	0	0	0/0	0/0	0/0	0/0	0/0	0/0	0/0	0/0	0/0	0/0	0/0	0/0	0/0	1/1
Chromosome2-3184735	DEL	PRECISE	1884	0	0	0/0	0/0	0/0	0/0	0/0	0/0	0/0	0/0	0/0	0/0	0/0	0/0	0/0	1/1
Chromosome2-3184927	DEL	PRECISE	1269	0	0	0/0	0/0	0/0	0/0	0/0	0/0	0/0	0/0	0/0	0/0	0/0	0/0	0/0	1/1
Chromosome2-3185159	DUP	PRECISE	6456	0	0	0/0	0/0	0/0	0/0	0/0	0/1	0/0	0/1	0/1	0/1	0/0	0/0	0/0	0/0
Chromosome2-3185182	BND	PRECISE	0	0	0	0/0	0/0	0/0	0/0	0/0	0/0	0/0	0/0	0/1	0/0	0/0	0/0	0/0	0/0
Chromosome2-3185631	INS	PRECISE	120	0	0	0/0	0/0	0/0	0/0	1/1	1/1	1/1	1/1	1/1	1/1	0/0	0/0	0/0	0/0
Chromosome2-3185716	DEL	PRECISE	329	0	0	0/0	0/0	0/0	0/0	1/1	1/1	1/1	1/1	1/1	1/1	0/0	0/0	0/0	0/0
Chromosome2-3187697	DEL	PRECISE	42424	0	0	0/0	0/0	0/0	0/0	0/1	1/1	0/0	0/0	0/0	0/0	0/0	0/0	0/0	0/0
Chromosome2-3188889	DEL	PRECISE	676	0	0	0/0	0/0	0/0	0/0	1/1	1/1	1/1	0/0	0/0	1/1	0/0	1/1	1/1	1/1
Chromosome2-3189858	INS	PRECISE	864	0	0.707	0/0	0/0	0/0	0/0	0/0	0/0	0/0	0/0	0/0	0/0	0/0	0/0	0/1	0/0
Chromosome2-3191859	BND	PRECISE	0	0	0	0/0	0/0	0/0	0/0	0/0	0/0	0/0	0/0	0/0	0/0	0/0	0/1	0/1	0/0
Chromosome2-3192363	DEL	PRECISE	53	0	0	0/0	0/0	0/0	0/0	0/0	0/0	0/0	0/0	0/0	0/0	0/0	0/0	0/1	0/0
Chromosome2-3192565	INS	PRECISE	4608	0	0	0/0	0/0	0/0	0/0	0/0	0/0	0/0	0/0	0/0	0/0	0/0	0/0	0/1	0/0
Chromosome2-3192579	DEL	PRECISE	9865	0	0	0/0	0/0	0/0	0/0	0/0	0/0	0/0	0/0	0/0	0/0	0/0	0/0	0/1	0/0
Chromosome2-3192685	INS	PRECISE	5520	0	0	0/0	0/0	0/0	0/0	0/1	0/0	0/0	0/0	0/0	0/0	0/0	0/0	0/0	0/0
Chromosome2-3192801	INS	PRECISE	887	0	0	0/0	0/0	0/0	0/0	0/0	0/0	0/0	0/0	0/0	0/0	0/0	0/0	0/0	1/1
Chromosome2-3192999	DEL	PRECISE	80	0	0	0/0	0/0	0/0	0/0	0/0	0/0	0/0	0/0	0/0	0/0	0/0	0/0	0/1	0/0
Chromosome2-3193136	BND	PRECISE	0	0	0	0/0	0/0	0/0	1/1	0/0	0/0	1/1	0/0	0/0	0/0	0/0	0/0	0/0	0/0
Chromosome2-3193161	INS	PRECISE	288	0	0	0/0	0/0	0/0	0/0	0/0	0/0	0/0	0/0	0/0	0/0	0/0	0/0	0/1	0/0
Chromosome2-3193161	BND	PRECISE	0	0	0	0/0	0/0	0/1	0/0	0/0	0/0	0/0	0/0	0/0	0/0	0/0	0/0	0/0	0/0
Chromosome2-3193300	BND	PRECISE	0	0	0	0/0	0/0	0/1	0/0	0/0	0/0	0/0	0/0	0/0	0/0	0/0	0/0	0/0	0/0
Chromosome2-3193540	INS	PRECISE	1200	0	0	0/0	0/0	0/0	0/0	0/0	0/0	0/0	0/0	0/0	0/0	0/0	0/0	0/0	1/1
Chromosome2-3193963	BND	PRECISE	0	0	0	0/0	0/0	0/0	0/0	0/0	0/0	0/0	0/0	0/0	0/0	0/1	0/0	0/0	0/0
Chromosome2-3193986	DEL	PRECISE	141	0	0	0/0	0/0	0/1	1/1	0/0	0/0	1/1	1/1	0/0	0/0	0/0	0/0	0/0	0/0
Chromosome2-3194296	INS	PRECISE	5044	0	0	0/0	0/0	0/0	0/0	0/0	0/0	0/0	0/0	0/0	0/0	0/1	0/0	0/0	0/0
Chromosome2-3194302	DEL	PRECISE	181	0.5	0.5	0/0	0/0	0/0	1/1	0/0	0/0	1/1	1/1	0/0	0/0	0/0	0/0	0/0	0/0
Chromosome2-3194341	INS	PRECISE	180	0.577	0.577	0/0	0/0	0/0	1/1	0/0	0/0	1/1	1/1	0/0	0/0	0/0	0/0	0/0	0/0
Chromosome2-3194496	INS	PRECISE	301	0	0.447	0/0	0/1	0/0	0/0	0/0	0/0	0/0	0/0	0/0	1/1	1/1	1/1	0/0	1/1
Chromosome2-3194615	DEL	PRECISE	3857	0	0	0/0	0/0	0/0	0/0	0/0	0/0	0/0	0/0	0/0	0/0	1/1	0/0	0/0	0/0
Chromosome2-3197345	BND	PRECISE	0	0	0	0/0	0/0	0/0	0/0	0/0	0/0	0/0	0/1	0/0	0/0	0/0	0/0	0/0	0/0
Chromosome2-3197470	INS	PRECISE	5341	0	0	0/0	0/0	0/0	0/0	0/0	0/0	0/0	0/0	0/0	0/0	0/1	0/0	0/0	0/0
Chromosome2-3197687	DEL	PRECISE	1282	0	0	0/0	0/0	0/0	0/0	0/0	0/0	0/1	0/0	0/0	0/0	0/0	0/0	0/0	0/0
Chromosome2-3197694	INS	PRECISE	1293	0	0.707	0/0	0/0	0/0	0/0	0/0	0/0	0/0	0/0	0/1	0/0	0/0	0/0	0/0	0/0
Chromosome2-3197938	DEL	PRECISE	460	0	0	0/0	0/0	0/0	0/0	0/1	0/0	0/0	0/0	0/0	0/0	0/0	0/0	0/0	0/0
Chromosome2-3197963	INS	PRECISE	63	0	0	0/0	0/0	0/1	0/1	0/0	0/0	0/0	0/0	0/0	0/0	0/0	0/0	0/1	0/0
Chromosome2-3198334	INS	PRECISE	54	0	0	0/0	0/0	0/0	0/0	0/1	0/0	0/0	0/0	0/0	0/0	0/0	0/0	0/0	0/0
Chromosome2-3198430	DEL	PRECISE	672	0	0	0/0	0/0	0/1	1/1	1/1	1/1	1/1	1/1	0/1	0/1	0/0	0/0	0/1	1/1
Chromosome2-3198472	INS	PRECISE	63	0	0.378	0/0	0/1	0/0	0/0	0/0	0/0	0/0	0/0	0/1	0/1	1/1	1/1	0/1	0/0
Chromosome2-3198694	BND	PRECISE	0	0	0	0/0	0/0	0/1	0/0	0/0	0/0	0/1	0/0	0/0	0/0	0/0	0/0	0/0	0/0
Chromosome2-3201224	INS	PRECISE	3928	0	0	0/0	0/0	0/1	0/0	0/0	0/0	0/0	0/0	0/0	0/0	0/0	0/0	0/0	0/0
Chromosome2-3201292	DEL	PRECISE	863	0	0	0/0	0/1	0/0	0/0	0/0	0/0	0/0	0/0	0/1	0/1	0/1	1/1	0/1	0/1
Chromosome2-3201924	DEL	PRECISE	3534	0	0	0/0	0/0	0/0	0/0	0/0	0/0	0/0	0/0	0/0	0/0	1/1	0/0	0/0	0/0
Chromosome2-3203490	DEL	PRECISE	985	0	0	0/0	0/1	0/0	0/0	0/0	0/0	0/0	0/0	0/1	0/1	0/1	1/1	0/1	0/1
Chromosome2-3204710	INS	PRECISE	90	0.577	0.577	0/0	0/1	0/0	0/0	0/0	0/0	0/0	0/0	0/0	0/0	0/0	0/0	0/0	0/1
Chromosome2-3204717	INS	PRECISE	1409	0	0	0/0	0/0	0/0	0/1	0/0	0/0	0/0	0/0	0/0	0/0	0/0	0/0	0/0	0/0
Chromosome2-3204957	DEL	PRECISE	118	0	0	0/0	0/1	0/1	0/0	1/1	0/0	0/0	0/0	0/1	0/1	0/0	1/1	1/1	1/1
Chromosome2-3205433	DEL	PRECISE	709	0	0	0/0	0/0	0/1	0/0	0/1	0/0	0/0	0/0	0/0	0/0	0/0	0/0	0/1	0/1
Chromosome2-3206300	INS	PRECISE	5992	0	0	0/0	0/0	0/0	0/0	0/0	0/0	0/0	0/0	0/0	0/0	0/0	0/0	0/0	1/1
Chromosome2-3206301	BND	PRECISE	0	0	0	0/0	0/0	0/0	0/0	0/0	0/0	0/0	0/0	0/0	0/0	0/0	0/0	0/0	0/1
Chromosome2-3206541	INS	PRECISE	165	0	0.707	0/0	0/0	0/0	0/0	0/0	0/0	0/0	0/0	0/1	0/0	0/0	0/0	0/1	0/0
Chromosome2-3206762	DEL	PRECISE	54	0	0	0/0	0/0	0/0	0/0	0/0	0/0	0/0	0/0	0/1	0/0	0/0	0/0	0/1	0/0
Chromosome2-3206944	BND	PRECISE	0	0	0	0/0	0/0	0/0	0/0	0/0	0/0	0/0	0/0	0/1	0/0	0/0	0/0	0/1	0/0
Chromosome2-3206958	INS	PRECISE	7700	0	0	0/0	0/0	0/0	0/0	1/1	0/0	0/0	0/0	0/0	0/0	0/0	0/0	0/0	0/0

Chromosome2-3206959 BND	PRECISE	0	0	0	0/0	0/0	0/0	0/0	0/1	0/0	0/0	0/0	0/0	0/0	0/0	0/0	0/0	0/0
Chromosome2-3206962 DEL	PRECISE	2430	0	0	0/0	0/0	0/0	0/0	0/0	0/0	0/0	0/0	0/0	0/1	0/0	1/1	0/0	0/0
Chromosome2-3207553 DEL	PRECISE	796	0	0	0/0	0/1	0/0	0/0	0/0	0/0	0/0	0/0	0/1	0/1	0/1	1/1	1/1	0/1
Chromosome2-3207696 INS	PRECISE	5208	0	0	0/0	0/0	0/0	0/0	0/0	0/0	0/0	0/0	0/0	0/0	1/1	0/0	0/0	0/0
Chromosome2-3207925 DEL	PRECISE	66	0	0	0/0	0/0	0/0	0/0	0/0	0/0	0/0	0/0	0/1	0/0	0/0	0/1	1/1	0/0
Chromosome2-3208308 DEL	PRECISE	114	0	0	0/0	0/1	0/0	0/0	0/0	0/0	0/0	0/0	0/0	0/0	0/1	0/0	0/0	0/1
Chromosome2-3208371 INS	PRECISE	535	0	0	0/0	0/0	0/1	0/0	0/0	0/0	0/0	0/0	0/0	0/0	0/0	0/0	0/0	0/0
Chromosome2-3208522 DEL	PRECISE	57	0	0	0/0	0/0	0/0	0/0	0/1	0/1	0/0	0/1	0/1	0/1	0/0	0/0	0/0	0/0
Chromosome2-3208640 INS	PRECISE	10564	0	0	0/0	0/0	0/0	0/0	0/0	0/0	0/0	0/0	0/0	0/0	0/0	0/0	0/0	0/1
Chromosome2-3208998 DEL	PRECISE	4234	0	0	0/0	0/1	0/0	0/0	0/0	0/0	0/0	0/0	0/1	0/1	0/1	1/1	0/1	0/1
Chromosome2-3209996 INS	PRECISE	653	0	0	0/0	0/0	0/1	0/0	0/0	0/0	0/0	0/0	0/0	0/0	0/0	0/0	0/0	0/0
Chromosome2-3210432 DEL	PRECISE	9359	0	0	0/0	1/1	0/0	0/0	0/0	0/0	0/0	0/0	0/1	1/1	1/1	0/0	0/1	0/1
Chromosome2-3210987 DEL	PRECISE	287	0	0	0/0	0/0	0/0	0/0	0/0	0/0	0/0	0/0	0/0	0/0	0/0	0/0	1/1	1/1
Chromosome2-3211230 BND	PRECISE	0	0	0	0/0	0/0	0/0	0/0	0/0	0/0	0/0	0/0	0/0	0/0	0/0	0/0	0/1	0/1
Chromosome2-3212268 DEL	PRECISE	7573	0	0	0/0	0/1	0/0	0/0	0/0	0/0	0/0	0/0	0/0	0/0	0/1	0/0	0/0	0/1
Chromosome2-3212278 INS	PRECISE	73	0	0.577	0/0	0/0	0/0	0/0	0/0	0/0	0/0	0/0	0/1	0/0	0/0	0/1	0/0	0/0
Chromosome2-3212436 DEL	PRECISE	767	0	0	0/0	0/0	0/0	0/0	0/0	0/0	0/0	0/0	0/1	0/1	0/0	1/1	0/1	0/0
Chromosome2-3212895 DEL	PRECISE	54	0	0	0/0	0/0	0/0	0/0	0/0	0/0	0/0	0/0	0/0	0/0	0/0	0/0	0/1	0/1
Chromosome2-3213381 INS	PRECISE	144	0	0.707	0/0	0/0	0/0	0/0	0/0	0/0	0/0	0/0	0/0	0/1	0/0	0/0	0/0	0/0
Chromosome2-3213868 DEL	PRECISE	204	0	0	0/0	0/1	0/0	0/0	0/0	0/0	0/0	0/0	0/1	0/1	0/1	1/1	0/1	1/1
Chromosome2-3214284 DEL	PRECISE	139	0	0	0/0	0/0	0/1	0/0	0/0	0/0	0/0	0/0	0/0	0/0	0/0	0/0	0/0	0/0
Chromosome2-3215084 DEL	PRECISE	209	0.976	0.976	0/0	0/1	0/0	0/0	0/0	0/0	0/0	0/0	0/1	0/1	0/1	1/1	0/1	0/1
Chromosome2-3215623 DEL	PRECISE	233	0	0	0/0	0/0	0/0	0/0	0/0	0/0	0/0	0/0	0/1	0/0	0/0	0/1	1/1	0/0
Chromosome2-3215741 INS	PRECISE	622	0	0.577	0/0	0/1	0/0	0/0	0/0	0/0	0/0	0/0	0/0	0/0	0/1	0/0	0/0	0/1
Chromosome2-3215782 DEL	PRECISE	860	0	0	0/0	0/0	0/0	0/0	0/0	0/0	0/0	0/0	0/1	0/0	0/0	0/1	1/1	0/0
Chromosome2-3216009 INS	PRECISE	5412	0	0	0/0	0/0	0/0	1/1	0/0	0/0	0/0	0/0	0/0	0/0	0/0	0/0	0/0	0/0
Chromosome2-3216077 DEL	PRECISE	1027	0	0	0/0	0/1	0/0	0/0	0/0	0/0	0/0	0/0	0/1	0/1	0/1	1/1	1/1	0/1
Chromosome2-3216525 BND	PRECISE	0	0.976	0	0/0	0/1	0/0	0/0	0/0	0/0	0/0	0/0	0/0	0/0	0/0	1/1	0/1	0/1
Chromosome2-3217376 DEL	PRECISE	182	0	0	0/0	0/1	0/0	0/0	0/0	0/0	0/0	0/0	0/1	0/1	0/1	1/1	0/1	0/1
Chromosome2-3217520 INS	PRECISE	116	0	0	0/0	0/1	0/0	0/0	0/0	0/0	0/0	0/0	0/1	0/1	0/1	1/1	0/1	0/1
Chromosome2-3218040 DEL	PRECISE	243	0	0	0/0	0/1	0/0	0/0	0/0	0/0	0/0	0/0	0/1	0/1	0/1	1/1	0/1	0/0
Chromosome2-3218198 INS	PRECISE	4511	0	0	0/0	0/0	0/0	0/0	1/1	0/0	0/0	0/0	0/0	0/0	0/0	0/0	0/0	0/0
Chromosome2-3218474 INS	PRECISE	1018	0	0	0/0	0/0	0/0	0/0	0/0	0/0	0/1	0/1	0/0	0/0	0/0	0/0	0/0	0/0
Chromosome2-3219520 DEL	PRECISE	457	0.577	0.577	0/0	0/0	0/0	0/0	0/0	0/0	0/0	0/0	0/0	0/0	0/0	0/1	0/0	0/0
Chromosome2-3219804 INS	PRECISE	2426	0	0	0/0	0/0	0/0	0/0	0/1	0/0	0/0	0/0	0/0	0/0	0/0	0/0	0/0	0/0
Chromosome2-3220241 INS	PRECISE	162	0	0.488	0/0	0/1	0/0	0/0	0/0	0/0	0/0	0/0	0/1	0/1	0/1	1/1	0/1	0/1
Chromosome2-3220258 DEL	PRECISE	138	0	0	0/0	0/1	0/0	0/0	0/0	0/0	0/0	0/0	0/1	0/1	0/1	1/1	0/1	0/1
Chromosome2-3220936 DEL	PRECISE	2364	0	0	0/0	0/1	0/0	0/0	0/0	0/0	0/0	0/0	0/1	0/1	0/1	1/1	1/1	1/1
Chromosome2-3221654 DEL	PRECISE	5132	0.751	0	0/0	0/1	0/1	1/1	1/1	1/1	1/1	1/1	1/1	1/1	1/1	1/1	1/1	1/1
Chromosome2-3224160 INS	PRECISE	658	0	0	0/0	0/0	0/0	0/0	0/1	0/0	0/0	0/0	0/0	0/0	0/0	0/0	0/0	0/0
Chromosome2-3224276 DEL	PRECISE	183	0	0	0/0	0/0	0/0	0/0	0/0	0/0	0/0	0/0	0/0	0/1	0/0	0/1	0/0	0/0
Chromosome2-3224609 DEL	PRECISE	2624	0	0	0/0	0/0	0/1	0/0	0/0	0/0	0/0	0/0	0/0	0/0	0/0	0/0	0/0	0/0
Chromosome2-3225499 INS	PRECISE	626	0	0	0/0	0/0	0/1	0/0	0/0	0/0	0/0	0/0	0/0	0/0	0/0	0/0	0/0	0/0
Chromosome2-3226816 DEL	PRECISE	82	0	0	0/0	0/0	0/0	1/1	0/0	0/0	0/0	0/0	0/0	0/0	0/0	0/0	0/0	0/0
Chromosome2-3228210 INS	PRECISE	823	0	0.707	0/0	0/0	0/0	0/0	0/0	0/0	0/0	0/0	0/0	0/0	0/0	0/0	0/1	1/1
Chromosome2-3228400 DEL	PRECISE	1033	0	0	0/0	0/0	0/0	0/0	0/1	0/0	0/0	0/0	0/0	0/0	0/0	0/0	0/0	0/0
Chromosome2-3228728 DEL	PRECISE	52	0	0	0/0	0/1	0/1	1/1	1/1	1/1	1/1	1/1	1/1	1/1	1/1	1/1	1/1	1/1
Chromosome2-3229113 DEL	PRECISE	1415	0	0	0/0	0/1	0/0	0/0	0/0	0/0	0/0	0/0	0/1	0/1	0/1	1/1	1/1	1/1
Chromosome2-3229180 DEL	PRECISE	702	0.447	0.447	0/0	0/0	0/0	0/0	0/1	0/1	0/0	0/1	0/1	0/1	0/0	0/0	0/0	0/0
Chromosome2-3229896 INS	PRECISE	66	0	0	0/0	0/0	0/0	0/0	0/0	0/0	0/0	0/0	0/1	0/0	0/0	0/1	0/1	0/0
Chromosome2-3230703 DEL	PRECISE	388	0	0	0/0	0/1	0/0	0/0	0/0	0/0	0/0	0/0	0/1	0/1	1/1	1/1	0/1	0/1
Chromosome2-3230724 INS	PRECISE	93	0	0.707	0/0	0/0	0/0	0/0	0/0	0/0	0/0	0/0	0/0	0/0	0/0	0/0	0/1	0/1
Chromosome2-3231426 INS	PRECISE	132	0	0	0/0	0/0	0/0	1/1	0/0	0/0	0/0	0/0	0/0	0/0	0/0	0/0	0/0	0/0
Chromosome2-3232394 DEL	PRECISE	81	0	0	0/0	0/1	0/0	0/0	0/0	0/0	0/0	0/0	0/1	0/1	0/1	1/1	0/1	0/1
Chromosome2-3234808 DEL	PRECISE	65	0	0	0/0	0/0	0/0	0/0	0/0	0/0	0/0	0/0	0/0	0/0	0/0	0/0	1/1	0/1
Chromosome2-3235337 DEL	PRECISE	58	0	0	0/0	0/1	0/0	0/0	0/0	0/0	0/1	0/0	0/0	0/0	0/1	0/0	0/0	0/1
Chromosome2-3235560 INS	PRECISE	57	0	0	0/0	0/0	0/0	0/0	0/0	0/0	0/0	0/0	0/0	0/1	0/0	0/0	0/0	0/0
Chromosome2-3235716 DEL	PRECISE	946	0	0	0/0	0/0	0/0	0/0	0/0	0/0	0/0	0/0	0/0	0/0	0/0	0/0	1/1	0/1
Chromosome2-3236823 INS	PRECISE	111	0	0	0/0	0/0	0/0	0/0	0/1	0/0	0/0	0/0	0/0	0/0	0/0	0/0	0/0	0/0
Chromosome2-3237092 INS	PRECISE	149	0	0	0/0	0/0	0/0	1/1	0/1	0/1	0/1	1/1	0/0	0/1	0/0	0/0	0/0	0/0
Chromosome2-3238040 DEL	PRECISE	88	0	0	0/0	0/0	0/0	0/0	0/0	0/0	0/0	0/0	0/0	0/0	0/0	0/0	0/1	0/1
Chromosome2-3238085 INS	PRECISE	211	0	0	0/0	0/0	0/0	1/1	0/0	0/0	0/0	0/0	0/0	0/0	0/0	0/0	0/0	0/0
Chromosome2-3238161 INS	PRECISE	2634	0	0	0/0	0/0	0/0	1/1	0/0	0/0	0/0	0/0	0/0	0/0	0/0	0/0	0/0	0/0
Chromosome2-3238162 BND	PRECISE	0	0	0	0/0	0/0	0/0	0/1	0/0	0/0	0/0	0/0	0/0	0/0	0/0	0/0	0/0	0/0
Chromosome2-3238190 INS	PRECISE	211	0	0	0/0	0/0	0/0	1/1	0/0	0/0	0/0	0/0	0/0	0/0	0/0	0/0	0/0	0/0
Chromosome2-3238573 INS	PRECISE	121	0	0.707	0/0	0/0	0/0	0/0	0/0	0/0	0/0	0/0	0/0	0/0	0/0	0/0	0/1	0/1
Chromosome2-3239613 BND	PRECISE	0	0	0	0/0	0/0	0/0	0/0	0/0	0/0	0/0	0/0	0/1	0/0	0/0	0/0	0/0	0/0
Chromosome2-3239703 INS	PRECISE	9975	0	0	0/0	0/0	0/0	0/0	0/0	0/0	0/1	0/0	0/0	0/0	0/0	0/0	0/0	0/0
Chromosome2-3240101 DEL	PRECISE	420	0	0	0/0	0/1	0/0	0/0	0/0	0/0	0/1	0/0	0/1	0/1	0/1	1/1	0/1	0/1
Chromosome2-3240308 DEL	PRECISE	222	0	0	0/0	0/0	0/0	0/0	0/0	0/0	0/0	0/0	0/0	0/0	0/0	0/0	0/0	0/1
Chromosome2-3240852 INS	PRECISE	1422	0	0	0/0	0/0	0/1	0/0	0/0	0/0	0/0	0/0	0/0	0/0	0/0	0/0	0/0	0/0
Chromosome2-3241009 DEL	PRECISE	426	0	0	0/0	0/0	0/0	0/0	1/1	0/0	0/0	0/0	0/0	1/1	0/0	0/0	1/1	1/1
Chromosome2-3241118 DEL	PRECISE	561	0	0	0/0	0/0	0/0	0/0	0/1	0/0	0/0	0/0	0/0	0/1	0/0	0/0	1/1	1/1

Chromosome2-3241220 DEL	PRECISE	378	0	0	0/0	0/0	0/0	0/0	0/1	0/0	0/0	0/0	0/0	0/1	0/0	1/1	1/1	1/1
Chromosome2-3241446 INS	PRECISE	54	0	0.518	0/0	0/0	0/0	0/0	0/0	0/0	0/0	0/0	0/0	0/1	0/0	1/1	1/1	0/1
Chromosome2-3241739 DEL	PRECISE	63	0	0	0/0	0/1	0/0	0/0	0/0	0/0	0/1	0/0	0/1	0/1	0/0	1/1	1/1	1/1
Chromosome2-3241981 DEL	PRECISE	432	0	0	0/0	0/0	0/0	0/0	0/0	1/1	0/1	0/1	0/1	0/0	0/1	0/0	0/0	0/0
Chromosome2-3242052 DEL	PRECISE	77	0	0	0/0	0/0	0/1	0/0	0/0	0/0	0/0	0/0	0/0	0/0	0/0	0/0	0/0	0/0
Chromosome2-3242452 DEL	PRECISE	5272	0	0	0/0	0/1	0/0	0/0	0/0	0/0	0/1	0/0	0/1	0/1	0/0	1/1	1/1	1/1
Chromosome2-3243273 DEL	PRECISE	179	0	0	0/0	0/0	0/1	0/0	1/1	1/1	0/1	1/1	0/1	0/1	0/1	0/0	0/0	0/1
Chromosome2-3243856 BND	PRECISE	0	0	0	0/0	0/1	0/0	0/0	0/0	0/0	0/1	0/0	0/0	0/0	0/1	0/1	0/0	0/1
Chromosome2-3243948 BND	PRECISE	0	0	0	0/0	0/1	0/0	0/0	0/0	0/0	0/0	0/0	0/1	0/0	0/1	0/0	0/1	0/1
Chromosome2-3243998 DEL	PRECISE	1408	0	0	0/0	0/0	0/0	0/0	0/1	0/0	0/0	0/0	0/0	0/0	0/0	0/0	0/0	0/0
Chromosome2-3244055 DEL	PRECISE	10986	0	0	0/0	0/0	0/0	0/0	0/0	0/0	0/0	0/0	0/0	0/0	0/0	0/0	0/1	0/1
Chromosome2-3244199 DEL	PRECISE	1354	0	0	0/0	0/0	0/0	0/0	0/0	0/0	0/0	0/0	0/0	0/1	0/0	1/1	0/0	0/0
Chromosome2-3245145 DEL	PRECISE	71	0	0	0/0	0/0	0/1	0/0	1/1	0/0	0/0	0/1	0/0	0/1	0/1	0/0	0/0	0/0
Chromosome2-3245157 INS	PRECISE	2074	0	0	0/0	0/0	0/0	0/0	0/0	0/0	0/0	0/0	0/0	0/1	0/0	0/0	0/0	0/0
Chromosome2-3245198 INS	PRECISE	48	0	0.756	0/0	0/1	0/0	0/0	0/0	0/0	0/1	0/0	0/1	0/0	0/1	0/1	0/1	0/1
Chromosome2-3245393 DEL	PRECISE	264	0	0	0/0	0/0	0/1	0/0	1/1	0/0	0/0	0/0	0/0	0/0	0/0	0/0	0/0	0/0
Chromosome2-3245585 DEL	PRECISE	51	0	0	0/0	0/1	0/0	0/0	0/0	0/0	0/1	0/0	0/1	0/0	1/1	0/1	0/1	0/1
Chromosome2-3245740 INS	PRECISE	102	0	0	0/0	0/0	0/0	0/0	0/0	0/0	0/0	0/0	0/0	1/1	0/0	1/1	0/0	0/0
Chromosome2-3245780 INS	PRECISE	5323	0	0	0/0	0/0	1/1	0/0	0/0	0/0	0/0	0/0	0/0	0/0	0/0	0/0	0/0	0/0
Chromosome2-3246064 DEL	PRECISE	184	0	0	0/0	0/0	0/0	0/0	0/0	0/0	0/0	0/0	0/0	1/1	0/0	1/1	0/0	0/0
Chromosome2-3246154 DUP	PRECISE	206	0	0	0/0	0/0	0/0	0/0	0/1	0/1	0/0	0/1	0/0	0/1	0/1	0/0	0/0	0/0
Chromosome2-3246181 INS	PRECISE	113	0	0.488	0/0	0/0	0/1	0/0	1/1	0/1	0/0	0/1	0/0	1/1	1/1	1/1	0/0	0/0
Chromosome2-3246314 DEL	PRECISE	3185	0	0	0/0	0/0	0/1	0/0	1/1	0/1	0/0	0/1	0/0	1/1	1/1	1/1	0/0	0/0
Chromosome2-3246588 DEL	PRECISE	73	0.707	0.707	0/0	0/0	0/0	0/0	0/0	0/1	0/0	0/0	1/1	0/0	0/0	0/0	0/0	0/0
Chromosome2-3247120 DEL	PRECISE	85	0	0	0/0	0/0	0/0	0/0	0/0	0/1	0/0	0/0	0/1	0/0	0/0	0/0	0/1	0/1
Chromosome2-3247263 DEL	PRECISE	425	0	0	0/0	0/0	0/1	0/0	1/1	0/1	0/0	0/1	0/0	0/1	0/1	0/0	0/0	0/0
Chromosome2-3247529 INS	PRECISE	5586	0	0	0/0	0/0	1/1	0/0	0/0	0/0	0/0	0/0	0/0	0/0	0/0	0/0	0/0	0/0
Chromosome2-3247589 INS	PRECISE	715	0	0	0/0	0/0	0/1	0/0	0/0	0/0	0/0	0/0	0/0	0/0	0/0	0/0	0/0	0/0
Chromosome2-3248148 BND	PRECISE	0	0	0	0/0	0/0	0/0	0/0	0/0	0/0	0/0	0/0	0/0	0/0	0/0	0/1	0/0	0/0
Chromosome2-3248164 DEL	PRECISE	57	0	0	0/0	0/1	0/0	0/0	0/0	0/0	0/1	0/0	0/1	0/0	1/1	1/1	0/1	0/1
Chromosome2-3248610 INS	PRECISE	378	0	0.577	0/0	0/0	0/1	0/0	1/1	0/1	0/0	0/0	0/0	1/1	0/1	0/1	0/0	0/0
Chromosome2-3248746 INS	PRECISE	11083	0	0	0/0	0/1	0/0	0/0	0/0	0/0	0/0	0/0	0/0	0/0	0/0	0/0	0/0	0/0
Chromosome2-3248747 BND	PRECISE	0	0	0	0/0	0/1	0/0	0/0	0/0	0/0	0/1	0/0	0/0	0/0	0/0	0/0	0/0	0/1
Chromosome2-3249025 DEL	PRECISE	85	0	0	0/0	0/0	0/0	0/0	0/1	0/0	0/0	0/0	0/0	0/0	0/0	0/0	0/0	0/0
Chromosome2-3249300 INS	PRECISE	5312	0	0	0/0	0/0	0/0	0/0	1/1	0/0	0/0	0/0	0/0	0/0	0/0	0/0	0/0	0/0
Chromosome2-3249434 DEL	PRECISE	66	0	0	0/0	0/0	0/1	0/0	1/1	0/1	0/0	0/0	0/0	0/1	1/1	0/0	0/0	0/0
Chromosome2-3249561 DEL	PRECISE	330	0	0	0/0	0/0	0/1	0/0	1/1	0/1	0/0	0/0	0/0	0/1	1/1	0/0	0/0	0/0
Chromosome2-3250998 INS	PRECISE	338	0	0.9	0/0	0/1	0/0	0/0	0/0	0/0	0/1	0/0	0/1	0/0	0/1	0/1	0/1	0/1
Chromosome2-3251129 DEL	PRECISE	3317	0	0	0/0	0/0	0/0	0/0	0/0	0/0	0/0	0/0	0/0	1/1	0/0	0/1	0/0	0/0
Chromosome2-3251202 DEL	PRECISE	57	0	0	0/0	0/0	0/0	0/0	0/0	0/0	0/0	0/0	0/1	0/0	0/0	0/1	0/1	0/0
Chromosome2-3251284 DEL	PRECISE	1043	0	0	0/0	0/0	1/1	0/0	1/1	0/1	0/0	0/1	0/0	0/1	0/1	0/0	0/0	0/0
Chromosome2-3251579 INS	PRECISE	276	0	0.535	0/0	0/1	0/0	0/0	0/0	0/0	0/1	0/0	0/1	0/0	1/1	0/1	1/1	0/1
Chromosome2-3251782 DEL	PRECISE	98	0.463	0	0/0	0/1	0/0	0/0	0/0	0/0	0/1	0/0	0/1	1/1	1/1	1/1	1/1	1/1
Chromosome2-3252474 DEL	PRECISE	120	0	0	0/0	0/1	0/0	0/0	1/1	0/0	0/1	0/0	1/1	1/1	1/1	1/1	1/1	1/1
Chromosome2-3253357 INS	PRECISE	442	0	0.5	0/0	0/0	0/1	0/0	1/1	1/1	0/0	0/0	0/1	0/0	0/0	0/0	0/0	0/0
Chromosome2-3253577 DEL	PRECISE	222	0	0	0/0	0/0	0/1	0/0	1/1	0/1	0/0	0/0	0/0	0/0	0/1	0/0	0/0	0/0
Chromosome2-3253984 DEL	PRECISE	475	0	0	0/0	0/1	0/1	0/0	1/1	1/1	0/1	0/0	1/1	1/1	1/1	1/1	1/1	1/1
Chromosome2-3254115 DEL	PRECISE	1205	0	0	0/0	0/0	0/0	0/0	0/0	0/0	0/0	0/0	0/1	1/1	0/0	0/1	0/1	0/0
Chromosome2-3254307 DEL	PRECISE	876	0	0	0/0	0/0	0/0	0/0	0/1	0/0	0/0	0/0	0/0	0/0	0/0	0/0	0/0	0/0
Chromosome2-3254314 BND	PRECISE	0	0	0	0/0	0/0	0/0	0/0	0/0	0/0	0/0	0/1	0/0	0/0	0/0	0/0	0/0	0/0
Chromosome2-3254678 INS	PRECISE	164	0	0	0/0	0/0	0/0	0/0	0/0	0/0	0/0	0/0	0/0	0/1	0/0	0/1	0/0	0/0
Chromosome2-3255165 DEL	PRECISE	355	0	0	0/0	0/0	0/0	0/0	0/0	0/0	0/0	0/0	0/1	0/0	0/0	0/1	0/1	0/0
Chromosome2-3255730 DEL	PRECISE	1422	0	0	0/0	0/0	0/0	0/0	1/1	1/1	0/0	0/1	1/1	0/1	1/1	0/0	1/1	1/1
Chromosome2-3255998 BND	PRECISE	0	0	0	0/0	0/0	0/0	0/0	0/0	0/0	0/0	0/0	0/1	0/0	0/0	0/0	0/1	0/0
Chromosome2-3256266 INS	PRECISE	294	0	0	0/0	0/0	0/1	0/0	0/0	0/0	0/0	0/0	0/0	0/0	0/0	0/0	0/0	0/0
Chromosome2-3256829 INS	PRECISE	5406	0	0	0/0	0/0	0/0	0/0	1/1	0/0	0/0	0/0	0/0	0/0	0/0	0/0	0/0	0/0
Chromosome2-3257124 DEL	PRECISE	4534	0	0	0/0	0/0	0/0	0/0	0/0	0/0	0/0	0/0	1/1	0/0	0/0	1/1	0/1	0/0
Chromosome2-3257718 DEL	PRECISE	713	0	0	0/0	0/0	0/0	0/0	0/0	0/0	0/0	0/0	1/1	0/0	0/0	1/1	0/1	0/0
Chromosome2-3258011 DEL	PRECISE	5243	0	0	0/0	0/0	0/0	0/0	0/1	0/0	0/0	0/0	0/1	0/0	0/0	1/1	1/1	1/1
Chromosome2-3258880 INS	PRECISE	136	0	0	0/0	0/0	0/0	0/0	0/1	0/0	0/0	0/0	0/1	0/0	0/0	1/1	0/1	0/0
Chromosome2-3259019 DEL	PRECISE	73	0.957	0.957	0/0	0/1	0/0	0/0	0/0	0/0	0/0	0/0	0/0	0/0	0/0	0/0	0/0	0/0
Chromosome2-3259573 INS	PRECISE	3585	0	0.577	0/0	0/0	0/0	0/0	0/0	0/0	0/0	0/0	0/1	0/0	0/0	0/1	0/1	0/0
Chromosome2-3259817 BND	PRECISE	0	0	0	0/0	0/0	0/0	0/0	0/0	0/0	0/1	0/0	0/0	0/0	0/0	0/0	0/0	0/0
Chromosome2-3259879 INS	PRECISE	710	0	0	0/0	0/0	0/0	0/0	0/0	0/0	0/0	0/0	0/0	0/1	0/0	0/1	0/0	0/0
Chromosome2-3259906 DEL	PRECISE	61	0	0	0/0	0/0	0/0	0/0	0/0	0/0	0/0	0/0	0/1	0/0	0/0	0/1	0/1	0/0
Chromosome2-3260104 INS	PRECISE	202	0	0	0/0	0/0	0/0	0/0	0/1	0/0	0/0	0/0	0/0	0/0	0/0	0/0	0/0	0/0
Chromosome2-3260416 BND	PRECISE	0	0	0	0/0	0/0	0/1	0/0	0/0	0/0	0/0	0/0	0/0	0/0	0/0	0/0	0/0	0/0
Chromosome2-3260490 INS	PRECISE	5395	0	0	0/0	0/0	0/0	1/1	0/0	0/0	0/0	0/0	0/0	0/0	0/0	0/0	0/0	0/0
Chromosome2-3260595 INS	PRECISE	1643	0	0.535	0/0	0/1	0/0	0/0	0/0	0/0	0/1	0/0	0/1	0/0	0/1	0/1	0/1	0/1
Chromosome2-3260599 INS	PRECISE	74	0	0	0/0	0/0	0/0	0/0	0/0	0/0	0/0	0/0	0/0	0/1	0/0	0/1	0/0	0/0
Chromosome2-3260739 INS	PRECISE	279	0	0	0/0	0/0	0/0	0/0	0/0	0/0	0/0	0/0	0/0	0/1	0/0	0/1	0/0	0/0
Chromosome2-3261040 INS	PRECISE	1165	0	0	0/0	0/0	0/0	0/0	0/0	0/0	0/0	0/0	0/0	0/1	0/0	0/1	0/0	0/0
Chromosome2-3261086 DEL	PRECISE	58	0	0	0/0	0/0	0/0	0/0	0/0	0/0	0/0	0/0	0/0	0/1	0/0	0/1	0/0	0/0

Chromosome2-3262596	DEL	PRECISE	62	0	0	0/0	0/1	0/0	0/0	0/0	0/0	0/1	0/0	0/1	0/0	0/1	0/1	1/1	0/1
Chromosome2-3263238	INS	PRECISE	229	0	0.5	0/0	0/1	0/0	0/0	0/0	0/0	0/1	0/0	0/0	0/0	0/1	0/0	0/0	0/1
Chromosome2-3263301	INS	PRECISE	603	0	0.707	0/0	0/0	0/0	1/1	0/0	0/0	0/0	0/0	0/0	0/0	0/1	0/0	0/0	0/0
Chromosome2-3264067	INS	PRECISE	902	0	0	0/0	0/0	0/1	0/0	0/0	0/0	0/0	0/0	0/0	0/0	0/0	0/0	0/0	0/0
Chromosome2-3264185	DEL	PRECISE	1662	0	0	0/0	0/0	0/0	1/1	0/0	0/0	1/1	0/1	0/0	0/0	0/0	0/0	0/0	0/0
Chromosome2-3264643	DEL	PRECISE	180	0	0	0/0	0/0	0/0	0/0	0/1	0/0	0/0	0/0	0/0	0/0	0/0	0/0	0/0	0/0
Chromosome2-3264936	DEL	PRECISE	90	0	0	0/0	0/0	0/0	0/0	0/1	0/0	0/0	0/0	0/0	0/0	0/0	0/0	1/1	1/1
Chromosome2-3264972	BND	PRECISE	0	0	0	0/0	0/0	0/0	0/0	0/1	0/0	0/0	0/0	0/0	0/0	0/0	0/0	0/0	0/0
Chromosome2-3264972	BND	PRECISE	0	0	0	0/0	0/0	0/1	0/0	0/1	0/0	0/0	0/0	0/0	0/0	0/0	0/0	0/0	0/0
Chromosome2-3265073	BND	PRECISE	0	0	0	0/0	0/1	0/0	0/0	0/0	0/0	0/0	0/0	1/1	0/0	0/0	0/0	0/0	0/0
Chromosome2-3265154	INS	PRECISE	233	0	0	0/0	0/0	0/0	1/1	0/0	0/0	0/0	0/0	0/1	0/0	0/0	0/0	0/0	0/0
Chromosome2-3265406	INS	PRECISE	62	0	0	0/0	0/0	0/0	0/0	0/0	0/0	1/1	0/1	0/0	0/0	0/0	0/0	0/0	0/0
Chromosome2-3265680	DEL	PRECISE	50	0	0	0/0	0/0	0/0	0/0	0/0	0/1	0/0	0/0	1/1	0/0	0/0	0/0	0/0	0/0
Chromosome2-3274616	DEL	PRECISE	5102	0	0	0/0	0/0	0/0	0/0	0/0	0/0	1/1	0/1	0/0	0/0	0/0	0/0	0/0	0/0
Chromosome2-3274935	INS	PRECISE	7455	0	0	0/0	0/0	0/0	0/0	0/0	0/0	0/0	0/0	0/0	0/0	1/1	0/0	0/0	0/0
Chromosome2-3276332	DEL	PRECISE	58	0	0	0/0	0/0	0/0	0/0	0/0	0/0	1/1	0/1	0/0	0/0	0/0	0/0	0/0	0/0
Chromosome2-3276579	DEL	PRECISE	1255	0	0	0/0	0/0	0/0	0/0	0/0	0/0	1/1	0/1	0/0	0/0	0/0	0/0	0/0	0/0
Chromosome2-3278021	DEL	PRECISE	1035	0	0	0/0	0/0	0/1	0/0	0/1	0/0	0/0	0/0	0/0	0/0	0/0	0/0	0/0	0/0
Chromosome2-3278125	INS	PRECISE	4432	0	0	0/0	0/0	0/0	1/1	0/0	0/0	0/0	0/0	0/0	0/0	0/0	0/0	0/0	0/0
Chromosome2-3278125	BND	PRECISE	0	0	0	0/0	0/0	0/0	0/1	0/0	0/0	0/0	0/0	0/0	0/0	0/0	0/0	0/0	0/0
Chromosome2-3278163	INS	PRECISE	132	0	0.408	0/0	0/0	0/1	1/1	0/1	0/0	1/1	0/0	0/0	0/0	0/0	0/0	0/0	1/1
Chromosome2-3278372	INS	PRECISE	3973	0	0	0/0	0/0	0/0	1/1	0/0	0/0	0/0	0/0	0/0	0/0	0/0	0/0	0/0	0/0
Chromosome2-3278502	INS	PRECISE	623	0	0.707	0/0	0/0	0/1	0/0	0/1	0/0	0/0	0/0	0/0	0/0	0/0	0/0	0/0	0/0
Chromosome2-3278972	INS	PRECISE	718	0	0	0/0	0/0	0/0	0/0	0/0	0/0	0/1	0/0	0/0	0/0	0/0	0/0	0/0	0/0
Chromosome2-3279000	DEL	PRECISE	833	0	0	0/0	0/0	0/0	1/1	0/0	0/0	0/0	0/0	0/0	0/0	0/0	0/0	0/0	0/0
Chromosome2-3279199	INS	PRECISE	212	0	0	0/0	0/0	0/0	0/0	0/0	0/1	0/0	0/0	1/1	0/0	0/0	0/0	0/0	0/0
Chromosome2-3279431	INS	PRECISE	8207	0	0	0/0	0/0	0/0	1/1	0/0	0/0	0/0	0/0	0/0	0/0	0/0	0/0	0/0	0/0
Chromosome2-3280336	INS	PRECISE	51	0	0.577	0/0	0/0	0/0	1/1	0/0	0/0	0/0	0/0	0/0	0/0	0/0	0/0	1/1	1/1
Chromosome2-3280456	INS	PRECISE	52	0	0	0/0	0/0	0/1	1/1	0/1	0/1	0/1	0/0	1/1	0/0	0/0	0/0	1/1	1/1
Chromosome2-3280888	INS	PRECISE	489	0	0.707	0/0	0/0	0/0	0/0	0/0	0/0	0/0	0/0	0/0	0/0	0/0	0/0	1/1	1/1
Chromosome2-3281153	DEL	PRECISE	72	0	0	0/0	0/0	0/1	0/0	0/1	0/1	0/0	0/0	1/1	0/0	1/1	0/0	0/0	0/0
Chromosome2-3281277	DEL	PRECISE	169	0	0	0/0	0/0	0/0	0/0	0/0	0/0	1/1	0/1	0/0	0/0	0/0	0/0	0/0	0/0
Chromosome2-3281598	DEL	PRECISE	95	0	0	0/0	0/0	0/0	0/0	0/0	0/0	0/0	0/0	1/1	0/1	0/1	1/1	0/1	0/1
Chromosome2-3281621	INS	PRECISE	13032	0	0	0/0	0/0	0/0	0/0	0/0	0/0	0/0	0/0	1/1	0/0	0/0	0/0	0/0	0/0
Chromosome2-3281621	BND	PRECISE	0	0	0	0/0	0/0	0/0	0/0	0/0	0/1	0/0	0/0	0/1	0/0	0/0	0/0	0/0	0/0
Chromosome2-3281920	DEL	PRECISE	50	0	0	0/0	0/0	0/1	0/0	0/1	0/1	0/0	0/0	0/1	0/0	1/1	0/0	0/0	0/0
Chromosome2-3282088	DEL	PRECISE	72	0	0	0/0	0/0	0/0	1/1	0/0	0/0	0/0	0/0	0/0	0/0	0/0	0/0	0/1	0/1
Chromosome2-3282659	BND	PRECISE	0	0	0	0/0	0/0	0/0	0/0	0/0	0/0	0/0	0/0	0/0	0/0	0/0	0/0	0/0	0/1
Chromosome2-3282901	INS	PRECISE	1983	0	0	0/0	0/0	0/1	0/0	0/0	0/0	0/0	0/0	0/0	0/0	0/0	0/0	0/0	0/0
Chromosome2-3283077	INS	PRECISE	112	0	0	0/0	0/1	0/0	0/0	0/0	0/0	0/1	0/0	0/1	0/1	0/1	1/1	1/1	0/1
Chromosome2-3283301	INS	PRECISE	55	0	0	0/0	0/0	0/1	1/1	0/1	0/1	1/1	0/1	1/1	0/0	1/1	0/0	1/1	0/0
Chromosome2-3283416	INS	PRECISE	290	0	0.527	0/0	0/0	0/1	1/1	0/1	0/1	1/1	0/1	1/1	0/0	1/1	0/0	1/1	0/0
Chromosome2-3283847	DEL	PRECISE	4470	0	0	0/0	0/0	0/0	0/0	0/0	0/0	0/0	0/0	0/0	0/0	0/0	0/0	0/1	0/0
Chromosome2-3283872	DEL	PRECISE	1986	0.751	0.751	0/0	0/1	0/1	1/1	0/1	1/1	1/1	0/1	1/1	0/1	1/1	1/1	0/1	1/1
Chromosome2-3284429	DEL	PRECISE	55	0	0	0/0	0/0	0/0	1/1	0/0	0/0	0/0	0/0	0/0	0/0	0/0	0/0	0/1	0/0
Chromosome2-3284498	INS	PRECISE	1293	0	0	0/0	0/0	0/0	0/0	0/1	0/0	0/0	0/0	0/0	0/0	0/0	0/0	0/0	0/0
Chromosome2-3284799	INS	PRECISE	404	0	0	0/0	0/0	0/0	0/0	0/0	0/0	0/0	0/0	0/0	0/0	0/0	0/0	0/1	0/0
Chromosome2-3285953	INS	PRECISE	1453	0	0	0/0	0/0	0/0	0/0	0/0	0/0	0/0	0/0	0/0	0/0	1/1	0/0	0/0	0/0
Chromosome2-3287537	INS	PRECISE	657	0	0	0/0	0/0	0/0	0/0	0/0	0/0	0/0	0/0	0/0	0/0	1/1	0/0	0/0	0/0
Chromosome2-3287740	INS	PRECISE	97	0	0	0/0	0/1	0/0	0/0	0/0	0/0	0/1	0/0	0/0	0/0	0/1	0/0	0/0	0/1
Chromosome2-3288898	DEL	PRECISE	4102	0	0	0/0	0/0	0/0	0/0	0/0	0/0	0/0	0/0	0/0	1/1	0/0	0/1	0/0	0/0
Chromosome2-3288997	DEL	PRECISE	3112	0	0	0/0	1/1	0/0	0/0	0/0	0/0	0/1	0/0	0/0	0/0	0/1	0/0	0/0	1/1
Chromosome2-3289122	DEL	PRECISE	1858	0	0	0/0	0/1	0/1	0/0	1/1	1/1	1/1	1/1	1/1	1/1	1/1	1/1	1/1	1/1
Chromosome2-3289433	INS	PRECISE	206	0	0.577	0/0	0/0	0/0	0/0	0/0	0/0	0/0	0/0	0/1	0/0	0/0	0/1	0/1	0/0
Chromosome2-3289947	DEL	PRECISE	75	0	0	0/0	0/0	0/0	0/0	0/0	0/0	0/0	0/0	0/1	0/0	0/0	0/1	0/1	0/0
Chromosome2-3290385	INS	PRECISE	10815	0	0	0/0	0/0	0/0	0/0	0/1	0/0	0/0	0/0	0/0	0/0	0/0	0/0	0/0	0/0
Chromosome2-3290386	BND	PRECISE	0	0	0	0/0	0/0	0/0	0/0	0/1	0/0	0/0	0/0	0/0	0/0	0/0	0/0	0/0	0/0
Chromosome2-3291226	INS	PRECISE	396	0	0	0/0	0/0	0/0	0/0	0/1	0/0	0/0	0/0	0/0	0/0	0/0	0/0	0/0	0/0
Chromosome2-3291891	INS	PRECISE	93	0	0.333	0/0	0/1	0/0	0/0	0/0	0/1	0/1	0/0	1/1	0/1	1/1	1/1	0/1	0/1
Chromosome2-3292342	INS	PRECISE	147	0	0	0/0	0/0	0/0	0/0	0/1	0/0	0/0	0/0	0/0	0/0	0/0	0/0	0/1	0/1
Chromosome2-3292688	INS	PRECISE	334	0	0.577	0/0	0/0	0/0	0/0	0/0	0/1	0/0	0/0	0/1	0/0	0/1	0/0	0/0	0/0
Chromosome2-3292812	INS	PRECISE	66	0	0	0/0	0/0	0/0	0/0	0/0	0/0	0/0	0/0	0/0	0/0	0/0	0/0	0/1	0/1
Chromosome2-3292825	DEL	PRECISE	57	0	0	0/0	0/1	0/0	0/0	0/0	0/0	0/1	0/0	0/1	0/1	0/1	1/1	0/1	0/1
Chromosome2-3293093	DEL	PRECISE	83	0	0	0/0	0/1	0/0	0/0	0/0	0/0	0/1	0/0	0/1	0/0	0/1	0/1	1/1	0/1
Chromosome2-3293160	INS	PRECISE	168	0	0.707	0/0	0/0	0/0	0/0	0/0	0/0	0/0	0/0	0/0	0/1	0/0	0/0	0/0	0/0
Chromosome2-3293190	DEL	PRECISE	172	0	0	0/0	0/1	0/0	0/0	0/0	0/0	0/0	0/0	0/1	0/0	0/1	1/1	0/1	0/1
Chromosome2-3293431	DEL	PRECISE	90	0	0	0/0	0/0	0/0	0/0	0/0	0/0	0/0	0/0	0/0	0/1	0/0	0/0	0/0	0/0
Chromosome2-3293503	INS	PRECISE	11224	0	0	0/0	0/0	0/0	0/0	0/1	0/0	0/0	0/0	0/0	0/0	0/0	0/0	0/0	0/0
Chromosome2-3293661	INS	PRECISE	5610	0	0	0/0	0/0	0/0	1/1	0/0	0/0	0/0	0/0	0/0	0/0	0/0	0/0	0/0	0/0
Chromosome2-3293995	INS	PRECISE	6317	0	0	0/0	0/0	0/0	0/0	1/1	0/0	0/0	0/0	0/0	0/0	0/0	0/0	0/0	0/0
Chromosome2-3293996	BND	PRECISE	0	0	0	0/0	0/0	0/0	0/0	0/1	0/0	0/0	0/0	0/0	0/0	0/0	0/0	0/0	0/0
Chromosome2-3295381	INS	PRECISE	55	0	0	0/0	0/0	0/0	0/0	0/0	0/0	0/0	0/0	0/0	0/0	0/1	0/0	0/0	0/0
Chromosome2-3295391	DEL	PRECISE	2077	0	0	0/0	0/1	0/0	0/0	0/0	0/0	1/1	0/0	0/1	0/0	0/1	1/1	0/1	1/1

Chromosome2-3296506	DEL	PRECISE	70	0	0	0/0	0/0	0/0	1/1	0/0	0/0	0/1	0/1	0/0	0/0	0/0	0/0	0/0	0/0
Chromosome2-3297401	DEL	PRECISE	336	0	0	0/0	0/0	0/0	0/0	0/1	0/0	0/0	0/0	0/0	0/0	0/0	0/0	0/0	0/0
Chromosome2-3297771	INS	PRECISE	63	0	0	0/0	0/1	0/0	1/1	0/0	0/0	0/1	0/0	0/1	0/1	1/1	1/1	0/1	0/1
Chromosome2-3297804	DEL	PRECISE	76	0	0	0/0	0/1	0/0	0/0	0/0	0/0	0/1	0/0	0/1	0/1	0/1	1/1	0/1	0/1
Chromosome2-3298039	INS	PRECISE	10866	0	0	0/0	0/0	0/0	0/0	0/1	0/0	0/0	0/0	0/0	0/0	0/0	0/0	0/0	0/0
Chromosome2-3298249	INS	PRECISE	1614	0	0.707	0/0	0/0	0/0	0/0	0/0	0/0	0/1	0/1	0/0	0/0	0/0	0/0	0/0	0/0
Chromosome2-3299431	INS	PRECISE	63	0	0	0/0	0/0	0/1	1/1	0/0	0/0	1/1	0/1	0/1	0/0	1/1	1/1	1/1	0/0
Chromosome2-3301686	DEL	PRECISE	1016	0	0	0/0	0/0	0/0	0/0	1/1	0/1	0/0	1/1	0/0	1/1	0/0	0/0	0/0	0/0
Chromosome2-3302225	DEL	PRECISE	54	0	0	0/0	0/0	0/0	0/0	0/0	0/0	0/0	0/0	0/0	0/0	0/0	1/1	0/0	0/0
Chromosome2-3303546	DEL	PRECISE	750	0	0	0/0	0/0	0/1	1/1	0/1	0/0	1/1	0/0	0/0	0/0	1/1	1/1	0/0	0/0
Chromosome2-3303656	DEL	PRECISE	2187	0	0	0/0	0/0	0/1	1/1	0/1	0/0	1/1	0/0	0/0	0/0	1/1	1/1	0/0	0/0
Chromosome2-3304387	DEL	PRECISE	473	0	0	0/0	0/0	0/0	1/1	0/0	0/0	1/1	0/0	0/0	0/0	1/1	0/0	0/0	0/0
Chromosome2-3307062	DEL	PRECISE	126	0	0	0/0	0/1	0/0	0/0	0/0	0/0	0/1	0/0	0/0	0/0	0/1	0/0	0/1	0/1
Chromosome2-3310716	BND	PRECISE	0	0	0	0/0	0/0	0/0	0/0	0/0	0/0	0/0	0/0	0/0	0/0	0/0	0/1	0/0	0/0
Chromosome2-3310744	INS	PRECISE	159	0	0.707	0/0	0/0	0/0	0/0	0/0	0/0	0/1	0/1	0/0	0/0	0/0	0/0	0/0	0/0
Chromosome2-3310857	INS	PRECISE	1428	0	0	0/0	0/0	0/0	0/0	0/0	0/0	0/0	0/0	0/0	0/0	0/0	0/0	1/1	1/1
Chromosome2-3310897	DEL	PRECISE	1154	0	0	0/0	0/0	0/1	0/0	0/1	0/0	0/0	0/0	0/0	0/0	0/0	0/0	1/1	1/1
Chromosome2-3311679	BND	PRECISE	0	0	0	0/0	0/1	0/0	0/0	0/0	0/0	0/0	0/0	0/0	0/0	0/1	0/0	0/0	0/0
Chromosome2-3313395	DEL	PRECISE	64	0.707	0	0/0	0/1	0/0	0/0	0/0	0/0	0/1	0/0	0/1	0/1	0/1	1/1	0/1	0/1
Chromosome2-3313858	INS	PRECISE	357	0	0	0/0	0/0	0/0	0/0	0/1	0/0	0/0	0/0	0/0	0/0	0/0	0/0	0/0	0/0
Chromosome2-3314008	INS	PRECISE	50	0	0	0/0	0/1	0/0	0/0	0/0	0/0	0/0	0/0	0/0	0/1	0/1	1/1	0/0	0/0
Chromosome2-3314326	DEL	PRECISE	434	0	0	0/0	0/0	0/0	0/0	0/0	0/0	0/0	0/0	0/0	0/0	0/0	0/0	0/1	0/1
Chromosome2-3315203	INS	PRECISE	1027	0	0.577	0/0	0/0	0/0	0/0	0/0	0/0	0/0	0/0	0/1	0/0	0/0	0/0	0/1	0/0
Chromosome2-3315674	DEL	PRECISE	2981	0	0	0/0	0/0	0/1	0/0	0/0	0/0	1/1	0/1	0/0	0/0	1/1	0/0	0/1	1/1
Chromosome2-3316331	BND	PRECISE	0	0	0	0/0	0/0	0/0	0/0	0/0	0/0	0/0	0/0	0/0	0/0	0/0	0/0	0/1	0/0
Chromosome2-3317773	BND	PRECISE	0	0	0	0/0	0/0	0/1	0/0	0/1	0/0	0/0	0/0	0/0	1/1	0/0	0/0	0/0	0/0
Chromosome2-3318343	INS	PRECISE	6243	0	0	0/0	0/0	0/0	0/0	0/0	0/0	0/0	0/0	0/0	0/0	0/0	0/0	1/1	0/0
Chromosome2-3318484	INS	PRECISE	52	0	0.378	0/0	0/0	0/0	0/0	0/1	0/1	1/1	1/1	0/0	1/1	1/1	0/0	1/1	0/0
Chromosome2-3319394	DEL	PRECISE	4993	0	0	0/0	0/0	0/0	1/1	1/1	0/0	0/0	0/0	0/0	0/0	0/0	0/0	0/0	0/0
Chromosome2-3320422	DEL	PRECISE	223	0	0	0/0	0/0	0/0	1/1	1/1	0/0	0/0	0/0	0/0	0/0	0/0	0/0	0/0	0/0
Chromosome2-3322592	INS	PRECISE	113	0	0	0/0	0/0	0/0	1/1	1/1	0/0	0/0	0/0	0/0	0/0	0/0	0/0	0/0	0/0
Chromosome2-3322798	INS	PRECISE	1485	0	0.577	0/0	0/0	0/0	0/0	0/0	0/0	0/0	0/0	0/1	0/0	0/0	1/1	0/1	0/0
Chromosome2-3322803	DEL	PRECISE	233	0	0	0/0	0/0	0/0	0/0	0/0	0/0	0/0	0/0	0/0	0/0	0/0	0/0	0/1	0/0
Chromosome2-3322807	INS	PRECISE	6017	0	0	0/0	0/0	0/0	0/0	0/0	0/0	0/0	0/0	0/0	0/0	0/0	0/0	0/0	1/1
Chromosome2-3322808	BND	PRECISE	0	0	0	0/0	0/0	0/0	0/0	0/0	0/0	0/0	0/0	0/0	0/0	0/0	0/0	0/0	0/1
Chromosome2-3323238	DEL	PRECISE	151	0	0	0/0	0/0	0/0	0/0	0/0	0/0	0/0	0/0	0/1	0/0	0/0	0/1	0/1	0/0
Chromosome2-3323587	INS	PRECISE	7772	0	0	0/0	0/0	0/0	0/0	0/0	0/0	0/0	0/0	0/0	0/0	0/0	0/0	0/0	0/0
Chromosome2-3323601	INS	PRECISE	942	0	0	0/0	0/0	0/1	0/0	0/0	0/0	0/0	0/0	0/0	0/0	0/0	0/0	0/0	0/0
Chromosome2-3323897	INS	PRECISE	211	0	0	0/0	0/0	0/0	1/1	0/1	0/0	0/0	0/0	0/0	0/0	0/0	0/0	0/0	0/0
Chromosome2-3324917	DEL	PRECISE	181	0	0	0/0	0/1	0/0	0/0	0/0	0/0	0/1	0/0	0/0	0/1	1/1	0/0	0/0	0/1
Chromosome2-3325023	DEL	PRECISE	675	0	0	0/0	0/0	0/0	0/0	0/0	0/0	0/0	0/0	0/0	0/1	0/0	0/0	0/0	0/0
Chromosome2-3325201	INS	PRECISE	136	0	0	0/0	0/0	0/0	0/0	0/0	0/0	0/0	0/0	0/0	0/0	0/0	0/0	0/1	0/0
Chromosome2-3325248	INS	PRECISE	1389	0	0	0/0	0/0	0/1	0/0	0/0	0/0	0/0	0/0	0/0	0/0	0/0	0/0	0/0	0/0
Chromosome2-3325250	DEL	PRECISE	1584	0	0	0/0	0/1	0/0	0/0	0/0	0/0	0/1	0/0	0/0	0/1	1/1	0/1	0/0	0/1
Chromosome2-3328203	INS	PRECISE	1299	0	0	0/0	0/0	0/0	0/0	0/0	0/0	0/0	0/0	0/0	0/0	0/0	0/0	0/0	0/1
Chromosome2-3328765	DEL	PRECISE	363	0	0	0/0	0/1	0/0	0/0	0/0	0/0	0/1	0/0	0/1	0/1	1/1	1/1	0/1	1/1
Chromosome2-3328899	INS	PRECISE	871	0	0	0/0	0/0	1/1	0/0	0/0	0/0	0/0	0/0	0/0	0/0	0/0	0/0	0/0	0/0
Chromosome2-3328981	DEL	PRECISE	912	0	0	0/0	0/1	0/1	0/0	0/0	0/0	0/1	0/0	0/1	0/1	1/1	1/1	1/1	1/1
Chromosome2-3329369	INS	PRECISE	161	0	0	0/0	0/0	0/1	0/0	0/0	0/0	0/0	0/0	0/0	0/0	0/0	0/0	0/0	0/0
Chromosome2-3329574	INS	PRECISE	364	0	0.577	0/0	0/0	0/0	0/0	0/0	0/0	0/0	0/0	0/1	0/1	0/0	1/1	0/1	0/0
Chromosome2-3329881	DEL	PRECISE	901	0	0	0/0	0/0	0/1	0/0	0/0	0/0	0/0	0/0	0/1	0/1	0/0	1/1	1/1	0/0
Chromosome2-3330358	DEL	PRECISE	218	0	0	0/0	0/1	0/0	0/0	0/0	0/0	0/1	0/0	0/1	0/1	1/1	1/1	0/1	0/1
Chromosome2-3331312	INS	PRECISE	462	0	0.957	0/0	0/1	0/0	0/0	0/0	0/0	0/1	0/0	0/0	0/0	1/1	0/0	0/0	0/1
Chromosome2-3331847	INS	PRECISE	137	0	0	0/0	0/0	0/1	0/0	0/0	0/0	0/0	0/0	0/0	0/0	0/0	0/0	0/0	0/0
Chromosome2-3332358	DEL	PRECISE	1337	0	0	0/0	0/0	0/1	0/0	0/0	0/0	0/0	0/0	0/0	0/0	0/0	0/0	0/0	0/0
Chromosome2-3335149	INS	PRECISE	276	0.707	0	0/0	0/0	0/0	0/0	0/0	0/0	1/1	0/1	0/0	0/0	0/0	0/0	0/0	0/0
Chromosome2-3336696	BND	PRECISE	0	0	0	0/0	0/0	0/0	0/0	0/0	0/0	1/1	0/1	0/0	0/0	0/0	0/0	0/0	0/0
Chromosome2-3336697	BND	PRECISE	0	0	0	0/0	0/0	0/0	0/0	0/0	0/0	0/1	0/0	0/0	0/0	0/0	0/0	0/0	0/0
Chromosome2-3337130	INS	PRECISE	870	0	0.5	0/0	0/1	0/0	0/0	0/0	0/0	0/1	0/0	0/0	0/0	0/0	0/0	0/0	0/0
Chromosome2-3337252	INS	PRECISE	174	0	0	0/0	0/0	0/0	0/0	0/0	0/0	0/0	0/0	0/0	0/0	0/0	0/0	0/1	0/1
Chromosome2-3337795	DEL	PRECISE	946	0	0	0/0	0/1	0/0	0/0	0/0	0/0	1/1	0/1	0/1	0/1	1/1	1/1	1/1	1/1
Chromosome2-3338327	BND	PRECISE	0	0	0	0/0	0/0	0/0	0/1	0/0	0/0	0/0	0/0	0/0	0/0	0/0	0/0	0/0	0/0
Chromosome2-3338478	DEL	PRECISE	252	0	0	0/0	0/1	0/1	0/0	0/0	0/0	1/1	0/1	0/1	0/0	1/1	1/1	1/1	1/1
Chromosome2-3338754	DEL	PRECISE	237	0	0	0/0	0/0	0/0	0/0	0/0	0/0	1/1	0/1	0/1	0/0	1/1	1/1	1/1	1/1
Chromosome2-3339337	DEL	PRECISE	158	0	0	0/0	0/0	0/0	0/0	0/0	0/0	0/0	0/0	0/0	0/0	0/0	0/0	0/1	0/0
Chromosome2-3339873	INS	PRECISE	703	0	0.816	0/0	0/0	0/0	0/0	0/0	0/0	0/1	0/0	0/0	0/0	1/1	0/0	0/0	0/1
Chromosome2-3340345	INS	PRECISE	930	0	0	0/0	0/0	0/0	0/0	0/0	0/0	0/0	0/0	0/0	0/0	0/0	0/0	0/0	0/1
Chromosome2-3341826	INS	PRECISE	55	0	0.354	0/0	0/1	0/0	0/0	0/0	0/0	0/1	0/0	0/1	0/1	1/1	1/1	0/1	0/1
Chromosome2-3341962	INS	PRECISE	999	0	0	0/0	0/0	0/0	0/0	0/0	0/0	0/0	0/0	0/0	0/0	0/0	0/0	0/0	0/1
Chromosome2-3342241	DEL	PRECISE	248	0.354	0.354	0/0	0/1	0/0	0/0	0/0	0/0	0/1	0/0	0/1	0/1	1/1	1/1	0/1	0/1
Chromosome2-3343758	DEL	PRECISE	145	0	0	0/0	0/0	0/0	0/0	0/0	0/0	0/0	0/0	0/0	0/0	0/0	0/0	0/1	0/1
Chromosome2-3344213	INS	PRECISE	201	0	0.378	0/0	0/1	0/0	0/0	0/0	0/0	0/1	0/0	0/1	0/0	1/1	0/1	0/0	0/1
Chromosome2-3345996	INS	PRECISE	58	0	0	0/0	0/0	0/1	0/0	0/0	0/0	0/0	0/0	0/0	0/0	0/0	0/0	0/0	0/0

Chromosome2-3346531	DEL	PRECISE	2984	0	0	0/0	0/1	0/0	0/0	0/0	0/0	0/1	0/0	0/1	0/0	1/1	1/1	0/1	0/1
Chromosome2-3346832	DEL	PRECISE	2770	0	0	0/0	0/1	0/0	0/0	0/0	0/0	0/1	0/0	0/1	0/0	1/1	1/1	0/1	0/1
Chromosome2-3347887	DEL	PRECISE	79	0	0	0/0	0/1	0/0	0/0	0/0	0/0	0/1	0/0	0/1	0/1	1/1	1/1	0/1	0/1
Chromosome2-3348534	INS	PRECISE	64	0	0	0/0	0/1	0/0	0/0	0/0	0/0	0/1	0/0	0/1	0/1	1/1	1/1	0/1	0/1
Chromosome2-3348950	DEL	PRECISE	77	0	0	0/0	0/0	0/0	0/0	0/0	0/0	0/0	0/0	0/0	0/0	0/0	0/0	0/1	1/1
Chromosome2-3349933	INS	PRECISE	132	0	0	0/0	0/0	0/0	0/0	0/0	0/0	0/0	0/0	0/1	0/1	0/1	1/1	0/1	0/0
Chromosome2-3350304	DEL	PRECISE	51	0	0	0/0	0/1	0/0	0/0	0/0	0/0	0/1	0/0	0/0	0/0	0/1	0/0	0/0	0/1
Chromosome2-3350622	INS	PRECISE	3348	0	0	0/0	0/0	1/1	0/0	0/0	0/0	0/0	0/0	0/0	0/0	0/0	0/0	0/0	0/0
Chromosome2-3350795	INS	PRECISE	7006	0	0	0/0	0/0	1/1	0/0	0/0	0/0	0/0	0/0	0/0	0/0	0/0	0/0	0/0	0/0
Chromosome2-3350868	DEL	PRECISE	722	0	0	0/0	0/1	0/0	0/0	0/0	0/0	0/1	0/0	0/0	0/1	1/1	0/0	0/0	0/0
Chromosome2-3351381	INS	PRECISE	72	0	0.5	0/0	0/1	0/1	0/0	0/0	0/0	0/1	0/0	0/1	0/1	1/1	1/1	0/1	0/1
Chromosome2-3351421	DEL	PRECISE	326	0	0	0/0	0/0	0/0	1/1	0/1	0/1	0/1	1/1	0/0	0/1	0/0	0/0	0/0	0/0
Chromosome2-3351506	INS	PRECISE	164	0	0.463	0/0	0/1	0/0	0/0	0/0	0/0	0/1	0/0	0/1	0/1	1/1	1/1	0/1	0/1
Chromosome2-3351709	INS	PRECISE	94	0	0	0/0	0/0	0/1	0/0	0/0	0/0	0/0	0/0	0/0	0/0	0/0	0/0	0/0	0/0
Chromosome2-3352036	DEL	PRECISE	59	0	0	0/0	0/0	0/0	0/0	0/1	0/0	0/0	0/0	0/0	0/0	0/0	0/0	0/0	0/0
Chromosome2-3352198	BND	PRECISE	0	0	0	0/0	0/0	0/0	0/0	0/0	0/0	0/0	0/0	0/1	0/0	0/0	0/0	0/0	0/0
Chromosome2-3352201	INS	PRECISE	4584	0	0.707	0/0	0/0	0/0	0/0	0/0	0/0	0/0	0/0	0/0	0/0	0/0	0/0	1/1	1/1
Chromosome2-3352202	BND	PRECISE	0	0	0	0/0	0/0	0/0	0/0	0/0	0/0	0/0	0/0	0/0	0/0	0/0	0/0	0/1	0/1
Chromosome2-3352990	DUP	PRECISE	24823	0	0	0/0	0/0	0/0	0/1	0/0	0/0	0/0	0/0	0/0	0/0	0/0	0/0	0/0	0/0
Chromosome2-3355170	INS	PRECISE	50	0	0	0/0	0/0	0/1	0/0	0/0	0/0	0/0	0/0	0/0	0/0	0/0	0/0	0/0	0/0
Chromosome2-3356018	DEL	PRECISE	72	0	0	0/0	0/0	0/0	0/0	0/1	0/0	0/0	0/0	0/0	0/0	0/0	0/0	0/0	0/0
Chromosome2-3356161	DEL	PRECISE	905	0.447	0.447	0/0	0/0	0/0	0/0	0/0	0/0	0/1	0/0	0/0	0/1	1/1	1/1	0/0	0/0
Chromosome2-3356699	DEL	PRECISE	9190	0	0	0/0	0/1	0/0	0/0	0/0	0/0	0/1	0/0	0/0	0/0	0/0	0/1	0/0	1/1
Chromosome2-3357169	INS	PRECISE	5310	0	0	0/0	0/0	0/0	1/1	0/0	0/0	0/0	0/0	0/0	0/0	0/0	0/0	0/0	0/0
Chromosome2-3357601	INS	PRECISE	93	0	0	0/0	0/0	0/0	0/0	0/0	0/0	0/0	0/0	0/0	0/1	1/1	1/1	0/0	0/0
Chromosome2-3358041	DEL	PRECISE	98	0	0	0/0	0/0	0/0	0/0	0/0	0/0	0/0	0/0	0/0	0/1	1/1	1/1	0/0	0/0
Chromosome2-3358800	INS	PRECISE	7202	0	0	0/0	0/0	1/1	0/0	0/0	0/0	0/0	0/0	0/0	0/0	0/0	0/0	0/0	0/0
Chromosome2-3358800	BND	PRECISE	0	0	0	0/0	0/0	0/1	0/0	0/0	0/0	0/0	0/0	0/0	0/0	0/0	0/0	0/0	0/0
Chromosome2-3359764	DEL	PRECISE	11486	0	0	0/0	0/0	0/1	0/0	0/0	0/0	0/0	0/0	0/0	0/0	0/0	0/0	0/0	0/0
Chromosome2-3359811	INS	PRECISE	1133	0	0	0/0	0/0	0/0	0/0	0/1	0/0	0/0	0/0	0/0	0/0	0/0	0/0	0/0	0/0
Chromosome2-3361646	INS	PRECISE	128	0	0	0/0	0/1	0/0	0/0	0/0	0/0	0/1	0/0	0/0	0/0	0/1	0/0	0/0	0/1
Chromosome2-3361649	DEL	PRECISE	1170	0	0	0/0	0/0	0/0	0/0	0/0	0/0	0/0	0/0	0/1	0/0	0/0	0/0	0/1	0/0
Chromosome2-3361908	DEL	PRECISE	1327	0	0	0/0	0/1	0/0	0/0	0/0	0/0	0/1	0/0	0/1	0/1	1/1	1/1	0/1	0/1
Chromosome2-3362687	DEL	PRECISE	122	0	0	0/0	0/1	0/0	0/0	0/0	0/0	0/1	0/0	0/1	0/1	1/1	1/1	1/1	1/1
Chromosome2-3362886	DEL	PRECISE	12611	0	0	0/0	0/0	0/1	0/0	0/0	0/0	0/0	0/0	0/0	0/0	0/0	0/0	0/0	0/0
Chromosome2-3365436	DEL	PRECISE	150	0	0	0/0	0/1	0/1	0/0	1/1	0/1	0/1	0/1	0/0	1/1	1/1	0/0	1/1	1/1
Chromosome2-3366351	INS	PRECISE	872	0	0.577	0/0	0/0	0/0	0/0	1/1	0/1	0/0	0/0	0/0	0/1	0/0	0/0	0/0	0/0
Chromosome2-3366850	INS	PRECISE	1200	0	0.518	0/0	0/1	0/0	0/0	0/0	0/0	0/1	0/0	0/1	0/1	1/1	1/1	0/1	0/1
Chromosome2-3367233	DEL	PRECISE	995	0	0	0/0	0/1	0/1	1/1	1/1	0/1	1/1	1/1	0/1	1/1	1/1	1/1	1/1	1/1
Chromosome2-3367936	INS	PRECISE	211	0	0.816	0/0	0/0	0/0	0/0	0/0	0/0	0/0	0/0	0/1	0/0	1/1	1/1	0/1	0/0
Chromosome2-3368685	INS	PRECISE	239	0	0	0/0	0/0	0/1	0/0	0/0	0/0	0/0	0/0	0/0	0/0	0/0	0/0	0/0	0/0
Chromosome2-3368867	INS	PRECISE	795	0	0.991	0/0	0/1	0/0	0/0	0/0	0/0	0/1	0/0	0/1	0/1	1/1	1/1	0/1	0/1
Chromosome2-3369241	INS	PRECISE	160	0	0.753	0/0	0/1	0/0	0/0	0/0	0/0	0/1	0/0	0/0	0/1	1/1	0/0	0/0	0/0
Chromosome2-3369407	DEL	PRECISE	87	0	0	0/0	0/0	0/1	0/0	0/0	0/0	0/0	0/0	0/0	0/0	0/0	0/0	0/0	0/0
Chromosome2-3369488	INS	PRECISE	3100	0	0	0/0	0/0	0/0	0/0	0/1	0/0	0/0	0/0	0/0	0/0	0/0	0/0	0/0	0/0
Chromosome2-3369936	DEL	PRECISE	128	0	0	0/0	0/1	0/0	0/0	0/0	0/0	0/1	0/0	0/1	0/1	1/1	1/1	0/1	0/1
Chromosome2-3370188	DEL	PRECISE	136	0	0	0/0	0/1	0/1	1/1	1/1	1/1	1/1	1/1	0/1	1/1	1/1	1/1	1/1	1/1
Chromosome2-3371475	DUP	PRECISE	7124133	0	0	0/0	0/0	0/0	0/1	0/0	0/0	0/0	0/0	0/0	0/0	0/0	0/0	0/0	0/0
Chromosome2-3371475	BND	PRECISE	0	0	0	0/0	0/0	0/0	0/0	0/0	0/0	0/0	0/0	0/0	0/1	0/0	0/0	0/0	0/0
Chromosome2-3371541	DEL	PRECISE	111	0	0	0/0	0/0	0/0	0/0	0/0	0/0	0/0	0/0	0/0	1/1	0/0	0/0	0/0	0/0
Chromosome2-3371631	INS	PRECISE	1369	0	0	0/0	0/0	0/0	0/0	0/0	0/0	0/0	0/0	0/0	1/1	0/0	0/0	0/0	0/0
Chromosome2-3372209	BND	PRECISE	0	0	0	0/0	0/0	0/0	0/0	0/1	0/0	0/0	0/0	0/0	0/0	0/0	0/0	0/0	0/0
Chromosome2-3372210	BND	PRECISE	0	0	0	0/0	0/0	0/0	0/1	0/1	0/0	0/0	0/0	0/1	0/0	0/0	0/0	0/0	0/0
Chromosome2-3372465	DEL	PRECISE	2522	0	0	0/0	0/0	0/0	1/1	0/0	1/1	0/0	0/1	0/0	1/1	0/0	0/0	0/0	0/0
Chromosome2-3372653	INS	PRECISE	1249	0	0	0/0	0/0	0/1	0/0	0/0	0/0	0/0	0/0	0/0	0/0	0/0	0/0	0/0	0/0
Chromosome2-3372761	BND	PRECISE	0	0	0	0/0	0/0	0/0	0/0	0/0	0/1	0/0	0/1	0/0	0/1	0/0	0/0	0/0	0/0
Chromosome2-3372833	INS	PRECISE	54	0	0.5	0/0	0/0	0/0	1/1	0/0	0/1	0/0	0/1	0/0	1/1	0/0	0/0	0/0	0/0
Chromosome2-3372851	BND	PRECISE	0	0.816	0	0/0	0/0	0/0	0/0	0/0	0/0	0/1	0/0	0/1	0/0	1/1	0/1	0/1	0/0
Chromosome2-3372890	INS	PRECISE	932	0	0	0/0	0/0	0/0	0/0	1/1	0/0	0/0	0/0	0/0	0/0	0/0	0/0	0/0	0/0
Chromosome2-3373661	DEL	PRECISE	317	0	0	0/0	0/1	0/0	0/0	0/0	0/0	0/1	0/0	1/1	0/1	1/1	1/1	0/1	0/1
Chromosome2-3374607	DEL	PRECISE	74	0	0	0/0	0/0	0/0	0/0	0/0	0/0	0/0	0/0	0/1	0/0	0/0	0/1	0/1	0/0
Chromosome2-3374772	DEL	PRECISE	5202	0	0	0/0	0/1	0/1	0/0	0/0	0/0	0/1	0/0	0/1	0/1	1/1	1/1	0/1	0/1
Chromosome2-3375572	DEL	PRECISE	1705	0	0	0/0	0/1	0/0	0/0	0/0	0/0	0/1	0/0	0/1	0/1	1/1	1/1	0/1	0/1
Chromosome2-3376062	DEL	PRECISE	483	0	0	0/0	0/1	0/0	0/0	0/0	0/0	0/1	0/0	0/1	0/1	1/1	1/1	0/1	0/1
Chromosome2-3376639	INS	PRECISE	137	0	0	0/0	0/0	0/1	0/0	0/0	0/0	0/0	0/0	0/0	0/0	0/0	0/0	0/0	0/0
Chromosome2-3376667	DEL	PRECISE	8386	0	0	0/0	0/1	0/1	0/0	0/1	0/0	0/1	0/0	0/1	0/1	1/1	1/1	1/1	1/1
Chromosome2-3378162	DEL	PRECISE	3063	0	0	0/0	0/0	0/0	1/1	0/1	0/0	0/0	0/0	0/0	0/0	0/0	0/0	0/0	0/0
Chromosome2-3378649	DEL	PRECISE	2328	0	0	0/0	0/0	0/0	0/0	0/0	0/0	0/0	0/0	0/0	0/0	0/0	0/1	0/0	0/0
Chromosome2-3378967	INS	PRECISE	1479	0	0	0/0	0/0	0/0	0/0	0/0	0/0	0/0	0/0	0/0	0/0	0/1	0/0	0/0	0/0
Chromosome2-3379012	DEL	PRECISE	107	0	0	0/0	0/0	0/0	0/0	0/0	0/0	0/0	0/0	0/0	0/0	0/0	0/0	0/1	0/0
Chromosome2-3379314	DEL	PRECISE	444	0	0	0/0	0/1	0/0	1/1	1/1	0/1	1/1	1/1	0/1	1/1	1/1	1/1	1/1	1/1
Chromosome2-3380708	INS	PRECISE	203	0	0	0/0	0/0	0/1	0/0	0/0	0/0	0/0	0/0	0/0	0/0	0/0	0/0	0/1	0/1
Chromosome2-3382677	DEL	PRECISE	78	0	0	0/0	0/0	0/0	0/0	0/0	0/0	0/0	0/0	0/0	0/0	0/0	0/0	0/1	0/1

Chromosome2-3382893	INS	PRECISE	51	0	0	0/0	0/0	0/0	0/0	0/0	0/0	0/0	0/0	0/0	0/0	0/0	0/0	1/1	1/1
Chromosome2-3383199	DEL	PRECISE	82	0	0	0/0	0/0	0/0	0/0	0/1	1/1	0/0	0/1	0/0	0/1	0/0	0/0	0/0	0/0
Chromosome2-3383306	DEL	PRECISE	77	0	0	0/0	0/1	0/0	0/0	0/0	0/0	0/1	0/0	0/0	0/1	1/1	1/1	0/1	0/1
Chromosome2-3384101	DEL	PRECISE	1470	0	0	0/0	0/1	0/0	0/0	0/0	0/0	0/1	0/0	0/1	0/1	1/1	1/1	1/1	0/1
Chromosome2-3386209	INS	PRECISE	5160	0	0	0/0	0/0	0/0	0/0	0/0	0/0	0/0	0/0	0/0	0/0	0/0	0/0	0/0	1/1
Chromosome2-3386826	DEL	PRECISE	959	0	0	0/0	0/1	0/0	0/0	0/0	0/0	0/1	0/0	0/1	0/1	1/1	1/1	0/1	0/1
Chromosome2-3386937	DEL	PRECISE	723	0	0	0/0	0/0	0/0	0/0	0/0	0/0	0/0	0/0	0/1	0/0	0/0	0/1	0/1	0/0
Chromosome2-3388080	DEL	PRECISE	4970	0	0	0/0	0/0	0/0	0/0	0/0	0/0	0/0	0/1	0/0	0/0	0/0	0/0	0/0	0/0
Chromosome2-3388583	DEL	PRECISE	428	0	0	0/0	0/0	0/0	0/1	0/0	0/0	0/0	0/1	0/0	0/0	0/0	0/0	0/0	0/0
Chromosome2-3388628	DEL	PRECISE	4617	0	0	0/0	0/0	0/0	0/1	0/0	0/0	0/0	0/1	0/0	0/0	0/0	0/0	0/0	0/0
Chromosome2-3388748	INS	PRECISE	1104	0	0	0/0	0/0	0/0	0/0	0/1	0/0	0/0	0/0	0/0	0/0	0/0	0/0	0/0	0/0
Chromosome2-3388979	INS	PRECISE	982	0	0	0/0	0/0	0/0	0/0	0/0	0/0	0/0	0/0	0/0	0/0	0/0	0/0	0/0	1/1
Chromosome2-3389136	INS	PRECISE	116	0	0	0/0	0/0	0/1	0/0	0/0	0/0	0/0	0/0	0/0	0/0	0/0	0/0	0/0	0/0
Chromosome2-3389521	INS	PRECISE	150	0	0	0/0	0/0	0/0	0/0	0/0	0/0	0/0	0/0	0/0	0/0	0/0	0/0	0/0	0/1
Chromosome2-3389938	DEL	PRECISE	2904	0	0	0/0	0/0	0/0	0/0	0/1	0/0	0/0	0/0	0/0	0/0	0/0	0/0	0/0	0/0
Chromosome2-3390033	DEL	PRECISE	1922	0	0	0/0	0/0	0/1	0/0	0/1	0/1	1/1	1/1	0/0	1/1	0/0	0/0	1/1	0/0
Chromosome2-3390262	DEL	PRECISE	918	0	0	0/0	0/0	0/1	0/0	0/1	0/0	0/0	0/0	0/0	0/0	0/0	0/0	1/1	0/0
Chromosome2-3390970	DUP	PRECISE	19501	0	0	0/0	0/1	0/0	0/0	0/0	0/0	0/0	0/0	0/0	0/0	0/1	0/0	0/0	0/1
Chromosome2-3391500	INS	PRECISE	709	0	0	0/0	0/0	0/0	1/1	0/0	0/0	0/0	0/0	0/0	0/0	0/0	0/0	0/0	0/0
Chromosome2-3391602	BND	PRECISE	0	0	0	0/0	0/0	0/0	0/0	0/0	0/0	0/0	0/0	0/0	0/0	0/0	0/0	0/0	0/1
Chromosome2-3392836	BND	PRECISE	0	0	0	0/0	0/0	0/0	0/0	0/0	0/0	0/0	0/0	0/0	1/1	0/1	0/0	0/0	0/0
Chromosome2-3395419	DEL	PRECISE	59	0	0	0/0	0/0	0/0	0/0	0/1	0/0	0/0	0/0	0/0	0/0	0/0	0/0	0/0	0/0
Chromosome2-3396218	BND	PRECISE	0	0	0	0/0	0/0	0/0	0/0	0/1	0/0	0/0	0/0	0/0	0/0	0/0	0/0	0/0	0/0
Chromosome2-3396554	DEL	PRECISE	2338	0	0	0/0	0/0	0/0	0/0	0/0	0/0	0/1	1/1	0/0	0/0	0/0	0/0	0/0	0/0
Chromosome2-3396563	INV	PRECISE	357	0	0	0/0	0/0	0/0	0/0	0/1	0/1	0/0	0/1	0/0	0/1	0/0	0/0	0/0	0/0
Chromosome2-3397049	DEL	PRECISE	1537	0	0	0/0	0/0	0/0	1/1	0/0	0/0	0/0	0/0	0/1	0/0	0/0	1/1	1/1	1/1
Chromosome2-3397290	DEL	PRECISE	59	0	0	0/0	0/0	0/0	0/0	0/0	0/0	0/0	0/0	0/1	0/0	0/0	0/1	1/1	0/0
Chromosome2-3397786	DEL	PRECISE	1036	0	0	0/0	0/1	0/1	1/1	1/1	0/1	1/1	1/1	0/1	1/1	1/1	1/1	1/1	1/1
Chromosome2-3399342	DEL	PRECISE	675	0	0	0/0	0/0	0/0	0/0	0/0	0/0	0/0	0/0	0/0	0/0	0/0	0/0	0/1	1/1
Chromosome2-3400410	INS	PRECISE	454	0	0	0/0	0/0	0/1	0/0	0/0	0/0	0/0	0/0	0/0	0/0	0/0	0/0	0/0	0/0
Chromosome2-3400659	INS	PRECISE	139	0	0.577	0/0	0/0	0/0	0/0	0/0	0/0	0/0	0/0	0/1	0/0	0/0	1/1	0/0	0/0
Chromosome2-3401065	DEL	PRECISE	120	0	0	0/0	0/0	0/0	0/0	0/0	0/0	0/0	0/0	0/0	0/0	0/0	0/0	0/0	1/1
Chromosome2-3401153	INS	PRECISE	9968	0	0	0/0	0/0	0/0	1/1	0/0	0/0	0/0	0/0	0/0	0/0	0/0	0/0	0/0	0/0
Chromosome2-3401603	DEL	PRECISE	1552	0	0	0/0	0/0	0/0	0/0	0/0	0/0	0/1	0/0	0/0	0/1	1/1	0/0	0/0	1/1
Chromosome2-3402060	INS	PRECISE	137	0	0	0/0	0/0	0/0	0/0	0/0	0/0	0/0	0/0	0/0	0/0	0/0	0/0	1/1	0/0
Chromosome2-3402187	DEL	PRECISE	210	0	0	0/0	0/0	0/0	0/0	0/0	0/0	0/0	0/0	0/0	0/0	0/0	0/0	0/1	0/1
Chromosome2-3402324	INS	PRECISE	137	0	0	0/0	0/0	0/1	0/0	0/0	0/0	0/0	0/0	0/0	0/0	0/0	0/0	0/0	0/0
Chromosome2-3402822	INS	PRECISE	865	0	0.408	0/0	0/1	0/0	0/0	0/0	0/0	0/1	0/0	0/0	0/1	1/1	0/1	0/0	0/1
Chromosome2-3403069	INS	PRECISE	8171	0	0	0/0	0/0	0/0	0/0	0/0	0/0	0/0	0/0	0/0	0/0	0/0	0/0	1/1	0/0
Chromosome2-3403070	BND	PRECISE	0	0	0	0/0	0/0	0/0	0/0	0/0	0/0	0/0	0/0	0/0	0/0	0/0	0/0	1/1	0/0
Chromosome2-3403073	DEL	PRECISE	63	0	0	0/0	0/0	0/0	0/0	0/0	0/0	0/0	0/0	0/0	0/0	0/0	0/0	0/0	0/1
Chromosome2-3403567	DEL	PRECISE	73	0	0	0/0	0/1	0/0	0/0	0/0	0/0	0/1	0/0	0/0	0/1	1/1	1/1	0/0	0/1
Chromosome2-3403632	INS	PRECISE	211	0	0	0/0	0/0	0/0	1/1	0/1	0/0	0/1	0/1	0/0	0/0	0/0	0/0	0/0	0/0
Chromosome2-3403855	INS	PRECISE	401	0	0	0/0	0/0	0/0	0/0	0/0	0/0	0/0	0/0	0/0	0/0	0/0	0/0	1/1	0/0
Chromosome2-3403939	INS	PRECISE	211	0	0	0/0	0/0	0/0	0/0	0/0	0/0	0/0	0/0	0/0	0/0	0/0	0/0	1/1	0/0
Chromosome2-3404092	INS	PRECISE	58	0	0	0/0	0/1	0/0	0/0	0/0	0/0	0/1	0/0	0/1	0/1	1/1	1/1	0/0	1/1
Chromosome2-3404343	INS	PRECISE	5938	0	0.5	0/0	0/0	0/0	0/0	1/1	1/1	0/0	0/1	0/0	1/1	0/0	0/0	0/0	0/0
Chromosome2-3404343	BND	PRECISE	0	0	0	0/0	0/0	0/0	0/0	0/1	0/1	0/0	0/1	0/0	0/1	0/0	0/0	0/0	0/0
Chromosome2-3404504	INS	PRECISE	132	0	0.816	0/0	0/1	0/0	0/0	0/0	0/0	0/1	0/0	0/0	0/1	1/1	0/0	0/0	0/1
Chromosome2-3404858	DEL	PRECISE	1382	0	0	0/0	0/0	0/0	0/0	0/0	0/0	0/0	0/0	0/0	0/0	0/0	0/0	1/1	0/0
Chromosome2-3405008	DEL	PRECISE	706	0	0	0/0	0/0	0/0	0/0	0/0	0/0	0/0	0/0	0/0	0/0	0/0	0/0	0/0	1/1
Chromosome2-3405068	INS	PRECISE	52	0	0	0/0	0/0	0/1	0/0	0/0	0/0	0/0	0/0	0/0	0/0	0/0	0/0	1/1	0/0
Chromosome2-3405149	DEL	PRECISE	90	0	0	0/0	0/1	0/0	0/0	0/0	0/0	0/1	0/0	0/1	0/1	1/1	1/1	0/1	1/1
Chromosome2-3407810	INS	PRECISE	8463	0	0	0/0	0/0	0/1	0/0	0/0	0/0	0/0	0/0	0/0	0/0	0/0	0/0	0/0	0/0
Chromosome2-3407924	DEL	PRECISE	1675	0	0	0/0	0/0	0/0	0/0	0/0	0/0	0/0	0/0	0/0	0/0	0/0	0/0	1/1	1/1
Chromosome2-3408178	INS	PRECISE	1488	0	0.535	0/0	0/1	0/0	0/0	0/0	0/0	0/1	0/0	0/1	0/1	1/1	1/1	0/1	0/1
Chromosome2-3408476	BND	PRECISE	0	0	0	0/0	0/0	0/0	0/0	0/0	0/0	0/0	0/0	0/0	0/0	0/0	0/0	0/0	0/1
Chromosome2-3408512	INS	PRECISE	368	0	0	0/0	0/0	0/0	0/0	0/0	0/0	0/0	0/0	0/1	0/0	0/0	0/1	0/1	0/0
Chromosome2-3408839	INS	PRECISE	1084	0	0	0/0	0/0	0/1	0/0	0/0	0/0	0/0	0/0	0/0	0/0	0/0	0/0	0/0	0/0
Chromosome2-3409144	DEL	PRECISE	23251	0	0	0/0	0/1	0/0	0/0	0/0	0/0	0/1	0/0	0/1	0/1	0/0	0/0	0/1	0/1
Chromosome2-3409391	DEL	PRECISE	75	0	0	0/0	0/0	0/1	0/0	0/0	0/0	0/0	0/0	0/0	0/0	0/0	0/0	0/0	0/0
Chromosome2-3409785	INS	PRECISE	76	0	0	0/0	0/0	0/1	0/0	0/0	0/0	0/0	0/0	0/0	0/0	0/0	0/0	0/0	0/0
Chromosome2-3410091	DEL	PRECISE	12497	0	0	0/0	0/0	0/1	0/0	0/0	0/0	0/0	0/0	0/0	0/0	0/0	0/0	1/1	1/1
Chromosome2-3410297	BND	PRECISE	0	0.447	0	0/0	0/0	0/0	0/1	0/1	0/0	0/0	0/0	0/0	0/1	0/0	0/0	0/0	0/0
Chromosome2-3411845	DEL	PRECISE	445	0	0	0/0	0/0	0/1	1/1	1/1	0/1	1/1	1/1	0/0	1/1	0/0	0/0	1/1	1/1
Chromosome2-3412279	DEL	PRECISE	15125	0	0	0/0	1/1	0/0	0/0	0/0	0/0	0/1	0/0	0/1	1/1	0/0	0/0	0/1	0/1
Chromosome2-3412488	DEL	PRECISE	1389	0	0	0/0	0/0	0/1	1/1	1/1	0/1	1/1	1/1	0/0	1/1	0/0	0/0	1/1	1/1
Chromosome2-3413221	DEL	PRECISE	5081	0	0	0/0	0/0	0/1	1/1	1/1	0/1	1/1	1/1	0/0	1/1	0/0	0/0	0/0	0/0
Chromosome2-3414069	INS	PRECISE	915	0	0.886	0/0	0/1	0/0	0/0	0/0	0/0	0/1	0/0	0/1	0/1	1/1	1/1	0/1	0/1
Chromosome2-3414470	DEL	PRECISE	53	0	0	0/0	0/0	0/0	0/0	0/0	0/0	0/0	0/0	0/0	0/0	0/0	0/0	0/1	0/1
Chromosome2-3414734	DEL	PRECISE	53	0	0	0/0	0/1	0/0	0/0	0/0	0/0	0/1	0/0	0/1	0/1	1/1	1/1	0/1	0/1
Chromosome2-3415810	BND	PRECISE	0	0	0	0/0	0/0	0/0	0/1	0/0	0/0	0/0	0/0	0/0	0/0	0/0	0/0	0/0	0/0
Chromosome2-3415810	BND	PRECISE	0	0	0	0/0	0/0	0/0	0/0	0/0	0/0	0/0	0/0	0/0	0/0	0/0	0/1	0/0	0/0

Chromosome2-3416279	BND	PRECISE	0	0	0	0/0	0/0	0/0	0/0	0/0	0/0	0/0	0/0	0/0	0/0	0/0	0/0	0/1	1/1
Chromosome2-3416311	INS	PRECISE	212	0	0	0/0	0/0	0/0	1/1	0/0	0/0	0/0	0/0	0/0	0/0	0/0	0/0	0/0	0/0
Chromosome2-3416427	BND	PRECISE	0	0	0	0/0	0/0	0/0	0/0	0/0	0/0	0/0	0/0	0/0	0/0	0/0	0/0	0/0	0/1
Chromosome2-3416562	DEL	PRECISE	62	0	0	0/0	0/0	0/0	0/0	0/0	0/0	0/0	0/0	0/0	0/0	0/0	0/0	1/1	1/1
Chromosome2-3416707	DEL	PRECISE	54	0	0	0/0	0/0	0/0	0/0	1/1	0/1	1/1	1/1	0/0	1/1	0/0	0/0	0/0	0/0
Chromosome2-3416722	INS	PRECISE	1477	0	0	0/0	0/0	0/0	0/0	0/0	0/0	0/0	0/0	0/0	0/0	0/0	0/0	1/1	1/1
Chromosome2-3417237	DUP	PRECISE	220	0	0	0/0	0/0	0/0	0/0	0/0	0/0	0/0	0/0	0/0	0/0	0/1	0/0	0/0	0/0
Chromosome2-3417245	DEL	PRECISE	682	0	0	0/0	0/0	0/0	0/0	0/0	0/0	0/0	0/0	0/1	0/0	0/0	0/1	0/1	0/0
Chromosome2-3417265	INS	PRECISE	137	0	0	0/0	0/1	0/0	0/0	0/0	0/0	0/1	0/0	0/0	0/1	1/1	0/1	0/0	0/1
Chromosome2-3418400	INS	PRECISE	122	0	0	0/0	0/0	0/0	0/0	0/0	0/0	0/0	0/0	0/0	0/0	0/0	0/0	0/0	0/1
Chromosome2-3419026	INS	PRECISE	624	0	0	0/0	0/0	0/1	0/0	0/0	0/0	0/0	0/0	0/0	0/0	0/0	0/0	0/0	0/0
Chromosome2-3419140	INS	PRECISE	2687	0	0.707	0/0	0/0	0/0	1/1	0/1	0/0	0/0	0/0	0/0	0/0	0/0	0/0	0/0	0/0
Chromosome2-3419292	INS	PRECISE	238	0	0	0/0	0/0	0/1	0/0	0/0	0/0	0/0	0/0	0/0	0/0	0/0	0/0	0/0	0/0
Chromosome2-3419824	INS	PRECISE	234	0	0.707	0/0	0/0	0/0	0/0	0/0	0/0	0/1	0/0	0/1	0/1	1/1	1/1	0/1	0/1
Chromosome2-3421080	INS	PRECISE	1297	0	0.707	0/0	0/0	0/0	0/0	0/0	0/0	0/0	0/0	0/0	0/0	0/0	0/0	1/1	1/1
Chromosome2-3421080	INS	PRECISE	12689	0	0	0/0	0/0	0/0	0/0	0/0	0/0	0/0	0/0	0/0	0/0	0/0	0/0	0/1	0/0
Chromosome2-3421097	INS	PRECISE	11425	0	0	0/0	0/0	0/0	0/0	0/0	0/0	0/0	0/0	0/0	0/0	0/0	0/0	0/1	0/0
Chromosome2-3421157	BND	PRECISE	0	0	0	0/0	0/0	0/0	0/0	0/0	0/0	0/0	0/0	0/0	0/0	0/0	0/0	0/1	0/0
Chromosome2-3421209	INS	PRECISE	4761	0	0	0/0	0/0	1/1	0/0	0/0	0/0	0/0	0/0	0/0	0/0	0/0	0/0	0/0	0/0
Chromosome2-3421356	INS	PRECISE	7279	0	0	0/0	0/0	0/0	0/0	1/1	0/0	0/0	0/0	0/0	0/0	0/0	0/0	0/0	0/0
Chromosome2-3421462	BND	PRECISE	0	0	0	0/0	0/0	0/0	0/0	0/0	0/0	0/1	0/0	0/0	0/1	0/0	0/0	0/0	0/1
Chromosome2-3421619	BND	PRECISE	0	0	0	0/0	0/0	0/1	0/0	0/0	0/0	0/0	0/0	0/0	0/0	0/0	0/0	0/0	0/0
Chromosome2-3421650	DEL	PRECISE	17441	0	0	0/0	0/1	0/0	0/0	0/0	0/0	0/1	0/0	0/1	0/1	0/0	0/0	1/1	0/1
Chromosome2-3421912	INS	PRECISE	1391	0	0	0/0	0/0	0/0	0/0	0/1	0/0	0/0	0/0	0/0	0/0	0/0	0/0	0/0	0/0
Chromosome2-3422372	DEL	PRECISE	70	0	0	0/0	0/0	0/0	1/1	0/1	0/1	1/1	1/1	0/0	1/1	0/0	0/0	0/0	0/0
Chromosome2-3424072	DEL	PRECISE	53	0	0	0/0	0/1	0/0	0/0	0/0	0/0	0/1	0/0	0/1	0/1	0/1	0/1	0/1	0/1
Chromosome2-3424113	DEL	PRECISE	4074	0	0	0/0	0/0	0/0	0/0	0/1	1/1	0/0	0/1	0/0	0/1	0/0	0/0	0/0	0/0
Chromosome2-3424289	DEL	PRECISE	317	0	0	0/0	0/1	0/0	0/0	0/0	0/0	0/1	0/0	0/1	0/1	0/1	1/1	0/1	0/1
Chromosome2-3424885	DEL	PRECISE	671	0	0	0/0	0/0	0/0	0/0	0/0	0/0	0/0	0/0	0/0	0/0	0/0	0/0	0/1	0/1
Chromosome2-3426118	DEL	PRECISE	644	0	0	0/0	0/1	0/0	0/0	0/0	0/0	0/1	0/0	0/1	0/1	1/1	1/1	0/1	0/1
Chromosome2-3426212	DEL	PRECISE	50	0	0	0/0	0/0	0/0	0/0	0/0	0/0	0/1	0/1	0/0	0/0	0/0	0/0	0/0	0/0
Chromosome2-3427626	DEL	PRECISE	364	0	0	0/0	0/0	0/1	0/0	0/0	0/0	0/0	0/0	0/0	0/0	0/0	0/0	0/0	0/0
Chromosome2-3429135	DEL	PRECISE	1101	0	0	0/0	0/1	0/0	0/0	0/0	0/0	0/1	0/0	0/0	0/1	1/1	0/1	0/0	0/1
Chromosome2-3429429	INS	PRECISE	1039	0	0	0/0	0/0	0/1	0/0	0/0	0/0	0/0	0/0	0/0	0/0	0/0	0/0	0/0	0/0
Chromosome2-3429495	DEL	PRECISE	645	0	0	0/0	0/1	0/0	1/1	1/1	0/1	1/1	1/1	0/1	1/1	1/1	1/1	1/1	1/1
Chromosome2-3429675	INS	PRECISE	236	0	0.707	0/0	0/0	0/0	0/0	0/0	0/0	0/1	0/1	0/0	0/0	0/0	0/0	0/0	0/0
Chromosome2-3430150	DEL	PRECISE	51	0	0	0/0	0/1	0/0	0/0	0/0	0/0	0/1	0/0	0/0	0/1	1/1	0/1	0/0	0/1
Chromosome2-3430170	INS	PRECISE	129	0	0	0/0	0/0	0/0	0/0	0/0	0/0	0/0	0/0	0/1	0/0	0/0	0/1	0/1	0/0
Chromosome2-3430616	DEL	PRECISE	64	0	0	0/0	0/1	0/0	0/0	0/0	0/0	0/1	0/0	0/0	0/1	1/1	0/1	0/0	0/1
Chromosome2-3431047	DEL	PRECISE	626	0	0	0/0	0/0	0/1	1/1	1/1	0/1	1/1	1/1	0/0	1/1	1/1	1/1	1/1	1/1
Chromosome2-3431334	DEL	PRECISE	222	0	0	0/0	0/0	0/0	0/0	0/0	0/0	0/0	0/0	0/0	1/1	1/1	1/1	0/0	1/1
Chromosome2-3432394	INS	PRECISE	661	0	0	0/0	0/0	0/0	0/1	0/0	0/0	0/0	0/0	0/0	0/0	0/0	0/0	0/0	0/0
Chromosome2-3432779	DEL	PRECISE	90	0	0	0/0	0/1	0/0	0/0	0/0	0/0	0/1	0/0	0/1	0/1	1/1	1/1	0/1	0/1
Chromosome2-3433508	DEL	PRECISE	76	0	0	0/0	0/1	0/0	0/0	0/0	0/0	0/1	0/0	0/0	0/1	1/1	0/1	0/0	0/1
Chromosome2-3434280	DEL	PRECISE	67	0	0	0/0	0/1	0/0	0/0	0/0	0/0	0/1	0/0	0/1	0/1	1/1	1/1	0/1	0/1
Chromosome2-3434328	INS	PRECISE	341	0	0	0/0	0/0	0/1	0/1	0/0	0/0	0/0	0/0	0/0	0/0	0/0	0/0	0/0	0/0
Chromosome2-3434723	DEL	PRECISE	739	0	0	0/0	0/1	0/0	0/0	0/0	0/0	0/1	0/0	0/1	0/1	1/1	1/1	0/1	0/1
Chromosome2-3435857	INS	PRECISE	180	0	0	0/0	0/1	0/0	0/0	0/0	0/0	0/1	0/0	0/1	0/1	1/1	1/1	0/1	0/1
Chromosome2-3435997	INS	PRECISE	777	0	0.577	0/0	0/0	0/0	0/0	0/0	0/0	0/0	0/0	0/1	0/0	0/0	0/1	0/1	0/0
Chromosome2-3436110	INS	PRECISE	7352	0	0	0/0	0/0	0/0	0/0	1/1	0/0	0/0	0/0	0/0	0/0	0/0	0/0	0/0	0/0
Chromosome2-3436179	INS	PRECISE	1522	0	0	0/0	0/0	0/0	0/0	0/1	0/0	0/0	0/0	0/0	0/0	0/0	0/0	0/0	0/0
Chromosome2-3437063	DEL	PRECISE	8141	0.527	0.527	0/0	0/1	0/0	0/0	0/0	0/0	0/1	0/0	0/1	0/1	1/1	0/0	0/0	1/1
Chromosome2-3438771	DEL	PRECISE	392	0	0	0/0	0/1	0/1	1/1	0/1	0/1	1/1	1/1	0/1	1/1	1/1	1/1	1/1	1/1
Chromosome2-3439494	INS	PRECISE	120	0	0	0/0	0/1	0/0	0/0	0/0	0/0	0/1	0/0	0/0	0/1	1/1	0/1	0/0	0/1
Chromosome2-3439638	INS	PRECISE	95	0	0.354	0/0	1/1	0/0	0/0	0/0	0/0	0/1	0/0	0/1	0/1	0/0	1/1	0/1	0/1
Chromosome2-3439740	INS	PRECISE	1311	0	0.577	0/0	0/0	0/0	0/0	0/0	0/0	0/0	0/0	0/1	0/0	0/0	1/1	0/1	0/0
Chromosome2-3440708	INS	PRECISE	905	0	0	0/0	0/0	0/1	0/0	0/0	0/0	0/0	0/0	0/0	0/0	0/0	0/0	0/0	0/0
Chromosome2-3441206	INS	PRECISE	161	0	0	0/0	0/0	0/0	0/1	0/0	0/0	0/0	0/0	0/0	0/0	0/0	0/0	0/0	0/0
Chromosome2-3441640	BND	PRECISE	0	0	0	0/0	0/0	0/0	0/0	0/0	0/0	0/0	0/0	0/0	0/0	0/0	0/0	0/1	0/1
Chromosome2-3442250	DUP	PRECISE	121	0	0	0/0	0/0	0/0	0/0	0/0	0/0	0/0	0/0	0/0	0/1	0/1	1/1	0/0	0/0
Chromosome2-3442667	DEL	PRECISE	348	0.354	0.354	0/0	0/1	0/0	0/0	0/0	0/0	0/1	0/0	0/1	0/1	1/1	1/1	0/1	0/1
Chromosome2-3443151	DEL	PRECISE	109	0	0	0/0	0/1	0/1	0/1	0/0	0/0	0/1	0/0	0/1	0/1	1/1	1/1	1/1	1/1
Chromosome2-3444127	INS	PRECISE	150	0	0	0/0	0/0	0/0	0/0	0/0	0/0	0/0	0/0	0/0	0/1	0/1	1/1	0/0	0/0
Chromosome2-3444258	INS	PRECISE	125	0	0	0/0	0/0	0/1	0/1	0/0	0/0	0/0	0/0	0/0	0/0	0/0	0/0	0/0	0/0
Chromosome2-3444559	INS	PRECISE	1369	0	0	0/0	0/0	0/0	0/1	0/0	0/0	0/0	0/0	0/0	0/0	0/0	0/0	0/0	0/0
Chromosome2-3444728	INS	PRECISE	180	0	0.951	0/0	0/1	0/0	0/0	0/0	0/0	0/1	0/0	0/0	0/0	0/1	1/1	0/0	0/0
Chromosome2-3444897	DEL	PRECISE	223	0	0	0/0	0/0	0/0	0/0	0/0	0/0	0/0	0/0	0/0	0/0	0/0	0/0	1/1	0/0
Chromosome2-3445580	INS	PRECISE	122	0	0.577	0/0	0/0	0/0	0/0	0/0	0/0	0/0	0/0	0/0	0/1	0/1	0/1	0/0	0/0
Chromosome2-3446113	BND	PRECISE	0	0	0	0/0	0/0	0/0	0/1	0/0	0/0	0/0	0/0	0/0	0/0	0/0	0/0	0/0	0/0
Chromosome2-3446182	DEL	PRECISE	56	0	0	0/0	0/0	0/0	0/1	0/0	0/0	0/0	0/0	0/0	0/0	0/0	0/0	0/0	0/0
Chromosome2-3446196	INS	PRECISE	56	0	0	0/0	0/0	0/0	0/0	0/0	0/0	0/0	0/0	0/0	0/0	0/0	0/0	0/1	0/0
Chromosome2-3446288	DEL	PRECISE	309	0	0	0/0	0/0	0/0	0/1	0/0	0/0	0/0	0/0	0/0	0/0	0/0	0/0	0/0	0/0
Chromosome2-3446454	INS	PRECISE	1760	0	0	0/0	0/0	0/0	0/0	0/0	0/0	0/0	0/0	0/0	0/0	0/0	0/0	0/0	0/1

Chromosome2-3446846	INS	PRECISE	51	0	0	0/0	0/0	0/1	0/1	1/1	0/1	1/1	1/1	0/0	1/1	0/0	0/0	1/1	1/1
Chromosome2-3446934	DEL	PRECISE	54	0	0	0/0	0/0	0/0	1/1	0/0	0/0	0/0	0/0	0/0	0/0	0/0	0/0	0/0	0/0
Chromosome2-3447273	INS	PRECISE	1369	0	0	0/0	0/0	0/0	0/0	0/0	0/0	0/0	0/0	0/0	0/0	0/0	0/0	0/0	0/1
Chromosome2-3447316	INS	PRECISE	5316	0	0	0/0	0/0	0/0	0/0	0/0	0/0	0/0	0/0	0/0	0/0	0/0	0/0	0/0	1/1
Chromosome2-3447459	DEL	PRECISE	6469	0	0	0/0	0/0	0/0	0/1	0/0	0/0	0/0	0/0	0/0	0/0	0/0	0/0	0/0	0/0
Chromosome2-3447590	INS	PRECISE	257	0	0.744	0/0	0/1	0/0	0/0	0/0	0/0	0/1	0/0	0/1	0/1	1/1	1/1	0/1	0/1
Chromosome2-3447816	BND	PRECISE	0	0	0	0/0	0/0	0/0	0/0	0/0	0/0	0/0	0/0	0/0	0/1	0/1	0/0	0/0	0/0
Chromosome2-3447873	INS	PRECISE	9078	0	0	0/0	0/0	1/1	0/0	0/0	0/0	0/0	0/0	0/0	0/0	0/0	0/0	0/0	0/0
Chromosome2-3447966	INS	PRECISE	146	0	0	0/0	0/1	0/0	0/0	0/0	0/0	0/1	0/0	0/1	0/1	1/1	1/1	0/1	0/1
Chromosome2-3448163	INS	PRECISE	1093	0	0.707	0/0	0/0	0/0	0/0	0/0	0/0	0/1	0/1	0/0	0/0	0/0	0/0	0/0	0/0
Chromosome2-3448176	INS	PRECISE	7092	0	0	0/0	0/0	0/0	1/1	0/0	0/0	0/0	0/0	0/0	0/0	0/0	0/0	0/0	0/0
Chromosome2-3448505	DEL	PRECISE	52	0	0	0/0	0/0	0/0	0/0	0/0	0/0	0/0	0/0	0/0	0/0	0/0	0/0	0/1	0/1
Chromosome2-3448649	DEL	PRECISE	150	0	0	0/0	0/1	0/0	0/0	0/0	0/0	0/1	0/0	0/1	0/1	1/1	1/1	0/1	0/1
Chromosome2-3449107	INS	PRECISE	160	0	0	0/0	0/0	0/0	0/0	0/0	0/0	0/0	0/0	0/0	0/0	0/0	0/0	0/1	0/1
Chromosome2-3449375	INS	PRECISE	1073	0	0.707	0/0	0/0	0/0	0/0	0/0	0/0	0/0	0/0	0/0	0/0	0/0	0/0	0/1	0/1
Chromosome2-3450179	INS	PRECISE	3920	0	0	0/0	0/0	0/0	0/1	0/0	0/0	0/0	0/0	0/0	0/0	0/0	0/0	0/0	0/0
Chromosome2-3450510	BND	PRECISE	0	0	0	0/0	0/0	0/0	0/0	0/0	0/0	0/0	0/1	0/0	0/0	0/0	0/0	0/0	0/0
Chromosome2-3450575	DEL	PRECISE	140	0	0	0/0	0/0	0/0	0/1	0/0	0/0	0/0	0/0	0/0	0/0	0/0	0/0	0/0	0/0
Chromosome2-3451154	DEL	PRECISE	161	0	0	0/0	0/1	0/0	0/0	0/0	0/0	0/1	0/0	0/0	0/0	0/1	0/0	0/0	0/1
Chromosome2-3451283	DEL	PRECISE	54	0	0	0/0	0/1	0/0	0/0	0/0	0/0	0/1	0/0	0/1	0/1	1/1	1/1	0/1	0/1
Chromosome2-3451374	DEL	PRECISE	211	0	0	0/0	0/1	0/1	1/1	1/1	0/1	1/1	1/1	0/1	1/1	1/1	1/1	1/1	1/1
Chromosome2-3452336	INS	PRECISE	211	0	0	0/0	0/0	0/0	0/0	0/1	0/0	0/0	0/0	0/0	0/0	0/0	0/0	0/0	0/0
Chromosome2-3452524	INS	PRECISE	123	0	0	0/0	0/0	0/0	0/1	0/0	0/0	0/0	0/0	0/0	0/0	0/0	0/0	0/0	0/0
Chromosome2-3452550	DEL	PRECISE	257	0	0	0/0	0/0	0/1	0/0	0/0	0/0	0/0	0/0	0/0	0/0	0/0	0/0	0/0	0/0
Chromosome2-3454481	DEL	PRECISE	882	0	0	0/0	0/1	0/1	0/1	1/1	0/1	0/1	0/1	0/1	1/1	1/1	1/1	1/1	1/1
Chromosome2-3454923	DEL	PRECISE	633	0	0	0/0	0/1	0/1	0/1	1/1	0/1	0/1	0/1	0/1	1/1	1/1	1/1	1/1	1/1
Chromosome2-3455382	INS	PRECISE	65	0	0	0/0	0/0	0/0	0/0	0/0	0/0	0/0	0/0	0/0	0/1	0/1	1/1	0/0	0/0
Chromosome2-3456017	DEL	PRECISE	108	0	0	0/0	0/1	0/0	0/0	0/0	0/0	0/1	0/0	0/1	0/1	1/1	1/1	0/1	0/1
Chromosome2-3456695	INS	PRECISE	354	0	0.577	0/0	0/0	0/0	0/0	0/0	0/0	0/0	0/0	0/1	0/0	0/0	0/1	0/1	0/0
Chromosome2-3458162	DEL	PRECISE	107	0	0	0/0	0/0	0/0	0/0	0/0	0/0	0/0	0/0	0/1	0/0	0/0	0/1	0/1	0/0
Chromosome2-3458165	INS	PRECISE	5378	0	0	0/0	0/0	1/1	0/0	0/0	0/0	0/0	0/0	0/0	0/0	0/0	0/0	0/0	0/0
Chromosome2-3458925	INS	PRECISE	888	0	0	0/0	0/0	0/1	0/0	0/0	0/0	0/0	0/0	0/0	0/0	0/0	0/0	0/0	0/0
Chromosome2-3460162	DEL	PRECISE	1001	0	0	0/0	0/0	0/1	1/1	1/1	0/1	1/1	1/1	0/0	0/1	0/0	0/0	0/1	0/1
Chromosome2-3460694	INS	PRECISE	137	0	0	0/0	0/0	0/0	0/0	1/1	0/1	1/1	1/1	0/0	1/1	0/0	0/0	0/0	0/0
Chromosome2-3460848	DEL	PRECISE	862	0	0	0/0	0/0	0/0	0/0	0/0	0/0	0/0	0/0	0/1	0/1	1/1	1/1	0/1	0/0
Chromosome2-3461174	INS	PRECISE	12373	0	0	0/0	0/0	0/0	1/1	0/0	0/0	0/0	0/0	0/0	0/0	0/0	0/0	0/0	0/0
Chromosome2-3461174	BND	PRECISE	0	0	0	0/0	0/0	0/0	0/1	0/0	0/0	0/0	0/0	0/0	0/0	0/0	0/0	0/0	0/0
Chromosome2-3461300	DEL	PRECISE	807	0	0	0/0	0/0	0/0	0/1	0/0	0/0	0/0	0/0	0/0	0/0	0/0	0/0	0/0	0/0
Chromosome2-3461840	DEL	PRECISE	58	0	0	0/0	0/1	0/0	0/0	0/0	0/0	0/1	0/0	0/1	0/1	1/1	1/1	0/1	0/1
Chromosome2-3463195	DEL	PRECISE	6639	0	0	0/0	0/1	0/0	0/0	0/0	0/0	0/1	0/0	0/1	0/1	1/1	1/1	0/1	0/1
Chromosome2-3463540	BND	PRECISE	0	0	0	0/0	0/0	0/0	0/0	0/0	0/0	0/0	0/0	0/0	0/1	0/0	0/0	0/0	0/0
Chromosome2-3464195	INS	PRECISE	10434	0	0	0/0	0/0	0/0	1/1	0/0	0/0	0/0	0/0	0/0	0/0	0/0	0/0	0/0	0/0
Chromosome2-3464195	INS	PRECISE	1377	0	0.5	0/0	0/0	0/0	0/0	0/1	0/1	0/0	0/1	0/0	0/1	0/0	0/0	0/0	0/0
Chromosome2-3464415	INS	PRECISE	12538	0	0	0/0	0/0	0/0	0/1	0/0	0/0	0/0	0/0	0/0	0/0	0/0	0/0	0/0	0/0
Chromosome2-3464415	BND	PRECISE	0	0	0	0/0	0/0	0/0	0/1	0/0	0/0	0/0	0/0	0/0	0/0	0/0	0/0	0/0	0/0
Chromosome2-3464543	INS	PRECISE	78	0	0	0/0	0/0	0/0	0/1	0/0	0/0	0/0	0/0	0/0	0/0	0/0	0/0	0/0	0/0
Chromosome2-3464705	INS	PRECISE	7187	0	0	0/0	0/0	0/0	1/1	0/0	0/0	0/0	0/0	0/0	0/0	0/0	0/0	0/0	0/0
Chromosome2-3465805	DEL	PRECISE	280	0	0	0/0	0/0	0/1	0/1	0/0	0/0	0/0	0/0	0/0	0/0	0/0	0/0	0/0	0/0
Chromosome2-3466500	INS	PRECISE	49	0	0.535	0/0	1/1	0/0	0/0	0/0	0/0	1/1	0/0	1/1	0/1	0/0	0/0	0/1	0/1
Chromosome2-3466667	INS	PRECISE	60	0	0	0/0	0/0	0/0	0/0	0/1	0/1	0/0	0/1	0/0	1/1	0/0	0/0	0/0	0/0
Chromosome2-3467720	DEL	PRECISE	72	0	0	0/0	0/0	0/0	0/1	0/0	0/0	0/0	0/0	0/0	0/0	0/0	0/0	0/0	0/0
Chromosome2-3468081	INS	PRECISE	4681	0	0	0/0	0/0	0/0	0/1	0/0	0/0	0/0	0/0	0/0	0/0	0/0	0/0	0/0	0/0
Chromosome2-3468835	INS	PRECISE	6658	0	0	0/0	0/0	0/0	1/1	0/0	0/0	0/0	0/0	0/0	0/0	0/0	0/0	0/0	0/0
Chromosome2-3468988	DEL	PRECISE	153	0	0	0/0	0/1	0/0	0/1	0/0	0/0	0/1	0/0	0/0	0/1	1/1	0/1	0/0	0/1
Chromosome2-3469496	INS	PRECISE	362	0	0	0/0	0/0	0/0	0/1	0/0	0/0	0/0	0/0	0/0	0/0	0/0	0/0	0/0	0/0
Chromosome2-3469625	INS	PRECISE	3131	0	0	0/0	0/0	0/0	1/1	0/0	0/0	0/0	0/0	0/0	0/0	0/0	0/0	0/0	0/0
Chromosome2-3470795	DEL	PRECISE	1121	0	0	0/0	0/0	0/0	0/0	0/0	0/0	0/0	0/0	0/1	0/0	0/0	0/1	0/1	0/0
Chromosome2-3472165	INS	PRECISE	867	0	0.816	0/0	0/0	0/0	0/0	0/1	0/1	0/0	0/1	0/0	0/1	0/0	0/0	0/0	0/0
Chromosome2-3472885	INS	PRECISE	208	0	0.463	0/0	0/1	0/0	0/0	0/0	0/0	0/1	0/0	0/1	0/1	1/1	1/1	0/1	0/1
Chromosome2-3472935	DEL	PRECISE	951	0	0	0/0	0/0	0/1	0/0	0/0	0/0	0/0	0/0	0/0	0/0	0/0	0/0	0/0	0/0
Chromosome2-3474170	DEL	PRECISE	580	0	0	0/0	0/1	0/0	0/0	0/0	0/0	0/1	0/0	0/1	0/1	1/1	1/1	0/1	0/1
Chromosome2-3474242	INS	PRECISE	401	0	0	0/0	0/0	0/0	0/0	0/0	0/0	0/0	0/0	0/0	0/0	0/0	0/0	0/1	0/1
Chromosome2-3474330	BND	PRECISE	0	0	0	0/0	0/0	0/1	0/0	0/0	0/0	0/0	0/0	0/0	0/0	0/0	0/0	0/0	0/0
Chromosome2-3474381	DEL	PRECISE	66	0	0	0/0	0/0	0/0	0/0	0/0	0/0	0/0	0/0	0/0	0/1	0/1	0/1	0/0	0/0
Chromosome2-3474658	DEL	PRECISE	52	0	0	0/0	0/0	0/0	0/0	0/0	0/0	0/0	0/0	0/0	0/0	0/0	0/0	0/1	0/1
Chromosome2-3475266	DEL	PRECISE	6103	0	0	0/0	0/1	0/1	1/1	1/1	0/1	1/1	1/1	0/1	1/1	1/1	1/1	1/1	1/1
Chromosome2-3477697	DEL	PRECISE	57	0	0	0/0	0/1	0/1	1/1	1/1	0/1	1/1	1/1	0/1	1/1	1/1	1/1	1/1	1/1
Chromosome2-3479152	INS	PRECISE	160	0	0	0/0	0/0	0/0	0/1	0/0	0/0	0/0	0/0	0/0	0/0	0/0	0/0	0/0	0/0
Chromosome2-3479293	DEL	PRECISE	134	0	0	0/0	0/1	0/0	0/0	0/0	0/0	0/1	0/0	0/1	0/1	1/1	1/1	0/1	0/1
Chromosome2-3479525	INS	PRECISE	359	0	0	0/0	0/0	0/1	0/0	0/0	0/0	0/0	0/0	0/0	0/0	0/0	0/0	0/0	0/0
Chromosome2-3479738	INS	PRECISE	998	0	0	0/0	0/0	0/0	0/1	0/0	0/0	0/0	0/0	0/0	0/0	0/0	0/0	0/0	0/0
Chromosome2-3481020	INS	PRECISE	282	0	0	0/0	0/0	0/0	0/0	0/0	0/0	0/0	0/0	0/0	0/0	0/1	0/0	0/0	0/0
Chromosome2-3481636	DEL	PRECISE	703	0	0	0/0	0/0	0/0	0/0	0/0	0/0	0/0	0/0	0/0	0/0	1/1	1/1	0/0	0/0

Chromosome2-3481724 BND	PRECISE	0	0	0	0/0	0/0	0/0	0/0	0/0	0/0	0/0	0/0	0/0	0/0	0/0	0/1	0/0	0/1
Chromosome2-3481849 DEL	PRECISE	148	0	0	0/0	0/1	0/0	0/0	0/0	0/0	0/1	0/0	0/1	0/1	0/1	1/1	0/1	0/0
Chromosome2-3484050 INS	PRECISE	62	0	0	0/0	0/0	0/0	0/0	0/0	0/0	0/0	0/0	0/0	0/0	0/0	0/0	0/1	0/1
Chromosome2-3485162 DEL	PRECISE	211	0	0	0/0	0/0	0/1	0/0	0/0	0/0	0/0	0/0	0/0	0/0	0/0	0/0	0/0	0/0
Chromosome2-3485230 INS	PRECISE	351	0	0	0/0	0/0	0/1	0/0	0/0	0/0	0/0	0/0	0/0	0/0	0/0	0/0	0/0	0/0
Chromosome2-3485301 DEL	PRECISE	780	0	0	0/0	0/0	0/0	0/0	0/0	0/0	0/0	0/0	0/0	0/0	0/0	0/0	1/1	0/1
Chromosome2-3486396 DEL	PRECISE	50	0	0	0/0	0/0	0/0	0/0	0/0	0/0	0/0	0/0	0/0	0/0	0/1	0/0	0/0	0/0
Chromosome2-3487625 INS	PRECISE	393	0	0	0/0	0/1	0/0	0/0	0/0	0/0	0/1	0/0	0/0	0/0	0/1	0/0	0/0	0/1
Chromosome2-3487676 INS	PRECISE	995	0	0.707	0/0	0/0	0/0	0/0	0/0	0/1	0/0	0/0	0/1	0/0	0/0	0/0	0/0	0/0
Chromosome2-3487953 DEL	PRECISE	138	0	0	0/0	0/1	0/0	0/0	0/0	0/0	0/1	0/0	0/1	0/1	1/1	1/1	0/1	0/1
Chromosome2-3488321 DEL	PRECISE	51	0	0	0/0	0/0	0/0	0/0	0/0	0/0	0/0	0/0	0/0	0/0	0/0	0/0	0/1	0/1
Chromosome2-3488739 DEL	PRECISE	248	0	0	0/0	0/1	0/0	0/0	0/0	0/0	0/1	0/0	0/1	0/1	1/1	1/1	0/1	0/1
Chromosome2-3489062 DEL	PRECISE	132	0	0	0/0	0/1	0/0	0/0	0/0	0/0	0/1	0/0	0/1	0/1	1/1	1/1	0/1	0/1
Chromosome2-3489792 DEL	PRECISE	137	0	0	0/0	0/1	0/1	0/1	1/1	1/1	1/1	1/1	1/1	1/1	1/1	1/1	1/1	1/1
Chromosome2-3489902 INS	PRECISE	53	0	0	0/0	0/1	0/0	0/0	0/0	0/0	0/1	0/0	0/1	0/1	1/1	1/1	1/1	1/1
Chromosome2-3490821 DEL	PRECISE	806	0	0	0/0	0/0	0/0	0/1	0/0	0/0	0/0	0/0	0/0	0/0	0/0	0/0	0/0	0/0
Chromosome2-3491148 BND	PRECISE	0	0	0	0/0	0/0	0/1	0/0	0/1	0/1	0/1	0/1	0/0	0/1	0/0	0/0	0/0	0/0
Chromosome2-3491148 BND	PRECISE	0	0	0	0/0	0/0	0/0	0/1	0/0	0/0	0/0	0/0	0/0	0/1	0/0	0/0	0/0	0/0
Chromosome2-3491422 DEL	PRECISE	50	0	0	0/0	0/0	0/0	0/0	0/0	0/0	0/0	0/0	0/0	0/0	0/0	0/0	1/1	0/1
Chromosome2-3491944 DEL	PRECISE	670	0	0	0/0	0/0	0/1	0/0	1/1	0/1	0/0	0/1	0/0	0/1	0/0	0/0	0/0	0/0
Chromosome2-3492428 INS	PRECISE	439	0	0.577	0/0	0/0	0/0	0/0	0/0	0/0	0/0	0/0	0/1	0/1	0/0	1/1	0/1	0/0
Chromosome2-3492544 INS	PRECISE	318	0	0.447	0/0	0/0	0/0	0/1	1/1	0/1	0/0	0/1	0/0	0/1	0/0	0/0	0/0	0/0
Chromosome2-3493054 DEL	PRECISE	983	0	0	0/0	0/1	0/0	0/0	0/0	0/0	0/1	0/0	0/0	0/0	1/1	0/0	0/0	0/1
Chromosome2-3493317 INS	PRECISE	363	0	0.707	0/0	0/0	0/0	0/0	0/0	0/0	0/0	0/0	0/0	0/1	0/0	0/1	0/0	0/0
Chromosome2-3493687 INS	PRECISE	5938	0	0	0/0	0/0	0/0	0/0	0/0	0/0	0/0	0/0	0/0	0/0	0/0	0/0	1/1	0/0
Chromosome2-3493691 BND	PRECISE	0	0	0	0/0	0/0	0/0	0/0	0/0	0/0	0/0	0/0	0/0	0/0	0/0	0/0	0/0	0/1
Chromosome2-3494302 INS	PRECISE	86	0	0	0/0	0/0	0/1	0/0	0/0	0/0	0/0	0/0	0/0	0/0	0/0	0/0	0/0	0/0
Chromosome2-3494378 INS	PRECISE	3918	0	0	0/0	0/0	0/0	1/1	0/0	0/0	0/0	0/0	0/0	0/0	0/0	0/0	0/0	0/0
Chromosome2-3495185 DEL	PRECISE	52	0	0	0/0	0/1	0/0	0/0	0/0	0/0	0/1	0/0	0/1	0/1	1/1	1/1	0/1	0/1
Chromosome2-3495294 DEL	PRECISE	332	0	0	0/0	0/1	0/0	0/1	1/1	1/1	0/1	0/1	1/1	1/1	1/1	1/1	1/1	1/1
Chromosome2-3495428 DEL	PRECISE	463	0	0	0/0	0/0	0/1	0/1	0/0	0/0	0/0	0/0	0/0	0/0	0/0	0/0	0/0	0/0
Chromosome2-3495771 INS	PRECISE	260	0	0.5	0/0	1/1	0/0	0/0	0/0	0/0	0/1	0/0	0/0	0/0	0/0	0/0	0/0	0/1
Chromosome2-3496020 INS	PRECISE	49	0	0.707	0/0	0/0	0/0	0/0	0/0	1/1	0/0	0/0	0/1	0/0	0/0	0/0	0/0	0/0
Chromosome2-3496371 DEL	PRECISE	755	0	0	0/0	0/1	0/1	0/1	0/0	0/0	1/1	0/1	0/1	0/1	1/1	1/1	0/0	1/1
Chromosome2-3496389 INS	PRECISE	77	0	0	0/0	0/0	0/0	0/0	0/1	0/0	0/0	0/0	0/0	0/0	0/0	0/0	0/0	0/0
Chromosome2-3496921 INS	PRECISE	887	0	0.5	0/0	0/1	0/0	0/0	0/0	0/0	0/1	0/0	0/0	0/0	0/1	0/0	0/0	0/1
Chromosome2-3497542 BND	PRECISE	0	0	0	0/0	0/0	0/0	0/1	0/0	0/0	0/0	0/0	0/0	0/0	0/0	0/0	0/0	0/0
Chromosome2-3498138 BND	PRECISE	0	0	0	0/1	0/1	0/1	0/0	0/0	0/0	0/0	0/0	0/0	0/0	0/0	0/0	0/0	0/0
Chromosome2-3498164 INS	PRECISE	143	0	0	0/0	0/0	0/1	0/0	0/0	0/0	0/0	0/0	0/0	0/0	0/0	0/0	0/0	0/0
Chromosome2-3498299 BND	PRECISE	0	0	0	0/0	0/0	0/0	0/0	0/0	0/0	0/0	0/0	0/0	0/1	0/0	0/0	0/0	0/1
Chromosome2-3498426 BND	PRECISE	0	0	0	0/0	0/1	0/0	0/0	0/0	0/0	0/1	0/0	0/1	0/0	0/0	0/0	0/1	0/1
Chromosome2-3499508 DEL	PRECISE	878	0	0	0/0	0/0	0/1	1/1	0/1	0/0	0/0	0/0	0/0	0/0	0/0	0/0	1/1	1/1
Chromosome2-3499745 BND	PRECISE	0	0	0	0/0	0/1	0/0	0/0	0/0	0/0	0/1	0/0	0/0	0/0	0/0	0/0	0/0	0/0
Chromosome2-3499960 DEL	PRECISE	822	0	0	0/0	0/1	0/0	0/0	0/0	0/0	0/1	0/0	0/1	0/1	1/1	1/1	0/1	0/1
Chromosome2-3500094 DEL	PRECISE	446	0	0	0/0	0/1	0/0	0/0	0/0	0/0	0/1	0/0	0/1	0/1	1/1	1/1	0/1	0/1
Chromosome2-3500610 DEL	PRECISE	165	0	0	0/0	0/1	0/0	0/0	0/0	0/0	0/1	0/0	0/0	0/0	0/1	0/0	0/0	0/1
Chromosome2-3500832 DEL	PRECISE	327	0	0	0/0	0/1	0/0	0/0	0/0	0/0	0/1	0/0	0/1	0/1	1/1	1/1	0/0	0/1
Chromosome2-3502237 INS	PRECISE	142	0	0	0/0	0/0	0/0	0/0	0/1	0/0	0/0	0/0	0/0	0/0	0/0	0/0	0/0	0/0
Chromosome2-3502406 DEL	PRECISE	211	0.976	0	0/0	0/1	0/0	0/0	0/0	0/0	0/1	0/0	0/0	0/1	1/1	1/1	1/1	1/1
Chromosome2-3502686 DEL	PRECISE	160	0	0	0/0	0/1	0/1	1/1	1/1	1/1	1/1	1/1	1/1	1/1	1/1	1/1	1/1	1/1
Chromosome2-3502820 INS	PRECISE	150	0	0.707	0/0	0/0	0/0	0/0	0/0	0/0	0/0	0/0	0/0	0/0	0/0	0/0	0/0	1/1
Chromosome2-3502863 INS	PRECISE	795	0	0.707	0/0	0/0	0/0	0/0	0/0	0/0	0/0	0/0	0/0	0/0	0/0	0/0	0/0	1/1
Chromosome2-3502983 INS	PRECISE	362	0	0	0/0	0/0	0/0	0/1	0/0	0/0	0/0	0/0	0/0	0/0	0/0	0/0	0/0	0/0
Chromosome2-3503106 DEL	PRECISE	57	0	0	0/0	0/1	0/0	0/0	0/0	0/0	0/0	0/0	0/0	0/1	1/1	1/1	0/0	0/1
Chromosome2-3503245 INS	PRECISE	993	0	0	0/0	0/0	0/0	0/1	0/0	0/0	0/0	0/0	0/0	0/0	0/0	0/0	0/0	0/0
Chromosome2-3503970 DEL	PRECISE	140	0	0	0/0	0/1	0/0	0/0	0/0	0/0	0/0	0/0	0/1	0/0	1/1	1/1	0/1	0/1
Chromosome2-3504097 INS	PRECISE	3248	0	0.707	0/0	0/0	0/0	0/0	0/0	0/0	0/0	0/0	0/0	0/0	0/0	0/1	0/0	0/0
Chromosome2-3505591 INS	PRECISE	59	0	0	0/0	0/0	0/0	0/0	0/0	0/0	0/0	0/0	0/0	0/0	0/0	0/0	0/1	0/1
Chromosome2-3505617 DEL	PRECISE	350	0	0	0/0	0/0	0/0	0/0	0/0	0/0	0/0	0/0	0/0	0/1	0/0	0/1	0/1	0/1
Chromosome2-3505973 DEL	PRECISE	617	0	0	0/0	0/0	0/0	0/0	0/0	0/0	0/0	0/0	0/0	0/1	0/0	0/1	0/0	0/0
Chromosome2-3506190 DEL	PRECISE	140	0	0	0/0	0/0	0/0	0/0	0/1	0/1	0/0	0/1	0/0	0/1	0/0	0/0	0/0	0/0
Chromosome2-3506309 INS	PRECISE	132	0	0	0/0	0/1	0/0	0/0	0/0	0/0	0/1	0/0	0/0	0/0	1/1	0/1	0/1	0/1
Chromosome2-3506372 DEL	PRECISE	250	0	0	0/0	0/0	0/0	0/0	0/0	0/0	0/0	0/0	0/0	0/1	0/0	0/1	0/0	0/0
Chromosome2-3507187 INS	PRECISE	5393	0	0.707	0/0	0/0	0/0	0/0	0/0	1/1	0/0	0/0	1/1	0/0	0/0	0/0	0/0	0/0
Chromosome2-3507701 INS	PRECISE	1553	0	0.707	0/0	0/0	0/0	0/0	0/0	0/0	1/1	0/1	0/0	0/0	0/0	0/0	0/0	0/0
Chromosome2-3508477 DEL	PRECISE	1486	0	0	0/0	0/0	0/0	0/0	0/0	0/0	0/0	0/0	0/0	0/0	0/0	0/0	0/0	0/1
Chromosome2-3509717 INS	PRECISE	710	0	0.707	0/0	0/0	0/0	0/0	0/0	0/1	0/0	0/0	0/1	0/0	0/0	0/0	0/0	0/0
Chromosome2-3509996 INS	PRECISE	747	0	0	0/0	0/0	0/0	0/0	0/0	0/0	0/0	0/0	0/0	0/0	0/1	0/0	0/0	0/0
Chromosome2-3510248 INS	PRECISE	132	0	0	0/0	0/0	0/0	0/1	0/1	0/0	0/1	0/1	0/0	0/0	0/0	0/0	0/0	0/0
Chromosome2-3510908 DEL	PRECISE	541	0	0	0/0	0/0	0/0	0/1	0/0	0/0	1/1	0/1	0/0	0/0	0/0	0/0	0/0	0/0
Chromosome2-3511276 INS	PRECISE	225	0	0	0/0	0/0	0/0	0/0	0/0	0/0	0/0	0/0	0/0	0/1	0/0	1/1	0/0	0/0
Chromosome2-3511727 BND	PRECISE	0	0.577	0	0/0	0/0	0/0	0/0	0/0	0/0	0/0	0/0	0/1	0/0	0/0	0/1	0/0	0/0
Chromosome2-3511856 BND	PRECISE	0	0	0	0/0	0/1	0/0	0/0	0/0	0/0	0/0	0/0	0/0	0/0	1/1	0/0	0/0	0/1

Chromosome2-3512497	BND	PRECISE	0	0	0	0/0	0/0	0/0	0/0	0/0	0/0	0/0	0/0	0/1	0/0	0/1	0/0	0/0	0/0
Chromosome2-3512825	INS	PRECISE	716	0	0.707	0/0	0/0	0/0	0/0	0/0	0/0	0/0	0/0	0/0	0/1	0/0	0/1	0/0	0/0
Chromosome2-3513029	INS	PRECISE	276	0	0.707	0/0	0/0	0/1	0/1	0/0	0/0	0/0	0/0	0/0	0/0	0/0	0/0	0/0	0/0
Chromosome2-3513101	DEL	PRECISE	97	0	0	0/0	0/0	0/0	0/0	0/0	0/0	0/0	0/0	0/1	0/1	0/1	1/1	0/1	0/0
Chromosome2-3513323	INS	PRECISE	240	0	0	0/0	0/0	0/1	0/0	0/0	0/0	0/0	0/0	0/0	0/0	0/0	0/0	0/0	0/0
Chromosome2-3513901	DEL	PRECISE	3599	0.5	0.5	0/0	0/0	0/0	0/0	0/0	0/0	0/0	0/0	0/1	0/1	0/0	1/1	0/1	0/0
Chromosome2-3514044	DEL	PRECISE	1853	0	0	0/0	0/1	0/0	0/0	0/0	0/0	0/1	0/0	0/0	0/0	1/1	0/0	1/1	1/1
Chromosome2-3514767	INS	PRECISE	1453	0	0	0/0	0/0	0/0	0/1	0/0	0/0	0/0	0/0	0/0	0/0	0/0	0/0	0/0	0/0
Chromosome2-3514935	INS	PRECISE	3117	0	0	0/0	0/0	0/0	0/0	0/0	0/0	0/0	0/0	0/0	0/0	0/1	0/0	0/0	0/0
Chromosome2-3515087	INS	PRECISE	687	0	0.577	0/0	0/0	0/1	1/1	0/0	0/0	0/0	0/0	0/0	0/0	0/0	0/0	0/1	0/1
Chromosome2-3515474	DEL	PRECISE	73	0	0	0/0	0/1	0/0	0/0	0/0	0/0	0/1	0/0	0/0	0/1	0/1	0/1	0/0	0/1
Chromosome2-3516178	INS	PRECISE	7649	0	0	0/0	0/0	0/0	0/0	0/0	0/0	0/0	0/0	0/0	0/0	0/0	0/0	0/0	1/1
Chromosome2-3516340	INS	PRECISE	66	0	0.707	0/0	0/0	0/0	0/0	0/0	0/0	0/0	0/0	0/0	0/0	0/0	0/0	0/1	0/1
Chromosome2-3516538	DEL	PRECISE	216	0	0	0/0	0/0	0/0	0/0	0/1	0/0	0/0	0/0	0/0	0/0	0/0	0/0	0/0	0/0
Chromosome2-3517344	INS	PRECISE	77	0	0.354	0/0	0/1	0/0	0/0	0/0	0/0	0/1	0/0	0/1	0/1	1/1	1/1	0/1	0/1
Chromosome2-3517745	DEL	PRECISE	582	0.354	0	0/0	0/0	0/0	0/0	0/0	0/0	0/1	0/0	0/1	0/1	1/1	1/1	0/0	0/1
Chromosome2-3518211	DEL	PRECISE	90	0	0	0/0	0/1	0/0	0/0	0/0	0/0	0/1	0/0	0/0	0/0	1/1	0/0	0/0	0/1
Chromosome2-3518330	DEL	PRECISE	167	0	0	0/0	0/1	0/0	0/0	0/0	0/0	0/1	0/0	0/0	0/0	0/1	0/0	0/0	0/1
Chromosome2-3518414	INS	PRECISE	50	0	0.577	0/0	0/0	0/0	0/0	0/0	0/0	0/0	0/0	0/1	0/0	0/0	0/1	0/0	0/0
Chromosome2-3519602	INS	PRECISE	626	0	0	0/0	0/0	0/0	0/1	0/0	0/0	0/0	0/0	0/0	0/0	0/0	0/0	0/0	0/0
Chromosome2-3520330	DEL	PRECISE	143	0	0	0/0	0/1	0/0	0/0	0/0	0/0	0/1	0/0	0/1	0/1	1/1	1/1	0/1	0/1
Chromosome2-3520511	DEL	PRECISE	194	0	0	0/0	0/0	0/0	0/0	0/0	0/0	0/0	0/0	0/0	0/0	0/0	0/0	0/1	0/1
Chromosome2-3520669	DEL	PRECISE	2319	0	0	0/0	0/0	0/0	0/1	0/0	0/0	0/0	0/0	0/0	0/0	0/0	0/0	0/0	0/0
Chromosome2-3520816	DEL	PRECISE	1086	0	0	0/0	0/1	0/0	0/0	0/0	0/0	0/1	0/0	0/0	0/1	1/1	0/1	0/0	0/1
Chromosome2-3521312	DEL	PRECISE	960	0	0	0/0	0/0	0/0	0/1	0/0	0/0	0/0	0/0	0/0	0/0	0/0	0/0	0/0	0/0
Chromosome2-3521379	DEL	PRECISE	128	0	0	0/0	0/0	0/0	0/0	0/0	0/0	0/0	0/0	0/1	0/0	0/0	0/1	0/1	0/0
Chromosome2-3521499	INS	PRECISE	216	0	0	0/0	0/0	0/0	0/0	0/0	0/1	0/0	0/0	0/1	0/0	0/0	0/0	0/0	0/0
Chromosome2-3521607	DEL	PRECISE	149	0	0	0/0	0/1	0/0	0/0	0/0	0/0	0/1	0/0	0/1	0/1	1/1	1/1	0/1	0/1
Chromosome2-3523329	DEL	PRECISE	425	0	0	0/0	0/0	0/0	0/0	0/1	0/0	0/0	0/0	0/0	0/0	0/0	0/0	0/0	0/0
Chromosome2-3523415	INS	PRECISE	993	0	0	0/0	0/0	0/0	0/0	0/0	0/0	0/0	0/0	0/0	0/1	0/0	0/1	0/0	0/0
Chromosome2-3523799	DEL	PRECISE	351	0	0	0/0	0/1	0/0	0/0	0/0	0/0	0/1	0/0	0/0	0/0	0/1	0/0	0/0	0/1
Chromosome2-3524759	INS	PRECISE	163	0	0	0/0	0/0	0/0	0/1	0/0	0/0	0/0	0/0	0/0	0/0	0/0	0/0	0/0	0/0
Chromosome2-3524769	DEL	PRECISE	82	0	0	0/0	0/0	0/0	0/0	0/1	0/0	0/0	0/0	0/0	0/0	0/0	0/0	0/0	0/0
Chromosome2-3525808	INS	PRECISE	885	0	0.957	0/0	0/0	0/0	0/0	0/1	0/1	0/0	0/1	0/0	0/1	0/0	0/0	0/0	0/0
Chromosome2-3525892	DEL	PRECISE	99	0	0	0/0	0/0	0/0	0/0	0/1	0/0	0/0	0/0	0/0	0/0	0/0	0/0	0/0	0/0
Chromosome2-3526255	INS	PRECISE	506	0	0.518	0/0	0/1	0/0	0/0	0/0	0/0	0/1	0/0	0/1	0/1	1/1	1/1	0/1	1/1
Chromosome2-3526574	DEL	PRECISE	210	0	0	0/0	0/1	0/0	0/0	0/0	0/0	0/1	0/0	0/1	0/1	1/1	1/1	0/1	1/1
Chromosome2-3526595	INS	PRECISE	625	0	0	0/0	0/0	0/1	0/0	0/0	0/0	0/0	0/0	0/0	0/0	0/0	0/0	0/0	0/0
Chromosome2-3527030	INS	PRECISE	102	0	0	0/0	0/1	0/0	0/0	0/0	0/0	0/1	0/0	0/0	0/0	0/1	0/0	0/0	1/1
Chromosome2-3527473	INS	PRECISE	2800	0	0	0/0	0/0	0/0	1/1	0/0	0/0	0/0	0/0	0/0	0/0	0/0	0/0	0/0	0/0
Chromosome2-3528202	INS	PRECISE	137	0	0	0/0	0/0	0/0	0/0	0/0	0/0	0/0	0/0	0/1	0/0	0/0	0/1	0/1	0/0
Chromosome2-3528529	DEL	PRECISE	602	0	0	0/0	0/0	0/0	0/1	0/0	0/0	0/0	0/0	0/0	0/0	0/0	0/0	0/0	0/0
Chromosome2-3528702	DEL	PRECISE	104	0	0	0/0	0/0	0/0	0/0	0/0	0/0	0/0	0/0	0/1	0/0	0/0	0/1	0/1	0/0
Chromosome2-3528736	INS	PRECISE	277	0	0.707	0/0	0/0	0/0	0/0	0/0	0/0	0/0	0/0	0/0	0/0	0/0	0/0	0/1	0/1
Chromosome2-3528798	DEL	PRECISE	162	0	0	0/0	0/1	0/1	1/1	1/1	1/1	1/1	1/1	1/1	1/1	1/1	1/1	1/1	1/1
Chromosome2-3528979	DEL	PRECISE	782	0	0	0/0	0/1	0/0	0/0	0/0	0/0	0/1	0/0	0/0	0/0	1/1	0/0	0/0	0/0
Chromosome2-3529526	DEL	PRECISE	211	0.548	0	0/0	0/0	0/0	0/0	0/0	0/0	0/0	0/0	0/1	0/0	0/1	1/1	0/1	0/0
Chromosome2-3529878	DEL	PRECISE	74	0	0	0/0	0/0	0/0	0/0	0/0	0/0	0/0	0/0	0/0	0/1	0/1	0/1	0/0	0/0
Chromosome2-3530187	INS	PRECISE	273	0	0	0/0	0/0	0/0	0/1	0/0	0/0	0/0	0/0	0/0	0/0	0/0	0/0	0/0	0/0
Chromosome2-3530494	INS	PRECISE	368	0	0	0/0	0/0	0/0	0/1	0/0	0/0	0/0	0/0	0/0	0/0	0/0	0/0	0/0	0/0
Chromosome2-3530796	INS	PRECISE	206	0	0.641	0/0	0/1	0/0	0/0	0/0	0/0	0/1	0/0	0/1	0/1	1/1	1/1	1/1	1/1
Chromosome2-3531381	DEL	PRECISE	50	0	0	0/0	0/0	0/0	0/0	0/0	0/0	0/1	0/0	0/1	0/0	1/1	1/1	0/1	0/1
Chromosome2-3531684	DEL	PRECISE	71	0	0	0/0	0/0	0/0	0/0	0/0	0/0	0/0	0/0	0/0	0/0	0/0	0/0	0/1	0/1
Chromosome2-3532167	INS	PRECISE	238	0	0	0/0	0/0	0/0	0/0	0/0	0/0	0/0	0/0	0/0	0/0	0/0	0/0	0/1	0/1
Chromosome2-3533002	INS	PRECISE	69	0	0.535	0/0	0/1	0/0	0/0	0/0	0/0	0/0	0/0	0/1	0/1	1/1	1/1	0/1	0/1
Chromosome2-3533013	DEL	PRECISE	1238	0.378	0.378	0/0	0/1	0/0	0/0	0/0	0/0	0/0	0/0	0/1	0/1	1/1	1/1	0/1	0/1
Chromosome2-3533645	DEL	PRECISE	5439	0	0	0/0	0/1	0/0	0/0	0/0	0/0	0/1	0/0	0/0	0/0	1/1	0/0	0/0	0/1
Chromosome2-3534650	INS	PRECISE	455	0	0	0/0	0/0	0/0	0/1	0/0	0/0	0/0	0/0	0/0	0/0	0/0	0/0	0/0	0/0
Chromosome2-3534666	DEL	PRECISE	55	0	0	0/0	0/0	0/0	0/1	0/0	0/0	0/0	0/0	0/0	0/0	0/0	0/0	0/0	0/0
Chromosome2-3535791	DEL	PRECISE	5995	0	0	0/0	0/1	0/0	0/1	0/0	0/1	1/1	0/1	1/1	0/1	1/1	1/1	1/1	1/1
Chromosome2-3536849	INS	PRECISE	2646	0	0.707	0/0	0/0	0/0	0/0	0/0	0/0	0/0	0/0	0/0	0/0	0/0	0/0	1/1	1/1
Chromosome2-3537331	DEL	PRECISE	3469	0	0	0/0	0/0	0/0	0/1	0/0	0/0	0/0	0/0	0/0	0/0	0/0	0/0	0/0	0/0
Chromosome2-3537954	INS	PRECISE	119	0	0	0/0	0/0	0/0	0/0	0/0	0/0	0/0	0/0	0/0	0/0	0/0	0/0	1/1	1/1
Chromosome2-3537975	DEL	PRECISE	805	0	0	0/0	0/0	0/1	0/1	0/1	0/1	0/0	0/1	0/0	1/1	0/0	0/0	1/1	1/1
Chromosome2-3538696	INS	PRECISE	18565	0	0	0/0	0/0	0/0	0/0	0/0	0/0	0/0	0/0	0/1	0/0	0/0	0/0	0/0	0/0
Chromosome2-3538697	BND	PRECISE	0	0	0	0/0	0/0	0/0	0/1	0/0	0/1	0/0	0/0	0/1	0/0	0/0	0/0	0/0	0/0
Chromosome2-3538834	INS	PRECISE	53	0	0	0/0	0/1	0/0	0/0	0/0	0/0	0/1	0/0	0/0	0/0	1/1	0/1	0/0	0/1
Chromosome2-3538839	INS	PRECISE	8102	0	0	0/0	0/0	0/0	0/1	0/0	0/0	0/0	0/0	0/0	0/0	0/0	0/0	0/0	0/0
Chromosome2-3539051	INS	PRECISE	7303	0	0	0/0	0/0	0/0	1/1	0/0	0/0	0/0	0/0	0/0	0/0	0/0	0/0	0/0	0/0
Chromosome2-3539078	INS	PRECISE	625	0	0.707	0/0	0/0	0/1	0/1	0/0	0/0	0/0	0/0	0/0	0/0	0/0	0/0	0/0	0/0
Chromosome2-3539521	INS	PRECISE	188	0	0.707	0/0	0/0	0/0	0/0	0/0	0/0	0/0	0/0	0/0	0/0	0/0	0/0	1/1	0/1
Chromosome2-3539674	DEL	PRECISE	234	0	0	0/0	0/0	0/0	0/0	0/1	0/0	0/0	0/0	0/0	0/0	0/0	0/0	0/0	0/0
Chromosome2-3541266	INS	PRECISE	1375	0	0	0/0	0/0	0/0	0/1	0/0	0/0	0/0	0/0	0/0	0/0	0/0	0/0	0/0	0/0

Chromosome2-3541466	DEL	PRECISE	10725	0	0	0/0	0/0	0/1	1/1	1/1	1/1	1/1	1/1	1/1	1/1	1/1	1/1	1/1	1/1
Chromosome2-3542682	DEL	PRECISE	1383	0	0	0/0	0/0	0/0	0/0	0/0	0/0	0/0	0/0	0/0	0/0	0/0	0/0	0/1	0/1
Chromosome2-3544043	DEL	PRECISE	274	0	0	0/0	0/0	0/0	0/0	0/1	0/1	0/0	0/1	0/0	1/1	0/0	0/0	0/0	0/0
Chromosome2-3545757	INS	PRECISE	50	0	0	0/0	0/0	0/1	0/0	0/0	0/0	0/0	0/0	0/0	0/0	0/0	0/0	0/0	0/0
Chromosome2-3546039	DEL	PRECISE	51	0	0	0/0	0/1	0/0	0/0	0/0	0/0	0/1	0/0	0/1	0/1	1/1	1/1	0/1	0/1
Chromosome2-3548933	INS	PRECISE	74	0	0.5	0/0	0/1	0/1	0/0	0/0	0/0	0/1	0/0	0/1	0/1	1/1	1/1	1/1	1/1
Chromosome2-3549090	INS	PRECISE	67	0	0.5	0/0	0/0	0/0	0/0	0/0	0/1	0/1	0/1	0/1	0/0	0/0	0/0	0/0	0/0
Chromosome2-3549144	INS	PRECISE	548	0	0	0/0	0/0	0/0	0/1	0/0	0/0	0/0	0/0	0/0	0/0	0/0	0/0	0/0	0/0
Chromosome2-3549489	DEL	PRECISE	73	0	0	0/0	0/0	0/0	0/0	0/0	0/0	0/0	0/0	0/0	0/0	0/0	0/0	0/1	0/1
Chromosome2-3549728	INS	PRECISE	479	0.843	0.699	0/0	0/1	0/1	0/1	0/0	0/0	0/1	0/0	0/1	0/1	1/1	1/1	0/1	0/1
Chromosome2-3550226	INS	PRECISE	1141	0	0	0/0	0/0	0/0	0/1	0/0	0/0	0/0	0/0	0/0	0/0	0/0	0/0	0/0	0/0
Chromosome2-3551083	INS	PRECISE	721	0	0.707	0/0	0/0	0/0	0/0	0/0	0/0	0/0	0/0	0/0	0/0	0/0	0/0	0/1	0/1
Chromosome2-3551254	DUP	PRECISE	436	0	0	0/0	0/1	0/0	0/0	0/0	0/0	0/1	0/0	0/0	0/0	0/1	0/0	0/0	0/1
Chromosome2-3551793	DEL	PRECISE	76	0	0	0/0	0/1	0/0	0/0	0/0	0/0	0/1	0/0	0/0	0/1	1/1	0/1	0/0	0/1
Chromosome2-3552085	INS	PRECISE	383	0	0	0/0	0/0	0/0	0/0	0/0	0/0	0/0	0/0	0/0	0/0	0/0	0/0	0/1	0/1
Chromosome2-3552802	DEL	PRECISE	7235	0	0	0/0	0/1	0/1	1/1	1/1	1/1	1/1	1/1	1/1	1/1	1/1	1/1	1/1	1/1
Chromosome2-3555449	DEL	PRECISE	55	0	0	0/0	0/0	0/0	0/1	0/0	0/0	0/0	0/0	0/0	0/0	0/0	0/0	0/0	0/0
Chromosome2-3555948	DEL	PRECISE	93	0	0	0/0	0/1	0/0	0/0	0/0	0/0	0/1	0/0	0/1	0/1	1/1	1/1	0/1	0/1
Chromosome2-3556557	INS	PRECISE	51	0	0.463	0/0	1/1	0/0	0/0	0/0	0/0	0/1	0/0	1/1	0/1	1/1	1/1	0/1	0/1
Chromosome2-3557213	DEL	PRECISE	196	0.333	0.333	0/0	0/1	0/1	0/0	0/0	0/0	0/1	0/0	0/1	0/1	1/1	1/1	0/1	0/1
Chromosome2-3557817	INS	PRECISE	92	0	0	0/0	0/0	0/0	0/0	0/1	0/1	1/1	0/1	1/1	0/0	0/0	0/0	0/0	0/0
Chromosome2-3558707	INS	PRECISE	77	0	0	0/0	0/1	0/0	0/0	0/0	0/0	0/1	0/0	0/1	0/1	1/1	1/1	0/1	0/1
Chromosome2-3559624	DEL	PRECISE	56	0	0	0/0	0/1	0/0	0/0	0/0	0/0	0/1	0/0	0/0	0/0	1/1	0/0	0/0	0/1
Chromosome2-3559903	DUP	PRECISE	18284	0	0	0/0	0/0	0/0	0/0	0/0	0/0	0/0	0/0	0/0	0/0	0/0	0/0	0/1	0/1
Chromosome2-3559945	BND	PRECISE	0	0	0	0/0	0/1	0/0	0/0	0/0	0/0	0/0	0/0	0/0	0/0	0/1	0/0	0/0	0/0
Chromosome2-3560237	INS	PRECISE	306	0	0.518	0/0	0/1	0/0	0/0	0/0	0/0	0/1	0/0	0/1	0/1	1/1	1/1	0/1	0/1
Chromosome2-3560494	INS	PRECISE	189	0	0.632	0/0	0/0	0/1	0/1	1/1	1/1	0/1	1/1	0/1	0/1	0/0	0/0	0/1	0/1
Chromosome2-3562631	INS	PRECISE	348	0	0.5	0/0	0/0	0/0	0/0	0/0	0/0	0/1	0/0	0/0	0/0	0/1	0/0	0/0	0/1
Chromosome2-3563362	DEL	PRECISE	1015	0	0	0/0	0/0	0/1	1/1	1/1	1/1	1/1	1/1	1/1	1/1	1/1	1/1	1/1	1/1
Chromosome2-3564073	DEL	PRECISE	57	0	0	0/0	0/0	0/1	0/0	0/0	0/0	0/0	0/0	0/0	0/0	0/0	0/0	0/0	0/0
Chromosome2-3564344	DEL	PRECISE	92	0.354	0.354	0/0	0/1	0/0	0/0	0/0	0/0	0/1	0/0	0/1	0/1	1/1	1/1	0/1	0/1
Chromosome2-3564505	DEL	PRECISE	104	0	0	0/0	0/1	0/0	0/0	0/0	0/0	0/1	0/0	0/1	0/1	1/1	1/1	0/1	0/0
Chromosome2-3564777	DEL	PRECISE	213	0	0	0/0	0/0	0/0	0/0	0/0	0/0	0/0	0/0	0/1	0/1	0/0	1/1	0/1	0/0
Chromosome2-3565823	DEL	PRECISE	288	0	0	0/0	0/1	0/0	0/0	0/0	0/0	0/1	0/0	0/1	0/1	1/1	1/1	0/1	0/1
Chromosome2-3565923	INS	PRECISE	284	0	0	0/0	0/0	0/0	0/1	0/0	0/0	0/0	0/0	0/0	0/0	0/0	0/0	0/0	0/0
Chromosome2-3565942	DEL	PRECISE	1994	0	0	0/0	0/1	0/0	0/0	0/0	0/0	0/1	0/0	0/1	0/1	1/1	1/1	0/1	0/1
Chromosome2-3566171	INS	PRECISE	50	0	0.354	0/0	0/1	0/0	0/0	0/0	0/0	0/1	0/0	0/1	0/1	1/1	1/1	0/1	0/1
Chromosome2-3566183	DEL	PRECISE	503	0	0	0/0	0/1	0/0	0/0	0/0	0/0	0/1	0/0	0/1	0/1	1/1	1/1	0/1	0/1
Chromosome2-3566316	BND	PRECISE	0	0	0	0/0	0/1	0/0	0/0	0/0	0/0	0/0	0/0	0/1	0/0	0/1	0/0	0/0	0/1
Chromosome2-3567631	DEL	PRECISE	622	0	0	0/0	0/0	0/0	0/0	0/0	0/0	0/0	0/0	0/1	0/0	0/0	1/1	0/0	0/0
Chromosome2-3567990	DEL	PRECISE	54	0	0	0/0	0/1	0/0	0/0	0/0	0/0	0/0	0/0	0/1	0/0	0/1	1/1	0/0	0/1
Chromosome2-3568418	INS	PRECISE	57	0	0.787	0/0	0/1	0/0	0/0	0/0	0/0	0/0	0/0	0/1	0/0	1/1	1/1	0/0	0/1
Chromosome2-3568738	INS	PRECISE	132	0	0	0/0	0/0	0/1	0/0	0/1	0/0	0/0	0/0	0/0	0/0	0/0	0/0	0/0	0/0
Chromosome2-3569003	INS	PRECISE	145	0	0	0/0	0/0	0/1	0/0	0/0	0/0	0/0	0/0	0/0	0/0	0/0	0/0	0/0	0/0
Chromosome2-3569433	INS	PRECISE	724	0	0	0/0	0/0	0/0	0/0	0/0	0/0	0/1	0/1	0/0	0/0	0/0	0/0	0/0	0/0
Chromosome2-3570257	DEL	PRECISE	158	0	0	0/0	0/1	0/0	1/1	1/1	1/1	1/1	1/1	1/1	1/1	1/1	1/1	1/1	1/1
Chromosome2-3570294	INS	PRECISE	59	0	0	0/0	0/1	0/0	0/0	0/0	0/0	0/1	0/0	0/1	0/1	1/1	1/1	0/1	0/1
Chromosome2-3570828	DEL	PRECISE	132	0	0	0/0	0/1	0/0	1/1	1/1	1/1	1/1	1/1	1/1	1/1	1/1	1/1	1/1	1/1
Chromosome2-3570962	DEL	PRECISE	345	0	0	0/0	0/0	0/0	0/0	0/0	0/0	0/0	0/0	0/1	0/1	0/1	1/1	0/1	0/0
Chromosome2-3571260	INS	PRECISE	11438	0	0	0/0	0/0	0/0	0/0	0/0	0/0	0/0	0/0	0/0	0/0	0/0	0/0	0/0	0/0
Chromosome2-3571277	BND	PRECISE	0	0	0	0/0	0/0	0/0	0/0	0/1	0/0	0/0	0/1	0/0	0/1	0/0	0/0	0/0	0/0
Chromosome2-3571414	INS	PRECISE	276	0	0.548	0/0	0/0	0/0	1/1	0/0	1/1	0/1	0/1	0/1	0/0	0/0	0/0	0/0	0/0
Chromosome2-3571977	DEL	PRECISE	66	0	0	0/0	0/0	0/0	0/1	0/0	0/0	0/0	0/0	0/0	0/0	0/0	0/0	0/0	0/0
Chromosome2-3572015	BND	PRECISE	0	0	0	0/0	0/0	0/0	0/0	0/0	0/0	0/0	0/0	0/0	0/0	0/0	0/1	0/1	0/0
Chromosome2-3572016	BND	PRECISE	0	0	0	0/0	0/0	0/0	0/0	0/0	0/0	0/0	0/0	0/0	0/0	0/0	0/0	0/1	0/0
Chromosome2-3572250	DEL	PRECISE	102	0	0	0/0	0/1	0/0	0/0	0/0	0/0	0/1	0/0	0/0	0/0	0/1	0/0	0/0	0/1
Chromosome2-3573480	DEL	PRECISE	70	0	0	0/0	0/1	0/0	0/0	0/0	0/0	0/1	0/0	0/1	0/1	1/1	1/1	0/1	0/1
Chromosome2-3573522	INS	PRECISE	8297	0	0	0/0	0/0	0/0	1/1	0/0	0/0	0/0	0/0	0/0	0/0	0/0	0/0	0/0	0/0
Chromosome2-3573848	DEL	PRECISE	56	0	0	0/0	0/1	0/0	0/0	0/0	0/0	0/1	0/0	0/0	0/0	0/1	0/0	0/0	0/1
Chromosome2-3575374	INS	PRECISE	476	0	0.707	0/0	0/0	0/0	0/0	0/0	0/0	0/1	1/1	0/0	0/0	0/0	0/0	0/0	0/0
Chromosome2-3577031	DEL	PRECISE	96	0	0	0/0	0/0	0/0	0/1	0/0	0/0	0/0	0/0	0/0	0/0	0/0	0/0	0/0	0/0
Chromosome2-3577900	INS	PRECISE	79	0	0.354	0/0	0/1	0/0	0/0	0/0	0/0	0/1	0/0	0/1	0/1	1/1	1/1	0/1	0/1
Chromosome2-3577971	DEL	PRECISE	254	0	0	0/0	0/0	0/0	0/1	0/0	0/0	0/0	0/0	0/0	0/0	0/0	0/0	0/0	0/0
Chromosome2-3578103	DEL	PRECISE	56	0	0	0/0	0/0	0/0	0/0	0/0	0/0	0/0	0/0	0/0	0/0	0/0	0/0	0/1	0/1
Chromosome2-3578347	INS	PRECISE	67	0	0.354	0/0	0/1	0/0	0/0	0/0	0/0	0/1	0/0	0/1	0/1	1/1	1/1	0/1	0/1
Chromosome2-3578493	DEL	PRECISE	53	0	0	0/0	0/0	0/0	0/0	0/0	0/0	0/0	0/0	0/0	0/0	0/0	0/0	0/1	0/1
Chromosome2-3578879	DEL	PRECISE	2703	0	0	0/0	0/1	0/0	0/0	0/0	0/0	0/1	0/0	0/1	0/1	1/1	1/1	0/1	0/1
Chromosome2-3579246	DEL	PRECISE	71	0	0	0/0	0/1	0/0	0/0	0/0	0/0	0/1	0/0	0/1	1/1	1/1	1/1	0/1	0/1
Chromosome2-3580268	DEL	PRECISE	1073	0	0	0/0	0/0	0/0	1/1	0/0	0/0	0/0	0/0	0/0	0/0	0/0	0/0	0/0	0/0
Chromosome2-3580395	INS	PRECISE	240	0	0	0/0	0/0	0/0	0/1	0/0	0/0	0/0	0/0	0/0	0/0	0/0	0/0	0/0	0/0
Chromosome2-3580664	DEL	PRECISE	50	0	0	0/0	0/0	0/0	0/0	0/0	0/0	0/0	0/0	0/0	0/0	0/0	0/0	0/1	0/1
Chromosome2-3581010	DEL	PRECISE	1308	0	0	0/0	0/0	0/1	0/0	0/0	0/0	0/0	0/0	0/0	0/0	0/0	0/0	0/0	0/0
Chromosome2-3581278	DEL	PRECISE	959	0	0	0/0	0/1	0/0	0/0	0/0	0/0	0/0	0/0	0/0	0/0	0/1	0/0	0/1	1/1

Chromosome2-3582559 INS	PRECISE	85	0	0	0/0	0/1	0/0	0/0	0/0	0/0	0/1	0/0	0/1	0/1	1/1	1/1	0/1	0/1
Chromosome2-3582641 BND	PRECISE	0	0	0	0/0	0/0	0/0	0/0	0/0	0/0	0/1	0/1	0/0	0/0	0/0	0/0	0/0	0/0
Chromosome2-3583111 DEL	PRECISE	9924	0	0	0/0	0/0	0/0	0/0	0/0	0/0	0/0	0/0	0/0	0/0	0/0	0/0	0/0	1/1
Chromosome2-3583425 DEL	PRECISE	106	0	0	0/0	0/0	0/0	0/0	0/0	0/0	0/1	0/1	0/0	0/0	0/0	0/0	0/0	0/0
Chromosome2-3584022 INS	PRECISE	96	0	0	0/0	0/0	0/0	0/1	0/0	0/0	0/0	0/0	0/0	0/0	0/0	0/0	0/0	0/0
Chromosome2-3584496 BND	PRECISE	0	0	0	0/0	0/0	0/0	0/1	0/0	0/0	0/0	0/0	0/0	0/0	0/0	0/0	0/0	0/0
Chromosome2-3584756 BND	PRECISE	0	0	0	0/0	0/0	0/0	0/0	0/0	0/0	0/0	0/0	0/1	0/1	0/1	0/1	1/1	0/0
Chromosome2-3585759 DEL	PRECISE	811	0	0	0/0	0/1	0/0	0/0	0/0	0/0	1/1	0/0	0/1	0/1	1/1	1/1	1/1	1/1
Chromosome2-3586113 INS	PRECISE	276	0	0	0/0	0/0	0/0	1/1	0/0	0/0	0/0	0/0	0/0	0/0	0/0	0/0	0/0	0/0
Chromosome2-3586204 DEL	PRECISE	561	0	0	0/0	0/1	0/0	0/0	0/0	0/0	0/1	0/0	0/1	0/0	0/1	0/1	0/1	0/1
Chromosome2-3586413 DEL	PRECISE	60	0	0	0/0	0/0	0/0	0/0	0/0	0/0	0/0	0/0	0/0	0/1	0/1	0/1	0/0	0/0
Chromosome2-3586435 INS	PRECISE	162	0	0	0/0	0/0	0/0	0/1	0/0	0/0	0/0	0/0	0/0	0/0	0/0	0/0	0/0	0/0
Chromosome2-3587176 INS	PRECISE	1062	0	0	0/0	0/0	0/0	0/1	0/0	0/0	0/0	0/0	0/0	0/0	0/0	0/0	0/0	0/0
Chromosome2-3587876 DEL	PRECISE	78	0	0	0/0	0/0	0/0	0/1	0/0	0/0	0/0	0/0	0/0	0/0	0/0	0/0	0/0	0/0
Chromosome2-3588314 INS	PRECISE	130	0	0.548	0/0	0/0	0/0	0/0	0/0	0/0	0/0	0/0	0/1	0/1	0/1	1/1	0/1	0/0
Chromosome2-3588495 DEL	PRECISE	581	0	0	0/0	0/1	0/1	0/1	1/1	1/1	1/1	1/1	1/1	1/1	1/1	1/1	1/1	1/1
Chromosome2-3589864 INS	PRECISE	105	0	0	0/0	0/0	0/0	0/1	0/0	0/0	0/0	0/0	0/0	0/0	0/0	0/0	0/0	0/0
Chromosome2-3590184 DEL	PRECISE	2520	0	0	0/0	0/1	0/1	0/1	1/1	1/1	1/1	1/1	1/1	1/1	1/1	1/1	1/1	1/1
Chromosome2-3590736 DEL	PRECISE	1236	0	0	0/0	0/0	0/0	0/0	0/0	0/0	0/0	0/0	0/1	0/1	0/1	1/1	0/1	0/0
Chromosome2-3590789 DEL	PRECISE	67	0	0	0/0	0/0	0/0	0/1	0/0	0/0	0/0	0/0	0/0	0/0	0/0	0/0	0/0	0/0
Chromosome2-3591007 DEL	PRECISE	998	0	0	0/0	0/1	0/0	0/0	0/0	0/0	0/1	0/0	0/1	0/1	1/1	1/1	0/1	0/1
Chromosome2-3592280 DEL	PRECISE	53	0	0	0/0	0/1	0/0	0/0	0/0	0/0	0/1	0/0	0/0	0/0	1/1	1/1	0/1	0/0
Chromosome2-3595492 DEL	PRECISE	69	0	0	0/0	0/1	0/0	0/0	0/0	0/0	0/1	0/0	1/1	0/0	0/1	0/1	0/1	0/1
Chromosome2-3597583 DEL	PRECISE	1377	0	0	0/0	0/1	0/0	0/0	0/0	0/0	0/1	0/0	0/1	0/1	1/1	1/1	0/1	0/1
Chromosome2-3597891 INS	PRECISE	232	0	0.488	0/0	0/0	0/0	0/1	1/1	1/1	0/1	1/1	0/1	0/1	0/0	0/0	0/0	0/0
Chromosome2-3598475 INS	PRECISE	8501	0	0.707	0/0	0/0	0/0	0/0	0/0	0/0	0/0	0/0	0/0	0/0	0/0	0/0	0/1	0/1
Chromosome2-3598535 DEL	PRECISE	379	0	0	0/0	0/1	0/0	0/0	0/0	0/0	0/1	0/0	0/1	0/1	1/1	1/1	0/1	0/1
Chromosome2-3599610 INS	PRECISE	996	0	0	0/0	0/0	0/0	0/1	0/0	0/0	0/0	0/0	0/0	0/0	0/0	0/0	0/0	0/0
Chromosome2-3600030 DEL	PRECISE	95	0	0	0/0	0/1	0/0	0/0	0/0	0/0	0/0	0/0	0/1	0/1	1/1	1/1	0/1	0/1
Chromosome2-3600156 DEL	PRECISE	1677	0.452	0.452	0/0	0/0	0/1	1/1	1/1	1/1	1/1	1/1	1/1	1/1	1/1	1/1	1/1	1/1
Chromosome2-3600394 DEL	PRECISE	124	0	0	0/0	0/0	0/0	0/1	0/0	0/0	0/0	0/0	0/0	0/0	0/0	0/0	0/0	0/0
Chromosome2-3600516 DEL	PRECISE	277	0	0	0/0	0/0	0/0	0/0	0/0	0/1	0/0	0/0	0/1	0/0	0/0	0/0	0/0	0/0
Chromosome2-3600726 DEL	PRECISE	59	0	0	0/0	0/0	0/0	0/0	1/1	0/1	0/0	0/1	0/0	0/1	0/0	0/0	0/0	0/0
Chromosome2-3601115 DEL	PRECISE	53	0	0	0/0	0/1	0/1	0/1	1/1	1/1	1/1	1/1	1/1	1/1	1/1	1/1	1/1	1/1
Chromosome2-3601494 INS	PRECISE	74	0	0.333	0/0	0/1	0/0	0/1	0/0	0/0	0/1	0/0	0/1	0/1	1/1	1/1	0/1	0/1
Chromosome2-3601833 INS	PRECISE	52	0	0	0/0	0/0	0/0	0/1	0/0	0/0	0/0	0/0	0/0	0/0	0/0	0/0	0/0	0/0
Chromosome2-3601872 INS	PRECISE	757	0	0	0/0	0/0	0/1	0/0	0/0	0/0	0/0	0/0	0/0	0/0	0/0	0/0	0/0	0/0
Chromosome2-3602290 INS	PRECISE	3317	0	0	0/0	0/0	0/0	0/0	0/0	0/0	0/0	0/0	0/0	0/0	0/0	0/0	0/0	0/0
Chromosome2-3602670 DEL	PRECISE	6261	0	0	0/0	0/0	0/0	0/0	0/1	0/0	0/0	0/0	0/0	0/0	0/0	0/0	0/0	0/0
Chromosome2-3602676 DEL	PRECISE	2897	0	0	0/0	0/1	0/1	0/1	0/0	0/0	0/1	0/0	0/0	0/1	0/1	0/1	0/1	0/1
Chromosome2-3602912 DEL	PRECISE	525	0	0	0/0	0/1	0/0	0/0	0/0	0/0	0/1	0/0	0/1	0/1	0/1	0/1	0/1	0/1
Chromosome2-3603025 DEL	PRECISE	2575	0.832	0	0/0	0/1	0/1	0/1	0/1	1/1	1/1	1/1	1/1	1/1	1/1	1/1	1/1	1/1
Chromosome2-3603290 INS	PRECISE	60	0	0	0/0	0/0	0/0	0/1	0/0	0/0	0/0	0/0	0/0	0/0	0/0	0/0	0/0	0/0
Chromosome2-3603438 INS	PRECISE	5498	0	0	0/0	0/0	0/1	0/0	0/0	0/0	0/0	0/0	0/0	0/0	0/0	0/0	0/0	0/0
Chromosome2-3603629 INS	PRECISE	679	0	0.707	0/0	0/0	0/0	0/0	0/0	0/0	0/0	0/0	0/0	0/0	0/0	0/0	0/1	0/1
Chromosome2-3604678 BND	PRECISE	0	0	0	0/0	0/0	0/1	0/0	0/0	0/0	0/0	0/0	0/0	0/0	0/0	0/0	0/0	0/0
Chromosome2-3604739 INS	PRECISE	84	0	0.378	0/0	0/0	0/0	0/0	1/1	1/1	1/1	0/0	1/1	1/1	0/0	0/0	1/1	1/1
Chromosome2-3606070 DUP	PRECISE	21365	0	0	0/0	0/1	0/0	0/0	0/0	0/0	0/1	0/0	0/1	0/1	0/0	0/0	0/0	0/1
Chromosome2-3607077 BND	PRECISE	0	0	0	0/0	0/0	0/0	0/0	0/0	0/0	0/1	0/0	0/0	0/0	0/1	0/0	0/0	0/0
Chromosome2-3607202 INS	PRECISE	7694	0	0	0/0	0/0	0/1	0/0	0/0	0/0	0/0	0/0	0/0	0/0	0/0	0/0	0/0	0/0
Chromosome2-3607766 INS	PRECISE	7542	0	0	0/0	0/0	0/0	0/0	0/0	0/0	0/0	0/0	0/0	0/0	0/0	0/0	0/0	0/0
Chromosome2-3607910 DEL	PRECISE	85	0	0	0/0	0/1	0/0	0/0	0/0	0/0	0/1	0/0	0/1	0/1	1/1	1/1	0/1	0/0
Chromosome2-3608368 DEL	PRECISE	875	0	0	0/0	0/0	0/0	0/1	0/0	0/0	0/0	0/0	0/0	0/0	0/0	0/0	0/0	0/0
Chromosome2-3608464 DEL	PRECISE	52	0	0	0/0	0/0	1/1	0/0	0/0	0/0	0/0	0/0	0/0	0/0	0/0	0/0	1/1	1/1
Chromosome2-3608647 DEL	PRECISE	12527	0	0	0/0	0/0	0/1	0/1	0/0	0/0	0/0	0/0	0/0	0/0	0/0	0/0	1/1	1/1
Chromosome2-3610072 DEL	PRECISE	1453	0	0	0/0	0/0	0/0	0/0	0/0	0/0	1/1	1/1	0/0	0/0	0/0	0/0	0/0	0/0
Chromosome2-3610956 DEL	PRECISE	91	0	0	0/0	0/0	0/0	0/0	0/1	0/0	1/1	1/1	0/0	0/1	0/0	0/0	0/0	0/0
Chromosome2-3611219 INS	PRECISE	4559	0	0	0/0	0/0	0/0	0/1	0/0	0/0	0/0	0/0	0/0	0/0	0/0	0/0	0/0	0/0
Chromosome2-3611570 INS	PRECISE	5799	0	0	0/0	0/0	0/0	0/1	0/0	0/0	0/0	0/0	0/0	0/0	0/0	0/0	0/0	0/0
Chromosome2-3611943 BND	PRECISE	0	0	0	0/0	0/0	0/0	0/0	0/0	0/0	0/0	0/0	0/0	0/0	0/1	0/0	0/0	0/0
Chromosome2-3612243 DEL	PRECISE	484	0	0	0/0	0/0	0/0	0/1	0/0	0/0	0/0	0/0	0/0	0/0	0/0	0/0	0/0	0/0
Chromosome2-3612380 BND	PRECISE	0	0	0	0/0	0/0	0/0	0/0	0/0	0/1	0/0	0/1	0/0	0/0	0/0	0/0	0/0	0/0
Chromosome2-3615707 INS	PRECISE	669	0	0	0/0	0/0	0/0	0/0	0/1	0/0	0/0	0/0	0/0	0/0	0/0	0/0	0/0	0/0
Chromosome2-3615760 INS	PRECISE	1200	0	0	0/0	0/0	0/0	0/0	0/1	0/0	0/0	0/0	0/0	0/0	0/0	0/0	0/0	0/0
Chromosome2-3616176 DEL	PRECISE	461	0	0	0/0	0/0	0/1	0/1	1/1	0/1	1/1	1/1	0/0	1/1	0/0	0/0	0/0	0/0
Chromosome2-3616318 DEL	PRECISE	349	0	0	0/0	0/1	0/1	0/1	1/1	0/1	1/1	1/1	0/0	1/1	1/1	1/1	1/1	1/1
Chromosome2-3616640 DEL	PRECISE	99	0	0	0/0	0/1	0/0	0/0	0/1	0/0	0/1	0/0	0/0	0/1	1/1	1/1	0/1	0/1
Chromosome2-3616898 INS	PRECISE	56	0	0.463	0/0	0/1	0/0	0/0	0/0	0/0	0/1	0/0	0/1	0/1	1/1	1/1	0/1	0/1
Chromosome2-3621087 BND	PRECISE	0	0	0	0/0	0/0	0/0	0/0	0/1	1/1	0/0	0/0	0/1	0/0	0/0	0/0	0/0	0/0
Chromosome2-3621663 DEL	PRECISE	750	0	0	0/0	0/1	0/0	0/0	0/0	0/0	0/1	0/0	0/1	0/1	1/1	1/1	0/1	0/1
Chromosome2-3621757 INS	PRECISE	999	0	0	0/0	0/0	0/0	0/1	0/0	0/0	0/0	0/0	0/0	0/0	0/0	0/0	0/0	0/0
Chromosome2-3622254 DEL	PRECISE	140	0	0	0/0	0/0	0/0	0/0	0/0	1/1	0/0	0/1	0/1	0/0	0/0	0/0	0/0	0/0
Chromosome2-3622626 DEL	PRECISE	1185	0	0	0/0	0/1	0/0	0/0	0/0	0/0	0/1	0/0	0/1	0/1	1/1	1/1	0/1	0/1

Chromosome2-3622937	DEL	PRECISE	2692	0	0	0/0	0/1	0/0	0/0	0/0	0/0	0/1	0/0	0/1	0/1	1/1	1/1	1/1	1/1
Chromosome2-3623282	INS	PRECISE	193	0	0.354	0/0	0/1	0/0	0/0	0/0	0/0	0/1	0/0	0/1	0/1	1/1	1/1	0/1	0/1
Chromosome2-3623378	INS	PRECISE	157	0	0.354	0/0	0/1	0/0	0/0	0/0	0/0	0/1	0/0	0/1	0/1	1/1	1/1	0/1	0/1
Chromosome2-3623590	DEL	PRECISE	159	0	0	0/0	0/1	0/0	0/0	0/0	0/0	0/1	0/0	1/1	0/1	1/1	1/1	0/1	0/1
Chromosome2-3623622	BND	PRECISE	0	0	0	0/0	0/0	0/0	0/0	0/1	0/0	0/1	0/1	0/1	0/0	0/0	0/0	0/0	0/0
Chromosome2-3623628	INS	PRECISE	247	0	0.354	0/0	0/1	0/0	0/0	0/0	0/0	0/1	0/0	1/1	0/1	1/1	1/1	0/1	0/1
Chromosome2-3624031	INS	PRECISE	138	0	0.707	0/0	0/0	0/0	0/0	0/0	0/0	0/0	0/0	0/0	0/0	0/0	0/0	0/1	0/1
Chromosome2-3624167	DEL	PRECISE	165	0	0	0/0	0/0	0/1	0/0	1/1	1/1	0/1	1/1	0/1	0/1	0/0	0/0	0/0	0/0
Chromosome2-3625448	INS	PRECISE	90	0	0	0/0	0/0	0/0	0/0	0/0	0/0	0/1	0/0	0/1	0/1	1/1	1/1	0/1	0/1
Chromosome2-3625671	INS	PRECISE	2082	0	0	0/0	0/0	0/1	0/0	0/0	0/0	0/0	0/0	0/0	0/0	0/0	0/0	0/0	0/0
Chromosome2-3626082	DEL	PRECISE	139	0	0	0/0	0/1	0/0	0/0	0/0	0/0	0/1	0/0	0/1	0/1	1/1	1/1	0/1	0/1
Chromosome2-3626443	DEL	PRECISE	150	0	0	0/0	0/1	0/0	0/0	0/0	0/0	0/1	0/0	0/1	0/1	1/1	1/1	0/1	0/1
Chromosome2-3627719	INS	PRECISE	389	0	0	0/0	0/0	0/0	0/1	0/0	0/0	0/0	0/0	0/0	0/0	0/0	0/0	0/0	0/0
Chromosome2-3628220	DEL	PRECISE	83	0	0	0/0	0/0	0/0	0/0	0/0	0/0	0/0	0/0	0/0	0/0	0/0	0/0	0/1	0/1
Chromosome2-3629312	DEL	PRECISE	211	0	0	0/0	0/0	0/1	0/1	0/1	0/0	0/0	0/0	0/0	0/0	0/0	0/0	0/0	0/0
Chromosome2-3629861	DEL	PRECISE	588	0	0	0/0	0/1	0/0	0/0	0/0	0/0	0/1	0/0	0/1	0/1	1/1	1/1	0/1	0/1
Chromosome2-3630050	DEL	PRECISE	1278	0.354	0.354	0/0	0/1	0/0	0/0	0/0	0/0	0/1	0/0	0/1	0/1	1/1	1/1	0/1	0/1
Chromosome2-3630349	BND	PRECISE	0	0.707	0	0/0	0/0	0/0	0/0	0/0	0/0	0/0	0/0	0/0	0/1	0/0	0/0	0/0	0/1
Chromosome2-3630680	INS	PRECISE	382	0	0	0/0	0/0	0/0	0/1	0/0	0/0	0/0	0/0	0/0	0/0	0/0	0/0	0/0	0/0
Chromosome2-3630727	INS	PRECISE	5374	0	0	0/0	0/0	1/1	0/0	0/0	0/0	0/0	0/0	0/0	0/0	0/0	0/0	0/0	0/0
Chromosome2-3630846	INS	PRECISE	4961	0	0	0/0	0/0	0/0	1/1	0/0	0/0	0/0	0/0	0/0	0/0	0/0	0/0	0/0	0/0
Chromosome2-3631024	DEL	PRECISE	75	0	0	0/0	0/1	0/0	0/0	0/0	0/0	0/1	0/0	0/0	0/1	1/1	0/1	0/0	0/1
Chromosome2-3631217	DEL	PRECISE	75	0	0	0/0	0/0	0/0	0/0	0/0	0/0	0/1	0/1	0/0	0/0	0/0	0/0	0/0	0/0
Chromosome2-3631255	INS	PRECISE	150	0.577	0	0/0	0/0	0/0	0/0	0/1	0/0	0/1	0/1	0/0	0/0	0/0	0/0	0/0	0/0
Chromosome2-3631837	DEL	PRECISE	63	0	0	0/0	0/1	0/0	0/0	0/0	0/0	0/1	0/0	0/1	0/1	1/1	1/1	0/1	0/1
Chromosome2-3632456	INS	PRECISE	62	0	0	0/0	0/0	0/1	0/0	0/0	0/0	0/0	0/0	0/0	0/0	0/0	0/0	0/0	0/0
Chromosome2-3633606	DEL	PRECISE	1445	0	0	0/0	0/0	0/0	0/0	0/1	0/0	1/1	0/1	0/0	0/0	0/0	0/0	0/0	0/0
Chromosome2-3633975	INS	PRECISE	130	0	0.408	0/0	0/0	0/0	0/0	0/1	0/1	0/0	0/1	0/0	1/1	0/0	0/0	1/1	1/1
Chromosome2-3634152	DEL	PRECISE	60	0	0	0/0	0/0	0/0	0/0	0/1	0/0	1/1	0/1	0/0	0/0	0/0	0/0	0/0	0/0
Chromosome2-3634543	DEL	PRECISE	111	0	0	0/0	0/0	0/0	0/0	0/1	0/1	0/0	0/1	0/0	1/1	0/0	0/0	0/0	0/0
Chromosome2-3635503	DEL	PRECISE	78	0	0	0/0	0/0	0/0	0/0	0/0	0/0	0/0	0/0	0/0	0/0	0/0	0/0	0/1	0/1
Chromosome2-3635508	INS	PRECISE	78	0	0.707	0/0	0/0	0/0	0/0	0/0	0/0	0/0	0/0	0/0	0/0	0/0	0/1	0/0	0/0
Chromosome2-3635935	INS	PRECISE	766	0	0.548	0/0	0/0	0/0	0/0	0/0	0/0	0/0	0/0	0/1	0/1	0/1	1/1	0/1	0/0
Chromosome2-3636632	INS	PRECISE	132	0	0	0/0	0/0	0/1	0/0	0/0	0/0	0/0	0/0	0/0	0/0	0/0	0/0	0/0	0/0
Chromosome2-3636936	DEL	PRECISE	116	0	0	0/0	0/0	0/0	0/0	0/0	0/0	0/0	0/0	0/0	0/0	0/0	0/0	0/1	0/1
Chromosome2-3637167	INS	PRECISE	132	0	0	0/0	0/0	0/0	0/0	0/0	0/0	0/0	0/0	0/0	0/0	0/0	0/0	0/0	0/1
Chromosome2-3637181	DEL	PRECISE	58	0	0	0/0	0/0	0/0	0/0	1/1	0/1	0/0	0/1	0/1	1/1	0/1	1/1	1/1	0/1
Chromosome2-3637589	DUP	PRECISE	25208	0	0	0/0	0/0	0/0	0/1	0/0	0/0	0/0	0/0	0/0	0/0	0/0	0/0	0/0	0/0
Chromosome2-3637964	INS	PRECISE	179	0	0	0/0	0/0	0/0	0/0	0/0	0/0	0/0	0/0	0/0	0/1	0/1	0/1	0/0	0/0
Chromosome2-3638215	INS	PRECISE	1621	0	0.548	0/0	0/0	0/0	0/0	0/1	1/1	0/0	0/0	1/1	1/1	0/0	0/0	0/0	0/0
Chromosome2-3638310	INS	PRECISE	12233	0	0	0/0	0/0	0/0	0/0	1/1	0/0	0/0	0/0	0/0	0/0	0/0	0/0	0/0	0/0
Chromosome2-3640303	DEL	PRECISE	70	0.707	0.707	0/0	0/0	0/0	0/0	0/0	0/0	0/0	0/0	0/0	0/0	0/0	0/0	0/1	0/1
Chromosome2-3640360	DEL	PRECISE	658	0.354	0.354	0/0	0/1	0/0	0/0	0/0	0/0	0/1	0/0	0/1	0/1	1/1	1/1	0/1	0/1
Chromosome2-3640400	INS	PRECISE	976	0	0	0/0	0/0	0/0	0/1	0/0	0/0	0/0	0/0	0/0	0/0	0/0	0/0	0/0	0/0
Chromosome2-3641250	INS	PRECISE	67	0.5	0.816	0/0	0/0	0/0	0/0	0/0	0/0	0/0	0/0	0/0	0/0	0/0	0/1	0/0	0/0
Chromosome2-3641590	DEL	PRECISE	212	0	0	0/0	0/1	0/1	0/0	1/1	1/1	0/1	0/0	1/1	1/1	1/1	1/1	0/0	1/1
Chromosome2-3641912	INS	PRECISE	293	0	0	0/0	0/0	0/0	0/1	0/0	0/0	0/0	0/0	0/0	0/0	0/0	0/0	0/0	0/0
Chromosome2-3642789	DEL	PRECISE	994	0	0	0/0	0/0	0/1	0/1	1/1	1/1	1/1	1/1	1/1	1/1	1/1	1/1	1/1	1/1
Chromosome2-3643054	INS	PRECISE	69	0	0	0/0	0/0	0/0	0/1	0/0	0/0	0/0	0/0	0/0	0/0	0/0	0/0	0/0	0/0
Chromosome2-3643108	INS	PRECISE	1411	0	0	0/0	0/0	0/0	0/0	0/1	0/0	0/0	0/0	0/0	0/0	0/0	0/0	0/0	0/0
Chromosome2-3644524	DEL	PRECISE	5096	0	0	0/0	0/1	0/0	0/0	0/1	0/1	0/1	0/1	0/1	1/1	1/1	1/1	1/1	1/1
Chromosome2-3644784	DEL	PRECISE	2243	0	0	0/0	0/0	0/0	0/1	0/0	0/0	0/0	0/0	0/0	0/0	0/0	0/0	0/0	0/0
Chromosome2-3645563	DEL	PRECISE	133	0	0	0/0	0/1	0/1	0/1	1/1	1/1	1/1	1/1	1/1	1/1	1/1	1/1	1/1	1/1
Chromosome2-3645733	DEL	PRECISE	105	0	0	0/0	0/0	0/0	0/0	0/0	0/0	0/0	0/0	0/0	0/0	0/0	0/0	0/1	0/1
Chromosome2-3645859	DEL	PRECISE	393	0	0.5	0/0	0/0	0/1	0/1	0/0	0/0	0/0	0/0	0/0	0/0	0/0	0/0	0/1	0/1
Chromosome2-3646260	INS	PRECISE	196	0	0.5	0/0	0/0	0/0	0/0	0/1	0/1	0/0	0/1	0/0	1/1	0/0	0/0	0/0	0/0
Chromosome2-3647792	INS	PRECISE	376	0	0.535	0/0	0/1	0/0	0/0	0/0	0/0	0/1	0/0	0/1	0/1	1/1	1/1	0/1	0/1
Chromosome2-3648273	DEL	PRECISE	132	0	0	0/0	0/1	0/0	0/0	0/1	0/1	0/1	0/1	0/1	1/1	1/1	1/1	1/1	0/1
Chromosome2-3648610	INS	PRECISE	73	0	0	0/0	0/0	0/0	0/1	0/0	0/0	0/0	0/0	0/0	0/0	0/0	0/0	0/0	0/0
Chromosome2-3650841	INS	PRECISE	2183	0	0	0/0	0/0	0/0	0/0	0/0	0/0	0/0	0/0	0/0	0/0	0/1	0/0	0/0	0/0
Chromosome2-3650841	DUP	PRECISE	6791	0	0	0/0	0/0	0/0	0/0	0/0	0/0	0/0	0/0	0/0	0/0	0/1	0/0	0/0	0/0
Chromosome2-3650842	DEL	PRECISE	4576	0	0	0/0	0/0	0/0	0/0	0/0	0/0	0/0	0/0	0/0	1/1	0/1	0/0	0/0	0/0
Chromosome2-3650846	BND	PRECISE	0	0	0	0/0	0/0	0/0	0/0	0/0	0/0	0/0	1/1	0/0	1/1	0/0	0/0	0/0	0/0
Chromosome2-3651130	DEL	PRECISE	81	0	0	0/0	0/0	0/0	0/0	0/0	0/0	0/1	0/0	0/0	1/1	0/1	0/0	0/0	0/0
Chromosome2-3651173	INS	PRECISE	72	0	0.996	0/0	0/0	0/1	0/1	0/0	1/1	1/1	1/1	1/1	1/1	1/1	0/0	1/1	0/0
Chromosome2-3651713	DEL	PRECISE	152	0	0	0/0	0/1	0/0	0/0	0/0	0/0	0/1	0/0	0/0	0/0	0/1	0/0	0/0	0/1
Chromosome2-3651792	DEL	PRECISE	854	0	0	0/0	0/1	0/0	0/0	0/0	0/0	0/1	0/0	0/0	0/0	0/1	0/0	0/0	0/1
Chromosome2-3652103	DEL	PRECISE	501	0	0	0/0	0/1	0/0	0/0	0/0	0/0	0/1	0/0	0/0	0/0	0/1	0/0	0/0	0/1
Chromosome2-3652602	DEL	PRECISE	200	0	0	0/0	0/0	0/0	0/0	0/1	0/0	0/0	0/1	0/0	0/0	0/0	0/0	0/0	0/0
Chromosome2-3652809	BND	PRECISE	0	0	0	0/0	0/0	0/0	0/0	0/0	0/0	0/1	0/0	0/0	0/0	0/0	0/0	0/0	0/0
Chromosome2-3653990	INS	PRECISE	266	0.289	0.835	0/0	0/1	0/0	0/1	0/0	0/1	0/1	0/1	0/1	1/1	1/1	1/1	1/1	1/1
Chromosome2-3654605	DEL	PRECISE	85	0	0	0/0	0/0	0/0	0/0	0/0	0/0	0/0	0/0	0/0	0/0	0/0	0/0	0/1	0/1
Chromosome2-3654867	DEL	PRECISE	454	0	0	0/0	0/1	0/0	0/1	1/1	0/1	1/1	1/1	0/1	1/1	1/1	1/1	1/1	1/1

Chromosome2-3655234 DEL	PRECISE	236	0	0	0/0	0/0	0/0	0/0	0/1	0/1	0/0	0/1	0/0	1/1	0/1	0/1	0/0	0/0
Chromosome2-3656671 DEL	PRECISE	381	0.5	0	0/0	0/0	0/0	0/0	0/1	0/1	0/0	0/1	0/0	1/1	0/0	0/0	0/0	0/0
Chromosome2-3657266 DEL	PRECISE	1097	0.467	0	0/0	0/1	0/0	0/0	0/1	1/1	0/1	0/1	0/1	1/1	1/1	1/1	1/1	0/1
Chromosome2-3657916 INS	PRECISE	833	0	0	0/0	0/0	0/0	0/0	0/0	0/0	0/0	0/0	0/0	0/0	0/0	0/0	0/0	0/1
Chromosome2-3658700 DEL	PRECISE	147	0	0	0/0	0/0	0/0	0/0	0/1	0/1	0/0	0/0	0/1	1/1	1/1	1/1	0/1	0/1
Chromosome2-3659159 INS	PRECISE	256	0	0.707	0/0	0/0	0/0	0/0	0/0	0/0	0/0	0/0	0/0	0/0	0/0	0/0	0/1	0/1
Chromosome2-3659668 INS	PRECISE	66	0	0	0/0	0/0	0/0	0/1	0/0	0/0	0/0	0/0	0/0	0/0	0/0	0/0	0/0	0/0
Chromosome2-3659958 INS	PRECISE	909	0	0	0/0	0/0	0/0	0/0	0/0	0/0	0/0	0/0	0/0	0/0	0/0	0/0	0/0	1/1
Chromosome2-3660470 INS	PRECISE	132	0	0	0/0	0/0	0/0	0/0	0/0	0/0	0/0	0/0	0/1	0/0	0/0	1/1	0/0	0/0
Chromosome2-3660479 DEL	PRECISE	212	0	0	0/0	0/1	0/0	0/0	0/1	0/1	0/1	0/1	0/1	1/1	1/1	1/1	0/1	0/1
Chromosome2-3660604 DEL	PRECISE	187	0	0	0/0	0/0	0/0	0/0	0/0	0/0	0/0	0/0	0/0	1/1	0/1	0/0	0/1	0/0
Chromosome2-3660622 INS	PRECISE	364	0	0.707	0/0	0/1	0/0	0/0	0/1	0/1	0/1	0/1	0/0	0/0	0/1	0/0	0/0	0/1
Chromosome2-3660723 DEL	PRECISE	105	0	0	0/0	0/0	0/0	0/0	0/0	0/0	0/0	0/0	0/1	0/0	0/0	0/1	0/0	0/0
Chromosome2-3660784 INS	PRECISE	712	0	0	0/0	0/0	0/0	0/1	0/0	0/0	0/0	0/0	0/0	0/0	0/0	0/0	0/0	0/0
Chromosome2-3660853 INS	PRECISE	80	0	0.405	0/0	0/1	0/0	0/0	0/1	0/1	0/1	0/1	0/1	1/1	1/1	1/1	0/1	0/1
Chromosome2-3661354 INS	PRECISE	1402	0	0	0/0	0/0	0/0	0/0	0/0	0/0	0/0	0/0	0/0	0/0	0/0	0/0	0/1	0/0
Chromosome2-3662072 INS	PRECISE	924	0	0	0/0	0/0	0/1	0/0	0/0	0/0	0/0	0/0	0/0	0/0	0/0	0/0	0/0	0/0
Chromosome2-3662250 DEL	PRECISE	348	0	0	0/0	0/1	0/0	0/0	0/1	0/1	0/1	0/1	0/1	1/1	1/1	1/1	0/1	0/1
Chromosome2-3662760 INS	PRECISE	212	0	0	0/0	0/0	0/1	0/0	0/0	0/0	0/0	0/0	0/0	0/0	0/0	0/0	0/0	0/0
Chromosome2-3663573 INS	PRECISE	146	0	0.577	0/0	0/1	0/0	0/0	0/0	0/0	0/1	0/0	0/0	0/0	1/1	0/0	0/0	0/1
Chromosome2-3663748 INS	PRECISE	711	0	0	0/0	0/0	0/0	0/0	0/0	0/0	0/0	0/0	0/0	0/0	0/1	0/0	0/0	0/0
Chromosome2-3665422 DEL	PRECISE	60	0	0	0/0	0/0	0/0	0/1	0/0	0/0	0/0	0/0	0/0	0/0	0/0	0/0	0/0	0/0
Chromosome2-3665863 DEL	PRECISE	91	0	0	0/0	0/0	0/0	0/0	0/0	0/0	0/0	0/0	0/1	0/0	0/0	0/1	0/0	0/0
Chromosome2-3666058 INS	PRECISE	654	0	0	0/0	0/0	0/0	0/0	1/1	0/0	0/0	0/0	0/0	0/0	0/0	0/0	0/0	0/0
Chromosome2-3666164 DEL	PRECISE	54	0	0	0/0	0/0	0/0	0/1	0/0	0/0	0/0	0/0	0/0	0/0	0/0	0/0	0/0	0/0
Chromosome2-3666256 INS	PRECISE	274	0	0.707	0/0	0/0	0/0	0/0	0/0	0/0	0/0	0/0	0/0	0/0	0/0	0/0	0/1	0/1
Chromosome2-3666956 DEL	PRECISE	1336	0	0	0/0	0/1	0/1	0/1	1/1	1/1	1/1	1/1	1/1	1/1	1/1	1/1	1/1	1/1
Chromosome2-3667842 INS	PRECISE	313	0	0.577	0/0	0/0	0/0	0/0	0/1	0/1	0/0	0/1	0/0	0/1	0/0	0/0	0/0	0/0
Chromosome2-3667921 DEL	PRECISE	1570	0	0	0/0	0/0	0/0	0/0	0/1	1/1	0/0	1/1	0/1	0/0	0/0	0/0	0/0	0/0
Chromosome2-3668084 INS	PRECISE	631	0	0	0/0	0/0	0/0	0/1	0/0	0/0	0/0	0/0	0/0	0/0	0/0	0/0	0/0	0/0
Chromosome2-3668550 BND	PRECISE	0	0	0	0/0	0/0	0/0	0/0	0/0	0/0	0/0	0/0	0/0	0/0	0/0	0/0	0/1	0/0
Chromosome2-3668612 INS	PRECISE	8104	0	0	0/0	0/0	0/0	0/0	0/0	0/0	0/0	0/0	0/0	0/0	0/0	0/0	0/0	1/1
Chromosome2-3668627 INS	PRECISE	1907	0	0.577	0/0	0/0	0/0	0/0	0/0	0/0	0/0	0/0	0/0	0/1	0/0	0/1	0/1	0/0
Chromosome2-3668642 DUP	PRECISE	15918	0	0	0/0	0/0	0/0	0/0	0/0	0/0	0/0	0/0	0/1	0/1	0/1	0/1	0/0	0/0
Chromosome2-3668998 INS	PRECISE	5198	0	0	0/0	0/0	0/1	0/0	0/0	0/0	0/0	0/0	0/0	0/0	0/0	0/0	0/0	0/0
Chromosome2-3669175 INS	PRECISE	1656	0	0	0/0	0/0	0/0	0/0	0/0	0/0	0/0	0/0	0/0	0/1	0/0	0/0	0/0	0/0
Chromosome2-3669998 INS	PRECISE	210	0	0	0/0	0/0	0/1	0/0	0/0	0/0	0/0	0/0	0/0	0/0	0/0	0/0	0/0	0/0
Chromosome2-3670012 DEL	PRECISE	51	0	0	0/0	0/0	0/0	0/0	0/0	0/1	0/0	0/0	0/1	0/0	0/0	0/0	0/0	0/0
Chromosome2-3670115 INS	PRECISE	5275	0	0	0/0	0/0	0/0	0/0	0/0	0/0	0/0	0/0	0/0	0/0	0/1	0/0	0/0	0/0
Chromosome2-3670294 BND	PRECISE	0	0	0	0/0	0/0	0/0	0/0	0/0	0/0	0/0	0/0	0/0	0/1	0/0	0/1	0/1	0/0
Chromosome2-3670947 INS	PRECISE	9526	0	0	0/0	0/0	0/0	0/0	0/0	0/0	0/0	0/0	0/0	0/0	1/1	0/0	0/0	0/0
Chromosome2-3670947 BND	PRECISE	0	0	0	0/0	0/0	0/0	0/0	0/0	0/0	0/0	0/0	0/0	0/0	0/1	0/0	0/0	0/0
Chromosome2-3670947 BND	PRECISE	0	0	0	0/0	0/0	0/0	0/0	0/0	0/0	0/0	0/0	0/0	0/0	0/1	0/0	0/0	0/0
Chromosome2-3671452 INS	PRECISE	748	0	0	0/0	0/0	0/0	0/0	0/1	0/0	0/0	0/0	0/0	0/0	0/0	0/0	0/0	0/0
Chromosome2-3671645 DEL	PRECISE	259	0	0	0/0	0/0	0/0	0/0	0/0	0/0	0/0	0/0	0/0	0/0	0/1	0/0	0/0	0/0
Chromosome2-3672064 DEL	PRECISE	287	0	0	0/0	0/0	0/0	0/0	1/1	0/0	0/0	0/1	1/1	0/0	0/0	0/0	1/1	0/0
Chromosome2-3673171 BND	PRECISE	0	0	0	0/0	0/0	0/0	0/1	0/0	0/0	0/0	0/0	0/0	0/0	0/0	0/0	0/0	0/0
Chromosome2-3674301 DEL	PRECISE	87	0	0	0/0	0/1	0/0	0/0	0/1	0/1	0/1	0/1	0/1	1/1	1/1	1/1	0/1	1/1
Chromosome2-3674705 DEL	PRECISE	443	0	0	0/0	0/1	0/0	0/0	0/0	0/0	0/1	0/0	0/0	0/0	1/1	1/1	0/0	0/1
Chromosome2-3674904 INS	PRECISE	2641	0	0	0/0	0/0	0/0	0/0	1/1	0/0	0/0	0/0	0/0	0/0	0/0	0/0	0/0	0/0
Chromosome2-3674922 INS	PRECISE	65	0	0	0/0	0/1	0/0	0/1	1/1	1/1	0/1	0/1	1/1	1/1	1/1	1/1	1/1	1/1
Chromosome2-3674933 DEL	PRECISE	783	0.302	0.647	0/0	0/1	0/0	0/0	0/1	0/1	0/1	0/1	0/1	1/1	1/1	1/1	1/1	1/1
Chromosome2-3675214 DEL	PRECISE	215	0.506	0	0/0	0/1	0/1	0/1	1/1	1/1	1/1	1/1	1/1	1/1	1/1	1/1	0/1	0/1
Chromosome2-3676346 INS	PRECISE	724	0	0.467	0/0	0/1	0/0	0/0	0/1	0/1	0/1	0/1	0/1	1/1	1/1	1/1	0/1	0/1
Chromosome2-3677223 INS	PRECISE	93	0	0	0/0	0/0	0/0	0/1	0/0	0/0	0/0	0/0	0/0	0/0	0/0	0/0	0/0	0/0
Chromosome2-3678340 DEL	PRECISE	682	0	0	0/0	0/1	0/0	0/0	0/1	0/1	0/1	0/1	0/0	1/1	1/1	0/0	0/0	0/1
Chromosome2-3678340 BND	PRECISE	0	0	0	0/0	0/0	0/0	0/0	0/0	0/0	0/0	0/0	0/0	0/1	0/0	0/0	0/1	0/0
Chromosome2-3678439 DEL	PRECISE	2941	0	0	0/0	0/1	0/0	0/0	0/1	0/1	0/1	1/1	0/0	1/1	1/1	0/0	0/0	0/1
Chromosome2-3678733 BND	PRECISE	0	0	0	0/0	0/0	0/0	0/0	0/0	0/0	0/0	0/0	0/0	0/1	0/0	0/0	0/0	0/0
Chromosome2-3679813 DEL	PRECISE	79	0	0	0/0	0/1	0/0	0/0	0/1	0/1	0/1	1/1	0/1	1/1	1/1	1/1	0/1	0/1
Chromosome2-3680160 INS	PRECISE	98	0	0.302	0/0	0/1	0/0	0/0	0/1	0/1	0/1	1/1	0/1	1/1	1/1	1/1	0/1	0/1
Chromosome2-3680476 INS	PRECISE	70	0	0	0/0	0/1	0/0	0/0	0/1	0/1	0/1	1/1	0/1	1/1	1/1	1/1	0/1	0/1
Chromosome2-3680768 INS	PRECISE	188	0	0.647	0/0	0/1	0/0	0/0	0/1	0/1	0/1	0/1	0/1	1/1	1/1	1/1	0/1	0/1
Chromosome2-3680947 INS	PRECISE	132	0	0	0/0	0/0	0/0	0/0	0/0	0/0	0/0	0/0	0/0	0/0	0/1	0/0	0/0	0/0
Chromosome2-3680982 DEL	PRECISE	297	0	0	0/0	0/1	0/0	0/0	0/1	0/1	0/1	0/1	0/1	1/1	1/1	1/1	0/1	0/1
Chromosome2-3681181 DEL	PRECISE	997	0	0	0/0	0/1	0/0	0/0	0/1	1/1	0/1	0/1	0/1	1/1	1/1	1/1	0/1	0/1
Chromosome2-3681337 INS	PRECISE	1389	0	0	0/0	0/0	0/0	0/1	0/0	0/0	0/0	0/0	0/0	0/0	0/0	0/0	0/0	0/0
Chromosome2-3681534 DEL	PRECISE	755	0	0	0/0	0/1	0/0	0/0	0/1	0/1	0/1	0/1	0/1	1/1	1/1	1/1	0/1	0/1
Chromosome2-3682277 INS	PRECISE	161	0	0	0/0	0/0	0/0	0/0	0/0	0/0	0/0	0/0	0/1	0/0	0/0	0/1	0/0	0/0
Chromosome2-3682319 INS	PRECISE	874	0	0	0/0	0/0	0/0	0/1	0/0	0/0	0/0	0/0	0/0	0/0	0/0	0/0	0/0	0/0
Chromosome2-3683606 DEL	PRECISE	3093	0	0	0/0	0/0	0/0	0/1	0/0	0/0	0/0	0/0	0/0	0/0	0/0	0/0	1/1	1/1
Chromosome2-3684056 DEL	PRECISE	207	0	0	0/0	0/1	0/0	0/0	0/1	0/1	0/0	0/0	0/1	1/1	1/1	1/1	0/0	0/1
Chromosome2-3684221 INS	PRECISE	331	0	0	0/0	0/0	0/0	0/0	0/0	0/0	1/1	1/1	0/0	0/0	0/0	0/0	0/0	0/0

Chromosome2-3684850	INS	PRECISE	133	0	0	0/0	0/0	0/0	0/0	0/0	0/0	0/0	0/0	0/0	0/0	0/0	0/0	0/0	1/1
Chromosome2-3686499	DEL	PRECISE	61	0	0	0/0	0/1	0/0	0/0	0/0	0/0	0/1	0/0	0/0	0/1	0/1	0/1	0/1	0/1
Chromosome2-3686959	INS	PRECISE	93	0	0	0/0	0/0	0/0	0/0	0/0	0/0	0/0	0/0	0/0	0/0	0/0	0/0	0/1	0/1
Chromosome2-3687690	DEL	PRECISE	94	0	0	0/0	0/1	0/0	0/0	0/0	0/0	0/1	0/0	0/0	0/1	0/1	0/1	0/1	0/1
Chromosome2-3689049	DEL	PRECISE	994	0	0	0/0	0/1	0/1	0/1	1/1	1/1	1/1	1/1	1/1	1/1	1/1	1/1	1/1	1/1
Chromosome2-3689184	INS	PRECISE	800	0	0.577	0/0	0/0	0/0	0/0	0/0	0/0	0/0	0/0	0/1	0/0	0/1	0/1	0/0	0/0
Chromosome2-3689273	INS	PRECISE	4745	0	0	0/0	0/0	0/0	0/0	1/1	0/0	0/0	0/0	0/0	0/0	0/0	0/0	0/0	0/0
Chromosome2-3689327	DEL	PRECISE	1009	0	0	0/0	0/1	0/0	0/0	0/1	0/1	0/1	0/1	0/0	1/1	1/1	1/1	0/1	0/1
Chromosome2-3690008	DEL	PRECISE	64	0	0	0/0	0/0	0/0	0/0	0/1	0/1	0/0	0/1	0/0	0/1	0/0	0/0	0/0	0/0
Chromosome2-3690820	INS	PRECISE	820	0	0	0/0	0/0	0/0	0/1	0/0	0/0	0/0	0/0	0/0	0/0	0/0	0/0	0/0	0/0
Chromosome2-3691283	INS	PRECISE	190	0	0	0/0	1/1	0/0	0/0	0/0	0/0	0/1	0/0	0/0	0/1	1/1	0/1	0/1	0/1
Chromosome2-3691393	INS	PRECISE	656	0	0	0/0	0/0	0/0	0/1	0/0	0/0	0/0	0/0	0/0	0/0	0/0	0/0	0/0	0/0
Chromosome2-3691516	BND	PRECISE	0	0	0	0/0	0/0	0/0	0/0	0/0	0/0	0/0	0/0	0/0	0/1	0/0	0/0	0/0	0/0
Chromosome2-3692065	INS	PRECISE	147	0	0.467	0/0	0/1	0/0	0/0	0/1	0/1	0/1	0/0	0/1	1/1	1/1	1/1	0/1	0/1
Chromosome2-3692731	INS	PRECISE	1546	0	0.707	0/0	0/0	0/0	0/0	0/0	0/0	0/0	0/0	0/0	0/0	0/0	0/0	0/1	0/1
Chromosome2-3692926	INS	PRECISE	955	0	0.707	0/0	0/0	0/0	0/0	0/0	0/0	0/0	0/0	0/0	0/0	0/0	0/0	0/1	0/1
Chromosome2-3694799	DEL	PRECISE	50	0	0	0/0	0/0	0/0	0/0	0/0	0/0	0/0	0/0	0/1	0/0	0/0	1/1	0/0	0/0
Chromosome2-3695776	INS	PRECISE	325	0	0.577	0/0	0/0	0/0	0/0	0/1	0/0	0/1	0/1	0/0	0/0	0/0	0/0	0/0	0/0
Chromosome2-3695866	DEL	PRECISE	93	0	0	0/0	0/0	0/0	0/0	0/0	0/0	0/0	0/0	0/1	0/0	0/0	0/1	0/0	0/0
Chromosome2-3696122	INS	PRECISE	1188	0	0	0/0	0/0	0/0	0/0	0/0	0/0	0/0	0/0	0/0	0/0	0/0	0/0	0/1	0/1
Chromosome2-3696151	INS	PRECISE	5932	0	0	0/0	0/0	0/0	0/0	1/1	0/0	0/0	0/0	0/0	0/0	0/0	0/0	0/0	0/0
Chromosome2-3698349	DEL	PRECISE	80	0	0	0/0	0/0	0/0	0/0	0/0	0/0	0/0	0/0	0/0	0/0	0/0	0/0	0/1	0/1
Chromosome2-3699501	DEL	PRECISE	89	0	0	0/0	0/0	0/0	0/0	0/0	0/0	0/0	0/0	0/1	0/0	0/0	1/1	0/0	1/1
Chromosome2-3699605	DEL	PRECISE	97	0	0	0/0	0/0	0/0	0/0	0/1	0/0	0/0	0/1	0/0	1/1	0/0	0/0	0/0	0/0
Chromosome2-3702925	DEL	PRECISE	51	0	0	0/0	0/1	0/0	0/0	0/0	0/0	0/1	0/0	0/0	1/1	1/1	1/1	0/0	0/0
Chromosome2-3703107	INS	PRECISE	4942	0	0	0/0	0/0	0/0	0/0	1/1	0/0	0/0	0/0	0/0	0/0	0/0	0/0	0/0	0/0
Chromosome2-3704812	DEL	PRECISE	144	0	0	0/0	0/1	0/0	0/0	0/1	0/1	0/1	0/1	0/1	1/1	1/1	1/1	0/1	0/1
Chromosome2-3705046	DEL	PRECISE	211	0	0	0/0	0/1	0/1	0/1	1/1	0/1	1/1	1/1	0/1	1/1	1/1	1/1	1/1	1/1
Chromosome2-3706863	INS	PRECISE	150	0	0.707	0/0	0/0	0/0	0/0	0/0	0/1	0/0	0/0	0/1	0/0	0/0	0/0	0/0	0/0
Chromosome2-3707477	DEL	PRECISE	625	0	0	0/0	0/1	0/0	0/1	0/1	1/1	0/1	0/1	1/1	1/1	1/1	1/1	1/1	1/1
Chromosome2-3707979	INS	PRECISE	697	0	0	0/0	0/0	0/0	0/0	0/0	0/0	0/0	0/0	0/0	0/0	0/0	0/0	0/1	0/0
Chromosome2-3708413	INS	PRECISE	106	0	0	0/0	0/1	0/0	0/0	0/0	0/0	0/1	0/0	0/0	1/1	0/0	0/1	0/0	0/1
Chromosome2-3708531	DEL	PRECISE	271	0	0	0/0	0/1	0/0	0/0	0/1	0/1	0/1	0/1	0/1	1/1	1/1	1/1	0/1	0/1
Chromosome2-3708642	DEL	PRECISE	194	0	0	0/0	0/1	0/0	0/0	0/1	0/1	0/1	0/1	0/1	1/1	1/1	1/1	0/1	0/1
Chromosome2-3709277	INS	PRECISE	317	0	0.408	0/0	0/1	0/0	0/0	0/0	0/0	0/1	0/0	0/0	0/1	0/1	0/1	0/0	0/1
Chromosome2-3709857	DEL	PRECISE	53	0	0	0/0	0/0	0/0	0/0	0/0	0/1	0/0	0/1	0/1	0/1	0/1	0/1	0/1	0/0
Chromosome2-3709960	INS	PRECISE	994	0	0.837	0/0	0/1	0/0	0/0	0/0	0/0	0/1	0/0	0/0	0/1	0/1	0/1	0/0	0/1
Chromosome2-3710294	DEL	PRECISE	370	0	0	0/0	0/0	0/0	0/0	0/0	0/0	0/0	0/0	0/0	0/0	0/0	0/0	0/1	0/0
Chromosome2-3711327	INS	PRECISE	889	0	0	0/0	0/0	0/0	0/0	0/0	0/0	0/0	0/0	0/0	0/0	0/0	0/0	0/1	0/0
Chromosome2-3711660	INS	PRECISE	231	0	0.707	0/0	0/0	0/0	0/0	0/0	0/0	0/1	0/1	0/0	0/0	0/0	0/0	0/0	0/0
Chromosome2-3711853	INS	PRECISE	121	0	0	0/0	0/0	0/0	0/0	0/0	0/0	0/1	0/1	0/0	0/0	0/0	0/0	0/0	0/0
Chromosome2-3712315	INS	PRECISE	165	0	0	0/0	0/0	0/0	0/0	0/0	0/0	0/0	0/0	0/0	0/0	0/1	0/0	0/0	0/0
Chromosome2-3712547	INS	PRECISE	160	0	0	0/0	0/0	0/0	0/0	0/0	0/0	0/0	0/0	0/0	0/0	0/0	0/0	1/1	0/1
Chromosome2-3713301	INS	PRECISE	5369	0	0	0/0	0/0	0/0	1/1	0/0	0/0	0/0	0/0	0/0	0/0	0/0	0/0	0/0	0/0
Chromosome2-3713759	DEL	PRECISE	276	0	0	0/0	0/0	0/0	0/1	0/0	0/0	0/0	0/0	0/0	0/0	0/0	0/0	0/0	0/0
Chromosome2-3714394	INS	PRECISE	133	0	0.516	0/0	0/1	0/0	0/0	0/0	0/0	0/1	0/0	0/0	0/1	0/1	0/1	0/0	0/1
Chromosome2-3714808	INS	PRECISE	6868	0	0	0/0	0/0	0/0	0/0	0/0	0/0	0/0	0/0	0/0	0/0	0/0	0/0	0/0	0/1
Chromosome2-3715093	DEL	PRECISE	825	0.905	0.905	0/0	0/1	0/0	0/0	0/1	0/1	0/1	0/1	1/1	1/1	1/1	1/1	1/1	1/1
Chromosome2-3715244	DEL	PRECISE	152	0	0	0/0	0/0	0/0	0/0	0/0	0/0	0/0	0/0	0/1	0/0	0/0	0/0	0/0	0/0
Chromosome2-3715336	INS	PRECISE	211	0	0	0/0	0/0	0/0	0/0	0/0	0/0	0/0	0/0	0/0	0/0	0/0	0/0	0/1	0/0
Chromosome2-3716224	INS	PRECISE	6993	0	0	0/0	0/0	0/0	1/1	0/0	0/0	0/0	0/0	0/0	0/0	0/0	0/0	0/0	0/0
Chromosome2-3716338	DEL	PRECISE	58	0	0	0/0	0/0	0/0	0/0	0/0	0/0	0/0	0/0	0/1	0/0	0/1	0/1	0/1	0/0
Chromosome2-3716348	INS	PRECISE	997	0	0	0/0	0/0	0/1	0/0	0/0	0/0	0/0	0/0	0/0	0/0	0/0	0/0	0/0	0/0
Chromosome2-3716397	DEL	PRECISE	697	0	0	0/0	0/0	0/0	0/0	0/0	0/1	0/0	0/0	0/1	0/0	0/0	0/0	0/0	0/0
Chromosome2-3716405	BND	PRECISE	0	0	0	0/0	0/0	0/0	0/0	0/0	0/0	0/0	0/0	0/1	0/0	0/0	0/1	0/0	0/0
Chromosome2-3718381	DEL	PRECISE	1043	0	0	0/0	0/0	0/0	0/0	0/1	0/0	0/0	0/0	0/0	0/0	0/0	0/0	0/0	0/0
Chromosome2-3718450	DEL	PRECISE	5826	0	0	0/0	0/0	0/0	0/0	0/0	0/0	0/0	0/0	0/0	0/0	0/0	0/0	1/1	1/1
Chromosome2-3718647	DEL	PRECISE	1191	0	0	0/0	0/0	0/0	0/0	1/1	0/0	0/0	0/0	0/0	0/0	0/0	0/0	0/0	0/0
Chromosome2-3718771	DEL	PRECISE	2624	0	0	0/0	0/0	0/0	0/0	1/1	0/0	0/0	0/0	0/0	0/0	0/0	0/0	0/0	0/0
Chromosome2-3719002	DEL	PRECISE	212	0	0	0/0	0/0	0/1	0/0	0/0	0/0	0/0	0/0	0/0	0/0	0/0	0/0	0/0	0/0
Chromosome2-3719101	DEL	PRECISE	97	0	0	0/0	0/0	0/0	0/1	0/0	0/0	0/0	0/0	0/0	0/0	0/0	0/0	0/0	0/0
Chromosome2-3719528	DEL	PRECISE	246	0	0	0/0	0/1	0/0	0/0	0/1	0/0	0/1	0/1	0/1	1/1	1/1	1/1	0/1	0/1
Chromosome2-3719819	DEL	PRECISE	125	0	0	0/0	0/1	0/0	0/0	0/1	0/1	0/1	0/1	0/0	1/1	1/1	0/1	0/1	0/1
Chromosome2-3720075	DEL	PRECISE	50	0	0	0/0	0/0	0/0	0/0	0/0	0/0	0/0	0/0	0/0	0/0	0/0	0/0	0/1	0/1
Chromosome2-3720834	DEL	PRECISE	411	0	0	0/0	0/0	0/0	0/0	0/0	0/0	0/0	0/0	0/0	0/0	0/0	0/0	0/1	0/0
Chromosome2-3720970	INS	PRECISE	4945	0	0	0/0	0/0	0/0	0/0	1/1	0/0	0/0	0/0	0/0	0/0	0/0	0/0	0/0	0/0
Chromosome2-3721335	INS	PRECISE	4942	0	0	0/0	0/0	0/1	0/0	0/0	0/0	0/0	0/0	0/0	0/0	0/0	0/0	0/0	0/0
Chromosome2-3721652	INS	PRECISE	211	0	0.5	0/0	0/1	0/0	0/0	0/1	0/1	0/1	0/1	0/0	1/1	0/1	0/1	0/0	0/1
Chromosome2-3722078	INS	PRECISE	61	0	0.5	0/0	0/0	0/0	0/0	0/1	0/1	0/0	0/1	0/0	1/1	0/0	0/0	0/0	0/0
Chromosome2-3722369	DEL	PRECISE	64	0	0	0/0	0/1	0/0	0/0	0/1	0/1	0/0	0/1	0/1	1/1	1/1	1/1	0/0	0/1
Chromosome2-3722382	INS	PRECISE	246	0	0	0/0	0/0	0/0	0/1	0/0	0/0	0/0	0/0	0/0	0/0	0/0	0/0	0/0	0/0
Chromosome2-3722590	INS	PRECISE	5427	0	0	0/0	0/0	1/1	0/0	0/0	0/0	0/0	0/0	0/0	0/0	0/0	0/0	0/0	0/0
Chromosome2-3723846	DEL	PRECISE	679	0	0	0/0	0/1	0/0	0/0	0/1	0/1	0/1	0/1	0/1	1/1	1/1	1/1	0/1	0/1

Chromosome2-3724720	INS	PRECISE	1518	0	0.957	0/0	0/0	0/0	0/0	0/1	0/1	0/0	0/1	0/0	0/1	0/0	0/0	0/0	0/0
Chromosome2-3725917	INS	PRECISE	5365	0	0	0/0	0/0	0/0	1/1	0/0	0/0	0/0	0/0	0/0	0/0	0/0	0/0	0/0	0/0
Chromosome2-3726051	INS	PRECISE	7876	0	0	0/0	0/0	0/0	0/0	0/0	0/0	0/0	0/0	0/0	0/0	0/0	0/0	0/0	0/1
Chromosome2-3726184	DEL	PRECISE	56	0	0	0/0	0/1	0/0	0/0	0/1	1/1	0/1	0/1	0/1	1/1	1/1	1/1	0/1	0/1
Chromosome2-3726502	DEL	PRECISE	174	0	0	0/0	0/1	0/0	0/0	0/1	1/1	0/1	0/1	0/1	1/1	1/1	1/1	0/1	0/1
Chromosome2-3727774	INS	PRECISE	12276	0	0.707	0/0	0/0	0/0	0/0	0/0	0/0	0/1	0/1	0/0	0/0	0/0	0/0	0/0	0/0
Chromosome2-3727774	BND	PRECISE	0	0	0	0/0	0/0	0/0	0/0	0/0	0/0	0/1	0/1	0/0	0/0	0/0	0/0	0/0	0/0
Chromosome2-3727807	DEL	PRECISE	180	0	0	0/0	0/1	0/0	0/1	0/1	0/1	1/1	1/1	0/1	1/1	1/1	1/1	1/1	1/1
Chromosome2-3728207	INS	PRECISE	65	0	0	0/0	0/0	0/0	0/1	0/0	0/0	0/0	0/0	0/0	0/0	0/0	0/0	0/0	0/0
Chromosome2-3728218	INS	PRECISE	1734	0	0.816	0/0	0/0	0/0	0/0	0/0	0/0	0/0	0/0	0/0	0/0	0/1	0/1	0/1	0/0
Chromosome2-3729198	INS	PRECISE	329	0	0.707	0/0	0/0	0/0	0/0	0/0	0/0	0/0	0/0	0/0	0/0	0/0	0/0	0/1	0/1
Chromosome2-3729521	DEL	PRECISE	5482	0	0	0/0	0/0	0/1	0/1	0/1	0/1	1/1	0/1	0/1	0/1	0/1	0/1	0/1	1/1
Chromosome2-3730793	INS	PRECISE	5122	0	0	0/0	0/0	0/0	1/1	0/0	0/0	0/0	0/0	0/0	0/0	0/0	0/0	0/0	0/0
Chromosome2-3731192	DEL	PRECISE	110	0	0	0/0	0/1	0/0	0/0	0/0	0/0	0/1	0/0	0/0	0/1	0/1	0/1	0/0	0/1
Chromosome2-3731451	INS	PRECISE	77	0.577	0	0/0	0/1	0/1	0/0	0/1	0/0	0/1	0/1	0/1	1/1	0/1	1/1	1/1	1/1
Chromosome2-3731475	INS	PRECISE	4233	0	0	0/0	0/0	1/1	0/0	0/0	0/0	0/0	0/0	0/0	0/0	0/0	0/0	0/0	0/0
Chromosome2-3731770	DEL	PRECISE	774	0.277	0	0/0	0/0	0/1	0/1	0/1	0/0	0/1	0/1	0/1	1/1	1/1	1/1	1/1	1/1
Chromosome2-3731816	DEL	PRECISE	56	0	0	0/0	0/0	0/0	0/0	0/1	0/0	0/0	0/0	0/0	0/0	0/0	0/0	0/0	0/0
Chromosome2-3732082	DEL	PRECISE	411	0	0	0/0	0/0	0/0	0/0	0/1	0/0	0/1	0/1	0/1	1/1	1/1	1/1	0/1	0/1
Chromosome2-3732270	INS	PRECISE	239	0	0.707	0/0	0/0	0/0	0/0	0/0	0/0	0/0	0/0	0/0	0/0	0/0	0/0	0/1	0/1
Chromosome2-3732691	INS	PRECISE	124	0	0	0/0	0/1	0/0	0/0	0/0	0/0	0/1	0/0	0/0	0/1	0/1	0/1	0/0	0/1
Chromosome2-3732908	DEL	PRECISE	469	0	0	0/0	0/1	0/0	0/0	0/1	0/1	0/1	0/1	0/1	1/1	1/1	1/1	0/1	0/1
Chromosome2-3733355	INS	PRECISE	3006	0	0	0/0	0/0	0/0	0/1	0/0	0/0	0/0	0/0	0/0	0/0	0/0	0/0	0/0	0/0
Chromosome2-3734863	BND	PRECISE	0	0	0	0/0	0/0	0/0	0/0	0/0	0/1	0/0	0/0	0/1	0/0	0/0	0/0	0/0	0/0
Chromosome2-3735350	INS	PRECISE	276	0	0	0/0	0/0	0/0	0/0	0/1	0/1	0/0	0/1	0/0	1/1	0/0	0/0	0/0	0/0
Chromosome2-3736509	DEL	PRECISE	946	0	0	0/0	0/0	0/0	0/0	0/0	0/0	0/0	0/0	0/0	0/0	1/1	0/0	0/0	0/0
Chromosome2-3736770	DEL	PRECISE	206	0	0	0/0	0/0	0/0	0/0	0/0	0/0	0/0	0/0	0/0	0/0	0/1	0/0	0/1	0/0
Chromosome2-3736875	DEL	PRECISE	75	0	0	0/0	0/0	0/0	0/0	0/1	0/0	0/0	0/1	0/1	0/1	0/1	0/1	0/1	0/0
Chromosome2-3739636	DEL	PRECISE	171	0	0	0/0	0/1	0/0	0/0	0/1	0/1	0/1	0/1	0/1	1/1	1/1	1/1	0/1	0/0
Chromosome2-3740213	DEL	PRECISE	731	0	0	0/0	0/1	0/0	0/0	0/1	0/1	0/1	0/1	0/1	1/1	1/1	1/1	0/1	0/1
Chromosome2-3740894	INS	PRECISE	59	0	0	0/0	0/0	0/0	0/1	0/0	0/0	0/0	0/0	0/0	0/0	0/0	0/0	0/0	0/0
Chromosome2-3741137	INS	PRECISE	84	0	0.408	0/0	0/1	0/0	0/0	0/0	0/0	0/1	0/0	0/0	0/1	0/1	0/0	0/0	0/1
Chromosome2-3741622	INS	PRECISE	161	0	0	0/0	0/0	0/0	0/0	0/0	0/0	0/0	0/0	0/0	0/0	1/1	0/0	0/0	0/0
Chromosome2-3741935	INS	PRECISE	701	0	0	0/0	0/0	0/0	0/0	0/0	0/0	0/0	0/0	0/0	0/0	0/0	0/0	0/0	0/1
Chromosome2-3742462	INS	PRECISE	151	0	0.577	0/0	0/0	0/0	0/0	0/0	0/0	0/0	0/0	0/1	0/0	0/0	1/1	1/1	0/0
Chromosome2-3744113	DEL	PRECISE	78	0	0	0/0	0/0	0/0	0/0	0/0	0/0	0/0	0/0	0/0	0/0	1/1	0/0	0/0	0/0
Chromosome2-3744358	DEL	PRECISE	152	0	0	0/0	0/0	0/0	0/0	0/0	0/0	0/0	0/0	0/1	1/1	1/1	1/1	0/0	0/0
Chromosome2-3744814	INS	PRECISE	658	0	0	0/0	0/0	0/0	0/0	0/0	0/0	0/0	0/0	0/1	0/0	0/0	0/0	0/0	0/0
Chromosome2-3746108	INS	PRECISE	454	0	0.707	0/0	0/0	0/0	0/0	0/0	0/1	0/0	0/0	0/1	0/0	0/0	0/0	0/0	0/0
Chromosome2-3746867	INS	PRECISE	871	0	0	0/0	0/0	0/0	0/1	0/0	0/0	0/0	0/0	0/0	0/0	0/0	0/0	0/0	0/0
Chromosome2-3748060	DEL	PRECISE	2110	0	0	0/0	0/1	0/0	0/0	0/0	0/0	0/1	0/0	0/0	0/0	0/1	0/0	0/0	0/1
Chromosome2-3749269	INS	PRECISE	295	0	0	0/0	0/0	0/0	0/0	0/0	0/0	0/0	0/0	0/0	0/0	0/0	0/0	1/1	0/0
Chromosome2-3749826	DEL	PRECISE	97	0	0	0/0	0/0	0/0	0/0	0/0	0/0	0/0	0/0	0/0	0/0	0/0	0/0	1/1	1/1
Chromosome2-3750359	DEL	PRECISE	646	0.467	0.467	0/0	0/1	0/0	0/0	1/1	0/1	1/1	0/1	0/1	0/0	0/0	0/0	0/0	1/1
Chromosome2-3751763	DEL	PRECISE	80	0	0	0/0	0/1	0/0	0/0	0/1	0/1	0/1	0/1	0/1	1/1	1/1	1/1	0/1	0/1
Chromosome2-3752452	INS	PRECISE	210	0	0.816	0/0	0/0	0/0	0/0	0/1	0/1	0/0	0/1	0/0	0/1	0/0	0/0	0/0	0/0
Chromosome2-3753352	INS	PRECISE	8078	0	0.707	0/0	0/0	0/0	0/0	0/0	1/1	0/0	0/0	1/1	0/0	0/0	0/0	0/0	0/0
Chromosome2-3753715	INS	PRECISE	77	0.289	0.577	0/0	0/1	0/0	1/1	1/1	1/1	0/1	0/1	1/1	1/1	1/1	1/1	1/1	1/1
Chromosome2-3754565	DEL	PRECISE	211	0.5	0.5	0/0	0/0	0/0	0/0	0/0	0/0	0/0	0/1	0/0	0/1	0/0	0/0	0/0	0/0
Chromosome2-3755882	DEL	PRECISE	5371	0.289	0	0/0	0/1	0/1	0/0	0/1	1/1	0/1	0/1	1/1	1/1	1/1	1/1	1/1	1/1
Chromosome2-3758631	INS	PRECISE	623	0	0.707	0/0	0/0	0/0	0/0	0/0	0/0	0/0	0/0	0/0	0/0	0/0	0/0	0/1	0/1
Chromosome2-3758990	INS	PRECISE	5589	0	0	0/0	0/0	0/0	1/1	0/0	0/0	0/0	0/0	0/0	0/0	0/0	0/0	0/0	0/0
Chromosome2-3759171	DEL	PRECISE	5399	0.555	0.277	0/0	0/1	0/1	1/1	0/0	0/0	0/0	0/0	0/0	0/0	0/0	0/0	0/0	0/0
Chromosome2-3761351	INS	PRECISE	84	0	0	0/0	0/0	0/1	0/0	0/0	0/0	0/0	0/0	0/0	0/0	0/0	0/0	0/0	0/0
Chromosome2-3764995	INS	PRECISE	636	0	0	0/0	0/0	0/0	0/0	0/0	0/0	0/0	0/0	0/0	0/0	0/0	0/0	0/1	0/1
Chromosome2-3765025	DEL	PRECISE	69	0	0	0/0	0/0	0/0	0/0	0/0	0/0	0/0	0/0	0/0	0/0	0/0	0/0	0/1	0/1
Chromosome2-3765210	DEL	PRECISE	79	0	0	0/0	0/0	0/0	0/0	0/0	0/0	0/0	0/0	0/0	0/1	0/0	0/1	0/0	0/0
Chromosome2-3765425	INS	PRECISE	130	0	0	0/0	0/0	0/0	0/0	0/0	0/1	0/0	0/0	0/1	0/0	0/0	0/0	0/0	0/0
Chromosome2-3765746	INS	PRECISE	3991	0	0	0/0	0/0	0/0	0/0	0/0	0/0	0/0	0/0	0/0	0/0	1/1	0/0	0/0	0/0
Chromosome2-3765959	INS	PRECISE	55	0	0	0/0	0/0	0/0	0/0	0/0	0/0	0/0	0/1	0/0	0/0	0/0	0/0	0/0	0/0
Chromosome2-3766498	DEL	PRECISE	169	0	0	0/0	0/1	0/0	0/0	0/1	0/1	0/1	0/1	0/0	0/1	0/1	0/0	0/1	0/1
Chromosome2-3767202	INS	PRECISE	53	0	0	0/0	0/0	0/0	0/0	0/0	0/0	0/0	0/0	0/0	0/0	0/1	0/0	0/0	0/0
Chromosome2-3767716	INS	PRECISE	5334	0	0	0/0	0/0	0/0	1/1	0/0	0/0	0/0	0/0	0/0	0/0	0/0	0/0	0/0	0/0
Chromosome2-3768498	INS	PRECISE	5037	0	0	0/0	0/0	1/1	0/0	0/0	0/0	0/0	0/0	0/0	0/0	0/0	0/0	0/0	0/0
Chromosome2-3768878	BND	PRECISE	0	0	0	0/0	0/0	0/0	0/0	0/0	0/0	0/0	0/1	0/0	1/1	0/0	0/0	0/0	0/0
Chromosome2-3770183	INS	PRECISE	768	0	0.5	0/0	0/0	0/1	0/0	1/1	0/0	1/1	1/1	0/0	0/0	0/0	0/0	0/0	0/0
Chromosome2-3770550	INS	PRECISE	66	0	0.894	0/0	0/1	0/0	0/0	0/0	0/0	0/1	0/0	0/0	0/0	1/1	0/0	1/1	1/1
Chromosome2-3770641	INS	PRECISE	457	0	0.707	0/0	0/0	0/0	0/0	0/0	0/0	0/0	0/0	0/1	0/0	0/0	0/0	0/0	0/0
Chromosome2-3770687	DEL	PRECISE	801	0	0	0/0	0/1	0/0	0/0	0/0	0/0	0/1	0/0	0/1	1/1	1/1	1/1	1/1	1/1
Chromosome2-3771104	DEL	PRECISE	5296	0	0	0/0	0/1	0/0	0/0	0/0	0/0	0/1	0/0	0/1	1/1	1/1	1/1	1/1	1/1
Chromosome2-3771720	DEL	PRECISE	157	0	0	0/0	0/0	0/0	0/0	1/1	0/0	0/0	0/0	0/0	0/0	0/0	0/0	0/0	0/0
Chromosome2-3771899	DEL	PRECISE	58	0	0	0/0	0/0	0/0	0/0	0/0	0/0	0/0	0/0	0/0	0/0	0/0	0/0	0/1	0/1
Chromosome2-3773469	DEL	PRECISE	155	0	0	0/0	0/0	0/0	0/0	0/0	0/0	0/0	0/0	0/0	0/0	1/1	0/0	1/1	0/0

Chromosome2-3773558	DEL	PRECISE	680	0	0	0/0	0/0	0/0	0/1	0/0	0/0	0/0	0/0	0/0	0/0	0/0	0/0	0/0	0/0
Chromosome2-3773691	DEL	PRECISE	227	0	0	0/0	0/0	0/0	0/0	0/0	0/0	0/0	0/0	0/0	0/0	0/0	0/0	1/1	0/0
Chromosome2-3774453	INS	PRECISE	2425	0	0	0/0	0/0	0/0	0/0	0/0	0/0	0/0	0/0	0/0	0/0	0/1	0/0	0/0	0/0
Chromosome2-3774513	INS	PRECISE	1185	0	0	0/0	0/0	0/1	0/0	0/0	0/0	0/0	0/0	0/0	0/0	0/0	0/0	0/0	0/0
Chromosome2-3774662	INS	PRECISE	279	0	0.548	0/0	0/1	0/0	0/0	0/0	0/0	0/1	0/0	0/0	0/1	0/1	0/0	0/0	0/1
Chromosome2-3776976	INS	PRECISE	6913	0	0	0/0	0/0	0/0	0/0	1/1	0/0	0/0	0/0	0/0	0/0	0/0	0/0	0/0	0/0
Chromosome2-3777669	INS	PRECISE	989	0	0	0/0	0/0	0/0	0/0	0/1	0/0	0/0	0/0	0/0	0/0	0/0	0/0	0/0	0/0
Chromosome2-3777685	DEL	PRECISE	1314	0	0	0/0	0/1	0/0	0/1	1/1	1/1	1/1	1/1	1/1	1/1	1/1	1/1	1/1	1/1
Chromosome2-3777931	INS	PRECISE	279	0	0	0/0	0/0	0/0	0/1	0/0	0/0	0/0	0/0	0/0	0/0	0/0	0/0	0/0	0/0
Chromosome2-3778043	INS	PRECISE	368	0	0.707	0/0	0/0	0/0	0/0	0/0	0/0	0/0	0/0	0/0	0/0	0/0	0/0	0/1	0/1
Chromosome2-3778059	DEL	PRECISE	144	0	0	0/0	0/0	0/1	0/0	0/1	0/1	1/1	0/0	0/1	0/0	0/0	0/0	0/1	0/1
Chromosome2-3778957	INS	PRECISE	703	0	0	0/0	0/0	0/0	0/1	0/0	0/0	0/0	0/0	0/0	0/0	0/0	0/0	0/0	0/0
Chromosome2-3779160	INS	PRECISE	201	0	0	0/0	0/0	0/0	0/0	0/0	0/0	0/0	0/0	0/0	0/0	0/0	0/0	0/1	0/1
Chromosome2-3779697	DEL	PRECISE	302	0	0	0/0	0/1	0/0	0/0	0/1	0/1	1/1	1/1	0/1	1/1	1/1	1/1	1/1	1/1
Chromosome2-3780639	INS	PRECISE	67	0	0.577	0/0	0/0	0/0	0/1	0/0	0/0	0/0	0/0	0/0	0/0	0/0	0/0	0/1	0/0
Chromosome2-3780950	DEL	PRECISE	221	0	0	0/0	0/1	0/0	0/0	0/0	0/0	0/1	0/1	0/1	0/1	1/1	1/1	1/1	1/1
Chromosome2-3782032	INS	PRECISE	151	0	0	0/0	0/0	0/0	0/0	0/0	0/0	0/0	0/0	0/0	0/0	0/0	0/0	0/0	0/1
Chromosome2-3782032	DUP	PRECISE	19501	0	0	0/0	0/1	0/0	0/0	0/0	0/0	0/0	0/0	0/0	0/0	0/1	0/0	0/0	0/1
Chromosome2-3782116	DEL	PRECISE	75	0	0	0/0	0/1	0/0	0/0	0/0	0/0	0/1	0/0	0/0	0/0	0/1	0/0	0/0	1/1
Chromosome2-3782526	INS	PRECISE	82	0	0.447	0/0	0/1	0/0	0/0	0/1	0/0	0/1	0/1	0/1	1/1	1/1	1/1	1/1	1/1
Chromosome2-3783136	INS	PRECISE	180	0	0	0/0	0/0	0/1	0/0	0/0	0/0	0/0	0/0	0/0	0/0	0/0	0/0	0/0	0/0
Chromosome2-3783179	DEL	PRECISE	13857	0	0	0/0	0/0	0/0	0/1	0/0	0/0	0/0	0/0	0/0	0/0	0/0	0/0	0/0	0/0
Chromosome2-3783931	INS	PRECISE	5245	0	0	0/0	0/0	0/1	0/0	0/0	0/0	0/0	0/0	0/0	0/0	0/0	0/0	0/0	0/0
Chromosome2-3783942	DEL	PRECISE	10795	0	0	0/0	0/0	0/0	0/0	0/1	0/0	0/0	0/1	0/0	0/0	0/0	0/0	0/0	0/0
Chromosome2-3784365	DEL	PRECISE	2482	0	0	0/0	0/0	0/0	0/0	1/1	0/0	0/0	0/0	0/0	0/0	0/0	0/0	0/0	0/0
Chromosome2-3784467	DEL	PRECISE	678	0	0	0/0	0/0	0/0	0/0	0/0	0/0	0/0	0/0	0/1	1/1	1/1	1/1	0/0	0/0
Chromosome2-3784490	INS	PRECISE	994	0	0.707	0/0	0/0	0/0	0/0	0/0	0/0	1/1	1/1	0/0	0/0	0/0	0/0	0/0	0/0
Chromosome2-3785762	DEL	PRECISE	856	0	0	0/0	0/0	0/0	0/0	1/1	0/0	0/0	0/0	0/1	1/1	0/1	0/0	0/0	0/0
Chromosome2-3786374	INS	PRECISE	56	0	0	0/0	0/1	0/0	0/0	0/0	0/0	0/0	0/0	1/1	0/0	0/1	0/0	0/0	0/0
Chromosome2-3786406	BND	PRECISE	0	0	0	0/0	0/0	0/0	0/0	0/1	0/1	0/0	0/0	0/1	0/0	0/1	0/0	0/0	0/0
Chromosome2-3786441	DEL	PRECISE	748	0	0	0/0	0/0	0/0	0/0	0/0	0/0	0/0	0/0	0/0	0/0	1/1	0/0	1/1	1/1
Chromosome2-3787340	INS	PRECISE	13084	0	0	0/0	0/0	0/1	0/0	0/0	0/0	0/0	0/0	0/0	0/0	0/0	0/0	0/0	0/0
Chromosome2-3788125	INS	PRECISE	13655	0	0	0/0	0/0	0/0	0/0	0/0	0/0	0/0	0/0	0/0	0/0	0/0	0/0	0/0	0/0
Chromosome2-3788215	DEL	PRECISE	141	0	0	0/0	0/0	0/0	0/0	0/1	0/0	0/0	0/0	0/0	0/1	0/1	0/1	0/1	0/0
Chromosome2-3788460	DEL	PRECISE	140	0.289	0.289	0/0	0/0	0/0	0/1	0/1	0/1	0/1	0/1	0/1	1/1	1/1	1/1	0/1	0/1
Chromosome2-3788910	INS	PRECISE	972	0	0	0/0	0/0	0/0	0/1	0/0	0/0	0/0	0/0	0/0	0/0	0/0	0/0	0/0	0/0
Chromosome2-3789059	DEL	PRECISE	75	0	0	0/0	0/1	0/0	0/0	0/1	0/1	0/1	0/1	0/1	1/1	1/1	1/1	0/1	0/1
Chromosome2-3789296	INS	PRECISE	1378	0	0	0/0	0/0	0/0	0/1	0/0	0/0	0/0	0/0	0/0	0/0	0/0	0/0	0/0	0/0
Chromosome2-3789331	INS	PRECISE	415	0	0	0/0	0/0	0/0	0/0	0/0	0/0	0/0	0/0	0/0	0/0	0/1	0/0	0/0	0/0
Chromosome2-3789432	INS	PRECISE	150	0	0	0/0	0/0	0/0	0/0	0/0	0/0	0/0	0/0	0/0	0/0	0/1	0/0	0/0	0/0
Chromosome2-3790583	INS	PRECISE	137	0	0	0/0	0/0	0/0	0/0	0/0	0/0	0/0	0/0	0/0	0/0	0/0	0/0	0/0	0/1
Chromosome2-3790894	INS	PRECISE	66	0	0	0/0	0/0	0/1	0/0	0/0	0/0	0/0	0/0	0/0	0/0	0/0	0/0	0/0	0/0
Chromosome2-3791118	INS	PRECISE	542	0	0.447	0/0	0/0	0/0	0/0	0/1	0/1	0/0	0/1	0/0	1/1	0/0	0/1	0/0	0/0
Chromosome2-3792102	BND	PRECISE	0	0	0	0/0	0/0	0/0	0/0	0/0	0/0	0/0	0/0	0/0	0/0	0/1	0/0	0/0	0/0
Chromosome2-3792103	BND	PRECISE	0	0	0	0/0	0/0	0/0	0/0	0/0	0/0	0/1	0/0	0/0	0/0	0/0	0/0	0/0	0/0
Chromosome2-3792454	DEL	PRECISE	74	0	0	0/0	0/0	0/0	0/0	0/1	0/0	0/1	0/1	0/0	0/0	0/0	0/0	0/1	0/1
Chromosome2-3793122	INS	PRECISE	99	0	0	0/0	0/0	0/1	0/0	0/0	1/1	0/0	0/0	0/1	0/0	0/0	0/0	0/0	0/0
Chromosome2-3793315	INS	PRECISE	1479	0	0.707	0/0	0/0	0/0	0/0	0/0	0/0	0/0	0/0	0/1	0/0	0/0	0/1	0/0	0/0
Chromosome2-3794636	INS	PRECISE	2508	0	0.707	0/0	0/0	0/0	0/0	0/0	0/0	0/0	0/0	0/1	0/0	0/0	1/1	0/0	0/0
Chromosome2-3794724	DEL	PRECISE	52	0	0	0/0	0/1	0/0	0/0	0/1	0/1	0/1	0/1	0/0	1/1	1/1	1/1	0/1	0/1
Chromosome2-3794803	INS	PRECISE	85	0	0.408	0/0	0/1	0/0	0/0	0/0	0/0	0/0	0/0	0/0	0/1	1/1	0/1	0/1	0/1
Chromosome2-3794875	INS	PRECISE	495	0	0	0/0	0/0	0/0	0/0	0/0	0/1	0/0	0/0	1/1	0/0	0/0	0/0	0/0	0/0
Chromosome2-3795610	DEL	PRECISE	54	0	0	0/0	0/0	0/0	0/0	0/0	0/0	0/0	0/0	0/0	0/1	0/0	0/1	0/0	0/0
Chromosome2-3796040	INS	PRECISE	991	0	0.577	0/0	0/0	0/0	0/0	0/1	0/0	0/1	0/1	0/0	0/0	0/0	0/0	0/0	0/0
Chromosome2-3796616	INS	PRECISE	455	0	0	0/0	0/0	0/1	0/0	0/0	0/0	0/0	0/0	0/0	0/0	0/0	0/0	0/0	0/0
Chromosome2-3796903	INS	PRECISE	62	0	0	0/0	0/0	0/0	0/0	0/1	0/0	0/1	0/1	0/0	0/0	0/0	0/0	0/1	0/0
Chromosome2-3796904	INS	PRECISE	4938	0	0.707	0/0	0/0	0/0	0/0	0/0	1/1	0/0	0/0	0/1	0/0	0/0	0/0	0/0	0/0
Chromosome2-3796976	DEL	PRECISE	444	0	0	0/0	0/0	0/0	1/1	0/1	0/0	0/1	0/1	0/0	0/0	0/0	0/0	0/0	0/0
Chromosome2-3797508	INS	PRECISE	702	0	0.983	0/0	0/0	0/0	0/0	0/1	0/1	0/1	0/1	0/0	0/1	0/1	0/0	0/0	0/0
Chromosome2-3797904	INS	PRECISE	888	0	0	0/0	0/0	0/0	0/0	0/0	0/0	0/0	0/0	0/0	0/0	0/1	0/0	0/0	0/0
Chromosome2-3797991	INS	PRECISE	13183	0	0	0/0	0/0	0/0	0/0	0/1	0/0	0/0	0/0	0/0	0/0	0/0	0/0	0/0	0/0
Chromosome2-3798538	DEL	PRECISE	379	0	0	0/0	0/0	0/0	0/0	0/0	0/0	0/0	0/0	0/0	0/0	0/0	0/0	0/1	0/1
Chromosome2-3799080	DEL	PRECISE	56	0	0	0/0	0/0	0/0	0/0	0/1	0/1	0/1	0/1	0/1	0/1	0/0	0/1	0/0	0/0
Chromosome2-3799117	INS	PRECISE	53	0	0	0/0	0/1	0/1	1/1	1/1	0/1	1/1	1/1	0/1	1/1	1/1	1/1	1/1	1/1
Chromosome2-3799927	DEL	PRECISE	95	0	0	0/0	0/0	0/0	1/1	0/0	0/0	0/0	0/0	0/0	0/0	0/0	0/0	0/0	0/0
Chromosome2-3800351	DEL	PRECISE	702	0	0	0/0	0/1	0/0	1/1	1/1	0/1	1/1	1/1	0/1	1/1	1/1	1/1	1/1	1/1
Chromosome2-3800484	DEL	PRECISE	191	0	0	0/0	0/0	0/1	0/0	0/0	0/1	0/0	0/0	0/1	0/0	0/0	0/0	0/0	0/0
Chromosome2-3801150	DEL	PRECISE	85	0	0	0/0	0/0	0/0	1/1	0/0	0/0	0/0	0/0	0/0	0/0	0/0	0/0	0/0	0/0
Chromosome2-3802741	DEL	PRECISE	56	0	0	0/0	0/0	0/0	0/0	0/1	0/0	0/0	0/0	0/0	0/0	0/0	0/0	0/0	0/0
Chromosome2-3802791	INS	PRECISE	138	0	0	0/0	0/0	0/0	0/0	0/1	0/0	0/0	0/0	0/0	0/0	0/0	0/0	0/0	0/0
Chromosome2-3802838	DEL	PRECISE	2171	0	0	0/0	0/1	0/0	1/1	0/1	0/1	0/1	0/1	0/1	1/1	1/1	1/1	1/1	1/1
Chromosome2-3803195	INS	PRECISE	189	0	0	0/0	0/0	0/0	0/0	0/0	0/0	0/0	0/0	0/0	0/0	0/1	0/0	0/0	0/0
Chromosome2-3804586	INS	PRECISE	542	0	0.837	0/0	0/0	0/0	0/0	0/1	0/1	0/1	0/1	0/0	0/1	0/0	0/0	0/0	0/0

Chromosome2-3804879	INS	PRECISE	4584	0	0	0/0	0/0	0/0	0/0	0/0	0/0	0/0	0/0	0/0	0/0	0/0	0/0	1/1	0/0
Chromosome2-3805206	INS	PRECISE	268	0	0	0/0	0/0	0/0	0/0	0/0	0/0	0/0	0/0	0/0	0/0	0/1	0/0	0/1	0/0
Chromosome2-3805927	INS	PRECISE	9173	0	0	0/0	0/0	0/0	0/0	0/0	0/0	0/0	0/0	0/0	0/0	0/0	0/0	0/0	1/1
Chromosome2-3806016	DEL	PRECISE	286	0	0	0/0	0/0	0/0	0/0	0/0	0/0	0/0	0/0	0/1	0/0	0/0	0/1	0/0	0/0
Chromosome2-3806133	INS	PRECISE	556	0	0	0/0	0/0	0/0	1/1	0/0	0/0	0/0	0/0	0/0	0/0	0/0	0/0	0/0	0/0
Chromosome2-3806432	DEL	PRECISE	67	0	0	0/0	0/0	0/0	1/1	0/0	0/0	0/0	0/0	0/0	0/0	0/0	0/0	0/0	0/0
Chromosome2-3807573	DEL	PRECISE	624	0	0	0/0	0/0	0/1	1/1	1/1	1/1	0/0	0/0	1/1	0/0	0/0	0/0	1/1	1/1
Chromosome2-3808735	DEL	PRECISE	1751	0.277	0.277	0/0	0/1	0/1	1/1	1/1	1/1	1/1	1/1	1/1	1/1	1/1	1/1	1/1	1/1
Chromosome2-3809000	DEL	PRECISE	56	0.577	0.577	0/0	0/0	0/0	0/0	0/0	0/0	0/0	0/0	0/0	0/1	0/0	0/1	0/1	0/0
Chromosome2-3810798	INS	PRECISE	337	0	0	0/0	0/0	0/0	0/0	0/0	0/0	0/0	0/0	0/0	0/0	0/0	0/0	0/1	0/0
Chromosome2-3810800	DEL	PRECISE	65	0	0	0/0	0/1	0/0	0/0	0/1	0/1	0/1	0/1	0/1	1/1	1/1	1/1	0/1	0/1
Chromosome2-3811817	DEL	PRECISE	447	0.866	0.289	0/0	0/1	0/0	1/1	0/1	0/1	0/1	0/1	0/1	1/1	1/1	1/1	1/1	1/1
Chromosome2-3812424	DEL	PRECISE	1496	0	0	0/0	0/1	0/0	0/0	0/1	0/1	0/1	0/1	0/1	1/1	1/1	1/1	1/1	1/1
Chromosome2-3813887	INS	PRECISE	357	0	0	0/0	0/0	0/0	0/0	0/0	0/0	0/0	0/0	0/0	0/0	0/1	0/0	0/0	0/0
Chromosome2-3813903	DEL	PRECISE	375	0	0	0/0	0/0	0/0	0/0	0/0	0/0	0/0	0/0	0/0	0/0	0/0	0/0	0/0	0/1
Chromosome2-3814151	INS	PRECISE	54	0	0	0/0	0/0	0/0	0/0	0/1	0/0	0/0	0/0	0/0	0/0	0/0	0/0	0/0	0/0
Chromosome2-3814269	INS	PRECISE	52	0	0	0/0	0/1	0/0	1/1	0/1	0/1	0/1	0/1	0/1	0/1	1/1	0/1	0/1	1/1
Chromosome2-3814441	INS	PRECISE	403	0	0.622	0/0	0/1	0/0	1/1	0/1	0/1	0/1	0/1	0/1	1/1	1/1	1/1	0/1	1/1
Chromosome2-3814522	INS	PRECISE	89	0	0.707	0/0	0/0	0/0	0/0	0/0	0/0	0/1	0/1	0/0	0/0	0/0	0/0	0/0	0/0
Chromosome2-3814623	INS	PRECISE	63	0	0	0/0	0/0	0/0	1/1	0/0	0/0	0/0	0/0	0/0	0/0	0/0	0/0	0/0	0/0
Chromosome2-3814919	DEL	PRECISE	218	0	0	0/0	0/0	0/0	1/1	0/0	0/0	0/0	0/0	0/0	0/0	0/0	0/0	0/0	0/0
Chromosome2-3815020	DEL	PRECISE	282	0	0	0/0	0/1	0/0	0/0	0/1	0/1	0/1	0/1	0/1	1/1	1/1	1/1	0/1	0/1
Chromosome2-3815468	INS	PRECISE	886	0	0	0/0	0/0	0/0	1/1	0/0	0/0	0/0	0/0	0/0	0/0	0/0	0/0	0/0	0/0
Chromosome2-3815801	INS	PRECISE	211	0	0.467	0/0	0/1	0/0	0/0	0/1	0/1	0/1	0/1	0/1	1/1	1/1	1/1	0/1	0/1
Chromosome2-3816023	INS	PRECISE	158	0	0	0/0	0/0	0/0	0/0	0/0	0/0	0/1	0/1	0/0	0/0	0/0	0/0	0/0	0/0
Chromosome2-3817384	DEL	PRECISE	112	0	0	0/0	0/0	0/0	0/0	0/0	0/0	0/0	0/0	0/0	0/0	0/0	0/0	0/1	0/1
Chromosome2-3818104	INS	PRECISE	796	0	0.707	0/0	0/0	0/0	0/0	0/0	0/0	0/0	0/0	0/0	0/0	0/0	0/0	0/1	0/1
Chromosome2-3818713	DEL	PRECISE	92	0	0	0/0	0/0	0/0	0/0	0/0	0/0	0/0	0/0	0/0	0/1	0/0	0/1	0/1	0/0
Chromosome2-3820092	DEL	PRECISE	810	0	0	0/0	0/0	0/1	0/0	0/0	0/0	0/0	0/0	0/1	0/0	1/1	1/1	0/0	0/0
Chromosome2-3820991	INS	PRECISE	152	0	0	0/0	0/0	0/0	0/0	0/0	0/0	0/0	0/0	0/0	0/0	0/0	0/0	0/1	0/0
Chromosome2-3821088	DEL	PRECISE	79	0	0	0/0	0/1	0/0	1/1	0/1	1/1	0/1	0/1	0/1	1/1	1/1	1/1	1/1	0/1
Chromosome2-3822238	INS	PRECISE	160	0	0	0/0	0/0	0/0	0/0	0/0	0/0	0/0	0/0	0/0	0/0	0/0	0/0	1/1	0/0
Chromosome2-3824245	DEL	PRECISE	1485	0	0	0/0	0/0	0/1	0/0	0/0	0/0	0/0	0/0	0/0	0/0	0/0	0/0	0/0	0/0
Chromosome2-3826181	INS	PRECISE	510	0	0	0/0	0/0	0/0	1/1	0/0	0/0	0/0	0/0	0/0	0/0	0/0	0/0	0/0	0/0
Chromosome2-3826312	DUP	PRECISE	1904	0	0	0/0	0/1	0/0	0/0	0/0	0/0	0/0	0/1	0/0	0/1	0/1	0/0	0/0	0/1
Chromosome2-3826375	INS	PRECISE	805	0	0	0/0	0/0	0/0	0/0	0/0	0/0	0/0	0/0	0/0	0/0	0/0	0/0	0/0	0/1
Chromosome2-3826724	DEL	PRECISE	2662	0	0	0/0	0/0	0/0	1/1	0/0	0/0	0/0	0/0	0/0	0/0	0/0	0/0	0/0	0/0
Chromosome2-3827438	INS	PRECISE	133	0	0.316	0/0	0/1	0/0	0/0	0/1	0/1	0/1	0/1	0/0	1/1	1/1	0/1	0/1	0/1
Chromosome2-3827450	DEL	PRECISE	131	0	0	0/0	0/1	0/0	0/0	0/1	0/1	0/1	0/1	0/1	1/1	1/1	1/1	0/1	0/1
Chromosome2-3828784	INS	PRECISE	369	0	0	0/0	0/0	0/0	1/1	0/0	0/0	0/0	0/0	0/0	0/0	0/0	0/0	0/0	0/0
Chromosome2-3828784	DEL	PRECISE	203	0	0	0/0	0/0	0/0	0/0	0/0	0/0	0/0	0/0	0/1	0/0	0/1	0/0	0/0	0/0
Chromosome2-3829147	DEL	PRECISE	923	0	0	0/0	0/0	0/0	0/0	0/0	0/0	0/0	0/0	0/1	1/1	1/1	1/1	0/1	0/0
Chromosome2-3829189	DEL	PRECISE	53	0	0	0/0	0/0	0/0	0/0	0/0	0/0	0/0	0/0	0/0	0/0	0/0	0/0	0/1	1/1
Chromosome2-3829809	DEL	PRECISE	1272	0	0	0/0	1/1	0/0	0/0	1/1	1/1	1/1	0/1	1/1	0/0	1/1	0/0	1/1	1/1
Chromosome2-3831356	DEL	PRECISE	125	0	0	0/0	0/0	0/0	0/0	0/0	0/0	0/0	0/0	0/0	0/1	0/0	0/1	1/1	0/0
Chromosome2-3832084	DEL	PRECISE	472	0	0	0/0	0/0	0/0	0/0	0/0	0/1	0/0	0/0	0/1	0/0	0/0	0/0	0/0	0/0
Chromosome2-3833075	DEL	PRECISE	56	0	0	0/0	0/0	0/1	0/0	0/0	0/0	0/0	0/0	0/0	0/0	0/0	0/0	0/0	0/0
Chromosome2-3834933	DEL	PRECISE	274	0	0	0/0	0/1	0/0	0/0	0/1	0/1	0/1	0/1	0/1	1/1	1/1	1/1	0/1	0/1
Chromosome2-3835392	INS	PRECISE	61	0	0	0/0	0/0	0/0	0/0	0/0	0/0	0/0	0/0	0/0	0/0	0/0	0/0	0/1	0/1
Chromosome2-3835457	DEL	PRECISE	1000	0	0	0/0	0/1	0/0	1/1	1/1	1/1	1/1	1/1	1/1	1/1	1/1	1/1	1/1	1/1
Chromosome2-3836274	DEL	PRECISE	539	0	0	0/0	0/1	0/0	1/1	1/1	1/1	1/1	1/1	1/1	1/1	1/1	1/1	1/1	1/1
Chromosome2-3837257	DEL	PRECISE	1182	0	0	0/0	0/1	0/1	1/1	1/1	1/1	1/1	1/1	1/1	1/1	1/1	1/1	1/1	1/1
Chromosome2-3838500	DEL	PRECISE	2443	0	0	0/0	0/1	0/0	0/0	1/1	1/1	1/1	1/1	1/1	1/1	1/1	1/1	1/1	1/1
Chromosome2-3840174	DEL	PRECISE	1873	0	0	0/0	0/1	0/0	0/0	0/1	0/1	0/1	0/0	0/1	1/1	1/1	1/1	0/1	0/1
Chromosome2-3841017	INS	PRECISE	482	0	0	0/0	0/0	0/0	0/0	0/0	0/0	0/0	0/0	0/0	0/0	0/0	0/0	0/1	0/0
Chromosome2-3841376	DEL	PRECISE	260	0	0	0/0	0/1	0/0	0/0	0/1	0/1	0/1	0/0	0/1	1/1	1/1	1/1	0/1	0/1
Chromosome2-3841425	INS	PRECISE	69	0	0.756	0/0	0/1	0/0	0/0	0/1	0/1	0/1	0/0	0/0	0/1	0/1	0/0	0/0	0/1
Chromosome2-3841968	INS	PRECISE	275	0	0.707	0/0	0/0	0/0	0/0	0/0	0/0	0/0	0/0	0/0	0/1	0/0	0/1	0/0	0/0
Chromosome2-3842382	INS	PRECISE	226	0	0	0/0	0/0	0/0	0/0	0/0	0/0	0/0	0/0	0/1	0/0	0/0	0/1	0/0	0/0
Chromosome2-3842406	DEL	PRECISE	65	0	0	0/0	0/0	0/0	0/0	0/0	0/0	0/0	0/0	0/0	0/0	0/1	0/0	0/0	0/0
Chromosome2-3842463	DEL	PRECISE	905	0	0	0/0	0/1	0/1	1/1	1/1	0/1	1/1	1/1	0/1	1/1	1/1	1/1	1/1	1/1
Chromosome2-3843365	DEL	PRECISE	173	0	0	0/0	0/1	0/0	0/0	0/1	0/1	0/1	0/1	0/1	1/1	1/1	1/1	0/1	0/1
Chromosome2-3843690	DEL	PRECISE	748	0	0	0/0	0/1	0/0	0/0	0/0	0/1	0/0	0/1	0/0	1/1	1/1	1/1	0/1	0/1
Chromosome2-3844085	INS	PRECISE	453	0	0.527	0/0	0/1	0/0	0/0	0/1	0/0	0/0	0/1	0/0	1/1	1/1	1/1	0/1	0/1
Chromosome2-3844211	DEL	PRECISE	131	0.316	0.316	0/0	0/1	0/0	0/0	0/1	0/0	0/0	0/1	0/0	1/1	1/1	1/1	0/1	0/1
Chromosome2-3844706	DEL	PRECISE	1208	0	0	0/0	0/0	0/0	0/1	0/0	0/0	0/0	0/0	0/0	0/0	0/0	0/0	0/0	0/0
Chromosome2-3845065	DEL	PRECISE	65	0.905	0.302	0/0	1/1	0/0	0/0	0/1	0/1	0/1	0/1	0/1	0/0	1/1	1/1	0/1	1/1
Chromosome2-3845461	INS	PRECISE	1859	0	0	0/0	0/0	0/0	0/1	0/0	0/0	0/0	0/0	0/0	0/0	0/0	0/0	0/0	0/0
Chromosome2-3846466	INS	PRECISE	358	0	0.707	0/0	0/0	0/0	0/0	0/0	0/0	0/0	0/0	0/1	0/0	0/0	0/1	0/0	0/0
Chromosome2-3847154	DEL	PRECISE	66	0	0	0/0	0/1	0/0	0/0	0/1	0/0	0/1	0/1	0/1	1/1	1/1	1/1	0/1	0/1
Chromosome2-3850147	DEL	PRECISE	211	0	0	0/0	0/1	0/0	0/1	0/1	0/1	0/0	0/1	0/0	1/1	1/1	1/1	0/0	0/0
Chromosome2-3850371	INS	PRECISE	363	0.894	0.548	0/0	0/0	0/1	0/1	0/1	0/0	0/1	0/1	0/0	0/0	0/0	0/0	0/0	0/0
Chromosome2-3850783	INS	PRECISE	5904	0	0	0/0	0/0	0/0	0/1	0/0	0/0	0/0	0/0	0/0	0/0	0/0	0/0	0/0	0/0

Chromosome2-3850958	INS	PRECISE	233	0	0	0/0	0/0	0/0	0/0	0/0	0/0	0/0	0/0	0/0	0/0	0/1	0/0	0/0	0/0
Chromosome2-3850971	DEL	PRECISE	77	0	0	0/0	0/1	0/0	0/0	0/1	0/1	0/1	0/1	0/1	1/1	1/1	1/1	1/1	1/1
Chromosome2-3851391	INS	PRECISE	1235	0	0	0/0	0/0	0/0	0/1	0/0	0/0	0/0	0/0	0/0	0/0	0/0	0/0	0/0	0/0
Chromosome2-3852435	INV	PRECISE	690	0	0	0/0	0/0	0/0	0/0	0/0	0/0	0/0	0/0	0/0	0/0	0/0	0/0	0/1	0/1
Chromosome2-3852673	DEL	PRECISE	81	0	0	0/0	0/0	0/0	0/0	0/0	0/0	0/0	0/0	0/1	0/1	0/0	1/1	0/0	0/0
Chromosome2-3854689	INS	PRECISE	739	0	0	0/0	0/0	0/0	0/1	0/0	0/0	0/0	0/0	0/0	0/0	0/0	0/0	0/0	0/0
Chromosome2-3855315	INS	PRECISE	445	0	0.707	0/0	0/0	0/0	0/0	0/0	0/0	0/0	0/0	0/0	0/0	0/0	0/0	0/1	0/1
Chromosome2-3855573	INS	PRECISE	4743	0	0	0/0	0/0	0/1	0/0	0/0	0/0	0/0	0/0	0/0	0/0	0/0	0/0	0/0	0/0
Chromosome2-3855667	DEL	PRECISE	773	0	0	0/0	0/0	0/0	0/0	0/1	0/1	0/1	0/1	0/1	1/1	1/1	1/1	0/1	0/1
Chromosome2-3855733	INS	PRECISE	193	0	0.548	0/0	0/0	0/1	0/1	0/1	0/0	0/1	0/1	0/0	0/0	0/0	0/0	0/0	0/0
Chromosome2-3855877	DEL	PRECISE	1289	0.463	0	0/0	0/0	0/0	0/0	0/1	0/1	0/0	0/0	0/0	0/0	1/1	0/0	0/0	0/0
Chromosome2-3856172	INS	PRECISE	1005	0	0	0/0	0/0	0/0	0/1	0/0	0/0	0/0	0/0	0/0	0/0	0/0	0/0	0/0	0/0
Chromosome2-3856484	INS	PRECISE	73	0	0.378	0/0	0/1	0/0	0/0	0/1	0/1	0/0	0/1	0/0	0/1	0/1	0/0	0/0	0/0
Chromosome2-3856884	INS	PRECISE	104	0	0.405	0/0	0/1	0/0	0/0	0/1	0/1	0/0	0/1	0/1	1/1	1/1	1/1	0/1	0/1
Chromosome2-3856930	BND	PRECISE	0	0	0	0/0	0/0	0/0	0/0	0/0	0/0	0/0	0/0	0/1	0/0	0/1	0/1	0/0	0/0
Chromosome2-3857140	INS	PRECISE	5070	0	0.707	0/0	0/0	0/0	0/0	0/0	0/0	0/0	0/0	0/0	0/0	0/0	0/0	1/1	1/1
Chromosome2-3857336	INS	PRECISE	197	0	0	0/0	0/0	0/0	0/1	0/0	0/0	0/0	0/0	0/0	0/0	0/0	0/0	0/0	0/0
Chromosome2-3858581	DEL	PRECISE	223	0	0	0/0	0/0	0/0	0/0	0/1	0/1	0/1	0/1	0/1	1/1	1/1	1/1	0/1	0/1
Chromosome2-3858836	INS	PRECISE	5678	0	0	0/0	0/0	1/1	0/0	0/0	0/0	0/0	0/0	0/0	0/0	0/0	0/0	0/0	0/0
Chromosome2-3859154	INS	PRECISE	86	0	0	0/0	0/0	0/0	0/0	0/0	0/0	0/0	0/0	0/0	0/0	0/0	0/0	1/1	1/1
Chromosome2-3859245	INS	PRECISE	264	0	0.707	0/0	0/0	0/0	0/0	0/0	0/0	0/0	0/0	0/0	0/0	0/0	0/0	1/1	1/1
Chromosome2-3859429	INS	PRECISE	5275	0	0	0/0	0/0	0/0	0/0	0/0	0/0	0/0	0/0	0/0	0/0	0/0	0/0	1/1	1/1
Chromosome2-3859753	DEL	PRECISE	2718	0	0	0/0	0/0	0/0	0/1	0/0	0/0	0/0	0/0	0/0	0/0	0/0	0/0	0/0	0/0
Chromosome2-3860097	BND	PRECISE	0	0	0	0/0	0/0	0/0	0/0	0/0	0/0	0/0	0/0	0/0	0/0	0/0	0/0	0/0	1/1
Chromosome2-3860259	INS	PRECISE	94	0	0.441	0/0	0/0	0/1	1/1	1/1	1/1	1/1	1/1	1/1	0/0	0/0	0/0	1/1	1/1
Chromosome2-3862421	INS	PRECISE	145	0	0.707	0/0	0/0	0/0	0/0	0/0	0/0	0/0	0/0	0/0	0/0	0/0	0/0	1/1	1/1
Chromosome2-3862888	DEL	PRECISE	80	0	0	0/0	0/0	0/0	0/0	0/0	0/0	0/0	0/0	0/1	0/0	0/0	0/0	0/0	0/0
Chromosome2-3862895	BND	PRECISE	0	0	0	0/0	0/0	0/0	0/0	1/1	0/0	0/1	1/1	0/0	0/0	0/0	0/0	0/0	0/0
Chromosome2-3862908	INS	PRECISE	64	0	0	0/0	0/0	0/0	0/0	0/0	0/0	0/0	0/0	0/1	0/0	0/0	0/0	0/0	0/0
Chromosome2-3863038	INS	PRECISE	5686	0	0	0/0	0/0	0/1	0/0	0/0	0/0	0/0	0/0	0/0	0/0	0/0	0/0	0/0	0/0
Chromosome2-3863918	DEL	PRECISE	570	0	0	0/0	0/0	0/1	0/0	0/0	0/0	0/0	0/0	0/0	0/0	0/0	0/0	0/0	0/0
Chromosome2-3863983	INS	PRECISE	996	0	0	0/0	0/0	0/0	0/0	0/0	0/0	0/0	0/0	0/0	0/0	0/0	0/0	1/1	1/1
Chromosome2-3864029	INS	PRECISE	373	0	0.667	0/0	0/0	0/0	0/0	0/0	0/0	0/1	0/1	0/1	1/1	1/1	1/1	0/0	0/0
Chromosome2-3864471	DEL	PRECISE	1124	0	0	0/0	0/0	0/1	1/1	1/1	0/0	1/1	1/1	0/0	0/0	0/0	0/0	0/0	0/0
Chromosome2-3866629	BND	PRECISE	0	0	0	0/0	0/0	0/0	0/0	0/0	0/0	0/0	0/0	0/0	0/0	0/0	0/0	0/1	0/0
Chromosome2-3866652	INS	PRECISE	2026	0	0	0/0	0/0	0/0	0/0	0/0	0/0	0/0	0/0	0/0	0/0	0/0	0/0	0/0	0/1
Chromosome2-3867381	DEL	PRECISE	65	0	0	0/0	0/1	0/0	0/0	0/1	0/1	0/1	0/1	0/0	0/1	0/1	0/0	0/0	0/0
Chromosome2-3867485	BND	PRECISE	0	0	0	0/0	0/0	0/0	0/0	0/0	0/0	0/0	0/0	0/0	0/0	0/1	0/0	0/0	0/0
Chromosome2-3867585	DEL	PRECISE	102	0	0	0/0	0/0	0/0	0/0	0/0	0/0	0/0	0/0	0/0	0/0	0/0	0/1	0/0	0/0
Chromosome2-3868065	INS	PRECISE	82	0	0.302	0/0	0/1	0/0	0/0	0/1	0/1	0/1	0/1	0/1	1/1	1/1	1/1	0/1	0/1
Chromosome2-3868088	DEL	PRECISE	132	0	0	0/0	0/0	0/0	0/0	0/0	0/0	0/0	0/0	0/1	0/1	0/1	1/1	0/1	0/1
Chromosome2-3869465	DEL	PRECISE	1612	0	0	0/0	0/1	0/1	1/1	1/1	1/1	1/1	1/1	1/1	1/1	1/1	1/1	1/1	1/1
Chromosome2-3869761	DEL	PRECISE	5854	0	0	0/0	0/1	0/0	0/0	0/1	0/1	0/1	0/0	0/1	0/1	1/1	1/1	0/0	0/0
Chromosome2-3870021	DEL	PRECISE	5922	0	0	0/0	0/0	0/0	0/1	0/0	0/0	0/0	0/0	0/0	0/0	0/0	0/0	0/0	0/0
Chromosome2-3870308	BND	PRECISE	0	0	0	0/0	0/0	0/0	0/0	0/1	0/0	0/1	0/1	0/0	0/0	0/0	0/0	0/0	0/0
Chromosome2-3870374	DEL	PRECISE	835	0	0	0/0	0/1	0/0	0/1	0/1	0/1	0/1	0/1	0/1	1/1	1/1	1/1	0/0	0/0
Chromosome2-3871831	INS	PRECISE	5651	0	0	0/0	0/0	1/1	0/0	0/0	0/0	0/0	0/0	0/0	0/0	0/0	0/0	0/0	0/0
Chromosome2-3872790	INS	PRECISE	90	0	0	0/0	0/0	0/0	0/0	0/0	0/0	0/0	0/0	0/1	0/0	1/1	1/1	0/0	0/0
Chromosome2-3873139	BND	PRECISE	0	0	0	0/0	0/0	0/0	0/0	0/0	0/0	0/1	0/0	0/0	1/1	0/0	0/0	0/0	0/0
Chromosome2-3873141	INS	PRECISE	703	0	0	0/0	0/0	0/0	0/1	0/0	0/0	0/0	0/0	0/0	0/0	0/0	0/0	0/0	0/0
Chromosome2-3873588	DEL	PRECISE	2405	0	0	0/0	0/1	0/0	0/0	0/0	0/1	0/1	0/1	0/1	1/1	1/1	1/1	0/0	0/1
Chromosome2-3874118	DEL	PRECISE	4610	0	0	0/0	0/1	0/1	0/1	1/1	0/1	1/1	1/1	0/1	1/1	1/1	0/1	1/1	1/1
Chromosome2-3875309	INS	PRECISE	211	0	0	0/0	0/0	0/0	0/1	0/0	0/0	0/0	0/0	0/0	0/0	0/0	0/0	0/0	0/0
Chromosome2-3875525	DEL	PRECISE	115	0	0	0/0	0/1	0/0	0/0	0/1	0/1	0/1	0/1	0/0	1/1	1/1	1/1	0/0	0/0
Chromosome2-3876168	DEL	PRECISE	74	0	0	0/0	0/1	0/0	0/0	0/1	0/1	0/1	0/1	0/0	0/1	0/1	0/0	0/1	0/1
Chromosome2-3877393	DEL	PRECISE	874	0.577	0.289	0/0	0/1	0/0	1/1	1/1	1/1	1/1	1/1	1/1	1/1	1/1	1/1	1/1	1/1
Chromosome2-3877914	DEL	PRECISE	179	0	0	0/0	0/0	0/0	0/0	0/0	0/0	0/0	0/0	0/0	0/0	0/1	0/0	1/1	1/1
Chromosome2-3878214	DEL	PRECISE	71	0	0	0/0	0/0	0/0	0/1	0/0	0/0	0/0	0/0	0/0	0/0	0/0	0/0	0/0	0/0
Chromosome2-3878670	DEL	PRECISE	54	0	0	0/0	0/1	0/0	0/0	0/1	0/0	0/1	0/1	0/0	1/1	1/1	0/0	0/0	0/0
Chromosome2-3879261	INS	PRECISE	4766	0	0	0/0	0/0	0/0	0/0	0/0	0/0	0/0	0/0	0/0	0/0	1/1	0/0	0/0	0/0
Chromosome2-3879261	BND	PRECISE	0	0	0	0/0	0/0	0/0	0/0	0/0	0/0	0/0	0/0	0/0	0/0	0/1	0/0	0/0	0/0
Chromosome2-3879292	INS	PRECISE	2484	0	0	0/0	0/0	0/0	0/0	0/0	1/1	0/0	0/0	0/1	0/0	0/0	0/0	0/0	0/0
Chromosome2-3879317	DEL	PRECISE	324	0	0	0/0	0/1	0/0	0/0	0/1	0/0	0/1	0/1	0/1	1/1	0/1	1/1	0/1	0/1
Chromosome2-3879437	DEL	PRECISE	58	0	0	0/0	0/1	0/0	0/0	0/1	0/0	0/1	0/1	0/1	1/1	0/1	1/1	0/1	0/0
Chromosome2-3879480	INS	PRECISE	467	0	0	0/0	0/0	0/0	0/0	0/0	1/1	0/0	0/0	0/1	0/0	0/0	0/0	0/0	0/0
Chromosome2-3879709	DEL	PRECISE	1351	0	0	0/0	1/1	0/0	0/0	1/1	0/1	1/1	1/1	1/1	1/1	0/0	0/0	1/1	0/1
Chromosome2-3880508	INS	PRECISE	211	0	0	0/0	0/0	0/0	0/1	0/0	0/1	0/0	0/0	1/1	0/0	0/0	0/0	0/0	0/0
Chromosome2-3880572	INS	PRECISE	2824	0	0	0/0	0/0	0/0	1/1	0/0	0/0	0/0	0/0	0/0	0/0	0/0	0/0	0/0	0/0
Chromosome2-3880640	DEL	PRECISE	358	0.548	0	0/0	0/0	0/0	1/1	0/0	1/1	0/0	0/0	1/1	0/0	0/0	0/0	1/1	1/1
Chromosome2-3881190	INS	PRECISE	1549	0	0.707	0/0	0/0	0/0	0/0	0/0	0/0	0/0	0/0	0/1	0/0	0/0	0/1	0/0	0/0
Chromosome2-3881260	INS	PRECISE	312	0	0	0/0	0/0	0/0	0/1	0/0	0/0	0/0	0/0	0/0	0/0	0/0	0/0	0/0	0/0
Chromosome2-3881486	INS	PRECISE	535	0	0	0/0	0/0	0/0	1/1	0/0	0/0	0/0	0/0	0/0	0/0	0/0	0/0	0/0	0/0
Chromosome2-3881545	DEL	PRECISE	775	0	0	0/0	0/0	0/0	0/1	0/0	0/0	0/0	0/0	0/0	0/0	0/0	0/0	0/1	0/1

Chromosome2-3881676	DEL	PRECISE	120	0	0	0/0	0/0	0/0	0/0	0/0	0/0	0/0	0/0	0/0	0/0	0/0	0/0	0/1	0/1
Chromosome2-3882613	DEL	PRECISE	80	0	0	0/0	0/0	0/0	0/0	0/0	0/0	0/0	0/0	0/0	0/0	0/0	0/0	0/1	0/1
Chromosome2-3883477	DEL	PRECISE	60	0	0	0/0	0/1	0/0	0/0	0/1	0/1	0/1	0/1	0/1	1/1	1/1	1/1	0/1	1/1
Chromosome2-3884917	DEL	PRECISE	850	0.982	0.405	0/0	0/1	0/0	0/0	0/1	0/1	0/1	0/1	0/1	1/1	1/1	1/1	1/1	1/1
Chromosome2-3885680	INS	PRECISE	870	0	0.707	0/0	0/0	0/0	0/0	0/0	0/0	0/0	0/0	0/1	0/0	0/0	0/1	0/0	0/0
Chromosome2-3885753	DEL	PRECISE	62	0	0	0/0	0/1	0/0	0/0	0/1	0/1	0/1	0/1	0/0	0/1	0/1	0/0	0/0	0/0
Chromosome2-3886382	DEL	PRECISE	1168	0	0	0/0	0/1	0/0	1/1	1/1	0/1	1/1	1/1	0/1	1/1	1/1	1/1	1/1	1/1
Chromosome2-3886410	INS	PRECISE	67	0	0	0/0	0/0	0/1	0/0	0/0	0/0	0/0	0/0	0/0	0/0	0/0	0/0	0/0	0/0
Chromosome2-3888122	INS	PRECISE	97	0	0.277	0/0	0/1	0/1	0/1	1/1	1/1	1/1	1/1	0/1	1/1	1/1	1/1	0/1	0/1
Chromosome2-3889017	INS	PRECISE	971	0	0.707	0/0	0/0	0/0	0/0	0/0	0/0	0/0	0/0	0/0	0/1	0/0	1/1	0/0	0/0
Chromosome2-3889477	DEL	PRECISE	88	0	0	0/0	0/0	0/1	0/0	0/0	0/0	0/0	0/0	0/0	0/0	0/0	0/0	0/0	0/0
Chromosome2-3890497	DEL	PRECISE	61	0	0	0/0	0/0	0/1	0/0	0/0	0/1	0/0	0/0	0/1	0/0	0/0	0/0	0/0	0/0
Chromosome2-3890517	INS	PRECISE	387	0	0.378	0/0	0/0	0/1	1/1	0/1	0/1	0/0	0/1	0/1	0/0	0/0	0/0	0/0	0/0
Chromosome2-3890661	DEL	PRECISE	155	0	0	0/0	0/0	0/0	0/1	0/0	0/0	0/0	0/0	0/0	0/0	0/0	0/0	0/0	0/0
Chromosome2-3892290	DEL	PRECISE	57	0	0	0/0	0/0	0/0	0/0	0/0	0/0	0/0	0/0	0/1	0/0	0/0	1/1	0/0	0/0
Chromosome2-3892436	DEL	PRECISE	200	0	0	0/0	0/1	0/0	0/0	0/1	0/1	0/1	0/1	0/1	1/1	1/1	1/1	0/1	1/1
Chromosome2-3893413	DEL	PRECISE	130	0	0	0/0	0/1	0/0	0/0	0/1	0/1	0/1	0/1	0/0	0/1	0/1	0/0	0/0	0/0
Chromosome2-3894135	DEL	PRECISE	866	0	0	0/0	0/0	0/0	0/0	0/0	0/0	0/0	0/0	0/1	0/0	0/1	0/1	0/0	0/0
Chromosome2-3894888	DEL	PRECISE	91	0	0	0/0	0/0	0/0	0/1	0/0	0/0	0/0	0/0	0/0	0/0	0/0	0/0	0/0	0/0
Chromosome2-3894987	DEL	PRECISE	59	0	0	0/0	0/1	0/0	0/0	0/1	0/1	0/1	0/1	0/0	0/1	1/1	0/0	0/0	0/0
Chromosome2-3896737	INS	PRECISE	281	0	0.707	0/0	0/0	0/0	0/0	0/0	0/0	0/0	0/0	0/0	0/0	0/0	0/0	0/1	0/1
Chromosome2-3896963	INS	PRECISE	211	0	0	0/0	0/0	0/0	0/0	0/0	0/0	0/0	0/0	0/0	0/0	0/0	0/0	0/1	0/1
Chromosome2-3897288	BND	PRECISE	0	0	0	0/0	0/0	0/0	0/0	0/0	0/0	0/0	0/0	0/0	0/0	0/0	0/1	0/0	0/0
Chromosome2-3897296	INS	PRECISE	369	0	0.568	0/0	0/1	0/0	0/0	1/1	0/1	0/1	0/0	0/0	1/1	1/1	0/1	0/1	0/1
Chromosome2-3897561	DEL	PRECISE	56	0	0	0/0	0/1	0/0	0/0	0/1	0/1	0/1	0/1	0/1	1/1	1/1	1/1	0/1	0/1
Chromosome2-3898252	INS	PRECISE	886	0	0.707	0/0	0/0	0/0	0/0	0/0	0/1	0/0	0/0	0/1	0/0	0/0	0/0	0/0	0/0
Chromosome2-3898594	INS	PRECISE	57	0	0	0/0	0/1	0/0	0/0	0/1	0/0	0/1	0/1	0/0	1/1	0/1	0/1	0/1	0/1
Chromosome2-3899270	INS	PRECISE	132	0	0	0/0	0/0	0/0	0/0	0/0	0/0	0/1	0/1	0/0	0/0	0/0	0/0	0/0	0/0
Chromosome2-3899270	INS	PRECISE	1748	0	0	0/0	0/0	0/0	0/0	0/1	0/0	0/0	0/0	0/0	0/0	0/0	0/0	0/0	0/0
Chromosome2-3899406	INS	PRECISE	685	0	0	0/0	0/0	0/0	0/0	0/1	0/0	0/0	0/0	0/0	0/0	0/0	0/0	0/0	0/0
Chromosome2-3899596	INS	PRECISE	445	0	0	0/0	0/0	0/0	0/0	0/0	0/0	0/0	0/0	0/0	0/0	0/0	0/0	1/1	1/1
Chromosome2-3899659	DEL	PRECISE	372	0	0	0/0	0/1	0/0	0/0	1/1	0/0	1/1	0/1	0/1	1/1	1/1	1/1	0/1	0/0
Chromosome2-3900337	INS	PRECISE	247	0	0	0/0	0/0	0/0	0/0	0/0	0/0	0/0	0/0	0/0	0/1	0/0	0/1	0/0	0/0
Chromosome2-3900571	INS	PRECISE	176	0	0	0/0	0/0	0/0	0/0	0/0	0/0	0/0	0/0	0/0	0/1	0/0	0/1	0/0	0/0
Chromosome2-3902390	INS	PRECISE	60	0.905	0.389	0/0	0/1	0/0	0/1	1/1	0/0	0/1	0/1	0/1	1/1	1/1	1/1	0/0	0/1
Chromosome2-3902496	INS	PRECISE	500	0	0	0/0	0/0	0/0	0/1	0/0	0/0	0/0	0/0	0/0	0/0	0/0	0/0	0/0	0/0
Chromosome2-3902552	INS	PRECISE	2408	0	0	0/0	0/0	0/0	0/1	0/0	0/0	0/0	0/0	0/0	0/0	0/0	0/0	0/0	0/0
Chromosome2-3902757	INS	PRECISE	1059	0	0	0/0	0/0	0/1	0/0	0/0	0/0	0/0	0/0	0/0	0/0	0/0	0/0	0/0	0/0
Chromosome2-3903058	INS	PRECISE	11988	0	0	0/0	0/0	0/0	0/0	0/0	0/0	0/0	0/1	0/0	0/0	0/0	0/0	0/0	0/0
Chromosome2-3903135	INS	PRECISE	6989	0	0	0/0	0/0	0/0	0/0	0/0	0/0	0/0	0/0	0/0	0/0	0/0	0/0	0/0	0/0
Chromosome2-3903413	DEL	PRECISE	67	0	0	0/0	0/0	0/0	0/0	0/0	0/0	0/0	0/0	0/1	0/0	0/0	0/1	0/0	0/0
Chromosome2-3903768	INS	PRECISE	340	0	0.535	0/0	0/1	0/0	0/0	1/1	0/1	0/1	0/1	0/0	0/1	1/1	0/0	0/0	0/0
Chromosome2-3903801	DEL	PRECISE	78	0	0	0/0	0/0	0/0	0/0	0/0	0/0	0/0	0/0	0/0	0/1	0/0	0/1	0/1	0/1
Chromosome2-3904792	INS	PRECISE	115	0	0	0/0	0/0	0/0	0/0	0/1	0/0	0/0	0/0	0/0	0/0	0/0	0/0	0/0	0/0
Chromosome2-3904907	INS	PRECISE	2348	0	0	0/0	0/0	0/0	1/1	0/0	0/0	0/0	0/0	0/0	0/0	0/0	0/0	0/0	0/0
Chromosome2-3904957	INS	PRECISE	137	0	0	0/0	0/0	0/0	0/0	0/0	1/1	0/0	0/0	0/1	0/0	0/0	0/0	0/0	0/0
Chromosome2-3904999	INS	PRECISE	931	0	0	0/0	0/0	0/0	0/0	0/1	0/0	0/0	0/0	0/0	0/0	0/0	0/0	0/0	0/0
Chromosome2-3905479	DEL	PRECISE	96	0	0	0/0	0/0	0/0	0/0	0/0	0/1	0/0	0/0	0/1	0/0	0/0	0/0	0/0	0/0
Chromosome2-3905705	BND	PRECISE	0	0	0	0/0	0/0	0/0	0/0	0/1	0/0	0/0	0/0	0/0	0/0	0/0	0/0	0/0	0/0
Chromosome2-3905734	INS	PRECISE	1105	0	0.707	0/0	0/0	0/0	0/0	0/0	0/0	0/0	0/0	0/1	0/0	0/0	0/1	0/0	0/0
Chromosome2-3905997	INS	PRECISE	246	0	0.302	0/0	0/1	0/0	0/0	1/1	0/1	0/1	0/1	0/1	1/1	1/1	1/1	0/1	0/1
Chromosome2-3906034	DEL	PRECISE	56	0	0	0/0	0/0	0/0	0/1	0/0	0/0	0/0	0/0	0/0	0/0	0/0	0/0	0/0	0/0
Chromosome2-3906082	INS	PRECISE	53	0	0	0/0	0/0	0/0	0/1	0/1	0/0	0/0	0/0	0/1	0/0	1/1	0/1	1/1	1/1
Chromosome2-3907141	INS	PRECISE	62	0	0	0/0	0/0	0/0	0/0	0/0	0/0	0/0	0/0	0/0	0/0	0/1	0/0	0/0	0/0
Chromosome2-3907188	INS	PRECISE	4908	0	0	0/0	0/0	0/0	0/0	0/0	0/0	0/0	0/0	0/0	0/0	1/1	0/0	0/0	0/0
Chromosome2-3908669	INS	PRECISE	161	0	0.707	0/0	0/0	0/0	0/0	0/0	0/0	0/0	0/0	0/0	0/0	0/0	0/0	0/1	0/1
Chromosome2-3908930	INS	PRECISE	312	0	0	0/0	0/0	0/0	0/1	0/0	0/0	0/0	0/0	0/0	0/0	0/0	0/0	0/0	0/0
Chromosome2-3909246	DEL	PRECISE	93	0	0	0/0	0/0	0/1	0/0	0/0	0/0	0/0	0/0	0/0	0/0	0/0	0/0	0/0	0/0
Chromosome2-3911678	DEL	PRECISE	258	0	0	0/0	0/0	0/0	0/0	1/1	0/0	0/0	0/0	0/0	0/1	0/1	0/1	0/0	0/0
Chromosome2-3912642	INS	PRECISE	68	0	0	0/0	0/0	0/0	0/0	0/0	0/0	0/0	0/0	0/1	0/1	0/1	1/1	0/1	1/1
Chromosome2-3913463	INS	PRECISE	66	0	0	0/0	0/0	0/0	0/0	0/0	0/0	0/0	0/0	0/0	0/0	0/0	0/0	0/1	0/1
Chromosome2-3914687	DEL	PRECISE	55	0.316	0	0/0	0/1	0/0	0/0	1/1	0/1	0/1	0/1	0/1	1/1	1/1	1/1	0/1	0/0
Chromosome2-3916233	INS	PRECISE	68	0	0	0/0	0/0	0/0	0/0	0/1	0/0	0/0	0/0	0/0	0/0	0/0	0/0	0/0	0/0
Chromosome2-3916478	INS	PRECISE	136	0	0	0/0	0/0	0/1	0/0	0/0	0/0	0/0	0/0	0/0	0/0	0/0	0/0	0/0	0/0
Chromosome2-3916701	INS	PRECISE	791	0	0	0/0	0/0	0/0	0/1	0/0	0/0	0/0	0/0	0/0	0/0	0/0	0/0	0/0	0/0
Chromosome2-3917833	INS	PRECISE	56	0	0	0/0	0/0	0/0	0/0	0/1	0/0	0/0	0/0	0/1	0/0	0/0	0/1	0/1	0/1
Chromosome2-3919097	DEL	PRECISE	51	0	0	0/0	0/0	0/1	0/0	0/0	1/1	0/1	1/1	0/1	0/0	0/0	0/0	0/0	0/0
Chromosome2-3919356	DEL	PRECISE	934	0	0	0/0	0/0	0/1	0/1	0/0	1/1	1/1	1/1	1/1	0/0	0/0	0/0	0/0	0/0
Chromosome2-3920217	INS	PRECISE	544	0	0	0/0	0/0	0/0	1/1	0/0	0/0	0/0	0/0	0/0	0/0	0/0	0/0	0/0	0/0
Chromosome2-3920358	INS	PRECISE	212	0	0	0/0	0/0	0/0	0/0	0/0	0/0	0/1	0/1	0/0	0/0	0/0	0/0	0/0	0/0
Chromosome2-3920389	DEL	PRECISE	123	0	0	0/0	0/1	0/0	0/0	0/1	0/0	0/1	0/1	0/0	0/1	0/1	0/0	0/0	0/0
Chromosome2-3920510	DEL	PRECISE	821	0	0	0/0	0/1	0/0	0/0	1/1	0/0	0/1	0/1	0/1	1/1	1/1	1/1	0/1	0/1
Chromosome2-3920720	INS	PRECISE	158	0	0.378	0/0	0/1	0/0	0/0	0/1	0/0	0/1	0/1	0/0	0/1	0/1	0/0	0/0	0/0

Chromosome2-3921291	INS	PRECISE	130	0	0	0/0	0/0	0/0	0/0	0/0	0/1	0/0	0/0	0/1	0/0	0/0	0/0	0/0	0/0
Chromosome2-3922257	INS	PRECISE	160	0	0	0/0	0/0	0/0	0/1	0/0	0/0	0/0	0/0	0/0	0/0	0/0	0/0	0/0	0/0
Chromosome2-3922659	DEL	PRECISE	67	0	0	0/0	0/0	0/0	0/1	0/0	0/0	0/0	0/0	0/0	0/0	0/0	0/0	0/0	0/0
Chromosome2-3922933	INS	PRECISE	613	0	0	0/0	0/0	0/0	0/0	0/1	0/0	0/0	0/0	0/0	0/0	0/0	0/0	0/0	0/0
Chromosome2-3924552	INS	PRECISE	428	0	0.816	0/0	0/0	0/0	0/0	0/0	0/1	0/1	0/1	0/1	0/0	0/0	0/0	0/0	0/0
Chromosome2-3924825	INS	PRECISE	761	0	0	0/0	0/0	0/0	0/0	0/1	0/0	0/0	0/0	0/0	0/0	0/0	0/0	0/0	0/0
Chromosome2-3924884	INS	PRECISE	277	0	0.535	0/0	0/1	0/0	0/0	0/1	0/1	0/1	0/1	0/0	0/1	0/1	0/0	0/0	0/0
Chromosome2-3927493	INS	PRECISE	353	0	0.831	0/0	0/1	0/0	0/0	1/1	0/1	0/1	0/1	0/1	1/1	1/1	1/1	0/1	0/1
Chromosome2-3927584	DEL	PRECISE	69	0	0	0/0	0/0	0/0	0/0	0/0	0/0	0/0	0/0	0/0	0/0	0/0	0/0	0/1	0/1
Chromosome2-3927589	INS	PRECISE	50	0	0	0/0	0/0	0/1	0/0	0/0	0/0	0/0	0/0	0/0	0/0	0/0	0/0	0/0	0/0
Chromosome2-3927694	DEL	PRECISE	152	0	0	0/0	0/0	0/0	0/0	0/1	0/0	0/0	0/0	0/0	0/0	0/0	0/0	0/0	0/0
Chromosome2-3928456	INS	PRECISE	363	0	0	0/0	0/0	0/0	0/1	0/0	0/0	0/0	0/0	0/0	0/0	0/0	0/0	0/0	0/0
Chromosome2-3928877	DEL	PRECISE	124	0	0	0/0	0/1	0/0	0/1	1/1	0/1	1/1	1/1	0/1	1/1	1/1	1/1	1/1	1/1
Chromosome2-3929604	INS	PRECISE	174	0	0	0/0	0/0	0/0	0/0	0/0	0/0	0/0	0/0	0/0	0/0	0/0	0/0	0/0	0/1
Chromosome2-3930369	INS	PRECISE	133	0	0	0/0	0/0	0/0	0/0	0/0	0/0	0/0	0/0	0/1	0/0	0/0	0/1	0/0	0/0
Chromosome2-3930578	INS	PRECISE	752	0	0.957	0/0	0/0	0/0	0/0	0/0	0/0	0/0	0/0	0/0	0/0	0/0	0/1	0/1	1/1
Chromosome2-3930980	INS	PRECISE	160	0	0.378	0/0	0/1	0/0	0/0	0/1	0/1	0/1	0/1	0/0	0/1	0/1	0/0	0/0	0/0
Chromosome2-3931482	DEL	PRECISE	259	0	0	0/0	0/1	0/0	0/0	1/1	0/1	0/1	0/1	0/1	1/1	1/1	1/1	0/1	0/1
Chromosome2-3931688	INS	PRECISE	3954	0	0	0/0	0/0	0/0	0/0	0/1	0/0	0/0	0/0	0/0	0/0	0/0	0/0	0/0	0/0
Chromosome2-3931829	INS	PRECISE	246	0	0.674	0/0	0/1	0/0	0/0	1/1	0/1	0/1	0/1	0/1	1/1	1/1	1/1	0/1	0/1
Chromosome2-3931930	INS	PRECISE	180	0	0	0/0	0/1	0/0	0/0	1/1	0/1	0/1	0/1	0/0	0/1	1/1	0/0	0/0	0/0
Chromosome2-3931965	DEL	PRECISE	139	0	0	0/0	0/1	0/0	0/0	1/1	0/1	0/1	0/1	0/0	0/1	1/1	0/0	0/0	0/0
Chromosome2-3932142	INS	PRECISE	993	0	0.707	0/0	0/0	0/0	0/0	0/0	0/0	0/1	0/1	0/0	0/0	0/0	0/0	0/0	0/0
Chromosome2-3932246	INS	PRECISE	162	0	0	0/0	0/0	0/0	0/1	0/0	0/0	0/0	0/0	0/0	0/0	0/0	0/0	0/0	0/0
Chromosome2-3932402	DEL	PRECISE	85	0	0	0/0	1/1	0/0	0/0	1/1	0/1	0/1	0/1	0/1	1/1	1/1	1/1	0/1	0/1
Chromosome2-3932878	INS	PRECISE	448	0	0	0/0	0/0	0/0	0/0	0/1	0/0	0/0	0/0	0/0	0/0	0/0	0/0	0/0	0/0
Chromosome2-3932885	DEL	PRECISE	69	0	0	0/0	0/0	0/0	0/0	0/0	0/0	0/0	0/0	0/1	0/0	0/0	0/1	0/0	0/0
Chromosome2-3933865	DEL	PRECISE	63	0	0	0/0	0/1	0/0	0/0	1/1	0/1	0/0	0/0	0/0	1/1	1/1	1/1	0/1	0/0
Chromosome2-3934097	INS	PRECISE	664	0	0	0/0	0/0	0/0	0/1	0/0	0/0	0/0	0/0	0/0	0/0	0/0	0/0	0/0	0/0
Chromosome2-3936443	DEL	PRECISE	552	0	0	0/0	0/1	0/0	0/0	0/1	0/1	0/1	0/1	0/0	0/1	0/1	0/0	0/0	0/0
Chromosome2-3936822	DEL	PRECISE	85	0	0	0/0	0/0	0/0	0/1	0/0	0/0	0/1	0/1	0/0	0/0	0/0	0/0	0/0	0/0
Chromosome2-3937150	DEL	PRECISE	995	0	0	0/0	0/0	0/0	0/0	0/1	0/1	0/0	0/1	0/0	0/1	1/1	0/0	0/0	0/0
Chromosome2-3937363	INS	PRECISE	169	0	0	0/0	0/0	0/0	0/0	0/0	0/0	0/0	0/0	0/0	0/0	1/1	0/0	0/0	0/0
Chromosome2-3937528	INS	PRECISE	1365	0	0	0/0	0/0	0/1	0/0	0/0	0/0	0/0	0/0	0/0	0/0	0/0	0/0	0/0	0/0
Chromosome2-3938106	DEL	PRECISE	87	0	0	0/0	0/0	0/0	0/0	0/0	0/0	0/0	0/0	0/0	0/0	0/0	0/0	0/1	0/1
Chromosome2-3938324	DEL	PRECISE	189	0	0	0/0	0/1	0/0	0/0	0/1	0/1	0/1	0/1	0/1	1/1	1/1	1/1	0/1	0/1
Chromosome2-3938700	DEL	PRECISE	135	0	0	0/0	0/0	0/0	0/0	0/0	0/0	0/0	0/0	0/0	0/0	0/0	0/0	1/1	0/1
Chromosome2-3939962	DEL	PRECISE	59	0	0	0/0	0/0	0/0	0/0	0/0	0/0	0/0	0/0	0/0	0/0	0/1	0/0	0/0	0/0
Chromosome2-3941561	DEL	PRECISE	1159	0	0	0/0	0/1	0/0	0/0	1/1	1/1	1/1	1/1	0/0	1/1	1/1	1/1	1/1	1/1
Chromosome2-3941998	INS	PRECISE	5758	0	0	0/0	0/0	0/0	0/1	0/0	0/0	0/0	0/0	0/0	0/0	0/0	0/0	0/0	0/0
Chromosome2-3942212	INS	PRECISE	151	0	0	0/0	0/0	0/0	0/0	0/0	0/1	0/0	0/0	0/1	0/0	0/0	0/0	0/0	0/0
Chromosome2-3942517	INS	PRECISE	184	0	0	0/0	0/0	0/0	0/0	0/0	0/0	0/0	0/0	0/1	0/0	0/0	0/1	0/0	0/0
Chromosome2-3942928	INS	PRECISE	1515	0	0.707	0/0	0/0	0/0	0/0	0/0	0/0	0/0	0/0	0/1	0/0	0/0	1/1	0/0	0/0
Chromosome2-3943058	INS	PRECISE	1370	0	0	0/0	0/0	0/0	0/0	0/0	0/0	0/0	0/0	0/1	0/0	0/0	0/1	0/0	0/0
Chromosome2-3943114	INS	PRECISE	253	0	0.874	0/0	0/1	0/0	0/0	1/1	0/1	0/1	0/1	0/1	1/1	1/1	1/1	0/1	0/1
Chromosome2-3943284	INS	PRECISE	51	0	0	0/0	0/0	0/0	0/0	0/0	0/0	0/0	0/0	0/0	0/0	0/1	0/0	0/0	0/0
Chromosome2-3943406	INS	PRECISE	623	0	0	0/0	0/0	0/0	0/0	0/0	0/0	0/1	0/0	0/0	0/0	0/0	0/0	0/0	0/0
Chromosome2-3944198	INS	PRECISE	1016	0	0	0/0	0/0	0/1	0/0	0/0	0/0	0/0	0/0	0/0	0/0	0/0	0/0	0/0	0/0
Chromosome2-3944317	INS	PRECISE	54	0	0	0/0	0/0	0/1	0/0	0/0	0/0	0/0	0/0	0/0	0/0	0/0	0/0	0/0	0/0
Chromosome2-3944408	DEL	PRECISE	208	0	0	0/0	0/0	0/1	0/0	0/0	0/0	0/0	0/0	0/0	0/0	0/0	0/0	0/0	0/0
Chromosome2-3945237	DEL	PRECISE	390	0	0	0/0	0/0	0/0	0/0	0/0	0/0	0/0	0/0	0/0	0/0	0/0	0/0	0/1	0/1
Chromosome2-3945342	INS	PRECISE	95	0	0	0/0	0/0	0/0	0/1	0/0	0/0	0/0	0/0	0/0	0/0	0/0	0/0	0/0	0/0
Chromosome2-3945960	INS	PRECISE	123	0	0.405	0/0	0/1	0/0	0/0	1/1	0/1	0/1	0/1	0/1	1/1	1/1	1/1	0/1	0/0
Chromosome2-3946782	INS	PRECISE	79	0	0	0/0	0/0	0/0	0/0	0/0	0/0	0/0	0/0	0/1	0/0	0/0	1/1	0/0	0/0
Chromosome2-3947634	INS	PRECISE	59	0	0.302	0/0	0/1	0/0	1/1	1/1	0/0	0/1	0/1	0/1	1/1	1/1	1/1	1/1	1/1
Chromosome2-3948234	DEL	PRECISE	54	0	0	0/0	0/0	0/0	0/0	0/0	0/0	0/0	0/0	0/0	0/0	0/1	0/0	0/0	0/0
Chromosome2-3950338	DEL	PRECISE	53	0	0	0/0	0/0	0/0	0/0	0/0	0/0	0/0	0/0	0/0	0/0	0/0	0/0	0/1	0/1
Chromosome2-3952813	INS	PRECISE	369	0	0	0/0	0/0	0/0	0/0	0/0	0/0	0/0	0/0	0/0	0/0	0/0	0/0	0/0	0/1
Chromosome2-3952894	BND	PRECISE	0	0	0	0/0	0/0	0/0	0/0	0/0	0/0	0/0	0/0	0/0	0/0	0/0	0/0	0/0	0/1
Chromosome2-3953016	INS	PRECISE	905	0	0.707	0/0	0/0	0/0	0/0	0/0	0/1	0/0	0/0	0/1	0/0	0/0	0/0	0/0	0/0
Chromosome2-3953027	DEL	PRECISE	86	0	0	0/0	0/0	0/0	0/0	0/0	0/0	0/0	0/0	0/0	0/0	0/1	0/0	0/0	0/0
Chromosome2-3953130	INS	PRECISE	119	0	0	0/0	0/1	0/0	0/0	1/1	0/1	0/1	0/1	0/1	1/1	1/1	1/1	0/1	0/1
Chromosome2-3954183	DEL	PRECISE	211	0	0	0/0	0/1	0/0	0/0	1/1	0/1	0/1	1/1	0/1	1/1	1/1	1/1	0/1	0/1
Chromosome2-3954547	INS	PRECISE	1352	0	0	0/0	0/0	0/0	0/1	0/0	0/0	0/0	0/0	0/0	0/0	0/0	0/0	0/0	0/0
Chromosome2-3955156	INS	PRECISE	150	0	0	0/0	0/0	0/1	0/0	0/0	0/0	0/0	0/0	0/0	0/0	0/0	0/0	0/0	0/0
Chromosome2-3956072	DEL	PRECISE	50	0	0	0/0	0/0	0/0	0/0	0/0	0/0	0/0	0/0	0/0	0/0	0/0	0/0	0/1	0/1
Chromosome2-3956263	INS	PRECISE	229	0	0	0/0	0/1	0/0	0/0	1/1	0/1	0/1	0/1	0/1	1/1	1/1	1/1	0/1	0/1
Chromosome2-3956356	INS	PRECISE	5173	0	0	0/0	0/0	0/0	1/1	0/0	0/0	0/0	0/0	0/0	0/0	0/0	0/0	0/0	0/0
Chromosome2-3956381	DEL	PRECISE	90	0.302	0.302	0/0	0/1	0/0	0/0	1/1	0/1	0/1	0/1	0/1	1/1	1/1	1/1	0/1	0/1
Chromosome2-3957873	INS	PRECISE	50	0	0	0/0	0/0	0/0	0/0	0/0	0/0	0/0	0/0	0/0	0/0	0/0	0/0	0/1	0/1
Chromosome2-3958245	INS	PRECISE	460	0	0	0/0	0/0	0/0	0/1	0/0	0/0	0/0	0/0	0/0	0/0	0/0	0/0	0/0	0/0
Chromosome2-3958412	DEL	PRECISE	94	0	0	0/0	0/0	0/0	0/1	0/0	0/0	0/0	0/0	0/0	0/0	0/0	0/0	0/0	0/0
Chromosome2-3959400	INS	PRECISE	1479	0	0	0/0	0/0	0/0	0/1	0/0	0/0	0/0	0/0	0/0	0/0	0/0	0/0	0/0	0/0

Chromosome2-3960313 INS	PRECISE	88	0	0	0/0	0/1	0/0	0/0	1/1	0/1	0/1	0/1	0/0	0/1	1/1	0/0	0/0	0/0
Chromosome2-3960862 INS	PRECISE	907	0	0.707	0/0	0/0	0/0	0/0	0/0	0/0	0/0	0/0	0/0	0/0	0/0	0/0	0/1	0/1
Chromosome2-3960866 BND	PRECISE	0	0	0	0/0	0/0	0/0	0/0	0/0	0/0	0/0	0/0	0/0	0/0	0/0	0/1	0/1	0/0
Chromosome2-3960901 INS	PRECISE	121	0	0	0/0	0/0	0/0	0/0	0/0	0/0	0/0	0/0	0/0	0/1	0/0	1/1	0/1	0/1
Chromosome2-3961757 DEL	PRECISE	139	0	0	0/0	0/1	0/0	1/1	1/1	0/1	0/1	0/1	0/1	1/1	1/1	1/1	1/1	1/1
Chromosome2-3961783 INS	PRECISE	7211	0	0	0/0	0/0	0/0	1/1	0/0	0/0	0/0	0/0	0/0	0/0	0/0	0/0	0/0	0/0
Chromosome2-3962793 INS	PRECISE	837	0	0	0/0	0/0	0/0	0/1	0/0	0/0	0/0	0/0	0/0	0/0	0/0	0/0	0/0	0/0
Chromosome2-3962882 INS	PRECISE	211	0	0	0/0	0/0	0/0	0/0	0/0	0/0	0/0	0/0	0/0	0/0	0/0	0/0	1/1	0/1
Chromosome2-3964429 INS	PRECISE	1056	0	0	0/0	0/0	0/0	0/1	0/0	0/0	0/0	0/0	0/0	0/0	0/0	0/0	0/0	0/0
Chromosome2-3964548 DEL	PRECISE	50	0	0	0/0	0/0	0/0	0/0	0/0	0/0	0/0	0/0	0/1	0/0	0/0	0/1	0/0	0/0
Chromosome2-3964816 DEL	PRECISE	1303	0	0	0/0	0/0	0/0	0/0	0/1	0/0	0/0	0/0	0/0	1/1	0/0	0/0	0/1	0/1
Chromosome2-3964949 DEL	PRECISE	759	0	0	0/0	0/0	0/0	0/0	0/1	0/0	0/0	0/0	0/0	1/1	0/0	0/0	0/1	0/1
Chromosome2-3965027 DEL	PRECISE	70	0	0	0/0	0/1	0/0	0/0	0/1	0/1	0/1	0/1	0/1	0/0	1/1	1/1	0/0	0/0
Chromosome2-3965241 DEL	PRECISE	945	0	0	0/0	0/1	0/0	0/0	0/1	0/1	0/1	0/1	0/1	0/0	0/1	0/1	0/0	0/0
Chromosome2-3965371 INS	PRECISE	807	0	0.837	0/0	0/0	0/0	0/0	0/1	0/0	0/0	0/0	0/0	0/1	0/1	0/1	0/1	0/1
Chromosome2-3965608 DEL	PRECISE	328	0	0	0/0	0/1	0/1	1/1	1/1	0/1	1/1	1/1	0/1	1/1	1/1	1/1	1/1	1/1
Chromosome2-3965817 INS	PRECISE	1347	0	0.707	0/0	0/0	0/0	0/0	0/0	0/0	0/0	0/0	0/0	0/0	0/0	0/0	0/1	0/1
Chromosome2-3965926 INS	PRECISE	114	0	0	0/0	0/0	0/0	0/0	0/0	0/1	0/0	0/0	0/1	0/0	0/0	0/0	0/0	0/0
Chromosome2-3966225 DEL	PRECISE	427	0	0	0/0	0/1	0/0	0/0	1/1	0/1	0/1	0/1	0/1	1/1	1/1	1/1	0/1	0/1
Chromosome2-3968112 DEL	PRECISE	63	0	0	0/0	0/0	0/0	0/0	0/0	1/1	1/1	1/1	1/1	0/0	0/0	0/0	0/0	0/0
Chromosome2-3970518 DEL	PRECISE	369	0	0	0/0	0/1	0/0	0/0	1/1	0/1	0/1	0/1	0/0	1/1	1/1	0/0	0/0	0/0
Chromosome2-3970649 INS	PRECISE	304	0	0.707	0/0	0/0	0/0	0/0	0/0	0/0	0/0	0/0	0/0	0/0	0/0	0/0	0/1	0/1
Chromosome2-3970974 INS	PRECISE	163	0	0.894	0/0	0/0	0/0	0/0	0/1	0/0	0/0	0/0	0/0	0/1	0/0	0/1	0/1	0/1
Chromosome2-3971202 INS	PRECISE	165	0	0.488	0/0	0/1	0/0	0/0	0/1	0/1	0/1	0/1	0/0	0/1	0/1	0/0	0/0	0/0
Chromosome2-3972037 INS	PRECISE	373	0	0.577	0/0	0/0	0/0	0/0	0/1	0/0	0/0	0/0	0/1	0/0	0/1	0/1	0/0	0/0
Chromosome2-3972053 DEL	PRECISE	72	0	0	0/0	0/0	0/0	0/0	0/1	0/0	0/0	0/0	0/1	0/1	0/1	1/1	0/1	0/1
Chromosome2-3972402 INS	PRECISE	1196	0	0	0/0	0/0	0/0	0/0	0/0	0/0	0/0	0/0	0/0	0/0	0/1	0/0	0/0	0/0
Chromosome2-3972456 DEL	PRECISE	127	0	0	0/0	0/1	0/0	0/0	1/1	1/1	0/1	0/1	0/1	1/1	1/1	1/1	0/1	0/1
Chromosome2-3972895 INS	PRECISE	1555	0	0	0/0	0/0	0/0	0/1	0/0	0/0	0/0	0/0	0/0	0/0	0/0	0/0	0/0	0/0
Chromosome2-3973117 DEL	PRECISE	289	0	0	0/0	0/0	0/0	0/0	0/1	0/0	0/0	0/0	0/0	1/1	0/1	0/1	0/1	0/1
Chromosome2-3973245 INS	PRECISE	322	0	0.488	0/0	0/1	0/0	0/0	0/1	0/1	0/1	0/1	0/0	0/1	0/1	0/0	0/0	0/0
Chromosome2-3973381 INS	PRECISE	57	0.302	0	0/0	0/1	0/0	0/0	1/1	0/1	0/1	0/1	0/1	1/1	1/1	1/1	0/1	0/1
Chromosome2-3974783 INS	PRECISE	6027	0	0	0/0	0/0	0/0	1/1	0/0	0/0	0/0	0/0	0/0	0/0	0/0	0/0	0/0	0/0
Chromosome2-3975000 DEL	PRECISE	81	0	0	0/0	0/0	0/0	0/0	0/0	0/0	0/0	0/0	0/0	0/0	0/0	0/0	0/1	0/1
Chromosome2-3976475 INS	PRECISE	55	0	0	0/0	0/1	0/0	0/0	1/1	0/1	0/1	0/1	0/1	1/1	1/1	1/1	1/1	1/1
Chromosome2-3976704 INS	PRECISE	641	0	0	0/0	0/0	0/0	0/1	0/0	0/0	0/0	0/0	0/0	0/0	0/0	0/0	0/0	0/0
Chromosome2-3976706 BND	PRECISE	0	0	0	0/0	0/0	0/0	0/0	0/1	0/0	0/0	0/0	0/0	0/0	0/0	0/1	0/0	0/0
Chromosome2-3976940 DEL	PRECISE	226	0.505	0.505	0/0	0/1	0/0	0/0	1/1	0/1	0/1	0/1	0/1	1/1	1/1	1/1	0/1	0/1
Chromosome2-3977231 INS	PRECISE	67	0	0	0/0	0/1	0/0	0/0	1/1	0/1	0/1	0/1	0/1	1/1	1/1	1/1	0/1	0/1
Chromosome2-3977325 INS	PRECISE	150	0	0	0/0	0/0	0/0	0/0	0/0	0/0	0/0	0/0	0/0	0/0	0/0	0/0	0/1	0/0
Chromosome2-3977345 DEL	PRECISE	70	0	0	0/0	0/0	0/0	0/0	0/0	0/0	0/0	0/0	0/0	0/0	0/0	0/0	0/1	0/1
Chromosome2-3977992 INS	PRECISE	813	0	0.707	0/0	0/0	0/0	0/0	0/0	0/1	0/0	0/0	0/1	0/0	0/0	0/0	0/0	0/0
Chromosome2-3978709 INS	PRECISE	2483	0	0	0/0	0/0	0/0	0/0	0/0	0/0	0/0	0/0	0/0	0/0	0/0	0/0	0/0	0/1
Chromosome2-3979143 INS	PRECISE	326	0	0.707	0/0	0/0	0/0	0/0	0/0	0/0	0/0	0/0	0/1	0/0	0/0	0/1	0/0	0/0
Chromosome2-3979578 DEL	PRECISE	109	0	0	0/0	0/0	0/0	0/1	0/0	0/0	0/0	0/0	0/0	0/0	0/0	0/0	0/0	0/0
Chromosome2-3979912 DEL	PRECISE	72	0	0	0/0	0/1	0/0	0/0	1/1	0/0	0/1	0/0	0/1	1/1	1/1	1/1	1/1	0/1
Chromosome2-3980005 DEL	PRECISE	1037	0	0	0/0	0/1	0/0	0/0	1/1	0/1	1/1	0/1	0/0	0/0	1/1	0/0	1/1	1/1
Chromosome2-3981040 DEL	PRECISE	91	0	0	0/0	0/0	0/0	0/0	0/0	0/0	0/0	0/0	0/0	0/0	0/1	0/0	0/0	0/0
Chromosome2-3981104 INS	PRECISE	415	0	0.577	0/0	0/1	0/0	0/0	0/1	0/1	0/1	0/1	0/0	0/1	0/1	0/0	0/0	0/0
Chromosome2-3981409 INS	PRECISE	210	0	0.447	0/0	0/0	0/0	0/0	0/1	0/0	0/0	0/0	0/1	0/0	0/0	0/1	0/1	0/1
Chromosome2-3981447 DEL	PRECISE	77	0	0	0/0	0/0	0/0	0/0	0/0	0/0	0/0	0/0	0/0	0/1	0/0	0/1	0/0	0/0
Chromosome2-3981951 BND	PRECISE	0	0	0	0/0	0/0	0/0	0/0	0/1	0/0	0/0	0/0	0/0	0/1	0/1	0/1	0/1	0/1
Chromosome2-3982334 INS	PRECISE	1460	0	0	0/0	0/0	0/0	0/0	0/0	0/0	0/0	0/0	0/0	0/0	0/0	0/1	0/0	0/0
Chromosome2-3982526 INS	PRECISE	460	0	0.577	0/0	0/0	0/0	0/1	0/0	0/0	0/0	0/0	0/0	0/0	0/0	0/0	0/1	0/1
Chromosome2-3982845 DEL	PRECISE	3460	0	0	0/0	0/0	0/0	0/0	0/1	0/0	0/0	0/0	0/1	0/1	0/1	0/1	0/1	0/1
Chromosome2-3983106 DEL	PRECISE	109	0	0	0/0	0/1	0/0	0/0	0/1	0/1	0/1	1/1	0/1	0/1	0/1	0/1	0/1	0/1
Chromosome2-3984057 INS	PRECISE	10604	0	0	0/0	0/0	1/1	0/0	0/0	0/0	0/0	0/0	0/0	0/0	0/0	0/0	0/0	0/0
Chromosome2-3984130 DEL	PRECISE	5337	0	0	0/0	0/0	0/0	0/1	0/0	0/0	0/0	0/0	0/0	0/0	0/0	0/0	0/0	0/0
Chromosome2-3984325 DEL	PRECISE	555	0	0	0/0	0/0	0/0	0/0	0/0	0/0	0/0	0/0	0/0	0/0	0/1	0/0	0/0	0/0
Chromosome2-3984640 DEL	PRECISE	69	0	0	0/0	0/1	0/0	0/0	1/1	0/1	0/1	0/1	0/1	1/1	1/1	1/1	0/1	0/1
Chromosome2-3985086 INS	PRECISE	72	0	0	0/0	0/0	0/0	0/1	0/0	0/0	0/0	0/0	0/0	0/0	0/0	0/0	0/0	0/0
Chromosome2-3985197 INS	PRECISE	203	0	0	0/0	0/0	0/0	0/0	0/1	0/0	0/0	0/0	0/0	0/0	0/0	0/0	0/0	0/0
Chromosome2-3985254 DEL	PRECISE	1847	0	0	0/0	0/0	0/0	0/1	1/1	0/1	1/1	1/1	0/0	0/1	0/1	0/1	0/1	0/1
Chromosome2-3985401 INS	PRECISE	1098	0	0.707	0/0	0/0	0/0	0/0	0/0	0/0	0/0	0/0	0/0	0/0	0/0	0/0	0/1	0/1
Chromosome2-3985884 DEL	PRECISE	76	0.5	0.5	0/0	0/0	0/0	0/0	0/0	0/0	0/0	0/0	0/1	0/0	0/0	1/1	1/1	1/1
Chromosome2-3986292 DEL	PRECISE	996	0	0	0/0	0/0	0/0	0/0	0/0	0/0	0/0	0/0	0/1	0/0	0/0	1/1	1/1	1/1
Chromosome2-3986913 DEL	PRECISE	908	0	0	0/0	0/0	0/0	0/0	1/1	0/0	1/1	0/0	0/0	0/0	0/0	0/0	0/0	0/0
Chromosome2-3986974 BND	PRECISE	0	0	0	0/0	0/0	0/1	0/1	0/0	0/0	0/0	0/0	0/0	0/0	0/0	0/0	0/0	0/0
Chromosome2-3987226 DEL	PRECISE	58	0	0	0/0	0/0	0/0	0/0	0/0	0/0	0/0	0/0	0/0	0/0	0/0	0/0	0/1	0/1
Chromosome2-3988226 INS	PRECISE	879	0	0.707	0/0	0/0	0/0	0/0	0/0	0/0	0/1	1/1	0/0	0/0	0/0	0/0	0/0	0/0
Chromosome2-3988615 DEL	PRECISE	58	0	0	0/0	0/0	0/0	0/0	0/0	0/0	0/0	0/0	0/0	1/1	1/1	1/1	0/0	0/1
Chromosome2-3988851 DEL	PRECISE	218	0	0	0/0	0/0	0/0	0/0	0/0	0/0	0/0	0/0	0/0	1/1	1/1	1/1	0/0	0/1
Chromosome2-3988928 INS	PRECISE	59	0	0.447	0/0	0/0	0/0	0/0	0/0	0/0	0/0	0/0	0/0	1/1	1/1	1/1	0/0	0/1

Chromosome2-3989040	INS	PRECISE	1078	0	0	0/0	0/0	0/0	0/1	0/0	0/0	0/0	0/0	0/0	0/0	0/0	0/0	0/0	0/0
Chromosome2-3989342	INS	PRECISE	3660	0	0	0/0	0/0	0/0	1/1	0/0	0/0	0/0	0/0	0/0	0/0	0/0	0/0	0/0	0/0
Chromosome2-3989842	INS	PRECISE	809	0	0.707	0/0	0/0	0/0	0/0	0/0	0/0	1/1	0/1	0/0	0/0	0/0	0/0	0/0	0/0
Chromosome2-3989959	DEL	PRECISE	2323	0	0	0/0	0/1	0/0	0/0	1/1	0/0	0/0	0/1	0/1	1/1	1/1	1/1	0/1	0/1
Chromosome2-3990137	INS	PRECISE	475	0	0	0/0	0/0	0/0	0/0	0/0	0/0	0/0	0/0	0/0	0/0	0/0	0/0	0/1	0/1
Chromosome2-3990194	DEL	PRECISE	4764	0	0	0/0	0/1	0/0	0/0	1/1	0/0	0/0	0/1	0/1	1/1	1/1	1/1	0/1	0/1
Chromosome2-3990329	BND	PRECISE	0	0	0	0/0	0/0	0/0	0/0	0/0	0/0	0/1	0/0	0/0	0/0	0/0	0/0	0/0	0/0
Chromosome2-3992644	DEL	PRECISE	160	0	0	0/0	0/0	0/0	0/0	0/1	0/0	0/0	0/0	0/0	0/0	0/0	0/0	0/0	0/0
Chromosome2-3992996	DEL	PRECISE	426	0	0	0/0	0/1	0/0	0/0	1/1	0/1	1/1	1/1	0/1	1/1	1/1	1/1	0/0	0/0
Chromosome2-3993287	INS	PRECISE	199	0	0.316	0/0	0/1	0/0	0/0	1/1	0/1	0/1	0/1	0/1	1/1	1/1	1/1	0/0	0/0
Chromosome2-3993920	INS	PRECISE	132	0	0	0/0	0/0	0/0	0/1	0/0	0/0	0/0	0/0	0/0	0/0	0/0	0/0	0/0	0/0
Chromosome2-3994147	INS	PRECISE	137	0	0	0/0	0/0	0/0	0/0	0/0	0/0	0/1	0/1	0/0	0/0	0/0	0/0	0/0	0/0
Chromosome2-3994423	DEL	PRECISE	53	0	0	0/0	0/0	0/1	0/1	0/0	0/0	1/1	0/1	0/0	0/0	0/0	0/0	0/0	0/0
Chromosome2-3995083	DEL	PRECISE	583	0	0	0/0	0/0	0/0	0/0	0/0	0/0	0/0	0/0	0/0	0/0	0/0	0/0	1/1	0/0
Chromosome2-3996605	DEL	PRECISE	152	0	0	0/0	0/0	0/0	0/0	0/0	0/0	0/0	0/0	0/1	0/1	0/1	1/1	0/1	0/1
Chromosome2-3997084	INS	PRECISE	322	0	0.603	0/0	0/1	0/0	0/0	1/1	0/1	0/0	0/1	0/1	1/1	1/1	1/1	1/1	1/1
Chromosome2-3997851	DEL	PRECISE	450	0	0	0/0	0/1	0/0	0/0	1/1	0/1	0/1	0/1	0/1	1/1	1/1	1/1	0/1	0/1
Chromosome2-3998440	DEL	PRECISE	361	0	0	0/0	0/1	0/0	0/0	1/1	0/1	0/1	0/1	0/1	1/1	1/1	1/1	0/1	0/1
Chromosome2-3999167	INS	PRECISE	726	0	0	0/0	0/0	0/0	0/1	0/0	0/0	0/0	0/0	0/0	0/0	0/0	0/0	0/0	0/0
Chromosome2-3999254	DEL	PRECISE	2347	0	0	0/0	0/0	0/0	0/0	0/1	0/0	0/0	0/0	0/0	0/0	0/0	0/0	0/0	0/0
Chromosome2-3999544	INS	PRECISE	136	0	0	0/0	0/0	0/0	0/0	0/0	0/0	0/0	0/0	0/0	0/1	0/0	0/1	0/0	0/0
Chromosome2-3999552	DEL	PRECISE	137	0	0	0/0	0/1	0/0	0/0	1/1	1/1	0/1	0/1	0/1	1/1	1/1	1/1	0/1	0/1
Chromosome2-3999877	DEL	PRECISE	868	0	0	0/0	0/0	0/1	1/1	1/1	1/1	1/1	1/1	0/0	1/1	1/1	0/0	1/1	1/1
Chromosome2-4000275	INS	PRECISE	138	0	0	0/0	0/0	0/0	0/1	0/0	0/0	0/0	0/0	0/0	0/0	0/0	0/0	0/0	0/0
Chromosome2-4000805	DEL	PRECISE	1825	0.949	0.949	0/0	1/1	0/0	0/0	0/0	1/1	1/1	0/1	0/0	0/0	1/1	1/1	1/1	1/1
Chromosome2-4001244	DEL	PRECISE	369	0	0	0/0	0/1	0/0	0/0	1/1	1/1	0/1	0/1	0/0	1/1	0/1	0/1	0/1	0/1
Chromosome2-4001308	INS	PRECISE	56	0	0.707	0/0	0/0	0/0	0/0	0/0	0/0	0/0	0/0	0/1	0/0	0/0	0/1	0/0	0/0
Chromosome2-4001966	DEL	PRECISE	73	0	0	0/0	0/0	0/0	0/0	0/0	0/0	0/0	0/0	0/0	0/0	0/1	0/0	0/0	0/0
Chromosome2-4002161	DEL	PRECISE	66	0	0	0/0	0/0	0/0	0/1	0/0	0/0	0/0	0/0	0/0	0/0	0/0	0/0	0/0	0/0
Chromosome2-4002852	INS	PRECISE	59	0	0	0/0	0/0	0/0	0/0	0/0	0/0	0/1	0/1	0/0	0/0	0/0	0/0	0/0	0/0
Chromosome2-4003018	INS	PRECISE	50	0	0	0/0	0/0	0/0	0/1	0/0	0/0	0/0	0/0	0/0	0/0	0/0	0/0	0/0	0/0
Chromosome2-4003058	DEL	PRECISE	152	0	0	0/0	0/1	0/0	0/0	1/1	0/1	0/1	0/1	0/0	0/1	1/1	0/0	0/0	0/0
Chromosome2-4003124	DEL	PRECISE	842	0	0	0/0	0/1	0/0	0/0	1/1	0/1	0/1	0/1	0/1	1/1	1/1	1/1	0/1	0/1
Chromosome2-4003276	DEL	PRECISE	200	0	0	0/0	0/1	0/0	0/1	1/1	1/1	0/1	0/1	0/1	1/1	1/1	1/1	1/1	1/1
Chromosome2-4004509	DEL	PRECISE	91	0	0	0/0	0/1	0/0	0/0	1/1	0/1	0/1	0/1	0/1	1/1	1/1	1/1	0/1	0/1
Chromosome2-4004687	DEL	PRECISE	70	0	0	0/0	0/1	0/0	0/1	1/1	1/1	1/1	1/1	1/1	1/1	1/1	1/1	0/1	0/1
Chromosome2-4004768	INS	PRECISE	997	0	0.577	0/0	0/0	0/0	0/0	0/1	0/0	0/0	0/0	0/0	0/0	0/1	0/0	0/1	0/1
Chromosome2-4005403	DEL	PRECISE	102	0	0	0/0	0/0	0/0	0/1	0/0	0/0	0/0	0/0	0/0	0/0	0/0	0/0	0/0	0/0
Chromosome2-4005561	DEL	PRECISE	61	0	0	0/0	0/0	0/0	0/0	0/0	0/0	0/0	0/0	0/0	0/0	0/0	0/0	0/1	0/1
Chromosome2-4005921	INS	PRECISE	445	0	0.707	0/0	0/0	0/0	0/0	0/0	0/0	1/1	1/1	0/0	0/0	0/0	0/0	0/0	0/0
Chromosome2-4006000	DEL	PRECISE	149	0	0	0/0	0/0	0/0	0/0	1/1	0/0	0/0	0/0	0/0	1/1	1/1	0/0	1/1	1/1
Chromosome2-4006333	INS	PRECISE	89	0	0.452	0/0	0/1	0/0	0/1	1/1	0/1	1/1	1/1	0/1	1/1	1/1	1/1	1/1	1/1
Chromosome2-4007991	INS	PRECISE	995	0	0	0/0	0/0	0/0	0/1	0/0	0/0	0/0	0/0	0/0	0/0	0/0	0/0	0/0	0/0
Chromosome2-4008009	INS	PRECISE	137	0	0.707	0/0	0/0	0/0	0/0	0/0	0/0	0/1	0/1	0/0	0/0	0/0	0/0	0/0	0/0
Chromosome2-4008041	DEL	PRECISE	59	0	0	0/0	0/0	0/0	0/0	0/0	0/0	0/0	0/0	0/0	0/1	0/0	0/0	0/0	0/0
Chromosome2-4008289	INS	PRECISE	480	0	0	0/0	0/0	0/0	0/1	0/0	0/0	0/0	0/0	0/0	0/0	0/0	0/0	1/1	1/1
Chromosome2-4008300	BND	PRECISE	0	0	0	0/0	0/0	0/0	0/0	0/0	0/0	0/0	0/0	0/0	0/1	0/0	0/1	0/0	0/0
Chromosome2-4008958	DEL	PRECISE	485	0	0	0/0	0/0	0/1	0/0	0/0	0/0	0/0	0/0	0/0	0/0	0/0	0/0	0/0	0/0
Chromosome2-4009748	INS	PRECISE	3812	0	0	0/0	0/0	1/1	0/0	0/0	0/0	0/0	0/0	0/0	0/0	0/0	0/0	0/0	0/0
Chromosome2-4010099	INS	PRECISE	781	0	0	0/0	0/0	0/0	0/0	0/1	0/0	0/0	0/0	0/0	0/0	0/0	0/0	0/0	0/0
Chromosome2-4010150	INS	PRECISE	69	0	0.378	0/0	0/1	0/0	0/0	1/1	0/1	0/1	0/1	0/0	0/1	1/1	0/0	0/0	0/0
Chromosome2-4010955	INS	PRECISE	137	0	0	0/0	0/0	0/0	0/0	0/1	0/0	0/0	0/0	0/0	0/0	0/0	0/0	0/0	0/0
Chromosome2-4011020	BND	PRECISE	0	0	0	0/0	0/0	0/0	0/0	0/0	0/0	0/0	0/0	0/0	0/0	0/0	0/0	0/1	1/1
Chromosome2-4011113	INS	PRECISE	69	0	0	0/0	0/0	0/0	0/0	0/0	0/0	0/0	0/0	0/0	0/0	0/0	0/0	1/1	1/1
Chromosome2-4011221	INS	PRECISE	115	0	0	0/0	0/0	0/0	0/0	0/1	0/0	0/0	0/0	0/0	0/0	0/0	0/0	0/0	0/0
Chromosome2-4011513	BND	PRECISE	0	0	0	0/0	0/0	0/1	0/1	0/0	0/0	0/1	0/1	0/1	0/0	0/0	0/0	0/0	0/0
Chromosome2-4011513	BND	PRECISE	0	0	0	0/0	0/0	0/0	0/1	0/0	0/0	0/0	0/0	0/0	0/0	0/0	0/0	0/0	0/0
Chromosome2-4011805	INS	PRECISE	714	0	0	0/0	0/0	0/0	1/1	0/0	0/0	0/0	0/0	0/0	0/0	0/0	0/0	0/0	0/0
Chromosome2-4012180	DEL	PRECISE	443	0.302	0.302	0/0	0/1	0/0	0/0	1/1	0/1	0/1	0/1	0/1	1/1	1/1	1/1	0/0	0/1
Chromosome2-4012390	DEL	PRECISE	217	0	0	0/0	0/1	0/0	0/0	1/1	0/1	0/1	0/1	0/1	1/1	1/1	1/1	0/1	0/1
Chromosome2-4012833	DEL	PRECISE	128	0	0	0/0	0/1	0/0	0/0	1/1	0/1	0/1	0/1	0/1	0/1	1/1	0/1	0/1	0/1
Chromosome2-4013336	INS	PRECISE	146	0	0.647	0/0	0/1	0/0	0/0	1/1	0/1	0/1	0/1	0/1	1/1	1/1	1/1	0/1	0/1
Chromosome2-4013369	INS	PRECISE	4382	0	0	0/0	0/0	0/0	0/0	0/1	0/0	0/0	0/0	0/0	0/0	0/0	0/0	0/0	0/0
Chromosome2-4013686	INS	PRECISE	929	0	0	0/0	0/0	0/0	0/1	0/0	0/0	0/0	0/0	0/0	0/0	0/0	0/0	0/0	0/0
Chromosome2-4013962	INS	PRECISE	149	0	0	0/0	0/0	0/0	0/0	0/0	0/0	0/0	0/0	0/1	0/0	0/0	0/1	0/0	0/0
Chromosome2-4014073	INS	PRECISE	999	0	0	0/0	0/0	0/0	0/0	0/0	0/0	0/0	0/0	0/0	0/0	0/0	0/0	0/1	0/0
Chromosome2-4014558	DEL	PRECISE	744	0	0	0/0	0/0	0/1	0/1	0/0	0/1	0/0	0/0	0/1	0/0	0/0	0/0	0/0	0/0
Chromosome2-4014664	INS	PRECISE	150	0	0.577	0/0	0/0	0/1	0/0	0/0	0/1	0/0	0/0	0/1	0/0	0/0	0/0	0/0	0/0
Chromosome2-4015584	INS	PRECISE	6992	0	0	0/0	0/0	0/0	0/0	0/0	0/0	0/0	0/0	0/0	0/0	1/1	0/0	0/0	0/0
Chromosome2-4016815	INS	PRECISE	132	0	0	0/0	0/0	0/0	0/0	0/0	0/0	0/1	0/1	0/0	0/0	0/0	0/0	0/0	0/0
Chromosome2-4017035	INS	PRECISE	5875	0	0	0/0	0/0	1/1	0/0	0/0	0/0	0/0	0/0	0/0	0/0	0/0	0/0	0/0	0/0
Chromosome2-4017305	DEL	PRECISE	76	0	0	0/0	0/1	0/0	0/0	1/1	0/1	0/1	0/1	0/1	1/1	1/1	1/1	0/1	0/1
Chromosome2-4017437	DEL	PRECISE	106	0	0	0/0	0/0	0/0	0/0	0/0	0/0	0/0	0/0	0/0	0/0	0/0	0/0	0/1	0/1

Chromosome2-4017863	DEL	PRECISE	99	0	0	0/0	0/0	0/0	0/0	0/0	0/1	0/0	0/0	0/1	0/0	0/0	0/0	0/1	0/1
Chromosome2-4018063	INS	PRECISE	5052	0	0	0/0	0/0	0/0	0/0	0/0	0/0	0/0	0/0	0/0	0/0	0/0	0/0	0/0	1/1
Chromosome2-4018094	DEL	PRECISE	118	0	0	0/0	0/0	0/0	0/1	0/0	0/0	0/1	0/1	0/0	0/0	0/0	0/0	0/0	0/0
Chromosome2-4018489	INS	PRECISE	98	0	0	0/0	0/0	0/0	0/0	0/0	0/0	0/0	0/0	0/0	0/0	0/0	0/0	0/0	0/1
Chromosome2-4018840	INS	PRECISE	3521	0	0	0/0	0/0	0/0	0/0	0/0	0/0	0/0	0/0	0/0	0/0	0/0	0/0	1/1	0/1
Chromosome2-4019101	INS	PRECISE	738	0	0.996	0/0	0/1	0/0	0/1	1/1	0/1	0/1	0/1	0/1	1/1	1/1	1/1	1/1	1/1
Chromosome2-4019324	INS	PRECISE	264	0	0.488	0/0	0/1	0/0	0/0	0/1	0/1	0/1	0/1	0/0	0/1	0/1	0/0	0/0	0/0
Chromosome2-4020025	DEL	PRECISE	58	0	0	0/0	0/1	0/0	0/0	1/1	0/1	0/1	0/1	0/1	1/1	1/1	1/1	0/0	0/0
Chromosome2-4021610	DEL	PRECISE	342	0	0	0/0	0/0	0/0	0/0	0/0	0/0	0/0	0/0	0/0	0/0	0/0	0/0	1/1	1/1
Chromosome2-4021723	INS	PRECISE	14131	0	0	0/0	0/0	0/0	0/0	0/0	0/0	0/0	0/0	0/1	0/0	0/0	0/0	0/0	0/0
Chromosome2-4021832	DEL	PRECISE	601	0	0	0/0	0/0	0/0	0/0	1/1	1/1	0/1	0/1	0/1	1/1	1/1	1/1	0/1	0/1
Chromosome2-4021895	DEL	PRECISE	222	0	0	0/0	0/0	0/0	0/0	0/0	0/0	0/0	0/0	0/0	0/0	0/0	0/0	0/1	0/1
Chromosome2-4022022	DEL	PRECISE	93	0	0	0/0	0/0	0/0	0/0	0/0	0/0	0/0	0/0	0/0	0/0	0/0	0/0	0/0	0/1
Chromosome2-4022198	DEL	PRECISE	148	0.982	0.539	0/0	0/0	0/0	0/0	1/1	0/1	0/1	0/1	0/1	1/1	1/1	1/1	0/1	0/1
Chromosome2-4022320	DEL	PRECISE	56	0	0	0/0	0/0	0/0	0/0	0/0	0/0	0/0	0/0	0/0	0/0	0/0	0/0	0/0	0/1
Chromosome2-4023641	DEL	PRECISE	689	0.289	0.289	0/0	0/1	0/0	0/1	1/1	0/1	0/1	0/1	0/1	1/1	1/1	1/1	1/1	1/1
Chromosome2-4024419	DEL	PRECISE	56	0	0.934	0/0	0/1	0/0	0/0	1/1	1/1	0/1	0/1	0/0	1/1	1/1	0/1	1/1	1/1
Chromosome2-4024519	DEL	PRECISE	164	0	0	0/0	0/0	0/0	0/0	0/0	0/0	0/1	0/1	0/0	0/0	0/0	0/0	0/0	0/0
Chromosome2-4026274	DEL	PRECISE	234	0.289	0.492	0/0	0/1	0/1	0/0	1/1	0/1	1/1	1/1	0/1	1/1	1/1	1/1	0/1	0/0
Chromosome2-4026552	DEL	PRECISE	96	0	0	0/0	0/1	0/0	0/0	1/1	0/1	0/1	0/1	0/0	1/1	0/1	0/1	0/1	0/1
Chromosome2-4027410	DEL	PRECISE	107	0	0	0/0	0/0	0/0	0/0	0/1	0/0	0/0	0/0	0/1	0/1	0/1	1/1	1/1	0/1
Chromosome2-4027937	INS	PRECISE	137	0	0	0/0	0/0	0/1	0/0	0/0	0/0	0/0	0/0	0/0	0/0	0/0	0/0	0/0	0/0
Chromosome2-4028174	BND	PRECISE	0	0	0	0/0	0/0	0/0	0/0	0/0	0/0	0/0	0/0	0/1	0/0	0/0	0/1	0/0	0/0
Chromosome2-4028385	INS	PRECISE	1982	0	0	0/0	0/0	0/0	0/1	0/0	0/0	0/0	0/0	0/0	0/0	0/0	0/0	0/0	0/0
Chromosome2-4028678	DEL	PRECISE	58	0	0	0/0	0/0	0/0	0/0	0/0	0/0	0/0	0/0	0/0	0/0	0/1	0/0	0/0	0/0
Chromosome2-4029406	DEL	PRECISE	67	0	0	0/0	0/0	0/0	0/0	0/1	0/0	0/0	0/0	0/0	0/1	0/0	0/1	0/0	0/0
Chromosome2-4029579	INS	PRECISE	323	0	0	0/0	0/0	0/0	0/0	0/1	0/0	0/0	0/0	0/0	0/0	0/0	0/0	0/0	0/0
Chromosome2-4029977	DEL	PRECISE	241	0	0	0/0	0/0	0/0	0/0	0/0	0/1	0/0	0/0	0/1	0/0	0/0	0/0	0/0	0/0
Chromosome2-4030154	INS	PRECISE	137	0.707	0.707	0/0	0/0	0/0	0/0	0/0	0/0	0/0	0/0	0/0	0/0	0/0	0/0	0/1	0/1
Chromosome2-4030318	DEL	PRECISE	813	0	0	0/0	0/1	0/0	0/0	1/1	0/1	0/1	0/1	0/1	1/1	1/1	1/1	0/1	0/1
Chromosome2-4030837	DEL	PRECISE	452	0	0	0/0	0/1	0/0	0/0	1/1	0/1	0/1	0/1	0/1	1/1	1/1	1/1	0/1	0/1
Chromosome2-4031071	INS	PRECISE	1288	0	0	0/0	0/0	0/0	0/1	0/0	0/0	0/0	0/0	0/0	0/0	0/0	0/0	0/0	0/0
Chromosome2-4031280	INS	PRECISE	1840	0	0	0/0	0/0	0/0	0/0	0/0	0/0	0/0	0/0	0/0	0/0	0/1	0/0	0/0	0/0
Chromosome2-4031361	DEL	PRECISE	84	0	0	0/0	0/0	0/0	0/0	0/0	0/0	0/0	0/0	0/0	0/1	0/0	0/1	0/0	0/0
Chromosome2-4031559	DEL	PRECISE	357	0	0	0/0	0/1	0/0	0/0	1/1	0/1	0/1	0/1	0/1	1/1	1/1	1/1	0/1	0/1
Chromosome2-4031586	INS	PRECISE	55	0	0	0/0	0/0	0/1	0/0	0/0	0/0	0/0	0/0	0/0	0/0	0/0	0/0	0/0	0/0
Chromosome2-4031636	INS	PRECISE	6472	0	0	0/0	0/0	0/0	0/0	0/0	0/0	0/0	0/0	0/0	0/0	0/0	0/0	1/1	0/0
Chromosome2-4032234	DEL	PRECISE	76	0	0	0/0	0/0	0/0	0/0	0/0	0/0	0/0	0/0	0/1	0/1	0/0	1/1	0/0	0/0
Chromosome2-4032708	DEL	PRECISE	80	0	0	0/0	0/0	0/0	0/0	0/0	0/0	0/0	0/0	0/0	0/0	0/0	0/0	0/0	0/1
Chromosome2-4032805	DEL	PRECISE	165	0	0	0/0	0/0	0/1	0/1	0/0	0/0	0/0	0/0	0/0	0/0	0/0	0/0	0/0	0/0
Chromosome2-4033593	INS	PRECISE	609	0	0	0/0	0/0	0/1	0/0	0/0	0/0	0/0	0/0	0/0	0/0	0/0	0/0	0/0	0/0
Chromosome2-4034157	INS	PRECISE	50	0	0	0/0	0/0	0/0	0/1	0/0	0/0	0/0	0/0	0/0	0/0	0/0	0/0	0/0	0/0
Chromosome2-4034404	DEL	PRECISE	810	0	0	0/0	0/1	0/0	0/0	1/1	1/1	0/1	0/1	0/1	1/1	1/1	1/1	1/1	0/1
Chromosome2-4034564	INS	PRECISE	1834	0	0	0/0	0/0	0/0	0/1	0/0	0/0	0/0	0/0	0/0	0/0	0/0	0/0	0/0	0/0
Chromosome2-4034906	DEL	PRECISE	98	0	0	0/0	0/0	0/0	0/0	0/0	0/0	0/0	0/0	0/0	0/0	0/0	0/0	0/0	1/1
Chromosome2-4034907	BND	PRECISE	0	0	0	0/0	0/0	0/0	0/0	0/0	0/1	0/0	0/0	0/0	0/0	0/0	0/0	0/0	0/0
Chromosome2-4035138	INS	PRECISE	60	0	0	0/0	0/0	0/0	0/1	0/0	0/0	0/0	0/0	0/0	0/0	0/0	0/0	0/0	0/0
Chromosome2-4035631	INS	PRECISE	73	0	0	0/0	0/0	0/0	0/0	0/1	0/0	0/0	0/0	0/0	0/1	0/1	0/1	0/1	0/1
Chromosome2-4035744	DEL	PRECISE	86	0	0	0/0	0/0	0/0	0/0	0/1	0/0	0/0	0/0	0/0	0/0	0/0	0/0	0/0	0/0
Chromosome2-4036780	INS	PRECISE	1338	0	0	0/0	0/0	0/0	0/1	0/0	0/0	0/0	0/0	0/0	0/0	0/0	0/0	0/0	0/0
Chromosome2-4037302	INS	PRECISE	67	0	0.378	0/0	0/0	0/0	0/0	0/1	0/0	0/0	0/0	0/1	0/1	0/1	1/1	0/1	0/1
Chromosome2-4038023	DEL	PRECISE	105	0	0	0/0	0/0	0/0	0/0	0/1	0/0	0/0	0/0	0/0	0/0	0/0	0/0	0/0	0/0
Chromosome2-4038370	INS	PRECISE	132	0	0	0/0	0/1	0/0	0/0	1/1	0/1	0/1	0/1	0/1	1/1	1/1	1/1	1/1	0/1
Chromosome2-4039750	INS	PRECISE	74	0	0	0/0	0/1	0/1	1/1	1/1	1/1	1/1	1/1	1/1	1/1	1/1	1/1	1/1	1/1
Chromosome2-4040028	DEL	PRECISE	57	0	0	0/0	0/1	0/0	0/0	1/1	0/1	0/1	0/0	0/1	1/1	1/1	1/1	1/1	0/1
Chromosome2-4040509	INS	PRECISE	51	0	0	0/0	0/0	0/1	0/1	0/0	0/0	0/0	0/0	0/0	0/0	0/0	0/0	0/0	0/0
Chromosome2-4040989	DEL	PRECISE	109	0	0	0/0	0/1	0/0	0/0	1/1	0/1	0/0	0/1	0/0	1/1	1/1	1/1	1/1	0/0
Chromosome2-4041875	INS	PRECISE	687	0	0	0/0	0/0	0/0	0/0	0/0	0/0	0/0	0/0	0/0	0/0	0/1	0/0	0/0	0/0
Chromosome2-4041904	DEL	PRECISE	83	0	0	0/0	0/1	0/0	0/0	0/1	1/1	0/1	0/1	0/0	0/1	0/1	0/0	0/1	0/0
Chromosome2-4042658	DEL	PRECISE	84	0	0	0/0	0/0	0/1	0/0	0/0	0/0	0/0	0/0	0/0	0/0	0/0	0/0	0/0	0/0
Chromosome2-4042930	DEL	PRECISE	131	0.277	0	0/0	0/1	0/1	0/1	1/1	1/1	1/1	1/1	1/1	1/1	1/1	1/1	1/1	1/1
Chromosome2-4043373	DEL	PRECISE	66	0	0	0/0	0/0	0/0	0/0	0/0	0/0	0/0	0/0	0/0	0/0	0/1	0/0	0/0	0/0
Chromosome2-4043874	DUP	PRECISE	9692	0	0	0/0	0/0	0/0	0/1	0/0	0/1	0/0	0/0	0/1	0/0	0/0	0/0	0/0	0/0
Chromosome2-4044343	INS	PRECISE	211	0	0	0/0	0/0	0/0	0/0	0/0	0/0	0/0	0/0	0/0	0/0	0/0	0/0	0/0	0/1
Chromosome2-4044390	DEL	PRECISE	153	0.707	0.707	0/0	0/0	0/0	0/0	0/0	0/0	0/0	0/0	0/1	0/0	0/0	0/1	0/0	0/0
Chromosome2-4044709	INS	PRECISE	88	0	0	0/0	0/0	0/0	0/0	0/0	0/0	0/0	0/0	0/0	0/0	0/0	0/0	0/0	0/1
Chromosome2-4044825	INS	PRECISE	9695	0	0	0/0	0/0	0/0	0/0	0/0	0/1	0/0	0/0	0/0	0/0	0/0	0/0	0/0	0/0
Chromosome2-4044943	INS	PRECISE	258	0	0	0/0	0/0	0/0	0/0	0/0	0/0	0/0	0/0	0/0	0/0	0/0	0/0	0/0	0/1
Chromosome2-4045197	DEL	PRECISE	211	0	0	0/0	0/1	0/0	0/1	1/1	0/1	0/1	0/1	0/1	1/1	1/1	1/1	1/1	0/1
Chromosome2-4045225	BND	PRECISE	0	0	0	0/0	0/0	0/0	0/0	0/0	0/0	0/1	0/1	0/0	0/0	0/0	0/0	0/0	0/0
Chromosome2-4045856	DEL	PRECISE	1261	0	0	0/0	0/1	0/1	1/1	1/1	1/1	1/1	1/1	1/1	1/1	1/1	1/1	1/1	1/1
Chromosome2-4046020	DEL	PRECISE	107	0	0	0/0	0/0	0/0	0/0	0/0	0/0	0/0	0/0	0/0	0/0	0/0	0/0	0/0	0/1
Chromosome2-4046305	DEL	PRECISE	167	0	0	0/0	0/0	0/0	0/0	0/0	0/0	0/0	0/0	1/1	0/0	0/0	1/1	0/0	0/0

Chromosome2-4046823	INS	PRECISE	240	0	0	0/0	0/0	0/1	0/0	0/0	0/0	0/0	0/0	0/0	0/0	0/0	0/0	0/0	0/0
Chromosome2-4047177	INS	PRECISE	359	0	0	0/0	0/0	0/0	0/0	0/1	0/0	0/0	0/0	0/0	0/0	0/0	0/0	0/0	0/0
Chromosome2-4047400	INS	PRECISE	79	0	0.277	0/0	0/1	0/1	1/1	1/1	1/1	1/1	1/1	1/1	1/1	1/1	1/1	1/1	0/1
Chromosome2-4047770	DEL	PRECISE	94	0	0	0/0	0/0	0/1	0/1	0/0	0/0	0/0	0/0	0/0	0/0	0/0	0/0	0/0	0/0
Chromosome2-4048559	DEL	PRECISE	362	0	0	0/0	0/0	0/1	1/1	0/0	0/0	0/0	0/0	0/0	0/0	0/0	0/0	1/1	0/1
Chromosome2-4048931	INS	PRECISE	65	0	0.333	0/0	0/1	0/0	0/0	1/1	1/1	0/1	0/1	0/0	1/1	0/0	1/1	1/1	0/1
Chromosome2-4049107	INS	PRECISE	11236	0	0	0/0	0/0	1/1	0/0	0/0	0/0	0/0	0/0	0/0	0/0	0/0	0/0	0/0	0/0
Chromosome2-4049673	INS	PRECISE	62	0	0	0/0	0/1	0/0	0/0	1/1	0/1	0/1	0/1	0/1	1/1	1/1	1/1	1/1	0/1
Chromosome2-4049900	DEL	PRECISE	348	0	0	0/0	0/1	0/1	1/1	1/1	1/1	1/1	1/1	1/1	1/1	1/1	1/1	1/1	1/1
Chromosome2-4051700	INS	PRECISE	64	0	0	0/0	0/0	0/0	0/0	0/0	0/0	0/0	0/0	0/1	0/0	0/0	0/1	0/0	0/0
Chromosome2-4051886	INS	PRECISE	401	0	0	0/0	0/0	0/0	0/0	0/0	0/1	0/0	0/0	0/1	0/0	0/0	0/0	0/0	0/0
Chromosome2-4052009	INS	PRECISE	214	0	0	0/0	0/0	0/0	0/0	0/0	0/0	0/0	0/0	1/1	0/0	0/0	0/0	0/0	0/0
Chromosome2-4052137	INS	PRECISE	62	0	0.5	0/0	0/0	0/0	0/1	0/0	0/0	0/0	0/0	0/0	0/0	0/0	1/1	0/0	1/1
Chromosome2-4052156	INS	PRECISE	1321	0	0	0/0	0/0	0/1	0/0	0/0	0/0	0/0	0/0	0/0	0/0	0/0	0/0	0/0	0/0
Chromosome2-4052173	DEL	PRECISE	1959	0	0	0/0	0/0	0/0	0/0	0/0	0/0	0/0	0/0	0/0	0/0	0/0	1/1	0/0	0/0
Chromosome2-4052287	INS	PRECISE	239	0	0	0/0	0/0	0/0	0/1	0/0	0/0	0/0	0/0	0/0	0/0	0/0	0/0	0/0	0/0
Chromosome2-4052400	DEL	PRECISE	61	0	0	0/0	0/0	0/1	0/1	0/0	1/1	0/0	0/0	1/1	0/0	0/0	0/0	0/0	0/0
Chromosome2-4052491	INS	PRECISE	10850	0	0	0/0	0/0	0/0	0/0	0/0	0/0	0/0	0/0	0/0	0/0	0/0	0/0	0/0	0/0
Chromosome2-4052716	DEL	PRECISE	179	0	0	0/0	0/0	0/1	1/1	0/0	1/1	1/1	1/1	1/1	1/1	1/1	1/1	0/0	1/1
Chromosome2-4052748	INS	PRECISE	132	0	0	0/0	0/0	0/0	0/1	0/0	0/0	0/0	0/0	0/0	0/0	0/0	0/0	0/0	0/0
Chromosome2-4053631	INS	PRECISE	160	0	0.518	0/0	0/1	0/0	0/0	1/1	0/1	0/1	1/1	0/0	1/1	0/1	0/0	1/1	0/0
Chromosome2-4055957	INS	PRECISE	645	0	0	0/0	0/0	0/0	0/1	0/0	0/0	0/0	0/0	0/0	0/0	0/0	0/0	0/0	0/0
Chromosome2-4056236	INS	PRECISE	596	0	0	0/0	0/0	0/1	0/0	0/0	0/0	0/0	0/0	0/0	0/0	0/0	0/0	0/0	0/0
Chromosome2-4056866	INS	PRECISE	76	0	0	0/0	0/1	0/0	0/1	1/1	0/1	1/1	1/1	0/0	1/1	1/1	1/1	1/1	1/1
Chromosome2-4057524	DEL	PRECISE	5239	0	0	0/0	0/1	0/1	1/1	1/1	0/1	1/1	1/1	0/1	1/1	1/1	1/1	1/1	1/1
Chromosome2-4058331	BND	PRECISE	0	0	0	0/0	0/0	0/0	0/0	0/0	0/0	0/0	0/0	0/0	0/0	0/0	0/0	0/0	0/1
Chromosome2-4058997	INS	PRECISE	104	0	0	0/0	0/0	0/0	0/0	0/0	0/0	0/0	0/0	0/0	0/0	0/0	0/0	0/0	0/1
Chromosome2-4059058	DEL	PRECISE	335	0	0	0/0	0/0	0/0	0/0	0/0	0/0	0/0	0/0	0/0	0/0	1/1	0/1	0/0	0/0
Chromosome2-4059373	INS	PRECISE	137	0	0	0/0	0/0	0/0	0/1	0/0	0/0	0/1	0/1	0/0	0/0	0/0	0/0	0/0	0/0
Chromosome2-4059535	DEL	PRECISE	275	0	0	0/0	0/1	0/0	0/1	1/1	1/1	0/1	0/1	0/1	1/1	1/1	1/1	1/1	0/0
Chromosome2-4059884	DEL	PRECISE	174	0	0	0/0	0/1	0/0	0/0	1/1	0/1	0/1	0/1	0/1	1/1	1/1	1/1	1/1	0/1
Chromosome2-4059987	DEL	PRECISE	335	0	0	0/0	0/0	0/0	0/0	0/0	0/0	0/0	0/0	0/0	1/1	0/0	0/0	0/0	0/0
Chromosome2-4060257	DEL	PRECISE	65	0	0	0/0	0/0	0/0	0/0	0/0	0/0	0/0	0/0	0/0	0/0	0/0	0/0	0/0	0/1
Chromosome2-4060287	INS	PRECISE	595	0	0	0/0	0/0	0/1	0/0	0/0	0/0	0/0	0/0	0/0	0/0	0/0	0/0	0/0	0/0
Chromosome2-4060512	DEL	PRECISE	99	0	0	0/0	0/0	0/0	0/0	0/0	0/0	0/0	0/0	0/0	0/0	0/0	0/0	0/0	0/1
Chromosome2-4061154	INS	PRECISE	293	0	0	0/0	0/0	0/0	0/0	0/0	0/0	0/0	0/0	0/0	0/0	0/0	0/0	0/0	0/1
Chromosome2-4061161	DEL	PRECISE	265	0	0	0/0	0/0	0/0	0/0	0/0	0/0	0/0	0/0	0/0	0/0	0/1	0/1	0/0	0/0
Chromosome2-4061517	INS	PRECISE	10013	0	0	0/0	0/0	0/0	0/0	0/1	0/0	0/0	0/0	0/0	0/0	0/0	0/0	0/0	0/0
Chromosome2-4061518	BND	PRECISE	0	0	0	0/0	0/0	0/0	0/0	1/1	0/0	0/0	0/0	0/0	0/0	0/0	0/0	0/0	0/0
Chromosome2-4061620	INS	PRECISE	5316	0	0	0/0	0/0	1/1	0/0	0/0	0/0	0/0	0/0	0/0	0/0	0/0	0/0	0/0	0/0
Chromosome2-4062791	DEL	PRECISE	217	0	0	0/0	0/1	0/0	0/0	1/1	0/1	0/1	0/1	0/1	1/1	1/1	1/1	1/1	1/1
Chromosome2-4062886	INS	PRECISE	1289	0	0.707	0/0	0/0	0/0	0/0	0/0	0/0	0/0	0/0	0/0	0/0	0/1	1/1	0/0	0/0
Chromosome2-4063087	INS	PRECISE	5230	0	0	0/0	0/0	0/0	0/0	0/0	0/0	0/0	0/0	0/0	0/0	0/0	0/0	0/0	0/1
Chromosome2-4063087	BND	PRECISE	0	0	0	0/0	0/0	0/0	0/0	0/0	0/0	0/0	0/0	0/0	0/0	0/0	0/0	0/0	0/1
Chromosome2-4063256	DEL	PRECISE	334	0	0	0/0	0/1	0/0	0/0	1/1	0/1	0/0	0/1	0/1	1/1	1/1	1/1	1/1	1/1
Chromosome2-4064278	DEL	PRECISE	212	0	0	0/0	0/1	0/0	0/0	1/1	0/1	0/0	0/1	0/1	1/1	1/1	1/1	1/1	0/1
Chromosome2-4064615	DEL	PRECISE	383	0	0	0/0	0/1	0/0	0/0	1/1	0/1	0/0	0/1	0/1	1/1	1/1	1/1	1/1	0/1
Chromosome2-4065158	DEL	PRECISE	383	0	0	0/0	0/0	0/0	0/0	0/1	0/0	0/0	0/0	0/1	0/0	0/1	0/1	0/1	0/1
Chromosome2-4065456	DEL	PRECISE	161	0	0	0/0	0/0	0/0	0/0	0/0	0/0	0/0	0/0	0/1	0/0	0/0	0/0	0/0	0/0
Chromosome2-4065468	INS	PRECISE	703	0	0	0/0	0/0	0/1	0/1	0/0	0/0	0/0	0/0	0/0	0/0	0/0	0/0	0/0	0/0
Chromosome2-4065772	DEL	PRECISE	131	0	0	0/0	0/0	0/1	1/1	1/1	1/1	1/1	1/1	1/1	0/0	0/0	1/1	1/1	1/1
Chromosome2-4066541	INS	PRECISE	374	0	0.577	0/0	0/0	0/0	0/0	0/1	0/0	0/0	0/0	0/0	0/0	0/0	0/0	0/1	0/1
Chromosome2-4067507	INS	PRECISE	106	0	0	0/0	0/0	0/0	0/1	0/0	0/0	0/0	0/0	0/0	0/0	0/0	0/0	0/0	0/0
Chromosome2-4067728	INS	PRECISE	52	0	0	0/0	0/0	0/0	0/0	0/0	0/1	0/1	0/1	0/1	0/0	0/0	0/0	0/0	0/0
Chromosome2-4068811	DEL	PRECISE	698	0	0	0/0	0/1	0/1	0/1	1/1	0/1	0/1	0/1	0/1	1/1	1/1	1/1	1/1	1/1
Chromosome2-4068886	DEL	PRECISE	313	0	0	0/0	0/0	0/0	0/0	0/0	0/0	0/0	0/0	0/1	0/1	0/1	1/1	0/0	0/0
Chromosome2-4069349	DEL	PRECISE	57	0	0	0/0	0/1	0/0	0/0	0/1	0/1	0/1	0/1	0/0	0/1	0/1	0/0	0/1	0/0
Chromosome2-4069780	DEL	PRECISE	55	0	0	0/0	0/1	0/0	0/0	0/1	0/1	0/1	0/1	0/0	0/1	0/1	0/0	0/1	0/0
Chromosome2-4069931	INS	PRECISE	2863	0	0	0/0	0/0	0/0	0/1	0/0	0/0	0/0	0/0	0/0	0/0	0/0	0/0	0/0	0/0
Chromosome2-4070058	INS	PRECISE	612	0	0	0/0	0/0	0/0	0/0	0/0	0/0	0/0	0/0	0/1	0/0	0/0	0/0	0/0	0/0
Chromosome2-4072219	DEL	PRECISE	145	0	0	0/0	0/1	0/0	0/0	1/1	0/1	0/1	0/1	0/1	1/1	1/1	1/1	1/1	0/1
Chromosome2-4073614	DEL	PRECISE	53	0	0	0/0	0/1	0/0	0/0	1/1	0/1	0/1	0/1	0/1	1/1	1/1	1/1	1/1	0/1
Chromosome2-4073625	INS	PRECISE	976	0	0	0/0	0/0	0/0	0/0	0/0	0/0	0/0	0/0	0/1	0/0	0/0	0/0	0/0	0/0
Chromosome2-4075321	INS	PRECISE	1251	0	0	0/0	0/0	0/0	0/0	0/0	0/0	0/0	0/0	0/1	0/0	0/0	0/0	0/0	0/0
Chromosome2-4076698	DEL	PRECISE	53	0.568	0.422	0/0	0/1	0/0	0/0	1/1	0/1	0/1	0/1	0/1	1/1	1/1	1/1	0/1	0/0
Chromosome2-4079343	INS	PRECISE	270	0	0.467	0/0	0/1	0/0	0/0	1/1	0/1	0/1	0/1	0/1	1/1	1/1	1/1	1/1	0/1
Chromosome2-4079955	DEL	PRECISE	1396	0.832	0	0/0	0/1	0/1	1/1	1/1	1/1	1/1	1/1	1/1	1/1	1/1	1/1	1/1	1/1
Chromosome2-4081326	DUP	PRECISE	271	0	0	0/0	0/0	0/0	0/0	0/0	0/0	0/0	0/0	0/0	0/0	0/0	0/0	0/0	0/1
Chromosome2-4081364	DEL	PRECISE	1684	0	0	0/0	0/1	0/0	0/0	1/1	0/1	0/1	1/1	0/1	1/1	1/1	1/1	1/1	0/0
Chromosome2-4081746	INS	PRECISE	330	0	0.707	0/0	0/0	0/1	0/1	0/0	0/0	0/0	0/0	0/0	0/0	0/0	0/0	0/0	0/0
Chromosome2-4082804	DEL	PRECISE	834	0	0	0/0	0/1	0/0	0/0	1/1	0/1	0/1	1/1	0/1	1/1	1/1	1/1	1/1	0/1
Chromosome2-4082854	INS	PRECISE	18772	0	0	0/0	0/0	0/0	0/0	0/0	0/0	0/0	0/0	0/1	0/0	0/0	0/0	0/0	0/0
Chromosome2-4082855	BND	PRECISE	0	0.753	0	0/0	0/0	0/0	0/0	0/0	0/1	0/0	0/0	0/1	0/0	0/0	0/0	0/0	0/0

Chromosome2-4083887	INS	PRECISE	7244	0	0	0/0	0/0	0/0	1/1	0/0	0/0	0/0	0/0	0/0	0/0	0/0	0/0	0/0	0/0
Chromosome2-4084314	INS	PRECISE	553	0	0	0/0	0/0	0/0	0/1	0/0	0/0	0/1	0/0	0/0	0/0	0/0	0/0	0/0	0/0
Chromosome2-4085093	DEL	PRECISE	68	0	0	0/0	0/0	0/0	0/0	0/0	0/0	0/0	0/0	0/0	0/0	0/0	0/0	0/0	1/1
Chromosome2-4085522	INS	PRECISE	50	0	0	0/0	0/0	0/0	0/0	0/0	0/0	0/0	0/0	0/0	0/0	0/0	0/0	0/0	1/1
Chromosome2-4086214	DEL	PRECISE	64	0	0	0/0	0/1	0/0	0/0	1/1	0/1	1/1	1/1	1/1	1/1	1/1	1/1	1/1	0/1
Chromosome2-4087001	INS	PRECISE	118	0	0.354	0/0	0/1	0/0	0/0	0/1	0/1	0/1	1/1	0/0	0/1	0/1	0/0	0/1	0/0
Chromosome2-4087136	DEL	PRECISE	123	0	0	0/0	0/1	0/0	0/0	1/1	0/1	0/1	1/1	0/0	0/1	0/1	0/0	1/1	0/1
Chromosome2-4087213	INS	PRECISE	693	0	0.707	0/0	0/0	0/0	0/0	0/0	0/0	0/0	0/0	0/0	0/0	0/0	0/0	0/1	0/1
Chromosome2-4088003	DEL	PRECISE	53	0	0	0/0	0/1	0/0	0/0	1/1	0/1	0/1	1/1	0/1	1/1	1/1	1/1	1/1	0/1
Chromosome2-4088346	INS	PRECISE	51	0	0	0/0	0/1	0/0	0/0	1/1	0/1	0/1	1/1	0/1	1/1	1/1	1/1	1/1	0/1
Chromosome2-4088458	INS	PRECISE	840	0	0.674	0/0	0/1	0/0	0/0	1/1	0/1	0/1	1/1	0/1	1/1	1/1	1/1	1/1	0/1
Chromosome2-4090236	BND	PRECISE	0	0	0	0/0	0/0	0/0	0/0	0/0	0/0	0/0	0/0	0/0	0/1	0/0	0/0	0/0	0/0
Chromosome2-4091488	DEL	PRECISE	60	0	0	0/0	0/0	0/0	0/0	0/1	0/0	0/0	0/0	0/0	0/0	0/0	0/0	0/0	0/0
Chromosome2-4091947	INS	PRECISE	701	0	0	0/0	0/0	0/0	0/0	0/0	0/0	0/0	0/0	0/0	0/0	0/0	0/0	0/0	0/1
Chromosome2-4092821	DEL	PRECISE	66	0	0	0/0	0/0	0/0	0/0	0/0	0/0	0/0	0/0	0/1	0/1	0/0	0/1	0/0	0/0
Chromosome2-4092837	INS	PRECISE	692	0	0	0/0	0/0	0/0	1/1	0/0	0/0	0/1	0/0	0/0	0/0	0/0	0/0	0/0	0/0
Chromosome2-4093574	INS	PRECISE	216	0	0	0/0	0/0	0/0	0/0	0/0	0/0	0/0	0/0	0/0	0/0	0/0	0/0	0/0	0/1
Chromosome2-4093687	INS	PRECISE	210	0	0.707	0/0	0/0	0/0	0/0	0/0	0/0	0/0	0/0	0/0	0/0	0/1	0/1	0/0	0/0
Chromosome2-4093856	DEL	PRECISE	229	0.302	0.302	0/0	0/1	0/0	0/0	1/1	0/0	0/1	1/1	0/1	1/1	1/1	1/1	1/1	0/1
Chromosome2-4094002	INS	PRECISE	60	0	0	0/0	0/0	0/1	0/1	0/0	0/0	0/1	0/0	0/0	0/0	0/0	0/0	0/0	0/1
Chromosome2-4094245	INS	PRECISE	241	0.408	0.894	0/0	0/0	0/0	0/0	0/0	0/0	0/0	0/0	0/1	0/1	0/1	1/1	0/1	0/1
Chromosome2-4094865	DEL	PRECISE	1061	0	0	0/0	0/0	0/0	0/1	0/0	0/0	0/0	0/0	0/0	0/0	0/0	0/0	0/0	0/0
Chromosome2-4095712	DEL	PRECISE	2960	0	0	0/0	0/0	0/0	0/0	0/0	0/0	0/0	0/0	0/0	0/0	0/0	0/0	0/0	1/1
Chromosome2-4096099	DEL	PRECISE	162	0	0	0/0	0/0	0/1	0/1	0/0	0/0	0/0	0/0	0/0	0/0	0/0	0/0	0/0	0/1
Chromosome2-4096225	DEL	PRECISE	77	0	0	0/0	0/0	0/0	0/0	1/1	0/1	0/1	1/1	0/1	1/1	1/1	1/1	1/1	0/1
Chromosome2-4096241	INS	PRECISE	150	0	0.302	0/0	0/0	0/0	0/0	1/1	0/1	0/1	1/1	0/1	1/1	1/1	1/1	1/1	0/1
Chromosome2-4096593	DEL	PRECISE	73	0.333	0.333	0/0	0/1	0/0	0/0	0/1	0/1	0/1	1/1	0/0	1/1	0/1	0/1	0/1	0/0
Chromosome2-4096915	DEL	PRECISE	122	0	0	0/0	0/0	0/0	0/0	0/1	0/0	0/0	0/0	0/1	0/0	0/0	0/0	0/0	1/1
Chromosome2-4097702	INS	PRECISE	363	0	0.5	0/0	0/1	0/0	0/0	0/1	0/1	0/1	1/1	0/0	1/1	0/1	0/1	1/1	0/0
Chromosome2-4097998	INS	PRECISE	585	0	0	0/0	0/0	0/0	0/0	0/0	0/0	0/0	0/0	0/0	0/0	0/0	0/0	0/0	0/0
Chromosome2-4098101	DEL	PRECISE	342	0	0	0/0	0/1	0/1	0/1	1/1	0/1	0/1	1/1	0/1	1/1	1/1	1/1	1/1	1/1
Chromosome2-4099368	INS	PRECISE	12288	0	0	0/0	0/0	0/0	1/1	0/0	0/0	0/0	0/0	0/0	0/0	0/0	0/0	0/0	0/0
Chromosome2-4100346	DEL	PRECISE	864	0	0	0/0	0/1	0/0	0/0	1/1	0/0	0/0	1/1	0/1	1/1	1/1	1/1	1/1	0/1
Chromosome2-4100825	DEL	PRECISE	294	0	0	0/0	0/0	0/0	0/1	0/0	0/0	0/0	0/0	0/0	0/0	0/0	0/0	0/0	0/0
Chromosome2-4101016	INS	PRECISE	51	0	0.707	0/0	0/1	0/0	0/0	0/0	0/0	0/0	1/1	0/0	0/0	1/1	0/0	0/1	0/0
Chromosome2-4102024	INS	PRECISE	6089	0	0	0/0	0/0	0/0	0/0	0/0	0/0	0/0	0/0	0/0	0/0	0/0	1/1	0/0	0/0
Chromosome2-4102756	DEL	PRECISE	59	0	0	0/0	0/0	0/0	0/0	0/0	0/0	0/0	0/0	0/0	0/0	0/1	0/1	0/0	0/0
Chromosome2-4103503	INS	PRECISE	1027	0	0	0/0	0/0	0/0	0/1	0/0	0/0	0/0	0/0	0/0	0/0	0/0	0/0	0/0	0/0
Chromosome2-4103652	INS	PRECISE	211	0	0	0/0	0/0	0/0	0/0	0/0	0/0	0/0	0/0	0/0	0/0	0/0	0/0	0/0	0/1
Chromosome2-4103699	DEL	PRECISE	159	0	0	0/0	0/1	0/0	0/0	1/1	0/1	0/1	1/1	0/1	1/1	1/1	1/1	1/1	1/1
Chromosome2-4104041	INS	PRECISE	54	0	0	0/0	0/0	0/0	0/0	0/0	0/0	0/0	0/0	0/0	0/0	0/0	0/0	0/0	0/1
Chromosome2-4105134	INS	PRECISE	55	0	0	0/0	0/1	0/0	0/0	0/1	0/1	0/1	1/1	0/0	0/1	0/1	0/0	0/1	0/0
Chromosome2-4105452	DEL	PRECISE	237	0.405	0.405	0/0	0/1	0/0	0/0	0/1	0/1	0/1	1/1	0/1	1/1	1/1	1/1	1/1	0/1
Chromosome2-4105945	INS	PRECISE	209	0	0	0/0	0/0	0/0	0/0	0/0	0/0	0/0	0/0	0/0	0/0	0/1	0/1	0/0	0/0
Chromosome2-4106138	INS	PRECISE	1456	0	0.707	0/0	0/0	0/1	0/1	0/0	0/0	0/0	0/0	0/0	0/0	0/0	0/0	0/0	0/0
Chromosome2-4106295	INS	PRECISE	288	0	0	0/0	0/0	0/1	0/1	0/0	0/0	0/0	0/0	0/0	0/0	0/0	0/0	0/0	0/0
Chromosome2-4110177	DEL	PRECISE	56	0	0	0/0	0/0	0/0	0/0	0/0	0/0	0/0	0/0	0/0	0/0	0/0	0/0	0/0	0/1
Chromosome2-4112970	INS	PRECISE	55	0	0	0/0	0/0	0/1	1/1	0/0	0/0	0/0	0/0	0/0	0/0	0/0	0/0	0/0	0/0
Chromosome2-4113823	INS	PRECISE	654	0	0.707	0/0	0/0	0/1	0/1	0/0	0/0	0/0	0/0	0/0	0/0	0/0	0/0	0/0	0/0
Chromosome2-4113874	INS	PRECISE	164	0	0.354	0/0	0/1	0/0	0/0	0/1	0/1	0/1	1/1	0/0	0/1	0/1	0/0	0/1	0/0
Chromosome2-4114428	INS	PRECISE	132	0	0.463	0/0	0/1	0/0	0/0	0/1	0/1	0/1	1/1	0/0	0/1	0/1	0/0	0/1	0/0
Chromosome2-4114793	DEL	PRECISE	124	0	0	0/0	0/0	0/0	0/0	0/0	0/0	0/0	0/0	0/0	0/0	0/0	0/0	0/0	0/1
Chromosome2-4116148	INS	PRECISE	276	0	0	0/0	0/1	0/0	0/0	0/1	0/1	0/1	1/1	0/1	0/1	0/1	0/0	1/1	0/1
Chromosome2-4116708	INS	PRECISE	130	0	0	0/0	0/0	0/1	0/1	0/0	0/0	0/0	0/0	0/0	0/0	0/0	0/0	0/0	0/0
Chromosome2-4117021	DEL	PRECISE	356	0	0	0/0	0/1	0/0	0/0	0/1	1/1	0/1	1/1	0/0	0/1	0/1	0/0	0/1	0/0
Chromosome2-4117714	INS	PRECISE	815	0	0	0/0	0/0	0/1	0/1	0/0	0/0	0/0	0/0	0/0	0/0	0/0	0/0	0/0	0/0
Chromosome2-4117744	DEL	PRECISE	426	0	0	0/0	0/1	0/0	0/0	0/1	1/1	0/1	1/1	0/1	1/1	1/1	1/1	1/1	0/1
Chromosome2-4117963	DEL	PRECISE	491	0	0	0/0	0/1	0/0	0/0	0/1	1/1	0/1	1/1	0/1	1/1	1/1	1/1	1/1	0/1
Chromosome2-4118666	INS	PRECISE	276	0	0.707	0/0	0/0	0/0	0/0	0/0	0/0	0/0	0/0	0/0	0/0	0/1	0/1	0/0	0/0
Chromosome2-4118766	INS	PRECISE	245	0	0.926	0/0	0/1	0/0	0/0	0/1	1/1	0/1	1/1	0/0	0/1	0/1	0/0	0/1	0/0
Chromosome2-4120577	DEL	PRECISE	68	0	0	0/0	0/0	0/0	0/0	0/0	0/0	0/0	0/0	0/1	0/0	0/0	0/0	0/0	0/0
Chromosome2-4121231	INS	PRECISE	2463	0	0.707	0/0	0/0	0/1	0/1	0/0	0/0	0/0	0/0	0/0	0/0	0/0	0/0	0/0	0/0
Chromosome2-4122036	INS	PRECISE	356	0	0	0/0	0/0	0/0	0/1	0/0	0/0	0/0	0/0	0/0	0/0	0/0	0/0	0/0	0/0
Chromosome2-4122038	DEL	PRECISE	77	0	0	0/0	0/0	0/0	0/0	0/0	0/0	0/0	0/0	0/0	0/0	0/0	0/0	0/0	0/1
Chromosome2-4122666	INS	PRECISE	132	0	0	0/0	0/0	0/0	0/0	0/0	0/0	0/0	0/0	0/0	0/0	0/0	0/0	0/0	0/1
Chromosome2-4123418	INS	PRECISE	236	0	0.707	0/0	0/0	0/0	0/0	0/0	0/0	0/0	0/0	0/1	0/0	0/1	0/1	0/1	0/1
Chromosome2-4123708	INS	PRECISE	149	0	0.707	0/0	0/0	0/1	0/1	0/0	0/0	0/0	0/0	0/0	0/0	0/0	0/0	0/0	0/0
Chromosome2-4124763	INS	PRECISE	58	0	0.577	0/0	0/0	0/1	0/1	0/0	0/0	0/0	0/0	0/0	0/0	0/0	0/0	0/0	0/1
Chromosome2-4125184	INS	PRECISE	995	0	0.707	0/0	0/0	0/0	0/0	0/0	0/1	0/0	0/0	0/1	0/0	0/0	0/0	0/0	0/0
Chromosome2-4125415	INS	PRECISE	1295	0	0	0/0	0/0	0/0	0/1	0/0	0/0	0/0	0/0	0/0	0/0	0/0	0/0	0/0	0/0
Chromosome2-4125966	INS	PRECISE	621	0	0.577	0/0	0/0	0/0	0/1	0/1	0/0	0/1	0/0	0/0	0/0	0/0	0/0	0/0	0/0
Chromosome2-4126200	DEL	PRECISE	106	0	0	0/0	0/0	0/0	0/0	0/0	0/0	0/0	0/0	0/0	0/0	0/0	0/0	0/0	0/1
Chromosome2-4127088	DEL	PRECISE	130	0.277	0.277	0/0	0/1	0/1	0/1	0/1	0/1	0/1	1/1	1/1	1/1	1/1	1/1	1/1	1/1

Chromosome2-4127695	INS	PRECISE	703	0	0	0/0	0/0	0/0	0/1	0/0	0/0	0/0	0/0	0/0	0/0	0/0	0/0	0/0	0/0
Chromosome2-4128426	DEL	PRECISE	774	0	0	0/0	0/1	0/1	0/1	0/1	0/0	0/0	1/1	0/1	1/1	1/1	1/1	1/1	1/1
Chromosome2-4128831	INS	PRECISE	4999	0	0	0/0	0/0	0/0	0/0	0/0	0/0	0/0	0/0	0/0	0/0	0/0	0/0	0/0	1/1
Chromosome2-4129014	INS	PRECISE	541	0	0.966	0/0	0/1	0/0	0/0	0/0	0/0	0/1	0/1	0/1	0/1	0/1	1/1	0/1	0/1
Chromosome2-4129449	INS	PRECISE	62	0	0.378	0/0	0/0	0/1	1/1	0/1	0/1	0/1	0/0	1/1	0/0	0/0	0/0	0/0	0/1
Chromosome2-4131249	INS	PRECISE	1377	0	0	0/0	0/0	0/0	0/0	0/0	0/0	0/0	0/0	0/0	0/0	0/0	0/0	0/0	1/1
Chromosome2-4132156	BND	PRECISE	0	0	0	0/0	0/0	0/0	0/0	0/0	0/0	0/0	0/0	0/0	0/0	0/1	0/0	0/0	0/0
Chromosome2-4132458	INS	PRECISE	57	0	0.707	0/0	0/0	0/0	0/0	0/0	0/0	0/0	0/0	0/0	0/0	0/1	0/1	0/0	0/0
Chromosome2-4133287	INS	PRECISE	724	0	0.991	0/0	0/1	0/0	0/0	0/1	0/0	0/1	1/1	0/0	0/1	0/1	0/0	0/1	0/0
Chromosome2-4133611	DEL	PRECISE	102	0	0	0/0	0/0	0/0	0/1	0/1	0/0	0/0	0/0	0/0	0/0	0/0	0/0	0/0	0/0
Chromosome2-4134614	INS	PRECISE	605	0.934	0.701	0/0	0/1	0/0	0/0	0/1	0/1	0/1	1/1	0/1	1/1	1/1	1/1	1/1	0/1
Chromosome2-4134738	DEL	PRECISE	87	0	0	0/0	0/0	0/1	0/1	0/0	0/0	0/0	0/0	0/0	0/0	0/0	0/0	0/0	0/0
Chromosome2-4134803	BND	PRECISE	0	0	0	0/0	0/0	0/0	0/0	0/0	0/0	0/1	0/0	0/0	0/0	0/0	0/0	0/0	0/0
Chromosome2-4134889	INS	PRECISE	804	0	0	0/0	0/0	0/0	0/0	0/0	0/0	0/0	0/0	0/0	0/0	0/0	0/0	0/0	0/1
Chromosome2-4136607	INS	PRECISE	52	0	0	0/0	0/0	0/0	0/0	0/0	0/0	0/0	0/0	0/0	0/1	0/0	0/0	0/0	0/0
Chromosome2-4137084	INS	PRECISE	701	0	0.707	0/0	0/0	0/0	0/0	0/0	0/0	0/1	0/0	0/0	0/0	0/0	0/0	0/0	0/1
Chromosome2-4137611	DEL	PRECISE	92	0	0	0/0	0/1	0/0	0/0	0/1	1/1	0/1	1/1	0/0	0/1	0/1	0/0	0/1	0/0
Chromosome2-4138099	INS	PRECISE	647	0	0.707	0/0	0/0	0/1	0/1	0/0	0/0	0/0	0/0	0/0	0/0	0/0	0/0	0/0	0/0
Chromosome2-4138757	INS	PRECISE	1000	0	0	0/0	0/0	0/0	0/1	0/0	0/0	0/0	0/0	0/0	0/0	0/0	0/0	0/0	0/0
Chromosome2-4138947	INS	PRECISE	211	0	0	0/0	0/0	0/0	0/1	0/0	0/0	0/0	0/0	0/0	0/0	0/0	0/0	0/0	0/0
Chromosome2-4138992	DEL	PRECISE	858	0	0	0/0	0/1	0/0	0/0	1/1	1/1	1/1	1/1	1/1	1/1	1/1	1/1	1/1	1/1
Chromosome2-4140359	BND	PRECISE	0	0	0	0/0	0/0	0/0	0/0	0/0	0/0	0/0	0/1	0/0	0/1	0/1	0/0	0/0	0/0
Chromosome2-4140369	INS	PRECISE	784	0	0	0/0	0/0	0/0	0/0	0/0	0/0	0/0	0/0	0/0	0/0	0/0	0/0	0/1	0/1
Chromosome2-4140779	DEL	PRECISE	62	0	0	0/0	0/0	0/0	0/0	0/0	0/0	0/0	0/0	0/0	0/0	0/0	0/0	0/1	1/1
Chromosome2-4141690	INS	PRECISE	63	0	0	0/0	0/1	0/0	0/0	0/1	0/1	0/1	1/1	0/1	0/1	1/1	0/1	1/1	0/1
Chromosome2-4141703	DEL	PRECISE	416	0	0	0/0	0/1	0/0	0/0	0/1	0/1	0/1	1/1	0/1	0/1	1/1	0/1	1/1	0/1
Chromosome2-4141770	INS	PRECISE	12090	0	0	0/0	0/0	0/0	0/0	1/1	0/0	0/0	0/0	0/0	0/0	0/0	0/0	0/0	0/0
Chromosome2-4142142	INS	PRECISE	6084	0	0	0/0	0/0	0/0	0/0	0/0	0/0	0/1	0/0	0/0	0/0	0/0	0/0	0/0	0/0
Chromosome2-4142225	DEL	PRECISE	5440	0.277	0.277	0/0	0/1	0/1	1/1	1/1	1/1	1/1	1/1	0/1	1/1	1/1	1/1	1/1	1/1
Chromosome2-4142826	INS	PRECISE	81	0	0	0/0	0/0	0/0	0/0	0/0	0/0	0/1	0/0	0/0	0/0	0/0	0/0	0/0	0/1
Chromosome2-4143601	DEL	PRECISE	51	0	0	0/0	0/0	0/0	0/0	0/0	0/0	0/0	0/0	0/1	0/0	0/0	0/0	0/0	0/0
Chromosome2-4144117	INS	PRECISE	56	0	0.302	0/0	0/1	0/0	0/0	0/1	0/1	0/1	1/1	0/1	1/1	1/1	1/1	1/1	0/1
Chromosome2-4145267	INS	PRECISE	998	0	0	0/0	0/0	0/0	0/0	0/1	0/0	0/0	0/0	0/0	0/0	0/0	0/0	0/0	0/0
Chromosome2-4146247	DEL	PRECISE	55	0	0	0/0	0/1	0/0	0/0	0/1	0/1	0/1	1/1	0/1	1/1	1/1	1/1	1/1	0/1
Chromosome2-4147255	INS	PRECISE	137	0	0	0/0	0/0	0/0	0/0	0/0	0/0	0/0	0/0	0/0	0/0	0/0	0/0	0/1	0/1
Chromosome2-4147560	INS	PRECISE	5510	0	0	0/0	0/0	0/0	0/0	0/0	0/0	0/0	0/0	0/0	0/0	0/0	0/0	0/0	1/1
Chromosome2-4147560	BND	PRECISE	0	0	0	0/0	0/0	0/0	0/0	0/0	0/0	0/0	0/0	0/0	0/0	0/0	0/0	0/0	0/1
Chromosome2-4147619	DEL	PRECISE	89	0	0	0/0	0/0	0/0	0/0	0/0	0/1	0/0	0/0	0/1	0/0	0/0	0/0	0/0	0/0
Chromosome2-4148149	DEL	PRECISE	75	0	0	0/0	0/0	0/0	0/1	0/0	0/0	0/0	0/0	0/0	0/0	0/0	0/0	0/0	0/0
Chromosome2-4148808	INS	PRECISE	242	0	0	0/0	0/0	0/0	0/0	0/0	0/0	0/0	0/0	0/0	0/0	0/0	0/0	0/1	1/1
Chromosome2-4148943	DEL	PRECISE	81	0	0	0/0	0/0	0/0	0/0	0/0	0/1	0/0	0/0	0/1	0/0	0/0	0/0	0/0	0/0
Chromosome2-4149635	DEL	PRECISE	51	0	0	0/0	0/0	0/0	0/0	0/0	0/0	0/0	0/0	0/0	0/0	0/1	0/1	0/1	0/1
Chromosome2-4149913	DEL	PRECISE	61	0	0	0/0	0/1	0/1	1/1	1/1	1/1	1/1	1/1	1/1	1/1	1/1	1/1	1/1	1/1
Chromosome2-4150045	DEL	PRECISE	61	0	0	0/0	0/0	0/0	0/0	0/0	0/0	0/0	0/0	0/0	0/0	0/1	0/1	0/0	0/0
Chromosome2-4150653	INS	PRECISE	627	0	0	0/0	0/0	0/0	0/1	0/0	0/0	0/0	0/0	0/0	0/0	0/0	0/0	0/0	0/0
Chromosome2-4150707	DEL	PRECISE	276	0	0	0/0	0/1	0/0	0/0	0/1	0/1	0/1	1/1	0/1	1/1	1/1	1/1	1/1	0/1
Chromosome2-4151302	INS	PRECISE	3010	0	0	0/0	0/0	0/0	0/0	0/0	0/0	0/0	0/0	0/0	1/1	0/0	0/0	0/0	0/0
Chromosome2-4151467	DEL	PRECISE	62	0	0	0/0	0/0	0/0	0/0	0/0	0/1	0/0	0/0	0/1	0/0	0/0	0/0	0/0	0/0
Chromosome2-4151620	DEL	PRECISE	446	0	0	0/0	0/1	0/1	0/1	0/1	0/1	0/1	1/1	0/1	1/1	1/1	1/1	1/1	1/1
Chromosome2-4152141	INS	PRECISE	67	0	0.707	0/0	0/0	0/0	0/0	0/0	0/0	0/1	0/0	0/0	0/0	0/0	0/0	0/0	0/1
Chromosome2-4152532	INS	PRECISE	155	0	0	0/0	0/0	0/0	0/0	0/0	0/0	0/1	0/0	0/0	0/0	0/0	0/0	0/0	0/1
Chromosome2-4153395	DEL	PRECISE	487	0	0	0/0	1/1	0/0	0/0	1/1	1/1	0/1	0/0	1/1	0/0	0/0	0/0	0/0	0/1
Chromosome2-4154200	DEL	PRECISE	64	0	0	0/0	0/0	0/0	0/0	0/0	0/0	0/0	0/0	1/1	0/0	0/0	0/0	0/0	1/1
Chromosome2-4154304	DEL	PRECISE	51	0	0	0/0	0/0	0/0	0/0	0/0	0/0	0/0	0/0	0/1	0/0	0/0	0/0	0/1	0/1
Chromosome2-4155630	DEL	PRECISE	284	0	0	0/0	0/1	0/0	0/0	0/1	0/1	0/1	1/1	0/0	0/1	0/1	0/0	0/1	0/0
Chromosome2-4156955	DEL	PRECISE	249	0	0	0/0	0/0	0/1	1/1	0/0	0/0	0/0	0/0	0/0	0/0	0/0	0/0	0/0	0/0
Chromosome2-4157236	INS	PRECISE	6893	0	0	0/0	0/0	0/0	0/0	0/0	0/0	0/0	0/0	0/0	0/0	0/0	0/0	0/0	1/1
Chromosome2-4158763	INS	PRECISE	455	0	0.707	0/0	0/0	0/1	0/1	0/0	0/0	0/0	0/0	0/0	0/0	0/0	0/0	0/0	0/0
Chromosome2-4159096	INS	PRECISE	332	0	0	0/0	0/0	0/0	0/0	0/0	0/0	0/0	0/0	0/0	0/0	0/0	0/0	0/0	0/1
Chromosome2-4160386	INS	PRECISE	9826	0	0	0/0	0/0	0/0	0/0	0/0	0/0	1/1	0/0	0/0	0/0	0/0	0/0	0/0	0/0
Chromosome2-4161104	INS	PRECISE	4906	0	0	0/0	0/0	0/0	0/0	0/0	0/0	0/0	0/0	0/0	0/0	0/0	0/0	0/0	1/1
Chromosome2-4162630	INS	PRECISE	838	0	0	0/0	0/0	0/1	0/1	0/0	0/0	0/0	0/0	0/0	0/0	0/0	0/0	0/0	0/0
Chromosome2-4162727	DEL	PRECISE	53	0	0	0/0	0/1	0/0	0/0	0/1	0/1	0/1	1/1	0/1	1/1	1/1	1/1	1/1	0/0
Chromosome2-4163177	DEL	PRECISE	1293	0	0	0/0	0/1	0/0	0/0	1/1	0/1	0/1	1/1	1/1	1/1	1/1	1/1	1/1	0/1
Chromosome2-4163297	DEL	PRECISE	60	0	0	0/0	0/0	0/0	0/0	0/0	0/1	0/0	0/0	0/1	0/0	0/0	0/0	0/0	0/0
Chromosome2-4163768	DEL	PRECISE	137	0	0	0/0	0/1	0/0	0/0	1/1	0/1	0/1	1/1	0/1	1/1	1/1	1/1	1/1	1/1
Chromosome2-4165685	DEL	PRECISE	54	0	0	0/0	0/1	0/0	0/0	0/1	0/1	0/0	1/1	0/0	1/1	1/1	1/1	1/1	0/1
Chromosome2-4165941	DEL	PRECISE	78	0	0	0/0	0/0	0/0	0/0	0/0	0/0	0/0	0/0	0/0	0/0	0/0	0/0	0/1	0/1
Chromosome2-4166545	INS	PRECISE	244	0	0.447	0/0	0/0	0/0	0/0	0/1	0/0	0/0	1/1	0/1	1/1	1/1	1/1	1/1	0/1
Chromosome2-4167549	INS	PRECISE	223	0	0	0/0	0/0	0/0	0/0	0/0	0/0	0/0	0/0	0/0	0/0	0/0	0/0	0/0	0/1
Chromosome2-4168384	INS	PRECISE	294	0	0.667	0/0	0/1	0/0	0/0	0/1	0/0	0/1	1/1	0/1	1/1	1/1	1/1	1/1	0/1
Chromosome2-4168701	INS	PRECISE	96	0	0	0/0	0/0	0/0	0/0	0/0	0/0	0/0	0/0	0/0	0/0	0/1	0/1	0/0	0/0
Chromosome2-4168745	DEL	PRECISE	875	0	0	0/0	0/0	0/1	0/1	0/0	0/0	0/0	0/0	0/0	0/0	0/0	0/0	0/0	0/0

Chromosome2-4169658 DEL	PRECISE	1621	0	0	0/0	0/0	0/0	0/1	0/0	0/0	1/1	0/0	0/0	0/0	0/0	0/0	0/0	0/0
Chromosome2-4170192 DEL	PRECISE	51	0	0	0/0	0/1	0/0	0/0	0/0	0/0	0/1	1/1	0/0	1/1	1/1	1/1	0/1	0/0
Chromosome2-4171031 INS	PRECISE	211	0	0.447	0/0	0/0	0/0	0/0	0/0	0/0	0/0	0/0	0/0	0/0	0/1	0/1	0/1	0/1
Chromosome2-4172269 DEL	PRECISE	691	0	0	0/0	0/1	0/0	0/0	0/1	0/1	0/1	1/1	0/1	1/1	1/1	1/1	1/1	0/1
Chromosome2-4172702 INS	PRECISE	390	0	0	0/0	0/0	0/0	0/0	0/0	0/0	0/0	0/0	0/0	0/0	0/0	0/0	0/0	0/1
Chromosome2-4172890 DEL	PRECISE	78	0	0	0/0	0/0	0/0	0/0	0/1	0/0	0/0	0/0	0/0	0/0	0/0	0/0	0/0	0/1
Chromosome2-4173779 INS	PRECISE	139	0	0.354	0/0	0/1	0/0	0/0	0/1	0/1	0/1	1/1	0/0	0/1	0/1	0/0	0/1	0/0
Chromosome2-4173872 INS	PRECISE	930	0	0	0/0	0/0	0/1	0/1	0/0	0/0	0/0	0/0	0/0	0/0	0/0	0/0	0/0	0/0
Chromosome2-4175147 DEL	PRECISE	57	0	0	0/0	0/1	0/0	0/0	0/1	0/1	0/1	1/1	0/0	0/1	0/1	0/0	0/1	0/0
Chromosome2-4175767 DEL	PRECISE	67	0	0	0/0	0/0	0/0	0/0	0/0	0/0	0/0	0/0	0/1	0/0	0/1	0/1	0/1	0/1
Chromosome2-4175831 DEL	PRECISE	830	0	0	0/0	0/1	0/0	0/0	0/1	0/1	0/1	1/1	0/0	1/1	1/1	1/1	1/1	0/1
Chromosome2-4176944 INS	PRECISE	106	0	0.577	0/0	0/0	0/0	0/0	0/0	0/0	0/0	0/0	0/1	0/0	0/0	0/0	0/1	0/1
Chromosome2-4177015 INS	PRECISE	417	0	0	0/0	0/0	0/0	0/0	0/0	0/0	0/0	0/0	0/0	0/0	0/0	0/0	0/0	0/1
Chromosome2-4177442 INS	PRECISE	150	0	0	0/0	0/1	0/0	0/0	0/1	0/1	0/1	1/1	0/0	0/1	0/1	0/0	0/1	0/0
Chromosome2-4177492 DEL	PRECISE	80	0	0	0/0	0/0	0/0	0/1	0/0	0/0	0/1	0/0	0/0	0/0	0/0	0/0	0/0	0/0
Chromosome2-4178530 DEL	PRECISE	693	0	0	0/0	0/0	0/0	0/0	0/0	0/0	0/0	0/0	0/1	1/1	1/1	1/1	1/1	0/1
Chromosome2-4178993 DEL	PRECISE	779	0	0	0/0	0/1	0/0	0/0	0/1	0/0	0/1	1/1	0/1	1/1	1/1	1/1	1/1	0/1
Chromosome2-4179319 INS	PRECISE	343	0	0.632	0/0	0/1	0/0	0/0	0/1	0/1	0/1	1/1	0/1	1/1	1/1	1/1	0/1	0/0
Chromosome2-4179847 INS	PRECISE	888	0	0	0/0	0/0	0/0	0/0	0/0	0/0	0/0	0/0	0/0	0/0	0/0	0/0	0/0	0/1
Chromosome2-4181669 INS	PRECISE	1148	0	0	0/0	0/0	0/0	0/0	0/0	0/0	0/0	0/0	0/0	0/0	0/1	0/1	0/0	0/0
Chromosome2-4181791 DEL	PRECISE	81	0	0	0/0	0/0	0/0	0/0	0/0	0/0	0/0	0/0	0/0	0/0	0/0	0/0	0/0	0/1
Chromosome2-4182743 INS	PRECISE	97	0	0.5	0/0	0/0	0/0	0/0	0/0	0/0	0/0	0/0	0/0	0/0	1/1	0/1	0/1	0/1
Chromosome2-4183249 INS	PRECISE	675	0	0.707	0/0	0/0	0/0	0/0	0/0	0/0	0/0	0/0	0/0	0/0	0/1	0/1	0/0	0/0
Chromosome2-4184609 INS	PRECISE	74	0	0	0/0	0/0	0/0	0/0	0/0	0/0	0/0	0/0	0/0	0/0	0/0	0/0	0/0	0/1
Chromosome2-4184694 DEL	PRECISE	825	0	0	0/0	0/1	0/0	0/1	1/1	0/0	0/1	1/1	0/0	1/1	1/1	1/1	1/1	1/1
Chromosome2-4184818 DEL	PRECISE	61	0	0	0/0	0/0	0/0	0/1	0/1	0/0	0/0	0/0	0/0	0/0	0/0	0/0	0/0	0/0
Chromosome2-4185256 DEL	PRECISE	236	0	0	0/0	0/1	0/0	0/0	0/1	0/0	0/1	1/1	0/1	1/1	1/1	1/1	1/1	0/0
Chromosome2-4185754 INS	PRECISE	1548	0	0.707	0/0	0/0	0/0	0/0	0/0	0/0	0/0	0/0	0/0	0/0	0/1	0/1	0/0	0/0
Chromosome2-4186035 DEL	PRECISE	363	0.302	0.302	0/0	0/1	0/0	0/0	0/1	1/1	0/1	1/1	1/1	1/1	1/1	1/1	1/1	1/1
Chromosome2-4186263 DEL	PRECISE	137	0	0	0/0	0/1	0/0	0/0	0/1	0/0	0/1	1/1	0/0	1/1	1/1	1/1	1/1	0/0
Chromosome2-4186441 INS	PRECISE	161	0.333	0.5	0/0	0/1	0/0	0/0	0/1	0/0	0/0	1/1	0/0	1/1	1/1	1/1	0/1	0/0
Chromosome2-4186580 DEL	PRECISE	318	0	0	0/0	0/0	0/0	0/0	0/0	0/0	1/1	0/0	0/0	0/0	0/0	0/0	0/0	0/0
Chromosome2-4186794 DEL	PRECISE	503	0	0	0/0	0/0	0/0	0/0	0/0	0/0	0/0	0/0	0/0	0/0	0/0	0/0	0/0	1/1
Chromosome2-4186804 DUP	PRECISE	2723	0	0	0/0	0/0	0/0	0/0	0/0	0/0	0/0	0/0	0/0	0/1	0/0	0/0	0/0	0/0
Chromosome2-4186913 INS	PRECISE	2738	0	0.707	0/0	0/0	0/0	0/0	0/0	0/0	0/0	0/0	0/0	1/1	0/0	1/1	0/0	0/0
Chromosome2-4187650 DEL	PRECISE	129	0	0	0/0	0/0	0/0	0/0	0/0	0/0	0/0	0/0	0/0	0/1	0/0	1/1	0/0	0/0
Chromosome2-4188626 DEL	PRECISE	61	0	0	0/0	0/0	0/0	0/0	1/1	0/0	0/0	0/0	0/0	0/0	0/0	0/0	0/0	0/0
Chromosome2-4189643 DEL	PRECISE	58	0	0	0/0	0/1	0/0	0/0	0/0	0/1	0/1	1/1	0/0	1/1	1/1	1/1	1/1	0/1
Chromosome2-4189676 INS	PRECISE	996	0	0.707	0/0	0/0	0/0	0/0	0/0	0/1	0/0	0/0	0/1	0/0	0/0	0/0	0/0	0/0
Chromosome2-4190675 INS	PRECISE	942	0	0	0/0	0/0	0/0	0/0	0/0	0/0	0/0	0/0	0/0	0/0	0/0	0/0	0/0	0/1
Chromosome2-4190874 DEL	PRECISE	197	0	0	0/0	0/0	0/0	0/0	0/0	0/0	0/0	0/0	0/0	0/1	0/0	0/1	0/0	0/0
Chromosome2-4191468 DEL	PRECISE	87	0	0	0/0	0/0	0/1	1/1	0/0	0/0	0/0	0/0	0/0	0/0	0/0	0/0	0/0	0/0
Chromosome2-4192408 INS	PRECISE	346	0	0	0/0	0/0	0/0	0/0	0/0	0/0	0/0	0/0	0/0	0/0	0/1	0/1	0/0	0/0
Chromosome2-4192656 INS	PRECISE	705	0	0	0/0	0/0	0/0	0/0	0/0	0/0	0/0	0/0	0/0	0/0	0/0	0/0	0/0	0/1
Chromosome2-4193965 DEL	PRECISE	727	0	0	0/0	0/1	0/0	0/0	0/1	0/1	0/0	1/1	0/1	1/1	1/1	1/1	1/1	1/1
Chromosome2-4194047 DEL	PRECISE	70	0	0	0/0	0/0	0/0	0/0	0/0	0/0	0/0	0/0	0/0	0/0	0/0	0/0	0/0	0/1
Chromosome2-4195735 DEL	PRECISE	281	0.302	0.302	0/0	0/1	0/0	0/0	0/1	0/1	0/1	1/1	0/1	1/1	1/1	1/1	1/1	0/0
Chromosome2-4196439 DEL	PRECISE	905	0	0	0/0	0/1	0/1	1/1	1/1	1/1	1/1	1/1	1/1	1/1	1/1	1/1	1/1	1/1
Chromosome2-4198803 INS	PRECISE	52	0.603	0.674	0/0	0/1	0/0	0/0	0/1	0/1	0/1	1/1	0/1	1/1	1/1	1/1	1/1	0/1
Chromosome2-4199102 INS	PRECISE	161	0	0	0/0	0/0	0/0	0/0	0/0	0/0	0/0	0/0	0/0	0/0	0/1	1/1	0/0	0/0
Chromosome2-4199964 INS	PRECISE	996	0	0.707	0/0	0/0	0/0	0/0	0/0	1/1	0/0	0/0	0/1	0/0	0/0	0/0	0/0	0/0
Chromosome2-4201183 DEL	PRECISE	54	0	0	0/0	0/0	0/0	0/0	0/0	0/0	0/0	0/0	0/1	0/0	0/1	0/1	0/1	0/1
Chromosome2-4203944 INS	PRECISE	75	0	0	0/0	0/0	0/0	0/0	0/0	0/1	0/0	0/1	0/1	0/0	0/1	0/1	0/1	0/0
Chromosome2-4204434 DEL	PRECISE	247	0.905	0.905	0/0	0/1	0/0	0/0	0/1	1/1	0/1	1/1	1/1	1/1	1/1	1/1	1/1	0/1
Chromosome2-4204544 INS	PRECISE	100	0	0	0/0	0/1	0/0	0/0	0/1	1/1	0/1	1/1	1/1	1/1	1/1	1/1	1/1	1/1
Chromosome2-4204575 INS	PRECISE	3325	0	0	0/0	0/0	0/0	0/0	0/0	0/0	0/0	0/0	0/0	0/0	0/0	0/0	0/0	1/1
Chromosome2-4204751 DEL	PRECISE	127	0	0	0/0	0/1	0/0	0/0	0/1	0/1	0/1	1/1	0/0	0/1	0/1	0/0	0/1	0/0
Chromosome2-4205396 INS	PRECISE	86	0	0.302	0/0	0/1	0/0	0/0	0/1	0/1	0/1	1/1	0/1	1/1	0/1	0/1	1/1	0/1
Chromosome2-4205935 DEL	PRECISE	1046	0	0	0/0	0/0	0/0	0/0	0/0	0/0	0/0	0/0	0/0	0/0	0/1	0/1	0/0	0/0
Chromosome2-4207148 DEL	PRECISE	148	0	0	0/0	0/1	0/0	0/0	0/1	0/1	0/1	1/1	0/1	1/1	1/1	1/1	1/1	0/1
Chromosome2-4209251 INS	PRECISE	151	0	0.707	0/0	0/0	0/0	0/0	0/0	0/0	0/0	0/0	0/0	0/0	0/1	0/1	0/0	0/0
Chromosome2-4209602 INS	PRECISE	774	0	0	0/0	0/0	0/0	0/1	0/0	0/0	0/0	0/0	0/0	0/0	0/0	0/0	0/0	0/0
Chromosome2-4209607 DUP	PRECISE	4509	0	0	0/0	0/0	0/0	0/0	0/0	0/1	0/0	0/0	0/1	0/0	0/0	0/0	0/0	0/0
Chromosome2-4209792 DEL	PRECISE	142	0	0	0/0	0/1	0/0	0/0	0/1	0/1	0/1	1/1	0/1	1/1	1/1	1/1	1/1	1/1
Chromosome2-4209939 INS	PRECISE	561	0.333	0.972	0/0	0/1	0/0	0/0	0/1	0/1	0/1	1/1	0/0	1/1	0/1	0/0	1/1	0/1
Chromosome2-4210265 INS	PRECISE	2787	0	0	0/0	0/0	0/0	0/0	1/1	0/0	0/0	0/0	0/0	0/0	0/0	0/0	0/0	0/0
Chromosome2-4211031 DEL	PRECISE	80	0	0	0/0	0/0	0/0	0/0	0/0	0/0	0/0	0/0	0/0	0/0	0/1	1/1	0/0	0/0
Chromosome2-4211364 DEL	PRECISE	841	0	0	0/0	0/1	0/0	0/0	0/1	0/1	0/1	1/1	0/1	1/1	1/1	1/1	1/1	0/0
Chromosome2-4211456 INS	PRECISE	54	0	0	0/0	0/0	0/0	0/0	0/0	0/0	0/0	0/0	0/0	0/0	0/0	0/0	0/0	1/1
Chromosome2-4213060 DEL	PRECISE	246	0	0	0/0	0/0	0/0	0/0	0/0	0/0	0/0	0/0	0/0	0/0	0/0	0/0	0/0	1/1
Chromosome2-4213162 DEL	PRECISE	426	0	0	0/0	0/0	0/1	1/1	0/0	0/0	1/1	0/0	0/1	0/0	1/1	1/1	0/0	0/0
Chromosome2-4214055 INS	PRECISE	17263	0	0	0/0	0/0	0/0	0/1	0/0	0/0	0/0	0/0	0/0	0/0	0/0	0/0	0/0	0/0
Chromosome2-4214056 BND	PRECISE	0	0	0	0/0	0/0	0/0	0/1	0/0	0/0	0/0	0/0	0/0	0/0	0/0	0/0	0/0	0/0

Chromosome2-4214208	DEL	PRECISE	241	0.354	0	0/0	0/1	0/0	0/0	0/1	0/1	0/1	1/1	0/0	1/1	1/1	1/1	0/0	0/0
Chromosome2-4215523	DEL	PRECISE	82	0	0	0/0	0/1	0/0	0/0	0/1	0/1	0/1	1/1	0/1	1/1	1/1	1/1	0/1	0/0
Chromosome2-4216308	INS	PRECISE	150	0	0	0/0	0/0	0/0	0/0	0/0	0/0	0/0	0/0	0/0	0/0	0/0	0/0	0/0	0/1
Chromosome2-4218172	DEL	PRECISE	70	0	0	0/0	0/0	0/0	0/0	0/1	0/1	0/1	1/1	0/1	1/1	1/1	1/1	1/1	0/1
Chromosome2-4218187	INS	PRECISE	9041	0	0	0/0	0/0	0/0	0/0	1/1	0/0	0/0	0/0	0/0	0/0	0/0	0/0	0/0	0/0
Chromosome2-4218809	DEL	PRECISE	416	0	0	0/0	0/0	0/0	0/0	0/0	0/1	0/0	1/1	0/1	1/1	1/1	1/1	1/1	1/1
Chromosome2-4219058	DEL	PRECISE	213	0	0	0/0	0/1	0/0	0/0	0/1	0/1	0/1	1/1	0/1	1/1	1/1	1/1	1/1	1/1
Chromosome2-4219242	DEL	PRECISE	57	0	0	0/0	0/0	0/0	0/0	0/0	0/0	0/0	0/0	0/0	0/0	0/0	0/0	0/1	1/1
Chromosome2-4220746	DEL	PRECISE	69	0	0	0/0	0/1	0/0	0/0	0/1	0/0	0/1	1/1	0/0	0/1	0/1	0/0	0/1	0/0
Chromosome2-4222496	INS	PRECISE	930	0	0	0/0	0/0	0/0	0/0	0/1	0/0	0/0	0/0	0/0	0/0	0/0	0/0	0/0	0/0
Chromosome2-4223362	INS	PRECISE	108	0	0	0/0	0/0	0/0	0/0	1/1	0/0	0/0	0/0	0/0	0/0	0/0	0/0	0/0	0/0
Chromosome2-4223421	INS	PRECISE	789	0	0	0/0	0/0	0/0	0/0	0/0	0/0	0/0	0/0	0/0	0/0	0/0	0/0	0/0	1/1
Chromosome2-4224975	DEL	PRECISE	482	0	0	0/0	0/1	0/0	0/1	1/1	0/1	0/1	1/1	0/1	1/1	1/1	1/1	1/1	0/1
Chromosome2-4225802	DEL	PRECISE	128	0	0	0/0	0/1	0/0	0/0	0/1	0/1	0/1	1/1	0/1	1/1	1/1	1/1	1/1	0/1
Chromosome2-4226638	DEL	PRECISE	138	0	0	0/0	0/1	0/0	0/0	0/1	0/1	0/1	1/1	0/1	1/1	1/1	1/1	1/1	0/1
Chromosome2-4229420	INS	PRECISE	1181	0	0	0/0	0/0	0/0	0/0	0/1	0/0	0/0	0/0	0/0	0/0	0/0	0/0	0/0	0/0
Chromosome2-4229690	DEL	PRECISE	57	0	0	0/0	0/0	0/0	0/0	0/0	0/0	0/0	0/0	0/1	1/1	0/1	1/1	0/0	0/0
Chromosome2-4230092	INS	PRECISE	190	0	0.603	0/0	0/1	0/0	0/0	0/1	0/1	0/1	1/1	0/1	1/1	1/1	1/1	1/1	0/1
Chromosome2-4230143	INS	PRECISE	864	0	0.944	0/0	1/1	0/0	0/0	1/1	1/1	1/1	0/0	0/1	1/1	0/0	0/1	0/0	1/1
Chromosome2-4230199	DEL	PRECISE	93	0	0	0/0	0/0	0/0	0/0	0/0	0/0	0/0	0/0	0/0	0/0	0/0	0/0	0/0	1/1
Chromosome2-4230764	INS	PRECISE	1253	0	0	0/0	0/0	0/0	0/0	0/0	0/0	0/0	0/0	0/0	0/1	0/0	1/1	0/0	0/0
Chromosome2-4231822	INS	PRECISE	146	0	0.905	0/0	0/1	0/0	0/0	0/1	0/1	0/1	1/1	0/1	1/1	1/1	1/1	1/1	0/1
Chromosome2-4233331	DEL	PRECISE	53	0	0	0/0	0/0	0/1	0/1	0/0	0/0	0/0	0/0	0/0	0/0	0/0	0/0	0/0	0/0
Chromosome2-4233413	INS	PRECISE	447	0	0	0/0	0/0	0/0	0/0	0/0	0/0	0/1	0/0	0/0	0/0	0/0	0/0	0/0	0/0
Chromosome2-4233443	DEL	PRECISE	211	0	0	0/0	0/1	0/0	0/0	0/1	0/1	0/1	1/1	0/1	1/1	1/1	1/1	1/1	0/1
Chromosome2-4233675	INS	PRECISE	99	0	0	0/0	0/0	0/0	0/0	0/0	0/0	0/0	0/0	0/0	0/0	0/0	0/0	0/0	0/1
Chromosome2-4233904	INS	PRECISE	11086	0	0	0/0	0/0	0/0	1/1	0/0	0/0	0/0	0/0	0/0	0/0	0/0	0/0	0/0	0/0
Chromosome2-4233989	BND	PRECISE	0	0	0	0/0	0/0	0/1	0/0	0/0	0/1	0/0	0/0	0/1	0/0	0/0	0/0	0/0	0/0
Chromosome2-4234006	INS	PRECISE	53	0	0.378	0/0	0/0	0/0	0/0	0/0	0/0	0/0	1/1	0/0	1/1	0/0	0/0	1/1	1/1
Chromosome2-4234626	DEL	PRECISE	729	0	0	0/0	0/0	0/0	0/0	0/0	0/0	0/0	0/0	0/0	0/0	0/0	0/0	0/0	1/1
Chromosome2-4235035	DEL	PRECISE	120	0	0	0/0	0/1	0/0	0/0	0/1	0/1	0/1	1/1	0/0	1/1	1/1	1/1	0/1	0/0
Chromosome2-4235279	INS	PRECISE	211	0	0	0/0	0/0	0/0	0/1	0/0	0/0	0/0	0/0	0/0	0/0	0/0	0/0	0/0	0/0
Chromosome2-4235379	INS	PRECISE	735	0	0	0/0	0/0	0/0	0/0	0/0	0/0	0/1	0/0	0/0	0/0	0/0	0/0	0/0	0/0
Chromosome2-4235589	INS	PRECISE	53	0	0.934	0/0	0/1	0/0	0/0	0/1	0/1	0/1	1/1	0/1	1/1	1/1	1/1	1/1	0/1
Chromosome2-4236152	INS	PRECISE	4082	0	0	0/0	0/0	0/0	0/0	0/0	0/0	0/0	0/0	0/0	0/1	0/0	0/0	0/0	0/0
Chromosome2-4236927	INS	PRECISE	4021	0	0	0/0	0/0	0/0	0/0	0/0	0/0	0/0	0/0	0/0	0/0	0/0	0/0	0/0	0/1
Chromosome2-4237061	DEL	PRECISE	375	0.603	0.302	0/0	0/1	0/0	0/0	0/1	0/1	0/1	1/1	0/1	1/1	1/1	1/1	1/1	0/1
Chromosome2-4237143	INS	PRECISE	546	0	0	0/0	0/0	0/0	0/0	0/0	0/0	0/0	0/0	0/0	0/0	0/0	0/0	0/0	0/1
Chromosome2-4237408	DEL	PRECISE	50	0	0	0/0	0/1	0/0	0/0	0/1	0/1	0/1	1/1	0/1	1/1	1/1	1/1	1/1	0/1
Chromosome2-4237637	INS	PRECISE	636	0	0.924	0/0	0/1	0/0	0/0	0/1	0/1	0/1	1/1	0/1	1/1	1/1	1/1	1/1	0/1
Chromosome2-4239657	INS	PRECISE	57	0	0.535	0/0	0/1	0/0	0/0	0/1	0/1	0/1	1/1	0/0	0/1	0/1	0/0	0/1	0/0
Chromosome2-4240034	INS	PRECISE	73	0	0.316	0/0	0/1	0/0	0/0	0/1	0/1	0/1	1/1	0/1	0/1	1/1	0/1	0/1	0/0
Chromosome2-4240221	DEL	PRECISE	478	0	0	0/0	0/1	0/0	0/0	0/1	0/1	0/1	1/1	0/1	1/1	1/1	1/1	1/1	1/1
Chromosome2-4240750	INS	PRECISE	51	0	0.316	0/0	0/1	0/0	0/0	0/1	0/1	0/1	1/1	0/1	0/1	1/1	0/1	0/1	0/0
Chromosome2-4241224	INS	PRECISE	98	0	0	0/0	0/0	0/0	0/0	0/1	0/0	0/0	0/0	0/0	0/0	0/0	0/0	0/0	0/0
Chromosome2-4241395	INS	PRECISE	365	0	0.753	0/0	0/0	0/0	0/0	0/0	0/0	0/0	0/0	0/1	0/1	0/1	1/1	0/1	0/1
Chromosome2-4241662	INS	PRECISE	59	0	0	0/0	0/0	0/0	0/0	0/0	0/1	0/0	0/0	0/1	0/0	0/0	0/0	0/0	0/0
Chromosome2-4242299	DEL	PRECISE	352	0	0	0/0	0/1	0/0	0/0	0/1	1/1	0/1	1/1	1/1	1/1	1/1	1/1	1/1	1/1
Chromosome2-4244812	INS	PRECISE	50	0	0	0/0	0/0	0/0	0/0	0/0	0/0	0/0	0/0	0/0	0/0	0/0	0/0	0/0	0/1
Chromosome2-4245226	INS	PRECISE	211	0	0	0/0	0/0	0/1	1/1	0/0	0/1	0/0	0/0	0/1	0/0	0/0	0/0	0/0	0/0
Chromosome2-4245273	DEL	PRECISE	3386	0	0	0/0	0/1	0/0	0/0	0/1	0/1	0/1	1/1	0/1	1/1	1/1	1/1	1/1	0/1
Chromosome2-4245362	DEL	PRECISE	472	0	0	0/0	0/0	0/1	1/1	0/0	0/1	0/0	0/0	0/1	0/0	0/0	0/0	0/0	0/0
Chromosome2-4245617	DEL	PRECISE	110	0	0	0/0	0/0	0/1	1/1	0/0	0/1	0/0	0/0	0/1	0/0	0/0	0/0	0/0	0/0
Chromosome2-4246321	INS	PRECISE	1394	0	0	0/0	0/0	0/0	0/0	1/1	0/0	0/0	0/0	0/0	0/0	0/0	0/0	0/0	0/0
Chromosome2-4246885	BND	PRECISE	0	0	0	0/0	0/0	0/0	0/0	0/0	0/0	0/0	0/0	0/0	0/1	0/0	0/0	0/0	0/0
Chromosome2-4247257	DEL	PRECISE	161	0	0	0/0	0/1	0/0	0/0	0/1	0/1	0/1	1/1	0/1	1/1	1/1	1/1	1/1	0/1
Chromosome2-4247346	INS	PRECISE	315	0	0	0/0	0/0	0/0	0/0	0/0	0/0	0/0	0/0	0/0	0/0	0/0	0/0	0/1	0/1
Chromosome2-4248174	DEL	PRECISE	9961	0	0	0/0	0/0	0/0	0/0	1/1	0/0	0/0	0/0	0/0	0/0	0/0	0/0	0/0	0/0
Chromosome2-4248175	BND	PRECISE	0	0	0	0/0	0/0	0/0	0/1	0/1	0/1	0/0	1/1	0/1	0/1	0/0	0/1	0/0	0/0
Chromosome2-4248233	INS	PRECISE	86	0	0.378	0/0	0/0	0/0	0/1	0/1	1/1	0/0	1/1	0/1	1/1	0/0	1/1	0/0	0/0
Chromosome2-4249168	INS	PRECISE	852	0	0.577	0/0	0/0	0/1	1/1	0/0	0/0	0/0	0/0	0/0	0/0	0/0	0/0	0/0	1/1
Chromosome2-4249170	BND	PRECISE	0	0	0	0/0	0/0	0/0	1/1	1/1	0/0	0/0	0/0	1/1	0/0	0/0	0/0	0/0	0/0
Chromosome2-4251035	BND	PRECISE	0	0	0	0/0	0/0	0/1	1/1	0/0	0/0	0/0	0/0	0/0	0/0	0/0	0/0	0/0	0/0
Chromosome2-4251036	BND	PRECISE	0	0	0	0/0	0/0	0/1	1/1	0/0	0/0	0/0	0/0	0/0	0/0	0/0	0/0	0/0	0/0
Chromosome2-4252163	INS	PRECISE	112	0	0	0/0	0/0	0/0	0/0	0/0	0/0	0/0	0/0	0/0	0/1	0/0	0/0	0/0	0/0
Chromosome2-4252333	DEL	PRECISE	634	0	0	0/0	0/0	0/0	0/0	0/0	0/0	0/0	0/0	0/0	1/1	1/1	1/1	1/1	0/1
Chromosome2-4252785	DEL	PRECISE	363	0	0	0/0	0/1	0/0	0/0	0/1	0/0	0/1	1/1	0/1	1/1	1/1	1/1	1/1	0/0
Chromosome2-4252976	INS	PRECISE	346	0	0	0/0	0/0	0/0	0/0	0/0	0/0	0/0	0/0	0/0	0/0	0/0	0/0	0/0	1/1
Chromosome2-4253304	INS	PRECISE	854	0.333	0.972	0/0	0/1	0/0	0/0	0/1	0/1	0/1	1/1	0/0	0/1	1/1	0/0	1/1	0/0
Chromosome2-4254458	DEL	PRECISE	348	0.302	0.302	0/0	0/1	0/0	0/0	0/1	0/1	0/1	1/1	0/1	1/1	1/1	1/1	1/1	1/1
Chromosome2-4254891	DEL	PRECISE	222	0	0	0/0	0/0	0/0	0/0	0/0	0/0	0/0	0/0	0/0	0/1	0/0	0/1	0/0	0/0
Chromosome2-4255254	INS	PRECISE	2825	0	0	0/0	0/0	0/0	0/0	0/0	0/0	0/0	0/0	0/0	0/0	0/0	0/0	1/1	0/0
Chromosome2-4255407	INS	PRECISE	1180	0	0	0/0	0/0	0/0	0/0	0/0	0/0	0/0	0/0	0/0	0/0	0/0	0/0	0/0	0/1

Chromosome2-4255714	INS	PRECISE	987	0	0	0/0	0/0	0/0	0/0	0/0	0/0	0/0	0/0	0/0	0/1	0/0	0/1	0/0	0/0
Chromosome2-4255865	DEL	PRECISE	233	0	0	0/0	0/0	0/0	0/0	0/0	0/0	0/0	0/0	0/1	0/0	0/1	0/1	0/0	0/1
Chromosome2-4257289	DEL	PRECISE	273	0	0	0/0	0/0	0/0	0/0	0/0	0/0	0/0	0/0	0/0	0/0	0/0	0/0	0/0	0/1
Chromosome2-4258039	DEL	PRECISE	349	0.603	0	0/0	0/1	0/0	0/0	0/1	0/1	0/1	1/1	0/1	1/1	1/1	1/1	1/1	0/1
Chromosome2-4258234	DEL	PRECISE	58	0	0	0/0	0/1	0/0	0/0	0/1	0/1	0/1	1/1	0/1	1/1	1/1	1/1	1/1	0/1
Chromosome2-4260601	INS	PRECISE	58	0	0	0/0	0/0	0/0	0/0	0/0	0/0	0/0	0/0	0/0	0/0	0/1	0/1	0/0	0/1
Chromosome2-4260787	INS	PRECISE	68	0	0.707	0/0	0/0	0/0	0/0	0/1	0/0	0/0	0/0	0/0	0/0	0/0	0/0	0/0	0/0
Chromosome2-4260898	DEL	PRECISE	64	0	0	0/0	0/0	0/0	0/0	0/0	0/0	0/0	0/0	0/0	0/1	0/0	0/1	0/0	0/0
Chromosome2-4261182	INS	PRECISE	233	0	0	0/0	0/0	0/0	0/0	0/0	0/0	0/0	0/0	0/0	0/0	0/0	0/0	0/1	0/0
Chromosome2-4261507	INS	PRECISE	1148	0	0	0/0	0/0	0/0	0/0	0/0	0/0	0/0	0/0	0/0	0/0	0/0	0/0	0/0	0/1
Chromosome2-4262115	DEL	PRECISE	55	0	0	0/0	0/0	0/0	0/0	0/0	0/0	0/0	0/0	0/1	0/0	0/1	0/1	0/0	0/1
Chromosome2-4262706	DEL	PRECISE	99	0	0	0/0	0/0	0/0	0/0	0/0	0/0	0/0	0/0	0/0	0/0	0/0	0/0	0/0	0/1
Chromosome2-4263645	DEL	PRECISE	53	0	0	0/0	0/1	0/0	0/0	0/1	0/1	0/1	1/1	0/1	1/1	1/1	1/1	1/1	0/1
Chromosome2-4264144	INS	PRECISE	228	0	0.707	0/0	0/0	0/0	0/0	0/0	0/0	0/0	0/0	0/0	0/1	0/0	0/0	0/0	0/0
Chromosome2-4265479	INS	PRECISE	64	0	0	0/0	0/1	0/0	0/0	0/1	0/1	0/1	1/1	0/0	0/1	0/1	0/0	0/1	0/0
Chromosome2-4265975	DEL	PRECISE	565	0	0	0/0	0/1	0/0	0/0	0/1	0/1	0/1	1/1	0/1	1/1	1/1	1/1	1/1	0/1
Chromosome2-4266242	DEL	PRECISE	2364	0	0	0/0	0/1	0/0	0/0	0/1	0/1	0/1	1/1	0/1	1/1	1/1	1/1	1/1	0/1
Chromosome2-4266306	DEL	PRECISE	1454	0	0	0/0	0/0	0/0	0/0	0/0	0/1	0/0	0/0	0/1	0/0	0/0	0/0	0/0	0/0
Chromosome2-4266517	DEL	PRECISE	62	0	0	0/0	0/1	0/0	0/0	0/1	1/1	0/1	1/1	1/1	1/1	1/1	1/1	1/1	0/1
Chromosome2-4268255	DEL	PRECISE	612	0	0	0/0	0/1	0/0	0/0	0/1	0/1	0/1	1/1	0/1	1/1	1/1	1/1	1/1	1/1
Chromosome2-4268621	INS	PRECISE	4997	0	0	0/0	0/0	0/0	0/0	0/0	0/0	0/1	0/0	0/0	0/0	0/0	0/0	0/0	0/0
Chromosome2-4269148	DEL	PRECISE	799	0	0	0/0	0/1	0/0	0/0	0/1	0/1	0/1	1/1	0/1	1/1	1/1	1/1	1/1	0/1
Chromosome2-4269290	DEL	PRECISE	984	0	0	0/0	0/1	0/0	0/0	0/1	0/1	0/1	1/1	0/1	1/1	1/1	1/1	1/1	0/1
Chromosome2-4269341	DEL	PRECISE	124	0	0	0/0	0/0	0/0	0/0	0/0	0/0	0/0	0/0	0/0	0/0	0/0	0/0	0/0	0/1
Chromosome2-4270181	DEL	PRECISE	85	0	0	0/0	0/1	0/0	0/0	0/0	0/1	0/1	1/1	0/1	1/1	1/1	1/1	1/1	0/1
Chromosome2-4270471	DEL	PRECISE	66	0	0	0/0	0/0	0/0	0/0	0/0	0/0	0/0	0/0	0/0	0/0	0/0	0/0	0/0	1/1
Chromosome2-4271080	INS	PRECISE	150	0	0	0/0	0/0	0/1	1/1	0/0	0/0	0/0	0/0	0/0	0/0	0/0	0/0	0/0	0/0
Chromosome2-4271214	DEL	PRECISE	60	0	0	0/0	0/1	0/0	0/0	0/1	0/1	0/1	1/1	0/1	1/1	1/1	1/1	1/1	0/1
Chromosome2-4271707	DEL	PRECISE	338	0	0	0/0	0/1	0/0	0/0	0/1	0/1	0/1	1/1	0/1	1/1	1/1	1/1	1/1	0/1
Chromosome2-4271752	INS	PRECISE	402	0	0.577	0/0	0/0	0/0	0/0	0/0	0/0	0/0	0/0	0/1	0/0	0/1	0/1	0/0	0/0
Chromosome2-4271970	INS	PRECISE	460	0	0	0/0	0/0	0/0	0/0	0/1	0/0	0/0	0/0	0/0	0/0	0/0	0/0	0/0	0/0
Chromosome2-4272312	DEL	PRECISE	116	0	0	0/0	0/0	0/0	0/0	0/0	0/0	0/0	0/0	0/1	0/0	0/1	1/1	0/0	0/0
Chromosome2-4272539	INS	PRECISE	708	0	0.837	0/0	0/0	0/0	0/0	0/1	0/0	0/1	0/0	0/0	0/1	0/1	0/0	0/0	0/0
Chromosome2-4273426	INS	PRECISE	242	0	0.707	0/0	0/0	0/0	0/0	0/0	0/1	0/0	0/0	0/1	0/0	0/0	0/0	0/0	0/0
Chromosome2-4273489	DEL	PRECISE	62	0	0	0/0	0/0	0/0	0/0	0/0	0/0	0/0	0/0	0/0	0/0	0/0	0/0	0/0	0/1
Chromosome2-4273623	INS	PRECISE	997	0	0	0/0	0/0	0/0	0/0	1/1	0/0	0/0	0/0	0/0	0/0	0/0	0/0	0/0	0/0
Chromosome2-4273716	INS	PRECISE	5450	0	0	0/0	0/0	0/0	0/0	0/0	0/0	0/0	0/0	0/0	0/0	0/0	0/0	0/0	0/1
Chromosome2-4273717	BND	PRECISE	0	0	0	0/0	0/0	0/0	0/0	0/0	0/0	0/0	0/0	0/0	0/0	0/0	0/0	0/0	0/1
Chromosome2-4274037	INS	PRECISE	104	0	0	0/0	0/0	0/0	0/0	0/0	0/0	0/0	0/0	0/0	0/0	0/0	0/0	0/0	0/1
Chromosome2-4274429	DEL	PRECISE	172	0	0	0/0	0/1	0/0	0/0	0/1	0/1	0/1	1/1	0/1	1/1	1/1	1/1	1/1	0/1
Chromosome2-4274471	DEL	PRECISE	994	0	0	0/0	0/1	0/0	0/0	0/1	0/1	0/1	1/1	0/1	1/1	1/1	1/1	1/1	0/1
Chromosome2-4274652	INS	PRECISE	5986	0	0	0/0	0/0	0/0	0/0	0/0	0/0	0/0	0/0	0/0	0/0	0/0	0/0	0/0	1/1
Chromosome2-4274728	DEL	PRECISE	392	0.577	0.577	0/0	0/0	0/0	0/0	0/0	0/0	0/0	0/0	0/1	0/0	0/1	0/1	0/0	0/0
Chromosome2-4275523	DEL	PRECISE	1216	0	0	0/0	0/1	0/0	0/0	0/1	0/1	0/1	1/1	0/1	1/1	1/1	1/1	1/1	0/1
Chromosome2-4275860	INS	PRECISE	159	0	0	0/0	0/0	0/0	0/0	0/0	0/0	0/0	0/0	0/0	0/0	0/0	0/0	0/0	0/1
Chromosome2-4276139	INS	PRECISE	85	0	0	0/0	0/0	0/0	0/0	0/0	0/0	0/0	0/0	0/0	1/1	0/0	0/1	0/0	0/0
Chromosome2-4276240	INS	PRECISE	85	0	0	0/0	0/0	0/0	0/0	0/0	0/0	0/0	0/0	0/0	0/0	0/0	0/0	0/1	0/0
Chromosome2-4276270	DEL	PRECISE	379	0	0	0/0	0/1	0/0	0/0	0/1	0/1	0/1	1/1	0/1	1/1	1/1	1/1	1/1	0/1
Chromosome2-4276907	DEL	PRECISE	564	0	0	0/0	0/0	0/0	0/0	0/0	0/0	0/0	0/0	0/1	0/0	0/1	0/1	0/0	0/0
Chromosome2-4277102	DEL	PRECISE	55	0	0	0/0	0/0	0/0	0/0	0/0	0/0	0/0	0/0	0/0	0/1	0/0	0/1	0/0	0/1
Chromosome2-4277725	INS	PRECISE	217	0	0	0/0	0/0	0/0	0/0	0/0	0/0	0/0	0/0	0/0	0/1	0/0	0/1	0/0	0/0
Chromosome2-4277849	INS	PRECISE	252	0	0	0/0	0/0	0/0	0/0	0/0	0/0	0/0	0/0	0/0	0/0	0/0	0/0	0/1	0/0
Chromosome2-4277904	DEL	PRECISE	65	0	0	0/0	0/1	0/0	0/0	0/1	0/1	0/1	1/1	0/1	1/1	1/1	1/1	1/1	0/1
Chromosome2-4279908	DEL	PRECISE	86	0	0	0/0	0/1	0/0	0/0	0/1	0/0	0/1	1/1	0/1	1/1	1/1	1/1	1/1	0/1
Chromosome2-4280709	INS	PRECISE	8560	0	0	0/0	0/0	0/0	0/0	0/0	0/0	0/0	0/0	0/0	0/0	0/0	0/0	0/0	1/1
Chromosome2-4280710	BND	PRECISE	0	0	0	0/0	0/0	0/0	0/0	0/0	0/0	0/0	0/0	0/0	0/0	0/0	0/0	0/0	0/1
Chromosome2-4281087	DEL	PRECISE	355	0	0	0/0	0/1	0/0	0/0	0/1	0/0	0/1	1/1	0/1	1/1	1/1	1/1	1/1	0/1
Chromosome2-4281238	DEL	PRECISE	165	0	0	0/0	0/0	0/0	0/0	0/0	0/0	0/0	0/0	0/0	0/1	0/0	0/1	0/0	0/0
Chromosome2-4283097	DEL	PRECISE	213	0	0	0/0	0/0	0/0	0/0	0/0	0/0	0/0	0/0	0/0	0/0	0/0	0/1	0/0	0/0
Chromosome2-4283262	INS	PRECISE	170	0	0	0/0	0/0	0/0	0/0	0/0	0/0	0/0	0/0	0/0	0/0	0/0	0/1	0/0	0/0
Chromosome2-4284312	DEL	PRECISE	619	0	0	0/0	1/1	0/0	0/0	1/1	1/1	0/1	0/0	0/0	0/0	0/0	0/0	0/0	0/1
Chromosome2-4284516	DEL	PRECISE	51	0	0	0/0	0/0	0/0	0/0	0/0	0/0	0/0	0/0	0/0	0/0	0/0	0/0	0/0	0/1
Chromosome2-4284662	INS	PRECISE	222	0	0	0/0	0/0	0/0	0/0	0/0	0/1	0/0	0/0	0/1	0/0	0/0	0/0	0/0	0/0
Chromosome2-4285394	BND	PRECISE	0	0	0	0/0	0/0	0/0	0/0	0/0	0/0	0/0	0/0	0/1	0/0	0/1	0/0	0/0	0/0
Chromosome2-4285510	DUP	PRECISE	88	0	0	0/0	0/0	0/0	0/0	0/0	0/0	0/0	0/0	0/0	0/0	0/0	0/0	0/0	0/1
Chromosome2-4285596	INS	PRECISE	863	0	0	0/0	0/0	0/0	0/0	0/1	0/0	0/0	0/0	0/0	0/0	0/0	0/0	0/0	0/0
Chromosome2-4286253	DEL	PRECISE	1156	0.577	0	0/0	0/0	0/0	0/0	0/0	0/0	0/0	0/0	0/1	0/0	0/1	0/0	1/1	0/0
Chromosome2-4286401	INS	PRECISE	355	0	0.577	0/0	0/0	0/0	0/0	0/0	0/0	0/0	0/0	0/0	1/1	0/0	1/1	0/0	0/0
Chromosome2-4286695	INS	PRECISE	8948	0	0	0/0	0/0	0/0	0/0	0/0	0/0	0/0	0/0	0/0	0/0	0/0	0/0	0/0	1/1
Chromosome2-4286695	BND	PRECISE	0	0	0	0/0	0/0	0/0	0/0	0/0	0/0	0/0	0/0	0/0	0/0	0/0	0/0	0/0	0/1
Chromosome2-4286758	DEL	PRECISE	180	0	0	0/0	0/1	0/0	0/0	1/1	1/1	1/1	1/1	1/1	1/1	1/1	1/1	1/1	0/1
Chromosome2-4287290	INS	PRECISE	1307	0	0	0/0	0/0	0/0	0/0	0/0	0/0	0/0	0/0	0/0	0/0	0/0	0/1	0/0	0/0
Chromosome2-4289959	DEL	PRECISE	276	0	0	0/0	0/1	0/0	0/0	0/1	0/1	1/1	1/1	0/1	1/1	1/1	1/1	1/1	0/1

Chromosome2-4290039	DEL	PRECISE	965	0	0	0/0	0/0	0/0	0/0	0/0	0/0	0/0	0/0	0/0	0/0	0/0	0/0	0/0	0/1
Chromosome2-4290051	INS	PRECISE	252	0	0.707	0/0	0/0	0/0	0/0	0/0	0/0	0/0	0/0	0/0	0/1	0/0	0/1	0/0	0/0
Chromosome2-4290662	DEL	PRECISE	160	0	0	0/0	0/1	0/1	1/1	0/1	1/1	0/1	1/1	1/1	1/1	1/1	1/1	1/1	1/1
Chromosome2-4292256	DEL	PRECISE	227	0	0	0/0	0/0	0/0	0/0	0/0	0/0	0/0	0/0	0/0	0/0	0/0	0/0	0/0	0/1
Chromosome2-4292525	INS	PRECISE	137	0	0	0/0	0/0	0/0	0/0	0/1	0/0	0/0	0/0	0/0	0/0	0/0	0/0	0/0	0/0
Chromosome2-4295643	DEL	PRECISE	403	0	0	0/0	0/1	0/0	0/0	0/1	0/1	0/1	1/1	0/0	0/1	1/1	0/1	0/1	0/1
Chromosome2-4296253	INS	PRECISE	322	0	0.809	0/0	0/1	0/0	0/0	0/1	1/1	0/1	1/1	0/0	1/1	1/1	1/1	1/1	0/1
Chromosome2-4296268	DEL	PRECISE	84	0	0	0/0	0/1	0/0	0/0	0/1	1/1	0/1	1/1	0/0	0/1	1/1	0/0	0/1	0/0
Chromosome2-4296521	DEL	PRECISE	251	0.422	0.699	0/0	0/1	0/0	0/0	0/1	1/1	0/1	1/1	0/1	0/1	1/1	0/1	1/1	0/0
Chromosome2-4296874	DEL	PRECISE	108	0	0	0/0	0/0	0/0	0/0	0/0	0/0	0/0	0/0	0/0	0/0	0/0	0/0	0/0	0/1
Chromosome2-4296895	INS	PRECISE	152	0	0	0/0	0/0	0/0	0/0	0/0	0/0	0/0	0/0	0/0	0/0	0/0	0/0	0/0	0/1
Chromosome2-4296996	DEL	PRECISE	53	0	0	0/0	0/0	0/0	0/0	0/0	0/0	0/0	0/0	0/0	0/0	0/0	0/0	0/0	0/1
Chromosome2-4297042	INS	PRECISE	517	0	0	0/0	0/0	0/0	0/0	0/0	0/0	0/1	0/0	0/0	0/0	0/0	0/0	0/0	0/0
Chromosome2-4297057	INS	PRECISE	1983	0	0	0/0	0/0	0/0	0/0	0/0	0/0	0/0	0/0	0/0	0/0	0/0	0/0	0/0	0/1
Chromosome2-4297573	INS	PRECISE	1040	0	0	0/0	0/0	0/0	0/0	0/1	0/0	0/0	0/0	0/0	0/0	0/0	0/0	0/0	0/0
Chromosome2-4297756	DEL	PRECISE	66	0	0	0/0	0/1	0/0	0/0	0/1	0/1	0/1	1/1	0/1	1/1	1/1	1/1	1/1	0/1
Chromosome2-4297771	INS	PRECISE	540	0	0	0/0	0/0	0/0	0/0	0/0	0/0	0/0	0/0	0/0	0/0	0/0	0/0	0/0	0/1
Chromosome2-4297881	BND	PRECISE	0	0	0	0/0	0/0	0/0	0/0	0/0	0/1	0/0	0/1	0/0	0/0	0/0	0/0	0/0	0/0
Chromosome2-4298489	DEL	PRECISE	236	0	0	0/0	0/1	0/0	0/0	0/1	0/1	0/1	1/1	0/1	1/1	1/1	1/1	1/1	0/1
Chromosome2-4299497	DEL	PRECISE	950	0	0	0/0	0/0	0/0	0/0	0/0	0/0	0/0	0/0	0/0	1/1	0/0	0/1	0/0	0/0
Chromosome2-4299521	DEL	PRECISE	51	0	0	0/0	0/0	0/0	0/0	0/0	0/0	0/0	0/0	0/1	0/0	1/1	0/1	1/1	0/1
Chromosome2-4299579	INS	PRECISE	10980	0	0	0/0	0/0	0/0	0/0	0/0	0/0	0/0	0/0	0/0	0/0	0/0	0/0	0/0	0/1
Chromosome2-4299654	DEL	PRECISE	226	0	0	0/0	0/1	0/0	0/0	0/1	0/1	0/1	1/1	0/1	1/1	1/1	1/1	1/1	0/1
Chromosome2-4299921	DEL	PRECISE	184	0.302	0.674	0/0	0/1	0/0	0/0	0/1	0/1	0/1	1/1	0/1	1/1	1/1	1/1	1/1	0/1
Chromosome2-4302105	INS	PRECISE	75	0	0.833	0/0	0/0	0/0	0/0	0/1	0/1	0/1	1/1	0/0	1/1	1/1	1/1	1/1	0/0
Chromosome2-4302577	INS	PRECISE	926	0	0	0/0	0/0	0/0	0/0	0/0	0/0	0/1	0/0	0/0	0/0	0/0	0/0	0/0	0/0
Chromosome2-4303130	INS	PRECISE	2984	0	0	0/0	0/0	0/0	0/0	0/0	0/0	0/0	0/0	0/0	0/0	0/0	0/0	0/0	1/1
Chromosome2-4303205	DEL	PRECISE	54	0	0	0/0	0/0	0/0	0/0	0/0	0/0	0/0	0/0	0/0	0/0	0/0	0/0	0/0	0/1
Chromosome2-4303662	DEL	PRECISE	58	0	0	0/0	0/1	0/0	0/0	0/1	0/1	0/1	1/1	0/1	1/1	1/1	0/1	0/1	0/0
Chromosome2-4303770	INS	PRECISE	197	0	0	0/0	0/0	0/0	0/0	0/0	0/0	0/0	0/0	0/0	0/0	0/0	0/0	0/0	0/1
Chromosome2-4304539	INS	PRECISE	235	0	0	0/0	0/0	0/0	0/0	0/1	0/0	0/0	0/0	0/0	0/0	0/0	0/0	0/0	0/0
Chromosome2-4305423	INS	PRECISE	74	0	0	0/0	0/0	0/0	0/0	0/0	0/0	0/0	0/0	0/0	0/0	0/0	0/1	0/0	0/0
Chromosome2-4305524	INS	PRECISE	335	0	0.707	0/0	0/0	0/0	0/0	0/0	0/0	0/0	0/0	0/0	0/1	0/0	0/1	0/0	0/0
Chromosome2-4306562	DEL	PRECISE	137	0	0	0/0	0/1	0/1	1/1	1/1	0/1	1/1	1/1	0/1	1/1	1/1	1/1	1/1	1/1
Chromosome2-4309776	INS	PRECISE	9475	0	0	0/0	0/0	0/0	0/0	0/0	0/0	0/0	0/0	0/0	0/0	0/0	0/0	0/0	1/1
Chromosome2-4309989	INS	PRECISE	997	0	0	0/0	0/0	0/1	1/1	0/0	0/0	0/0	0/0	0/0	0/0	0/0	0/0	0/0	0/0
Chromosome2-4310448	INS	PRECISE	997	0	0	0/0	0/0	0/1	1/1	0/0	0/0	0/0	0/0	0/0	0/0	0/0	0/0	0/0	0/0
Chromosome2-4311404	INS	PRECISE	445	0	0	0/0	0/0	0/0	0/0	0/0	0/0	0/0	0/0	0/0	0/0	0/0	0/0	0/0	0/1
Chromosome2-4311488	DEL	PRECISE	957	0	0	0/0	0/0	0/0	0/0	0/0	0/0	0/0	0/0	0/0	0/0	0/0	0/0	0/0	0/1
Chromosome2-4312356	INS	PRECISE	696	0	0	0/0	0/0	0/0	0/0	0/0	0/0	0/0	0/0	0/0	0/0	0/0	0/0	0/1	0/0
Chromosome2-4312425	DEL	PRECISE	709	0	0	0/0	0/1	0/0	0/0	0/1	0/1	0/1	1/1	0/1	1/1	1/1	1/1	1/1	0/1
Chromosome2-4312976	INS	PRECISE	546	0	0.577	0/0	0/0	0/0	0/0	0/1	0/1	0/0	0/0	0/1	0/0	0/0	0/0	0/0	0/0
Chromosome2-4313517	DEL	PRECISE	63	0	0	0/0	0/0	0/0	0/0	1/1	0/0	0/0	0/0	0/0	0/0	0/0	0/0	0/0	1/1
Chromosome2-4313986	INS	PRECISE	139	0	0.707	0/0	0/0	0/0	0/0	0/0	0/0	0/0	0/0	0/1	0/0	0/0	0/0	0/0	0/0
Chromosome2-4314039	INS	PRECISE	1102	0	0.835	0/0	0/1	0/0	0/0	0/1	0/1	0/1	1/1	0/0	0/1	1/1	0/0	1/1	0/0
Chromosome2-4314218	INS	PRECISE	87	0	0.316	0/0	0/1	0/0	0/0	0/1	0/1	0/1	1/1	0/1	1/1	1/1	1/1	1/1	0/0
Chromosome2-4314491	DEL	PRECISE	521	0	0	0/0	0/1	0/0	0/0	0/1	0/1	0/1	1/1	0/1	1/1	1/1	1/1	1/1	0/1
Chromosome2-4315329	DEL	PRECISE	82	0	0	0/0	0/1	0/0	0/0	0/1	0/1	0/1	1/1	0/1	1/1	1/1	1/1	1/1	0/1
Chromosome2-4316962	INS	PRECISE	531	0	0	0/0	0/0	0/0	0/0	0/1	0/0	0/0	0/0	0/0	0/0	0/0	0/0	0/0	0/0
Chromosome2-4317092	DEL	PRECISE	96	0.447	0.447	0/0	0/0	0/1	1/1	0/0	0/1	0/0	0/0	0/1	0/0	0/0	0/0	0/0	0/1
Chromosome2-4318850	DEL	PRECISE	138	0	0	0/0	0/0	0/0	0/0	0/0	0/0	0/0	0/0	0/1	0/0	0/1	0/0	0/0	0/0
Chromosome2-4319033	DEL	PRECISE	265	0	0	0/0	0/0	0/0	0/0	0/1	0/1	0/1	1/1	0/1	1/1	1/1	1/1	1/1	0/1
Chromosome2-4319154	BND	PRECISE	0	0	0	0/0	0/0	0/0	0/0	0/0	0/0	0/0	0/0	0/0	0/0	0/0	0/0	0/1	0/0
Chromosome2-4319734	INS	PRECISE	112	0	0.603	0/0	0/0	0/0	0/0	0/1	0/1	0/1	1/1	0/1	1/1	1/1	1/1	1/1	0/1
Chromosome2-4319819	DEL	PRECISE	105	0	0	0/0	0/1	0/0	0/0	0/1	0/1	0/1	1/1	0/1	1/1	1/1	1/1	1/1	0/1
Chromosome2-4320187	DEL	PRECISE	63	0	0	0/0	0/0	0/0	0/0	0/0	0/0	0/0	0/0	0/0	0/0	0/0	0/1	0/1	0/0
Chromosome2-4320768	DEL	PRECISE	868	0	0	0/0	1/1	0/0	0/0	1/1	1/1	1/1	1/1	1/1	1/1	1/1	0/0	0/0	1/1
Chromosome2-4320845	DEL	PRECISE	68	0	0	0/0	0/0	0/0	0/0	0/0	1/1	1/1	0/0	1/1	0/0	0/0	0/0	0/0	0/0
Chromosome2-4321176	DEL	PRECISE	57	0	0	0/0	0/0	0/0	0/0	0/0	0/0	0/0	0/0	0/0	0/0	0/0	0/0	0/0	0/1
Chromosome2-4321735	INS	PRECISE	1645	0	0	0/0	0/0	0/0	0/0	0/0	0/0	0/0	0/0	0/0	0/0	0/0	0/0	0/0	1/1
Chromosome2-4321839	DEL	PRECISE	140	0	0	0/0	0/0	0/0	0/0	0/1	0/0	0/0	1/1	0/1	1/1	1/1	1/1	1/1	0/1
Chromosome2-4322395	DEL	PRECISE	834	0.302	0.302	0/0	0/1	0/0	0/0	0/1	0/0	0/1	1/1	0/1	1/1	1/1	1/1	1/1	0/1
Chromosome2-4322827	INS	PRECISE	1878	0	0	0/0	0/0	0/0	0/0	0/0	0/0	0/1	0/0	0/0	0/0	0/0	0/0	0/0	0/0
Chromosome2-4323950	INS	PRECISE	150	0	0	0/0	0/0	0/0	0/0	0/0	0/0	0/0	0/0	0/0	0/0	0/0	0/0	0/1	0/0
Chromosome2-4323953	DEL	PRECISE	72	0	0	0/0	0/1	0/0	0/0	0/1	0/1	0/1	1/1	0/1	1/1	1/1	1/1	0/1	0/0
Chromosome2-4324313	INS	PRECISE	379	0	0	0/0	0/0	0/0	0/0	0/0	0/0	0/0	0/0	0/0	0/0	0/0	0/0	0/0	0/1
Chromosome2-4324337	DEL	PRECISE	86	0	0	0/0	0/0	0/0	0/0	0/0	0/0	0/0	0/0	0/0	0/0	0/0	0/0	0/0	0/1
Chromosome2-4324457	DEL	PRECISE	82	0	0	0/0	0/0	0/0	0/0	0/0	0/0	0/0	0/0	0/0	0/0	0/0	0/1	0/0	0/0
Chromosome2-4325310	INS	PRECISE	775	0	0	0/0	0/0	0/0	0/0	0/0	0/0	0/0	0/0	0/0	0/0	0/0	0/1	0/0	0/0
Chromosome2-4325342	INS	PRECISE	55	0	0.302	0/0	0/1	0/0	0/0	0/1	0/0	0/1	1/1	0/1	1/1	1/1	1/1	1/1	0/1
Chromosome2-4325365	DEL	PRECISE	221	0	0	0/0	0/0	0/0	0/0	0/0	0/0	0/0	0/0	0/0	0/0	0/0	0/0	0/0	0/1
Chromosome2-4325663	INS	PRECISE	6991	0	0	0/0	0/0	0/0	0/0	0/0	0/0	0/0	0/0	0/0	0/0	0/0	0/0	0/0	1/1
Chromosome2-4325847	INS	PRECISE	995	0	0	0/0	0/0	0/0	0/0	1/1	0/0	0/0	0/0	0/0	0/0	0/0	0/0	0/0	0/0

Chromosome2-4327122	INS	PRECISE	140	0	0.756	0/0	0/1	0/0	0/0	1/1	0/1	0/1	1/1	0/0	1/1	0/0	0/0	1/1	0/0
Chromosome2-4327584	DEL	PRECISE	664	0	0	0/0	0/1	0/0	0/0	0/1	0/1	0/1	1/1	0/0	1/1	1/1	1/1	1/1	0/1
Chromosome2-4327737	INS	PRECISE	159	0	0.405	0/0	0/1	0/0	0/0	0/1	0/1	0/1	1/1	0/0	1/1	1/1	1/1	1/1	0/1
Chromosome2-4328467	DEL	PRECISE	338	0	0	0/0	0/1	0/0	0/0	1/1	0/1	0/1	1/1	0/1	1/1	1/1	1/1	1/1	1/1
Chromosome2-4328743	INS	PRECISE	50	0	0	0/0	0/1	0/0	0/0	0/1	0/1	0/1	1/1	0/1	1/1	1/1	1/1	1/1	0/1
Chromosome2-4329470	DEL	PRECISE	250	0.647	0.647	0/0	0/1	0/0	0/0	0/1	0/1	0/1	1/1	0/1	1/1	1/1	1/1	1/1	0/1
Chromosome2-4329574	INS	PRECISE	151	0	0.518	0/0	0/1	0/0	0/0	0/1	0/1	0/1	1/1	0/0	0/1	0/1	0/0	0/1	0/0
Chromosome2-4330085	DEL	PRECISE	995	0	0	0/0	0/1	0/1	1/1	1/1	1/1	1/1	1/1	1/1	1/1	1/1	1/1	1/1	1/1
Chromosome2-4330323	DEL	PRECISE	54	0	0	0/0	0/1	0/0	0/0	0/1	0/1	0/1	1/1	0/1	1/1	1/1	1/1	1/1	0/1
Chromosome2-4330837	INS	PRECISE	653	0	0	0/0	0/0	0/0	0/0	0/0	0/0	0/0	0/0	0/0	0/0	0/0	0/0	0/0	0/1
Chromosome2-4331227	DEL	PRECISE	73	0	0	0/0	0/1	0/0	0/0	0/0	0/1	0/1	1/1	0/1	1/1	1/1	1/1	1/1	0/1
Chromosome2-4332631	INS	PRECISE	5447	0	0	0/0	0/0	0/0	0/0	0/0	0/0	0/0	0/0	1/1	0/0	0/0	0/0	0/0	0/0
Chromosome2-4332742	INS	PRECISE	674	0	0	0/0	0/0	0/0	0/0	0/0	0/0	0/0	0/0	0/0	0/0	0/0	0/0	0/0	0/1
Chromosome2-4333132	DEL	PRECISE	2267	0.333	0.333	0/0	1/1	0/0	0/0	0/1	1/1	0/1	1/1	0/0	1/1	1/1	0/0	1/1	1/1
Chromosome2-4333430	INS	PRECISE	4243	0	0	0/0	0/0	0/0	0/0	0/0	0/0	0/0	0/0	0/0	0/0	0/0	0/0	0/0	1/1
Chromosome2-4333650	INS	PRECISE	132	0	0.333	0/0	0/1	0/0	0/0	0/1	0/1	0/1	1/1	0/0	0/1	1/1	0/0	0/1	0/0
Chromosome2-4334136	INS	PRECISE	3653	0	0	0/0	0/0	0/0	0/0	1/1	0/0	0/0	0/0	0/0	0/0	0/0	0/0	0/0	0/0
Chromosome2-4334258	DEL	PRECISE	1224	0.333	0.333	0/0	0/1	0/0	0/0	1/1	1/1	0/1	1/1	0/1	0/0	1/1	0/0	1/1	0/0
Chromosome2-4334499	DEL	PRECISE	90	0	0	0/0	0/0	0/0	0/0	0/0	0/0	0/0	0/0	0/0	0/0	0/0	0/0	0/0	1/1
Chromosome2-4336415	DEL	PRECISE	137	0.333	0.333	0/0	0/1	0/0	0/0	0/0	0/0	0/1	1/1	0/1	1/1	1/1	0/0	1/1	0/1
Chromosome2-4336610	DEL	PRECISE	728	0	0	0/0	0/1	0/0	0/0	1/1	0/0	1/1	1/1	0/1	1/1	1/1	0/0	1/1	1/1
Chromosome2-4336769	INS	PRECISE	73	0	0.316	0/0	0/1	0/0	0/0	0/1	0/0	0/1	1/1	0/1	1/1	1/1	0/0	1/1	1/1
Chromosome2-4336984	INS	PRECISE	161	0	0.707	0/0	0/0	0/0	0/0	0/0	0/0	0/0	0/0	0/1	0/0	0/1	0/0	0/0	0/0
Chromosome2-4337779	DEL	PRECISE	431	0.949	0.949	0/0	0/1	0/0	0/0	0/1	1/1	0/1	1/1	0/1	1/1	1/1	1/1	1/1	0/0
Chromosome2-4337948	INS	PRECISE	266	0	0.354	0/0	1/1	0/0	0/0	0/1	0/0	0/1	0/0	0/0	0/0	0/0	0/0	0/0	0/0
Chromosome2-4338069	DEL	PRECISE	96	0	0	0/0	0/1	0/0	0/0	0/1	1/1	0/1	1/1	0/1	1/1	1/1	1/1	1/1	0/1
Chromosome2-4338857	INS	PRECISE	161	0	0	0/0	0/0	0/0	0/0	0/0	0/0	0/0	0/0	0/0	0/0	0/0	0/1	0/0	0/0
Chromosome2-4339373	DEL	PRECISE	616	0	0	0/0	0/1	0/0	0/0	0/1	0/1	0/1	1/1	0/0	0/1	0/1	0/0	0/1	0/0
Chromosome2-4339473	INS	PRECISE	665	0	0	0/0	0/0	0/0	0/0	0/0	0/0	0/0	0/0	0/0	0/0	0/0	0/0	0/0	0/1
Chromosome2-4340775	INS	PRECISE	880	0	0	0/0	0/0	0/0	0/0	0/1	0/0	0/0	0/0	0/0	0/0	0/0	0/0	0/0	0/0
Chromosome2-4342216	DEL	PRECISE	4802	0	0	0/0	0/0	0/0	0/0	0/0	0/0	0/0	0/0	0/0	1/1	0/0	1/1	1/1	0/1
Chromosome2-4342259	INS	PRECISE	1647	0	0	0/0	0/0	0/0	0/0	1/1	0/0	0/0	0/0	0/0	0/0	0/0	0/0	0/0	0/0
Chromosome2-4343442	INS	PRECISE	333	0	0	0/0	0/0	0/0	0/0	0/0	0/0	0/0	0/0	0/0	0/0	0/0	0/0	0/0	0/1
Chromosome2-4343953	DEL	PRECISE	167	0	0	0/0	0/1	0/0	0/0	0/1	0/1	0/1	1/1	0/0	1/1	0/1	0/1	1/1	0/0
Chromosome2-4345036	INS	PRECISE	4968	0	0	0/0	0/0	0/0	0/0	0/0	0/0	0/0	0/0	0/0	0/0	0/0	0/0	0/0	1/1
Chromosome2-4345057	DEL	PRECISE	335	0	0	0/0	0/0	0/1	1/1	0/0	0/0	0/0	0/0	0/0	0/0	0/0	0/0	0/0	0/0
Chromosome2-4345611	INS	PRECISE	285	0	0	0/0	0/0	0/0	0/0	0/0	0/0	0/0	0/0	0/0	0/1	0/0	0/1	0/0	0/0
Chromosome2-4346437	INS	PRECISE	1070	0	0	0/0	0/0	0/0	0/0	0/0	0/0	0/0	0/0	0/0	0/0	0/0	0/0	0/1	0/0
Chromosome2-4346708	BND	PRECISE	0	0	0	0/0	0/0	0/1	0/1	0/0	0/0	0/0	0/0	0/0	0/0	0/0	0/0	0/0	0/0
Chromosome2-4346712	INS	PRECISE	994	0	0	0/0	0/0	0/0	0/0	0/0	0/0	0/1	0/0	0/0	0/0	0/0	0/0	0/0	0/0
Chromosome2-4346964	INS	PRECISE	1067	0	0	0/0	0/0	0/0	0/0	0/0	0/0	0/0	0/0	0/0	0/0	0/0	0/0	0/0	0/1
Chromosome2-4347104	INS	PRECISE	1228	0	0	0/0	0/0	0/0	0/0	0/0	0/0	0/0	0/0	0/0	0/0	0/0	0/0	0/0	0/1
Chromosome2-4347152	INS	PRECISE	132	0	0	0/0	0/0	0/0	0/0	0/1	0/0	0/0	0/0	0/0	0/0	0/0	0/0	0/0	0/0
Chromosome2-4347623	INS	PRECISE	150	0	0	0/0	0/0	0/0	0/0	0/0	0/0	0/0	0/0	0/0	0/0	0/0	0/0	0/0	0/1
Chromosome2-4347797	INS	PRECISE	825	0	0.951	0/0	1/1	0/0	0/0	1/1	0/0	1/1	0/0	0/0	1/1	1/1	0/0	0/1	0/0
Chromosome2-4347894	INS	PRECISE	3851	0	0	0/0	0/0	0/0	0/0	0/0	0/0	0/0	0/0	0/0	0/0	0/0	1/1	0/0	0/0
Chromosome2-4347977	INS	PRECISE	210	0	0.518	0/0	0/1	0/0	0/0	0/1	0/0	0/1	1/1	0/0	0/1	0/1	0/0	0/0	0/0
Chromosome2-4348117	INS	PRECISE	3621	0	0	0/0	0/0	0/0	0/0	0/0	0/0	0/0	0/0	0/0	0/0	0/0	1/1	0/0	0/0
Chromosome2-4349091	INS	PRECISE	132	0	0	0/0	0/0	0/0	0/0	0/0	0/0	0/0	0/0	0/1	0/1	0/1	0/1	0/0	0/1
Chromosome2-4350316	DEL	PRECISE	56	0	0	0/0	0/0	0/1	1/1	0/1	0/0	1/1	0/0	0/0	0/0	0/0	0/0	0/0	0/1
Chromosome2-4350538	INS	PRECISE	137	0	0.354	0/0	0/0	0/0	0/0	0/1	0/1	0/1	1/1	0/0	0/1	0/0	0/0	1/1	0/0
Chromosome2-4350701	INS	PRECISE	133	0	0.522	0/0	0/1	0/0	0/0	0/1	0/1	0/1	1/1	0/1	1/1	1/1	0/1	0/1	0/1
Chromosome2-4350975	INS	PRECISE	191	0	0.5	0/0	0/1	0/0	0/0	0/1	0/0	0/1	1/1	0/0	0/0	0/1	0/1	0/1	0/1
Chromosome2-4351295	DEL	PRECISE	795	0	0	0/0	0/0	0/0	0/0	0/0	0/0	0/0	0/0	0/0	0/0	0/0	0/0	0/0	0/1
Chromosome2-4351610	INS	PRECISE	106	0	0.302	0/0	0/1	0/0	0/0	0/1	0/1	0/0	1/1	0/0	1/1	1/1	1/1	1/1	0/0
Chromosome2-4351740	DEL	PRECISE	110	0	0	0/0	0/0	0/0	0/0	0/0	0/0	0/0	0/0	0/0	0/1	0/1	1/1	0/0	0/0
Chromosome2-4351823	DEL	PRECISE	583	0	0	0/0	0/0	0/0	0/0	0/0	0/0	0/0	0/0	0/0	0/0	0/0	0/0	0/0	0/1
Chromosome2-4351921	INS	PRECISE	133	0	0	0/0	0/0	0/0	0/0	0/0	0/0	0/0	0/0	0/0	0/0	0/0	0/1	0/0	0/0
Chromosome2-4353436	INS	PRECISE	103	0	0	0/0	0/0	0/0	0/0	0/1	0/0	0/0	0/0	0/0	0/0	0/0	0/0	0/0	0/0
Chromosome2-4353687	INS	PRECISE	702	0	0	0/0	0/0	0/0	0/0	0/0	0/0	0/0	0/0	0/0	0/0	0/0	0/0	0/1	0/0
Chromosome2-4353765	DEL	PRECISE	58	0	0	0/0	0/0	0/0	0/0	0/0	0/0	0/0	0/0	0/0	0/0	0/0	0/0	0/0	1/1
Chromosome2-4355586	INS	PRECISE	614	0	0	0/0	0/0	0/0	0/0	0/0	0/0	0/0	0/0	0/0	0/0	0/0	0/0	0/0	0/1
Chromosome2-4355703	INS	PRECISE	4940	0	0	0/0	0/0	0/0	0/0	0/0	0/0	0/1	0/0	0/0	0/0	0/0	0/0	0/0	0/0
Chromosome2-4356560	INS	PRECISE	67	0	0	0/0	0/1	0/0	0/0	0/1	0/1	0/1	1/1	0/1	1/1	1/1	1/1	1/1	1/1
Chromosome2-4357393	DEL	PRECISE	99	0	0	0/0	0/0	0/0	0/0	0/0	0/0	0/0	0/0	0/0	0/0	0/0	0/0	0/0	0/1
Chromosome2-4357513	INS	PRECISE	197	0	0.354	0/0	0/1	0/0	0/0	0/1	1/1	0/1	1/1	0/0	0/1	0/0	0/0	0/1	0/0
Chromosome2-4358326	DEL	PRECISE	51	0	0	0/0	0/0	0/0	0/0	0/0	0/0	0/0	0/0	0/0	0/0	0/0	0/0	0/0	0/1
Chromosome2-4358391	INS	PRECISE	79	0	0.744	0/0	0/1	0/0	0/0	0/1	0/1	0/1	1/1	0/0	0/1	0/0	0/0	0/1	0/0
Chromosome2-4359149	INS	PRECISE	813	0	0	0/0	0/0	0/0	0/0	0/0	0/0	0/0	0/0	0/0	0/0	0/0	0/0	0/1	0/0
Chromosome2-4360554	INS	PRECISE	77	0	0.302	0/0	0/1	0/0	0/0	0/0	0/1	0/1	1/1	0/1	1/1	1/1	1/1	1/1	0/1
Chromosome2-4360755	BND	PRECISE	0	0	0	0/0	0/0	0/0	0/0	0/0	0/0	0/0	0/0	0/0	0/0	0/1	0/1	0/0	0/0
Chromosome2-4360759	INS	PRECISE	349	0	0	0/0	0/0	0/0	0/0	0/0	0/0	0/0	0/0	0/0	0/0	0/0	0/0	0/0	0/1
Chromosome2-4360954	INS	PRECISE	161	0	0.405	0/0	0/1	0/0	0/0	0/1	0/1	0/1	1/1	0/0	1/1	1/1	1/1	1/1	0/1

Chromosome2-4361251	INS	PRECISE	1684	0	0	0/0	0/0	0/0	0/0	0/0	0/0	0/0	0/0	0/0	0/0	0/0	0/0	0/0	0/1
Chromosome2-4361311	INS	PRECISE	98	0	0.966	0/0	0/1	0/0	0/0	0/1	0/0	0/1	1/1	0/0	1/1	1/1	1/1	1/1	0/1
Chromosome2-4361492	DEL	PRECISE	52	0.316	0.316	0/0	0/1	0/0	0/0	0/1	0/0	0/1	1/1	0/1	1/1	1/1	1/1	1/1	0/1
Chromosome2-4362453	INS	PRECISE	1961	0	0	0/0	0/0	0/0	0/0	0/0	0/0	0/0	0/0	0/0	0/0	0/0	0/0	0/0	0/1
Chromosome2-4362978	INS	PRECISE	139	0	0	0/0	0/0	0/0	0/0	0/0	0/0	0/0	0/0	0/0	0/0	0/0	0/0	0/0	0/1
Chromosome2-4363386	INS	PRECISE	1122	0	0	0/0	0/0	0/1	1/1	0/0	0/0	0/0	0/0	0/0	0/0	0/0	0/0	0/0	0/0
Chromosome2-4363748	DEL	PRECISE	55	0	0	0/0	0/0	0/0	0/0	0/1	0/0	0/0	0/0	0/0	0/0	0/0	0/0	0/0	0/0
Chromosome2-4364097	INS	PRECISE	63	0	0.302	0/0	0/1	0/0	0/0	0/1	0/1	0/1	1/1	0/1	1/1	1/1	1/1	1/1	0/1
Chromosome2-4364750	INS	PRECISE	60	0	0.302	0/0	0/1	0/0	0/0	0/0	0/1	0/0	1/1	0/1	1/1	1/1	1/1	1/1	0/1
Chromosome2-4364931	DEL	PRECISE	720	0	0	0/0	0/0	0/0	0/0	0/0	0/0	0/0	0/0	0/0	0/0	1/1	1/1	1/1	1/1
Chromosome2-4365407	INS	PRECISE	255	0	0	0/0	0/1	0/0	0/0	0/1	0/0	0/1	1/1	0/0	1/1	1/1	1/1	1/1	0/0
Chromosome2-4366417	DEL	PRECISE	57	0.302	0	0/0	0/1	0/0	0/0	0/1	0/0	0/1	1/1	0/1	1/1	1/1	1/1	1/1	0/1
Chromosome2-4367736	DEL	PRECISE	106	0	0	0/0	0/0	0/0	0/0	0/0	0/0	0/0	0/0	0/0	0/0	0/0	0/0	0/0	0/1
Chromosome2-4367889	INS	PRECISE	1068	0	0	0/0	0/0	0/0	0/0	0/0	0/0	0/1	0/0	0/0	0/0	0/0	0/0	0/0	0/0
Chromosome2-4369394	DEL	PRECISE	5429	0	0	0/0	0/1	0/1	1/1	1/1	0/1	1/1	1/1	0/1	1/1	1/1	1/1	1/1	1/1
Chromosome2-4369996	INS	PRECISE	105	0	0	0/0	0/1	0/0	0/0	0/1	0/1	0/1	1/1	0/1	1/1	1/1	1/1	1/1	0/1
Chromosome2-4371587	INS	PRECISE	95	0	0	0/0	0/1	0/0	0/0	0/1	0/1	0/1	1/1	0/1	1/1	1/1	1/1	0/1	0/0
Chromosome2-4371895	DEL	PRECISE	1335	0	0	0/0	0/1	0/0	0/0	0/1	0/1	0/1	1/1	0/1	1/1	1/1	1/1	1/1	0/1
Chromosome2-4372377	DEL	PRECISE	68	0	0	0/0	0/1	0/0	0/0	0/1	0/1	0/1	1/1	0/0	1/1	1/1	1/1	1/1	1/1
Chromosome2-4372573	BND	PRECISE	0	0	0	0/0	0/0	0/0	0/1	0/0	0/0	0/0	0/0	0/0	0/0	0/0	0/0	0/0	0/0
Chromosome2-4372744	INS	PRECISE	1013	0	0	0/0	0/0	0/0	0/0	0/0	0/0	0/0	0/0	0/0	0/0	0/0	0/0	0/0	0/1
Chromosome2-4372835	DEL	PRECISE	90	0	0	0/0	0/0	0/0	0/0	0/0	0/0	0/0	0/0	0/0	0/0	0/0	0/0	0/0	0/1
Chromosome2-4372906	INS	PRECISE	684	0.603	0.674	0/0	0/1	0/0	0/0	0/1	0/1	0/1	1/1	0/1	1/1	1/1	1/1	1/1	0/1
Chromosome2-4372959	DEL	PRECISE	57	0	0	0/0	0/0	0/1	1/1	0/1	0/1	0/1	0/0	0/1	0/0	0/0	0/0	0/0	0/0
Chromosome2-4373448	DEL	PRECISE	267	0	0	0/0	0/1	0/0	0/0	0/1	0/1	0/1	1/1	0/0	1/1	0/1	0/1	1/1	0/1
Chromosome2-4373972	DEL	PRECISE	513	0	0	0/0	0/1	0/0	0/0	0/1	0/1	0/1	1/1	0/1	1/1	1/1	1/1	1/1	0/1
Chromosome2-4374275	DEL	PRECISE	295	0	0	0/0	0/1	0/0	0/0	0/1	0/1	0/1	1/1	0/1	1/1	1/1	1/1	1/1	0/1
Chromosome2-4375276	DEL	PRECISE	91	0.577	0.577	0/0	0/0	0/0	0/0	0/0	0/0	0/0	0/0	0/0	0/0	1/1	1/1	0/0	0/0
Chromosome2-4375376	INS	PRECISE	683	0	0.894	0/0	0/1	0/0	0/0	0/1	0/0	0/1	1/1	0/0	1/1	1/1	1/1	1/1	0/1
Chromosome2-4375604	INS	PRECISE	117	0	0.405	0/0	0/1	0/0	0/0	0/1	0/1	0/1	1/1	0/0	1/1	1/1	1/1	1/1	0/1
Chromosome2-4376167	INS	PRECISE	1126	0	0	0/0	0/0	0/0	0/0	0/0	0/0	0/0	0/0	0/0	0/0	0/0	0/0	0/0	0/1
Chromosome2-4376169	INS	PRECISE	77	0	0	0/0	0/0	0/0	0/0	0/0	0/0	0/0	0/0	0/0	0/0	0/0	0/0	0/1	0/0
Chromosome2-4376630	DEL	PRECISE	306	0	0	0/0	0/0	0/0	0/0	0/0	0/0	0/0	0/0	0/0	0/0	0/0	0/0	0/0	0/1
Chromosome2-4376947	DEL	PRECISE	87	0	0	0/0	0/0	0/0	0/0	0/0	0/0	0/0	0/0	0/0	0/0	0/0	0/0	0/0	0/1
Chromosome2-4377103	INS	PRECISE	1309	0	0	0/0	0/0	0/0	0/0	0/0	0/0	0/0	0/0	0/0	0/0	0/0	0/0	0/0	0/1
Chromosome2-4377130	DEL	PRECISE	52	0	0	0/0	0/1	0/0	0/0	0/1	0/1	0/1	1/1	0/0	0/1	0/1	0/0	0/1	0/0
Chromosome2-4377230	INS	PRECISE	7767	0	0	0/0	0/0	0/0	0/0	0/0	0/0	0/0	0/0	0/0	0/0	0/0	0/0	0/1	0/0
Chromosome2-4378773	DEL	PRECISE	529	0	0	0/0	0/1	0/0	0/0	0/1	0/0	0/1	1/1	0/1	1/1	1/1	1/1	1/1	0/0
Chromosome2-4379184	INS	PRECISE	1813	0	0	0/0	0/0	0/0	0/0	0/0	0/0	0/0	0/0	0/0	0/0	0/0	0/1	0/0	0/0
Chromosome3-10812	BND	PRECISE	0	0	0	0/1	0/1	1/1	0/0	0/1	0/1	0/0	0/1	0/0	0/0	0/1	0/0	0/0	0/0
Chromosome3-11289	DEL	PRECISE	938	0	0	0/0	0/0	0/1	0/0	0/0	0/0	0/0	0/0	0/0	0/0	0/0	0/0	0/0	0/0
Chromosome3-12900	INS	PRECISE	138	0	0.707	0/0	0/0	0/1	1/1	0/0	0/0	0/0	0/0	0/0	0/0	0/0	0/0	0/0	0/0
Chromosome3-20698	DEL	PRECISE	166	0	0	0/0	0/0	0/1	1/1	0/0	0/0	0/0	0/0	0/0	0/0	0/0	0/0	0/0	1/1
Chromosome3-23079	DEL	PRECISE	1030	0.316	0.316	0/0	0/1	0/0	0/1	0/0	0/0	0/0	0/0	1/1	0/0	0/0	0/0	0/0	0/0
Chromosome3-24737	DEL	PRECISE	2479	0	0	0/1	0/1	0/0	0/1	0/0	0/0	0/0	0/0	1/1	0/0	0/0	0/0	0/0	0/0
Chromosome3-29432	DEL	PRECISE	6002	0	0	0/0	0/0	0/1	0/1	0/0	0/0	0/0	0/0	0/0	0/0	0/0	0/0	0/0	0/1
Chromosome3-37213	DEL	PRECISE	67	0	0	0/0	0/0	0/1	0/1	0/0	0/0	0/0	0/0	0/0	0/0	0/0	0/0	0/0	0/0
Chromosome3-37356	INS	PRECISE	383	0	0	0/0	0/0	0/1	0/1	0/0	0/0	0/0	0/0	0/0	0/0	0/0	0/0	0/0	0/0
Chromosome3-37720	DEL	PRECISE	3811	0	0	0/0	0/0	0/0	0/0	0/0	0/0	0/0	0/0	0/1	0/0	0/1	0/0	0/0	0/1
Chromosome3-38347	DEL	PRECISE	12800	0	0	0/0	0/0	0/1	0/1	0/0	0/0	0/0	0/0	0/0	0/0	0/0	0/0	0/0	0/1
Chromosome3-41563	DEL	PRECISE	2556	0	0	0/0	0/0	0/0	0/0	0/0	0/0	0/0	0/0	0/1	0/0	0/1	0/0	0/0	0/1
Chromosome3-43357	DEL	PRECISE	771	0	0	0/0	0/0	0/0	0/1	0/0	0/0	0/0	0/0	0/1	0/1	0/0	0/0	0/0	0/0
Chromosome3-44150	DEL	PRECISE	198	0	0	0/1	0/0	0/0	0/0	0/0	0/0	0/0	0/0	0/1	0/0	0/1	0/0	0/0	0/1
Chromosome3-51199	DEL	PRECISE	1508	0	0	0/0	0/0	0/0	0/0	0/0	0/0	0/0	0/0	0/0	0/0	0/0	0/0	0/0	0/1
Chromosome3-53385	INS	PRECISE	5454	0	0	0/0	0/0	0/0	0/0	0/0	0/0	1/1	0/0	0/0	0/0	0/0	0/0	0/0	0/0
Chromosome3-55474	INS	PRECISE	54	0	0	0/0	0/0	0/1	0/1	0/0	0/0	0/0	0/0	0/0	0/0	0/0	0/0	0/0	0/1
Chromosome3-62060	DEL	PRECISE	86	0.577	0.577	0/0	0/0	0/0	0/1	0/0	1/1	0/0	0/0	0/1	0/0	0/0	0/0	0/0	0/0
Chromosome3-71014	DEL	PRECISE	105	0	0	0/0	0/0	0/0	0/0	0/0	0/0	0/0	0/0	0/0	0/0	0/0	0/0	0/0	0/1
Chromosome3-73317	DEL	PRECISE	262	0.289	0.289	0/1	0/1	1/1	0/1	1/1	0/0	0/1	1/1	0/0	0/1	0/1	0/1	0/1	0/1
Chromosome3-80440	DEL	PRECISE	248	0	0	0/1	0/1	1/1	0/1	0/1	0/0	0/1	1/1	0/0	0/1	1/1	0/1	0/1	0/0
Chromosome3-81698	DEL	PRECISE	270	0	0	0/0	0/0	0/1	0/1	0/0	0/0	0/0	0/0	0/0	0/0	0/0	0/0	0/0	0/0
Chromosome3-82132	INS	PRECISE	179	0	0	0/0	0/0	0/0	0/0	0/0	0/0	0/0	0/0	0/0	0/0	0/0	0/0	0/0	0/1
Chromosome3-82754	BND	PRECISE	0	0	0	0/0	0/1	0/0	0/0	0/0	0/0	0/0	0/1	0/0	0/0	0/1	0/0	0/0	0/0
Chromosome3-82882	INS	PRECISE	7180	0	0	0/0	0/0	0/0	0/0	0/0	0/0	0/0	0/0	0/0	0/0	0/0	0/0	0/0	0/1
Chromosome3-83013	BND	PRECISE	0	0	0	0/1	0/1	0/1	0/0	0/1	0/1	0/0	1/1	0/0	0/1	0/1	0/1	0/1	0/0
Chromosome3-96029	INS	PRECISE	54	0	0.577	0/0	0/0	0/1	0/1	0/0	0/0	0/0	0/0	0/0	0/0	0/0	0/0	0/0	0/0
Chromosome3-96029	DEL	PRECISE	54	0	0	0/1	0/1	0/1	0/0	0/1	0/1	0/0	0/1	0/0	0/1	0/1	0/1	0/1	0/0
Chromosome3-117583	DEL	PRECISE	216	0	0	0/1	0/1	1/1	0/1	0/1	0/1	0/1	0/1	0/0	0/1	0/1	0/1	0/1	0/1
Chromosome3-119710	DEL	PRECISE	250	0	0	0/1	1/1	1/1	0/1	0/1	0/1	0/1	0/1	0/0	0/1	0/1	0/1	0/1	0/1
Chromosome3-146484	DEL	PRECISE	3850	0	0	0/0	0/0	0/1	0/0	0/0	0/0	0/0	0/0	0/0	0/0	0/0	0/0	0/0	0/0
Chromosome3-149162	DEL	PRECISE	218	0	0	0/1	0/1	0/1	0/1	0/1	0/1	0/1	0/1	0/0	0/1	0/1	0/1	0/1	0/1
Chromosome3-158477	DEL	PRECISE	831	0	0	0/1	0/1	1/1	0/1	0/1	0/1	0/1	0/1	0/0	1/1	1/1	0/1	0/1	0/1
Chromosome3-165994	DEL	PRECISE	50	0.852	0	0/1	0/1	1/1	1/1	1/1	1/1	0/1	0/1	0/1	1/1	1/1	0/1	1/1	0/1

Chromosome3-167035	INS	PRECISE	5394	0	0	0/0	0/0	0/0	0/0	0/0	0/0	0/0	0/0	0/0	1/1	0/0	0/0	0/0	0/0
Chromosome3-169060	DEL	PRECISE	86	0	0	0/1	0/1	1/1	0/1	0/1	1/1	0/1	0/1	0/0	0/1	0/1	0/1	0/1	0/0
Chromosome3-174972	INS	PRECISE	4325	0	0	0/0	0/0	0/0	0/0	0/0	0/0	0/0	0/0	0/0	0/0	0/0	0/0	0/0	1/1
Chromosome3-180890	INS	PRECISE	654	0	0.866	0/1	0/1	1/1	0/1	0/1	1/1	0/1	0/1	0/0	0/1	0/1	0/1	0/1	0/0
Chromosome3-186304	INS	PRECISE	50	0	0	0/1	0/1	1/1	0/1	0/1	0/1	0/1	0/1	0/0	0/1	0/1	0/1	0/1	0/1
Chromosome3-187769	INS	PRECISE	236	0	0	0/0	0/0	0/0	0/0	0/0	0/0	0/0	0/0	0/0	0/0	0/1	0/0	0/0	0/0
Chromosome3-190183	DEL	PRECISE	289	0.277	0.277	0/1	0/1	1/1	0/1	0/1	0/1	0/1	0/1	0/0	0/1	0/1	0/1	0/1	0/1
Chromosome3-191693	DEL	PRECISE	252	0	0	0/1	0/1	0/1	0/0	0/1	0/1	0/0	0/1	0/0	0/1	0/1	0/1	0/1	0/0
Chromosome3-195187	INS	PRECISE	137	0	0	0/0	0/0	0/0	0/0	0/0	0/0	0/0	0/0	0/0	0/0	0/1	0/0	0/0	0/0
Chromosome3-203942	INS	PRECISE	997	0	0	0/0	0/0	0/0	0/0	0/0	0/0	0/0	0/0	0/0	0/0	0/0	0/0	0/0	0/1
Chromosome3-206498	DEL	PRECISE	278	0	0	0/1	0/1	1/1	0/1	0/1	0/1	0/1	0/1	0/0	0/1	0/1	0/1	0/1	0/1
Chromosome3-206993	INS	PRECISE	807	0	0	0/0	0/0	0/0	0/0	0/0	0/0	0/0	0/0	0/0	0/1	0/0	0/0	0/0	0/0
Chromosome3-210790	DEL	PRECISE	74	0	0	0/0	0/0	0/0	0/0	0/0	0/0	0/0	0/0	0/0	0/0	0/0	0/0	0/0	0/1
Chromosome3-214727	INS	PRECISE	99	0	0.277	0/1	0/1	1/1	0/1	0/1	0/1	0/1	0/1	0/0	0/1	0/1	0/1	0/1	0/1
Chromosome3-223999	DEL	PRECISE	93	0	0	0/0	0/0	0/0	0/1	0/1	0/1	0/0	0/0	0/1	0/0	0/1	0/0	0/0	0/0
Chromosome3-227138	INS	PRECISE	241	0	0.707	0/0	0/0	0/1	0/1	0/0	0/0	0/0	0/0	0/0	0/0	0/0	0/0	0/0	0/0
Chromosome3-233906	INS	PRECISE	674	0	0	0/0	0/0	0/0	0/0	0/0	0/0	0/0	0/0	0/0	0/1	0/0	0/0	0/0	0/0
Chromosome3-235106	DEL	PRECISE	63	0	0	0/1	0/1	0/1	0/1	0/1	0/1	0/1	0/1	0/1	0/1	0/1	0/1	0/0	0/1
Chromosome3-242018	INS	PRECISE	132	0	0	0/0	0/0	0/1	0/1	0/0	0/0	0/0	0/0	0/0	0/0	0/0	0/0	0/0	0/0
Chromosome3-269233	BND	PRECISE	0	0	0	0/1	0/1	0/1	0/0	0/1	0/1	0/1	0/0	0/0	0/0	0/1	0/1	0/1	0/0
Chromosome3-273572	INS	PRECISE	1465	0	0.837	0/0	0/0	0/0	1/1	0/1	0/0	0/0	0/0	0/1	0/0	0/0	0/0	0/0	0/0
Chromosome3-285720	DEL	PRECISE	749	0	0	0/0	0/0	0/0	0/0	0/0	0/0	0/0	0/0	0/0	0/1	0/1	0/0	0/0	0/0
Chromosome3-292456	DEL	PRECISE	83	0	0	0/0	0/0	0/0	1/1	0/1	0/0	0/0	0/0	0/1	0/1	0/0	0/0	0/0	0/0
Chromosome3-293114	BND	PRECISE	0	0	0	0/0	0/0	0/0	0/0	0/0	0/0	0/0	0/0	0/0	0/0	0/1	0/0	0/0	0/0
Chromosome3-293114	BND	PRECISE	0	0	0	0/0	0/0	0/0	0/0	0/0	0/0	0/0	0/0	0/1	0/0	0/0	0/0	0/0	0/0
Chromosome3-293114	BND	PRECISE	0	0	0	0/0	0/1	0/0	0/0	0/0	0/0	0/0	0/0	0/0	0/0	0/0	0/0	0/1	0/0
Chromosome3-293114	BND	PRECISE	0	0	0	0/0	0/0	0/0	0/0	0/0	0/0	0/0	0/0	0/0	0/0	0/0	0/0	0/0	0/1
Chromosome3-293115	DEL	PRECISE	10955	0	0	0/1	0/1	0/0	0/1	0/0	0/0	0/0	1/1	0/0	0/0	0/0	0/0	0/1	0/0
Chromosome3-294685	INS	PRECISE	1342	0	0	0/0	0/1	0/0	0/0	0/0	0/0	0/0	0/0	0/0	0/0	0/0	0/0	0/1	0/0
Chromosome3-296864	INS	PRECISE	68	0	0	0/0	0/0	0/0	0/0	0/0	0/0	0/0	0/0	0/0	0/1	0/1	0/0	0/0	0/0
Chromosome3-299144	DEL	PRECISE	434	0	0	0/0	0/1	0/0	0/0	0/0	0/0	0/1	0/0	0/0	0/0	0/0	0/0	0/0	0/1
Chromosome3-304541	DEL	PRECISE	343	0	0	0/0	0/0	0/0	0/0	0/0	0/0	0/1	0/0	0/0	0/0	0/0	0/0	0/0	0/0
Chromosome3-309552	INS	PRECISE	56	0	0.5	0/0	0/0	0/0	0/1	0/1	0/1	0/0	0/0	0/1	0/0	0/0	0/0	0/0	0/0
Chromosome3-310670	DEL	PRECISE	88	0	0	0/0	0/0	0/0	0/1	0/1	0/1	0/0	0/0	0/1	0/1	0/0	0/0	0/0	0/0
Chromosome3-315149	INS	PRECISE	237	0	0	0/0	0/0	0/1	0/0	0/0	0/0	0/0	0/0	0/0	0/0	0/0	0/0	0/0	0/0
Chromosome3-322955	INS	PRECISE	5084	0	0	0/0	0/0	1/1	0/0	0/0	0/0	0/0	0/0	0/0	0/0	0/0	0/0	0/0	0/0
Chromosome3-322961	BND	PRECISE	0	0	0	0/0	0/0	0/1	0/0	0/0	0/0	0/0	0/0	0/0	0/0	0/0	0/0	0/0	0/0
Chromosome3-328726	DEL	PRECISE	70	0	0	0/0	0/0	0/0	0/0	0/0	0/0	0/0	0/0	0/0	0/0	0/0	0/0	0/0	0/1
Chromosome3-329449	INS	PRECISE	1708	0	0	0/0	0/0	0/0	0/0	0/0	0/0	0/0	0/0	0/0	0/0	0/1	0/0	0/0	0/0
Chromosome3-338917	INS	PRECISE	867	0	0	0/0	0/0	0/0	0/0	0/0	0/0	0/0	0/0	0/0	0/0	0/0	0/0	0/0	0/1
Chromosome3-339253	BND	PRECISE	0	0	0	0/1	0/0	0/1	0/0	0/1	0/0	0/0	0/0	0/0	0/1	0/1	0/0	0/0	0/0
Chromosome3-339253	BND	PRECISE	0	0	0	0/0	0/1	0/1	0/0	0/0	0/0	0/0	0/0	0/0	0/0	0/1	0/0	0/0	0/0
Chromosome3-348646	DEL	PRECISE	328	0	0	0/1	0/1	1/1	0/1	0/1	0/1	0/1	0/0	0/1	0/1	0/1	0/1	0/1	0/1
Chromosome3-351767	INS	PRECISE	1781	0	0	0/0	0/0	0/0	0/1	0/0	0/0	0/0	0/0	0/0	0/0	0/0	0/0	0/0	0/0
Chromosome3-353723	DEL	PRECISE	198	0	0	0/1	0/1	1/1	0/1	0/1	0/1	0/1	0/0	0/1	0/1	0/1	0/1	0/1	0/0
Chromosome3-361194	INS	PRECISE	904	0	0	0/0	0/0	0/0	0/0	0/0	0/0	0/0	0/0	0/0	0/1	0/0	0/0	0/0	0/0
Chromosome3-362935	INS	PRECISE	6017	0	0	0/0	0/0	0/0	0/0	0/0	0/0	0/0	0/0	0/0	0/0	1/1	0/0	0/0	0/0
Chromosome3-376356	INS	PRECISE	185	0	0	0/0	0/0	0/0	0/0	0/0	0/0	0/0	0/0	0/0	0/0	0/0	0/1	0/0	0/0
Chromosome3-378096	DEL	PRECISE	2679	0	0	0/0	0/0	0/1	0/0	0/0	0/0	0/0	0/0	0/0	0/0	0/0	0/0	0/0	0/0
Chromosome3-379049	INS	PRECISE	73	0	0.267	0/1	0/1	0/1	0/1	0/1	0/1	0/1	0/1	0/1	0/1	0/1	0/1	0/1	0/1
Chromosome3-393391	INS	PRECISE	332	0	0	0/0	0/0	0/0	0/0	0/0	0/0	0/0	0/0	0/0	0/0	0/0	0/0	0/0	0/1
Chromosome3-394214	DEL	PRECISE	136	0	0	0/1	0/0	1/1	0/1	0/1	0/1	0/0	0/0	0/1	0/1	0/0	0/0	0/1	0/1
Chromosome3-406993	DEL	PRECISE	237	0.267	0.267	0/1	0/1	1/1	0/1	0/1	1/1	0/1	0/1	0/1	0/1	0/1	0/1	0/1	0/1
Chromosome3-410033	DEL	PRECISE	531	0	0	0/1	0/1	0/1	0/0	0/1	0/1	0/1	0/1	0/1	0/1	0/1	0/1	0/1	0/0
Chromosome3-414771	DEL	PRECISE	874	0	0	0/1	0/1	1/1	0/1	0/1	0/1	0/1	0/1	0/1	1/1	0/1	1/1	0/1	0/1
Chromosome3-418984	DEL	PRECISE	291	0.267	0.267	0/1	0/1	1/1	0/1	0/1	0/1	0/1	0/1	0/1	0/1	0/1	1/1	0/1	0/1
Chromosome3-419749	DEL	PRECISE	886	0.802	0	0/1	0/1	1/1	0/1	0/1	0/1	0/1	0/1	0/0	0/1	0/1	1/1	0/1	0/1
Chromosome3-436669	INS	PRECISE	1079	0	0	0/0	0/0	0/0	0/0	0/0	0/0	0/0	0/0	0/0	0/0	0/0	0/0	0/0	0/1
Chromosome3-441382	INS	PRECISE	1183	0	0	0/0	0/0	0/1	0/0	0/0	0/0	0/0	0/0	0/0	0/0	0/0	0/0	0/0	0/0
Chromosome3-446584	INV	PRECISE	10057351	0	0	0/0	0/0	0/0	0/1	0/0	0/0	0/0	0/0	0/1	0/1	0/0	0/0	0/0	0/0
Chromosome3-447813	INS	PRECISE	1159	0	0.837	0/0	0/0	0/0	0/1	0/1	0/1	0/0	0/0	0/1	0/1	0/0	0/0	0/0	0/0
Chromosome3-456613	DEL	PRECISE	322	0.277	0.277	0/1	0/0	1/1	0/1	0/1	0/0	0/1	0/1	0/0	0/0	0/1	0/1	0/1	0/1
Chromosome3-456714	INS	PRECISE	227	0.447	0.548	0/0	0/0	0/0	0/1	0/1	1/1	0/0	0/0	1/1	1/1	0/0	0/0	0/0	0/0
Chromosome3-462256	DEL	PRECISE	740	0	0	0/0	0/1	1/1	0/0	0/0	0/0	0/0	0/0	0/0	0/0	0/1	0/0	0/0	0/1
Chromosome3-464117	INS	PRECISE	71	0	0	0/0	0/0	0/0	0/0	0/0	0/0	0/0	0/0	0/0	0/0	0/0	1/1	0/0	0/0
Chromosome3-465132	DEL	PRECISE	55	0	0	0/0	0/0	0/0	0/1	0/1	1/1	0/0	0/0	1/1	1/1	0/0	0/0	0/0	0/0
Chromosome3-466479	DEL	PRECISE	74	0	0	0/1	0/1	1/1	0/0	0/1	0/0	0/1	0/1	0/0	0/0	0/0	0/0	0/0	0/1
Chromosome3-470809	INS	PRECISE	3331	0	0	0/0	0/0	0/0	0/0	0/0	0/0	0/0	0/0	0/0	0/0	0/0	1/1	0/0	0/0
Chromosome3-472407	DEL	PRECISE	51	0.611	0.611	0/1	0/1	1/1	0/1	0/1	0/1	0/1	0/1	0/0	0/1	0/1	0/1	0/1	0/1
Chromosome3-476807	INS	PRECISE	51	0	0	0/0	0/0	0/0	0/0	0/0	0/0	0/0	0/0	0/0	0/0	0/0	0/0	0/0	0/1
Chromosome3-483710	DEL	PRECISE	87	0	0	0/1	0/0	1/1	0/1	0/0	0/0	0/1	0/1	0/0	0/0	0/1	0/1	0/1	0/1
Chromosome3-488673	DEL	PRECISE	128	0	0	0/0	0/1	1/1	0/1	0/1	0/1	0/0	0/0	0/1	0/1	0/1	0/1	0/1	0/1
Chromosome3-509633	INS	PRECISE	161	0	0	0/0	0/0	0/0	0/0	0/0	0/0	0/0	0/0	0/0	0/0	0/0	1/1	0/0	0/0

Chromosome3-517026	INS	PRECISE	89	0	0	0/0	0/0	0/1	0/0	0/0	0/0	0/0	0/0	0/0	0/0	0/0	0/0	0/0	0/1
Chromosome3-517426	DEL	PRECISE	64	0	0	0/0	0/0	0/1	0/0	0/0	0/0	0/0	0/0	0/0	0/0	0/0	0/0	0/0	0/0
Chromosome3-527127	DEL	PRECISE	66	0	0	0/1	0/1	1/1	0/1	0/1	0/1	0/1	0/1	0/1	0/1	0/1	0/1	0/1	0/0
Chromosome3-529874	INS	PRECISE	705	0	0	0/0	0/0	0/0	0/0	0/0	0/0	0/0	0/0	0/0	0/0	0/0	0/1	0/0	0/0
Chromosome3-532980	DEL	PRECISE	1160	0	0	0/0	0/0	1/1	1/1	1/1	1/1	0/0	0/0	1/1	1/1	1/1	1/1	0/0	0/1
Chromosome3-541718	INV	PRECISE	748	0	0.378	0/0	0/0	0/0	0/1	0/1	0/1	0/0	0/0	0/1	0/1	0/1	0/1	0/0	0/0
Chromosome3-541742	DEL	PRECISE	716	0	0	0/1	0/1	1/1	1/1	1/1	1/1	0/1	0/1	1/1	1/1	1/1	1/1	0/1	0/0
Chromosome3-548176	INS	PRECISE	1032	0	0	0/0	0/0	0/0	0/0	0/0	0/0	0/0	0/0	0/0	0/0	0/0	0/0	0/0	0/1
Chromosome3-551835	DEL	PRECISE	842	0	0	0/0	0/0	0/0	1/1	1/1	1/1	0/0	0/0	1/1	1/1	1/1	1/1	0/0	0/0
Chromosome3-552764	DEL	PRECISE	427	0	0	0/0	0/0	0/0	0/0	0/0	0/0	0/0	0/0	0/0	0/0	0/0	1/1	0/0	0/0
Chromosome3-557851	DEL	PRECISE	225	0	0	0/1	0/1	1/1	1/1	1/1	0/1	0/1	0/0	0/1	0/1	0/1	0/1	0/1	0/1
Chromosome3-560492	DEL	PRECISE	135	0.267	0	0/1	0/1	1/1	0/1	0/1	0/1	0/1	0/0	0/1	0/1	0/1	0/1	0/1	0/1
Chromosome3-568279	INS	PRECISE	1005	0	0	0/0	0/0	0/0	0/0	0/0	0/0	0/0	0/0	0/0	0/0	0/0	0/0	0/0	0/1
Chromosome3-578826	INS	PRECISE	74	0	0.392	0/1	0/1	1/1	1/1	1/1	1/1	0/1	0/1	1/1	1/1	1/1	1/1	0/1	0/1
Chromosome3-599193	DEL	PRECISE	352	0	0	0/1	0/1	1/1	0/1	0/1	0/1	0/1	0/1	0/1	0/1	0/1	0/1	0/1	0/1
Chromosome3-602495	INS	PRECISE	331	0	0	0/0	0/0	0/0	0/0	0/0	0/0	0/0	0/0	0/0	0/0	0/0	0/0	0/0	0/1
Chromosome3-608472	INS	PRECISE	1206	0	0	0/0	0/0	0/0	0/0	0/0	0/0	0/0	0/0	0/0	0/0	0/0	0/0	0/0	0/1
Chromosome3-610788	DEL	PRECISE	2540	0	0	0/1	0/1	1/1	0/1	0/0	0/1	0/1	0/1	0/1	0/1	0/1	0/1	0/1	0/0
Chromosome3-628088	DEL	PRECISE	424	0	0	0/1	0/1	1/1	0/1	0/1	0/1	0/0	0/1	0/1	0/1	0/1	0/1	0/1	0/0
Chromosome3-630329	INS	PRECISE	61	0	0.363	0/1	0/1	1/1	0/1	0/1	0/1	0/0	0/1	0/1	0/1	0/1	0/1	0/1	0/1
Chromosome3-631239	DEL	PRECISE	135	0	0	0/0	0/0	0/0	0/1	0/1	0/1	0/0	0/0	0/1	0/1	0/1	0/1	0/0	0/0
Chromosome3-634033	DEL	PRECISE	3663	0	0	0/1	0/1	1/1	0/1	0/1	0/1	0/0	0/1	0/1	0/1	0/1	0/1	0/1	0/1
Chromosome3-649631	DUP	PRECISE	11800	0	0	0/0	0/0	0/0	0/0	0/0	0/0	0/0	0/0	0/0	0/1	0/0	0/0	0/0	0/0
Chromosome3-658441	DEL	PRECISE	160	0	0	0/0	0/0	0/0	0/0	0/0	0/0	0/0	0/0	0/0	0/0	0/1	0/0	0/0	0/0
Chromosome3-658920	INS	PRECISE	66	0	0	0/0	0/0	0/1	0/0	0/0	0/0	0/0	0/0	0/0	0/0	0/0	0/0	0/0	0/0
Chromosome3-663071	DEL	PRECISE	363	0	0	0/1	0/1	1/1	1/1	1/1	1/1	0/0	0/1	1/1	1/1	1/1	1/1	0/1	0/0
Chromosome3-663964	INS	PRECISE	1434	0	0	0/0	0/0	0/0	0/0	0/0	0/0	0/0	0/0	0/0	0/0	0/0	0/1	0/0	0/0
Chromosome3-665773	DEL	PRECISE	686	0	0	0/0	0/0	0/1	0/0	0/0	0/0	0/0	0/0	0/0	0/0	0/0	0/0	0/0	0/1
Chromosome3-680445	DEL	PRECISE	138	0	0	0/1	0/1	1/1	0/1	0/1	0/1	0/1	1/1	0/0	0/0	0/1	0/1	0/0	0/0
Chromosome3-680527	INS	PRECISE	75	0	0	0/0	0/0	0/0	0/0	0/0	0/0	0/0	0/0	0/0	0/0	0/0	0/1	0/0	0/0
Chromosome3-681337	INS	PRECISE	625	0	0	0/0	0/0	0/0	0/0	0/0	0/0	0/0	0/0	0/0	0/0	0/0	0/0	0/0	0/1
Chromosome3-696150	INS	PRECISE	247	0	0.333	0/0	0/0	0/1	0/0	0/0	0/0	0/0	0/1	0/0	0/0	0/0	0/1	0/0	0/0
Chromosome3-696526	DEL	PRECISE	56	0	0	0/0	0/0	1/1	0/0	0/0	0/0	0/0	0/1	0/0	0/0	0/0	0/1	0/0	0/0
Chromosome3-697581	DEL	PRECISE	3777	0	0	0/0	0/0	0/0	0/0	0/0	0/0	0/0	0/0	0/0	0/0	0/0	1/1	0/0	0/0
Chromosome3-698055	DEL	PRECISE	2900	0.408	0.408	0/0	0/0	0/0	1/1	1/1	1/1	0/0	0/0	1/1	1/1	1/1	0/0	0/0	0/0
Chromosome3-702717	DEL	PRECISE	82	0	0	0/1	0/0	1/1	0/1	0/1	0/0	0/1	0/1	0/1	0/1	0/0	0/1	0/0	0/1
Chromosome3-702908	INS	PRECISE	114	0	0	0/0	0/0	0/0	0/0	0/0	0/0	0/0	0/0	0/0	0/0	0/0	0/0	0/0	0/1
Chromosome3-709804	INS	PRECISE	161	0	0.842	0/1	0/1	1/1	0/1	0/1	0/1	0/1	1/1	0/1	0/1	0/0	0/1	0/1	0/1
Chromosome3-720854	INS	PRECISE	907	0	0	0/0	0/0	0/0	0/0	0/0	0/0	0/1	0/0	0/0	0/0	0/0	0/0	0/0	0/0
Chromosome3-738035	DEL	PRECISE	436	0	0	0/1	0/1	1/1	0/1	0/1	0/1	0/1	0/1	0/1	0/1	0/1	0/1	0/1	0/1
Chromosome3-747665	INS	PRECISE	145	0	0	0/0	0/0	0/0	0/0	0/0	0/0	0/0	0/0	0/0	0/0	0/0	0/0	0/0	0/1
Chromosome3-750505	INS	PRECISE	54	0	0	0/0	0/0	0/0	0/0	0/0	0/0	0/1	0/0	0/0	0/0	0/0	0/0	0/0	0/0
Chromosome3-753070	INS	PRECISE	5441	0	0	0/0	0/0	1/1	0/0	0/0	0/0	0/0	0/0	0/0	0/0	0/0	0/0	0/0	0/0
Chromosome3-766620	INS	PRECISE	55	0	0.376	0/1	0/1	1/1	0/1	0/1	0/1	0/1	0/1	0/1	0/1	0/1	0/1	0/1	0/0
Chromosome3-772954	DEL	PRECISE	342	0	0	0/1	0/1	1/1	0/1	0/1	0/1	0/0	0/1	0/1	0/1	0/1	1/1	0/1	0/0
Chromosome3-773629	INS	PRECISE	560	0	0	0/0	0/0	0/0	0/0	0/0	0/0	0/0	0/0	0/0	0/0	0/0	0/0	0/0	0/1
Chromosome3-781893	BND	PRECISE	0	0	0	0/1	0/0	0/0	0/0	0/0	0/0	0/0	0/0	0/1	0/0	0/0	0/0	0/0	0/0
Chromosome3-789410	DEL	PRECISE	204	0	0	0/1	0/1	1/1	0/1	0/1	0/1	0/1	0/1	0/1	0/1	0/1	0/1	0/1	0/0
Chromosome3-791565	INS	PRECISE	120	0	0	0/0	0/0	0/0	0/1	0/1	0/1	0/0	0/0	0/1	0/1	0/1	0/0	0/0	0/0
Chromosome3-801822	INS	PRECISE	80	0	0	0/0	0/0	0/0	0/1	0/1	0/1	0/0	0/0	0/1	0/1	0/1	0/0	0/0	0/0
Chromosome3-809243	DEL	PRECISE	73	0	0	0/0	0/0	0/0	0/0	0/0	0/0	0/1	0/0	0/0	0/0	0/0	0/0	0/0	0/0
Chromosome3-819574	DEL	PRECISE	66	0	0	0/1	0/1	1/1	0/1	0/1	0/1	0/1	0/1	0/1	0/1	0/1	0/1	0/1	0/1
Chromosome3-820465	INS	PRECISE	160	0	0	0/0	0/0	0/1	0/0	0/0	0/0	0/0	0/0	0/0	0/0	0/0	0/0	0/0	0/0
Chromosome3-820796	DEL	PRECISE	50	0	0	0/1	0/1	1/1	0/1	0/1	0/1	0/1	0/1	0/1	0/1	0/1	0/1	0/1	0/1
Chromosome3-821361	INS	PRECISE	422	0	0	0/0	0/0	0/0	0/0	0/0	0/0	0/0	0/0	0/0	0/0	0/0	0/0	0/0	0/1
Chromosome3-822624	INS	PRECISE	881	0	0	0/0	0/0	0/0	0/0	0/0	0/0	0/0	0/0	0/0	0/0	0/0	0/1	0/0	0/0
Chromosome3-826190	DEL	PRECISE	1195	0	0	0/1	0/1	1/1	1/1	1/1	1/1	0/1	0/1	1/1	1/1	0/1	1/1	0/1	0/1
Chromosome3-832450	INS	PRECISE	394	0	0.967	0/1	0/1	1/1	0/1	0/1	0/1	0/0	0/0	0/1	0/1	0/1	0/1	0/1	0/1
Chromosome3-832681	DEL	PRECISE	94	0	0	0/0	0/0	0/0	0/0	0/0	0/0	0/1	0/0	0/0	0/0	0/0	0/0	0/0	0/0
Chromosome3-836796	DEL	PRECISE	247	0	0.354	0/0	0/0	1/1	0/0	0/0	0/0	0/0	0/0	0/0	0/0	0/0	0/0	0/0	0/1
Chromosome3-849329	INS	PRECISE	623	0	0	0/0	0/0	0/0	0/0	0/0	0/0	0/0	0/0	0/0	0/0	0/0	0/1	0/0	0/0
Chromosome3-856875	INS	PRECISE	193	0	0.707	0/0	0/0	0/0	0/0	0/0	0/0	0/0	0/0	0/0	0/0	0/1	1/1	0/0	0/0
Chromosome3-862417	DEL	PRECISE	81	0	0	0/0	0/0	0/1	0/0	0/0	0/0	0/0	0/0	0/0	0/0	0/0	0/0	0/0	0/0
Chromosome3-867732	DEL	PRECISE	710	0	0	0/0	0/0	0/0	1/1	1/1	1/1	0/0	0/0	1/1	1/1	0/0	0/0	0/0	0/0
Chromosome3-869801	INS	PRECISE	262	0	0.548	0/0	0/0	0/0	0/1	0/1	0/1	0/0	0/0	0/1	0/1	0/0	0/0	0/0	0/0
Chromosome3-880890	DEL	PRECISE	1867	0	0	0/1	0/1	1/1	1/1	1/1	1/1	0/0	0/1	1/1	1/1	1/1	1/1	0/1	0/1
Chromosome3-884524	INS	PRECISE	121	0	0	0/0	0/0	0/0	0/0	0/0	0/0	0/0	0/0	0/0	0/0	0/0	0/0	0/0	0/1
Chromosome3-888274	INS	PRECISE	736	0	0	0/0	0/0	0/1	0/0	0/0	0/0	0/0	0/0	0/0	0/0	0/0	0/0	0/0	0/0
Chromosome3-904787	DEL	PRECISE	1014	0	0	0/0	0/0	0/0	0/0	0/0	0/0	0/0	0/0	0/0	0/0	0/0	0/0	0/0	0/1
Chromosome3-913298	DEL	PRECISE	54	0	0	0/0	0/0	0/0	0/0	0/0	0/0	0/0	0/0	0/0	0/0	1/1	0/0	0/0	0/0
Chromosome3-914057	DEL	PRECISE	1004	0.577	0	0/1	0/0	0/0	0/0	0/0	0/0	0/1	0/0	0/0	0/0	1/1	0/0	0/0	1/1
Chromosome3-917008	INS	PRECISE	2257	0	0	0/0	0/0	0/0	0/0	0/0	0/0	0/0	0/0	0/0	0/0	0/0	0/0	0/0	0/1
Chromosome3-922638	INS	PRECISE	1889	0	0	0/0	0/0	0/0	0/0	0/0	0/0	0/0	0/0	0/0	0/0	0/0	0/0	0/0	0/1

Chromosome3-923778	INS	PRECISE	5894	0	0	0/0	0/0	0/0	0/0	0/0	0/0	0/0	0/0	0/0	0/0	0/0	0/0	0/0	1/1
Chromosome3-929682	DEL	PRECISE	83	0	0	0/1	0/1	1/1	0/1	0/1	0/1	0/1	0/1	0/1	0/1	0/1	0/1	0/1	0/1
Chromosome3-935553	INS	PRECISE	266	0	0.63	0/1	0/1	1/1	0/1	0/1	0/1	0/1	0/1	0/1	0/1	0/1	0/1	0/1	0/0
Chromosome3-944390	DEL	PRECISE	852	0	0	0/1	0/1	1/1	1/1	1/1	1/1	0/1	0/1	1/1	1/1	0/1	1/1	0/1	0/1
Chromosome3-945464	DEL	PRECISE	212	0	0	0/1	0/1	0/1	1/1	1/1	1/1	0/0	0/1	1/1	1/1	0/1	0/1	0/1	0/0
Chromosome3-949177	INS	PRECISE	4650	0	0	0/0	0/0	0/0	0/0	0/0	0/0	0/0	0/0	0/0	0/0	0/0	0/0	0/0	0/1
Chromosome3-953640	DEL	PRECISE	617	0	0	0/1	0/1	1/1	1/1	1/1	1/1	0/1	0/0	1/1	1/1	0/1	0/1	0/1	0/0
Chromosome3-960584	INS	PRECISE	335	0	0	0/0	0/0	0/0	0/0	0/0	0/0	0/0	0/0	0/0	0/0	0/1	0/0	0/0	0/0
Chromosome3-966833	INS	PRECISE	80	0	0.802	0/1	0/0	1/1	0/1	0/1	0/1	0/1	0/1	0/1	0/1	0/1	0/1	0/1	0/1
Chromosome3-968073	INS	PRECISE	1749	0	0	0/0	0/0	0/0	0/0	0/0	0/0	0/0	0/0	0/0	0/0	0/0	0/0	0/0	1/1
Chromosome3-969608	INS	PRECISE	132	0	0.913	0/1	0/1	1/1	0/1	0/1	0/1	0/1	0/1	0/1	0/0	0/1	0/1	0/1	0/0
Chromosome3-970108	INS	PRECISE	992	0	0	0/0	0/0	1/1	0/0	0/0	0/0	0/0	0/0	0/0	0/0	0/0	0/0	0/0	0/0
Chromosome3-978906	DEL	PRECISE	55	0	0	0/0	0/0	0/0	0/0	0/0	0/0	0/0	0/0	0/0	0/0	0/0	0/0	0/0	0/1
Chromosome3-1002229	INS	PRECISE	56	0	0	0/1	0/1	1/1	0/1	0/1	0/0	0/1	0/1	0/0	0/1	0/1	0/1	0/1	0/1
Chromosome3-1011830	DEL	PRECISE	159	0	0	0/1	0/1	1/1	0/1	0/1	0/1	0/1	0/0	0/1	0/1	0/1	0/1	0/1	0/1
Chromosome3-1012741	INS	PRECISE	132	0	0.516	0/1	0/1	0/1	0/0	0/0	0/0	0/0	0/0	0/1	0/0	0/1	0/0	0/1	0/0
Chromosome3-1013848	INS	PRECISE	993	0	0	0/0	0/0	0/1	0/0	0/0	0/0	0/0	0/0	0/0	0/0	0/0	0/0	0/0	0/0
Chromosome3-1016180	INS	PRECISE	10396	0	0	0/0	0/0	0/0	0/0	0/0	0/0	0/1	0/0	0/0	0/0	0/0	0/0	0/0	0/0
Chromosome3-1017222	DEL	PRECISE	76	0	0	0/0	0/0	0/0	0/0	0/0	0/0	0/0	0/0	0/0	0/0	0/1	0/0	0/0	0/0
Chromosome3-1022389	INS	PRECISE	4934	0	0	0/0	0/0	0/0	0/0	0/0	0/0	0/0	0/0	0/0	0/0	1/1	0/0	0/0	0/0
Chromosome3-1022396	BND	PRECISE	0	0	0	0/0	0/0	0/0	0/0	0/0	0/0	0/0	0/0	0/0	0/0	0/1	0/0	0/0	0/0
Chromosome3-1023592	DEL	PRECISE	160	0	0	0/0	0/0	0/1	0/0	0/0	0/0	0/0	0/0	0/0	0/0	0/0	0/0	0/0	0/0
Chromosome3-1025896	INS	PRECISE	130	0.864	0.611	0/1	0/1	1/1	0/1	0/1	0/1	0/1	0/1	0/1	0/1	0/1	0/1	0/1	0/1
Chromosome3-1029159	DEL	PRECISE	489	0	0	0/0	0/0	0/0	0/1	0/1	0/1	0/0	0/0	0/1	0/1	0/0	0/0	0/0	0/0
Chromosome3-1035177	INS	PRECISE	620	0	0	0/0	0/0	0/0	0/0	0/0	0/0	0/0	0/0	0/0	0/0	0/0	0/0	0/0	0/1
Chromosome3-1045133	BND	PRECISE	0	0	0	0/0	0/0	0/1	0/0	0/0	0/0	0/0	0/0	0/0	0/0	0/0	0/0	0/0	0/0
Chromosome3-1045136	BND	PRECISE	0	0	0	0/0	0/0	0/1	0/0	0/0	0/0	0/0	0/0	0/0	0/0	0/0	0/0	0/0	0/0
Chromosome3-1047023	DEL	PRECISE	296	0	0	0/1	0/1	1/1	0/1	0/1	0/1	0/1	0/1	0/1	0/1	1/1	1/1	0/1	0/1
Chromosome3-1049098	DEL	PRECISE	96	0	0	0/0	0/0	0/1	0/0	0/0	0/0	0/0	0/0	0/0	0/0	0/0	0/0	0/0	0/0
Chromosome3-1049543	INS	PRECISE	64	0	0	0/0	0/0	0/0	0/0	0/0	0/0	0/0	0/0	0/0	0/0	0/0	0/0	0/0	0/1
Chromosome3-1050158	DEL	PRECISE	62	0	0	0/0	0/0	0/0	0/0	0/0	0/0	0/0	0/0	0/0	0/0	0/0	0/0	0/0	0/1
Chromosome3-1054631	INS	PRECISE	133	0	0	0/1	0/1	1/1	0/1	0/1	0/1	0/1	0/1	0/1	0/1	0/1	0/1	0/1	0/0
Chromosome3-1055764	DEL	PRECISE	61	0	0	0/0	0/0	0/0	0/0	0/0	0/0	0/0	0/0	0/0	0/1	0/0	0/0	0/0	0/0
Chromosome3-1058065	DEL	PRECISE	57	0	0	0/0	0/0	0/0	0/0	0/0	0/0	0/0	0/0	0/0	0/0	0/0	0/0	0/0	0/1
Chromosome3-1070324	DEL	PRECISE	2793	0.467	0.302	0/1	0/1	1/1	0/0	0/0	0/1	0/1	0/0	0/1	0/0	0/1	0/0	0/0	0/0
Chromosome3-1071071	DEL	PRECISE	2044	0.333	0.333	0/0	0/0	0/0	0/0	0/0	0/0	0/0	0/0	0/0	0/0	0/0	0/0	0/0	0/1
Chromosome3-1071305	DEL	PRECISE	2822	0	0	0/0	0/0	0/0	0/0	0/0	0/0	0/0	0/0	0/0	0/0	0/0	1/1	0/0	0/0
Chromosome3-1074768	INS	PRECISE	286	0	0	0/0	0/0	0/0	0/0	0/0	0/0	0/0	0/0	0/0	0/0	0/0	0/1	0/0	0/0
Chromosome3-1079388	INS	PRECISE	54	0	0	0/0	0/0	0/0	0/0	0/0	0/0	0/0	0/0	0/0	0/1	0/0	0/0	0/0	0/0
Chromosome3-1099644	INS	PRECISE	133	0	0	0/0	0/0	0/0	0/0	0/0	0/0	0/0	0/0	0/0	0/0	0/0	0/1	0/0	0/0
Chromosome3-1099662	INS	PRECISE	2320	0	0	0/0	0/0	0/0	0/0	0/0	0/0	0/0	0/0	0/0	0/0	0/1	0/0	0/0	0/0
Chromosome3-1105407	INS	PRECISE	242	0	0.816	0/1	1/1	0/0	0/0	0/0	0/0	0/0	0/0	1/1	0/0	0/1	0/0	1/1	0/0
Chromosome3-1106869	DEL	PRECISE	55	0	0	0/1	0/1	1/1	0/1	0/1	0/1	0/1	0/0	0/1	0/1	0/1	0/1	0/1	0/0
Chromosome3-1122726	INS	PRECISE	151	0	0.516	0/0	0/0	0/0	0/1	0/1	0/1	0/0	0/1	0/0	0/1	0/0	0/1	0/0	0/0
Chromosome3-1128732	INS	PRECISE	150	0	0.267	0/1	0/1	1/1	0/1	0/1	0/1	0/1	0/1	0/1	0/1	0/1	0/1	0/1	0/1
Chromosome3-1134041	INS	PRECISE	303	0	0.837	0/0	0/1	0/1	0/0	0/0	0/0	0/0	0/0	0/0	0/0	0/1	0/0	0/1	0/0
Chromosome3-1134634	DEL	PRECISE	2875	0	0	0/0	0/0	0/0	0/1	0/1	0/1	0/0	0/1	0/0	0/1	0/0	1/1	0/0	0/1
Chromosome3-1142293	DEL	PRECISE	206	0	0	0/0	0/0	0/0	0/0	0/0	0/0	0/1	0/0	0/0	0/0	0/0	0/0	0/0	0/0
Chromosome3-1144318	DEL	PRECISE	188	0	0	0/0	0/0	0/1	0/0	0/0	0/0	0/1	0/0	0/0	0/0	0/0	0/0	0/0	0/0
Chromosome3-1144409	INS	PRECISE	626	0	0	0/0	0/0	0/0	0/0	0/0	0/0	0/0	0/0	0/0	0/0	0/0	0/0	0/0	0/1
Chromosome3-1146438	INS	PRECISE	95	0	0	0/0	0/0	0/0	0/1	0/1	0/0	0/1	0/1	0/0	0/0	0/0	0/0	0/0	0/0
Chromosome3-1146564	DEL	PRECISE	636	0	0	0/0	0/0	0/0	0/0	0/0	0/0	0/1	0/0	0/0	0/0	0/0	0/0	0/0	0/0
Chromosome3-1151887	INS	PRECISE	355	0	0	0/0	0/0	0/0	0/0	0/0	0/0	0/0	0/0	0/0	0/0	0/0	0/0	0/0	0/1
Chromosome3-1154868	DEL	PRECISE	76	0.577	0.577	0/1	0/1	1/1	0/0	0/0	0/0	0/1	0/0	0/1	0/0	0/0	0/0	0/1	0/0
Chromosome3-1155882	INS	PRECISE	75	0	0	0/0	0/0	0/0	0/0	0/0	0/0	0/0	0/0	0/0	0/0	0/0	1/1	0/0	0/0
Chromosome3-1158842	INS	PRECISE	150	0	0	0/0	0/0	0/0	0/0	0/0	0/0	0/0	0/0	0/0	0/1	0/0	0/0	0/0	0/0
Chromosome3-1159967	INS	PRECISE	339	0	0	0/0	0/0	0/0	0/0	0/0	0/0	0/0	0/0	0/0	0/1	0/0	0/0	0/0	0/0
Chromosome3-1164021	DEL	PRECISE	86	0	0	0/0	0/0	0/0	0/0	0/0	0/0	0/1	0/0	0/0	0/0	0/0	0/0	0/0	0/0
Chromosome3-1164206	INS	PRECISE	1419	0	0	0/0	0/0	0/0	0/0	0/0	0/0	0/0	0/0	0/0	0/0	1/1	0/0	0/0	0/0
Chromosome3-1169379	DEL	PRECISE	59	0	0	0/0	0/0	0/0	0/0	0/0	0/0	0/1	0/0	0/0	0/0	0/0	0/0	0/0	0/0
Chromosome3-1173863	DEL	PRECISE	215	0	0.535	0/1	0/1	1/1	0/1	0/1	0/1	0/1	0/1	0/1	0/1	0/0	0/1	0/1	0/1
Chromosome3-1182067	INS	PRECISE	161	0	0	0/0	0/0	0/0	0/0	0/0	0/0	0/0	0/0	0/0	1/1	0/0	0/0	0/0	0/0
Chromosome3-1182322	INS	PRECISE	6186	0	0	0/0	0/0	0/0	0/0	0/0	0/0	0/0	0/0	0/0	1/1	0/0	0/0	0/0	0/0
Chromosome3-1182324	DEL	PRECISE	1420	0.726	0.726	0/0	0/0	1/1	0/1	0/1	1/1	0/1	1/1	0/0	0/1	0/1	0/1	0/1	0/1
Chromosome3-1189089	DEL	PRECISE	69	0	0	0/1	0/0	1/1	0/1	0/1	0/1	0/1	0/1	0/1	0/1	0/1	0/1	0/1	0/1
Chromosome3-1193786	INS	PRECISE	57	0	0.392	0/1	0/1	1/1	0/1	0/1	0/1	0/1	0/1	0/1	1/1	1/1	0/1	0/1	0/1
Chromosome3-1202103	INS	PRECISE	2006	0	0	0/0	0/0	0/0	0/0	0/0	0/0	0/0	0/0	0/0	0/0	0/0	0/0	0/0	0/1
Chromosome3-1203809	DEL	PRECISE	93	0	0	0/0	0/0	0/0	0/0	0/0	0/0	0/0	0/0	0/0	0/0	0/0	0/0	0/0	0/1
Chromosome3-1204983	DEL	PRECISE	58	0	0	0/0	0/0	0/0	0/0	0/0	0/0	0/0	0/0	0/0	0/0	0/0	0/0	0/0	0/1
Chromosome3-1206791	DEL	PRECISE	264	0	0	0/0	0/0	0/0	0/0	0/0	0/0	0/0	0/0	0/0	0/0	0/1	0/1	0/0	0/0
Chromosome3-1209435	INS	PRECISE	68	0	0	0/0	0/0	0/0	0/0	0/0	0/0	0/0	0/0	0/0	0/1	0/1	0/0	0/0	0/0
Chromosome3-1210108	DEL	PRECISE	68	0	0	0/1	0/1	1/1	0/0	0/1	0/1	0/1	0/1	0/1	0/1	0/1	0/1	0/1	0/1
Chromosome3-1211620	INS	PRECISE	9858	0	0	0/0	0/0	0/0	0/0	0/0	0/0	0/0	0/0	0/0	0/0	0/0	0/0	0/1	0/0

Chromosome3-1211625	BND	PRECISE	0	0	0	0/0	0/0	0/0	0/0	0/0	0/0	0/0	0/0	0/0	0/0	0/0	0/0	0/1	0/0
Chromosome3-1211773	DEL	PRECISE	624	0	0	0/0	0/0	0/0	0/0	0/0	0/0	0/0	0/0	0/0	0/0	0/0	0/0	0/0	0/1
Chromosome3-1217878	INS	PRECISE	455	0	0	0/0	0/0	0/0	0/0	0/0	0/0	0/1	0/0	0/0	0/0	0/0	0/0	0/0	0/0
Chromosome3-1218414	DEL	PRECISE	93	0	0	0/1	0/1	1/1	0/0	0/1	0/1	0/1	0/1	0/1	0/1	0/1	0/1	0/1	0/1
Chromosome3-1224677	INS	PRECISE	73	0	0.475	0/1	0/1	1/1	0/1	0/1	0/1	0/1	0/1	0/1	0/1	0/1	0/1	0/1	0/1
Chromosome3-1225112	INS	PRECISE	2903	0	0	0/0	0/0	0/0	0/0	0/0	0/0	0/0	0/0	0/0	1/1	0/0	0/0	0/0	0/0
Chromosome3-1226492	DEL	PRECISE	201	0	0	0/1	0/1	1/1	0/1	0/1	0/1	0/1	0/1	0/1	0/1	0/1	0/1	0/1	0/1
Chromosome3-1228268	DEL	PRECISE	113	0	0	0/1	0/1	1/1	0/1	0/1	0/1	0/1	0/1	0/1	0/1	0/1	0/1	0/1	0/0
Chromosome3-1231881	DEL	PRECISE	124	0	0	0/1	0/1	0/1	0/0	0/0	0/0	0/0	0/0	0/1	0/0	0/1	0/0	0/1	0/0
Chromosome3-1231981	INS	PRECISE	1057	0	0	0/0	0/0	0/0	0/0	0/0	0/0	0/0	0/0	0/0	0/0	0/0	0/0	0/0	0/1
Chromosome3-1233851	INS	PRECISE	1199	0	0	0/0	0/0	0/1	0/0	0/0	0/0	0/0	0/0	0/0	0/0	0/0	0/0	0/0	0/0
Chromosome3-1235185	DEL	PRECISE	164	0	0	0/1	0/1	1/1	0/1	0/1	0/1	0/0	0/1	0/1	0/1	0/1	0/1	0/1	0/0
Chromosome3-1238576	INS	PRECISE	68	0	0.363	0/1	0/1	1/1	1/1	1/1	1/1	0/1	0/1	1/1	1/1	1/1	1/1	0/1	0/1
Chromosome3-1240809	INS	PRECISE	4463	0	0	0/0	0/0	0/0	0/0	0/0	0/0	0/0	0/0	0/0	0/0	0/0	0/0	0/0	0/1
Chromosome3-1240884	DEL	PRECISE	161	0	0	0/1	0/1	0/1	0/0	0/0	0/0	0/0	0/0	0/1	0/0	1/1	0/0	0/1	0/0
Chromosome3-1242406	DEL	PRECISE	69	0	0	0/0	0/0	0/0	0/1	0/1	1/1	0/0	0/0	0/1	0/1	0/1	0/0	0/0	0/0
Chromosome3-1243603	DEL	PRECISE	650	0	0	0/0	0/1	1/1	1/1	1/1	1/1	0/0	0/1	1/1	1/1	1/1	1/1	0/1	0/1
Chromosome3-1249321	INS	PRECISE	657	0	0.548	0/1	0/1	0/1	0/0	0/0	0/0	0/0	0/0	0/1	0/0	0/0	0/0	0/1	0/0
Chromosome3-1252852	INS	PRECISE	686	0	0.745	0/1	0/1	1/1	0/1	0/1	0/1	0/1	0/1	0/1	0/1	0/0	0/1	0/1	0/1
Chromosome3-1255282	DEL	PRECISE	52	0	0	0/0	0/0	0/0	0/1	0/1	0/1	0/0	0/0	0/1	0/0	0/0	0/0	0/0	0/0
Chromosome3-1275700	DEL	PRECISE	150	0	0	0/1	0/1	1/1	0/1	0/1	0/1	0/1	0/1	0/1	0/0	0/1	0/1	0/1	0/1
Chromosome3-1276443	DEL	PRECISE	562	0	0	1/1	1/1	0/0	0/1	0/1	1/1	1/1	1/1	1/1	0/1	1/1	1/1	1/1	1/1
Chromosome3-1281194	DEL	PRECISE	1308	0.726	0.267	0/1	0/1	1/1	0/1	0/1	0/1	0/1	0/1	0/1	0/1	0/1	0/1	0/1	0/1
Chromosome3-1294995	DEL	PRECISE	90	0	0	0/0	0/0	0/0	0/0	0/0	0/0	0/0	0/0	0/0	0/0	0/0	0/0	0/0	0/1
Chromosome3-1295846	INS	PRECISE	57	0	0	0/0	0/0	0/1	0/0	0/0	0/0	0/0	0/0	0/0	0/0	0/0	0/0	0/0	0/0
Chromosome3-1296449	DEL	PRECISE	92	0.277	0.277	0/1	0/1	1/1	0/1	0/1	1/1	0/0	0/1	1/1	1/1	1/1	0/1	0/1	0/1
Chromosome3-1297048	DEL	PRECISE	1112	0	0	0/0	0/0	0/0	1/1	1/1	0/0	0/0	0/0	0/0	0/0	0/0	0/0	0/0	0/0
Chromosome3-1317034	INS	PRECISE	2198	0	0	0/0	0/0	0/0	0/0	0/0	0/0	0/0	0/0	0/0	0/0	0/0	0/0	0/0	0/1
Chromosome3-1323759	INS	PRECISE	207	0	0.447	0/0	0/0	0/0	1/1	1/1	1/1	0/0	0/0	1/1	0/0	1/1	0/0	0/0	0/0
Chromosome3-1335107	INS	PRECISE	6831	0	0	0/0	0/0	0/0	0/0	0/0	0/0	0/1	0/0	0/0	0/0	0/0	0/0	0/0	0/0
Chromosome3-1339074	INS	PRECISE	102	0	0	0/0	0/0	0/0	1/1	1/1	1/1	0/0	0/0	1/1	0/0	0/0	0/0	0/0	0/0
Chromosome3-1339774	DEL	PRECISE	58	0	0	0/0	0/0	0/0	0/0	0/0	0/0	0/0	0/0	0/0	0/0	0/0	1/1	0/0	0/0
Chromosome3-1341575	INS	PRECISE	1379	0	0	0/0	0/0	0/0	0/0	0/0	0/0	0/0	0/0	0/0	0/0	0/0	1/1	0/0	0/0
Chromosome3-1344126	DEL	PRECISE	73	0	0	0/1	0/1	1/1	0/1	0/1	0/1	0/1	0/1	0/1	0/1	0/1	0/1	0/0	0/1
Chromosome3-1353444	INS	PRECISE	8479	0	0	0/0	0/0	0/0	0/0	0/0	0/0	0/0	0/0	0/0	0/0	0/0	0/0	0/0	0/1
Chromosome3-1356786	INS	PRECISE	121	0	0	0/0	0/0	0/0	0/0	0/0	0/0	0/0	0/0	0/0	0/0	0/0	0/1	0/0	0/0
Chromosome3-1362858	INS	PRECISE	230	0	0	0/0	0/0	0/0	0/0	0/0	0/0	0/0	0/0	0/0	0/0	0/0	0/0	0/0	0/1
Chromosome3-1366689	INS	PRECISE	276	0	0.469	0/1	0/1	1/1	0/1	0/1	0/1	0/1	0/1	0/1	0/1	0/1	0/1	0/1	0/1
Chromosome3-1366775	INS	PRECISE	1371	0	0	0/0	0/0	0/0	0/0	0/0	0/0	0/0	0/0	0/0	0/0	0/0	0/1	0/0	0/0
Chromosome3-1366777	DUP	PRECISE	11853849	0	0	0/0	0/0	0/0	0/0	0/0	0/0	0/0	0/0	0/0	0/0	0/0	0/1	0/0	0/0
Chromosome3-1368001	DEL	PRECISE	107	0	0	0/0	0/0	0/0	0/0	0/0	0/0	0/0	0/0	0/0	0/0	0/0	0/1	0/0	0/0
Chromosome3-1368090	INS	PRECISE	1215	0	0	0/0	0/0	0/1	0/0	0/0	0/0	0/0	0/0	0/0	0/0	0/0	0/0	0/0	0/0
Chromosome3-1369105	INS	PRECISE	81	0	0	0/0	0/0	0/0	0/0	0/0	0/0	0/1	0/0	0/0	0/0	0/0	0/0	0/0	0/0
Chromosome3-1386930	DEL	PRECISE	361	0	0	0/0	0/0	0/0	0/0	0/0	0/0	0/0	0/0	0/0	0/0	0/0	0/1	0/0	0/0
Chromosome3-1398457	DEL	PRECISE	199	0	0	0/0	0/0	0/0	0/0	0/0	0/0	0/0	0/0	0/0	0/1	1/1	0/1	0/0	0/0
Chromosome3-1405729	INS	PRECISE	125	0	0	0/1	0/0	0/1	0/0	0/0	0/0	0/0	0/0	0/0	0/0	0/0	0/0	0/0	0/0
Chromosome3-1414729	INS	PRECISE	200	0	0	0/0	0/0	0/0	0/0	0/0	0/0	0/0	0/0	0/0	0/0	0/0	0/0	0/0	0/1
Chromosome3-1417788	INS	PRECISE	1275	0	0	0/0	0/0	0/0	0/0	0/0	0/0	0/0	0/0	0/0	0/0	0/0	0/0	0/0	0/1
Chromosome3-1428095	INS	PRECISE	401	0	0	0/0	0/0	0/0	0/0	0/0	0/0	0/0	0/0	0/0	0/0	0/0	0/0	0/0	0/1
Chromosome3-1444094	DEL	PRECISE	612	0	0	0/1	0/1	1/1	0/1	0/1	0/1	0/1	0/1	0/1	0/1	0/1	0/1	0/1	0/1
Chromosome3-1447871	INS	PRECISE	277	0	0.5	0/0	0/0	0/0	0/1	0/1	0/1	0/0	0/0	0/1	0/0	0/0	0/0	0/0	0/0
Chromosome3-1468082	INS	PRECISE	1808	0	0	0/0	0/0	0/0	0/0	0/0	0/0	0/0	0/0	0/0	0/0	0/0	0/1	0/0	0/0
Chromosome3-1477512	INS	PRECISE	1636	0	0	0/0	0/0	0/0	0/0	0/0	0/0	0/0	0/0	0/0	0/0	0/0	0/0	0/0	0/1
Chromosome3-1477545	INS	PRECISE	179	0	0	0/0	0/0	0/0	0/0	0/0	0/0	0/1	0/0	0/0	0/0	0/0	0/0	0/0	0/0
Chromosome3-1481573	INS	PRECISE	7875	0	0	0/0	0/0	0/0	0/0	0/0	0/0	0/0	0/0	0/0	1/1	0/0	0/0	0/0	0/0
Chromosome3-1482485	INS	PRECISE	2046	0	0	0/0	0/0	0/0	0/0	0/0	0/0	0/0	0/0	0/0	1/1	0/0	0/0	0/0	0/0
Chromosome3-1483723	DEL	PRECISE	363	0	0	0/0	0/0	1/1	1/1	1/1	1/1	0/1	0/0	1/1	1/1	1/1	1/1	0/0	0/1
Chromosome3-1484611	INS	PRECISE	137	0	0	0/0	0/0	0/0	0/0	0/0	0/0	0/0	0/0	0/0	0/0	0/0	1/1	0/0	0/0
Chromosome3-1485700	DEL	PRECISE	50	0	0	0/0	0/0	0/0	0/0	0/0	0/0	0/0	0/0	0/0	0/0	0/0	1/1	0/0	0/0
Chromosome3-1498990	DEL	PRECISE	81	0	0	0/0	0/0	0/0	0/0	0/0	0/0	0/0	0/0	0/0	1/1	0/0	0/0	0/0	0/0
Chromosome3-1499609	INS	PRECISE	4590	0	0	0/0	0/0	0/0	0/0	0/0	0/0	0/0	0/0	0/0	1/1	0/0	0/0	0/0	0/0
Chromosome3-1509365	INS	PRECISE	97	0	0	0/0	0/0	0/0	0/0	0/0	0/0	0/0	0/0	0/0	0/0	0/0	0/0	0/0	0/1
Chromosome3-1511456	DEL	PRECISE	63	0	0	0/0	0/1	1/1	0/0	0/0	0/0	0/0	0/0	0/1	0/0	0/1	0/0	0/1	0/1
Chromosome3-1530124	DEL	PRECISE	363	0	0	0/1	0/1	1/1	1/1	1/1	1/1	0/1	0/0	1/1	0/0	1/1	1/1	0/1	0/1
Chromosome3-1550409	DEL	PRECISE	942	0	0	0/1	0/1	1/1	1/1	1/1	1/1	0/1	1/1	1/1	1/1	0/1	1/1	0/1	0/0
Chromosome3-1552769	INS	PRECISE	153	0	0	0/0	0/0	0/0	0/0	0/0	0/0	0/0	0/0	0/0	0/0	0/0	0/0	0/0	0/1
Chromosome3-1553879	INS	PRECISE	65	0	0	0/0	0/0	0/0	0/0	0/0	0/0	0/0	0/0	0/0	0/0	0/0	0/1	0/0	0/0
Chromosome3-1554194	DEL	PRECISE	72	0	0	0/0	0/0	0/0	0/0	0/0	0/0	0/1	0/0	0/0	0/0	0/0	0/0	0/0	0/0
Chromosome3-1558225	INS	PRECISE	1253	0	0	0/0	0/0	0/0	0/0	0/0	0/0	0/0	0/0	0/0	0/0	0/0	0/1	0/0	0/0
Chromosome3-1575812	DEL	PRECISE	88	0	0	0/1	0/1	1/1	0/0	0/0	0/0	0/1	0/0	0/1	0/0	0/1	0/0	0/0	0/0
Chromosome3-1599607	INS	PRECISE	61	0	0.267	0/1	1/1	0/0	1/1	1/1	0/1	0/1	1/1	1/1	1/1	1/1	0/1	0/1	0/1
Chromosome3-1600122	DEL	PRECISE	318	0.535	0.267	0/1	0/1	1/1	0/1	0/1	0/1	0/1	0/1	0/1	0/1	0/1	0/1	0/1	0/1
Chromosome3-1600931	DEL	PRECISE	83	0	0	0/1	0/1	1/1	0/1	0/1	0/1	0/1	0/1	0/1	0/1	0/1	0/1	0/1	0/1

Chromosome3-1601662	BND	PRECISE	0	0	0	0/0	0/0	0/0	0/0	0/0	0/0	0/0	0/0	0/1	0/0	0/0	0/0	0/0	0/0
Chromosome3-1612461	INS	PRECISE	162	0	0.426	0/1	0/1	1/1	0/1	0/1	0/1	0/1	0/1	0/1	0/1	0/1	0/1	0/1	0/1
Chromosome3-1614244	DEL	PRECISE	571	0	0	0/1	0/1	1/1	0/1	0/1	0/1	0/1	0/1	0/1	0/1	0/1	0/1	0/1	0/1
Chromosome3-1616398	DEL	PRECISE	65	0	0	0/0	0/0	0/0	0/0	0/0	0/0	0/0	0/0	0/0	0/1	0/0	0/0	0/0	0/0
Chromosome3-1624202	DEL	PRECISE	42941905	0	0	0/1	0/0	0/1	0/0	0/1	0/1	0/1	0/0	0/0	0/1	0/1	0/0	0/0	0/0
Chromosome3-1626835	DEL	PRECISE	89	0	0	0/1	0/1	1/1	0/1	0/0	0/0	0/1	0/1	0/0	0/1	0/1	0/1	0/1	0/1
Chromosome3-1631679	INS	PRECISE	137	0	0	0/0	0/0	0/0	0/0	0/0	0/0	0/0	0/0	0/0	0/0	0/0	0/1	0/0	0/0
Chromosome3-1635673	DEL	PRECISE	718	0.535	0.267	0/1	0/1	1/1	0/1	0/1	0/0	0/1	0/1	0/0	0/1	0/1	0/1	0/1	0/1
Chromosome3-1638080	DEL	PRECISE	386	0	0	0/1	0/1	1/1	1/1	1/1	1/1	0/1	0/1	1/1	1/1	1/1	1/1	0/1	0/1
Chromosome3-1638607	INS	PRECISE	371	0	0	0/0	0/0	0/0	0/0	0/0	0/0	0/0	0/0	0/0	0/0	0/0	0/0	0/0	0/1
Chromosome3-1644236	DEL	PRECISE	1664	0	0	0/0	0/1	1/1	0/1	0/1	0/1	0/1	0/1	0/1	0/1	0/1	0/1	0/1	0/1
Chromosome3-1644625	INS	PRECISE	92	0	0	0/0	0/0	0/0	0/0	0/0	0/0	0/0	0/0	0/0	0/0	0/0	0/1	0/0	0/0
Chromosome3-1645974	DEL	PRECISE	369	0	0	0/0	0/1	1/1	0/1	1/1	1/1	0/1	0/1	0/1	0/1	1/1	0/1	0/1	0/1
Chromosome3-1647372	DEL	PRECISE	209	0.267	0	0/0	0/1	1/1	0/1	0/1	0/1	0/1	0/1	0/1	0/1	0/1	0/1	0/1	0/1
Chromosome3-1648709	DEL	PRECISE	119	0	0	0/0	0/0	0/0	0/0	0/0	0/0	0/0	0/0	0/0	0/1	0/0	0/0	0/0	0/0
Chromosome3-1657925	INS	PRECISE	136	0	0	0/0	0/0	0/0	0/1	0/1	0/1	0/0	0/1	0/0	0/1	0/0	0/1	0/0	0/0
Chromosome3-1658293	INS	PRECISE	849	0	0	0/0	0/0	0/0	0/0	0/0	0/0	0/0	0/0	0/0	0/1	0/0	0/0	0/0	0/0
Chromosome3-1665103	DEL	PRECISE	1395	0	0	0/0	0/0	0/0	0/0	0/0	0/0	0/0	0/0	0/0	0/1	0/1	0/0	0/0	0/0
Chromosome3-1669365	DEL	PRECISE	968	0	0	0/1	0/1	1/1	0/1	0/0	0/1	0/1	0/1	0/1	0/1	0/1	0/0	0/1	0/1
Chromosome3-1669936	INS	PRECISE	211	0	0	0/0	0/0	0/0	0/0	0/0	0/0	0/0	0/0	0/0	0/0	0/0	1/1	0/0	0/0
Chromosome3-1675908	DEL	PRECISE	364	0	0	0/1	0/1	1/1	1/1	1/1	1/1	0/1	0/1	1/1	1/1	1/1	1/1	0/1	0/1
Chromosome3-1680015	DEL	PRECISE	123	0	0	0/0	0/0	0/0	0/0	0/0	0/0	0/0	0/0	0/0	0/1	0/0	0/1	0/0	0/0
Chromosome3-1681893	DEL	PRECISE	51	0	0	0/1	0/0	0/1	0/0	0/0	0/0	0/1	0/0	0/0	0/0	0/0	0/0	0/0	0/0
Chromosome3-1695278	DEL	PRECISE	201	0.535	0.267	0/1	0/1	1/1	0/1	0/1	0/0	0/1	0/1	0/1	0/1	0/1	0/1	0/1	0/1
Chromosome3-1699671	BND	PRECISE	0	0	0	0/0	0/0	0/1	0/0	0/0	0/0	0/0	0/0	0/0	0/0	0/0	0/0	0/0	0/0
Chromosome3-1699960	INS	PRECISE	238	0	0.5	0/0	0/0	0/0	0/1	0/1	0/1	0/0	0/0	0/1	0/0	0/0	0/0	0/0	0/0
Chromosome3-1706759	INS	PRECISE	623	0	0	0/0	0/0	0/0	0/0	0/0	0/0	0/0	0/0	0/0	0/0	0/0	0/1	0/0	0/0
Chromosome3-1708022	INS	PRECISE	927	0	0	0/0	0/0	0/1	0/0	0/0	0/0	0/0	0/0	0/0	0/0	0/0	0/0	0/0	0/0
Chromosome3-1711487	DEL	PRECISE	2349	0.802	0.802	0/1	0/1	1/1	1/1	1/1	1/1	0/1	0/1	1/1	1/1	1/1	1/1	0/1	1/1
Chromosome3-1714256	DEL	PRECISE	389	0	0	0/1	0/1	1/1	0/1	0/1	0/1	0/0	0/1	0/1	0/1	0/1	0/1	0/1	0/1
Chromosome3-1727387	BND	PRECISE	0	0	0	0/0	0/0	0/0	0/0	0/0	0/0	0/0	0/0	0/0	0/0	0/1	0/0	0/0	0/0
Chromosome3-1728674	DEL	PRECISE	650	0	0	0/0	0/0	1/1	1/1	0/1	0/1	0/1	0/1	0/0	0/1	1/1	1/1	0/0	0/0
Chromosome3-1733829	DEL	PRECISE	54	0	0	0/0	0/0	0/0	0/1	0/1	0/1	0/0	0/0	1/1	0/0	0/0	0/0	0/0	0/0
Chromosome3-1736343	INS	PRECISE	453	0	0	0/0	0/0	0/0	0/0	0/0	0/0	0/0	0/0	0/0	0/0	0/0	0/0	0/0	0/1
Chromosome3-1740034	INS	PRECISE	126	0	0	0/0	0/0	0/0	0/0	0/0	0/0	0/0	0/0	0/0	0/0	0/0	0/0	0/0	0/1
Chromosome3-1740937	DEL	PRECISE	369	0	0	0/1	0/0	1/1	0/1	0/1	0/1	0/1	0/1	0/1	0/1	0/1	0/1	0/1	0/1
Chromosome3-1746368	DEL	PRECISE	92	0	0	0/1	0/1	1/1	0/1	0/1	0/1	0/1	0/1	0/1	0/1	0/1	0/1	0/1	0/1
Chromosome3-1755160	DEL	PRECISE	57	0	0	0/0	0/0	0/0	0/0	0/0	0/0	0/0	0/0	0/0	0/0	0/0	0/0	0/0	0/1
Chromosome3-1761454	INS	PRECISE	4470	0	0	0/0	0/0	0/0	0/0	0/0	0/0	0/0	0/0	0/0	0/1	0/0	0/0	0/0	0/0
Chromosome3-1761666	INS	PRECISE	2525	0	0	0/0	0/0	0/0	0/0	0/0	0/0	0/0	0/0	0/0	0/0	0/0	1/1	0/0	0/0
Chromosome3-1769221	INS	PRECISE	611	0	0	0/0	0/0	0/0	0/0	0/0	0/0	0/0	0/0	0/0	0/0	0/1	0/0	0/0	0/0
Chromosome3-1769346	DEL	PRECISE	785	0	0	0/1	0/1	1/1	0/1	0/1	0/1	0/1	0/0	0/1	0/1	0/1	0/1	0/1	0/1
Chromosome3-1782022	DEL	PRECISE	647	0	0	0/0	0/0	0/0	0/0	0/0	0/0	0/0	0/0	0/0	0/0	1/1	0/0	0/0	0/0
Chromosome3-1782692	DEL	PRECISE	189	0	0	0/0	0/0	0/0	0/0	0/0	0/0	0/0	0/0	0/0	0/0	1/1	0/0	0/0	0/0
Chromosome3-1785549	DEL	PRECISE	168	0	0	0/0	0/0	0/0	0/0	0/0	0/0	0/0	0/0	0/0	0/0	1/1	0/0	0/0	0/0
Chromosome3-1786482	DEL	PRECISE	275	0.408	0	0/0	0/0	0/0	1/1	1/1	1/1	0/0	0/0	1/1	1/1	1/1	0/0	0/0	0/0
Chromosome3-1787224	DUP	PRECISE	89	0	0	0/0	0/0	0/0	0/0	0/0	0/0	0/0	0/0	0/0	0/0	0/0	0/0	0/0	0/1
Chromosome3-1787348	BND	PRECISE	0	0	0	0/0	0/0	0/0	0/0	0/0	0/0	0/0	0/0	0/0	0/0	0/0	0/0	0/0	0/1
Chromosome3-1787937	DEL	PRECISE	180	0	0	0/0	0/0	0/0	1/1	0/1	1/1	0/0	0/0	1/1	1/1	0/1	0/0	0/0	0/0
Chromosome3-1796221	DEL	PRECISE	50	0.516	0.516	0/0	0/0	0/0	0/1	1/1	1/1	0/0	0/0	1/1	1/1	1/1	0/0	0/0	0/0
Chromosome3-1798948	INV	PRECISE	30820730	0.816	0	0/0	0/0	0/0	0/1	0/0	0/0	0/0	0/0	0/1	0/1	0/0	0/0	0/0	0/0
Chromosome3-1799757	INS	PRECISE	50	0	0.408	0/0	0/0	0/0	1/1	1/1	1/1	0/0	0/0	1/1	1/1	0/1	0/0	0/0	0/0
Chromosome3-1810049	INS	PRECISE	3369	0	0	0/0	0/0	0/0	0/0	0/0	0/0	0/0	0/0	0/0	0/0	0/0	0/0	0/0	0/1
Chromosome3-1830742	DEL	PRECISE	137	0	0	0/1	0/1	1/1	1/1	1/1	1/1	0/1	0/0	1/1	1/1	1/1	1/1	0/1	0/1
Chromosome3-1843973	DEL	PRECISE	211	0	0	0/1	0/1	1/1	1/1	1/1	1/1	0/1	0/0	1/1	1/1	1/1	0/0	0/1	0/1
Chromosome3-1845342	INS	PRECISE	85	0	0.577	0/0	0/0	0/0	0/1	0/1	0/0	0/0	0/0	0/0	0/0	0/0	0/0	0/0	0/0
Chromosome3-1845910	INS	PRECISE	657	0	0.707	0/1	0/0	0/1	0/0	0/0	0/0	0/0	0/0	0/0	0/0	0/0	0/0	0/0	0/0
Chromosome3-1849637	INS	PRECISE	139	0	0.688	0/1	0/1	1/1	0/1	0/1	0/0	0/0	0/0	0/1	0/1	0/1	0/0	0/1	0/1
Chromosome3-1852567	INS	PRECISE	652	0	0	0/0	0/0	0/0	0/0	0/0	0/0	0/1	0/0	0/0	0/0	0/0	0/0	0/0	0/0
Chromosome3-1860406	INS	PRECISE	2502	0	0	0/0	0/0	0/0	0/0	0/0	0/0	0/0	0/0	0/0	0/0	0/0	0/0	0/0	1/1
Chromosome3-1862822	INS	PRECISE	52	0	0.267	0/1	0/1	1/1	1/1	1/1	1/1	0/1	0/1	1/1	1/1	0/1	1/1	0/1	0/1
Chromosome3-1881278	INS	PRECISE	57	0	0	0/0	0/0	0/0	0/0	0/0	0/0	0/0	0/0	0/0	0/0	0/1	0/0	0/0	0/0
Chromosome3-1883553	DEL	PRECISE	86	0	0	0/0	0/1	0/1	0/1	0/1	0/1	0/0	0/1	0/1	0/1	0/1	0/1	0/1	0/0
Chromosome3-1896349	INS	PRECISE	121	0	0	0/0	0/0	0/0	0/0	0/0	0/0	0/0	0/0	0/0	0/0	0/1	0/1	0/0	0/0
Chromosome3-1901725	DEL	PRECISE	551	0	0	0/0	0/0	0/0	0/0	0/0	0/0	0/0	0/0	0/0	0/0	0/1	0/0	0/0	0/0
Chromosome3-1907308	DEL	PRECISE	67	0	0	0/1	0/1	1/1	1/1	1/1	1/1	0/1	0/1	1/1	1/1	0/1	1/1	0/1	0/1
Chromosome3-1911936	INS	PRECISE	1001	0	0	0/0	0/0	0/0	0/0	0/0	0/0	0/0	0/0	0/0	0/0	0/0	0/0	0/0	0/1
Chromosome3-1913121	INS	PRECISE	149	0	0	0/0	0/0	0/0	0/0	0/0	0/0	0/0	0/0	0/0	0/0	0/0	0/0	0/0	0/1
Chromosome3-1914423	DEL	PRECISE	1476	0	0	0/0	0/0	0/0	0/1	0/1	0/0	0/0	0/0	0/1	0/1	0/0	0/1	0/0	0/0
Chromosome3-1919833	DEL	PRECISE	1022	0	0	0/1	0/1	1/1	0/1	0/1	1/1	0/1	0/1	0/1	0/1	0/1	1/1	0/1	0/1
Chromosome3-1921003	DEL	PRECISE	78	0	0	0/1	0/1	1/1	0/1	0/1	0/1	0/1	0/1	0/1	0/1	0/1	0/1	0/1	0/1
Chromosome3-1922927	DEL	PRECISE	333	0	0	0/1	0/1	1/1	0/1	0/1	1/1	0/1	0/1	0/1	0/1	0/1	0/1	0/1	0/1
Chromosome3-1923118	INS	PRECISE	74	0	0	0/0	0/0	0/0	0/0	0/0	0/0	0/0	0/0	0/0	0/0	0/1	0/0	0/0	0/0

Chromosome3-1924004	INS	PRECISE	888	0	0	0/0	0/0	0/0	0/0	0/0	0/0	0/0	0/0	0/0	0/0	0/1	0/0	0/0	0/0
Chromosome3-1925298	DEL	PRECISE	235	0	0	0/1	0/1	1/1	0/1	0/1	0/1	0/1	0/1	0/1	0/1	0/1	0/1	0/1	0/1
Chromosome3-1931579	INS	PRECISE	72	0	0.447	0/0	0/0	0/0	0/1	0/1	0/1	0/0	0/0	0/1	0/0	0/1	0/0	0/0	0/0
Chromosome3-1937689	DEL	PRECISE	68	0.786	0.786	0/0	0/1	0/1	0/1	0/1	0/1	0/0	0/1	0/1	0/1	0/1	0/1	0/1	0/0
Chromosome3-1942108	DEL	PRECISE	103	0	0	0/0	0/0	0/0	0/1	0/1	0/1	0/0	0/1	0/0	0/1	0/0	0/1	0/0	0/1
Chromosome3-1942343	INS	PRECISE	207	0	0	0/0	0/0	0/0	0/0	0/0	0/0	0/0	0/0	0/0	0/0	0/0	0/1	0/0	0/0
Chromosome3-1961140	DEL	PRECISE	951	0	0	0/0	0/0	0/0	1/1	1/1	1/1	0/1	0/0	1/1	0/0	0/0	0/1	0/0	0/0
Chromosome3-1965551	BND	PRECISE	0	0	0	0/0	0/0	0/0	0/1	0/1	0/0	0/0	0/0	0/1	0/0	0/0	0/0	0/0	0/0
Chromosome3-1969227	DEL	PRECISE	1363	0	0	0/0	0/0	0/0	0/0	0/0	0/0	0/0	0/0	0/0	0/0	0/0	0/0	0/0	0/1
Chromosome3-1972202	INS	PRECISE	141	0	0.363	0/1	0/1	1/1	1/1	1/1	1/1	0/1	0/1	1/1	0/1	0/1	0/1	0/1	0/1
Chromosome3-1976367	INS	PRECISE	61	0	0.876	0/0	0/0	1/1	0/0	0/0	0/1	0/1	0/0	0/0	0/0	0/0	0/0	0/0	0/0
Chromosome3-1976766	INS	PRECISE	985	0	0.707	0/0	0/0	0/0	0/0	0/0	0/0	0/0	0/0	0/0	1/1	1/1	0/0	0/0	0/0
Chromosome3-1983715	INS	PRECISE	1757	0	0	0/0	0/0	0/0	0/0	0/0	0/0	0/0	0/0	0/0	0/1	1/1	0/0	0/0	0/0
Chromosome3-1987986	INS	PRECISE	5631	0	0	0/0	0/0	0/0	0/0	0/0	0/0	0/0	0/0	0/0	0/0	0/0	1/1	0/0	0/0
Chromosome3-1992249	DEL	PRECISE	295	0	0	0/1	0/1	1/1	0/1	0/1	0/1	0/1	0/1	0/1	1/1	1/1	1/1	0/1	0/1
Chromosome3-2005631	DEL	PRECISE	165	0	0	0/0	0/0	0/0	0/1	0/1	0/1	0/0	0/0	0/1	1/1	0/1	0/0	0/0	0/0
Chromosome3-2025898	INS	PRECISE	350	0	0.577	0/0	0/0	0/0	0/1	0/1	0/1	0/0	0/0	0/1	0/0	0/0	0/0	0/0	0/0
Chromosome3-2034517	INS	PRECISE	53	0	0.267	0/1	0/1	1/1	0/1	0/1	0/1	0/1	0/1	0/1	0/1	0/1	0/1	0/1	0/1
Chromosome3-2040786	INS	PRECISE	428	0	0	0/0	0/0	0/0	0/0	0/0	0/0	0/0	0/0	0/0	0/0	0/1	0/0	0/0	0/0
Chromosome3-2044397	DEL	PRECISE	830	0	0	0/0	0/0	0/0	0/0	0/0	0/0	0/0	0/0	0/0	0/0	0/0	0/1	0/0	0/0
Chromosome3-2046865	INS	PRECISE	127	0	0	0/1	0/1	1/1	0/1	0/1	0/1	0/1	0/1	0/1	0/1	0/1	0/1	0/1	0/1
Chromosome3-2050285	DEL	PRECISE	108	0	0	0/0	0/0	0/0	0/0	0/0	0/0	0/0	0/0	0/0	0/0	0/1	0/0	0/0	0/0
Chromosome3-2054188	DEL	PRECISE	401	0	0	0/0	0/0	0/0	0/1	0/1	0/1	0/0	0/0	0/1	0/0	0/0	0/1	0/0	0/0
Chromosome3-2060853	DEL	PRECISE	621	0	0	0/1	0/1	1/1	0/1	0/1	0/1	0/1	0/1	0/1	0/1	1/1	0/1	0/1	0/1
Chromosome3-2066045	INS	PRECISE	131	0	0	0/0	0/0	0/0	0/0	0/0	0/0	0/0	0/0	0/0	0/0	0/0	0/0	0/0	0/1
Chromosome3-2072592	DEL	PRECISE	121	0	0	0/1	0/1	1/1	1/1	1/1	1/1	0/1	0/1	1/1	1/1	1/1	1/1	0/1	0/1
Chromosome3-2073172	INS	PRECISE	109	0	0.267	0/1	0/1	1/1	0/1	0/1	0/1	0/1	0/1	0/1	0/1	0/1	0/1	0/1	0/1
Chromosome3-2091104	DEL	PRECISE	150	0	0	0/1	0/1	1/1	0/1	0/1	0/1	0/1	0/1	0/1	0/1	0/1	0/1	0/1	0/1
Chromosome3-2098990	DEL	PRECISE	161	0.5	0.5	0/0	0/0	0/0	0/1	0/1	0/1	0/0	0/0	0/1	0/0	0/0	0/0	0/0	0/0
Chromosome3-2117091	DEL	PRECISE	1708	0	0	0/1	0/1	1/1	0/1	0/1	0/1	0/1	0/0	0/1	0/0	0/1	0/0	0/1	0/0
Chromosome3-2118214	INS	PRECISE	369	0	0	0/0	0/0	0/0	0/0	0/0	0/0	0/0	0/0	0/0	0/0	0/0	1/1	0/0	0/0
Chromosome3-2132376	DEL	PRECISE	100	0	0	0/0	0/0	0/0	0/0	0/0	0/0	0/0	0/0	0/0	0/0	0/0	0/0	0/0	0/1
Chromosome3-2132553	DUP	PRECISE	6325612	0	0	0/0	0/0	0/0	0/0	0/1	0/0	0/0	0/0	0/0	0/0	0/0	0/0	0/0	0/0
Chromosome3-2144503	DEL	PRECISE	2475	0	0	0/0	0/0	0/0	0/0	0/0	0/0	0/0	0/0	0/0	0/0	0/0	0/0	0/0	1/1
Chromosome3-2145500	DEL	PRECISE	1292	0	0	0/0	0/0	0/0	0/0	0/0	0/0	0/0	0/0	0/0	0/0	0/1	0/0	0/0	0/0
Chromosome3-2146632	DEL	PRECISE	65	0	0	0/0	0/1	0/1	0/0	0/0	0/0	0/0	0/0	0/1	0/0	0/1	0/0	0/1	0/0
Chromosome3-2157831	INS	PRECISE	137	0	0.363	0/1	0/1	1/1	0/1	0/1	0/1	0/1	0/1	0/1	0/1	0/1	0/1	1/1	0/1
Chromosome3-2160252	DEL	PRECISE	69	0	0	0/1	0/1	1/1	0/1	0/1	0/1	0/0	0/1	0/1	0/1	0/1	0/1	1/1	0/0
Chromosome3-2173053	DEL	PRECISE	142	0	0	0/1	0/1	1/1	0/1	0/1	0/1	0/1	0/1	0/1	0/1	0/1	0/1	0/1	0/0
Chromosome3-2184537	DEL	PRECISE	348	0	0	0/0	0/0	0/0	0/0	0/0	0/0	0/0	0/0	0/0	0/0	0/0	0/0	0/0	0/1
Chromosome3-2186420	DEL	PRECISE	99	0	0	0/1	0/1	1/1	0/1	0/1	0/1	0/1	0/1	0/1	0/1	0/1	0/1	0/1	0/1
Chromosome3-2193358	DEL	PRECISE	98	0	0	0/0	0/0	0/0	0/0	0/0	0/0	0/0	0/0	0/0	0/0	0/0	0/0	0/0	0/1
Chromosome3-2209291	DEL	PRECISE	98	0	0	0/0	0/0	0/0	0/0	0/0	0/0	0/0	0/0	0/0	0/0	0/0	0/0	0/0	0/1
Chromosome3-2228015	INS	PRECISE	102	0	0	0/0	0/0	0/0	0/0	0/0	0/0	0/1	0/0	0/0	0/0	0/0	0/0	0/0	0/0
Chromosome3-2231577	DEL	PRECISE	714	0.267	0.267	0/1	0/1	1/1	1/1	1/1	1/1	0/1	0/1	1/1	0/1	1/1	1/1	0/1	0/1
Chromosome3-2232486	INS	PRECISE	195	0	0.707	0/0	0/0	0/0	0/0	0/0	0/0	0/0	0/0	0/0	0/0	0/1	0/1	0/0	0/0
Chromosome3-2232634	DEL	PRECISE	422	0	0	0/1	0/1	1/1	0/1	0/1	0/1	0/1	0/1	0/1	0/1	0/1	0/1	0/1	0/1
Chromosome3-2233865	INS	PRECISE	205	0.535	0.611	0/1	0/1	1/1	0/1	0/1	0/1	0/1	0/1	0/1	0/1	1/1	1/1	0/1	0/1
Chromosome3-2235189	DEL	PRECISE	60	0	0	0/1	0/1	1/1	0/1	0/1	0/1	0/1	0/1	0/1	0/1	0/1	0/1	0/1	0/1
Chromosome3-2238515	DEL	PRECISE	314	0	0	0/0	0/0	0/0	0/0	0/0	0/0	0/1	0/0	0/0	0/0	0/0	0/0	0/0	0/0
Chromosome3-2239801	DEL	PRECISE	75	0	0	0/0	0/1	0/1	0/1	0/1	0/1	0/0	0/1	0/1	0/1	0/1	0/1	0/1	0/0
Chromosome3-2242586	DEL	PRECISE	78	0	0	0/0	0/0	0/0	0/0	0/0	0/0	0/0	0/0	0/0	0/0	0/1	0/0	0/0	0/1
Chromosome3-2253150	INS	PRECISE	5073	0	0	0/0	0/0	0/0	0/0	0/0	0/0	0/0	0/0	0/0	0/0	0/0	0/0	0/0	1/1
Chromosome3-2262889	INS	PRECISE	2365	0	0	0/0	0/0	0/0	0/0	0/0	0/0	0/0	0/0	0/0	0/0	0/0	0/0	0/0	0/1
Chromosome3-2265587	DEL	PRECISE	219	0	0	0/1	0/1	1/1	0/1	0/1	0/1	0/1	0/1	0/1	0/1	0/1	0/1	0/0	0/1
Chromosome3-2265867	INS	PRECISE	123	0	0	0/0	0/0	0/0	0/0	0/0	0/0	0/0	0/0	0/0	0/0	0/1	0/0	0/0	0/0
Chromosome3-2268766	INS	PRECISE	1162	0	0	0/0	0/0	0/0	0/0	0/0	0/0	0/0	0/0	0/0	0/0	0/0	0/0	0/0	0/1
Chromosome3-2275617	INS	PRECISE	114	0	0.426	0/1	0/1	1/1	0/1	0/1	0/1	0/1	0/1	0/1	0/1	0/1	0/1	0/1	0/1
Chromosome3-2282385	INS	PRECISE	1556	0	0	0/0	0/0	0/0	0/0	0/0	0/0	0/0	0/0	0/0	0/0	0/0	0/0	0/0	0/1
Chromosome3-2286075	INS	PRECISE	1308	0	0	0/0	0/0	0/0	0/0	0/0	0/0	0/0	0/0	0/0	0/0	0/0	0/0	0/0	0/1
Chromosome3-2287622	INS	PRECISE	343	0	0.707	0/1	0/0	0/1	0/0	0/0	0/0	0/0	0/0	0/0	0/0	0/0	0/0	0/0	0/0
Chromosome3-2290932	INS	PRECISE	150	0	0	0/0	0/0	0/0	0/0	0/0	0/0	0/0	0/0	0/0	0/0	0/0	0/0	0/0	0/1
Chromosome3-2299588	INS	PRECISE	151	0	0.802	0/1	0/1	1/1	0/1	0/1	0/1	0/1	1/1	0/1	0/1	0/1	0/1	0/1	0/1
Chromosome3-2302832	INS	PRECISE	132	0	0.548	0/0	0/1	0/1	0/0	0/0	0/0	0/0	0/0	0/1	0/0	0/1	0/0	0/1	0/0
Chromosome3-2304982	INS	PRECISE	121	0	0.408	0/0	0/0	0/0	0/1	0/1	0/1	0/0	1/1	0/0	0/1	0/0	0/1	0/0	0/0
Chromosome3-2306381	INS	PRECISE	1498	0	0	0/0	0/0	0/0	0/0	0/0	0/0	0/1	0/0	0/0	0/0	0/0	0/0	0/0	0/0
Chromosome3-2311294	INS	PRECISE	56	0	0	0/1	0/1	1/1	1/1	1/1	1/1	0/1	0/1	1/1	0/1	1/1	1/1	0/1	0/1
Chromosome3-2311370	DEL	PRECISE	61	0	0	0/1	0/1	1/1	0/1	0/1	0/1	0/1	0/1	0/1	0/1	0/1	0/1	0/1	0/1
Chromosome3-2320948	INS	PRECISE	545	0	0	0/0	0/0	0/0	0/0	0/0	0/0	0/1	0/0	0/0	0/0	0/0	0/0	0/0	0/0
Chromosome3-2326807	INS	PRECISE	890	0	0	0/0	0/0	0/0	0/0	0/0	0/0	0/0	0/0	0/0	0/0	0/0	0/0	0/0	1/1
Chromosome3-2328740	DEL	PRECISE	611	0	0	0/1	1/1	0/0	0/0	0/0	0/0	0/1	1/1	0/0	1/1	1/1	0/1	0/1	1/1
Chromosome3-2334824	DEL	PRECISE	1152	0	0	0/0	0/0	0/0	0/0	0/0	0/0	0/0	0/0	0/0	0/1	0/0	0/0	0/0	0/0
Chromosome3-2340474	INS	PRECISE	98	0	0.886	0/0	0/0	0/0	0/0	0/0	0/0	0/0	0/0	0/0	0/0	0/0	1/1	0/0	0/0

Chromosome3-2341880	INS	PRECISE	60	0	0	0/0	0/0	0/0	0/0	0/0	0/0	0/0	0/0	0/0	0/0	0/0	0/1	0/0	0/0
Chromosome3-2342845	INS	PRECISE	702	0	0	0/0	0/0	0/0	0/0	0/0	0/0	0/0	0/0	0/0	0/0	0/1	0/0	0/0	0/0
Chromosome3-2343541	DEL	PRECISE	3916	0	0	0/0	0/0	0/1	0/1	0/1	0/0	0/1	0/1	0/0	0/0	0/0	0/0	0/0	0/1
Chromosome3-2347478	INS	PRECISE	98	0	0.579	0/0	0/1	0/1	0/1	0/1	1/1	0/0	0/0	1/1	0/1	0/1	1/1	0/0	0/0
Chromosome3-2355565	INS	PRECISE	7557	0	0	0/0	0/0	0/0	0/0	0/0	0/0	0/0	0/0	0/0	1/1	0/0	0/0	0/0	0/0
Chromosome3-2358390	INS	PRECISE	64	0	0	0/0	0/0	0/0	0/0	0/0	0/0	0/0	0/0	0/0	0/0	0/0	0/1	0/0	0/0
Chromosome3-2376565	DEL	PRECISE	911	0	0	0/0	0/0	0/0	0/1	0/1	0/1	0/0	0/0	0/1	0/0	0/0	0/0	0/0	0/0
Chromosome3-2377758	INS	PRECISE	803	0	0	0/0	0/0	0/0	0/0	0/0	0/0	0/0	0/0	0/0	0/0	0/0	0/1	0/0	0/0
Chromosome3-2378542	DUP	PRECISE	36245197	0	0	0/0	0/0	0/0	0/0	0/0	0/0	0/0	0/0	0/0	0/0	0/1	0/0	0/0	0/0
Chromosome3-2381676	DEL	PRECISE	117	0	0	0/1	0/1	1/1	0/1	0/1	0/1	0/1	0/1	0/1	0/1	0/1	0/1	0/1	0/1
Chromosome3-2386006	DEL	PRECISE	1148	0	0	0/1	0/1	1/1	0/1	0/1	0/1	0/1	0/1	0/1	0/1	0/1	0/1	0/1	0/1
Chromosome3-2386032	DEL	PRECISE	63	0	0	0/0	0/0	0/0	0/0	0/0	0/0	0/0	0/0	0/0	0/0	0/0	0/1	0/0	0/0
Chromosome3-2405088	DEL	PRECISE	58	0	0	0/0	0/0	0/0	0/0	0/0	0/0	0/0	0/0	0/0	0/1	0/0	0/0	0/0	0/0
Chromosome3-2406582	DEL	PRECISE	69	0	0	0/0	0/0	0/0	0/0	0/0	0/0	0/0	0/0	0/0	0/0	0/0	0/0	0/0	0/1
Chromosome3-2410602	DEL	PRECISE	1231	0	0	0/1	0/1	1/1	0/1	0/1	0/1	0/0	0/1	0/0	0/1	0/1	1/1	0/1	0/0
Chromosome3-2412711	INS	PRECISE	845	0	0	0/0	0/0	0/0	0/0	0/0	0/0	0/0	0/0	0/0	0/0	0/0	0/0	0/0	0/1
Chromosome3-2416880	INS	PRECISE	312	0	0.937	0/0	0/1	1/1	0/1	0/0	0/1	0/0	0/1	0/0	0/1	1/1	0/1	0/0	0/1
Chromosome3-2417389	DEL	PRECISE	3350	0	0	0/1	0/0	0/0	0/0	0/0	0/0	0/0	0/0	0/0	0/0	0/0	0/0	0/0	0/0
Chromosome3-2418244	DEL	PRECISE	1128	0	0	0/0	0/0	0/0	0/0	0/0	0/0	0/0	0/0	0/0	0/0	0/0	0/0	0/0	0/1
Chromosome3-2420451	INS	PRECISE	6056	0	0	0/0	0/0	0/0	0/0	0/0	0/0	0/0	0/0	0/0	0/0	0/0	0/0	0/0	1/1
Chromosome3-2421743	BND	PRECISE	0	0	0	0/0	0/0	0/1	0/0	0/0	0/0	0/0	0/0	0/0	0/1	0/0	0/0	0/0	0/0
Chromosome3-2437589	INS	PRECISE	9331	0	0	0/0	0/0	0/0	0/0	0/0	0/0	0/0	0/0	0/0	0/0	0/0	0/0	0/0	0/1
Chromosome3-2451652	DEL	PRECISE	114	0	0	0/1	0/1	1/1	0/1	0/1	0/1	0/1	0/1	0/1	0/1	0/1	0/1	0/1	0/1
Chromosome3-2451959	INS	PRECISE	77	0	0.535	0/1	0/1	1/1	0/1	0/1	0/1	0/1	0/1	0/1	0/1	0/1	0/1	0/1	0/1
Chromosome3-2459339	DEL	PRECISE	525	0	0	0/0	0/0	0/0	0/0	0/0	0/0	0/0	0/0	0/0	0/0	0/0	1/1	0/0	0/0
Chromosome3-2467376	DEL	PRECISE	363	0	0	0/1	0/1	1/1	1/1	1/1	1/1	0/1	0/1	1/1	1/1	1/1	1/1	0/1	0/1
Chromosome3-2478990	INS	PRECISE	51	0	0.5	0/0	0/0	0/0	0/1	1/1	0/1	0/0	0/0	1/1	0/0	0/0	0/0	0/0	0/0
Chromosome3-2482647	DEL	PRECISE	226	0	0	0/1	0/1	1/1	1/1	1/1	1/1	0/1	0/1	1/1	1/1	1/1	0/1	0/1	0/1
Chromosome3-2488766	INS	PRECISE	59	0	0.5	0/0	0/0	0/0	1/1	1/1	0/1	0/0	0/0	0/1	0/0	0/0	0/0	0/0	0/0
Chromosome3-2492504	INS	PRECISE	1318	0	0	0/0	0/0	0/0	0/0	0/0	0/0	0/0	0/0	0/0	1/1	0/0	0/0	0/0	0/0
Chromosome3-2515333	INS	PRECISE	5455	0	0	0/0	0/0	0/0	0/0	0/0	0/0	0/0	0/0	0/0	0/0	1/1	0/0	0/0	0/0
Chromosome3-2519627	INS	PRECISE	209	0	0.267	0/1	0/1	1/1	0/1	0/1	0/1	0/1	0/1	0/1	0/1	0/1	0/1	0/1	0/1
Chromosome3-2537573	INS	PRECISE	5690	0	0	0/0	0/0	0/0	0/0	0/0	0/0	1/1	0/0	0/0	0/0	0/0	0/0	0/0	0/0
Chromosome3-2537578	BND	PRECISE	0	0	0	0/0	0/0	0/0	0/0	0/0	0/0	0/1	0/0	0/0	0/0	0/0	0/0	0/0	0/0
Chromosome3-2540353	INS	PRECISE	5380	0	0	0/0	0/0	0/0	0/0	0/0	0/0	0/0	0/0	0/0	0/0	0/0	0/0	0/0	1/1
Chromosome3-2541889	INS	PRECISE	886	0	0.707	0/0	0/0	0/1	0/0	0/1	0/0	0/0	0/1	0/1	0/1	0/1	0/1	0/1	0/0
Chromosome3-2547340	DEL	PRECISE	77	0	0	0/1	0/0	0/1	0/0	0/0	0/0	0/1	0/0	0/0	0/0	0/0	0/0	0/0	0/0
Chromosome3-2557626	INS	PRECISE	1184	0	0	0/0	0/0	0/0	0/0	0/0	0/0	0/0	0/0	0/0	0/0	0/0	0/0	0/0	0/1
Chromosome3-2565742	INS	PRECISE	329	0	0	0/0	0/0	0/0	0/0	0/0	0/0	0/0	0/0	0/0	0/0	0/0	0/1	0/0	0/0
Chromosome3-2567370	DUP	PRECISE	723	0.632	0	0/0	0/0	0/0	0/0	0/0	0/0	0/0	0/0	0/0	0/1	0/0	0/1	0/0	0/0
Chromosome3-2567413	INS	PRECISE	5513	0	0	0/0	0/0	0/0	0/0	0/0	0/0	0/0	0/0	0/0	0/0	0/0	0/0	0/0	1/1
Chromosome3-2571955	DEL	PRECISE	9434	0	0	0/1	0/0	0/1	0/1	0/0	0/0	0/0	0/0	0/0	0/0	0/0	0/0	0/0	0/0
Chromosome3-2583420	DEL	PRECISE	704	0	0	0/1	0/1	1/1	1/1	1/1	1/1	0/1	0/1	1/1	1/1	0/1	0/1	0/1	0/1
Chromosome3-2606340	INS	PRECISE	211	0	0.447	0/0	0/0	0/0	0/1	0/1	0/1	0/0	0/0	0/1	1/1	0/0	0/0	0/0	0/0
Chromosome3-2607030	INS	PRECISE	5734	0	0	0/0	0/0	0/0	1/1	0/0	0/0	0/0	0/0	0/0	0/0	0/0	0/0	0/0	0/0
Chromosome3-2609483	DEL	PRECISE	122	0	0	0/0	0/0	0/0	0/1	0/0	0/0	0/0	0/0	0/0	0/0	0/0	0/0	0/0	0/0
Chromosome3-2617470	INS	PRECISE	2227	0	0	0/0	0/0	0/0	0/0	0/0	0/0	0/0	0/0	0/0	0/0	0/0	0/0	0/0	0/1
Chromosome3-2618983	DEL	PRECISE	337	0	0	0/1	0/1	1/1	0/1	0/1	0/1	0/1	0/1	0/1	0/1	1/1	0/1	0/1	0/1
Chromosome3-2620224	DEL	PRECISE	87	0	0	0/1	0/1	1/1	0/1	0/1	0/1	0/1	0/1	0/1	0/1	1/1	0/1	0/1	0/1
Chromosome3-2627193	INS	PRECISE	134	0	0.452	0/0	0/1	0/1	0/1	0/1	0/0	0/1	0/1	0/1	0/1	0/1	0/1	0/1	0/0
Chromosome3-2641110	DEL	PRECISE	89	0	0	0/0	0/1	0/1	0/0	0/0	0/1	0/0	0/1	0/1	0/1	0/1	0/1	0/1	0/0
Chromosome3-2643520	INS	PRECISE	1227	0	0.707	0/1	0/0	0/1	0/0	0/0	0/0	0/0	0/0	0/0	0/0	0/0	0/0	0/0	0/0
Chromosome3-2648852	INS	PRECISE	2881	0	0	0/0	0/0	0/0	0/0	0/0	0/0	0/0	0/0	0/0	0/0	0/0	0/0	0/0	0/1
Chromosome3-2649564	DEL	PRECISE	830	0	0	0/1	0/1	1/1	0/0	0/0	0/1	0/1	0/1	0/1	0/1	0/1	0/1	0/1	0/0
Chromosome3-2659614	DEL	PRECISE	55	0	0	0/0	0/0	0/0	1/1	1/1	0/1	0/0	0/0	0/1	0/0	0/0	0/0	0/0	1/1
Chromosome3-2663657	INS	PRECISE	633	0	0	0/0	0/0	0/0	0/0	0/0	0/0	0/0	0/0	0/0	0/0	0/0	0/0	0/0	0/1
Chromosome3-2669458	INS	PRECISE	2101	0	0	0/0	0/0	0/0	0/0	0/0	0/0	0/0	0/0	0/0	0/0	0/0	0/0	0/0	0/1
Chromosome3-2670625	INS	PRECISE	55	0	0	0/0	0/0	0/0	0/1	0/1	0/1	0/0	0/0	0/1	0/1	0/1	0/1	0/0	0/0
Chromosome3-2674641	DEL	PRECISE	69	0	0	0/0	0/0	0/0	0/0	0/0	0/0	0/0	0/0	0/0	0/0	0/1	0/1	0/0	0/0
Chromosome3-2690216	INS	PRECISE	90	0	0	0/0	0/0	0/0	0/0	0/0	0/0	0/0	0/0	0/0	0/0	0/0	0/0	0/0	0/1
Chromosome3-2709168	INS	PRECISE	1017	0	0.707	0/0	0/1	0/1	0/0	0/0	0/0	0/0	0/0	0/1	0/0	0/1	0/0	0/1	0/0
Chromosome3-2720901	INS	PRECISE	2109	0	0	0/0	0/0	0/0	0/0	0/0	0/0	0/0	0/0	0/0	0/0	0/0	0/0	0/0	0/1
Chromosome3-2725890	INS	PRECISE	137	0	0	0/0	0/0	0/0	0/0	0/0	0/1	0/0	0/1	0/0	0/1	0/0	0/1	0/0	0/0
Chromosome3-2726635	INS	PRECISE	1223	0	0	0/0	0/0	0/0	0/0	0/0	0/0	0/0	0/0	0/0	0/0	0/0	0/0	0/0	0/1
Chromosome3-2727851	DEL	PRECISE	52	0.555	0.555	0/1	0/1	1/1	0/1	0/1	0/1	0/1	0/1	0/1	0/1	0/1	0/1	0/1	0/0
Chromosome3-2728096	DEL	PRECISE	1362	0	0	0/1	0/1	1/1	0/1	0/1	0/1	0/1	0/1	0/1	0/1	0/1	0/1	0/1	0/1
Chromosome3-2737390	DEL	PRECISE	52	0	0	0/1	0/1	1/1	0/1	0/0	0/1	0/1	0/1	0/1	0/1	0/1	0/0	0/1	0/1
Chromosome3-2739030	INS	PRECISE	11090	0	0	0/0	0/0	0/0	0/0	0/0	0/0	0/0	0/0	0/0	1/1	0/0	0/0	0/0	0/0
Chromosome3-2739045	BND	PRECISE	0	0	0	0/0	0/0	0/0	0/0	0/0	0/0	0/0	0/0	0/0	0/1	0/0	0/0	0/0	0/0
Chromosome3-2739125	INS	PRECISE	888	0	0.447	0/0	0/1	0/1	0/0	0/0	0/0	0/0	0/0	0/1	0/0	0/1	0/0	0/1	0/0
Chromosome3-2748791	DEL	PRECISE	50	0	0	0/0	0/0	0/0	0/0	0/0	0/0	0/0	0/0	0/0	0/0	0/0	0/0	0/0	0/1
Chromosome3-2752451	INS	PRECISE	1883	0	0	0/0	0/0	0/0	0/0	0/0	0/0	0/0	0/0	0/0	0/0	0/0	0/0	0/0	0/1
Chromosome3-2759271	INS	PRECISE	148	0	0	0/0	0/0	0/0	0/0	0/0	0/0	0/0	0/0	0/0	0/1	0/0	0/0	0/0	0/0

Chromosome3-2760297	DEL	PRECISE	358	0	0	0/1	0/1	1/1	0/1	0/1	0/1	0/1	0/1	0/1	0/1	0/1	0/1	0/1	0/1
Chromosome3-2762492	INS	PRECISE	705	0	0	0/0	0/0	0/0	0/1	0/0	0/0	0/0	0/0	0/0	0/0	0/0	0/0	0/0	0/0
Chromosome3-2762868	DEL	PRECISE	561	0	0	0/0	0/0	0/0	0/0	0/0	0/0	0/0	0/0	0/0	0/0	0/1	0/1	0/0	0/0
Chromosome3-2775092	INS	PRECISE	7283	0	0	0/0	0/0	0/0	0/0	0/0	0/0	0/0	0/0	0/0	0/0	0/0	0/0	0/0	1/1
Chromosome3-2778651	INS	PRECISE	887	0	0	0/0	0/0	0/0	0/0	0/0	0/0	0/0	0/0	0/0	0/0	0/0	0/0	0/0	0/1
Chromosome3-2789270	DEL	PRECISE	72	0	0	0/1	0/1	1/1	1/1	0/1	0/1	0/1	0/1	0/1	0/1	0/1	0/1	0/1	0/0
Chromosome3-2793914	INS	PRECISE	910	0	0	0/0	0/0	0/0	0/0	0/0	0/0	0/0	0/0	0/0	0/1	0/0	0/0	0/0	0/0
Chromosome3-2795252	INS	PRECISE	1157	0	0	0/0	0/0	0/0	0/0	0/0	0/0	0/1	0/0	0/0	0/0	0/0	0/0	0/0	0/0
Chromosome3-2812702	DEL	PRECISE	349	0.535	0.535	0/1	0/1	1/1	0/1	0/1	0/1	0/1	0/1	0/0	1/1	0/1	0/1	0/1	0/1
Chromosome3-2815167	DEL	PRECISE	210	0	0	0/1	0/1	1/1	0/1	0/1	0/1	0/1	0/1	0/0	1/1	0/1	0/1	0/1	0/1
Chromosome3-2818867	INS	PRECISE	365	0	0	0/0	0/0	0/0	0/0	0/0	0/0	0/0	0/0	0/0	0/0	0/0	0/1	0/0	0/0
Chromosome3-2838196	DEL	PRECISE	91	0	0	0/1	0/1	1/1	0/1	0/1	0/1	0/0	0/1	0/1	0/1	0/1	0/1	0/1	0/1
Chromosome3-2838881	DEL	PRECISE	623	0.363	0	0/1	0/1	1/1	1/1	1/1	1/1	0/1	0/1	1/1	1/1	1/1	1/1	0/1	0/1
Chromosome3-2846892	DEL	PRECISE	59	0	0	0/1	0/0	0/1	0/1	0/0	0/0	0/0	0/0	0/0	0/0	0/0	0/0	0/0	0/0
Chromosome3-2850257	INS	PRECISE	60	0	0	0/1	0/0	0/1	0/1	0/1	0/1	0/1	0/1	0/0	0/1	0/0	0/1	0/0	0/0
Chromosome3-2851160	INS	PRECISE	325	0.894	0.548	0/0	0/0	0/0	0/1	0/1	0/1	0/0	0/0	0/1	0/0	0/0	0/1	0/0	0/0
Chromosome3-2851200	DEL	PRECISE	732	0	0	0/0	0/0	0/0	0/0	0/0	0/0	0/0	0/0	0/0	0/1	0/0	0/0	0/0	0/0
Chromosome3-2853010	INS	PRECISE	81	0.447	0.894	0/0	0/0	0/0	1/1	0/1	0/1	0/0	0/0	1/1	0/0	0/0	0/1	0/0	0/0
Chromosome3-2853862	DEL	PRECISE	231	0	0	0/0	0/0	0/0	0/1	0/1	0/1	0/1	0/1	0/0	0/1	0/0	0/1	0/0	0/0
Chromosome3-2873540	DEL	PRECISE	4937	0.535	0	0/1	0/1	1/1	1/1	1/1	1/1	0/1	0/1	1/1	1/1	1/1	1/1	0/1	0/1
Chromosome3-2900158	DEL	PRECISE	100	0	0	0/0	0/0	0/0	0/0	0/0	0/0	0/0	0/0	0/0	1/1	0/0	0/0	0/0	0/0
Chromosome3-2910048	DEL	PRECISE	108	0	0	0/0	0/0	0/0	0/0	0/0	0/0	0/0	0/0	0/0	0/0	0/0	0/0	0/0	0/1
Chromosome3-2917231	DEL	PRECISE	1615	0	0	0/0	0/0	0/0	0/0	0/0	0/0	0/0	0/0	0/0	0/0	0/0	0/0	0/0	0/1
Chromosome3-2918318	INS	PRECISE	59	0	0.707	0/0	0/0	0/0	0/0	0/0	0/0	0/0	0/0	0/0	0/0	0/1	0/0	0/1	0/0
Chromosome3-2919130	INS	PRECISE	886	0	0	0/0	0/0	0/0	0/0	0/0	0/0	0/0	0/0	0/0	0/0	0/0	0/0	0/0	0/1
Chromosome3-2919567	INS	PRECISE	5397	0	0	0/0	0/0	0/0	0/0	0/0	0/0	0/0	0/0	0/0	0/0	0/0	0/0	0/0	1/1
Chromosome3-2925187	INS	PRECISE	1533	0	0	0/0	0/0	0/0	0/0	0/0	0/0	0/0	0/0	0/0	0/1	0/0	0/0	0/0	0/0
Chromosome3-2925378	INS	PRECISE	212	0	0	0/0	0/0	0/0	0/1	0/0	0/0	0/0	0/0	0/0	0/0	0/0	0/0	0/0	0/0
Chromosome3-2925568	DEL	PRECISE	147	0	0	0/0	0/0	0/0	0/0	0/0	0/0	0/1	0/0	0/0	0/0	0/0	0/0	0/0	0/0
Chromosome3-2932486	DEL	PRECISE	157	0	0	0/1	0/1	1/1	0/1	0/1	1/1	0/1	0/1	0/1	1/1	0/1	1/1	0/1	0/1
Chromosome3-2945916	DEL	PRECISE	199	0	0	0/1	0/1	1/1	0/0	0/1	0/1	0/1	0/1	0/1	0/1	0/1	1/1	0/0	0/1
Chromosome3-2950774	DEL	PRECISE	147	0	0	0/0	0/1	0/1	0/0	0/0	0/0	0/0	0/0	0/1	0/0	0/1	0/0	0/0	0/0
Chromosome3-2952384	DEL	PRECISE	64	0	0	0/0	0/0	0/0	0/1	1/1	0/0	0/0	1/1	0/0	1/1	0/0	0/0	0/0	0/0
Chromosome3-2953699	DEL	PRECISE	329	0.267	0.267	0/1	0/1	1/1	0/1	0/1	0/1	0/1	0/1	0/1	1/1	1/1	1/1	1/1	0/1
Chromosome3-2955341	DEL	PRECISE	76	0	0	0/0	0/0	0/0	0/0	0/0	0/0	0/0	0/0	0/0	0/1	0/0	0/0	0/0	0/0
Chromosome3-2956017	INS	PRECISE	1792	0	0.707	0/1	0/0	0/1	0/0	0/0	0/0	0/0	0/0	0/0	0/0	0/0	0/0	0/0	0/0
Chromosome3-2963687	INS	PRECISE	72	0	0	0/0	0/0	0/0	0/0	0/0	0/0	0/0	0/0	0/0	0/0	0/0	0/0	0/0	0/1
Chromosome3-2964251	INS	PRECISE	625	0	0	0/0	0/0	0/0	0/0	0/0	0/0	0/0	0/0	0/0	0/0	0/0	0/0	0/0	0/1
Chromosome3-2973253	DEL	PRECISE	71	0	0	0/1	0/1	1/1	1/1	1/1	1/1	0/1	0/1	1/1	1/1	0/1	0/1	0/1	0/1
Chromosome3-2973321	INS	PRECISE	61	0.577	0	0/0	0/0	0/0	0/0	0/0	0/0	0/0	0/0	0/0	0/0	0/1	0/1	0/1	0/0
Chromosome3-2973794	INS	PRECISE	828	0	0	0/1	0/0	0/0	0/0	0/0	0/0	0/0	0/0	0/0	0/0	0/0	0/0	0/0	0/0
Chromosome3-2977080	INS	PRECISE	1067	0	0	0/0	0/0	0/0	1/1	0/0	0/0	0/0	0/0	0/0	0/0	0/0	0/0	0/0	0/0
Chromosome3-2979608	INS	PRECISE	196	0	0	0/0	0/0	0/0	0/0	0/0	0/0	0/0	0/0	0/0	1/1	0/0	0/0	0/0	0/0
Chromosome3-2979857	DEL	PRECISE	1381	0	0	0/0	0/0	0/0	0/0	0/0	0/0	0/0	0/0	0/0	1/1	0/0	0/0	0/0	0/0
Chromosome3-2985998	DEL	PRECISE	92	0	0	0/1	0/0	0/1	0/0	0/0	0/0	0/0	0/0	0/0	0/0	0/0	0/0	0/0	0/0
Chromosome3-2988393	DEL	PRECISE	152	0	0	0/0	0/0	0/0	0/0	0/0	0/0	0/0	0/0	0/0	0/0	1/1	1/1	1/1	0/0
Chromosome3-2991940	DEL	PRECISE	64	0	0	0/1	0/1	1/1	0/0	1/1	0/1	0/1	0/1	0/0	0/1	0/1	0/1	0/1	0/1
Chromosome3-2992893	DEL	PRECISE	148	0	0	0/1	0/1	1/1	0/0	0/1	0/1	0/1	0/1	0/0	0/1	0/1	0/1	0/1	0/0
Chromosome3-2992898	INS	PRECISE	7651	0	0	0/0	0/0	0/0	0/0	0/0	0/0	0/0	0/0	0/0	0/0	0/0	0/0	0/0	1/1
Chromosome3-2993088	INS	PRECISE	636	0	0	0/0	0/0	0/0	1/1	0/0	0/0	0/0	0/0	0/0	0/0	0/0	0/0	0/0	0/0
Chromosome3-2994289	INS	PRECISE	83	0	0	0/1	0/1	1/1	0/1	0/1	0/1	0/1	0/1	0/1	0/1	0/1	0/1	0/1	0/0
Chromosome3-3002166	INS	PRECISE	270	0	0.5	0/0	0/0	0/0	0/1	0/1	0/1	0/0	0/0	0/1	0/0	0/0	0/0	0/0	0/0
Chromosome3-3012297	DEL	PRECISE	190	0	0	0/1	0/1	1/1	0/1	0/1	0/1	0/1	0/1	0/1	0/1	0/1	0/1	1/1	0/1
Chromosome3-3017376	DEL	PRECISE	779	0	0.535	0/1	0/1	1/1	0/1	0/1	0/1	0/1	0/1	0/1	0/1	0/1	0/1	1/1	0/1
Chromosome3-3017597	INS	PRECISE	62	0	0	0/0	0/0	0/0	0/0	0/0	0/0	0/0	0/0	0/0	0/1	0/0	0/0	0/0	0/0
Chromosome3-3019491	DEL	PRECISE	1470	0	0	0/1	0/1	1/1	1/1	1/1	1/1	0/1	0/1	1/1	1/1	1/1	1/1	1/1	0/1
Chromosome3-3028736	INS	PRECISE	13036	0	0	0/0	0/0	0/0	0/0	0/0	0/0	0/0	0/0	0/0	0/1	0/0	0/0	0/0	0/0
Chromosome3-3029659	DEL	PRECISE	3921	0	0	0/0	0/0	0/0	0/1	0/1	0/1	0/0	0/0	0/1	0/0	0/0	0/0	0/0	0/0
Chromosome3-3031468	DEL	PRECISE	1363	0	0	0/0	0/0	0/0	0/0	0/0	0/0	0/0	0/0	0/0	0/0	0/1	0/0	0/0	0/0
Chromosome3-3047285	DEL	PRECISE	612	0	0	0/0	0/0	0/0	0/0	0/0	0/0	0/0	0/0	0/0	0/0	0/0	0/0	0/0	0/1
Chromosome3-3050637	INS	PRECISE	424	0	0	0/0	0/0	0/0	0/1	0/0	0/0	0/0	0/0	0/0	0/0	0/0	0/0	0/0	0/0
Chromosome3-3054957	INS	PRECISE	364	0	0	0/0	0/0	0/0	0/0	0/0	0/0	0/0	0/0	0/0	0/1	0/0	0/0	0/0	0/0
Chromosome3-3055737	BND	PRECISE	0	0	0	0/0	0/0	0/0	0/1	0/1	0/1	0/0	0/0	0/1	0/0	0/0	0/0	0/0	0/0
Chromosome3-3055737	BND	PRECISE	0	0	0	0/0	0/0	0/0	0/1	0/1	0/0	0/0	0/0	0/1	0/0	0/0	0/0	0/0	0/0
Chromosome3-3059877	DEL	PRECISE	173	0	0	0/0	0/0	0/0	0/0	0/0	0/0	0/0	0/0	0/0	0/0	0/0	0/0	0/0	0/1
Chromosome3-3061177	DEL	PRECISE	253	0	0	0/1	0/1	1/1	0/1	0/1	0/1	0/1	0/1	0/1	0/1	0/1	0/1	1/1	0/1
Chromosome3-3063133	INS	PRECISE	821	0	0.837	0/0	0/1	0/1	0/0	0/0	0/0	0/0	0/0	0/1	0/0	0/1	0/0	0/1	0/0
Chromosome3-3063133	BND	PRECISE	0	0	0	0/0	0/0	0/0	0/0	0/1	0/1	0/0	0/1	0/0	0/0	0/0	0/0	0/0	0/0
Chromosome3-3063972	DEL	PRECISE	52	0.832	0.277	0/1	0/1	1/1	0/1	0/1	0/1	0/1	0/1	0/1	0/1	0/1	0/1	0/1	0/0
Chromosome3-3068153	INS	PRECISE	131	0.802	0.475	0/1	0/1	1/1	0/1	0/0	0/1	0/1	0/0	0/1	0/1	0/1	0/1	0/1	0/1
Chromosome3-3068337	DEL	PRECISE	72	0	0	0/0	0/0	0/0	0/0	0/0	0/0	0/0	0/0	0/0	0/0	0/0	0/0	0/0	0/1
Chromosome3-3071176	DEL	PRECISE	500	0	0	0/0	0/0	0/0	0/0	0/0	0/0	0/0	0/0	0/0	0/0	0/0	0/1	0/1	0/0
Chromosome3-3074432	INS	PRECISE	3814	0	0	0/0	0/0	0/0	0/0	0/0	0/0	0/0	0/0	0/0	0/0	0/0	0/0	0/0	1/1

Chromosome3-3075398	DEL	PRECISE	63	0	0	0/0	0/0	0/0	0/1	0/0	0/0	0/1	0/0	0/0	0/0	0/0	0/0	0/0	0/0		
Chromosome3-3081143	INS	PRECISE	245	0	0	0/0	0/0	0/0	0/0	0/0	0/0	0/0	0/0	0/0	0/0	0/0	0/1	0/1	0/0		
Chromosome3-3081559	DEL	PRECISE	815	0	0	0/1	1/1	1/1	0/0	0/0	0/1	1/1	0/1	0/1	0/1	0/1	0/1	0/1	0/1		
Chromosome3-3081905	DEL	PRECISE	65	0.707	0.707	0/0	0/0	0/0	0/0	0/0	0/0	0/0	0/0	0/0	0/0	0/0	0/1	0/1	0/0		
Chromosome3-3093510	INS	PRECISE	238	0	0	0/0	0/0	0/0	0/0	0/0	0/0	0/0	0/0	0/0	0/0	0/0	0/1	0/1	0/0		
Chromosome3-3114708	INS	PRECISE	126	0	0.267	0/1	0/1	1/1	0/1	0/1	0/1	0/1	0/1	0/1	0/1	0/1	0/1	0/1	0/1		
Chromosome3-3131132	INS	PRECISE	211	0	0	0/0	0/0	0/0	0/0	0/1	1/1	0/1	1/1	0/0	1/1	0/0	1/1	0/0	0/0		
Chromosome3-3139821	INS	PRECISE	8611	0	0	0/0	0/0	0/0	0/0	0/0	0/0	0/0	0/0	0/0	0/0	0/0	0/1	0/0	0/0		
Chromosome3-3142636	DEL	PRECISE	84	0	0	0/1	0/0	0/1	1/1	1/1	0/1	0/1	0/1	0/0	1/1	0/0	1/1	0/0	0/0		
Chromosome3-3143956	INS	PRECISE	454	0	0	0/1	0/0	0/1	0/0	0/0	0/0	0/0	0/0	0/0	0/0	0/0	0/0	0/0	0/0		
Chromosome3-3146202	INS	PRECISE	68	0	0	0/1	0/0	0/1	1/1	0/1	0/1	0/1	0/1	0/0	1/1	0/0	1/1	0/0	0/1		
Chromosome3-3148910	INS	PRECISE	938	0	0	0/0	0/0	0/0	0/0	0/0	0/0	0/0	0/0	0/0	0/0	0/0	0/0	0/0	0/1		
Chromosome3-3149470	INS	PRECISE	138	0	0	0/0	0/0	0/0	0/0	0/0	0/0	0/0	0/0	0/0	0/0	0/0	0/0	0/0	0/1		
Chromosome3-3150531	INS	PRECISE	507	0	0.463	0/0	0/1	0/1	0/1	0/1	0/0	0/0	0/0	1/1	0/0	1/1	0/0	0/1	0/0		
Chromosome3-3156514	INS	PRECISE	876	0	0	0/0	0/0	0/0	0/0	0/0	0/0	0/0	0/0	0/0	0/0	0/0	0/0	0/0	0/1		
Chromosome3-3165651	DEL	PRECISE	53	0	0	0/1	0/0	0/1	0/1	0/1	0/1	0/1	0/1	0/0	0/1	0/0	0/1	0/0	0/1		
Chromosome3-3177394	INS	PRECISE	141	0	0	0/0	0/0	0/0	0/0	0/0	0/0	0/0	0/0	0/0	0/0	0/0	0/0	0/0	0/1		
Chromosome3-3182968	DEL	PRECISE	631	0	0	0/0	0/1	1/1	0/1	0/1	1/1	0/0	0/0	1/1	0/0	0/1	0/0	0/1	0/0		
Chromosome3-3189051	INS	PRECISE	53	0	0.632	0/1	0/0	0/1	0/1	0/1	0/1	0/1	0/1	0/0	0/1	0/0	0/1	0/0	0/1		
Chromosome3-3198917	INS	PRECISE	260	0	0	0/0	0/0	0/0	0/0	0/0	0/0	0/0	0/0	0/0	0/1	0/0	0/0	0/0	0/0		
Chromosome3-3200125	INS	PRECISE	150	0	0	0/0	0/0	0/0	0/0	0/0	0/0	0/0	0/0	0/0	0/0	0/1	0/0	0/0	0/0		
Chromosome3-3201107	DEL	PRECISE	89	0	0	0/0	0/0	0/0	0/0	0/0	0/0	0/0	0/0	0/0	0/1	0/0	0/0	0/0	0/0		
Chromosome3-3215915	DEL	PRECISE	248	0	0	0/0	0/1	0/1	1/1	0/1	0/1	0/0	0/0	1/1	0/1	1/1	0/0	1/1	0/0		
Chromosome3-3223978	INS	PRECISE	283	0	0.518	0/0	0/1	0/1	0/1	0/1	0/1	0/0	0/0	1/1	0/0	0/1	0/0	0/1	0/0		
Chromosome3-3231618	INS	PRECISE	536	0	0.518	0/0	0/1	0/1	0/1	0/1	0/1	0/0	0/0	1/1	0/0	0/1	0/0	0/1	0/0		
Chromosome3-3250173	DEL	PRECISE	81	0	0	0/1	0/0	0/1	0/1	0/1	0/1	0/1	0/1	0/0	0/1	0/0	0/1	0/0	0/1		
Chromosome3-3253414	INS	PRECISE	150	0	0.316	0/1	0/0	0/1	0/1	0/1	0/1	0/1	0/1	0/0	0/1	0/0	0/1	0/0	0/1		
Chromosome3-3267970	DEL	PRECISE	119	0	0	0/0	0/1	0/1	0/1	0/1	0/1	0/0	0/0	1/1	0/0	0/1	0/1	1/1	0/0		
Chromosome3-3276377	DEL	PRECISE	52	0	0	0/1	0/0	0/1	0/1	0/1	0/1	0/1	0/1	0/0	0/1	0/0	0/1	0/0	0/1		
Chromosome3-3286344	DEL	PRECISE	104	0	0	0/1	0/0	0/1	0/1	0/1	0/1	0/1	0/1	0/0	0/1	0/0	0/1	0/0	0/1		
Chromosome3-3291933	DEL	PRECISE	150	0	0	0/1	0/1	1/1	1/1	1/1	1/1	0/1	0/1	1/1	0/1	0/1	1/1	1/1	0/1		
Chromosome3-3351348	BND	PRECISE	0	0	0	0/0	0/0	0/0	0/0	0/1	0/1	0/0	0/1	0/0	0/1	0/0	0/1	0/0	0/0		
Chromosome3-3351348	BND	PRECISE	0	0	0	0/0	0/0	0/0	0/0	0/0	0/0	0/0	0/0	0/0	0/0	0/0	0/1	0/0	0/0		
Chromosome3-3351960	DEL	PRECISE	828	0	0	0/0	0/0	0/0	0/0	0/0	0/0	0/0	0/0	0/0	1/1	0/0	0/0	0/0	0/0		
Chromosome3-3353234	INS	PRECISE	139	0	0	0/0	0/0	0/0	0/0	0/0	0/0	0/0	0/0	0/0	1/1	0/1	0/0	0/0	0/0		
Chromosome3-3373680	INS	PRECISE	265	0	0.707	0/1	0/0	0/1	0/1	0/1	1/1	0/1	0/1	0/0	1/1	0/0	0/1	0/0	0/1		
Chromosome3-3381465	DEL	PRECISE	775	0	0	0/1	0/0	0/0	0/1	0/1	0/1	0/1	0/1	0/0	0/1	0/0	0/1	0/0	0/1		
Chromosome3-3381714	INS	PRECISE	58	0	0.447	0/0	0/0	0/0	0/0	0/0	0/0	0/0	0/0	0/0	0/1	0/1	0/1	0/1	0/0		
Chromosome3-3386414	INS	PRECISE	92	0	0.667	0/0	0/1	0/1	0/1	0/1	0/1	0/0	0/0	1/1	0/1	0/1	0/0	0/1	0/0		
Chromosome3-3389031	INS	PRECISE	123	0	0	0/0	0/0	0/0	0/0	0/0	0/0	0/0	0/0	0/0	0/0	0/0	0/0	0/0	0/1		
Chromosome3-3396871	INS	PRECISE	822	0	0	0/0	0/0	0/0	0/0	0/0	0/0	0/0	0/0	0/0	0/0	0/1	0/0	0/0	0/0		
Chromosome3-3401837	INS	PRECISE	466	0	0	0/0	0/0	0/0	0/0	0/0	0/0	0/0	0/0	0/0	0/0	0/0	0/0	0/0	0/1		
Chromosome3-3419172	DEL	PRECISE	923	0	0	0/0	0/0	0/0	0/0	0/0	0/0	0/0	0/0	0/0	0/0	0/1	0/0	0/0	0/0		
Chromosome3-3423096	INS	PRECISE	1009	0	0.577	0/1	0/0	0/1	0/0	0/0	0/0	0/1	0/0	0/0	0/0	0/0	0/0	0/0	0/0		
Chromosome3-3426064	DEL	PRECISE	191	0	0	0/0	0/0	0/0	0/1	0/0	0/0	0/0	0/0	0/0	0/0	0/0	0/0	0/0	0/0		
Chromosome3-3427049	INS	PRECISE	198	0	0	0/1	0/0	0/1	0/1	0/1	0/1	0/1	0/1	0/0	0/1	0/0	0/1	0/0	0/1		
Chromosome3-3427864	DEL	PRECISE	78	0	0	0/1	0/0	0/1	0/1	0/1	0/1	0/1	0/1	0/0	0/1	0/0	0/1	0/0	0/1		
Chromosome3-3437968	DEL	PRECISE	227	0	0	0/0	0/1	0/1	0/1	0/1	0/1	0/0	0/0	1/1	0/1	1/1	0/0	0/1	0/0		
Chromosome3-3442404	INS	PRECISE	231	0	0	0/0	0/0	0/0	0/0	0/0	0/0	0/0	0/0	0/0	0/0	0/1	0/0	0/0	0/0		
Chromosome3-3445325	INS	PRECISE	93	0	0.289	0/1	0/0	1/1	0/1	0/1	0/1	0/1	0/1	0/0	1/1	0/1	1/1	1/1	0/1		
Chromosome3-3447824	DEL	PRECISE	84	0	0	0/0	0/0	0/0	0/0	0/0	0/0	0/0	0/0	0/0	0/0	0/1	0/0	0/0	0/0		
Chromosome3-3449507	INS	PRECISE	211	0	0	0/1	0/0	0/1	0/0	0/0	0/0	0/1	0/0	0/0	0/0	0/0	0/0	0/0	0/0		
Chromosome3-3451702	INS	PRECISE	195	0	0.707	0/0	0/0	0/0	0/0	0/0	0/0	0/0	0/0	0/0	0/0	0/0	0/1	1/1	0/0		
Chromosome3-3458752	INS	PRECISE	55	0	0	0/1	0/0	0/1	0/1	0/1	0/1	0/1	0/1	0/0	1/1	0/1	0/1	0/0	0/1		
Chromosome3-3462316	BND	PRECISE	0	0	0	0/1	0/0	0/0	0/0	0/0	0/0	0/0	0/0	0/0	0/0	0/0	0/0	0/0	0/0		
Chromosome3-3468159	INS	PRECISE	70	0	0	0/1	0/1	1/1	1/1	1/1	1/1	0/1	0/1	1/1	1/1	0/1	1/1	1/1	0/1		
Chromosome3-3469246	INS	PRECISE	3291	0	0	0/0	0/0	0/0	0/0	0/0	0/0	0/0	0/0	0/0	1/1	0/0	0/0	0/0	0/0		
Chromosome3-3473244	INS	PRECISE	56	0	0	0/0	0/0	0/0	0/0	0/0	0/0	0/0	0/0	0/0	0/1	0/0	0/0	0/0	0/0		
Chromosome3-3476999	DEL	PRECISE	242	0	0	0/1	0/0	0/1	0/1	0/1	0/1	0/1	0/1	0/0	0/1	0/0	0/1	0/0	0/1		
Chromosome3-3479965	DEL	PRECISE	411	0	0	0/1	0/0	0/1	0/1	0/1	0/1	0/1	0/1	0/0	0/1	0/0	0/1	0/0	0/1		
Chromosome3-3486574	INS	PRECISE	623	0	0.738	0/0	0/1	0/1	0/1	0/1	0/1	0/0	0/0	1/1	0/1	0/1	0/1	1/1	0/0		
Chromosome3-3492273	BND	PRECISE	0	0	0	0/0	0/0	0/0	0/0	0/0	0/0	0/0	0/0	0/0	0/1	0/0	0/0	0/0	0/0		
Chromosome3-3493292	DEL	PRECISE	435	0	0	0/1	0/0	0/1	0/1	0/1	0/0	0/1	0/1	0/0	0/1	0/0	0/1	0/0	0/1		
Chromosome3-3495201	INS	PRECISE	9891	0	0	0/0	0/0	0/0	0/0	0/0	0/0	0/0	0/0	0/0	0/0	0/1	0/0	0/0	0/0		
Chromosome3-3504088	DEL	PRECISE	131	0	0	0/0	0/0	0/0	0/0	0/0	0/0	0/0	0/0	0/0	0/0	0/1	0/0	0/0	0/0		
Chromosome3-3504395	INS	PRECISE	1126	0	0.707	0/1	0/0	0/1	0/0	0/0	0/0	0/0	0/0	0/0	0/0	0/0	0/0	0/0	0/0		
Chromosome3-3509371	DEL	PRECISE	365	0	0	0/0	0/0	0/0	0/0	0/0	0/0	0/0	0/0	0/0	0/1	0/0	0/0	0/0	0/0		
Chromosome3-3531710	INS	PRECISE	6095	0	0	0/0	0/0	0/0	0/0	0/0	0/0	0/0	0/0	0/0	0/0	0/0	0/0	0/0	1/1		
Chromosome3-3531933	INS	PRECISE	133	0	0.707	0/0	0/0	0/0	0/0	0/0	0/0	0/1	0/0	0/0	0/0	0/0	0/0	0/0	0/0		
Chromosome3-3534395	INS	PRECISE	8249	0	0	0/0	0/0	0/0	0/0	0/0	0/0	0/0	0/0	0/0	0/0	0/0	0/0	0/0	0/1		
Chromosome3-3536603	DEL	PRECISE	379	0.949	0.316	0/1	0/0	0/1	0/1	0/1	0/0	0/1	0/0	0/0	0/1	0/0	0/1	0/0	0/1		
Chromosome3-3547213	INS	PRECISE	87	0	0	0/0	0/0	0/0	0/0	0/0	0/0	0/0	0/0	0/0	0/0	0/1	0/0	0/0	0/0		
Chromosome3-3552150	INS	PRECISE	1028	0	0	0/0	0/0	0/0	0/0	0/0	0/0	0/0	0/0	0/0	0/0	0/1	0/0	0/0	0/0		

Chromosome3-3556396	DEL	PRECISE	195	0	0	0/0	0/0	0/0	0/1	0/1	0/1	0/0	0/1	0/0	0/1	0/0	0/1	0/0	0/0
Chromosome3-3557842	DEL	PRECISE	379	0	0	0/0	0/1	1/1	0/1	0/1	0/1	0/0	0/0	1/1	0/0	0/1	0/1	1/1	0/0
Chromosome3-3559180	INS	PRECISE	1091	0	0	0/0	0/0	0/0	0/0	0/0	0/0	0/0	0/0	0/0	0/1	0/0	0/0	0/0	0/0
Chromosome3-3560038	DEL	PRECISE	364	0	0	0/1	0/0	0/1	0/1	0/1	0/1	0/1	0/1	0/0	0/1	0/0	0/1	0/0	0/1
Chromosome3-3572145	BND	PRECISE	0	0	0	0/0	0/0	0/0	0/0	0/0	0/0	0/1	0/0	0/0	0/0	0/0	0/0	0/0	0/0
Chromosome3-3585625	DEL	PRECISE	81	0	0	0/1	0/0	0/1	0/1	0/1	0/1	0/1	0/1	0/0	0/1	0/0	0/1	0/0	0/1
Chromosome3-3587559	INS	PRECISE	56	0	0	0/1	0/0	0/0	0/1	0/1	0/1	0/1	0/1	0/0	0/1	0/0	0/1	0/0	0/1
Chromosome3-3593391	INS	PRECISE	65	0	0	0/1	0/0	0/1	0/1	0/1	0/1	0/1	0/1	0/0	0/1	0/0	0/1	0/0	0/0
Chromosome3-3602210	DEL	PRECISE	94	0	0	0/1	0/0	0/1	0/1	0/0	0/0	0/1	0/0	0/0	0/0	0/0	0/0	0/0	0/1
Chromosome3-3603072	INS	PRECISE	163	0	0	0/0	0/0	0/0	0/0	0/1	0/1	0/0	0/1	0/0	0/1	0/0	1/1	0/0	0/0
Chromosome3-3603184	BND	PRECISE	0	0	0	0/0	0/0	0/1	0/0	0/0	0/0	0/0	0/0	0/0	0/0	0/0	0/0	0/0	0/0
Chromosome3-3613263	DEL	PRECISE	957	0	0	0/0	0/0	0/0	0/0	0/0	0/0	0/0	0/0	0/0	0/1	0/0	0/0	0/0	0/0
Chromosome3-3613543	DEL	PRECISE	113	0	0	0/1	0/0	0/1	0/1	0/1	0/1	0/1	0/1	0/0	0/1	0/0	0/1	0/0	0/0
Chromosome3-3613562	INS	PRECISE	90	0	0	0/0	0/0	0/0	0/0	0/0	0/0	0/0	0/0	0/0	0/0	0/0	0/0	0/0	0/1
Chromosome3-3627153	INS	PRECISE	865	0	0.876	0/0	0/1	0/1	0/1	0/1	0/1	0/0	0/1	1/1	0/0	0/1	0/1	1/1	0/0
Chromosome3-3636478	DEL	PRECISE	326	0	0	0/0	0/0	0/0	0/0	0/0	0/0	0/0	0/0	0/0	0/0	0/0	0/0	0/0	0/1
Chromosome3-3636726	DEL	PRECISE	49696230	0	0	0/0	0/0	0/0	0/0	0/0	0/0	0/0	0/0	0/0	0/0	0/0	0/0	0/1	0/0
Chromosome3-3643748	INS	PRECISE	5571	0	0	0/0	0/0	0/0	0/0	0/1	0/0	0/0	0/0	0/0	0/0	0/0	0/0	0/0	0/0
Chromosome3-3643958	DEL	PRECISE	132	0	0	0/1	0/0	0/1	0/1	0/1	0/1	0/1	0/0	0/0	0/1	0/0	0/1	0/0	0/1
Chromosome3-3648513	INS	PRECISE	356	0	0	0/0	0/0	0/0	0/0	0/0	0/0	0/0	0/0	0/0	0/0	0/0	0/0	0/0	0/1
Chromosome3-3655187	INS	PRECISE	5968	0	0	0/0	0/0	0/0	0/0	0/0	0/0	0/0	0/0	0/0	0/0	0/0	0/0	0/0	1/1
Chromosome3-3665269	DEL	PRECISE	5586	0	0	0/1	0/0	0/1	0/1	0/1	0/1	0/1	0/0	0/0	0/1	0/0	0/1	0/0	0/1
Chromosome3-3668694	INS	PRECISE	4467	0	0	0/0	0/0	0/0	0/0	0/0	0/0	0/0	0/0	0/0	0/0	1/1	0/0	0/0	0/0
Chromosome3-3674083	DEL	PRECISE	243	0	0	0/1	0/0	0/1	0/1	0/1	0/1	0/1	0/0	0/0	1/1	0/0	0/1	0/0	0/1
Chromosome3-3680549	DEL	PRECISE	5080	0	0	0/0	0/0	0/0	0/0	0/0	0/0	0/0	0/0	0/0	0/0	0/1	0/0	0/0	0/0
Chromosome3-3681824	DEL	PRECISE	1027	0	0	0/1	0/0	0/1	0/0	0/0	0/0	0/1	0/0	0/0	0/0	0/0	0/0	0/0	0/0
Chromosome3-3685346	DEL	PRECISE	91	0	0	0/0	0/1	0/1	0/1	0/1	0/1	0/0	0/1	1/1	0/0	0/1	0/1	1/1	0/0
Chromosome3-3696254	DEL	PRECISE	50	0	0	0/1	0/0	0/1	0/1	0/1	0/1	0/1	0/0	0/0	0/1	0/0	0/1	0/0	0/0
Chromosome3-3696701	DEL	PRECISE	1004	0	0	0/0	0/0	0/0	0/0	0/0	0/0	0/0	0/0	0/0	0/0	0/0	0/0	0/0	0/1
Chromosome3-3697256	DEL	PRECISE	226	0	0	0/1	0/0	0/1	0/1	0/1	0/1	0/1	0/0	0/0	0/1	0/0	0/1	0/0	0/0
Chromosome3-3700838	DEL	PRECISE	51	0	0	0/1	0/0	0/1	0/1	0/1	0/1	0/1	0/0	0/0	0/1	0/0	0/1	0/0	0/0
Chromosome3-3707978	DEL	PRECISE	213	0	0	0/1	0/0	0/1	0/1	0/1	0/1	0/1	0/0	0/0	0/1	0/0	0/1	0/0	0/0
Chromosome3-3708962	DEL	PRECISE	78	0	0	0/0	0/0	0/0	0/0	0/0	0/0	0/0	0/0	0/0	0/0	0/0	0/0	0/0	0/1
Chromosome3-3716370	DEL	PRECISE	187	0.316	0.316	0/1	0/0	0/1	0/1	0/1	0/1	0/1	0/0	0/0	0/1	0/1	0/1	0/0	0/1
Chromosome3-3716948	INS	PRECISE	93	0	0	0/1	0/0	0/1	0/1	0/1	0/1	0/1	0/0	0/0	0/1	0/0	0/1	0/0	0/1
Chromosome3-3718697	INS	PRECISE	6281	0	0	0/0	0/0	0/0	1/1	0/0	0/0	0/0	0/0	0/0	0/0	0/0	0/0	0/0	0/0
Chromosome3-3720477	INS	PRECISE	401	0	0	0/0	0/1	0/1	0/1	0/1	0/1	0/0	0/1	1/1	0/0	0/1	0/1	1/1	0/0
Chromosome3-3736365	DEL	PRECISE	52	0	0	0/1	0/0	0/1	0/0	0/0	0/0	0/1	0/0	0/0	1/1	0/1	0/0	0/0	0/0
Chromosome3-3737406	INS	PRECISE	146	0	0	0/0	0/0	0/0	0/0	0/0	0/0	0/0	0/0	0/0	0/0	0/1	0/0	0/0	0/0
Chromosome3-3741637	INS	PRECISE	54	0	0	0/0	0/1	0/1	1/1	0/1	0/1	0/0	0/1	1/1	0/0	0/1	0/1	1/1	0/0
Chromosome3-3753032	INS	PRECISE	5576	0	0	0/0	0/0	0/0	0/0	0/0	0/0	0/0	0/0	0/0	0/0	1/1	0/0	0/0	0/0
Chromosome3-3754390	INS	PRECISE	1407	0	0	0/0	0/0	0/0	0/0	0/0	0/0	0/0	0/0	0/0	0/1	0/0	0/0	0/0	0/0
Chromosome3-3754844	DEL	PRECISE	355	0	0	0/1	0/0	0/1	0/1	0/1	0/1	0/1	0/0	0/0	1/1	0/1	0/1	0/0	1/1
Chromosome3-3761342	INS	PRECISE	700	0	0	0/0	0/0	0/0	0/0	0/0	0/0	0/0	0/0	0/0	0/0	0/1	0/0	0/0	0/0
Chromosome3-3773655	INS	PRECISE	242	0	0.577	0/0	0/0	0/0	0/1	0/0	0/0	0/0	0/0	0/0	0/1	0/1	0/0	0/0	0/0
Chromosome3-3781266	INS	PRECISE	111	0	0.69	0/1	0/0	0/1	0/0	0/1	1/1	0/1	0/0	0/0	0/1	0/0	0/1	0/0	0/0
Chromosome3-3781852	DEL	PRECISE	98	0	0	0/0	0/1	0/1	0/1	0/1	0/1	0/0	0/1	1/1	0/0	0/1	0/1	1/1	0/0
Chromosome3-3784483	DEL	PRECISE	786	0.882	0.882	0/1	0/0	0/1	0/0	0/1	1/1	0/1	0/0	0/0	0/1	0/0	0/1	0/0	0/1
Chromosome3-3795278	INS	PRECISE	340	0	0.577	0/0	0/0	0/0	0/0	0/0	0/0	0/0	0/1	0/0	0/0	0/0	1/1	0/1	0/0
Chromosome3-3800438	INS	PRECISE	51	0	0	0/1	0/1	1/1	1/1	1/1	1/1	0/1	0/1	1/1	1/1	0/1	1/1	1/1	0/1
Chromosome3-3803190	INS	PRECISE	202	0	0	0/0	0/0	0/0	0/0	0/0	0/0	0/0	0/0	0/0	0/0	0/0	0/0	0/0	0/1
Chromosome3-3812491	DEL	PRECISE	166	0	0	0/1	0/0	0/1	0/1	0/1	0/1	0/1	0/0	0/0	0/1	0/0	0/1	0/0	0/1
Chromosome3-3816108	INS	PRECISE	623	0.316	0.876	0/0	0/1	0/1	0/1	0/1	0/1	0/0	0/1	1/1	0/0	1/1	0/1	1/1	0/0
Chromosome3-3817140	DEL	PRECISE	74	0	0	0/1	0/0	0/1	0/0	0/0	0/0	0/1	0/0	0/0	0/0	0/0	0/0	0/0	0/0
Chromosome3-3819252	DEL	PRECISE	238	0	0	0/0	0/1	0/1	0/1	0/1	0/1	0/0	0/1	1/1	0/0	1/1	0/1	1/1	0/0
Chromosome3-3823642	DEL	PRECISE	61	0	0	0/0	0/0	0/0	0/0	0/0	0/0	0/0	0/0	0/0	0/0	0/0	0/0	0/0	0/1
Chromosome3-3833184	INS	PRECISE	95	0	0.333	0/1	0/0	0/1	1/1	0/1	0/1	0/1	0/0	0/0	0/1	0/0	0/1	0/0	0/1
Chromosome3-3842228	DEL	PRECISE	624	0	0	0/0	0/0	0/0	0/0	0/0	0/0	0/0	0/1	0/0	0/0	1/1	1/1	1/1	0/0
Chromosome3-3847271	DEL	PRECISE	200	0	0	0/0	0/0	0/0	0/0	0/0	0/0	0/0	0/0	0/0	0/1	0/0	0/0	0/0	0/0
Chromosome3-3848480	DEL	PRECISE	61	0	0	0/0	0/0	0/0	0/0	0/0	0/0	0/0	0/0	0/0	0/1	0/0	0/0	0/0	0/0
Chromosome3-3850950	DEL	PRECISE	75	0	0	0/0	0/0	0/0	0/0	0/1	0/1	0/0	0/0	0/0	0/1	0/0	0/1	0/0	0/0
Chromosome3-3854200	DEL	PRECISE	300	0	0.726	0/1	0/1	1/1	1/1	1/1	1/1	0/1	0/1	1/1	1/1	0/1	1/1	1/1	0/1
Chromosome3-3863729	DEL	PRECISE	78	0	0	0/1	0/0	0/1	0/1	0/1	0/1	0/1	0/0	0/0	0/1	0/0	0/1	0/0	0/1
Chromosome3-3872424	INS	PRECISE	348	0	0	0/0	0/0	0/0	0/0	0/0	0/0	0/1	0/0	0/0	0/0	0/0	0/0	0/0	0/0
Chromosome3-3874971	INS	PRECISE	329	0	0	0/0	0/0	0/0	0/0	0/0	0/0	0/0	0/0	0/0	0/0	0/0	0/0	0/0	0/1
Chromosome3-3883266	BND	PRECISE	0	0	0	0/0	0/0	0/0	0/0	0/0	0/0	0/0	0/0	0/0	0/0	0/0	0/1	0/0	0/0
Chromosome3-3884121	INS	PRECISE	66	0	0	0/0	0/1	0/1	0/1	1/1	0/1	0/0	0/0	1/1	0/0	0/1	0/0	0/1	0/0
Chromosome3-3894546	INS	PRECISE	137	0	0	0/0	0/1	0/1	0/1	0/1	0/1	0/0	0/0	1/1	0/0	0/1	0/0	0/1	0/0
Chromosome3-3894564	INS	PRECISE	1700	0	0	0/0	0/0	0/0	0/0	0/0	0/0	0/0	0/0	0/0	0/1	0/0	0/0	0/0	0/0
Chromosome3-3905091	INS	PRECISE	132	0	0.333	0/1	0/0	0/1	0/1	0/1	0/1	0/1	0/0	0/0	0/1	0/0	0/1	0/0	0/1
Chromosome3-3909636	DEL	PRECISE	77	0	0	0/1	0/0	0/1	0/1	0/1	0/1	0/1	0/0	0/0	0/1	0/0	0/1	0/0	0/1
Chromosome3-3910431	INS	PRECISE	61	0	0.577	0/0	0/0	0/0	0/0	0/0	0/0	0/0	0/1	0/0	0/0	0/0	0/1	0/1	0/0
Chromosome3-3916415	INS	PRECISE	596	0	0.5	1/1	0/0	1/1	1/1	0/0	0/0	0/1	0/0	0/0	0/0	0/0	0/0	0/0	0/0

Chromosome3-3916945	DEL	PRECISE	219	0	0	0/0	0/0	0/0	0/0	0/0	0/0	0/0	0/0	0/0	0/0	0/0	0/0	0/0	1/1
Chromosome3-3918058	DEL	PRECISE	782	0	0	0/0	0/1	1/1	1/1	1/1	1/1	1/1	1/1	1/1	1/1	1/1	1/1	1/1	0/0
Chromosome3-3921504	DEL	PRECISE	81	0	0	0/0	0/0	0/0	0/0	0/0	0/0	0/0	0/0	0/0	0/1	0/0	0/1	0/0	0/0
Chromosome3-3924143	INS	PRECISE	6422	0	0	0/0	0/0	0/0	0/0	0/0	0/0	1/1	0/0	0/0	0/0	0/0	0/0	0/0	0/0
Chromosome3-3934309	DEL	PRECISE	451	0	0	0/1	0/0	0/1	0/1	0/1	0/0	0/1	0/0	0/0	0/1	0/0	0/0	0/0	0/0
Chromosome3-3948330	DEL	PRECISE	186	0	0	0/1	0/0	0/1	0/1	0/1	0/1	0/0	0/0	0/0	0/1	0/0	0/0	0/0	0/0
Chromosome3-3953497	INS	PRECISE	211	0	0.548	0/1	0/0	0/1	0/1	0/1	0/1	0/0	0/0	0/0	0/1	0/0	0/0	0/0	0/0
Chromosome3-3954044	DEL	PRECISE	94	0	0	0/1	0/0	0/1	0/1	0/1	0/1	0/0	0/0	0/0	0/1	0/0	0/0	0/0	0/0
Chromosome3-3955667	INS	PRECISE	157	0	0.302	0/1	0/0	0/1	0/1	0/1	0/1	0/1	0/1	0/0	0/1	0/0	0/1	0/1	0/0
Chromosome3-3963380	INS	PRECISE	107	0	0.408	0/1	0/0	0/1	0/1	0/1	0/1	0/0	0/0	0/0	0/1	0/0	0/0	0/0	0/0
Chromosome3-3966250	INS	PRECISE	91	0	0.354	0/1	0/0	0/1	0/1	0/1	0/1	0/1	0/0	0/0	0/1	0/0	0/0	0/0	0/1
Chromosome3-3976125	INS	PRECISE	624	0	0.894	0/1	0/0	0/1	0/0	0/1	0/1	0/0	0/0	0/0	0/1	0/0	0/0	0/0	0/0
Chromosome3-3982175	INS	PRECISE	349	0	0	0/0	0/0	0/0	0/0	0/0	0/0	0/0	0/0	0/0	0/0	0/0	0/0	0/0	0/1
Chromosome3-3989889	INS	PRECISE	1608	0	0.463	0/0	0/1	0/1	0/1	1/1	0/1	0/0	0/0	1/1	0/0	0/1	0/0	0/1	0/0
Chromosome3-3990363	DEL	PRECISE	1031	0	0	0/0	0/0	0/0	0/0	0/0	0/0	0/0	0/0	0/0	0/0	0/0	0/0	0/0	0/1
Chromosome3-3995306	DEL	PRECISE	295	0	0	0/1	0/0	0/1	0/1	0/1	0/1	0/1	0/0	0/0	0/1	0/0	0/0	0/0	0/1
Chromosome3-3997438	DUP	PRECISE	48752523	0	0	0/0	0/0	0/0	0/0	0/0	0/0	0/0	0/0	0/0	0/0	0/0	0/1	0/0	0/0
Chromosome3-4000859	INS	PRECISE	1805	0	0	0/0	0/0	0/0	0/0	0/0	0/0	0/0	0/0	0/0	0/0	0/1	0/0	0/0	0/0
Chromosome3-4012116	INS	PRECISE	1025	0	0	0/0	0/0	0/0	0/0	0/0	0/0	0/0	0/0	0/0	0/0	0/1	0/0	0/0	0/0
Chromosome3-4022344	INS	PRECISE	1025	0	0.354	0/1	0/0	0/1	0/1	0/1	0/1	0/1	0/0	0/0	0/1	0/0	0/0	0/0	0/1
Chromosome3-4030245	BND	PRECISE	0	0	0	0/0	0/0	0/0	0/0	0/1	0/1	0/0	0/0	0/0	0/1	0/0	0/0	0/0	0/0
Chromosome3-4045230	DUP	PRECISE	29082	0	0	0/0	0/0	0/0	0/0	0/0	0/1	0/0	0/0	0/0	0/1	0/0	0/0	0/0	0/0
Chromosome3-4047629	INS	PRECISE	107	0	0	0/1	0/0	0/1	0/1	0/1	0/1	0/1	0/0	0/0	0/1	0/0	0/0	0/0	0/1
Chromosome3-4059743	DEL	PRECISE	283	0	0	0/1	0/0	0/0	0/0	0/1	0/1	0/1	0/0	0/0	0/1	0/0	0/0	0/0	0/1
Chromosome3-4060684	INS	PRECISE	824	0	0	0/0	0/0	0/0	0/0	0/0	0/0	1/1	0/0	0/0	0/0	0/0	0/0	0/0	0/0
Chromosome3-4060827	BND	PRECISE	0	0	0	0/1	0/0	0/0	0/0	0/1	0/1	0/0	0/0	0/0	0/0	0/0	0/0	0/0	0/0
Chromosome3-4061787	DEL	PRECISE	138	0	0	0/0	0/0	0/0	0/0	0/0	0/0	0/0	0/0	0/0	1/1	0/0	1/1	0/0	0/0
Chromosome3-4065741	INS	PRECISE	997	0	0	0/0	0/0	0/0	0/0	0/0	0/0	0/0	0/0	0/0	0/0	0/0	0/0	0/0	1/1
Chromosome3-4070278	DEL	PRECISE	171	0	0	0/0	0/0	0/0	0/1	0/1	0/1	0/1	0/0	0/0	0/1	0/0	0/0	0/0	0/0
Chromosome3-4088156	INS	PRECISE	64	0	0	0/0	0/0	0/0	0/0	0/0	0/0	0/0	0/0	0/0	0/0	0/1	0/0	0/0	0/0
Chromosome3-4093163	DEL	PRECISE	121	0	0	0/1	0/0	0/1	0/0	0/0	0/0	0/0	0/0	0/0	0/0	0/0	0/0	0/0	0/0
Chromosome3-4096787	DEL	PRECISE	58	0	0	0/1	0/0	0/1	0/1	0/1	0/1	0/1	0/0	0/0	0/1	0/1	0/0	0/0	0/1
Chromosome3-4101994	INS	PRECISE	161	0	0.577	0/0	0/0	0/0	0/0	0/0	0/0	0/0	0/1	0/0	0/0	0/0	0/1	0/1	0/0
Chromosome3-4105866	DEL	PRECISE	132	0	0	0/1	0/1	1/1	1/1	1/1	1/1	0/1	0/1	1/1	1/1	1/1	1/1	1/1	0/1
Chromosome3-4111848	DEL	PRECISE	189	0	0	0/0	0/0	0/0	0/0	0/1	0/1	0/1	0/0	0/0	0/1	0/0	0/0	0/0	0/0
Chromosome3-4112758	INS	PRECISE	1089	0	0.707	1/1	0/0	0/1	0/0	0/0	0/0	0/0	0/0	0/0	0/0	0/0	0/0	0/0	0/0
Chromosome3-4113035	INV	PRECISE	413	0	0	0/0	0/0	0/0	0/1	0/0	0/0	0/0	0/0	0/0	0/0	0/0	0/0	0/0	0/0
Chromosome3-4114194	DEL	PRECISE	1199	0	0	0/1	0/1	1/1	1/1	1/1	1/1	0/1	0/0	1/1	1/1	1/1	0/0	1/1	0/0
Chromosome3-4119906	DEL	PRECISE	58	0	0	0/1	0/0	0/1	0/1	0/1	0/1	0/1	0/0	0/0	0/1	0/0	0/0	0/0	0/1
Chromosome3-4125870	INS	PRECISE	52	0	0	0/1	0/0	0/1	0/0	0/1	0/1	0/0	0/0	0/0	0/1	0/0	0/0	0/0	0/0
Chromosome3-4126078	INS	PRECISE	3062	0	0	0/0	0/0	0/0	0/0	0/0	0/0	0/0	0/0	0/0	0/0	0/0	0/0	0/0	1/1
Chromosome3-4127059	INS	PRECISE	140	0	0.408	0/1	0/0	0/1	0/0	0/1	0/1	0/0	0/0	0/0	0/1	0/0	0/0	0/0	0/0
Chromosome3-4128971	DEL	PRECISE	155	0	0	0/0	0/0	0/0	0/0	0/0	0/0	0/0	0/0	0/0	0/0	0/0	0/0	0/0	0/1
Chromosome3-4129388	DEL	PRECISE	2313	0	0	0/0	0/0	0/0	0/0	0/0	0/0	0/0	0/0	0/0	0/0	0/0	0/0	0/0	0/1
Chromosome3-4136969	DEL	PRECISE	276	0	0	0/1	0/0	0/1	0/1	0/1	0/1	0/1	0/0	0/0	0/1	0/0	0/0	0/0	0/1
Chromosome3-4137771	INS	PRECISE	51	0	0	0/1	0/0	0/1	0/0	0/0	0/0	0/0	0/0	0/0	0/0	0/0	0/0	0/0	0/0
Chromosome3-4144545	INS	PRECISE	1128	0	0	0/0	0/0	0/0	0/1	0/0	0/0	0/0	0/0	0/0	0/0	0/0	0/0	0/0	0/0
Chromosome3-4146711	DEL	PRECISE	5448	0	0	0/0	0/1	1/1	1/1	1/1	1/1	0/1	0/1	1/1	1/1	0/1	1/1	1/1	0/1
Chromosome3-4154157	INS	PRECISE	139	0	0.505	0/1	0/0	0/0	0/1	0/1	0/1	0/1	0/1	0/0	0/1	0/0	0/1	0/1	0/1
Chromosome3-4159258	DEL	PRECISE	117	0	0	0/0	0/1	1/1	1/1	1/1	1/1	0/0	0/0	1/1	1/1	0/1	0/1	0/0	0/0
Chromosome3-4163906	DEL	PRECISE	80	0	0	0/0	0/0	0/0	0/0	0/0	0/0	0/0	0/0	0/0	0/0	0/0	0/0	0/0	0/1
Chromosome3-4164150	INS	PRECISE	52	0	0	0/0	0/0	0/0	0/0	0/0	0/0	0/1	0/0	0/0	0/0	0/0	0/0	0/0	0/0
Chromosome3-4176788	DEL	PRECISE	138	0	0	0/1	0/0	0/1	0/1	0/1	0/1	0/1	0/0	0/0	0/1	0/0	0/0	0/0	0/1
Chromosome3-4184885	BND	PRECISE	0	0	0	0/0	0/0	0/0	0/0	0/0	0/0	0/0	0/0	0/0	0/0	0/0	0/1	0/0	0/0
Chromosome3-4203464	BND	PRECISE	0	0	0	0/0	0/0	0/0	0/0	0/0	0/0	0/0	0/0	0/0	0/1	0/0	0/0	0/0	0/0
Chromosome3-4204573	DEL	PRECISE	42945442	0	0	0/0	0/0	0/0	0/0	0/0	0/0	0/0	0/0	0/0	0/1	0/0	0/0	0/0	0/0
Chromosome3-4218007	DEL	PRECISE	85	0	0	0/1	0/0	0/1	0/1	0/1	0/1	0/1	0/0	0/0	0/1	0/0	0/0	0/0	0/1
Chromosome3-4219704	DEL	PRECISE	59	0	0	0/1	0/0	0/1	0/1	0/1	0/1	0/1	0/0	0/0	0/1	0/0	0/0	0/0	0/1
Chromosome3-4225805	INV	PRECISE	47107897	0	0	0/0	0/0	0/0	0/0	0/0	0/0	0/0	0/0	0/0	0/0	0/0	0/0	0/0	0/1
Chromosome3-4246180	DEL	PRECISE	2479	0.707	0.707	1/1	0/0	1/1	1/1	1/1	1/1	1/1	0/0	0/0	0/1	0/0	0/0	0/0	1/1
Chromosome3-4248424	DEL	PRECISE	235	0	0	0/0	0/0	0/0	0/0	0/0	0/0	0/0	0/1	0/0	1/1	0/0	1/1	0/1	0/0
Chromosome3-4252242	INS	PRECISE	50	0	0	0/0	0/0	0/0	0/0	0/0	0/0	0/0	0/0	0/0	0/0	0/0	0/0	0/0	0/1
Chromosome3-4274316	DEL	PRECISE	58	0	0	0/1	0/0	0/1	0/1	0/1	0/1	0/1	0/0	0/0	0/1	0/0	0/0	0/0	0/1
Chromosome3-4278276	DEL	PRECISE	93	0	0	0/1	0/0	0/1	1/1	0/1	0/1	0/1	0/0	0/0	0/1	0/0	0/0	0/0	0/0
Chromosome3-4296693	INS	PRECISE	651	0	0.707	0/0	0/0	0/0	0/0	0/0	0/0	0/0	0/0	0/0	1/1	0/0	0/0	0/0	0/0
Chromosome3-4296765	DEL	PRECISE	367	0	0	0/1	0/0	0/1	0/1	0/1	0/1	0/0	0/0	0/0	1/1	0/0	0/0	0/0	0/0
Chromosome3-4297274	DEL	PRECISE	1815	0	0	0/1	0/0	0/1	0/1	0/1	0/1	0/1	0/0	0/0	1/1	0/1	1/1	0/0	0/1
Chromosome3-4303249	DEL	PRECISE	149	0	0	0/1	0/0	0/1	0/1	0/1	0/1	0/1	0/0	0/0	0/1	0/0	0/0	0/0	0/1
Chromosome3-4305845	DEL	PRECISE	96	0	0	0/0	0/0	0/0	0/0	0/0	0/0	0/0	0/0	0/0	0/0	0/0	0/0	0/0	0/1
Chromosome3-4306243	INS	PRECISE	132	0	0	0/1	0/0	0/1	0/0	0/0	0/0	0/0	0/0	0/0	0/0	0/0	0/0	0/0	0/0
Chromosome3-4331558	DEL	PRECISE	51	0	0	0/0	0/0	0/0	0/1	0/0	0/0	0/0	0/1	0/0	0/1	0/1	1/1	0/1	0/0
Chromosome3-4334270	DEL	PRECISE	61	0	0	0/0	0/0	0/0	0/0	0/0	0/0	0/0	0/0	0/0	0/0	0/1	0/0	0/0	0/0
Chromosome3-4338026	DEL	PRECISE	697	0	0	0/1	0/0	0/1	0/1	0/1	0/1	0/0	0/0	0/0	0/1	0/0	0/0	0/0	0/1

Chromosome3-4342512 INS	PRECISE	70	0	0.354	0/1	0/0	0/1	0/1	0/1	0/1	0/1	0/0	0/0	0/1	0/0	0/0	0/0	0/1
Chromosome3-4344324 INS	PRECISE	1187	0	0.707	0/0	0/0	0/0	0/0	0/0	0/0	0/0	0/0	0/0	0/1	0/0	0/1	0/0	0/0
Chromosome3-4344856 DEL	PRECISE	77	0	0	0/0	0/0	0/0	0/0	0/0	0/0	0/0	0/0	0/0	0/0	0/1	0/0	0/0	0/0
Chromosome3-4349891 INS	PRECISE	641	0	0	0/0	0/0	0/0	0/0	0/0	0/0	0/0	0/0	0/0	0/0	0/1	0/0	0/0	0/0
Chromosome3-4359306 DEL	PRECISE	64	0	0	0/1	0/0	0/1	0/1	0/1	0/1	0/1	0/0	0/0	0/1	0/0	0/0	0/0	0/1
Chromosome3-4364906 DEL	PRECISE	54	0	0	0/1	0/0	0/1	0/1	0/1	0/1	0/1	0/0	0/0	0/1	0/0	0/0	0/0	0/1
Chromosome3-4383304 DEL	PRECISE	157	0	0	0/0	0/0	0/0	0/0	0/0	0/0	0/0	0/0	0/0	0/0	0/0	0/0	0/0	0/1
Chromosome3-4383723 DEL	PRECISE	774	0	0	0/0	0/0	0/0	0/0	0/0	0/0	0/0	0/0	0/0	0/0	0/0	0/0	0/0	0/1
Chromosome3-4384622 DEL	PRECISE	97	0	0	0/0	0/0	0/0	0/0	0/0	0/0	0/0	0/0	0/0	0/0	0/0	0/0	0/0	0/1
Chromosome3-4393433 DEL	PRECISE	61	0	0	0/0	0/0	0/0	0/1	0/1	0/1	0/1	0/0	0/0	0/1	0/0	0/0	0/0	0/1
Chromosome3-4398430 INS	PRECISE	281	0	0	0/0	0/0	0/0	0/0	0/0	0/0	0/0	0/0	0/0	0/0	0/0	0/0	0/0	0/1
Chromosome3-4400006 INS	PRECISE	491	0	0	0/0	0/0	0/0	0/0	0/0	0/0	0/0	0/1	0/0	0/0	0/0	1/1	0/0	0/0
Chromosome3-4400721 BND	PRECISE	0	0	0	0/0	0/0	0/0	0/0	0/0	0/0	0/0	0/0	0/0	0/0	0/0	0/0	0/0	0/1
Chromosome3-4406675 DEL	PRECISE	58	0	0	0/0	0/0	0/0	0/1	0/0	0/0	0/0	0/0	0/0	0/0	0/0	0/0	0/0	0/0
Chromosome3-4410184 INS	PRECISE	73	0	0	0/1	0/0	0/1	0/0	0/0	0/0	0/0	0/0	0/0	0/0	0/0	0/0	0/0	0/0
Chromosome3-4418304 INS	PRECISE	703	0	0	0/0	0/0	0/0	0/0	0/0	0/0	0/0	0/0	0/0	0/0	0/0	0/0	0/1	0/0
Chromosome3-4424039 INS	PRECISE	49	0	0.577	0/0	0/0	0/0	0/0	0/1	0/1	0/0	0/0	0/0	0/1	0/0	0/0	0/0	0/0
Chromosome3-4431111 INS	PRECISE	53	0	0.452	0/1	0/0	0/1	0/1	0/1	1/1	0/1	0/1	0/0	1/1	0/1	1/1	0/1	0/1
Chromosome3-4432649 INS	PRECISE	336	0	0	0/0	0/0	0/0	0/1	0/0	0/0	0/0	0/0	0/0	0/0	0/0	0/0	0/0	0/0
Chromosome3-4433250 DEL	PRECISE	52	0	0	0/0	0/0	0/0	0/0	0/0	0/0	0/0	0/0	0/0	0/0	0/0	0/0	0/0	0/1
Chromosome3-4436896 DEL	PRECISE	1893	0	0	0/0	0/0	0/0	0/0	0/0	0/0	0/0	0/1	0/0	0/0	0/0	1/1	0/0	0/0
Chromosome3-4438781 INS	PRECISE	67	0	0.354	0/1	0/0	0/1	0/1	0/0	0/1	0/1	0/0	0/0	0/1	0/0	0/0	0/0	0/1
Chromosome3-4445671 INS	PRECISE	1072	0	0	0/0	0/0	0/0	0/0	0/0	0/0	0/0	0/0	0/0	0/0	0/0	0/0	1/1	0/0
Chromosome3-4447452 INS	PRECISE	148	0	0.69	0/1	0/0	0/1	0/1	0/1	0/0	0/1	0/0	0/0	0/1	0/0	0/0	0/0	0/1
Chromosome3-4449411 DEL	PRECISE	350	0	0	0/1	0/0	0/1	0/1	0/1	0/1	0/1	0/0	0/0	1/1	0/0	0/1	0/1	0/1
Chromosome3-4450233 INS	PRECISE	141	0	0.577	0/0	0/0	0/0	0/0	0/1	0/1	0/0	0/0	0/0	0/1	0/0	0/0	0/0	0/0
Chromosome3-4450241 INS	PRECISE	1521	0	0	0/0	0/0	0/0	0/0	0/0	0/0	0/0	0/0	0/0	0/0	0/1	0/0	0/0	0/0
Chromosome3-4451028 DEL	PRECISE	173	0	0	0/1	0/0	0/1	0/1	0/1	0/1	0/1	0/0	0/0	0/1	0/0	0/0	0/0	0/1
Chromosome3-4452139 INS	PRECISE	568	0.354	0.744	0/1	0/0	0/1	0/1	0/1	0/1	0/1	0/0	0/0	0/1	0/0	0/0	0/0	0/1
Chromosome3-4463756 INS	PRECISE	938	0	0.957	0/1	0/0	0/1	0/1	0/0	0/0	0/0	0/0	0/0	0/0	0/0	0/0	0/0	1/1
Chromosome3-4464454 DEL	PRECISE	121	0	0	0/1	0/0	0/1	0/1	0/0	0/0	0/0	0/0	0/0	0/0	0/0	0/0	0/0	1/1
Chromosome3-4464770 INS	PRECISE	3416	0	0	0/0	0/0	0/0	0/0	0/0	0/0	0/0	0/0	0/0	0/0	1/1	0/0	0/0	0/0
Chromosome3-4464770 DUP	PRECISE	4379325	0	0	0/0	0/0	0/0	0/0	0/0	0/0	0/0	0/0	0/0	0/0	0/1	0/0	0/0	0/0
Chromosome3-4465878 INS	PRECISE	1927	0	0	0/0	0/0	0/0	0/0	0/0	0/0	0/0	0/0	0/0	0/0	1/1	0/0	0/0	0/0
Chromosome3-4466240 INS	PRECISE	339	0	0.5	0/0	0/0	0/0	0/0	0/1	0/1	0/1	0/0	0/0	1/1	0/0	0/0	0/0	0/0
Chromosome3-4466734 DEL	PRECISE	53	0	0	0/0	0/0	0/0	0/0	0/1	0/1	0/1	0/0	0/0	1/1	0/0	0/0	0/0	0/0
Chromosome3-4469788 INS	PRECISE	646	0	0	0/0	0/0	0/0	0/0	0/0	0/0	0/0	0/0	0/0	0/0	0/1	0/0	0/0	0/0
Chromosome3-4473464 DEL	PRECISE	66	0	0	0/0	0/0	0/0	0/0	0/1	0/1	0/1	0/0	0/0	1/1	0/0	0/0	0/0	0/0
Chromosome3-4480206 DEL	PRECISE	746	0	0	0/0	0/0	0/0	0/0	0/0	0/0	0/0	0/1	0/0	0/0	0/1	1/1	0/0	0/0
Chromosome3-4487317 DEL	PRECISE	3548	0	0	0/0	0/0	0/0	0/0	0/0	0/1	0/1	0/0	0/0	0/0	0/0	0/0	0/0	0/0
Chromosome3-4488563 DEL	PRECISE	86	0	0	0/0	0/0	0/0	0/0	0/0	0/0	0/0	0/0	0/0	0/0	0/0	0/0	0/1	0/0
Chromosome3-4496255 DEL	PRECISE	1104	0	0	0/0	0/0	0/0	0/0	0/0	0/0	0/0	0/0	0/0	0/0	0/1	0/0	0/0	0/0
Chromosome3-4496526 INS	PRECISE	599	0	0	0/0	0/0	0/0	0/0	0/0	0/1	0/0	0/0	0/0	1/1	0/0	0/0	0/0	0/0
Chromosome3-4511118 DEL	PRECISE	83	0	0	0/1	0/0	0/1	0/1	0/1	0/1	0/0	0/0	0/0	0/1	0/0	0/0	0/0	0/1
Chromosome3-4511274 INS	PRECISE	105	0	0	0/0	0/0	0/0	0/0	0/0	0/0	0/0	0/0	0/0	0/0	0/0	0/0	0/0	0/1
Chromosome3-4515807 DEL	PRECISE	94	0	0	0/0	0/0	0/0	0/0	0/0	0/0	0/0	0/0	0/0	0/0	0/0	0/0	0/0	0/1
Chromosome3-4524421 INS	PRECISE	296	0	0.354	0/1	0/0	0/1	0/1	0/1	0/1	0/1	0/0	0/0	0/1	0/0	0/0	0/0	0/1
Chromosome3-4537752 DEL	PRECISE	100	0	0	0/1	0/0	0/1	0/1	0/1	0/1	0/1	0/0	0/0	0/0	0/0	0/0	0/0	0/1
Chromosome3-4542623 INS	PRECISE	4971	0	0	0/0	0/0	0/0	0/0	0/0	0/0	1/1	0/0	0/0	0/0	0/0	0/0	0/0	0/0
Chromosome3-4549117 DEL	PRECISE	504	0	0	0/0	0/0	0/0	0/0	0/0	0/0	0/0	0/0	0/0	1/1	0/0	0/1	0/0	0/0
Chromosome3-4551199 DEL	PRECISE	7181	0.441	0.441	1/1	0/0	1/1	1/1	0/1	1/1	0/1	0/0	0/0	1/1	0/0	0/1	0/0	1/1
Chromosome3-4559942 INS	PRECISE	7119	0	0	0/0	0/0	0/0	0/1	0/0	0/0	0/0	0/0	0/0	0/0	0/0	0/0	0/0	0/0
Chromosome3-4559942 BND	PRECISE	0	0	0	0/0	0/0	0/0	0/1	0/0	0/0	0/0	0/0	0/0	0/0	0/0	0/0	0/0	0/0
Chromosome3-4560903 INS	PRECISE	171	0	0.707	0/0	0/0	0/0	0/0	0/0	0/0	0/0	0/1	0/0	0/0	0/0	0/1	0/0	0/0
Chromosome3-4564235 INS	PRECISE	67	0	0	0/0	0/0	0/0	0/0	0/0	0/0	0/0	0/0	0/0	0/0	0/0	0/0	0/1	0/0
Chromosome3-4568392 DEL	PRECISE	51	0	0	0/1	0/0	0/1	0/1	0/1	0/1	0/1	0/0	0/0	0/1	0/0	0/0	0/0	0/1
Chromosome3-4569324 INS	PRECISE	1020	0	0	0/1	0/0	0/1	0/0	0/0	0/0	0/0	0/0	0/0	0/0	0/0	0/0	0/0	0/0
Chromosome3-4584875 DEL	PRECISE	211	0	0	0/0	0/0	0/0	0/0	0/0	0/0	0/0	0/1	0/0	1/1	0/0	1/1	0/1	0/0
Chromosome3-4595499 DEL	PRECISE	117	0	0	0/1	0/0	0/1	0/1	0/1	0/1	0/1	0/0	0/0	0/1	0/0	0/0	0/0	0/1
Chromosome3-4597255 DEL	PRECISE	148	0	0	0/0	0/0	0/0	0/0	0/0	0/0	0/0	0/0	0/0	0/0	0/1	0/0	0/0	0/0
Chromosome3-4601302 INS	PRECISE	184	0	0	0/0	0/0	0/0	0/1	0/0	0/0	0/0	0/0	0/0	0/0	0/0	0/0	0/0	0/0
Chromosome3-4601730 DEL	PRECISE	64	0	0	0/0	0/0	0/0	0/0	0/0	0/0	0/0	0/0	0/0	0/0	0/0	0/0	0/0	0/1
Chromosome3-4604060 DEL	PRECISE	170	0	0	0/0	0/0	0/0	0/0	0/0	0/0	0/0	0/1	0/0	0/0	0/1	1/1	0/0	0/0
Chromosome3-4606089 INS	PRECISE	5633	0	0	0/0	0/0	0/0	0/0	0/0	0/0	0/0	0/0	0/0	0/0	0/0	0/0	0/0	1/1
Chromosome3-4608155 DEL	PRECISE	77	0	0	0/1	0/0	0/1	0/1	0/1	1/1	0/1	0/0	0/0	0/1	0/0	0/0	0/0	0/0
Chromosome3-4616872 INS	PRECISE	90	0	0	0/0	0/0	0/0	0/1	0/0	0/0	0/0	0/0	0/0	0/0	0/0	0/0	0/0	0/0
Chromosome3-4617363 INS	PRECISE	610	0	0	0/0	0/0	0/0	0/1	0/0	0/0	0/0	0/0	0/0	0/0	0/0	0/0	0/0	0/0
Chromosome3-4618289 INS	PRECISE	1201	0	0.894	0/1	0/0	0/1	0/0	1/1	0/1	0/1	0/0	0/0	0/1	0/0	0/0	0/0	0/0
Chromosome3-4619950 DEL	PRECISE	3361	0	0	0/0	0/0	0/0	0/0	0/0	0/0	0/0	0/1	0/0	0/0	0/0	0/1	0/0	0/0
Chromosome3-4621528 DEL	PRECISE	87	0	0	0/0	0/0	0/0	0/1	0/0	0/0	0/0	0/0	0/0	0/0	0/0	0/0	0/0	0/1
Chromosome3-4622533 BND	PRECISE	0	0.5	0	0/0	0/0	0/0	0/1	0/0	0/0	0/0	0/0	0/0	0/0	0/0	0/0	0/0	0/1
Chromosome3-4623721 INS	PRECISE	70	0	0.707	0/0	0/0	0/0	0/0	0/0	0/0	0/0	0/1	0/0	0/0	0/0	0/1	0/0	0/0
Chromosome3-4637204 DEL	PRECISE	1045	0	0	0/0	0/0	0/0	0/1	0/0	0/0	0/0	0/0	0/0	0/0	0/0	0/0	0/0	0/0

Chromosome3-4638660 DEL	PRECISE	261	0	0	0/0	0/0	0/0	0/0	0/0	0/0	0/0	0/0	0/0	0/0	0/0	0/0	0/0	0/1
Chromosome3-4642963 DEL	PRECISE	416	0	0	0/1	0/0	0/0	0/1	0/0	0/0	0/0	0/0	0/0	0/0	0/0	0/0	0/1	0/1
Chromosome3-4643715 BND	PRECISE	0	0	0	0/0	0/0	0/0	0/0	0/1	0/1	0/1	0/0	0/0	0/1	0/0	0/0	0/0	0/0
Chromosome3-4646096 INS	PRECISE	58	0	0	0/1	0/0	0/1	0/0	0/0	0/0	0/0	0/0	0/0	0/0	0/0	0/0	0/0	0/0
Chromosome3-4651933 DEL	PRECISE	74	0.463	0	0/1	0/0	0/1	0/1	0/1	0/1	0/1	0/0	0/0	0/1	0/0	0/0	0/0	0/1
Chromosome3-4656427 INS	PRECISE	4968	0	0	0/0	0/0	0/0	0/0	0/0	0/0	0/0	0/0	0/0	0/0	0/0	0/0	1/1	0/0
Chromosome3-4669073 INS	PRECISE	1687	0	0	0/0	0/0	0/0	0/0	0/0	0/0	0/0	0/0	0/0	0/0	0/0	0/0	0/0	0/1
Chromosome3-4670057 INS	PRECISE	2638	0	0	0/0	0/0	0/0	0/0	0/0	0/0	0/0	0/0	0/0	0/0	0/0	0/0	0/0	1/1
Chromosome3-4670187 INS	PRECISE	1417	0	0	0/0	0/0	0/0	0/0	0/0	0/0	0/0	0/0	0/0	0/1	0/0	0/1	0/0	0/0
Chromosome3-4682395 BND	PRECISE	0	0	0	0/0	0/0	0/0	0/0	0/0	0/0	0/0	0/0	0/0	0/1	0/0	0/0	0/0	0/0
Chromosome3-4682399 BND	PRECISE	0	0	0	0/0	0/0	0/0	0/0	0/0	0/0	0/0	0/0	0/0	0/1	0/0	0/0	0/0	0/0
Chromosome3-4684735 DEL	PRECISE	204	0	0	0/0	0/0	0/0	0/0	0/0	0/0	0/0	0/0	0/0	0/0	0/0	0/0	0/0	0/1
Chromosome3-4684974 INS	PRECISE	546	0	0	0/1	0/0	0/1	0/1	0/0	0/0	0/0	0/0	0/0	0/0	0/0	0/0	0/0	0/0
Chromosome3-4687729 INS	PRECISE	316	0	0.707	0/0	0/0	0/0	0/0	0/0	0/0	0/0	0/0	0/0	0/1	0/0	0/1	0/0	0/0
Chromosome3-4689887 DEL	PRECISE	90	0	0	0/0	0/0	0/0	0/0	0/0	0/0	0/0	0/1	0/0	0/0	0/0	0/1	0/0	0/0
Chromosome3-4701047 DEL	PRECISE	377	0	0	0/1	0/0	0/1	0/1	0/1	0/1	0/1	0/0	0/0	0/1	0/0	0/0	0/0	0/1
Chromosome3-4701312 INS	PRECISE	137	0	0	0/0	0/0	0/0	0/0	0/0	0/0	0/0	0/0	0/0	0/0	0/1	0/0	0/0	0/0
Chromosome3-4702894 INS	PRECISE	642	0	0	0/0	0/0	0/0	0/0	0/0	0/0	0/0	0/0	0/0	0/0	0/0	0/0	0/1	0/0
Chromosome3-4705605 INS	PRECISE	53	0	0	0/1	0/0	0/1	0/1	0/1	0/1	0/1	0/0	0/0	0/1	0/0	0/0	0/1	0/1
Chromosome3-4720404 INS	PRECISE	189	0	0	0/0	0/0	0/0	0/0	0/0	0/0	0/0	0/1	0/0	0/0	0/0	0/1	0/0	0/0
Chromosome3-4731397 DEL	PRECISE	67	0	0	0/1	0/0	0/1	0/1	0/1	0/1	0/1	0/0	0/0	0/1	0/0	0/0	0/0	0/0
Chromosome3-4742366 DEL	PRECISE	56	0	0	0/1	0/0	1/1	0/0	0/1	0/1	0/1	0/0	0/0	1/1	0/0	0/0	0/0	0/1
Chromosome3-4743863 INS	PRECISE	712	0	0	0/0	0/0	0/0	0/1	0/0	0/0	0/0	0/0	0/0	0/0	0/0	0/0	0/0	0/0
Chromosome3-4759546 DEL	PRECISE	771	0	0	0/1	0/0	0/0	0/0	0/1	0/1	0/1	0/1	0/0	0/1	0/0	0/0	0/1	0/1
Chromosome3-4771570 DEL	PRECISE	52	0	0	0/0	0/0	0/0	0/0	0/0	0/0	0/0	0/0	0/0	0/1	0/0	0/1	0/1	0/0
Chromosome3-4772762 INS	PRECISE	217	0	0	0/0	0/0	0/0	0/0	0/0	0/0	0/0	0/0	0/0	0/0	0/1	0/0	0/0	0/0
Chromosome3-4779914 DEL	PRECISE	579	0	0	0/0	0/0	0/0	0/0	0/0	0/1	0/0	0/0	0/0	1/1	0/0	0/0	0/0	0/1
Chromosome3-4781113 DEL	PRECISE	1057	0	0	0/1	0/0	0/1	0/0	0/0	0/0	0/0	0/0	0/0	0/0	0/0	0/0	0/0	0/0
Chromosome3-4785202 DEL	PRECISE	199	0	0	0/1	0/0	0/1	0/0	0/0	0/1	0/0	0/0	0/0	1/1	0/0	0/0	0/0	0/0
Chromosome3-4786867 DEL	PRECISE	302	0	0	0/1	0/0	0/1	0/0	0/0	0/1	0/0	0/0	0/0	1/1	0/0	0/0	0/0	0/0
Chromosome3-4792969 DEL	PRECISE	12762	0	0	0/0	0/0	0/0	0/0	0/0	0/0	0/0	0/1	0/0	0/0	0/0	1/1	0/0	0/0
Chromosome3-4792974 BND	PRECISE	0	0	0	0/0	0/0	0/0	0/0	0/0	0/0	0/0	0/0	0/0	0/0	0/0	0/0	0/1	0/0
Chromosome3-4805731 INV	PRECISE	18293871	0	0	0/0	0/0	0/0	0/0	0/0	0/0	0/0	0/0	0/0	0/1	0/0	0/0	0/0	0/0
Chromosome3-4810361 DEL	PRECISE	23388	0	0	0/0	0/0	0/0	0/0	0/0	0/0	0/0	1/1	0/0	0/0	0/1	1/1	0/0	0/0
Chromosome3-4837798 INS	PRECISE	122	0	0	0/0	0/0	0/0	0/1	0/0	0/0	0/0	0/0	0/0	0/0	0/0	0/0	0/0	0/0
Chromosome3-4845040 DEL	PRECISE	400	0	0	0/1	0/0	0/1	0/1	0/0	0/0	0/0	0/0	0/0	0/0	0/0	0/0	0/0	0/1
Chromosome3-4845756 DEL	PRECISE	76	0	0	0/0	0/0	0/0	0/1	0/0	0/0	0/0	0/0	0/0	0/0	0/0	0/0	0/0	0/0
Chromosome3-4852182 INS	PRECISE	1988	0	0	0/0	0/0	0/0	0/0	0/0	0/0	0/0	0/0	0/0	0/0	0/0	0/0	0/0	0/1
Chromosome3-4853264 INS	PRECISE	1440	0	0.957	0/0	0/0	0/0	0/0	0/1	0/1	0/1	0/0	0/0	0/1	0/0	0/0	0/0	0/0
Chromosome3-4856459 DEL	PRECISE	91	0	0	0/0	0/0	0/0	0/0	0/0	0/0	0/0	0/0	0/0	0/0	0/0	0/0	0/0	0/1
Chromosome3-4866283 INS	PRECISE	323	0	0	0/0	0/0	0/0	0/0	0/0	0/0	0/0	0/0	0/0	0/0	0/1	0/0	0/0	0/0
Chromosome3-4866788 DEL	PRECISE	67	0	0	0/0	0/0	0/0	0/0	0/0	0/0	0/0	0/0	0/0	0/0	0/0	0/0	0/0	0/1
Chromosome3-4869109 DEL	PRECISE	766	0	0	0/1	0/0	0/1	0/0	0/0	0/0	0/0	0/0	0/0	0/0	0/0	0/0	0/0	0/0
Chromosome3-4870022 INS	PRECISE	89	0	0	0/1	0/0	0/1	0/0	0/0	0/0	0/0	0/0	0/0	0/0	0/0	0/0	0/0	0/0
Chromosome3-4876109 INS	PRECISE	69	0	0	0/0	0/0	0/0	0/0	0/0	0/0	0/0	0/0	0/0	0/0	0/1	0/0	0/0	0/0
Chromosome3-4880312 INS	PRECISE	1297	0	0	0/0	0/0	0/0	0/0	0/0	0/0	0/0	0/0	0/0	0/0	0/1	0/0	0/0	0/0
Chromosome3-4896183 INS	PRECISE	380	0	0	0/0	0/0	0/0	0/0	0/0	0/0	0/0	0/0	0/0	0/0	0/0	0/0	0/0	0/1
Chromosome3-4900622 INS	PRECISE	751	0	0	0/0	0/0	0/0	0/0	0/0	0/0	0/0	0/0	0/0	0/0	0/1	0/0	0/0	0/0
Chromosome3-4915897 INS	PRECISE	65	0	0.378	0/1	0/0	0/1	0/0	0/1	0/1	1/1	0/0	0/0	0/1	0/0	0/0	0/0	0/1
Chromosome3-4916087 DEL	PRECISE	859	0	0	0/1	0/0	0/1	0/0	0/1	0/1	0/1	0/0	0/0	0/1	0/0	0/0	0/0	0/1
Chromosome3-4916203 INS	PRECISE	998	0	0	0/0	0/0	0/0	0/0	0/0	0/0	0/0	0/0	0/0	0/0	0/0	0/0	0/1	0/0
Chromosome3-4920493 INS	PRECISE	137	0	0	0/0	0/0	0/0	0/0	0/0	0/0	0/0	0/0	0/0	0/0	0/1	0/0	0/0	0/0
Chromosome3-4927124 INS	PRECISE	80	0	0	0/0	0/0	0/0	0/0	0/1	0/1	0/0	0/0	0/0	0/1	0/0	0/0	0/0	0/0
Chromosome3-4927971 INS	PRECISE	643	0	0	0/0	0/0	0/0	0/0	0/0	0/0	0/0	0/0	0/0	0/0	0/0	0/0	0/0	0/1
Chromosome3-4928568 INS	PRECISE	163	0	0	0/0	0/0	0/0	0/0	0/1	0/1	0/0	0/0	0/0	0/1	0/0	0/0	0/0	0/0
Chromosome3-4929885 INS	PRECISE	134	0	0.835	0/1	0/0	0/1	0/0	0/1	0/1	0/1	0/0	0/0	0/1	0/0	0/0	0/0	0/1
Chromosome3-4935289 DEL	PRECISE	50	0	0	0/1	0/0	0/1	0/1	0/0	0/1	0/1	0/0	0/0	0/1	0/0	0/0	0/0	0/0
Chromosome3-4938454 DEL	PRECISE	58	0	0	0/1	0/0	0/1	0/1	0/0	0/1	0/1	0/0	0/0	0/1	0/0	0/0	0/0	0/1
Chromosome3-4945993 DEL	PRECISE	338	0.632	0.408	0/0	0/0	0/0	0/1	0/0	0/0	0/0	0/1	0/0	1/1	0/1	1/1	0/1	0/0
Chromosome3-4946524 INS	PRECISE	4856	0	0	0/0	0/0	0/0	1/1	0/0	0/0	0/0	0/0	0/0	0/0	0/0	0/0	0/0	0/0
Chromosome3-4949867 INS	PRECISE	5259	0	0	0/0	0/0	0/0	0/0	0/0	0/0	1/1	0/0	0/0	0/0	0/0	0/0	0/0	0/0
Chromosome3-4949867 BND	PRECISE	0	0	0	0/0	0/0	0/0	0/0	0/0	0/0	0/1	0/0	0/0	0/0	0/0	0/0	0/0	0/0
Chromosome3-4950074 DEL	PRECISE	50	0	0	0/0	0/0	0/0	0/0	0/0	0/0	0/0	0/1	0/0	0/1	0/0	1/1	0/1	0/0
Chromosome3-4958427 INS	PRECISE	3775	0	0	0/0	0/0	0/0	0/0	0/0	0/0	0/0	0/0	0/0	0/0	0/1	0/0	0/0	0/0
Chromosome3-4959572 DEL	PRECISE	50	0	0	0/0	0/0	0/0	0/0	0/0	0/0	0/0	0/0	0/0	0/0	0/0	0/0	0/0	0/1
Chromosome3-4970271 INS	PRECISE	51	0	0	0/0	0/0	0/0	0/1	0/0	0/0	0/0	0/0	0/0	0/0	0/0	0/0	0/0	0/0
Chromosome3-4975218 DEL	PRECISE	1151	0	0	0/1	0/0	0/1	0/1	0/1	0/1	0/1	0/0	0/0	0/1	0/0	0/0	0/0	0/1
Chromosome3-4976745 DEL	PRECISE	152	0	0	0/0	0/0	0/0	0/0	0/0	0/0	0/0	0/0	0/0	0/0	0/0	0/0	0/0	0/1
Chromosome3-4978241 DEL	PRECISE	4200	0	0	0/0	0/0	0/0	0/0	0/0	0/0	0/0	0/0	0/0	0/0	0/0	0/0	0/0	0/1
Chromosome3-4989946 DEL	PRECISE	17273	0.577	0.577	0/0	0/0	0/0	0/0	0/0	0/0	0/0	0/1	0/0	0/0	0/0	0/1	0/0	0/0
Chromosome3-4999144 DEL	PRECISE	5631	0	0	0/1	0/0	0/1	1/1	0/1	0/1	0/1	0/0	0/0	1/1	0/0	0/1	0/1	0/0
Chromosome3-5006340 INS	PRECISE	6986	0	0	0/0	0/0	0/0	0/0	0/0	0/0	0/0	0/0	0/0	0/0	0/0	0/1	0/0	0/0
Chromosome3-5007639 DEL	PRECISE	3908	0	0	0/1	0/0	0/1	0/0	0/0	0/0	0/0	0/0	0/0	0/0	0/0	0/0	0/0	0/0

Chromosome3-5007670	INS	PRECISE	6970	0	0	0/0	0/0	0/0	0/0	0/0	0/0	0/0	0/0	0/0	0/0	0/0	0/1	0/0	0/0
Chromosome3-5008780	INS	PRECISE	108	0	0	0/0	0/0	0/0	0/1	0/1	0/1	0/1	0/0	0/0	1/1	0/0	0/0	0/0	0/0
Chromosome3-5015658	INS	PRECISE	3976	0	0	0/0	0/0	0/0	0/0	0/0	0/0	0/0	0/0	0/0	0/0	0/0	0/1	0/0	0/0
Chromosome3-5021776	DEL	PRECISE	196	0	0	0/0	0/0	0/0	0/0	0/0	0/0	0/0	0/0	0/0	0/1	0/0	0/0	0/0	0/0
Chromosome3-5021935	BND	PRECISE	0	0	0	0/0	0/0	0/0	0/0	0/0	0/1	0/0	0/0	0/0	0/0	0/0	0/0	0/0	0/0
Chromosome3-5023608	DEL	PRECISE	1194	0.333	0	0/1	0/0	0/0	0/1	0/1	0/1	0/1	0/1	0/0	1/1	0/0	1/1	0/0	0/0
Chromosome3-5023739	INS	PRECISE	84	0	0	0/0	0/0	0/0	0/0	0/0	0/0	0/0	0/0	0/0	0/0	0/1	0/0	0/0	0/0
Chromosome3-5029663	INS	PRECISE	52	0	0	0/0	0/0	0/0	0/0	0/0	0/0	0/0	0/0	0/0	0/0	0/1	0/0	0/0	0/0
Chromosome3-5040377	DEL	PRECISE	249	0	0	0/0	0/0	0/0	0/0	0/0	0/0	0/0	0/1	0/0	1/1	0/1	1/1	0/1	0/0
Chromosome3-5042420	INS	PRECISE	68	0	0	0/0	0/0	0/0	0/0	0/0	0/0	0/1	0/0	0/0	0/0	0/0	0/0	0/0	0/0
Chromosome3-5053335	BND	PRECISE	0	0	0	0/0	0/1	0/0	0/0	0/0	0/0	0/1	1/1	0/0	0/0	0/1	0/0	0/0	0/0
Chromosome3-5057825	INS	PRECISE	1770	0	0	0/0	0/0	0/0	0/0	0/0	0/0	0/0	0/0	0/0	0/0	0/1	0/0	0/0	0/0
Chromosome3-5061949	DEL	PRECISE	5026	0.577	0.577	0/0	0/0	0/0	0/0	0/0	0/0	0/0	0/1	0/0	0/0	0/0	0/1	1/1	0/0
Chromosome3-5063157	DEL	PRECISE	239	0	0	0/0	0/0	0/0	0/0	0/0	0/0	0/0	0/0	0/0	1/1	0/1	0/1	0/0	0/0
Chromosome3-5065041	DEL	PRECISE	76	0	0	0/0	0/0	0/0	0/0	0/0	0/0	0/0	0/0	0/0	0/0	0/1	0/0	0/0	0/0
Chromosome3-5069107	DEL	PRECISE	942	0	0	0/0	0/0	0/0	0/0	0/0	0/0	0/0	0/0	0/0	0/0	0/1	0/0	0/0	0/0
Chromosome3-5070592	INS	PRECISE	4981	0	0	0/0	0/0	0/0	0/0	0/0	0/0	1/1	0/0	0/0	0/0	0/0	0/0	0/0	0/0
Chromosome3-5081407	INS	PRECISE	17874	0	0	0/0	0/0	0/0	0/0	0/0	0/0	0/0	0/0	0/0	0/0	1/1	0/0	0/0	0/0
Chromosome3-5081407	DUP	PRECISE	1424675	0	0	0/0	0/0	0/0	0/0	0/0	0/0	0/0	0/0	0/0	0/0	0/1	0/0	0/0	0/0
Chromosome3-5081412	BND	PRECISE	0	0	0	0/0	0/0	0/0	0/0	0/0	0/0	0/0	0/0	0/0	0/0	1/1	0/0	0/0	0/0
Chromosome3-5082011	DEL	PRECISE	409	0	0	0/0	0/0	0/0	0/1	0/0	0/0	0/1	0/0	0/0	0/0	0/0	1/1	0/1	0/0
Chromosome3-5085159	DEL	PRECISE	59245030	0	0	0/0	0/1	0/0	0/0	0/0	0/0	0/0	0/0	0/0	0/0	0/0	0/0	0/0	0/0
Chromosome3-5086470	DEL	PRECISE	1690	0	0	0/1	0/0	0/1	0/0	0/0	0/0	0/0	0/0	0/0	0/0	0/0	0/0	0/0	0/0
Chromosome3-5088207	DEL	PRECISE	2568	0	0	0/1	0/0	0/1	0/0	0/0	0/0	0/0	0/0	0/0	0/0	0/0	0/0	0/0	0/0
Chromosome3-5089489	INS	PRECISE	4513	0	0	0/0	0/0	0/0	0/1	0/0	0/0	0/0	0/0	0/0	0/0	0/0	0/0	0/0	0/0
Chromosome3-5094500	DEL	PRECISE	909	0	0	0/1	0/0	0/1	1/1	0/1	0/1	1/1	1/1	0/0	1/1	0/0	1/1	0/0	1/1
Chromosome3-5099121	DEL	PRECISE	1897	0	0	0/0	0/0	0/0	0/0	0/0	0/0	0/0	0/0	0/0	0/0	0/0	0/0	0/0	0/1
Chromosome3-5109838	INS	PRECISE	2786	0	0	0/0	0/0	0/0	0/0	0/0	0/0	0/0	0/0	0/0	0/0	0/0	0/0	0/0	0/0
Chromosome3-5113890	DEL	PRECISE	50	0	0	0/0	0/0	0/0	0/0	0/0	0/0	0/0	1/1	0/0	0/0	0/0	1/1	0/0	0/0
Chromosome3-5117451	INS	PRECISE	2534	0	0	0/0	0/0	0/0	0/0	0/0	0/0	0/0	0/0	0/0	0/0	0/0	0/0	0/1	0/0
Chromosome3-5117769	DEL	PRECISE	2728	0	0	0/0	0/0	0/0	0/0	0/0	0/0	0/0	0/0	0/0	0/0	0/0	0/0	0/0	0/1
Chromosome3-5120079	BND	PRECISE	0	0	0	0/0	0/0	0/0	0/0	0/0	0/1	0/0	0/0	0/0	0/1	0/0	0/0	0/0	0/0
Chromosome3-5122520	INS	PRECISE	6081	0	0	0/0	0/0	0/0	0/0	0/0	0/0	1/1	0/0	0/0	0/0	0/0	0/0	0/0	0/0
Chromosome3-5127084	INS	PRECISE	317	0	0	0/0	0/0	0/0	0/0	0/0	0/0	0/0	0/0	0/0	0/0	0/0	0/0	0/0	0/1
Chromosome3-5129766	INS	PRECISE	1370	0	0	0/0	0/0	0/0	0/0	0/0	0/0	1/1	0/0	0/0	0/0	0/0	0/0	0/0	0/0
Chromosome3-5133340	DEL	PRECISE	1247	0	0	0/1	0/0	0/1	0/1	0/1	0/1	0/1	0/0	0/0	0/1	0/0	0/0	0/0	0/1
Chromosome3-5135442	INS	PRECISE	137	0	0	0/0	0/0	0/0	0/0	0/0	0/0	0/0	0/0	0/0	0/0	0/1	0/0	0/0	0/0
Chromosome3-5135860	BND	PRECISE	0	0	0	0/1	0/0	0/1	0/0	0/0	0/0	0/0	0/0	0/0	0/0	0/0	0/0	0/0	0/0
Chromosome3-5150190	DEL	PRECISE	363	0	0.463	1/1	0/0	1/1	0/1	0/1	0/1	0/1	0/0	0/0	0/1	0/0	0/0	0/0	0/1
Chromosome3-5155135	INS	PRECISE	50	0	0	0/1	0/0	0/1	0/1	0/1	0/1	1/1	0/0	0/0	0/1	0/0	0/0	0/0	0/1
Chromosome3-5158564	INS	PRECISE	150	0	0	0/0	0/0	0/0	0/0	0/0	0/0	0/0	0/0	0/0	0/0	0/0	0/0	0/0	0/1
Chromosome3-5159880	DEL	PRECISE	50	0	0	0/1	0/0	0/1	0/1	0/1	0/1	1/1	0/0	0/0	0/1	0/0	0/0	0/0	0/1
Chromosome3-5160878	INS	PRECISE	87	0.809	0.302	0/1	0/0	0/1	0/1	0/1	0/1	0/1	0/1	0/0	1/1	0/0	1/1	0/1	0/1
Chromosome3-5161192	DEL	PRECISE	53	0	0	0/0	0/0	0/0	0/0	0/0	0/0	0/0	0/1	0/0	0/1	0/0	1/1	0/1	0/0
Chromosome3-5165965	INS	PRECISE	211	0	0	0/0	0/0	0/0	0/1	0/0	0/0	0/0	0/0	0/0	0/0	0/0	0/0	0/0	0/0
Chromosome3-5169235	INS	PRECISE	132	0	0	0/0	0/0	0/0	0/0	0/0	0/0	0/0	0/0	0/0	0/0	0/0	0/0	0/0	0/1
Chromosome3-5174721	DEL	PRECISE	79	0.707	0.707	0/0	0/0	0/0	0/0	0/0	0/0	0/0	0/1	0/0	0/0	0/0	0/1	0/0	0/0
Chromosome3-5174767	INS	PRECISE	779	0	0.641	0/0	0/1	0/1	0/1	1/1	1/1	0/0	0/0	1/1	0/0	1/1	0/0	0/1	0/0
Chromosome3-5186080	INS	PRECISE	1233	0	0.991	0/0	0/1	0/1	0/1	1/1	0/1	0/0	0/0	1/1	0/0	0/1	0/0	0/1	0/0
Chromosome3-5186690	DEL	PRECISE	53	0.378	0.378	0/0	0/0	0/1	0/1	0/0	0/0	1/1	0/0	0/0	0/1	0/0	0/0	0/0	0/0
Chromosome3-5192147	INS	PRECISE	367	0	0	0/1	0/0	0/1	0/0	0/0	0/0	0/0	0/0	0/0	0/0	0/0	0/0	0/0	0/0
Chromosome3-5195816	DEL	PRECISE	79	0	0	0/0	0/0	0/0	0/0	0/0	0/0	0/0	0/0	0/0	0/0	0/0	0/0	0/0	0/1
Chromosome3-5199294	DEL	PRECISE	64	0	0	0/0	0/1	0/1	0/1	0/1	0/1	0/0	0/0	1/1	0/0	0/1	0/0	0/1	0/0
Chromosome3-5201220	DEL	PRECISE	112	0	0	0/0	0/0	0/0	0/1	0/0	0/0	0/0	0/0	0/0	0/0	0/0	0/0	0/0	0/0
Chromosome3-5202065	INS	PRECISE	263	0	0.463	0/0	0/1	0/1	0/1	0/1	0/1	0/0	0/0	1/1	0/0	0/1	0/0	0/1	0/0
Chromosome3-5205549	INS	PRECISE	124	0	0	0/0	0/0	0/0	0/0	0/0	0/0	0/0	0/1	0/0	0/0	0/0	0/1	0/0	0/0
Chromosome3-5218755	DEL	PRECISE	121	0	0	0/1	0/1	1/1	0/1	0/1	0/0	0/0	0/1	1/1	0/0	1/1	0/1	1/1	0/0
Chromosome3-5220414	DEL	PRECISE	1323	0.354	0	0/1	0/0	1/1	1/1	1/1	0/0	0/1	0/0	0/0	0/1	0/0	0/0	0/0	0/1
Chromosome3-5221321	INS	PRECISE	710	0	0	0/0	0/0	0/0	0/0	0/0	0/0	0/0	0/0	0/0	0/0	0/0	0/0	1/1	0/0
Chromosome3-5221566	DEL	PRECISE	121	0	0	0/0	0/0	0/0	0/0	0/0	0/0	0/0	0/0	0/0	0/0	0/0	0/0	1/1	0/0
Chromosome3-5226875	INS	PRECISE	1963	0	0	0/0	0/0	0/0	0/0	0/0	0/0	0/0	0/0	0/0	0/0	0/0	0/0	0/0	0/1
Chromosome3-5227485	DEL	PRECISE	323	0	0	0/0	0/0	0/0	0/0	0/0	0/0	0/0	0/1	0/0	0/0	0/1	0/1	0/0	0/0
Chromosome3-5232329	DEL	PRECISE	179	0	0	0/0	0/0	0/0	0/0	0/0	0/0	0/0	0/0	0/0	0/0	0/0	0/0	0/0	0/1
Chromosome3-5235597	DEL	PRECISE	140	0	0	1/1	0/0	1/1	0/1	0/1	0/1	0/1	0/0	0/0	0/1	0/0	0/0	0/0	0/1
Chromosome3-5242141	DEL	PRECISE	56	0	0	0/0	0/0	0/0	0/0	0/0	0/0	0/0	0/0	0/0	0/0	0/0	0/0	0/0	1/1
Chromosome3-5245873	INS	PRECISE	700	0	0	0/0	0/0	0/0	0/0	0/0	0/0	0/0	0/0	0/0	0/0	0/0	0/0	0/0	1/1
Chromosome3-5246577	BND	PRECISE	0	0	0	0/1	0/0	0/1	0/0	0/0	0/0	0/0	0/0	0/0	0/0	0/0	0/0	0/0	0/0
Chromosome3-5261588	INS	PRECISE	172	0	0	0/1	0/0	0/1	0/1	0/1	0/1	0/1	0/0	0/0	0/1	0/0	0/0	0/0	0/1
Chromosome3-5264389	DEL	PRECISE	123	0	0	0/0	0/1	0/1	0/1	0/1	0/1	0/0	0/0	1/1	0/0	1/1	0/0	1/1	0/0
Chromosome3-5273812	INS	PRECISE	210	0	0.707	0/0	0/0	0/0	0/0	0/0	0/0	0/0	0/1	0/0	0/0	0/0	0/1	0/0	0/0
Chromosome3-5282818	DEL	PRECISE	153	0	0	0/0	0/0	0/0	0/0	0/0	0/0	0/0	0/0	0/0	0/0	0/0	0/0	0/0	0/1
Chromosome3-5293952	DEL	PRECISE	4849	0	0	0/0	0/0	0/0	0/1	0/1	0/0	0/1	0/0	0/0	0/0	0/0	0/0	0/0	0/0
Chromosome3-5297028	DEL	PRECISE	1772	0	0	0/0	0/0	0/1	0/0	0/1	0/0	0/0	0/0	0/0	0/1	0/0	0/0	0/0	0/0

Chromosome3-5298739	DEL	PRECISE	61	0	0	0/0	0/0	0/0	0/0	0/0	0/0	0/0	0/1	0/0	0/1	0/1	1/1	1/1	0/0
Chromosome3-5299580	DEL	PRECISE	387	0	0	0/0	0/0	0/0	0/0	0/0	0/0	0/0	0/0	0/0	0/0	0/0	0/0	0/0	0/1
Chromosome3-5301655	INS	PRECISE	150	0	0.354	0/0	0/1	0/1	0/1	0/1	1/1	0/0	0/0	1/1	0/0	1/1	0/0	1/1	0/0
Chromosome3-5304621	INS	PRECISE	150	0	0	0/0	0/0	0/0	0/0	0/0	0/0	0/0	0/0	0/0	0/1	0/0	0/1	0/0	0/0
Chromosome3-5307676	INS	PRECISE	650	0	0.707	0/1	0/0	0/1	0/0	0/0	0/0	0/0	0/0	0/0	0/0	0/0	0/0	0/0	0/0
Chromosome3-5314182	INS	PRECISE	1037	0	0.707	0/0	0/0	0/0	0/0	0/0	0/0	0/0	0/1	0/0	0/0	0/0	0/1	0/0	0/0
Chromosome3-5318635	DEL	PRECISE	770	0.802	0.802	1/1	0/1	1/1	1/1	1/1	1/1	1/1	0/1	1/1	1/1	1/1	1/1	1/1	0/1
Chromosome3-5322624	INS	PRECISE	360	0	0	0/0	0/0	0/0	0/0	0/0	0/0	0/0	0/0	0/0	0/0	0/0	0/1	0/0	0/0
Chromosome3-5325747	DEL	PRECISE	50	0	0	0/0	0/0	0/0	0/0	0/0	0/0	0/0	0/0	0/0	0/0	0/0	0/1	0/0	0/0
Chromosome3-5331418	DEL	PRECISE	118	0.463	0.463	0/1	0/0	0/1	0/1	0/1	0/1	0/1	0/0	0/0	0/1	0/0	0/0	0/0	0/1
Chromosome3-5332348	INS	PRECISE	322	0	0	0/1	0/0	0/1	0/0	0/0	0/0	0/0	0/0	0/0	0/0	0/0	0/0	0/0	0/0
Chromosome3-5335000	INS	PRECISE	68	0	0	0/1	0/0	0/1	0/1	0/1	0/1	0/1	0/0	0/0	0/1	0/0	0/0	0/0	0/0
Chromosome3-5345014	INS	PRECISE	121	0	0	0/0	0/1	0/1	0/1	0/1	0/1	0/0	0/0	1/1	0/0	0/1	0/0	0/1	0/0
Chromosome3-5351584	DEL	PRECISE	711	0	0	0/0	0/0	0/0	0/0	0/0	0/0	0/0	0/1	0/0	0/0	0/0	0/1	0/0	0/0
Chromosome3-5352384	DEL	PRECISE	131	0	0	0/1	0/0	0/1	0/1	0/1	0/1	0/1	0/0	0/0	0/1	0/0	0/0	0/0	0/1
Chromosome3-5363615	DEL	PRECISE	223	0	0	0/1	0/0	0/1	0/1	0/1	0/1	0/1	0/0	0/0	0/1	0/0	0/0	0/0	0/1
Chromosome3-5368370	DEL	PRECISE	101	0	0	0/1	0/0	0/1	0/1	0/1	0/1	0/1	0/0	0/0	0/1	0/0	0/0	0/0	0/0
Chromosome3-5370205	DEL	PRECISE	56	0	0	0/0	0/0	0/0	0/0	0/0	0/0	0/0	0/0	0/0	0/0	0/1	0/0	0/1	0/0
Chromosome3-5372675	INS	PRECISE	4922	0	0	0/0	0/0	0/0	0/0	0/0	0/0	0/0	0/0	0/0	0/0	0/0	0/0	0/0	0/1
Chromosome3-5377711	DEL	PRECISE	62	0	0	0/0	0/0	0/0	0/0	0/1	0/1	0/1	0/0	0/0	0/1	0/0	0/0	0/0	0/0
Chromosome3-5379199	DEL	PRECISE	342	0	0	0/1	0/0	0/1	0/1	0/1	0/1	0/1	0/0	0/0	0/1	0/0	0/0	0/0	0/1
Chromosome3-5384892	INS	PRECISE	88	0	0	0/0	0/0	0/0	0/0	0/0	0/0	0/0	0/0	0/0	0/0	0/0	0/0	0/0	0/1
Chromosome3-5384901	DEL	PRECISE	77	0	0	0/0	0/0	0/0	0/0	0/0	0/0	0/0	0/0	0/0	0/0	0/1	0/0	0/1	0/0
Chromosome3-5402245	INS	PRECISE	124	0	0	0/1	0/0	0/1	0/0	0/0	0/0	0/0	0/0	0/0	0/0	0/0	0/0	0/0	0/0
Chromosome3-5418262	DEL	PRECISE	117	0	0	0/0	0/0	0/0	0/0	0/0	0/0	0/0	0/0	0/0	0/0	0/0	0/0	0/0	0/1
Chromosome3-5434395	DEL	PRECISE	72	0	0	0/0	0/0	0/0	0/0	0/0	0/0	0/0	0/1	0/0	0/0	0/0	0/1	0/0	0/0
Chromosome3-5435613	DEL	PRECISE	250	0	0	0/1	0/0	0/1	0/1	0/1	0/1	0/1	0/0	0/0	0/1	0/0	0/0	0/0	0/1
Chromosome3-5437063	INS	PRECISE	1109	0	0	0/0	0/0	0/0	0/0	0/0	0/0	0/0	0/0	0/0	0/0	0/1	0/0	0/1	0/0
Chromosome3-5443618	DEL	PRECISE	58	0	0	0/0	0/0	0/0	0/0	0/0	0/0	0/0	0/0	0/0	0/0	0/1	0/0	0/1	0/0
Chromosome3-5444759	DEL	PRECISE	112	0	0	0/1	0/0	0/1	0/0	0/0	0/0	0/0	0/0	0/0	0/0	0/0	0/0	0/0	0/0
Chromosome3-5447050	INS	PRECISE	1039	0	0	0/0	0/0	0/0	0/1	0/0	0/0	0/0	0/0	0/0	0/0	0/0	0/0	0/0	0/0
Chromosome3-5466167	INS	PRECISE	201	0	0.354	0/1	0/0	0/1	0/1	0/1	0/1	0/1	0/0	0/0	0/1	0/0	0/0	0/0	0/1
Chromosome3-5486683	DEL	PRECISE	1131	0.983	0.894	0/1	0/0	0/1	0/0	0/1	0/1	0/1	0/0	0/0	0/1	0/0	0/0	0/0	0/0
Chromosome3-5489127	INS	PRECISE	133	0	0	0/0	0/1	0/1	1/1	0/1	0/1	0/0	0/0	1/1	0/0	0/1	0/0	0/0	0/0
Chromosome3-5496892	DEL	PRECISE	220	0	0	0/1	0/0	0/1	0/1	0/0	0/0	0/1	0/0	0/0	0/0	0/0	0/0	0/0	0/0
Chromosome3-5501422	INS	PRECISE	3135	0	0	0/0	0/0	0/0	0/0	0/0	0/0	0/0	0/0	0/0	0/1	0/0	1/1	0/0	0/0
Chromosome3-5517851	DEL	PRECISE	60	0	0	0/0	0/1	0/1	0/1	0/1	0/1	0/0	0/0	1/1	0/0	0/1	0/0	0/1	0/0
Chromosome3-5521837	DEL	PRECISE	90	0	0	0/0	0/1	0/1	0/1	0/1	0/1	0/0	0/0	1/1	0/0	0/1	0/0	0/1	0/0
Chromosome3-5526903	DEL	PRECISE	213	0	0	0/1	0/0	0/1	0/1	0/1	0/1	0/1	0/0	0/0	0/1	0/0	0/0	0/0	0/1
Chromosome3-5546429	INS	PRECISE	56	0	0.707	0/0	0/0	0/0	0/0	0/0	0/0	0/0	0/0	0/0	0/0	0/1	0/0	0/1	0/0
Chromosome3-5547596	INS	PRECISE	308	0	0	0/0	0/0	0/0	0/0	0/0	0/0	0/0	0/0	0/0	0/0	0/0	0/0	0/0	0/1
Chromosome3-5550318	INS	PRECISE	277	0	0.641	0/1	0/0	0/1	0/1	0/1	0/1	0/1	0/0	0/0	0/1	0/0	0/0	0/0	0/1
Chromosome3-5552551	INS	PRECISE	122	0	0.463	0/1	0/0	0/1	0/1	0/1	0/1	0/1	0/0	0/0	0/1	0/0	0/0	0/0	0/1
Chromosome3-5561637	DEL	PRECISE	106	0	0	0/1	0/0	0/1	0/1	0/1	0/1	0/1	0/0	0/0	0/1	0/0	0/0	0/0	0/0
Chromosome3-5582990	DEL	PRECISE	646	0	0	0/1	0/0	0/1	0/1	0/1	0/1	0/1	0/0	0/0	0/1	0/0	0/0	0/0	0/0
Chromosome3-5583281	INS	PRECISE	60	0	0	0/0	0/0	0/0	0/0	0/0	0/0	0/0	0/0	0/0	0/1	0/0	0/1	0/0	0/0
Chromosome3-5595227	DEL	PRECISE	86	0	0	0/0	0/0	0/0	0/0	0/0	0/0	0/0	0/1	0/0	0/1	0/0	1/1	0/0	0/0
Chromosome3-5596293	INS	PRECISE	196	0	0.707	0/0	0/0	0/0	0/0	0/0	0/0	0/0	0/1	0/0	0/0	0/0	1/1	0/0	0/0
Chromosome3-5600695	INS	PRECISE	447	0	0	0/0	0/0	0/0	0/0	0/0	0/0	0/0	0/0	0/0	0/0	0/0	0/0	0/0	0/1
Chromosome3-5603797	DEL	PRECISE	73	0	0	0/0	0/0	0/0	0/0	0/1	0/1	0/1	0/0	0/0	0/1	0/0	0/0	0/0	0/1
Chromosome3-5612399	DEL	PRECISE	117	0	0	0/0	0/1	0/1	0/1	0/1	0/1	0/0	0/0	1/1	0/1	0/1	0/1	0/1	0/0
Chromosome3-5620523	INS	PRECISE	192	0	0	0/0	0/0	0/0	0/0	0/0	0/0	0/0	0/0	0/0	0/0	0/0	0/0	0/0	0/1
Chromosome3-5620765	DEL	PRECISE	20056163	0	0	0/0	0/0	0/0	0/0	0/0	0/0	0/0	0/1	0/0	0/0	0/1	0/0	0/1	0/0
Chromosome3-5626035	INS	PRECISE	385	0	0	0/0	0/0	0/0	0/0	0/0	0/0	0/0	0/0	0/0	0/0	0/0	0/0	0/0	0/1
Chromosome3-5634907	DEL	PRECISE	621	0	0	0/0	0/1	1/1	1/1	1/1	1/1	0/0	0/0	1/1	1/1	0/1	0/1	0/1	0/0
Chromosome3-5635582	BND	PRECISE	0	0	0	0/0	0/0	0/0	0/0	0/1	0/0	0/0	0/0	0/0	0/1	0/0	0/0	0/0	0/0
Chromosome3-5641008	DEL	PRECISE	201	0	0	0/0	0/0	0/0	0/0	0/1	0/0	0/0	0/0	0/0	0/1	0/0	0/0	0/0	0/1
Chromosome3-5641286	INS	PRECISE	171	0	0.408	0/0	0/0	0/0	0/0	0/1	0/0	0/0	0/0	0/0	0/1	0/0	0/0	0/0	0/1
Chromosome3-5641384	DEL	PRECISE	1702	0	0	0/0	0/0	0/0	0/0	0/1	0/0	0/0	0/0	0/0	0/1	0/0	0/0	0/0	0/1
Chromosome3-5641408	INS	PRECISE	5347	0	0	0/0	0/0	0/0	0/0	0/0	0/0	0/0	0/0	0/0	1/1	0/0	0/0	0/0	0/0
Chromosome3-5646715	INS	PRECISE	711	0	0	0/0	0/0	0/0	0/0	0/0	0/0	0/0	0/1	0/0	0/0	0/0	1/1	0/0	0/0
Chromosome3-5648234	INS	PRECISE	659	0	0	0/0	0/0	0/0	0/0	0/0	0/0	0/0	0/0	0/0	0/0	0/0	0/0	0/0	0/1
Chromosome3-5649522	DEL	PRECISE	64	0	0	0/0	0/0	0/0	0/0	0/1	0/1	0/0	0/0	0/0	0/1	0/0	0/0	0/0	0/0
Chromosome3-5651040	DEL	PRECISE	381	0	0	0/1	0/0	0/1	0/1	0/1	0/1	0/1	0/0	0/0	0/1	0/0	0/0	0/0	0/1
Chromosome3-5663894	INS	PRECISE	1503	0	0.707	0/1	0/0	0/1	0/0	0/0	0/0	0/0	0/0	0/0	0/0	0/0	0/0	0/0	0/0
Chromosome3-5664106	BND	PRECISE	0	0	0	0/0	0/0	0/0	0/0	0/0	0/0	0/1	0/0	0/0	0/0	0/0	0/0	0/0	0/0
Chromosome3-5665059	INS	PRECISE	588	0	0.916	0/0	0/0	1/1	0/1	1/1	1/1	0/0	0/0	1/1	0/0	0/1	0/0	0/1	0/0
Chromosome3-5666974	INS	PRECISE	342	0	0.886	0/0	0/1	1/1	1/1	1/1	0/0	0/0	0/0	1/1	0/0	1/1	0/0	0/1	0/0
Chromosome3-5671611	DEL	PRECISE	54	0.577	0.577	0/0	0/0	0/0	0/0	0/0	0/1	0/0	0/0	0/0	0/1	0/0	0/0	0/0	0/0
Chromosome3-5675730	INS	PRECISE	5954	0	0	0/0	0/0	0/0	0/0	0/0	0/0	0/0	0/0	0/0	0/0	0/0	0/0	0/0	0/1
Chromosome3-5686525	DEL	PRECISE	63	0	0	0/1	0/0	0/1	0/0	0/0	0/0	0/0	0/0	0/0	0/0	0/0	0/0	0/0	0/0
Chromosome3-5686638	INS	PRECISE	369	0	0	0/0	0/0	0/0	0/0	0/0	0/0	0/0	0/0	0/0	0/0	0/0	0/0	0/0	0/1
Chromosome3-5690346	DEL	PRECISE	172	0	0	0/0	0/0	0/0	0/0	0/0	0/0	0/0	0/0	0/0	0/0	0/0	0/0	0/0	0/1

Chromosome3-5694172	DEL	PRECISE	1233	0	0	0/1	0/1	1/1	1/1	1/1	1/1	0/1	0/0	1/1	0/1	0/1	0/0	0/1	0/1
Chromosome3-5696613	DEL	PRECISE	64	0	0	0/0	0/0	0/0	0/0	0/0	0/0	0/0	0/1	0/0	0/0	0/0	0/1	0/0	0/0
Chromosome3-5700392	DEL	PRECISE	836	0	0	0/1	0/1	1/1	1/1	1/1	1/1	0/1	0/0	1/1	0/1	0/1	0/0	0/1	0/1
Chromosome3-5704535	DEL	PRECISE	194	0	0	0/0	0/0	0/0	0/0	0/0	0/0	0/0	0/0	0/0	0/1	0/0	0/1	0/0	0/0
Chromosome3-5710663	DEL	PRECISE	216	0	0	0/0	0/0	0/0	0/0	0/0	0/0	0/0	0/0	0/0	0/1	0/0	0/1	0/0	0/0
Chromosome3-5718255	DEL	PRECISE	67	0	0	0/0	0/0	0/0	0/0	0/1	0/1	0/0	0/0	0/0	0/1	0/0	0/0	0/0	0/0
Chromosome3-5724977	INS	PRECISE	152	0	0.937	0/1	0/1	0/0	0/0	0/0	0/0	0/0	0/0	0/0	0/1	0/1	0/0	0/1	0/1
Chromosome3-5725711	DEL	PRECISE	309	0.497	0	0/1	0/1	1/1	1/1	1/1	1/1	0/0	1/1	1/1	1/1	1/1	1/1	1/1	0/1
Chromosome3-5728607	BND	PRECISE	0	0	0	0/0	0/0	0/0	0/0	0/0	0/1	0/0	0/0	0/0	0/0	0/0	0/0	0/0	0/0
Chromosome3-5741065	DEL	PRECISE	551	0	0	0/0	0/0	0/0	0/0	0/0	0/0	0/0	0/0	0/0	0/1	0/0	0/1	0/0	0/0
Chromosome3-5741794	BND	PRECISE	0	0	0	0/0	0/0	0/1	0/1	0/1	0/0	0/0	0/0	0/1	0/0	1/1	0/0	0/0	0/0
Chromosome3-5748789	INS	PRECISE	623	0	0.965	0/1	0/0	1/1	1/1	1/1	1/1	0/1	0/0	1/1	0/1	0/1	0/0	0/1	0/0
Chromosome3-5749181	INV	PRECISE	43178871	0	0	0/0	0/1	0/1	0/1	0/1	0/1	0/0	0/0	0/1	0/0	0/1	0/0	0/0	0/0
Chromosome3-5754274	DEL	PRECISE	831	0	0	0/1	0/1	1/1	1/1	1/1	1/1	0/1	0/0	1/1	0/1	0/1	0/0	0/1	0/0
Chromosome3-5755239	DEL	PRECISE	470	0	0	0/0	0/0	0/0	0/1	0/0	0/1	0/1	0/0	0/0	0/1	0/0	0/0	0/1	0/0
Chromosome3-5766207	DEL	PRECISE	183	0	0	0/0	0/1	1/1	1/1	1/1	1/1	0/0	0/0	1/1	0/1	0/0	0/0	0/0	0/0
Chromosome3-5769671	DEL	PRECISE	1368	0	0	0/0	0/0	0/0	0/0	0/0	0/0	0/0	0/0	0/0	0/0	0/0	0/0	0/1	0/1
Chromosome3-5771622	DEL	PRECISE	63	0	0	0/0	0/0	0/0	0/0	0/0	0/0	0/0	0/0	0/0	0/1	0/1	1/1	0/1	0/0
Chromosome3-5775445	DEL	PRECISE	80	0	0	0/0	0/0	0/0	0/0	0/0	0/0	0/0	0/0	0/0	0/0	0/1	0/0	1/1	0/0
Chromosome3-5777083	DEL	PRECISE	338	0	0	0/0	0/0	0/0	0/0	0/0	0/0	0/0	0/1	0/0	0/0	0/0	0/1	0/0	0/0
Chromosome3-5779628	INS	PRECISE	206	0	0	0/0	0/0	0/0	0/0	0/0	0/0	0/0	0/0	0/0	0/1	0/0	0/1	0/0	0/0
Chromosome3-5780115	DEL	PRECISE	217	0	0	0/0	0/0	0/0	0/0	0/0	0/0	0/0	0/0	0/0	0/0	0/1	0/0	1/1	0/0
Chromosome3-5783125	INS	PRECISE	2625	0	0	0/0	0/0	0/0	0/0	0/0	0/0	1/1	0/0	0/0	0/0	0/0	0/0	0/0	0/0
Chromosome3-5790863	INS	PRECISE	1553	0	0.577	0/0	0/0	0/0	0/0	0/1	0/1	0/0	0/0	0/0	0/1	0/0	0/0	0/0	0/0
Chromosome3-5791477	DEL	PRECISE	54	0	0	0/0	0/0	0/0	0/0	0/0	0/0	0/0	0/0	0/0	0/0	0/0	0/0	0/1	0/1
Chromosome3-5793414	DEL	PRECISE	507	0	0	0/1	0/1	1/1	1/1	1/1	1/1	0/1	0/1	1/1	1/1	1/1	1/1	1/1	0/1
Chromosome3-5796791	INS	PRECISE	714	0	0.577	0/0	0/0	0/0	0/0	0/1	0/1	0/0	0/0	0/0	0/1	0/0	0/0	0/0	0/0
Chromosome3-5800764	DEL	PRECISE	219	0	0	0/1	0/1	1/1	1/1	1/1	1/1	0/1	0/0	1/1	0/1	0/1	0/0	0/1	0/1
Chromosome3-5808490	DEL	PRECISE	235	0	0	0/1	0/0	1/1	1/1	0/0	0/0	0/1	0/0	0/0	0/0	0/0	0/0	0/1	0/1
Chromosome3-5815422	INS	PRECISE	1440	0	0	0/0	0/0	0/0	0/0	0/0	0/0	0/1	0/0	0/0	0/0	0/0	0/0	0/0	0/0
Chromosome3-5815461	INS	PRECISE	364	0	0.707	0/0	0/0	0/0	0/0	0/0	0/0	0/0	0/0	0/0	0/1	0/0	0/1	0/0	0/0
Chromosome3-5817335	INS	PRECISE	132	0	0	0/0	0/0	0/0	0/0	0/0	0/0	0/1	0/0	0/0	0/0	0/0	0/0	0/0	0/0
Chromosome3-5817993	INS	PRECISE	817	0	0	0/0	0/0	0/0	0/0	0/0	0/0	0/0	0/0	0/0	0/1	0/0	0/1	0/0	0/0
Chromosome3-5831252	DEL	PRECISE	90	0	0	0/1	0/1	1/1	0/1	1/1	1/1	0/1	0/0	1/1	0/1	0/1	0/0	0/1	0/1
Chromosome3-5833075	DEL	PRECISE	162	0	0	0/0	0/0	0/0	0/0	0/0	0/0	0/0	0/0	0/0	0/0	0/1	0/0	0/1	0/0
Chromosome3-5842354	BND	PRECISE	0	0	0	0/1	0/0	0/1	0/0	0/0	0/0	0/0	0/0	0/0	0/0	0/0	0/0	0/0	0/0
Chromosome3-5847067	DEL	PRECISE	58	0	0	0/1	0/0	0/1	0/0	0/0	0/0	0/0	0/0	0/0	0/0	0/0	0/0	0/0	0/0
Chromosome3-5849881	DEL	PRECISE	708	0	0	1/1	0/1	0/0	1/1	1/1	1/1	0/1	1/1	0/0	1/1	0/0	0/0	1/1	0/1
Chromosome3-5853418	DEL	PRECISE	133	0	0	0/0	0/0	0/0	0/0	0/0	0/0	0/0	0/0	0/0	0/0	0/0	0/0	0/1	0/0
Chromosome3-5853601	INS	PRECISE	56	0.577	0	0/1	0/1	1/1	1/1	1/1	1/1	0/1	0/0	1/1	1/1	1/1	0/0	1/1	0/1
Chromosome3-5854019	DEL	PRECISE	8155311	0.577	0.577	0/0	0/0	0/0	0/0	0/0	0/0	0/0	0/1	0/0	0/1	0/0	0/1	0/0	0/0
Chromosome3-5859893	DEL	PRECISE	107	0	0	0/1	0/1	1/1	1/1	1/1	1/1	0/1	0/0	1/1	0/1	0/1	0/0	0/0	0/1
Chromosome3-5860046	INS	PRECISE	237	0	0.707	0/0	0/0	0/0	0/0	0/0	0/0	0/0	0/0	0/0	0/1	0/0	0/1	0/0	0/0
Chromosome3-5863084	DEL	PRECISE	113	0	0	0/0	0/0	0/0	0/0	0/0	0/0	0/0	0/0	0/0	0/1	0/0	0/1	0/0	0/0
Chromosome3-5864631	DEL	PRECISE	1065	0	0	0/1	0/1	1/1	1/1	1/1	1/1	0/1	0/0	1/1	0/1	0/1	0/0	0/1	0/1
Chromosome3-5866863	INS	PRECISE	5955	0	0	0/0	0/0	0/0	0/0	0/0	0/0	0/0	0/0	0/0	0/0	0/0	0/0	0/1	0/0
Chromosome3-5866875	BND	PRECISE	0	0	0	0/0	0/0	0/0	0/0	0/0	0/0	0/0	0/0	0/0	0/0	0/0	0/0	0/1	0/0
Chromosome3-5867342	DEL	PRECISE	203	0	0	0/1	0/1	1/1	1/1	1/1	1/1	0/1	0/0	1/1	0/1	1/1	0/0	1/1	0/1
Chromosome3-5867843	DEL	PRECISE	864	0	0	0/1	0/1	1/1	1/1	1/1	1/1	0/1	0/0	1/1	0/1	0/1	0/0	0/0	0/1
Chromosome3-5872888	INS	PRECISE	226	0	0	0/0	0/0	0/0	0/0	0/0	0/0	0/1	0/0	0/0	0/0	0/0	0/0	0/0	0/0
Chromosome3-5879605	INS	PRECISE	882	0	0	0/0	0/0	0/0	0/0	0/0	0/0	0/1	0/0	0/0	0/0	0/0	0/0	0/0	0/0
Chromosome3-5882241	INS	PRECISE	345	0	0	0/1	0/0	0/1	0/0	0/0	0/0	0/0	0/0	0/0	0/0	0/0	0/0	0/0	0/0
Chromosome3-5886832	DEL	PRECISE	279	0	0	0/1	0/1	1/1	1/1	1/1	1/1	0/1	0/0	1/1	0/1	0/1	0/0	0/1	0/1
Chromosome3-5901863	DEL	PRECISE	773	0	0	0/1	0/1	1/1	1/1	1/1	1/1	0/1	0/1	1/1	1/1	1/1	1/1	1/1	0/1
Chromosome3-5903681	INS	PRECISE	124	0.376	0.408	0/1	0/1	1/1	1/1	1/1	1/1	0/1	0/0	1/1	1/1	0/0	0/1	0/1	0/1
Chromosome3-5906211	INS	PRECISE	59	0	0	0/1	0/1	1/1	1/1	1/1	1/1	0/1	0/1	1/1	1/1	1/1	1/1	0/1	0/1
Chromosome3-5958137	INS	PRECISE	141	0	0	0/0	0/0	0/0	0/0	0/0	0/0	0/0	0/1	0/0	0/0	0/1	0/1	0/1	0/0
Chromosome3-5958592	INS	PRECISE	760	0	0	0/0	0/0	0/0	0/1	0/0	0/0	0/0	0/0	0/0	0/0	0/0	0/0	0/0	0/0
Chromosome3-5965361	DEL	PRECISE	161	0	0	0/1	0/1	1/1	1/1	1/1	1/1	0/1	0/1	1/1	1/1	1/1	1/1	1/1	0/1
Chromosome3-5968873	DEL	PRECISE	1074	0	0	0/1	0/1	1/1	1/1	1/1	1/1	0/1	0/1	1/1	1/1	1/1	1/1	1/1	0/1
Chromosome3-5971552	BND	PRECISE	0	0	0	0/0	0/1	0/1	0/0	0/1	0/1	0/0	0/0	1/1	0/0	0/0	0/0	0/0	0/0
Chromosome3-5973831	INS	PRECISE	313	0	0	0/0	0/0	0/0	0/0	0/0	0/0	0/0	0/0	0/0	0/1	0/0	0/1	0/0	0/0
Chromosome3-5980533	DEL	PRECISE	79	0	0	0/1	0/0	0/1	0/0	0/0	0/0	0/0	0/0	0/0	0/0	0/0	0/0	0/0	0/0
Chromosome3-5981681	DEL	PRECISE	84	0	0	0/0	0/0	0/0	0/1	0/0	0/0	0/1	0/0	0/0	0/0	0/0	0/0	0/0	0/0
Chromosome3-5984938	DEL	PRECISE	211	0	0	0/1	0/1	1/1	1/1	1/1	1/1	0/1	0/0	1/1	1/1	0/1	0/1	0/0	0/1
Chromosome3-5985691	INS	PRECISE	879	0	0	0/0	0/0	0/0	0/0	0/0	0/0	0/0	0/0	0/0	0/0	0/0	0/0	0/1	0/0
Chromosome3-5992154	INS	PRECISE	531	0	0	0/0	0/0	0/0	0/0	0/0	0/0	0/0	0/0	0/0	0/1	0/0	0/1	0/0	0/0
Chromosome3-6000666	DEL	PRECISE	362	0.333	0.333	0/1	0/0	1/1	1/1	1/1	0/0	0/1	0/0	1/1	0/0	0/0	0/0	0/1	0/1
Chromosome3-6004368	DEL	PRECISE	190	0	0	0/0	0/0	0/0	0/0	0/0	0/0	0/0	0/1	0/0	0/0	0/0	0/1	0/0	0/0
Chromosome3-6006186	DEL	PRECISE	748	0	0	0/0	1/1	0/0	1/1	0/0	0/0	1/1	0/0	0/0	1/1	1/1	0/0	1/1	0/1
Chromosome3-6009717	BND	PRECISE	0	0	0	0/0	0/0	0/0	0/1	0/1	0/0	0/0	0/0	0/0	0/1	0/0	0/0	0/0	0/0
Chromosome3-6019109	BND	PRECISE	0	0	0	0/0	0/0	0/0	0/0	0/0	0/0	0/0	0/0	0/0	0/0	0/0	0/0	0/0	0/1
Chromosome3-6021388	BND	PRECISE	0	0	0	0/0	0/0	0/0	0/0	0/0	0/0	0/1	0/0	0/0	0/0	0/0	0/0	0/0	0/0

Chromosome3-6023542	INV	PRECISE	871	0	0	0/0	0/1	0/1	0/1	0/1	0/1	0/0	0/0	1/1	0/0	0/1	0/0	0/0	0/0
Chromosome3-6025367	INS	PRECISE	82	0	0.548	0/0	0/0	0/0	0/0	0/0	0/0	0/0	0/1	0/0	0/1	1/1	1/1	0/1	0/0
Chromosome3-6030347	INS	PRECISE	994	0	0	0/0	0/0	0/1	0/0	0/0	0/0	0/0	0/0	0/0	0/0	0/0	0/0	0/0	0/0
Chromosome3-6033709	DEL	PRECISE	121	0.408	0.408	0/0	0/0	0/1	1/1	1/1	0/0	0/0	0/0	0/1	0/0	0/1	0/0	0/0	0/0
Chromosome3-6041501	DUP	PRECISE	51	0	0	0/0	0/1	0/0	0/0	0/1	1/1	0/0	0/0	0/1	0/0	0/0	0/0	0/0	0/0
Chromosome3-6042127	INS	PRECISE	6221	0	0	0/0	0/0	0/0	0/0	0/0	0/0	0/0	0/0	0/0	0/0	0/0	0/0	0/1	0/0
Chromosome3-6055613	DEL	PRECISE	497	0	0	0/1	0/1	1/1	1/1	1/1	1/1	0/1	0/0	1/1	0/1	0/1	0/0	0/1	0/1
Chromosome3-6059321	DEL	PRECISE	939	0	0	0/0	0/1	0/1	0/1	0/1	0/1	0/0	0/0	1/1	0/0	0/1	0/0	0/0	0/0
Chromosome3-6063615	INS	PRECISE	211	0	0	0/1	0/1	1/1	1/1	1/1	1/1	0/1	0/1	1/1	1/1	1/1	1/1	1/1	0/1
Chromosome3-6063957	DEL	PRECISE	63	0.333	0.333	0/1	0/0	0/1	0/1	1/1	0/0	0/1	0/0	0/0	0/1	0/0	0/0	0/1	0/1
Chromosome3-6067516	INS	PRECISE	312	0	0	0/0	0/0	0/0	0/0	0/0	0/0	0/0	0/0	0/0	0/1	0/0	0/1	0/0	0/0
Chromosome3-6068974	INS	PRECISE	2403	0	0	0/0	0/0	0/0	0/0	0/0	0/0	0/0	0/0	0/0	0/1	0/0	0/1	0/0	0/0
Chromosome3-6071764	INS	PRECISE	113	0	0.707	0/0	0/0	0/0	0/0	0/0	0/0	0/0	0/0	0/0	0/0	0/1	0/0	0/0	0/0
Chromosome3-6073877	INS	PRECISE	211	0	0	0/0	0/0	0/0	0/0	0/0	0/0	0/1	0/0	0/0	0/0	0/0	0/0	0/0	0/0
Chromosome3-6079896	BND	PRECISE	0	0	0	0/0	0/0	0/0	0/0	0/0	0/0	0/0	0/0	0/0	0/0	0/0	0/0	0/0	0/1
Chromosome3-6082267	BND	PRECISE	0	0	0	0/0	0/0	0/1	0/1	0/1	0/1	0/0	0/0	0/1	0/0	0/0	0/0	0/0	0/0
Chromosome3-6090324	DEL	PRECISE	1028	0.632	0	0/1	0/1	1/1	1/1	1/1	1/1	0/1	0/0	1/1	0/1	0/1	0/0	0/0	0/0
Chromosome3-6097708	DEL	PRECISE	66	0	0	0/0	0/0	0/0	0/0	0/0	0/0	0/0	0/1	0/0	0/0	0/0	0/1	0/0	0/0
Chromosome3-6100925	DEL	PRECISE	50	0	0	0/0	0/0	0/0	0/0	0/0	0/0	0/0	0/0	0/0	0/0	0/0	0/0	0/1	0/1
Chromosome3-6105026	INS	PRECISE	874	0	0.707	0/1	0/0	0/1	0/0	0/0	0/0	0/0	0/0	0/0	0/0	0/0	0/0	0/0	0/0
Chromosome3-6108463	DEL	PRECISE	148	0	0	0/0	0/0	1/1	1/1	1/1	1/1	0/1	0/0	1/1	0/0	0/1	0/0	0/0	0/0
Chromosome3-6109754	BND	PRECISE	0	0	0	0/0	0/0	0/0	0/0	0/0	0/0	0/0	0/0	0/0	0/0	0/0	0/0	0/1	0/1
Chromosome3-6115216	DEL	PRECISE	62	0.316	0.316	0/1	0/1	1/1	1/1	1/1	1/1	0/0	0/0	1/1	0/1	0/1	0/0	0/0	0/0
Chromosome3-6121286	DEL	PRECISE	52	0	0	0/0	0/0	0/0	0/0	0/0	0/0	0/0	0/0	0/0	0/1	0/0	0/1	0/0	0/0
Chromosome3-6135900	INS	PRECISE	82	0	0	0/0	0/0	0/0	0/1	0/1	0/1	0/0	0/0	0/0	0/1	0/0	0/0	0/0	0/0
Chromosome3-6138614	INS	PRECISE	135	0	0	0/0	0/0	0/0	0/0	0/0	0/0	0/0	0/0	0/0	0/1	0/0	0/1	0/0	0/0
Chromosome3-6144248	DEL	PRECISE	1943	0	0	0/0	0/1	0/1	0/1	0/1	0/1	0/0	0/0	1/1	0/0	0/1	0/0	0/0	0/0
Chromosome3-6147241	DEL	PRECISE	102	0	0	0/0	0/0	0/0	0/0	0/0	0/0	0/0	0/0	0/0	0/0	0/0	0/0	0/1	0/1
Chromosome3-6156223	INS	PRECISE	985	0	0	0/0	0/0	0/0	0/0	0/0	0/0	0/0	0/0	0/0	0/0	0/0	0/0	0/1	0/1
Chromosome3-6158317	INS	PRECISE	62	0	0	0/0	0/0	0/0	0/0	0/0	0/0	0/0	0/0	0/0	0/0	0/1	0/0	0/1	0/1
Chromosome3-6165031	DEL	PRECISE	723	0	0	0/1	0/1	1/1	1/1	1/1	1/1	0/1	0/0	1/1	0/1	0/1	0/0	0/1	0/0
Chromosome3-6169575	INS	PRECISE	349	0	0	0/0	0/0	0/0	0/0	0/0	0/0	0/0	0/0	0/0	0/0	0/0	0/0	0/1	0/0
Chromosome3-6172243	INS	PRECISE	1606	0	0	0/0	0/0	0/0	0/0	0/0	0/0	0/1	0/0	0/0	0/0	0/0	0/0	0/0	0/0
Chromosome3-6174489	DEL	PRECISE	69	0	0	0/0	0/0	0/0	0/0	0/0	0/0	0/0	0/0	0/0	0/0	0/0	0/0	0/1	0/1
Chromosome3-6179350	DEL	PRECISE	71	0	0	0/0	0/0	0/0	0/0	0/0	0/0	0/0	0/0	0/0	0/1	1/1	0/0	0/1	0/0
Chromosome3-6185774	DEL	PRECISE	221	0	0	0/0	0/0	0/0	0/0	0/0	0/0	0/0	0/1	0/0	0/0	0/0	0/1	0/0	0/0
Chromosome3-6197103	INS	PRECISE	213	0	0.894	0/0	0/0	0/0	0/1	0/1	0/1	0/1	0/0	0/0	0/1	0/0	0/0	0/0	0/0
Chromosome3-6210136	INS	PRECISE	1093	0	0	0/0	0/0	0/0	0/0	0/0	0/0	0/0	0/0	0/0	0/0	0/0	0/0	0/0	0/1
Chromosome3-6212340	INS	PRECISE	353	0	0.5	0/0	0/0	0/0	0/0	0/0	0/0	0/0	0/1	0/0	0/0	0/1	0/1	0/1	0/0
Chromosome3-6212425	DEL	PRECISE	447	0	0	0/1	0/0	0/1	0/0	0/0	0/0	0/0	0/0	0/0	0/0	0/0	0/0	0/0	0/0
Chromosome3-6213993	DEL	PRECISE	1122	0	0	0/1	0/1	1/1	1/1	1/1	1/1	0/1	0/0	1/1	0/1	0/1	0/0	0/1	0/1
Chromosome3-6223617	INS	PRECISE	131	0	0.376	0/1	0/1	1/1	1/1	1/1	1/1	0/1	0/0	1/1	1/1	1/1	0/0	1/1	0/1
Chromosome3-6226220	INS	PRECISE	5056	0	0	0/0	0/0	0/0	0/0	0/0	0/0	0/0	0/0	0/0	0/0	0/0	0/0	0/0	0/1
Chromosome3-6227882	DEL	PRECISE	187	0	0	0/0	0/0	0/0	0/0	0/0	0/0	0/0	0/0	0/0	0/1	0/1	0/0	0/1	0/0
Chromosome3-6229390	INS	PRECISE	100	0	0	0/0	0/0	0/0	0/0	0/0	0/0	0/0	0/0	0/0	0/0	0/0	0/0	0/0	0/1
Chromosome3-6233462	INS	PRECISE	1251	0	0	0/0	0/0	0/0	0/0	0/0	0/0	0/0	0/0	0/0	0/1	0/0	0/0	0/0	0/0
Chromosome3-6235432	INS	PRECISE	352	0	0.707	0/1	0/0	1/1	0/0	0/0	0/0	0/0	0/0	0/0	0/0	0/0	0/0	0/0	0/0
Chromosome3-6254598	DEL	PRECISE	437	0	0	0/0	0/1	1/1	0/0	1/1	0/0	0/0	0/0	1/1	0/0	0/0	0/0	0/0	0/0
Chromosome3-6255523	DEL	PRECISE	131	0	0	0/0	0/1	1/1	0/0	1/1	0/0	0/0	0/0	1/1	0/0	0/0	0/0	0/0	0/0
Chromosome3-6257123	INS	PRECISE	402	0	0	0/0	0/0	0/0	0/0	0/0	0/0	0/0	0/0	0/0	0/0	0/0	0/0	0/0	0/1
Chromosome3-6267854	INS	PRECISE	276	0	0	0/0	0/0	0/0	0/0	0/0	0/0	0/0	0/1	0/0	0/1	0/0	1/1	0/0	0/0
Chromosome3-6269663	DEL	PRECISE	981	0	0	0/1	0/0	1/1	1/1	1/1	1/1	0/1	0/0	0/0	0/1	0/0	0/0	0/0	0/0
Chromosome3-6270906	DEL	PRECISE	498	0	0	0/1	0/0	1/1	1/1	1/1	1/1	0/1	0/1	0/0	1/1	1/1	1/1	1/1	1/1
Chromosome3-6271756	DEL	PRECISE	813	0	0	0/1	0/0	1/1	1/1	1/1	1/1	0/1	0/0	0/0	0/1	0/0	0/0	0/0	0/0
Chromosome3-6272880	DEL	PRECISE	705	0	0	0/1	0/0	1/1	1/1	1/1	1/1	0/1	0/0	0/0	0/1	0/0	0/0	0/0	0/0
Chromosome3-6274856	INS	PRECISE	1031	0	0	0/0	0/0	0/0	0/0	0/0	0/0	0/0	0/0	0/0	0/0	0/0	0/0	0/0	0/1
Chromosome3-6276767	DEL	PRECISE	60	0	0	0/0	0/0	0/0	0/0	0/0	0/0	0/0	0/1	0/0	0/1	0/1	1/1	0/1	0/1
Chromosome3-6294124	INS	PRECISE	406	0	0	0/0	0/0	0/0	0/0	0/0	0/0	0/0	0/0	0/0	0/0	0/0	0/0	0/0	0/1
Chromosome3-6300019	INS	PRECISE	137	0	0	0/0	0/0	0/0	0/0	0/0	0/0	0/0	0/0	0/0	0/0	1/1	0/0	0/1	1/1
Chromosome3-6303277	INS	PRECISE	227	0.603	0.603	0/1	0/1	0/0	0/0	0/0	0/0	1/1	0/0	0/0	0/1	0/1	0/0	0/0	0/0
Chromosome3-6306542	DEL	PRECISE	759	0	0	0/1	0/1	1/1	1/1	1/1	1/1	0/1	0/0	1/1	0/1	0/1	0/0	0/1	0/1
Chromosome3-6314493	DEL	PRECISE	228	0	0	0/0	0/1	0/1	1/1	1/1	1/1	0/1	0/0	1/1	0/1	0/1	0/0	0/1	0/0
Chromosome3-6327597	INS	PRECISE	137	0	0	0/1	0/0	0/1	0/0	0/0	0/0	0/0	0/0	0/0	0/0	0/0	0/0	0/0	0/0
Chromosome3-6327833	DEL	PRECISE	86	0	0	0/0	0/1	0/1	0/0	0/1	0/0	0/0	0/0	1/1	0/0	0/1	0/0	0/1	0/0
Chromosome3-6339080	DEL	PRECISE	56	0	0	0/0	0/1	0/1	0/1	1/1	1/1	0/0	0/0	1/1	0/0	0/1	0/0	0/0	0/0
Chromosome3-6340559	DEL	PRECISE	329	0	0	0/1	0/0	1/1	1/1	1/1	1/1	0/0	0/1	1/1	1/1	0/1	1/1	0/0	0/0
Chromosome3-6342426	INS	PRECISE	846	0	0	0/0	0/0	0/0	0/0	0/0	0/0	0/0	0/1	0/0	0/0	0/0	0/1	0/0	0/0
Chromosome3-6344313	DEL	PRECISE	2751	0	0	0/0	0/0	0/0	0/0	0/0	0/0	0/0	0/0	0/0	1/1	0/0	0/1	0/0	0/0
Chromosome3-6346538	DEL	PRECISE	449	0	0	0/1	0/0	0/1	0/0	0/0	0/0	0/0	0/0	0/0	0/0	0/0	0/0	0/0	0/0
Chromosome3-6352240	DEL	PRECISE	157	0	0	0/0	0/0	0/0	0/1	0/0	0/0	0/0	0/0	0/0	0/0	0/0	0/0	0/0	0/0
Chromosome3-6354194	INS	PRECISE	121	0	0	0/0	0/0	0/0	0/0	0/0	0/0	0/0	0/1	0/0	0/0	0/0	0/1	0/0	0/0
Chromosome3-6355236	INS	PRECISE	171	0	0	0/0	0/0	0/0	0/0	0/0	0/0	0/0	0/0	0/0	0/0	0/0	0/0	0/0	0/1
Chromosome3-6364831	INS	PRECISE	161	0	0	0/0	0/0	0/0	0/0	0/0	0/0	0/0	0/0	0/0	0/0	0/0	0/0	0/1	0/1

Chromosome3-6365549	DEL	PRECISE	473	0	0	0/1	0/1	1/1	1/1	1/1	1/1	0/1	0/0	1/1	0/1	1/1	0/0	0/1	0/1
Chromosome3-6372167	INS	PRECISE	10975	0	0	0/0	0/0	0/1	0/0	0/0	0/0	0/0	0/0	0/0	0/0	0/0	0/0	0/0	0/0
Chromosome3-6372167	INV	PRECISE	26617529	0	0	0/1	0/0	0/0	0/0	0/0	0/0	0/0	0/0	0/0	0/0	0/0	0/0	0/0	0/0
Chromosome3-6384691	DEL	PRECISE	217	0	0	0/0	0/0	0/0	0/0	0/0	0/0	0/0	0/1	0/0	0/0	0/0	0/1	0/0	0/0
Chromosome3-6397799	DEL	PRECISE	153	0	0	0/1	0/1	1/1	1/1	1/1	1/1	0/0	0/0	1/1	0/1	0/1	0/0	0/1	0/1
Chromosome3-6401056	INS	PRECISE	1676	0	0	0/0	0/0	0/0	0/0	0/0	0/0	0/0	0/0	0/0	0/1	0/0	0/1	0/0	0/0
Chromosome3-6401776	INS	PRECISE	59	0	0	0/0	0/0	0/0	0/0	0/0	0/0	0/0	0/0	0/0	0/0	0/0	0/0	0/0	1/1
Chromosome3-6405431	INS	PRECISE	63	0	0.463	0/0	0/0	0/1	0/1	0/1	0/1	0/0	0/0	0/1	0/0	0/0	0/0	0/1	0/0
Chromosome3-6406993	INS	PRECISE	78	0	0.378	0/0	0/0	0/0	0/1	0/1	0/1	0/0	0/1	0/0	1/1	0/0	1/1	0/0	0/1
Chromosome3-6406995	DEL	PRECISE	1393	0	0	0/0	0/0	0/0	0/1	0/1	0/1	0/0	0/0	0/0	0/0	0/0	0/0	0/0	0/1
Chromosome3-6408405	DEL	PRECISE	759	0	0	0/0	0/0	0/0	0/1	0/1	0/1	0/0	0/0	0/0	0/0	0/0	0/0	0/0	0/1
Chromosome3-6409219	DEL	PRECISE	3913	0	0	0/0	0/0	0/0	0/1	0/1	0/1	0/0	0/0	0/0	0/0	0/0	0/0	0/0	0/1
Chromosome3-6410255	DEL	PRECISE	75	0	0	0/0	0/0	0/0	0/0	0/0	0/0	0/0	0/0	0/0	0/0	0/1	0/0	1/1	0/1
Chromosome3-6415899	INS	PRECISE	151	0	0	0/0	0/0	0/0	0/0	0/0	0/0	0/0	0/1	0/0	0/0	0/0	0/1	0/0	0/0
Chromosome3-6417643	INS	PRECISE	75	0	0	0/0	0/0	0/0	0/0	0/0	0/0	0/0	0/0	0/0	0/1	0/0	0/1	0/0	0/0
Chromosome3-6417643	DEL	PRECISE	75	0	0	0/1	0/1	1/1	1/1	1/1	1/1	0/1	0/0	1/1	0/1	0/1	0/0	0/0	0/1
Chromosome3-6418846	DEL	PRECISE	111	0	0	0/1	0/1	1/1	1/1	1/1	1/1	0/1	0/0	1/1	0/1	0/1	0/0	0/1	0/1
Chromosome3-6423433	DEL	PRECISE	109	0	0	0/0	0/0	0/0	0/0	0/0	0/0	0/0	0/0	0/0	0/1	0/0	0/1	0/0	0/0
Chromosome3-6427002	DEL	PRECISE	114	0	0	0/0	0/0	0/0	0/0	0/0	0/0	0/0	0/0	0/0	0/1	0/0	0/1	0/0	0/0
Chromosome3-6441435	INS	PRECISE	393	0	0	0/0	0/0	0/0	0/0	0/0	0/0	0/0	0/0	0/0	0/0	0/0	0/0	0/0	0/1
Chromosome3-6443161	DEL	PRECISE	329	0	0	0/1	0/1	1/1	1/1	1/1	1/1	0/1	0/0	1/1	1/1	0/1	1/1	1/1	0/1
Chromosome3-6450984	BND	PRECISE	0	0	0	0/1	0/0	0/0	0/1	0/1	0/0	0/0	0/0	0/0	0/1	0/0	0/0	0/0	0/0
Chromosome3-6450984	BND	PRECISE	0	0	0	0/0	0/0	0/1	0/1	0/1	0/0	0/0	0/0	0/0	0/0	0/0	0/0	0/0	0/0
Chromosome3-6456893	DEL	PRECISE	73	0	0	0/0	0/0	0/0	0/0	0/0	0/0	0/0	0/1	0/0	0/0	0/0	0/1	0/0	0/0
Chromosome3-6468416	DEL	PRECISE	691	0	0	0/0	0/0	0/0	0/0	0/0	0/0	0/0	0/0	0/0	0/0	0/0	0/0	0/0	0/1
Chromosome3-6469331	DEL	PRECISE	895	0	0	0/0	0/0	0/0	0/0	0/0	0/0	0/0	0/0	0/0	0/0	0/0	0/0	0/0	0/1
Chromosome3-6476655	INS	PRECISE	131	0.707	0.707	0/0	0/0	0/0	0/0	0/0	0/0	0/0	0/0	0/0	1/1	0/0	0/1	0/0	0/0
Chromosome3-6483272	DEL	PRECISE	199	0	0	0/1	0/0	0/1	0/0	0/0	0/0	0/0	0/0	0/0	0/0	0/0	0/0	0/0	0/0
Chromosome3-6485889	INS	PRECISE	67	0	0	0/0	0/0	0/0	0/0	0/0	0/0	0/0	0/0	0/0	0/0	0/0	0/0	0/0	0/1
Chromosome3-6486890	BND	PRECISE	0	0	0	0/0	0/0	0/0	0/0	0/0	0/0	0/0	0/0	0/1	0/0	0/0	0/0	0/0	0/0
Chromosome3-6495423	INS	PRECISE	225	0	0	0/0	0/0	0/0	0/0	0/0	0/0	0/0	0/0	0/0	0/0	0/0	0/0	0/0	1/1
Chromosome3-6495532	DEL	PRECISE	1190	0	0	0/1	0/1	1/1	1/1	1/1	1/1	0/1	0/0	1/1	0/1	0/1	0/0	0/1	0/0
Chromosome3-6500187	DEL	PRECISE	51	0	0	0/1	0/0	0/1	0/0	0/0	0/0	0/0	0/0	0/0	0/0	0/0	0/0	0/0	0/1
Chromosome3-6500515	DEL	PRECISE	5566	0.267	0	0/1	0/1	1/1	1/1	1/1	1/1	0/1	0/1	1/1	0/1	1/1	0/1	1/1	1/1
Chromosome3-6509358	INS	PRECISE	326	0	0.577	0/0	0/0	0/0	0/0	0/0	0/0	0/0	0/0	0/0	0/0	0/0	0/0	0/1	0/1
Chromosome3-6525288	INS	PRECISE	144	0	0	0/0	0/0	0/0	0/0	0/0	0/0	0/0	0/0	0/0	0/0	0/0	0/0	0/0	0/1
Chromosome3-6526155	INS	PRECISE	1331	0	0.707	0/0	0/0	0/0	0/0	0/0	0/0	0/0	0/1	0/0	0/0	0/0	0/1	0/0	0/0
Chromosome3-6528444	INS	PRECISE	1871	0	0	0/0	0/0	0/0	0/0	0/0	0/0	0/0	0/0	0/0	0/0	0/0	0/0	0/0	0/1
Chromosome3-6531670	INS	PRECISE	144	0	0.289	0/1	0/1	1/1	1/1	1/1	1/1	0/1	0/0	1/1	0/1	0/1	0/0	0/1	1/1
Chromosome3-6532915	DEL	PRECISE	586	0	0	0/0	0/0	0/0	0/0	0/0	0/0	0/0	0/1	0/0	0/0	0/0	0/1	0/0	0/0
Chromosome3-6533210	BND	PRECISE	0	0	0	0/0	0/0	0/0	0/0	1/1	0/1	0/0	0/0	0/1	0/1	0/1	0/0	0/0	0/0
Chromosome3-6533210	BND	PRECISE	0	0	0	0/0	0/0	0/0	0/0	0/0	0/0	0/0	0/0	0/1	0/1	0/0	0/0	0/1	0/0
Chromosome3-6546813	INS	PRECISE	12842	0	0	0/0	0/0	0/0	0/0	0/1	0/0	0/0	0/0	0/0	0/0	0/0	0/0	0/0	0/0
Chromosome3-6548248	DEL	PRECISE	197	0	0	0/0	0/0	0/0	0/0	0/0	0/0	0/0	0/0	0/0	0/1	0/0	0/1	0/0	0/0
Chromosome3-6549448	DEL	PRECISE	332	0	0	0/1	0/1	1/1	1/1	1/1	1/1	0/1	0/0	1/1	0/1	0/1	0/0	0/1	0/1
Chromosome3-6560279	INS	PRECISE	11677	0	0	0/0	0/0	0/0	0/0	0/0	0/0	0/0	0/0	0/0	0/0	0/0	0/0	0/0	0/1
Chromosome3-6561661	INS	PRECISE	76	0.519	0	0/1	0/1	1/1	1/1	1/1	1/1	0/1	0/1	1/1	1/1	1/1	1/1	1/1	1/1
Chromosome3-6563066	INS	PRECISE	174	0	0	0/0	0/0	0/0	0/0	0/0	0/0	0/0	0/0	0/0	0/0	0/0	0/0	0/0	0/1
Chromosome3-6569561	INS	PRECISE	357	0	0	0/0	0/0	0/0	0/0	0/0	0/0	0/0	0/0	0/0	0/0	0/0	0/0	0/0	0/1
Chromosome3-6569714	DEL	PRECISE	58	0	0	0/0	0/0	0/0	0/0	0/0	0/0	0/0	0/0	0/0	0/1	0/0	0/1	0/0	0/0
Chromosome3-6572266	INS	PRECISE	1149	0	0	0/0	0/0	0/0	0/0	0/0	0/0	0/0	0/0	0/0	0/0	0/1	0/0	0/1	0/1
Chromosome3-6577771	DEL	PRECISE	996	0	0	0/0	0/0	0/0	0/0	0/0	0/0	0/0	0/0	0/0	1/1	0/0	0/1	0/0	0/0
Chromosome3-6578781	DEL	PRECISE	159	0	0	0/0	0/0	0/0	1/1	1/1	1/1	0/0	0/0	0/0	0/1	0/0	0/0	0/0	0/0
Chromosome3-6584821	DEL	PRECISE	445	0	0	0/0	0/0	0/0	0/0	0/0	0/0	0/0	0/0	0/0	0/0	0/1	0/0	0/1	0/1
Chromosome3-6601195	INS	PRECISE	7803	0	0	0/0	0/0	0/0	0/0	0/0	0/0	0/0	0/0	0/0	0/0	0/0	0/0	0/0	0/0
Chromosome3-6611573	INS	PRECISE	307	0	0.707	0/0	0/0	0/0	0/0	0/0	0/0	0/0	0/0	0/0	0/1	0/0	0/1	0/0	0/0
Chromosome3-6612633	DEL	PRECISE	130	0	0	0/0	0/0	0/0	0/1	0/1	0/1	0/0	0/0	0/0	0/1	0/0	0/0	0/0	0/0
Chromosome3-6626165	DEL	PRECISE	164	0.289	0.289	0/1	0/1	1/1	1/1	1/1	1/1	0/1	0/0	1/1	0/1	0/1	0/0	0/1	0/0
Chromosome3-6627061	INS	PRECISE	111	0	0	0/0	0/0	0/0	0/0	0/0	0/0	0/0	0/0	0/0	0/1	0/0	0/1	0/0	0/0
Chromosome3-6630649	INS	PRECISE	1168	0	0	0/0	0/0	0/0	0/0	0/0	0/0	0/0	0/0	0/0	0/0	0/0	0/0	0/0	0/1
Chromosome3-6630916	DEL	PRECISE	84	0.289	0	0/1	0/0	1/1	1/1	1/1	1/1	0/1	0/0	1/1	0/1	0/1	0/0	0/1	0/1
Chromosome3-6631701	DEL	PRECISE	395	0	0	0/1	0/0	1/1	0/0	0/0	0/0	0/0	0/0	0/0	0/0	0/0	0/0	0/0	0/0
Chromosome3-6632790	DEL	PRECISE	219	0	0	0/1	0/1	1/1	1/1	1/1	1/1	0/0	0/0	1/1	0/1	0/1	0/0	0/1	0/1
Chromosome3-6637916	INS	PRECISE	3214	0	0.707	1/1	0/0	1/1	0/0	0/0	0/0	0/0	0/0	0/0	0/0	0/0	0/0	0/0	0/0
Chromosome3-6639034	DEL	PRECISE	230	0	0	0/1	0/1	1/1	1/1	1/1	1/1	0/1	0/0	1/1	0/1	0/1	0/0	0/1	0/1
Chromosome3-6648301	DEL	PRECISE	109	0	0	0/1	0/1	1/1	1/1	1/1	1/1	0/1	0/0	1/1	0/1	0/1	0/0	0/1	0/1
Chromosome3-6652144	DEL	PRECISE	211	0	0	0/1	0/1	1/1	1/1	1/1	1/1	0/1	0/0	1/1	0/1	0/1	0/1	0/1	0/1
Chromosome3-6657183	INS	PRECISE	855	0	0.707	0/1	0/0	0/1	0/0	0/0	0/0	0/0	0/0	0/0	0/0	0/0	0/0	0/0	0/0
Chromosome3-6658938	DEL	PRECISE	4379	0	0	0/1	0/1	1/1	1/1	1/1	1/1	0/1	0/0	1/1	0/1	0/1	0/0	0/1	0/1
Chromosome3-6674375	DEL	PRECISE	52	0	0	0/1	0/0	1/1	1/1	1/1	1/1	0/0	0/0	1/1	0/0	0/0	0/0	0/1	0/0
Chromosome3-6675898	DEL	PRECISE	832	0	0	0/0	1/1	0/0	0/0	0/0	0/0	1/1	0/0	0/0	0/0	1/1	0/0	1/1	1/1
Chromosome3-6678427	INS	PRECISE	166	0	0	0/0	0/0	0/0	0/0	0/0	0/0	0/0	0/1	0/0	0/0	0/0	0/1	0/0	0/0
Chromosome3-6697253	DEL	PRECISE	452	0	0	0/0	0/0	0/0	0/0	0/0	0/0	0/0	0/1	0/0	1/1	1/1	1/1	1/1	1/1

Chromosome3-6698843	INS	PRECISE	6938	0	0	0/0	0/0	0/0	0/0	0/0	0/0	0/0	0/0	0/0	1/1	0/0	0/0	0/0	0/0
Chromosome3-6698852	BND	PRECISE	0	0	0	0/0	0/0	0/0	0/0	0/0	0/0	0/0	0/0	0/0	0/1	0/0	0/0	0/0	0/0
Chromosome3-6698852	BND	PRECISE	0	0	0	0/0	0/0	0/0	0/0	0/0	0/0	0/0	0/0	0/0	0/1	0/0	0/0	0/0	0/0
Chromosome3-6700451	INS	PRECISE	54	0	0	0/1	0/1	1/1	1/1	1/1	1/1	0/1	0/0	1/1	0/1	0/1	0/0	1/1	0/1
Chromosome3-6729039	INS	PRECISE	455	0	0	0/0	0/0	0/0	0/1	0/0	0/0	0/0	0/0	0/0	0/0	0/0	0/0	0/0	0/0
Chromosome3-6732041	INS	PRECISE	60	0	0	0/1	0/1	1/1	1/1	1/1	1/1	0/1	0/0	1/1	1/1	1/1	0/0	1/1	1/1
Chromosome3-6739681	DEL	PRECISE	128	0	0	0/1	0/1	1/1	1/1	1/1	1/1	0/1	0/0	1/1	0/1	0/1	0/0	0/1	0/1
Chromosome3-6748195	DEL	PRECISE	996	0	0	0/1	0/1	1/1	1/1	1/1	1/1	0/1	0/0	1/1	1/1	1/1	0/1	1/1	1/1
Chromosome3-6757048	BND	PRECISE	0	0	0	0/0	0/1	1/1	0/0	0/0	0/0	0/1	0/0	0/0	0/1	0/1	0/0	0/1	0/0
Chromosome3-6766577	DEL	PRECISE	182	0	0	0/1	0/1	1/1	1/1	1/1	1/1	0/1	0/0	1/1	0/1	0/1	0/0	0/1	0/1
Chromosome3-6769292	INS	PRECISE	5782	0	0	0/0	0/0	0/0	0/0	0/0	0/0	0/0	0/0	0/0	0/0	0/0	0/0	0/0	1/1
Chromosome3-6769292	BND	PRECISE	0	0	0	0/0	0/0	0/0	0/0	0/0	0/0	0/0	0/0	0/0	0/0	0/0	0/0	0/0	0/1
Chromosome3-6773547	INS	PRECISE	1881	0	0	0/0	0/0	0/0	0/1	0/0	0/0	0/0	0/0	0/0	0/0	0/0	0/0	0/0	0/0
Chromosome3-6778053	INS	PRECISE	702	0	0	0/0	0/0	0/0	0/0	0/0	0/0	0/0	0/0	0/0	0/0	0/0	0/0	0/0	0/1
Chromosome3-6780126	INS	PRECISE	82	0	0	0/0	0/0	0/0	0/0	0/0	0/0	0/0	0/0	0/0	0/0	0/1	0/0	0/1	0/1
Chromosome3-6784835	INS	PRECISE	97	0	0	0/1	0/1	1/1	1/1	1/1	1/1	0/1	0/0	1/1	1/1	0/1	0/0	0/1	0/1
Chromosome3-6792306	BND	PRECISE	0	0	0	0/0	0/1	0/1	0/1	1/1	1/1	0/0	0/0	0/0	0/1	0/1	0/0	0/1	0/1
Chromosome3-6792944	BND	PRECISE	0	0	0	0/0	0/0	0/1	0/1	0/1	0/1	0/1	0/0	0/1	0/0	0/0	0/0	0/0	0/0
Chromosome3-6792944	BND	PRECISE	0	0	0	0/0	0/0	0/0	0/0	0/1	0/0	0/0	0/0	0/0	0/0	0/0	0/0	0/0	0/0
Chromosome3-6797468	DEL	PRECISE	273	0	0	0/1	0/0	0/1	0/0	0/0	0/0	0/0	0/0	0/0	0/0	0/0	0/0	0/0	0/0
Chromosome3-6797829	INS	PRECISE	84	0	0.539	0/1	0/0	0/1	1/1	0/1	0/1	0/1	0/0	0/1	0/1	0/1	0/0	0/1	1/1
Chromosome3-6799589	INS	PRECISE	226	0	0.707	0/0	0/0	0/0	0/0	0/0	0/0	0/0	0/1	0/0	0/0	0/0	0/1	0/0	0/0
Chromosome3-6807213	INS	PRECISE	426	0	0.894	0/0	0/0	0/0	0/1	0/1	0/1	0/0	0/0	0/1	0/1	0/0	0/0	0/0	0/0
Chromosome3-6814013	INS	PRECISE	57	0	0.707	0/0	0/0	0/0	0/0	0/0	0/0	0/0	0/0	0/0	0/0	0/0	0/0	0/1	0/1
Chromosome3-6817309	DEL	PRECISE	135	0	0	0/1	0/1	1/1	1/1	1/1	1/1	0/1	0/0	1/1	0/1	0/1	0/0	0/1	0/1
Chromosome3-6821373	DEL	PRECISE	3083	0.866	0.866	0/1	0/1	1/1	1/1	1/1	1/1	0/1	0/0	1/1	0/1	0/1	0/0	0/1	0/1
Chromosome3-6828020	INS	PRECISE	1184	0	0.707	0/0	0/0	0/0	0/0	0/0	0/0	0/0	0/0	0/0	0/1	0/0	0/1	0/0	0/0
Chromosome3-6828472	DEL	PRECISE	1199	0	0	0/0	0/0	0/0	0/0	0/0	0/0	0/1	0/0	0/0	0/0	0/0	0/0	0/0	0/0
Chromosome3-6830028	INS	PRECISE	148	0	0.408	0/0	0/0	0/0	0/0	0/0	0/0	0/0	0/1	0/0	0/1	0/1	1/1	0/1	0/1
Chromosome3-6835693	DEL	PRECISE	213	0.548	0.548	0/0	0/0	0/0	0/0	0/0	0/0	0/0	0/1	0/0	0/0	0/1	0/1	0/1	0/1
Chromosome3-6851834	DEL	PRECISE	1155	0	0	0/1	0/1	1/1	1/1	1/1	1/1	0/1	0/0	1/1	0/1	0/1	0/0	0/1	0/1
Chromosome3-6853002	INS	PRECISE	50	0	0	0/0	0/0	0/0	0/0	0/0	0/0	0/0	0/1	0/0	0/1	0/1	1/1	0/0	0/0
[truncated: 6,400,873 more chars]
